# Supplementary material for: Early Inoculation of Microbial Suspension in Suckling Piglets Affects the Transmission of Maternal Microbiota and the Associated Antibiotic Resistance Genes
Source: Microorganisms. 2020 Oct 13;8(10):1576. doi: 10.3390/microorganisms8101576 (PMC7602062; doi:10.3390/microorganisms8101576)
Supplement: Supplementary file 1 [file microorganisms-08-01576-s001.pdf]

Supplementary figures for **Early inoculation of microbial suspension in suckling piglets affected the transmission of maternal microbiota and the associated antibiotic resistance genes**

O. Zemb*[,1,a], C.S. Achard[1,2,a], V. Dupouy[3], L. Cauquil[1], N. Arpaillange[3], A. Bousquet-Melou[3], N. Le Floc'h[4]

[1]GenPhySE, Université de Toulouse, INRAE, INPT, ENVT, Castanet Tolosan, France

[2] Lallemand SAS, 19 rue des Briquetiers, BP 59, 31702 Blagnac CEDEX, France

[3] INTHERES, Universite de Toulouse, INRAE, ENVT, Toulouse, France

[4] PEGASE, INRAE, Agrocampus-Ouest, 35042, RENNES, France

* corresponding author : Olivier.zemb@inrae.fr

1. Comparing the lactating sows and the donor sows 5
1.1 Housing conditions 5
1.2 Ratio of resistant Enterobacteria (Table S1) 5
1.3 ARG abundance in the donor and lactating sows before the experiment 6
1.4 ARG abundance in the donor and lactating sows during the experiment (Table S2) 7
2. Comparing the gavaged and control piglets 8
2.1 Ratio of resistant Enterobacteria in piglets (Fig S3 and S4) 8
2.2 Graphical representation of the impact of S1 and S2 on ARGs (Fig S5 and S6) 9
2.3 Principle of the DAPC 11
2.4 OTUs that separate the piglets are rare in the donor sows (Fig S6) 12
2.5 ARG ratios in the sows and in the S1 piglets at weaning 12
2.5.1 Table of ARG in S1 piglets at weaning (Table S3) 12
2.5.2 Table of ARGs in S2 piglets (Table S4) 14
2.5.3 Visualization of the ARG ratio in S1 and S2 piglets (Fig S2) 16
3. Neutral model applied to the piglets 18
3.1 Rationale of the neutral model 18
3.2 Necessity of estimating the metacommunity from piglets (Fig S8) 18
Figure S8: Repartition of the frequency of the OTUs in S1, S2 and control piglets compared to the abundance of the OTUs in the lactating sows 18
3.3 Fitting the neutral model to control, S1 and S2 piglets of sow722 (Fig S7) 19
4. Linking OTUs and ARGs 20
4.1 Rationale of the MINE method 20
4.2 Examples of ARG-OTU relationships (Fig S10) 21
Figure S10: Representative relationships found between OTUs and ARGs for linear (left), co-exclusion (middle), and uncharacterized (right) relationships. 21
4.3 Distribution of the MIC under real and randomized conditions (Fig S11) 21
4.4 Network of ARGs and OTUs at weaning (Fig S12) 22
5. Technical tables (Table S5) 23
1.1 Table S5: List of primers used in this paper 23
4.5 Identification of samples 24
4.6 OTU table 27
4.7 Table of significant links between ARGs and OTUs 4613

4.8 Fasta of representative sequences 4617

List of Tables:	
Table S1: Counts of tetracycline and sulfamide-resistant Enterobacteria	Page 5
Table S2 : ARG abundances in the 6 samples from the donor sows and in the 12 samples from the lactating sows	Page 7
Table S3 : Significance of the exclusion and relative abundance of targeted genes in the S1 and control piglets at weaning	Page 12
Table S4 : Significance of the exclusion and relative abundance of targeted genes in the S2 donor	Page 14
Table S5: List of primers used in this paper	Page 22
**List of Figures:**	
Figure S1: Control of ARG variability in the donor and lactating sows 3 months before the experiment.	Page 6
Figure S2: Relative abundance of ARGs in S1 and S2 piglets at weaning vs the abundance of ARGs in the donor sow	Page 16
Figure S3: Proportion of Enterobacteriaceae resistant to sulfnoamide (left) and tetracycline (right) in piglets at 14 days and at 4 weeks (i.e. weaning)	Page 8
Figure S4 : Lactating sow effect on the Enterobacteriaceae resistant to tetracycline in piglets at 4 weeks (weaning).	Page 8
Figure S5 : Graphical representation of the impact of the S1 suspension on ARGs at 14 days and 4 weeks	Page 9
Figure S6 : Graphical representation of the impact of the S2 suspension on ARGs at 14 days and 4 weeks	Page 10
Figure S7 : Fitting the neutral model to control, S1 and S2 piglets of sow722.	Page 19
Figure S8: Repartition of the frequency of the OTUs in S1, S2 and control piglets compared to the abundance of the OTUs in the lactating sows.	Page 18
Figure S9: Number of reads of the OTUs in the donor sows by their global contribution in the DAPC	Page 12
Figure S10: Representative relationships found between OTUs and ARGs	Page 21
Figure S11 : comparison of the distribution of the maximal information coefficient index in the dataset before and after randomization.	Page 21
Figure S12 : Network of significant ARGs and OTUs relationships at weaning	Page 22

1. Comparing the lactating sows and the donor sows

1.1 Housing conditions

The pigs were housed in farming conditions as authorized by the French regulation on animal experimentation for experimentations conducted with an agronomic purpose.
At 106 days of gestation, sows were moved to the farrowing room with natural (windows) and artificial light. They were kept in individual farrowing crates (2 x 2.5 m) equipped with a though and a drinker. The floor of the farrowing pens was made of slatted plastic. The farrowing crates were equipped with 2 infrared heat bulbs for piglets. The ambient temperature was kept at 24 to 25 °C in the lactation rooms with heating and ventilation. Farrowing was induced by an intramuscular injection of prostaglandin F2α (2 mL of Dinolytic, Zoetis, France) on day 114 of gestation. Usual farm practices for newly farrowed piglets were individual identification by tagging, iron injection, tail docking, and castration of male piglets. Cross-fostering, if needed, was performed intra-treatment within 2 days after birth but piglets selected for blood and feces samplings were maintained with her biological mother.

1.2 Ratio of resistant Enterobacteria (Table S1)

Table S1: Counts of tetracycline and sulfamide-resistant Enterobacteria, Ntot number of strains isolated, Ntet and Ptet, number and proportion of strains resistant to tetracylcine, Nsul, number and proportion of strains resistant to sulfonamide. The averages and standard errors are reported as well. It should be noted that the unexpected rise of the proportion of antibiotic-resistant enterobacteria in the donor sows is not due to the use of antibiotics (since no antibiotics were used in the experiment), but could be due to the evolution of the microbiota around parturition [*Huang, X.; Gao, J.; Zhao, Y.; He, M.; Ke, S.; Wu, J.; Zhou, Y.; Fu, H.; Yang, H.; Chen, C., et al. Dramatic Remodeling of the Gut Microbiome Around Parturition and Its Relationship With Host Serum Metabolic Changes in Sows.* Front Microbiol ***2019***, 10, *2123, doi:10.3389/fmicb.2019.02123.]*

Sow	use	date	Ntot	Ntet	Nsul	P_tet	tet_average	tet_stdev	P_sul	sul_average	sul_stdev
sow268	donor	prior	9	1	0	11%	6%	8%	0%	0%	0
sow206	donor	prior	10	0	0	0%			0%		
sow512	Lact.	prior	10	4	5	40%	36%	15%	50%	31%	17%
sow709	Lact.	prior	10	2	4	20%			40%		
sow710	Lact.	prior	10	3	1	30%			10%		
sow722	Lact.	prior	9	5	2	56%			22%		
sow512	Lact.	05fev (14 days)	10	2	0	20%	68%	33%	0%	50%	52%
sow709	Lact.	05fev (14 days)	10	9	10	90%			100%		
sow710	Lact.	05fev (14 days)	10	9	9	90%			90%		
sow722	Lact.	05fev (14 days)	10	7	1	70%			10%		
sow268	donor	16fev (weaning)	10	3	4	30%	50%	28%	40%	40%	0.00
sow206	donor	16fev (weaning)	10	7	4	70%			40%		
sow512	Lact.	16fev (weaning)	10	4	0	40%	48%	37%	0%	0%	0
sow709	Lact.	16fev (weaning)	8	5	0	63%			0%		
sow710	Lact.	16fev (weaning)	10	0	0	0%			0%		
sow722	Lact.	16fev (weaning)	8	7	0	88%			0%		

1.3 ARG abundance in the donor and lactating sows before the experiment

In addition to the ratio of resistant Enterobacteria, we performed a screening based on *blaTEM*, *TetM*, *sul2* and *FlorR* 3 months before the begin of the experiment to ensure that some variability in ARGs profiles was present. The results are indicated in the figure below:

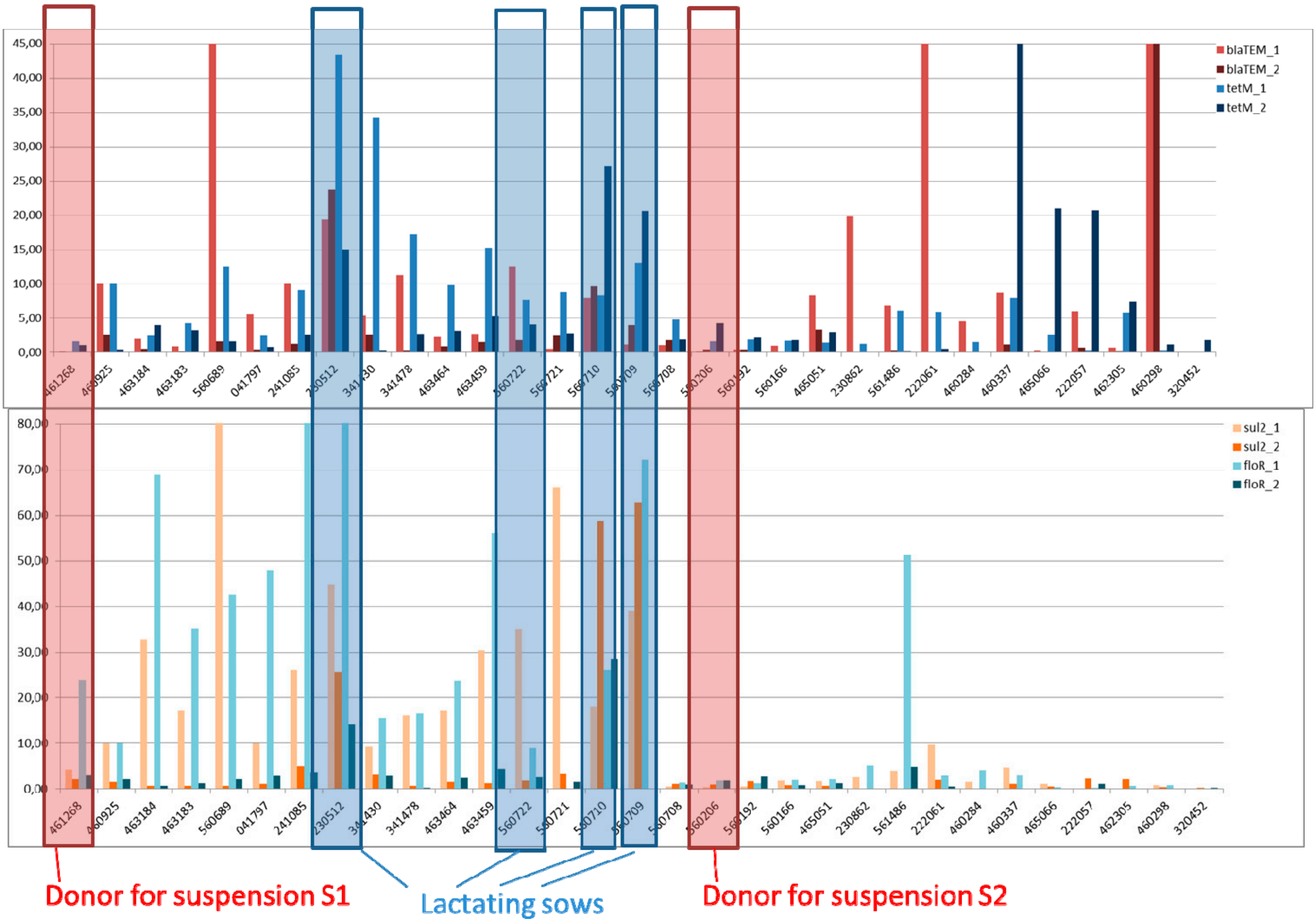

Figure S1: Control of ARG variability in the donor and lactating sows 3 months before the experiment. The ARGs are quantified as described previously.

1.4 ARG abundance in the donor and lactating sows during the experiment (Table S2)

Table S2 : ARG abundances in the 6 samples from the donor sows and in the 12 samples from the lactating sows. The abundances are normalized by the abundance of the 16S rRNA gene. The numbers are not between 0 and 1 because the ARGs were pre-amplified whereas the 16S rRNA genes were not. The measures performed on the day of weaning are in bold. The ARGs aadA, aadA2, lnuB and tetB are highlighted because their uncorrected p-value (according to a wilcoxon test) is lower than 0.05.

			Donor sows (2 sows sampled 3 times)						Lactating sows (4 sows sampled 3 times)											
	raw p-value	pval_corrected_for_FDR	D206_2511	D206_0312	D206J2	D268_2511	D268_0312	D268J2	T512_J0	T215_J1	T512J2	T709_J0	T709_J1	T709J2	T710_J0	T710_J1	T710J2	T722_J0	T722_J1	T722J2
aadA	0.041	0.381	1.3	1.0	**2.4**	0.2	0.1	**2.6**	1.0	59.5	**4.0**	3.0	12.5	**6.1**	3.5	1.2	**0.4**	6.5	0.4	**8.6**
aadA2_2	0.044	0.381	2.2	1.9	**4.1**	0.6	0.4	**4.0**	1.5	60.9	**5.8**	4.6	14.6	**6.4**	6.1	2.7	**1.0**	6.5	1.0	**8.1**
aadE	0.750	0.931	8.7	9.4	**6.0**	5.5	5.0	**6.3**	8.0	54.1	**16.3**	1.9	13.1	**6.7**	3.6	3.3	**2.8**	4.1	3.3	**9.8**
aadE.1	0.750	0.931	8.9	9.9	**6.2**	5.7	5.4	**6.4**	8.5	55.8	**16.0**	2.0	13.0	**7.5**	3.8	3.3	**2.9**	4.3	3.4	**10.5**
aadE_2	0.053	0.381	7.1	9.9	**7.5**	4.7	8.6	**9.8**	7.3	31.6	**18.1**	8.7	27.2	**10.6**	10.9	12.3	**7.5**	7.9	8.8	**20.8**
ant6Ib	0.553	0.866	6.6	9.3	**12.9**	4.2	8.9	**17.9**	7.5	0.7	**32.1**	4.7	38.1	**14.3**	8.9	11.8	**9.5**	7.5	12.7	**31.3**
ant6Ib.1	0.682	0.931	7.1	10.8	**15.1**	4.3	9.5	**18.1**	6.9	0.7	**37.4**	5.1	39.9	**13.1**	10.3	12.3	**12.2**	7.1	15.1	**27.3**
aph3Ib	0.102	0.461	55.1	94.9	**15.6**	146.8	57.8	**92.6**	0.8	36.7	**46.3**	13.0	41.2	**16.2**	20.7	18.3	**5.4**	607.7	15.3	**184.4**
aphA3	0.494	0.866	2.2	2.6	**1.9**	1.5	1.3	**2.7**	1.7	60.7	**3.5**	1.5	8.1	**3.4**	1.9	2.5	**1.5**	1.3	1.5	**5.9**
aphA3.1	0.750	0.931	2.5	2.8	**2.0**	1.7	1.4	**2.9**	1.8	60.3	**3.7**	1.6	8.4	**3.7**	1.9	2.5	**1.6**	1.3	1.6	**6.5**
blaTEM	0.291	0.698	0.6	1.2	**1.8**	0.7	0.1	**18.9**	0.7	3.8	**0.5**	0.5	44.8	**11.7**	1.5	23.4	**1.5**	124.4	0.8	**2.8**
dfrA1	0.682	0.931	0.1	0.1	**0.0**	0.0	0.0	**0.1**	0.0	72.0	**0.0**	0.0	0.3	**0.3**	0.2	0.0	**0.0**	4.8	0.0	**0.1**
ermB	0.892	0.964	34.5	13.3	**3.6**	4.1	5.8	**5.3**	3.0	43.7	**7.1**	4.7	14.3	**7.6**	4.4	6.5	**5.9**	4.7	2.4	**16.7**
ermF	0.553	0.866	15.6	13.7	**5.1**	12.0	14.5	**23.5**	3.9	3.6	**6.1**	8.8	42.7	**10.6**	17.5	24.9	**14.3**	6.4	12.1	**14.1**
ermG	0.213	0.698	4.8	4.5	**2.6**	1.8	1.6	**3.3**	1.5	71.7	**3.6**	1.3	12.2	**2.4**	9.7	5.4	**5.1**	2.3	10.3	**9.6**
is26	0.213	0.698	2.0	1.5	**0.2**	3.5	1.0	**4.9**	102.5	78.5	**14.6**	0.6	0.6	**0.8**	13.0	2.4	**67.2**	15.6	0.4	**6.6**
is6100	0.494	0.866	17.1	12.3	**1.3**	14.1	6.1	**3.1**	3.3	57.5	**5.5**	1.0	13.2	**4.8**	1.3	2.6	**1.7**	22.3	1.7	**3.6**
lnuB	0.032	0.381	201.7	209.5	**41.2**	105.6	74.5	**56.3**	143.7	2.4	**174.2**	7.9	27.5	**31.4**	17.2	5.9	**12.9**	72.9	16.2	**84.5**
lnuC	0.250	0.698	13.3	13.2	**9.0**	10.6	13.3	**9.0**	9.7	9.9	**16.1**	10.6	38.7	**13.6**	15.8	17.4	**12.1**	8.2	11.4	**20.9**
mefA	0.385	0.814	11.3	16.3	**10.3**	8.1	15.6	**11.0**	7.9	11.6	**11.8**	10.8	31.2	**13.1**	16.3	19.3	**11.6**	10.8	11.0	**19.1**
MGtetM	0.892	0.964	11.7	8.1	**16.7**	4.8	2.1	**7.1**	329.9	73.0	**9.0**	1.0	8.8	**2.4**	53.4	11.5	**8.4**	4.3	3.7	**4.2**
strB	0.083	0.427	76.0	68.3	**18.3**	180.7	54.0	**87.6**	2.2	27.0	**34.4**	5.5	37.8	**20.3**	19.0	11.5	**3.2**	555.0	10.6	**196.7**
tet32	0.291	0.698	10.1	6.5	**3.8**	5.8	9.0	**11.4**	15.0	9.7	**10.2**	4.6	30.8	**7.5**	9.1	29.9	**5.8**	6.4	9.6	**25.2**
tet40_2	0.553	0.866	14.0	12.6	**7.2**	8.8	12.4	**10.5**	7.8	7.3	**17.4**	9.3	42.8	**12.1**	15.1	21.2	**11.3**	7.3	10.3	**19.0**
tetA_2	0.892	0.964	17.5	12.0	**4.8**	4.5	9.7	**22.1**	610.3	1.6	**71.8**	4.8	49.4	**11.4**	5.0	32.0	**3.6**	8.4	5.8	**12.9**
tetB_2	0.041	0.381	0.2	0.1	**0.0**	0.1	0.2	**0.7**	0.3	72.0	**0.8**	0.8	0.6	**0.3**	0.6	0.3	**0.0**	6.5	0.1	**2.2**
tetG	0.892	0.964	9.4	24.5	**13.2**	7.5	14.5	**4.8**	2.4	1.0	**15.0**	5.4	43.6	**2.1**	10.2	16.3	**12.3**	5.5	16.9	**23.4**
tetL	0.437	0.866	168.1	192.8	**2.6**	104.4	71.7	**1.4**	387.7	57.8	**4.2**	4.5	9.4	**2.1**	12.0	17.6	**8.4**	5.7	8.7	**4.8**
tetM	0.291	0.698	17.0	28.4	**3.7**	6.7	6.6	**40.4**	127.2	10.6	**43.4**	44.0	5.5	**51.4**	26.4	5.9	**31.9**	6.5	6.9	**94.2**
tetM_2	0.250	0.698	18.0	34.5	**4.6**	6.2	7.3	**45.3**	129.9	12.9	**54.1**	55.1	5.9	**47.3**	33.0	6.6	**41.4**	5.8	9.1	**89.3**
tetO	0.964	0.964	12.6	11.0	**4.8**	8.0	11.4	**7.5**	6.1	9.7	**10.3**	6.6	40.4	**10.8**	9.8	13.8	**8.2**	4.9	7.9	**11.7**
tetO.1	0.964	0.964	12.6	11.0	**4.6**	7.9	11.6	**7.8**	6.1	9.3	**10.0**	6.4	41.3	**11.4**	10.1	13.5	**8.3**	5.2	7.7	**12.4**
tetQ	0.616	0.925	13.5	11.7	**7.5**	7.7	12.6	**11.1**	5.1	9.3	**13.4**	8.4	33.7	**11.1**	13.0	15.7	**10.0**	7.9	11.3	**16.6**
tetR	0.964	0.964	16.2	13.0	**5.0**	6.6	12.4	**25.1**	576.6	2.5	**74.6**	5.9	45.6	**10.2**	6.6	29.6	**5.4**	9.3	6.6	**13.7**
tetW.1	0.083	0.427	11.6	10.4	**7.3**	7.8	10.8	**10.8**	9.8	17.7	**15.7**	12.1	29.2	**12.0**	13.1	13.0	**7.4**	9.5	9.2	**22.1**
tetX	0.385	0.814	18.1	25.4	**6.6**	9.5	16.6	**19.7**	3.4	6.2	**4.9**	8.0	29.5	**11.6**	9.1	27.6	**10.1**	9.0	11.7	**23.7**

2. Comparing the gavaged and control piglets

2.1 Ratio of resistant Enterobacteria in piglets (Fig S3 and S4)

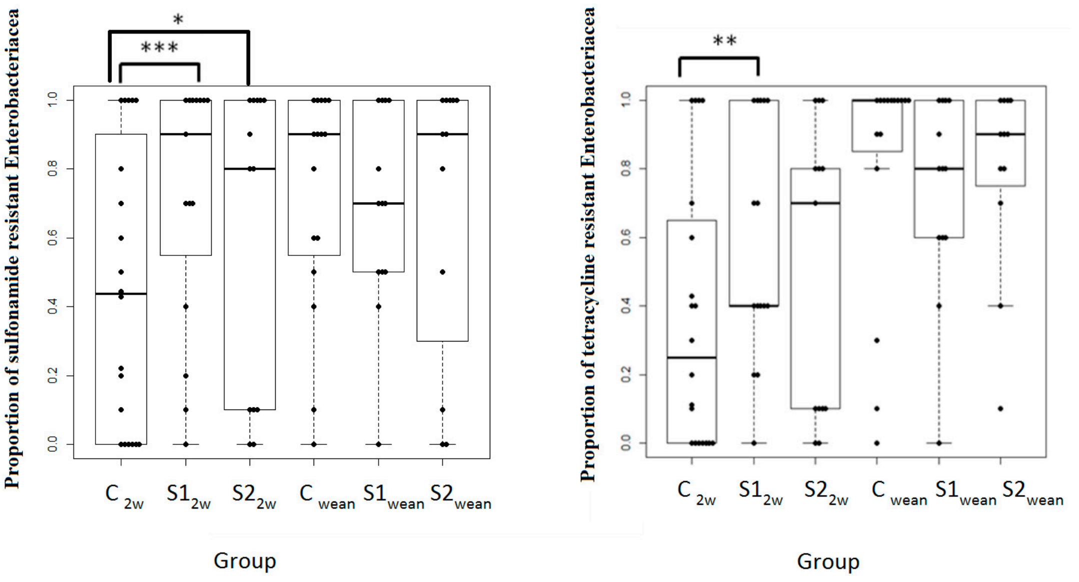

Figure S3: Proportion of Enterobacteriaceae resistant to sulfamethoxazole (left) and tetracycline (right) in piglets at 14 days and at 4 weeks (i.e. weaning). The proportion was measured by counting the Enterobacteria plated on a selective medium and then screening for the antibiotic-resistance of 10 isolates per sample. Stars indicate p-values below 0.05,0.01 and 0.001 respectively. The control piglets are labeled 'C' while the piglets force-fed with the microbial suspensions are labeled 'S1' and 'S2' respectively.

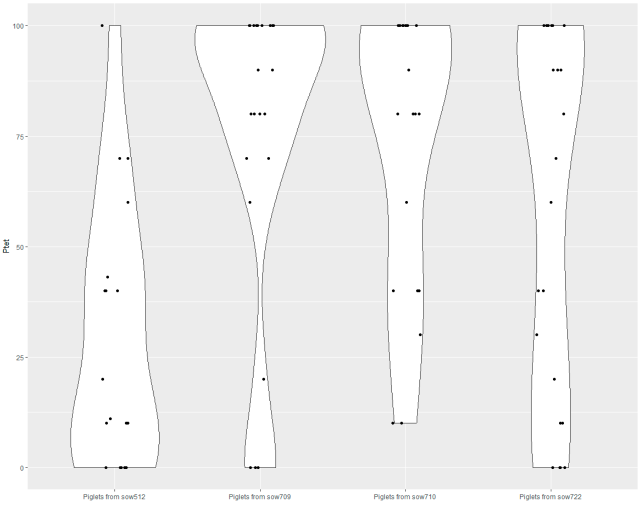

Figure S4 : Lactating sow effect on the Enterobacteriaceae resistant to tetracycline in piglets at 4 weeks (weaning). We used the violin plot from ggplot2 to visualize the impact of the lactating sow at weaning.

2.2 Graphical representation of the impact of S1 and S2 on ARGs (Fig S5 and S6)

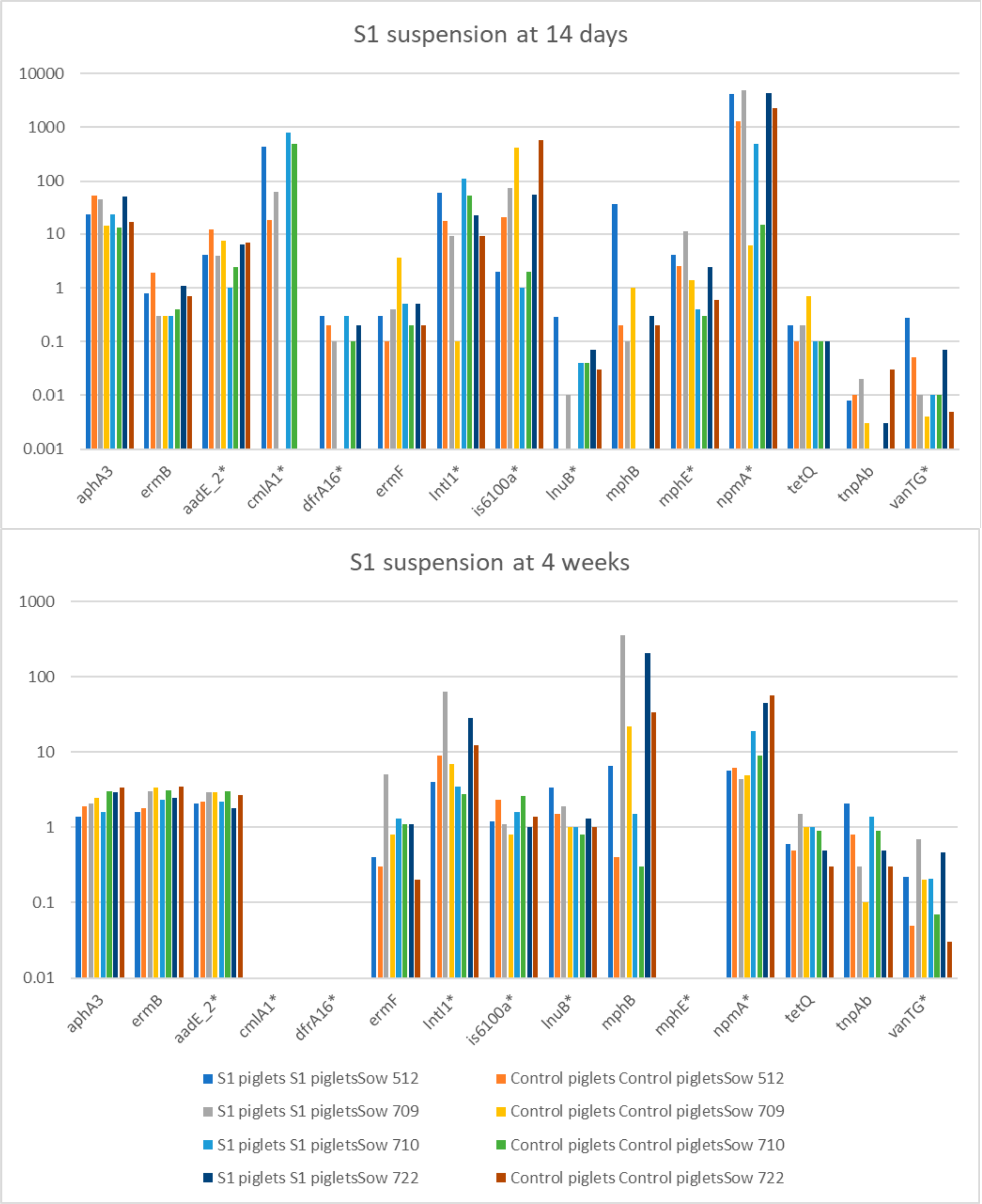

Figure S5 : Graphical representation of the impact of the S1 suspension on ARGs at 14 days and 4 weeks

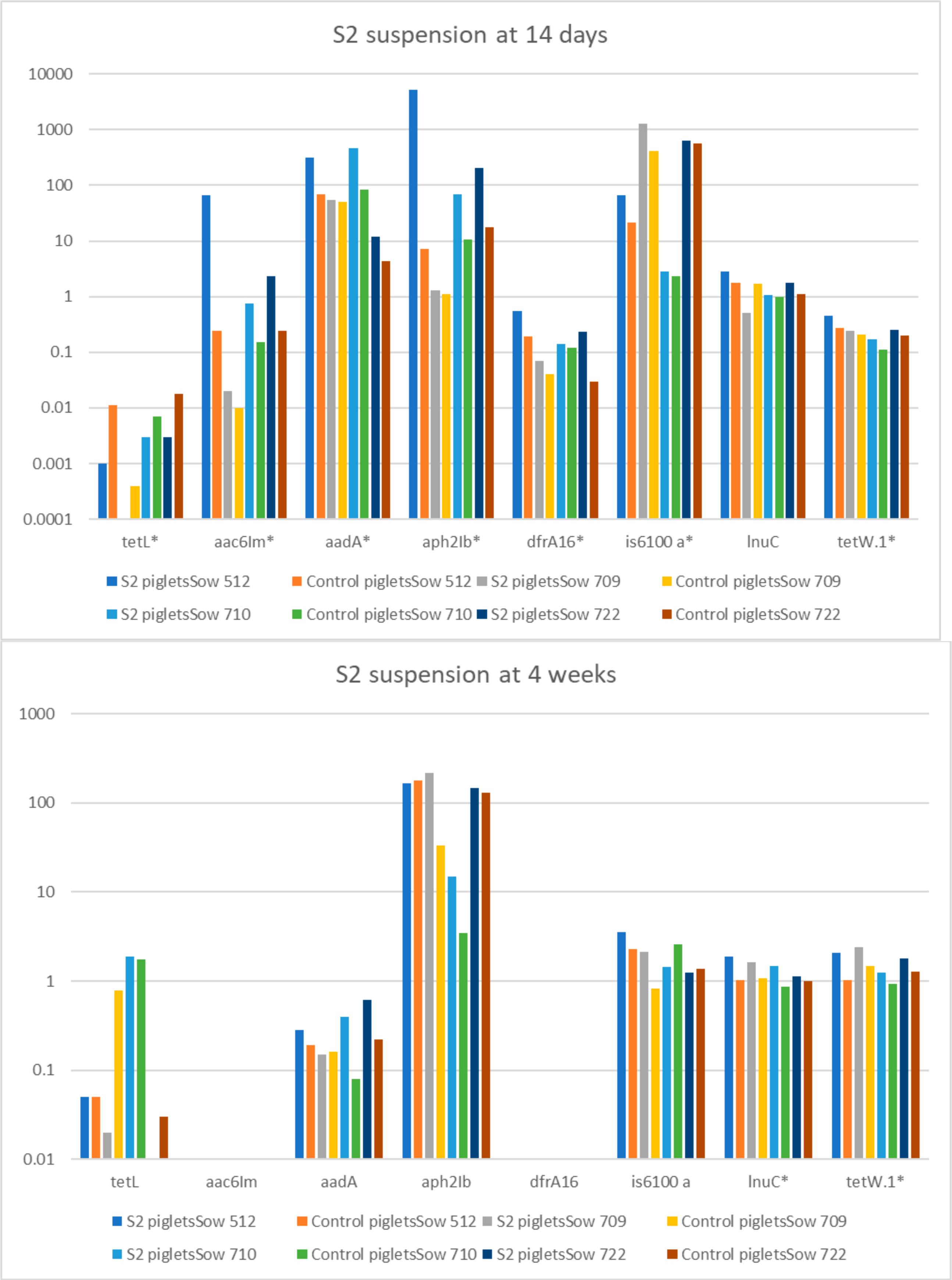

Figure S6 : Graphical representation of the impact of the S1 suspension on ARGs at 14 days and 4 weeks

2.3 Principle of the DAPC

Here we give more detail about the statistics that we used to determine the separation of the gavaged and control piglets via DAPC. DAPC (Jombart et al., 2010) stands for Discriminant Analysis of Principal Components. The first advantage of DAPC lies in the fact that many OTUs are in fact colinear, making them difficult to use in a discriminant analysis. DAPC solves this issue by using a PCA to get orthogonal axes. The second advantage of DAPC lies in the power of the discriminant analysis, which allows to isolate differences that are related to a particular treatment by optimizing the variance between groups while minimizing the variance within groups.

The pitfall of this method is that it will obtain some degree of separation for any groups of individual, especially given the high number of OTUs. The overfitting can be mesured by the a-score, which is the difference between the proportion of successful reassignment and values obtained using random groups. In practice, the p-values corresponds to the proportion of simulation with a better a-score than the 'real' groups. In other words, we performed 1000 simulations with random groups, and we count how many have a better separation than the real observation (i.e. how many a-scores are negative out of the 1000 a-scores obtained). If the separation is due to overfitting, then many simulations will obtain a similar proportion of successful reassignments, leading to many a-scores being negative. In contrast, very few simulations will obtain the a-score of a separation that is not random.

Therefore, the use of DAPC allows us to pool the two sampling dates and the use of a-scores generates an estimation of the statistical significance that takes into account the overfitting. The use of the randomization is close to the rationale of a PERMANOVA. However, in our case the PERMANOVA is not significant (p=0.4) because the major effect is the date of sampling, making the effect of inoculation negligible.

The interested reader is invited to read the initial publication :

T. Jombart, S. Devillard, and F. Balloux, Discriminant analysis of principal components: a new method for the analysis of genetically structured populations. BMC Genet 11 (2010) 94.

2.4 OTUs that separate the piglets are rare in the donor sows (Fig S6)

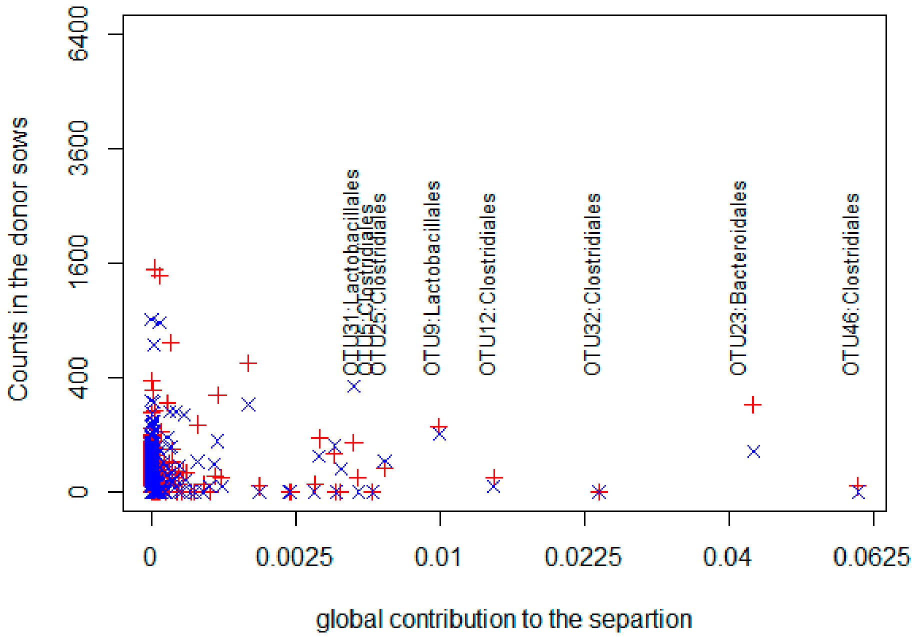

Figure S9: Number of reads of the OTUs in the donor sows by their global contribution in the DAPC. The 8 OTUs needed to significant separation are labeled.

2.5 ARG ratios in the sows and in the S1 piglets at weaning

2.5.1 Table of ARG in S1 piglets at weaning (Table S3)

Table S3 : Significance of the exclusion and relative abundance of targeted genes in the S1 and control piglets at weaning. The abundances are normalized by the abundance of the 16S rRNA gene. The numbers are not between 0 and 1 because the ARGs were pre-amplified whereas the 16S rRNA genes were not (see material and methods). For example the control piglets of Sow512 had up to 33.57/0.42=79.9 fold less mphB than the control piglets from Sow722. The p-values correspond to a 2-way non-parametric Friedman test based on the medians.

		Lactating Sow 512		Lactating Sow 709		Lactating Sow 710		Lactating Sow 722						
ARG ID	p-value for piglets	S1_piglets_with_sow512	Control_piglets_with_sow512	S1_piglets_with_sow709	Control_piglets_with_sow709	S1_piglets_with_sow710	Control_piglets_with_sow710	S1_piglets_with_sow722	Control_piglets_with_sow722	S1 donor (sow268)	Mean_Lactating_Sow512	Mean_Lactating _Sow709	Mean_Lactating _Sow710	Mean_Lactating _Sow722
aadA	0.32	0.05	0.19	1.43	0.16	0.11	0.08	1.22	0.22	0.09	2.36	0.41	0.16	0.36
aadA2_2	0.32	0.10	0.27	1.74	0.28	0.23	0.22	1.56	0.34	0.15	2.45	0.50	0.30	0.37
aadE	0.32	2.11	2.91	2.83	3.67	2.54	3.76	2.59	2.33	0.59	2.52	0.40	0.29	0.38
aadE.1	0.32	2.26	2.91	2.92	3.84	2.60	3.91	2.70	2.35	0.62	2.59	0.43	0.30	0.39
aadE_2	0.32	2.11	2.23	2.91	2.86	2.18	3.03	1.84	2.66	0.73	1.68	0.89	0.86	0.81
ant6Ib	0.32	1.22	1.12	0.29	1.04	0.50	0.79	0.82	0.96	0.92	0.73	0.93	0.85	1.07

ant6Ib.1	0.32	1.35	1.09	0.34	1.20	0.53	0.75	0.74	1.17	0.95	0.81	0.91	1.01	1.06
aph2Ib	1.00	23.32	179.3	43.02	33.25	19.77	3.51	88.24	129.6	NA	NA	0.87	0.66	0.24
aph3Ib	0.32	25.11	17.81	286.9	13.62	2.71	2.64	241.3	509.6	11.98	2.19	1.34	1.24	23.68
aphA1	1.00	17.68	10.53	1.73	1.45	2.92	3.21	4.69	7.96	27.94	1.75	NA	NA	NA
aphA3	0.05	1.40	1.89	2.05	2.48	1.57	3.03	2.91	3.42	0.19	2.41	0.23	0.16	0.17
aphA3.1	0.05	1.41	2.13	2.16	2.48	1.67	3.26	2.93	3.35	0.20	2.40	0.25	0.17	0.18
blaTEM	1.00	0.31	0.91	80.32	29.89	12.94	37.80	33.12	28.68	0.56	0.17	0.71	0.47	4.26
blaTEM_2	0.32	0.27	0.98	383.0	130.7	39.52	105.5	158.4	247.9	NA	0.11	1.00	0.64	9.12
cepA	1.00	0.08	0.06	0.02	0.09	0.01	0.03	2.88	2.85	NA	NA	0.02	NA	0.12
dfrA1	0.32	0.01	0.10	2.06	0.13	0.01	0.00	1.91	0.39	0.00	2.75	0.01	0.01	0.16
ermB	0.05	1.58	1.82	2.99	3.43	2.32	3.15	2.46	3.53	0.50	1.85	0.55	0.47	0.47
ermF	0.05	0.42	0.28	5.13	0.78	1.28	1.11	1.09	0.16	1.64	0.33	0.98	1.54	0.75
ermG	0.32	5.00	5.67	6.83	9.95	7.27	5.21	7.87	9.64	0.23	2.83	0.21	0.62	0.50
IntI1[a]	0.32	4.00	8.89	63.72	6.99	3.45	2.83	28.47	12.51	1.61	NA	0.46	0.90	3.63
IntI2[a]	1.00	0.01	0.02	0.16	0.06	0.00	0.02	0.06	0.02	0.00	3.05	0.01	NA	0.01
is26[a]	0.32	1.52	0.74	3.76	0.68	0.98	0.26	2.28	5.91	0.35	5.60	0.06	2.95	0.63
is6100[a]	0.32	1.17	2.31	1.09	0.82	1.63	2.58	0.98	1.36	1.00	2.36	0.29	0.15	0.85
lnuB	0.05	3.43	1.48	1.86	1.03	0.99	0.79	1.28	0.96	9.09	6.29	1.63	1.19	4.17
lnuC	1.00	0.94	1.03	1.67	1.07	1.32	0.86	0.96	1.00	1.13	0.87	1.14	1.29	0.90
mefA	1.00	0.96	0.73	1.31	0.86	0.81	1.26	0.35	0.36	1.10	0.82	1.09	1.32	0.95
MGtetM	0.32	0.44	1.84	26.21	2.38	11.07	14.58	2.41	2.68	0.51	10.54	0.18	2.46	0.31
mphB	0.05	6.48	0.42	359.4	21.92	1.48	0.28	206.2	33.57	1.65	NA	2.05	2.43	2.11
npmA	0.32	5.74	6.23	4.45	4.94	19.33	9.00	45.31	57.07	NA	1.27	0.81	0.28	0.06
strB	1.00	14.22	11.98	243.1	15.73	2.25	2.28	190.8	317.6	13.63	1.65	1.18	0.99	21.97
sul2	1.00	0.08	0.13	2.74	0.17	0.06	0.12	2.46	1.62	0.06	NA	NA	0.01	0.18
tet32	1.00	1.07	1.86	0.81	0.66	0.71	0.94	1.24	0.74	0.84	0.88	0.63	1.00	0.86
tet40_2	0.32	1.42	0.97	1.81	1.75	1.37	1.37	1.46	1.39	1.06	0.75	1.05	1.29	0.81
tetA_2	1.00	4.25	1.78	46.62	14.54	18.32	20.06	11.51	15.08	1.08	15.32	0.89	0.80	0.63
tetB_2	1.00	0.17	0.20	0.83	0.55	0.15	0.18	2.05	0.91	0.03	2.77	0.05	0.03	0.25
tetG	0.32	1.51	0.78	1.07	1.44	1.08	0.89	0.29	0.20	0.88	0.34	0.54	1.07	0.99
tetH	0.32	1.50	1.93	1.02	1.06	1.06	2.06	0.54	0.41	0.25	NA	0.28	NA	NA
tetL	1.00	0.06	0.05	1.01	0.78	0.42	1.77	0.02	0.03	7.45	11.21	0.31	1.01	0.50
tetM	1.00	0.20	0.42	4.30	0.49	1.71	2.42	0.46	0.40	1.63	4.06	3.65	2.22	1.83
tetM_2	0.32	0.17	0.40	3.86	0.47	1.94	2.39	0.44	0.48	1.75	4.38	3.93	2.82	1.79
tetO	1.00	1.22	1.02	1.87	1.63	1.68	2.25	1.32	1.41	0.91	0.68	0.89	0.87	0.55
tetO.1	1.00	1.26	1.06	1.99	1.67	1.67	2.35	1.34	1.43	0.92	0.66	0.91	0.88	0.57
tetQ	0.05	0.61	0.55	1.46	1.01	1.02	0.94	0.46	0.33	1.02	0.69	0.93	1.08	0.82
tetR	1.00	4.15	1.72	31.30	14.06	4.38	9.41	7.71	9.73	1.34	14.62	0.87	0.89	0.70
tetT	0.32	1.95	2.07	1.63	1.03	4.29	2.56	4.09	2.01	1.87	NA	NA	NA	NA
tetW.1	0.32	1.00	1.03	1.79	1.47	1.02	0.93	1.38	1.28	0.98	1.16	1.08	0.95	0.90
tetX	1.00	0.70	0.71	1.47	0.52	1.02	2.11	1.71	0.31	1.45	0.40	0.91	1.12	0.97
tnpA[a]	0.05	2.11	0.83	0.33	0.16	1.47	0.94	0.59	0.39	27.75	NA	1.59	2.15	NA
vanTG	0.05	0.22	0.05	0.70	0.23	0.22	0.07	0.47	0.03	1.77	NA	1.71	4.11	1.29
X16SZhu	NA	1.00	1.00	1.00	1.00	1.00	1.00	1.00	1.00	1.00	1.00	1.00	1.00	1.00

[a]:Not an ARG

2.5.2 Table of ARGs in S2 piglets (Table S4)

Table S4 : Significance of the exclusion and relative abundance of targeted genes in the S2 donor. The abundances are normalized by the abundance of the 16S rRNA gene. The numbers are not between 0 and 1 because the ARGs were pre-amplified whereas the 16S rRNA genes were not (see material and methods). The p-values correspond to a 2-way non-parametric Friedman test based on the medians.

		Lactating Sow 512		Lactating Sow 709		Lactating Sow 710		Lactating Sow 722						
ARG ID	p-value piglets	S2_piglets_with_sow512	Control_piglets_with_sow512	S2_piglets_with_sow709	Control_piglets_with_sow709	S2_piglets_with_sow710	Control_piglets_with_sow710	S2_piglets_with_sow722	Control_piglets_with_sow722	S2 donor (sow206)	Mean_Lactating_Sow512	Mean_Lactating_Sow709	Mean_Lactating_Sow710	Mean_Lactating_Sow722
aadA	0.32	0.28	0.19	0.15	0.16	0.40	0.08	0.62	0.22	0.12	2.36	0.41	0.16	0.36
aadA2_2	0.05	0.42	0.27	0.31	0.28	0.73	0.22	0.58	0.34	0.21	2.45	0.50	0.30	0.37
aadE	0.32	2.73	2.91	3.55	3.67	3.61	3.76	4.39	2.33	0.56	2.52	0.40	0.29	0.38
aadE.1	0.32	2.83	2.91	3.76	3.84	3.77	3.91	4.62	2.35	0.58	2.59	0.43	0.30	0.39
aadE_2	1.00	3.53	2.23	2.65	2.86	2.58	3.03	2.86	2.66	0.59	1.68	0.89	0.86	0.81
ant6Ib	0.32	1.03	1.12	0.85	1.04	0.65	0.79	1.03	0.96	0.72	0.73	0.93	0.85	1.07
ant6Ib.1	0.32	1.11	1.09	0.97	1.20	0.58	0.75	0.97	1.17	0.83	0.81	0.91	1.01	1.06
aph2Ib	0.32	167.9	179.3	218.1	33.25	14.91	3.51	147.8	129.6	NA	NA	0.87	0.66	0.24
aph3Ib	0.05	19.23	17.81	21.88	13.62	15.04	2.64	964.5	509.6	3.67	2.19	1.34	1.24	23.68
aphA1	0.32	16.87	10.53	5.31	1.45	3.49	3.21	3.21	7.96	4.49	1.75	NA	NA	NA
aphA3	0.32	2.32	1.89	4.23	2.48	2.27	3.03	4.31	3.42	0.16	2.41	0.23	0.16	0.17
aphA3.1	0.32	2.25	2.13	4.12	2.48	2.34	3.26	4.15	3.35	0.17	2.40	0.25	0.17	0.18
blaTEM	0.32	0.21	0.91	11.44	29.89	51.90	37.80	13.72	28.68	0.09	0.17	0.71	0.47	4.26
blaTEM_2	0.32	0.18	0.98	34.50	130.7	189.9	105.5	37.55	247.9	0.04	0.11	1.00	0.64	9.12
cepA	0.32	0.18	0.06	0.58	0.09	0.03	0.03	0.19	2.85	NA	NA	0.02	NA	0.12
dfrA1	0.32	0.01	0.10	0.14	0.13	0.01	0.00	0.50	0.39	0.00	2.75	0.01	0.01	0.16
ermB	1.00	3.47	1.82	3.86	3.43	2.96	3.15	3.52	3.53	1.12	1.85	0.55	0.47	0.47
ermF	1.00	0.17	0.28	0.41	0.78	1.89	1.11	0.42	0.16	0.77	0.33	0.98	1.54	0.75
ermG	1.00	7.87	5.67	9.49	9.95	9.95	5.21	5.99	9.64	0.27	2.83	0.21	0.62	0.50
IntI1[a]	0.32	4.61	8.89	9.89	6.99	9.98	2.83	20.44	12.51	0.79	NA	0.46	0.90	3.63
IntI2[a]	1.00	0.03	0.02	0.06	0.06	0.01	0.02	0.01	0.02	NA	3.05	0.01	NA	0.01
is26[a]	0.05	2.17	0.74	0.79	0.68	1.55	0.26	10.23	5.91	0.08	5.60	0.06	2.95	0.63
is6100[a]	1.00	3.56	2.31	2.15	0.82	1.43	2.58	1.25	1.36	0.66	2.36	0.29	0.15	0.85
lnuB	0.32	4.17	1.48	3.84	1.03	0.64	0.79	2.54	0.96	9.98	6.29	1.63	1.19	4.17
lnuC	0.05	1.87	1.03	1.62	1.07	1.47	0.86	1.12	1.00	0.83	0.87	1.14	1.29	0.90
mefA	0.32	0.67	0.73	1.09	0.86	1.29	1.26	0.60	0.36	0.89	0.82	1.09	1.32	0.95
MGtetM	1.00	0.74	1.84	4.29	2.38	17.67	14.58	1.00	2.68	0.91	10.54	0.18	2.46	0.31
mphB	1.00	0.55	0.42	13.08	21.92	1.72	0.28	1.94	33.57	2.44	NA	2.05	2.43	2.11
npmA	0.32	0.22	6.23	3.24	4.94	24.02	9.00	0.67	57.07	NA	1.27	0.81	0.28	0.06
strB	0.05	15.55	11.98	23.82	15.73	15.39	2.28	728.9	317.6	3.61	1.65	1.18	0.99	21.97
sul2	0.32	0.41	0.13	0.24	0.17	0.15	0.12	0.80	1.62	0.03	NA	NA	0.01	0.18
tet32	0.32	2.48	1.86	2.92	0.66	0.70	0.94	1.94	0.74	0.47	0.88	0.63	1.00	0.86
tet40_2	0.32	1.66	0.97	1.79	1.75	1.57	1.37	1.12	1.39	0.78	0.75	1.05	1.29	0.81

tetA_2	1.00	17.94	1.78	9.46	14.54	41.23	20.06	1.32	15.08	0.77	15.32	0.89	0.80	0.63
tetB_2	0.32	0.52	0.20	0.13	0.55	0.25	0.18	3.58	0.91	0.01	2.77	0.05	0.03	0.25
tetG	1.00	0.58	0.78	0.98	1.44	3.30	0.89	1.19	0.20	1.12	0.34	0.54	1.07	0.99
tetH	1.00	1.73	1.93	3.26	1.06	0.94	2.06	0.43	0.41	NA	NA	0.28	NA	NA
tetL	0.32	0.05	0.05	0.02	0.78	1.90	1.77	0.01	0.03	7.78	11.21	0.31	1.01	0.50
tetM	0.32	0.18	0.42	0.69	0.49	2.22	2.42	0.24	0.40	1.08	4.06	3.65	2.22	1.83
tetM_2	1.00	0.21	0.40	0.74	0.47	2.66	2.39	0.24	0.48	1.26	4.38	3.93	2.82	1.79
tetO	0.32	1.22	1.02	1.30	1.63	2.22	2.25	1.00	1.41	0.65	0.68	0.89	0.87	0.55
tetO.1	0.32	1.23	1.06	1.41	1.67	2.17	2.35	1.06	1.43	0.64	0.66	0.91	0.88	0.57
tetQ	1.00	0.40	0.55	0.56	1.01	1.94	0.94	0.54	0.33	0.76	0.69	0.93	1.08	0.82
tetR	1.00	14.26	1.72	8.04	14.06	27.85	9.41	1.33	9.73	0.77	14.62	0.87	0.89	0.70
tetT	0.32	2.73	2.07	1.80	1.03	5.37	2.56	1.82	2.01	1.79	NA	NA	NA	NA
tetW.1	0.05	2.08	1.03	2.39	1.47	1.25	0.93	1.78	1.28	0.68	1.16	1.08	0.95	0.90
tetX	1.00	0.28	0.71	0.69	0.52	1.25	2.11	0.77	0.31	1.12	0.40	0.91	1.12	0.97
tnpA[a]	0.32	2.32	0.83	0.78	0.16	0.43	0.94	0.55	0.39	32.39	NA	1.59	2.15	NA
vanTG	0.32	0.09	0.05	0.13	0.23	0.26	0.07	0.88	0.03	0.90	NA	1.71	4.11	1.29
X16SZhu	NA	1.00	1.00	1.00	1.00	1.00	1.00	1.00	1.00	1.00	1.00	1.00	1.00	1.00

[a] : not an ARG

2.5.3 Visualization of the ARG ratio in S1 and S2 piglets (Fig S2)

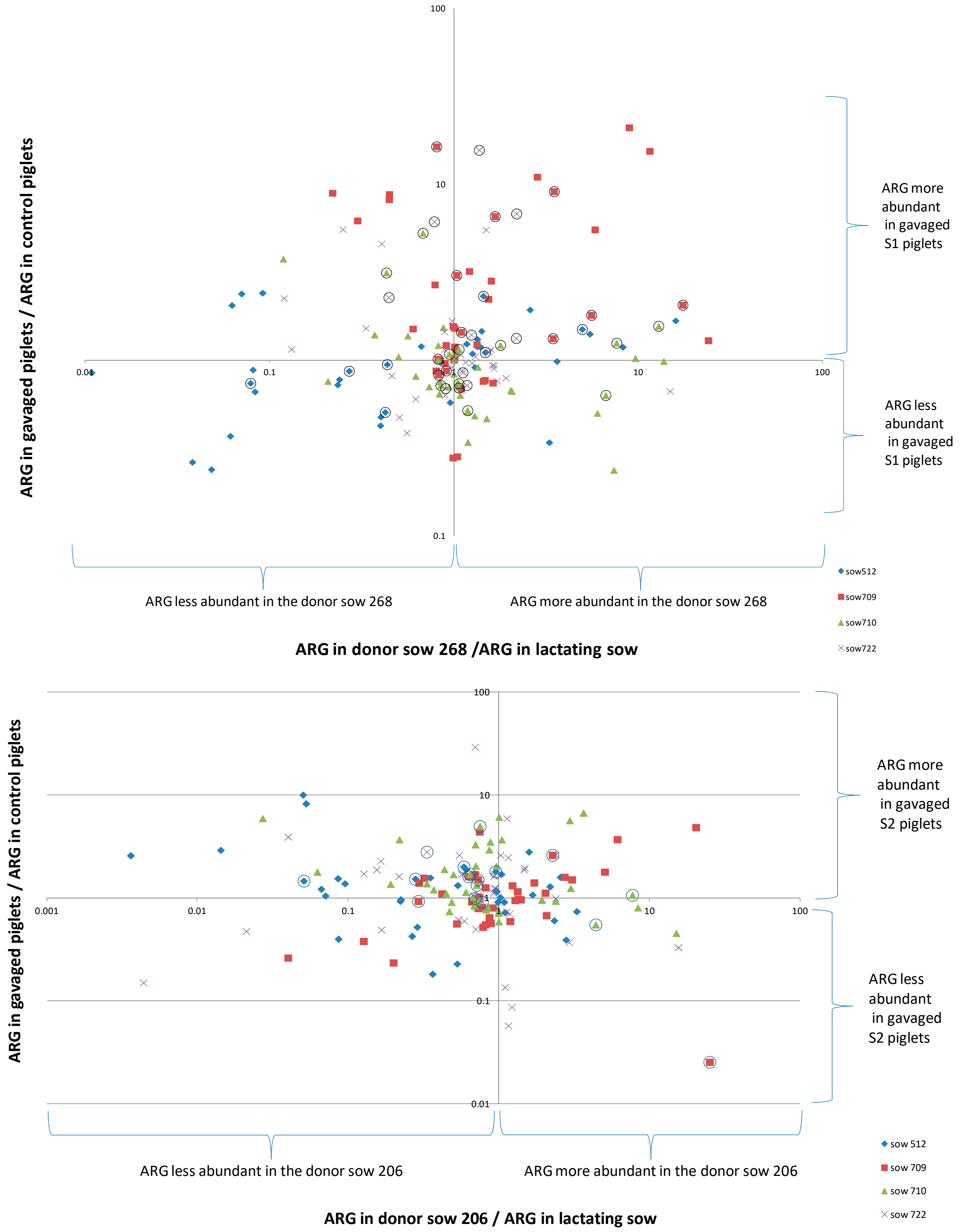

Figure S2: Relative abundance of ARGs in S1 and S2 piglets at weaning (as ratio of the ARG in the control piglets) vs the abundance of ARGs in the donor sow (as ratio of the ARG in the lactating sow). For each ARG in Table S1 (top, corresponding to S1 donor sow 268) or Table S2 (bottom, corresponding to S2 donor sow 206), the X axis indicated the ratio between the relative abundance in the piglets gavaged with suspension

S1 over the relative abundance in the control piglets. The Y-axis indicates the ratio between the relative abundance in the donor sow over the relative abundance in the lactating sow. ARGs that are significantly impacted by the inoculation of suspension are circled. Each sow has a different marker. The axes use a logarithmic scale. The green areas indicate the areas where a low (resp. high) abundance of ARG in the donor translates to a low(resp.high) abundance in the gavaged piglets, i.e the hypothesis that the ARG level in the sows is mirrored in the piglets.
This figure illustrates the challenge of predicting the success of the exclusion based solely on the ARG level in the donor and the lactating sow because a strong ARG contrast is not enough to guarantee a successful exclusion of the ARG.

3. Neutral model applied to the piglets

3.1 Rationale of the neutral model

The rationale of the neutral model is that a metacommunity (microbes surrounding the piglet in our case) feed a local community (microbes in the gut of the piglets). The neutral model assumes that species are 'neutral' in their ecological fitness (i.e. each species has the same growth, death and dispersion rates), and the assembly is the resulting stochastic phenomenon.

The metacommunity can either be objectively measured (such as the microbiota of the milking sow, and the microbiota of the donor sow), or it can be derived from the observation across all the samples. The basic idea is that an OTU that is rare in the metacommunity will be observed in very few local community while an OTU that is abundant in the metacomunity will be observed in every local comunity.

In our case, we first show that the link between the microbiota of the sows and the microbiota of the piglets is tenuous, as most of the OTUs observed in every piglet is not observed in the sows (with identical sequencing depth, Fig S4). We then proceed to fit the neutral model by estimating the metacommunity from the observations across all the piglets (Fig S5). The interested reader is invited to read the publication describing the algorithm fitting the neutral model : A.R. Burns, W.Z. Stephens, K. Stagaman, S. Wong, J.F. Rawls, K. Guillemin, and B.J. Bohannan, Contribution of neutral processes to the assembly of gut microbial communities in the zebrafish over host development. The ISME journal 10 (2016) 655-64.

3.2 Necessity of estimating the metacommunity from piglets (Fig S8)

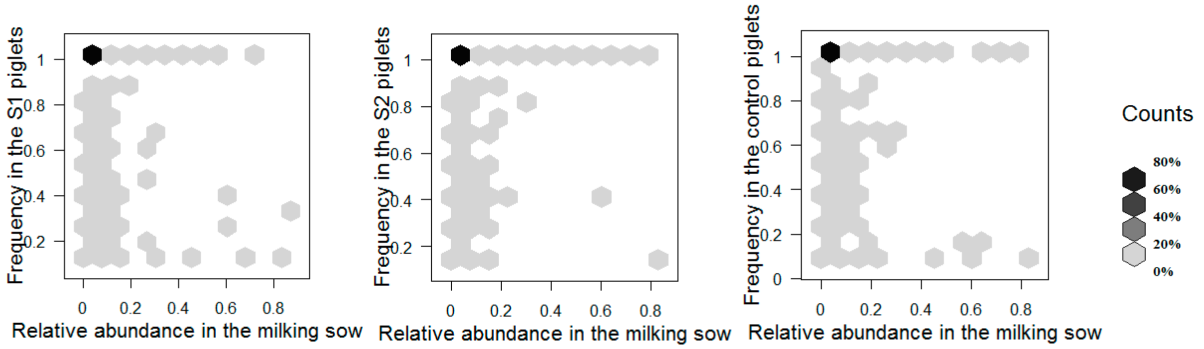

Figure S8: Repartition of the frequency of the OTUs in S1, S2 and control piglets compared to the abundance of the OTUs in the lactating sows. Each OTU is plotted with its abundance in the milking sow (X-axis) vs the number of piglets in which it was observed across the 4 lactating sows (i.e. out of 3x4 piglets for S1 or S2, out of 4x4 piglets for the control) (Frequency on the Y-Axis). The area was divided in hexagons with the hexbin package of R, and the density of each hexagon was color-coded. This figure shows that the piglets gavaged with S1, S2 or saline water all had more than 80% of their OTUs that were present in every piglet, yet undetectable in their milking sow sampled at the same sequencing depth. This contrast is in clear opposition with the idea that the milking sow provides a metacommunity that colonize the gut of the piglet following a neutral model (at least in the control piglets), and justifies the use of an extrapolated metacommunity to fit the neutral model on the gut microbes of the piglets (Fig.2).

3.3 Fitting the neutral model to control, S1 and S2 piglets of sow722 (Fig S7)

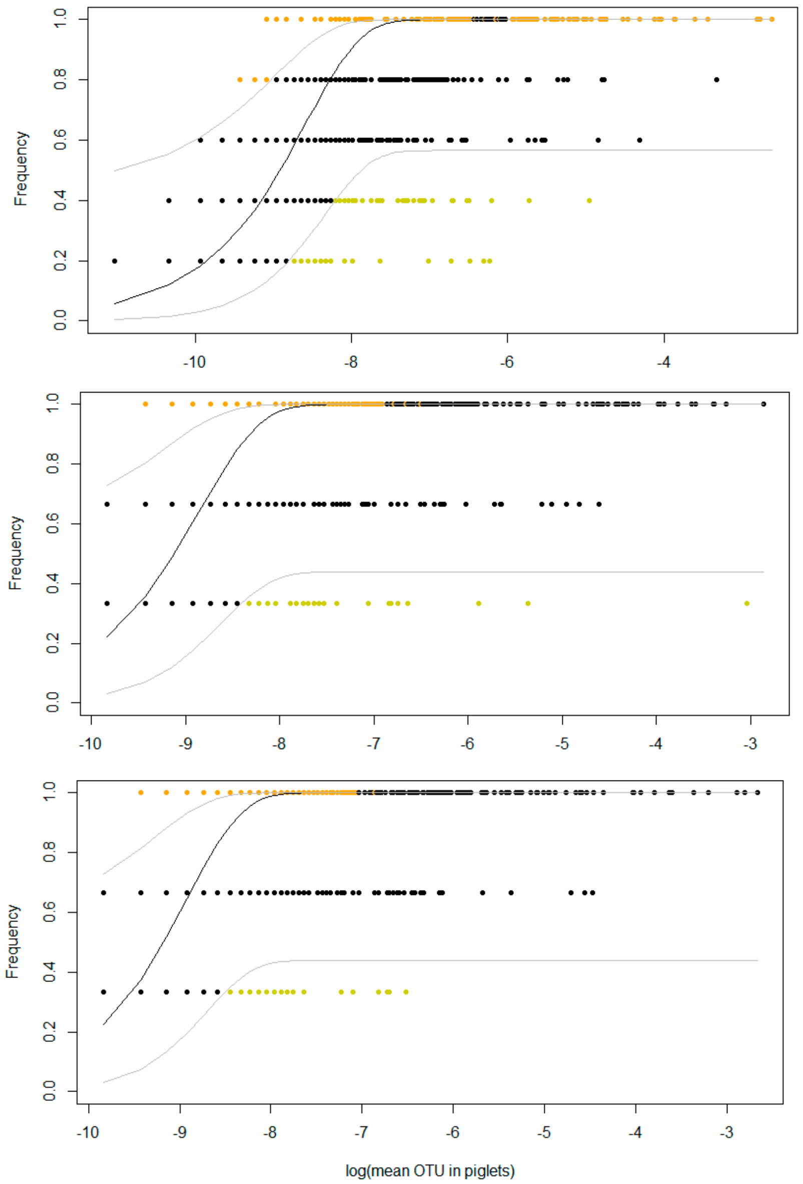

Figure S7 : Fitting the neutral model to control, S1 and S2 piglets of sow722. The controls piglets (TOP) show an acceptable fit (R^2=0.45) while piglets force-fed with S1 (MIDDLE) and S2 (BOTTOM) are not well described by the model (R^2<0.04). OTUs more frequent than expected are in orange, OTUs less frequent than expected are in green. The lack of fit of the neutral model for the gavaged piglets (in contrast to the control piglets for which the neutral model offers a decent fit) indicates that the inoculation of microbial suspension in the early life modified the rules of assembly of the microbial communities.

4. Linking OTUs and ARGs

4.1 Rationale of the MINE method

In our dataset, we merged the data of ARG abundance and 16S gene for each sample. We then look at the relationships between ARG and OTU using the MINE software (see Material and Method section), which can detect simultaneously linear and non-linear relationships. The linear and non-linear relationships between the selected OTUs and ARGs were characterized on a filtered OTU table so that the total count of each OTU across the samples is higher than 1000 (i.e., 0.07% of the total counts), which was merged with the ARG dataset using the sample Id. We then computed the maximal information coefficient index using the application provided with Java SE 1.7.0_02[49] with the percentage of the records necessary to have data in them for both variables before those variables are compared (cv) and the exponent (α) parameters set to 0.6.

To keep only relationships that are statistically significant, we used the same randomization strategy as for the DAPC. Indeed, the empirical null distribution of the maximal information coefficient was computed using 1000 random permutations of the relative abundances within the variables in order to account for the baseline correlation bound to occur between any two vectors. The maximal value of the null distribution was set as the significant threshold for the real dataset, i.e., the dataset before randomization. The relationships were labeled as linear when the regression coefficient was higher than 0.5. They were labeled as non-linear when the regression coefficient was negative and the nonlinearity coefficient was higher than 0.3 (see Fig S7 for examples). The dataset at 14 days and the dataset at weaning were analyzed separately to avoid relationships due to the impact of age.

4.2 Examples of ARG-OTU relationships (Fig S10)

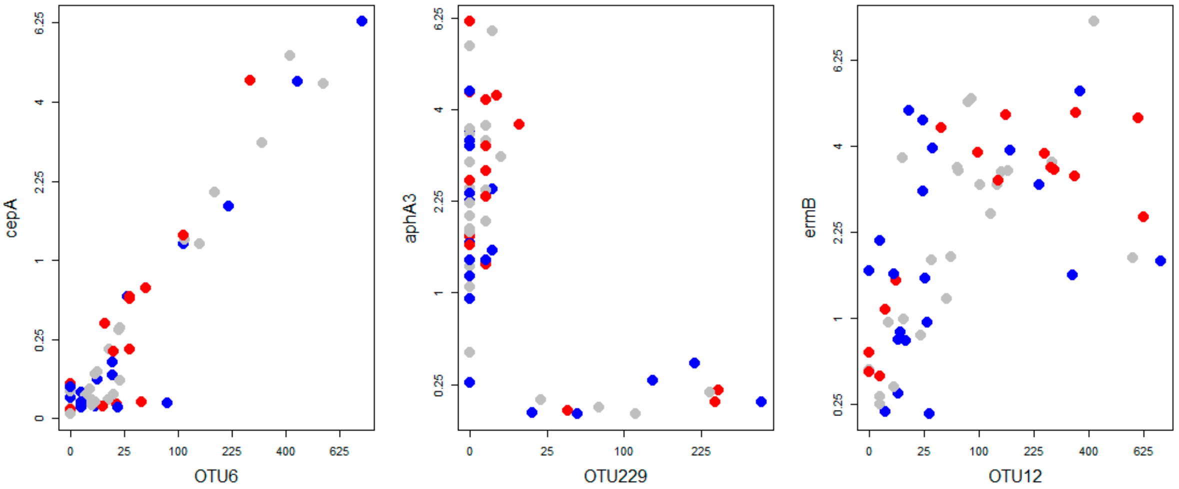

Figure S10: Representative relationships found between OTUs and ARGs for linear (left), co-exclusion (middle), and uncharacterized (right) relationships. The X-axis indicates the number of counts of the OTU and the Y-axis reports the quantification of the antibiotic resistance gene. The points are color-coded for their groups (Red : S1 piglets, blue : S2 piglets, gray : control piglets)

4.3 Distribution of the MIC under real and randomized conditions (Fig S11)

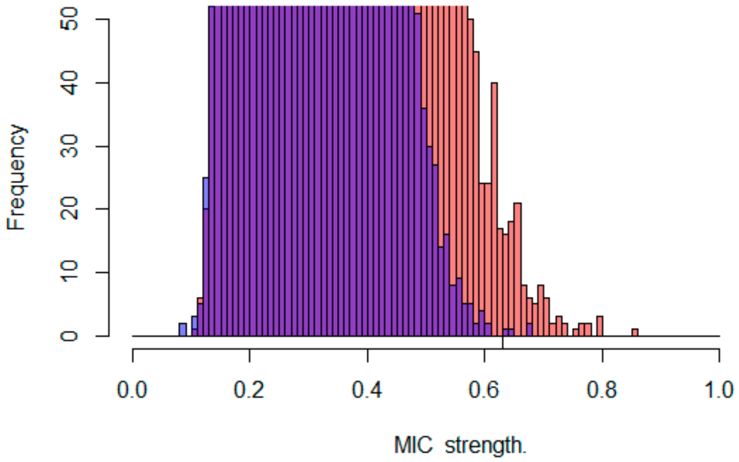

Figure S11 : comparison of the distribution of the maximal information coefficient index in the dataset before and after randomization. The maximal value of the null distribution was set as the significant threshold for the real dataset, i.e. the dataset before randomization (red). The rationale is that the MIC values that were never observed in the randomized dataset (blue) are not an artifact due to the high number of pairwise comparison.

4.4 Network of ARGs and OTUs at weaning (Fig S12)

The statistically significant interactions between ARGs and OTU, i.e. the interactions whose MIC was never observed in the randomized dataset (see List above) are represented below using Gephi 0.9.2.

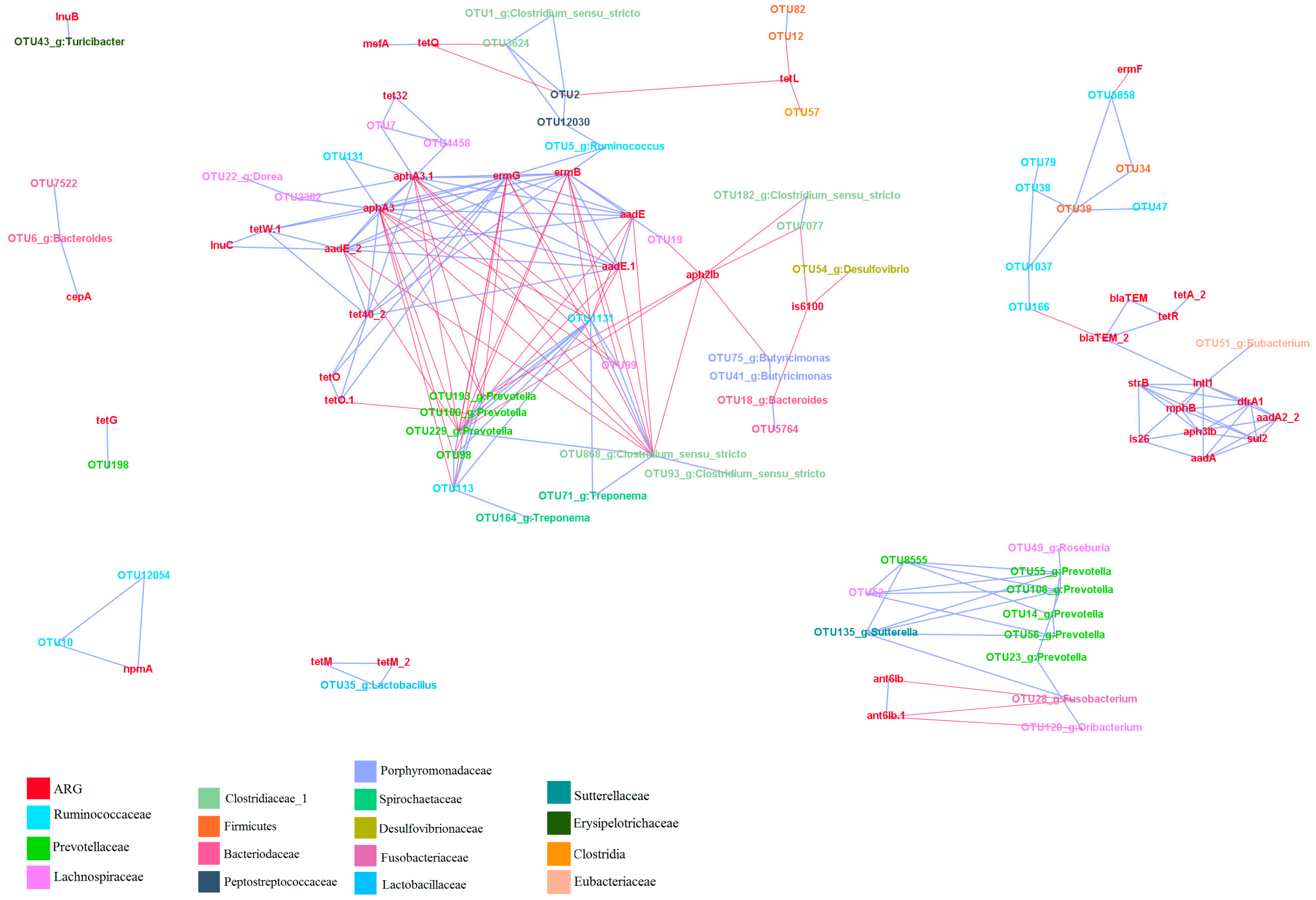

Figure S12 : Network of significant ARGs and OTUs relationships at weaning (excluding relationships between OTUs that were not linked to ARGs at all). The network representation of the non-linear relationships as assessed by the MIC, in which ARGs are indicated by their name in red, OTUs are represented by their number and color-coded by their order. The linear clusters in which at least one ARG is present are represented in blue, and co-exclusive relationships between ARGs and OTUs are represented in red.

5. Technical tables (Table S5)

1.1 Table S5: List of primers used in this paper

Primer in the paper	initial primer name	FWD primer	REV primer	Reference
aac6Im	aac6Im	CGCCCGATGAATGAGGATGA	CACGGTCATCTGTCAGCCAT	This study
aacA.aphD	aacA/aphD	AGAGCCTTGGGAAGATGAAGTTT	TTGATCCATACCATAGACTATCTCATCA	[48]
aadA	aadA	GTTGTGCACGACGACATCATT	GGCTCGAAGATACCTGCAAGAA	[37]
aadA2_2	aadA2_2	CAATGACATTCTTGCGGGTATC	GACCTACCAAGGCAACGCTATG	[37]
aadA9_2	aadA9_2	GGATGCACGCTTGGATGAA	CCTCTAGCGGCCGGAGTATT	[37]
aadE	aadE	TACCTTATTGCCCTTGGAAGAGTTA	GGAACTATGTCCCTTTTAATTCTACAATCT	[48]
aadE.1	aadE	TACCTTATTGCCCTTGGAAGAGTTA	GGAACTATGTCCCTTTTAATTCTACAATCT	[48]
aadE_2	aadE_2	GCTTCGCATGATTTCCTGGC	CCTCCCGGAACAATGCCATA	This study
ant6Ib	ant6-Ib	ACATCCGACAGCACGTTCTT	CGCAGTAATTCCATACGCACA	This study
ant6Ib.1	ant6-Ib	ACATCCGACAGCACGTTCTT	CGCAGTAATTCCATACGCACA	This study
aph2Ib	aph2-Ib	TCAAATCCCTGCGGTAGTGT	CGTCGCTTCATCATATGCAAGG	This study
aphA1	aphA1	TGAACAAGTCTGGAAAGAAATGCA	CCTATTAATTTCCCCTCGTCAAAAA	[37]
aphA3	aphA3	CGGAATTGAAAAAACTGATCGAA	ATACCGGCTGTCCGTCATTT	[37]
aphA3.1	aphA3	CGGAATTGAAAAAACTGATCGAA	ATACCGGCTGTCCGTCATTT	[37]
blaTEM	blaTEM	TTCCTGTTTTTGCTCACCCAG	CTCAAGGATCTTACCGCTGTTG	[49]
blaTEM_2	blaTEM_2	AGCATCTTACGGATGGCATGA	TCCTCCGATCGTTGTCAGAAGT	[37]
cepA	cepA	AGTTGCGCAGAACAGTCCTCTT	TCGTATCTTGCCCGTCGATAAT	[48]
dfrA1	dfrA1	GGAATGGCCCTGATATTCCA	AGTCTTGCGTCCAACCAACAG	[37]
dfrA16	dfrA16	TCGTTTCTGGTGGTGGTGAA	ATGTCTCCATCTCGCTCGAT	This study
ermB	ermB	TAAAGGGCATTTAACGACGAAACT	TTTATACCTCTGTTTGTTAGGGAATTGAA	[48]
ermF	ermF	CAGCTTTGGTTGAACATTTACGAA	AAATTCCTAAAATCACAACCGACAA	[48]
ermG	ermG	ACAAACAGATCACTAGCATTGC	GCGCTATCCACTTTAGGTTTTGG	This study
Intl1	Intl1	TGCCGTGATCGAAATCCAGATCCT	TTTCTGGAAGGCGAGCATCGTTTG	[50]
Intl2	Intl2	TGCTTTTCCCACCCTTACC	GACGGCTACCCTCTGTTATCTC	[51]
is6100	is6100	CCGATCACGGAAAGCTCAAG	GGCTCGCATGACTTCGAATC	[37]
lnuB	lnuB	TGAACATAATCCCCTCGTTTAAAGAT	TAATTGCCCTGTTTCATCGTAAATAA	[48]
lnuC	lnuC	TTTCTTGATGGTGGCTGGGG	TGGGCTCTTGACTGATATCCA	This study
mcr1_2	mcr1_2	TGGCGTTCAGCAGTCATTAT	AGCTTACCCACCGAGTAGAT	[52]
mefA	mefA	CCGTAGCATTGGAACAGCTTTT	AAACGGAGTATAAGAGTGCTGCAA	[37]
MGtetM	MGtetM	GAAGTGCCGCCAAATCCTTT	CTGCATTCCACTTCCCAACG	This study
mphB	mphB	CGCAGCGCTTGATCTTGTAG	TTACTGCATCCATACGCTGCTT	[48]
npmA	npmA	GGTCAGTTTGATCGTGTGCA	TTTCTACCGTCTCCAGTACCCA	This study
str	str	AATGAGTTTTGGAGTGTCTCAACGTA	AATCAAAACCCCTATTAAAGCCAAT	[48]
sul2	sul2	TCATCTGCCAAACTCGTCGTTA	GTCAAAGAACGCCGCAATGT	[37]
tet32	tet32	CCATTACTTCGGACAACGGTAGA	CAATCTCTGTGAGGGCATTTAACA	[48]
tet40_2	tet40_2	TGCTATTGAGGAACGGCAGG	ACAAAGCCCAGGTTGAGCTT	This study
tetA_2	tetA	CTCACCAGCCTGACCTCGAT	CACGTTGTTATAGAAGCCGCATAG	[48]
tetB_2	tetB_2	GCCCAGTGCTGTTGTTGTCAT	TGAAAGCAAACGGCCTAAATACA	[37]
tetG	tetG	TCAACCATTGCCGATTCGA	TGGCCCGGCAATCATG	[37]
tetH	tetH	TTTGGGTCATCTTACCAGCATTAA	TTGCGCATTATCATCGACAGA	[48]
tetL	tetL	ATGGTTGTAGTTGCGCGCTATAT	ATCGCTGGACCGACTCCTT	[37]
tetM	tetM	CATCATAGACACGCCAGGACATAT	CGCCATCTTTTGCAGAAATCA	[48]
tetO	tetO	ATGTGGATACTACAACGCATGAGATT	TGCCTCCACATGATATTTTTCCT	[37]
tetQ	tetQ	CGCCTCAGAAGTAAGTTCATACACTAAG	TCGTTCATGCGGATATTATCAGAAT	[48]
tetR	tetR	CGCGATAGACGCCTTCGA	TCCTGACAACGAGCCTCCTT	[37]
tetT	tetT	CCATATAGAGGTTCCACCAAATCC	TGACCCTATTGGTAGTGGTTCTATTG	[48]
tetW.1	tetW.1	ATGAACATTCCCACCGTTATCTTT	ATATCGGCGGAGAGCTTATCC	[37]
tetX	tetX	AAATTTGTTACCGACACGGAAGTT	CATAGCTGAAAAAATCCAGGACAGTT	[48]
tnpA	tnpA	AATTGATGCGGACGGCTTAA	TCACCAAACTGTTTATGGAGTCGTT	[48]
vanTG	vanTG	CGTGTAGCCGTTCCGTTCTT	CGGCATTACAGGTATATCTGGAAA	[48]

X16SZhu	16S_Zhu	GGGTTGCGCTCGTTGC	ATGGYTGTCGTCAGCTCGTG	48

4.5 Identification of samples

ID_R	shortID	sowshortID	sowlongID	SALLE	LOGE	piglet	sex	lot	color	Sampling_date
CA1601001J2	001J2	722	560722	0	0	1	0	S1	BLEU	J2
CA1601002J2	002J2	722	560722	0	0	2	0	S2	JAUNE	J2
CA1601003J2	003J2	722	560722	0	0	3	0	S1	BLEU	J2
CA1601004J2	004J2	722	560722	0	0	4	0	T	BLANC	J2
CA1601005J2	005J2	722	560722	0	0	5	0	T	BLANC	J2
CA1601006J2	006J2	722	560722	0	0	6	0	S1	BLEU	J2
CA1601007J2	007J2	722	560722	0	0	7	0	T	BLANC	J2
CA1601008J2	008J2	722	560722	0	0	8	0	T	BLANC	J2
CA1601009J2	009J2	722	560722	0	0	9	0	S2	JAUNE	J2
CA1601010J2	010J2	722	560722	0	0	10	0	S2	JAUNE	J2
CA1601011J2	011J2	722	560722	0	0	11	0	S1	BLEU	J2
CA160110J1	10J1	722	560722	0	0	10	0	S2	JAUNE	J1
CA160111J1	11J1	722	560722	0	0	11	0	S1	BLEU	J1
CA160112J1	12J1	722	560722	0	0	12	0	S2	JAUNE	J1
CA160112J2	12J2	722	560722	0	0	14	0	T	BLANC	J2
CA1601138J1	138J1	709	560709	15	2	138	M	S2	JAUNE	J1
CA1601138J2	138J2	709	560709	15	2	139	M	S1	BLEU	J2
CA1601139J1	139J1	709	560709	15	2	139	M	S1	BLEU	J1
CA1601140J1	140J1	709	560709	15	2	140	M	S2	BLANC	J1
CA1601140J2	140J2	709	560709	15	2	140	M	S2	BLANC	J2
CA1601141J1	141J1	709	560709	15	2	141	M	S1	BLEU	J1
CA1601141J2	141J2	709	560709	15	2	141	M	S1	BLEU	J2
CA1601142J1	142J1	709	560709	15	2	142	M	T	BLANC	J1
CA1601142J2	142J2	709	560709	15	2	142	M	T	BLANC	J2
CA1601143J1	143J1	709	560709	15	2	143	M	T	BLANC	J1
CA1601143J2	143J2	709	560709	15	2	143	M	T	BLANC	J2
CA1601144J1	144J1	709	560709	15	2	144	F	T	BLANC	J1
CA1601144J2	144J2	709	560709	15	2	144	F	T	BLANC	J2

CA1601145J2	145J2	709	560709	15	2	146	F	T	BLANC	J2
CA1601146J1	146J1	709	560709	15	2	146	F	T	BLANC	J1
CA1601148J1	148J1	709	560709	15	2	148	F	S1	BLEU	J1
CA1601148J2	148J2	709	560709	15	2	148	F	S1	BLEU	J2
CA1601149J1	149J1	709	560709	15	2	149	F	S2	JAUNE	J1
CA1601149J2	149J2	709	560709	15	2	149	F	S2	JAUNE	J2
CA160114J1	14J1	722	560722	0	0	14	0	T	BLANC	J1
CA160114J2	14J2	722	560722	0	0	14	0	T	BLANC	J2
CA1601150J1	150J1	709	560709	15	2	150	F	S1	BLEU	J1
CA1601150J2	150J2	709	560709	15	2	150	F	S1	BLEU	J2
CA1601151J1	151J1	709	560709	15	2	151	F	T	BLANC	J1
CA1601151J2	151J2	709	560709	15	2	151	F	T	BLANC	J2
CA1601152J1	152J1	709	560709	15	2	152	F	S2	JAUNE	J1
CA1601152J2	152J2	709	560709	15	2	152	F	S2	JAUNE	J2
CA1601197J1	197J1	512	230512	15	12	197	M	T	BLANC	J1
CA1601197J2	197J2	512	230512	15	12	197	M	T	BLANC	J2
CA1601198J1	198J1	512	230512	15	12	198	M	T	BLANC	J1
CA1601199J1	199J1	710	560710	15	11	199	M	S1	BLEU	J1
CA1601199J2	199J2	710	560710	15	11	199	M	S1	BLEU	J2
CA16011J1	1J1	722	560722	0	0	1	0	S1	BLEU	J1
CA1601200J2	200J2	710	560710	15	11	200	M	S2	JAUNE	J2
CA1601201J1	201J1	710	560710	15	11	201	M	S1	BLEU	J1
CA1601201J2	201J2	710	560710	15	11	201	M	S1	BLEU	J2
CA1601202J1	202J1	710	560710	15	11	202	M	S2	JAUNE	J1
CA1601202J2	202J2	710	560710	15	11	202	M	S2	JAUNE	J2
CA1601204J1	204J1	710	560710	15	11	204	M	T	BLANC	J1
CA1601204J2	204J2	710	560710	15	11	204	M	T	BLANC	J2
CA1601205J1	205J1	710	560710	15	11	205	M	S1	BLEU	J1
CA1601205J2	205J2	710	560710	15	11	205	M	S1	BLEU	J2
CA1601206J1	206J1	710	560710	15	11	206	F	T	BLANC	J1
CA1601206J2	206J2	710	560710	15	11	206	F	T	BLANC	J2
CA1601207J1	207J1	710	560710	15	11	207	F	S1	BLEU	J1
CA1601207J2	207J2	710	560710	15	11	207	F	S1	BLEU	J2

CA1601208J1	208J1	710	560710	15	11	208	F	S2	JAUNE	J1	
CA1601208J2	208J2	710	560710	15	11	208	F	S2	JAUNE	J2	
CA1601209J2	209J2	710	560710	15	11	209	F	T	BLANC	J2	
CA1601210J1	210J1	710	560710	15	11	210	F	T	BLANC	J1	
CA1601210J2	210J2	710	560710	15	11	210	F	T	BLANC	J2	
CA1601211J1	211J1	710	560710	15	11	211	F	T	BLANC	J1	
CA1601211J2	211J2	710	560710	15	11	211	F	T	BLANC	J2	
CA1601212J1	212J1	710	560710	15	11	212	F	S2	JAUNE	J1	
CA1601212J2	212J2	710	560710	15	11	212	F	S2	JAUNE	J2	
CA1601213J1	213J1	512	230512	15	12	213	M	S1	BLEU	J1	
CA1601213J2	213J2	512	230512	15	12	213	M	S1	BLEU	J2	
CA1601214J1	214J1	512	230512	15	12	214	M	S2	JAUNE	J1	
CA1601214J2	214J2	512	230512	15	12	214	M	S2	JAUNE	J2	
CA1601215J1	215J1				sow512			sow512			J1
CA1601216J1	216J1	512	230512	15	12	216	M	S1	BLEU	J1	
CA1601216J2	216J2	512	230512	15	12	216	M	S1	BLEU	J2	
CA1601217J1	217J1	512	230512	15	12	217	M	S2	JAUNE	J1	
CA1601217J2	217J2	512	230512	15	12	217	M	S2	JAUNE	J2	
CA1601218J1	218J1	512	230512	15	12	218	F	T	BLANC	J1	
CA1601218J2	218J2	512	230512	15	12	218	F	T	BLANC	J2	
CA1601219J1	219J1	512	230512	15	12	219	F	S1	BLEU	J1	
CA1601219J2	219J2	512	230512	15	12	219	F	S1	BLEU	J2	
CA1601220J1	220J1	512	230512	15	12	220	F	T	BLANC	J1	
CA1601220J2	220J2	512	230512	15	12	220	F	T	BLANC	J2	
CA1601221J1	221J1	512	230512	15	12	221	F	T	BLANC	J1	
CA1601221J2	221J2	512	230512	15	12	221	F	T	BLANC	J2	
CA1601222J1	222J1	512	230512	15	12	222	F	T	BLANC	J1	
CA1601222J2	222J2	512	230512	15	12	222	F	T	BLANC	J2	
CA1601223J1	223J1	512	230512	15	12	223	F	S2	JAUNE	J1	
CA1601223J2	223J2	512	230512	15	12	223	F	S2	JAUNE	J2	
CA16012J1	2J1	722	560722	0	0	2	0	S2	JAUNE	J1	
CA16013J1	3J1	722	560722	0	0	3	0	S1	BLEU	J1	
CA16014J1	4J1	722	560722	0	0	4	0	T	BLANC	J1	

CA1601512J0	512J0				sow512			sow512			J0
CA1601512J2	512J2				sow512			sow512			J2
CA16015J1	5J1	722	560722	0	0	5	0	T	BLANC	J1	
CA16016J1	6J1	722	560722	0	0	6	0	S1	BLEU	J1	
CA1601709J0	709J0				sow709			sow709			J0
CA1601709J1	709J1				sow709			sow709			J1
CA1601709J2	709J2				sow709			sow709			J2
CA1601710J0	710J0				sow710			sow710			J0
CA1601710J1	710J1				sow710			sow710			J1
CA1601710J2	710J2				sow710			sow710			J2
CA1601722J0	722J0				sow722			sow722			J0
CA1601722J1	722J1				sow722			sow722			J1
CA1601722J2	722J2				sow722			sow722			J2
CA16017J1	7J1	722	560722	0	0	7	0	T	BLANC	J1	
CA16018J1	8J1	722	560722	0	0	8	0	T	BLANC	J1	
CA16019J1	9J1	722	560722	0	0	9	0	S2	JAUNE	J1	
CA1601D206	D206		0		Donor206						
CA1601D268	D268		0		Donor268						

4.6 OTU table

The sequences used to produce the data below were submitted to NCBI's SRA archive[with accession number SRP124929.

#OTU ID	CA1601001J2_AACGCA_L001_R	CA1601002J2_TTCGAG_L001_R
CA1601003J2_AAGCTA_L001_R	CA1601004J2_AGTTTG_L001_R
CA1601005J2_TCCCCA_L001_R	CA1601006J2_AACTAG_L001_R
CA1601007J2_GTTCGC_L001_R	CA1601008J2_TGCCTT_L001_R
CA1601009J2_ATAAGA_L001_R	CA1601010J2_CACACT_L001_R
CA1601011J2_ACAGTT_L001_R	CA160110J1_GTCCTA_L001_R
CA160111J1_ACGGTA_L001_R	CA160112J1_ACACCG_L001_R
CA160112J2_AAACAA_L001_R	CA1601138J1_CAGTCT_L001_R
CA1601138J2_CGAGAA_L001_R	CA1601139J1_TTAAAT_L001_R
CA1601140J1_AATTGC_L001_R	CA1601140J2_CCGGGT_L001_R
CA1601141J1_ACTCGA_L001_R	CA1601141J2_GTCATT_L001_R
CA1601142J1_GTTACC_L001_R	CA1601142J2_CAGGAC_L001_R

CA1601143J1_CAGATG_L001_R	CA1601143J2_TAAGAC_L001_R
CA1601144J1_TCGCGC_L001_R	CA1601144J2_AGTTGA_L001_R
CA1601145J2_GTTAGT_L001_R	CA1601146J1_TAACTT_L001_R
CA1601148J1_CTGTAA_L001_R	CA1601148J2_CCAGCC_L001_R
CA1601149J1_CCATTG_L001_R	CA1601149J2_AGCGGT_L001_R
CA160114J1_AGTATT_L001_R	CA160114J2_AGGGAT_L001_R
CA1601150J1_TAGGCT_L001_R	CA1601150J2_GCTCAA_L001_R
CA1601151J1_TTCTTG_L001_R	CA1601151J2_TCCATT_L001_R
CA1601152J1_CCGACC_L001_R	CA1601152J2_ACCTCA_L001_R
CA1601197J1_TTCAAC_L001_R	CA1601197J2_CATGTT_L001_R
CA1601198J1_ACGAAT_L001_R	CA1601199J1_CAGAGC_L001_R
CA1601199J2_GGTATA_L001_R	CA16011J1_GGAGGT_L001_R
CA1601200J2_GTGCTG_L001_R	CA1601201J1_TCTATG_L001_R
CA1601201J2_CCCTCC_L001_R	CA1601202J1_GGACGG_L001_R
CA1601202J2_GAGAAC_L001_R	CA1601204J1_GAGTTG_L001_R
CA1601204J2_TGGTGT_L001_R	CA1601205J1_CGCACA_L001_R
CA1601205J2_AGAGGG_L001_R	CA1601206J1_AATTCT_L001_R
CA1601206J2_ATGAAC_L001_R	CA1601207J1_GGAAGA_L001_R
CA1601207J2_TACCTG_L001_R	CA1601208J1_CTAGGA_L001_R
CA1601208J2_CTAGAG_L001_R	CA1601209J2_GAGATA_L001_R
CA1601210J1_TGACCT_L001_R	CA1601210J2_ATGCTT_L001_R
CA1601211J1_CAGTTC_L001_R	CA1601211J2_GCATAC_L001_R
CA1601212J1_TGCAGT_L001_R	CA1601212J2_CCCAAA_L001_R
CA1601213J1_AATGAA_L001_R	CA1601213J2_CTACGC_L001_R
CA1601214J1_TGCCAG_L001_R	CA1601214J2_CTCTAC_L001_R
CA1601215J1_CACTGT_L001_R	CA1601216J1_GTGCCA_L001_R
CA1601216J2_ACTACT_L001_R	CA1601217J1_AAATGT_L001_R
CA1601217J2_ACCGTG_L001_R	CA1601218J1_GTGACG_L001_R
CA1601218J2_CCTCTT_L001_R	CA1601219J1_GGTCCG_L001_R
CA1601219J2_GTAGAA_L001_R	CA1601220J1_AACTTA_L001_R
CA1601220J2_TGGGTA_L001_R	CA1601221J1_TGTGAG_L001_R
CA1601221J2_CTGAGG_L001_R	CA1601222J1_CAACGA_L001_R
CA1601222J2_TCATCA_L001_R	CA1601223J1_AAGGTG_L001_R

CA1601223J2_CTTGCA_L001_R	CA16012J1_GCATGG_L001_R

CA16013J1_CTGCAC_L001_R	CA16014J1_TTTCCG_L001_R	CA1601512J0_CAATGC_L001_R

CA1601512J2_GATGCT_L001_R	CA16015J1_GACTCG_L001_R

CA16016J1_AATGGT_L001_R	CA1601709J0_GCAATA_L001_R

CA1601709J1_TTGCCC_L001_R	CA1601709J2_AGCCCT_L001_R

CA1601710J0_CAATTA_L001_R	CA1601710J1_TTAGCT_L001_R

CA1601710J2_CCCGAG_L001_R	CA1601722J0_TGTTCG_L001_R

CA1601722J1_GGATTC_L001_R	CA1601722J2_TGGATT_L001_R

CA16017J1_GTAACA_L001_R	CA16018J1_GAGAGG_L001_R

CA16019J1_CAGGCG_L001_R	CA1601D206_CTACCG_L001_R

CA1601D268_CCAACA_L001_R

OTU6	609	389	40	24	778	148	190	565	35	26	1038	1	260	230

709	543	6	1439	3953	39	414	0	3185	27	603	4	152	14

7	1732	129	2	1848	35	778	909	92	1	3270	2	2994	0

5	155	1141	4	1	1889	59	3	8	3	0	1	262	4

306	5	17	0	2	3	60	0	2	10	2	0	5	0

4	20	414	20	1180	1388	1	970	151	1506	6	525	17	84

5	1303	31	179	446	6	9	75	605	282	0	5	204	107

0	20	0	0	4	0	20	103	25	40	1247	103	0	0

OTU2	708	320	193	941	840	1133	982	458	248	1311	795	1544	689	565

1136	96	23	250	272	932	141	36	4	49	78	549	276	446

15	193	24	41	219	162	859	1537	49	115	57	1451	357	1188

207	17	120	65	770	529	1431	17	12	224	36	14	1039	2

1204	53	1163	44	29	11	65	25	54	25	43	1027	77	40

354	689	190	330	326	154	300	472	1124	63	159	163	707	750

226	153	450	790	1500	388	1612	295	211	476	404	865	119	300

94	89	364	69	57	78	495	267	439	620	323	70	695	301

OTU11	1500	32	13	9	254	54	314	462	33	81	196	250	337	26

247	1322	18	455	665	10	74	3	1117	46	1378	7	232	5

15	681	997	7	1131	124	250	973	1146	10	1656	6	628	0

74	6	258	2	0	909	6	4	6	7	12	1	54	5

14	3	6	1	17	3	33	24	9	19	5	1	4	1

7	85	783	0	749	247	3	605	30	829	12	297	6	8

	1	654	16	32	419	10	12	104	237	777	5	6	523	627
	10	26	12	5	5	12	3	49	25	184	482	845	8	7
OTU110		38	8	1	6	0	42	76	3	81	0	0	0	0
	0	67	0	13	0	0	50	0	0	0	3	0	98	2
	40	9	0	0	38	0	3	0	1	0	4	0	51	0
	17	0	5	102	10	27	0	46	6	12	7	4	0	6
	1	23	9	19	0	0	2	5	10	0	0	0	40	3
	2	1	2	2	77	7	0	10	2	1	1	16	16	45
	0	8	1	89	2	2	1	8	0	0	0	0	1	0
	0	0	0	0	0	0	0	1	0	0	5	0	2	0
	0													
OTU111		41	15	1	0	36	98	155	37	18	0	0	0	0
	0	12	1	0	0	0	0	0	0	0	0	0	10	0
	0	0	0	0	0	0	1	0	24	0	0	0	0	0
	0	0	0	6	13	4	0	29	3	0	2	0	0	2
	0	0	0	45	3	4	0	1	23	0	5	1	24	0
	1	0	10	9	34	1	0	3	0	0	0	41	0	1
	15	27	0	0	0	197	5	27	0	0	0	0	0	1
	0	0	0	0	0	0	0	0	0	0	0	0	1	0
	0													
OTU68	521	26	2	0	0	0	99	82	0	0	2	0	15	0
	0	0	2	12	11	4	47	0	0	247	0	0	1	0
	0	0	19	3	0	48	0	193	1	5	669	2	1	0
	1	20	44	2	0	43	0	0	2	0	0	0	3	0
	0	0	0	5	2	0	1	0	0	6	2	1	5	4
	0	5	13	0	0	0	0	0	0	4	5	3	0	1
	1	0	11	0	0	0	0	155	12	1	0	0	1	5
	0	1	7	0	0	0	0	17	3	3	132	1	0	0
OTU5	921	662	23	1233	1055	1722	1311	836	489	4	244	1	38	4
	247	6	21	33	43	67	18	8	8	25	59	1013	4159	127
	2	77	227	52	37	210	4	893	1	20	227	476	230	315
	1	10	202	322	1178	38	948	28	36	205	111	0	844	0
	233	68	2464	14	40	85	62	69	37	3	727	1919	0	47

	0	121	202	1809	378	78	53	0	891	984	44	13	791	29
	491	8	949	0	1287	1374	86	29	10	47	0	3	14	215
	1	1	1	0	2	2	2	4	4	24	22	1	0	0
OTU7	1143	1244	1724	5	17	34	1262	897	1211	1889	0	2	2	16
	791	0	12	0	0	2694	0	2	0	3	1	4	0	1258
	1	1	0	5	1	0	0	2	0	1	0	121	0	1374
	0	32	0	0	33	0	101	2	26	6	712	0	0	0
	478	0	231	0	18	0	29	146	0	14	0	91	0	38
	0	10	0	734	0	0	31	0	2212	0	1054	0	1653	0
	128	0	2003	0	3010	0	556	0	1	0	0	15	0	0
	0	0	1	0	0	0	0	0	1	1	0	0	0	0
OTU162		152	33	1	0	0	70	0	11	1	23	0	0	0
	0	8	0	0	0	0	0	0	0	0	0	0	0	2
	0	0	0	0	0	0	0	0	8	0	0	1	1	0
	2	0	0	0	0	0	0	2	0	0	0	0	0	10
	0	11	0	1	0	0	0	0	1	0	1	0	0	0
	0	0	100	0	23	0	0	0	0	63	41	2	0	1
	0	2	0	24	0	2	0	0	0	0	0	0	0	0
	0	0	0	0	0	0	0	0	0	1	0	0	0	0
	0													
OTU6831		2	2	3	2	4	0	0	0	2	5	2	1	0
	3	2	2	0	4	1	0	0	1	1	0	0	3	1
	0	1	2	0	1	0	0	0	1	0	1	3	2	3
	1	0	2	1	2	0	0	0	0	4	0	0	1	0
	1	0	0	0	2	1	0	0	1	0	1	2	3	0
	2	1	1	1	6	1	0	4	1	0	1	5	0	2
	1	2	2	1	0	8	1	1	1	0	2	0	1	1
	0	0	2	1	2	0	0	3	0	0	1	0	1	2
	3													
OTU13	437	76	162	127	58	80	75	485	296	287	30	211	39	30
	133	117	1	152	9	272	12	8	18	8	90	121	177	63
	3	51	87	10	190	14	3	23	1462	1078	0	689	79	315
	224	3	529	195	120	62	34	5	140	219	9	28	20	5

	166	6	421	68	33	33	234	23	2	7	1917	71	1	13
	620	43	45	315	1309	360	14	297	92	248	313	521	115	46
	125	15	101	77	66	946	507	2	174	9	390	386	109	31
	193	21	724	571	191	48	238	456	395	225	126	389	97	440
OTU47	64	66	491	98	107	118	166	304	285	295	8	296	8	7
	191	62	74	45	22	552	302	25	0	13	18	189	22	86
	25	26	35	45	12	83	151	24	73	44	3	168	32	126
	247	5	93	85	133	9	53	5	23	254	22	26	31	2
	123	56	294	22	6	32	8	43	17	3	13	160	25	23
	47	111	88	377	6	14	60	286	63	12	92	41	102	48
	94	54	183	62	37	21	148	0	265	128	65	36	84	56
	67	26	60	30	42	26	91	22	48	53	72	132	142	69
OTU887		31	18	29	20	67	32	16	33	46	62	22	21	9
	24	100	35	19	58	24	13	28	17	26	12	22	18	21
	12	12	58	33	17	24	10	26	12	25	19	32	67	28
	26	24	38	27	30	16	10	9	44	90	5	18	9	10
	21	14	31	32	15	22	15	26	30	22	28	18	33	11
	80	42	32	13	68	15	14	76	15	15	10	130	13	20
	33	22	11	11	18	46	10	25	13	19	33	20	22	8
	8	21	28	21	30	23	16	23	18	23	13	19	11	27
	125													
OTU981		468	285	374	45	4	345	262	179	138	64	17	67	10
	32	61	106	1	31	18	39	15	1	140	85	260	5	32
	31	1	142	42	2	544	16	77	198	14	12	50	22	3
	159	596	4	116	0	120	31	202	3	0	7	1	0	318
	2	360	1	96	2	4	5	0	0	12	5	1	15	58
	4	0	378	408	122	290	13	0	103	205	1	6	153	3
	188	32	8	316	169	9	8	8	8	73	14	0	8	96
	8	0	1	1	0	0	0	703	14	29	46	83	161	2
	0													
OTU34	95	10	102	234	183	61	228	48	66	96	54	234	50	69
	182	221	1	50	87	46	26	0	60	5	3	11	9	19
	0	40	29	8	19	0	206	63	273	0	0	12	7	112

	325	1	49	2	3	14	6	0	0	2	0	10	62	0
	149	0	26	1	1	4	2	1	1	1	1	40	1	1
	15	95	86	78	58	25	4	88	78	176	3	199	15	38
	6	27	38	328	23	23	12	11	544	355	41	27	18	19
	37	39	16	11	1	5	45	36	54	336	230	293	21	18
OTU18	329	181	107	71	7	145	791	284	106	26	13	60	97	83
	301	70	704	1458	42	18	270	931	1161	2427	1208	49	91	11
	1129	71	67	1441	991	332	30	1066	545	311	616	18	16	10
	21	1105	206	217	41	634	21	398	311	105	402	582	711	344
	218	1004	50	282	99	484	247	79	677	435	252	13	418	325
	1	603	213	7	314	679	10	161	107	523	5	146	28	53
	395	193	117	185	243	693	8	334	18	13	0	1	9	5
	0	1	19	0	1	0	268	4	3	97	49	177	0	1
OTU12054		47	0	77	186	175	57	0	42	0	2	10	7	4
	2	1	16	0	0	0	0	125	0	3	79	1	66	52
	1	2	2	24	5	5	112	76	38	0	3	11	3	9
	0	6	16	23	51	50	18	18	8	5	11	4	0	0
	0	10	2	93	25	1	24	6	12	2	0	178	3	22
	13	0	4	41	0	4	24	0	10	0	3	4	58	35
	0	6	76	3	186	1	21	0	11	9	32	0	0	24
	77	0	0	3	0	1	0	0	0	0	0	9	15	0
	0													
OTU849		27	17	30	24	38	26	18	29	42	43	33	15	10
	19	47	21	13	53	39	8	23	15	26	12	19	12	18
	13	7	41	27	14	22	8	19	10	17	13	26	46	22
	22	21	33	23	14	21	9	9	25	37	10	10	9	11
	17	20	35	22	15	21	12	28	47	16	34	9	31	14
	42	19	30	8	58	15	9	53	18	23	11	83	9	26
	19	18	8	13	16	55	19	24	11	14	25	14	20	11
	6	21	19	15	22	32	13	27	7	22	11	7	11	13
	68													
OTU21	801	739	213	10	129	553	193	45	180	173	5	11	2	0
	843	2	25	8	0	269	6	123	4	22	28	661	35	45

	77	2	238	504	0	245	1	25	5	371	0	350	0	22
	0	4	3	56	253	10	465	9	83	33	25	5	165	6
	97	192	653	10	58	42	48	95	1	38	165	229	5	66
	7	892	187	22	5	93	76	4	55	149	207	3	46	32
	602	0	14	3	90	158	1273	0	1	0	2	2	11	0
	2	58	33	13	24	82	1	15	22	0	4	3	15	4
OTU10	671	248	929	1637	1955	688	216	917	384	159	106	186	69	134
	406	409	60	3	274	89	1211	38	6	865	13	547	513	116
	48	84	304	184	69	1101	740	448	25	345	95	119	239	425
	695	143	540	522	488	248	319	69	142	138	72	52	199	25
	276	26	735	258	16	208	96	104	47	6	1487	154	236	173
	121	310	405	57	629	289	49	1359	111	94	58	1371	379	22
	77	754	985	1764	78	640	92	604	155	296	91	197	290	759
	130	47	91	82	62	43	153	110	164	675	297	306	125	53
OTU45	146	374	132	55	179	143	6	18	107	25	39	247	364	198
	56	236	184	111	74	45	381	142	53	239	68	54	387	10
	218	4	74	202	252	665	142	10	113	7	46	145	207	21
	66	99	105	318	50	650	143	188	114	285	9	288	38	133
	39	144	96	210	66	168	149	58	138	47	710	9	292	141
	290	83	103	35	61	295	183	414	59	670	37	230	10	495
	45	152	43	282	5	207	20	44	468	198	0	45	16	230
	0	53	62	76	63	8	0	175	193	217	667	326	342	121
OTU126		1	14	12	77	9	1	4	0	31	12	0	5	0
	1	1	4	0	4	1	2	24	0	0	4	33	95	0
	24	0	0	0	0	0	1	0	1	0	0	0	13	0
	27	65	0	6	1	1	0	1	0	33	0	2	48	0
	0	12	1	33	0	0	2	15	3	0	0	0	2	0
	1	10	10	24	9	5	1	0	24	5	0	4	35	13
	96	0	0	105	21	2	67	4	0	0	4	37	27	0
	55	29	15	23	15	4	42	14	5	18	3	0	112	7
	16													
OTU79	44	0	51	1	176	99	60	17	82	35	1	4	1	3
	33	0	1	0	4	165	4	0	0	1	0	9	0	11

	0	7	0	0	0	4	1	1	0	3	0	163	9	3
	8	2	1	4	28	4	99	0	1	3	1	0	22	0
	120	0	15	2	2	3	2	3	0	1	2	53	0	1
	28	95	1	106	0	0	4	1	65	12	13	2	113	1
	7	1	47	2	31	0	22	6	0	0	13	20	3	1
	29	29	20	21	10	7	23	8	72	6	2	0	12	19
OTU1	958	353	408	2200	677	1252	616	399	150	873	741	2117	312	154
	1055	734	35	1211	44	711	348	77	40	182	159	166	227	357
	9	39	113	64	982	169	1369	3426	274	109	519	576	0	465
	411	25	208	350	1437	807	2776	17	17	946	97	74	6717	38
	3779	287	964	195	22	56	59	45	141	13	300	605	149	26
	617	91	277	276	569	144	81	694	934	80	119	469	585	765
	67	259	395	1214	926	198	800	147	848	1251	879	1203	675	1720
	125	29	418	63	12	61	654	430	657	1192	1296	344	932	249
OTU65	84	64	0	81	54	9	0	105	88	33	15	4	1	3
	58	147	65	1	3	123	228	11	1	219	116	10	0	22
	23	74	47	391	36	148	33	44	0	39	0	12	17	22
	39	24	57	115	17	245	3	0	20	6	0	8	12	0
	1	2	33	45	5	141	5	7	52	2	9	4	49	12
	12	18	77	3	30	20	3	85	0	181	7	21	2	23
	9	2	30	63	7	76	15	56	2	92	3	34	0	39
	10	43	54	7	20	5	18	17	47	71	103	139	26	34
OTU115		3	2	12	6	17	3	2	11	19	3	2	5	4
	9	1	4	1	8	5	4	61	0	3	3	1	3	7
	5	0	1	5	3	0	6	18	0	0	4	2	2	3
	3	269	0	8	6	2	11	9	0	0	28	0	1	57
	0	14	2	9	4	0	0	2	0	2	0	28	7	1
	0	1	19	36	5	7	3	1	40	0	9	2	49	2
	75	7	36	42	83	4	31	2	0	90	30	1	8	0
	41	1	2	2	1	3	2	8	5	5	16	19	9	13
	0													
OTU143		44	0	0	0	0	1	0	0	0	0	0	0	0
	0	0	0	0	0	0	0	6	1825	0	3	0	0	0

	0	0	0	0	72	0	0	19	0	0	41	0	0	0
	0	0	0	0	26	0	1	0	0	79	12	0	1	6
	0	0	34	1	161	52	0	14	5	44	17	0	6	57
	471	0	1	1	0	0	2	11	0	0	0	0	0	0
	0	39	0	0	0	1	0	0	0	0	0	0	0	0
	23	0	0	0	0	0	0	0	0	0	0	0	10	0
	0													
OTU8	40	24	6	116	67	2	39	11	5	22	2338	282	1943	3429
	94	252	6	601	889	449	258	5	50	17	249	60	90	1
	0	1660	145	3	1324	4	1247	605	141	3	629	8	3817	2
	819	23	445	52	34	1347	80	8	0	158	8	40	349	24
	76	81	130	39	1	37	114	3	41	1	102	30	33	10
	906	16	1512	59	185	1718	10	1302	18	649	14	483	100	2643
	5	1515	13	3380	66	303	54	2530	140	1323	1229	191	511	911
	1571	18	317	150	6	86	38	121	58	885	726	89	16	226
OTU14	104	603	559	149	24	201	13	38	111	80	11	90	17	7
	4	61	353	136	6	256	1197	427	22	298	15	624	245	1469
	2377	10	182	173	157	588	110	0	156	2520	69	198	38	308
	7	869	346	2094	157	240	42	2044	809	2245	2330	3289	0	1700
	3	379	291	1733	1847	1620	880	4142	711	991	519	146	490	865
	15	19	215	107	66	210	2364	740	102	173	2341	779	600	1225
	1998	218	9	256	3	1346	14	5	487	26	0	2	14	163
	0	63	22	11	32	20	17	19	25	547	16	128	35	3
OTU8176		1	0	0	0	0	0	0	0	0	0	0	0	0
	0	0	0	0	0	0	0	0	0	0	0	0	0	0
	0	0	0	0	0	0	0	0	0	0	0	0	0	0
	0	0	0	0	0	0	0	0	0	0	0	0	0	0
	0	0	0	0	0	0	0	0	0	0	0	0	0	0
	0	0	0	0	0	0	0	0	0	0	0	0	0	0
	0	0	0	0	0	0	0	0	0	0	0	0	0	0
	0	0	0	0	0	0	0	0	0	0	0	0	0	0
	0													

OTU134 30 9 3 0 0 4 39 2 8 31 2 0 1
7 90 14 1 51 8 7 0 0 16 1 129 16 53
4 1 0 3 28 32 1 0 3 41 5 83 26 185
24 0 0 19 6 84 4 50 0 5 8 37 0 3
0 19 0 6 2 15 0 5 28 0 2 0 50 0
6 1 3 0 9 18 0 3 1 2 12 0 2 2
1 0 41 60 2 68 0 2 0 0 5 0 0 2
0 1 0 0 1 1 0 0 0 1 0 0 0 0
0
OTU323 20 13 0 0 0 5 0 0 0 0 0 0 0
0 0 1 0 0 0 8 8 1 1 1 49 1 6
4 0 0 0 4 1 4 0 0 4 29 1 3 0
2 0 0 0 0 9 0 7 0 0 0 0 0 3
0 30 2 0 2 1 0 2 2 0 0 4 2 0
1 0 14 0 2 0 0 0 0 5 0 0 0 0
0 16 0 0 0 0 0 1 0 2 0 0 0 0
0 0 0 1 0 0 0 0 0 0 0 0 0 0
0
OTU1009 8 1 0 4 9 10 12 1 3 0 1 0 0
0 5 0 0 0 0 0 0 0 0 0 0 7 8
3 0 0 0 0 0 0 0 25 0 1 0 18 1
9 0 0 1 0 17 0 17 0 0 1 1 0 14
0 7 0 17 0 0 1 0 0 0 0 0 16 0
0 0 0 0 5 0 0 0 0 9 2 0 0 9
0 1 0 8 0 15 7 2 0 0 0 0 0 0
0 0 0 0 0 0 0 0 0 0 0 1 0 0
0
OTU54 99 133 107 29 10 69 171 86 87 119 38 75 121 62
41 247 132 17 173 135 195 120 74 154 82 233 82 118
43 89 62 91 75 86 83 52 76 92 65 76 102 94
115 112 62 94 116 53 91 71 94 105 70 62 80 26
150 41 85 50 36 32 87 59 65 54 89 88 27 84
18 95 42 88 87 47 235 61 123 14 45 73 154 47

	88	73	86	16	74	34	7	35	100	124	17	19	98	142
	15	4	21	3	0	0	7	8	7	81	64	88	21	10
OTU29	190	4	19	13	111	138	271	45	170	136	2	4	0	1
	155	1	43	0	0	211	3	41	0	24	1	84	0	126
	76	0	1	127	0	62	3	37	1	21	1	358	0	181
	0	30	2	73	1174	1	96	15	64	35	93	5	27	2
	37	85	464	16	67	5	129	108	56	29	49	868	22	37
	28	51	4	26	2	1	178	8	156	31	130	4	389	13
	49	6	212	14	77	60	235	47	0	3	29	26	7	0
	84	339	241	106	66	213	87	85	100	16	12	4	82	74
OTU12212		1	0	0	0	0	0	0	0	0	0	0	0	0
	0	0	0	0	0	0	0	0	0	0	0	0	0	0
	0	0	0	0	0	0	0	0	0	0	0	0	0	0
	0	0	0	0	0	0	0	0	0	0	0	0	0	0
	0	0	0	0	0	0	0	0	0	0	0	0	0	0
	0	0	0	0	0	0	0	0	0	0	0	0	0	0
	0	0	0	0	0	0	0	0	0	0	0	0	0	0
	0	0	0	0	0	0	0	0	0	0	0	0	0	0
	0													
OTU1346		9	12	1	3	0	5	6	13	0	1	6	0	19
	3	7	5	2	11	4	0	7	5	3	30	14	0	1
	1	5	0	8	21	10	21	4	4	8	2	0	6	1
	0	0	5	13	9	0	12	0	2	1	3	0	7	1
	4	4	11	0	4	4	2	1	0	6	4	13	0	1
	4	0	1	15	5	41	51	5	4	1	49	0	9	0
	1	8	15	3	7	2	7	0	11	0	0	0	0	2
	6	0	0	0	0	0	0	0	0	0	9	1	8	0
	0													
OTU215		10	21	1	2	1	4	2	11	1	0	5	8	26
	2	6	1	7	11	3	1	7	0	3	35	15	1	0
	2	8	0	3	32	13	35	2	9	3	4	2	2	1
	0	1	6	13	7	0	9	0	2	4	2	0	5	4
	9	3	9	0	7	3	4	5	0	15	4	19	0	2

	13	0	0	21	2	47	72	13	16	0	63	0	5	0
	3	9	22	1	12	0	6	0	24	0	0	0	0	2
	2	0	0	0	0	0	0	0	0	0	9	7	6	0
	0													
OTU11031		1	0	0	0	0	0	0	0	0	0	0	0	0
	0	0	0	0	0	0	0	0	0	0	0	0	0	0
	0	0	0	0	0	0	0	0	0	0	0	0	0	0
	0	0	0	0	0	0	0	0	0	0	0	0	0	0
	0	0	0	0	0	0	0	0	0	0	0	0	0	0
	0	0	0	0	0	0	0	0	0	0	0	0	0	0
	0	0	0	0	0	0	0	0	0	0	0	0	0	0
	0	0	0	0	0	0	0	0	0	0	0	0	0	0
	0													
OTU19	460	266	460	519	163	876	275	688	44	416	40	1	4	1
	275	36	29	209	18	468	158	44	6	32	20	765	1206	145
	57	14	641	199	113	59	7	136	184	23	10	44	0	116
	9	27	271	113	830	1	41	103	267	430	87	15	138	0
	176	67	339	23	47	58	89	56	24	66	51	324	32	37
	7	331	23	15	14	104	204	40	62	592	66	22	640	797
	73	35	45	58	98	336	1280	5	5	48	5	1	32	133
	12	45	15	25	40	11	14	18	9	114	25	33	8	1
OTU25	219	925	195	397	649	761	10	198	67	52	35	273	6	117
	327	8	118	1	8	86	154	11	6	30	73	239	3	13
	27	373	489	1	134	102	835	144	337	176	130	85	579	16
	52	30	390	2	34	655	51	15	6	282	18	9	71	10
	7	2	143	29	18	36	10	12	4	13	64	34	183	16
	48	210	848	4	6	220	50	823	100	145	11	411	15	38
	40	1040	144	225	13	744	33	359	295	440	28	52	4	1365
	57	27	21	21	19	9	53	81	54	556	556	1041	27	36
OTU409		2	1	3	0	3	8	5	2	4	1	0	5	1
	9	0	3	3	0	1	5	2	14	0	15	1	11	4
	2	0	0	0	0	3	13	24	5	0	50	0	2	1
	3	0	0	0	8	16	4	2	0	2	5	1	0	5

	2	0	2	3	3	4	0	1	4	2	3	14	2	13
	2	8	1	10	0	0	1	7	0	9	2	1	3	1
	9	5	10	0	0	1	4	3	4	8	5	5	4	16
	19	5	52	29	34	41	84	14	10	3	9	3	24	7
	22													
OTU22	194	163	454	632	410	299	214	1126	178	186	40	52	57	60
	189	22	28	99	2	93	132	124	10	19	1	174	58	405
	27	33	660	23	41	143	10	207	3	52	4	91	22	57
	19	98	154	186	85	6	1065	77	220	119	81	29	98	28
	472	70	227	30	31	45	214	158	83	224	330	487	53	56
	16	219	509	423	18	4	53	28	788	19	139	43	218	243
	92	115	1015	147	425	563	131	6	81	25	1	14	38	115
	10	135	28	4	53	9	18	4	23	8	11	221	14	36
OTU868		28	89	10	4	6	28	96	3	39	47	2	7	0
	0	16	0	0	2	0	13	1	0	0	5	3	1	7
	0	0	0	0	1	3	1	1	15	0	14	0	17	0
	43	2	4	4	0	5	0	60	1	2	2	1	3	5
	0	13	2	3	0	1	7	0	27	0	4	2	4	0
	1	1174	23	0	13	0	0	20	1	119	32	19	1	13
	4	19	1	67	5	7	1	53	0	0	0	1329	3016	1
	1	149	260	810	226	153	326	1702	668	914	0	1	0	2173
	882													
OTU2302		41	18	43	61	30	6	12	74	16	38	2	2	9
	1	39	1	5	63	0	18	12	23	1	2	0	27	4
	51	7	2	50	2	2	23	2	24	0	8	1	13	1
	21	3	7	17	10	13	0	86	6	31	5	33	5	9
	2	17	10	13	5	6	1	31	20	5	29	29	32	4
	4	2	8	49	10	4	2	3	3	56	3	11	2	32
	33	14	12	65	19	44	51	7	12	3	3	0	0	4
	15	1	4	2	1	4	2	0	1	0	1	2	7	0
	0													
OTU421		17	24	33	33	16	13	8	11	89	24	22	22	75
	9	4	11	79	23	4	50	56	40	0	14	0	32	0

	29	5	0	0	32	13	147	3	3	1	150	4	13	71
	2	1	1	0	41	21	0	5	8	4	32	104	17	0
	10	2	39	5	20	19	0	1	8	44	1	104	32	23
	6	6	0	234	0	18	1	4	0	17	1	6	1	6
	0	10	29	0	0	0	34	8	2	18	18	0	3	54
	46	0	2	37	11	5	12	0	16	6	41	1	10	10
	7													
OTU59	30	9	8	0	78	11	141	8	95	68	4	7	3	2
	57	7	113	8	4	42	7	210	1	71	3	648	39	52
	103	36	11	137	9	156	5	24	1	398	1	390	9	89
	1	135	0	99	15	0	57	83	55	104	181	4	9	35
	13	109	181	22	277	51	134	86	44	57	20	410	49	69
	133	167	55	69	12	2	115	34	77	2	172	18	173	0
	349	10	319	12	39	9	146	2	3	1	7	11	7	0
	19	129	101	35	97	290	20	50	30	15	4	5	33	32
OTU39	239	113	389	251	435	69	203	323	289	218	88	152	141	27
	127	275	15	270	110	339	293	8	10	3	61	39	11	359
	5	20	200	15	67	11	59	80	464	14	44	67	43	302
	142	11	619	2	71	51	77	2	3	45	57	36	15	0
	160	6	172	0	7	14	13	24	2	8	0	210	8	12
	86	699	188	508	274	203	34	142	407	115	90	1033	222	7
	98	92	311	456	232	247	52	9	1292	105	147	80	17	43
	151	49	143	146	27	52	70	33	74	270	34	153	51	81
OTU23	7	162	34	3	4	26	0	6	80	5	0	3	15	0
	4	2	1987	0	0	14	1	1954	0	429	0	20	0	7
	563	0	0	988	0	464	26	0	1	236	0	24	0	13
	0	10	0	480	18	0	5	394	291	205	0	82	2	1260
	2	741	31	1308	1193	162	384	434	284	401	395	22	1444	1991
	0	20	3	12	6	0	1414	2	6	15	29	4	2	0
	33	0	3	1	0	13	74	1	1	0	0	0	0	1
	0	583	74	14	49	117	63	255	127	0	0	27	310	67
OTU1781		2	0	0	6	0	0	0	0	0	0	4	1	13
	5	0	0	1	2	0	1	2	0	0	0	4	0	3

	0	2	29	20	4	10	0	1	0	0	1	0	0	2
	0	0	0	8	1	0	1	0	3	0	2	0	13	0
	6	0	35	0	0	2	12	0	0	37	6	5	2	0
	9	0	0	1	0	0	25	0	0	0	0	0	2	0
	0	0	1	0	1	0	0	1	127	0	3	0	0	36
	2	1	1	1	0	0	0	2	0	0	0	2	7	0
	0													
OTU2850		11	0	4	5	7	4	5	7	13	50	0	44	6
	13	1	3	1	0	1	0	82	0	0	1	0	0	2
	18	0	0	11	0	39	3	19	2	12	0	0	0	0
	0	0	0	0	0	0	0	0	0	0	0	0	0	9
	0	0	0	1	0	0	0	0	0	2	0	0	1	0
	1	0	0	35	1	1	1	1	6	13	1	2	57	25
	2	1	93	27	4	1	0	0	4	6	58	0	0	52
	19	0	0	0	0	0	0	0	0	0	19	10	6	0
	0													
OTU891		10	3	3	11	1	15	8	5	1	11	3	32	1
	3	16	1	0	1	0	8	1	1	0	0	0	3	0
	8	0	0	0	0	1	1	15	71	0	5	0	30	0
	27	1	0	1	0	28	6	65	0	0	4	0	0	74
	0	45	1	20	0	1	0	0	0	1	0	0	11	0
	0	12	8	3	2	5	0	1	3	13	1	1	2	7
	10	0	1	2	12	11	1	20	1	3	2	6	18	0
	8	0	0	12	0	0	0	17	10	9	8	5	1	7
	0													
OTU825		27	23	35	28	64	26	29	43	43	47	38	24	21
	25	58	45	12	73	26	17	32	26	35	18	23	25	30
	18	10	41	28	25	34	13	28	13	24	13	33	64	25
	34	32	31	23	21	17	16	15	43	78	20	19	14	17
	19	27	31	26	13	31	12	19	47	23	32	24	23	8
	61	34	23	18	70	17	11	54	23	18	17	107	11	46
	28	25	8	23	20	70	17	34	9	20	29	18	20	16

9 31 34 27 24 37 15 26 14 35 27 12 12 23
89
OTU875 3 1 0 1 1 2 0 0 0 0 0 2 0
0 1 0 0 0 0 0 1 0 0 0 0 2 0
4 0 1 0 0 1 0 5 3 0 0 0 2 1
0 0 0 0 0 0 5 2 0 0 0 0 0 0
0 1 0 1 0 0 0 0 0 0 0 0 0 0
0 0 3 1 0 0 0 0 1 0 1 1 2 0
0 1 1 0 3 0 3 1 1 0 2 0 0 0
3 0 0 0 0 0 0 0 0 0 3 2 2 0
0
OTU3761 11 5 8 3 16 4 7 8 9 7 6 2 5
7 7 4 0 15 6 1 2 5 11 5 3 4 3
9 1 10 11 5 10 3 2 4 2 5 9 16 7
11 10 17 4 6 7 2 6 6 9 3 6 3 2
0 6 4 8 2 11 3 6 7 2 9 2 6 2
12 3 8 1 16 5 4 13 3 4 3 33 2 10
3 8 1 5 1 10 1 10 1 6 4 6 7 2
4 7 4 6 9 7 2 5 1 6 3 2 6 4
12
OTU189 39 6 4 0 0 1 6 1 3 4 10 3 0
21 4 3 0 7 8 0 2 0 9 0 6 2 114
1 0 9 3 0 24 1 0 0 13 0 4 0 0
1 1 0 3 1 0 0 0 0 0 4 0 1 0
0 0 0 2 0 0 2 0 0 0 0 0 0 0
0 3 23 2 8 3 1 1 4 3 2 1 0 1
2 0 0 0 1 1 1 0 39 5 0 1 1 0
4 3 0 3 1 1 0 7 0 3 5 0 0 2
0
OTU55 59 39 41 0 2 4 6 0 41 8 1 37 3 4
7 16 364 2 0 44 1 589 3 267 49 438 10 66
880 0 4 153 4 223 13 0 1 318 2 19 0 7
0 245 1 280 16 0 2 485 627 178 9 456 0 261

	4	163	11	628	654	545	563	578	507	510	82	15	251	324
	0	4	9	54	160	0	667	55	13	0	146	1	2	34
	764	1	0	1	0	191	65	1	41	0	0	1	1	1
	1	10	19	9	29	13	6	7	9	52	0	2	16	1
OTU1762		4	2	1	1	6	0	3	1	4	4	2	1	0
	0	9	3	0	6	4	1	2	1	2	1	1	0	3
	2	1	2	1	2	1	1	0	2	2	0	1	3	1
	3	5	4	2	1	2	0	0	5	5	3	1	0	0
	1	0	4	0	0	2	2	1	4	3	4	6	5	3
	5	1	0	1	2	2	0	6	0	2	0	5	0	2
	1	2	0	1	1	3	1	1	0	1	0	5	1	0
	2	3	2	2	4	2	0	2	1	2	0	0	0	2
	3													
OTU957		10	18	15	17	33	24	11	29	16	36	15	18	15
	13	16	18	6	37	21	10	14	15	18	10	15	13	16
	16	4	34	25	16	24	5	25	11	14	6	35	51	16
	24	18	36	15	9	8	6	5	31	72	14	7	9	12
	8	17	18	17	4	17	2	12	20	14	33	20	21	11
	22	10	12	7	42	14	7	46	14	19	5	98	6	17
	15	10	8	12	5	26	8	26	9	14	25	7	17	12
	5	22	17	13	12	28	7	20	4	26	9	7	4	12
	39													
OTU3102		4	3	9	2	9	2	4	5	7	9	0	5	2
	3	12	2	1	5	1	2	2	6	7	3	3	0	2
	0	2	5	4	1	4	3	2	0	4	1	2	10	3
	2	3	1	1	2	2	2	0	6	8	0	2	1	1
	2	2	6	5	3	6	4	3	4	2	3	3	5	2
	10	2	3	1	8	5	2	7	5	3	2	17	4	4
	3	2	0	4	2	7	0	3	1	5	4	2	4	1
	1	5	4	1	1	3	0	2	1	4	2	2	0	3
	9													
OTU15	207	186	386	48	121	631	36	340	29	34	388	810	1432	588
	377	393	168	372	326	13	454	51	574	58	1000	27	1330	4

4	143	692	1512	1414	117	577	362	1185	43	454	5	163	33
1023	124	386	270	60	623	21	25	120	109	3	9	313	13
72	156	200	131	30	33	44	11	121	192	1266	27	305	618
1	31	1213	28	292	434	38	145	4	21	46	262	39	341
165	172	40	401	19	156	1	323	747	408	38	0	1119	304
4	7	8	20	6	0	0	31	3	751	304	1057	0	83
OTU145	19	9	36	11	9	17	23	12	4	13	12	2	15
10	11	3	4	3	11	33	17	1	6	4	25	0	9
10	0	1	1	2	6	2	1	1	28	2	1	0	11
10	1	3	12	7	4	6	9	0	1	3	0	3	1
1	4	2	13	1	1	2	2	2	3	0	4	3	2
1	4	13	6	8	16	9	1	4	13	17	2	7	2
8	4	1	3	8	4	3	0	15	11	6	0	4	4
7	3	1	9	5	0	1	9	2	7	11	5	2	6
1													
OTU125	19	26	3	4	2	5	12	0	1	1	16	6	12
4	57	16	1	136	48	1	21	0	18	8	15	0	32
0	0	8	36	0	106	0	17	5	6	1	177	0	1
0	1	0	2	0	0	6	4	1	0	0	0	0	3
0	0	2	0	0	0	0	1	0	0	1	0	1	2
0	0	9	30	0	2	144	0	41	0	95	0	11	0
11	0	23	3	136	2	4	1	5	0	1	0	0	26
24	0	0	1	2	2	1	1	0	1	8	3	20	0
0													
OTU11301	1	0	0	0	0	0	1	0	0	0	0	0	0
0	0	0	0	0	0	0	0	0	0	0	0	1	0
0	0	0	0	0	0	0	0	0	0	0	0	0	0
0	0	0	0	0	0	0	0	0	0	0	0	0	0
0	0	0	0	0	0	0	0	0	0	0	0	0	0
0	0	0	0	0	0	0	0	0	0	0	0	0	0
0	0	0	0	1	0	0	0	0	0	0	0	0	0
0	0	0	0	0	1	0	0	0	0	0	0	0	0
0													

OTU291 4 7 13 0 0 1 3 1 13 1 0 0 0
0 0 0 0 0 0 3 0 0 0 0 2 1 0
3 0 0 0 0 0 0 0 0 0 13 0 7 0
1 1 0 1 0 2 0 0 13 2 0 5 0 0
0 2 0 1 0 2 0 2 0 0 0 0 10 0
0 51 1 0 1 0 0 2 0 0 3 6 0 4
2 4 0 3 1 1 1 4 0 0 0 35 11 0
0 51 22 59 53 35 72 44 33 14 0 0 1 75
30
OTU586 1 0 1 0 0 0 0 0 0 1 0 0 0
0 0 0 0 0 0 0 0 0 0 0 0 0 0
0 0 0 0 0 0 0 0 0 0 0 0 0 0
0 0 0 0 0 1 0 0 0 0 0 0 0 0
0 2 0 1 0 0 0 0 0 0 0 0 1 0
0 7 0 0 0 0 0 0 0 0 0 0 0 0
0 0 0 0 0 0 0 0 0 0 0 1 4 0
0 4 0 0 0 0 1 1 0 2 0 0 0 0
5
OTU52 23 38 1 1 17 12 44 49 15 7 1 0 0 1
52 0 203 0 0 6 11 364 0 4 0 46 7 44
531 1 0 36 0 41 8 8 0 73 0 61 0 20
0 157 0 0 9 0 122 1636 369 28 285 619 2 526
4 6 6 71 984 21 877 690 27 1773 12 40 18 569
1 23 1 1 0 0 335 1 20 0 79 7 71 0
302 0 10 5 1 0 16 0 0 0 3 3 0 2
1 3 3 0 2 0 4 1 2 2 1 1 1 6
OTU72 3 19 7 13 14 12 4 0 2 1 192 9 184 76
39 3 0 9 45 2 3 0 174 2 63 2 7 4
0 73 8 3 93 17 73 1 87 39 170 0 12 1
128 2 1 5 2 87 4 0 0 6 0 0 4 0
13 20 2 3 0 1 15 1 10 0 29 0 16 6
0 11 1 1 0 6 0 10 0 0 0 10 0 27

4	0	1	0	0	0	2	107	33	63	1	0	125	26
0	0	1	0	0	0	0	2	2	8	25	19	0	0
OTU1824	10	5	13	10	16	8	11	15	13	8	15	3	1
4	10	12	8	14	7	6	6	7	8	5	8	2	6
15	7	13	13	3	8	2	12	4	6	6	13	21	8
9	10	17	9	4	3	3	2	12	29	3	4	3	0
9	4	9	11	1	6	4	4	8	6	14	11	11	0
12	4	8	5	11	5	7	13	4	9	2	28	2	3
12	5	5	4	1	16	3	14	3	5	15	1	6	8
2	14	4	8	7	13	2	9	1	13	7	6	3	8
19													
OTU14829	2	0	0	2	3	2	0	2	1	6	1	2	0
0	0	0	0	3	0	1	1	0	0	0	0	1	0
3	0	0	0	0	0	0	2	4	2	0	0	1	0
5	1	0	0	0	0	0	0	0	0	0	1	0	2
0	4	0	1	0	0	0	0	0	0	0	0	0	0
0	0	0	0	0	0	1	0	4	3	0	0	7	2
0	0	2	0	3	1	0	0	0	9	2	0	0	0
2	0	0	0	1	0	1	0	1	1	6	2	1	0
0													
OTU127	35	11	10	153	26	6	12	11	6	4	0	10	31
7	32	49	2	2	26	6	7	0	2	23	0	0	23
9	3	1	0	12	0	5	0	17	56	9	121	8	23
3	4	10	4	14	4	4	1	0	5	26	0	1	7
2	1	5	7	1	15	2	2	12	13	15	24	4	1
0	10	10	0	3	64	0	24	1	5	0	8	25	5
0	13	0	12	0	0	83	1	4	1	0	0	4	0
8	0	0	8	1	1	0	4	1	14	1	7	31	4
0													
OTU4142	12	3	13	4	22	11	6	15	16	20	9	10	9
9	32	14	5	22	7	4	7	7	8	3	9	8	9
9	8	14	7	4	9	6	13	8	7	11	15	22	10
13	8	17	10	5	8	3	5	14	18	6	4	4	7

	10	4	7	5	3	11	4	8	16	7	11	8	14	6
	32	18	9	7	37	5	6	22	7	9	3	35	6	14
	18	7	2	3	4	19	7	9	2	7	6	11	13	6
	7	16	4	8	7	10	2	13	7	12	12	3	3	12
	34													
OTU20	129	1795	465	32	369	484	742	138	23	7	115	206	135	263
	452	91	28	234	22	3	76	3	41	73	1169	12	40	33
	4	290	162	61	90	5	46	74	100	43	1035	11	116	1
	13	11	136	29	17	98	205	47	6	7	1	5	99	4
	39	25	8	6	10	14	19	8	12	8	14	220	5	4
	3	394	171	19	312	255	0	51	12	1060	1	35	16	397
	32	136	22	25	48	133	38	56	258	90	8	2	258	129
	6	5	5	14	6	2	1	20	5	347	244	65	2	0
OTU120		4	0	0	0	0	1	0	1	6	1	0	0	5
	0	0	0	330	0	0	1	0	39	0	4	0	0	0
	1	63	0	0	42	0	163	1	14	1	83	0	7	0
	0	0	2	0	109	4	0	0	97	40	31	0	1	5
	104	0	17	1	65	68	50	34	12	39	92	18	0	228
	16	0	0	0	0	0	0	15	0	0	1	0	0	0
	1	0	0	0	0	0	0	6	1	0	0	0	0	0
	0	0	22	40	0	26	31	6	9	24	0	0	0	34
	0													
OTU12	318	354	948	120	199	495	366	554	355	466	37	680	182	366
	272	179	31	193	109	446	476	2	184	75	388	74	204	215
	4	64	138	213	399	781	269	101	577	39	1	103	395	111
	704	26	68	162	29	2	57	8	0	375	197	5	13	1
	32	25	191	0	18	338	13	41	154	12	561	111	1	4
	5	448	515	821	774	115	6	1602	369	32	825	193	16	144
	64	343	87	176	173	641	199	80	83	101	3	6	93	488
	3	2	9	0	4	0	0	43	11	296	467	431	10	2
OTU332		3	11	3	2	1	5	3	0	1	2	2	2	0
	4	0	0	1	0	2	1	0	1	0	2	0	1	1
	0	0	2	0	3	2	2	0	1	0	6	0	0	3

	0	1	0	4	5	2	2	1	0	1	2	0	1	2
	0	2	0	2	0	0	1	0	0	0	0	0	1	1
	1	4	0	2	2	1	0	0	3	2	3	0	0	0
	2	1	3	0	1	0	1	2	10	3	1	7	2	3
	3	5	3	12	8	6	3	10	5	9	0	1	0	2
	3													
OTU48	62	46	63	59	25	107	39	86	48	38	8	126	13	141
	97	9	65	2	0	27	1	70	0	55	0	44	44	11
	40	0	0	72	44	62	52	58	0	59	13	37	25	52
	0	54	1	41	53	51	117	26	13	25	11	23	62	25
	26	63	33	23	30	40	21	9	35	22	111	23	63	32
	0	389	0	9	1	0	60	16	68	1	39	1	89	19
	50	0	36	0	65	32	35	5	32	61	0	0	68	36
	0	0	5	0	2	4	0	6	12	15	56	69	4	0
OTU901		29	15	29	25	46	20	19	35	40	37	36	24	16
	14	36	38	15	43	20	11	18	13	43	15	17	17	15
	20	14	57	32	13	25	8	21	10	21	25	34	48	30
	37	26	29	20	18	15	12	14	40	71	10	15	7	16
	14	15	20	35	16	22	7	25	35	18	42	21	29	10
	39	25	34	17	59	11	10	43	9	14	10	91	18	29
	20	13	11	16	18	40	13	30	11	13	29	11	19	11
	10	29	21	14	23	32	10	26	10	19	6	13	14	21
	56													
OTU11027		1	0	0	0	0	0	0	0	0	0	0	0	0
	0	0	0	0	0	0	0	0	0	0	0	0	0	0
	0	0	0	0	0	0	0	0	0	0	0	0	0	0
	0	0	0	0	0	0	0	0	0	0	0	0	0	0
	0	0	0	0	0	0	0	0	0	0	0	0	0	0
	0	0	0	0	0	0	0	0	0	0	0	0	0	0
	0	0	0	0	0	0	0	0	0	0	0	0	0	0
	0	0	0	0	0	0	0	0	0	0	0	0	0	0
	0													

OTU150	5	10	2	3	4	7	3	11	6	7	0	3	0
0	6	44	14	2	14	34	15	2	5	5	28	0	2
12	2	0	7	18	22	7	0	5	16	0	4	1	11
6	4	45	0	1	0	0	0	0	3	19	4	2	0
0	3	1	0	2	4	0	14	6	0	2	0	2	6
6	0	26	0	17	17	0	16	1	36	0	8	5	6
0	25	2	6	0	1	1	4	1	2	0	0	8	3
0	0	0	1	0	1	1	0	1	3	1	0	8	0
0													
OTU881	22	25	27	18	54	21	25	47	31	48	23	19	20
25	19	32	13	48	19	8	30	27	35	9	14	21	20
14	18	50	35	16	29	9	22	15	27	20	50	64	23
33	17	44	17	15	11	13	13	35	75	12	8	5	13
15	18	23	28	13	30	7	16	47	21	31	21	24	13
37	24	35	16	67	17	13	44	17	14	10	120	12	37
17	13	8	31	12	34	15	32	7	13	38	8	20	7
18	28	38	22	18	37	9	25	10	37	20	17	9	26
45													
OTU8491	3	0	0	0	0	0	0	0	0	0	0	0	0
0	0	0	0	0	0	0	0	0	0	0	0	0	0
0	0	0	0	0	0	0	0	0	0	0	0	0	0
0	0	0	0	0	0	0	0	0	0	0	0	0	0
0	0	0	0	0	0	0	0	0	0	0	0	0	0
0	0	0	0	0	0	0	0	0	0	0	0	0	0
0	0	0	0	0	0	0	0	0	0	0	0	0	0
0	0	0	0	0	0	0	0	0	0	0	0	0	0
0													
OTU10380	1	0	1	0	0	0	0	0	0	1	0	1	0
0	0	0	0	0	0	0	0	0	0	1	1	1	2
0	0	0	2	1	1	0	1	0	0	1	0	2	1
2	3	0	0	0	0	0	1	0	1	0	0	0	1
0	0	0	1	0	2	1	0	1	0	2	0	1	0
1	1	1	0	2	0	1	0	0	0	0	2	0	0

0 0 0 0 0 0 0 0 0 0 1 0 0 0
0 0 1 0 0 1 0 0 0 0 0 0 0 0
3
OTU1586 2 0 1 3 0 1 1 1 0 3 0 4 0
1 1 2 0 0 0 2 1 0 0 0 2 0 1
0 0 0 0 0 2 1 7 2 0 0 0 0 0
0 1 0 0 0 0 0 0 0 0 3 0 0 0
0 0 0 1 0 0 0 0 0 0 0 0 0 0
0 0 0 1 1 2 1 0 6 3 0 1 2 0
0 1 1 0 2 0 2 2 0 1 0 0 0 0
4 0 0 0 0 0 0 0 0 0 3 3 2 0
0
OTU3624 118 42 55 299 95 160 71 79 19 118 271 326 59
36 151 247 4 294 29 157 128 11 8 25 41 21 93
61 0 12 45 8 320 13 149 248 93 17 146 90 5
67 157 2 58 49 137 210 163 3 3 124 6 11 303
4 227 31 50 24 1 6 14 3 40 1 56 67 19
0 31 15 34 43 95 23 6 159 104 13 21 75 82
243 18 38 27 222 184 49 120 37 157 166 22 84 145
238 2 10 35 5 2 12 29 30 63 134 157 45 56
17
OTU1048 22 14 9 15 34 13 13 28 19 28 13 14 1
7 10 20 5 28 15 6 17 8 18 5 14 6 8
6 7 26 22 6 16 10 25 6 11 9 21 46 12
16 8 19 13 6 11 6 5 30 41 8 9 6 5
5 7 22 17 3 18 2 13 11 10 20 18 9 6
23 10 21 7 42 8 4 37 6 10 5 77 10 10
10 9 4 5 2 20 3 27 3 4 16 10 10 5
4 9 22 12 13 16 5 16 4 11 6 4 6 12
26
OTU75 41 53 1 5 0 12 15 48 1 5 37 5 92 14
10 8 23 50 18 6 27 8 16 140 41 1 6 1
28 0 35 94 41 95 5 26 30 33 8 13 1 0

	0	14	59	29	1	68	0	5	7	10	0	13	13	24
	11	33	0	28	5	8	7	4	34	10	82	0	12	20
	0	2	84	13	177	313	45	34	4	269	3	26	0	4
	34	70	1	45	1	15	2	61	2	6	0	0	6	24
	0	0	1	0	1	0	0	2	0	55	13	23	0	0
OTU883		46	15	29	14	77	29	35	31	45	70	26	15	12
	19	126	26	15	48	24	7	34	24	27	9	16	13	20
	16	8	45	28	22	30	6	18	10	15	15	24	39	24
	43	25	16	22	19	13	5	8	29	86	12	16	15	13
	28	17	33	19	11	31	5	17	21	27	23	23	30	12
	109	26	41	13	54	15	10	49	19	21	6	82	10	16
	20	20	8	15	17	64	11	27	8	14	21	15	17	10
	8	26	22	18	38	25	9	18	6	26	16	8	16	23
	187													
OTU7432		6	7	31	0	0	0	0	0	0	0	0	0	0
	0	0	0	0	0	0	0	1	0	0	0	0	0	0
	0	0	0	0	0	0	0	0	0	0	0	4	0	3
	0	0	0	0	2	0	0	0	0	0	0	0	0	0
	0	0	0	0	0	0	0	0	0	0	0	0	0	0
	0	0	2	0	0	0	0	0	0	0	0	0	0	1
	0	2	0	0	0	2	0	2	0	0	0	0	0	0
	0	0	0	0	0	14	0	0	0	3	0	0	0	0
	0													
OTU3713		0	1	12	9	21	31	35	3	15	97	244	205	94
	67	6	91	0	155	39	57	9	0	3	2	21	1	6
	75	178	8	529	0	169	127	13	134	2	0	62	104	2
	1137	1	204	34	23	2	3	1	15	228	1	45	70	2
	35	190	33	29	2	146	10	11	360	3	1	26	136	12
	17	446	291	28	1	2	28	23	9	6	2	35	2	8
	0	191	13	108	2	0	4	132	69	252	57	10	318	298
	5	12	4	25	10	5	40	45	108	113	363	435	31	83
OTU13753		8	1	0	1	0	0	0	0	3	0	0	0	0
	0	0	0	0	0	0	0	0	0	0	0	0	1	0

0	0	0	0	0	0	0	0	0	0	1	0	3	0
0	0	0	2	0	1	0	0	0	0	0	0	0	0
0	0	0	0	0	0	0	0	0	0	0	0	0	0
0	1	0	0	2	0	0	0	0	3	0	0	0	0
0	0	0	7	0	0	0	0	0	0	0	0	0	0
0	0	1	2	0	1	0	1	0	1	0	0	0	3
0													
OTU104	22	28	8	1	1	1	31	6	17	24	0	0	1
0	11	2	2	5	0	15	0	4	0	2	3	25	0
1	7	0	3	1	0	5	0	4	1	12	0	10	2
3	1	0	35	5	2	1	1	1	15	1	13	0	0
0	0	1	15	1	5	0	13	12	0	3	0	34	2
11	30	11	0	4	4	7	43	2	34	9	54	2	22
1	163	9	27	4	32	0	51	0	1	0	7	15	0
0	43	20	76	10	9	7	85	9	56	0	1	3	63
8													
OTU12030	68	37	19	73	88	135	100	56	23	122	66	122	50
37	124	13	4	18	33	88	13	2	0	3	12	62	26
33	2	16	5	2	18	12	48	154	4	9	5	152	33
100	12	2	12	6	74	36	130	3	0	18	8	4	90
0	113	5	109	5	2	3	7	5	1	1	4	120	3
3	30	73	28	34	36	18	32	48	109	5	10	16	66
84	24	17	47	64	161	50	166	15	20	34	29	73	4
23	12	1	27	4	1	2	65	25	36	33	21	5	77
43													
OTU768	7	2	35	5	11	36	8	19	29	12	1	12	0
0	3	4	0	0	1	32	1	0	0	0	0	1	0
21	0	0	0	1	1	0	2	7	11	1	0	0	0
5	37	1	0	0	5	0	7	0	0	0	2	0	0
0	13	1	9	0	0	0	0	0	0	0	0	8	0
0	11	12	4	3	16	0	0	1	8	8	2	62	4
2	1	0	18	4	7	2	3	0	13	11	10	7	0

0	12	25	18	16	8	3	4	2	9	9	1	4	5
5													
OTU8137	5	5	7	37	4	10	7	51	45	9	403	43	150
29	6	147	3	137	27	0	27	25	18	0	4	1	57
3	6	27	174	14	8	31	4	6	35	2	1	0	7
28	12	16	5	51	115	17	47	12	5	36	0	8	5
19	103	46	39	25	6	19	53	16	19	3	38	51	24
38	7	1	30	2	21	8	10	5	10	8	0	23	4
26	13	12	4	3	4	63	17	28	18	50	1	29	21
24	9	182	0	46	128	36	94	71	28	9	64	9	30
24													
OTU708	1	0	0	0	0	0	0	0	0	0	0	0	0
0	0	0	0	0	0	0	1	0	0	1	0	0	0
0	1	0	0	0	0	0	0	0	0	0	0	0	0
0	0	0	0	2	0	0	1	0	0	0	0	0	0
0	0	1	0	1	0	1	0	0	1	0	0	0	0
0	0	0	0	0	0	0	0	0	0	0	0	0	2
0	0	0	0	0	0	0	1	0	0	0	0	1	0
0	0	1	8	0	0	0	1	0	4	0	0	0	9
0													
OTU762	28	33	42	25	75	35	32	39	55	86	27	18	22
15	108	35	19	61	29	12	25	20	35	11	28	17	26
17	21	65	36	17	39	14	26	20	30	18	25	79	41
39	38	48	21	30	10	5	14	48	62	17	20	14	9
20	21	23	39	19	28	18	22	46	20	30	12	38	6
93	40	33	14	85	24	17	61	19	26	10	82	13	31
36	23	15	18	13	68	19	31	7	31	35	22	26	13
12	40	28	19	34	30	10	28	13	25	31	18	6	21
119													
OTU398	12	0	0	0	0	0	0	0	0	0	0	0	0
0	0	0	0	0	0	0	0	0	0	0	0	0	0
0	0	0	0	0	0	0	0	0	0	0	0	0	0
0	0	0	0	0	3	0	0	0	2	0	5	0	0

0	0	0	0	0	13	0	2	1	0	0	0	0	0
2	0	0	0	0	0	0	0	0	0	2	4	0	3
0	30	0	0	4	14	0	6	0	0	0	0	0	0
0	0	0	0	0	0	0	0	0	0	0	0	0	0
0													
OTU15215	2	0	0	0	0	0	0	0	0	0	0	0	0
0	0	0	0	0	0	0	0	0	0	0	0	0	1
0	0	0	0	0	0	0	0	0	0	0	0	0	0
0	0	0	0	0	0	0	0	0	0	0	0	0	0
0	0	0	0	0	0	0	0	0	0	0	0	0	0
0	0	0	0	0	0	0	0	0	0	0	0	0	0
0	0	0	0	0	0	0	0	0	0	0	0	0	0
0	0	0	0	0	0	0	0	0	0	0	0	0	0
0													
OTU451	1	3	0	2	0	1	0	0	1	0	0	5	0
0	1	0	0	0	0	4	0	0	0	0	2	0	0
1	0	0	0	0	0	0	0	1	2	0	0	0	0
0	0	0	0	0	0	0	0	0	0	0	0	0	0
0	0	0	2	0	0	0	0	0	0	0	0	1	0
1	1	1	0	1	0	0	0	4	1	1	0	0	4
2	0	0	0	1	1	0	1	0	1	0	0	0	0
6	0	0	0	0	0	0	0	3	4	0	0	0	0
0													
OTU304	2	4	2	0	1	1	4	0	10	5	0	0	0
0	0	0	0	1	0	1	0	0	0	0	0	0	0
1	0	0	0	0	0	0	0	0	0	1	0	0	0
1	0	0	0	0	0	0	0	0	0	1	0	0	0
0	0	0	2	0	0	0	0	0	0	0	0	1	0
0	3	0	0	2	0	0	0	0	0	1	0	0	0
0	0	0	1	0	0	0	0	0	0	0	10	4	0
0	16	15	14	0	1	6	8	2	13	0	1	0	22
12													

OTU797		22	12	22	9	44	18	20	24	22	41	29	13	10
	9	40	23	8	32	11	13	24	11	14	13	15	4	18
	17	8	37	16	7	24	3	18	7	11	17	28	47	21
	18	30	26	14	8	7	7	8	38	50	4	10	8	4
	13	18	13	16	7	21	3	14	22	12	14	16	23	8
	33	14	19	9	48	3	4	36	8	18	8	76	2	19
	16	14	11	11	7	48	3	24	6	9	21	6	15	7
	8	18	20	12	16	13	4	12	9	15	13	7	4	15
	50													
OTU5645		2	1	1	0	0	2	0	3	0	2	0	0	1
	1	3	3	0	4	0	0	1	1	0	0	0	0	0
	1	1	1	1	2	0	0	1	0	0	2	1	5	0
	2	1	2	2	0	1	1	0	0	5	0	1	1	1
	2	0	0	0	0	0	1	2	1	2	0	0	0	0
	3	3	0	0	0	0	0	3	0	1	0	2	0	1
	2	0	1	0	1	0	0	0	0	2	1	0	1	0
	0	1	2	0	0	2	0	1	2	0	0	1	0	2
	9													
OTU6289		18	23	3	1	2	8	0	21	17	2	0	0	0
	1	0	8	0	3	0	7	5	1	0	6	213	2	0
	2	0	19	3	276	31	17	0	0	1	15	8	54	17
	0	2	1	0	47	4	10	6	1	0	77	0	0	122
	0	0	212	2	1	0	6	0	0	56	1	45	10	48
	1	0	71	0	0	4	0	2	3	8	1	3	1	3
	0	10	44	4	8	0	4	3	2	0	0	0	0	5
	0	0	1	21	0	0	2	0	0	6	0	1	1	0
	1													
OTU928		45	27	30	27	79	37	32	37	64	78	31	30	22
	23	210	25	19	53	24	12	29	21	35	19	27	31	22
	14	10	67	38	16	38	19	21	19	20	31	35	66	36
	49	27	41	28	23	21	11	14	45	88	15	28	13	11
	33	22	28	41	13	35	9	28	28	30	45	16	37	17
	143	37	50	19	84	19	7	71	20	24	18	95	18	39

	39	16	10	23	22	66	13	45	13	18	38	7	20	14
	21	37	30	25	45	44	18	37	17	19	25	10	9	38
	177													
OTU77	47	96	19	158	37	79	7	51	7	5	46	22	85	12
	105	0	10	187	9	9	213	0	1	1	6	3	20	8
	0	1	12	10	0	16	7	11	10	9	4	51	11	111
	0	1	2	6	68	55	10	4	2	11	0	1	80	0
	10	0	24	0	1	0	2	0	1	0	7	16	0	2
	0	43	113	6	3	31	3	2	7	2	12	9	16	8
	23	17	6	1	36	1	14	22	72	89	0	0	59	87
	0	0	1	0	0	0	0	11	0	2	87	0	0	0
OTU92	49	19	34	4	7	6	3	14	1	9	0	111	0	58
	10	4	5	0	15	2	0	17	0	33	0	7	0	0
	16	0	2	14	0	19	8	11	4	22	1	10	30	2
	0	1	0	132	1	0	0	3	13	424	3	0	0	3
	0	2	11	36	25	14	33	43	6	2	15	2	20	9
	1	4	0	11	5	0	4	20	2	0	0	1	2	0
	47	0	0	10	11	66	6	0	76	0	0	0	22	6
	7	0	0	4	4	0	2	1	7	5	11	24	1	0
OTU198		27	41	8	2	1	1	2	0	5	3	0	1	3
	2	0	3	3	8	1	22	1	23	0	17	5	62	4
	453	487	0	4	12	3	4	3	0	181	34	0	7	1
	16	4	0	27	5	22	0	8	17	217	87	260	86	0
	1	51	4	26	6	192	7	104	174	3	6	6	7	0
	13	22	1	20	19	15	3	7	23	1	6	81	38	29
	1	4	28	1	3	4	69	1	0	32	0	0	8	0
	3	3	10	15	13	3	8	3	13	51	12	2	75	6
	23													
OTU4458		113	99	133	1	2	3	117	91	92	171	0	0	0
	2	61	0	0	0	0	253	0	0	0	0	0	0	0
	119	0	0	0	1	0	0	0	0	0	0	0	13	0
	134	0	3	0	0	1	0	4	0	2	1	81	0	1
	0	31	0	23	0	0	0	2	21	0	1	0	12	0

0	0	0	0	74	0	0	4	0	263	0	124	0	166
0	18	0	204	0	343	0	43	0	0	0	0	2	0
0	0	0	0	0	0	0	0	0	0	0	0	0	0
0													
OTU7522	43	43	2	3	90	18	25	84	2	2	118	0	29
0	83	20	1	106	404	4	20	0	293	2	68	0	8
0	1	190	1	0	54	2	110	105	14	0	289	0	302
0	0	12	154	0	0	16	1	1	0	0	1	0	3
1	2	0	2	0	2	0	2	0	1	0	0	3	0
0	0	1	51	3	120	102	0	135	17	169	2	75	2
6	0	141	1	21	51	0	1	3	1	1	0	0	13
0	0	3	0	0	0	0	3	11	2	5	149	4	0
0													
OTU654	3	0	3	2	0	0	1	1	1	1	0	0	1
0	0	0	0	2	0	3	2	0	0	0	0	0	0
0	0	1	3	0	0	0	2	2	0	0	1	0	0
0	1	0	0	0	0	0	1	0	0	0	1	0	0
1	3	0	0	0	0	0	0	0	1	0	0	5	0
0	0	0	0	3	0	0	0	2	1	0	2	2	0
0	0	0	1	0	2	0	0	2	0	0	0	0	5
1	0	0	0	0	0	0	0	0	0	1	0	0	0
0													
OTU514	18	0	0	0	0	2	0	0	0	3	0	17	1
0	3	0	47	0	0	0	0	0	0	0	0	0	0
0	0	0	0	0	11	15	0	0	0	0	1	0	1
15	0	6	0	8	24	0	4	0	1	26	0	0	40
0	6	22	36	1	0	2	14	0	0	0	0	18	3
3	0	2	1	0	0	0	0	19	1	0	0	0	0
0	0	0	48	0	0	21	5	0	0	0	0	0	18
0	0	0	0	2	1	0	0	0	1	0	0	1	0
0													
OTU42 24	16	6	3	1	8	7	6	4	22	4	0	114	16
59	64	81	13	121	15	1	166	219	31	254	1	1	1

	3	32	133	21	133	28	79	1	91	7	83	2	245	22
	35	640	11	0	7	735	3	6	1	14	2	21	7	84
	3	3	6	0	6	0	28	0	5	1	1	0	0	50
	1	24	48	0	124	18	18	4	6	5	1	10	1	9
	45	129	1	2	39	1	1	2	0	226	0	0	12	9
	0	0	9	0	0	0	1	13	6	15	138	16	0	0
OTU7589	2	1	3	2	4	1	2	3	4	1	0	4	0	
	1	3	1	0	0	0	1	3	0	0	3	2	1	1
	1	0	0	0	0	0	16	2	0	0	0	0	0	1
	1	5	0	3	1	0	0	0	0	0	2	0	0	0
	0	0	0	0	0	1	3	0	0	0	0	6	0	0
	0	0	1	3	0	4	0	0	43	1	0	2	5	0
	0	0	6	1	2	0	6	1	0	0	0	0	0	2
	7	0	0	1	0	0	0	0	0	0	4	3	2	0
	0													
OTU900	23	15	20	12	43	19	18	15	24	46	22	14	9	
	16	86	22	13	41	16	10	14	12	33	3	21	9	19
	13	10	45	30	9	13	5	12	8	14	14	24	35	19
	36	14	26	14	15	15	9	3	23	50	14	8	6	4
	22	15	20	23	4	17	10	21	25	21	18	11	11	11
	53	23	25	6	43	2	10	42	10	20	8	49	8	25
	20	8	8	17	12	45	5	29	5	11	13	10	14	8
	7	21	17	15	20	23	8	19	11	23	12	12	6	16
	87													
OTU927	16	9	19	13	35	16	11	15	22	25	17	12	9	
	13	52	24	7	27	9	7	13	11	20	7	15	7	18
	6	6	36	22	8	14	3	8	5	9	8	20	25	12
	19	13	21	7	11	5	1	6	15	40	9	5	10	5
	12	10	25	9	9	15	7	13	23	13	16	16	16	8
	54	15	14	7	51	9	7	35	6	7	7	52	3	13
	11	6	4	11	11	32	3	12	9	17	13	3	10	6
	5	14	12	14	17	19	5	11	11	16	5	5	3	15
	47													

OTU2267		4	0	0	0	0	1	3	0	0	0	0	0	0
	0	2	0	0	0	0	1	1	0	0	0	0	0	0
	0	0	0	0	0	0	4	0	0	0	0	0	4	0
	0	0	1	0	2	10	0	0	0	0	1	5	0	0
	0	0	1	16	0	0	0	0	3	0	0	2	1	0
	2	0	0	0	3	0	0	0	2	2	0	1	0	0
	0	0	0	2	0	0	0	15	0	0	0	0	0	0
	0	0	0	0	0	0	0	0	0	0	0	0	0	0
	0													
OTU73	76	36	49	60	9	23	21	13	189	37	12	100	7	7
	86	176	12	14	35	94	5	23	182	2	114	40	212	78
	44	2	125	8	7	35	5	16	33	33	8	18	15	18
	87	5	135	195	37	39	11	12	32	74	40	8	1	3
	6	46	150	208	64	97	25	101	52	18	68	182	65	13
	31	20	89	147	444	199	22	106	33	24	30	537	133	92
	110	268	132	164	105	86	66	3	53	30	26	57	15	9
	87	50	98	47	40	35	71	24	92	25	31	124	55	62
OTU312		12	0	27	0	0	0	2	0	0	1	0	0	1
	0	1	0	0	0	0	2	0	1	0	0	0	0	0
	107	1	0	0	0	0	0	0	0	0	0	0	0	0
	1	0	1	0	0	0	0	0	2	21	0	38	0	0
	0	1	0	0	0	0	0	3	4	0	2	0	3	0
	1	9	9	0	2	0	0	95	0	10	0	41	1	184
	0	4	0	7	0	32	0	4	0	4	0	0	3	0
	0	1	0	0	0	0	0	1	0	0	0	0	0	0
	0													
OTU53	12	0	4	18	34	20	11	12	72	11	414	35	377	3
	11	82	12	18	0	3	19	0	0	1	9	3	0	1
	6	1	218	3	0	63	68	1	2	0	0	4	0	21
	18	143	1	78	45	207	5	29	42	136	3	57	238	23
	61	28	38	31	48	31	165	50	21	11	6	127	19	143
	2	18	66	74	5	85	7	52	17	1	29	55	101	15

32 41 79 3 9 5 18 892 55 189 0 0 500 56

0 0 2 4 1 1 1 18 0 32 117 51 0 0

OTU11028 1 0 0 0 0 0 0 0 0 0 0 0 0

0 0 0 0 0 0 0 0 0 0 0 0 0 0

0 0 0 0 0 0 0 0 0 0 0 0 0 0

0 0 0 0 0 0 0 0 0 0 0 0 0 0

0 0 0 0 0 0 0 0 0 0 0 0 0 0

0 0 0 0 0 0 0 0 0 0 0 0 0 0

0 0 0 0 0 0 0 0 0 0 0 0 0 0

CTGGAATGCGGATGCTGTGGGAGGAATT

0

OTU526 29 0 50 0 0 0 2 0 0 9 0 0 0

0 0 0 0 0 0 0 0 0 0 0 0 0 0

10 0 0 0 0 0 0 0 0 0 0 0 0 0

3 0 0 0 0 0 0 0 0 0 0 93 0 0

0 0 0 0 0 0 1 10 6 0 2 0 0 0

0 11 16 0 0 0 0 0 0 5 0 79 0 7

0 3 0 5 0 2 0 0 0 19 0 1 11 0

0 4 0 0 0 1 1 3 0 0 1 0 0 0

0

OTU877 31 12 19 13 43 24 18 28 31 58 24 20 16

19 75 31 13 45 16 4 25 15 29 10 18 12 9

10 7 55 26 12 23 4 21 11 20 13 34 45 12

25 29 18 21 20 10 3 12 28 69 10 15 9 5

8 11 30 13 9 22 13 17 34 16 28 9 16 6

37 35 20 9 57 12 11 52 13 11 10 67 13 22

23 12 8 11 14 39 10 24 5 8 24 8 12 7

6 13 22 15 26 23 5 14 7 19 17 12 4 19

77

OTU1885 10 2 6 4 21 6 9 9 11 11 5 5 7

3 42 7 5 16 3 3 8 4 12 2 5 4 5

1 2 15 12 2 4 0 6 5 7 7 5 15 10

7 7 10 7 1 6 2 3 4 19 3 2 2 2

	8	5	6	12	7	7	1	3	9	7	6	9	10	3
	23	9	10	2	16	5	5	18	3	4	5	19	5	5
	3	2	6	1	7	23	4	5	7	4	15	5	8	3
	6	9	9	2	11	7	7	5	3	4	6	6	3	8
	57													
OTU57	59	12	158	6	80	690	51	11	76	46	0	52	0	0
	39	5	1	2	1	4	1	0	0	0	0	0	1	123
	0	0	0	0	0	0	6	6	4	1	0	5	0	29
	38	1	0	0	17	7	129	1	1	1	2	0	74	0
	64	2	35	0	6	0	0	2	2	4	0	24	0	0
	5	410	0	299	2	4	5	1	156	4	11	22	98	3
	6	1	46	5	126	2	79	1	40	1	2	3	1	5
	0	4	3	1	1	1	3	2	5	17	6	1	0	3
OTU12213		1	0	0	0	0	0	0	0	0	0	0	0	0
	0	0	0	0	0	0	0	0	0	0	0	0	0	0
	0	0	0	0	0	0	0	0	0	0	0	0	0	0
	0	0	0	0	0	0	0	0	0	0	0	0	0	0
	0	0	0	0	0	0	0	0	0	0	0	0	0	0
	0	0	0	0	0	0	0	0	0	0	0	0	0	0
	0	0	0	0	0	0	0	0	0	0	0	0	0	0
	0	0	0	0	0	0	0	0	0	0	0	0	0	0
	0													
OTU763		33	16	34	12	49	18	10	23	37	59	18	16	12
	15	111	29	13	50	21	8	17	12	37	6	14	15	26
	14	11	53	34	17	15	11	26	10	13	16	21	55	19
	33	29	33	22	12	14	12	8	36	55	17	9	12	11
	20	13	14	28	19	23	11	16	18	20	22	23	31	16
	78	27	27	16	51	15	7	55	13	18	9	83	11	26
	28	12	14	15	22	41	8	26	9	11	26	14	11	12
	11	36	19	18	14	25	11	25	13	19	16	7	9	21
	106													
OTU2184		25	0	48	6	4	2	46	8	6	2	0	0	0
	0	33	0	4	0	0	21	0	3	0	8	0	5	0

53	2	0	0	0	0	19	0	0	0	3	0	22	0
7	0	10	0	0	0	2	0	1	1	6	1	0	0
0	0	0	10	1	0	0	18	11	0	1	0	21	0
2	0	2	0	2	0	0	10	0	2	0	35	0	31
0	11	0	20	0	14	0	15	0	0	0	0	0	0
0	0	0	4	5	4	1	1	0	0	0	0	0	1
0													
OTU944	16	14	20	17	48	18	22	42	29	43	24	13	8
8	23	26	10	33	23	5	25	14	14	6	15	10	18
8	10	44	28	12	30	8	15	9	19	8	38	62	32
27	19	30	14	11	16	8	16	36	60	11	9	13	9
4	17	27	16	7	21	10	17	29	13	33	16	24	9
33	17	15	11	53	14	9	47	13	19	8	91	3	16
16	15	4	13	10	42	8	18	11	8	22	7	15	8
8	14	18	10	20	30	7	17	5	17	16	8	8	16
38													
OTU708	15	37	76	215	12	18	1	19	3	7	2	3	10
13	0	197	4	2	10	0	48	0	169	0	68	55	10
52	0	0	171	1	288	5	42	1	331	3	36	0	10
0	63	0	163	54	3	53	8	14	23	9	3	14	11
3	32	13	21	2	32	54	7	4	28	75	34	37	34
8	55	0	0	0	0	29	0	14	2	4	0	5	11
19	0	0	1	2	4	10	6	4	2	1	1	19	8
2	5	19	3	4	4	9	1	14	2	5	3	2	1
OTU199	5	2	7	1	7	5	8	3	4	7	0	5	2
0	6	2	0	10	4	5	5	1	0	1	2	1	3
1	0	2	1	2	4	2	3	0	4	2	0	2	5
2	1	0	9	7	3	4	1	0	0	7	1	0	0
0	0	4	2	2	0	9	0	2	5	1	10	0	3
1	4	2	3	2	9	3	0	2	0	4	0	14	0
0	0	2	1	2	0	4	3	2	15	11	0	3	2
20	3	5	5	2	0	0	1	3	10	6	1	5	6
4													

OTU1237		10	6	7	32	15	10	12	7	3	11	32	37	16
	7	17	33	1	43	8	30	19	1	0	0	4	3	8
	9	0	2	6	1	46	2	23	22	28	1	23	13	2
	6	14	0	8	3	26	27	19	0	0	15	0	4	20
	1	26	3	3	5	2	3	5	0	5	0	4	8	1
	0	2	5	3	8	11	0	0	15	14	0	8	8	7
	29	0	10	11	33	18	6	11	9	20	16	1	5	10
	20	0	1	2	0	3	1	4	1	4	14	22	7	9
	0													
OTU44	28	110	1	64	15	0	11	6	75	1	13	74	4	35
	8	82	195	7	16	61	144	222	3	38	9	70	24	179
	0	0	23	179	18	195	103	16	23	57	0	1	29	7
	3	37	2	143	260	36	2	0	0	55	97	1	40	47
	235	210	72	0	0	152	124	103	107	91	0	6	0	39
	0	34	39	1	0	4	2	70	2	46	49	9	2	23
	65	51	2	49	15	62	55	12	1	32	0	0	110	0
	0	1	0	1	1	0	0	8	0	114	66	105	0	0
OTU119		18	30	28	30	23	16	17	44	23	2	25	62	48
	39	4	96	31	3	41	10	129	18	13	36	55	10	21
	42	4	53	76	26	30	24	61	8	115	22	28	11	37
	8	70	7	58	27	25	13	0	1	3	20	12	22	17
	10	32	31	35	13	7	37	27	14	18	10	3	3	3
	5	0	27	92	6	1	1	52	53	1	9	8	6	5
	28	25	16	14	14	3	21	14	3	113	82	0	0	45
	65	0	0	1	0	0	0	0	2	0	78	35	65	0
	0													
OTU828		41	25	31	18	89	30	28	46	36	70	19	31	14
	20	119	29	22	53	23	14	34	29	27	13	30	27	19
	13	8	55	32	23	22	9	26	8	24	20	37	64	31
	51	34	27	30	32	13	10	12	43	111	17	26	16	14
	31	26	32	39	17	45	13	26	38	28	33	33	48	15
	124	46	38	12	77	22	14	67	14	24	13	117	18	19
	18	25	9	23	22	71	11	40	9	13	36	19	20	14

9 49 30 16 28 42 15 31 12 21 29 8 10 33

176

OTU12260 7 7 74 19 15 4 49 34 20 7 0 1 0

0 26 0 0 2 0 6 0 0 0 0 0 0 0

6 1 0 2 0 3 0 0 8 0 1 0 2 0

3 9 0 27 0 3 0 3 0 0 0 0 3 1

0 0 0 9 0 1 0 1 2 0 0 0 7 0

1 0 0 0 151 0 3 8 1 24 1 0 13 28

0 7 1 7 5 3 0 4 0 4 0 0 1 0

0 0 2 0 1 0 0 1 0 0 2 0 2 0

0

OTU13963 1 0 0 0 0 0 0 0 0 0 0 0 0

0 0 0 0 0 0 0 0 0 0 0 0 0 0

0 0 0 0 0 0 0 0 0 0 0 0 0 0

0 0 0 0 0 0 0 0 0 1 0 0 0 0

0 0 0 0 0 0 0 0 0 0 0 0 1 0

0 0 0 0 0 0 0 0 0 0 0 0 0 0

0 0 0 0 0 1 0 0 0 0 0 0 0 0

0 0 0 0 0 0 0 0 0 0 0 0 0 0

0

OTU892 21 19 26 14 53 35 11 27 39 48 25 17 15

22 77 28 8 51 27 5 24 22 23 10 23 15 26

22 12 49 26 14 23 17 20 7 13 21 28 58 26

36 33 31 26 20 16 5 11 30 59 16 10 9 4

14 17 29 15 14 28 8 18 24 17 29 19 31 11

53 27 25 11 55 21 9 40 15 19 15 81 10 21

25 15 10 11 12 54 13 29 6 17 23 14 27 6

10 26 25 17 26 33 7 28 8 13 15 11 12 21

129

OTU1037 49 5 105 48 88 5 124 89 147 65 1 80 0

0 7 0 5 1 26 115 13 0 0 0 0 0 0

71 0 0 2 1 1 1 17 5 2 2 0 55 36

195 41 5 51 0 122 0 5 0 5 1 50 6 0

	0	47	0	32	0	1	1	4	4	0	1	0	19	0
	3	51	36	10	229	0	4	19	38	229	1	78	28	115
	2	10	12	52	12	192	8	35	0	10	21	36	24	16
	2	52	72	70	108	45	74	67	18	60	7	15	1	126
	60													
OTU152		15	0	24	0	1	6	52	0	22	1	0	0	0
	0	13	0	1	19	0	5	2	1	10	0	15	17	1
	40	2	0	0	1	1	0	0	0	1	7	0	3	0
	4	2	12	2	7	39	1	8	22	24	14	22	4	0
	3	32	0	24	2	4	6	8	10	4	0	0	7	1
	0	12	8	0	8	1	0	27	0	4	3	7	3	47
	12	68	1	7	0	25	3	2	0	0	0	0	9	0
	0	8	10	1	17	10	11	12	10	2	0	0	0	0
	0													
OTU13771		2	0	1	0	1	1	0	4	3	0	2	0	2
	1	0	0	0	4	3	0	0	1	1	1	0	1	0
	0	0	1	1	0	2	0	1	0	0	1	1	1	0
	2	0	2	2	0	0	0	0	1	0	0	0	1	0
	0	2	1	3	0	1	1	1	0	3	0	0	1	2
	2	4	0	1	2	1	1	4	0	0	1	5	0	0
	0	1	2	0	1	2	1	2	0	0	3	0	2	1
	0	0	1	0	2	1	0	1	0	0	1	0	1	0
	1													
OTU41	25	21	3	0	0	5	10	26	4	8	48	4	62	48
	10	129	34	269	123	2	221	59	896	106	566	1	92	5
	38	210	56	63	553	34	30	14	361	14	319	2	134	1
	61	8	239	17	1	338	2	3	10	12	8	19	3	14
	21	29	0	23	3	14	21	52	42	18	61	0	51	14
	1	3	46	4	202	151	47	56	6	247	1	152	3	12
	24	290	4	30	7	7	1	78	41	66	0	0	38	20
	0	10	0	0	0	0	0	4	3	76	68	51	0	0
OTU56	27	3	72	27	2	0	0	7	5	1	1	19	3	1
	0	20	24	13	0	4	212	34	10	56	55	2	16	33

	203	1	32	54	92	32	12	1	70	89	35	7	2	4
	0	153	52	304	9	26	0	739	256	72	193	460	0	85
	0	115	2	164	254	354	396	658	136	307	32	7	138	133
	9	1	30	7	2	86	316	104	11	3	116	122	20	75
	485	18	2	41	0	157	0	0	64	4	1	1	3	21
	12	107	6	13	18	29	11	7	11	125	0	35	40	10
OTU33	6	13	74	117	20	20	57	24	28	26	5	149	7	30
	12	14	16	1	1	23	72	246	1	20	2	71	22	39
	143	21	25	8	1	89	83	230	4	203	0	118	0	7
	1	16	54	248	72	6	74	287	34	109	71	48	5	72
	13	162	203	181	29	526	34	147	287	76	267	64	38	22
	0	23	279	19	86	0	57	25	35	5	30	25	74	4
	29	3	374	23	22	572	293	5	68	48	0	1	173	21
	0	0	0	0	1	0	0	9	0	32	160	153	0	0
OTU349		8	2	0	0	3	2	1	3	1	0	3	0	0
	0	0	0	0	0	0	0	3	0	0	0	3	0	2
	0	0	0	1	0	2	0	0	0	0	0	1	0	0
	0	0	1	0	0	0	0	0	6	0	2	0	0	0
	0	3	0	11	0	0	0	3	1	0	5	0	3	0
	1	0	1	0	1	0	0	2	0	1	0	0	0	6
	0	1	0	1	0	0	0	1	0	0	0	0	1	0
	0	0	0	0	0	2	0	0	0	3	0	0	0	0
	0													
OTU230		8	10	11	9	3	5	1	18	3	2	0	2	0
	1	5	7	0	0	0	5	9	0	2	0	8	9	24
	1	0	0	5	0	1	4	3	5	6	0	1	1	0
	3	3	2	0	1	5	0	2	0	0	3	1	0	0
	0	3	2	4	0	0	3	1	0	1	0	2	7	0
	0	3	3	3	7	2	0	0	1	3	0	0	2	7
	7	3	4	15	2	3	3	13	0	2	4	0	0	4
	3	3	0	2	0	2	2	5	1	3	3	3	3	2
	0													

OTU188	4	5	0	1	0	0	0	2	0	5	0	2	0
0	0	3	7	0	0	21	27	18	9	0	0	31	4
12	32	0	9	8	0	30	0	0	7	52	0	19	0
7	2	0	8	3	6	0	4	0	53	9	19	0	5
0	3	0	14	1	58	5	6	11	2	2	7	4	1
2	65	0	11	0	2	0	1	8	0	4	29	13	0
1	31	0	1	1	0	0	3	0	10	0	17	18	1
0	165	180	37	96	161	12	26	53	20	18	5	0	123
73													
OTU879	20	11	19	11	32	24	20	17	23	49	13	17	8
12	33	13	4	39	14	7	10	18	15	14	14	11	13
12	10	42	23	14	30	3	13	15	21	12	25	48	13
21	19	22	19	11	9	4	6	24	51	10	7	9	7
15	9	21	20	12	21	4	11	31	16	24	13	17	14
37	18	29	8	45	13	6	35	13	16	7	81	11	23
20	13	7	15	14	41	17	15	8	10	20	14	12	9
10	22	20	14	22	22	9	17	7	18	9	3	10	16
41													
OTU873	0	0	1	0	0	0	0	0	0	92	0	21	117
0	8	7	19	152	0	12	0	0	0	0	1	31	0
0	462	34	0	4	0	35	0	2	0	20	0	78	0
3	0	8	0	0	2	0	0	0	0	1	1	4	2
1	0	1	0	0	0	2	0	0	0	0	1	2	0
0	0	8	2	0	0	0	0	0	0	0	2	0	3
0	76	0	3	0	1	0	272	1	73	0	0	54	3
0	4	0	0	0	0	0	6	0	0	52	1	0	0
OTU886	25	15	23	11	67	11	18	15	29	53	18	19	14
7	63	22	8	31	18	12	13	14	17	8	19	19	20
14	11	39	18	13	25	13	16	8	17	12	23	48	23
30	24	20	12	14	8	10	6	28	54	14	10	6	5
16	7	17	18	7	21	10	20	21	13	16	17	26	13
66	23	24	6	42	17	11	47	8	14	10	64	13	24
14	9	10	8	12	44	7	17	8	9	20	18	16	8

13	19	13	6	18	20	4	23	14	12	11	8	11	25
91													
OTU10806	12	210	26	1	41	51	83	13	3	1	12	18	7
24	40	10	0	21	4	0	5	0	3	4	105	0	6
1	0	29	11	2	5	0	4	4	7	1	96	2	11
1	0	0	12	2	0	6	17	2	0	2	0	1	8
0	4	0	1	0	0	0	2	0	1	1	0	26	0
0	0	39	15	3	32	20	0	2	0	79	1	3	2
29	2	15	4	2	2	8	1	5	26	7	0	0	14
13	0	0	0	0	0	0	0	4	0	29	19	6	0
0													
OTU11022	1	0	0	0	0	0	0	0	0	0	0	0	0
0	0	0	0	0	0	0	0	0	0	0	0	0	0
0	0	0	0	0	0	0	0	0	0	0	0	0	0
0	0	0	0	0	0	0	0	0	0	0	0	0	0
0	0	0	0	0	0	0	0	0	0	0	0	0	0
0	0	0	0	0	0	0	0	0	0	0	0	0	0
0	0	0	0	0	0	0	0	0	0	0	0	0	0
0	0	0	0	0	0	0	0	0	0	0	0	0	0
0													
OTU913	13	5	19	11	24	11	10	15	16	26	15	8	7
9	10	16	9	30	16	3	13	9	17	2	6	7	14
8	7	25	23	6	18	1	11	4	4	4	21	33	14
9	11	16	8	14	9	5	1	14	26	7	7	4	1
9	9	12	13	5	13	2	11	18	8	14	8	14	7
12	8	11	7	25	6	4	29	9	10	4	65	2	4
7	15	5	7	9	31	3	12	9	3	13	8	8	11
4	13	9	9	10	20	7	15	3	11	6	3	2	14
21													
OTU1548	10	8	11	14	29	12	11	14	18	26	16	8	9
15	10	14	2	23	9	10	11	9	6	6	16	8	9
17	9	28	24	8	15	11	10	1	16	10	30	40	12
7	13	20	8	13	7	5	1	15	44	6	8	3	5

	10	9	15	15	8	15	3	7	19	10	14	13	12	4
	14	6	25	6	40	6	3	21	13	9	9	80	5	10
	6	11	7	5	9	23	7	21	2	9	26	12	9	4
	6	19	17	10	9	22	4	18	7	15	9	3	3	11
	26													
OTU142	2	61	11	55	9	12	11	1	0	3	7	30	30	
	23	21	28	26	27	29	1	6	25	76	8	75	6	4
	3	0	24	22	52	20	3	30	6	57	9	41	1	11
	2	54	12	136	5	1	27	3	18	4	7	2	23	21
	16	6	15	0	20	1	16	5	5	27	3	54	3	29
	13	0	6	120	6	119	97	5	81	4	97	0	77	28
	100	25	136	8	33	4	20	1	7	74	31	0	0	9
	24	0	0	0	0	0	0	0	1	0	53	37	84	0
	0													
OTU11746	75	2	112	87	144	3	17	111	14	5	0	122	0	
	0	6	0	0	0	41	137	29	0	0	1	0	0	0
	46	0	0	4	0	1	0	25	4	0	0	0	22	49
	0	66	0	10	0	14	2	2	1	0	2	42	10	0
	0	20	0	30	0	2	1	1	7	0	3	0	24	0
	5	3	0	28	170	0	2	4	45	70	0	25	24	78
	5	7	8	3	14	20	8	25	1	5	38	7	2	22
	2	19	6	7	8	1	0	4	0	3	13	12	0	7
	3													
OTU770	29	15	26	12	59	29	21	21	31	56	16	10	7	
	14	66	26	10	28	21	7	20	13	31	5	21	13	15
	12	4	39	14	10	18	11	8	6	11	17	29	55	23
	29	22	33	14	10	13	12	9	36	63	15	6	7	6
	12	11	19	19	14	25	10	13	21	14	23	15	29	8
	73	24	18	5	66	9	6	45	8	20	4	90	8	17
	23	12	13	19	11	48	6	24	4	16	23	11	15	7
	7	23	24	17	13	26	8	16	4	22	12	5	9	21
	104													

OTU211	29	22	4	0	0	14	2	1	1	11	0	1	0
0	26	0	0	1	0	10	0	0	0	0	2	30	0
0	0	0	0	0	0	0	0	0	0	2	0	3	0
2	0	0	0	0	6	0	6	0	0	0	0	0	4
0	3	0	40	0	0	0	0	0	0	0	2	15	0
0	8	66	0	0	0	0	0	0	0	0	2	0	0
0	1	0	3	0	0	0	4	0	0	0	3	3	0
0	3	3	1	1	4	0	13	0	14	0	0	0	2
1													
OTU8175	1	0	0	0	0	0	0	0	0	0	0	0	0
0	0	0	0	0	0	0	0	0	0	0	0	0	0
0	0	0	0	0	0	0	0	0	0	0	0	0	0
0	0	0	0	0	0	0	0	0	0	0	0	0	0
0	0	0	0	0	0	0	0	0	0	0	0	0	0
0	0	0	0	0	0	0	0	0	0	0	0	0	0
0	0	0	0	0	0	0	0	0	0	0	0	0	0
0	0	0	0	0	0	0	0	0	0	0	0	0	0
0													
OTU325	1	12	6	1	0	2	5	3	15	0	0	1	0
0	3	0	0	0	0	3	0	0	0	0	0	1	0
8	1	0	5	0	0	0	0	1	0	2	0	4	0
14	4	0	4	1	24	0	7	0	0	2	4	0	0
0	1	0	5	0	0	0	0	3	0	0	0	5	0
0	0	1	0	4	0	0	0	2	24	1	1	0	10
0	0	0	3	0	0	0	0	0	1	2	2	5	0
0	5	7	2	4	2	0	0	2	5	0	2	0	1
0													
OTU6511	2	1	0	0	1	1	0	1	0	0	0	0	0
2	0	0	1	0	1	0	0	0	0	3	0	0	0
0	0	1	0	0	3	0	0	2	0	1	1	2	0
0	0	1	0	0	0	1	1	0	2	2	1	0	0
0	2	1	0	1	0	0	3	0	0	0	1	0	1
4	2	0	0	1	1	0	0	0	0	0	1	0	0

0	0	0	0	1	0	0	0	0	0	0	1	2	0
0	1	0	0	0	0	0	0	0	0	0	1	0	1
1													
OTU7508	3	1	1	0	2	0	0	0	0	1	0	1	0
2	2	1	0	2	2	1	0	1	0	1	0	0	0
0	0	0	1	1	0	2	3	0	0	1	1	1	0
1	0	0	1	1	0	0	0	0	2	0	0	0	0
0	0	3	1	1	0	0	0	0	0	1	0	1	1
1	2	1	0	0	0	0	0	1	1	1	2	0	1
1	0	1	1	1	2	0	1	0	0	1	2	0	0
0	5	0	1	2	2	2	1	0	1	1	0	0	1
5													
OTU8743	2	20	0	17	0	7	5	3	6	0	9	10	27
23	0	35	0	7	20	0	5	0	4	1	5	1	24
0	0	13	60	0	133	0	4	7	11	0	20	0	72
0	0	0	40	1	0	4	0	0	0	1	0	0	8
0	0	5	0	2	0	1	1	0	0	0	4	0	1
0	1	9	7	0	28	73	0	1	0	129	0	8	0
6	1	51	0	13	0	1	4	9	0	4	0	0	8
13	0	0	0	0	0	0	0	1	0	16	6	32	0
0													
OTU279	3	11	0	0	0	0	13	27	6	0	3	0	0
0	0	0	11	0	0	0	0	7	0	3	3	8	7
3	6	0	0	4	1	6	4	0	0	4	2	1	0
1	0	6	0	0	0	1	0	0	2	1	0	0	0
0	0	4	0	2	2	0	3	0	1	2	5	0	3
1	0	5	0	0	0	0	21	0	7	0	0	0	0
0	0	0	0	0	0	0	4	0	0	1	0	0	0
5	0	0	0	0	0	0	0	0	0	0	0	0	0
0													
OTU497	1	0	0	1	1	0	0	0	0	0	1	0	0
0	0	3	3	0	1	0	8	0	0	0	0	0	6
0	1	2	4	0	0	0	0	4	5	0	0	0	3

0	0	0	13	0	2	2	1	2	0	0	1	1	0
3	13	2	1	0	0	0	0	0	2	2	0	0	0
0	0	0	2	2	0	2	0	0	1	0	0	1	0
0	2	1	0	0	1	1	0	6	0	3	0	0	61
3	0	0	0	0	0	0	9	0	3	2	0	0	0
0													
OTU8555	18	18	26	0	4	1	5	1	21	3	0	21	1
0	1	2	112	0	0	18	0	157	0	77	10	169	1
22	231	0	1	50	1	103	2	0	1	54	0	14	0
3	0	67	0	75	5	0	0	140	239	55	3	83	0
57	2	40	3	129	223	149	205	210	107	190	17	10	50
102	0	3	2	25	50	0	276	19	6	0	29	1	0
6	257	0	0	0	0	63	34	0	11	0	0	0	0
0	0	1	2	0	5	5	2	1	2	20	0	1	5
0													
OTU161	22	0	6	15	5	31	4	27	7	12	0	15	0
0	5	9	2	7	0	40	0	0	0	2	0	23	4
15	1	1	0	48	0	7	18	6	1	0	0	20	0
1	0	0	0	10	27	0	48	0	0	8	0	0	6
0	2	1	1	6	0	9	2	0	2	0	5	10	16
0	0	10	3	1	0	0	0	1	11	1	2	21	0
1	1	2	0	1	0	1	0	0	22	3	0	0	2
1	0	0	0	0	0	0	0	0	0	13	0	20	0
0													
OTU835	32	16	23	16	54	30	17	38	35	67	13	24	14
13	147	28	15	44	20	10	17	16	19	14	20	12	14
14	12	59	18	18	24	10	21	10	25	10	28	55	29
39	29	27	15	24	16	4	11	42	72	15	8	7	7
22	15	22	31	13	23	5	24	40	24	21	27	22	17
93	34	22	9	60	15	10	64	15	11	10	66	12	24
26	19	15	24	11	49	17	31	10	26	21	14	25	16
10	37	31	24	36	23	15	28	8	24	30	10	10	24
140													

OTU62	8	104	54	0	3	12	36	6	144	2	0	1	1	1
	4	1	61	0	1	25	1	450	0	60	4	53	5	483
	1164	0	0	69	0	224	12	4	5	635	0	117	1	40
	0	8	1	501	15	1	52	535	276	76	230	10	6	219
	50	37	12	336	586	148	605	321	92	446	58	155	211	180
	61	2	0	35	1	0	151	0	13	22	203	0	30	0
	177	0	6	3	0	3	51	0	2	0	5	6	0	1
	10	540	159	353	890	576	65	434	158	0	16	1	287	152
OTU1942		16	8	8	7	20	7	10	8	15	20	16	17	2
	13	54	23	9	24	13	2	13	4	12	4	6	8	7
	9	5	15	7	2	8	5	5	5	11	14	11	17	9
	16	1	4	14	9	6	6	4	16	36	2	6	4	2
	9	10	6	7	7	3	5	7	10	8	11	6	11	6
	51	13	7	6	22	4	3	20	5	8	8	37	6	9
	13	5	2	3	11	26	1	9	1	10	9	10	6	9
	4	11	8	5	10	14	9	7	5	9	7	1	12	11
	65													
OTU218		2	27	12	0	1	0	5	0	15	3	0	0	0
	0	0	0	0	0	0	1	0	0	0	0	0	0	0
	0	0	0	0	0	0	0	0	0	0	0	0	5	0
	3	0	0	0	0	1	0	0	0	0	0	0	0	0
	0	1	0	5	0	0	0	0	1	0	0	0	2	0
	0	18	0	0	6	0	0	0	0	0	1	1	0	6
	2	1	0	7	0	1	0	6	0	0	0	19	31	0
	0	27	16	42	9	6	12	62	14	21	0	0	0	16
	3													
OTU11023		1	0	0	0	0	0	0	0	0	0	0	0	0
	0	0	0	0	0	0	0	0	0	0	0	0	0	0
	0	0	0	0	0	0	0	0	0	0	0	0	0	0
	0	0	0	0	0	0	0	0	0	0	0	0	0	0
	0	0	0	0	0	0	0	0	0	0	0	0	0	0
	0	0	0	0	0	0	0	0	0	0	0	0	0	0
	0	0	0	0	0	0	0	0	0	0	0	0	0	0

	0	0	0	0	0	0	0	0	0	0	0	0	0	0
	0													
OTU43	93	153	29	295	60	127	65	44	53	92	14	148	10	2
	46	147	14	242	4	136	77	17	10	99	111	46	21	106
	3	0	68	27	168	121	38	78	72	27	358	223	0	332
	35	5	18	24	108	53	215	4	2	99	11	11	67	1
	99	25	31	14	3	12	1	10	17	1	17	32	20	10
	216	215	2	62	21	3	88	17	151	5	62	22	46	26
	26	11	139	15	122	13	191	0	58	50	445	563	115	39
	23	43	183	69	14	61	308	51	180	58	83	24	470	314
OTU953		18	13	19	13	28	15	16	29	16	16	14	9	13
	6	11	19	5	26	10	7	15	10	16	6	13	7	10
	10	7	24	14	8	11	0	13	4	11	11	21	45	9
	14	9	21	13	10	5	4	6	26	39	6	5	4	4
	9	6	8	12	2	13	3	7	30	9	16	8	15	9
	17	9	16	5	31	7	5	28	11	8	8	67	6	9
	12	6	6	5	9	25	2	13	7	3	21	9	7	7
	14	14	23	6	11	13	6	19	3	12	6	4	4	13
	12													
OTU128		31	2	18	19	2	6	13	10	25	4	0	8	1
	3	0	0	13	26	1	2	25	14	0	22	54	12	7
	15	14	0	7	31	2	37	5	38	1	28	6	12	22
	3	22	28	15	20	3	42	4	21	11	4	2	2	27
	6	10	1	1	8	7	3	23	14	0	14	0	3	7
	23	0	24	3	4	19	0	36	35	2	11	0	4	15
	1	28	36	6	7	4	8	2	0	5	8	0	0	1
	0	0	0	0	0	2	0	0	0	3	12	0	1	0
	0													
OTU40	6	129	17	60	25	11	17	9	18	9	14	69	8	16
	13	29	414	27	3	10	129	190	0	427	149	18	2	75
	153	5	1	482	11	152	6	9	102	143	31	27	0	23
	156	23	168	30	7	1	108	6	47	48	45	52	14	48
	22	155	10	163	23	186	83	57	93	11	87	33	136	92

	2	1	65	3	59	68	115	134	37	286	16	86	4	414
	70	192	8	188	7	139	25	15	128	30	22	9	39	21
	1	11	21	0	2	15	0	17	36	204	153	151	11	4
OTU801		38	18	22	17	64	43	36	38	37	43	33	28	17
	19	126	36	15	39	26	18	27	16	29	17	20	17	16
	16	13	51	27	7	31	18	21	18	21	22	27	60	23
	43	30	28	40	11	16	9	10	33	63	25	11	14	13
	23	12	30	33	17	34	9	28	33	22	30	23	27	12
	97	47	38	11	65	22	7	73	16	25	18	86	9	27
	32	17	10	18	27	61	9	35	9	14	28	26	15	11
	11	37	30	10	23	16	14	23	15	17	14	14	10	29
	162													
OTU9	62	78	144	520	80	177	74	574	585	132	4254	516	1859	642
	61	2511	93	1588	540	2	460	292	248	6	90	33	658	45
	66	635	2616	178	81	434	150	84	623	39	47	5	227	396
	170	358	77	683	1123	353	583	149	69	478	16	150	100	312
	1212	723	415	296	212	352	661	370	377	55	353	578	388	503
	64	47	384	99	200	240	98	66	102	171	15	344	48	376
	146	92	86	9	109	799	281	547	243	587	6	481	519	442
	63	2032	8	570	1174	433	747	946	254	128	1183	134	196	128
OTU272		17	81	10	42	0	7	38	22	22	6	0	2	0
	0	7	0	13	0	0	11	4	18	0	9	6	51	0
	36	7	0	0	3	2	16	0	1	7	0	0	5	0
	11	3	4	2	3	1	0	2	24	16	12	0	2	0
	6	20	0	7	10	5	25	21	14	5	7	9	6	8
	9	38	22	13	20	6	18	60	6	3	3	28	13	55
	6	53	8	10	8	25	17	22	0	1	0	8	19	1
	0	24	47	3	39	64	29	15	32	21	8	5	19	11
	10													
OTU12214		1	0	0	0	0	1	0	1	0	1	0	0	0
	0	1	0	0	0	0	0	0	0	0	0	0	0	0
	0	0	0	0	0	0	0	0	0	0	0	0	0	0
	0	0	0	0	0	0	0	0	0	0	0	0	0	0

0	0	0	0	0	0	0	0	0	0	0	1	0	0
0	0	0	3	0	0	0	0	0	0	0	1	0	0
0	0	0	0	0	0	0	0	0	0	0	0	0	0
0	0	0	0	0	0	0	0	0	0	0	0	0	0
0													
OTU1077	23	18	21	24	57	30	21	28	36	56	19	21	10
27	61	22	11	32	20	11	21	14	25	12	16	13	21
16	18	59	17	13	22	8	18	6	26	23	29	55	21
35	29	14	19	19	10	9	9	36	64	12	16	5	8
14	11	20	31	8	25	18	16	30	19	24	17	20	7
56	28	11	12	65	13	12	50	14	18	11	85	12	20
22	17	16	18	19	51	10	26	10	8	25	10	18	10
13	24	24	12	35	20	11	25	9	23	14	8	7	25
89													
OTU931	15	20	17	14	34	22	22	23	25	36	20	13	8
9	33	18	11	37	17	11	11	16	24	7	15	18	14
14	15	40	22	10	13	5	13	12	21	13	23	43	15
14	24	19	21	10	12	6	9	28	52	4	12	8	5
15	11	23	19	13	15	6	9	30	9	21	18	22	10
29	30	16	7	41	21	7	29	16	11	6	63	5	15
22	15	8	9	14	32	8	18	7	10	19	10	24	12
4	22	15	9	16	12	8	17	7	17	18	5	12	9
49													
OTU238	13	10	12	5	2	8	11	15	4	12	2	2	3
3	6	7	0	5	3	14	11	0	0	3	1	2	10
1	0	1	1	0	9	4	5	2	7	0	0	5	3
4	9	0	7	1	3	3	1	0	0	2	0	1	3
0	1	0	7	1	0	0	1	0	1	0	0	1	0
0	6	10	5	5	0	0	2	6	12	9	1	13	0
5	3	6	12	7	2	5	17	0	12	6	0	1	3
0	3	2	4	2	1	0	0	1	9	12	1	4	5
0													

OTU381	1	2	4	3	0	1	0	4	2	0	1	2	0
0	0	1	0	3	0	0	5	0	2	5	0	0	0
0	0	2	1	1	0	3	3	0	8	6	0	1	0
1	2	0	13	2	1	2	0	0	0	2	1	0	1
0	0	1	5	0	1	0	2	2	3	0	6	1	0
1	0	0	1	0	4	4	0	4	1	9	1	7	1
0	1	3	1	7	3	2	0	1	0	7	0	0	0
1	0	0	0	0	0	0	0	0	0	3	2	5	0
0													
OTU7258	4	1	3	6	5	8	2	7	5	3	3	0	2
4	7	2	2	2	5	3	1	5	4	1	1	1	7
9	0	9	2	1	7	4	5	1	1	7	3	11	5
5	2	0	5	2	2	2	0	6	10	3	1	2	1
2	3	0	6	1	3	1	3	4	3	4	5	0	5
4	6	1	2	5	1	1	15	4	4	3	11	2	4
2	3	2	1	4	9	2	3	2	0	7	0	2	4
0	6	6	1	3	3	1	3	4	5	1	0	4	1
11													
OTU2784	37	59	10	33	89	46	170	49	54	97	480	49	65
22	270	2	112	35	43	200	4	10	2	35	6	8	12
0	38	155	0	126	0	288	26	405	0	107	1	11	34
987	0	590	1	111	90	36	2	0	28	0	2	103	0
44	0	54	1	1	1	7	1	2	1	0	19	1	2
65	136	146	71	178	104	4	299	48	425	13	1052	8	196
5	98	42	308	12	29	61	16	250	357	23	27	14	665
11	40	58	14	21	3	17	34	65	210	133	120	11	28
OTU968	14	9	94	0	1	1	0	0	0	0	0	0	0
0	0	0	0	0	1	0	0	0	2	0	0	0	1
0	0	0	0	0	0	0	0	0	0	0	17	0	31
0	0	1	0	18	0	0	0	0	0	0	0	0	0
0	0	0	0	0	0	0	1	0	0	0	0	0	0
0	0	0	0	1	0	0	1	0	0	0	0	0	2
0	9	0	1	0	6	0	4	0	0	0	0	0	0

	0	0	1	0	0	33	0	0	0	1	0	0	0	0
	0													
OTU186		3	3	7	6	0	1	20	1	89	11	0	2	1
	0	15	2	1	3	0	0	1	0	0	0	2	0	1
	2	0	0	3	0	0	1	0	8	4	0	1	8	0
	12	14	2	6	4	18	1	24	1	9	3	6	0	2
	0	8	0	10	4	3	5	1	1	4	1	2	7	1
	1	10	6	1	2	23	1	3	5	46	2	5	2	12
	37	5	0	1	3	6	46	2	0	10	4	17	3	1
	0	3	16	3	34	50	6	28	56	79	4	8	19	128
	0													
OTU177		29	2	0	7	22	2	1	29	1	4	8	28	59
	105	1	4	6	59	135	2	192	69	8	12	9	6	17
	0	6	36	7	25	181	38	4	16	255	1	178	0	16
	0	0	2	1	1	0	53	0	0	0	25	0	1	18
	2	1	5	1	24	0	5	0	0	0	0	5	0	38
	4	0	1	1	2	40	3	5	0	0	0	0	5	1
	1	1	8	1	2	0	1	0	172	1	107	0	0	45
	1	0	0	0	0	0	0	0	0	1	2	11	11	0
	0													
OTU378		2	0	2	0	0	3	5	2	0	3	4	2	1
	1	4	6	0	3	2	0	2	0	0	0	0	1	1
	0	0	2	0	2	0	0	0	1	0	0	0	2	2
	0	2	0	3	0	4	0	1	0	0	0	0	0	0
	0	0	0	2	0	0	0	0	0	0	0	0	5	0
	1	0	1	1	2	3	0	0	0	2	0	1	0	1
	0	2	0	3	2	2	1	1	1	0	2	0	2	0
	1	0	2	0	0	0	0	0	1	0	2	1	0	0
	0													
OTU2382		5	2	2	1	2	1	3	3	1	3	2	0	0
	1	1	0	0	5	1	1	2	1	0	1	2	1	0
	2	1	6	3	0	0	0	2	0	1	2	4	3	0
	1	1	2	1	3	0	0	0	2	4	0	0	1	0

	1	0	0	2	1	0	0	2	2	0	1	2	2	1
	6	1	0	0	4	0	0	3	2	3	1	9	2	1
	0	1	1	0	2	3	0	1	1	0	2	1	0	0
	0	2	1	0	2	2	1	3	1	1	1	2	1	1
	3													
OTU11962		5	1	4	6	8	4	2	3	7	7	5	1	2
	5	19	2	0	4	1	2	1	2	6	3	2	2	2
	2	2	7	2	0	2	1	3	0	4	4	2	13	6
	10	3	1	8	1	1	2	1	2	15	1	3	1	1
	0	5	4	4	4	4	1	0	3	1	2	3	6	3
	16	5	2	3	7	0	2	5	4	5	2	8	3	2
	2	0	0	3	0	7	1	1	2	3	5	2	0	1
	2	7	2	5	3	1	1	2	2	1	7	1	1	2
	36													
OTU82	11	17	16	12	6	18	21	67	18	23	1	17	3	9
	30	26	6	46	3	35	96	1	12	13	46	5	3	14
	3	2	9	13	18	0	14	5	38	2	10	7	11	19
	39	0	80	2	0	6	1	0	0	26	2	1	1	1
	2	9	18	0	0	1	0	0	0	0	1	6	13	1
	9	30	61	18	55	47	4	18	15	33	11	95	2	16
	1	40	17	16	18	20	13	0	7	24	5	9	13	60
	3	0	4	0	0	0	14	3	27	23	21	28	8	2
OTU367		1	0	0	0	0	0	0	0	0	10	1	1	0
	4	0	0	0	1	0	1	0	0	0	12	0	0	0
	0	0	0	0	2	0	0	0	0	0	0	0	0	12
	1	0	0	0	0	0	3	0	0	0	4	1	0	2
	0	0	0	1	0	0	0	0	6	0	0	1	0	0
	0	3	5	0	0	0	16	2	1	7	2	14	3	0
	0	0	0	2	0	1	0	3	2	1	0	0	3	0
	0	2	2	0	0	0	0	0	1	10	0	1	0	2
	0													
OTU13602		1	2	2	0	1	0	1	1	3	0	2	0	0
	0	1	2	0	2	3	0	2	1	0	0	1	0	1

	1	1	1	1	1	2	1	2	0	2	0	1	3	1
	0	0	2	0	0	1	0	1	2	5	0	0	1	1
	0	0	2	0	0	1	1	0	1	1	0	1	3	0
	3	0	0	0	3	0	2	0	0	2	0	3	0	2
	0	0	0	2	0	1	0	1	0	0	0	1	0	2
	0	0	1	3	1	1	0	0	2	2	0	0	0	0
	1													
OTU67	5	7	9	0	4	2	3	2	30	21	50	2	76	26
	1	77	0	1	28	17	323	0	3	4	1	46	4	461
	1	9	2	5	8	0	10	0	21	1	0	13	1	39
	159	1	0	132	37	0	26	0	51	35	3	10	0	0
	101	1	0	3	2	11	11	29	1	1	19	35	0	2
	7	0	3	15	14	0	15	5	23	17	99	3	12	22
	0	0	9	78	1	7	0	4	1	14	0	7	28	0
	0	14	9	6	32	3	10	11	30	8	4	2	18	23
OTU114		17	12	2	0	2	2	2	0	0	1	0	36	0
	1	1	0	0	0	0	9	0	0	0	0	0	0	0
	0	0	0	1	0	0	0	0	0	0	0	0	11	0
	13	0	0	0	15	2	0	11	2	0	0	151	0	0
	0	0	0	19	0	18	4	30	57	1	16	0	3	0
	2	4	3	24	0	1	1	0	1	0	0	9	2	0
	435	0	1	4	2	10	6	0	0	2	8	0	2	0
	0	1	10	4	12	6	3	2	2	9	2	1	0	3
	9													
OTU11529		3	0	1	1	1	2	0	2	1	3	0	3	0
	1	8	2	0	3	0	0	1	0	0	0	1	1	0
	0	0	3	1	2	1	0	1	0	0	1	1	5	0
	1	3	2	2	1	0	0	1	0	1	0	1	0	0
	1	1	2	3	2	1	1	1	2	3	0	2	1	1
	1	1	0	0	0	0	0	1	0	0	0	2	0	0
	3	1	1	1	0	1	0	1	3	0	0	1	0	1
	1	1	2	1	0	1	0	1	1	1	1	1	0	2
	4													

OTU621	1	0	0	2	0	1	1	3	1	0	0	0	0
0	0	1	1	2	0	0	0	0	0	0	0	1	0
0	0	0	0	0	0	0	0	1	0	2	0	1	0
3	0	0	1	0	0	1	0	0	0	0	0	0	0
0	2	0	0	0	0	0	0	0	0	0	0	1	0
0	0	1	0	0	0	1	0	0	0	0	0	0	0
0	2	0	0	2	0	0	4	0	0	1	0	0	0
0	0	0	0	0	0	0	0	0	0	0	0	1	0
0													
OTU14265	1	0	0	0	0	0	0	0	0	0	0	0	0
0	0	0	0	0	0	0	0	0	0	0	0	0	0
0	0	0	0	0	0	0	0	0	0	0	0	0	0
0	0	0	0	0	0	0	0	0	0	0	0	0	0
0	0	0	0	0	0	0	0	0	0	0	0	0	0
0	0	0	0	0	0	0	0	0	0	0	0	0	0
0	0	0	0	0	0	0	0	0	0	0	0	0	0
0	0	0	0	0	0	0	0	0	0	0	0	0	0
0													
OTU3073	0	21	4	13	0	2	34	62	7	0	64	37	547
2	1768	4	9	229	11	799	1	4	328	741	2	1	12
2	6	552	0	175	0	460	1	270	3	2	184	716	4
21	0	4	0	22	35	0	0	0	17	2	0	8	0
4	0	99	0	0	0	1	0	0	1	66	7	0	0
1	26	0	231	1	1	9	7	8	3	12	125	12	1
2	1	0	2	7	4	0	4	29	861	0	0	76	300
0	5	5	0	1	0	0	23	0	177	260	1190	0	0
OTU11020	1	0	0	0	0	0	0	0	0	0	0	0	0
0	0	0	0	0	0	0	0	0	0	0	0	0	0
0	0	0	0	0	0	0	0	0	0	0	0	0	0
0	0	0	0	0	0	0	0	0	0	0	0	0	0
0	0	0	0	0	0	0	0	0	0	0	0	0	0
0	0	0	0	0	0	0	0	0	0	0	0	0	0
0	0	0	0	0	0	0	0	0	0	0	0	0	0

	0	0	0	0	0	0	0	0	0	0	0	0	0	0
	0													
OTU2326		1	3	1	2	0	0	2	0	2	0	0	0	0
	0	0	0	0	0	0	0	0	1	0	0	0	2	0
	0	3	0	0	0	0	1	0	0	0	0	0	0	0
	0	0	1	0	0	0	0	0	2	1	0	0	0	0
	0	0	0	0	1	2	1	1	1	0	0	0	1	0
	2	0	0	0	0	0	0	2	1	0	0	4	0	2
	1	4	0	0	1	5	0	0	0	0	0	0	0	0
	0	3	1	0	1	2	1	0	0	1	0	0	0	0
	0													
OTU76	12	15	2	18	23	2	4	16	10	4	14	6	14	7
	4	0	14	13	28	72	118	4	14	110	7	123	22	30
	8	2	5	48	128	74	12	23	30	126	0	37	0	11
	0	1	0	72	44	24	7	70	82	76	61	5	23	7
	96	0	31	25	24	18	221	2	4	51	40	37	25	9
	3	11	0	0	0	11	96	0	6	2	92	60	19	0
	133	0	4	0	0	0	7	2	5	40	2	13	24	15
	11	2	1	15	19	8	49	48	34	6	2	17	23	4
OTU9437		22	8	22	2	11	16	15	54	34	11	15	5	40
	0	5	1	4	0	0	11	5	0	0	0	0	6	0
	11	1	0	14	1	0	0	1	0	1	3	4	5	0
	23	1	0	12	0	1	0	0	0	0	5	0	0	0
	0	1	2	0	0	1	1	0	0	0	0	0	0	2
	0	2	55	5	17	4	7	1	2	63	4	6	10	4
	0	1	1	8	11	3	2	7	0	1	6	1	1	0
	0	0	4	8	4	1	1	0	2	0	14	4	15	4
	2													
OTU3	19	4	5	23	15	31	60	24	789	2	2	9	25	5
	12	67	58	7	3	23	10	44	2	0	0	6	27	51
	30	49	154	42	0	154	12	10	11	40	1	60	2	28
	24	2859	71	424	919	2	198	1316	3911	340	100	1105	127	2729
	219	1861	210	2791	3123	1312	2704	1474	1759	609	55	803	1941	1407

226	45	33	118	15	16	47	12	125	58	340	67	1340	13
1791	148	377	9	181	18	639	202	0	1	58	259	358	9
671	89	11	770	802	1712	1799	716	1665	12	33	38	17	252
OTU5764	26	17	1	6	0	32	124	34	26	3	1	4	7
9	48	6	136	234	6	3	38	180	175	497	202	8	15
8	178	12	5	237	92	48	1	98	56	63	86	6	7
2	3	225	25	42	10	47	4	64	51	10	29	75	128
51	32	182	6	45	14	82	43	16	136	59	50	2	81
59	0	61	14	1	30	63	0	15	10	54	1	17	6
4	69	25	25	23	38	68	2	34	1	4	0	0	1
0	0	1	6	0	0	0	42	1	0	6	8	12	0
0													
OTU796	5	0	3	2	1	2	4	0	3	21	0	14	3
6	0	1	0	0	1	1	28	0	0	0	0	2	2
7	0	1	0	0	11	2	6	1	7	1	0	1	0
1	0	0	1	1	0	0	2	0	1	1	0	0	8
0	0	0	1	1	0	0	0	0	1	1	0	0	0
1	7	0	10	1	0	1	2	4	6	0	0	22	8
1	0	25	7	2	0	0	1	2	1	20	3	3	21
7	19	12	28	24	7	11	9	14	5	6	3	3	3
0													
OTU1072	23	19	18	2	24	2	15	22	26	11	0	4	0
0	12	26	3	1	2	3	0	2	0	2	2	1	0
3	1	3	0	8	0	1	0	0	3	2	0	0	1
17	25	0	9	0	0	0	3	0	0	3	0	2	13
0	12	0	2	0	0	4	1	1	1	0	0	6	0
1	4	31	0	2	16	0	0	50	15	0	4	21	12
0	3	0	59	0	3	17	11	1	2	0	0	10	0
0	4	1	1	2	4	0	3	3	4	22	0	8	1
1													
OTU160	11	11	10	12	9	11	5	6	17	6	3	15	13
16	2	21	2	5	1	1	17	0	5	3	4	4	13
3	0	2	6	2	6	5	4	1	3	9	5	6	4

5 38 0 24 3 12 3 9 0 1 8 0 1 7
0 3 6 6 1 0 1 0 0 2 0 13 5 2
0 0 4 8 7 21 6 0 25 10 21 2 44 0
8 5 8 1 15 2 9 7 0 17 2 1 3 3
7 14 5 7 1 1 2 6 7 10 21 10 8 9
5
OTU400 2 0 0 0 0 0 0 0 0 0 0 0 0
0 0 0 0 0 0 0 0 0 0 0 0 0 0
0 0 0 0 0 0 0 0 0 0 0 0 0 0
0 0 0 0 0 0 0 1 0 0 0 0 0 0
0 0 0 0 0 0 0 0 0 0 0 0 0 0
0 1 0 0 0 0 0 0 0 0 0 0 0 0
0 0 0 0 0 0 0 0 0 0 0 7 1 0
0 0 0 0 0 1 0 1 0 0 0 0 0 55
34
OTU964 9 6 8 14 21 8 7 21 9 25 21 10 5
5 10 18 2 38 11 8 13 13 17 4 11 9 11
11 7 31 20 11 16 3 18 3 14 15 14 35 16
16 12 27 7 3 14 3 4 36 31 10 4 10 6
4 8 19 16 6 18 5 10 17 3 22 11 15 7
16 5 7 6 43 14 3 30 9 13 12 64 13 8
11 14 8 9 6 32 6 7 8 6 24 4 6 5
4 9 15 2 14 19 10 19 4 15 11 5 3 14
25
OTU871 31 21 29 13 75 29 17 24 24 63 20 18 13
18 117 25 16 48 15 10 23 12 14 6 21 11 18
11 14 38 25 17 28 5 12 13 27 19 32 55 25
40 25 24 24 10 12 6 5 26 58 12 11 4 4
16 16 19 21 13 24 8 14 21 14 23 15 22 9
78 24 21 4 64 17 12 53 19 9 6 65 9 23
29 16 3 13 12 42 5 25 5 12 32 10 12 24
6 17 23 18 16 21 14 20 17 13 21 5 11 23
144

OTU270 11 4 0 0 0 19 0 9 3 0 0 1 5
0 0 0 0 5 0 8 21 0 0 1 1 1 0
0 0 0 0 4 0 0 1 6 0 0 0 7 3
1 0 0 18 29 0 0 0 0 0 6 0 0 0
0 0 1 3 7 0 1 1 2 1 0 9 0 6
0 0 2 3 2 5 0 2 0 0 0 2 6 0
0 0 0 0 0 3 7 21 0 0 0 0 0 2
4 0 0 2 0 0 0 0 0 1 6 1 0 0
2
OTU259 11 17 15 3 0 3 12 2 2 0 0 7 0
0 5 2 4 9 0 7 3 9 20 16 19 66 2
34 26 2 36 20 2 15 0 2 4 1 0 29 0
19 0 4 1 4 1 0 2 5 14 6 0 1 1
0 9 2 4 4 6 20 34 13 8 6 8 9 2
6 21 1 1 5 10 11 24 0 3 3 2 5 35
3 89 1 3 17 8 6 7 0 0 0 9 7 0
0 26 49 12 20 19 23 14 14 8 3 0 11 28
11
OTU415 7 0 0 0 0 0 0 0 0 0 0 0 0
0 0 0 3 0 0 2 0 7 0 36 0 2 0
0 1 0 0 30 0 3 0 0 0 34 0 1 0
0 0 0 0 0 0 0 0 0 0 0 0 0 0
0 0 0 0 0 0 0 0 0 0 0 0 0 0
0 0 0 0 0 0 0 0 0 0 0 0 0 0
0 0 0 0 0 0 0 0 0 0 0 0 0 0
0 0 0 1 0 0 0 0 0 0 0 0 0 0
0
OTU769 36 14 36 27 50 28 15 32 63 49 34 33 14
23 77 26 8 52 31 13 22 23 28 10 25 23 13
14 16 53 42 18 36 7 21 18 17 19 34 74 30
29 32 42 29 25 21 8 9 34 74 12 14 11 7
30 28 31 36 23 39 12 37 44 25 29 24 27 11
59 54 32 13 75 12 18 70 16 17 16 103 12 32

37 30 11 20 22 52 13 32 9 12 26 21 19 13
15 44 33 17 25 19 10 26 15 23 17 12 9 35
88
OTU203 2 0 0 0 0 0 0 0 0 0 0 0 0
0 0 0 26 0 0 0 0 412 0 12 0 15 0
0 11 0 0 17 0 23 3 0 0 2 0 0 0
0 0 0 0 0 0 0 0 0 25 0 1 0 0
0 0 0 0 1 20 0 4 10 2 0 0 0 0
49 0 0 0 0 0 0 0 0 0 0 0 0 0
0 0 0 0 0 0 0 6 0 0 0 0 0 0
0 0 0 0 0 0 0 0 0 0 0 0 2 0
0
OTU327 5 3 0 0 0 4 10 0 0 0 1 0 0
0 0 0 0 5 0 0 0 0 1 0 1 2 2
0 0 0 0 0 0 2 0 0 1 1 0 0 0
3 0 0 1 0 1 3 2 0 0 0 0 0 1
0 1 1 4 0 0 0 0 0 0 0 2 2 0
0 0 1 0 2 12 1 1 0 0 1 0 0 0
2 0 0 5 0 11 1 3 4 0 0 3 0 0
0 4 0 0 0 0 1 0 0 0 1 2 0 0
2
OTU90 28 5 2 8 1 6 9 6 2 0 0 5 1 0
5 0 14 17 0 2 21 15 1 37 12 2 7 16
23 0 3 36 29 22 5 9 0 39 10 0 0 0
13 18 50 166 26 26 8 79 19 46 62 30 10 32
19 61 12 57 18 110 74 173 73 48 22 10 40 13
0 3 52 0 1 0 4 93 22 145 5 23 0 77
34 0 0 92 23 32 0 3 19 12 0 0 0 3
0 0 0 0 1 0 0 0 0 9 4 37 0 0
OTU178 24 11 23 3 2 11 10 15 47 11 0 12 0
0 7 4 2 0 0 12 2 0 0 0 0 4 0
1 1 0 0 4 0 2 0 1 2 7 0 0 0
6 0 1 0 0 2 0 1 0 0 7 1 0 0

0	5	0	3	1	0	0	1	0	1	0	2	28	0
0	0	58	0	8	8	0	3	0	5	0	1	0	0
0	6	0	0	0	0	1	29	0	5	0	0	0	0
1	0	0	33	0	0	24	0	2	9	6	2	0	68
23													
OTU121	24	26	23	25	14	8	10	24	19	7	0	18	0
0	17	0	2	10	0	28	5	0	0	0	10	2	1
22	0	0	0	1	0	1	5	4	1	6	0	3	1
12	29	0	10	7	4	7	20	0	4	5	7	4	10
1	14	0	10	0	3	7	0	1	2	0	0	10	0
0	3	35	6	44	31	17	3	20	27	0	9	36	25
12	12	6	2	25	26	8	20	0	18	6	5	2	0
5	6	1	6	4	1	0	7	1	3	7	4	20	2
1													
OTU275	2	2	6	6	3	3	3	3	0	1	2	24	1
14	6	20	5	0	1	3	65	8	1	4	9	14	0
3	8	8	7	12	1	2	10	4	28	4	2	13	3
3	10	1	50	13	2	0	1	5	3	11	3	3	2
2	2	12	3	8	4	7	4	3	12	2	2	5	2
2	1	5	21	0	8	3	3	12	1	1	1	3	2
2	5	4	1	7	0	28	4	1	43	22	0	0	11
16	0	0	1	0	1	1	1	2	2	21	10	27	1
0													
OTU872	20	16	21	12	43	26	20	28	33	44	22	16	10
21	54	17	7	34	19	3	10	10	21	4	16	11	12
15	6	34	25	13	18	7	15	9	14	16	23	45	18
19	23	23	18	27	5	8	5	29	72	15	10	7	4
7	16	24	12	10	18	9	16	14	26	29	7	14	6
66	22	21	10	41	10	8	32	8	16	5	92	3	21
18	10	5	7	7	43	5	19	9	17	28	10	14	4
6	25	22	6	20	27	5	11	8	13	12	2	9	17
116													

OTU83	13	30	18	0	8	10	14	24	16	23	5	5	14	71
	6	24	32	7	102	29	2	32	0	39	6	33	34	10
	5	0	9	11	0	33	17	8	0	42	1	45	15	5
	2	20	3	193	4	9	5	1	18	115	5	8	2	16
	9	45	8	59	31	43	9	27	46	3	46	15	87	24
	0	38	17	11	15	9	98	25	7	26	38	0	3	11
	111	1	12	3	8	10	29	52	15	7	0	0	13	10
	0	24	3	1	5	0	0	2	1	8	11	48	11	2
OTU1709		22	19	22	0	15	41	15	75	31	10	10	7	18
	0	6	0	7	0	0	6	7	0	0	1	1	5	0
	11	0	0	6	1	0	0	1	1	4	2	6	4	0
	26	1	0	6	0	0	0	0	0	0	5	0	1	1
	0	1	0	2	0	1	2	2	0	0	1	0	0	0
	1	1	41	3	15	1	2	3	2	77	1	4	7	3
	0	3	1	6	19	5	3	4	0	1	8	1	2	0
	0	0	4	7	4	5	0	0	0	3	11	1	15	1
	4													
OTU854		38	22	42	32	71	29	21	38	41	71	26	36	15
	20	69	40	18	69	29	10	28	17	33	13	27	22	27
	17	14	52	38	13	35	8	25	12	32	20	39	62	30
	40	30	44	22	18	13	5	8	52	74	14	25	10	10
	25	17	27	36	14	29	8	27	50	24	34	22	33	22
	74	36	41	17	74	16	10	55	15	19	8	115	20	31
	28	26	16	20	15	49	19	43	7	24	41	17	19	16
	9	47	40	26	28	37	12	40	10	23	16	11	21	27
	81													
OTU12311		2	3	31	0	0	0	0	0	0	0	0	0	0
	0	0	0	0	0	0	0	0	0	0	0	0	0	0
	0	0	0	0	0	0	0	0	0	0	0	8	0	1
	0	0	0	0	2	0	0	0	0	0	0	0	0	0
	0	0	0	0	0	0	0	0	0	0	0	0	0	0
	1	0	0	0	0	0	0	0	0	0	0	0	0	0
	0	1	0	0	0	0	0	2	0	0	0	0	0	0

0	0	0	0	0	8	0	0	0	1	0	0	0	0
0
OTU200	10	28	11	7	11	11	6	25	13	13	0	45	0
3	22	11	2	1	11	8	31	0	0	7	4	153	16
25	3	4	4	8	3	2	1	19	31	119	0	7	19
13	26	1	24	4	13	8	10	2	0	30	0	3	41
1	12	0	5	1	0	4	2	1	1	1	8	15	1
1	12	17	238	5	37	2	2	40	12	41	10	5	3
11	2	519	11	124	6	21	7	1	37	3	1	4	2
9	7	21	15	9	2	5	18	9	29	14	22	5	19
26
OTU205	5	2	3	1	3	3	1	4	1	3	10	3	0
2	4	12	2	10	2	9	9	1	5	3	2	4	16
0	0	1	1	2	11	2	3	3	6	4	1	1	11
2	2	0	5	1	8	9	4	0	0	2	0	1	7
0	6	1	1	0	0	1	1	0	0	0	1	2	0
0	3	12	1	2	4	1	0	6	0	12	0	7	0
6	0	2	1	2	4	6	5	2	9	1	1	4	2
8	0	0	0	1	1	0	1	0	6	2	2	6	0
2
OTU10404	3	1	0	3	9	0	0	0	0	0	0	0	0
0	1	0	0	0	0	0	0	0	0	0	0	0	0
0	0	0	1	0	0	1	2	0	0	0	0	0	0
0	0	0	5	0	0	1	0	0	0	1	0	0	0
0	0	0	0	0	0	0	0	0	0	0	2	0	1
0	0	0	1	0	0	1	0	7	0	0	0	2	0
0	0	6	3	7	0	0	0	0	0	2	0	0	0
7	0	0	0	0	0	0	0	0	0	1	0	0	0
0
OTU4	58	440	126	841	1701	216	507	78	270	27	355	10	172	4
105	612	953	1019	862	49	466	225	4412	1771	1594	75	122	65
266	1317	657	275	613	606	265	112	149	40	1557	200	276	62
143	156	72	580	400	212	591	642	6	1221	28	1875	385	1049

	678	1378	72	1012	57	2399	57	59	1290	260	140	60	4964	842
	3	545	579	273	510	158	57	58	232	198	14	84	60	572
	116	714	62	154	29	27	21	29	210	40	218	8	1694	50
	5	13	14	13	5	34	206	22	10	23	182	79	0	18
OTU9649		1	0	2	0	0	2	1	2	6	1	0	0	0
	0	0	1	0	1	2	0	1	0	1	0	0	0	0
	1	0	0	1	0	1	1	1	1	0	0	1	2	0
	1	1	0	3	0	2	0	0	0	1	0	0	1	0
	0	0	2	0	0	0	0	0	1	1	0	0	0	0
	1	0	1	0	1	0	0	3	0	1	0	0	0	0
	0	1	0	0	0	0	0	1	0	0	1	0	0	1
	1	0	1	0	0	0	0	1	0	0	0	1	1	1
	0													
OTU11736		1	0	0	0	0	0	1	0	0	0	0	1	0
	0	0	0	0	1	0	0	0	0	0	0	0	1	0
	1	0	0	0	0	0	0	0	0	0	0	0	0	0
	0	0	0	0	1	0	0	0	0	0	0	0	0	0
	0	0	0	0	0	0	0	0	0	0	0	0	0	0
	0	0	1	0	0	0	0	0	0	1	0	0	0	0
	0	0	0	0	0	0	0	1	0	0	0	0	0	0
	0	1	0	0	0	0	0	0	0	0	0	0	0	0
	0													
OTU5858		20	0	29	22	36	21	59	10	17	27	1	29	1
	8	17	11	1	15	6	10	7	1	0	0	17	2	0
	8	0	35	4	6	1	0	0	18	0	2	0	6	4
	10	59	0	90	3	10	1	16	5	0	8	1	7	0
	0	12	1	38	2	1	5	1	0	3	2	0	18	1
	0	7	27	20	15	51	0	0	5	45	8	4	55	11
	7	3	43	23	33	9	4	12	1	38	24	6	7	5
	10	7	8	5	7	3	2	7	12	9	18	20	2	4
	7													
OTU306		3	2	2	0	0	0	6	0	11	1	0	0	1
	0	2	0	3	0	3	1	2	33	0	6	0	87	0

	39	39	0	0	0	0	2	0	0	0	45	0	2	0
	0	0	0	2	0	6	0	3	4	53	4	169	2	1
	0	1	2	2	2	7	0	9	33	0	4	0	19	0
	2	0	4	0	2	0	0	6	0	4	0	91	0	11
	0	1	0	4	0	0	1	1	0	0	0	1	4	0
	0	5	2	15	32	11	36	8	14	8	0	0	0	22
	13													
OTU2998		3	14	7	5	1	1	8	3	10	1	0	1	0
	0	0	0	4	0	0	3	1	11	0	6	3	15	0
	15	0	0	0	1	0	3	1	2	5	0	0	2	0
	3	1	0	3	0	2	0	1	5	8	3	0	2	0
	1	7	0	1	2	3	8	9	4	4	3	0	4	0
	5	18	3	2	2	0	7	20	3	3	2	8	4	22
	3	26	2	1	6	13	8	10	0	0	0	1	11	0
	0	15	20	2	21	23	19	3	8	11	1	0	3	3
	0													
OTU38	69	92	375	111	65	23	555	404	247	497	2	49	1	4
	56	0	19	1	6	491	6	1	0	1	3	5	0	90
	0	0	6	2	3	15	6	40	16	34	0	557	9	478
	35	8	90	1	160	2	51	0	5	7	88	4	8	0
	161	0	145	0	7	2	15	25	0	13	2	100	2	23
	101	1040	9	668	6	3	56	30	802	2	192	44	862	2
	37	3	687	29	517	2	275	0	28	11	65	59	3	1
	99	98	168	107	80	83	70	34	111	11	13	2	87	72
OTU767		1	0	0	0	0	0	0	0	0	0	0	0	0
	0	0	0	0	0	0	0	0	0	0	0	0	0	0
	0	0	0	0	0	0	0	0	0	0	0	0	0	0
	0	0	0	0	0	0	0	0	0	0	0	0	0	0
	0	0	0	0	0	0	0	0	0	0	0	0	0	0
	0	4	0	0	0	0	0	0	0	2	0	0	0	0
	0	0	0	0	0	0	0	0	0	0	0	0	3	0
	0	0	2	0	0	5	0	0	1	0	0	0	0	0
	0													

OTU11021 1 0 0 0 1 0 0 0 0 1 0 0 0
0 0 0 0 0 0 0 0 0 0 0 0 0 0
0 0 0 0 0 0 0 0 0 0 0 0 0 0
0 0 0 0 0 0 0 0 0 0 0 0 0 0
0 0 0 0 0 1 0 0 0 0 0 0 0 0
1 0 1 0 0 0 0 0 0 0 0 0 0 1
0 1 0 0 0 0 0 0 0 0 0 0 0 1
0 0 0 0 0 0 0 0 0 0 0 0 0 0
0
OTU1411 1 0 0 0 0 1 0 0 2 0 0 0 0
0 0 0 8 0 0 0 0 3 0 1 0 1 0
0 0 0 0 2 0 6 0 0 0 3 0 1 0
0 0 0 0 1 0 0 1 0 2 0 0 0 0
0 1 4 0 0 1 0 0 0 0 1 0 0 0
0 0 1 0 0 0 0 1 0 0 0 0 0 0
0 2 0 0 0 0 0 0 0 0 0 0 0 0
0 0 0 1 0 0 0 0 0 0 0 0 0 0
0
OTU1741 35 98 42 60 210 183 101 534 244 58 352 32 88
64 459 399 476 17 127 6 204 50 472 258 637 960 238
46 24 122 211 29 320 578 27 255 710 64 260 2 452
10 184 418 202 52 9 115 13 48 280 76 48 64 4
275 244 58 219 11 114 13 29 243 3 263 93 219 20
65 346 46 9 42 35 105 24 65 164 12 560 61 508
32 82 63 166 20 109 241 285 502 589 127 47 95 276
51 55 98 94 7 30 180 36 52 213 165 394 196 37
OTU173 25 27 1 0 0 30 2 1 12 9 3 2 6
1 8 0 0 3 0 5 0 1 0 0 15 64 14
2 0 0 3 7 0 2 0 2 1 1 0 2 6
3 0 0 4 0 10 1 20 0 0 2 0 0 7
0 1 1 18 0 0 0 0 0 0 1 5 12 1
0 11 51 0 1 4 1 2 0 0 6 1 0 1
0 2 0 5 1 2 0 5 2 0 0 10 17 0

	0	8	18	8	4	9	4	24	2	11	0	1	3	8
	1													
OTU61	3	1	3	0	8	1	2	2	6	6	10	103	13	57
	1	201	1	147	9	2	117	1	50	70	159	0	12	0
	0	6	88	3	106	10	105	0	484	9	59	1	15	1
	83	0	111	16	0	16	0	3	0	17	0	5	4	2
	0	14	0	3	1	1	0	0	11	0	165	1	10	0
	10	4	47	1	16	6	0	16	0	8	1	158	1	9
	0	1	2	44	2	3	0	7	189	129	4	1	13	246
	0	9	4	1	2	0	5	3	1	11	35	243	8	1
OTU311		16	0	23	6	0	37	0	0	12	0	0	3	0
	0	0	0	0	0	0	11	0	0	0	0	1	1	6
	0	0	0	0	0	0	0	0	0	0	0	0	0	0
	1	0	0	0	0	6	0	6	2	0	0	0	0	2
	0	0	0	1	0	0	0	0	0	0	0	0	31	0
	0	0	17	0	6	0	0	0	0	0	0	0	0	1
	0	0	0	0	0	0	4	6	0	0	0	1	1	0
	0	10	5	1	2	0	53	4	9	12	0	0	0	1
	8													
OTU1130		16	16	29	12	59	12	21	28	32	57	23	15	10
	26	86	23	9	44	24	6	23	12	11	10	21	19	14
	12	10	40	22	12	19	6	11	4	12	14	28	45	19
	33	30	28	26	21	15	10	5	28	60	12	4	7	9
	18	12	16	15	12	18	11	18	22	16	19	17	19	9
	63	31	13	6	48	15	8	42	13	18	9	69	11	22
	17	10	10	3	13	47	5	19	9	14	20	18	14	13
	9	17	27	14	28	24	9	15	14	15	21	9	7	21
	91													
OTU387		5	9	8	0	0	0	0	0	5	0	0	0	0
	0	1	0	1	0	0	11	0	4	0	0	0	0	0
	7	4	0	0	3	0	1	1	1	2	1	0	1	0
	1	0	0	0	2	2	0	1	5	1	1	9	0	0
	0	0	0	0	0	1	2	10	8	4	4	2	1	2

	0	1	1	1	7	0	0	17	0	4	0	3	0	6
	0	11	2	0	0	4	0	1	0	1	0	1	3	0
	0	1	1	0	0	3	0	2	0	2	0	0	0	2
	4													
OTU85	20	0	29	21	67	42	16	10	59	2	6	7	1	23
	18	29	18	0	10	8	22	17	0	2	0	53	92	4
	28	0	2	28	9	40	18	15	0	14	0	10	19	13
	0	48	0	22	38	4	41	28	18	25	18	2	15	9
	13	20	14	2	11	21	36	21	37	8	0	24	13	30
	10	64	0	15	0	0	27	0	1	2	9	6	26	0
	90	0	1	31	30	77	38	24	1	14	3	7	32	29
	0	21	17	13	9	5	0	12	14	7	14	16	15	3
OTU9000	2	3	6	0	0	0	2	2	2	9	0	0	0	
	0	4	0	0	0	0	17	0	0	0	0	0	0	0
	1	0	0	0	0	0	0	0	0	0	0	0	1	0
	7	0	0	0	0	0	0	0	0	0	0	2	0	0
	0	1	0	0	0	0	0	0	0	0	0	0	0	0
	0	0	0	0	6	0	0	0	0	7	0	3	0	10
	0	0	0	9	0	7	0	0	0	0	0	0	0	0
	0	0	0	0	0	0	0	0	0	0	0	0	0	0
	0													
OTU11024	1	0	0	0	0	0	0	0	0	0	0	0	0	
	0	0	0	0	0	0	0	0	0	0	0	0	0	0
	0	0	0	0	0	0	0	0	0	0	0	0	0	0
	0	0	0	0	0	0	0	0	0	0	0	0	0	0
	0	0	0	0	0	0	0	0	0	0	0	0	0	0
	0	0	0	0	0	0	0	0	0	0	0	0	0	0
	0	0	0	0	0	0	0	0	0	0	0	0	0	0
	0	0	0	0	0	0	0	0	0	0	0	0	0	0
	0													
OTU322	2	4	0	0	1	0	1	0	2	1	0	0	0	
	1	1	0	0	2	0	1	0	0	0	0	6	0	0
	0	0	0	0	0	0	1	1	0	0	4	0	0	0

	3	0	0	0	1	2	18	9	0	0	0	2	0	0
	0	1	0	1	0	0	0	0	1	0	0	1	7	0
	0	0	0	0	1	0	1	0	0	4	0	1	1	0
	15	0	0	0	0	0	0	2	8	0	2	0	0	1
	0	0	0	0	0	0	0	2	0	0	5	2	0	0
	0													
OTU105	15	0	17	3	0	37	97	195	152	33	0	0	1	
	0	1	0	8	1	0	85	0	29	0	0	0	37	0
	31	9	0	0	4	0	1	1	1	1	0	0	72	1
	18	0	1	4	0	90	0	47	0	17	2	60	0	0
	0	6	1	79	0	1	0	15	14	0	3	0	219	0
	4	10	90	0	13	0	0	55	0	18	0	16	0	87
	0	15	0	49	1	79	28	102	0	0	0	5	8	0
	0	12	21	3	11	10	34	26	5	7	0	0	0	7
	3													
OTU7813	1	2	0	0	0	0	1	0	0	0	0	0	0	
	0	0	0	0	0	0	0	0	0	0	0	0	3	1
	0	0	0	0	0	0	0	0	0	0	0	0	1	0
	0	0	0	0	0	0	0	0	0	0	0	1	0	1
	0	0	0	2	0	0	0	0	0	0	0	0	2	0
	0	0	0	0	0	0	0	0	0	0	1	0	0	0
	0	0	0	0	0	0	1	0	0	0	0	0	0	0
	0	0	0	0	0	0	0	0	0	0	0	0	0	0
	0													
OTU9478	7	3	4	3	8	1	2	2	0	9	1	1	1	
	4	9	1	4	1	0	1	2	1	5	3	2	3	1
	1	0	3	2	2	2	0	0	0	2	0	5	7	0
	2	1	1	4	4	1	0	1	3	7	0	1	1	3
	0	1	1	3	2	2	2	1	3	1	2	1	2	1
	6	0	4	1	4	2	0	4	2	0	1	13	3	2
	1	1	1	0	0	8	2	2	2	1	4	2	3	3
	0	1	4	1	2	2	1	2	0	3	1	1	1	4
	14													

OTU210	1	13	0	0	0	0	6	0	1	2	0	0	0
0	0	1	0	0	0	0	0	0	1	0	13	0	0
0	0	0	0	0	0	0	0	0	2	0	0	0	0
0	1	0	5	0	0	0	7	0	0	0	0	0	0
0	0	0	1	0	0	0	0	0	0	0	1	0	0
0	83	1	0	0	1	0	0	0	5	1	1	0	0
1	0	0	6	0	2	0	0	0	0	0	92	38	0
0	110	12	13	23	10	1	50	2	6	0	3	0	14
77													
OTU138	2	55	8	4	1	11	12	4	0	1	26	2	156
74	12	7	13	81	31	1	4	3	22	10	114	0	41
0	1	106	11	6	53	6	5	4	9	21	91	0	77
0	13	43	66	1	0	94	0	1	1	2	22	2	2
4	1	10	0	4	0	3	2	0	1	3	4	0	7
2	0	5	29	1	4	19	0	3	3	22	0	0	0
23	1	30	2	10	4	2	0	97	2	18	0	0	7
3	0	0	0	0	0	0	1	0	0	5	4	1	0
0													
OTU15794	4	3	7	5	4	4	5	4	11	8	5	3	2
3	16	4	1	10	6	0	8	2	5	4	2	7	2
3	4	9	2	3	4	1	4	0	3	1	3	9	3
3	2	9	3	3	1	0	1	4	7	1	2	1	6
2	2	8	5	2	4	1	2	4	4	10	6	3	4
6	6	3	1	9	2	4	8	4	2	1	16	2	8
4	0	2	2	1	10	1	3	1	4	7	1	7	1
1	10	5	0	5	2	2	5	2	2	4	1	0	5
24													
OTU3976	2	1	2	1	5	3	0	2	1	1	1	3	1
1	2	4	0	2	2	1	2	1	0	0	0	3	0
1	4	2	4	1	0	0	1	0	1	1	1	6	1
3	3	2	3	0	1	0	1	2	9	0	0	2	0
2	3	1	2	0	1	1	1	4	3	0	0	0	0
4	1	2	1	3	2	0	4	0	1	1	7	0	4

3	0	2	1	2	3	1	2	0	2	1	1	3	0
0	1	1	3	1	0	1	2	1	3	0	0	1	0
5													
OTU978	4	18	1	2	10	2	2	10	17	4	0	5	0
1	9	6	0	1	1	6	1	0	0	4	66	1	1
2	1	8	1	78	8	3	0	1	0	5	1	16	4
9	11	0	5	10	2	5	7	0	0	7	0	0	51
0	2	54	1	0	0	0	0	0	13	1	18	22	8
0	24	25	0	0	14	0	0	29	4	2	3	11	1
0	6	10	13	2	1	11	0	2	2	0	141	59	1
0	307	38	39	19	21	11	142	20	15	10	2	4	23
35													
OTU14015	2	0	1	1	1	2	0	1	2	2	1	0	0
1	3	0	0	0	1	1	1	1	0	0	0	0	0
0	0	1	0	0	0	0	0	0	0	2	2	3	0
2	3	0	0	0	0	0	0	3	2	0	0	1	0
1	2	0	1	0	2	0	1	3	0	0	0	0	0
2	2	1	1	1	0	0	2	0	1	1	2	1	3
0	0	0	0	0	1	0	0	0	0	3	0	0	0
0	0	0	0	1	1	0	1	0	1	0	1	0	1
4													
OTU2428	12	0	0	0	5	0	0	2	0	0	1	8	2
0	6	6	0	2	0	0	0	0	0	0	1	0	0
0	0	0	0	0	5	0	2	45	1	0	6	0	0
0	2	0	1	0	0	14	0	0	0	0	0	0	5
0	1	0	0	0	0	0	0	0	0	0	0	0	0
0	0	0	2	0	5	1	0	6	0	0	0	2	0
0	0	1	0	0	4	0	0	0	3	7	0	0	3
7	0	0	0	0	0	0	0	2	0	4	3	4	0
0													
OTU31 4	10	2	123	2	2	16	1	7	1	375	69	406	2215
0	79	85	64	1830	0	17	5	73	9	16	1	41	1
57	1022	265	344	29	5	267	5	13	0	8	0	548	12

	7	661	68	189	10	100	0	377	4	34	13	563	5	654
	3	517	5	19	190	459	171	54	167	172	50	22	100	196
	9	0	39	7	85	423	0	5	9	13	0	15	2	34
	61	68	1	10	5	65	1	1245	69	202	9	12	1102	204
	39	16	2	4	11	3	25	32	3	2	497	218	7	0
OTU14122		7	2	4	6	7	6	6	8	7	12	6	8	0
	6	19	3	2	5	4	2	10	6	3	1	2	5	4
	5	2	8	9	2	8	2	3	4	3	5	8	13	10
	7	8	8	5	2	3	1	3	8	14	4	1	4	3
	6	2	7	7	0	9	0	3	11	2	3	5	8	0
	21	9	6	1	10	2	3	9	4	3	2	13	2	6
	7	0	1	5	4	5	1	6	1	5	6	3	7	2
	1	6	2	3	6	6	2	1	1	6	3	2	1	6
	22													
OTU35	11	1	10	31	37	1	12	5	12	6	2	21	6	0
	15	54	262	13	13	24	62	585	0	15	5	11	41	29
	22	0	10	140	0	57	170	6	54	16	0	9	1	22
	8	35	9	421	108	43	12	368	31	83	13	222	0	748
	20	332	68	86	313	267	119	125	1229	189	294	135	135	252
	0	3	11	6	4	51	0	0	2	4	0	3	1	15
	79	0	0	1	9	88	0	45	37	127	0	2	76	78
	0	0	6	12	5	32	0	19	0	27	231	36	0	0
OTU84	13	6	5	45	21	8	15	5	4	1	33	11	38	19
	5	27	27	31	10	41	23	27	49	26	26	25	3	26
	5	27	12	34	20	20	13	5	38	25	19	7	15	16
	43	7	20	2	24	32	48	6	24	7	29	3	6	5
	26	4	24	4	8	5	19	14	3	6	13	26	7	8
	2	6	25	1	29	17	9	11	17	24	8	9	7	39
	1	53	9	16	3	2	7	45	16	66	12	8	28	26
	13	2	5	6	2	3	4	4	2	28	49	16	1	4
OTU1363		10	1	0	0	0	0	0	0	0	0	1	0	0
	0	2	0	2	9	0	0	3	0	0	0	3	1	1
	0	0	0	1	4	11	1	0	0	0	0	4	0	0

0	0	0	1	0	0	0	0	0	0	0	0	0	0
0	0	1	0	0	0	0	0	0	0	0	0	0	2
0	0	0	8	0	3	0	0	2	0	6	0	0	0
0	0	1	0	1	0	2	0	0	0	0	0	0	0
1	0	0	0	0	0	0	0	0	1	0	0	1	0
0													
OTU571	12	0	4	1	0	3	0	1	0	0	0	0	0
0	1	0	0	0	0	0	0	0	0	0	0	0	0
0	0	0	0	0	0	0	0	0	0	0	0	0	0
0	0	0	0	0	0	0	0	0	0	0	0	0	0
0	0	0	0	0	0	0	0	0	0	0	0	0	0
0	1	0	0	0	0	0	0	0	0	1	0	0	2
0	0	0	0	0	0	0	3	0	0	0	0	0	0
0	0	0	0	0	2	1	0	1	0	1	0	0	0
0													
OTU131	19	151	56	14	93	40	161	126	20	121	0	4	0
0	58	0	10	1	0	22	1	10	0	12	3	24	1
74	4	0	3	32	6	7	0	9	0	23	0	12	0
22	6	3	105	7	4	0	15	0	1	16	2	9	2
2	9	1	27	0	3	10	3	5	8	1	0	34	1
2	1	86	0	107	417	1	7	3	26	1	2	43	48
0	23	1	421	3	68	7	22	1	9	22	2	9	9
0	2	3	0	2	4	2	13	1	7	1	0	5	2
0													
OTU107	27	2	81	28	3	35	62	21	73	406	0	165	0
1	130	0	1	0	0	255	0	0	0	0	0	1	0
29	2	0	0	3	0	1	0	0	1	1	0	13	1
35	12	1	2	0	0	0	1	0	0	0	33	0	0
0	0	0	27	0	4	1	6	2	0	3	0	51	0
2	24	3	0	82	15	0	20	0	23	3	408	1	145
0	4	0	196	0	9	0	73	0	11	0	62	340	0
0	21	60	5	11	26	7	161	27	241	53	2	3	4
0													

OTU14273 1 0 0 0 0 0 0 0 0 0 0 0 0
0 0 0 0 0 0 0 0 0 0 0 0 0 0
0 0 0 0 0 0 0 0 0 0 0 0 0 0
0 0 0 0 0 0 0 0 0 0 0 0 0 0
0 0 0 0 0 0 0 0 0 0 0 0 0 0
0 0 0 0 0 0 0 0 0 0 0 0 0 0
0 0 0 0 0 0 0 0 0 0 0 0 0 0
0 0 0 0 0 0 0 0 0 0 0 0 0 0
0

OTU2090 12 3 5 0 18 23 7 15 3 5 13 20 48
7 5 5 0 71 98 2 0 0 1 0 1 1 19
20 0 24 62 4 0 2 0 4 5 3 1 7 35
30 2 0 8 3 9 5 4 0 1 3 4 1 0
0 1 2 99 0 5 10 18 6 6 3 1 7 1
1 103 39 6 1 112 19 36 11 12 7 50 76 1
0 27 9 11 26 1 1 1 12 28 21 50 117 18
12 102 48 42 27 35 8 34 32 33 1 4 2 17
27

OTU147 8 31 13 16 9 8 19 38 26 10 2 4 7
1 8 2 20 5 0 9 16 5 0 5 9 36 13
36 2 2 3 16 0 25 6 10 10 8 1 13 1
8 3 0 9 1 6 6 10 1 6 4 3 4 1
0 11 1 10 0 2 0 4 6 11 1 1 55 1
0 12 0 14 6 1 6 7 7 40 8 6 2 53
21 10 0 3 7 3 7 12 3 13 1 16 5 2
7 14 29 40 47 16 30 27 14 10 2 5 3 15
8

OTU16817 2 2 0 0 9 1 1 1 0 0 9 0 2
0 5 1 0 4 17 1 0 0 11 0 10 0 0
0 0 11 1 0 5 0 3 8 0 0 14 0 14
0 0 1 12 0 0 1 0 0 0 0 1 0 0
0 1 0 0 0 0 0 0 0 0 0 0 0 0
0 0 0 2 0 10 7 0 1 0 8 0 4 0

1	0	9	0	2	1	0	0	0	3	0	0	0	2
0	0	0	0	0	0	0	0	0	0	0	7	0	0
0													
OTU379	4	1	0	0	2	0	0	0	0	0	0	2	1
7	0	0	5	0	1	0	0	7	0	0	0	0	2
0	9	0	0	2	0	3	0	1	0	0	0	0	0
0	0	1	0	2	0	0	5	0	1	7	0	0	1
0	1	0	0	2	6	0	7	0	0	2	0	1	0
11	1	0	0	1	0	0	2	0	0	1	0	0	0
3	2	0	0	0	0	0	0	4	0	0	0	0	0
0	0	0	1	0	1	2	2	6	0	0	1	0	4
9													
OTU676	1	9	0	2	0	0	0	0	1	2	0	1	0
0	0	0	1	0	0	19	0	15	0	0	1	0	0
10	216	0	0	0	2	0	3	0	4	35	1	3	1
0	0	0	0	1	0	0	1	0	4	29	26	0	0
0	0	6	14	3	21	0	35	44	0	1	1	0	0
8	1	0	1	0	5	0	3	8	0	1	15	0	2
0	6	3	2	0	0	0	0	0	0	0	0	1	1
0	0	1	3	4	4	0	1	8	33	2	1	22	3
19													
OTU4011	21	106	18	16	60	61	2	11	6	4	2	19	0
12	19	0	4	0	0	9	19	7	0	3	11	12	0
0	1	45	58	0	11	5	67	8	34	20	4	5	64
0	7	2	20	0	3	71	2	3	0	20	3	3	1
1	0	0	23	5	2	9	3	1	1	1	1	2	14
4	0	20	126	0	1	19	3	52	8	18	2	18	3
2	2	95	13	14	2	60	4	31	41	34	0	0	0
118	0	0	1	0	0	0	0	7	2	58	58	89	1
0													
OTU273	28	0	3	0	0	2	0	0	0	0	12	0	4
0	0	0	0	3	8	11	0	0	0	0	0	0	1
14	0	0	0	0	0	0	0	0	12	3	1	0	0

3	0	0	9	0	8	5	0	0	0	7	8	0	0
0	0	0	10	0	0	0	0	0	1	0	0	0	0
0	1	1	0	6	0	0	0	0	0	0	0	3	4
0	1	1	9	0	0	0	0	0	3	0	0	3	0
1	0	0	0	2	0	0	1	1	4	0	0	0	0
0													
OTU318	2	0	2	0	0	1	4	1	0	1	0	0	0
0	2	0	3	0	0	1	0	0	1	5	3	65	0
4	7	0	0	6	0	2	0	1	2	0	0	6	0
10	0	0	1	0	0	0	0	2	9	1	0	0	0
1	7	1	1	0	3	7	5	5	4	1	0	4	1
4	8	3	0	2	2	1	42	0	1	1	11	0	18
5	33	0	2	3	1	3	1	0	0	0	1	6	1
0	4	9	2	6	2	1	4	4	14	6	0	5	2
0													
OTU11547	3	4	0	0	1	4	3	0	0	2	0	0	0
0	13	0	0	0	0	3	0	0	0	0	0	2	0
0	0	0	0	2	0	0	0	0	0	2	0	3	0
1	0	0	0	0	2	0	8	0	0	0	0	0	1
0	0	0	0	0	0	0	0	0	0	0	0	0	0
0	1	0	2	0	0	0	0	0	0	0	0	0	0
0	0	0	0	0	0	0	3	0	0	0	0	0	0
0	0	0	1	0	0	0	0	0	0	0	0	0	0
0													
25	17	28	27	11	17	19	20	13	17	24	12	12	1
16	36	4	16	0	59	4	0	1	2	7	11	13	11
0	5	13	1	3	10	0	4	53	13	0	17	0	8
1	1	1	10	35	28	55	0	0	24	1	0	21	0
16	6	31	2	1	0	1	1	2	0	5	12	2	0
1	13	5	16	10	9	2	3	46	27	4	39	10	50
1	0	31	17	6	0	9	1	88	64	2	2	2	50
0	5	14	4	0	6	1	1	2	57	14	14	2	1

OTU2301 4 2 5 4 6 1 2 1 3 8 2 3 3
3 2 2 1 2 4 2 3 3 6 0 4 2 4
2 2 6 4 4 6 1 0 2 2 5 5 12 4
5 2 3 3 1 1 1 3 3 12 0 1 0 0
2 2 1 5 2 5 2 0 4 2 4 9 6 0
6 0 2 3 11 4 2 7 2 2 0 16 1 2
3 2 0 0 2 9 2 2 3 1 5 1 1 3
1 3 6 4 1 4 1 3 2 3 0 1 2 4
11

OTU10897 7 1 1 10 11 10 5 5 2 7 3 5 5
3 3 1 0 1 4 6 0 0 0 0 0 1 2
1 0 2 0 0 2 1 1 7 0 0 1 7 2
4 0 1 0 0 5 3 8 0 0 0 0 0 2
0 9 1 7 2 0 0 1 0 1 0 0 7 0
1 2 6 1 3 3 1 4 3 7 1 0 0 3
0 3 0 3 2 4 0 3 2 1 3 0 2 0
0 1 0 1 0 0 0 3 3 2 2 0 0 5
1

OTU244 5 6 10 9 19 18 31 10 16 30 19 57 12
11 15 4 0 12 3 25 9 1 0 1 3 4 4
16 1 2 0 1 6 4 31 29 3 5 3 58 8
46 4 1 2 13 67 17 87 0 0 30 4 0 31
0 125 0 41 19 1 2 5 1 9 2 0 124 1
3 30 19 4 15 3 1 5 4 57 6 3 1 18
6 4 0 15 13 21 3 45 11 10 15 23 33 3
9 49 95 69 27 42 18 26 58 69 16 9 2 78
54

OTU80 12 5 2 37 4 1 5 2 0 0 11 230 42 25
14 0 4 0 0 6 3 1 4 0 2 0 23 0
0 0 1 16 6 0 65 22 9 4 14 1 0 1
6 2 7 10 0 7 2 5 0 62 5 6 6 6
2 42 2 5 0 4 0 2 19 1 123 2 0 9
1 2 5 0 91 8 0 0 3 13 0 6 0 3

2 160 2 67 0 0 0 46 0 38 0 0 19 68

0 0 1 0 0 0 0 10 0 15 95 212 0 0

OTU289 24 0 1 1 0 4 4 2 0 76 0 0 0

0 0 0 0 0 0 21 1 0 0 0 4 1 0

0 0 0 0 0 0 0 0 0 11 0 0 0 0

0 0 0 0 0 0 0 0 0 0 0 1 0 2

0 2 0 0 0 1 0 0 0 0 0 0 2 0

0 1 0 0 3 0 0 0 0 55 0 2 0 0

0 0 0 0 0 0 0 0 0 0 0 0 0 0

0 0 0 0 0 0 0 0 7 24 0 0 0 0

11

OTU15320 2 6 2 0 0 1 3 2 4 1 0 1 2

7 0 1 3 0 9 1 0 3 0 7 1 4 5

2 1 0 1 4 1 3 1 0 0 4 0 2 1

0 0 4 0 17 1 1 0 1 2 7 1 0 0

1 0 12 0 13 5 6 2 11 8 1 2 0 9

6 0 5 7 1 1 1 20 2 1 1 7 0 0

2 5 0 3 1 0 2 3 3 0 0 0 0 1

0 0 1 0 0 0 0 0 0 0 1 5 4 1

0

OTU946 14 9 18 14 39 19 12 19 22 30 32 18 12

10 28 21 5 42 20 4 14 20 20 8 9 13 8

15 11 38 22 12 19 5 26 10 14 8 22 33 18

21 19 30 9 14 18 9 9 29 49 9 17 9 4

9 8 15 13 9 20 6 19 22 17 34 19 17 12

25 14 16 12 43 13 2 40 5 11 6 79 9 13

13 15 9 18 15 29 7 25 9 16 19 9 19 7

10 24 26 16 18 18 2 16 11 10 11 9 6 17

35

OTU5318 3 2 0 13 5 9 8 5 0 0 0 1 0

0 9 0 0 0 0 1 0 0 0 0 0 4 9

0 0 0 0 0 0 2 0 50 0 1 0 2 0

3 0 0 1 0 11 1 25 0 0 3 0 0 51

0	4	1	11	0	0	0	0	0	0	0	4	7	0
0	0	0	1	2	0	0	0	0	5	0	0	0	3
0	1	0	2	0	11	3	0	0	0	0	0	0	0
2	0	0	0	0	0	0	0	0	0	1	0	0	0
0													
OTU10261	2	1	1	0	0	0	1	2	17	0	0	1	7
0	1	0	1	3	1	4	0	5	17	3	3	4	0
3	4	0	0	2	1	1	0	1	2	7	0	2	0
1	0	28	1	23	2	6	1	6	5	36	1	5	0
4	3	11	12	7	4	3	29	4	4	2	4	1	0
7	0	8	1	1	0	2	16	4	0	0	6	1	2
3	38	1	1	4	0	2	13	0	0	0	0	0	0
0	0	2	4	4	1	3	18	6	0	0	0	0	7
5													
OTU11012	1	0	0	0	0	0	0	0	0	0	0	0	0
0	0	0	0	0	0	0	0	0	0	0	0	0	0
0	0	0	0	0	0	0	0	0	0	0	0	0	0
0	0	0	0	0	0	0	0	0	0	0	0	0	0
0	0	0	0	0	0	0	0	0	0	0	0	0	0
0	0	0	0	0	0	0	0	0	0	0	0	0	0
0	0	0	0	0	0	0	0	0	0	0	0	0	0
0	0	0	0	0	0	0	0	0	0	0	0	0	0
0													
OTU89 10	31	8	4	13	1	4	3	3	1	0	39	0	56
2	0	0	5	0	5	114	0	0	7	0	6	0	14
0	0	0	18	11	5	43	1	5	41	0	3	24	10
2	3	1	68	176	0	4	34	1	114	225	13	0	5
11	74	69	10	19	8	6	0	12	16	18	64	36	2
5	0	0	0	1	0	1	1	2	7	3	3	9	0
1	1	0	0	3	60	7	1	72	12	4	2	7	35
2	9	56	1	94	46	2	84	13	9	5	91	43	33
OTU1023	14	10	17	12	38	17	8	19	23	28	14	17	13
18	15	19	8	38	10	10	16	14	19	7	8	12	11

12	6	40	28	15	18	3	19	7	16	12	32	55	17
18	14	29	13	16	10	5	8	27	50	11	11	8	10
18	10	20	9	11	18	7	12	10	15	23	5	23	10
35	13	19	8	45	6	12	33	11	10	11	85	5	17
19	10	6	9	9	30	10	21	10	12	23	8	19	8
10	21	12	14	17	15	6	17	7	14	16	9	4	12
31													
OTU6111	2	1	3	0	3	3	1	3	3	1	2	1	0
2	3	4	2	2	2	0	2	0	0	1	0	2	2
5	1	2	2	0	3	0	1	0	1	1	0	6	1
1	0	2	2	0	0	0	1	3	1	0	0	0	0
0	0	1	2	0	2	1	4	2	0	1	1	2	1
2	2	2	1	5	2	1	5	1	0	0	8	0	1
3	0	1	0	0	5	0	5	2	0	4	1	2	3
0	4	2	2	1	3	0	2	0	1	1	0	0	1
3													
OTU1361	1	12	0	0	2	0	1	0	8	2	0	0	0
0	0	0	0	0	0	1	0	0	0	0	0	0	0
0	0	0	0	0	0	0	0	1	0	1	0	2	0
2	0	0	0	0	0	0	0	0	0	0	0	0	0
0	1	0	0	0	0	0	0	0	0	0	0	1	0
0	0	2	0	0	0	0	0	0	0	0	3	0	13
0	1	0	4	0	0	0	6	0	0	0	0	1	0
0	3	0	12	1	0	10	2	1	2	0	0	0	11
0													
OTU109	20	16	41	51	14	104	198	34	343	324	0	20	2
1	33	4	5	27	7	168	22	201	1	14	2	71	6
306	5	16	0	6	2	42	7	64	13	568	1	860	4
655	37	10	12	69	231	1	448	0	4	296	24	2	371
7	449	30	233	28	6	50	3	26	156	5	3	432	14
29	1177	297	3	82	3	4	80	12	476	64	158	10	375
5	36	3	256	29	444	10	568	2	11	0	1014	2771	11

1 248 340 1485 290 445 639 1117 871 1236 4 14 7 2053
1158
OTU11013 1 0 0 0 0 0 0 0 0 0 0 0 0
0 0 0 0 0 0 0 0 0 0 0 0 0 0
0 0 0 0 0 0 0 0 0 0 0 0 0 0
0 0 0 0 0 0 0 0 0 0 0 0 0 0
0 0 0 0 0 0 0 0 0 0 0 0 0 0
0 0 0 0 0 0 0 0 0 0 0 0 0 0
0 0 0 0 0 0 0 0 0 0 0 0 0 0
0 0 0 0 0 0 0 0 0 0 0 0 0 0
0
OTU10266 5 0 1 1 8 1 1 2 4 4 1 2 2
1 14 2 1 4 2 3 1 3 2 2 2 0 2
3 1 3 1 1 1 1 1 1 0 1 0 12 3
6 4 1 3 2 0 1 2 2 6 2 3 1 1
0 0 4 4 3 2 1 5 5 3 0 1 3 1
13 2 3 0 0 3 2 10 2 1 0 5 1 2
5 0 3 0 3 5 0 5 0 3 2 0 1 0
1 3 2 1 5 5 2 5 2 0 1 0 1 3
11
OTU8465 10 0 5 19 4 27 3 26 0 10 1 0 0
0 4 0 1 1 0 14 0 0 0 0 0 27 26
5 0 0 1 0 0 0 0 0 0 0 0 0 0
0 0 0 0 0 0 0 0 0 0 1 0 0 0
0 0 0 0 0 0 0 0 0 0 0 0 3 0
0 0 0 0 0 0 6 0 0 0 0 2 0 1
0 1 0 0 0 0 16 41 0 0 2 0 0 0
4 3 0 0 0 2 0 0 0 1 0 0 0 0
0
OTU7332 8 1 0 0 0 0 0 0 0 0 0 6 0
0 0 2 0 0 0 1 0 49 0 37 10 4 0
17 43 0 1 8 0 1 0 0 0 10 0 5 0
0 0 67 0 1 0 0 0 8 8 3 0 0 0

3	0	3	0	6	3	15	12	0	1	10	2	1	1
10	0	0	0	0	4	0	0	0	2	0	0	1	0
5	100	0	0	0	0	16	4	0	1	0	0	0	0
0	0	1	3	0	1	1	0	1	0	4	0	1	0
0													
OTU336	2	1	1	0	3	3	1	4	5	5	0	1	0
1	1	2	1	0	6	3	4	0	1	3	0	1	1
1	1	0	1	1	0	0	0	0	3	1	0	3	2
0	2	0	2	0	6	0	0	0	1	0	1	0	0
0	1	0	2	0	1	0	0	3	0	0	0	1	1
1	4	4	2	3	13	0	1	2	4	0	0	2	1
0	2	2	12	0	2	0	2	0	4	0	0	1	0
4	1	15	9	5	17	23	2	8	8	2	0	5	16
9													
OTU130	1	7	2	8	1	1	0	1	1	1	0	2	0
3	1	0	4	0	3	370	0	0	0	0	2	3	0
20	1	0	0	0	0	0	0	1	120	0	0	12	0
1	2	0	1	0	0	0	0	0	0	0	1	0	0
0	0	0	5	0	0	0	0	0	0	0	0	2	0
0	0	10	0	0	0	0	0	0	2	0	5	4	1
4	0	0	11	0	16	0	22	0	0	0	0	0	0
24	0	1	1	0	0	0	1	0	0	66	2	94	0
0													
OTU8177	1	0	1	0	0	0	0	0	0	0	0	0	0
0	0	0	0	0	0	0	0	0	0	0	0	0	0
0	0	0	0	0	0	0	0	0	0	0	0	0	0
0	0	0	0	0	0	0	0	0	0	0	0	0	0
0	0	0	0	0	0	0	0	0	0	0	0	0	0
0	0	0	0	0	0	0	0	0	0	0	0	0	0
0	0	0	0	0	0	0	0	0	0	0	0	0	0
0	0	0	0	0	0	0	0	0	0	0	0	0	0
0													

OTU1677 1 1 1 2 1 2 0 0 0 1 1 0 1
1 0 3 0 3 0 2 1 0 0 0 0 0 0
1 0 0 0 0 1 0 2 4 0 0 0 0 0
0 0 0 0 1 3 1 0 0 0 2 0 0 1
0 1 0 1 0 0 0 0 0 0 0 0 1 1
1 1 0 1 0 1 0 0 2 0 0 0 0 2
1 0 0 0 0 1 0 1 0 2 1 3 1 0
0 0 0 1 0 0 1 1 0 1 1 0 0 3
0
OTU144 14 1 1 0 0 0 0 0 3 1 1 3 23
13 0 2 17 0 0 6 11 44 0 3 0 6 1
0 2 0 1 6 0 32 3 0 0 0 0 3 0
3 0 20 0 31 0 6 0 13 3 29 0 16 1
9 0 2 0 101 19 8 35 7 22 7 30 0 40
26 0 1 0 0 0 0 0 29 1 0 0 0 5
0 0 0 4 0 0 1 0 1 3 0 0 0 1
31 0 2 0 0 1 1 0 3 4 0 5 16 6
0
OTU692 1 0 2 0 0 1 0 0 2 1 0 3 0
0 0 1 0 0 2 1 0 0 0 0 0 0 0
1 1 0 0 0 0 0 0 0 0 0 0 0 0
0 4 0 1 0 4 0 0 0 0 3 0 0 0
0 0 0 3 0 0 0 0 0 0 0 0 3 0
0 1 0 2 1 0 0 0 0 0 0 0 0 0
0 0 0 0 0 0 1 1 0 0 1 0 1 0
0 6 1 3 1 2 0 1 2 6 1 1 0 2
1
OTU187 12 13 9 0 0 2 5 5 10 21 0 2 0
0 2 0 0 1 0 3 0 0 0 0 4 3 1
0 0 0 0 0 2 0 0 1 2 11 1 1 0
3 1 0 1 0 0 0 1 0 1 0 2 0 0
0 0 0 0 1 0 0 1 0 0 0 0 0 0
0 126 2 0 2 1 0 1 0 2 1 7 0 8

0	2	0	13	0	0	1	2	0	2	0	82	110	0
1	21	18	48	5	4	2	72	9	34	1	1	2	23
19													
OTU7897	1	1	0	0	0	2	0	1	0	0	0	1	0
0	0	0	0	0	0	1	0	0	0	0	0	0	1
0	0	0	0	0	0	0	2	0	0	0	0	2	0
0	0	0	0	0	0	0	1	0	0	0	0	0	1
0	0	0	0	0	0	0	0	0	0	0	0	0	0
0	0	0	0	0	0	0	0	0	0	0	0	0	0
0	0	0	0	0	2	0	1	0	0	1	0	0	0
0	0	0	0	0	0	0	0	0	0	0	0	0	0
0													
OTU156	2	12	12	4	2	12	12	4	22	21	1	9	0
3	5	0	1	1	14	1	0	0	1	1	12	0	20
20	0	1	3	0	8	0	4	3	3	0	2	8	21
9	7	0	7	24	3	12	0	0	0	130	4	2	0
0	0	9	6	4	2	0	0	1	3	0	2	10	2
0	2	22	22	11	4	57	4	10	14	9	32	19	24
26	1	42	11	22	15	17	22	2	1	2	0	4	2
6	9	23	15	23	95	6	9	31	23	2	6	3	26
11													
OTU176	5	4	4	0	0	1	9	3	3	1	1	2	1
2	2	0	4	19	2	0	3	5	33	2	4	2	1
0	3	0	3	1	2	14	0	0	6	9	1	3	0
5	10	0	229	2	3	1	8	6	2	3	2	4	0
1	7	12	2	5	8	17	6	2	1	3	0	8	0
7	11	1	1	2	27	28	10	24	5	14	12	29	6
4	28	13	1	18	1	14	0	0	1	1	11	9	0
0	36	12	1	6	7	2	1	4	5	3	1	0	6
7													
OTU1107	6	5	8	10	28	12	11	14	23	24	11	17	3
9	28	16	7	24	13	5	16	8	15	3	11	7	10
5	8	25	13	6	14	0	5	6	6	9	11	23	9

13	10	17	7	6	4	5	4	15	31	3	7	3	4
6	7	9	14	7	11	5	11	15	7	15	7	10	7
28	7	17	2	24	6	2	24	1	14	6	52	4	7
6	3	9	5	7	24	7	15	3	10	13	4	4	2
8	16	10	9	12	11	0	6	3	3	10	1	2	14
32													
OTU283	3	2	1	0	0	0	0	0	30	43	0	0	0
0	0	1	0	0	0	21	3	0	0	0	1	0	0
1	0	0	0	0	0	0	0	0	1	0	0	1	0
0	0	0	0	0	0	0	0	0	0	0	0	0	0
0	1	0	0	0	0	0	0	0	0	0	0	0	0
0	2	40	0	10	0	0	1	0	20	0	0	5	0
0	0	0	1	0	2	0	4	0	0	0	0	6	0
0	0	0	1	2	6	4	0	2	2	0	0	0	4
0													
OTU12211	1	0	0	0	0	0	0	0	0	0	0	0	0
0	0	0	0	0	0	0	0	0	0	0	0	0	0
0	0	0	0	0	0	0	0	0	0	0	0	0	0
0	0	0	0	0	0	0	0	0	0	0	0	0	0
0	0	0	0	0	0	0	0	0	0	0	0	0	0
0	0	0	0	0	0	0	0	0	0	0	0	0	0
0	0	0	0	0	0	0	0	0	0	0	0	0	0
0	0	0	0	0	0	0	0	0	0	0	0	0	0
0													
OTU11512	1	0	0	0	0	1	2	0	0	0	0	0	0
0	2	0	0	0	0	2	0	0	0	0	0	0	0
0	0	0	0	0	0	0	0	0	0	0	0	2	0
0	0	0	0	0	0	0	0	0	0	0	0	0	0
0	1	0	0	0	0	0	0	0	0	0	0	0	0
0	0	0	0	0	0	0	0	0	0	0	0	0	0
0	0	0	0	0	0	0	0	0	0	0	0	0	0
0	0	0	0	0	0	0	0	0	0	0	0	0	0
0													

OTU3155	2	0	0	0	0	0	0	2	0	2	0	0	1
0	1	0	0	1	0	0	0	0	0	0	0	0	0
0	0	2	0	0	0	0	0	0	0	0	0	2	0
0	0	0	0	0	0	0	0	1	1	0	0	0	1
0	1	0	0	0	0	0	0	1	0	0	2	2	0
0	0	0	0	0	0	0	0	0	0	0	4	0	2
0	0	0	0	1	0	1	0	0	1	0	0	0	0
0	2	0	0	0	4	1	0	0	1	0	0	0	1
1													
OTU884	23	10	22	13	62	15	21	18	22	31	20	12	12
18	88	25	19	30	19	11	20	12	20	13	14	20	10
10	8	42	22	10	18	6	16	9	18	15	22	39	22
31	8	26	22	10	12	6	4	26	72	9	10	6	7
15	12	22	23	9	23	9	16	34	16	22	13	25	11
67	25	24	8	52	9	15	52	12	14	10	63	8	21
21	11	4	16	26	44	2	14	8	10	19	8	13	10
10	31	15	9	19	20	7	10	16	16	9	7	5	20
84													
OTU14854	8	0	12	0	0	0	0	0	0	0	0	0	0
0	0	0	1	0	0	0	0	0	0	0	0	0	0
3	0	0	0	0	0	0	0	0	0	0	0	0	0
1	0	0	0	0	0	0	0	0	0	0	35	0	0
0	0	0	0	0	0	0	9	1	0	1	0	2	0
0	2	0	0	0	0	0	0	0	0	0	8	0	1
0	5	0	1	0	0	0	3	0	3	0	0	1	0
0	0	0	0	0	0	0	0	0	0	0	0	0	0
0													
OTU14244	13	0	0	0	0	0	0	18	0	0	0	0	0
0	0	0	0	0	0	0	0	0	0	0	0	0	0
0	0	0	0	0	1	0	0	0	0	0	0	0	0
0	0	0	0	0	0	9	0	0	0	0	0	0	0
0	0	0	0	0	0	0	0	0	0	0	0	0	0
0	0	0	0	0	0	0	0	0	0	0	0	0	0

0	0	0	0	0	0	0	0	0	0	0	0	0	0
0	0	0	0	0	0	0	0	0	0	0	4	0	0
0													
OTU1152	4	0	0	0	0	0	0	0	1	0	0	0	0
0	2	0	0	0	0	0	0	0	0	0	0	0	0
0	0	0	0	0	0	0	0	0	0	0	0	0	0
0	0	0	0	0	0	0	0	0	0	0	0	0	0
0	0	0	0	0	0	0	0	0	0	0	0	0	0
0	0	0	0	0	0	0	0	0	0	0	0	0	0
0	0	0	0	0	0	0	0	0	0	0	0	0	0
0	0	0	0	0	0	0	0	0	0	0	0	0	0
0													
OTU5453	3	0	2	2	8	3	3	2	5	4	2	5	1
7	7	2	0	4	0	0	3	5	4	0	4	1	0
1	1	8	0	2	1	0	4	2	4	3	4	7	3
2	5	2	3	3	1	2	0	2	9	1	3	3	0
2	0	2	4	0	1	2	3	3	1	1	3	4	1
5	6	2	1	6	1	0	2	0	2	2	6	2	3
7	1	2	0	3	5	1	3	1	0	1	0	3	1
4	5	1	3	4	2	2	4	1	0	1	0	1	1
12													
OTU14274	1	0	0	0	0	0	0	0	0	0	0	0	0
0	0	0	0	0	0	0	0	0	0	0	0	0	0
0	0	0	0	0	0	0	0	0	0	0	0	0	0
0	0	0	0	0	0	0	0	0	0	0	0	0	0
0	0	0	0	0	0	0	0	0	0	0	0	0	0
0	0	0	0	0	0	0	0	0	0	0	0	0	0
0	0	0	0	0	0	0	0	0	0	0	0	0	0
0	0	0	0	0	0	0	0	0	0	0	0	0	0
0													
OTU11157	1	0	0	0	0	0	0	0	0	0	0	0	0
0	0	0	0	0	0	0	0	0	0	0	0	0	0
0	0	0	0	0	0	0	0	0	0	0	0	0	0

0 0 0 0 0 0 0 0 0 0 0 0 0 0
0 0 0 0 0 0 0 0 0 0 0 0 0 0
0 0 0 0 0 0 0 0 0 0 0 0 0 0
0 0 0 0 0 0 0 0 1 0 0 0 0 0
0 0 0 0 0 0 0 0 0 0 0 0 0 0
0
OTU831 14 10 16 19 35 12 10 14 24 36 13 16 10
11 32 22 7 39 15 12 18 13 18 6 8 11 16
12 8 44 21 12 20 2 16 8 15 12 15 61 15
24 17 20 15 6 9 5 8 22 51 10 4 14 6
13 8 16 17 8 29 9 10 13 18 19 15 20 9
45 11 27 11 54 12 9 42 13 10 8 74 7 14
17 9 11 14 11 32 5 9 5 9 34 7 12 9
14 14 13 16 18 18 9 17 10 12 4 5 3 15
45
OTU15209 1 0 0 0 0 1 1 0 0 0 0 0 0
0 0 0 0 0 0 0 0 0 0 0 0 0 2
0 0 0 0 0 0 0 0 1 0 0 0 0 0
0 0 0 0 0 1 0 0 0 0 0 0 0 0
0 0 0 1 0 0 0 0 0 0 0 0 0 0
0 0 0 0 1 1 0 0 0 0 1 0 0 0
0 0 0 1 0 1 0 0 0 0 0 0 0 0
0 0 0 0 0 0 0 0 0 0 0 0 0 0
0
OTU12159 2 2 4 0 0 3 0 1 0 2 0 0 2
1 1 1 0 1 0 2 2 0 0 0 1 0 1
1 0 0 3 1 3 2 1 0 1 0 0 0 2
3 2 0 0 2 0 0 0 0 0 2 1 0 0
0 0 0 1 0 0 1 0 0 0 0 4 2 0
0 0 0 3 3 3 2 0 3 0 0 1 1 0
1 0 2 0 1 0 2 1 0 3 0 0 0 1
1 0 0 0 0 0 0 0 0 0 0 0 0 0
0

OTU170		8	5	9	13	1	4	0	15	11	36	0	14	1
	6	2	21	0	1	4	19	43	2	1	19	0	2	2
	9	0	1	7	3	0	8	3	1	20	0	5	8	4
	4	12	1	19	23	3	3	2	0	0	13	0	2	1
	0	7	1	19	8	1	10	1	1	8	1	24	3	11
	0	4	5	5	9	0	0	5	11	11	4	2	7	1
	6	4	1	12	8	0	12	0	8	25	8	1	0	2
	8	10	3	26	3	3	2	1	1	4	6	1	9	8
	2													
OTU508		2	0	0	10	0	0	0	0	0	0	0	0	0
	0	0	0	3	0	0	1	1	3	0	14	0	1	0
	4	0	0	0	1	0	3	0	0	1	2	0	0	0
	0	0	0	0	0	0	1	0	0	6	0	0	0	0
	0	2	1	0	0	0	0	0	0	0	0	0	0	0
	0	0	0	0	2	0	0	0	0	0	0	0	0	0
	0	0	0	0	0	0	0	1	0	4	0	0	0	0
	0	0	0	0	0	0	0	0	0	0	0	0	0	0
	0													
OTU69	8	4	8	7	6	7	5	19	12	2	11	11	4	2
	9	7	55	6	4	55	9	10	3	16	4	8	9	5
	4	9	4	7	1	10	3	8	4	63	5	16	1	20
	6	3	6	6	41	23	24	4	9	5	7	4	29	3
	6	2	19	4	5	2	4	1	6	6	8	26	2	7
	8	6	3	5	3	16	4	19	11	3	4	2	5	22
	7	4	14	3	6	2	15	8	6	5	5	13	4	11
	15	15	10	13	10	11	105	12	9	4	9	14	148	1174
OTU890		1	0	0	0	0	0	0	0	0	0	0	0	0
	0	0	0	0	0	0	0	0	0	0	0	0	0	0
	0	0	0	0	0	0	0	0	0	0	0	0	0	0
	0	0	0	0	0	0	0	0	0	0	0	0	0	0
	0	0	0	0	0	0	0	0	0	0	0	0	0	0
	0	1	0	0	0	0	0	0	0	0	0	0	0	1
	0	0	0	0	0	0	0	0	0	0	0	1	1	0

0	0	0	0	0	0	0	3	0	0	0	0	0	1
0													
OTU3461	3	20	2	0	0	2	29	8	26	6	0	0	0
0	0	0	0	0	0	0	0	0	0	0	0	0	0
0	16	0	0	0	0	0	0	0	0	0	0	0	0
6	0	11	0	0	0	0	0	0	0	1	0	0	0
0	0	0	0	0	1	0	2	1	0	0	0	3	0
1	1	7	0	0	0	0	33	0	12	0	3	0	0
0	51	0	3	0	0	0	10	0	0	0	0	0	0
0	0	0	0	2	0	0	2	0	1	0	0	0	0
0													
OTU2223	1	1	6	0	2	0	3	2	17	21	1	7	1
0	0	1	0	3	3	12	52	0	0	0	0	1	0
69	0	0	3	0	0	0	5	0	6	1	0	0	6
1	5	0	0	0	1	1	0	0	1	0	0	0	0
0	10	0	1	0	1	1	1	2	0	0	0	2	0
2	6	28	2	0	1	1	0	2	1	1	5	2	0
3	3	1	14	1	5	0	0	0	0	9	5	4	0
0	4	3	6	3	2	8	14	3	6	2	0	0	10
1													
OTU4302	4	0	0	0	0	0	2	0	0	0	0	1	0
0	0	1	0	0	0	1	3	0	11	1	0	1	0
3	0	0	0	1	1	0	0	0	6	0	0	0	0
0	2	0	3	0	1	0	0	5	1	4	40	2	0
1	0	0	14	1	2	6	4	12	1	0	0	2	0
0	0	0	3	0	3	24	10	9	1	4	2	13	14
0	3	0	2	3	3	0	0	0	0	0	0	0	0
0	0	0	0	0	0	0	1	0	0	0	0	0	0
0													
OTU240	19	0	0	5	0	0	2	1	1	3	33	5	143
14	1	42	0	3	2	0	97	6	2	1	11	2	19
0	0	3	37	0	29	2	5	5	12	2	8	0	1
1	35	1	26	0	1	7	1	9	1	10	1	11	1

19 0 1 0 1 6 2 2 4 7 5 2 0 0
0 1 1 39 33 37 23 0 66 4 2 9 30 8
10 5 33 0 14 0 12 0 16 4 24 0 0 6
36 0 2 0 0 0 0 0 2 0 11 26 11 0
0
OTU14676 2 0 1 0 2 0 1 1 2 0 1 0 0
0 0 0 0 2 0 0 1 0 1 0 0 0 0
0 0 1 0 0 1 0 0 1 0 0 2 1 1
2 1 0 1 0 0 0 0 1 1 0 1 1 0
1 0 0 0 0 0 0 0 0 0 0 1 0 0
1 1 2 0 0 0 0 3 1 0 0 1 0 1
0 0 0 0 0 2 0 0 1 0 0 0 1 0
0 2 0 0 1 0 0 2 0 0 0 0 0 0
0
OTU9877 2 2 2 0 0 0 2 8 11 6 0 0 0
0 5 1 0 0 0 3 0 0 0 0 0 0 0
3 0 0 0 0 0 0 0 0 0 1 0 1 0
17 0 0 0 0 0 0 0 0 0 0 0 0 0
0 0 0 0 0 0 0 0 0 0 0 0 0 0
0 0 1 0 1 1 0 0 1 1 0 0 0 2
0 0 0 14 0 2 1 0 0 0 0 0 0 0
0 2 0 0 0 0 0 0 0 0 0 0 1 0
0
OTU12391 2 0 0 1 0 0 0 3 0 5 0 0 0
1 0 1 0 2 0 0 1 1 0 0 0 0 1
0 0 3 0 1 1 0 0 0 0 0 0 0 1
0 0 1 0 0 0 0 0 1 0 1 0 0 0
0 1 0 0 0 0 0 1 0 0 0 0 0 0
1 0 0 0 2 0 0 1 0 0 0 3 0 1
0 0 0 2 0 2 0 0 0 0 0 0 0 0
0 0 0 0 1 1 0 0 0 0 0 0 0 0
0

OTU1093	1	0	0	0	0	1	1	2	0	0	0	0	1
2	0	1	0	0	0	0	1	0	1	30	0	0	0
0	0	3	0	1	3	0	0	1	0	0	0	0	0
0	1	1	0	0	0	4	0	0	0	0	2	0	1
0	0	0	0	0	0	1	0	1	0	0	2	0	1
0	0	0	1	0	1	7	0	2	0	5	0	3	0
0	0	9	0	0	3	2	0	8	1	0	0	0	0
1	0	0	0	0	0	0	0	0	0	0	3	9	0
0													
OTU6248	1	1	0	0	0	0	0	0	0	0	0	0	0
0	0	0	0	0	0	0	0	0	0	0	0	0	0
0	0	0	0	0	0	0	1	0	0	0	0	0	0
0	0	0	0	0	1	0	0	1	0	0	0	0	0
0	0	0	0	0	0	1	0	0	1	0	0	1	0
0	0	0	0	0	0	0	0	0	0	0	2	0	0
0	0	0	0	0	0	0	0	0	1	0	0	0	0
0	0	1	0	0	0	0	0	0	0	0	0	0	0
1													
OTU11015	1	0	0	0	0	0	0	0	0	0	0	0	0
0	0	0	0	0	0	0	0	0	0	0	0	0	0
0	0	0	0	0	0	0	0	0	0	0	0	0	0
0	0	0	0	0	0	0	0	0	0	0	0	0	0
0	0	0	0	0	0	0	0	0	0	0	0	0	0
0	0	0	0	0	0	0	0	0	0	0	0	0	0
0	0	0	0	0	0	0	0	0	0	0	0	0	0
0	0	0	0	0	0	0	0	0	0	0	0	0	0
0													
OTU15787	1	0	1	0	0	0	0	0	0	1	0	0	0
0	0	0	0	0	0	0	0	0	0	0	0	0	0
0	0	0	0	0	0	0	0	0	0	0	0	0	0
0	0	0	0	0	0	0	0	0	0	0	0	0	0
0	0	0	0	0	0	0	0	0	0	0	0	0	0
0	0	0	0	0	0	0	0	0	0	0	0	0	0

0	0	0	0	0	0	0	0	0	0	0	0	0	0
0	0	0	0	0	0	0	0	0	0	0	0	0	0
0													
OTU4935	2	1	0	0	2	0	1	2	0	0	0	0	0
0	1	1	0	1	2	0	0	1	2	0	1	0	0
1	0	3	3	2	1	0	0	0	0	0	2	3	0
0	2	1	1	0	3	1	0	0	2	0	0	0	0
0	1	2	1	0	0	2	3	1	0	0	1	0	0
0	0	0	0	1	0	0	1	0	0	1	4	0	1
0	0	0	2	1	1	1	4	0	0	3	0	0	0
0	2	2	0	0	1	0	0	0	1	0	0	1	1
3													
OTU16568	4	0	0	0	0	0	4	2	0	0	0	0	1
0	0	0	0	0	0	0	0	1	0	15	0	0	0
0	0	0	0	0	0	0	0	12	0	1	10	0	0
0	0	2	0	0	0	2	0	0	0	0	0	0	0
0	0	0	0	0	0	0	0	0	0	0	0	0	1
0	0	0	0	0	0	0	0	0	0	0	0	0	0
0	0	0	0	0	0	0	0	2	0	0	0	0	0
0	0	0	0	0	0	0	0	0	0	0	2	0	0
0													
OTU842	2	0	3	5	0	3	2	0	0	1	16	11	10
5	3	2	0	7	2	12	1	0	0	0	0	0	0
2	0	0	0	0	21	0	25	46	2	0	10	1	0
1	8	0	6	0	3	19	5	0	0	7	0	0	26
0	18	0	2	1	0	0	0	0	0	0	1	0	1
0	9	1	5	1	7	6	0	10	5	1	0	5	4
20	0	5	2	41	1	3	1	2	3	24	7	2	10
30	0	2	4	0	0	0	18	5	1	20	12	3	1
1													
OTU1584	1	0	0	4	2	1	3	0	0	4	4	9	2
1	7	0	0	0	0	1	0	0	0	0	0	0	0
0	0	0	0	0	0	0	8	9	0	0	0	0	0

5 3 0 0 0 0 0 0 0 0 0 0 0 2
0 4 0 0 0 0 0 0 0 0 0 0 2 0
0 0 0 2 1 1 0 0 2 1 0 0 0 0
0 0 0 1 5 0 0 0 0 2 7 0 1 0
0 0 0 3 0 0 0 2 0 3 7 2 0 0
0
OTU74 8 319 35 102 27 19 13 19 13 3 13 16 8 2
96 43 14 186 15 4 67 0 138 17 239 30 218 0
2 125 192 9 125 25 9 24 289 12 273 3 57 1
12 0 158 8 4 24 0 57 4 3 60 8 11 5
7 6 1 2 1 7 2 0 5 0 14 1 2 2
0 12 27 0 251 323 3 25 9 463 1 93 19 435
15 55 23 187 9 22 3 8 13 5 0 0 13 14
0 2 0 0 1 0 0 4 5 33 10 15 2 0
OTU4423 4 1 1 2 6 4 0 2 2 10 4 3 4
3 15 4 5 10 1 0 1 3 2 3 3 2 1
5 1 3 3 2 1 2 7 3 3 3 3 13 2
6 3 1 6 1 3 2 1 1 17 0 3 2 0
5 1 2 2 1 2 0 2 3 2 2 4 2 2
9 4 2 2 3 2 1 7 3 4 1 4 2 0
5 2 1 1 1 7 0 1 1 2 3 2 2 3
3 7 2 2 3 1 1 5 0 2 5 1 3 3
21
OTU10969 2 0 0 0 0 0 0 0 0 0 0 0 0
0 0 0 0 0 0 0 0 0 0 0 0 0 0
0 0 0 0 0 0 0 0 0 0 0 0 0 0
0 0 0 0 0 0 0 0 0 0 0 0 0 0
0 0 0 0 0 0 0 0 0 0 0 0 0 0
0 0 0 0 0 0 0 0 0 0 0 0 0 0
0 0 0 0 0 0 0 0 0 0 0 0 0 0
0 0 0 0 0 0 0 0 0 0 0 0 0 0
0

OTU961	19	27	139	0	0	0	0	1	0	0	2	0	0
0	0	0	0	0	0	0	0	0	0	0	0	0	0
0	0	0	0	0	0	0	0	0	0	0	46	0	45
0	0	3	0	22	0	0	0	0	0	0	0	0	0
0	0	0	0	0	0	0	0	1	0	1	0	1	0
2	0	0	0	3	0	0	1	0	1	0	1	0	2
0	13	0	0	0	8	0	6	0	0	0	0	1	0
0	0	0	0	0	34	0	0	0	20	0	0	0	0
0													
OTU14270	1	0	0	0	0	0	0	0	0	0	0	0	0
0	0	0	0	0	0	0	0	0	0	0	0	0	0
0	0	0	0	0	0	0	0	0	0	0	0	0	0
0	0	0	0	0	0	0	0	0	0	0	0	0	0
0	0	0	0	0	0	0	0	0	0	0	0	0	0
0	0	0	0	0	0	0	0	0	0	0	0	0	0
0	0	0	0	0	0	0	0	0	0	0	0	0	0
0	0	0	0	0	0	0	0	0	0	0	0	0	0
0													
OTU213	12	1	16	0	6	14	5	12	5	7	0	4	3
0	1	3	4	34	2	3	27	0	1	1	1	6	2
8	0	0	16	8	2	0	2	0	71	0	5	11	0
0	5	0	3	0	0	6	26	1	0	5	1	3	5
0	21	0	10	1	0	1	1	0	4	0	8	10	4
1	2	13	7	3	0	3	0	2	0	0	1	5	0
2	3	1	10	1	14	1	3	0	62	0	0	0	0
0	0	1	3	0	1	0	0	0	1	1	1	4	0
0													
OTU493	4	0	2	4	4	0	0	4	1	1	0	0	0
0	0	0	0	0	0	0	0	0	0	0	0	0	0
0	0	0	0	0	0	0	0	1	5	0	0	0	0
0	0	0	0	0	0	0	0	0	0	0	5	0	0
0	0	0	0	0	0	0	0	0	0	0	0	1	0
0	0	1	0	0	0	0	0	0	0	0	0	0	0

0 0 0 0 0 0 0 0 0 17 0 0 0 0
0 0 0 0 0 0 0 0 0 2 0 0 0 0
0
OTU12569 2 0 0 1 1 0 0 2 0 1 0 2 1
0 4 1 0 0 1 0 0 0 0 0 0 2 0
0 0 2 4 1 1 0 2 0 0 1 0 2 0
2 3 1 1 0 0 0 0 1 0 1 0 1 1
0 0 0 0 0 1 0 1 1 0 0 0 1 0
3 0 0 0 2 1 0 2 0 0 0 0 0 0
0 1 0 1 0 1 0 0 0 0 3 0 0 1
0 1 2 2 0 2 0 0 0 1 0 0 0 1
4
OTU246 9 4 15 1 0 3 2 1 14 3 0 1 0
0 2 0 0 0 0 1 0 0 0 0 0 1 3
0 0 0 0 0 0 1 0 1 0 0 0 0 0
4 1 0 0 0 0 2 0 0 0 0 1 0 0
0 0 0 2 0 0 0 0 0 0 0 0 2 0
0 9 1 0 8 0 1 3 1 6 0 3 0 5
1 4 0 1 0 6 3 8 0 0 1 5 1 0
8 7 22 13 22 41 6 5 8 25 1 1 0 5
0
OTU669 11 33 21 7 5 6 13 22 7 15 2 6 2
2 15 15 3 6 3 5 0 1 0 5 0 14 42
7 1 1 16 9 0 5 0 40 2 0 0 5 0
18 27 0 21 18 7 0 7 1 0 12 0 1 3
1 12 1 14 4 0 4 0 2 2 1 11 19 2
0 7 3 1 6 49 9 1 54 3 67 2 1 1
25 2 35 13 0 10 31 4 2 14 0 8 11 0
5 11 8 10 13 4 1 8 3 30 11 0 1 5
11
OTU2747 2 0 1 0 0 0 3 0 0 4 0 0 0
0 1 0 0 0 0 6 7 0 0 0 2 0 2
0 0 0 0 0 0 0 1 0 0 0 0 0 0

	0	0	0	0	0	0	0	0	0	0	0	0	0	0
	0	0	0	1	0	0	0	0	0	0	0	0	0	0
	0	0	1	0	0	0	0	0	0	4	0	1	0	0
	0	0	0	0	0	0	0	0	0	0	1	2	1	0
	6	0	0	0	0	0	0	0	0	0	0	0	1	0
	3													
OTU58	3	1	0	0	2	1	3	1	2	0	280	12	257	69
	0	69	2	5	417	0	33	0	245	0	59	0	1	0
	0	758	32	0	45	0	66	0	5	0	12	0	21	0
	27	0	11	0	0	368	0	0	0	1	0	0	14	0
	2	0	0	1	0	1	12	0	0	0	0	0	1	0
	0	0	67	1	18	6	0	6	1	4	0	3	0	8
	0	193	0	5	0	2	1	294	8	87	0	0	231	15
	0	10	0	0	0	0	12	35	0	8	325	3	0	0
OTU3240		2	0	0	0	1	2	0	2	4	5	3	1	3
	1	6	1	0	0	1	0	2	2	1	1	0	0	2
	0	0	4	0	0	1	1	0	0	2	2	0	3	1
	4	1	1	1	0	0	1	2	2	3	0	2	1	0
	0	0	1	2	0	1	0	0	3	1	0	1	2	1
	6	3	0	1	4	1	0	3	0	1	1	8	1	1
	0	1	0	2	1	4	0	0	0	2	0	1	1	0
	0	0	0	2	3	1	1	1	1	0	1	0	0	1
	7													
OTU11014		1	0	0	0	0	0	0	0	0	0	0	0	0
	0	0	0	0	0	0	0	0	0	0	0	0	0	0
	0	0	0	0	0	0	0	0	0	0	0	0	0	0
	0	0	0	0	0	0	0	0	0	0	0	0	0	0
	0	0	0	0	0	0	0	0	0	0	0	0	0	0
	0	0	0	0	0	0	0	0	0	0	0	0	0	0
	0	0	0	0	0	0	0	0	0	0	0	0	0	0
	0	0	0	0	0	0	0	0	0	0	0	0	0	0
	0													

OTU226		5	9	20	0	2	3	15	2	31	49	0	5	0
	3	3	1	0	5	0	18	0	0	0	0	9	3	4
	17	2	5	31	1	0	1	0	1	0	0	2	0	6
	20	4	0	5	8	5	13	1	0	0	15	9	0	1
	0	8	0	32	3	1	0	1	1	0	1	0	15	3
	1	12	5	2	8	1	1	2	1	15	0	5	60	30
	2	4	0	8	6	17	6	15	0	3	1	2	8	0
	0	37	8	6	9	3	5	2	9	9	7	4	10	31
	13													
OTU253		11	0	33	0	0	18	3	0	0	0	0	8	0
	0	0	0	0	0	0	36	0	0	0	0	0	0	0
	10	0	0	1	0	0	0	0	0	0	0	0	8	0
	6	24	0	4	0	0	0	0	0	1	0	0	0	0
	0	1	0	40	0	1	0	0	0	0	1	0	53	0
	1	0	6	0	7	89	114	0	29	12	1	46	1	4
	0	5	0	0	32	13	0	0	0	0	0	1	0	0
	0	9	0	0	14	1	0	2	0	0	5	1	0	0
	3													
OTU3506		3	0	0	0	1	1	2	0	1	0	0	0	0
	0	4	0	0	0	0	0	0	0	0	0	0	2	0
	0	0	0	0	0	0	0	0	0	0	0	0	0	0
	1	0	0	0	0	8	0	2	0	0	0	1	0	0
	0	0	0	7	0	0	0	0	0	1	0	0	3	0
	0	0	0	0	0	0	0	0	0	0	1	0	0	2
	0	1	0	0	0	0	0	0	0	0	0	0	0	0
	0	0	0	0	0	0	0	0	0	0	0	0	0	0
	0													
OTU296		6	11	4	42	28	3	6	8	47	3	0	0	0
	0	18	0	1	0	0	0	0	0	0	0	0	1	0
	25	0	0	0	0	0	0	0	1	4	0	0	1	0
	4	0	0	9	0	2	0	11	0	2	0	6	0	0
	0	3	0	0	0	0	0	0	2	0	1	0	13	0
	1	0	0	0	0	0	0	5	1	1	0	2	2	7

	0	0	0	12	0	1	0	0	0	0	0	2	2	0
	0	3	0	0	3	2	5	0	1	0	0	0	0	0
	2													
OTU4737		9	4	5	4	20	5	6	12	10	17	8	3	3
	1	22	6	1	11	4	3	8	1	8	5	2	5	4
	3	2	12	6	5	4	4	2	1	8	4	9	3	12
	6	6	5	7	5	3	0	2	10	10	3	4	1	5
	0	1	6	8	4	4	1	1	7	3	5	2	8	0
	13	7	2	1	11	2	1	14	6	5	2	17	4	6
	6	5	4	2	4	11	1	3	1	5	4	10	7	6
	1	0	5	4	5	7	0	1	2	5	4	5	3	3
	26													
OTU288		2	1	16	10	0	1	25	16	20	0	0	2	0
	0	0	0	16	0	0	0	4	4	0	1	0	0	0
	1	17	0	0	0	0	0	0	0	1	0	0	3	0
	8	10	2	0	0	6	0	0	14	38	3	10	11	0
	1	3	0	5	1	6	1	8	8	0	0	0	2	0
	5	2	5	0	0	0	0	3	0	0	0	3	0	18
	1	41	0	8	0	5	0	73	0	0	1	5	3	0
	0	3	6	3	6	2	0	5	8	9	1	0	0	9
	2													
OTU7251		1	0	0	0	0	0	0	0	0	0	0	0	0
	0	0	0	0	0	0	0	0	0	0	0	0	0	0
	0	0	0	0	0	0	0	0	0	0	0	0	0	0
	0	0	0	0	0	0	0	0	0	0	0	0	0	0
	0	0	0	0	0	0	0	0	0	0	0	0	0	0
	0	0	0	0	1	0	0	0	0	0	0	0	0	0
	0	0	0	0	0	0	0	0	0	0	0	0	0	0
	0	0	0	0	0	0	0	0	0	0	0	0	0	0
	0													
OTU214		3	34	9	1	4	5	3	3	12	1	0	3	0
	0	3	0	0	0	0	3	1	0	0	0	14	6	5
	1	0	0	1	2	1	1	2	2	2	12	1	5	0

19	2	0	18	1	3	1	0	0	0	2	0	0	0
1	17	0	2	0	0	0	0	1	1	0	3	2	1
1	2	13	1	5	5	0	3	2	7	3	5	4	11
8	6	1	3	0	1	1	4	0	0	0	0	5	0
0	0	9	3	20	12	1	8	18	33	3	1	4	56
0													
OTU12222	1	0	0	0	0	0	0	0	0	0	0	0	0
0	0	0	0	0	0	0	0	0	0	0	0	0	0
0	0	0	0	0	0	0	0	0	0	0	0	0	0
0	0	0	0	0	0	0	0	0	0	0	0	0	0
0	0	0	0	0	0	0	0	0	0	0	0	0	0
0	0	0	0	0	0	0	0	0	0	0	0	0	0
0	0	0	0	0	0	0	0	0	0	0	0	0	0
0	0	0	0	0	0	0	0	0	0	0	0	0	0
0													
OTU1158	2	0	0	11	9	8	0	3	0	0	2	0	0
0	0	0	0	0	0	0	0	0	0	1	0	8	21
0	0	1	0	0	0	1	0	9	0	0	1	1	0
0	0	0	1	0	7	0	2	0	0	1	0	0	0
0	0	0	6	0	0	1	0	0	1	0	11	0	0
0	0	0	2	0	0	1	0	0	0	0	0	0	2
0	0	0	0	0	0	7	0	0	0	0	0	0	0
2	0	0	0	0	0	0	0	0	0	0	0	0	0
0													
OTU1530	1	0	0	0	0	0	0	0	1	0	0	1	
0	0	0	0	0	0	0	0	0	0	0	0	0	0
0	0	0	1	0	0	0	0	0	0	0	0	1	0
2	0	0	0	0	0	0	0	0	0	0	0	0	0
0	0	0	0	0	0	0	0	0	0	0	1	0	0
0	0	0	0	0	0	0	2	0	1	0	0	0	0
0	0	0	0	0	0	0	0	0	0	0	0	0	0
0	0	0	0	0	0	0	1	0	0	1	0	0	1
0													

OTU1098 1 0 0 0 1 1 0 0 0 0 0 0 0
0 0 0 0 0 0 0 0 0 0 0 0 0 13
0 0 0 0 0 0 0 0 1 0 0 0 0 0
0 0 0 0 0 0 0 0 0 0 0 0 0 6
0 0 1 4 0 0 0 0 0 0 0 2 0 0
0 0 0 2 0 0 0 0 0 0 0 0 0 0
0 0 0 0 0 0 0 0 0 0 0 0 0 0
0 0 0 0 0 0 0 0 0 0 0 0 0 0
0
OTU93 10 4 5 2 4 0 26 3 1 21 1 3 0 1
7 0 0 3 1 4 0 0 2 0 10 0 3 1
0 0 0 0 0 1 2 1 0 98 0 4 0 4
22 0 17 1 0 4 12 0 0 0 0 0 0 0
7 0 3 0 0 0 0 3 0 0 1 5 1 0
287 4 1 8 23 4 2 3 4 43 3 18 3 2
1 6 10 7 3 6 2 0 4 0 843 181 1 1
79 22 91 138 59 77 67 29 88 2 13 7 175 209
OTU297 3 0 4 1 0 0 0 0 8 0 0 1 1
0 0 0 0 0 0 5 0 0 0 0 0 24 1
1 0 0 0 3 0 0 0 0 0 107 0 2 0
1 0 0 1 0 1 0 0 0 0 0 0 0 0
0 0 0 2 0 0 0 0 1 0 0 0 1 0
1 1 0 0 1 0 0 3 1 1 1 1 0 4
0 10 0 2 1 0 0 0 0 0 0 1 1 0
0 5 3 11 9 3 0 1 3 46 0 0 0 70
0
OTU618 5 3 3 0 0 0 1 2 0 0 1 0 0
0 2 0 0 0 0 2 2 0 1 1 2 0 15
0 0 0 2 0 0 0 0 0 0 0 0 0 0
0 1 0 0 1 0 0 0 0 0 0 0 0 0
0 1 0 0 0 0 0 0 1 0 1 2 0 0
0 0 8 0 0 0 0 0 0 0 0 0 1 0
0 1 0 2 0 6 0 3 0 1 0 0 1 0

0	0	0	0	0	0	0	0	0	1	0	0	1	0
0													
OTU1778	1	2	0	0	1	2	3	0	1	2	3	0	1
0	2	2	0	3	0	0	2	2	0	0	2	1	0
1	0	2	1	1	1	0	2	0	1	1	5	6	1
1	1	0	2	0	0	0	1	1	5	1	0	0	0
3	0	2	0	1	1	0	1	3	0	1	1	1	1
6	0	3	0	5	2	0	0	0	1	0	4	0	2
2	1	2	0	1	3	1	1	0	0	3	0	1	2
1	0	2	0	3	0	2	0	0	1	1	1	0	2
2													
OTU3470	2	0	3	0	1	2	2	0	4	5	0	1	0
0	0	0	11	1	0	12	0	11	0	1	0	2	0
2	10	0	0	0	0	0	0	1	0	0	0	13	0
0	3	13	0	0	0	0	1	1	20	1	13	0	0
5	5	0	5	0	5	0	8	15	0	6	1	4	0
1	0	1	1	0	2	0	4	0	2	0	7	0	0
0	1	0	5	0	2	0	0	0	0	0	0	0	0
0	0	0	0	0	0	0	0	0	0	1	0	3	0
0													
OTU46 11	2	3	0	0	0	1	0	64	12	0	1	5	7
3	1	10	0	0	7	27	17	2	14	1	1154	0	1704
10	0	0	1	1	238	0	0	3	0	0	524	0	19
0	0	0	0	0	0	0	0	0	0	0	2	0	0
0	0	0	0	0	70	0	67	0	1	1	6	0	27
0	0	0	0	0	0	0	0	14	0	0	0	0	0
591	0	0	0	8	0	0	0	0	0	1	0	0	0
0	0	1	2	4	1	0	0	1	2	0	88	2	0
OTU153	12	11	19	8	1	12	31	2	21	9	2	6	4
3	7	26	13	6	2	7	9	15	15	4	24	60	40
11	4	0	4	2	9	9	0	1	52	0	0	5	0
10	7	6	6	2	3	1	3	31	9	20	14	18	0
9	19	1	24	2	1	10	5	6	1	12	14	27	6

3	11	6	1	24	18	12	16	56	28	4	15	32	40
8	14	20	8	6	10	19	30	0	4	1	3	8	1
0	2	14	3	7	7	8	10	14	42	7	1	3	4
1													
OTU14272	1	0	0	0	0	0	0	0	0	0	0	0	0
0	0	0	0	0	0	0	0	0	0	0	0	0	0
0	0	0	0	0	0	0	0	0	0	0	0	0	0
0	0	0	0	0	0	0	0	0	0	0	0	0	0
0	0	0	0	0	0	0	0	0	0	0	0	0	0
0	0	0	0	0	0	0	0	0	0	0	0	0	0
0	0	0	0	0	0	0	0	0	0	0	0	0	0
0	0	0	0	0	0	0	0	0	0	0	0	0	0
0													
OTU4681	1	0	2	1	2	1	1	0	1	2	0	0	0
0	1	0	0	3	0	1	1	0	1	0	0	0	0
1	1	2	1	0	1	0	0	0	2	0	1	2	3
1	1	1	0	0	0	0	0	1	5	1	0	1	0
1	0	0	1	1	2	0	1	2	0	0	0	3	1
6	1	1	1	1	1	0	5	0	3	0	3	1	1
2	0	1	0	0	5	0	2	0	0	2	0	1	0
1	4	0	0	0	1	0	1	0	3	0	1	0	1
4													
OTU744	7	3	0	0	3	0	12	10	2	0	2	0	5
5	30	5	0	78	13	0	1	0	154	2	36	0	2
0	0	14	0	0	47	0	3	92	4	0	66	0	5
0	0	14	41	0	0	66	0	0	0	0	0	0	12
0	2	0	1	0	0	1	0	0	0	0	0	0	0
0	0	1	7	1	27	65	0	12	1	55	0	11	0
1	0	15	0	3	11	0	0	7	0	1	0	0	1
1	0	0	0	0	0	0	2	0	0	2	6	2	0
0													
OTU862	1	0	1	1	2	0	1	1	1	1	14	1	24
10	1	11	18	4	37	1	9	4	8	2	2	0	6

0	2	16	70	23	31	3	3	2	13	8	28	0	61
1	1	1	7	4	0	1	0	7	10	4	0	1	6
7	1	23	0	3	8	3	2	4	2	0	6	1	7
0	3	0	7	0	7	9	7	7	0	9	4	5	0
4	9	31	0	2	0	1	1	25	0	12	6	12	21
5	13	7	15	17	5	6	2	2	5	4	4	28	17
2													
OTU166	8	6	22	2	12	1	44	10	241	50	0	10	0
0	7	0	1	0	2	9	32	0	0	0	0	0	0
10	0	0	1	0	1	0	2	2	7	0	0	3	0
61	8	1	41	2	23	0	1	0	1	0	7	1	2
0	2	0	7	0	5	0	1	1	0	1	0	4	0
2	53	167	0	96	0	0	13	2	129	1	24	14	24
2	1	1	103	6	35	0	125	0	2	9	82	43	2
0	56	56	41	156	66	26	94	16	35	0	11	0	81
22													
OTU14271	1	0	0	0	0	0	0	0	0	0	0	0	0
0	0	0	0	0	0	0	0	0	0	0	0	0	0
0	0	0	0	0	0	0	0	0	0	0	0	0	0
0	0	0	0	0	0	0	0	0	0	0	0	0	0
0	0	0	0	0	0	0	0	0	0	0	0	0	0
0	0	0	0	0	0	0	0	0	0	0	0	0	0
0	0	0	0	0	0	0	0	0	0	0	0	0	0
0	0	0	0	0	0	0	0	0	0	0	0	0	0
0													
OTU12221	1	0	0	0	0	0	0	0	0	0	0	0	0
0	0	0	0	0	0	0	0	0	0	0	0	0	0
0	0	0	0	0	0	0	0	0	0	0	0	0	0
0	0	0	0	0	0	0	0	0	0	0	0	1	0
0	0	0	0	0	0	0	0	0	0	0	0	0	0
0	0	0	0	0	0	0	0	0	0	0	0	0	0
0	0	0	0	0	0	0	0	0	0	0	0	1	0

1	0	0	0	0	0	0	0	0	0	0	0	0	0
0													
OTU11006	1	0	0	0	0	0	0	0	0	0	0	0	0
0	0	0	0	0	0	0	0	0	0	0	0	0	0
0	0	0	0	0	0	0	0	0	0	0	0	0	0
0	0	0	0	0	0	0	0	0	0	0	0	0	0
0	0	0	0	0	0	0	0	0	0	0	0	0	0
0	0	0	0	0	0	0	0	0	0	0	0	0	0
0	0	0	0	0	0	0	0	0	0	0	0	0	0
0	0	0	0	0	0	0	0	0	0	0	0	0	0
0													
OTU1907	2	3	1	0	0	0	1	0	2	4	0	1	0
0	2	0	0	0	1	0	1	2	0	1	1	5	2
1	0	2	0	4	2	3	0	0	0	2	0	10	2
1	2	0	1	12	5	0	5	0	0	7	0	0	12
0	2	1	1	1	0	0	2	2	1	0	14	4	0
0	1	10	3	1	2	0	0	2	2	0	0	4	0
1	0	3	2	2	0	0	4	0	0	0	0	1	0
3	0	0	4	0	5	0	3	0	6	2	0	3	0
1													
OTU204	4	1	0	0	0	0	26	0	1	0	0	0	1
0	0	0	0	0	0	0	0	0	0	1	0	0	0
0	0	0	0	1	0	1	0	0	0	1	12	0	0
0	0	4	0	1	0	1	9	2	0	0	0	0	0
0	0	0	2	1	0	0	0	1	0	5	0	0	3
0	0	483	0	0	0	0	0	0	0	0	0	0	3
0	1	0	0	0	0	0	0	0	0	0	0	2	1
0	0	0	0	0	0	0	137	0	0	0	0	0	0
1													
OTU12012	2	1	5	1	2	4	1	1	7	1	6	2	0
1	9	3	0	2	0	2	1	1	1	3	1	5	2
2	0	7	4	1	1	1	1	0	1	1	3	6	3
1	3	1	1	1	0	0	1	1	8	1	3	1	0

7	0	3	0	0	3	0	3	5	0	3	2	2	2
5	0	3	0	5	2	1	5	1	0	2	7	1	1
2	1	0	4	1	4	1	2	1	2	2	0	0	2
2	3	1	1	4	3	0	3	1	1	3	0	4	0
18													
OTU8503	1	0	0	0	0	0	0	0	0	0	0	0	0
0	0	0	0	0	0	0	0	0	0	0	0	0	0
0	0	0	0	0	0	0	0	0	0	0	0	0	0
0	0	0	0	0	0	0	0	0	0	0	0	0	0
0	0	0	0	0	0	0	0	0	0	0	0	0	0
0	0	0	0	0	0	0	0	0	0	0	0	0	0
0	0	0	0	0	0	0	0	0	0	0	0	0	0
0	0	0	0	0	0	0	0	0	0	0	0	0	0
0													
OTU6116	7	11	3	4	24	9	8	12	15	20	13	9	6
4	19	8	3	13	8	3	11	7	12	3	3	3	4
4	3	22	10	2	4	2	6	3	5	9	12	24	15
12	12	18	10	6	3	2	7	17	31	4	4	2	5
4	2	8	8	4	7	1	7	8	6	7	7	9	2
19	13	9	3	14	0	4	14	9	2	5	32	3	2
7	9	2	12	5	23	1	12	1	4	7	3	5	2
5	8	10	10	8	4	7	10	3	9	5	3	1	7
22													
OTU8206	2	1	0	0	0	0	0	0	0	0	0	0	0
0	0	0	0	2	0	2	0	5	0	0	0	0	0
2	39	0	2	1	0	0	0	0	20	1	0	1	0
0	0	0	0	0	0	0	1	0	3	6	7	0	0
0	0	0	1	3	16	0	5	4	0	0	0	0	0
1	1	0	2	0	0	0	0	0	0	0	5	2	2
0	1	0	0	0	1	0	0	0	1	0	0	1	0
0	0	1	1	2	1	1	0	3	6	3	0	0	1
2													

OTU7486	4	3	2	2	8	0	2	0	11	9	2	0	0
0	14	3	2	6	3	4	2	1	1	2	3	1	0
2	2	5	5	1	0	1	2	3	0	4	3	11	1
3	7	4	2	0	3	0	1	4	17	5	0	2	1
3	0	1	2	3	5	0	4	3	1	4	2	0	2
14	4	2	0	7	0	1	2	2	0	3	15	1	4
4	1	1	0	3	3	1	6	0	2	2	1	3	1
1	5	2	1	2	2	1	2	1	2	2	0	1	3
23													
OTU7796	1	0	1	0	0	0	1	0	2	1	1	0	0
0	4	0	0	0	2	0	2	1	0	0	0	0	1
0	0	0	1	0	0	0	2	0	1	0	0	1	0
2	2	2	2	0	1	1	0	1	2	0	2	0	0
0	1	0	0	0	0	1	1	0	1	2	1	0	1
1	1	0	0	3	1	0	1	1	0	0	0	1	0
0	0	0	0	2	1	0	1	0	0	0	0	1	1
1	1	0	0	0	0	0	0	0	0	1	0	0	1
5													
OTU1512	2	4	3	1	0	0	10	1	5	15	0	0	0
0	6	0	0	0	0	26	0	0	0	0	0	0	0
19	0	0	0	0	0	0	0	0	0	0	0	1	0
28	0	0	0	0	0	0	2	0	0	0	1	0	0
0	5	0	1	0	0	0	0	0	0	0	0	0	0
0	0	0	0	2	0	0	1	0	23	0	5	0	11
0	0	0	12	0	34	0	12	0	0	0	0	0	0
0	0	0	0	0	0	0	0	0	0	0	0	0	0
0													
OTU351	2	2	1	7	3	0	0	5	6	1	0	6	0
0	0	0	0	0	0	2	7	0	0	0	0	1	0
0	0	0	0	0	0	0	4	0	1	2	0	6	0
4	0	0	0	1	0	0	1	0	0	1	0	0	0
0	1	0	1	0	0	0	0	0	0	0	0	2	0
0	0	6	3	8	0	0	0	0	8	0	1	1	1

	0	0	0	18	0	0	0	11	0	9	1	0	1	16
	0	5	1	2	1	0	1	1	0	5	3	0	13	1
	1													
OTU175		7	130	18	1	1	34	0	11	0	1	0	17	0
	0	4	0	0	5	1	2	0	0	0	0	17	0	1
	1	0	0	0	0	0	0	0	0	87	0	0	0	0
	0	0	0	0	0	0	0	0	0	0	0	0	0	0
	0	0	0	0	0	2	0	0	0	0	0	0	0	0
	0	0	9	0	47	0	0	0	0	11	0	5	33	0
	0	3	0	1	0	8	0	1	0	31	0	0	0	0
	13	0	1	0	0	0	0	0	0	4	0	0	0	0
	3													
OTU7228		1	0	1	0	0	0	0	1	0	0	0	0	0
	0	0	0	0	0	0	0	0	1	0	0	0	0	0
	0	0	0	0	0	0	0	0	0	0	0	0	0	0
	0	0	0	1	0	0	0	0	0	1	0	0	0	0
	0	0	0	0	0	0	0	0	0	0	0	0	0	0
	0	0	0	0	1	0	0	0	1	0	0	1	0	0
	0	0	0	0	0	1	0	0	0	0	1	0	0	1
	0	0	0	0	1	0	0	0	0	0	0	0	0	0
	1													
OTU1163		2	0	0	0	0	0	0	0	2	0	0	0	0
	0	1	0	0	0	0	0	0	0	0	0	0	0	0
	0	0	0	0	0	0	0	0	0	0	0	0	0	0
	0	0	0	0	0	0	0	0	0	0	0	0	0	0
	0	0	0	0	0	0	0	0	0	0	0	0	0	0
	0	0	0	0	0	0	0	2	0	0	0	0	0	3
	0	0	0	0	0	0	0	0	0	0	0	0	0	0
	0	0	0	0	0	0	0	0	1	0	0	0	0	0
	0													
OTU3129		4	5	1	3	5	2	2	5	5	4	7	8	3
	1	2	3	1	8	3	1	3	7	6	2	2	2	3
	1	3	11	4	4	5	1	7	0	4	2	4	13	5

6	8	4	4	0	7	1	6	8	13	3	2	2	0
2	2	7	1	2	7	0	1	2	6	8	5	2	3
9	2	8	2	12	2	3	7	2	1	2	18	1	4
4	1	5	3	2	4	1	3	1	2	6	2	2	1
3	7	4	0	8	2	1	5	0	5	0	1	2	9
10													
OTU7482	1	0	0	1	2	1	0	2	2	1	2	1	3
1	6	0	1	4	1	0	1	1	2	0	1	1	0
1	0	2	0	0	1	0	2	1	0	0	0	4	1
1	0	0	1	0	0	0	0	1	3	0	0	0	0
1	1	2	1	0	1	1	2	0	1	3	3	1	1
2	3	1	0	2	1	0	3	2	0	1	2	1	1
0	1	0	2	0	4	1	1	0	1	0	0	0	1
0	3	0	0	2	2	0	1	0	1	0	0	1	1
4													
OTU3972	8	4	0	32	3	2	1	4	1	0	21	21	59
47	3	70	310	58	58	2	86	74	7	382	8	11	25
3	68	61	117	117	95	65	39	41	90	70	36	1	102
0	2	86	13	39	1	19	0	49	30	26	0	15	16
59	0	84	0	50	13	13	10	5	38	62	43	0	85
58	0	5	54	1	54	56	4	49	2	107	1	17	2
4	2	116	0	30	2	10	3	33	9	16	0	0	26
34	0	2	2	0	1	0	0	6	2	49	47	144	0
1													
OTU7618	2	0	0	1	0	0	0	0	0	1	1	1	0
0	2	1	0	3	0	0	0	0	0	0	0	0	0
0	0	0	0	0	0	0	1	1	0	0	0	0	0
0	0	0	0	0	2	0	1	0	0	0	0	0	5
0	2	0	1	0	0	0	0	0	0	0	0	0	0
0	0	0	0	0	0	0	0	0	0	0	0	0	0
0	0	0	0	0	0	0	0	0	0	0	0	0	0
0	0	0	0	0	0	0	0	0	0	0	1	0	1
0													

OTU11007	1	0	0	0	0	0	0	0	0	0	0	0	0
0	0	0	0	0	0	0	0	0	0	0	0	0	0
0	0	0	0	0	0	0	0	0	0	0	0	0	0
0	0	0	0	0	0	0	0	0	0	0	0	0	0
0	0	0	0	0	0	0	0	0	0	0	0	0	0
0	0	0	0	0	0	0	0	0	0	0	0	0	0
0	0	0	0	0	0	0	0	0	0	0	0	0	0
0	0	0	0	0	0	0	0	0	0	0	0	0	0
0													
OTU2464	1	0	0	99	0	0	0	0	0	0	6	2	2
3	4	2	0	0	1	0	2	0	0	0	0	0	1
0	0	5	0	0	1	0	2	16	0	4	0	3	2
1	0	1	0	19	0	0	1	0	0	0	0	0	0
0	0	0	0	0	0	6	1	0	4	0	0	0	0
0	5	1	1	0	0	0	0	0	0	0	2	0	0
0	1	0	1	0	4	1	1	0	19	0	0	2	3
0	3	36	3	0	13	3	2	1	3	0	1	50	10
17													
OTU10579	2	0	8	89	3	1	6	14	13	9	0	9	0
0	2	25	7	6	0	1	24	0	1	3	6	5	1
1	46	7	6	13	0	0	13	0	9	0	1	7	0
0	1	6	46	2	0	0	0	9	6	10	0	11	0
6	1	0	21	0	0	22	7	4	17	3	11	1	0
0	0	2	82	0	0	1	0	9	6	2	2	5	5
0	5	1	5	4	2	135	6	0	16	16	0	0	1
35	0	0	1	0	0	0	0	0	0	15	1	26	0
0													
OTU805	1	0	2	0	0	0	0	0	0	1	0	0	0
0	0	0	0	0	0	0	0	0	0	0	0	0	0
7	0	0	0	0	0	0	0	0	0	0	0	0	0
0	0	0	0	0	0	0	0	0	0	0	1	0	0
0	0	0	0	0	6	0	0	0	0	0	0	0	0
0	0	2	0	0	0	0	0	0	0	0	0	0	0

11 0 0 1 7 0 0 0 0 0 0 0 1 0

0 4 0 0 0 0 0 0 0 0 0 0 0 0

0

OTU261 3 0 1 0 0 0 0 0 1 0 0 1 30

0 0 0 0 0 0 1 2 0 0 0 7 1 0

1 0 0 0 0 0 2 0 0 0 0 0 1 0

0 0 0 0 7 3 0 1 1 120 0 434 0 0

0 1 0 0 1 11 1 17 7 0 1 0 2 1

0 2 0 1 0 0 0 3 3 0 11 9 8 0

0 0 22 2 2 3 10 1 0 1 0 0 2 0

0 1 0 2 7 1 1 1 0 2 1 0 1 11

0

OTU14612 1 1 1 0 0 2 3 0 2 1 0 0 0

0 1 0 0 1 0 0 0 0 0 0 0 0 0

0 0 0 0 0 0 0 0 0 0 0 1 0 0

1 0 0 2 1 2 0 1 0 0 0 0 0 0

0 1 0 1 0 0 0 1 0 0 0 0 2 0

0 2 2 0 0 2 0 1 0 0 0 0 0 0

1 0 0 2 0 0 1 1 0 0 0 1 0 0

0 5 0 2 1 1 1 8 0 3 0 0 0 0

0

OTU12791 2 1 0 0 2 3 2 0 2 5 3 1 0

2 1 4 0 4 3 0 0 1 1 2 0 1 1

1 0 1 2 1 1 2 2 0 1 1 4 4 3

2 0 3 0 2 3 0 1 2 5 0 0 1 0

1 1 1 3 1 2 1 0 1 0 3 2 0 0

4 2 0 1 4 2 0 5 0 0 1 5 0 1

3 4 0 1 0 6 2 0 1 0 1 0 1 0

0 1 4 1 3 1 0 1 0 0 1 2 5 3

4

OTU2645 4 5 5 4 7 7 5 7 9 10 2 4 1

4 24 5 0 4 3 1 3 1 2 2 1 3 1

5 5 9 7 1 3 2 6 1 3 0 4 10 4

10	1	4	1	2	4	1	2	3	10	0	4	1	2
4	0	6	5	1	9	3	1	3	2	2	4	6	5
18	5	8	1	13	4	4	5	3	4	4	12	1	3
4	0	1	2	3	7	1	5	2	5	5	4	4	2
2	7	3	1	4	1	1	3	2	2	1	1	4	2
26													
OTU11010	1	0	0	0	0	0	0	0	0	0	0	0	0
0	0	0	0	0	0	0	0	0	0	0	0	0	0
0	0	0	0	0	0	0	0	0	0	0	0	0	0
0	0	0	0	0	0	0	0	0	0	0	0	0	0
0	0	0	0	0	0	0	0	0	0	0	0	0	0
0	0	0	0	0	0	0	0	0	0	0	0	0	0
0	0	0	0	0	0	0	0	0	0	0	0	0	0
0	0	0	0	0	0	0	0	0	0	0	0	0	0
0													
OTU15570	1	0	0	0	0	0	0	0	0	1	0	0	0
0	0	0	0	0	0	0	0	0	0	0	0	0	0
0	0	1	0	0	0	0	0	0	0	0	0	0	0
0	0	0	0	0	0	0	0	0	0	0	0	0	0
1	0	1	0	0	0	0	0	0	0	0	0	0	0
0	0	0	0	0	0	0	0	1	0	0	0	0	0
0	0	0	0	0	0	0	0	0	0	0	0	1	0
0	0	0	1	0	0	0	0	0	0	0	0	0	0
0													
OTU9383	1	0	0	0	0	0	0	0	0	0	0	0	0
0	0	0	0	2	2	0	0	0	0	0	0	0	0
0	0	4	0	0	1	0	0	0	0	0	2	0	0
0	0	0	0	0	0	0	0	0	0	0	0	0	0
0	0	0	0	0	0	0	0	0	0	0	0	0	0
0	0	0	1	0	0	1	0	1	0	0	0	0	0
0	0	1	0	0	0	0	0	0	0	0	0	0	0
0	0	0	0	0	0	0	0	0	0	0	1	0	0
0													

OTU9054 6 0 0 0 2 0 2 0 0 1 2 1 2
0 0 3 0 2 2 0 0 0 4 0 6 0 3
0 0 9 4 0 7 0 0 0 2 0 13 0 7
0 0 0 0 0 0 0 0 0 0 0 0 0 1
0 0 0 0 0 0 0 0 0 0 0 0 0 0
0 0 0 0 0 0 0 0 0 0 0 0 0 0
0 0 0 0 0 2 0 0 0 0 0 0 0 0
0 0 0 0 0 0 0 0 0 0 0 0 0 0
0
OTU12226 1 0 0 0 0 0 0 0 0 0 0 0 0
0 0 0 0 0 0 0 0 0 0 0 0 0 0
0 0 0 0 0 0 0 0 0 0 0 0 0 0
0 0 0 0 0 0 0 0 0 0 0 0 0 0
0 0 0 0 0 0 0 0 0 0 0 0 0 0
0 0 0 0 0 0 0 0 0 0 0 0 0 0
0 0 0 0 0 0 0 0 0 0 0 0 0 0
0 0 0 0 0 0 0 0 0 0 0 0 0 0
0
OTU8431 3 0 0 1 8 2 3 3 4 2 4 2 1
2 1 3 0 4 1 0 0 2 3 1 1 0 1
2 1 6 1 0 1 0 1 2 1 1 0 6 2
2 0 3 1 2 0 0 1 2 1 0 1 0 0
0 3 7 3 1 5 3 1 5 1 4 1 1 1
2 3 0 1 4 3 0 4 1 1 0 12 0 4
1 2 1 0 3 2 0 0 2 1 3 0 1 0
1 1 0 1 0 3 0 2 0 2 4 0 1 2
5
OTU10247 1 7 5 0 0 1 1 0 6 0 0 9 0
1 0 0 7 0 0 1 0 51 0 60 14 36 6
12 51 0 0 23 0 10 0 0 0 31 1 3 0
0 0 74 0 25 2 0 0 33 22 22 1 3 0
2 0 10 1 10 25 19 22 28 9 65 12 3 12
29 0 3 1 2 15 0 5 4 4 0 15 0 0

7	195	0	0	0	0	22	15	0	5	0	0	1	0
0	0	5	3	0	4	0	0	1	0	10	0	1	3
0													
OTU555	1	0	1	0	0	0	1	1	0	0	0	0	0
0	0	0	0	0	0	1	0	0	0	0	0	0	0
0	0	0	0	0	0	0	0	0	0	0	0	0	0
0	0	0	0	0	0	0	0	0	0	0	0	0	0
0	0	0	3	0	0	0	0	0	0	0	0	0	0
0	0	0	0	0	0	0	1	0	0	0	0	0	0
0	0	0	0	0	0	0	0	0	0	0	0	0	0
0	0	0	0	0	0	1	0	0	0	21	0	0	0
0													
OTU915	27	2	1	0	9	3	15	116	0	3	4	23	10
5	2	15	10	3	34	6	29	168	21	15	52	5	4
26	0	6	9	5	9	0	1	1	47	0	28	2	10
0	191	24	114	10	34	2	18	23	273	9	58	6	20
3	72	49	45	11	29	243	49	33	26	36	6	19	23
3	62	15	16	4	22	145	40	7	11	28	3	8	16
257	7	0	30	3	6	89	2	0	0	0	3	0	4
4	34	33	34	12	31	91	53	20	2	1	4	44	11
OTU7646	1	0	0	0	0	0	1	0	0	5	0	1	0
0	2	0	0	0	0	0	0	0	0	0	0	0	0
1	0	0	0	0	0	0	0	0	0	0	0	0	0
0	0	0	0	0	0	0	0	0	0	0	0	0	0
0	0	0	0	0	0	0	0	0	0	0	0	0	0
0	0	0	0	0	0	0	0	0	0	0	2	0	0
0	0	0	0	0	0	0	0	0	0	0	0	0	0
0	0	2	0	0	0	0	1	0	0	0	0	0	0
0													
OTU9921	2	0	0	1	1	3	0	0	0	0	0	0	0
0	0	0	0	0	0	0	0	0	0	0	0	2	1
0	0	0	0	0	0	0	0	1	0	0	0	0	0
0	0	0	1	0	1	0	2	0	0	0	0	0	1

0	0	0	0	0	0	0	0	0	0	0	0	1	0
0	0	0	0	0	0	0	0	0	1	0	0	0	1
0	0	0	3	0	3	2	0	0	0	0	0	0	0
0	0	0	0	0	0	0	0	0	0	0	0	0	0
0													
OTU4759	1	0	2	2	4	0	1	1	2	3	1	0	0
1	3	0	0	1	1	1	1	0	1	0	0	0	1
2	0	2	0	0	1	0	1	1	0	0	1	0	1
1	1	2	0	1	0	0	1	4	3	0	0	0	1
1	2	2	0	0	1	1	0	2	0	2	1	0	1
7	0	0	0	2	1	2	2	0	1	1	2	3	1
1	0	1	0	0	1	1	1	0	1	0	0	0	0
2	0	0	1	2	0	2	1	0	1	1	0	3	0
0													
OTU2373	4	3	4	0	11	3	3	1	5	8	1	1	1
2	10	2	1	4	0	0	2	2	5	3	3	3	3
1	1	6	2	4	1	1	1	1	2	1	2	5	4
0	0	0	1	1	1	0	2	2	8	0	0	1	4
2	2	2	4	2	3	1	0	2	0	0	8	1	0
14	5	6	2	9	1	1	3	0	5	1	10	0	0
1	0	2	2	2	3	2	4	1	0	3	2	1	0
2	2	3	1	2	2	2	5	0	1	3	1	0	1
11													
OTU11011	1	0	0	0	0	0	0	0	0	0	0	0	0
0	0	0	0	0	0	0	0	0	0	0	0	0	0
0	0	0	0	0	0	0	0	0	0	0	0	0	0
0	0	0	0	0	0	0	0	0	0	0	0	0	0
0	0	0	0	0	0	0	0	0	0	0	0	0	0
0	0	0	0	0	0	0	0	0	0	0	0	0	0
0	0	0	0	0	0	0	0	0	0	0	0	0	0
0	0	0	0	0	0	0	0	0	0	0	0	0	0
0													

OTU14293 1 0 0 0 0 0 0 0 0 0 0 0 0
0 0 0 0 0 0 0 0 0 0 0 0 0 0
0 0 0 0 0 0 0 0 0 0 0 0 0 0
0 0 0 0 0 0 0 0 0 0 0 0 0 0
0 0 0 0 0 0 0 0 0 0 0 0 0 0
0 0 0 0 0 0 0 0 0 0 0 0 0 0
0 0 0 0 0 0 0 0 0 0 0 0 0 0
0 0 0 0 0 0 0 0 0 0 0 0 0 0
0
OTU10134 1 0 0 1 1 0 0 2 0 0 0 1 0
0 0 0 0 0 0 0 3 0 0 0 0 2 0
0 0 0 0 0 0 0 2 0 0 0 0 0 1
0 2 0 0 2 1 0 1 0 0 0 0 0 0
0 0 0 0 0 0 0 0 0 0 0 1 0 0
0 0 0 1 0 0 1 0 1 0 0 0 1 0
0 0 2 0 5 0 0 0 0 1 0 0 0 0
1 0 0 0 0 0 0 0 0 1 1 0 0 0
0
OTU154 3 0 0 20 2 10 0 6 3 0 9 3 15
7 1 35 1 3 9 1 12 9 4 6 38 8 63
12 3 1 9 12 0 1 3 1 7 1 1 18 10
3 1 0 1 1 1 1 0 0 0 6 1 2 1
1 13 0 2 0 0 0 1 1 1 0 0 0 5
0 0 0 5 0 0 3 4 0 12 18 0 4 28
0 2 2 1 16 1 13 4 0 4 4 2 1 0
1 2 1 0 8 0 0 0 2 4 12 19 6 4
1
OTU2140 1 0 0 0 2 0 0 1 0 0 1 1 2
2 0 0 0 0 2 0 5 0 0 1 0 0 0
0 0 0 1 0 3 0 0 0 7 0 2 0 0
0 0 0 0 0 0 0 0 0 0 0 0 0 1
0 0 0 0 0 0 0 0 0 0 0 1 0 0
1 0 0 0 0 0 0 0 0 0 0 0 0 0

0	0	1	0	0	0	0	0	5	0	3	0	0	1
0	0	0	0	0	0	0	0	0	0	0	0	0	0
0													
OTU103	4	6	2	3	11	1	17	7	9	2	44	22	16
38	4	38	29	8	13	4	37	0	4	11	15	5	25
0	4	17	21	4	2	11	19	3	29	2	1	2	0
0	31	3	29	9	3	3	0	6	0	4	0	5	8
7	0	6	4	16	0	6	19	10	5	0	8	0	28
12	0	6	19	2	14	5	1	24	0	6	0	10	0
7	13	15	0	5	0	17	2	7	5	17	1	0	10
28	2	1	0	0	0	0	0	1	0	27	18	60	0
0													
OTU155	19	0	0	0	1	0	1	1	0	0	9	0	0
0	0	1	0	35	9	3	40	0	8	0	8	0	161
3	0	0	1	0	348	0	0	0	0	0	93	0	0
0	0	0	0	1	0	68	0	1	0	0	0	0	33
0	0	0	0	0	0	0	0	0	0	0	0	0	0
0	0	0	0	0	0	0	0	0	0	0	0	0	0
0	0	0	3	2	1	0	0	0	0	1	0	0	0
0	0	0	0	0	0	0	0	0	1	3	0	0	0
0													
OTU95 1	2	49	0	1	0	27	4	33	25	1	19	1	0
1	8	6	15	0	53	84	10	83	11	6	45	0	71
0	0	4	12	8	3	0	1	123	5	0	4	0	13
181	1	62	4	14	2	6	34	10	60	611	28	0	1
2	1	189	17	22	165	69	262	16	3	12	22	28	3
3	6	82	82	133	334	344	132	34	47	141	213	127	1
30	1	36	163	252	87	1	0	11	0	4	0	0	0
23	1	2	1	0	3	6	1	0	54	0	0	0	0
OTU12761	2	0	2	10	1	7	4	5	0	5	2	11	0
1	7	12	0	4	1	5	0	0	0	0	0	0	0
0	0	0	2	0	0	0	8	0	7	0	0	0	2
3	11	0	8	0	2	2	3	1	0	0	0	1	2

0	3	0	1	0	0	0	0	0	0	0	1	0	1
0	0	3	3	0	26	0	1	2	0	1	0	9	1
0	0	0	1	2	0	0	0	0	5	1	0	1	4
2	0	0	0	0	0	0	0	0	0	13	2	3	0
0													
OTU9638	7	2	5	5	4	4	5	8	9	10	3	2	3
2	12	3	2	8	3	0	1	7	3	2	3	2	1
2	0	8	1	6	1	2	5	1	1	6	4	9	5
5	6	3	3	6	2	0	1	6	12	0	2	3	3
5	0	6	2	1	4	2	6	8	6	2	8	5	5
13	9	5	2	12	4	2	7	2	2	1	16	0	6
4	1	2	0	1	5	0	6	6	3	13	4	0	2
3	3	3	3	4	5	0	6	1	3	6	1	1	4
9													
OTU15334	1	1	2	1	2	0	4	2	1	4	3	0	0
0	1	1	0	2	2	0	1	0	1	0	0	0	0
0	0	1	0	0	0	0	0	0	0	0	0	3	2
1	2	2	0	1	1	0	0	2	0	0	0	0	0
0	0	0	0	1	2	1	0	4	0	2	0	0	0
0	0	1	0	4	0	0	2	0	0	0	2	0	0
1	0	0	0	0	0	1	3	0	2	0	0	2	0
0	0	1	0	0	3	0	1	0	0	1	0	0	2
0													
OTU12223	1	0	0	0	0	0	0	0	0	0	0	0	0
0	0	0	0	0	0	0	0	0	0	0	0	0	0
0	0	0	0	0	0	0	0	0	0	0	0	0	0
0	0	0	0	0	0	0	0	0	0	0	0	0	0
0	0	0	0	0	0	0	0	0	0	0	0	0	0
0	0	0	0	0	0	0	0	0	0	0	0	0	0
0	0	0	0	0	0	0	0	0	0	0	0	0	0
0	0	0	0	0	0	0	0	0	0	0	0	0	0
0													

OTU1936	4	0	0	1	2	5	1	5	0	0	0	0	0
1	4	0	0	0	0	1	0	0	0	0	0	17	33
1	0	0	0	0	0	0	0	4	0	0	0	0	0
1	0	0	1	0	11	0	1	0	1	0	0	0	3
0	1	0	1	0	0	2	2	0	0	0	1	2	0
0	0	0	0	1	0	0	0	0	0	4	0	1	2
1	0	0	0	0	0	7	0	0	0	0	0	0	0
0	0	0	0	0	0	0	0	0	0	0	0	0	0
0													
OTU149	10	81	22	111	3	36	106	33	16	158	0	6	0
0	57	0	6	0	4	81	3	8	0	6	2	0	0
17	5	1	4	7	0	12	1	0	0	3	0	55	2
20	13	1	238	4	24	0	49	2	14	16	9	1	1
2	20	5	12	2	1	1	3	4	0	2	1	45	3
5	13	10	12	7	267	0	4	0	55	7	9	8	21
1	3	1	89	1	4	0	13	0	1	1	8	10	0
0	9	24	17	2	1	0	6	5	14	1	1	10	13
9													
OTU137	3	1	0	0	0	0	0	1	145	1	1	0	0
0	0	0	11	0	0	14	0	25	0	0	0	1	15
6	5	0	1	34	0	43	0	0	0	136	0	201	0
3	0	6	0	10	7	0	3	1	6	0	0	1	0
3	0	3	5	2	6	0	6	2	3	7	2	2	8
7	21	2	0	0	0	0	6	0	0	14	12	0	0
0	3	0	29	0	1	0	19	0	0	0	0	9	0
0	1	222	36	35	63	49	15	60	22	0	0	1	42
20													
OTU12225	1	0	0	0	0	0	0	0	0	0	0	0	0
0	0	0	0	0	0	0	0	0	0	0	0	0	0
0	0	0	0	0	0	0	0	0	0	0	0	0	0
0	0	0	0	0	0	0	0	0	0	0	0	0	0
0	0	0	0	0	0	0	0	0	0	0	0	0	0
0	0	0	0	0	0	0	0	0	0	0	0	0	0

0	0	0	0	0	0	0	0	0	0	0	0	0	0
0	0	0	0	0	0	0	0	0	0	0	0	0	0
0													
OTU425	2	2	0	0	1	1	1	0	1	1	1	0	0
0	0	0	0	0	2	1	2	0	2	1	2	3	4
0	0	0	0	1	0	0	1	1	3	2	2	1	0
6	1	0	1	0	3	0	2	0	0	1	1	0	1
0	4	1	2	1	0	0	0	0	1	0	2	4	0
1	0	2	0	2	0	0	1	0	2	0	1	4	3
1	0	0	3	0	0	3	1	0	1	1	0	18	0
0	0	0	2	0	0	0	1	0	0	1	1	2	0
0													
OTU8502	1	0	0	0	0	0	0	0	0	0	0	0	0
0	0	0	0	0	0	0	0	0	0	0	0	0	0
0	0	0	0	0	0	0	0	0	0	0	0	0	0
0	0	0	0	0	0	0	0	0	0	0	0	0	0
0	0	0	0	0	0	0	0	0	0	0	0	0	0
0	0	0	0	0	0	0	0	0	0	0	0	0	0
0	0	0	0	0	0	0	0	0	0	0	0	0	0
0	0	0	0	0	0	0	0	0	0	0	0	0	0
0													
OTU184	3	7	20	1	9	20	17	12	6	60	0	3	1
0	4	0	0	0	0	32	46	0	0	0	0	3	0
4	0	0	0	0	0	0	27	11	3	2	0	4	0
0	0	0	12	0	0	0	0	0	3	1	1	0	0
0	0	0	39	0	0	0	0	1	0	0	2	6	1
0	4	0	0	0	0	0	3	0	0	0	8	1	1
0	1	0	0	0	0	0	2	0	10	4	0	8	12
12	0	0	1	1	0	0	0	2	2	7	5	6	2
0													
OTU503	1	11	3	0	1	0	1	1	3	0	0	0	1
0	1	0	0	1	0	0	5	0	0	0	0	0	0
1	0	0	0	0	0	0	0	0	0	0	0	1	0

0 0 0 0 0 1 0 2 0 0 1 3 0 0

0 3 0 0 0 0 1 1 0 0 0 0 0 0

2 5 1 0 2 0 0 4 0 35 0 2 0 1

0 9 0 2 0 0 0 0 0 0 0 7 5 0

0 7 8 8 4 9 1 5 4 0 0 0 2 47

1

OTU51 16 6 3 66 27 9 7 9 0 3 66 100 56 128

65 53 79 165 2 1 31 22 3 114 43 4 61 0

15 47 20 39 42 10 93 12 0 70 289 1 1 1

0 6 1 0 0 112 1 4 0 8 1 37 6 9

0 76 2 1 0 0 8 0 76 0 74 0 46 2

0 7 53 0 2 35 7 30 6 16 1 3 3 557

41 155 2 56 6 15 10 3 23 57 0 0 47 61

0 0 4 0 0 0 0 6 0 26 176 168 0 0

OTU13278 1 0 1 0 0 2 0 2 0 0 1 0 0

1 0 0 0 0 0 0 1 0 0 0 0 0 0

0 0 0 0 0 1 0 0 0 0 0 1 0 0

1 0 0 0 0 0 0 0 0 0 0 0 0 0

0 0 0 0 0 0 0 0 0 0 1 0 0 0

1 1 0 0 0 0 0 1 0 0 0 1 0 0

1 1 0 0 0 1 0 0 0 0 1 0 1 0

1 1 1 0 1 0 0 0 0 0 0 0 0 0

0

OTU14292 1 0 0 0 0 0 0 0 0 0 0 0 0

0 0 0 0 0 0 0 0 0 0 0 0 0 0

0 0 0 0 0 0 0 0 0 0 0 0 0 0

0 0 0 0 0 0 0 0 0 0 0 0 0 0

0 0 0 0 0 0 0 0 0 0 0 0 0 0

0 0 0 0 0 0 0 0 0 0 0 0 0 0

0 0 0 0 0 0 0 0 0 0 0 0 0 0

0 0 0 0 0 0 0 0 0 0 0 0 0 0

0

OTU11008	1	0	0	0	0	0	0	0	0	0	0	0	0
0	0	0	0	0	0	0	0	0	0	0	0	0	0
0	0	0	0	0	0	0	0	0	0	0	0	0	0
0	0	0	0	0	0	0	0	0	0	0	0	0	0
0	0	0	0	0	0	0	0	0	0	0	0	0	0
0	0	0	0	0	0	0	0	0	0	0	0	0	0
0	0	0	0	0	0	0	0	0	0	0	0	0	0
0	0	0	0	0	0	0	0	0	0	0	0	0	0
0													
OTU315	3	4	3	2	0	1	4	0	3	2	0	1	1
1	3	0	0	1	1	3	1	0	1	0	4	0	0
0	0	0	1	0	3	2	1	2	2	0	4	6	1
4	1	0	1	0	5	4	4	0	0	0	1	0	1
0	4	1	2	0	0	0	0	0	1	0	0	5	2
0	4	4	0	1	0	5	1	0	5	6	0	2	2
0	0	2	0	3	1	3	4	0	0	1	4	2	0
0	2	2	5	6	5	1	6	4	4	5	0	0	6
0													
OTU3844	1	1	0	0	0	0	0	0	0	0	0	0	0
0	0	0	0	0	0	0	0	0	0	0	0	0	0
0	0	0	0	1	0	0	0	0	0	0	0	0	0
0	0	0	0	1	0	0	0	0	0	0	0	0	0
0	0	0	0	0	0	0	0	0	0	0	0	0	0
0	0	0	0	0	0	0	0	0	0	0	0	0	0
0	0	0	0	0	0	0	0	0	0	0	0	0	0
0	0	0	0	0	0	0	0	0	0	0	0	1	0
0													
OTU12224	1	0	0	0	0	0	0	0	0	0	0	0	0
0	0	0	0	0	0	0	0	0	0	0	0	0	0
0	0	0	0	0	0	0	0	0	0	0	0	0	0
0	0	0	0	0	0	0	0	0	0	0	0	0	0
0	0	0	0	0	0	0	0	0	0	0	0	0	0
0	0	0	0	0	0	0	0	0	0	0	0	0	0

0	0	0	0	0	0	0	0	0	0	0	0	0	0
0	0	0	0	0	0	0	0	0	0	0	0	0	0
0													
OTU10197	4	0	1	0	0	0	0	0	1	0	0	3	0
0	0	0	28	0	0	2	0	24	0	21	0	38	0
5	48	0	0	8	1	8	1	0	0	20	1	2	0
0	0	9	0	10	0	0	0	25	27	12	2	31	0
11	0	9	0	30	30	52	22	24	17	20	2	0	11
7	0	0	0	0	11	0	33	10	0	0	7	0	0
2	40	0	0	0	0	21	3	1	0	0	0	0	0
0	0	0	0	0	0	0	0	0	0	5	0	0	1
0													
OTU7202	1	0	1	0	1	0	0	1	0	0	0	3	0
1	0	0	0	0	1	1	0	0	0	0	0	0	0
1	0	0	0	0	0	0	1	0	0	0	0	0	0
0	1	0	0	0	0	0	0	0	0	0	0	0	0
0	0	0	1	0	0	0	0	0	0	0	0	0	0
0	0	0	1	1	0	0	0	1	0	0	2	1	3
0	0	0	0	0	0	1	0	0	0	0	4	0	0
0	1	0	0	0	0	0	1	0	0	0	0	0	0
0													
OTU11009	1	0	0	0	0	0	0	0	0	0	0	0	0
0	0	0	0	0	0	0	0	0	0	0	0	0	0
0	0	0	0	0	0	0	0	0	0	0	0	0	0
0	0	0	0	0	0	0	0	0	0	0	0	0	0
0	0	0	0	0	0	0	0	0	0	0	0	0	0
0	0	0	0	0	0	0	0	0	0	0	0	0	0
0	0	0	0	0	0	0	0	0	0	0	0	0	0
0	0	0	0	0	0	0	0	0	0	0	0	0	0
0													
OTU12215	1	0	0	0	0	0	0	0	0	0	0	0	0
0	0	0	0	0	0	0	0	0	0	0	0	0	0
0	0	0	0	0	0	0	0	0	0	0	0	0	0

0	0	0	0	0	0	0	0	0	0	0	0	0	0
0	0	0	0	0	0	0	0	0	0	0	0	0	0
0	0	0	0	0	0	0	0	0	0	0	0	0	0
0	0	0	0	0	0	0	0	0	0	0	0	0	0
0	0	0	0	0	0	0	0	0	0	0	0	0	0
0													
OTU16231	2	3	1	1	0	0	1	1	3	3	3	0	2
0	11	1	0	0	0	1	1	1	2	1	0	0	0
1	1	2	1	1	1	1	1	1	0	1	0	6	1
6	0	0	2	1	1	0	2	1	5	1	3	1	0
3	0	1	1	2	0	0	1	3	2	0	1	1	0
2	5	1	2	2	1	2	0	1	3	0	0	0	1
1	1	0	2	3	1	3	4	0	1	2	1	0	0
1	1	0	0	1	2	1	3	2	2	1	1	1	4
3													
OTU6660	5	3	7	0	8	7	3	5	6	6	5	6	3
4	37	8	4	8	5	5	1	5	3	2	4	4	3
4	0	8	1	3	2	1	9	1	2	6	5	9	1
9	5	1	7	1	1	1	1	1	15	2	5	3	2
6	2	1	3	3	5	3	5	5	3	5	7	8	0
13	11	3	3	17	6	3	8	2	2	0	13	2	2
9	4	4	0	4	12	2	4	1	5	6	1	4	2
4	7	4	5	7	2	8	2	1	1	6	5	5	3
25													
OTU12216	3	0	0	0	0	0	0	0	0	0	0	0	0
0	0	0	0	0	0	0	0	0	0	0	0	0	0
0	0	0	0	0	0	0	0	0	0	0	0	0	0
0	0	0	0	0	0	0	0	0	0	0	0	0	0
0	0	0	0	0	0	0	0	0	0	0	0	0	0
0	0	0	0	0	0	0	0	0	0	0	0	0	0
0	0	0	0	0	0	0	0	0	0	0	0	0	0
0	0	0	0	0	0	0	0	0	0	0	0	0	0
0													

OTU3547	4	3	0	0	2	4	1	2	3	2	0	2	2
3	1	2	0	8	4	1	1	1	0	1	2	0	1
3	1	6	3	1	3	0	4	2	0	0	1	4	2
1	2	2	3	2	2	1	0	5	2	3	0	0	0
0	1	1	1	0	0	0	0	2	0	1	2	0	2
1	1	2	3	4	1	0	5	3	0	1	7	0	4
2	1	0	2	0	2	1	1	0	0	1	0	0	1
0	0	2	1	1	1	0	5	0	1	0	2	1	2
7													
OTU11018	1	0	0	0	0	0	0	0	0	0	0	1	0
0	0	0	0	0	0	0	0	0	0	0	0	0	0
0	0	0	0	0	0	0	0	0	0	0	0	0	0
0	0	0	0	0	0	0	0	0	0	0	0	0	0
0	0	0	0	0	0	0	0	0	0	0	0	0	0
0	0	0	0	0	0	0	0	0	0	0	0	0	0
0	0	0	0	0	0	0	0	0	0	0	0	0	0
0	0	0	0	0	0	0	0	0	0	0	0	0	0
0													
OTU14296	1	0	0	0	0	0	0	0	0	0	0	0	0
0	0	0	0	0	0	0	0	0	0	0	0	0	0
0	0	0	0	0	0	0	0	0	0	0	0	0	0
0	0	0	0	0	0	0	0	0	0	0	0	0	0
0	0	0	0	0	0	0	0	0	0	0	0	0	0
0	0	0	0	0	0	0	0	0	0	0	0	0	0
0	0	0	0	0	0	0	0	0	0	0	0	0	0
0	0	0	0	0	0	0	0	0	0	0	0	0	0
0													
OTU316	5	0	1	0	5	0	0	0	0	7	0	0	0
0	0	1	0	0	0	0	0	0	0	0	0	0	0
0	0	0	1	0	0	0	0	0	6	0	0	0	0
1	13	0	1	0	1	2	0	0	0	0	0	0	0
0	0	0	1	0	0	0	0	0	0	0	0	5	0
0	0	0	0	76	2	0	0	0	1	11	0	0	0

2	0	0	2	0	0	0	0	0	1	0	3	1	0
0	6	0	0	0	0	0	3	0	0	0	0	0	0
0													
OTU3872	1	0	0	0	0	0	0	0	0	0	0	0	0
0	0	0	1	0	0	0	0	0	0	0	0	1	0
0	0	1	0	1	0	0	0	0	0	2	0	1	0
0	0	0	0	0	0	0	0	0	0	0	0	0	0
0	0	0	0	0	0	0	0	0	0	0	0	0	0
0	0	0	0	0	0	0	0	0	0	0	0	0	0
0	0	0	0	0	0	0	0	0	0	0	0	0	0
0	0	0	0	0	0	1	0	0	0	0	0	0	0
0													
OTU14444	2	2	1	2	6	3	1	3	3	4	4	5	3
1	13	2	0	6	4	1	2	3	3	2	0	3	3
0	2	5	2	2	2	1	6	2	1	2	8	13	1
3	9	6	1	1	2	1	2	5	7	2	2	1	3
3	0	8	3	1	6	0	2	4	5	1	1	2	0
3	1	3	1	4	4	1	8	3	3	1	7	0	6
6	4	0	2	2	2	1	2	0	5	5	3	4	1
2	7	0	1	4	2	2	1	1	1	3	1	2	3
9													
OTU551	4	0	0	0	0	2	0	0	3	1	0	0	0
0	1	0	0	0	0	0	0	0	0	0	0	0	0
0	0	0	0	1	0	0	0	0	0	0	0	0	0
0	0	0	0	1	0	0	2	1	5	1	0	0	0
0	2	0	0	2	2	3	4	0	1	1	0	0	0
1	1	0	0	0	0	0	4	0	7	0	0	0	0
0	1	0	1	0	0	1	0	0	0	0	0	1	0
0	0	0	0	0	1	1	2	0	0	0	0	0	0
0													
OTU8508	1	0	0	0	0	0	0	0	0	0	0	0	0
0	0	0	0	0	0	0	0	0	0	0	0	0	0
0	0	0	0	0	0	0	0	0	0	0	0	0	0

0 0 0 0 0 0 0 0 0 0 0 0 0 0
0 0 0 0 0 0 0 0 0 0 0 0 0 0
0 0 0 0 0 0 0 0 0 0 0 0 0 0
0 0 0 0 0 0 0 0 0 0 0 0 0 0
0 0 0 0 0 0 0 0 0 0 0 0 0 0
0

OTU453 1 0 0 0 0 2 0 0 0 0 1 0 1
0 0 20 0 0 1 0 6 0 0 0 2 1 0
1 0 2 1 0 1 0 2 0 6 0 0 0 0
0 0 0 0 0 0 0 0 0 0 0 0 0 0
0 0 0 0 0 0 0 0 0 0 0 0 0 0
0 0 0 1 0 0 0 0 0 0 0 0 0 0
0 0 0 1 0 0 0 0 0 1 0 0 0 2
5 0 0 0 0 0 0 0 0 0 2 0 0 0
0

OTU14294 1 0 0 0 0 0 0 0 0 0 0 0 0
0 0 0 0 0 0 0 0 0 0 0 0 0 0
0 0 0 0 0 0 0 0 0 0 0 0 0 0
0 0 0 0 0 0 0 0 0 0 0 0 0 0
0 0 0 0 0 0 0 0 0 0 0 0 0 0
0 0 0 0 0 0 0 0 0 0 0 0 0 0
0 0 0 0 0 0 0 0 0 0 0 0 0 0
0 0 0 0 0 0 0 0 0 0 0 0 0 0
0

OTU135 1 5 3 0 0 0 1 1 7 1 1 2 3
0 0 0 88 0 0 4 0 129 0 90 2 6 25
30 50 0 0 26 2 38 1 0 1 41 0 7 0
4 0 51 0 97 2 0 2 178 41 6 34 3 0
23 3 36 8 75 121 27 157 148 47 268 2 19 8
160 2 2 0 6 1 1 64 0 7 3 16 0 18
2 30 0 2 1 4 0 8 2 0 1 0 0 1
0 0 2 2 2 18 3 3 2 8 1 0 1 16
0

OTU14295 1 0 0 0 0 0 0 0 0 0 0 0 0
0 0 0 0 0 0 0 0 0 0 0 0 0 0
0 0 0 0 0 0 0 0 0 0 0 0 0 0
0 0 0 0 0 0 0 0 0 0 0 0 0 0
0 0 0 0 0 0 0 0 0 0 0 0 0 0
0 0 0 0 0 0 0 0 0 0 0 0 0 0
0 0 0 0 0 0 0 0 0 0 0 0 0 0
0 0 0 0 0 0 0 0 0 0 0 0 0 0
0
OTU8475 2 1 0 0 0 3 3 1 1 0 0 0 0
0 0 0 0 1 0 0 0 0 0 0 0 0 6
1 0 0 0 0 0 0 0 0 0 0 0 1 0
0 0 0 0 0 0 0 1 0 0 2 1 0 0
0 0 0 0 0 0 1 0 0 0 0 2 2 0
0 0 0 1 9 1 0 0 0 1 0 0 0 0
0 0 0 0 0 0 5 0 0 0 0 0 0 0
2 0 0 0 0 0 0 0 0 0 0 0 0 0
0
OTU11017 1 0 0 0 0 0 0 0 0 0 0 0 0
0 0 0 0 0 0 0 0 0 0 0 0 0 0
0 0 0 0 0 0 0 0 0 0 0 0 0 0
0 0 0 0 0 0 0 0 0 0 0 0 0 0
0 0 0 0 0 0 0 0 0 0 0 0 0 0
0 0 0 0 0 0 0 0 0 0 0 0 0 0
0 0 0 0 0 0 0 0 0 0 0 0 0 0
0 0 0 0 0 0 0 0 0 0 0 0 0 0
0
OTU8507 1 0 0 0 0 0 0 0 0 0 0 0 0
0 0 0 0 0 0 0 0 0 0 0 0 0 0
0 0 0 0 0 0 0 0 0 0 0 0 0 0
0 0 0 0 0 0 0 0 0 0 0 0 0 0
0 0 0 0 0 0 0 0 0 0 0 0 0 0
0 0 0 0 0 0 0 0 0 0 0 0 0 0

0	0	0	0	0	0	0	0	0	0	0	0	0	0
0	0	0	0	0	0	0	0	0	0	0	0	0	0
0													
OTU2220	2	0	4	0	5	8	2	4	0	3	0	0	0
0	3	3	0	33	1	2	0	0	0	1	0	1	7
14	0	0	23	1	0	0	0	3	2	3	0	0	0
12	0	0	0	1	8	1	1	0	0	0	3	0	1
0	1	1	0	0	0	0	1	0	1	0	0	5	0
0	7	13	1	0	35	14	0	12	1	5	3	37	2
0	7	3	7	9	0	0	0	0	6	2	3	6	2
6	14	2	4	3	2	2	2	0	5	1	0	0	5
6													
OTU7322	1	0	0	0	0	0	0	0	0	0	0	6	2
0	2	0	0	0	0	0	0	0	1	0	0	1	5
0	0	0	0	0	0	0	0	1	0	0	1	0	0
0	2	0	3	0	0	0	0	1	0	0	0	0	0
0	0	0	0	0	0	0	0	0	0	0	0	0	0
0	0	0	4	0	8	1	0	1	0	0	0	2	0
3	0	1	0	0	0	0	0	0	0	1	0	0	0
1	0	0	0	0	0	0	0	1	0	0	0	6	0
0													
OTU345	2	0	0	0	0	0	0	0	1	0	0	0	0
0	0	0	0	0	0	0	0	0	0	0	0	0	0
0	0	0	0	0	0	0	0	0	0	0	0	0	0
0	0	0	0	0	0	0	1	0	0	0	0	0	0
0	0	0	0	0	0	0	0	0	0	0	0	0	0
0	0	1	0	0	0	0	0	0	0	1	0	0	0
0	0	0	0	0	0	0	51	0	0	0	0	0	0
0	0	0	0	0	14	1	0	6	24	0	0	0	0
15													
OTU124	4	2	19	1	14	14	26	3	69	8	0	0	0
0	3	0	0	1	0	8	0	0	0	1	0	13	0
1	0	0	0	1	0	8	0	3	0	2	0	22	0

35	2	1	0	0	14	0	4	0	0	6	2	0	0
0	29	0	2	0	0	1	0	0	0	0	0	29	0
1	23	35	0	2	0	1	1	0	21	0	15	1	45
9	16	0	49	0	11	3	69	0	0	0	11	19	0
0	7	30	44	6	28	10	34	21	125	0	0	0	24
48													
OTU8054	1	0	0	0	0	0	0	0	0	0	0	0	0
0	0	0	0	0	0	0	0	0	0	0	0	0	0
0	0	0	0	0	0	0	0	0	0	0	0	0	0
0	0	0	0	0	0	0	0	0	0	0	0	0	0
0	0	0	0	0	0	0	0	0	0	0	0	0	0
0	0	1	0	0	0	0	0	0	0	0	0	0	0
0	0	0	0	0	0	0	0	0	0	0	0	0	0
0	0	0	0	0	1	0	0	0	0	0	0	0	0
0													
OTU7405	2	2	3	6	6	4	1	2	3	8	4	4	1
3	14	3	2	4	2	1	3	0	4	1	2	5	1
3	2	4	4	3	0	1	2	0	4	1	1	10	1
6	4	1	4	0	0	2	0	2	7	1	3	1	2
4	1	5	5	0	4	3	2	5	3	2	1	1	3
6	5	1	2	6	2	3	4	1	1	1	8	0	2
2	0	3	1	1	15	1	2	1	3	4	4	3	1
1	9	3	3	6	3	4	2	3	2	2	1	0	2
16													
OTU15800	1	0	0	2	0	0	0	1	1	2	0	1	0
1	5	0	0	4	1	0	0	3	0	1	1	0	1
2	1	0	0	0	0	0	0	0	1	0	2	0	1
0	2	2	2	2	0	0	0	1	0	0	0	2	1
0	1	4	0	0	1	0	0	0	1	0	0	1	0
2	1	2	1	4	2	1	4	2	2	1	3	0	0
1	1	0	0	0	2	0	0	0	0	0	1	1	1
0	0	0	2	0	0	1	2	1	1	0	0	0	0
4													

OTU265 0 5 248 14 121 1 79 13 0 62 14 364 94
2 834 4 11 361 1 412 14 17 407 1 4 0 1
1 1090 306 22 69 0 512 10 98 0 15 0 492 0
679 8 6 0 0 127 0 3 1 1 0 10 16 4
2 9 2 2 0 1 2 0 11 0 21 0 12 0
0 0 469 8 0 21 0 187 0 9 0 3 0 16
0 1242 1 21 1 1 0 221 779 809 0 0 225 1259
0 18 2 0 0 0 0 52 0 98 507 851 0 0
OTU8765 1 0 0 2 1 0 1 0 0 1 0 0 0
0 0 0 0 0 0 0 0 0 0 0 0 0 0
0 0 0 0 0 1 0 0 3 0 0 1 0 0
0 0 0 0 0 0 1 0 0 0 0 0 0 1
0 1 0 0 0 0 0 0 0 0 0 0 0 0
0 0 0 0 0 0 0 0 0 1 0 0 2 0
1 0 0 0 0 0 0 0 0 0 0 0 0 0
1 0 0 0 0 0 0 0 0 0 0 0 0 0
0
OTU3522 1 0 0 0 0 0 2 0 0 0 0 0 0
0 2 0 0 0 1 0 0 0 0 0 0 11 17
0 0 0 0 0 0 2 0 0 0 0 0 1 0
1 0 0 0 0 0 0 1 0 0 0 0 0 1
0 0 1 2 0 0 0 0 0 0 0 1 0 0
0 0 0 0 1 0 0 0 0 0 1 0 0 0
0 0 0 1 0 0 2 0 1 0 0 0 0 0
1 0 0 0 0 0 0 0 0 0 0 0 0 0
0
OTU12220 1 0 0 0 0 0 0 0 0 0 0 0 0
0 0 0 0 0 0 0 0 0 0 0 0 0 0
0 0 0 0 0 0 0 0 0 0 0 0 0 0
0 0 0 0 0 0 0 0 0 0 0 0 0 0
0 0 0 0 0 0 0 0 0 0 0 0 0 0
0 0 0 0 0 0 0 0 0 0 0 0 0 0
0 0 0 0 0 0 0 0 0 0 0 0 0 0

0	0	0	0	0	0	0	0	0	0	0	0	0	0
0													
OTU12219	1	0	0	0	0	0	0	0	0	0	0	0	0
0	0	0	0	0	0	0	0	0	0	0	0	0	0
0	0	0	0	0	0	0	0	0	0	0	0	0	0
0	0	0	0	0	0	0	0	0	0	0	0	0	0
0	0	0	0	0	0	0	0	0	0	0	0	0	0
0	0	0	0	0	0	0	0	0	0	0	0	0	0
0	0	0	0	0	0	0	0	0	0	0	0	0	0
0	0	0	0	0	0	0	0	0	0	0	0	0	0
0													
OTU8509	1	0	1	0	0	0	0	0	1	0	0	0	0
0	4	0	0	0	0	1	0	0	0	0	0	2	0
0	1	0	0	0	0	3	0	0	0	1	0	2	0
0	0	0	0	0	0	0	1	0	0	0	0	0	0
0	0	0	0	0	0	0	0	0	0	0	1	0	0
0	0	2	1	0	0	0	0	0	0	0	0	0	0
0	0	0	0	0	0	0	0	0	0	0	0	0	0
0	0	0	0	0	0	0	0	0	0	0	0	0	0
0													
OTU14526	1	0	0	0	5	0	1	5	4	3	0	1	0
2	2	3	1	5	1	2	1	2	2	0	2	1	3
1	3	4	1	3	3	0	2	0	2	0	4	3	3
1	1	7	0	0	3	1	2	1	6	1	1	0	1
0	1	1	1	1	3	0	0	1	5	4	3	3	3
3	3	1	2	6	0	0	2	1	1	1	12	1	1
3	0	1	3	0	3	0	2	2	3	2	1	1	3
0	1	1	1	3	4	1	3	0	2	2	0	0	1
2													
OTU8510	1	0	0	0	0	0	0	0	0	0	0	0	0
0	0	0	0	0	0	0	0	0	0	0	0	0	0
0	0	0	0	0	0	0	0	0	0	0	0	0	0
0	0	0	0	0	0	0	0	0	0	0	0	0	0

0 0 0 0 0 0 0 0 0 0 0 0 0 0
0 0 0 0 0 0 0 0 0 0 0 0 0 0
0 0 0 0 0 0 0 0 0 0 0 0 0 0
0 0 0 0 0 0 0 0 0 0 0 0 0 0
0

OTU3189 2 1 0 0 0 0 0 0 1 0 0 0 0
0 0 0 9 0 0 2 0 5 0 2 1 33 0
1 9 0 0 5 0 3 0 0 0 12 0 0 0
0 0 2 0 2 0 0 0 20 10 3 0 5 0
0 0 0 0 1 12 4 15 39 0 15 0 2 0
2 0 0 0 0 3 0 6 2 0 0 15 0 0
0 4 0 0 0 0 11 2 0 0 0 0 0 0
0 0 0 0 0 0 0 0 0 0 2 0 0 0
0

OTU13554 1 0 1 1 1 2 2 0 2 1 1 1 0
0 1 0 0 1 0 0 0 0 1 1 0 0 0
1 0 2 1 0 0 0 0 0 0 0 0 1 0
1 0 0 0 0 0 1 1 0 2 0 0 1 0
0 0 0 1 1 1 0 1 0 0 0 0 0 0
0 0 1 1 4 0 1 1 0 0 0 5 0 0
1 1 1 1 0 1 0 1 0 0 1 0 0 0
0 1 0 1 0 1 0 1 0 1 1 1 0 0
1

OTU10624 2 0 2 1 4 3 1 3 4 1 2 3 1
4 13 3 5 2 1 0 1 0 1 4 2 0 1
0 1 6 2 0 2 0 3 0 0 1 3 6 2
5 2 0 2 0 1 5 0 2 3 0 0 1 0
1 1 2 1 1 3 0 0 3 1 3 3 4 1
5 3 1 1 2 1 0 4 1 1 1 4 0 1
4 0 1 2 2 8 1 0 4 1 4 0 2 0
0 2 2 0 4 2 1 1 2 3 1 3 1 3
7

OTU101	2	18	0	0	8	10	3	7	1	0	10	6	22
15	2	11	1	20	17	1	4	2	40	2	239	11	11
0	0	21	24	2	41	1	0	2	31	1	171	0	12
0	3	0	22	5	3	7	4	0	0	4	1	1	12
0	3	5	0	1	0	2	1	0	2	0	4	0	0
0	0	6	10	0	95	61	0	17	2	32	0	10	1
19	0	4	2	7	19	1	0	36	3	1	0	1	11
14	0	0	0	0	1	0	1	0	1	7	4	11	1
0													
OTU343	5	15	12	8	3	3	28	10	13	6	3	2	2
1	1	1	1	0	3	15	6	0	0	3	8	0	15
3	0	0	0	3	0	3	0	2	8	0	0	8	2
10	6	1	12	11	13	1	14	0	1	1	0	1	1
0	3	5	3	1	1	0	1	0	2	0	13	7	4
1	4	20	3	0	2	5	3	19	12	7	1	12	4
9	4	0	19	11	3	3	16	0	0	0	7	5	3
2	9	7	2	7	0	1	4	0	8	3	4	5	2
6													
OTU11019	1	0	0	0	0	0	0	0	0	0	0	0	0
0	0	0	0	0	0	0	0	0	0	0	0	0	0
0	0	0	0	0	0	0	0	0	0	0	0	0	0
0	0	0	0	0	0	0	0	0	0	0	0	0	0
0	0	0	0	0	0	0	0	0	0	0	0	0	0
0	0	0	0	0	0	0	0	0	0	0	0	0	0
0	0	0	0	0	0	0	0	0	0	0	0	0	0
0	0	0	0	0	0	0	0	0	0	0	0	0	0
0													
OTU224	1	0	0	0	1	2	28	6	43	9	0	0	0
0	1	0	0	0	2	0	0	2	0	0	0	0	0
3	0	0	0	3	1	0	0	3	0	0	0	17	0
26	6	0	7	0	1	0	0	1	1	29	5	2	0
0	0	0	5	1	0	0	1	2	0	0	0	6	0
0	11	0	0	12	0	0	0	17	28	0	0	0	22

0	5	0	42	0	0	1	12	0	0	0	4	19	0
0	15	17	8	17	11	3	5	13	14	2	1	0	32
8													
OTU237	8	25	25	0	3	19	24	2	45	33	0	0	0
0	6	0	0	0	0	4	1	0	0	0	2	3	11
1	0	0	1	0	0	0	0	0	0	5	0	6	0
20	0	0	1	0	8	0	0	0	0	0	0	0	0
0	9	0	4	0	0	0	2	1	0	1	0	3	0
0	23	62	0	11	0	0	2	0	35	2	2	0	9
0	5	0	64	1	2	2	23	0	1	0	37	21	0
1	15	12	33	12	0	1	22	7	19	1	0	0	19
8													
OTU5626	3	0	0	0	0	0	2	1	1	0	0	0	
0	0	0	0	0	0	0	0	0	0	0	0	1	0
1	0	0	1	0	0	0	0	0	0	0	0	0	0
0	0	0	3	0	1	0	0	0	0	0	0	0	0
0	0	0	4	0	0	0	0	1	0	0	0	1	0
0	0	0	0	0	0	0	0	0	0	0	1	0	1
0	1	0	1	0	2	0	1	0	0	0	1	0	0
0	0	0	0	0	0	0	0	0	0	0	0	0	1
0													
OTU374	1	1	0	0	0	1	3	2	3	0	0	0	17
0	0	0	0	0	0	3	0	1	10	0	11	3	3
0	0	0	0	0	0	0	0	0	0	37	0	1	0
2	0	1	0	0	0	1	8	4	0	1	3	0	0
0	1	1	6	5	3	6	0	1	1	0	6	2	0
0	4	0	0	1	1	0	0	0	0	0	0	0	16
0	0	0	1	0	0	0	0	0	0	0	0	6	0
0	3	3	4	2	1	2	1	1	5	2	0	2	24
33													
OTU269	1	0	5	16	0	2	0	1	11	9	0	0	0
0	2	3	41	0	2	0	2	2	0	0	0	0	0
3	1	0	0	0	0	1	1	0	0	1	0	1	0

2	0	1	0	1	17	0	1	0	4	3	41	0	1
0	0	30	1	0	2	0	48	12	0	6	0	13	0
0	12	1	5	2	0	0	5	0	3	0	14	0	2
0	2	0	0	1	2	3	39	0	9	0	4	8	0
0	5	15	36	11	35	8	16	12	11	3	2	3	24
2													
OTU14289	1	0	0	0	0	0	0	0	0	0	0	0	0
0	0	0	0	0	0	0	0	0	0	0	0	0	0
0	0	0	0	0	0	0	0	0	0	0	0	0	0
0	0	0	0	0	0	0	0	0	0	0	0	0	0
0	0	0	0	0	0	0	0	0	0	0	0	0	0
0	0	0	0	0	0	0	0	0	0	0	0	0	0
0	0	0	0	0	0	0	0	0	0	0	0	0	0
0	0	0	0	0	0	0	0	0	0	0	0	0	0
0													
OTU9498	3	0	0	0	0	0	1	0	2	1	0	0	1
0	1	1	1	0	0	0	0	0	2	0	0	1	0
1	0	3	3	0	2	1	0	0	1	0	1	2	1
1	0	4	0	0	1	0	0	1	0	0	0	0	0
0	2	0	1	0	1	0	1	1	1	0	2	1	0
2	1	0	0	4	0	0	2	1	0	0	3	1	0
0	2	0	1	0	3	0	1	0	0	1	1	1	0
0	2	0	0	2	2	0	0	0	0	0	0	0	0
5													
OTU12217	1	0	0	0	0	0	0	0	0	0	0	0	0
0	0	0	0	0	0	0	0	0	0	0	0	0	0
0	0	0	0	0	0	0	0	0	0	0	0	0	0
0	0	0	0	0	0	0	0	0	0	0	0	0	0
0	0	0	0	0	0	0	0	0	0	0	0	0	0
0	0	0	0	0	0	0	0	0	0	0	0	0	0
0	0	0	0	0	0	0	0	0	0	0	0	0	0
0	0	0	0	0	0	0	0	0	0	0	0	0	0
0													

OTU14288	1	0	0	0	0	0	0	0	0	0	0	0	0
0	0	0	0	0	0	0	0	0	0	0	0	0	0
0	0	0	0	0	0	0	0	0	0	0	0	0	0
0	0	0	0	0	0	0	0	0	0	0	0	0	0
0	0	0	0	0	0	0	0	0	0	0	0	0	0
0	0	0	0	0	0	0	0	0	0	0	0	0	0
0	0	0	0	0	0	0	0	0	0	0	0	0	0
0	0	0	0	0	0	0	0	0	0	0	0	0	0
0													
OTU4455	1	0	0	0	0	0	0	0	0	0	0	0	0
0	0	0	1	0	0	29	0	0	0	0	0	0	0
3	0	0	0	0	0	0	0	0	13	0	0	2	0
0	0	0	0	0	0	0	0	0	0	0	0	0	0
0	0	0	0	0	0	0	0	0	0	0	0	0	0
0	0	0	0	0	0	0	0	0	0	0	0	0	0
2	0	0	0	0	1	0	4	0	0	0	0	0	0
0	0	0	0	0	0	0	0	0	0	3	0	10	0
0													
OTU8504	1	0	0	0	0	0	0	0	0	0	0	0	0
0	0	0	0	0	0	0	0	0	0	0	0	0	0
0	0	0	0	0	0	0	0	0	0	0	0	0	0
0	0	0	0	0	0	0	0	0	0	0	0	0	0
0	0	0	0	0	0	0	0	0	0	0	0	0	0
0	0	0	0	0	0	0	0	0	0	0	0	0	0
0	0	0	0	0	0	0	0	0	0	0	0	0	0
0	0	0	0	0	0	0	0	0	0	0	0	0	0
0													
OTU12218	1	0	0	0	0	0	0	0	0	0	0	0	0
0	0	0	0	0	0	0	0	0	0	0	0	0	0
0	0	0	0	0	0	0	0	0	0	0	0	0	0
0	0	0	0	0	0	0	0	0	0	0	0	0	0
0	0	0	0	0	0	0	0	0	0	0	0	0	0
0	0	0	0	0	0	0	0	0	0	0	0	0	0

0	0	0	0	0	0	0	0	0	0	0	0	0	0
0	0	0	0	0	0	0	0	0	0	0	0	0	0
0													
OTU11688	1	0	0	1	0	0	0	0	0	0	0	0	0
0	0	0	0	0	1	0	0	0	0	0	0	2	0
0	0	0	0	0	0	1	0	0	0	0	0	0	1
0	0	0	1	1	0	0	0	0	0	0	0	0	0
0	0	0	0	0	0	0	0	0	0	0	0	0	0
0	0	0	0	0	0	0	0	0	0	0	0	0	0
0	0	0	0	0	0	1	0	0	0	0	0	0	0
0	0	0	0	0	0	0	0	0	0	0	1	0	0
0													
OTU11016	1	0	0	0	0	0	0	0	0	0	0	0	0
0	0	0	0	0	0	0	0	0	0	0	0	0	0
0	0	0	0	0	0	0	0	0	0	0	0	0	0
0	0	0	0	0	0	0	0	0	0	0	0	0	0
0	0	0	0	0	0	0	0	0	0	0	0	0	0
0	0	0	0	0	0	0	0	0	0	0	0	0	0
0	0	0	0	0	0	0	0	0	0	0	0	0	0
0	0	0	0	0	0	0	0	0	0	0	0	0	0
0													
OTU3468	1	0	3	1	1	4	0	0	0	12	0	6	2
1	0	0	0	0	0	0	7	0	0	0	0	0	1
1	0	0	0	0	2	0	2	1	1	0	0	0	0
0	0	0	0	0	0	0	0	0	0	0	0	0	4
0	0	0	0	0	0	0	0	0	0	0	0	0	0
0	0	0	2	0	0	0	0	1	1	0	0	7	2
1	0	11	1	1	0	0	0	0	1	13	0	0	5
2	0	0	0	0	0	0	0	0	0	3	2	0	0
0													
OTU1290	2	2	2	0	0	1	7	10	5	4	0	5	0
0	9	0	0	0	0	0	0	1	0	0	0	0	0
0	0	0	0	3	0	2	0	0	0	1	0	0	0

7	7	0	6	0	0	0	0	0	0	0	0	0	0
0	3	0	0	0	0	0	0	0	0	0	0	0	0
0	2	6	0	0	17	0	0	7	1	0	1	5	5
0	0	0	19	0	0	0	1	0	0	0	0	0	0
0	0	0	0	0	0	1	0	0	0	2	0	1	0
0													
OTU15181	1	0	0	0	2	0	0	0	0	0	2	0	0
0	0	0	0	0	0	0	0	0	1	0	2	0	0
0	0	1	0	0	0	0	0	0	0	0	3	0	1
0	0	0	3	0	0	0	0	0	0	0	0	0	0
0	1	0	0	0	0	0	0	0	0	0	0	0	0
0	0	0	0	0	0	0	0	1	0	1	0	0	0
1	0	0	0	0	0	0	0	0	0	0	0	0	0
0	0	0	0	0	0	0	0	0	0	0	1	0	0
0													
OTU10069	1	0	0	0	0	0	0	0	0	0	0	0	0
0	0	0	0	0	0	0	3	2	0	2	2	0	0
0	0	0	0	0	1	2	0	0	1	0	0	0	0
0	0	1	0	1	0	0	0	5	4	0	1	10	0
1	0	4	0	1	0	2	3	1	3	3	0	0	4
0	0	0	0	0	0	3	0	1	0	0	0	1	0
0	6	0	0	0	0	3	0	0	0	0	0	0	0
0	0	0	0	0	0	0	0	0	0	0	0	1	0
0													
OTU1789	1	0	0	0	1	0	2	4	8	1	0	1	0
0	0	0	2	0	0	0	1	5	0	0	0	0	0
0	2	0	0	6	0	12	0	0	0	24	0	2	0
5	0	0	0	0	0	0	0	0	0	0	0	0	0
0	0	0	0	0	0	0	0	0	0	0	0	0	1
0	21	0	0	0	0	0	0	0	1	3	13	0	1
0	0	0	0	0	3	0	3	0	0	0	0	4	0
0	0	101	40	0	2	3	2	34	5	0	1	0	1
0													

OTU98 3	20	16	0	0	5	1	5	8	0	0	0	0	0
4	1	0	1	0	1	1	1	0	1	0	2	2	0
0	0	0	0	0	1	0	1	1	17	0	16	0	4
0	1	0	1	5	0	7	0	0	0	1	2	1	0
10	0	1	1	3	0	1	1	0	0	2	2	0	4
118	2	0	1	2	0	21	1	0	1	3	1	2	0
24	0	5	0	0	0	0	0	0	0	25	29	0	0
247	332	55	87	135	141	23	22	79	0	2	0	94	85
OTU227	2	3	0	2	0	0	4	1	1	8	0	0	0
0	2	0	0	0	0	2	0	0	0	1	0	0	1
3	0	0	0	0	0	3	0	0	0	0	0	0	0
4	0	0	0	0	1	0	0	0	0	2	5	0	1
0	4	0	21	0	0	0	0	1	0	0	1	9	0
2	15	1	0	112	0	2	2	0	2	1	2	1	11
1	7	0	3	1	0	0	3	0	0	0	9	8	0
0	12	15	11	71	64	13	23	40	36	0	3	0	29
23													
OTU15601	1	0	0	0	2	1	0	1	1	2	2	0	0
0	1	0	0	0	0	0	0	0	0	0	1	0	1
0	0	0	0	0	1	0	0	0	1	0	0	0	0
0	1	0	0	0	0	0	0	2	0	1	0	0	1
0	0	0	0	0	0	1	0	0	0	0	0	0	0
1	0	0	0	0	0	0	0	1	1	0	1	0	0
0	1	0	0	0	0	0	1	1	0	0	0	0	0
0	1	0	0	0	0	0	0	0	0	0	0	0	0
0													
OTU256	3	10	5	1	8	10	23	12	4	25	0	3	1
2	10	14	0	2	0	24	5	1	1	1	18	0	0
6	0	7	1	0	0	1	12	2	5	0	0	0	0
17	27	0	9	0	4	3	18	0	0	0	3	2	3
0	20	2	4	1	3	0	1	0	1	1	0	20	2
2	0	12	1	11	32	1	4	5	31	12	2	13	11
11	4	0	14	5	5	0	5	0	8	5	0	1	4

2	0	1	3	2	0	3	2	1	10	3	5	13	1
10													
OTU5089	1	0	3	9	1	2	9	3	18	3	0	1	0
0	1	0	0	0	0	10	1	2	0	5	0	13	0
0	0	0	0	3	0	6	0	1	0	0	0	1	0
25	0	1	0	0	1	0	1	1	4	0	3	3	0
0	1	0	1	3	5	1	4	11	0	1	0	6	0
0	0	3	0	2	0	0	2	3	7	5	3	2	4
3	1	2	6	3	18	0	0	0	0	0	0	0	0
0	0	0	0	0	2	0	0	0	0	4	0	0	0
0													
OTU236	6	0	1	1	3	4	1	4	0	0	1	0	4
6	4	4	0	6	0	0	3	2	2	4	3	6	0
0	1	8	2	5	1	8	1	3	1	0	1	0	0
0	3	2	2	0	9	0	3	1	1	0	0	0	3
4	4	11	1	0	0	2	0	2	8	0	0	5	2
3	2	7	6	3	1	5	0	2	1	1	1	5	1
4	0	6	0	5	1	1	0	21	0	5	0	1	16
0	0	0	0	0	0	2	0	1	0	3	2	2	0
0													
OTU14291	1	0	0	0	0	0	0	0	0	0	0	0	0
0	0	0	0	0	0	0	0	0	0	0	0	0	0
0	0	0	0	0	0	0	0	0	0	0	0	0	0
0	0	0	0	0	0	0	0	0	0	0	0	0	0
0	0	0	0	0	0	0	0	0	0	0	0	0	0
0	0	0	0	0	0	0	0	0	0	0	0	0	0
0	0	0	0	0	0	0	0	0	0	0	0	0	0
0	0	0	0	0	0	0	0	0	0	0	0	0	0
0													
OTU6563	1	0	0	0	0	0	0	1	2	0	0	0	0
0	0	0	0	0	0	0	0	0	0	0	0	0	0
1	0	0	1	0	0	2	0	0	0	0	0	0	0
0	0	0	0	1	1	0	0	0	0	5	2	0	0

0	0	1	0	0	0	1	0	0	2	0	1	2	0
0	0	1	1	2	0	0	0	3	1	0	1	0	0
0	0	1	0	0	0	0	1	0	0	0	0	0	0
0	0	0	0	0	0	0	0	0	0	0	0	0	0
0													
OTU15879	1	0	0	0	0	0	0	0	0	0	0	0	0
0	0	0	0	1	0	0	0	0	0	0	0	0	0
0	0	0	0	0	0	0	0	0	0	0	0	0	0
0	0	0	0	0	0	0	0	0	0	0	0	0	0
0	0	0	0	0	0	0	0	0	0	0	0	0	0
0	0	0	0	0	0	0	0	0	0	0	0	0	0
0	0	0	0	0	0	0	0	0	0	0	0	0	0
0	0	0	0	0	0	0	0	0	0	0	0	0	0
0													
OTU8506	1	0	0	0	0	0	0	0	0	0	0	0	0
0	0	0	0	0	0	0	0	0	0	0	0	0	0
0	0	0	0	0	0	0	0	0	0	0	0	0	0
0	0	0	0	0	0	0	0	0	0	0	0	0	0
0	0	0	0	0	0	0	0	0	0	0	0	0	0
0	0	0	0	0	0	0	0	0	0	0	0	0	0
0	0	0	0	0	0	0	0	0	0	0	0	0	0
0	0	0	0	0	0	0	0	0	0	0	0	0	0
0													
OTU14290	1	0	0	0	0	0	0	0	0	0	0	0	0
0	0	0	0	0	0	0	0	0	0	0	0	0	0
0	0	0	0	0	0	0	0	0	0	0	0	0	0
0	0	0	0	0	0	0	0	0	0	0	0	0	0
0	0	0	0	0	0	0	0	0	0	0	0	0	0
0	0	0	0	0	0	0	0	0	0	0	0	0	0
0	0	0	0	0	0	0	0	0	0	0	0	0	0
0	0	0	0	0	0	0	0	0	0	0	0	0	0
0													

OTU242 2 1 17 50 5 3 11 14 32 13 0 23 2
1 2 5 6 2 0 39 62 4 0 1 0 3 1
100 11 0 2 4 0 2 36 4 5 3 0 17 1
19 6 15 11 4 11 0 0 2 18 4 32 3 1
1 7 2 54 9 4 9 30 27 4 15 3 69 6
5 4 1 15 12 7 0 4 6 10 0 53 5 43
4 0 5 15 21 8 34 12 0 8 18 3 4 10
21 4 5 6 9 2 1 1 4 6 12 6 12 5
1
OTU14298 1 0 0 0 0 0 0 0 0 0 0 0 0
0 0 0 0 0 0 0 0 0 0 0 0 0 0
0 0 0 0 0 0 0 0 0 0 0 0 0 0
0 0 0 0 0 0 0 0 0 0 0 0 0 0
0 0 0 0 0 0 0 0 0 0 0 0 0 0
0 0 0 0 0 0 0 0 0 0 0 0 0 0
0 0 0 0 0 0 0 0 0 0 0 0 0 0
0 0 0 0 0 0 0 0 0 0 0 0 0 0
0
OTU8505 1 0 0 0 0 0 0 0 0 0 0 0 0
0 0 0 0 0 0 0 0 0 0 0 0 0 0
0 0 0 0 0 0 0 0 0 0 0 0 0 0
0 0 0 0 0 0 0 0 0 0 0 0 0 0
0 0 0 0 0 0 0 0 0 0 0 0 0 0
0 0 0 0 0 0 0 0 0 0 0 0 0 0
0 0 0 0 0 0 0 0 0 0 0 0 0 0
0 0 0 0 0 0 0 0 0 0 0 0 0 0
0
OTU14426 1 0 3 0 0 0 0 1 2 0 0 0 0
0 0 0 0 0 0 0 0 0 0 0 0 0 0
0 0 0 0 0 0 0 0 0 0 0 0 0 0
0 0 0 0 0 0 0 0 0 0 0 0 0 0
0 0 0 0 0 0 0 0 0 0 0 0 0 0
0 0 0 0 2 0 0 0 0 1 0 0 0 0

0	0	0	0	0	2	0	0	0	0	0	0	0	0
0	0	0	0	0	0	0	0	0	0	0	0	0	0
0													
OTU3153	2	2	0	2	5	5	0	2	6	5	1	1	1
1	8	2	3	2	2	0	1	0	2	0	5	0	1
2	1	4	3	0	2	0	0	1	1	0	3	6	2
2	0	0	0	1	2	0	1	0	2	1	1	0	1
0	0	2	0	2	2	0	3	2	2	1	3	0	1
2	6	0	1	3	0	1	6	2	3	0	9	0	0
3	0	1	2	1	4	0	1	1	1	2	2	1	0
1	1	3	2	0	0	1	2	1	0	1	1	1	3
11													
OTU5049	1	0	0	0	1	2	0	0	1	2	0	0	1
0	0	1	0	1	0	0	0	1	0	0	1	1	0
0	0	1	1	0	1	0	1	0	1	1	0	1	1
0	2	0	0	1	0	0	0	1	0	0	0	0	0
0	2	1	0	1	0	0	0	0	1	0	0	0	1
2	1	0	0	3	0	0	1	0	1	0	1	0	0
0	1	0	0	2	1	1	0	1	0	1	0	0	0
0	1	3	0	0	1	0	2	1	1	1	0	0	0
3													
OTU8493	1	0	0	0	0	0	0	0	0	0	0	0	0
0	0	0	0	0	0	0	0	0	0	0	0	0	0
0	0	0	0	0	0	0	0	0	0	0	0	0	0
0	0	0	0	0	0	0	0	0	0	0	0	0	0
0	0	0	0	0	0	0	0	0	0	0	0	0	0
0	0	0	0	0	0	0	0	0	0	0	0	0	0
0	0	0	0	0	0	0	0	0	0	0	0	0	0
0	0	0	0	0	0	0	0	0	0	0	0	0	0
0													
OTU572	4	0	0	0	0	0	0	0	0	1	0	0	0
0	0	0	0	0	0	1	1	0	9	0	24	1	1
0	0	0	0	0	0	0	0	0	1	1	0	0	0

0 0 1 0 0 0 0 0 0 0 1 0 0 0
0 1 0 1 0 0 0 0 0 0 0 0 3 0
0 3 1 0 0 1 0 0 1 1 0 0 0 0
0 0 0 0 1 4 2 1 0 0 0 4 2 0
0 4 5 8 1 1 0 1 1 4 0 0 0 2
7
OTU1131 3 0 0 0 1 0 5 5 11 5 0 2 0
0 0 0 0 0 0 0 0 0 0 0 0 1 0
0 0 0 0 0 0 0 0 5 0 0 0 14 3
7 0 0 2 1 4 0 2 0 0 1 1 0 1
0 0 0 2 0 0 1 0 1 0 2 0 4 0
2 74 0 0 10 0 0 1 0 10 3 3 0 3
0 2 1 3 0 0 0 12 0 0 0 58 53 0
0 87 93 157 135 107 87 103 39 118 0 14 0 137
52
OTU13194 1 1 0 0 0 0 0 0 0 0 0 0 0
0 1 0 0 0 0 0 3 0 0 0 0 0 0
0 0 0 0 0 0 0 0 1 0 0 0 1 0
1 0 0 0 0 0 1 1 0 0 0 0 0 3
0 0 0 0 0 0 0 0 0 0 0 0 0 0
0 0 0 0 0 0 0 0 0 0 0 0 0 0
0 0 0 0 0 0 0 0 0 0 1 0 0 0
3 0 0 0 0 0 0 0 0 0 0 0 0 0
0
OTU116 4 0 8 28 32 22 7 88 5 10 2 9 1
1 7 1 22 0 10 1 23 9 0 4 0 30 1
14 3 10 0 0 0 0 16 8 16 2 1 7 2
209 11 4 21 0 13 1 15 2 5 3 9 5 16
1 29 8 37 2 3 7 4 1 4 0 0 7 4
9 1 24 0 55 34 1 1 1 66 5 14 24 6
0 14 0 0 23 4 6 40 1 0 9 3 2 3
6 1 1 3 2 1 4 1 4 2 6 1 16 7
0

OTU13511	1	0	0	0	0	0	0	0	0	0	0	0	0
0	0	0	0	0	0	0	0	0	0	0	0	0	0
0	0	0	0	0	0	0	0	0	0	0	0	0	0
0	0	0	0	0	0	0	0	0	0	0	0	0	0
0	0	0	0	0	0	0	0	0	0	0	0	0	0
0	0	0	0	0	0	0	0	0	0	0	0	0	0
0	1	0	0	0	0	0	0	0	0	0	0	0	0
0	0	0	0	0	0	0	0	0	0	0	0	0	0
0													
OTU8494	1	0	0	0	0	0	0	0	0	0	0	0	0
0	0	0	0	0	0	0	1	0	0	0	1	0	0
0	0	0	0	0	0	0	0	0	0	0	0	0	0
0	0	0	0	0	0	0	0	0	0	0	0	0	0
0	0	0	0	0	0	0	0	0	0	0	0	0	0
0	0	0	0	0	0	0	0	0	0	0	0	0	0
0	0	0	0	0	0	0	0	0	0	0	0	0	0
0	0	0	0	0	0	0	0	0	0	0	0	0	0
0													
OTU11606	1	0	0	0	0	0	0	0	0	0	0	0	0
0	0	0	3	0	0	0	2	3	0	2	1	0	0
0	10	0	0	3	0	0	0	0	0	3	0	0	0
0	0	6	1	1	0	1	0	3	2	1	0	1	0
2	0	1	0	2	1	4	0	1	2	1	0	0	0
3	0	0	0	0	0	0	0	0	0	0	0	3	0
1	2	0	0	0	0	1	0	0	0	0	0	0	0
0	0	0	0	0	0	0	0	0	0	0	0	0	0
0													
OTU12897	4	4	0	24	50	0	1	0	0	0	12	0	0
0	3	0	28	0	0	0	1	0	1	86	0	4	0
0	4	12	3	5	0	9	7	1	0	3	1	0	18
0	2	1	1	43	9	14	6	67	0	43	0	185	14
53	14	87	1	35	3	178	0	0	68	1	6	1	3
1	0	1	2	0	0	4	6	3	5	2	0	2	0

2 8 15 2 3 2 1 0 1 2 2 3 0 47
0 0 0 0 0 2 0 4 2 0 1 0 5 0
1
OTU468 3 0 0 0 0 5 0 0 0 0 1 0 2
0 0 0 0 5 0 0 8 0 0 5 0 2 0
0 0 0 3 4 0 0 0 0 7 0 0 0 0
0 0 0 0 0 0 0 0 0 1 0 0 0 0
0 0 3 0 0 0 0 0 0 0 0 0 0 0
0 0 0 2 0 0 0 0 0 0 0 0 0 0
0 0 3 0 0 0 0 0 0 1 0 0 0 0
1 0 0 1 0 0 0 0 0 0 0 0 0 0
0
OTU2957 4 0 0 0 0 0 0 0 0 0 0 0 0
0 0 0 0 0 0 0 0 0 0 0 0 0 0
0 0 0 0 0 0 0 0 0 0 0 0 0 0
0 0 0 0 0 0 0 0 0 0 0 0 0 0
0 0 0 0 0 1 0 0 0 0 0 0 0 0
0 0 0 0 0 0 0 0 0 0 0 0 0 4
0 1 0 0 0 0 0 0 0 0 0 0 0 0
0 0 0 0 0 0 0 0 0 1 0 0 0 0
0
OTU14892 1 0 0 0 0 0 0 0 0 0 0 0 0
0 0 0 0 0 0 0 0 0 0 0 0 0 0
0 0 0 0 0 0 0 0 0 0 0 0 1 0
0 0 0 0 0 0 0 0 0 0 0 0 0 0
0 0 0 0 0 0 0 0 0 0 0 1 0 0
0 0 0 0 0 0 0 0 0 0 0 0 0 0
0 0 0 0 0 0 0 0 0 0 0 0 1 0
0 0 0 0 0 0 0 0 0 0 0 0 0 0
0
OTU925 11 9 15 8 25 16 9 11 15 22 14 8 7
18 9 11 5 27 8 2 12 6 19 4 11 9 6
11 5 27 15 2 13 1 15 3 14 11 18 36 13

11	8	23	9	5	9	5	4	15	43	8	9	5	8
2	4	10	9	7	10	4	16	19	9	14	11	13	6
25	16	13	9	25	9	4	17	8	9	4	55	6	10
13	6	7	5	6	38	5	20	5	2	17	5	12	3
5	13	18	6	9	14	7	16	1	15	3	7	1	10
27													
OTU13595	2	0	2	1	1	1	3	2	3	4	2	1	1
2	4	1	0	6	2	0	2	2	2	0	0	0	1
1	1	3	2	1	2	0	2	1	1	0	1	4	2
5	2	1	1	2	0	1	1	1	5	1	1	0	0
1	2	4	2	1	0	0	4	4	1	1	2	4	2
6	4	2	0	4	0	0	6	1	2	0	8	1	2
4	1	1	2	1	6	0	1	1	3	4	1	0	1
1	2	2	0	0	2	1	3	0	2	1	1	0	1
5													
OTU243	4	1	0	0	0	0	1	0	3	1	0	1	1
0	0	0	1	1	0	6	0	0	0	2	0	20	1
2	1	0	0	2	1	1	0	0	0	1	0	1	0
2	3	0	0	3	0	0	0	0	2	6	3	0	0
1	0	2	6	0	1	0	4	1	0	0	0	5	0
2	9	0	2	4	0	16	2	3	4	1	12	4	7
1	11	0	2	2	0	24	0	0	0	0	1	8	0
0	24	0	15	2	12	24	4	3	11	0	1	1	0
9													
OTU8492	1	0	0	0	0	0	0	0	0	0	0	0	0
0	0	0	0	0	0	0	0	0	0	0	0	0	0
0	0	0	0	0	0	0	0	0	0	0	0	0	0
0	0	0	0	0	0	0	0	0	0	0	0	0	0
0	0	0	0	0	0	0	0	0	0	0	0	0	0
0	0	0	0	0	0	0	0	0	0	0	0	0	0
0	0	0	0	0	0	0	0	0	0	0	0	0	0
0	0	0	0	0	0	0	0	0	0	0	0	0	0
0													

OTU319 3 0 12 6 3 7 3 6 4 9 0 6 0

3 4 3 8 0 8 2 15 2 3 3 0 0 0

8 0 0 0 2 0 0 10 0 10 2 0 8 0

1 22 12 6 0 11 3 3 0 2 0 4 1 1

10 9 0 10 0 0 3 5 2 1 0 2 5 0

1 4 0 17 17 0 1 8 5 8 2 1 2 3

3 1 4 16 3 4 2 4 0 1 0 1 13 0

4 13 1 13 7 1 0 4 4 12 1 2 5 14

11

OTU9372 4 0 10 6 3 5 15 7 7 44 0 38 7

17 5 7 1 0 0 4 48 0 0 1 0 4 1

15 0 0 7 0 8 13 14 0 13 0 0 6 0

2 0 0 3 0 0 0 1 0 0 1 3 0 12

0 0 0 1 0 0 0 0 4 0 0 0 4 1

1 4 0 24 5 2 1 2 6 7 1 13 32 24

6 2 69 23 2 3 0 2 1 6 40 3 2 55

15 10 17 29 20 17 6 11 6 0 17 9 5 6

1

OTU3005 2 0 0 0 0 0 0 0 1 0 1 1 0

1 0 0 1 0 0 0 0 0 0 0 0 0 0

0 0 3 0 0 1 0 0 0 0 1 1 1 0

0 0 0 1 1 0 0 0 1 0 0 0 0 0

0 0 0 0 0 0 0 0 1 0 0 0 0 1

1 0 2 0 1 1 0 1 0 0 1 2 0 1

0 0 0 0 0 0 0 0 0 0 0 0 0 0

1 0 0 0 0 0 0 1 0 0 1 0 0 0

1

OTU11372 3 2 3 1 1 2 2 4 2 4 4 3 1

2 4 1 0 1 1 1 2 1 5 2 4 2 1

3 0 4 3 0 3 1 2 0 1 1 3 4 2

2 3 5 4 3 2 2 2 2 6 1 3 1 2

0 0 3 2 2 3 0 2 6 2 3 1 1 2

1 2 2 0 5 1 0 7 2 2 2 8 1 4

2	0	2	1	0	5	0	4	0	1	0	2	3	0
1	0	1	3	2	1	1	3	2	0	0	0	0	1
3													
OTU5219	1	0	0	0	0	0	1	0	1	0	0	0	0
0	0	0	0	0	0	1	0	0	0	0	0	1	0
0	0	0	0	0	0	0	0	0	0	0	0	4	0
1	0	0	0	0	0	0	1	0	0	0	1	0	0
0	0	0	0	0	0	0	0	1	0	0	0	1	0
0	0	3	1	0	0	0	0	0	0	0	0	0	3
0	0	1	3	0	0	0	0	0	0	0	0	0	0
0	0	0	2	1	0	0	0	0	0	0	0	0	0
0													
OTU24 5	0	2	2	1	0	0	1	0	5	10	2	40	11
1	1	2592	1	0	1	4	254	3	232	1	7	12	6
929	3	18	629	3	25	29	0	4	35	5	309	2	8
0	3172	16	629	3	10	0	1053	357	298	584	1645	5	1284
3	903	10	1352	52	550	971	311	1264	487	168	20	162	532
0	0	1	0	0	0	2547	12	25	0	7	5	0	8
70	85	0	5	5	54	1	64	8	5	0	0	23	8
0	0	0	0	3	0	0	9	2	10	71	9	4	0
OTU9668	1	1	1	0	0	0	0	1	1	0	0	0	0
0	0	0	0	0	0	0	0	0	0	0	8	0	0
0	0	5	0	7	2	1	0	0	0	0	0	5	1
0	0	0	0	1	0	2	0	0	0	3	0	0	6
0	0	4	0	0	0	0	0	0	1	0	0	0	2
0	0	7	0	0	0	0	0	0	0	0	2	0	0
0	2	5	0	0	0	0	0	0	0	0	0	0	0
0	0	0	0	0	0	0	0	0	5	0	0	0	0
0													
OTU1402	2	1	2	3	4	1	1	1	1	2	1	3	0
5	8	4	2	2	0	2	0	2	1	2	3	2	1
1	0	0	1	2	1	0	0	4	3	0	1	3	1
2	2	3	3	0	4	1	1	1	4	1	0	0	1

0	3	2	0	1	2	1	1	1	0	4	1	2	0
3	2	1	2	8	2	0	5	2	1	1	4	0	1
1	2	1	2	3	4	0	1	1	0	2	1	0	1
1	0	0	1	0	4	0	2	1	1	2	1	1	2
14													
OTU9094	1	0	0	1	1	0	0	1	1	0	0	0	0
0	0	0	1	0	2	0	0	0	1	0	0	0	0
0	0	0	1	1	0	0	0	0	1	1	0	0	0
0	0	0	0	0	0	0	0	1	0	0	0	0	0
0	0	0	0	0	1	0	0	1	0	0	0	0	0
0	0	0	0	0	0	0	0	0	0	0	0	0	0
0	0	0	0	0	0	0	1	0	1	0	0	1	1
1	0	0	0	0	1	0	1	0	1	0	0	0	0
0													
OTU6939	2	1	0	0	4	1	1	1	2	4	0	2	0
3	17	1	4	1	1	0	1	2	3	1	1	0	1
0	0	4	3	3	3	2	2	1	0	2	1	5	2
3	2	4	4	1	2	2	3	0	3	2	3	2	0
3	3	0	0	0	3	2	0	0	1	3	2	0	1
5	3	1	1	1	3	0	3	0	2	2	4	2	1
2	0	1	1	5	5	1	4	1	2	6	4	1	3
2	2	3	1	3	1	3	3	1	2	1	2	1	0
12													
OTU377	3	1	5	1	2	0	1	3	1	0	0	0	0
0	7	0	1	0	0	0	0	0	0	0	0	20	1
0	1	0	0	0	0	0	0	0	0	4	0	1	0
1	0	0	0	1	4	0	4	0	0	2	0	0	7
0	0	0	4	1	0	0	1	0	1	0	2	1	0
1	1	1	1	2	1	0	1	0	0	0	2	0	3
0	0	0	0	0	0	0	3	0	0	0	0	0	0
0	0	0	0	0	0	0	1	0	1	1	0	2	3
0													

OTU64 4 18 17 0 5 11 44 37 248 27 0 17 3 2
3 0 58 3 21 45 0 90 3 42 10 146 2 118
53 3 0 169 0 91 0 4 4 6 0 69 3 101
15 52 87 57 100 1 259 9 40 37 48 24 12 20
140 1 82 44 21 7 77 65 2 26 17 87 15 12
38 88 33 52 10 0 375 5 227 2 127 6 55 1
56 0 42 1 31 72 48 4 14 0 22 64 4 2
62 50 33 31 14 16 36 20 38 3 0 3 40 17
OTU836 2 2 4 0 0 0 0 0 7 0 0 0 0
0 0 0 0 0 0 0 0 0 0 0 0 2 0
0 0 0 0 0 0 0 0 0 0 0 0 4 0
1 0 0 0 0 0 1 0 0 0 0 0 0 1
0 0 0 0 0 0 0 0 0 0 0 0 0 0
0 4 0 0 3 0 0 0 0 4 0 0 0 0
0 1 0 42 0 0 0 0 0 0 0 3 0 0
0 0 0 2 1 0 0 3 2 2 0 0 0 7
0
OTU4726 1 0 0 0 0 0 2 0 1 3 0 4 0
0 2 1 1 1 0 1 2 0 0 2 0 0 0
1 0 0 0 0 1 1 2 6 0 0 0 0 0
0 0 0 0 0 2 0 1 0 0 1 2 2 5
0 0 0 0 2 0 0 0 1 0 1 0 0 1
0 0 0 0 0 1 0 2 1 0 0 0 1 0
1 0 2 0 0 1 1 0 0 2 4 1 0 3
4 0 0 0 0 0 0 0 0 1 0 1 1 0
0
OTU12207 1 0 0 0 0 0 0 0 0 0 0 0 0
0 0 0 0 0 0 0 0 0 0 0 0 0 0
0 0 0 0 0 0 0 0 0 0 0 0 0 0
0 0 0 0 0 0 0 0 0 0 0 0 0 0
0 0 0 0 0 0 0 0 0 0 0 0 0 0
0 0 0 0 0 0 0 0 0 0 0 0 0 0
0 0 0 0 0 0 0 0 0 0 0 0 0 0

0	0	0	0	0	0	0	0	0	0	0	0	0	0
0													
OTU2815	5	2	40	0	0	0	0	0	0	0	0	0	0
0	0	0	0	0	0	0	1	0	0	0	0	0	0
0	0	0	0	0	0	0	0	0	1	0	10	0	5
0	0	0	0	10	0	0	0	0	0	0	0	0	0
0	0	0	0	0	0	0	0	0	0	0	0	1	0
0	0	0	0	1	0	0	2	0	0	0	1	0	0
0	4	0	0	0	3	0	0	0	0	0	0	0	0
0	0	0	0	0	13	0	0	0	9	0	0	0	0
0													
OTU245	1	4	5	0	2	44	0	1	2	2	0	0	0
0	0	0	0	0	0	0	0	0	0	0	0	7	0
0	0	0	0	0	0	0	0	0	0	0	0	0	0
1	0	0	2	0	0	0	2	0	0	0	0	0	4
0	11	0	4	0	0	0	0	0	0	0	0	0	0
0	6	1	0	12	0	0	1	0	0	1	0	0	15
0	1	0	20	0	4	0	0	0	0	0	8	26	0
0	4	14	32	14	4	8	6	2	3	0	0	0	91
19													
OTU286	1	139	16	0	0	0	0	1	0	5	0	1	0
0	0	0	0	0	0	0	0	0	0	0	0	0	0
0	0	0	0	0	0	0	0	0	0	0	0	0	0
2	2	0	0	0	0	0	1	0	1	0	1	0	0
0	1	0	2	0	0	0	0	0	0	0	0	4	0
0	3	0	0	25	7	0	0	0	4	8	0	0	0
0	4	0	0	0	2	0	15	0	0	0	0	1	0
0	1	0	0	1	0	0	10	0	0	0	0	0	0
3													
OTU7613	1	0	0	0	1	2	0	1	2	2	1	1	0
0	2	1	0	3	2	2	2	2	3	0	0	1	1
1	2	0	0	0	0	0	3	1	1	0	2	2	0
1	1	0	2	0	0	0	0	0	5	0	0	1	0

1	0	1	0	0	0	0	2	2	0	1	0	1	1
4	1	3	1	1	2	2	3	1	1	0	7	0	2
2	0	1	0	1	2	0	1	0	0	0	0	0	1
0	4	0	2	3	2	1	4	1	2	0	0	1	3
2													
OTU14299	1	0	0	0	0	0	0	0	0	0	0	0	0
0	0	0	0	0	0	0	0	0	0	0	0	0	0
0	0	0	0	0	0	0	0	0	0	0	0	0	0
0	0	0	0	0	0	0	0	0	0	0	0	0	0
0	0	0	0	0	0	0	0	0	0	0	0	0	0
0	0	0	0	0	0	0	0	0	0	0	0	0	0
0	0	0	0	0	0	0	0	0	0	0	0	0	0
0	0	0	0	0	0	0	0	0	0	0	0	0	0
0													
OTU841	1	7	3	3	0	1	1	1	0	1	0	2	0
1	6	3	0	0	1	2	3	0	1	0	1	0	1
0	0	0	0	0	0	2	1	1	3	0	0	0	1
0	3	0	3	0	0	0	1	0	0	0	0	0	0
0	5	0	0	0	0	0	0	0	0	0	0	2	0
0	0	1	0	1	10	0	0	0	0	0	0	3	0
0	0	2	0	0	3	0	1	0	1	1	0	0	0
1	0	0	0	0	0	0	0	0	0	0	0	0	0
0													
OTU999	1	3	30	0	1	0	0	0	0	1	0	0	0
0	0	1	0	0	0	0	0	0	2	0	0	0	0
0	0	0	0	0	0	0	0	0	0	0	9	0	1
0	0	2	0	5	0	0	0	0	0	0	0	0	0
0	0	0	0	0	0	0	0	0	0	0	0	0	0
1	0	2	0	0	0	0	0	0	0	0	0	0	0
0	4	0	0	0	0	0	0	0	0	0	0	1	0
0	0	0	0	0	5	0	0	0	3	0	0	0	0
0													

OTU8489 1 0 0 0 0 0 0 0 0 0 0 0 0
0 0 0 0 0 0 0 0 0 0 0 1 0 0
0 0 0 0 0 0 0 0 0 0 0 0 0 0
0 0 0 0 0 0 0 0 0 0 0 0 0 0
0 0 0 0 1 0 0 0 0 0 0 0 0 0
0 0 0 0 0 0 0 0 0 0 0 0 0 0
0 0 0 0 0 0 0 0 0 0 0 0 0 0
0 0 0 0 0 0 0 0 0 0 0 0 0 0
0
OTU7721 1 0 0 0 0 0 1 0 0 0 1 0 0
0 0 0 0 0 0 0 0 0 0 0 0 0 0
0 0 0 0 0 0 0 0 0 0 0 0 0 0
0 0 0 0 0 0 0 0 0 0 0 0 0 0
0 0 0 0 0 0 0 0 0 0 0 0 0 0
1 0 0 0 1 0 0 0 0 0 0 0 0 0
0 0 0 0 0 0 0 0 0 0 0 0 0 0
0 0 0 0 0 0 0 0 0 1 0 0 0 0
0
OTU821 1 0 1 0 0 0 0 1 1 0 0 0 0
0 0 0 0 0 1 0 3 1 0 0 0 0 0
0 0 0 0 0 0 0 0 0 0 0 0 0 0
0 0 1 0 1 0 0 0 0 0 0 0 0 0
0 0 0 0 1 0 0 0 0 0 0 0 0 0
0 3 0 0 0 0 0 0 0 0 0 1 0 0
0 0 0 3 0 1 0 1 0 0 0 0 1 0
0 3 4 4 2 2 0 2 0 3 0 0 0 2
0
OTU5895 1 2 0 1 4 0 0 1 1 2 0 0 1
3 1 1 0 4 1 0 1 3 3 1 0 2 0
0 2 1 0 0 2 0 1 0 1 1 0 2 1
0 2 2 0 0 0 0 1 1 7 1 1 0 0
0 0 1 2 0 3 0 1 1 1 0 0 2 0
2 4 0 1 5 0 0 0 0 0 1 1 2 1

0	2	0	0	1	3	1	1	1	4	2	2	0	3
2	0	0	0	2	1	1	2	0	1	2	1	1	4
1													
OTU8488	2	0	0	0	0	1	0	0	0	0	0	0	0
0	0	0	0	0	0	0	0	0	0	0	0	0	0
0	0	0	1	1	0	0	0	0	0	0	0	0	0
0	0	0	0	0	0	0	0	0	0	0	0	0	0
0	0	0	1	0	0	0	0	0	0	0	0	0	0
0	0	1	0	0	0	0	0	0	0	0	0	0	0
0	0	0	0	0	0	0	0	0	0	0	0	0	0
0	0	0	0	0	0	0	0	0	0	0	0	0	0
0													
OTU10767	3	2	3	2	7	4	1	2	12	7	2	5	1
0	10	5	1	5	3	1	1	3	4	2	4	3	3
1	1	6	3	1	5	1	2	1	6	4	5	6	4
6	3	4	3	4	3	1	3	4	10	3	1	1	2
3	1	5	5	4	6	5	1	4	2	5	1	5	1
10	6	2	3	8	4	0	5	0	5	1	9	0	5
1	4	1	3	1	8	5	2	3	0	2	2	6	1
1	3	3	2	3	5	1	1	2	1	1	3	2	2
11													
OTU826	1	0	0	1	1	1	0	1	0	2	0	2	0
1	0	0	0	0	1	4	0	1	0	0	0	2	1
2	0	0	0	0	0	1	0	0	0	0	0	1	1
6	0	0	0	0	1	1	1	0	0	0	0	0	0
0	3	0	0	0	0	0	0	0	0	0	0	0	0
0	2	1	0	0	0	0	0	1	0	0	0	0	0
2	0	1	2	1	0	0	1	0	1	2	2	0	2
0	0	0	1	0	0	0	2	0	0	0	0	0	3
0													
OTU10972	1	0	0	0	0	0	0	0	0	0	0	0	0
0	0	1	0	0	0	0	0	0	0	0	1	0	0
1	0	0	1	0	0	0	0	0	0	0	0	0	0

0	1	0	1	1	0	0	0	0	0	0	0	0	0
0	1	0	0	0	0	0	0	0	0	1	0	0	0
0	0	0	0	0	0	0	0	0	0	0	1	0	0
0	1	0	0	0	0	0	0	0	0	0	0	0	0
0	1	0	0	0	1	0	0	0	0	0	0	0	0
0													
OTU10973	1	0	0	0	0	0	0	0	0	0	0	0	0
0	0	0	0	0	0	0	0	0	0	0	0	0	0
0	0	0	0	0	0	0	0	0	0	0	0	0	0
0	0	0	0	0	0	0	0	0	0	0	0	0	0
0	0	0	0	0	0	0	0	0	0	0	0	0	0
0	0	0	0	0	0	0	0	0	0	0	0	0	0
0	0	0	0	0	0	0	0	0	0	0	0	0	0
0	0	0	0	0	0	0	0	0	0	0	0	0	0
0													
OTU12205	1	0	0	0	1	0	0	0	1	0	1	0	0
0	0	0	0	1	0	0	0	0	0	0	0	1	0
0	0	0	0	0	0	0	0	0	0	0	0	0	0
0	0	1	0	0	1	0	0	0	1	0	0	0	0
0	2	0	0	0	0	0	0	0	0	0	1	0	0
2	0	0	0	0	0	0	0	0	1	1	2	0	0
0	1	0	0	0	0	0	0	0	0	0	0	0	0
0	2	1	0	0	0	0	0	0	0	0	0	0	0
1													
OTU3350	1	7	6	8	0	2	1	0	3	0	0	0	1
0	0	0	11	0	0	0	0	8	0	11	0	6	1
11	13	0	0	1	0	5	0	0	1	2	0	2	0
2	0	15	1	1	0	0	0	7	16	5	1	6	0
2	0	1	0	1	0	2	5	3	0	8	1	4	6
5	0	0	0	0	0	0	12	0	0	1	8	0	4
0	13	0	0	0	0	3	0	0	0	0	0	0	0
0	0	2	0	0	0	0	0	0	0	0	0	0	0
0													

OTU4358	1	0	0	0	0	0	1	0	2	1	0	0	0
0	0	0	0	0	0	5	0	0	0	0	0	0	0
1	0	0	0	0	0	1	0	0	0	0	0	0	0
1	1	0	1	0	0	0	0	0	0	0	1	0	0
0	0	0	0	0	0	0	0	0	0	0	0	0	0
0	0	1	1	3	0	0	0	0	1	0	0	0	0
0	0	0	0	0	0	0	0	0	0	0	0	0	0
0	0	0	0	0	0	0	0	0	0	0	0	0	0
0													
OTU341	1	3	6	0	0	0	2	2	4	3	1	4	0
0	1	0	0	4	1	9	0	0	0	0	12	0	7
1	0	0	0	1	0	1	0	0	5	0	0	3	0
1	0	0	0	8	0	0	0	0	0	2	0	0	0
0	0	0	0	0	0	0	0	0	0	0	1	2	0
0	3	3	0	3	0	3	0	0	3	0	1	4	0
0	0	0	2	2	1	0	2	0	2	0	0	2	3
0	6	1	9	0	3	2	0	1	10	2	0	0	4
1													
OTU8682	1	0	4	1	0	0	5	4	3	2	2	2	0
2	7	3	2	4	1	0	1	2	2	1	1	3	2
4	2	4	7	1	3	1	2	0	3	1	4	3	1
4	1	1	3	1	4	2	0	2	7	0	2	1	0
1	0	0	0	1	1	0	1	0	2	2	1	1	1
5	3	0	0	1	4	2	5	0	1	4	4	1	2
4	0	0	1	1	2	1	2	1	2	2	0	1	2
0	0	2	1	3	3	0	2	2	1	2	0	3	3
8													
OTU6780	8	1	10	0	1	0	2	9	3	2	0	0	0
0	6	0	0	0	0	5	0	0	0	0	0	0	0
0	0	0	0	0	0	0	0	0	0	0	0	1	0
9	0	0	0	0	0	0	1	0	0	0	1	0	0
0	0	0	1	0	0	0	0	0	0	0	0	0	0
0	0	0	0	1	0	0	0	0	6	0	1	0	8

0	0	0	15	0	1	0	2	0	0	0	0	0	0
0	0	0	0	0	0	0	0	0	0	0	0	0	0
0													
OTU129	3	4	20	13	1	4	4	3	17	7	1	4	1
1	8	27	19	0	0	4	11	3	1	0	7	30	2
53	46	0	0	0	3	1	3	5	9	14	1	22	0
9	3	19	8	26	25	1	1	10	14	13	62	0	1
3	13	9	97	6	27	11	32	41	3	12	9	17	2
7	24	3	29	18	2	0	0	4	30	2	42	0	6
2	22	2	16	2	15	37	26	2	0	12	20	14	3
15	54	34	48	52	24	19	28	10	11	8	2	20	30
28													
OTU1139	2	3	4	1	2	8	2	4	3	3	0	11	0
2	7	5	0	0	12	6	2	1	0	1	0	3	4
1	0	0	0	1	0	1	4	1	2	0	0	0	0
0	5	0	1	0	1	0	2	0	0	0	0	0	1
0	3	0	3	1	0	0	2	0	1	0	0	3	0
0	0	1	1	7	3	0	1	3	1	0	0	0	0
0	0	0	2	0	0	0	0	3	2	3	5	1	1
2	1	1	1	1	0	0	4	0	2	3	3	2	0
0													
OTU4279	5	1	2	1	3	2	3	4	4	4	1	2	0
2	5	0	2	4	4	4	2	1	1	1	0	1	3
1	3	4	6	0	3	0	2	1	1	2	3	1	1
2	2	1	4	2	0	0	3	2	6	2	1	0	0
0	4	3	1	4	3	0	4	1	3	3	4	1	0
6	8	1	1	5	0	0	5	0	3	0	9	1	4
2	4	1	1	1	4	0	5	1	2	1	3	2	2
1	1	2	0	3	2	1	2	1	3	2	2	0	1
7													
OTU4384	2	1	0	0	2	0	0	1	1	0	0	1	0
0	1	0	1	3	1	0	1	1	2	0	1	0	2
1	0	3	2	0	0	0	0	0	0	0	0	0	1

3	0	2	0	0	0	0	0	0	0	1	0	0	0
1	0	0	0	0	2	0	1	2	0	1	0	2	1
0	0	0	0	0	0	0	0	0	0	0	1	1	1
0	0	0	0	0	2	2	0	0	0	0	1	0	0
1	0	0	0	0	0	0	0	0	1	0	1	0	0
0													
OTU4230	4	4	6	2	11	6	5	15	5	10	5	9	1
2	5	8	5	11	10	3	6	7	6	2	2	3	3
1	1	15	10	2	12	1	5	6	6	8	7	15	8
6	10	10	3	11	5	2	3	14	22	6	3	1	2
3	2	8	4	3	4	1	5	7	2	2	4	6	5
9	3	12	4	15	4	2	9	2	0	1	28	1	6
8	8	1	4	1	9	1	11	2	1	10	2	2	2
2	3	4	3	5	9	2	4	0	9	3	3	2	4
9													
OTU9512	1	0	0	1	0	0	0	0	0	0	0	0	0
0	0	0	0	0	0	0	0	0	0	0	0	0	3
0	0	0	0	0	0	0	0	0	0	0	0	0	0
0	0	0	0	1	0	0	1	0	0	0	0	0	1
0	0	0	0	0	0	0	0	0	0	0	0	1	0
0	0	0	0	2	0	0	0	0	0	0	0	0	0
0	0	0	0	0	1	0	0	0	0	0	0	0	0
0	0	0	0	0	0	0	0	0	0	0	0	0	0
0													
OTU5527	1	0	0	0	0	0	0	0	0	1	0	1	0
0	0	0	0	0	0	0	1	0	0	0	0	1	0
0	0	0	0	0	0	0	2	2	0	0	0	0	0
0	0	0	0	0	0	1	2	0	0	0	0	0	0
0	0	0	0	0	0	0	0	0	0	0	0	1	0
0	0	0	0	0	0	0	0	0	0	0	0	0	0
0	0	0	1	0	0	0	0	0	0	0	0	0	0
1	0	0	0	0	0	0	0	0	0	0	1	0	0
0													

OTU13095 3 1 4 1 8 4 6 6 2 3 4 5 1

2 7 6 3 9 3 2 2 2 3 0 0 0 2

3 3 5 4 0 3 3 4 2 1 1 6 8 1

3 2 5 2 1 6 2 3 6 9 3 2 0 1

2 1 8 1 0 2 4 2 5 3 2 5 4 1

7 4 2 1 7 4 2 8 5 1 1 8 1 2

5 2 1 4 1 8 1 5 1 2 2 0 0 0

0 2 7 0 3 2 1 5 0 2 3 0 1 4

4

OTU4298 1 0 0 3 1 0 0 0 0 0 14 8 21

39 1 0 0 1 4 12 0 0 0 0 0 1 2

0 0 3 0 0 2 0 13 22 1 0 1 0 11

0 2 0 0 0 1 12 3 0 0 3 0 0 4

0 2 2 1 0 0 0 0 0 0 0 1 0 0

0 0 0 2 0 1 6 0 15 1 3 0 1 3

28 0 6 0 34 4 2 2 12 3 12 3 0 2

5 4 0 0 0 1 0 0 0 0 9 3 0 0

0

OTU12206 2 0 0 0 0 0 0 0 0 0 0 0 0

0 0 0 0 0 0 0 0 0 0 2 0 0 0

0 0 0 1 0 0 0 0 0 0 0 0 0 1

1 1 2 1 1 0 0 0 3 0 1 0 0 0

0 0 0 0 0 0 0 0 0 0 1 0 0 0

0 0 0 0 0 1 0 0 0 0 0 0 0 0

0 0 0 0 0 0 0 0 0 1 0 0 0 0

0 0 0 0 0 0 0 0 0 0 0 0 0 0

0

OTU1159 2 0 0 0 0 0 0 0 0 0 0 0 0

0 0 0 0 0 0 0 0 0 0 0 0 0 0

0 0 0 0 0 0 0 0 0 0 0 0 0 0

0 0 0 0 0 0 0 0 0 0 0 0 0 0

0 0 0 0 0 0 0 0 0 0 0 0 0 0

0 0 0 0 0 0 0 0 0 0 0 0 0 0

0	0	0	0	0	0	0	0	0	0	0	0	0	0
0	0	0	0	0	0	0	0	0	0	0	0	0	0
0													
OTU391	2	5	5	1	3	1	7	7	2	0	2	1	14
0	1	7	1	10	0	4	23	0	31	0	1	0	13
1	0	0	5	0	2	0	0	0	38	1	1	9	2
1	6	0	2	0	0	12	0	0	0	9	1	0	0
0	2	0	11	0	0	0	0	0	0	0	1	3	1
0	1	2	5	1	5	8	0	1	1	38	6	3	2
4	1	0	2	1	16	1	2	10	2	0	0	0	7
2	2	2	1	9	2	1	4	1	2	3	1	0	4
0													
OTU13507	1	0	0	0	0	0	0	0	9	0	0	1	1
0	1	0	0	0	0	0	0	3	0	0	0	4	0
0	2	0	2	0	0	0	0	0	0	0	0	0	0
0	0	7	0	18	1	3	0	0	1	13	0	1	0
0	0	1	6	3	1	0	21	2	3	1	8	0	0
0	0	3	0	0	0	0	2	2	1	0	1	0	0
1	16	1	0	0	0	0	5	0	0	0	0	0	0
0	0	3	0	1	2	0	5	2	0	0	0	0	1
1													
OTU445	1	12	4	0	3	1	4	2	0	1	0	0	0
0	1	1	0	0	0	1	0	0	0	0	0	0	0
0	0	0	0	0	0	0	0	1	0	2	0	0	0
1	0	1	0	0	0	0	0	0	0	0	0	0	0
0	0	0	0	0	0	0	0	0	0	0	0	1	0
0	0	9	0	1	0	0	2	0	2	0	1	1	0
0	1	0	5	0	0	0	0	0	0	0	0	2	0
0	0	0	3	0	0	1	0	1	5	0	0	0	0
0													
OTU208	4	77	2	5	4	12	21	3	17	0	1	0	0
0	9	0	0	0	2	5	0	0	0	0	10	6	16
0	1	0	0	1	2	0	0	2	3	1	4	2	0

4	0	0	0	3	8	2	17	0	0	6	8	0	5
0	3	0	2	0	0	0	1	1	0	0	1	24	0
3	3	3	0	15	13	0	1	0	6	11	10	0	3
0	1	0	24	0	13	1	9	0	0	0	2	3	0
5	4	3	3	4	4	8	10	3	5	2	0	0	11
6													
OTU146	2	61	2	2	30	6	7	12	19	13	5	2	9
16	4	0	0	2	39	13	14	0	0	8	40	7	11
0	0	0	0	0	0	10	0	2	9	0	2	8	22
23	80	0	7	0	0	0	2	0	0	2	2	1	0
0	12	2	3	0	1	2	0	1	2	0	2	3	1
0	1	28	5	2	36	0	5	11	15	3	3	20	5
38	0	2	16	55	3	9	6	28	0	0	6	1	3
5	3	3	0	2	0	2	2	4	2	18	0	3	3
1													
OTU14297	1	0	0	0	0	0	0	0	0	0	0	0	0
0	0	0	0	0	0	0	0	0	0	0	0	0	0
0	0	0	0	0	0	0	0	0	0	0	0	0	0
0	0	0	0	0	0	0	0	0	0	0	0	0	0
0	0	0	0	0	0	0	0	0	0	0	0	0	0
0	0	0	0	0	0	0	0	0	0	0	0	0	0
0	0	0	0	0	0	0	0	0	0	0	0	0	0
0	0	0	0	0	0	0	0	0	0	0	0	0	0
0													
OTU8918	2	2	2	2	2	1	2	4	4	7	1	5	1
4	18	1	2	6	1	0	3	3	4	0	1	5	2
0	1	7	3	0	0	0	5	2	1	2	2	4	0
7	2	5	4	2	3	3	0	2	11	1	3	2	2
5	0	4	5	2	2	0	2	4	5	5	0	4	2
3	6	5	1	2	3	0	10	2	2	2	5	0	2
6	2	4	0	3	7	2	2	0	4	6	0	1	0
1	6	1	0	3	0	1	4	0	0	1	2	1	2
15													

OTU3655 1 0 0 0 0 0 0 0 0 0 0 0 0
0 0 0 0 0 0 0 0 0 0 0 0 0 1
1 0 0 0 0 0 0 0 0 0 0 0 0 0
0 0 0 0 0 0 0 0 0 0 0 0 0 0
0 0 0 0 0 0 0 0 0 0 0 0 0 0
0 0 0 0 0 0 0 0 0 0 0 0 0 0
0 0 0 0 0 0 0 0 0 0 0 0 0 0
0 0 0 0 0 0 0 0 0 0 0 0 0 0
0
OTU151 1 26 3 18 1 1 10 4 3 0 2 20 11
18 19 38 3 6 5 4 45 2 19 5 42 3 4
3 3 15 26 9 22 2 27 4 156 8 7 5 6
0 7 2 110 4 5 1 1 3 3 3 1 1 0
1 4 7 2 1 1 2 0 1 2 3 2 7 1
4 0 4 8 1 45 2 5 8 3 5 4 11 2
25 64 12 7 19 7 16 6 0 11 30 2 5 30
30 0 2 0 0 0 0 0 3 0 26 11 181 0
1
OTU10970 1 0 0 0 0 0 0 0 0 0 0 0 0
0 0 0 0 0 0 0 0 0 0 0 0 0 0
0 0 0 0 0 0 0 0 0 0 0 0 0 0
0 0 0 0 0 0 0 0 0 0 0 0 0 0
0 0 0 0 0 0 0 0 0 0 0 0 0 0
0 0 0 0 0 0 0 0 0 0 0 0 0 0
0 0 0 0 0 0 0 0 0 0 0 0 0 0
0 0 0 0 0 0 0 0 0 0 0 0 0 0
0
OTU8500 1 0 0 0 0 0 0 0 0 0 0 0 0
0 0 0 0 0 0 0 0 0 0 0 0 0 0
0 0 0 0 0 0 0 0 0 0 0 0 0 0
0 0 0 0 0 0 0 0 0 0 0 0 0 0
0 0 0 0 0 0 0 0 0 0 0 0 0 0
0 0 0 0 0 0 0 0 0 0 0 0 0 0

0	0	0	0	0	0	0	0	0	0	0	0	0	0
0	0	0	0	0	0	0	0	0	0	0	0	0	0
0													
OTU2559	4	3	0	0	0	1	1	0	0	1	0	0	0
0	7	0	0	0	0	3	0	0	0	0	0	3	0
0	0	0	0	0	0	0	0	1	0	4	0	7	0
0	0	0	0	0	4	0	6	0	0	0	0	0	0
0	1	0	1	0	0	0	0	0	0	0	0	2	0
1	0	7	0	0	0	0	0	0	0	0	1	0	0
1	2	0	0	0	3	0	10	0	0	0	0	0	0
0	0	0	1	0	0	1	0	2	0	0	0	0	0
0													
OTU10968	1	0	0	0	0	0	0	0	0	0	0	0	0
0	0	0	0	0	0	0	0	0	0	0	0	0	0
0	0	0	0	0	0	0	0	0	0	0	0	0	0
0	0	0	0	0	0	0	0	0	0	0	0	0	0
0	0	0	0	0	0	0	0	0	0	0	0	0	0
0	0	0	0	0	0	0	0	0	0	0	0	0	0
0	0	0	0	0	0	0	0	0	0	0	0	0	0
0	0	0	0	0	0	0	0	0	0	0	0	0	0
0													
OTU8501	1	0	0	0	0	0	0	0	0	0	0	0	0
0	0	0	0	0	0	0	0	0	0	0	0	0	0
0	0	0	0	0	0	0	0	0	0	0	0	0	0
0	0	0	0	0	0	0	0	0	0	0	0	0	0
0	0	0	0	0	0	0	0	0	0	0	0	0	0
0	0	0	0	0	0	0	0	0	0	0	0	0	0
0	0	0	0	0	0	0	0	0	0	0	0	0	0
0	0	0	0	0	0	0	0	0	0	0	0	0	0
0													
OTU1701	2	6	0	8	5	4	0	1	0	4	0	2	0
0	6	3	4	17	2	0	8	0	5	0	27	4	27
0	0	26	21	0	10	0	3	1	6	1	10	0	5

	0	1	0	12	0	0	2	0	4	0	0	0	1	1
	0	1	0	0	0	0	0	1	0	0	0	0	0	0
	0	0	2	2	0	31	32	1	4	2	64	0	17	0
	41	1	0	0	17	2	2	0	0	0	0	0	0	5
	0	0	0	1	0	0	0	0	0	0	0	2	0	0
	0													
OTU49	6	1	3	0	0	0	2	2	7	1	0	0	1	0
	0	3	303	0	0	11	101	16	0	31	0	28	0	35
	884	0	0	296	15	335	5	5	0	330	0	4	0	3
	0	10	10	342	2	1	2	3	50	334	36	16	1	109
	0	151	1	66	120	1	23	9	226	14	3	6	40	234
	0	4	18	5	0	5	47	3	9	36	38	2	1	18
	23	5	1	24	6	192	1	0	0	0	2	0	17	0
	0	13	8	4	4	20	0	8	8	0	0	0	3	9
OTU1184		1	8	0	155	90	80	1	31	0	9	0	9	0
	11	119	0	0	0	0	1	0	0	0	0	5	29	0
	2	0	0	0	1	0	0	0	30	7	0	0	3	0
	0	13	0	9	1	21	0	4	0	0	0	1	1	95
	0	36	0	50	0	0	0	0	0	0	0	0	14	0
	2	1	146	2	0	6	0	0	3	10	2	0	0	0
	0	3	0	11	33	15	0	5	0	0	12	0	6	0
	0	1	0	1	2	0	0	0	1	0	2	1	0	0
	0													
OTU860		4	0	0	0	0	0	0	0	0	0	0	0	0
	0	0	0	0	0	0	0	0	0	0	0	0	0	0
	0	0	0	0	0	0	0	0	0	0	0	0	0	0
	0	0	0	0	0	0	0	0	0	0	0	0	0	0
	0	0	0	0	0	4	0	0	0	0	0	0	0	0
	0	0	0	0	0	0	0	0	0	0	0	1	0	0
	0	0	0	5	0	0	0	0	0	0	0	0	0	0
	0	0	0	0	0	0	0	0	0	0	0	0	0	0
	0													

OTU8499	1	0	0	0	0	0	0	0	0	0	0	0	0
0	0	0	0	0	0	0	0	0	0	0	0	0	0
0	0	0	0	0	0	0	0	0	0	0	0	0	0
0	0	0	0	0	0	0	0	0	0	0	0	0	0
0	0	0	0	0	0	0	0	0	0	0	0	0	0
0	0	0	0	0	0	0	0	0	0	0	0	0	0
0	0	0	0	0	0	0	0	0	0	0	0	0	0
0	0	0	0	0	0	0	0	0	0	0	0	0	0
0													
OTU4963	2	0	1	0	0	1	4	5	2	0	1	0	0
0	10	0	2	15	2	0	1	3	26	10	6	0	0
0	8	0	0	2	10	0	0	20	1	2	11	0	0
0	0	4	5	0	0	19	0	1	2	0	0	3	2
1	5	11	0	1	0	4	1	2	5	4	1	0	4
0	0	0	3	0	5	13	0	0	0	22	0	3	1
1	3	5	0	1	2	2	0	2	1	0	0	0	0
0	0	0	0	0	0	0	0	0	0	1	1	2	0
0													
OTU1341	3	1	2	2	6	9	4	2	6	5	0	22	2
4	11	1	0	1	0	15	1	0	0	1	0	1	0
0	0	0	0	0	0	9	5	6	1	0	0	8	5
7	3	0	0	0	0	0	0	0	0	0	0	0	0
0	0	0	3	0	0	0	0	0	0	0	0	0	0
0	0	5	3	5	8	4	0	19	5	0	8	3	0
3	0	1	0	5	4	9	2	1	3	1	0	0	0
2	0	0	0	0	0	0	0	0	0	2	3	0	0
0													
OTU233	2	2	2	2	8	3	0	4	2	14	1	7	0
0	3	1	0	2	0	9	3	0	0	0	1	7	2
2	0	0	0	0	0	0	0	0	11	0	0	1	0
6	1	0	0	0	7	0	0	0	0	0	1	0	0
0	93	0	9	0	0	0	0	0	0	0	0	73	0
1	0	0	2	0	0	0	2	0	8	1	1	2	17

1	1	1	1	1	5	1	0	0	1	1	0	3	0
0	3	0	0	0	0	0	0	0	0	2	0	1	0
0													
OTU10971	1	0	0	0	0	0	0	0	0	0	0	0	0
0	0	0	0	0	0	0	0	0	0	0	0	0	0
0	0	0	0	0	0	0	0	0	0	0	0	0	0
0	0	0	0	0	0	0	0	0	0	0	0	0	0
0	0	0	0	0	0	0	0	0	0	0	0	0	0
0	0	0	0	0	0	0	0	0	0	0	0	0	0
0	0	0	0	0	0	0	0	0	0	0	0	0	0
0	0	0	0	0	0	0	0	0	0	0	0	0	0
0													
OTU10355	1	0	0	0	1	0	0	1	0	0	0	0	0
0	0	0	1	1	0	0	0	1	1	0	0	0	1
0	1	1	0	1	0	0	1	0	0	1	1	0	0
0	1	0	0	0	0	0	0	1	1	2	0	1	0
0	0	0	0	1	0	0	0	0	1	0	0	0	0
2	0	1	0	0	0	0	0	0	0	0	2	0	0
0	1	0	0	0	0	0	0	0	0	0	0	1	0
0	0	0	0	1	0	0	1	0	0	1	0	0	0
0													
OTU6760	1	1	1	0	0	0	0	0	0	0	0	0	0
0	0	0	0	0	0	0	0	0	0	0	0	0	0
0	0	0	0	0	0	0	0	0	0	0	0	0	0
1	0	0	0	0	0	0	0	0	0	0	0	0	0
0	0	0	0	0	0	0	0	0	0	0	0	0	0
0	0	0	0	0	0	0	0	0	0	0	0	0	0
0	0	0	3	0	0	0	0	0	0	0	0	0	0
0	0	0	0	0	0	0	0	0	0	0	0	0	0
0													
OTU262	1	0	0	7	4	1	0	0	0	1	17	0	3
4	1	11	2	1	7	1	11	2	0	0	2	1	0
0	0	25	3	0	2	0	6	2	1	0	0	0	5

3	25	1	1	0	0	1	0	1	0	1	2	1	2
1	2	1	12	0	0	2	1	0	0	0	1	7	0
0	0	0	6	2	0	0	1	1	0	0	0	0	1
1	1	2	2	0	5	1	5	25	0	10	0	0	14
5	0	0	0	0	0	0	0	0	0	1	2	1	0
0													
OTU14280	1	0	0	0	0	0	0	0	0	0	0	0	0
0	0	0	0	0	0	0	0	0	0	0	0	0	0
0	0	0	0	0	0	0	0	0	0	0	0	0	0
0	0	0	0	0	0	0	0	0	0	0	0	0	0
0	0	0	0	0	0	0	0	0	0	0	0	0	0
0	0	0	0	0	0	0	0	0	0	0	0	0	0
0	0	0	0	0	0	0	0	0	0	0	0	0	0
0	0	0	0	0	0	0	0	0	0	0	0	0	0
0													
OTU12800	1	0	0	2	0	0	1	0	0	0	1	0	0
0	0	0	0	0	0	0	0	0	0	0	0	0	0
0	0	0	0	0	0	0	0	0	1	0	0	0	0
0	0	0	0	0	0	0	2	0	0	0	0	0	1
0	2	0	0	0	0	0	0	0	0	0	0	0	0
0	0	0	0	0	0	0	0	0	0	0	0	1	0
0	0	0	1	0	1	0	0	1	0	0	0	0	0
0	0	0	0	0	0	0	0	0	0	0	0	0	1
0													
OTU8496	1	0	0	0	0	0	0	0	0	0	0	0	0
0	0	0	0	0	0	0	0	0	0	0	0	0	0
0	0	0	0	0	0	0	0	0	0	0	0	0	0
0	0	0	0	0	0	0	0	0	0	0	0	0	0
0	0	0	0	0	0	0	0	0	0	0	0	0	0
0	0	0	0	0	0	0	0	0	0	0	0	0	0
0	0	0	0	0	0	0	0	0	0	0	0	0	0
0	0	0	0	0	0	0	0	0	0	0	0	0	0
0													

OTU8679	1	1	2	0	0	0	0	0	0	0	0	0	0
0	0	0	0	0	0	0	0	0	0	0	0	0	0
0	0	0	0	0	0	0	0	0	0	0	1	0	0
0	0	0	0	0	0	0	0	0	0	0	0	0	0
0	0	0	0	0	0	0	0	0	0	0	0	0	0
1	0	0	0	0	0	0	0	0	0	0	0	0	0
0	0	0	0	0	0	0	0	0	0	0	0	0	0
0	0	0	0	0	0	0	0	0	0	0	0	0	0
0													
OTU1188	2	2	1	1	0	2	0	1	0	0	0	4	1
1	1	0	0	0	0	2	0	0	0	0	1	1	0
0	0	0	0	0	0	0	4	3	0	0	0	0	1
3	2	0	0	0	0	0	0	0	0	1	0	0	0
0	0	0	4	0	0	0	0	0	0	0	0	0	0
0	0	1	0	3	1	0	0	4	3	0	0	0	0
0	0	1	1	0	2	1	3	0	0	0	0	0	0
1	0	0	0	0	0	0	0	0	0	1	1	1	0
0													
OTU8495	1	0	0	0	0	0	0	0	0	0	0	0	0
0	0	0	0	0	0	0	0	0	0	0	0	0	0
0	0	0	0	0	0	0	0	0	0	0	0	0	0
0	0	0	0	0	0	0	0	0	0	0	0	0	0
0	0	0	0	0	0	0	0	0	0	0	0	0	0
0	0	0	0	0	0	0	0	0	0	0	0	0	0
0	0	0	0	0	0	0	0	0	0	0	0	0	0
0	0	0	0	0	0	0	0	0	0	0	0	0	0
0													
OTU106	3	0	0	0	0	0	0	0	1	0	0	0	0
0	0	0	26	0	0	2	0	156	0	25	0	31	2
5	80	0	0	18	0	35	5	0	0	3	0	43	2
1	0	67	0	32	1	0	0	9	45	64	4	21	0
61	1	37	2	44	32	18	40	78	21	131	36	1	23
116	0	0	0	3	0	0	60	1	1	0	1	0	0

0	46	0	0	0	0	12	8	1	0	0	0	0	0
0	0	9	1	1	3	2	1	4	3	0	1	2	8
2													
OTU2134	1	0	0	1	0	0	0	0	1	2	1	0	0
0	0	0	0	1	0	0	0	0	0	0	1	0	0
0	0	0	0	0	0	0	0	0	0	1	0	0	0
1	1	0	1	0	0	0	0	0	2	0	1	0	0
0	0	1	0	0	0	0	1	1	0	0	0	0	0
1	0	1	0	2	0	0	0	0	1	1	0	0	0
1	0	0	1	0	1	0	1	0	0	0	0	0	0
0	0	0	0	1	0	0	0	0	0	0	0	0	0
2													
OTU6776	1	0	0	0	0	0	0	1	0	0	0	0	0
0	0	0	0	0	0	0	0	0	0	0	0	0	0
0	0	0	0	0	0	0	0	0	0	0	0	0	0
0	0	0	0	0	0	0	0	0	0	0	0	0	0
0	0	0	0	0	0	0	0	0	0	0	0	0	0
0	0	0	0	0	0	0	0	0	0	0	0	0	0
0	0	0	0	1	0	0	0	0	0	0	0	0	0
0	0	0	0	0	0	0	0	0	0	0	0	0	0
0													
OTU9223	1	0	0	0	0	0	0	0	0	0	1	2	0
0	0	0	0	0	0	1	0	0	0	0	0	1	0
0	0	0	0	0	0	0	0	1	0	0	0	0	0
0	0	0	0	0	0	0	0	0	0	0	0	0	0
0	1	0	0	0	0	0	0	0	0	0	0	0	0
0	0	0	0	0	0	0	0	0	0	0	0	0	0
0	0	0	0	0	0	0	0	0	0	1	0	0	0
0	0	0	0	0	0	0	0	0	0	0	0	0	0
0													
OTU1157	2	8	1	15	2	4	1	2	1	2	4	9	21
27	3	34	62	83	19	1	16	24	3	38	4	10	3
0	48	27	33	41	22	6	15	4	20	3	20	2	22

0 1 10 24 3 1 6 0 7 13 3 5 5 2
14 4 19 0 17 14 4 6 12 11 13 6 3 7
12 0 0 3 0 34 72 6 43 0 80 3 6 2
2 8 20 1 12 2 5 2 13 2 8 0 0 12
17 0 0 0 0 0 0 0 0 0 12 4 48 0
0
OTU3460 1 0 1 0 0 0 1 0 1 1 0 0 0
0 2 0 0 0 0 1 0 0 0 0 0 0 0
0 0 0 1 0 0 0 0 0 0 0 0 0 0
0 0 0 0 1 0 1 0 0 1 0 0 0 0
0 0 0 0 0 0 0 0 0 0 0 0 0 0
1 0 0 1 0 0 0 0 0 0 0 0 1 0
0 0 0 1 0 0 0 0 0 0 0 1 0 0
0 0 1 0 0 2 0 0 0 2 0 0 0 3
2
OTU3058 1 2 1 2 1 1 0 0 1 0 0 2 2
1 0 3 0 1 0 0 1 0 0 0 0 0 0
0 0 0 2 0 0 0 2 0 12 0 0 2 0
3 2 0 3 0 1 0 0 0 0 0 0 0 0
0 0 0 0 0 0 0 0 0 0 0 0 0 0
0 1 1 3 0 0 2 0 0 0 0 0 17 0
0 0 0 1 2 0 1 0 0 33 1 0 0 0
3 0 0 1 0 0 0 1 0 0 6 0 7 0
0
OTU5796 1 0 0 0 0 0 0 0 0 0 0 0 0
0 0 0 0 0 0 0 0 0 0 0 0 0 0
0 0 0 0 0 0 0 0 0 0 0 0 0 0
0 0 0 0 0 0 0 0 0 0 0 0 1 0
0 0 0 0 0 0 0 0 0 1 0 0 0 0
0 0 0 0 0 0 0 0 0 0 0 0 0 0
0 0 0 0 0 0 0 0 0 0 0 0 0 0
0 0 0 0 0 0 0 0 0 0 0 0 0 0
0

OTU3089 1 0 0 0 0 0 0 0 0 0 1 0 0
0 0 0 0 0 0 0 0 0 0 0 0 0 0
0 0 0 0 0 0 0 0 0 0 0 0 0 0
0 0 0 0 0 0 0 0 0 0 0 0 0 0
0 0 0 0 0 0 0 0 0 0 0 0 0 0
0 0 0 0 0 0 0 0 0 0 0 1 0 0
0 0 0 0 0 0 0 0 0 0 0 0 0 0
0 0 0 0 0 0 0 0 0 0 0 0 0 0
0
OTU12440 1 0 2 0 0 0 5 0 2 2 0 0 0
0 0 0 0 0 0 0 0 0 0 0 0 0 0
0 0 0 0 0 0 0 0 0 0 0 0 0 0
3 0 0 0 0 0 0 0 0 0 0 0 0 0
0 0 0 0 0 0 0 0 0 0 0 0 0 0
0 0 0 0 2 0 0 0 0 2 0 0 0 2
0 0 0 1 0 0 0 0 0 0 0 0 0 0
0 0 0 0 0 0 0 0 0 0 0 0 0 0
0
OTU3097 1 0 0 0 1 0 0 0 0 2 0 0 0
1 0 1 0 1 1 0 0 0 0 0 0 1 0
0 0 0 0 0 0 0 1 0 1 2 1 3 0
0 0 0 0 0 0 1 0 1 2 0 1 0 1
0 0 0 0 0 1 0 0 0 1 0 1 0 0
0 0 0 0 0 0 0 2 0 0 0 3 0 0
0 0 1 0 1 1 0 1 0 0 2 2 0 0
0 1 0 0 0 0 1 0 0 1 0 0 0 1
5
OTU14279 1 0 0 0 0 0 0 0 0 0 0 0 0
0 0 0 0 0 0 0 0 0 0 0 0 0 0
0 0 0 0 0 0 0 0 0 0 0 0 0 0
0 0 0 0 0 0 0 0 0 0 0 0 0 0
0 0 0 0 0 0 0 0 0 0 0 0 0 0
0 0 0 0 0 0 0 0 0 0 0 0 0 0

0	0	0	0	0	0	0	0	0	0	0	0	0	0
0	0	0	0	0	0	0	0	0	0	0	0	0	0
0													
OTU6886	1	0	0	0	0	0	0	0	0	0	0	2	0
0	0	0	0	0	0	1	0	0	0	0	0	0	0
0	0	0	0	0	0	0	0	0	0	0	0	0	0
0	0	0	0	0	1	0	4	0	0	0	0	0	0
0	3	0	2	0	1	0	0	0	0	0	0	1	0
1	1	0	0	0	0	0	0	0	3	0	0	0	0
0	0	0	0	0	2	0	2	0	0	0	0	0	0
0	0	1	1	0	0	0	0	1	0	0	0	0	1
0													
OTU12210	1	0	0	0	0	0	0	0	0	0	0	0	0
0	0	0	0	0	0	0	0	0	0	0	0	0	0
0	0	0	0	0	0	0	0	0	0	0	0	0	0
0	0	0	0	0	0	0	0	0	0	0	0	0	0
0	0	0	0	0	0	0	0	0	0	0	0	0	0
0	0	0	0	0	0	0	0	0	0	0	0	0	0
0	0	0	0	0	0	0	0	0	0	0	0	0	0
0	0	0	0	0	0	0	0	0	0	0	0	0	0
0													
OTU12209	1	0	0	0	0	0	0	0	0	0	0	0	0
0	0	0	0	0	0	0	0	0	0	0	0	0	0
0	0	0	0	0	0	0	0	0	0	0	0	0	0
0	0	0	0	0	0	0	0	0	0	0	0	0	0
0	0	0	0	0	0	0	0	0	0	0	0	0	0
0	0	0	0	0	0	0	0	0	0	0	0	0	0
0	0	0	0	0	0	0	0	0	0	0	0	0	0
0	0	0	0	0	0	0	0	0	0	0	0	0	0
0													
OTU5166	1	0	0	0	0	0	0	0	0	0	0	0	0
0	0	1	0	2	0	0	0	0	0	0	1	0	0
1	0	0	2	0	1	0	0	0	0	0	6	0	0

0	0	0	0	0	1	0	0	0	0	0	0	0	0
0	0	0	0	0	0	0	0	0	0	0	0	0	0
0	0	0	0	0	0	0	0	0	0	0	0	0	0
0	1	0	0	0	0	0	0	0	0	0	0	0	0
0	0	0	0	0	0	0	0	0	0	0	0	0	0
0													
OTU266	2	0	1	0	5	7	18	3	20	52	0	4	3
0	0	0	0	0	3	8	1	1	0	0	0	1	1
31	0	0	0	0	0	0	0	2	0	0	0	0	0
40	14	0	2	0	13	0	13	0	2	0	22	0	0
0	39	0	3	0	2	0	4	3	0	0	0	88	0
1	18	5	0	42	5	1	10	0	85	4	24	10	21
0	10	0	39	2	56	0	29	0	1	1	20	25	0
0	8	13	29	14	1	8	24	7	26	0	0	0	14
38													
OTU1931	1	1	0	1	1	1	0	0	0	0	0	0	0
0	5	0	0	0	0	0	0	1	0	2	0	0	0
1	0	0	0	5	0	0	0	1	1	6	0	0	0
0	0	0	1	10	10	0	0	2	3	4	12	0	0
2	9	0	1	1	0	0	2	4	8	1	0	15	0
0	0	0	0	0	0	0	0	0	0	0	0	0	0
0	0	0	0	0	0	0	0	0	0	0	0	0	0
0	0	0	0	0	8	5	0	1	1	0	0	0	0
10													
OTU8497	1	0	0	0	0	0	0	0	0	0	0	0	0
0	0	0	0	0	0	0	0	0	0	0	0	0	0
0	0	0	0	0	0	0	0	0	0	0	0	0	0
0	0	0	0	0	0	0	0	0	0	0	0	0	0
0	0	0	0	0	0	0	0	0	0	0	0	0	0
0	0	0	0	0	0	0	0	0	0	0	0	0	0
0	0	0	0	0	0	0	0	0	0	0	0	0	0
0	0	0	0	0	0	0	0	0	0	0	0	0	0
0													

OTU13676	5	0	1	0	0	1	0	2	6	3	0	0	0
0	0	0	0	3	0	0	0	0	0	0	0	0	1
0	0	0	0	0	2	0	0	0	10	0	0	0	2
0	1	0	0	1	0	0	0	0	0	0	0	0	0
0	2	0	1	0	0	0	1	0	0	0	0	0	0
0	0	2	0	0	0	0	0	0	0	0	2	0	0
0	2	0	0	0	1	0	3	0	0	0	1	2	0
0	0	0	0	0	1	0	0	0	0	0	1	0	0
0													
OTU994	2	1	2	0	0	2	0	2	2	2	0	0	0
0	0	0	0	0	0	0	0	0	0	0	0	1	0
0	0	0	0	0	0	0	0	0	0	0	1	0	0
0	0	0	0	0	0	0	0	0	0	0	2	0	0
0	0	0	0	0	0	0	0	0	0	0	0	0	0
0	0	0	0	0	0	0	1	0	0	0	0	2	0
0	0	0	0	0	1	0	0	0	2	0	1	1	0
0	1	0	0	1	0	0	0	0	0	0	0	0	0
0													
OTU756	1	0	1	0	0	0	1	0	2	2	0	0	0
1	0	0	0	0	0	0	6	0	0	0	0	0	0
0	0	0	0	0	0	0	0	0	4	0	0	0	0
0	1	0	2	0	0	0	0	0	0	0	0	0	0
0	0	0	1	1	0	0	0	0	0	0	0	0	0
0	2	0	0	0	0	0	0	2	1	0	0	0	0
0	0	2	0	0	2	0	1	0	0	0	2	2	0
2	0	3	2	0	2	1	1	1	0	0	1	0	0
0													
OTU15912	1	0	0	0	0	0	0	0	0	0	0	0	0
0	0	1	0	0	1	0	0	0	0	0	0	0	0
0	0	0	0	0	1	0	0	0	0	0	0	0	0
0	0	0	0	0	0	0	0	0	0	0	0	0	0
0	0	0	0	0	0	0	0	0	0	0	0	0	0
0	0	0	0	0	0	0	0	0	0	0	0	1	0

0	0	0	0	0	0	0	0	0	0	0	0	0	0
0	0	0	0	0	0	0	0	0	0	0	0	0	0
0													
OTU11108	2	1	3	2	5	0	1	3	5	3	4	3	0
2	10	3	1	5	2	1	2	2	4	1	4	1	4
1	2	5	3	0	5	0	2	3	0	2	3	4	1
4	3	2	3	1	0	0	1	3	6	3	1	1	1
7	2	2	5	3	3	1	2	4	1	2	2	3	1
3	5	1	0	9	0	2	6	0	4	1	9	3	3
7	2	0	3	1	4	0	6	1	1	1	2	1	1
1	4	3	1	3	5	1	4	3	4	3	2	1	4
17													
OTU12208	1	0	0	0	0	0	0	0	0	0	0	0	0
0	0	0	0	0	0	0	0	0	0	0	0	0	0
0	0	0	0	0	0	0	0	0	0	0	0	0	0
0	0	0	0	0	0	0	0	0	0	0	0	0	0
0	0	0	0	0	0	0	0	0	0	0	0	0	0
0	0	0	0	0	0	0	0	0	0	0	0	0	0
0	0	0	0	0	0	0	0	0	0	0	0	0	0
0	0	0	0	0	0	0	0	0	0	0	0	0	0
0													
OTU11479	1	0	0	0	0	0	0	0	0	0	0	0	0
0	1	0	0	0	0	0	0	0	0	0	0	0	0
0	0	0	0	1	0	0	0	0	0	0	0	0	0
0	0	1	1	0	1	0	0	0	1	0	0	0	0
1	0	1	0	0	0	0	0	1	1	0	0	1	0
0	1	0	0	2	0	0	1	0	0	0	1	0	0
0	0	1	0	0	0	0	0	1	0	0	0	0	0
1	1	0	0	0	0	0	0	0	0	2	0	0	0
2													
OTU16484	2	0	0	0	0	0	0	0	0	0	1	0	0
0	0	1	0	0	0	0	0	0	1	0	1	0	0
0	0	2	0	0	0	0	0	0	0	0	1	0	0

0	0	0	0	0	0	0	0	0	0	0	0	0	0
0	0	0	0	0	0	0	0	0	0	0	0	0	0
0	0	0	1	0	0	0	0	0	0	1	0	0	0
0	0	0	0	0	0	0	0	0	0	0	0	0	0
0	0	0	0	0	0	0	0	0	0	0	1	0	0
0													
OTU7955	3	1	3	3	1	0	0	1	8	1	0	4	0
0	6	23	0	1	0	1	0	0	13	0	1	0	0
4	1	1	15	0	0	0	0	1	1	1	0	0	0
0	5	0	6	2	0	0	1	1	0	1	1	0	0
0	0	0	1	0	0	0	0	1	0	1	0	2	0
0	2	0	9	11	46	18	2	1	0	1	1	46	13
9	3	31	3	14	2	3	0	0	3	1	0	0	1
0	2	2	1	3	0	1	1	2	8	1	0	0	0
0													
OTU8704	1	0	0	0	1	2	1	3	2	2	1	1	0
3	1	3	0	0	1	0	2	0	0	0	0	0	3
0	0	3	1	0	2	0	0	0	1	0	2	1	0
2	0	1	2	0	0	0	0	3	3	0	0	1	0
0	0	0	0	0	1	1	0	2	1	1	1	0	0
1	0	0	1	0	0	0	2	0	0	0	2	0	0
1	1	0	0	1	2	0	0	0	0	1	0	0	1
0	0	1	1	1	1	0	1	0	0	1	1	0	0
2													
OTU1385	3	0	2	0	5	4	2	5	2	1	2	0	1
0	0	0	0	0	0	0	1	0	2	0	1	0	0
0	0	0	1	0	0	0	0	0	1	0	0	0	0
0	0	0	0	0	0	0	0	0	0	0	0	0	0
0	0	0	0	0	0	0	1	0	0	0	0	0	0
0	0	4	0	4	0	0	1	0	18	0	0	0	1
0	0	0	1	0	0	0	1	0	0	0	0	0	0
0	0	2	0	0	0	0	0	0	0	0	0	0	0
0													

OTU8345	1	0	1	1	2	1	0	2	1	1	1	2	0
0	3	0	1	1	0	0	0	0	0	1	0	1	0
0	1	1	0	1	0	1	1	0	0	0	1	1	2
1	0	1	3	0	0	0	1	0	5	0	0	0	1
0	0	1	0	0	2	0	2	0	0	1	2	0	0
2	0	1	0	0	1	0	3	0	1	1	1	0	1
1	1	0	0	2	2	2	0	1	0	0	0	1	1
2	1	1	0	2	1	0	2	0	0	0	0	0	0
6													
OTU287	1	1	0	3	0	0	0	1	2	0	0	2	0
0	0	0	0	0	0	2	0	0	0	0	0	1	0
0	0	0	0	0	0	0	0	0	0	6	0	1	0
1	0	0	0	0	0	0	0	0	0	0	0	0	0
0	0	0	0	0	0	1	0	0	0	0	0	2	0
0	11	3	0	0	0	0	1	0	0	1	1	0	1
0	0	0	2	0	0	0	0	0	0	0	4	0	0
0	36	5	27	29	49	7	2	9	40	0	1	0	39
1													
OTU14281	1	0	0	0	0	0	0	0	0	0	0	0	0
0	0	0	0	0	0	0	0	0	0	0	0	0	0
0	0	0	0	0	0	0	0	0	0	0	0	0	0
0	0	0	0	0	0	0	0	0	0	0	0	0	0
0	0	0	0	0	0	0	0	0	0	0	0	0	0
0	0	0	0	0	0	0	0	0	0	0	0	0	0
0	0	0	0	0	0	0	0	0	0	0	0	0	0
0	0	0	0	0	0	0	0	0	0	0	0	0	0
0													
OTU280	1	19	1	0	0	0	0	0	4	3	0	4	0
1	0	0	2	1	0	11	2	0	8	0	31	1	0
7	0	0	8	3	0	0	0	0	53	0	0	0	0
26	71	1	30	0	1	0	4	6	1	0	5	0	0
0	0	0	1	0	0	0	0	2	0	0	0	9	0
0	0	1	0	0	4	1	6	0	3	0	27	3	3

1	0	2	17	0	0	0	1	0	0	0	1	8	0
0	37	0	4	2	20	10	7	2	0	0	0	0	1
0													
OTU6238	2	0	2	0	3	24	0	0	2	1	0	4	0
0	3	0	0	0	0	0	0	0	0	0	0	0	0
5	0	0	0	0	0	0	0	0	0	0	0	2	0
0	0	0	0	0	0	0	9	0	0	0	0	0	4
0	3	0	1	0	0	0	0	0	0	0	0	0	0
0	0	6	0	3	0	0	0	0	5	0	0	0	2
0	0	0	2	0	6	0	10	0	0	0	0	0	0
0	0	0	0	0	0	0	0	0	0	0	0	0	0
0													
OTU7273	1	3	0	0	0	0	0	0	0	0	0	0	0
0	0	0	0	0	0	0	0	0	0	0	0	0	0
0	0	0	0	0	0	0	0	0	0	0	0	0	0
0	0	0	0	3	0	0	0	0	0	4	0	0	0
0	0	0	0	0	0	0	0	1	0	0	0	0	0
0	0	0	0	1	0	0	0	0	0	0	0	0	0
0	1	0	0	0	0	0	0	0	0	0	0	0	0
0	0	0	0	0	0	0	0	0	0	0	0	0	0
0													
OTU466	3	0	3	5	0	0	0	3	3	0	0	0	0
0	0	0	0	0	0	0	0	0	0	0	8	0	0
1	0	0	0	0	4	0	0	1	2	0	0	0	0
4	0	0	0	0	0	0	0	5	3	1	5	0	0
0	1	0	1	0	6	0	1	8	0	1	3	1	0
0	7	1	1	0	17	0	0	1	1	6	4	0	1
3	0	3	1	4	0	0	0	0	0	0	0	2	0
0	3	14	0	11	26	16	5	5	0	0	1	0	0
0													
OTU1269	1	0	0	0	0	0	0	0	0	0	0	0	1
0	0	0	0	0	0	0	0	0	0	0	0	0	0
0	0	0	0	0	2	0	0	0	0	0	0	0	0

1 1 0 5 0 0 0 0 0 0 0 0 0 0
0 0 0 0 0 0 0 0 0 0 0 0 0 0
0 0 0 0 0 3 1 0 0 0 0 0 0 0
0 0 0 0 2 1 0 0 0 0 0 0 0 0
0 0 0 0 0 0 0 0 0 0 1 0 1 0
0
OTU5857 1 0 0 0 0 0 1 1 0 0 0 0 0
0 0 0 1 0 1 0 0 0 0 0 0 1 4
0 0 0 1 1 0 2 0 0 0 0 0 1 0
0 0 0 0 1 0 0 0 0 0 0 0 0 2
0 0 0 3 0 0 1 0 0 0 0 1 0 0
0 0 0 0 0 0 0 0 0 0 1 0 0 0
0 0 0 1 0 0 0 0 0 0 0 0 0 0
0 0 0 0 0 0 0 0 0 0 0 0 0 0
0
OTU16130 2 3 1 0 2 2 1 3 4 1 1 3 0
3 10 0 1 4 0 1 0 1 0 0 0 0 0
1 0 2 1 0 0 2 2 0 2 5 1 3 0
3 3 2 2 1 0 1 1 1 2 1 0 2 0
2 2 2 0 0 0 0 0 1 3 2 2 1 0
6 2 2 0 1 0 0 1 3 0 0 2 0 1
4 2 0 1 2 3 0 0 0 0 0 0 0 0
0 1 1 2 0 0 0 0 1 0 0 0 0 0
9
OTU7520 2 1 2 4 1 6 0 8 2 0 0 0 0
0 1 0 0 0 0 0 1 0 0 0 1 7 4
0 0 0 3 5 0 1 0 1 0 0 0 0 0
0 0 0 1 0 8 0 0 0 0 0 0 0 0
0 1 0 3 1 0 0 0 0 0 0 3 0 0
0 0 0 0 0 0 0 0 2 0 1 0 0 3
0 0 0 0 1 1 6 0 0 0 0 0 0 0
3 1 0 0 0 0 0 0 0 0 1 1 0 0
0

OTU2132	1	0	0	0	0	0	0	0	0	1	0	0	
0	0	0	0	1	0	0	0	0	0	0	0	0	0
1	0	1	0	0	0	0	0	0	0	0	0	0	0
0	0	1	1	1	0	0	0	0	0	0	0	0	0
0	0	0	0	0	1	0	0	0	1	0	0	0	0
1	0	0	0	0	1	0	0	0	0	0	0	0	0
0	0	0	0	0	0	0	0	0	0	0	0	0	0
0	0	0	0	0	0	0	0	0	1	0	0	0	0
1													
OTU10977	1	0	0	0	0	0	0	0	0	0	0	0	
0	0	0	0	0	0	0	0	0	0	0	0	0	0
0	0	0	0	0	0	0	0	0	0	0	0	0	0
0	0	0	0	0	0	0	0	0	0	0	0	0	0
0	0	0	0	0	0	0	0	0	0	0	0	0	0
0	0	0	0	0	0	0	0	0	0	0	0	0	0
0	0	0	0	0	0	0	0	0	0	0	0	0	0
0	0	0	0	0	0	0	0	0	0	0	0	0	0
0													
OTU11744	1	4	0	1	1	2	0	10	0	0	1	0	1
0	0	0	0	4	1	0	5	0	9	0	9	0	12
0	0	0	3	3	18	0	0	0	5	0	6	0	3
0	0	0	0	1	1	1	0	0	0	0	0	0	0
0	0	0	1	0	0	0	2	0	0	2	0	0	0
18	0	0	3	1	2	1	0	0	0	0	0	0	0
1	1	0	0	0	0	0	0	0	1	0	0	0	1
0	0	0	0	0	0	0	0	0	0	1	2	0	0
0													
OTU3941	1	0	0	0	0	1	0	0	0	0	0	0	0
0	2	1	0	0	1	0	0	1	1	1	1	0	1
0	0	1	0	1	0	0	1	0	0	0	1	2	0
2	1	1	0	0	0	0	1	1	1	1	0	0	0
2	0	2	0	1	0	0	0	0	1	0	0	0	0
1	0	1	0	0	0	0	1	1	0	0	0	0	1

0	0	0	0	0	2	0	0	1	1	0	0	2	0
0	2	0	1	1	0	1	0	0	0	0	0	0	0
2													
OTU12299	3	8	26	7	6	2	30	10	11	6	0	4	0
0	3	0	0	0	0	3	1	0	0	0	0	0	1
1	0	0	3	0	0	0	0	4	0	0	0	1	0
1	1	0	26	0	1	0	2	0	0	0	2	0	1
0	0	0	3	0	1	0	0	1	0	0	0	2	0
0	0	0	0	42	0	1	1	0	9	0	1	2	7
0	1	0	10	0	1	0	3	0	3	0	0	0	0
0	0	2	1	0	0	0	0	0	0	1	0	1	0
0													
OTU10975	1	0	0	0	0	0	0	0	0	0	0	0	0
0	0	0	0	0	0	0	0	0	0	0	0	0	0
0	0	0	0	0	0	0	0	0	0	0	0	0	0
0	0	0	0	0	0	0	0	0	0	0	0	0	0
0	0	0	0	0	0	0	0	0	0	0	0	0	0
0	0	0	0	0	0	0	0	0	0	0	0	0	0
0	0	0	0	0	0	0	0	0	0	0	0	0	0
0	0	0	0	0	0	0	0	0	0	0	0	0	0
0													
OTU276	1	8	7	0	1	0	4	1	16	1	0	0	0
0	1	0	0	0	0	2	0	0	0	0	0	4	0
0	0	0	0	0	0	0	0	1	0	0	0	0	0
5	0	0	1	0	0	0	0	0	0	0	0	0	0
0	0	0	1	0	0	0	0	0	0	0	0	1	0
0	19	1	0	14	0	0	2	0	5	0	2	0	7
5	3	0	14	0	0	0	2	0	0	0	14	3	0
0	10	26	9	14	26	11	13	5	36	0	0	0	48
6													
OTU5681	1	2	0	1	0	0	1	3	0	1	0	1	0
1	1	0	0	2	0	0	0	1	0	0	1	0	0
0	1	0	1	0	1	0	0	0	1	0	0	0	0

0 0 0 0 0 2 0 0 1 0 0 0 0 0
0 0 0 0 0 0 0 0 1 0 0 0 0 0
0 0 0 1 0 0 2 2 0 0 0 1 0 1
0 0 0 0 0 1 0 0 0 0 2 0 0 0
0 0 0 0 0 0 0 0 0 0 0 0 0 0
3
OTU1168 1 0 0 0 0 0 0 1 0 0 0 0 0
0 0 0 0 0 0 0 1 0 0 0 0 0 0
0 0 0 1 0 0 0 0 0 1 0 0 0 0
0 0 0 0 0 0 0 0 0 0 0 0 0 0
0 2 0 0 0 0 0 0 0 0 0 0 0 0
0 0 0 0 0 0 0 0 0 0 0 0 0 0
0 0 0 0 0 0 0 0 0 0 0 0 0 0
0 0 0 0 0 0 0 0 0 0 0 0 0 0
0
OTU8517 1 0 0 0 0 0 0 0 0 0 0 0 0
0 0 0 0 0 0 0 0 0 0 0 0 0 0
0 0 0 0 0 0 0 0 0 0 0 0 0 0
0 0 0 0 0 0 0 0 0 0 0 0 0 0
0 0 0 0 0 0 0 0 0 0 0 0 0 0
0 0 0 0 0 0 0 0 0 0 0 0 0 0
0 0 0 0 0 0 0 0 0 0 0 0 0 0
0 0 0 0 0 0 0 0 0 0 0 0 0 0
0
OTU382 3 0 1 0 0 0 0 0 1 0 0 0 0
0 0 0 5 0 0 0 0 16 0 0 0 0 0
0 0 0 0 1 0 48 1 4 0 0 0 2 0
1 0 0 0 1 2 0 6 0 2 8 1 1 0
0 0 3 2 62 3 12 1 2 17 0 0 0 18
1 0 0 0 0 0 0 1 0 0 0 0 0 0
0 1 0 0 0 0 0 0 0 0 0 0 0 0
0 0 0 0 0 18 1 0 1 1 0 2 10 0
0

OTU360 3 0 0 4 0 1 0 1 2 0 0 2 0
1 0 1 0 0 0 2 16 0 0 0 1 12 2
1 0 0 0 0 6 0 1 0 0 0 0 0 0
0 1 0 0 0 2 0 0 0 0 0 0 0 1
0 8 0 1 0 0 0 0 0 0 0 0 0 0
0 1 0 2 0 0 0 0 1 0 0 0 4 4
1 1 5 1 3 3 2 4 0 1 1 0 0 1
5 1 0 1 0 2 1 0 0 0 0 0 0 0
0
OTU14276 1 0 0 0 0 0 0 0 0 0 0 0 0
0 0 0 0 0 0 0 0 0 0 0 0 0 0
0 0 0 0 0 0 0 0 0 0 0 0 0 0
0 0 0 0 0 0 0 0 0 0 0 0 0 0
0 0 0 0 0 0 0 0 0 0 0 0 0 0
0 0 0 0 0 0 0 0 0 0 0 0 0 0
0 0 0 0 0 0 0 0 0 0 0 0 0 0
0 0 0 0 0 0 0 0 0 0 0 0 0 0
0
OTU1141 1 0 0 0 0 0 1 0 0 3 0 0 0
0 0 0 0 0 0 2 0 0 0 0 0 0 0
2 0 0 0 0 0 0 0 0 0 0 0 0 0
0 0 0 0 0 0 0 1 0 0 0 0 0 0
0 1 0 0 0 0 0 0 0 0 0 0 0 0
0 0 0 0 0 0 0 0 0 2 0 0 0 1
0 0 0 0 0 2 0 0 0 0 0 0 0 0
0 0 0 0 0 0 0 0 0 0 0 0 0 0
0
OTU8519 1 0 0 0 0 0 0 0 0 0 0 0 0
0 0 0 0 0 0 0 0 0 0 0 0 0 0
0 0 0 0 0 0 0 0 0 0 0 0 0 0
0 0 0 0 0 0 0 0 0 0 0 0 0 0
0 0 0 0 0 0 0 0 0 0 0 0 0 0
0 0 0 0 0 0 0 0 0 0 0 0 0 0

0	0	0	0	0	0	0	0	0	0	0	0	0	0
0	0	0	0	0	0	0	0	0	0	0	0	0	0
0													
OTU14275	1	0	0	0	0	0	0	0	0	0	0	0	0
0	0	0	0	0	0	0	0	0	0	0	0	0	0
0	0	0	0	0	0	0	0	0	0	0	0	0	0
0	0	0	0	0	0	0	0	0	0	0	0	0	0
0	0	0	0	0	0	0	0	0	0	0	0	0	0
0	0	0	0	0	0	0	0	0	0	0	0	0	0
0	0	0	0	0	0	0	0	0	0	0	0	0	0
0	0	0	0	0	0	0	0	0	0	0	0	0	0
0													
OTU14278	1	0	0	0	0	0	0	0	0	0	0	0	0
0	0	0	0	0	0	0	0	0	0	0	0	0	0
0	0	0	0	0	0	0	0	0	0	0	0	0	0
0	0	0	0	0	0	0	0	0	0	0	0	0	0
0	0	0	0	0	0	0	0	0	0	0	0	0	0
0	0	0	0	0	0	0	0	0	0	0	0	0	0
0	0	0	0	0	0	0	0	0	0	0	0	0	0
0	0	0	0	0	0	0	0	0	0	0	0	0	0
0													
OTU10974	1	0	0	0	0	0	0	0	0	0	0	0	0
0	0	0	0	0	0	0	0	0	0	0	0	0	0
0	0	0	0	0	0	0	0	0	0	0	0	0	0
0	0	0	0	0	0	0	0	0	0	0	0	0	0
0	0	0	0	0	0	0	0	0	0	0	0	0	0
0	0	0	0	0	0	0	0	0	0	0	0	0	0
0	0	0	0	0	0	0	0	0	0	0	0	0	0
0	0	0	0	0	0	0	0	0	0	0	0	0	0
0													
OTU14277	1	0	0	0	0	0	0	0	0	0	0	0	0
0	0	0	0	0	0	0	0	0	0	0	0	0	0
0	0	0	0	0	0	0	0	0	0	0	0	0	0

	0	0	0	0	0	0	0	0	0	0	0	0	0	0
	0	0	0	0	0	0	0	0	0	0	0	0	0	0
	0	0	0	0	0	0	0	0	0	0	0	0	0	0
	0	0	0	0	0	0	0	0	0	0	0	0	0	0
	0	0	0	0	0	0	0	0	0	0	0	0	0	0
	0													
OTU60	2	1	1	2	1	29	3	119	72	381	13	164	471	86
	2	0	2	0	492	140	317	4	0	636	1	292	2	19
	1	0	0	1	4	176	0	4	169	10	1	196	451	51
	582	1	116	1	0	1	0	4	0	115	4	0	8	0
	7	0	114	0	2	0	0	0	0	1	295	1	0	0
	0	53	5	0	0	0	2	1	11	0	22	6	12	2
	2	0	348	3	3	0	2	48	15	36	1	5	326	46
	8	4	5	11	1	4	1	0	10	117	2	3	1	6
OTU8910		1	7	3	1	0	1	0	1	0	1	2	0	0
	0	0	0	0	2	0	0	3	0	1	0	1	0	4
	0	1	1	0	0	0	1	0	0	0	0	0	0	1
	0	0	7	0	11	0	0	0	7	1	11	0	1	0
	0	0	0	2	0	5	0	4	9	0	5	0	1	0
	3	0	0	0	1	0	0	16	0	0	0	17	0	3
	0	7	0	0	1	0	0	0	0	0	0	0	0	0
	0	0	0	0	0	0	0	0	0	0	0	0	0	0
	0													
OTU5805		1	4	1	0	1	1	0	0	2	0	0	0	0
	0	0	0	3	0	0	1	0	2	0	0	1	2	0
	0	6	0	0	2	0	0	0	0	0	0	0	0	0
	0	0	4	0	12	0	0	0	8	1	3	0	4	0
	0	0	0	0	2	23	2	21	11	3	5	0	1	2
	10	0	0	0	4	1	0	14	0	1	0	5	0	0
	0	20	0	0	0	0	2	1	0	0	0	0	0	0
	0	0	0	0	0	3	0	0	0	0	0	0	0	0
	0													

OTU8518	1	0	0	0	0	0	0	0	0	0	0	0	0
0	0	0	0	0	0	0	0	0	0	0	0	0	0
0	0	0	0	0	0	0	0	0	0	0	0	0	0
0	0	0	0	0	0	0	0	0	0	0	0	0	0
0	0	0	0	0	0	0	0	0	0	0	0	0	0
0	0	0	0	0	0	0	0	0	0	0	0	0	0
0	0	0	0	0	0	0	0	0	0	0	0	0	0
0	0	0	0	0	0	0	0	0	0	0	0	0	0
0													
OTU258	2	3	0	4	0	70	1	50	15	2	0	0	0
0	1	0	0	0	0	53	0	8	0	0	1	12	0
1	0	0	0	1	0	1	0	1	0	0	0	1	0
0	0	0	0	0	1	0	5	1	0	0	3	0	0
0	3	0	3	0	4	1	1	4	0	1	0	50	0
0	2	39	0	16	0	0	0	0	0	0	3	1	50
0	0	0	2	0	6	52	68	0	0	0	2	0	0
0	1	0	2	4	4	2	7	0	1	0	0	0	0
2													
OTU16643	1	0	0	0	0	0	1	0	1	0	0	0	0
0	0	0	0	0	0	0	0	0	0	0	0	5	0
1	0	0	0	0	0	0	0	0	0	0	0	1	0
0	0	0	0	0	2	0	1	0	0	0	0	0	0
0	0	0	0	0	0	0	0	0	0	0	0	3	0
0	0	0	0	0	0	0	0	0	0	0	0	0	1
0	0	0	2	0	0	0	0	0	0	0	0	0	0
0	0	0	0	0	0	0	0	0	0	0	0	0	0
0													
OTU3098	5	4	6	2	5	6	2	6	5	9	0	4	0
4	6	3	0	3	5	0	2	2	2	1	1	2	0
0	2	2	2	3	4	0	0	0	4	2	3	4	2
3	4	5	2	3	1	0	0	4	5	0	0	1	1
1	1	1	2	0	3	4	3	2	1	3	3	2	1
4	1	2	1	7	1	1	7	2	2	1	10	0	3

1	1	1	2	1	6	1	1	3	0	3	2	1	0
0	1	1	1	2	2	0	0	0	2	2	1	0	0
10													
OTU10738	1	0	0	0	0	0	0	0	2	1	0	0	0
0	0	1	0	0	0	0	0	0	0	0	2	0	0
0	0	0	0	0	0	0	0	0	4	0	0	0	1
0	0	0	0	1	0	0	0	0	0	2	0	0	0
0	0	0	0	0	0	0	1	0	0	0	0	0	0
0	0	0	0	1	0	0	0	0	0	0	0	0	0
0	0	0	1	0	0	0	0	0	0	0	0	0	0
0	0	0	0	0	2	0	1	0	1	0	0	0	0
1													
OTU1394	1	0	0	0	0	0	0	0	0	0	0	0	0
0	0	0	0	0	0	0	0	0	0	0	0	0	0
0	0	0	0	0	0	0	0	0	0	0	0	0	0
0	0	0	0	0	0	0	0	0	0	0	0	0	0
0	0	0	0	0	0	0	0	0	0	0	0	0	0
0	0	2	0	0	0	0	0	0	0	0	0	0	0
0	0	0	0	0	0	0	0	0	0	0	0	0	0
0	0	0	0	0	0	0	0	0	0	0	0	0	0
0													
OTU15553	1	0	0	0	0	0	0	0	0	0	0	0	0
0	0	0	0	0	0	0	0	0	0	0	0	0	0
0	0	0	1	0	0	0	0	0	0	0	0	0	0
0	0	0	0	0	0	0	0	0	0	0	0	0	0
0	0	0	0	0	0	0	0	0	0	0	0	0	0
0	0	0	0	0	0	0	0	0	0	0	0	0	0
0	0	0	0	0	0	0	0	0	0	0	0	0	0
0	0	0	0	0	0	0	0	0	0	0	0	0	0
0													
OTU13737	1	0	0	0	0	0	1	1	2	3	0	0	0
0	0	0	0	0	0	0	0	0	0	0	0	0	0
0	0	0	0	0	0	0	0	0	0	0	0	0	0

0 0 0 0 0 0 0 0 0 0 0 0 0 0
0 0 0 0 0 0 0 0 0 0 0 0 0 0
0 0 0 0 0 0 0 0 0 0 0 3 0 0
0 0 0 4 0 0 0 0 0 0 0 0 3 0
0 0 1 0 0 0 0 0 0 3 0 0 0 0
0
OTU9704 2 14 15 3 0 11 0 0 2 3 0 0 0
0 0 1 0 0 0 0 13 0 0 0 0 0 3
0 0 1 8 0 4 0 0 0 3 0 1 0 1
0 0 18 0 35 0 0 0 22 0 37 1 0 0
0 0 0 4 0 27 0 14 32 0 8 0 2 0
14 0 1 0 3 0 0 22 0 4 0 69 0 14
0 32 0 0 0 0 1 0 0 0 0 0 0 0
1 0 1 0 0 0 0 0 0 0 0 0 0 0
0
OTU10976 1 0 0 0 0 0 0 0 0 0 0 0 0
0 0 0 0 0 0 0 0 0 0 0 0 0 0
0 0 0 0 0 0 0 0 0 0 0 0 0 0
0 0 0 0 0 0 0 0 0 0 0 0 0 0
0 0 0 0 0 0 0 0 0 0 0 0 0 0
0 0 0 0 0 0 0 0 0 0 0 0 0 0
0 0 0 0 0 0 0 0 0 0 0 0 0 0
0 0 0 0 0 0 0 0 0 0 0 0 0 0
0
OTU8522 1 0 0 0 0 0 0 0 0 0 0 0 0
0 0 0 0 0 0 0 0 0 0 0 0 0 0
0 0 0 0 0 0 0 0 0 0 0 0 0 0
0 0 0 0 0 0 0 0 0 0 0 0 0 0
0 0 0 0 0 0 0 0 0 0 0 0 0 0
0 0 0 0 0 0 0 0 0 0 0 0 0 0
0 0 0 0 0 0 0 0 0 0 0 0 0 0
0 0 0 0 0 0 0 0 0 0 0 0 0 0
0

OTU998	1	0	1	1	0	2	0	0	0	0	0	2	0
0	1	0	0	0	0	0	0	0	0	0	0	0	0
0	0	0	0	0	0	0	0	1	0	1	0	0	0
0	0	0	0	0	0	0	0	0	0	0	0	0	0
0	1	0	0	0	0	0	0	0	0	0	0	0	0
0	1	0	0	0	1	0	0	0	0	0	0	0	0
0	0	0	0	0	0	0	0	0	0	0	0	0	0
0	0	0	0	0	0	0	0	0	1	0	0	0	0
0													
OTU12241	1	0	0	0	0	0	0	0	0	0	0	0	0
0	0	0	0	0	0	0	0	0	0	0	0	0	0
0	0	0	0	0	0	0	0	0	0	0	0	0	0
0	0	0	0	0	0	0	0	0	0	0	0	0	0
0	0	0	0	0	0	0	0	0	0	0	0	0	0
0	0	0	0	0	0	0	0	0	0	0	0	0	0
0	0	0	0	0	0	0	0	0	0	0	0	0	0
0	0	0	0	0	0	0	0	0	0	0	0	0	0
0													
OTU132	1	0	1	1	2	1	55	1	1	12	0	3	0
0	0	27	0	1	0	15	0	13	0	0	0	0	2
2	27	0	0	0	0	1	9	0	2	0	0	0	0
15	41	7	11	0	2	0	28	1	33	4	10	9	8
0	7	0	33	0	3	21	1	34	2	0	0	0	1
5	5	24	6	17	22	0	1	3	27	1	0	33	13
5	5	0	1	34	60	3	11	0	1	5	11	3	4
0	27	4	0	17	0	1	5	0	0	11	8	0	0
0													
OTU8523	1	0	0	0	0	0	0	0	0	0	0	0	0
0	0	0	0	0	0	0	0	0	0	0	0	0	0
0	0	0	0	0	0	0	0	0	0	0	0	0	0
0	0	0	0	0	0	0	0	0	0	0	0	0	0
0	0	0	0	0	0	0	0	0	0	0	0	0	0
0	0	0	0	0	0	0	0	0	0	0	0	0	0

0	0	0	0	0	0	0	0	0	0	0	0	0	0
0	0	0	0	0	0	0	0	0	0	0	0	0	0
0													
OTU8526	1	0	0	0	0	0	0	0	0	0	0	0	0
0	0	0	0	0	0	0	0	0	0	0	0	0	0
0	0	0	0	0	0	0	0	0	0	0	0	0	0
0	0	0	0	0	0	0	0	0	0	0	0	0	0
0	0	0	0	0	0	0	0	0	0	0	0	0	0
0	0	0	0	0	0	0	0	0	0	0	0	0	0
0	0	0	0	0	0	0	0	0	0	0	0	0	0
0	0	0	0	0	0	0	0	0	0	0	0	0	0
0													
OTU1076	1	0	0	1	1	4	1	1	0	0	0	0	0
0	0	0	0	0	0	0	0	0	0	0	0	1	4
1	0	0	0	0	0	0	0	1	0	0	0	0	1
1	0	0	0	0	1	0	0	0	0	1	0	0	1
0	0	0	2	0	0	0	0	0	0	0	1	0	0
0	0	0	0	1	0	0	0	0	0	1	0	0	0
0	0	0	0	0	0	1	0	0	0	0	0	0	0
0	0	0	0	0	0	0	0	0	0	0	0	0	0
0													
OTU169	1	0	1	2	0	0	1	0	3	1	3	1	3
6	0	31	3	1	5	7	35	4	2	0	0	1	0
2	3	6	14	0	3	0	20	2	51	8	3	4	2
1	14	1	35	6	5	0	1	8	6	5	5	10	1
1	1	2	0	4	2	17	1	1	3	2	2	3	3
7	0	0	6	4	29	3	0	4	0	1	1	1	0
0	19	11	0	3	0	22	4	1	31	22	0	1	27
9	0	1	0	0	0	0	0	4	1	16	48	42	0
0													
OTU1329	1	7	0	0	0	0	2	2	0	0	0	0	0
0	0	0	0	0	0	0	0	0	0	0	0	0	0
1	0	0	0	0	0	0	0	0	0	0	0	1	0

5	0	0	0	0	1	0	1	0	0	0	0	0	0
0	0	0	0	0	0	0	0	0	0	0	0	0	0
0	0	0	0	3	0	0	0	0	4	0	0	0	5
0	0	0	1	0	3	0	0	0	0	0	0	0	0
0	0	0	0	0	0	0	0	0	0	0	0	0	0
0													
OTU10377	1	0	0	2	1	2	0	1	1	0	0	0	0
3	0	1	0	2	0	1	1	0	4	0	1	2	1
1	0	1	0	1	3	0	0	0	0	0	1	3	1
0	0	1	1	0	0	0	1	2	4	0	1	0	0
0	1	0	0	1	2	0	0	1	1	1	0	0	2
1	1	0	1	2	0	0	0	1	0	0	3	1	1
1	0	1	1	0	3	0	0	0	1	2	0	0	1
0	0	0	0	2	0	0	1	0	0	1	0	0	0
2													
OTU8524	1	0	0	0	0	0	0	0	0	0	0	0	0
0	0	0	0	0	0	0	0	0	0	0	0	0	0
0	0	0	0	0	0	0	0	0	0	0	0	0	0
0	0	0	0	0	0	0	0	0	0	0	0	0	0
0	0	0	0	0	0	0	0	0	0	0	0	0	0
0	0	0	0	0	0	0	0	0	0	0	0	0	0
0	0	0	0	0	0	0	0	0	0	0	0	0	0
0	0	0	0	0	0	0	0	0	0	0	0	0	0
0													
OTU10833	1	1	2	0	0	0	1	0	0	0	0	1	0
0	0	0	0	2	0	1	0	0	0	0	0	0	0
1	0	0	0	0	0	2	0	0	0	0	0	2	0
1	0	2	3	0	0	0	0	0	2	0	0	0	0
0	1	0	2	1	1	0	0	0	0	0	0	1	0
1	0	1	2	1	2	1	2	1	2	0	2	0	2
1	0	0	0	0	3	1	0	0	0	2	0	0	1
0	0	1	0	0	0	1	3	0	0	0	0	0	0
2													

OTU13069	1	0	0	2	0	2	0	1	0	1	0	0	0
0	1	0	0	1	0	0	0	0	0	0	0	2	0
0	0	0	0	1	0	0	0	5	0	0	1	0	0
0	0	0	0	0	2	0	0	0	0	0	0	0	5
0	1	0	0	0	0	0	0	0	0	0	0	2	0
0	0	0	0	0	0	0	0	0	0	0	0	0	1
1	0	1	0	0	0	0	3	0	0	1	0	0	0
0	0	0	0	0	0	0	0	0	0	0	0	0	0
0													
OTU8525	1	0	1	0	0	0	0	1	0	0	0	0	0
0	0	0	0	0	0	0	0	0	0	0	0	0	0
0	0	0	0	0	0	0	0	0	0	0	0	0	0
0	0	0	0	0	0	0	0	0	0	0	0	0	0
0	0	0	0	0	0	0	0	0	0	0	0	0	0
0	0	0	0	0	0	0	0	0	0	0	0	0	0
0	0	0	0	0	0	0	0	0	0	0	0	0	0
0	0	0	0	0	0	0	0	0	0	0	0	0	0
0													
OTU13382	2	1	0	0	2	0	0	1	0	0	1	2	0
2	14	0	0	4	2	0	3	0	2	2	1	0	0
0	0	2	1	0	0	2	2	0	1	2	0	5	2
1	2	3	1	1	0	0	1	0	5	1	1	0	0
0	0	3	1	0	0	0	0	0	3	0	0	1	2
5	0	0	1	1	1	1	2	0	0	0	4	1	1
1	2	0	2	2	0	0	2	1	1	1	2	1	0
0	2	0	0	1	0	2	1	1	2	1	0	1	0
8													
OTU8405	3	18	3	1	5	12	0	1	0	0	0	2	0
0	1	0	0	0	0	1	2	0	0	0	1	0	0
0	0	3	6	0	1	0	4	1	2	2	0	0	3
0	0	0	2	0	0	3	0	1	0	5	0	0	0
0	1	0	2	0	0	1	0	1	0	1	0	0	1
1	0	0	3	0	0	2	0	1	1	2	0	3	1

1	0	4	0	3	0	3	0	3	1	2	0	0	0
8	0	0	0	0	0	0	0	0	0	1	7	6	0
0													
OTU8527	1	0	0	0	0	0	0	0	0	0	0	0	0
0	0	0	0	0	0	0	0	0	0	0	0	0	0
0	0	0	0	0	0	0	0	0	0	0	0	0	0
0	0	0	0	0	0	0	0	0	0	0	0	0	0
0	0	0	0	0	0	0	0	0	0	0	0	0	0
0	0	0	0	0	0	0	0	0	0	0	0	0	0
0	0	0	0	0	0	0	0	0	0	0	0	0	0
0	0	0	0	0	0	0	0	0	0	0	0	0	0
0													
OTU14518	4	0	0	0	4	2	3	6	3	0	0	0	0
0	2	0	0	0	1	0	0	0	0	0	0	1	3
0	0	0	0	0	0	4	0	4	0	0	0	2	1
1	0	0	1	1	2	0	1	0	0	0	0	0	0
0	0	0	4	0	0	1	0	0	0	0	2	1	0
0	0	0	3	7	4	0	0	0	2	1	0	0	0
0	0	0	1	0	1	8	0	0	0	0	0	0	0
1	0	0	0	0	0	0	0	0	0	0	0	0	0
0													
OTU15624	1	0	0	3	1	0	0	2	0	0	20	5	42
55	3	8	0	12	10	8	4	0	0	0	9	6	1
0	0	23	2	0	19	0	12	11	3	0	22	1	49
0	13	1	14	1	0	21	0	0	0	1	0	1	2
1	0	3	1	0	0	0	1	0	2	0	3	1	0
1	0	0	44	0	2	26	0	21	0	24	1	5	1
53	0	34	0	18	5	7	1	26	3	32	0	0	9
15	4	0	0	1	0	0	0	1	0	15	10	3	0
0													
OTU12320	1	0	1	0	0	0	0	0	0	0	0	0	0
0	0	0	0	0	0	0	0	0	0	0	0	0	0
0	0	0	0	0	0	0	0	0	0	0	0	0	0

0	0	0	0	0	0	0	0	0	0	0	0	0	0
0	0	0	0	0	0	0	0	0	0	0	0	0	0
0	0	0	0	0	0	0	0	0	0	0	0	0	0
0	0	0	0	0	0	0	0	0	0	0	0	0	0
0	0	0	0	0	0	0	0	0	0	0	0	0	0
0													
OTU406	1	0	4	7	0	2	0	4	3	3	0	1	0
0	1	0	0	0	0	1	0	0	0	0	0	0	1
2	0	0	0	0	0	0	0	1	0	0	0	1	0
0	0	0	0	0	1	0	0	0	0	0	0	0	3
0	6	0	0	0	0	0	0	0	0	0	0	0	0
0	0	1	0	4	0	0	0	0	3	0	0	0	0
0	1	0	2	0	1	0	2	0	17	0	0	1	0
0	0	1	0	0	0	0	2	0	2	0	1	2	0
1													
OTU14285	1	0	0	0	0	0	0	0	0	0	0	0	0
0	0	0	0	0	0	0	0	0	0	0	0	0	0
0	0	0	0	0	0	0	0	0	0	0	0	0	0
0	0	0	0	0	0	0	0	0	0	0	0	0	0
0	0	0	0	0	0	0	0	0	0	0	0	0	0
0	0	0	0	0	0	0	0	0	0	0	0	0	0
0	0	0	0	0	0	0	0	0	0	0	0	0	0
0	0	0	0	0	0	0	0	0	0	0	0	0	0
0													
OTU2477	1	0	0	0	4	2	0	3	0	0	0	0	0
0	0	0	0	0	0	0	0	0	0	0	0	0	0
0	0	0	0	0	0	0	5	3	0	0	0	0	0
0	0	0	0	0	0	2	0	0	0	0	0	0	0
0	0	0	1	1	0	0	0	0	0	1	0	0	1
0	0	8	0	0	0	0	0	1	0	1	0	0	0
0	1	0	0	0	0	0	1	0	0	1	0	0	4
1	0	0	0	0	0	0	0	0	2	0	0	22	1
0													

OTU5057	1	2	0	10	0	6	5	2	0	0	0	0	1
0	0	0	0	0	0	1	0	0	0	0	0	3	4
1	0	0	0	0	0	0	0	25	0	0	0	2	0
1	0	0	1	1	8	1	17	0	0	4	0	0	23
0	0	0	4	0	0	0	0	0	0	0	1	4	0
0	0	0	0	2	0	0	0	0	7	0	0	0	1
0	0	0	3	0	3	1	0	0	0	1	0	0	0
4	0	0	0	0	0	0	0	0	0	0	0	0	0
0													
OTU799	5	6	247	0	0	0	0	0	0	0	0	0	0
0	0	0	0	0	1	0	0	0	0	0	0	0	0
0	0	0	0	0	0	0	0	0	0	0	26	0	13
0	0	1	0	40	0	0	0	0	0	0	0	0	0
0	0	0	0	0	0	0	1	0	0	0	0	2	0
2	0	0	0	0	0	0	0	0	0	0	0	0	0
0	17	0	2	0	3	0	0	0	0	0	0	0	0
0	0	0	0	0	21	0	0	0	9	0	0	0	0
0													
OTU1648	1	0	0	0	0	1	0	0	0	0	0	0	0
0	0	0	0	0	0	0	0	0	0	0	0	0	0
0	0	0	0	0	0	0	0	0	1	0	0	0	0
0	0	0	0	0	0	0	0	0	0	0	0	0	0
0	0	0	0	0	0	0	0	0	0	0	0	0	0
0	0	0	0	0	0	0	0	0	2	0	0	0	0
0	0	0	0	0	0	0	0	0	0	0	0	0	0
0	0	0	0	0	0	0	0	0	0	0	0	0	0
0													
OTU1082	1	0	0	0	2	0	0	0	0	0	0	0	0
0	0	0	2	0	0	0	0	0	0	0	0	0	0
0	0	0	0	0	0	0	0	0	0	0	0	1	0
1	0	0	0	0	1	0	0	0	0	0	0	0	0
0	1	0	0	0	0	1	0	0	0	1	0	0	1
0	1	0	0	0	0	0	0	0	0	0	0	0	0

0 1 0 0 0 0 0 1 0 0 0 0 0 0
0 0 0 0 1 0 0 0 0 0 0 0 0 0
0
OTU12243 1 0 0 0 0 0 0 0 0 0 0 0 0
0 0 0 0 0 0 0 0 0 0 0 0 0 0
0 0 0 0 0 0 0 0 0 0 0 0 0 0
0 0 0 0 0 0 0 0 0 0 0 0 0 0
0 0 0 0 0 0 0 0 0 0 0 0 0 0
0 0 0 0 0 0 0 0 0 0 0 0 0 0
0 0 0 0 0 0 0 0 0 0 0 0 0 0
0 0 0 0 0 0 0 0 0 0 0 0 0 0
0
OTU2955 1 0 1 2 1 0 0 0 0 0 0 0 0
0 0 0 0 0 0 0 0 0 0 0 0 0 0
0 0 0 0 0 0 0 0 0 0 0 0 0 0
0 0 0 4 0 0 0 0 0 0 0 0 0 0
0 0 0 1 0 0 0 0 0 0 0 0 1 0
0 0 0 0 2 0 0 0 0 0 0 0 0 3
0 0 0 0 0 0 0 0 0 0 0 0 0 0
0 0 0 0 0 0 0 0 0 0 0 0 0 0
0
OTU9239 1 0 0 0 2 0 0 0 0 0 0 1 0
0 0 0 0 0 0 3 0 0 0 0 0 0 0
0 0 0 0 0 0 0 3 0 0 0 0 0 0
0 0 0 0 0 0 0 0 0 0 0 0 0 0
0 0 0 0 0 0 0 0 0 0 0 0 0 0
0 0 0 0 0 0 0 0 1 0 0 0 1 0
0 0 1 0 0 0 1 0 0 0 1 0 0 0
0 0 0 0 0 0 0 0 0 0 0 1 1 0
0
OTU10963 1 0 0 0 0 0 0 0 0 0 0 0 0
0 0 0 0 0 0 0 0 0 0 0 0 0 0
0 0 0 0 1 0 0 0 0 0 0 0 1 0

0 0 0 0 0 0 0 0 0 0 0 0 0 1
0 0 1 1 0 0 0 0 0 0 0 0 0 0
0 0 0 0 0 0 0 0 0 0 0 0 0 0
0 1 0 0 0 0 0 0 0 0 0 0 0 0
0 0 0 0 0 0 0 0 0 0 0 0 0 0
0
OTU14286 1 0 0 0 0 0 0 0 0 0 0 0 0
0 0 0 0 0 0 0 0 0 0 0 0 0 0
0 0 0 0 0 0 0 0 0 0 0 0 0 0
0 0 0 0 0 0 0 0 0 0 0 0 0 0
0 0 0 0 0 0 0 0 0 0 0 0 0 0
0 0 0 0 0 0 0 0 0 0 0 0 0 0
0 0 0 0 0 0 0 0 0 0 0 0 0 0
0 0 0 0 0 0 0 0 0 0 0 0 0 0
0
OTU12242 1 0 0 0 0 0 0 0 0 0 0 0 0
0 0 0 0 0 0 0 0 0 0 0 0 0 0
0 0 0 0 0 0 0 0 0 0 0 0 0 0
0 0 0 0 0 0 0 0 0 0 0 0 0 0
0 0 0 0 0 0 0 0 0 0 0 0 0 0
0 0 0 0 0 0 0 0 0 1 0 0 0 0
0 0 0 0 0 0 0 0 0 0 0 0 0 0
0 0 0 0 0 0 0 0 0 0 0 0 0 0
0
OTU8874 1 0 0 0 0 0 0 0 0 0 0 0 0
0 0 0 0 0 0 0 0 0 0 0 0 0 0
0 0 0 0 0 0 0 0 0 2 0 0 0 0
0 1 0 0 0 0 0 0 0 0 0 0 0 0
0 0 0 0 0 0 0 0 0 0 0 0 0 0
0 0 0 0 0 0 0 0 0 0 0 0 1 0
1 0 0 0 0 0 0 0 0 0 0 0 0 0
0 0 0 0 0 0 0 0 0 0 0 0 0 0
0

OTU11293	1	1	0	0	0	0	0	0	0	0	0	2	0
0	0	0	0	0	0	0	0	0	0	0	0	0	0
0	0	0	0	0	0	1	0	0	0	0	0	0	0
0	0	0	1	0	1	0	0	0	0	0	0	0	0
0	0	0	0	0	0	0	0	0	0	0	0	2	0
0	0	0	0	0	0	0	0	0	0	0	0	0	1
0	0	0	0	0	0	0	2	0	0	0	0	0	0
0	1	1	2	0	0	0	0	0	0	0	0	0	0
0													
OTU2489	1	0	0	0	0	0	0	0	0	0	0	0	0
0	0	0	0	0	1	0	0	0	0	0	0	0	0
0	1	0	0	0	1	0	0	0	0	0	0	0	0
0	0	0	0	0	0	0	0	0	0	0	0	0	0
0	0	0	0	0	0	0	0	0	0	0	0	0	0
0	0	0	0	0	0	0	1	0	0	0	0	0	0
0	0	0	0	0	0	0	0	0	0	0	0	0	0
0	0	1	0	0	1	0	0	0	0	0	0	0	0
0													
OTU14287	1	0	0	0	0	0	0	0	0	0	0	0	0
0	0	0	0	0	0	0	0	0	0	0	0	0	0
0	0	0	0	0	0	0	0	0	0	0	0	0	0
0	0	0	0	0	0	0	0	0	0	0	0	0	0
0	0	0	0	0	0	0	0	0	0	0	0	0	0
0	0	0	0	0	0	0	0	0	0	0	0	0	0
0	0	0	0	0	0	0	0	0	0	0	0	0	0
0	0	0	0	0	0	0	0	0	0	0	0	0	0
0													
OTU11828	1	0	0	0	0	0	0	1	0	0	1	0	0
0	0	0	0	0	1	0	0	0	2	0	2	0	0
0	0	1	0	0	0	0	0	1	0	0	1	0	0
0	0	0	0	0	0	0	0	0	0	0	0	0	0
0	0	0	0	0	0	0	0	0	0	0	0	0	0
0	0	0	1	0	0	0	0	0	0	0	0	0	0

0 0 0 0 0 0 0 0 0 0 0 0 0 0
0 0 0 0 0 0 0 0 0 0 0 0 0 0
0

OTU1490 2 2 8 1 4 3 8 5 5 10 2 8 1
13 4 1 0 1 7 0 15 0 0 0 1 5 4
2 0 6 11 0 4 1 3 3 26 2 1 1 3
13 11 0 14 0 3 0 1 0 0 0 2 0 0
0 0 0 13 0 0 0 0 0 0 0 0 7 0
0 3 53 21 35 16 11 2 2 1 18 3 14 9
0 1 3 20 8 15 1 5 1 7 3 1 0 3
2 3 1 8 1 1 0 6 1 3 3 1 1 7
8

OTU1352 1 1 0 3 2 0 0 0 0 0 0 2 0
20 0 0 6 0 0 5 0 1 0 0 0 21 0
1 0 0 0 0 0 11 4 1 0 1 0 0 3
0 0 0 0 26 30 0 33 0 0 0 1 0 0
0 1 0 9 2 0 0 0 4 1 1 25 4 0
4 0 9 11 0 0 0 0 0 1 4 0 0 1
38 0 0 0 0 0 0 3 0 0 5 0 0 1
8 0 0 0 0 0 0 0 0 1 0 0 5 2
0

OTU888 1 0 0 0 0 0 0 0 4 14 0 0 0
0 0 0 0 0 0 0 0 0 0 0 0 8 0
2 0 0 0 0 0 0 0 0 0 0 0 0 0
1 0 0 0 0 0 0 0 0 0 0 0 0 0
0 0 0 0 0 0 0 0 0 0 0 0 1 0
0 8 3 0 0 0 0 0 0 0 0 0 0 0
0 0 0 1 0 0 0 0 0 0 0 1 5 0
0 6 9 12 1 10 0 1 8 3 0 0 0 1
2

OTU448 1 12 2 0 0 0 1 0 0 5 0 0 0
0 1 0 0 0 0 1 0 0 0 0 3 0 0
0 0 0 0 0 0 0 0 0 0 0 0 0 0

0 2 0 1 0 0 0 0 0 0 0 0 0 0
0 0 0 2 0 0 0 0 0 0 0 0 0 0
0 1 0 1 0 1 1 1 0 2 4 0 0 0
0 0 0 0 0 0 0 0 0 0 0 0 4 0
0 0 0 1 1 9 1 2 8 3 0 0 0 0
0
OTU4410 1 0 0 0 0 0 0 1 0 0 0 2 0
0 0 0 0 0 1 1 0 0 0 0 0 0 0
0 0 0 0 0 0 0 0 0 0 0 0 0 1
0 0 0 0 0 0 0 0 0 0 0 0 0 0
0 0 0 1 0 0 0 0 0 0 0 0 0 0
0 0 0 0 3 0 0 0 1 0 0 1 0 0
0 1 0 0 0 0 0 0 0 0 0 0 0 0
0 0 0 0 0 0 0 0 0 0 0 0 0 0
0
OTU4544 1 0 0 0 1 0 0 1 0 0 0 0 0
0 1 1 0 1 4 0 1 0 4 0 0 0 0
0 0 0 0 0 1 0 3 0 0 0 4 0 9
0 0 0 3 0 0 0 0 0 0 0 0 0 0
0 0 0 0 0 0 0 0 0 0 0 0 0 0
0 0 0 0 0 1 2 0 1 0 0 0 3 0
1 0 4 0 0 1 0 0 0 0 0 0 0 0
0 0 0 0 0 0 0 0 0 0 0 3 0 0
0
OTU12239 1 0 0 0 0 0 0 0 0 0 0 0 0
0 0 0 0 0 0 0 0 0 0 0 0 0 0
0 0 0 0 0 0 0 0 0 0 0 0 0 0
0 0 0 0 0 0 0 0 0 0 0 0 0 0
0 0 0 0 0 0 0 0 0 0 0 0 0 0
0 0 0 0 0 0 0 0 0 0 0 0 0 0
0 0 0 0 0 0 0 0 0 0 0 0 0 0
0 0 0 0 0 0 0 0 0 0 0 0 0 0
0

OTU5169	3	0	1	0	0	0	0	0	0	0	0	0	0
0	0	0	0	0	0	0	0	0	0	0	0	0	0
0	0	0	0	0	0	0	0	0	0	0	1	0	1
0	0	0	0	0	0	0	0	0	0	0	0	0	0
0	0	0	0	0	0	0	0	0	0	0	0	0	0
0	0	0	0	0	0	0	0	0	0	0	0	0	0
0	0	0	0	0	0	0	0	0	0	0	0	0	0
0	0	0	0	0	0	0	0	0	0	0	0	0	0
0													
OTU293	1	0	3	0	0	0	1	2	1	0	0	1	0
0	1	1	0	0	0	8	12	0	0	2	0	0	0
3	0	0	5	1	0	1	0	0	14	0	0	0	0
1	5	0	1	0	0	0	0	0	0	3	4	1	0
0	0	1	0	5	0	3	2	1	4	0	0	0	2
0	0	1	0	2	7	10	0	3	0	1	8	13	1
0	0	1	1	1	0	2	0	0	0	0	0	4	0
0	4	0	0	10	3	1	2	0	1	2	0	0	0
0													
OTU10925	1	0	0	0	2	0	0	0	0	0	0	0	0
0	1	0	0	0	0	1	0	0	0	0	0	0	0
0	0	1	0	0	1	1	0	0	1	0	1	0	0
0	0	0	1	0	0	0	0	0	0	0	0	0	0
0	0	0	0	0	0	0	0	0	0	0	0	0	0
1	0	1	0	1	0	0	1	0	1	0	1	0	1
1	0	0	0	1	0	0	0	0	0	0	0	1	0
0	0	0	0	0	0	0	0	0	0	0	0	0	0
1													
OTU354	3	1	0	3	1	3	1	1	0	0	1	0	1
1	5	2	0	3	3	1	2	0	13	5	16	4	10
0	0	40	1	3	1	0	3	2	3	1	7	3	1
0	0	0	0	0	1	2	0	0	0	0	0	0	5
0	1	0	0	0	0	0	0	2	1	0	2	0	1
0	0	14	3	0	2	0	0	0	0	0	0	0	1

0	0	4	2	0	2	0	0	0	1	0	0	0	1
0	0	0	0	0	0	0	0	0	0	0	3	0	0
0													
OTU12683	2	0	0	0	0	0	0	0	0	0	0	0	0
0	0	0	0	0	0	1	0	1	0	0	0	0	0
0	0	0	0	0	0	0	0	0	0	1	0	0	0
1	0	0	0	1	4	0	1	0	0	0	0	0	0
1	0	1	1	0	0	0	2	0	2	0	0	2	0
0	0	0	0	0	0	0	0	0	0	0	0	0	0
0	0	0	0	0	0	0	0	0	0	1	0	0	0
0	0	0	0	1	0	0	0	0	0	1	0	0	0
0													
OTU6208	1	0	0	1	1	0	0	0	0	0	0	0	0
0	0	0	0	0	0	0	0	0	0	0	0	0	0
0	0	0	0	1	0	0	0	0	0	0	1	0	0
0	1	0	0	0	1	0	1	1	0	0	0	6	0
4	0	1	0	0	0	9	0	0	3	0	0	0	0
0	0	0	0	0	1	0	0	0	0	0	0	0	0
0	0	0	0	0	0	1	0	0	0	0	0	0	2
0	0	0	0	0	0	0	0	0	0	0	0	0	0
0													
OTU6874	1	0	0	0	0	0	0	0	0	0	0	0	0
0	0	0	0	0	0	0	0	0	0	0	0	0	0
0	0	0	0	0	0	0	0	0	0	0	0	0	0
0	0	0	0	0	0	0	0	0	0	0	0	0	0
0	0	0	0	0	0	0	0	0	0	0	0	0	0
0	0	0	0	0	0	0	0	0	0	0	0	0	0
0	0	0	0	0	1	0	0	0	0	0	0	0	0
0	0	0	0	1	0	0	0	0	0	0	0	0	0
0													
OTU10965	2	0	0	0	0	0	0	0	0	0	0	0	0
0	0	0	0	0	0	0	0	0	0	0	0	0	0
0	0	0	0	0	0	0	0	0	0	0	0	0	0

0 0 0 0 0 0 0 0 0 0 0 0 0 0

0 0 0 0 0 0 0 0 0 0 0 0 0 0

0 0 0 0 0 0 0 0 0 0 0 0 0 0

0 0 0 0 0 0 0 0 0 0 0 0 0 0

0 0 0 0 0 0 0 0 0 0 0 0 0 0

0

OTU10959 1 0 0 0 0 0 0 0 0 0 0 0 0

0 0 0 0 0 0 0 0 0 0 0 0 0 0

0 0 0 0 0 0 0 0 0 0 0 0 0 0

0 0 0 0 0 0 0 0 0 0 0 0 0 0

0 0 0 0 0 0 0 0 0 0 0 0 0 0

0 0 0 0 0 0 0 0 0 0 0 0 0 0

0 0 0 0 0 0 0 0 0 0 0 0 0 0

0 0 0 0 0 0 0 0 0 0 0 0 0 0

0

OTU10960 1 0 0 0 0 0 0 0 0 0 0 0 0

0 0 0 0 0 0 0 0 0 0 0 0 0 0

0 0 0 0 0 0 0 0 0 0 0 0 0 0

0 0 0 0 0 0 0 0 0 0 0 0 0 0

0 0 0 0 0 0 0 0 0 0 0 0 0 0

0 0 0 0 0 0 0 0 0 0 0 0 0 0

0 0 0 0 0 0 0 0 0 0 0 0 0 0

0 0 0 0 0 0 0 0 0 0 0 0 0 0

0

OTU14282 1 0 0 0 0 0 0 0 0 0 0 0 0

0 0 0 0 0 0 0 0 0 0 0 0 0 0

0 0 0 0 0 0 0 0 0 0 0 0 0 0

0 0 0 0 0 0 0 0 0 0 0 0 0 0

0 0 0 0 0 0 0 0 0 0 0 0 0 0

0 0 0 0 0 0 0 0 0 0 0 0 0 0

0 0 0 0 0 0 0 0 0 0 0 0 0 0

0 0 0 0 0 0 0 0 0 0 0 0 0 0

0

OTU12240 1 0 0 0 0 0 0 0 0 0 0 0 0
0 0 0 0 0 0 0 0 0 0 0 0 0 0
0 0 0 0 0 0 0 0 0 0 0 0 0 0
0 0 0 0 0 0 0 0 0 0 0 0 0 0
0 0 0 0 0 0 0 0 0 0 0 0 0 0
0 0 0 0 0 0 0 0 0 0 0 0 0 0
0 0 0 0 0 0 0 0 0 0 0 0 0 0
0 0 0 0 0 0 0 0 0 0 0 0 0 0
0
OTU10961 1 0 0 0 0 0 0 0 0 0 0 0 0
0 0 0 0 0 0 0 0 0 0 0 0 0 0
0 0 0 0 0 0 0 0 0 0 0 0 0 0
0 0 0 0 0 0 0 0 0 0 0 0 0 0
0 0 0 0 0 0 0 0 0 0 0 0 0 0
0 0 0 0 0 0 0 0 0 0 0 0 0 0
0 0 0 0 0 0 0 0 0 0 0 0 0 0
0 0 0 0 0 0 0 0 0 0 0 0 0 0
0
OTU12789 1 0 1 0 0 0 0 0 0 0 0 0 1
0 0 0 0 0 0 0 4 0 0 0 0 0 0
0 0 0 2 0 0 1 0 0 0 0 0 0 0
0 0 0 0 1 1 2 0 0 0 0 0 0 2
0 3 1 1 0 0 0 0 0 0 0 0 0 0
0 0 0 0 4 0 0 0 0 0 0 0 2 0
0 0 0 1 0 0 0 0 0 7 0 0 0 0
0 0 0 2 0 0 0 0 0 0 0 0 0 0
0
OTU36 2 16 279 3 23 49 72 105 14 74 23 289 30 0
41 80 16 2 2 76 2 0 0 6 73 1 4 64
6 36 2 31 0 0 86 0 560 64 1 5 0 18
152 5 251 10 85 34 104 2 1 68 0 31 24 0
267 0 19 0 2 11 3 0 1 8 1 16 6 4
6 513 138 490 25 0 6 0 148 805 5 634 5 2

27 5 211 2 8 164 5 1 722 157 2 17 4 140

0 0 22 3 43 7 10 11 41 254 68 3 7 1

OTU278 1 0 0 0 0 4 0 18 40 2 0 0 0

0 0 1 1 0 0 142 12 0 0 0 0 1 0

15 0 0 0 0 0 0 0 0 1 0 0 1 0

0 14 0 12 3 0 0 0 1 0 0 0 0 0

0 0 0 3 0 0 0 0 0 0 0 0 19 0

0 0 6 0 23 5 3 1 0 3 1 2 1 0

0 0 2 2 0 0 19 1 3 10 0 0 0 0

1 0 0 0 0 0 0 0 0 13 3 0 2 9

0

OTU1485 3 0 1 0 0 2 1 0 1 1 1 4 1

3 0 4 0 1 0 2 0 0 0 0 8 0 0

1 0 5 0 0 24 1 12 12 0 0 5 1 1

0 26 0 6 0 0 5 4 0 0 0 0 0 6

1 0 0 1 0 0 0 0 0 0 0 0 0 0

0 0 1 42 1 6 4 0 11 0 0 0 4 0

16 0 3 1 29 0 2 0 0 1 2 0 0 4

3 1 0 0 0 0 0 0 0 0 6 3 0 0

0

OTU3373 1 0 0 0 0 0 0 0 0 0 0 0 0

1 0 0 0 0 0 0 0 1 0 2 0 2 0

1 4 0 0 1 0 0 0 0 0 0 0 0 0

0 0 3 0 0 0 0 0 0 3 2 0 1 0

1 0 0 0 0 1 0 0 1 1 5 0 0 0

0 0 0 0 0 0 0 1 1 0 0 1 0 0

0 11 0 0 0 0 4 1 0 0 0 0 0 0

0 0 0 0 0 0 0 0 0 0 0 0 0 0

0

OTU16570 1 0 0 0 1 0 0 0 0 1 0 0 0

0 0 0 0 0 0 0 0 0 0 0 0 0 0

0 0 0 0 0 0 0 3 0 0 0 0 0 1

0 1 0 0 0 0 0 0 0 0 0 0 0 0

0 0 0 0 0 0 0 0 0 0 0 0 0 0
0 0 0 0 0 0 0 0 1 0 0 0 1 0
0 0 0 0 0 0 2 0 0 0 0 0 0 0
1 0 0 0 0 0 0 0 0 0 1 1 1 0
0
OTU10980 1 2 0 0 0 0 0 0 0 0 0 0 0
1 0 0 0 0 0 1 0 0 0 0 0 1 0
0 0 0 0 0 0 0 0 0 0 0 0 0 0
0 0 0 0 0 0 0 0 0 0 1 0 0 0
0 0 0 0 0 0 0 0 0 0 0 0 0 0
0 0 0 2 0 0 0 0 0 0 0 0 1 0
0 0 1 0 1 0 1 0 0 0 0 0 0 0
0 0 0 0 0 0 0 0 0 0 0 1 1 0
0
OTU14283 1 0 0 0 0 0 0 0 0 0 0 0 0
0 0 0 0 0 0 0 0 0 0 0 0 0 0
0 0 0 0 0 0 0 0 0 0 0 0 0 0
0 0 0 0 0 0 0 0 0 0 0 0 0 0
0 0 0 0 0 0 0 0 0 0 0 0 0 0
0 0 0 0 0 0 0 0 0 0 0 0 0 0
0 0 0 0 0 0 0 0 0 0 0 0 0 0
0 0 0 0 0 0 0 0 0 0 0 0 0 0
0
OTU12507 2 1 0 2 2 1 1 0 1 0 1 0 0
0 0 0 0 0 0 0 0 0 0 0 0 0 6
0 0 0 0 0 0 0 0 0 0 0 1 0 0
0 0 0 0 0 1 0 1 0 0 0 0 0 1
0 1 0 3 0 0 0 1 0 0 0 1 2 0
0 0 0 0 4 1 0 0 0 2 0 0 0 0
0 0 0 0 0 0 1 0 0 0 0 0 0 0
0 0 0 0 0 0 0 0 0 0 0 0 0 0
0

OTU518	1	0	1	0	0	3	0	0	1	3	0	1	0
0	0	0	1	0	0	6	0	0	0	0	0	2	0
0	0	0	0	0	0	0	0	0	0	0	0	0	0
0	0	0	0	0	0	0	1	0	0	0	0	0	0
0	1	0	0	0	0	0	0	0	0	0	0	0	0
0	1	7	0	0	0	0	0	0	1	0	0	0	0
0	0	0	0	0	0	0	0	0	0	0	0	0	0
0	0	0	0	1	0	2	0	3	11	0	0	0	1
3													
OTU11635	1	1	0	0	0	0	0	1	0	1	1	0	0
1	0	2	0	1	0	0	1	0	2	0	1	2	0
0	1	3	1	0	1	0	1	1	1	0	1	1	2
0	0	3	1	0	1	0	1	0	2	1	0	0	1
0	1	1	0	0	0	0	0	0	0	2	0	1	0
2	1	0	1	1	2	0	1	0	0	0	3	0	1
0	1	0	1	1	2	0	2	0	0	0	0	0	0
0	1	1	0	0	0	0	1	1	0	0	0	0	0
1													
OTU1065	2	0	1	0	0	0	0	0	3	0	0	2	0
0	0	1	10	0	0	1	1	4	0	7	0	4	0
4	11	0	0	0	0	3	0	0	0	1	0	5	0
0	0	3	0	6	0	0	0	7	7	1	1	1	0
5	0	5	1	2	8	4	1	0	0	2	0	0	1
11	2	1	1	0	0	0	9	1	0	1	3	0	0
2	6	0	1	0	1	1	1	0	0	0	0	0	1
0	0	4	5	0	4	0	0	2	5	0	0	0	6
0													
OTU3989	1	0	0	0	0	0	0	0	0	0	0	0	0
0	0	0	0	0	0	0	0	0	0	0	0	0	0
0	1	0	0	0	0	0	0	0	0	0	0	0	0
0	0	0	0	0	0	0	0	0	0	0	0	0	0
0	0	0	0	0	0	0	0	0	0	0	0	2	0
0	0	0	0	0	0	0	0	0	0	0	0	0	0

0	0	0	0	0	0	0	0	0	0	0	0	0	0
0	0	0	0	0	0	0	0	0	0	0	0	0	0
0													
OTU9691	1	0	0	1	0	0	2	0	0	2	1	0	2
0	4	0	2	0	1	1	0	1	0	0	1	1	0
3	0	2	0	0	0	0	1	0	0	0	0	1	0
1	1	2	0	0	0	1	1	0	0	0	0	2	1
1	0	0	0	0	0	0	1	1	0	0	1	0	0
1	0	0	0	1	0	1	1	1	0	0	4	0	1
1	0	0	0	0	1	0	1	1	0	0	1	0	0
0	0	1	1	0	0	0	0	0	0	1	1	0	0
5													
OTU14227	1	1	1	1	3	2	1	0	2	1	0	0	1
0	6	1	0	1	0	1	0	1	0	1	1	0	0
0	0	2	2	0	0	0	1	0	2	1	1	2	2
6	2	0	0	0	0	0	1	3	1	0	0	0	0
0	0	1	1	0	1	1	0	2	0	2	1	1	1
1	3	3	0	1	1	0	2	2	0	0	1	0	2
1	0	1	0	1	0	0	3	1	0	2	1	0	2
0	0	2	0	0	1	0	1	0	1	0	0	3	3
2													
OTU14284	1	0	0	0	0	0	0	0	0	0	0	0	0
0	0	0	0	0	0	0	0	0	0	0	0	0	0
0	0	0	0	0	0	0	0	0	0	0	0	0	0
0	0	0	0	0	0	0	0	0	0	0	0	0	0
0	0	0	0	0	0	0	0	0	0	0	0	0	0
0	0	0	0	0	0	0	0	0	0	0	0	0	0
0	0	0	0	0	0	0	0	0	0	0	0	0	0
0	0	0	0	0	0	0	0	0	0	0	0	0	0
0													
OTU1230	1	0	8	2	4	4	1	7	4	4	3	4	2
2	3	10	3	9	1	1	1	4	5	0	4	5	3
5	3	5	5	2	3	2	8	2	0	3	7	20	4

3	6	4	5	1	2	3	1	4	7	2	4	1	1
6	2	6	3	1	3	3	2	4	4	8	3	3	1
6	4	2	2	10	2	1	9	3	1	4	17	1	5
3	3	1	5	2	6	2	5	2	1	2	3	3	2
1	4	2	4	3	2	4	9	0	3	2	0	2	3
14													
OTU206	1	4	5	0	0	0	0	1	3	3	0	4	0
1	0	24	0	3	0	29	97	0	19	14	151	8	1
23	8	0	16	1	3	1	0	0	5	0	1	7	0
0	1	1	7	4	2	0	0	33	44	40	88	29	0
5	2	6	19	4	31	12	59	32	4	61	2	3	0
6	3	4	8	16	73	2	7	5	0	1	52	1	6
2	39	6	2	3	1	3	12	1	4	0	0	0	2
0	3	1	6	2	1	5	4	3	5	0	0	11	7
13													
OTU10703	1	0	1	0	0	0	0	0	0	0	0	0	0
0	0	0	0	0	0	0	0	0	0	0	0	0	0
0	0	0	0	0	0	0	0	0	0	0	0	0	0
0	0	0	0	0	0	0	0	0	0	0	0	0	0
0	0	0	0	0	0	0	0	0	0	0	0	0	0
0	0	0	0	0	0	0	0	0	0	0	0	0	0
0	0	0	0	0	0	0	0	0	0	0	0	0	0
0	0	0	0	0	2	0	0	0	0	0	0	0	0
0													
OTU10962	1	0	0	0	0	0	0	0	0	0	0	0	0
0	0	0	0	0	0	0	0	0	0	0	0	0	0
0	0	0	0	0	0	0	0	0	0	0	0	0	0
0	0	0	0	0	0	0	0	0	0	0	0	0	0
0	0	0	0	0	0	0	0	0	0	0	0	0	0
0	0	0	0	0	0	0	0	0	0	0	0	0	0
0	0	0	0	0	0	0	0	0	0	0	0	0	0
0	0	0	0	0	0	0	0	0	0	0	0	0	0
0													

OTU3790	1	0	0	0	0	0	2	0	0	0	0	1	0
0	1	0	1	0	0	0	0	0	0	1	0	4	0
0	0	0	0	0	0	0	0	0	0	0	0	1	0
0	0	0	1	0	1	0	0	0	0	0	0	0	0
0	0	0	0	0	0	0	0	0	0	0	0	0	0
0	0	0	0	0	0	0	1	0	0	0	0	0	0
0	0	0	0	0	0	0	0	0	0	0	0	0	0
0	0	0	0	0	0	0	0	0	0	0	0	0	0
0													
OTU8512	1	0	0	0	0	0	0	0	0	0	0	0	0
0	0	0	0	0	0	0	0	0	0	0	0	0	0
0	0	0	0	0	0	0	0	0	0	0	1	0	0
0	0	0	0	0	0	0	0	0	0	0	0	0	0
0	0	0	0	0	0	0	0	0	0	0	0	0	0
0	0	0	0	0	0	0	0	0	0	0	0	0	0
0	0	0	0	0	0	0	0	0	0	0	0	0	0
0	0	0	0	0	0	0	0	0	0	0	0	0	0
0													
OTU13681	2	2	2	1	2	1	0	1	1	7	1	2	1
2	6	4	2	6	2	0	4	3	0	2	1	1	0
4	1	4	3	2	0	0	4	5	4	0	3	7	1
7	2	3	5	2	1	0	0	3	4	2	4	0	2
4	0	5	1	1	1	3	2	2	4	3	0	1	3
7	1	2	2	3	0	3	1	4	2	2	5	5	0
4	2	2	1	5	0	3	3	3	2	4	1	2	0
2	5	3	1	0	6	2	2	2	1	1	0	3	1
12													
OTU140	2	1	2	1	0	2	2	4	11	2	1	0	1
0	0	0	1	0	0	2	0	8	0	0	0	0	0
31	0	0	0	1	0	2	0	0	3	1	0	7	0
3	0	2	0	0	33	0	3	0	0	0	11	1	0
0	0	0	0	0	2	0	29	23	3	0	0	45	0
0	102	1	0	3	0	0	2	0	10	0	28	0	41

0	0	0	5	0	1	1	1	0	0	0	1	12	0
0	6	96	40	60	205	95	31	29	31	0	4	0	50
39													
OTU14242	1	0	0	0	0	0	0	0	0	0	0	0	0
0	0	0	0	0	0	0	0	0	0	0	0	0	0
0	0	0	0	0	0	0	0	0	0	0	0	0	0
0	0	0	0	0	0	0	0	0	0	0	0	0	0
0	0	0	0	0	0	0	0	0	0	0	0	0	0
0	0	0	0	0	0	0	0	0	0	0	0	0	0
0	0	0	0	0	0	0	0	0	0	0	0	0	0
0	0	0	0	0	0	0	0	0	0	0	0	0	0
0													
OTU12458	1	0	1	0	0	0	0	0	23	0	0	0	0
0	0	0	0	0	0	0	0	0	0	0	0	1	0
2	0	0	0	0	0	0	0	1	0	0	0	0	0
2	0	1	0	0	0	0	1	0	0	0	0	0	0
0	0	0	1	0	0	0	0	0	0	0	0	0	0
0	2	0	0	0	0	0	11	0	1	0	0	0	0
0	0	0	1	0	0	0	0	0	0	0	5	0	0
0	0	0	0	0	0	0	0	0	0	0	0	0	0
0													
OTU14243	1	0	0	0	0	0	0	0	0	0	0	0	0
0	0	0	0	0	0	0	0	0	0	0	0	0	0
0	0	0	0	0	0	0	0	0	0	0	0	0	0
0	0	0	0	0	0	0	0	0	0	0	0	0	0
0	0	0	0	0	0	0	0	0	0	0	0	0	0
0	0	0	0	0	0	0	0	0	0	0	0	0	0
0	0	0	0	0	0	0	0	0	0	0	0	0	0
0	0	0	0	0	0	0	0	0	0	0	0	0	0
0													
OTU10966	1	0	0	0	0	0	0	0	0	0	0	0	0
0	0	0	0	0	0	0	0	0	0	0	0	0	0
0	0	0	0	0	0	0	0	0	0	0	0	0	0

0	0	0	0	0	0	0	0	0	0	0	0	0	0
0	0	0	0	0	0	0	0	0	0	0	0	0	0
0	0	0	0	0	0	0	0	0	0	0	0	0	0
0	0	0	0	0	0	0	0	0	0	0	0	0	0
0	0	0	0	0	0	0	0	0	0	0	0	0	0
0													
OTU11792	2	0	0	0	1	1	0	2	1	0	0	2	1
0	0	0	0	0	0	2	0	1	0	0	1	0	2
0	0	1	1	0	2	0	3	0	4	2	2	2	0
3	1	2	0	0	0	0	0	0	1	0	1	0	1
0	0	0	1	1	2	0	0	0	0	2	1	0	0
3	0	1	0	2	1	2	1	0	0	0	2	0	0
1	1	0	0	0	2	0	2	0	0	1	0	0	1
0	0	0	1	2	0	0	3	0	1	0	0	0	0
1													
OTU8338	1	0	1	0	1	0	1	0	0	0	0	0	0
0	0	0	0	0	0	0	0	0	0	0	0	0	0
0	0	0	1	0	0	0	0	0	0	0	0	0	0
2	0	0	1	0	1	0	1	0	0	0	0	0	0
0	0	0	0	0	0	0	0	0	0	0	0	0	0
0	0	0	0	0	0	1	0	0	1	1	0	0	0
0	0	0	1	0	2	0	0	0	0	0	0	0	0
0	0	0	0	0	0	0	0	0	0	0	0	0	0
0													
OTU2236	1	0	0	0	0	0	0	0	0	0	0	1	0
0	1	0	0	0	0	1	0	0	0	0	0	0	0
0	0	0	0	0	0	0	1	1	0	0	0	0	0
1	1	0	0	0	2	0	1	0	0	0	0	0	2
0	1	0	0	0	0	0	0	0	0	0	0	0	0
0	2	0	0	0	1	0	0	1	1	0	0	1	0
0	0	0	0	0	0	0	1	0	0	0	0	1	1
3	0	0	1	0	0	0	0	0	0	1	0	0	1
0													

OTU752	2	0	0	2	5	0	0	0	0	0	5	2	0
1	1	0	2	0	0	2	0	0	0	0	0	0	0
0	0	0	3	0	0	5	3	4	0	0	0	1	0
0	0	0	0	0	1	6	1	0	0	0	0	0	1
0	0	0	0	0	0	0	1	0	0	0	0	0	0
1	0	0	0	2	0	0	0	0	0	0	0	0	1
0	0	0	0	0	0	0	0	8	0	1	0	0	7
1	0	0	0	0	0	0	0	0	0	0	2	0	0
0													
OTU522	1	0	0	0	0	0	0	0	0	3	0	1	0
2	2	0	1	0	2	15	0	0	0	1	0	0	0
0	0	0	0	0	2	0	0	0	2	2	0	2	0
0	11	0	0	0	0	0	0	0	0	0	1	0	0
0	0	0	0	0	0	0	0	1	0	0	0	2	0
0	0	4	0	0	1	0	0	3	0	0	0	1	0
0	0	0	0	1	0	0	0	0	0	0	0	0	1
4	0	0	1	3	2	0	0	2	6	1	2	0	1
0													
OTU7487	2	0	0	1	1	1	2	2	0	1	0	0	0
0	0	3	0	4	0	0	0	0	1	1	1	1	1
0	0	1	1	0	0	1	0	1	2	0	1	3	1
0	0	1	0	0	0	2	0	0	1	0	0	0	0
1	0	0	0	0	1	0	2	0	0	0	0	0	0
2	3	0	0	1	0	0	0	0	2	0	3	0	1
0	2	0	1	0	0	0	3	2	0	0	0	0	0
0	0	1	1	3	0	1	0	2	0	0	0	0	1
3													
OTU10739	1	1	0	5	1	3	0	1	0	0	0	3	0
0	6	0	0	3	0	1	1	0	0	0	2	0	0
1	0	0	0	0	3	2	3	11	0	0	0	3	0
6	1	0	0	0	3	2	12	0	0	2	0	0	9
0	6	0	1	0	0	0	0	0	0	0	0	1	0
0	12	0	0	0	3	0	1	1	3	0	0	0	0

0	0	0	0	1	2	0	3	0	1	1	15	31	0
1	1	0	3	1	0	0	13	2	4	3	0	0	15
1													
OTU1743	3	0	2	0	2	1	0	3	0	2	2	1	0
0	0	1	0	1	0	0	1	3	1	1	0	2	1
2	1	3	1	2	2	2	0	1	1	1	1	1	1
0	4	0	0	2	0	0	0	1	3	0	1	0	2
1	2	1	1	0	1	0	1	1	1	2	1	0	0
0	0	1	1	1	1	0	2	1	1	0	7	1	1
0	0	0	0	1	2	0	1	0	2	1	0	1	1
0	1	1	1	0	0	0	1	2	3	0	0	0	0
1													
OTU6145	1	1	0	0	1	1	0	0	0	0	0	0	1
0	1	0	0	1	0	0	0	0	0	0	1	0	0
0	0	1	1	1	2	0	0	0	0	0	0	0	2
0	0	0	0	0	0	0	0	0	1	0	0	1	0
0	0	0	1	0	0	0	0	0	0	0	0	1	1
0	0	1	0	0	1	0	1	0	0	0	1	0	0
0	0	0	0	0	0	0	0	0	0	0	0	0	0
0	0	0	0	0	0	0	0	0	0	0	0	0	0
1													
OTU7882	1	0	0	1	1	1	0	1	0	2	0	2	1
0	1	0	0	0	1	0	0	0	0	0	1	0	0
2	0	0	0	0	0	0	1	2	0	0	0	1	0
1	1	0	0	0	2	0	4	0	0	0	0	0	2
0	1	0	5	0	0	0	0	0	0	0	0	1	1
1	1	1	0	0	0	0	0	0	0	1	0	0	2
1	0	0	4	0	1	2	4	0	0	1	2	0	0
0	0	0	1	0	0	0	2	0	0	0	0	0	2
0													
OTU6192	1	2	0	0	0	2	0	0	1	0	0	0	0
0	0	0	0	0	0	0	0	0	0	0	0	0	0
0	0	0	0	0	0	0	0	0	0	0	0	0	0

0	0	0	0	0	0	0	0	0	0	0	0	0	0
0	0	0	0	0	0	0	0	0	0	0	0	1	0
0	0	0	0	0	0	0	0	0	0	0	0	0	0
0	0	0	0	0	0	0	0	0	1	0	0	0	0
0	0	0	0	0	0	0	0	0	1	0	0	0	0
0													
OTU6099	1	0	0	0	0	0	0	0	0	0	0	0	0
0	0	0	0	0	0	0	0	0	0	0	0	0	1
0	0	0	0	0	0	0	0	0	0	0	0	2	0
0	0	0	0	0	1	0	0	0	0	0	0	0	0
0	0	0	2	0	0	0	0	0	0	0	2	0	0
0	0	0	0	0	0	0	0	0	0	0	0	0	0
0	0	0	0	0	0	0	0	0	0	0	0	0	0
0	0	0	0	0	0	0	0	0	0	0	0	0	0
0													
OTU7394	2	2	5	4	5	3	2	5	3	4	0	2	4
1	5	5	2	3	2	0	3	1	0	3	1	2	1
2	0	2	2	2	2	1	4	3	1	0	1	8	1
2	1	3	1	1	1	1	1	6	7	1	3	0	2
0	2	6	4	2	3	1	1	4	1	3	1	2	0
4	3	0	2	7	1	2	5	1	0	1	9	0	3
0	2	0	1	2	4	0	3	0	0	3	4	1	2
0	5	0	4	1	1	1	2	2	3	2	1	1	0
7													
OTU14241	1	1	0	1	1	2	0	2	0	1	1	0	0
0	0	0	0	0	0	0	0	0	0	0	0	0	0
1	0	0	0	0	0	0	0	1	0	0	0	0	0
0	0	0	0	0	0	0	1	0	0	0	0	0	0
0	1	0	1	0	0	0	0	0	0	0	0	0	0
0	0	0	0	0	0	0	0	0	0	0	0	0	1
0	0	0	0	0	1	0	1	0	0	0	0	0	0
0	0	0	0	0	0	0	0	0	0	0	0	0	0
0													

OTU9203 1 0 0 0 0 0 0 0 0 0 0 0 0
0 0 0 0 0 0 0 0 0 0 1 0 0 0
0 0 0 0 0 0 0 0 0 0 1 0 0 0
0 0 0 0 0 0 0 0 0 0 0 0 0 1
0 0 0 0 0 0 0 0 0 1 0 0 0 0
0 0 0 0 0 0 0 0 0 0 0 0 0 0
0 0 0 0 0 0 0 0 0 0 0 0 0 0
0 0 0 0 0 0 0 0 0 0 0 1 0 0
0
OTU14094 1 0 0 0 0 0 5 0 0 0 0 0 0
0 2 0 0 0 0 0 0 1 0 0 0 1 0
0 0 0 0 0 0 3 0 0 0 0 1 4 0
0 0 0 0 0 16 0 0 0 0 0 5 0 1
0 0 0 27 0 1 0 1 6 0 2 4 3 0
1 0 0 0 6 0 0 0 2 9 0 1 0 0
0 3 0 3 0 0 0 36 0 0 0 0 0 0
0 0 0 0 0 0 0 0 0 0 0 0 0 0
0
OTU5349 1 0 0 6 0 0 1 0 0 0 0 0 0
0 0 0 0 0 0 0 0 0 0 0 0 3 0
1 0 0 0 0 0 0 0 3 0 0 0 0 0
0 0 0 0 1 0 0 0 0 0 0 0 0 0
0 0 0 0 0 0 0 0 0 0 0 0 0 0
0 0 0 0 1 0 0 0 0 0 0 0 1 0
0 0 0 0 0 0 0 0 0 0 0 0 0 0
0 0 0 0 0 0 0 0 0 0 0 0 0 0
0
OTU8739 1 1 0 0 0 1 0 0 0 0 0 0 0
1 0 1 0 5 0 0 20 1 0 0 0 2 1
0 1 0 0 0 28 0 0 0 36 0 5 0 1
0 1 0 0 0 0 3 0 0 0 3 0 0 0
0 0 0 0 0 0 0 0 0 0 0 0 0 4
0 0 0 0 0 4 0 0 0 0 0 0 2 0

0	0	0	0	0	0	0	0	8	0	1	0	0	0
1	0	0	0	0	0	0	0	0	0	0	0	0	0
0													
OTU7619	1	0	0	1	1	0	0	0	0	2	0	0	0
0	3	1	0	0	0	0	0	0	0	0	0	0	0
0	0	1	0	0	0	0	1	0	2	1	0	1	0
1	1	1	0	0	0	0	0	0	0	0	0	0	0
0	0	0	1	0	0	0	0	0	0	0	0	0	0
1	0	0	0	1	1	0	1	0	0	0	1	0	1
0	0	0	0	0	1	0	0	0	0	0	0	0	0
0	0	0	0	0	1	0	0	0	1	0	0	1	0
2													
OTU12244	1	0	0	0	0	0	0	0	0	0	0	0	0
0	0	0	0	0	0	0	0	0	0	0	0	0	0
0	0	0	0	0	0	0	0	0	0	0	0	0	0
0	0	0	0	0	0	0	0	0	0	0	0	0	0
0	0	0	0	0	0	0	0	0	0	0	0	0	0
0	0	0	0	0	0	0	0	0	0	0	0	0	0
0	0	0	0	0	0	0	0	0	0	0	0	0	0
0	0	0	0	0	0	0	0	0	0	0	0	0	0
0													
OTU10967	1	0	0	0	0	0	0	0	0	0	0	0	0
0	0	0	0	0	0	0	0	0	0	0	0	0	0
0	0	0	0	0	0	0	0	0	0	0	0	0	0
0	0	0	0	0	0	0	0	0	0	0	0	0	0
0	0	0	0	0	0	0	0	0	0	0	0	0	0
0	0	0	0	0	0	0	0	0	0	0	0	0	0
0	0	0	0	0	0	0	0	0	0	0	0	0	0
0	0	0	0	0	0	0	0	0	0	0	0	0	0
0													
OTU7274	2	0	0	1	1	1	3	6	2	2	2	0	1
1	2	5	0	3	3	1	1	1	3	1	2	2	0
3	1	4	3	2	1	1	0	1	0	1	7	4	2

3	3	3	2	0	1	2	1	1	7	1	1	0	0
0	0	2	2	1	4	0	2	2	1	2	1	1	0
1	2	2	2	3	0	2	2	0	1	1	7	2	1
0	2	3	1	1	4	1	0	1	1	1	1	2	0
2	5	1	1	1	3	2	4	1	0	3	0	0	3
5													
OTU12245	1	0	0	0	0	0	0	0	0	0	0	0	0
0	0	0	0	0	0	0	0	0	0	0	0	0	0
0	0	0	0	0	0	0	0	0	0	0	0	0	0
0	0	0	0	0	0	0	0	0	0	0	0	0	0
0	0	0	0	0	0	0	0	0	0	0	0	0	0
0	0	0	0	0	0	0	0	0	0	0	0	0	0
0	0	0	0	0	0	0	0	0	0	0	0	0	0
0	0	0	0	0	0	0	0	0	0	0	0	0	0
0													
OTU1035	1	0	0	0	0	0	1	0	0	0	0	0	0
0	0	0	0	0	0	0	0	0	0	0	0	0	0
0	0	0	0	0	0	0	0	0	0	0	0	0	0
0	0	0	0	0	0	0	0	0	0	0	0	1	0
0	1	0	0	0	0	0	0	0	0	0	0	0	1
0	0	0	0	0	0	0	0	0	0	0	0	0	0
0	0	0	0	0	0	0	0	0	0	0	0	0	0
0	0	0	0	0	0	0	0	0	0	0	0	0	0
0													
OTU12246	1	2	0	0	0	1	0	0	0	0	0	0	0
0	2	0	0	0	0	0	0	0	0	0	0	0	0
0	0	0	0	1	0	1	0	1	0	0	0	0	0
0	0	0	0	0	0	0	0	0	0	0	0	0	0
0	0	0	0	0	0	0	0	0	0	0	0	1	0
0	0	2	0	0	0	0	0	0	0	0	0	0	0
0	0	0	0	0	0	0	0	0	1	0	0	0	0
0	0	0	0	0	0	0	0	0	0	0	0	0	0
0													

OTU12247	1	0	0	0	0	0	0	0	0	0	0	0	0
0	0	0	0	0	0	0	0	0	0	0	0	0	0
0	0	0	0	0	0	0	0	0	0	0	0	0	0
0	0	0	0	0	0	0	0	0	0	0	0	0	0
0	0	0	0	0	0	0	0	0	0	0	0	0	0
0	0	0	0	0	0	0	0	0	0	0	0	0	0
0	0	0	0	0	0	0	0	0	0	0	0	0	0
0	0	0	0	0	0	0	0	0	0	0	0	0	0
0													
OTU8515	1	0	0	0	0	0	1	0	0	0	0	0	0
0	0	0	0	0	1	0	0	0	0	0	1	0	0
0	0	0	0	0	1	0	0	0	0	0	0	0	1
0	0	0	0	0	0	0	0	0	0	0	0	0	0
0	0	0	0	0	0	0	0	0	0	0	0	0	0
0	0	0	0	0	0	0	0	0	0	0	0	0	0
0	0	0	0	0	0	0	0	0	0	0	0	0	0
0	0	0	0	0	0	0	0	0	0	0	0	0	0
0													
OTU433	1	0	0	0	0	0	0	0	0	0	0	0	0
0	0	0	0	0	0	0	0	0	0	0	0	0	0
0	2	0	0	5	0	0	0	0	0	0	0	0	0
0	0	0	0	2	1	0	0	0	9	0	0	0	0
0	0	0	0	2	0	0	11	11	0	12	0	0	0
0	0	0	0	0	0	0	0	0	0	0	0	0	0
0	0	0	0	0	0	0	0	0	0	0	0	0	0
0	0	0	0	0	0	0	0	0	0	0	0	0	0
0													
OTU14245	1	0	0	0	0	0	0	0	0	0	0	0	0
0	0	0	0	0	0	0	0	0	0	0	0	0	0
0	0	0	0	0	0	0	0	0	0	0	0	0	0
0	0	0	0	0	0	0	0	0	0	0	0	0	0
0	0	0	0	0	0	0	0	0	0	0	0	0	0
0	0	0	0	0	0	0	0	0	0	0	0	0	0

0	0	0	0	0	0	0	0	0	0	0	0	0	0
0	0	0	0	0	0	0	0	0	0	0	0	0	0
0													
OTU12255	1	0	0	1	0	0	0	1	0	1	0	0	0
0	1	0	0	0	0	0	0	0	0	0	0	0	0
0	0	0	0	0	0	0	0	0	0	0	0	0	0
0	0	0	0	0	0	0	0	0	0	0	0	0	0
0	0	0	0	0	0	0	0	0	2	0	0	0	0
1	0	0	0	0	0	0	0	0	0	0	0	0	0
0	1	0	0	0	0	0	0	0	0	0	0	0	0
0	0	0	0	0	0	0	0	0	0	0	1	0	0
2													
OTU183	1	0	0	1	9	0	0	1	26	2	0	0	0
0	0	0	2	0	0	69	0	0	0	0	0	2	0
0	0	0	0	0	0	0	0	0	0	0	0	2	0
0	0	1	0	0	0	0	0	0	0	0	1	0	0
0	1	0	12	0	0	0	0	1	0	0	0	9	0
1	9	4	0	9	0	0	0	0	11	7	2	0	2
0	0	0	2	0	2	0	9	0	0	0	0	6	0
1	0	6	13	0	14	20	0	75	149	0	0	0	7
2													
OTU9046	1	0	1	0	3	0	0	1	0	1	1	0	0
2	7	1	0	0	0	0	0	0	0	0	0	0	0
0	1	0	0	0	0	0	0	1	0	1	1	3	1
0	1	1	0	0	0	2	0	0	5	0	0	1	0
1	0	1	0	1	0	0	0	0	0	0	0	0	1
1	0	1	0	0	1	0	3	0	1	0	1	0	0
1	0	0	0	0	0	0	1	0	0	0	0	1	0
1	2	1	0	1	2	0	0	0	0	1	2	1	0
5													
OTU8514	1	0	0	0	0	0	0	0	0	0	0	0	0
0	0	0	0	0	0	0	0	0	0	0	0	0	0
0	0	0	0	0	0	0	0	0	0	0	0	0	0

0	0	0	0	0	0	0	0	0	0	0	0	0	0
0	0	0	0	0	0	0	0	0	0	0	0	0	0
0	0	0	0	0	0	0	0	0	0	0	0	0	0
0	0	0	0	0	0	0	0	0	0	0	0	0	0
0	0	0	0	0	0	0	0	0	0	0	0	0	0
0													
OTU436	1	0	3	1	1	0	0	2	4	1	1	0	0
0	0	2	0	0	1	4	0	0	0	0	1	0	0
0	0	0	0	0	0	0	0	0	1	0	0	1	0
0	1	0	1	1	0	0	1	0	0	0	0	0	0
0	0	0	1	0	0	0	0	0	0	0	0	0	0
0	3	1	1	0	0	0	0	0	2	1	0	0	0
0	0	0	1	0	0	1	0	0	0	0	3	1	0
0	10	1	1	0	0	0	1	0	4	1	0	0	3
0													
OTU8516	1	0	0	0	0	0	0	0	0	0	0	0	0
0	0	0	0	0	0	0	0	0	0	0	0	0	0
0	0	0	0	0	0	0	0	0	0	0	0	0	0
0	0	0	0	0	0	0	0	0	0	0	0	0	0
0	0	0	0	0	0	0	0	0	0	0	0	0	0
0	0	0	0	0	0	0	0	0	0	0	0	0	0
0	0	0	0	0	0	0	0	0	0	0	0	0	0
0	0	0	0	0	0	0	0	0	0	0	0	0	0
0													
OTU3787	1	1	0	0	1	0	0	0	0	1	0	0	2
0	0	0	5	0	0	0	0	0	0	2	0	1	1
0	2	0	0	0	0	1	0	0	0	2	0	0	0
0	0	0	0	3	0	1	0	0	0	0	0	0	2
0	0	1	0	1	0	0	0	0	1	0	1	0	0
0	0	3	0	0	0	0	0	0	0	0	0	1	0
0	0	0	0	0	0	0	0	1	0	0	0	1	3
0	0	0	0	0	0	0	0	0	2	0	0	0	0
0													

OTU362 2 10 2 0 1 2 1 0 5 0 4 0 8
0 0 0 1 3 7 4 0 0 5 0 0 0 0
0 3 0 1 2 0 12 0 0 8 2 5 0 0
0 0 0 0 1 3 0 0 0 6 3 3 0 0
0 0 0 2 0 0 0 2 2 0 0 0 1 0
2 0 6 0 0 0 0 1 0 0 0 0 0 5
0 0 0 1 0 0 0 0 1 1 0 0 0 0
0 0 0 0 0 0 0 0 0 0 2 0 0 0
0
OTU6025 1 0 1 1 1 2 0 2 0 0 2 3 0
0 0 3 0 2 0 1 0 0 0 0 0 0 0
0 0 0 0 0 0 0 1 0 0 0 0 0 0
1 3 0 3 0 0 0 0 0 0 0 0 0 0
0 1 0 0 0 0 0 1 0 0 0 0 1 1
0 0 0 1 0 8 0 0 0 0 3 0 3 0
0 0 0 0 2 0 0 0 0 0 1 0 0 0
2 0 0 0 0 0 0 0 0 0 4 1 1 0
0
OTU305 1 0 12 5 0 7 3 7 2 0 0 3 1
0 13 14 0 1 0 16 1 1 0 0 0 6 0
17 0 0 0 0 0 1 0 5 0 0 0 6 0
5 0 0 0 0 9 1 0 0 1 0 1 4 1
0 10 0 2 0 0 10 0 0 0 0 0 12 0
0 8 10 1 1 0 0 4 0 2 1 19 18 44
0 1 0 1 0 2 0 1 0 3 0 8 20 0
0 15 6 16 10 10 2 16 3 21 0 1 0 11
9
OTU6330 4 0 4 18 8 4 0 1 0 0 0 1 0
0 0 1 0 0 0 1 3 0 0 2 0 2 1
0 0 0 0 0 1 6 15 17 0 0 0 0 0
0 0 0 2 2 11 2 6 0 0 3 0 0 0
0 0 0 6 2 0 0 0 0 1 0 4 0 1
0 0 0 3 0 0 1 0 2 0 0 0 1 0

	0	0	2	0	31	0	1	0	1	0	9	0	0	3
	12	0	0	0	0	0	0	0	0	0	0	2	1	0
	0													
OTU6333		1	0	0	1	0	2	0	0	0	0	0	4	0
	0	0	0	0	1	0	0	0	0	0	0	0	0	0
	0	0	0	0	0	1	0	0	0	0	0	0	0	0
	0	1	0	1	0	2	0	1	0	0	0	0	0	3
	0	0	0	0	0	0	0	0	0	0	0	0	0	0
	0	0	0	0	0	0	0	0	0	1	0	0	1	0
	0	0	0	0	0	0	0	0	0	0	0	1	0	0
	1	0	0	0	0	0	0	0	0	0	1	0	0	0
	0													
OTU8513		1	0	0	0	0	0	0	0	0	0	0	0	0
	0	0	0	0	0	0	0	0	0	0	0	0	0	0
	0	0	0	0	0	0	0	0	0	0	0	0	0	0
	0	0	0	0	0	0	0	0	0	0	0	0	0	0
	0	0	0	0	0	0	0	0	0	0	0	0	0	0
	0	0	0	0	0	0	0	0	0	0	0	0	0	0
	0	0	0	0	0	0	0	0	0	0	0	0	0	0
	0	0	0	0	0	0	0	0	0	0	0	0	0	0
	0													
OTU971		2	0	0	24	4	4	3	8	1	1	8	19	38
	52	3	53	278	30	29	4	50	62	9	291	8	6	29
	0	66	40	72	106	81	109	40	46	52	61	17	5	41
	1	2	61	11	33	1	24	0	38	40	18	4	3	7
	43	3	82	0	44	16	11	12	5	27	46	31	8	69
	45	0	8	54	0	60	35	4	55	1	83	1	21	0
	3	5	126	0	31	1	11	6	47	7	17	0	0	31
	37	0	0	7	0	1	0	0	2	0	58	34	154	0
	0													
OTU1979		1	2	29	31	4	29	43	30	25	42	2	8	2
	0	15	1	1	1	0	39	0	0	0	0	0	55	13
	79	0	0	0	2	0	20	0	7	0	0	0	25	0

51	6	2	0	1	12	0	40	0	0	0	11	1	0
0	4	1	6	0	0	0	10	2	15	0	0	89	0
1	20	0	0	1	0	0	21	14	31	0	54	4	112
0	2	0	24	0	19	1	6	0	8	9	25	22	18
3	9	59	35	18	8	11	25	7	32	0	1	1	25
9													
OTU15255	1	0	1	0	2	0	2	0	1	2	0	0	0
1	2	1	0	0	0	0	0	0	1	0	1	0	2
1	0	2	0	0	0	0	0	0	0	0	0	1	0
2	1	1	2	0	0	0	0	0	0	0	0	0	0
0	0	0	0	0	0	0	1	0	0	0	0	0	1
0	1	1	1	1	0	1	1	0	0	0	0	0	0
1	0	0	0	0	1	1	0	0	0	0	0	0	0
0	4	1	0	0	0	0	0	1	0	0	0	0	1
1													
OTU3244	1	0	1	1	0	0	1	0	0	1	0	0	0
0	0	0	0	0	0	0	0	0	0	0	0	0	0
0	0	0	0	0	0	0	0	0	1	0	0	0	0
1	0	0	5	0	0	0	0	0	0	0	0	0	0
0	0	0	0	0	0	0	0	0	0	0	0	0	0
0	0	0	2	2	3	0	1	0	5	0	2	0	0
0	0	0	69	2	1	0	0	0	0	0	0	0	0
0	0	0	0	0	0	0	0	0	0	0	0	3	0
0													
OTU6763	1	1	0	0	2	0	0	1	1	1	3	2	0
1	2	1	0	1	1	0	0	2	4	2	0	1	1
1	1	1	1	0	2	0	0	0	1	0	1	8	0
0	1	0	0	0	1	0	0	2	3	1	0	2	1
3	0	3	1	1	1	0	3	2	0	1	2	0	0
3	2	3	0	2	0	1	1	2	0	0	4	0	1
1	1	0	1	0	2	1	0	0	0	1	1	4	0
0	2	0	0	2	0	1	1	1	1	2	0	1	1
2													

OTU292	1	0	3	0	0	0	0	0	2	0	0	27	0
0	3	1	0	0	0	1	2	0	0	2	0	109	0
1	45	0	0	0	0	1	0	0	5	0	0	8	0
2	4	0	0	0	0	0	0	23	0	64	105	0	0
0	2	2	6	0	13	2	14	13	0	41	0	3	0
0	3	0	1	2	0	0	0	1	0	0	15	1	6
0	1	0	0	0	0	13	2	0	3	0	0	0	0
0	2	10	0	3	0	0	0	0	2	36	0	1	4
1													
OTU3007	2	0	0	0	2	1	0	1	1	0	0	0	0
0	0	1	0	1	0	0	0	0	0	0	0	1	0
0	0	0	0	2	2	1	0	1	0	0	0	0	0
0	5	0	1	0	0	0	2	0	0	0	0	1	0
0	0	0	0	0	0	0	0	0	0	0	2	1	0
0	0	0	0	0	0	0	0	0	0	0	0	1	1
0	0	2	2	0	1	1	0	0	1	0	0	0	0
0	0	0	0	1	1	0	0	0	2	0	1	2	0
0													
OTU7434	2	0	1	3	1	2	2	0	0	1	0	23	0
0	0	0	0	0	0	8	4	0	0	0	2	0	0
0	0	0	0	0	0	0	1	0	8	2	1	18	0
1	12	0	1	0	7	1	2	0	0	2	1	0	0
0	0	0	38	0	1	0	0	1	0	1	0	24	0
0	1	0	0	1	0	0	0	0	0	1	2	0	16
0	1	0	8	0	1	0	4	0	3	0	0	5	0
0	8	7	1	9	2	0	4	1	0	1	1	1	4
4													
OTU9580	1	12	1	1	1	7	0	0	3	3	0	7	6
4	0	1	5	1	0	0	9	1	0	0	2	0	10
0	4	0	2	1	6	10	8	0	3	0	1	3	5
0	4	2	4	14	1	14	2	0	1	8	0	5	0
2	1	4	1	2	0	2	1	0	2	0	14	1	6
6	10	0	5	1	2	6	2	9	2	14	0	3	1

22	1	5	0	6	0	5	0	4	10	2	0	1	1
3	0	0	0	0	2	0	0	4	3	2	19	11	7
1													
OTU14240	1	0	0	0	0	0	0	0	0	0	0	0	0
0	0	0	0	0	0	0	0	0	0	0	0	0	0
0	0	0	0	0	0	0	0	0	0	0	0	0	0
0	0	0	0	0	0	0	0	0	0	0	0	0	0
0	0	0	0	0	0	0	0	0	0	0	0	0	0
0	0	0	0	0	0	0	0	0	0	0	0	0	0
0	0	0	0	0	0	0	0	0	0	0	0	0	0
0	0	0	0	0	0	0	0	0	0	0	0	0	0
0													
OTU14239	1	0	0	0	0	0	0	0	0	0	0	0	0
0	0	0	0	0	0	0	0	0	0	0	0	0	0
0	0	0	0	0	0	0	0	0	0	0	0	0	0
0	0	0	0	0	0	0	0	0	0	0	0	0	0
0	0	0	0	0	0	0	0	0	0	0	0	0	0
0	0	0	0	0	0	0	0	0	0	0	0	0	0
0	0	0	0	0	0	0	0	0	0	0	0	0	0
0	0	0	0	0	0	0	0	0	0	0	0	0	0
0													
OTU2394	1	0	0	0	0	0	0	0	1	0	0	0	0
0	0	0	4	0	0	1	0	6	0	0	0	1	0
0	14	0	0	7	0	0	0	0	0	8	0	2	0
0	0	1	0	1	0	0	0	0	2	0	0	0	0
3	0	2	0	0	14	0	1	0	0	0	1	0	2
4	0	1	0	0	0	0	1	0	0	0	0	0	0
0	2	0	0	0	0	0	0	0	0	0	0	0	0
0	0	0	0	0	0	1	0	1	1	0	0	0	0
0													
OTU4224	12	6	1	0	18	2	4	9	1	0	13	0	0
0	0	11	0	30	65	0	6	0	48	0	6	0	5
0	0	35	3	0	19	0	1	0	1	0	76	0	79

0	0	1	0	0	0	6	0	1	0	0	0	0	0
0	2	0	0	0	0	0	1	0	0	0	0	0	0
0	0	0	0	0	2	1	0	0	6	0	1	0	0
0	0	0	0	0	5	0	0	0	0	0	0	0	0
0	0	0	0	0	0	0	0	0	0	0	1	0	0
0													
OTU9807	1	0	0	0	0	1	1	1	0	0	0	2	0
0	1	1	0	0	0	0	1	0	0	0	1	0	0
0	0	5	3	0	4	0	2	3	7	0	2	0	6
0	0	0	2	0	0	11	0	0	0	0	0	0	0
0	0	0	0	0	0	0	0	0	0	0	0	0	0
0	0	0	2	0	0	1	0	14	0	3	0	3	0
0	0	5	0	1	1	0	0	0	0	8	0	0	1
9	0	0	0	0	0	0	0	0	0	3	3	13	0
0													
OTU4063	2	0	0	0	1	0	2	1	0	1	0	0	1
1	3	0	0	2	0	0	0	1	0	0	1	1	0
0	1	0	1	3	0	0	0	0	0	1	1	4	2
1	1	1	2	0	1	0	0	0	2	0	1	1	0
0	0	0	0	1	1	3	0	0	0	2	0	0	0
2	1	0	0	3	1	0	3	1	0	0	3	0	0
1	0	0	0	1	0	0	0	0	1	2	0	0	2
0	2	0	0	0	1	0	0	1	1	1	1	0	0
7													
OTU10964	1	0	1	0	0	0	0	0	0	0	0	0	0
0	0	0	0	0	0	0	0	0	0	0	0	0	0
0	0	0	0	0	0	0	0	0	0	0	0	0	0
0	0	0	0	0	0	0	0	0	0	0	0	0	0
0	0	0	0	0	0	0	0	0	0	0	0	0	0
0	0	0	0	0	0	0	0	0	0	0	0	0	0
0	0	0	0	0	0	0	0	0	0	0	0	0	0
0	0	0	0	0	0	0	0	0	0	0	0	0	0
0													

OTU3265	1	0	0	0	0	0	0	0	0	0	0	0	0
0	0	0	0	0	0	0	0	0	0	0	0	0	0
0	0	1	0	0	0	0	0	0	0	0	0	0	0
0	0	0	0	0	0	0	0	0	0	0	0	0	0
0	0	0	0	0	0	0	0	0	0	0	0	0	0
0	0	0	0	0	0	0	0	0	0	0	0	0	0
0	0	0	1	0	0	1	0	1	1	0	0	0	0
1	0	0	0	0	0	0	0	0	0	1	0	0	0
0													
OTU2776	1	0	0	0	0	0	0	0	0	0	1	0	0
0	0	0	0	0	0	0	0	0	0	0	0	0	3
0	0	0	0	0	0	0	0	0	0	0	0	1	0
0	0	0	0	0	0	0	0	0	0	0	0	0	0
0	0	0	0	0	0	0	0	0	0	0	0	0	0
0	0	0	0	0	0	0	0	0	0	0	0	0	0
0	0	0	0	0	0	0	0	0	0	0	0	0	0
1	0	0	0	0	0	0	0	0	0	0	0	0	0
0													
OTU12233	1	0	0	0	0	0	0	0	0	0	0	0	0
0	0	0	0	0	0	0	0	0	0	0	0	0	0
0	0	0	0	0	0	0	0	0	0	0	0	0	0
0	0	0	0	0	0	0	0	0	0	0	0	0	0
0	0	0	0	0	0	0	0	0	0	0	0	0	0
0	0	0	0	0	0	0	0	0	0	0	0	0	0
0	0	0	0	0	0	0	0	0	0	0	0	0	0
0	0	0	0	0	0	0	0	0	0	0	0	0	0
0													
OTU12232	1	0	0	0	0	0	0	0	0	0	0	0	0
0	0	0	0	0	0	0	0	0	0	0	0	0	0
0	0	0	0	0	0	0	0	0	0	0	0	0	0
0	0	0	0	0	0	0	0	0	0	0	0	0	0
0	0	0	0	0	0	0	0	0	0	0	0	0	0
0	0	0	0	0	0	0	0	0	0	0	0	0	0

0	0	0	0	0	0	0	0	0	0	0	0	0	0
0	0	0	0	0	0	0	0	0	0	0	0	0	0
0													
OTU5192	3	0	0	4	1	2	1	4	1	0	7	0	0
0	0	1	0	1	4	0	6	0	5	0	14	0	17
0	0	2	17	0	23	0	0	0	10	0	12	0	3
0	0	0	0	1	0	1	0	0	0	0	0	0	0
0	0	0	2	0	0	0	0	0	0	3	0	0	0
9	0	0	0	2	0	1	0	0	0	0	0	0	0
0	2	0	0	0	0	0	0	0	0	0	0	0	1
0	0	0	0	0	0	0	0	0	0	0	0	0	0
0													
OTU16293	2	1	2	3	6	3	2	2	3	7	1	3	1
2	2	4	1	9	5	2	3	0	7	1	1	3	3
3	0	5	3	0	2	0	0	2	3	2	3	11	2
4	1	2	2	1	1	0	0	2	6	0	3	2	3
1	0	2	3	1	3	0	1	2	5	5	4	2	1
2	3	2	2	8	2	1	7	0	0	2	11	0	0
4	1	0	2	2	5	2	1	0	3	3	3	0	0
0	2	3	1	2	2	1	2	1	0	3	2	1	2
7													
OTU8461	1	0	0	0	0	0	0	0	0	0	0	0	0
0	0	0	0	0	0	0	0	0	0	0	0	0	0
0	0	0	0	0	0	0	0	0	0	0	0	0	0
0	0	0	0	0	0	0	0	0	0	0	0	0	0
0	0	0	0	0	0	0	0	0	0	0	0	0	0
0	0	0	0	0	0	0	0	0	0	0	0	0	0
0	0	0	0	0	0	0	0	0	0	0	0	0	0
0	0	0	0	0	0	0	0	0	0	0	0	0	0
0													
OTU12231	1	0	0	0	0	0	0	0	0	0	0	0	0
0	0	0	0	0	0	0	0	0	0	0	0	0	0
0	0	0	0	0	0	0	0	0	0	0	0	0	0

0	0	0	0	0	0	0	0	0	0	0	0	0	0
0	0	0	0	0	0	0	0	0	0	0	0	0	0
0	0	0	0	0	0	0	0	0	0	0	0	0	0
0	0	0	0	0	0	0	0	0	0	0	0	0	0
0	0	0	0	0	0	0	0	0	0	0	0	0	0
0													
OTU4915	1	0	0	0	0	0	0	0	0	0	0	0	0
0	0	0	0	0	0	0	0	0	1	0	0	0	0
0	0	0	0	0	0	0	0	0	0	0	0	0	0
0	0	0	0	3	0	0	0	1	0	3	0	0	0
0	0	0	4	0	0	0	2	1	0	0	0	0	0
0	0	0	0	0	0	0	1	0	0	0	0	0	0
0	0	0	0	0	0	0	2	0	0	0	0	0	0
0	0	0	0	0	0	0	0	0	0	0	0	0	0
0													
OTU15860	1	4	2	12	3	3	6	4	0	4	7	1	0
0	0	11	0	8	0	0	2	0	2	0	1	0	1
0	0	0	1	0	6	0	0	0	4	0	2	0	0
0	0	1	0	0	0	0	1	0	0	5	0	0	2
0	20	0	5	0	0	0	0	0	0	0	0	1	0
0	0	1	0	0	1	0	0	0	4	1	1	0	6
0	1	0	1	0	6	0	6	0	0	0	0	4	0
1	0	0	0	0	0	0	0	0	0	1	0	0	0
0													
OTU86 1	0	2	10	0	1	1	0	1	0	1	3	8	1
3	10	66	3	6	1	10	2	0	0	0	1	3	0
28	57	11	25	0	120	5	0	1	0	0	2	2	6
10	169	22	54	22	1	2	112	3	18	6	667	3	58
1	230	6	5	150	295	71	17	186	119	4	21	20	25
0	0	10	15	16	8	0	4	1	1	3	3	10	7
19	12	0	2	2	8	0	26	1	1	0	0	111	2
0	0	0	2	0	3	0	0	0	1	1	13	0	0

OTU8570	2	3	0	9	14	0	2	1	2	0	3	0	0
0	0	5	0	8	9	0	3	0	29	1	17	0	0
0	0	14	9	0	5	0	0	0	3	0	15	0	3
0	0	2	0	1	0	0	0	2	0	5	0	1	0
0	6	0	1	1	1	0	0	1	0	1	0	2	2
5	0	5	0	3	0	0	0	0	3	0	0	0	1
0	2	0	1	0	0	0	0	0	0	0	0	0	1
0	0	0	0	0	1	0	0	0	0	0	0	0	0
0													
OTU1234	1	0	0	0	0	0	0	0	0	0	0	0	0
0	0	0	0	0	0	0	0	0	0	0	0	0	0
0	0	0	0	0	0	0	0	0	0	0	0	0	0
0	0	0	0	0	0	0	0	0	0	0	0	0	0
0	0	0	0	0	0	0	0	0	0	0	0	1	0
0	0	0	0	0	0	0	0	0	2	0	0	0	0
0	0	0	0	0	0	0	0	0	1	0	0	0	0
0	0	0	0	0	0	0	0	0	5	0	0	0	0
0													
OTU9390	1	0	0	0	0	0	0	0	0	0	0	0	0
0	0	0	0	0	0	0	0	0	0	0	0	0	0
0	0	1	0	0	0	0	0	0	0	0	0	0	0
0	0	0	0	0	0	0	0	0	0	0	0	0	0
0	0	0	0	0	0	0	0	0	0	0	0	0	0
0	0	0	0	0	0	0	0	0	0	0	0	0	0
0	0	0	0	0	0	0	0	0	0	0	0	0	0
0	0	0	0	0	0	0	0	0	0	0	0	0	0
0													
OTU8547	1	0	16	0	1	25	2	0	0	1	0	0	0
0	0	12	0	3	0	0	1	0	2	0	5	0	0
0	0	1	1	0	0	0	0	0	21	0	0	0	1
0	0	0	0	3	0	0	0	0	0	5	0	0	0
0	0	0	1	0	0	0	0	0	0	0	2	0	2
0	0	2	1	1	0	0	1	0	0	0	1	0	5

0 2 0 3 1 0 1 0 0 0 0 0 0 0
0 0 0 0 0 0 0 0 0 0 0 0 0 0
0
OTU8462 1 0 0 0 0 0 0 0 0 0 0 0 0
0 0 0 0 0 0 0 0 0 0 0 0 0 0
0 0 0 0 0 0 0 0 0 0 0 0 0 0
0 0 0 0 0 0 0 0 0 0 0 0 0 0
0 0 0 0 0 0 0 0 0 0 0 0 0 0
0 0 0 0 0 0 0 0 0 0 0 0 0 0
0 0 0 0 0 0 0 0 0 0 0 0 0 0
0 0 0 0 0 0 0 0 0 0 0 0 0 0
0
OTU10987 1 0 0 0 0 0 0 0 0 0 0 0 0
0 0 0 0 0 0 0 0 0 0 0 0 0 0
0 0 0 0 0 0 0 0 0 0 0 0 0 0
0 0 0 0 0 0 0 0 0 0 0 0 0 0
0 0 0 0 0 0 0 0 0 0 0 0 0 0
0 0 0 0 0 0 0 0 0 0 0 0 0 0
0 0 0 0 0 0 0 0 0 0 0 0 0 0
0 0 0 0 0 0 0 0 0 0 0 0 0 0
0
OTU3698 1 0 0 0 0 0 0 0 1 1 1 0 0
0 1 0 0 0 0 0 0 0 1 1 0 2 0
0 0 0 0 0 0 0 0 0 1 0 0 0 0
0 0 1 0 0 0 0 0 1 0 0 0 0 0
0 0 0 1 0 0 0 0 0 0 0 0 1 0
0 0 0 0 0 0 1 0 0 0 1 2 0 0
0 0 0 0 0 0 0 0 0 0 0 1 0 0
1 0 0 0 0 0 0 1 0 0 0 1 0 0
1
OTU5164 1 0 0 1 0 0 0 0 0 1 0 0 1
0 0 0 0 0 0 0 0 0 0 0 0 0 0
0 0 0 0 0 2 0 0 0 0 0 1 0 0

0 0 0 0 1 0 1 1 0 0 0 0 0 0
0 0 0 1 1 0 0 0 0 0 0 0 1 0
0 0 0 0 0 0 0 0 0 0 0 0 0 0
0 0 0 0 1 1 0 0 0 0 0 0 0 0
0 0 0 0 0 0 0 0 0 1 0 0 0 0
0
OTU7918 1 0 0 0 0 0 0 1 0 0 0 0 0
0 0 1 0 0 0 0 0 0 0 0 0 0 0
0 0 0 0 0 1 0 0 0 2 0 1 0 0
0 0 0 0 0 0 0 0 0 0 0 0 0 0
0 0 0 0 0 0 0 0 0 0 0 0 0 0
0 0 0 0 0 0 0 0 0 0 0 0 0 0
0 0 0 0 0 0 0 0 0 0 0 0 0 0
0 0 0 0 0 0 0 0 0 0 0 0 0 0
0
OTU8458 1 0 0 0 0 0 0 0 0 0 0 0 0
0 0 0 0 0 0 0 0 0 0 0 0 0 0
0 0 0 0 0 0 0 0 0 0 0 0 0 0
0 0 0 0 0 0 0 0 0 0 0 0 0 0
0 0 0 0 0 0 0 0 0 0 0 0 0 0
0 0 0 0 0 0 0 0 0 0 0 0 0 0
0 0 0 0 0 0 0 0 0 0 0 0 0 0
0 0 0 0 0 0 0 0 0 0 0 0 0 0
0
OTU10988 1 0 0 0 0 0 0 0 0 0 0 0 0
0 0 0 0 0 0 0 0 0 0 0 0 0 0
0 0 0 0 0 0 0 0 0 0 0 0 0 0
0 0 0 0 0 0 0 0 0 0 0 0 0 0
0 0 0 0 0 0 0 0 0 0 0 0 0 0
0 0 0 0 0 0 0 0 0 0 0 0 0 0
0 0 0 0 0 0 0 0 0 0 0 0 0 0
0 0 0 0 0 0 0 0 0 0 0 0 0 0
0

OTU447	1	12	0	0	0	0	2	0	1	7	0	1	0
0	1	0	0	0	0	0	0	0	0	0	0	0	0
0	0	0	0	0	0	0	0	0	0	0	0	0	0
1	0	0	0	0	0	0	0	0	0	2	0	0	0
0	0	0	1	0	0	0	0	0	0	0	0	0	0
0	0	3	0	2	0	0	0	0	6	0	0	0	1
0	0	0	11	0	3	0	2	0	0	0	0	0	0
0	0	0	0	0	0	0	0	0	1	0	0	0	0
0													
OTU6307	2	3	0	5	9	7	4	3	2	7	6	3	3
1	3	7	1	5	4	3	3	4	9	0	4	1	0
2	3	17	3	1	3	0	2	2	3	3	5	7	1
0	2	6	3	2	4	1	4	6	10	2	0	1	2
4	0	5	2	3	6	2	1	3	1	7	4	4	2
5	5	6	0	16	3	0	10	2	4	2	17	1	5
2	2	2	8	2	5	0	3	3	2	6	3	1	3
1	4	4	2	1	7	0	5	3	4	4	2	2	4
2													
OTU235	1	0	0	1	0	0	1	0	0	0	0	0	0
0	0	0	0	0	0	0	0	0	0	0	0	1	0
0	0	0	0	0	0	0	0	0	0	0	0	5	0
8	1	0	0	0	0	0	1	0	1	0	0	0	0
0	8	0	2	0	0	1	0	0	0	0	0	0	0
0	10	1	0	0	0	0	0	0	0	0	0	0	0
0	0	0	0	0	0	0	0	0	0	0	0	5	0
0	9	15	97	10	14	61	2	4	18	0	1	0	17
4													
OTU473	1	0	0	0	0	0	4	0	2	0	0	0	0
0	0	0	2	0	0	1	0	1	0	0	0	2	0
0	29	0	0	2	0	2	0	0	0	5	0	0	0
1	0	0	0	0	0	0	0	1	0	0	0	0	0
0	0	6	0	0	72	0	0	0	2	0	0	0	0
0	0	0	0	0	0	0	0	0	0	0	0	0	7

0 0 0 0 0 0 0 0 0 0 0 0 0 0
0 0 10 5 0 0 16 1 1 0 1 0 0 1
0
OTU14250 1 0 0 0 0 0 0 0 0 0 0 0 0
0 0 0 0 0 0 0 0 0 0 0 0 0 0
0 0 0 0 0 0 0 0 0 0 0 0 0 0
0 0 0 0 0 0 0 0 0 0 0 0 0 0
0 0 0 0 0 0 0 0 0 0 0 0 0 0
0 0 0 0 0 0 0 0 0 0 0 0 0 0
0 0 0 0 0 0 0 0 0 0 0 0 0 0
0 0 0 0 0 0 0 0 0 0 0 0 0 0
0
OTU12227 1 0 0 0 2 0 0 1 0 0 0 0 0
0 0 0 0 0 0 0 0 0 0 0 0 0 0
0 0 0 0 0 0 0 1 0 0 0 0 0 0
0 0 0 0 0 0 0 0 0 0 0 0 0 0
0 0 0 0 0 0 0 0 0 0 0 0 0 0
0 0 0 0 0 0 0 0 0 0 0 1 0 0
0 0 0 0 0 0 0 0 0 0 0 0 0 0
1 0 0 0 0 0 0 0 0 0 0 0 0 0
0
OTU8460 1 0 0 0 0 0 0 0 0 0 0 0 0
0 0 0 0 0 0 0 0 0 0 0 0 0 0
0 0 0 0 0 0 0 0 0 0 0 0 0 0
0 0 0 0 0 0 0 0 0 0 0 0 0 0
0 0 0 0 0 0 0 0 0 0 0 0 0 0
0 0 0 0 0 0 0 0 0 0 0 0 0 0
0 0 0 0 0 0 0 0 0 0 0 0 0 0
0 0 0 0 0 0 0 0 0 0 0 0 0 0
0
OTU13074 1 0 0 0 0 0 0 0 0 0 0 0 0
0 1 0 1 0 0 0 0 5 0 2 0 13 0
0 5 0 0 2 0 0 0 0 0 1 0 0 0

1 0 1 0 1 0 0 0 0 0 0 0 0 0
0 0 0 0 0 2 0 0 1 0 0 0 0 1
0 1 1 1 0 0 0 0 0 0 0 0 0 0
0 1 0 0 0 0 0 0 0 0 0 0 0 0
0 0 0 0 0 1 0 0 0 0 0 0 0 0
0
OTU10982 1 0 0 0 0 0 0 0 0 0 0 1 0
0 1 0 0 0 0 0 0 0 0 0 0 0 0
0 0 0 1 0 0 0 0 0 0 2 0 0 0
0 1 0 2 0 0 0 1 0 0 0 0 0 0
0 0 0 0 0 0 0 0 0 0 0 0 0 0
0 0 0 0 0 1 0 0 2 0 2 0 0 0
0 0 1 0 0 0 0 0 0 1 0 0 0 0
1 0 0 0 0 0 0 0 0 0 2 0 0 0
0
OTU3660 1 0 0 0 0 0 2 0 1 0 0 0 0
0 0 0 0 0 0 0 0 0 0 0 0 0 3
0 0 0 0 0 0 0 0 0 0 0 0 0 0
0 0 0 0 0 1 0 0 0 0 0 0 0 0
0 0 0 0 0 0 0 0 0 0 0 0 1 0
0 0 0 0 2 0 0 0 0 0 0 0 0 0
0 0 0 0 0 1 0 0 0 0 0 0 0 0
0 0 0 0 0 0 0 0 0 0 0 0 0 0
0
OTU13099 1 1 10 4 0 1 3 2 18 1 0 0 0
0 2 0 2 0 0 8 1 0 0 6 0 17 0
5 0 0 0 8 0 9 0 0 0 0 0 2 0
33 0 1 0 0 5 1 2 2 3 1 3 7 0
0 0 0 2 0 4 0 2 10 0 1 0 5 0
0 0 6 0 0 0 2 2 3 4 6 2 1 5
5 0 3 7 4 13 0 0 0 0 0 1 0 0
0 0 0 0 0 1 0 2 0 0 9 0 0 0
2

OTU2777	1	1	2	3	2	6	0	0	0	1	3	4	0
0	8	0	0	0	0	2	0	0	0	0	0	0	0
1	0	1	2	0	4	0	15	12	3	1	0	0	0
0	1	0	2	0	1	10	0	0	0	6	0	0	6
0	1	0	1	0	0	0	0	0	0	0	0	0	1
0	0	0	1	0	0	0	0	7	0	0	0	1	0
5	0	1	0	2	0	5	1	0	4	9	0	0	1
25	0	0	0	0	0	0	0	2	0	9	9	2	0
0													
OTU1531	1	0	0	0	0	0	1	0	1	0	0	1	0
0	0	0	0	0	0	0	0	0	0	0	0	3	0
1	0	0	0	1	0	0	0	0	0	0	0	0	0
2	0	0	0	0	1	0	2	0	0	0	0	0	0
0	0	0	0	0	0	0	0	0	0	0	0	0	0
0	0	2	0	0	0	0	6	0	0	0	1	0	0
0	0	0	0	0	0	0	0	0	0	0	0	0	0
0	0	0	0	0	0	0	0	0	0	0	0	0	0
0													
OTU14246	1	0	0	0	0	0	0	0	0	0	0	0	0
0	0	0	0	0	0	0	0	0	0	0	0	0	0
0	0	0	0	0	0	0	0	0	0	0	0	0	0
0	0	0	0	0	0	0	0	0	0	0	0	0	0
0	0	0	0	0	0	0	0	0	0	0	0	0	0
0	0	0	0	0	0	0	0	0	0	0	0	0	0
0	0	0	0	0	0	0	0	0	0	0	0	0	0
0	0	0	0	0	0	0	0	0	0	0	0	0	0
0													
OTU15646	1	0	0	0	0	0	1	0	0	1	0	0	0
0	1	0	0	1	0	0	0	1	0	0	0	0	1
0	0	0	0	1	0	0	0	0	0	0	0	2	0
0	0	1	0	0	0	0	0	1	1	0	0	0	0
0	0	1	0	1	0	1	0	0	1	0	0	0	0
2	0	0	0	0	0	0	1	0	1	0	0	0	0

0	0	1	0	0	0	0	0	0	0	0	1	0	0
0	1	0	0	0	0	0	0	0	0	0	0	0	0
3													
OTU733	1	0	0	3	0	0	0	0	0	0	0	0	0
0	0	0	0	0	0	0	8	0	0	0	1	1	0
0	0	0	0	1	4	2	0	0	0	0	0	0	0
0	0	0	0	0	0	0	0	0	0	0	0	0	0
0	0	2	0	1	0	0	0	0	1	0	0	0	0
0	0	0	0	1	1	0	0	0	1	1	0	2	0
0	0	0	0	0	0	2	0	0	0	0	0	0	0
0	1	0	0	0	0	0	0	1	1	0	0	0	1
0													
OTU1534	1	0	0	0	0	0	0	0	0	0	0	0	0
0	0	0	0	0	0	0	0	0	0	0	0	0	0
0	0	0	0	0	0	0	0	0	0	0	2	0	1
0	0	0	0	0	0	0	0	0	0	0	0	0	0
0	0	0	0	0	0	0	0	0	0	1	0	0	0
0	0	0	0	0	0	0	0	0	0	0	0	0	0
0	0	0	0	0	1	0	0	0	0	0	0	0	0
0	0	0	0	0	2	0	1	0	0	0	0	0	0
0													
OTU560	1	1	0	0	0	0	0	1	0	0	0	0	0
1	0	0	0	1	0	0	0	0	0	0	0	0	1
0	0	0	0	0	0	0	0	0	1	0	1	0	0
3	0	0	0	0	1	1	2	0	0	0	0	0	0
0	0	0	0	0	0	2	0	2	0	0	0	0	0
0	1	2	0	1	0	1	0	0	3	0	0	0	0
0	0	0	0	0	0	2	1	0	0	0	1	4	1
1	1	1	0	0	0	2	2	0	1	0	0	0	2
2													
OTU12228	1	0	0	0	0	0	0	0	0	0	0	0	0
0	0	0	0	0	0	0	0	0	0	0	0	0	0
0	0	0	0	0	0	0	0	0	0	0	0	0	0

0 0 0 0 0 0 0 0 0 0 0 0 0 0
0 0 0 0 0 0 0 0 0 0 0 0 0 0
0 0 0 0 0 0 0 0 0 0 0 0 0 0
0 0 0 0 0 0 0 0 0 0 0 0 0 0
0 0 0 0 0 0 0 0 0 0 0 0 0 0
0
OTU4567 1 0 0 0 0 0 0 0 0 0 0 0 0
0 0 0 0 1 0 0 0 0 0 0 0 0 0
0 0 0 0 0 0 0 0 0 0 0 0 0 2
1 0 0 0 0 0 1 0 0 0 0 0 0 0
0 0 0 0 0 0 0 0 0 0 0 0 0 0
0 0 0 0 0 0 0 0 0 0 0 0 0 0
0 0 0 0 0 0 0 0 0 0 0 0 0 0
0 0 0 0 0 0 0 0 0 0 0 0 0 0
0
OTU6054 1 0 0 0 0 0 0 0 0 0 0 0 0
0 0 0 0 0 0 0 0 0 0 0 0 0 0
0 0 0 0 0 0 0 0 0 0 0 0 0 0
0 0 0 0 0 0 0 0 0 0 0 0 0 0
0 0 0 0 0 0 0 0 0 0 0 0 0 0
0 0 1 0 0 0 0 0 0 0 0 0 0 0
0 0 0 0 0 0 0 1 0 0 0 0 0 0
0 0 0 0 0 0 0 0 0 0 0 0 0 0
0
OTU1633 1 2 11 0 0 0 0 0 0 0 0 0 0
0 0 0 0 0 0 0 1 0 0 0 0 0 1
0 0 0 0 0 0 0 0 0 0 0 1 0 2
0 0 0 0 1 0 0 0 0 0 0 0 0 0
0 0 0 0 0 0 0 0 0 0 0 0 0 0
0 0 0 0 0 0 0 0 0 0 0 0 0 0
0 0 0 0 0 0 0 0 0 0 0 0 0 0
0 0 0 0 0 10 0 0 0 1 0 0 0 0
0

OTU10985	1	0	0	0	0	0	0	0	0	0	0	0	0
0	0	0	0	0	0	1	0	0	0	0	0	1	0
0	0	0	0	1	0	0	0	0	0	0	0	0	0
0	0	0	0	0	0	0	0	0	0	0	0	0	0
0	0	0	0	0	0	0	0	0	0	0	0	0	0
0	0	0	0	0	0	0	0	0	0	0	0	0	0
0	0	0	0	0	0	0	0	0	0	0	0	0	0
0	0	0	0	0	0	0	0	0	0	0	0	0	0
0													
OTU9887	1	1	0	1	4	3	0	1	1	1	0	0	1
0	2	1	0	2	1	1	3	3	0	0	1	0	1
1	0	2	1	0	2	0	1	2	0	0	1	5	1
0	0	1	1	1	1	0	0	3	0	0	1	0	0
1	0	0	3	0	0	0	2	1	0	0	0	0	0
2	0	0	0	2	0	1	0	1	0	0	2	1	1
0	0	1	1	1	3	0	0	0	1	1	0	1	1
0	0	0	0	1	1	1	0	0	2	0	0	0	0
1													
OTU12229	1	0	0	0	0	0	0	0	0	0	0	0	0
0	0	0	0	0	0	0	0	0	0	0	0	0	0
0	0	0	0	0	0	0	0	0	0	0	0	0	0
0	0	0	0	0	0	0	0	0	0	0	0	0	0
0	0	0	0	0	0	0	0	0	0	0	0	0	0
0	0	0	0	0	0	0	0	0	0	0	0	0	0
0	0	0	0	0	0	0	0	0	0	0	0	0	0
0	0	0	0	0	0	0	0	0	0	0	0	0	0
0													
OTU4584	1	2	6	6	13	7	4	3	3	6	4	3	3
4	11	4	2	8	2	5	2	3	3	4	1	4	4
0	3	7	3	6	4	6	4	4	1	4	5	8	3
10	4	4	6	3	2	2	7	10	9	0	2	2	3
6	5	3	8	5	3	3	5	10	5	8	2	4	4
5	8	6	2	7	5	7	8	3	2	1	17	2	7

6	8	3	5	1	6	2	3	1	9	3	3	6	2
2	6	3	4	2	8	3	7	2	1	5	1	4	6
11													
OTU8466	2	0	0	0	0	0	0	0	0	0	0	0	0
0	0	0	0	0	0	0	0	0	0	0	0	0	0
0	0	0	0	0	0	0	0	0	0	0	0	0	0
0	0	0	0	0	0	0	0	0	0	0	0	0	0
0	0	0	0	0	0	0	0	0	0	0	0	0	0
0	0	0	0	0	0	0	0	0	0	0	0	0	0
0	0	0	0	0	0	0	0	0	0	0	0	0	0
0	0	0	0	0	0	0	0	0	0	0	0	0	0
0													
OTU11620	1	0	2	0	1	2	0	4	2	2	1	2	0
0	1	1	0	4	0	0	2	3	0	0	2	1	1
2	0	1	2	0	2	0	1	1	2	1	2	2	2
1	1	1	0	1	0	0	1	3	3	0	0	0	1
1	1	2	4	1	2	1	0	1	0	4	1	2	0
2	1	2	0	2	1	2	3	1	1	0	6	0	0
0	0	0	0	0	6	2	0	2	0	1	0	0	0
1	1	0	0	3	1	1	0	1	0	1	0	0	0
4													
OTU12230	1	0	0	0	0	0	0	0	0	0	0	0	0
0	0	0	0	0	0	0	0	0	0	0	0	0	0
0	0	0	0	0	0	0	0	0	0	0	0	0	0
0	0	0	0	0	0	0	0	0	0	0	0	0	0
0	0	0	0	0	0	0	0	0	0	0	0	0	0
0	0	0	0	0	0	0	0	0	0	0	0	0	0
0	0	0	0	0	0	0	0	0	0	0	0	0	0
0	0	0	0	0	0	0	0	0	0	0	0	0	0
0													
OTU14683	1	0	0	2	4	0	0	4	0	0	0	0	0
0	0	0	0	0	0	0	3	0	0	1	0	2	1
0	0	0	1	0	0	4	1	0	0	0	0	0	0

	0	0	1	0	0	1	0	1	0	0	0	0	0	0
	0	0	0	2	1	0	0	0	2	0	0	2	0	1
	1	0	0	1	0	0	0	0	0	0	0	0	1	0
	0	0	2	0	2	0	1	0	1	0	0	0	0	1
	2	0	0	0	0	0	0	0	0	0	0	0	1	0
	0													
OTU232		2	4	0	0	0	10	2	1	5	1	1	6	3
	10	0	20	0	1	4	3	26	0	0	2	0	4	13
	7	1	0	4	0	3	10	10	2	10	1	2	2	3
	4	12	1	1	4	1	2	3	1	0	3	2	1	2
	0	0	1	6	2	1	0	1	0	0	0	0	7	0
	0	0	18	1	5	0	0	0	5	6	3	0	4	4
	7	0	2	3	4	8	1	8	0	2	8	0	0	8
	21	0	0	0	0	0	0	0	0	0	10	9	2	1
	0													
OTU1087		1	0	0	0	0	0	1	0	0	0	0	1	0
	1	0	0	0	0	0	0	0	0	0	0	0	0	0
	0	0	0	0	0	2	0	0	0	1	0	0	0	0
	0	1	0	0	1	0	0	0	0	0	0	0	0	0
	0	0	0	1	0	0	1	0	0	0	0	1	0	0
	0	0	1	1	2	1	0	0	1	0	0	0	0	0
	0	0	0	0	0	0	0	0	0	0	0	0	0	1
	0	0	0	0	0	0	0	0	0	0	0	0	0	0
	0													
OTU10986		1	0	0	0	0	0	0	0	0	0	0	0	0
	0	0	0	0	0	0	0	0	0	0	0	0	0	0
	0	0	0	0	0	0	0	0	0	0	0	0	0	0
	0	0	0	0	0	0	0	0	0	0	0	0	0	0
	0	0	0	0	0	0	0	0	0	0	0	0	0	0
	0	0	0	0	0	0	0	0	0	0	0	0	0	0
	0	0	0	0	0	0	0	0	0	0	0	0	0	0
	0	0	0	0	0	0	0	0	0	0	0	0	0	0
	0													

OTU10984	1	0	0	0	0	0	0	0	0	0	0	0	0
0	0	0	0	0	0	0	0	0	0	0	0	0	0
0	0	0	0	0	0	0	0	0	0	0	0	0	0
0	0	0	0	0	0	0	0	0	0	0	0	0	0
0	0	0	0	0	0	0	0	0	0	0	0	0	0
0	0	0	0	0	0	0	0	0	0	0	0	0	0
0	0	0	0	0	0	0	0	0	0	0	0	0	0
0	0	0	0	0	0	0	0	0	0	0	0	0	0
0													
OTU13019	1	0	0	0	0	0	0	0	0	0	0	0	0
0	0	0	0	0	0	1	0	0	0	0	0	0	0
0	0	0	0	0	0	0	1	1	0	1	0	0	0
0	0	0	0	0	2	0	0	0	0	1	0	0	0
0	0	0	0	0	0	0	0	0	0	0	0	0	0
0	0	0	0	1	0	0	0	0	0	0	0	0	0
0	0	0	1	0	0	0	0	0	0	0	0	0	0
0	0	0	0	0	0	0	0	0	0	0	1	0	0
0													
OTU1501	1	0	0	0	0	0	1	0	0	0	0	22	1
3	0	15	0	4	0	1	30	0	0	2	7	0	4
2	0	0	1	1	0	0	0	0	18	1	0	2	1
1	27	0	0	1	0	1	0	0	1	0	0	0	0
0	0	0	0	0	0	0	0	0	0	0	1	0	0
0	0	0	13	0	1	0	0	0	0	0	0	4	0
7	0	0	0	17	0	0	0	0	24	0	0	0	0
33	0	1	0	0	0	0	0	0	1	8	0	6	0
0													
OTU14249	1	0	0	0	0	0	0	0	0	0	0	0	0
0	0	0	0	0	0	0	0	0	0	0	0	0	0
0	0	0	0	0	0	0	0	0	0	0	0	0	0
0	0	0	0	0	0	0	0	0	0	0	0	0	0
0	0	0	0	0	0	0	0	0	0	0	0	0	0
0	0	0	0	0	0	0	0	0	0	0	0	0	0

0	0	0	0	0	0	0	0	0	0	0	0	0	0
0	0	0	0	0	0	0	0	0	0	0	0	0	0
0													
OTU14248	1	0	0	0	0	0	0	0	0	0	0	0	0
0	0	0	0	0	0	0	0	0	0	0	0	0	0
0	0	0	0	0	0	0	0	0	0	0	0	0	0
0	0	0	0	0	0	0	0	0	0	0	0	0	0
0	0	0	0	0	0	0	0	0	0	0	0	0	0
0	0	0	0	0	0	0	0	0	0	0	0	0	0
0	0	0	0	0	0	0	0	0	0	0	0	0	0
0	0	0	0	0	0	0	0	0	0	0	0	0	0
0													
OTU619	1	0	0	0	0	0	1	0	1	4	0	0	0
0	0	0	0	0	0	0	0	0	0	0	0	0	1
1	0	0	0	0	0	0	0	0	1	0	0	1	1
0	0	0	0	0	1	0	0	0	0	0	0	0	0
0	0	0	0	0	0	0	0	0	0	0	0	0	0
0	0	0	0	12	0	0	0	0	0	0	0	0	0
0	0	0	1	0	0	0	19	0	0	0	0	1	0
0	2	0	0	4	0	0	5	6	1	0	0	0	19
0													
OTU15723	1	1	3	0	2	1	2	1	1	1	3	1	2
1	12	1	1	1	1	0	2	5	0	1	0	2	0
0	1	3	0	1	1	1	3	1	1	0	2	7	0
5	3	3	4	0	0	0	0	5	5	0	0	1	2
1	0	2	1	0	3	1	1	2	3	0	0	1	1
6	5	0	0	2	0	0	3	1	1	0	3	1	0
3	2	0	0	2	3	2	0	0	0	2	3	1	2
2	4	0	1	2	1	2	1	0	1	1	1	1	1
14													
OTU6353	1	2	27	0	0	0	0	0	0	0	0	0	0
0	0	0	0	0	0	0	0	0	0	0	0	0	0
0	0	0	0	0	0	0	0	0	0	0	2	0	2

0 0 1 0 4 0 0 0 0 0 0 0 0 0
0 0 0 0 0 0 0 0 0 0 0 0 0 0
1 0 0 0 0 0 0 0 0 0 0 0 0 0
0 1 0 0 0 0 0 0 0 0 0 0 0 0
0 0 0 0 0 1 0 0 0 2 0 0 0 0
0
OTU11659 1 0 1 0 0 0 0 0 0 0 0 0 1
0 0 0 0 0 5 2 0 0 0 0 0 0 0
1 0 0 0 0 0 0 0 0 0 0 0 0 0
3 14 0 41 0 0 0 0 0 0 0 0 0 0
0 0 0 0 0 0 0 0 0 0 0 0 1 0
0 4 1 0 2 10 0 0 0 3 6 0 1 1
0 0 0 0 8 7 0 0 0 1 0 4 1 0
0 1 0 0 0 0 0 0 0 0 2 0 0 0
0
OTU88 1 0 6 307 368 0 2 0 12 0 1 0 0 1
26 0 19 0 1 2 0 12 0 197 0 1 0 0
4 20 0 70 0 0 3 0 0 0 0 0 21 0
5 106 1 4 0 4 4 0 0 13 0 2 19 0
92 26 7 22 0 14 0 0 43 7 1 0 78 8
0 0 1 17 37 3 0 0 7 3 1 1 1 3
0 0 0 0 0 0 0 0 0 12 2 1 0 0
0 0 3 1 0 1 3 5 0 0 129 3 0 0
OTU10983 1 0 0 0 0 0 0 0 0 0 0 0 0
0 0 0 0 0 0 0 0 0 0 0 0 0 0
0 0 0 0 0 0 0 0 0 0 0 0 0 0
0 0 0 0 0 0 0 0 0 0 0 0 0 0
0 0 0 0 0 0 0 0 0 0 0 0 0 0
0 0 0 0 0 0 0 0 0 0 0 0 0 0
0 0 0 0 0 0 0 0 0 0 0 0 0 0
0 0 0 0 0 0 0 0 0 0 0 0 0 0
0

OTU8467 1 0 0 0 0 0 0 0 0 0 0 0 0
0 0 0 0 0 0 0 0 0 0 0 0 0 0
0 0 0 0 0 0 0 0 0 0 0 0 0 0
0 0 0 0 0 0 0 0 0 0 0 0 0 0
0 0 0 0 0 0 0 0 0 0 0 0 0 0
0 0 0 0 0 0 0 0 0 0 0 0 0 0
0 0 0 0 0 0 0 0 0 0 0 0 0 0
0 0 0 0 0 0 0 0 0 0 0 0 0 0
0
OTU14247 1 0 0 0 0 0 0 0 0 0 0 0 0
0 0 0 0 0 0 0 0 0 0 0 0 0 0
0 0 0 0 0 0 0 0 0 0 0 0 0 0
0 0 0 0 0 0 0 0 0 0 0 0 0 0
0 0 0 0 0 0 0 0 0 0 0 0 0 0
0 0 0 0 0 0 0 0 0 0 0 0 0 0
0 0 0 0 0 0 0 0 0 0 0 0 0 0
0 0 0 0 0 0 0 0 0 0 0 0 0 0
0
OTU13677 1 0 1 0 0 1 2 0 1 0 0 0 0
0 0 0 1 1 2 0 0 0 2 0 2 0 0
0 0 3 1 0 0 0 0 0 0 0 0 0 1
0 0 0 0 0 0 1 0 0 0 0 0 0 0
1 0 0 0 0 0 1 1 1 2 1 0 0 0
2 0 1 0 2 0 0 0 0 0 0 1 0 1
0 0 0 0 0 1 0 1 0 0 1 0 1 0
0 0 0 0 0 0 0 1 0 0 0 0 0 0
0
OTU6826 1 0 0 0 0 0 0 0 0 0 0 0 0
0 1 0 0 0 0 0 0 0 0 0 0 1 2
0 0 0 0 0 0 0 0 0 0 0 0 0 0
0 0 0 0 0 0 0 0 0 0 0 0 0 0
0 0 0 0 0 0 0 0 0 0 0 0 0 0
0 0 0 0 0 0 0 0 0 0 0 0 0 0

0 0 0 0 0 1 0 0 0 0 0 0 0 0
0 0 0 1 0 0 0 0 0 0 0 0 0 0
0
OTU1263 1 0 1 0 0 0 0 1 0 1 0 2 0
0 0 0 0 0 0 0 1 0 0 0 0 0 1
0 0 0 1 0 0 0 0 0 0 0 0 0 0
1 0 0 0 0 0 0 0 0 0 0 0 0 0
0 0 0 0 0 0 0 0 0 0 0 0 0 0
0 0 1 0 1 3 0 0 0 1 1 0 1 1
0 0 0 0 0 0 0 0 0 0 0 0 0 0
0 0 0 0 0 0 0 0 0 0 0 0 0 0
0
OTU100 1 4 0 1 1 2 0 3 9 1 0 1 0
0 0 0 0 1 0 9 1 2 0 1 16 5 0
17 2 1 3 1 0 1 0 0 1 281 0 5 0
4 5 0 14 3 0 0 11 18 3 5 9 4 0
4 0 1 1 3 10 1 10 2 0 3 1 3 0
2 228 0 0 0 0 4 7 0 1 7 47 0 7
0 0 0 6 2 0 5 1 0 2 0 64 57 0
0 205 259 110 58 143 33 37 111 105 1 3 1 111
137
OTU7379 1 1 1 0 1 0 0 0 0 1 0 0 1
0 0 0 0 2 1 1 0 0 0 0 1 0 0
1 0 2 1 0 0 0 1 0 0 0 1 1 0
1 0 3 0 1 1 0 0 0 2 0 1 1 2
1 0 0 1 0 1 0 0 0 1 1 1 0 0
1 0 1 0 2 0 0 1 0 0 0 3 0 1
0 0 0 1 0 0 0 0 0 0 0 0 0 1
0 1 1 0 0 1 0 1 0 1 0 1 0 0
0
OTU13624 1 2 18 0 0 0 3 3 3 6 0 0 0
0 2 0 0 0 0 26 0 0 0 0 0 0 0
3 0 0 0 0 0 0 0 0 0 0 0 0 0

2	0	0	0	0	0	0	0	0	0	0	3	0	0
0	0	0	0	0	0	0	0	0	0	0	0	0	0
0	0	0	0	5	0	0	0	0	8	0	10	0	1
0	1	0	0	0	6	0	2	0	0	0	0	0	0
0	0	0	0	0	0	0	0	0	0	0	0	0	0
0													
OTU651	1	4	7	0	1	33	22	23	23	27	0	0	0
0	1	0	3	0	0	48	0	15	0	0	0	8	0
7	0	0	0	3	0	2	0	2	0	0	0	16	0
7	0	0	1	0	3	0	2	0	3	0	11	0	0
0	0	0	4	0	2	0	4	1	0	1	0	31	0
1	1	31	0	6	0	0	1	0	1	0	8	0	41
0	5	0	2	0	19	13	74	0	0	0	2	1	0
0	1	2	2	1	1	3	7	1	1	0	0	0	2
2													
OTU6262	1	0	0	3	2	1	0	2	0	0	1	1	2
0	2	1	0	3	0	1	0	0	0	1	0	1	0
3	0	0	0	1	1	0	0	0	2	1	1	0	0
1	1	0	0	1	1	0	0	0	6	1	0	2	0
0	1	1	1	0	1	0	1	1	0	1	0	1	0
1	1	1	2	0	1	0	3	0	2	0	4	0	0
0	1	0	0	0	1	1	1	1	0	0	0	1	0
0	4	0	0	0	1	0	1	1	2	0	0	0	0
2													
OTU212	2	4	0	4	0	7	7	7	8	2	0	0	1
2	4	1	45	0	0	15	2	27	0	3	1	45	0
15	9	0	1	10	0	16	4	8	0	32	0	6	0
12	3	0	0	19	17	0	4	1	9	10	3	1	0
1	5	12	9	8	6	4	10	10	6	17	4	12	5
16	1	4	4	2	3	0	23	0	4	2	11	2	8
0	36	5	6	0	2	2	14	0	0	1	2	4	2
2	5	12	3	10	9	4	1	7	4	4	1	8	0
2													

OTU10994	1	0	0	0	0	0	0	0	0	0	0	0	0
0	0	0	0	0	0	0	0	0	0	0	0	0	0
0	0	0	0	0	0	0	0	0	0	0	0	0	0
0	0	0	0	0	0	0	0	0	0	0	0	0	0
0	0	0	0	0	0	0	0	0	0	0	0	0	0
0	0	0	0	0	0	0	0	0	0	0	0	0	0
0	0	0	0	0	0	0	0	0	0	0	0	0	0
0	0	0	0	0	0	0	0	0	0	0	0	0	0
0													
OTU3200	1	1	1	0	0	1	0	5	1	0	0	0	0
0	0	0	0	1	0	3	0	0	0	0	0	0	3
0	0	0	8	0	1	1	0	0	1	0	0	0	0
0	0	0	0	0	0	0	0	0	0	0	0	0	0
0	0	0	0	0	0	0	0	0	0	0	0	0	0
0	0	1	0	0	0	5	0	0	0	0	1	0	0
0	0	0	0	0	0	0	1	0	0	0	0	0	0
1	0	0	0	0	0	0	0	0	0	0	0	0	0
0													
OTU10995	1	0	0	0	0	0	0	0	0	0	0	0	0
0	0	0	0	0	0	0	0	0	0	0	0	0	0
0	0	0	0	0	0	0	0	0	0	0	0	0	0
0	0	0	0	0	0	0	0	0	0	0	0	0	0
0	0	0	0	0	0	0	0	0	0	0	0	0	0
0	0	0	0	0	0	0	0	0	0	0	0	0	0
0	0	0	0	0	0	0	0	0	0	0	0	0	0
0	0	0	0	0	0	0	0	0	0	0	0	0	0
0													
OTU1194	1	0	0	0	0	0	0	2	1	0	0	0	0
0	1	0	1	0	0	2	5	0	0	0	1	1	0
1	0	0	0	0	0	1	0	0	0	0	0	2	0
0	0	0	0	0	0	0	0	0	0	1	0	0	0
0	1	0	0	0	0	0	0	1	0	0	0	2	0
0	0	0	0	2	1	0	1	0	0	0	0	0	0

0	0	0	0	0	0	0	0	0	0	0	0	0	0
0	0	0	0	0	0	0	0	0	1	2	0	0	0
0													
OTU14151	1	0	1	1	1	21	3	0	0	10	0	4	0
0	0	0	0	0	0	0	1	0	0	0	0	0	0
1	0	0	0	0	0	0	0	2	0	0	0	2	0
1	1	0	0	0	4	0	8	0	0	0	0	0	5
0	2	0	1	0	0	0	0	0	0	0	0	2	0
0	0	14	0	2	1	0	0	1	3	0	0	0	0
0	0	0	1	0	6	0	3	0	0	0	0	0	0
0	0	0	1	0	0	1	0	0	0	0	0	0	0
0													
OTU12235	1	0	0	0	0	0	0	0	0	0	0	0	0
0	0	0	0	0	0	0	0	0	0	0	0	0	0
0	0	0	0	0	0	0	0	0	0	0	0	0	0
0	0	0	0	0	0	0	0	0	0	0	0	0	0
0	0	0	0	0	0	0	0	0	0	0	0	0	0
0	0	0	0	0	0	0	0	0	0	0	0	0	0
0	0	0	0	0	0	0	0	0	0	0	0	0	0
0	0	0	0	0	0	0	0	0	0	0	0	0	0
0													
OTU4028	1	0	0	0	0	0	0	0	0	0	0	0	0
0	0	0	0	0	0	0	0	2	0	0	0	0	0
0	0	0	0	15	0	0	0	0	0	1	0	0	0
0	0	0	0	0	0	0	0	0	0	0	0	0	0
0	0	0	0	0	0	0	0	0	0	0	0	0	0
0	0	0	0	0	0	0	0	0	0	0	0	0	0
0	0	0	0	0	0	0	0	0	0	0	2	0	0
0	2	0	0	0	0	0	0	0	0	0	0	0	4
0													
OTU11511	1	1	0	1	1	0	0	0	2	0	0	0	1
0	0	1	0	1	1	0	1	0	0	0	0	0	0
0	1	0	2	1	0	1	1	2	1	0	0	0	0

0	0	0	1	0	1	0	0	1	1	0	0	0	0
1	0	0	0	0	1	0	0	1	0	3	0	0	0
1	0	1	0	3	1	0	0	0	0	0	1	0	0
0	0	0	0	1	0	0	0	1	0	2	1	0	0
1	0	1	0	2	2	0	0	1	0	0	0	0	0
0													
OTU10993	1	0	0	0	0	0	0	0	0	0	0	0	0
0	0	0	0	0	0	0	0	0	0	0	0	0	0
0	0	0	0	0	0	0	0	0	0	0	0	0	0
0	0	0	0	0	0	0	0	0	0	0	0	0	0
0	0	0	0	0	0	0	0	0	0	0	0	0	0
0	0	0	0	0	0	0	0	0	0	0	0	0	0
0	0	0	0	0	0	0	0	0	0	0	0	0	0
0	0	0	0	0	0	0	0	0	0	0	0	0	0
0													
OTU4764	1	7	0	0	2	0	9	7	2	0	0	0	1
0	3	0	22	0	0	0	2	32	0	1	0	6	0
2	44	0	0	7	0	2	3	0	0	7	0	7	0
1	0	12	0	0	0	0	14	181	28	5	30	70	0
68	0	0	2	5	131	1	106	99	4	225	0	3	1
82	0	2	0	0	0	0	14	0	1	0	9	0	6
0	31	0	0	1	0	0	1	0	0	0	0	0	0
0	0	0	0	0	0	1	0	0	0	0	0	0	0
1													
OTU12236	1	0	0	0	0	0	0	0	0	0	0	0	0
0	0	0	0	0	0	0	0	0	0	0	0	0	0
0	0	0	0	0	0	0	0	0	0	0	0	0	0
0	0	0	0	0	0	0	0	0	0	0	0	0	0
0	0	0	0	0	0	0	0	0	0	0	0	0	0
0	0	0	0	0	0	0	0	0	0	0	0	0	0
0	0	0	0	0	0	0	0	0	0	0	0	0	0
0	0	0	0	0	0	0	0	0	0	0	0	0	0
0													

OTU1289	1	2	1	0	0	0	0	0	1	0	0	3	0
1	1	0	0	0	0	0	0	0	0	0	1	2	1
0	0	0	0	0	1	0	0	0	3	14	0	0	0
1	1	0	1	0	2	0	1	0	0	0	0	0	0
0	4	0	1	0	0	1	0	0	0	0	1	1	1
0	1	2	1	1	2	0	0	0	0	1	0	1	0
1	0	0	2	1	0	0	1	0	4	1	0	1	0
0	0	1	2	0	0	0	0	0	0	1	3	0	0
0													
OTU446	1	0	2	0	0	0	1	0	0	1	0	0	0
0	0	0	0	0	0	1	0	0	0	0	0	0	0
2	0	0	0	0	0	0	0	0	0	0	0	11	0
2	2	0	0	0	0	0	0	0	1	0	3	0	0
0	0	0	3	0	0	0	0	1	0	0	0	0	0
0	1	0	0	0	0	0	0	0	0	0	7	0	0
0	0	0	4	0	2	0	1	0	0	0	2	1	0
0	3	10	1	4	2	1	3	5	5	0	0	0	9
1													
OTU5286	1	0	0	1	1	0	0	0	0	1	0	1	0
0	0	0	0	1	0	0	0	0	0	0	0	0	0
0	0	0	1	0	0	0	0	0	0	1	0	0	1
0	1	0	1	0	0	0	0	0	1	0	0	0	0
0	0	1	0	0	0	0	0	0	0	0	0	0	0
3	0	0	0	1	0	0	3	0	0	2	1	0	1
0	0	0	1	0	1	0	0	0	1	0	1	0	1
0	0	0	0	0	1	0	0	0	1	0	0	0	0
2													
OTU439	1	1	0	0	0	1	0	0	1	0	0	0	0
0	0	0	3	0	0	1	0	4	0	2	0	1	0
0	2	0	2	2	0	3	0	0	0	46	0	1	0
0	0	1	1	10	0	0	0	0	0	0	0	0	0
6	0	0	0	8	9	0	0	0	4	0	1	0	0
7	5	0	0	0	2	0	2	0	0	0	1	0	0

0	0	0	0	2	0	2	0	0	0	0	1	0	0
0	4	8	8	8	19	11	0	3	1	1	0	0	25
2													
OTU163	1	2	37	0	1	1	0	0	4	2	0	1	0
0	0	2	0	0	0	0	0	0	0	0	0	5	0
0	2	0	1	0	0	2	0	0	1	0	0	0	0
5	0	0	0	0	0	1	0	91	3	5	43	0	0
0	1	0	9	1	27	2	3	2	0	0	0	3	1
13	1	0	2	0	13	36	0	3	6	1	43	0	1
1	271	1	13	0	0	1	0	0	0	0	0	0	0
0	7	5	0	28	26	15	11	4	3	0	0	0	24
5													
OTU12237	1	0	0	0	0	0	0	0	0	0	0	0	0
0	0	0	0	0	0	0	0	1	0	0	0	0	0
0	0	0	0	0	0	0	0	0	0	0	0	0	0
0	0	0	0	0	0	0	0	0	0	0	0	0	0
0	0	0	0	1	0	0	0	0	0	0	0	0	1
0	0	0	0	0	0	0	0	0	0	0	0	0	0
0	0	0	1	0	0	0	0	0	0	0	0	0	0
0	0	1	0	0	0	0	0	0	0	0	0	0	0
0													
OTU12238	1	0	0	0	1	0	0	0	1	2	1	1	0
0	1	0	0	0	0	0	1	0	2	1	0	0	0
0	0	0	1	0	1	0	0	0	0	0	0	0	0
0	1	0	1	0	0	0	0	0	2	0	1	0	0
0	0	0	0	1	0	0	1	1	0	1	0	1	1
1	0	0	0	1	0	0	1	0	0	0	0	0	0
1	1	0	0	0	0	0	1	1	0	0	0	0	0
0	0	0	0	0	2	0	0	0	0	0	0	0	0
2													
OTU309	1	4	2	3	0	3	3	20	1	1	1	1	0
1	3	1	0	10	0	8	2	0	1	0	1	0	0
4	0	1	2	0	0	0	0	0	3	0	0	2	0

1 0 0 1 5 3 0 1 0 0 0 5 1 0
0 1 2 2 1 0 2 0 1 0 0 0 2 0
0 0 0 2 1 4 0 0 1 9 3 2 0 1
0 0 0 5 1 0 2 5 0 0 0 0 1 0
1 1 0 2 2 0 0 1 0 3 1 0 0 3
0
OTU14236 1 0 0 0 0 0 0 0 0 0 0 0 0
0 0 0 0 0 0 0 0 0 0 0 0 0 0
0 0 0 0 0 0 0 0 0 0 0 0 0 0
0 0 0 0 0 0 0 0 0 0 0 0 0 0
0 0 0 0 0 0 0 0 0 0 0 0 0 0
0 0 0 0 0 0 0 0 0 0 0 0 0 0
0 0 0 0 0 0 0 0 0 0 0 0 0 0
0 0 0 0 0 0 0 0 0 0 0 0 0 0
0
OTU437 1 0 1 0 0 1 1 1 5 0 0 0 0
0 0 0 0 0 0 0 0 7 0 1 0 0 0
0 4 0 0 0 0 0 0 0 0 0 1 0 0
3 0 0 0 0 0 0 1 0 0 0 0 12 0
0 1 0 1 0 0 0 6 18 0 0 0 0 0
0 2 3 0 0 0 0 42 0 6 0 24 0 8
0 12 0 3 0 0 0 2 0 0 0 8 0 0
0 5 12 15 33 41 73 5 14 14 0 0 0 21
5
OTU585 1 0 0 0 0 0 0 0 0 0 0 0 0
0 0 0 0 0 0 0 0 0 0 0 0 0 0
0 0 0 0 0 0 0 0 0 0 0 0 0 0
0 0 0 0 1 0 0 0 0 0 0 0 0 0
18 0 1 0 13 18 0 2 0 0 1 0 0 4
0 0 0 0 0 0 0 2 0 0 0 0 0 0
0 0 0 0 0 0 0 0 0 0 0 0 0 0
0 0 19 2 3 0 0 0 8 1 0 0 0 3
0

OTU8454	1	0	0	0	0	0	0	0	0	0	0	0	0
0	0	0	0	0	0	0	0	0	0	0	0	0	0
0	0	0	0	0	0	0	0	0	0	0	0	0	0
0	0	0	0	0	0	0	0	0	0	0	0	0	0
0	0	0	0	0	0	0	0	0	0	0	0	0	0
0	0	0	0	0	0	0	0	0	0	0	0	0	0
0	0	0	0	0	0	0	0	0	0	0	0	0	0
0	0	0	0	0	0	0	0	0	0	0	0	0	0
0													
OTU1102	1	0	0	0	0	0	0	0	0	0	0	0	0
0	0	0	0	0	0	0	0	0	0	0	0	1	0
0	0	0	0	0	0	0	0	0	0	0	0	8	0
1	0	0	0	0	0	0	0	0	0	0	0	0	0
0	0	0	0	0	0	0	0	0	0	0	0	0	0
0	0	0	0	0	0	0	0	0	0	0	0	0	0
0	1	0	0	0	0	0	0	0	0	0	0	0	0
0	0	0	0	0	0	0	0	0	0	0	0	0	0
0													
OTU8453	1	0	0	0	0	0	0	0	0	0	0	0	0
0	0	0	0	0	0	0	0	0	0	0	0	0	0
0	0	0	0	0	0	0	0	0	0	0	0	0	0
0	0	0	0	0	0	0	0	0	0	0	0	0	0
0	0	0	0	0	0	0	0	0	0	0	0	0	0
0	0	0	0	0	0	0	0	0	0	0	0	0	0
0	0	0	0	0	0	0	0	0	0	0	0	0	0
0	0	0	0	0	0	0	0	0	0	0	0	0	0
0													
OTU14235	1	0	0	0	0	0	0	0	0	0	0	0	0
0	0	0	0	0	0	0	0	0	0	0	0	0	0
0	0	0	0	0	0	0	0	0	0	0	0	0	0
0	0	0	0	0	0	0	0	0	0	0	0	0	0
0	0	0	0	0	0	0	0	0	0	0	0	0	0
0	0	0	0	0	0	0	0	0	0	0	0	0	0

0	0	0	0	0	0	0	0	0	0	0	0	0	0
0	0	0	0	0	0	0	0	0	0	0	0	0	0
0													
OTU9651	1	1	0	1	0	3	1	0	0	0	2	0	0
1	0	0	0	0	1	1	0	0	0	0	0	0	0
0	0	0	0	0	1	0	0	1	0	0	0	0	0
1	0	0	0	0	0	1	0	0	0	0	0	0	1
0	5	0	3	0	0	0	0	0	0	0	0	1	0
0	1	1	0	1	1	0	0	1	4	0	0	0	0
0	1	0	0	0	3	0	2	0	0	0	0	1	1
1	0	0	0	0	0	0	2	0	0	0	0	0	1
0													
OTU8456	1	0	0	0	0	0	0	0	0	0	0	0	0
0	0	0	0	0	0	0	0	0	0	0	0	0	0
0	0	0	0	0	0	0	0	0	0	0	0	0	0
0	0	0	0	0	0	0	0	0	0	0	0	0	0
0	0	0	0	0	0	0	0	0	0	0	0	0	0
0	0	0	0	0	0	0	0	0	0	0	0	0	0
0	0	0	0	0	0	0	0	0	0	0	0	0	0
0	0	0	0	0	0	0	0	0	0	0	0	0	0
0													
OTU8455	1	0	0	0	0	0	0	0	0	0	0	0	0
0	0	0	0	0	0	0	0	0	0	0	0	0	0
0	0	0	0	0	0	0	0	0	0	0	0	0	0
0	0	0	0	0	0	0	0	0	0	0	0	0	0
0	0	0	0	0	0	0	0	0	0	0	0	0	0
0	0	0	0	0	0	0	0	0	0	0	0	0	0
0	0	0	0	0	0	0	0	0	0	0	0	0	0
0	0	0	0	0	0	0	0	0	0	0	0	0	0
0													
OTU122	1	26	1	0	0	0	5	0	31	5	0	1	0
0	0	1	1	0	7	48	6	3	0	1	1	15	0
0	1	0	0	4	0	1	0	0	0	1	0	20	0

37	17	0	7	1	20	2	5	0	4	11	10	0	2
0	57	0	11	3	1	0	0	2	0	0	0	44	1
0	25	42	0	17	33	0	2	0	25	3	11	1	0
0	1	0	7	11	38	13	52	1	0	0	25	28	0
0	42	6	25	18	8	12	57	4	38	1	0	0	28
19													
OTU10996	1	0	0	0	0	0	0	0	0	0	0	0	0
0	0	0	0	0	0	0	0	0	0	0	0	0	0
0	0	0	0	0	0	0	0	0	0	0	0	0	0
0	0	0	0	0	0	0	0	0	0	0	0	0	0
0	0	0	0	0	0	0	0	0	0	0	0	0	0
0	0	0	0	0	0	0	0	0	0	0	0	0	0
0	0	0	0	0	0	0	0	0	0	0	0	0	0
0	0	0	0	0	0	0	0	0	0	0	0	0	0
0													
OTU1292	1	0	0	1	2	2	0	0	0	0	0	1	0
0	1	4	0	0	0	0	0	0	0	0	0	0	0
0	0	0	0	0	1	0	0	1	1	0	0	0	0
0	0	0	0	1	2	0	2	0	0	0	2	0	4
0	1	0	1	0	0	0	0	0	0	0	0	0	0
0	1	0	1	1	2	0	0	0	1	0	0	0	0
0	1	0	0	0	0	0	0	1	1	1	0	2	0
1	0	0	0	0	0	0	0	0	0	0	3	0	0
0													
OTU10990	1	0	0	0	0	0	0	0	0	0	0	0	0
0	0	0	0	0	0	0	0	0	0	0	0	0	0
0	0	0	0	0	0	0	0	0	0	0	0	0	0
0	0	0	0	0	0	0	0	0	0	0	0	0	0
0	0	0	0	0	0	0	0	0	0	0	0	0	0
0	0	0	0	0	0	0	0	0	0	0	0	0	0
0	0	0	0	0	0	0	0	0	0	0	0	0	0
0	0	0	0	0	0	0	0	0	0	0	0	0	0
0													

OTU878		1	0	0	0	0	2	0	0	0	2	0	0	0
	0	0	0	0	0	0	1	0	0	0	0	0	0	0
	2	0	0	0	0	0	0	0	0	0	0	0	0	0
	0	0	0	0	0	0	0	0	0	0	0	0	0	0
	0	0	0	2	0	0	0	0	0	0	0	0	0	0
	0	0	0	0	0	0	0	0	0	0	0	0	0	0
	0	0	0	0	0	0	0	0	0	0	0	0	0	0
	0	0	0	0	0	0	0	0	0	0	0	0	0	0
	0													
OTU97	1	4	238	0	0	44	0	0	0	726	2	11	0	0
	0	0	0	0	20	0	2	0	0	0	1	0	0	0
	0	0	0	0	0	0	0	0	0	0	0	0	12	0
	7	2	0	0	2	0	0	1	0	0	0	0	0	0
	5	1	0	0	2	0	0	0	0	0	0	2	0	3
	2	3	0	26	1	0	0	0	31	0	2	1	5	0
	11	0	6	0	0	0	0	0	0	1	0	1	0	0
	0	24	30	18	1	2	6	0	6	1	2	0	7	3
OTU221		1	0	0	0	0	0	2	0	0	0	13	0	7
	10	0	0	35	0	1	0	0	17	1	2	0	0	13
	0	3	6	0	45	0	5	1	2	0	0	28	0	0
	0	0	17	0	32	0	3	0	3	11	28	0	2	24
	9	0	65	0	25	0	1	17	0	21	12	45	2	5
	22	0	0	9	0	0	0	0	0	1	2	0	1	1
	36	18	2	0	0	0	2	0	47	0	0	0	0	2
	2	0	0	0	0	0	0	0	0	0	0	0	7	0
	0													
OTU282		0	2	48	0	0	0	0	1	2	0	0	0	0
	0	0	0	0	0	0	1	0	0	0	0	0	1	0
	0	0	0	0	0	0	1	0	0	1	27	0	0	0
	0	0	1	0	0	0	0	0	0	0	0	0	0	0
	0	0	0	0	0	0	0	0	0	0	0	0	0	0
	0	3	1	0	0	0	0	2	0	0	0	2	0	0
	0	6	0	0	0	0	0	0	0	0	0	2	7	0

0	2	44	42	13	16	46	7	21	18	0	2	0	62
3													
OTU310	0	3	2	2	0	0	0	0	0	0	1	0	0
0	0	0	0	1	0	0	0	0	0	0	6	0	6
0	0	0	1	0	2	0	0	0	0	0	0	0	0
0	1	0	0	0	0	0	3	0	1	0	0	0	0
0	1	0	0	0	0	0	0	0	0	0	0	0	0
0	15	0	0	0	8	0	0	0	3	3	0	1	1
0	0	0	1	0	1	0	0	0	1	0	4	4	0
0	28	5	9	20	4	1	20	5	3	0	1	0	3
0													
OTU313	0	1	0	0	0	0	0	0	0	0	0	4	0
0	0	0	0	1	0	0	0	0	1	0	0	0	0
0	0	1	5	0	0	0	0	0	12	0	0	0	0
0	4	0	4	1	6	1	6	13	0	0	6	0	0
0	2	0	4	1	2	0	3	1	0	0	0	42	3
0	8	0	0	0	0	0	0	0	0	0	7	4	0
1	0	0	0	0	0	0	0	0	0	0	32	8	0
0	77	0	0	32	15	6	39	0	0	0	0	0	0
2													
OTU1170	0	2	0	0	0	0	0	0	0	0	0	0	0
0	0	0	18	0	0	0	0	17	0	3	0	0	0
0	14	0	0	2	0	6	0	0	0	4	0	0	0
0	0	0	0	2	0	0	0	2	3	3	0	1	0
13	0	3	0	22	7	3	3	5	5	4	1	0	11
3	0	0	0	0	0	0	9	0	0	0	1	0	0
0	0	0	0	0	0	1	0	0	0	0	0	0	0
0	0	2	0	0	0	0	0	0	0	0	0	0	0
0													
OTU249	0	117	0	0	10	0	2	0	4	3	0	0	0
0	3	0	0	0	0	3	0	0	0	0	17	1	0
0	0	0	0	0	0	0	0	0	0	0	0	0	0
5	0	0	5	0	0	0	4	0	0	0	0	0	0

0 0 0 2 0 0 0 0 0 0 0 0 2 0
0 7 6 0 1 2 0 0 0 1 0 0 0 0
0 0 0 41 0 0 0 2 0 0 0 6 3 0
0 26 17 96 11 4 1 28 12 5 0 1 0 81
60
OTU158 0 13 10 7 1 0 2 1 16 6 0 1 0
0 0 1 0 0 0 1 0 0 1 0 0 0 0
4 0 0 0 0 0 0 2 0 1 0 0 3 0
3 1 2 3 0 0 0 2 0 0 0 2 0 0
0 0 0 23 0 0 1 0 0 0 0 0 9 0
0 88 3 0 3 0 0 0 3 9 7 12 0 2
1 0 0 6 0 15 5 21 0 0 0 56 89 0
0 158 70 142 42 23 27 109 25 80 1 1 0 39
5
OTU402 0 16 0 0 0 0 0 0 0 1 0 0 0
0 0 0 0 0 0 0 0 0 0 0 0 0 0
0 0 0 0 0 0 0 0 0 0 0 0 0 0
0 0 0 0 0 0 0 0 0 0 0 0 0 0
0 1 0 0 0 0 0 0 0 0 0 0 0 0
0 6 0 0 0 0 0 0 0 0 1 0 0 0
0 0 0 0 0 0 0 0 0 0 0 1 2 0
0 0 7 2 2 4 2 0 21 6 0 0 0 36
2
OTU12234 0 1 0 0 0 0 0 0 0 0 0 0 0
0 0 0 0 0 0 0 0 0 0 0 0 0 0
0 0 0 0 0 0 0 0 0 0 0 0 0 0
0 0 0 0 0 0 0 0 0 0 0 0 0 0
0 0 0 0 0 0 0 0 0 0 0 0 0 0
0 0 0 0 0 0 0 0 0 0 0 0 0 0
0 0 0 0 0 0 0 0 0 0 0 0 0 0
0 0 0 0 0 0 0 0 0 0 0 0 0 0
0

OTU219		0	60	0	3	2	0	4	1	28	0	0	0	0
	0	0	0	0	0	1	6	0	0	0	2	0	9	0
	1	0	0	0	0	0	0	0	0	0	0	0	14	0
	18	0	0	1	0	0	0	1	0	1	0	0	0	0
	0	1	0	0	0	0	0	0	1	0	0	0	0	0
	1	4	21	0	0	0	0	8	0	21	1	2	0	0
	0	0	0	1	35	18	0	45	0	0	0	3	3	0
	0	4	40	12	9	1	9	4	15	7	0	2	0	3
	24													
OTU7029		0	4	1	0	0	0	1	0	0	0	1	0	2
	0	0	0	0	1	0	0	0	0	0	0	10	0	0
	0	0	1	0	0	0	0	0	0	0	0	3	0	0
	0	0	0	0	0	0	1	1	0	0	0	0	0	1
	0	0	0	0	0	0	0	0	0	0	0	0	0	0
	0	0	1	0	0	1	0	0	0	0	0	0	0	0
	1	0	0	0	0	0	0	0	0	2	0	0	0	4
	0	0	0	0	0	0	0	0	0	0	0	0	0	0
	0													
OTU167		0	31	0	5	13	2	2	1	3	0	4	9	0
	3	5	6	8	42	4	1	1	1	5	7	6	2	4
	3	2	11	6	1	5	2	7	0	57	4	8	2	0
	0	1	2	58	5	8	5	3	4	3	1	1	4	0
	2	3	0	0	5	1	6	5	4	0	4	2	1	4
	9	0	0	9	0	8	14	0	5	0	37	1	2	0
	10	0	24	0	13	0	1	0	6	4	0	0	0	1
	12	1	0	1	0	0	0	0	2	1	9	19	4	0
	0													
OTU16	0	73	9	231	280	2	19	30	85	38	18	13	17	20
	9	31	782	64	12	6	23	684	2	18	6	71	1	123
	238	126	27	328	14	140	57	12	26	12	0	3	5	225
	2	56	1	38	600	29	98	1157	20	93	169	874	18	1377
	78	12	229	4	360	1178	347	384	93	260	55	413	35	248
	137	1	0	5	0	1	13	0	1	191	33	0	2	0

11	0	13	10	1	0	11	2	3	9	0	131	362	43
115	2866	27	1147	1919	314	13	2963	297	1	37	39	396	105
OTU165	0	3	30	0	0	3	0	0	0	5	0	0	0
0	0	0	0	0	0	0	0	0	0	0	0	0	0
0	0	0	0	0	0	0	0	0	0	0	0	0	0
0	0	0	0	0	0	0	3	0	0	0	0	0	1
0	0	0	0	0	0	0	0	0	0	0	0	0	0
0	46	8	0	3	0	0	1	0	0	0	0	0	2
0	3	0	1	0	0	0	6	0	0	0	33	30	0
0	267	31	63	6	25	2	11	1	144	0	2	0	45
28													
OTU1479	0	15	1	0	0	3	0	0	0	0	0	0	3
0	0	0	0	0	0	0	0	0	0	0	9	0	0
0	0	1	0	0	0	0	1	1	0	0	0	0	0
0	0	1	2	0	0	0	0	0	0	0	0	0	0
0	0	2	0	1	0	0	0	0	0	0	0	0	0
0	0	0	9	0	1	0	0	0	0	9	0	0	0
3	0	1	0	4	0	0	0	0	0	0	0	0	1
0	0	0	0	0	0	0	0	0	0	0	0	0	0
0													
OTU10052	0	1	0	1	0	0	0	0	0	0	0	0	0
0	1	0	0	2	0	1	0	0	0	0	0	1	0
0	0	0	0	0	1	0	0	0	0	0	0	0	0
0	0	0	0	0	2	0	0	2	0	2	0	0	0
0	0	3	0	0	0	0	0	0	1	0	0	0	1
0	0	0	0	0	0	0	0	0	0	1	0	0	0
2	0	0	0	0	0	0	0	0	0	0	0	0	0
0	0	0	0	0	0	0	0	0	0	0	0	0	0
0													
OTU10989	0	1	0	0	0	0	0	0	0	0	0	0	0
0	0	0	0	0	0	0	0	0	0	0	0	0	0
0	0	0	0	0	0	0	0	0	0	0	0	0	0
0	0	0	0	0	0	0	0	0	0	0	0	0	0

0	0	0	0	0	0	0	0	0	0	0	0	0	0
0	0	0	0	0	0	0	0	0	0	0	0	0	0
0	0	0	0	0	0	0	0	0	0	0	0	0	0
0	0	0	0	0	0	0	0	0	0	0	0	0	0
0													
OTU1068	0	1	3	0	0	0	0	0	0	0	0	1	0
0	0	0	0	0	0	0	15	1	0	1	0	0	0
1	29	0	1	0	0	2	1	0	1	7	0	0	0
0	0	5	1	15	0	0	0	31	10	6	24	56	0
12	0	4	0	11	16	32	6	55	5	10	3	0	2
5	0	0	0	0	0	2	19	6	0	0	9	7	0
8	22	1	0	2	0	13	0	0	1	0	0	0	0
2	0	0	0	0	0	0	0	0	0	8	0	0	0
0													
OTU2654	0	5	3	0	4	1	0	3	1	0	0	3	0
0	1	0	0	0	0	2	0	0	0	0	0	0	0
0	0	0	1	0	0	0	1	1	1	1	0	6	1
1	0	0	2	0	0	0	0	0	0	2	1	0	0
0	0	0	0	0	0	0	0	0	0	0	0	0	1
0	0	4	3	0	0	0	0	1	1	1	0	4	1
0	0	3	4	1	0	2	0	0	2	2	0	0	0
1	0	0	1	1	0	0	0	0	0	2	1	0	0
0													
OTU10991	0	2	0	0	0	0	0	0	0	0	0	0	0
0	0	0	0	0	0	0	0	0	0	0	0	0	0
0	0	0	0	0	0	0	0	0	0	0	0	0	0
0	0	0	0	0	0	0	0	0	0	0	0	0	0
0	0	0	0	0	0	0	0	0	0	0	0	0	0
0	0	0	0	0	0	0	0	0	0	0	0	0	0
0	0	0	0	0	0	0	0	0	0	0	0	0	0
0	0	0	0	0	0	0	0	0	0	0	0	0	0
0													

OTU217		0	25	0	0	0	0	0	1	1	0	0	4	0
	1	0	55	1	0	0	3	27	0	0	0	61	0	12
	1	0	0	6	0	1	1	0	0	3	12	15	4	0
	0	4	0	24	5	1	2	4	3	1	1	42	0	0
	0	3	0	7	0	0	6	0	3	0	1	5	2	0
	0	56	0	0	0	0	0	3	1	0	4	0	3	0
	0	0	7	0	13	0	0	0	1	1	0	1	33	0
	33	0	2	0	5	10	2	3	4	0	2	0	2	5
	0													
OTU66	0	55	157	41	3	27	78	1	2	57	1	50	0	0
	2	0	0	2	0	3	49	0	1	0	148	3	1	47
	0	0	0	0	39	0	2	0	34	0	0	0	8	0
	18	4	115	0	1	0	36	1	0	0	29	0	0	0
	21	0	370	0	2	0	0	4	0	0	0	79	0	1
	0	1	0	477	57	3	2	1	5	11	6	73	39	39
	5	5	16	14	107	0	0	0	0	2	0	0	0	0
	0	0	0	0	0	0	0	0	0	22	0	1	0	0
OTU1482		0	1	0	0	0	0	0	0	0	0	0	0	0
	0	0	0	0	0	0	0	0	0	0	0	0	0	0
	0	0	0	0	0	0	0	0	0	0	0	0	0	0
	0	0	0	0	0	0	0	0	0	0	0	0	0	0
	0	0	0	0	0	0	0	0	0	0	0	0	1	0
	0	0	1	0	0	0	0	0	0	1	0	0	0	0
	0	0	0	1	0	0	0	0	0	0	0	0	0	0
	0	0	0	0	0	0	0	0	0	0	0	0	0	0
	0													
OTU677		0	4	0	0	0	0	1	0	2	3	0	0	0
	0	0	0	0	0	0	0	0	0	0	0	0	0	0
	0	0	0	0	0	0	0	0	0	0	0	0	0	0
	0	0	0	0	0	0	0	0	0	0	0	0	0	0
	0	0	0	0	0	0	0	0	0	0	0	0	0	0
	0	0	0	0	0	0	0	0	0	2	0	1	0	0
	0	0	0	1	0	0	0	0	0	0	0	0	1	1

0 2 0 1 5 4 0 2 0 4 0 0 0 0

3

OTU14928 0 1 16 0 0 0 0 0 0 0 0 0 0

0 0 0 0 0 0 0 0 0 0 0 0 0 0

0 0 0 0 0 0 0 0 0 0 0 6 0 1

0 0 0 0 0 0 0 0 0 0 0 0 0 0

0 0 0 0 0 0 0 0 0 0 0 0 0 0

0 0 0 0 0 0 0 0 0 0 0 0 0 0

0 0 0 0 0 0 0 1 0 0 0 0 0 0

0 0 0 0 0 3 0 0 0 0 0 0 0 0

0

OTU9894 0 1 0 0 0 0 0 0 0 1 0 0 0

0 0 0 0 0 0 0 0 0 0 0 0 0 0

0 0 0 0 0 0 0 0 0 0 0 0 0 0

0 0 0 0 0 0 0 0 0 0 0 0 0 0

0 0 0 0 0 0 0 0 0 0 0 0 0 0

0 0 0 0 0 0 0 0 0 0 0 0 0 0

0 0 0 0 0 1 0 0 0 0 0 0 0 0

0 0 0 0 0 0 0 0 0 0 0 0 0 0

0

OTU14100 0 8 2 0 2 0 4 0 0 0 0 3 1

0 2 1 0 1 0 0 0 0 0 0 5 0 0

0 0 0 1 1 0 0 0 1 0 0 0 0 0

0 0 0 0 0 0 0 0 0 0 0 0 0 0

0 0 0 0 0 0 0 0 0 0 0 0 0 0

0 0 2 2 0 2 1 0 3 0 0 0 0 0

1 1 0 0 0 0 1 0 0 1 0 0 0 0

1 0 0 0 0 0 0 0 0 0 1 1 0 0

0

OTU1033 0 1 1 9 1 1 0 8 0 0 2 3 5

8 0 1 25 0 1 2 1 4 0 11 0 2 0

0 0 0 0 10 0 26 13 14 0 38 0 4 3

0 0 3 0 17 0 0 1 1 0 1 3 0 0

2	0	5	0	3	3	0	9	0	1	0	0	7	11
0	8	0	0	0	0	0	2	0	1	0	0	1	0
0	0	0	0	0	0	0	2	4	0	0	0	7	7
0	0	110	44	16	39	101	10	4	11	2	12	0	2
25													
OTU896	0	7	0	0	0	24	0	0	4	4	0	0	0
0	2	0	0	0	1	16	0	0	0	0	0	0	0
7	0	0	0	0	0	0	0	0	1	0	0	0	0
10	0	0	0	0	0	0	0	1	0	0	0	0	0
0	0	0	1	0	2	0	0	0	0	0	0	1	0
0	0	7	0	93	0	0	4	0	86	0	4	0	2
0	0	0	73	0	0	0	0	0	0	0	0	0	0
0	0	0	4	0	0	0	0	0	13	0	0	0	0
3													
OTU462	0	25	7	0	0	1	9	2	7	5	0	0	0
0	2	0	3	0	0	1	3	12	2	4	0	31	0
30	17	0	0	3	2	2	3	5	0	0	0	0	0
10	0	1	2	1	1	1	3	15	21	2	0	0	1
3	0	1	1	6	7	10	38	19	10	4	0	26	4
1	52	2	2	0	4	5	13	1	8	3	30	1	58
0	13	1	3	3	3	4	2	0	7	0	9	33	0
0	30	47	6	30	52	30	24	26	37	0	2	4	64
23													
OTU541	0	1	0	0	0	0	0	0	0	0	0	0	0
0	0	0	0	0	0	0	0	0	0	0	0	0	0
0	0	0	0	0	0	0	0	0	0	0	0	0	0
0	0	0	0	0	0	0	0	0	0	0	0	0	0
0	0	0	0	0	0	0	0	0	0	0	0	0	0
0	0	0	0	0	0	0	0	0	0	0	0	0	0
0	0	0	0	0	0	0	0	0	0	0	0	2	0
0	2	7	13	0	8	0	2	6	12	0	0	0	7
0													

OTU3881 0 2 0 0 0 0 0 0 0 0 0 1 0
0 0 0 1 0 0 2 1 4 0 0 0 2 0
2 0 0 0 2 0 7 1 0 0 0 0 0 0
0 0 1 0 1 4 0 0 0 0 1 2 0 0
2 1 1 0 0 0 0 2 0 0 1 0 0 0
0 0 1 0 0 0 0 0 0 0 1 0 1 0
0 0 1 0 0 0 0 1 0 0 1 0 0 1
0 0 0 0 0 0 0 0 1 0 1 0 0 0
0
OTU10992 0 1 0 0 0 0 0 0 0 0 0 0 0
0 0 0 0 0 0 0 0 0 0 0 0 0 0
0 0 0 0 0 0 0 0 0 0 0 0 0 0
0 0 0 0 0 0 0 0 0 0 1 0 0 0
0 0 0 0 0 0 0 0 0 0 0 0 0 0
0 0 0 0 0 0 0 0 0 0 0 0 0 0
0 0 0 0 0 0 0 0 0 0 0 0 0 0
0 0 0 0 0 0 0 0 0 0 0 0 0 0
0
OTU365 0 10 4 0 0 0 1 0 3 0 0 0 0
0 0 0 0 0 0 0 0 0 0 0 0 0 0
0 0 0 0 0 0 0 0 0 0 9 0 0 0
1 0 0 0 1 0 0 0 0 0 0 0 0 0
0 2 0 1 0 0 0 0 0 1 0 0 9 0
0 8 0 0 0 0 0 1 0 3 1 2 0 14
0 0 0 2 0 0 0 2 0 0 0 2 4 0
0 11 13 17 3 53 19 3 19 4 0 1 0 0
1
OTU1243 0 1 0 0 1 0 1 0 0 0 0 1 0
0 0 0 0 0 0 0 0 1 0 0 0 0 0
0 0 1 0 0 0 0 0 0 0 1 0 0 0
0 0 0 0 0 0 0 0 0 0 0 0 0 0
0 0 0 0 0 0 0 0 0 0 0 0 0 0
0 0 0 0 1 0 0 1 1 0 0 0 0 0

0	0	0	0	0	0	0	0	0	0	0	0	0	0
0	0	0	0	0	1	0	0	0	0	0	0	0	0
0													
OTU8450	0	1	0	0	0	0	0	0	0	0	0	0	0
0	0	0	0	0	0	0	0	0	0	0	0	0	0
0	0	0	0	0	0	0	0	0	0	0	0	0	0
0	0	0	0	0	0	0	0	0	0	0	0	0	0
0	0	0	0	0	0	0	0	0	0	0	0	0	0
0	0	0	0	0	0	0	0	0	0	0	0	0	0
0	0	0	0	0	0	0	0	0	0	0	0	0	0
0	0	0	0	0	0	0	0	0	0	0	0	0	0
0													
OTU13683	0	1	0	0	0	0	0	0	0	0	0	0	0
0	0	0	0	0	0	0	0	0	0	0	0	0	0
0	0	0	0	0	0	0	0	0	0	0	0	0	0
0	0	1	0	0	0	0	0	0	0	0	0	0	0
0	0	0	0	0	0	0	0	0	0	0	0	0	0
0	0	0	0	0	0	0	0	0	0	0	0	0	0
0	0	0	0	0	0	0	1	0	0	0	3	0	0
0	5	0	0	0	0	1	0	0	0	0	0	0	1
2													
OTU14233	0	1	0	0	0	0	0	0	0	0	0	0	0
0	0	0	0	0	0	0	0	0	0	0	0	0	0
0	0	0	0	0	0	0	0	0	0	0	0	0	0
0	0	0	0	0	0	0	0	0	0	0	0	0	0
0	0	0	0	0	0	0	0	0	0	0	0	0	0
0	0	0	0	0	0	0	0	0	0	0	0	0	0
0	0	0	0	0	0	0	0	0	0	0	0	0	0
0	0	0	0	0	0	0	0	0	0	0	0	0	0
0													
OTU8060	0	4	1	0	1	4	1	0	0	0	0	1	0
0	3	0	0	1	0	0	0	0	0	0	1	0	0
0	0	0	0	0	0	0	0	2	0	0	2	0	0

0 0 0 0 0 0 0 2 0 0 0 0 0 4
0 0 0 0 0 0 0 0 0 0 0 0 0 0
0 0 1 0 0 2 0 0 0 0 0 0 0 0
2 0 0 0 0 0 1 0 0 0 0 0 0 1
0 0 0 0 0 0 0 0 0 0 0 1 0 0
0
OTU8452 0 1 0 0 0 0 0 0 0 0 0 0 0
0 0 0 0 0 0 0 0 0 0 0 0 0 0
0 0 0 0 0 0 0 0 0 0 0 0 0 0
0 0 0 0 0 0 0 0 0 0 0 0 0 0
0 0 0 0 0 0 0 0 0 0 0 0 0 0
0 0 0 0 0 0 0 0 0 0 0 0 0 0
0 0 0 0 0 0 0 0 0 0 0 0 0 0
0 0 0 0 0 0 0 0 0 0 0 0 0 0
0
OTU5238 0 1 1 0 0 0 0 0 0 0 0 0 0
0 0 0 0 0 0 0 0 0 0 0 0 0 0
0 0 0 0 0 0 0 0 0 0 0 0 0 1
0 0 0 0 0 0 0 0 0 0 0 0 0 0
0 0 0 0 0 0 0 0 0 0 0 0 0 0
0 0 0 0 0 0 0 0 0 0 0 0 0 0
0 0 0 0 0 0 0 0 0 0 0 0 0 0
0 0 0 0 0 0 0 0 0 0 0 0 0 0
0
OTU13880 0 1 0 0 0 0 0 0 0 0 0 0 0
0 0 0 0 0 0 0 0 0 0 0 0 0 0
0 0 0 0 0 0 0 0 0 0 0 0 0 0
0 0 0 0 0 0 0 0 0 0 0 0 0 0
0 0 0 0 0 0 0 0 0 0 0 0 0 0
0 0 1 0 0 0 0 0 0 0 0 0 0 0
0 0 0 0 0 0 0 0 0 0 0 0 0 0
0 0 0 0 0 0 0 0 0 0 0 0 0 0
0

OTU1504	0	3	0	0	0	0	0	0	0	0	2	0	0
0	1	0	0	1	9	0	0	0	8	0	2	0	0
0	0	1	0	0	0	0	0	0	0	0	3	0	8
0	0	0	0	0	0	0	0	0	0	0	0	0	0
0	0	0	0	0	0	0	0	0	0	0	0	0	0
0	0	0	0	0	1	0	0	2	0	0	0	0	0
0	0	0	0	0	2	0	0	0	0	0	0	0	0
0	0	0	0	0	0	0	0	0	0	0	3	0	0
0													
OTU3396	0	1	0	0	0	0	1	0	3	0	0	1	2
0	0	0	1	0	0	0	0	0	0	0	0	0	0
2	0	0	0	0	0	1	0	1	0	0	0	2	0
0	0	1	0	0	7	0	2	0	0	0	0	0	3
0	2	2	1	2	0	0	1	0	3	0	0	1	2
1	1	0	0	1	0	0	1	0	0	0	0	0	3
0	2	0	1	0	2	0	9	1	0	0	0	6	1
0	1	1	0	2	0	4	9	4	3	0	0	1	0
1													
OTU8451	0	1	0	0	0	0	0	0	0	0	0	0	0
0	0	0	0	0	0	0	0	0	0	0	0	0	0
0	0	0	0	0	0	0	0	0	0	0	0	0	0
0	0	0	0	0	0	0	0	0	0	0	0	0	0
0	0	0	0	0	0	0	0	0	0	0	0	0	0
0	0	0	0	0	0	0	0	0	0	0	0	0	0
0	0	0	0	0	0	0	0	0	0	0	0	0	0
0	0	0	0	0	0	0	0	0	0	0	0	0	0
0													
OTU1464	0	1	2	2	4	2	2	2	4	2	5	6	3
3	10	2	1	2	0	1	0	6	4	1	1	0	1
2	1	3	4	1	4	2	3	0	1	2	2	9	3
4	2	5	3	1	1	1	3	3	12	1	1	2	1
3	1	4	5	2	3	0	3	4	2	2	1	1	2
9	6	2	2	5	3	2	1	0	1	1	12	1	2

	4	2	1	3	1	4	2	3	2	4	2	2	1	2
	1	5	3	6	0	3	0	4	3	1	4	2	1	4
	12													
OTU14234		0	1	0	0	0	0	0	0	0	0	0	0	0
	0	0	0	0	0	0	0	0	0	0	0	0	0	0
	0	0	0	0	0	0	0	0	0	0	0	0	0	0
	0	0	0	0	0	0	0	0	0	0	0	0	0	0
	0	0	0	0	0	0	0	0	0	0	0	0	0	0
	0	0	0	0	0	0	0	0	0	0	0	0	0	0
	0	0	0	0	0	0	0	0	0	0	0	0	0	0
	0	0	0	0	0	0	0	0	0	0	0	0	0	0
	0													
OTU81	0	32	74	0	0	15	143	2	74	50	0	7	0	0
	43	3	3	9	0	45	0	1	0	7	0	1	1	1
	4	5	0	5	0	0	0	0	2	2	10	6	2	68
	19	3	0	1	68	2	126	0	3	3	11	2	0	0
	51	0	29	0	0	1	2	1	1	0	1	304	0	2
	39	26	5	0	10	1	21	0	42	14	5	44	35	0
	6	0	1	2	92	1	6	0	42	0	37	52	1	0
	63	18	12	17	18	5	29	7	33	3	2	5	34	29
OTU12416		0	1	0	0	0	0	0	0	0	1	0	0	0
	0	0	0	0	0	0	0	0	0	0	0	0	0	0
	0	0	0	0	0	0	0	0	0	0	0	0	0	0
	0	0	0	0	0	0	0	0	0	1	0	0	0	0
	0	0	0	0	0	0	0	0	0	0	0	0	0	0
	0	0	0	0	0	0	0	0	0	1	0	0	0	0
	0	0	0	0	0	0	0	0	0	0	0	0	0	0
	0	0	0	0	0	0	0	1	0	0	0	0	0	0
	0													
OTU857		0	2	0	3	0	0	0	1	1	0	2	1	0
	1	0	1	1	0	0	0	0	0	0	1	0	0	0
	0	0	0	0	0	2	0	0	0	1	0	0	0	0
	0	0	0	0	0	0	0	1	0	0	0	0	0	0

0	0	0	0	0	0	0	0	0	0	0	0	0	0
0	0	0	0	0	0	0	0	0	0	0	0	0	0
0	1	0	0	0	0	0	0	1	0	0	2	0	0
0	0	0	1	0	0	0	0	0	0	0	0	0	0
0													
OTU11528	0	1	0	0	0	0	0	0	0	0	0	0	0
0	0	0	0	0	0	0	1	0	0	0	0	0	0
0	0	0	0	0	0	0	0	1	0	0	0	0	0
0	0	0	0	0	0	0	0	0	0	0	0	0	0
0	0	0	0	1	0	0	0	0	0	0	0	1	5
1	0	0	0	0	0	0	0	2	0	0	0	0	0
0	0	0	0	0	0	0	0	0	0	0	0	0	0
0	0	0	0	0	0	0	0	0	0	0	0	0	0
0													
OTU10814	0	2	0	0	0	0	0	0	0	0	0	0	0
0	0	0	0	0	0	0	0	0	0	0	0	0	0
0	0	0	0	0	0	0	0	0	0	0	0	0	0
0	0	0	0	0	0	0	0	0	0	0	0	0	0
0	0	0	0	0	0	0	0	0	0	0	0	0	0
0	0	0	0	0	0	0	0	0	0	0	0	0	0
0	0	0	0	0	0	0	0	0	0	0	0	0	0
0	0	0	0	0	0	0	0	0	0	0	0	0	0
0													
OTU180	0	43	0	0	0	0	0	0	4	0	0	1	0
0	0	0	0	1	0	0	0	0	0	0	0	1	0
0	0	0	0	0	0	0	0	0	0	0	0	0	1
1	0	0	1	0	0	0	4	0	0	0	1	0	0
0	0	0	0	0	0	0	0	1	0	0	1	0	0
0	35	0	0	6	0	0	1	0	1	1	4	0	2
0	1	0	0	1	0	0	0	0	0	0	27	28	0
0	126	22	124	72	56	71	12	28	14	0	1	0	91
54													

OTU399	0	4	0	0	0	0	0	0	0	0	0	0	0
0	0	0	0	0	0	0	0	0	0	0	0	0	0
0	0	0	0	0	0	0	0	0	0	0	0	0	0
0	0	0	0	0	0	0	0	0	0	0	0	0	0
0	0	0	0	0	0	0	0	0	0	0	0	0	0
0	10	0	0	1	0	0	0	0	0	0	0	0	0
0	0	0	1	0	0	0	0	0	0	0	1	6	0
0	5	22	0	6	3	2	0	15	12	0	0	0	0
0													
OTU3568	0	1	0	0	0	0	0	0	0	1	0	0	0
0	0	0	0	0	0	1	0	0	0	0	1	0	0
0	0	0	0	0	0	3	0	0	0	0	0	0	0
0	0	0	0	0	0	0	0	0	0	0	0	0	0
0	0	0	0	0	0	0	0	0	0	0	0	0	0
0	0	0	0	1	0	0	0	0	2	0	2	0	0
0	0	0	0	0	0	2	0	0	0	0	0	0	0
0	0	0	0	0	0	0	0	0	0	0	0	0	0
0													
OTU817	0	13	2	0	0	0	0	0	0	1	0	0	0
0	0	0	0	0	0	0	0	0	1	0	19	1	0
1	0	0	0	0	0	0	0	0	1	0	0	0	0
3	0	0	1	0	0	0	0	0	0	0	0	0	0
0	0	0	1	0	0	0	0	0	0	0	0	0	0
1	3	1	0	1	0	0	0	0	0	0	1	1	1
0	1	0	0	0	0	0	2	0	0	0	0	0	0
0	0	1	3	1	1	0	0	1	1	0	0	0	12
2													
OTU12173	0	1	0	0	0	0	0	0	0	0	0	0	0
0	0	0	0	0	0	0	0	0	0	0	0	0	0
0	0	0	0	0	0	0	0	0	0	0	0	0	0
0	0	0	0	0	0	0	0	0	0	0	0	0	0
0	0	0	0	0	0	0	0	0	0	0	0	0	0
0	0	0	0	0	0	0	0	0	0	0	0	0	0

0	0	0	0	0	0	0	0	0	0	0	0	0	0
0	0	0	0	0	0	0	0	0	0	0	0	0	0
0													
OTU535	0	1	4	0	0	2	1	0	4	0	0	0	0
0	0	0	0	0	0	0	0	0	2	0	0	0	0
1	0	0	0	0	0	0	0	0	0	0	0	0	0
5	0	0	0	0	0	0	0	0	0	0	0	0	0
0	0	0	0	0	0	0	0	0	0	0	0	0	0
0	0	0	0	0	0	0	0	0	3	0	0	0	1
0	0	0	0	0	2	0	4	0	0	0	0	0	0
0	0	1	0	0	0	0	0	2	0	0	0	0	1
0													
OTU8457	0	1	0	0	0	0	0	0	0	0	0	0	0
0	0	0	0	0	0	0	0	0	0	0	0	0	0
0	0	0	0	0	0	0	0	0	1	0	0	0	0
0	0	0	1	0	0	0	0	0	0	0	0	0	0
0	0	0	0	0	0	0	0	0	0	0	0	0	0
0	0	0	0	0	0	0	0	0	0	0	1	1	0
0	0	0	0	0	0	0	0	0	0	0	0	0	0
0	0	0	0	0	0	0	0	0	0	0	0	0	0
0													
OTU12285	0	2	15	0	0	0	0	0	0	0	0	0	0
0	0	0	1	0	0	0	0	0	0	0	0	0	0
0	0	0	0	0	0	0	0	0	0	0	0	0	0
0	0	0	0	0	0	0	0	0	0	0	0	0	0
0	0	0	0	0	0	0	0	0	0	0	0	0	0
0	0	0	0	0	0	0	0	0	0	0	0	0	1
0	0	0	0	0	0	0	0	0	0	0	1	0	0
0	0	0	0	0	0	0	0	0	0	0	0	0	2
0													
OTU7385	0	1	1	1	0	0	1	0	1	1	0	1	0
1	5	0	1	0	0	1	0	1	0	0	0	1	0
2	0	0	1	2	0	0	1	1	0	0	0	2	0

1	1	0	1	0	2	0	4	0	2	0	1	0	2
2	0	2	0	0	1	0	0	2	0	1	0	0	3
3	1	0	1	3	0	1	0	0	0	0	0	0	1
1	0	0	1	0	0	0	1	0	0	0	1	0	1
1	0	2	0	2	1	0	0	1	0	0	1	0	1
1													
OTU528	0	11	1	0	3	41	28	0	24	25	0	15	0
0	57	0	0	0	1	2	0	0	0	0	0	0	0
0	0	1	0	0	0	0	0	0	0	0	0	0	2
1	5	0	139	0	3	3	2	0	0	0	0	1	0
0	3	0	1	0	0	0	1	0	0	1	0	8	0
0	6	8	0	502	33	0	2	1	4	42	2	1	0
0	0	1	14	0	0	0	1	0	0	1	2	29	0
0	1	1	8	1	14	1	17	3	18	15	1	0	9
0													
OTU1730	0	5	0	1	0	0	0	1	0	0	0	4	2
2	0	0	102	1	0	0	28	32	0	2	0	0	1
1	57	0	0	0	3	9	3	4	2	26	0	0	0
0	0	2	0	4	0	0	4	0	1	5	0	0	0
0	0	1	0	16	4	1	1	1	0	6	46	1	36
17	0	0	0	0	0	0	1	32	7	0	1	0	0
1	3	23	0	2	0	0	0	1	11	1	0	0	0
4	0	0	2	1	0	2	0	1	1	5	3	24	2
0													
OTU3355	0	1	0	0	1	0	0	0	0	2	1	0	1
0	0	1	0	0	0	0	0	0	0	0	0	0	0
0	0	0	0	0	0	0	0	0	1	0	1	0	0
1	0	0	0	0	0	0	0	0	0	0	1	0	0
1	0	1	0	0	0	0	1	0	0	0	1	0	0
0	0	0	0	1	0	0	0	0	0	0	0	1	1
1	1	0	0	0	0	0	0	0	0	0	0	0	0
0	0	0	0	0	1	0	0	0	0	0	0	0	0
0													

OTU16415	0	2	0	0	2	0	0	0	1	1	1	0	1
0	0	0	0	0	1	0	1	0	1	0	0	1	0
0	0	1	0	0	0	0	0	0	0	1	1	1	1
0	1	0	0	0	0	0	0	0	0	0	0	0	0
1	0	0	1	0	1	1	0	0	0	0	1	1	0
1	1	0	0	1	0	1	1	0	0	0	0	0	0
1	0	0	0	1	1	0	2	0	0	0	0	1	1
0	0	0	0	0	0	0	0	0	0	0	0	0	0
0													
OTU13153	0	1	0	0	1	0	0	0	0	1	0	0	0
0	0	0	0	0	0	0	0	0	0	0	0	0	0
0	0	0	0	0	1	0	0	0	0	0	0	0	0
0	1	0	0	0	0	0	0	0	0	0	0	0	0
0	0	1	0	0	0	0	0	0	0	0	0	0	0
2	0	0	0	0	0	0	0	0	0	0	0	0	0
0	0	0	0	0	0	0	0	0	0	0	0	0	0
0	0	0	0	0	0	0	0	0	0	0	0	0	0
0													
OTU133	0	2	4	0	1	5	0	0	4	0	27	1	9
1	0	7	155	8	0	1	2	13	0	1	0	0	0
0	0	26	5	6	1	0	13	1	1	0	1	0	3
1	4	24	1	0	1	1	25	3	1	1	1	0	22
2	1	3	1	0	2	1	2	1	6	0	0	1	0
1	0	1	5	1	0	12	0	2	0	1	0	4	1
24	0	6	0	2	1	1	1	28	34	11	695	0	31
1	3	1	0	5	0	0	2	0	1	0	2	1	2
53													
OTU12174	0	1	0	0	0	0	0	0	0	0	0	0	0
0	0	0	0	0	0	0	0	0	0	0	0	0	0
0	0	0	0	0	0	0	0	0	0	0	0	0	0
0	0	0	0	0	0	0	0	0	0	0	0	0	0
0	0	0	0	0	0	0	0	0	0	0	0	0	0
0	0	0	0	0	0	0	0	0	0	0	0	0	0

	0	0	0	0	0	0	0	0	0	0	0	0	0	0
	0	0	0	0	0	0	0	0	0	0	0	0	0	0
	0													
OTU96	0	2	1	0	1	0	1	0	1	1	2	1	1	1
	0	0	3	22	0	1	8	31	11	64	154	163	29	192
	59	0	1	40	10	18	0	0	68	36	1	15	0	0
	0	12	0	81	0	0	2	132	37	22	73	67	1	82
	3	8	8	71	67	86	17	44	76	2	26	4	29	1
	0	4	0	0	0	0	116	0	0	0	5	15	37	0
	11	0	1	0	0	4	1	0	1	0	0	0	0	0
	2	13	46	2	5	3	1	0	2	1	0	0	7	7
OTU12175		0	1	0	0	0	0	0	0	0	0	0	0	0
	0	0	0	0	0	0	0	0	0	0	0	0	0	0
	0	0	0	0	0	0	0	0	0	0	0	0	0	0
	0	0	0	0	0	0	0	0	0	0	0	0	0	0
	0	0	0	0	0	0	0	0	0	0	0	0	0	0
	0	0	0	0	0	0	0	0	0	0	0	0	0	0
	0	0	0	0	0	1	0	0	0	0	0	0	0	0
	0	0	0	1	0	0	0	0	0	0	0	0	0	0
	0													
OTU247		0	2	18	1	0	10	8	0	14	49	0	1	0
	0	1	1	0	0	0	2	0	0	0	0	0	1	0
	0	0	0	0	0	0	0	0	0	1	0	0	0	0
	15	0	1	0	0	0	0	5	0	1	0	0	0	1
	0	1	0	1	0	0	0	0	1	0	1	1	6	0
	3	44	3	0	0	0	0	1	0	8	0	3	0	9
	0	3	0	3	0	0	0	29	0	0	0	72	73	0
	0	35	11	20	96	24	105	100	4	15	1	3	0	2
	29													
OTU14238		0	1	0	0	0	0	0	0	0	0	0	0	0
	0	0	0	0	0	0	0	0	0	0	0	0	0	0
	0	0	0	0	0	0	0	0	0	0	0	0	0	0
	0	0	0	0	0	0	0	0	0	0	0	0	0	0

0	0	0	0	0	0	0	0	0	0	0	0	0	0
0	0	0	0	0	0	0	0	0	0	0	0	0	0
0	0	0	0	0	0	0	0	0	0	0	0	0	0
0	0	0	0	0	0	0	0	0	0	0	0	0	0
0													
OTU16387	0	1	0	0	0	0	0	0	0	0	0	0	0
0	0	0	0	0	0	0	0	0	0	0	0	0	0
0	0	0	0	1	1	0	0	0	0	1	0	0	0
0	0	0	0	0	0	0	0	0	0	1	0	0	2
0	0	1	0	0	0	0	0	0	0	0	0	0	1
0	0	0	0	0	0	0	0	0	0	0	0	0	0
0	0	0	0	0	0	0	0	0	0	0	0	0	0
0	0	0	0	0	0	1	0	0	0	0	0	0	0
0													
OTU6441	0	1	0	1	0	0	1	0	0	0	0	0	0
0	0	0	0	1	0	0	0	0	0	0	0	0	0
0	0	0	0	0	0	0	1	1	0	0	0	0	0
0	0	0	0	1	1	0	0	0	0	0	0	0	1
0	2	0	2	0	0	0	0	0	0	0	0	1	0
0	0	0	0	0	0	0	0	1	0	0	0	0	1
0	0	0	0	1	1	0	0	0	0	1	0	0	0
1	0	0	0	0	0	0	0	0	0	0	2	1	0
0													
OTU10414	0	1	0	0	0	0	0	0	0	0	0	0	0
0	0	0	0	0	0	0	0	0	0	0	0	0	0
0	0	0	0	0	0	0	0	0	0	0	0	0	0
0	0	0	0	0	0	0	0	0	0	0	0	0	0
0	0	0	0	0	0	0	0	0	0	0	0	0	0
0	0	0	0	1	0	0	0	0	0	0	0	0	0
0	0	0	0	0	0	0	0	0	0	0	0	0	0
0	0	0	0	0	0	0	0	0	0	0	0	0	0
0													

OTU3389	0	1	3	1	2	1	0	2	2	0	0	1	1
1	1	0	1	0	2	0	0	0	0	1	1	1	0
0	1	2	1	1	2	1	0	1	0	0	0	2	1
0	0	1	2	2	0	2	1	0	1	0	1	0	0
1	0	0	0	0	1	1	1	2	0	0	2	0	1
4	1	1	0	2	1	0	5	1	0	0	1	0	1
0	1	0	2	1	0	1	0	0	1	0	1	0	0
0	1	1	0	0	2	0	4	0	0	0	2	0	0
2													
OTU7138	0	2	0	2	3	1	0	1	0	3	1	0	0
1	0	3	0	2	0	1	0	1	1	0	1	0	0
1	0	2	1	0	1	1	1	0	0	1	1	0	1
0	1	1	0	0	0	0	1	3	2	0	0	0	0
0	2	1	0	0	2	0	0	2	1	1	0	1	0
2	0	0	0	2	0	0	4	0	0	0	2	1	0
1	0	0	0	0	2	0	1	2	0	0	0	0	1
2	1	0	0	0	0	0	0	0	0	0	0	0	0
1													
OTU228	0	1	0	0	0	0	3	0	7	19	0	0	0
0	0	0	0	0	0	6	0	0	0	0	0	0	1
0	0	0	0	0	0	0	0	0	2	0	0	1	1
20	4	0	1	3	0	0	1	0	0	1	1	0	0
0	0	0	21	0	0	0	0	0	0	0	0	12	0
0	28	0	0	3	0	1	1	0	3	10	15	0	0
0	0	0	0	0	0	0	3	0	0	0	44	25	0
0	107	38	77	133	298	23	41	20	52	0	7	0	109
12													
OTU3731	0	2	0	0	0	0	6	0	0	2	1	0	13
1	0	0	2	7	6	0	1	3	4	0	87	1	2
0	0	2	0	5	1	4	0	0	1	25	16	0	0
0	4	7	5	0	0	4	1	0	0	0	0	0	2
0	1	0	0	0	0	0	0	0	0	2	9	0	0
0	0	0	0	8	1	19	0	3	2	37	0	2	0

10 3 4 2 2 8 0 0 67 0 0 0 0 0
0 0 0 0 0 0 0 0 0 0 0 0 0 0
0
OTU302 0 5 0 0 2 3 0 0 0 0 1 0 3
2 0 0 1 1 0 3 2 0 0 0 0 11 0
0 0 5 10 2 2 2 25 0 0 4 0 1 1
0 0 0 0 5 16 0 18 0 1 3 0 0 11
0 19 3 0 0 1 0 0 1 0 2 39 5 2
1 0 0 0 0 0 0 0 0 0 1 0 0 0
25 6 1 3 4 1 0 2 3 0 6 0 0 1
32 0 0 0 0 1 0 0 0 0 0 0 1 0
0
OTU14472 0 1 0 0 0 0 1 0 0 0 0 0 0
0 0 0 0 0 0 0 0 0 0 0 0 0 0
0 0 0 0 0 0 0 0 0 0 0 0 0 0
0 0 0 0 0 0 0 0 0 0 0 0 0 0
0 0 0 0 0 0 0 0 0 0 0 0 0 0
0 0 0 0 0 0 0 0 0 0 0 0 0 0
0 0 0 0 0 0 0 0 0 0 0 0 0 0
0 0 0 0 0 0 0 0 0 0 0 0 0 0
0
OTU748 0 2 0 0 3 0 1 2 0 1 0 0 0
0 1 0 0 0 0 0 0 0 0 0 0 0 0
0 0 0 0 1 0 0 0 1 0 0 0 0 0
0 0 0 0 0 1 0 3 0 0 0 0 0 1
0 1 0 0 1 0 0 0 0 0 0 0 2 0
0 0 0 0 0 0 0 0 0 0 0 0 0 0
0 0 0 0 0 1 0 1 0 0 0 0 0 0
0 0 0 0 0 0 0 0 0 0 0 0 0 0
0
OTU1169 0 12 6 7 11 4 2 6 1 0 0 1 1
1 5 1 0 0 1 0 6 0 0 9 11 5 3
0 2 1 4 6 0 4 1 4 1 11 1 1 2

2	2	1	13	1	0	1	3	0	2	2	0	0	5
0	2	6	2	5	0	1	1	1	2	0	11	0	2
0	2	7	3	0	7	5	0	4	2	3	1	12	5
1	2	4	12	15	0	9	1	3	1	1	0	0	4
5	0	0	3	1	0	0	1	0	0	13	0	0	0
0													
OTU2937	0	8	0	0	0	0	12	0	0	0	0	0	5
1	0	2	3	2	6	3	0	3	4	0	82	1	2
2	0	7	0	8	0	2	0	0	1	36	6	1	0
1	0	10	4	0	0	6	1	0	0	0	0	0	2
0	2	0	0	1	0	0	0	0	0	0	6	0	1
0	0	3	0	1	4	6	0	0	2	37	0	0	1
8	0	3	0	3	12	3	0	47	0	0	0	0	0
0	0	0	0	0	0	0	0	0	0	1	0	0	0
0													
OTU430	0	1	0	0	0	0	0	0	0	0	0	0	0
0	0	3	1	0	2	0	2	0	5	0	1	1	0
0	0	0	0	0	0	0	0	0	38	0	0	0	0
1	0	0	1	1	0	0	0	0	0	12	1	0	0
0	0	0	1	0	0	0	0	0	0	0	2	0	0
0	0	0	0	1	0	0	0	0	0	0	0	3	0
0	1	0	0	0	0	1	0	0	0	0	0	0	0
0	0	0	0	0	1	0	1	0	0	0	0	2	1
0													
OTU10429	0	2	2	0	2	0	0	1	46	1	0	0	0
0	2	0	0	0	0	0	0	0	0	0	0	0	0
0	0	0	0	0	0	0	0	0	0	0	0	1	0
0	0	0	0	0	0	0	0	0	0	0	0	0	0
0	0	0	0	0	0	0	0	0	0	0	0	0	0
0	0	0	0	18	0	0	2	0	11	0	0	0	0
0	0	0	0	0	0	0	0	0	0	0	1	2	0
0	0	1	0	2	2	0	5	1	0	0	0	0	0
2													

OTU10042	0	9	2	0	0	0	10	1	0	0	0	0	0
0	6	0	0	0	0	0	0	0	0	0	0	0	0
0	0	0	0	0	0	0	0	0	0	0	0	0	0
0	0	0	0	0	0	0	0	0	0	0	0	0	0
0	0	0	0	0	0	0	0	0	0	0	0	0	0
0	0	0	0	0	0	0	0	0	0	0	0	0	0
0	2	0	0	0	0	0	0	0	0	0	0	0	0
0	0	0	0	0	0	0	0	0	0	0	0	0	0
0													
OTU5216	0	2	0	0	0	0	0	0	0	0	0	0	0
0	0	0	0	0	2	0	0	0	2	0	0	0	0
0	0	2	0	0	1	0	0	0	0	0	3	0	3
0	0	0	0	0	0	0	0	0	0	0	0	0	0
0	0	0	0	0	0	0	0	0	0	0	0	0	0
0	0	0	0	0	0	0	0	1	0	1	0	0	0
0	0	1	0	0	0	0	0	0	0	0	0	0	0
0	0	0	0	0	0	0	0	0	0	0	0	0	0
0													
OTU13154	0	1	0	0	0	0	0	0	0	1	0	0	0
0	0	0	0	0	0	0	0	0	0	0	0	0	0
0	0	0	1	0	1	0	0	0	0	0	0	0	0
0	0	0	0	0	0	0	0	0	0	0	0	0	0
0	0	0	0	0	0	0	0	0	0	0	0	0	0
0	0	0	0	1	0	0	0	0	0	1	0	1	0
0	0	0	1	0	0	0	0	0	0	0	0	0	0
0	0	0	0	0	0	0	0	0	0	0	0	0	0
0													
OTU10187	0	2	2	0	2	1	1	1	0	1	2	0	1
2	1	1	3	2	1	0	0	2	2	2	1	1	0
0	0	3	4	0	3	0	0	0	1	1	3	4	0
1	1	1	1	0	1	0	3	2	4	1	1	3	0
3	2	2	3	0	2	2	1	1	2	3	2	1	4
0	1	0	1	8	1	0	5	0	1	0	3	1	0

0	1	0	0	1	4	0	2	1	0	0	1	0	0
0	4	1	4	0	3	1	2	0	2	0	2	0	3
5													
OTU14237	0	1	0	0	0	0	0	0	0	0	0	0	0
0	0	0	0	0	0	0	0	0	0	0	0	0	0
0	0	0	0	0	0	0	0	0	0	0	0	0	0
0	0	0	0	0	0	0	0	0	0	0	0	0	0
0	0	0	0	0	0	0	0	0	0	0	0	0	0
0	0	0	0	0	0	0	0	0	0	0	0	0	0
0	0	0	0	0	0	0	0	0	0	0	0	0	0
0	0	0	0	0	0	0	0	0	0	0	0	0	0
0													
OTU6230	0	5	3	0	0	0	0	0	0	0	0	1	0
0	1	1	1	0	0	0	0	0	0	7	0	0	0
0	2	0	0	2	0	0	0	0	0	0	0	0	0
0	0	0	0	1	0	0	0	1	1	4	0	0	0
1	0	0	0	1	7	0	2	3	0	5	0	0	0
7	0	0	0	0	0	0	1	0	0	0	0	1	0
0	0	0	0	0	0	1	1	0	0	0	0	1	0
0	0	0	0	0	0	0	0	0	0	0	0	0	0
0													
OTU12080	0	4	4	0	0	0	0	1	0	0	0	0	0
0	0	0	0	1	0	0	0	0	7	0	3	0	0
0	0	0	0	0	0	0	0	0	2	0	0	0	0
0	0	0	0	5	0	0	0	0	0	7	0	0	0
0	0	1	0	0	0	0	0	0	0	0	4	0	0
0	2	3	0	0	0	0	0	0	0	0	0	0	0
0	0	0	0	0	0	4	0	0	0	0	0	1	0
0	0	3	2	0	1	1	0	5	2	0	0	0	0
0													
OTU7857	0	17	1	1	0	1	11	0	16	7	0	0	0
0	0	0	0	0	0	0	0	0	0	0	0	0	0
0	0	0	0	0	0	0	0	1	0	0	0	9	0

2	0	0	0	0	0	0	1	0	0	0	0	0	0
0	1	0	0	0	0	0	0	0	0	0	0	2	0
0	0	3	0	0	0	0	1	0	0	0	4	0	41
0	0	0	5	0	0	0	7	0	0	0	0	0	0
0	3	1	17	3	3	22	1	1	11	0	1	0	4
0													
OTU1181	0	9	4	0	0	0	4	0	2	0	0	0	0
1	0	0	3	0	0	7	0	4	0	0	0	0	0
3	3	0	0	0	0	1	0	0	0	137	0	0	0
0	0	0	0	0	0	0	0	0	0	0	0	0	0
0	1	0	0	0	1	0	1	1	0	6	0	0	0
0	1	0	1	0	0	0	0	0	1	0	0	0	0
0	0	1	0	0	0	0	0	0	0	0	1	0	0
0	1	1	1	5	0	5	3	1	1	0	0	0	5
6													
OTU527	0	1	0	0	1	0	0	1	1	2	0	0	0
0	3	0	0	0	0	0	0	0	0	0	0	0	0
0	0	0	0	0	0	0	0	1	0	0	0	0	0
0	0	0	1	0	0	0	0	0	0	0	0	0	0
0	0	0	3	0	0	0	0	0	0	0	0	1	0
0	3	1	1	0	3	0	0	0	0	2	0	2	0
0	0	0	3	1	0	0	0	0	1	0	2	0	0
0	0	0	3	0	1	1	0	0	2	0	0	0	1
4													
OTU1123	0	2	0	0	0	0	0	0	0	1	0	0	0
0	0	0	0	0	0	1	0	0	0	0	0	0	0
0	0	0	0	0	0	0	0	0	0	0	0	0	0
0	0	0	0	0	0	0	0	0	0	0	0	0	0
0	0	0	0	0	0	1	0	0	0	0	0	0	0
0	2	0	0	1	0	0	0	0	0	1	0	0	0
0	0	0	0	0	0	0	0	0	2	0	0	0	0
0	0	1	0	1	1	0	0	7	2	0	0	0	0
0													

OTU10979 0 1 0 0 0 0 0 0 0 0 0 0 0
0 0 0 0 0 0 0 0 0 0 0 0 0 0
0 0 0 0 0 0 0 0 0 0 0 1 0 0
0 0 0 0 0 0 0 0 0 0 0 0 0 0
0 0 0 0 0 0 0 0 0 0 0 0 0 0
0 0 0 0 0 0 0 0 0 0 0 0 0 0
0 0 0 0 0 0 0 0 0 0 0 0 0 0
0 0 0 0 0 0 0 0 0 0 0 0 0 0
0
OTU324 0 4 0 0 0 0 0 0 3 0 0 1 0
0 0 0 0 0 0 0 0 0 0 0 0 0 0
0 0 0 0 0 0 0 0 0 0 0 0 0 1
0 0 0 0 0 0 0 0 0 0 0 0 0 0
0 0 0 0 0 0 0 0 0 0 0 0 0 0
0 2 0 0 8 0 0 1 0 0 1 1 0 0
0 0 0 0 0 0 0 2 0 0 0 2 1 0
0 12 6 93 13 44 6 9 7 15 0 0 0 2
25
OTU9075 0 1 1 0 1 0 0 1 0 0 0 0 1
0 0 1 0 1 0 1 2 0 2 0 1 0 1
0 0 1 0 0 0 0 0 0 0 0 0 2 0
0 0 0 0 0 0 0 0 1 0 0 0 0 0
0 1 0 1 0 1 1 0 1 1 0 0 0 0
2 0 0 1 1 0 0 0 1 0 0 3 0 0
1 0 0 0 0 1 0 1 0 0 1 0 0 1
0 1 1 0 0 0 1 0 0 0 0 0 0 0
3
OTU1432 0 5 0 0 0 0 0 0 1 0 0 0 0
0 1 0 0 0 0 0 0 0 0 0 0 0 0
0 0 0 0 0 0 0 0 0 0 0 0 0 0
0 0 0 0 0 1 0 0 0 0 0 0 0 0
0 1 0 0 0 0 0 0 0 0 0 0 3 0
0 0 0 0 0 0 0 0 0 0 0 0 0 2

0	0	0	1	0	0	0	0	0	0	0	1	4	0
0	1	1	6	1	4	2	0	1	4	0	0	0	9
4													
OTU12427	0	1	0	0	1	0	0	0	0	1	0	1	0
0	2	0	0	0	0	0	1	2	1	1	0	0	0
1	0	0	1	0	0	0	0	0	0	0	0	1	0
1	0	0	0	0	0	0	0	0	0	0	0	0	1
0	0	0	0	0	1	0	0	0	0	0	0	0	0
0	1	0	0	1	0	0	1	0	0	0	0	0	0
0	0	0	0	0	0	0	1	0	1	0	0	0	0
1	2	0	1	0	0	0	1	0	0	0	0	0	0
2													
OTU3491	0	1	0	0	0	0	0	0	0	0	0	0	0
0	0	0	0	0	0	0	0	0	0	0	3	0	0
0	0	0	0	0	0	0	0	1	0	0	0	0	0
0	0	0	0	0	0	0	0	0	0	0	0	0	0
0	0	0	0	0	0	0	0	0	0	0	0	0	0
0	0	0	0	0	0	0	0	0	0	3	0	0	0
1	0	1	0	0	0	0	0	0	0	0	0	0	0
0	0	0	0	0	0	0	0	0	0	0	0	0	0
0													
OTU196	0	4	17	0	0	0	0	1	4	33	2	20	9
2	0	10	1	0	14	22	32	0	5	2	30	8	40
7	0	7	18	1	12	0	4	0	27	17	0	21	17
3	5	1	7	141	2	0	0	0	1	127	0	0	0
1	0	0	1	0	0	2	3	2	9	0	60	6	1
1	5	1	8	39	0	2	0	2	4	0	51	9	8
0	1	4	0	0	0	2	3	16	289	21	0	8	7
31	0	6	6	0	7	28	0	5	5	14	5	19	0
1													
OTU5496	0	1	2	0	0	0	0	0	0	0	0	0	0
0	0	0	0	0	0	0	0	0	0	0	0	0	0
0	0	0	0	0	0	0	0	0	1	0	0	0	3

0	0	0	0	4	0	0	0	0	0	0	0	0	0
0	0	0	0	0	0	0	0	0	0	0	0	0	0
0	0	0	0	0	0	0	0	0	0	0	0	0	0
0	0	0	0	0	0	0	0	0	0	0	0	0	0
0	0	0	0	0	2	0	0	0	1	0	0	0	0
0													
OTU7801	0	1	0	0	0	0	0	0	0	0	0	0	0
0	0	0	0	0	0	0	0	0	0	0	0	0	0
0	0	0	0	0	0	0	0	0	0	0	0	0	0
0	0	0	0	0	0	0	0	0	0	0	0	0	0
0	0	0	0	0	0	0	0	0	0	0	0	0	0
0	0	0	0	0	0	0	0	0	0	0	0	0	0
0	0	0	0	0	0	0	0	0	0	0	0	0	0
0	0	0	0	0	1	1	0	0	0	0	0	0	0
0													
OTU10978	0	1	0	0	0	0	0	0	0	0	0	0	0
0	0	0	0	0	0	0	0	0	0	0	0	0	0
0	0	0	0	0	0	0	0	0	0	0	0	0	0
0	0	0	0	0	0	0	0	0	0	0	0	0	0
0	0	0	0	0	0	0	0	0	0	0	0	0	0
0	0	0	0	0	0	0	0	0	0	0	0	0	0
0	0	0	0	0	0	0	0	0	0	0	0	0	0
0	0	0	0	0	0	0	0	0	0	0	0	0	0
0													
OTU113	0	5	0	1	0	0	2	3	2	0	0	0	0
0	0	0	0	0	0	0	0	0	0	0	0	1	0
0	0	1	0	0	0	1	0	0	4	6	0	0	0
17	0	0	0	1	0	0	5	0	0	0	2	0	0
1	1	2	0	0	0	0	1	1	0	1	0	2	0
0	79	7	0	0	0	0	4	0	1	7	5	0	0
0	0	0	2	1	1	0	10	0	0	0	11	27	1
0	3	334	130	58	324	115	17	145	26	0	1	0	35
49													

OTU8480 0 6 0 0 0 0 1 0 0 0 0 0 0
0 0 0 0 0 0 0 0 0 0 0 0 1 0
0 1 0 0 0 0 0 0 0 0 0 0 0 0
0 0 0 0 0 0 0 0 0 1 0 0 0 0
0 0 0 0 0 0 1 0 0 0 0 0 0 0
0 0 0 0 0 1 0 0 0 0 0 0 0 3
0 0 0 0 0 0 0 0 0 0 0 0 0 0
0 1 0 0 0 0 0 0 0 0 0 0 0 1
0
OTU9762 0 3 0 1 2 1 0 0 0 0 0 0 0
0 0 0 0 0 0 0 0 0 0 0 0 0 0
0 0 0 0 0 0 0 3 0 1 1 0 0 1
0 0 0 0 0 0 3 0 0 0 0 0 0 1
0 0 0 1 0 0 0 0 1 0 0 0 0 0
0 0 0 2 0 0 0 0 1 0 1 1 2 0
0 0 1 0 0 0 0 0 0 2 2 0 0 0
3 0 0 0 0 0 0 0 0 0 1 0 4 0
0
OTU9214 0 2 1 0 0 0 1 0 5 0 0 2 0
0 0 0 6 0 0 4 0 7 0 37 0 0 0
7 7 0 0 12 0 5 1 0 0 0 0 0 0
3 0 6 1 4 2 0 2 2 12 0 19 1 0
1 0 3 0 3 3 3 22 26 1 2 1 6 1
12 0 0 0 2 0 0 33 0 2 1 13 1 7
0 7 0 2 0 0 0 0 0 0 0 0 0 1
0 1 1 1 0 5 1 0 1 1 1 0 0 0
0
OTU8418 0 1 0 0 2 0 0 0 0 0 1 0 1
0 0 0 0 0 1 0 0 1 0 0 0 0 0
1 0 0 0 0 0 0 0 0 0 0 0 1 0
1 0 1 1 0 0 0 2 0 1 0 0 0 0
0 0 0 1 0 0 0 0 0 0 0 1 0 0
0 0 0 0 0 0 0 0 0 0 0 2 1 1

0	1	0	0	0	1	0	0	0	0	1	0	0	0
0	0	0	0	0	0	0	0	0	1	1	0	0	0
2													
OTU3551	0	3	0	0	3	2	1	1	0	1	0	1	0
1	1	1	0	0	0	1	0	0	1	0	3	0	0
0	0	0	0	0	0	0	0	0	0	2	0	0	0
0	1	0	0	0	0	0	0	0	0	0	0	0	1
0	0	0	0	0	0	0	0	0	0	0	0	0	0
0	0	0	0	0	0	1	0	0	0	0	0	0	0
0	0	0	0	2	0	0	0	0	0	0	0	0	0
1	0	0	0	0	0	0	0	0	0	1	0	0	0
0													
OTU8479	0	1	0	0	0	0	0	0	0	0	0	0	0
0	0	0	0	0	0	0	0	0	0	0	0	0	0
0	0	0	0	0	0	0	0	0	0	0	0	0	0
0	0	0	0	0	0	0	0	0	0	0	0	0	0
0	0	0	0	0	0	0	0	0	0	0	0	0	0
0	0	0	0	0	0	0	0	0	0	0	0	0	0
0	0	0	0	0	0	0	0	0	0	0	0	0	0
0	0	0	0	0	0	0	0	0	0	0	0	0	0
0													
OTU7555	0	3	4	1	0	3	17	11	2	28	0	0	0
0	13	0	4	0	0	8	0	0	0	1	1	4	0
0	0	0	0	2	0	1	0	1	0	2	0	0	0
2	0	0	15	2	0	0	1	0	0	6	0	1	0
0	3	0	3	0	0	0	1	0	0	0	0	3	0
0	0	1	0	1	14	0	1	0	1	0	0	4	9
0	3	0	4	0	2	3	0	0	0	4	0	0	0
0	0	0	0	0	0	0	0	0	0	0	0	0	0
0													
OTU295	0	5	0	0	0	0	0	1	6	0	0	0	0
0	0	0	0	0	0	0	0	0	0	0	1	0	0
0	0	0	0	0	0	2	0	1	0	0	0	1	0

0	0	0	0	0	0	0	0	0	1	0	0	0	0
0	0	0	0	0	1	0	0	0	0	0	0	0	0
0	14	3	0	1	0	0	0	0	1	1	7	0	3
0	1	0	1	0	0	0	3	0	0	0	44	21	0
0	6	6	11	10	28	8	38	6	15	0	2	0	5
0													
OTU14251	0	1	0	0	0	0	0	0	0	0	0	0	0
0	0	0	0	0	0	0	0	0	0	0	0	0	0
0	0	0	0	0	0	0	0	0	0	0	0	0	0
0	0	0	0	0	0	0	0	0	0	0	0	0	0
0	0	0	0	0	0	0	0	0	0	0	0	0	0
0	0	0	0	0	0	0	0	0	0	0	0	0	0
0	0	0	0	0	0	0	0	0	0	0	0	0	0
0	0	0	0	0	0	0	0	0	0	0	0	0	0
0													
OTU1428	0	1	0	0	0	0	0	0	0	0	0	0	0
0	0	0	1	0	0	0	0	2	0	0	0	2	0
0	17	0	0	0	0	0	0	0	0	0	0	2	0
0	0	1	0	0	0	0	0	3	10	0	0	5	0
2	0	1	0	8	9	5	5	1	2	2	1	0	2
0	0	0	0	1	0	0	1	0	0	0	1	0	0
0	4	0	0	0	0	0	0	0	0	0	0	0	0
0	0	0	0	4	1	2	0	0	0	0	0	0	0
0													
OTU251	0	3	0	0	0	0	0	0	0	0	0	0	0
0	0	0	0	0	1	0	0	0	0	0	0	0	0
0	0	0	0	0	1	0	0	0	2	0	0	0	0
1	0	0	0	0	0	0	0	0	0	0	0	0	0
0	0	0	0	0	0	0	0	0	0	0	0	0	0
0	0	0	0	0	0	0	0	0	0	2	0	0	0
0	0	0	0	0	0	0	0	0	0	0	2	0	0
0	8	3	6	21	390	44	3	12	4	0	1	0	2
0													

OTU403	0	2	1	0	1	0	0	0	2	0	0	0	0
0	0	0	1	0	0	0	0	0	0	1	0	0	1
1	0	0	0	0	0	0	0	1	0	0	0	0	0
4	0	0	0	1	3	0	0	0	0	0	0	0	1
1	1	2	3	0	0	0	0	0	0	0	1	1	1
0	0	0	0	0	0	0	0	2	5	0	0	2	1
2	0	0	0	0	1	0	0	0	0	1	0	1	0
1	0	1	0	1	0	0	0	1	5	0	0	0	2
0													
OTU12181	0	1	0	0	0	0	0	0	0	0	0	0	0
0	0	0	0	0	0	0	0	0	0	0	0	0	0
0	0	0	0	0	0	0	0	0	0	0	0	0	0
0	0	0	0	0	0	0	0	0	0	0	0	0	0
0	0	0	0	0	0	0	0	0	0	0	0	0	0
0	0	0	0	0	0	0	0	0	0	0	0	0	0
0	0	0	0	0	0	0	0	0	0	0	0	0	0
0	0	0	0	0	0	0	0	0	0	0	0	0	0
0													
OTU12110	0	1	0	0	0	0	0	2	1	0	0	0	1
0	0	0	1	0	0	0	0	0	0	0	0	0	0
0	0	1	0	0	0	0	1	1	0	0	0	1	0
1	1	0	0	1	0	0	0	1	1	0	0	0	0
0	0	0	0	0	0	0	0	2	0	0	0	0	0
0	0	0	0	0	1	0	0	0	0	0	1	0	0
0	0	0	0	0	0	0	0	0	1	1	0	0	1
0	0	0	0	0	0	1	0	0	0	0	0	0	0
3													
OTU2672	0	5	0	0	0	0	0	0	0	0	0	0	0
0	0	0	0	0	0	0	0	0	0	0	0	0	0
0	0	0	0	0	0	0	0	1	0	0	0	0	0
0	0	0	0	0	0	0	0	0	0	0	0	0	0
0	0	0	0	0	0	0	0	0	0	0	0	1	0
0	0	0	0	0	0	0	0	0	0	0	0	0	0

0	0	0	0	0	0	0	2	0	0	0	2	1	0
0	5	0	0	0	0	2	0	1	1	0	0	0	0
1													
OTU3563	0	3	0	0	0	0	0	3	0	0	0	0	0
0	0	1	0	1	0	3	0	0	0	1	23	0	0
0	1	2	0	22	3	1	0	0	0	4	0	6	2
0	2	0	0	4	0	1	2	0	0	6	0	0	14
0	0	21	1	0	0	0	0	1	2	2	9	2	9
0	0	3	0	0	0	0	0	0	2	0	0	0	0
0	3	5	0	1	0	0	0	3	0	0	0	0	0
0	1	0	1	0	0	0	0	0	0	0	1	0	0
0													
OTU423	0	5	3	0	3	4	0	0	7	5	0	6	0
0	1	5	0	0	3	1	14	0	1	0	0	0	2
0	0	2	0	0	4	0	0	0	0	0	0	0	0
1	1	0	2	0	0	0	0	0	0	0	0	0	0
0	0	0	1	0	0	0	0	0	0	0	0	1	0
0	0	2	0	7	0	0	0	1	1	0	0	2	0
2	0	1	2	0	0	2	0	0	7	0	0	0	0
0	0	0	0	0	0	0	0	0	3	0	0	6	0
0													
OTU4600	0	3	0	0	0	1	0	0	0	0	0	0	0
0	0	0	1	0	0	0	0	0	0	0	0	0	0
0	0	0	0	0	0	0	2	0	0	0	0	0	0
0	0	0	0	0	0	0	0	0	0	0	0	0	0
0	0	0	0	0	0	0	0	0	0	0	0	0	1
0	0	0	1	0	0	0	0	1	0	0	0	0	0
0	0	1	0	0	0	0	0	0	0	0	0	0	0
1	0	0	0	0	0	0	0	0	0	1	0	0	0
0													
OTU2045	0	2	2	41	0	0	3	0	6	2	112	12	122
704	0	16	29	23	726	0	7	0	34	1	5	0	30
0	19	388	118	103	16	5	93	0	3	0	0	0	222

	4	3	183	16	75	5	30	0	135	1	12	2	137	1
	175	0	156	0	2	72	121	60	16	45	56	9	7	31
	65	0	0	7	3	23	107	0	1	3	1	1	3	1
	4	17	21	0	0	4	23	0	368	26	69	1	0	347
	67	20	3	0	0	1	1	1	15	1	0	115	85	0
	1													
OTU8477		0	1	0	0	0	0	0	0	0	0	0	0	0
	0	0	0	0	0	0	0	0	0	0	0	0	0	0
	0	0	0	0	0	0	0	0	0	0	0	0	0	0
	0	0	0	0	0	0	0	0	0	0	0	0	0	0
	0	0	0	0	0	0	0	0	0	0	0	0	0	0
	0	0	0	0	0	0	0	0	0	0	0	0	0	0
	0	0	0	0	0	0	0	0	0	0	0	0	0	0
	0	0	0	0	0	0	0	0	0	0	0	0	0	0
	0													
OTU675		0	1	0	0	0	0	0	2	0	6	0	6	0
	0	0	1	0	0	0	0	0	0	15	0	0	0	0
	0	0	0	0	0	0	0	0	0	0	0	0	0	0
	0	0	0	0	0	0	0	0	0	0	0	0	0	0
	0	0	0	1	0	0	0	0	0	0	0	0	0	0
	0	0	0	5	0	0	0	0	0	0	0	1	0	0
	0	0	2	0	31	0	0	0	0	4	0	0	0	0
	0	0	0	0	0	0	0	0	1	0	0	0	3	0
	0													
OTU12182		0	1	0	0	0	0	0	0	0	0	0	0	0
	0	0	0	0	0	0	0	0	0	0	0	0	0	0
	0	0	0	0	0	0	0	0	0	0	0	0	0	0
	0	0	0	0	0	0	0	0	0	0	0	0	0	0
	0	0	0	0	0	0	0	0	0	0	0	0	0	0
	0	0	0	0	0	0	0	0	0	0	0	0	0	0
	0	0	0	0	0	0	0	0	0	0	0	0	0	0
	0	0	0	0	0	0	0	0	0	0	0	0	0	0
	0													

OTU1005	0	4	0	0	0	0	0	0	0	0	0	0	0
0	0	0	0	0	0	0	0	0	0	0	0	0	0
0	0	0	0	0	0	0	0	0	0	0	0	0	0
0	0	0	0	0	0	0	0	0	0	0	0	0	0
0	0	0	0	0	0	0	0	0	0	0	0	0	0
0	0	0	0	0	0	0	0	0	0	0	0	0	0
0	0	0	0	0	0	0	0	0	0	0	1	1	0
0	0	0	0	0	0	1	0	0	7	0	0	0	0
0													
OTU300	0	1	5	2	1	1	0	1	17	9	0	0	0
1	0	0	1	0	0	16	1	0	0	1	0	1	0
6	0	0	0	0	0	0	0	0	0	0	0	0	0
4	0	2	0	0	0	0	0	0	0	0	1	0	1
0	0	0	3	0	0	0	0	0	0	0	0	12	0
0	22	2	0	0	0	0	1	0	1	3	6	0	2
0	2	0	0	0	1	0	4	0	0	0	0	0	0
0	3	5	2	110	49	22	22	48	85	0	5	0	0
0													
OTU10981	0	1	0	0	0	0	0	0	0	0	0	0	0
0	0	0	0	0	0	0	0	0	0	0	0	0	0
0	0	0	0	0	0	0	0	0	0	0	0	0	0
0	0	0	0	0	0	0	0	0	0	0	0	0	0
0	0	0	0	0	0	0	0	0	0	0	0	0	0
0	0	0	0	0	0	0	0	0	0	0	0	0	0
0	0	0	0	0	0	0	0	0	0	0	0	0	0
0	0	0	0	0	0	0	0	0	0	0	0	0	0
0													
OTU1765	0	1	0	0	0	0	0	0	0	0	0	0	0
0	1	0	0	0	0	0	0	0	0	0	0	0	0
0	0	0	1	0	0	0	0	0	0	0	0	0	0
0	0	0	0	0	0	0	0	0	0	0	0	0	0
0	0	0	0	0	0	0	0	0	0	0	0	0	0
0	0	0	0	0	0	1	0	0	1	0	0	0	2

0	0	0	0	0	0	0	0	0	0	0	0	0	0
0	0	0	0	0	0	0	0	0	0	0	0	0	0
0													
OTU3592	0	1	0	0	0	1	0	0	0	0	0	6	0
0	0	0	2	0	0	0	0	0	0	1	0	7	0
0	0	0	0	0	0	2	9	0	1	3	0	0	0
0	0	0	1	0	0	0	0	0	0	1	0	0	0
1	0	0	0	0	0	1	0	0	0	0	0	0	1
0	0	0	1	0	0	0	0	0	0	0	0	3	0
0	0	1	0	2	0	1	0	1	0	5	0	0	0
3	0	0	0	0	0	0	0	0	0	1	1	5	0
0													
OTU357	0	2	0	0	0	0	0	0	1	1	0	0	0
0	0	0	0	0	0	0	0	0	0	0	0	0	0
0	0	0	0	0	0	0	0	0	0	2	0	0	0
0	0	0	0	0	0	0	0	0	0	0	0	0	0
0	0	0	0	0	0	0	0	0	0	0	0	0	0
0	14	0	0	0	0	0	0	0	0	0	0	0	0
0	0	0	0	0	0	0	0	0	0	0	10	9	0
0	7	28	25	18	31	37	10	50	20	0	2	0	38
1													
OTU3339	0	2	0	1	5	4	1	2	6	1	0	2	2
3	15	2	2	3	0	1	1	2	2	2	2	2	2
1	2	3	1	2	1	2	2	1	1	1	3	6	4
2	2	1	2	1	2	2	0	0	8	1	3	1	1
2	0	3	1	1	1	1	3	1	3	2	2	1	2
4	2	0	1	1	1	2	2	2	2	0	3	1	0
3	1	3	0	3	2	1	0	1	0	2	0	0	0
1	4	0	1	4	6	0	3	2	0	2	0	1	4
12													
OTU8629	0	1	2	0	3	1	0	2	2	4	2	1	1
0	1	2	0	2	0	1	0	1	1	0	0	0	0
0	1	6	0	0	2	0	1	1	2	0	3	1	1

1	0	0	0	1	1	0	0	1	1	1	0	0	2
1	0	0	2	1	1	0	3	0	1	1	1	4	1
1	0	3	0	6	0	0	2	0	1	0	4	0	1
1	3	0	0	0	0	0	1	2	0	1	2	0	1
1	4	5	0	0	1	0	2	0	2	1	0	0	0
3													
OTU14261	0	1	0	0	0	0	0	0	0	0	0	0	0
0	0	0	0	0	0	0	0	0	0	0	0	0	0
0	0	0	0	0	0	0	0	0	0	0	0	0	0
0	0	0	0	0	0	0	0	0	0	0	0	0	0
0	0	0	0	0	0	0	0	0	0	0	0	0	0
0	0	0	0	0	0	0	0	0	0	0	0	0	0
0	0	0	0	0	0	0	0	0	0	0	0	0	0
0	0	0	0	0	0	0	0	0	0	0	0	0	0
0													
OTU12808	0	1	0	1	2	1	1	0	1	0	1	0	0
0	1	0	0	0	1	0	0	0	4	0	1	0	0
0	1	1	1	0	0	0	0	0	0	0	3	0	0
0	0	1	0	0	1	0	0	1	0	0	0	1	4
1	1	1	0	2	0	2	0	0	1	1	0	0	3
1	0	0	0	0	1	0	0	0	0	1	0	0	0
0	0	0	0	0	0	0	0	0	0	0	0	0	0
0	0	0	0	0	0	0	0	0	0	0	0	1	0
0													
OTU12176	0	1	0	0	0	0	0	0	0	0	0	0	0
0	0	0	0	0	0	0	0	0	0	0	0	0	0
0	0	0	0	0	0	0	0	0	0	0	0	0	0
0	0	0	0	0	0	0	0	0	0	0	0	0	0
0	0	0	0	0	0	0	0	0	0	0	0	0	0
0	0	0	0	0	0	0	0	0	0	0	0	0	0
0	0	0	0	0	0	0	0	0	0	0	0	0	0
0	0	0	0	0	0	0	0	0	0	0	0	0	0
0													

OTU620	0	5	0	4	2	0	3	1	3	0	2	0	1
0	1	2	1	1	0	2	1	0	4	0	3	1	1
1	0	0	0	0	3	1	0	0	0	2	3	3	0
1	0	0	3	0	0	1	1	0	0	1	0	0	0
0	0	0	1	0	0	0	2	0	0	0	1	0	0
0	0	0	0	2	2	0	0	0	2	3	0	4	3
3	0	1	0	3	2	1	0	1	0	0	0	0	1
0	0	0	0	0	0	0	0	0	1	0	0	2	0
0													
OTU193	0	4	0	1	0	0	0	3	0	0	0	0	0
0	0	0	0	0	0	0	0	0	0	0	1	0	0
1	0	0	0	0	0	0	0	1	0	0	0	0	0
0	0	0	0	0	0	1	5	0	0	0	1	0	4
0	0	0	0	1	0	0	0	0	0	1	1	0	0
0	4	0	0	0	0	0	0	0	0	10	0	0	0
0	0	0	1	1	0	0	1	0	0	0	3	2	0
0	4	194	61	309	292	253	29	126	15	0	15	0	149
55													
OTU117	0	4	21	24	0	18	69	52	11	44	1	3	7
0	21	0	0	0	19	0	0	0	0	1	0	0	0
0	99	0	0	0	0	0	0	0	8	0	0	27	3
73	83	0	63	0	4	0	26	26	34	0	13	31	1
0	1	1	0	0	0	0	1	1	0	0	0	170	0
12	18	0	6	32	40	1	25	16	81	2	15	15	147
0	3	0	89	0	18	0	0	4	28	40	25	39	0
0	23	27	32	22	14	3	27	13	20	0	1	0	28
19													
OTU14260	0	1	0	0	0	0	0	0	0	0	0	0	0
0	0	0	0	0	0	0	0	0	0	0	0	0	0
0	0	0	0	0	0	0	0	0	0	0	0	0	0
0	0	0	0	0	0	0	0	0	0	0	0	0	0
0	0	0	0	0	0	0	0	0	0	0	0	0	0
0	0	0	0	0	0	0	0	0	0	0	0	0	0

0	0	0	0	0	0	0	0	0	0	0	0	0	0
0	0	0	0	0	0	0	0	0	0	0	0	0	0
0													
OTU8589	0	1	2	0	0	0	0	0	0	0	0	0	0
0	0	0	0	0	0	0	0	0	0	0	0	0	0
0	0	0	0	0	0	0	0	0	0	0	3	0	0
0	0	0	0	0	0	0	0	0	0	0	0	0	0
0	0	0	0	0	0	0	0	0	0	0	0	0	0
0	0	0	0	0	0	0	0	0	0	0	0	0	0
0	0	0	0	0	0	0	1	0	0	0	0	0	0
0	0	0	0	0	0	0	0	0	0	0	0	0	0
0													
OTU6458	0	1	0	5	0	0	0	1	6	0	0	0	2
3	0	1	20	1	0	0	0	12	0	0	0	1	0
2	4	4	0	9	0	7	0	0	0	1	0	0	0
9	0	7	0	1	20	0	2	30	6	5	1	60	1
62	2	6	2	2	12	60	18	9	6	5	0	12	0
8	3	0	0	0	0	0	0	0	0	2	0	0	0
0	1	0	0	0	1	0	1	0	0	0	0	2	37
3	8	60	0	32	42	10	0	72	6	0	0	4	1
1													
OTU1249	0	1	0	0	0	0	0	0	0	2	0	0	0
0	0	0	0	0	0	2	0	0	0	0	1	0	0
0	0	0	0	1	0	0	0	1	0	0	0	0	1
171	0	2	0	0	4	0	0	0	0	0	0	0	0
0	29	0	0	0	0	0	0	27	0	2	0	14	0
0	4	0	0	8	0	0	0	0	1	0	112	0	5
0	0	0	0	0	1	0	0	0	0	0	4	2	0
0	32	17	1	64	70	28	4	8	3	0	3	0	0
7													
OTU6870	0	2	0	0	0	0	0	0	2	0	0	0	0
0	1	2	0	0	0	2	0	0	7	0	0	1	1
10	1	0	0	0	0	1	0	0	0	0	1	0	0

0	26	0	4	0	1	0	0	0	0	0	0	0	0
0	0	2	3	0	0	0	0	0	0	0	0	2	1
0	0	0	0	1	0	2	0	0	0	0	0	0	0
1	0	1	4	24	7	0	0	0	1	0	0	0	0
0	2	0	1	0	0	0	0	0	0	0	0	0	0
0													
OTU12180	0	1	0	0	0	0	0	0	0	0	0	0	0
0	0	0	0	0	0	0	0	0	0	0	0	0	0
0	0	0	0	0	0	0	0	0	0	0	0	0	0
0	0	0	0	0	0	0	0	0	0	0	0	0	0
0	0	0	0	0	0	0	0	0	0	0	0	0	0
0	0	0	0	0	0	0	0	0	0	0	0	0	0
0	0	0	0	0	0	0	0	0	0	0	0	0	0
0	0	0	0	0	0	0	0	0	0	0	0	0	0
0													
OTU1135	0	1	0	0	0	0	0	0	0	0	0	0	0
0	0	0	0	0	0	0	0	0	0	2	0	0	0
0	0	0	0	0	0	0	0	0	0	0	0	0	0
0	2	0	0	0	0	0	0	0	0	0	0	0	0
0	0	0	0	0	0	0	0	0	0	0	0	0	0
0	1	0	0	0	14	0	0	3	0	0	0	0	0
0	0	0	0	0	0	0	0	0	0	0	0	1	0
0	0	0	0	0	2	0	1	0	0	0	0	0	0
0													
OTU8486	0	1	1	0	0	0	0	0	0	0	0	0	0
0	0	0	0	0	0	0	0	0	0	0	0	0	0
0	0	0	0	0	0	0	0	0	0	0	0	0	0
0	0	0	0	0	0	0	0	0	0	0	0	0	0
0	0	0	0	0	0	0	0	0	0	0	0	0	0
0	0	0	0	0	0	0	0	0	0	0	0	0	0
0	0	0	0	0	0	0	0	0	0	0	0	0	0
0	0	0	0	0	0	0	0	0	0	0	0	0	0
0													

OTU8487 0 1 0 0 0 0 0 0 0 0 0 0 0
0 0 0 0 0 0 0 0 0 0 0 0 0 0
0 0 0 0 0 0 0 0 0 0 0 0 0 0
0 0 0 0 0 0 0 0 0 0 0 0 0 0
0 0 0 0 0 0 0 0 0 0 0 0 0 0
0 0 0 0 0 0 0 0 0 0 0 0 0 0
0 0 0 0 0 0 0 0 0 0 0 0 0 0
0 0 0 0 0 0 0 0 0 0 0 0 0 0
0
OTU8485 0 1 0 0 0 0 0 0 0 0 0 0 0
0 0 0 0 0 0 0 0 0 0 0 0 0 0
0 0 0 0 0 0 0 0 0 0 0 0 0 0
0 0 0 0 0 0 0 0 0 0 0 0 0 0
0 0 0 0 0 0 0 0 0 0 0 0 0 0
0 0 0 0 0 0 0 0 0 0 0 0 0 0
0 0 0 0 0 0 0 0 0 0 0 0 0 0
0 0 0 0 0 0 0 0 0 0 0 0 0 0
0
OTU14258 0 1 0 0 0 0 0 0 0 0 0 0 0
0 0 0 0 0 0 0 0 0 0 0 0 0 0
0 0 0 0 0 0 0 0 0 0 0 0 0 0
0 0 0 0 0 0 0 0 0 0 0 0 0 0
0 0 0 0 0 0 0 0 0 0 0 0 0 0
0 0 0 0 0 0 0 0 0 0 0 0 0 0
0 0 0 0 0 0 0 0 0 0 0 0 0 0
0 0 0 0 0 0 0 0 0 0 0 0 0 0
0
OTU12179 0 1 0 0 0 0 0 0 0 0 0 0 0
0 0 0 0 0 0 0 0 0 0 0 0 0 0
0 0 0 0 0 0 0 0 0 0 0 0 0 0
0 0 0 0 0 0 0 0 0 0 0 0 0 0
0 0 0 0 0 0 0 0 0 0 0 0 0 0
0 0 0 0 0 0 0 0 0 0 0 0 0 0

0 0 0 0 0 0 0 0 0 0 0 0 0 0
0 0 0 0 0 0 0 0 0 0 0 0 0 0
0
OTU14259 0 1 0 0 0 1 0 0 0 0 0 0 0
0 0 0 0 0 0 0 0 0 0 0 0 0 0
0 0 0 0 0 0 0 0 0 0 0 0 0 0
0 0 0 0 0 0 0 0 0 0 0 0 0 0
0 0 0 0 0 0 0 0 0 0 0 0 0 0
0 0 0 0 0 0 0 0 0 0 0 0 0 0
0 0 0 0 0 0 0 0 0 0 0 0 0 0
0 0 0 0 0 0 0 0 0 0 0 0 0 0
0
OTU12178 0 1 0 0 0 0 0 0 0 0 0 0 0
0 0 0 0 0 0 0 0 0 0 0 0 0 0
0 0 0 0 0 0 0 0 0 0 0 0 0 0
0 0 0 0 0 0 0 0 0 0 0 0 0 0
0 0 0 0 0 0 0 0 0 0 0 0 0 0
0 0 0 0 0 0 0 0 0 0 0 0 0 0
0 0 0 0 0 0 0 0 0 0 0 0 0 0
0 0 0 0 0 0 0 0 0 0 0 0 0 0
0
OTU8482 0 1 0 0 0 0 0 0 0 0 0 0 0
0 0 0 0 0 0 0 0 0 0 0 0 0 0
0 0 0 0 0 0 0 0 0 0 0 0 0 0
0 0 0 0 0 0 0 0 0 0 0 0 0 0
0 0 0 0 0 0 0 0 0 0 0 0 0 0
0 0 0 0 0 0 0 0 0 0 0 0 0 0
0 0 0 0 0 0 0 0 0 0 0 0 0 0
0 0 0 0 0 0 0 0 0 0 0 0 0 0
0
OTU2751 0 1 0 0 0 0 0 0 0 0 0 0 0
0 0 0 0 0 0 0 0 0 0 0 0 0 0
0 0 0 0 0 0 0 0 0 0 0 1 0 0

0	0	0	0	0	0	0	0	0	0	0	0	0	0
0	0	0	0	0	0	0	0	0	0	0	0	0	0
0	0	0	0	0	0	0	0	0	0	0	0	0	0
0	0	0	0	0	0	0	2	0	0	0	0	0	0
0	0	0	0	0	0	0	0	0	1	0	0	0	0
0													
OTU5158	0	3	69	0	2	0	0	0	0	0	0	0	0
0	0	0	0	0	1	0	0	0	0	0	1	0	0
0	0	0	0	0	0	0	0	0	0	0	9	0	4
0	0	1	0	8	0	0	0	0	0	0	0	0	0
0	0	0	0	0	0	0	0	0	0	0	0	0	0
1	0	0	0	2	0	0	0	0	0	0	0	0	0
0	4	0	1	0	0	0	1	0	0	0	0	0	0
0	0	0	0	0	3	0	0	0	3	0	0	0	0
0													
OTU923	0	1	0	0	1	0	0	0	0	0	0	0	2
0	0	0	0	0	1	0	0	0	1	0	1	0	0
0	0	3	0	0	1	0	3	1	0	0	0	0	3
0	0	0	1	0	0	1	0	0	0	0	0	0	0
0	0	0	0	0	0	0	0	0	0	0	0	0	0
0	0	0	0	0	0	0	0	0	0	1	0	0	0
0	0	0	0	0	0	0	0	0	0	0	0	0	0
0	0	0	0	0	0	0	0	1	0	0	1	0	0
0													
OTU347	0	8	0	0	1	3	7	0	49	7	0	0	0
0	1	0	0	0	0	0	0	0	0	0	0	0	0
0	0	0	0	0	0	0	0	0	0	16	0	0	0
5	0	0	0	0	0	0	0	0	0	1	0	0	0
0	0	0	5	0	0	0	0	0	0	0	0	1	0
0	0	0	0	16	0	0	1	0	4	0	0	0	1
0	0	0	7	0	0	0	1	0	0	0	1	0	0
0	1	0	0	9	1	3	3	2	1	0	0	0	1
2													

OTU11970	0	3	0	0	0	0	0	3	0	0	1	3	6
5	2	0	1	3	2	0	1	0	3	0	10	0	8
0	0	0	2	10	10	0	2	0	2	0	1	0	1
0	1	1	2	0	0	2	0	0	1	0	0	0	2
0	0	0	2	0	0	0	0	0	1	2	0	0	1
6	0	0	7	0	2	1	0	0	0	0	0	0	0
3	0	0	0	0	0	0	0	0	0	1	0	0	5
2	0	0	0	0	0	0	0	0	0	0	0	9	0
0													
OTU14645	0	2	2	3	3	2	5	7	4	2	4	0	2
1	3	1	1	2	2	0	5	1	3	0	1	2	0
0	1	5	3	0	1	2	5	2	1	1	4	2	3
2	1	4	0	2	1	0	0	3	4	1	3	1	2
1	1	1	3	0	3	1	2	0	1	3	0	2	1
1	0	3	1	7	2	0	4	0	0	0	2	1	1
1	0	0	1	2	4	0	2	0	0	0	1	1	1
0	2	2	2	3	2	1	1	2	4	1	0	0	3
2													
OTU8481	0	1	0	1	0	0	0	0	1	0	0	0	0
0	0	0	1	0	1	0	0	0	0	0	0	1	9
0	0	1	1	0	0	0	0	4	0	0	0	0	0
4	0	0	0	3	7	0	6	0	0	1	0	0	0
0	0	2	3	0	0	0	0	0	0	0	3	2	0
0	0	0	1	1	0	0	0	0	0	1	0	0	0
0	0	0	0	0	0	5	0	0	0	0	0	0	0
0	0	0	0	0	0	0	0	0	0	0	0	0	0
0													
OTU1951	0	5	0	0	1	10	2	0	18	0	0	0	0
0	0	0	2	0	0	5	0	11	0	2	0	39	0
18	36	0	0	0	0	0	0	0	0	2	0	17	0
5	0	4	0	1	16	0	4	16	35	2	38	4	3
3	5	1	10	0	12	4	36	47	0	5	0	17	0
11	0	0	0	0	0	0	30	0	2	0	1	0	4

0 87 0 5 0 2 0 14 0 0 0 0 0 0

0 0 0 0 0 0 0 0 0 0 0 0 0 1

0

OTU5835 0 1 0 0 0 0 0 0 2 0 0 0 0

0 0 0 0 0 0 1 1 1 0 0 0 0 1

0 0 0 0 1 0 0 0 0 0 16 0 1 0

0 0 1 0 0 0 0 0 1 1 5 0 0 0

0 0 4 4 0 3 0 1 1 1 0 2 0 10

0 1 0 0 0 0 0 0 0 0 9 3 0 0

0 1 0 0 0 0 0 4 0 0 0 0 1 0

0 0 2 0 0 0 1 0 1 4 0 0 0 0

0

OTU1565 0 6 1 0 0 0 0 0 0 0 0 0 0

0 0 0 0 0 0 0 0 0 0 0 0 0 0

0 0 0 0 0 0 0 0 0 0 0 0 0 0

0 0 0 0 0 0 0 0 0 0 0 0 0 0

0 0 0 0 0 0 0 0 0 0 0 0 1 0

0 0 1 0 0 0 0 0 0 0 0 0 0 0

0 1 0 0 0 0 0 1 0 0 0 0 0 0

0 0 0 0 0 9 0 0 0 0 0 0 0 0

0

OTU164 0 2 0 1 0 0 4 0 0 0 0 1 0

0 0 0 0 0 0 0 0 0 0 0 1 0 0

0 0 1 0 0 0 0 0 0 9 0 0 0 0

0 0 0 0 0 0 0 4 0 0 0 0 0 0

0 0 0 0 0 0 0 0 0 0 0 1 0 0

0 7 0 0 0 0 0 0 0 0 20 1 0 0

0 0 0 0 1 0 0 4 0 0 0 0 1 0

0 2 137 6 11 958 21 0 353 31 0 0 0 3

73

OTU10181 0 1 0 0 0 0 0 0 1 0 0 0 0

0 0 0 0 0 0 0 0 0 0 0 0 1 0

0 0 0 0 0 0 0 0 0 0 0 0 0 0

0	0	0	0	0	0	0	0	0	0	0	0	0	0
1	0	0	0	0	0	0	1	0	0	1	0	0	0
0	0	0	0	0	0	0	1	0	1	0	0	0	0
0	0	0	0	0	0	0	0	0	0	0	0	0	0
0	0	0	0	0	0	0	0	1	0	0	0	0	0
0													
OTU8483	0	1	0	0	0	0	0	0	0	0	0	0	0
0	0	0	0	0	0	0	0	0	0	0	0	0	0
0	0	0	0	0	0	0	0	0	0	0	0	0	0
0	0	0	0	0	0	0	0	0	0	0	0	0	0
0	0	0	0	0	0	0	0	0	0	0	0	0	0
0	0	0	0	0	0	0	0	0	0	0	0	0	0
0	0	0	0	0	0	0	0	0	0	0	0	0	0
0	0	0	0	0	0	0	0	0	0	0	0	0	0
0													
OTU8484	0	1	0	0	0	0	0	0	0	0	0	0	0
0	0	0	0	0	0	0	0	0	0	0	0	0	0
0	0	0	0	0	0	0	0	0	0	0	0	0	0
0	0	0	0	0	0	0	0	0	0	0	0	0	0
0	0	0	0	0	0	0	0	0	0	0	0	0	0
0	0	0	0	0	0	0	0	0	0	0	0	0	0
0	0	0	0	0	0	0	0	0	0	0	0	0	0
0	0	0	0	0	0	0	0	0	0	0	0	0	0
0													
OTU10805	0	1	0	0	0	0	0	0	0	0	0	0	0
0	0	0	0	0	0	0	0	0	0	0	0	0	0
0	0	0	0	0	0	0	0	0	0	0	0	0	0
0	0	0	0	0	0	0	0	0	0	0	0	0	0
0	0	0	0	0	0	0	0	0	0	0	0	0	0
0	0	0	0	0	0	0	0	0	0	0	0	0	0
0	0	0	0	0	0	0	0	0	0	0	0	0	0
0	0	0	0	0	0	0	0	0	0	0	0	0	0
0													

OTU14309	0	1	0	0	0	0	0	0	0	0	0	0	0
0	0	0	0	0	0	0	0	0	0	1	0	0	0
0	0	0	0	0	0	0	0	0	0	0	0	0	0
0	0	0	0	0	0	0	0	0	0	0	0	0	0
0	0	0	0	0	0	0	0	1	0	0	0	0	0
0	0	0	0	0	0	0	0	0	0	0	0	0	0
0	0	0	0	0	0	0	0	0	0	0	0	0	1
0	0	0	0	0	0	0	0	0	0	1	0	0	0
0													
OTU8205	0	2	3	0	4	0	2	3	2	4	0	2	0
1	1	1	1	4	2	0	1	2	3	0	0	1	2
2	0	3	1	1	2	2	1	1	1	3	1	5	1
0	0	2	2	0	1	0	1	2	4	0	2	1	1
1	0	1	3	0	2	0	1	2	1	1	1	2	2
2	0	2	1	5	0	1	2	0	1	0	7	2	1
1	2	1	1	1	2	0	2	1	0	0	0	2	1
0	1	3	0	2	1	0	1	0	2	1	0	0	1
6													
OTU10804	0	1	0	0	0	0	0	0	0	0	0	0	0
0	0	0	0	0	0	0	0	0	0	0	0	0	0
0	0	0	0	0	0	0	0	0	0	0	0	0	0
0	0	0	0	0	0	0	0	0	0	0	0	0	0
0	0	0	0	0	0	0	0	0	0	0	0	0	0
0	0	0	0	0	0	0	0	0	0	0	0	0	0
0	0	0	0	0	0	0	0	0	0	0	0	0	0
0	0	0	0	0	0	0	0	0	0	0	0	0	0
0													
OTU659	0	2	0	1	0	1	0	1	0	0	0	0	0
0	0	0	0	2	0	0	0	0	0	0	0	0	0
0	0	0	0	0	0	0	0	0	0	0	0	0	0
0	1	0	0	0	0	0	0	0	0	0	0	0	2
0	0	0	0	0	0	0	0	0	0	0	9	3	0
0	0	3	3	0	0	0	0	0	0	0	0	0	5

1	0	0	0	5	0	1	0	0	0	0	0	0	0
2	0	0	0	0	0	0	0	0	0	0	2	0	0
0													
OTU9884	0	1	0	0	0	0	0	0	0	0	0	0	0
0	0	0	1	0	0	0	0	0	0	0	0	0	1
0	0	0	1	0	0	0	0	0	0	0	0	0	0
0	0	1	0	0	0	0	0	0	1	0	0	0	0
0	0	0	0	0	1	0	0	0	0	0	0	0	0
1	0	0	0	0	0	0	0	0	0	0	0	0	0
0	0	0	0	0	1	0	0	0	0	0	0	0	0
0	0	0	0	0	0	0	0	0	0	0	0	0	0
0													
OTU8472	0	1	0	0	0	0	0	0	0	0	0	0	0
0	0	0	0	0	0	0	0	0	0	0	0	0	0
0	0	0	0	0	0	0	0	0	0	0	0	0	0
0	0	0	0	0	0	0	0	0	0	0	0	0	0
0	0	0	0	0	0	0	0	0	0	0	0	0	0
0	0	0	0	0	0	0	0	0	0	0	0	0	0
0	0	0	0	0	0	0	0	0	0	0	0	0	0
0	0	0	0	0	0	0	0	0	0	0	0	0	0
0													
OTU14393	0	1	0	0	0	0	0	1	0	0	0	0	0
0	0	0	0	0	0	0	0	0	0	1	0	0	0
0	0	0	0	0	0	1	0	0	0	0	2	0	1
0	0	0	0	0	0	0	0	0	0	0	0	0	0
0	0	0	0	0	0	0	0	0	0	0	0	0	0
0	0	0	0	0	0	0	0	0	0	0	0	0	0
0	0	0	0	0	0	0	0	0	0	0	0	0	0
0	0	0	0	0	0	0	0	0	0	0	1	0	0
0													
OTU570	0	1	0	0	0	0	1	0	0	0	0	0	0
0	0	0	0	0	0	0	0	0	0	0	0	0	0
0	0	0	0	0	0	0	0	0	0	0	0	0	0

0 0 0 0 0 0 0 0 0 0 0 0 0 0
0 0 0 0 0 1 0 0 0 0 0 0 1 0
0 5 0 0 2 0 0 1 0 1 0 40 0 3
0 4 0 1 0 2 0 5 0 0 0 0 1 0
0 2 4 0 0 0 0 0 0 0 0 0 4 0
0
OTU726 0 1 0 0 0 0 0 0 0 0 0 0 0
0 0 0 0 0 0 0 0 0 0 0 0 4 0
0 0 0 0 0 0 0 0 0 0 0 0 0 0
0 0 0 0 0 0 0 1 0 0 0 0 0 0
0 0 0 0 0 0 0 0 0 0 0 0 0 0
0 0 4 0 0 0 0 0 0 1 0 0 0 0
0 0 0 0 0 0 0 1 0 0 0 0 0 0
0 0 16 0 0 0 3 0 0 2 0 0 0 4
0
OTU3578 0 1 0 0 0 0 0 0 0 0 0 0 0
0 0 0 0 0 0 0 0 0 0 0 0 2 0
0 0 0 0 0 0 0 0 0 0 0 0 0 0
0 0 0 0 0 0 0 0 0 0 0 0 0 1
0 0 0 0 0 0 0 0 0 0 0 0 0 0
0 0 0 2 0 0 0 0 0 0 0 0 0 0
0 0 0 0 0 0 1 0 0 0 0 0 0 0
0 0 0 0 0 0 0 0 0 0 0 0 0 0
0
OTU8471 0 1 0 0 0 0 0 0 0 0 0 0 0
0 0 0 0 0 0 0 0 0 0 0 0 0 0
0 0 0 0 0 0 0 0 0 0 0 0 0 0
0 0 0 0 0 0 0 0 0 0 0 0 0 0
0 0 0 0 0 0 0 0 0 0 0 0 0 0
0 0 0 0 0 0 0 0 0 0 0 0 0 0
0 0 0 0 0 0 0 0 0 0 0 0 0 0
0 0 0 0 0 0 0 0 0 0 0 0 0 0
0

OTU12163	0	2	0	0	0	0	0	0	1	0	0	0	0
0	0	0	0	0	0	0	0	0	0	0	0	0	0
1	0	0	0	0	0	0	0	0	0	0	0	0	0
0	0	0	0	0	0	0	0	1	0	0	0	0	0
0	0	0	0	2	0	0	0	0	0	1	0	0	0
0	0	0	0	0	0	0	0	1	0	0	2	0	0
0	0	0	0	0	0	0	0	0	0	0	0	0	0
0	0	0	0	0	0	0	0	0	0	1	0	0	0
0													
OTU10807	0	1	0	0	0	0	0	0	0	0	0	0	0
0	0	0	0	0	0	0	0	0	0	0	0	0	0
0	0	0	0	0	0	0	0	0	0	0	0	0	0
0	0	0	0	0	0	0	0	0	0	0	0	0	0
0	0	0	0	0	0	0	0	0	0	0	0	0	0
0	0	0	0	0	0	0	0	0	0	0	0	0	0
0	0	0	0	0	0	0	0	0	0	0	0	0	0
0	0	0	0	0	0	0	0	0	0	0	0	0	0
0													
OTU4163	0	2	0	0	0	0	1	0	1	0	0	0	0
0	0	0	0	0	0	0	0	0	0	0	0	0	0
0	0	0	0	0	0	0	0	0	0	0	0	0	0
2	0	0	0	0	0	0	0	0	0	0	0	0	0
0	0	0	0	0	0	0	0	0	0	0	0	1	0
0	0	2	0	0	0	0	3	0	1	0	6	0	0
0	0	0	0	0	7	0	0	0	0	0	0	0	0
1	1	7	12	2	8	15	3	5	15	0	0	0	12
2													
OTU330	0	2	13	0	0	6	1	0	11	1	0	0	0
0	1	0	0	0	0	0	0	0	0	0	0	0	0
0	0	0	0	0	0	0	0	0	0	0	0	0	0
0	0	0	0	0	1	0	12	0	0	0	0	0	0
0	32	0	0	0	0	0	0	0	0	0	0	3	0
0	2	7	0	0	0	0	0	0	17	0	0	0	8

0	1	0	16	0	6	0	0	0	1	0	4	4	0
0	0	0	8	0	0	1	4	1	19	0	0	0	0
3													
OTU8473	0	1	0	0	0	0	0	0	0	0	0	0	0
0	0	0	0	0	0	0	0	0	0	0	0	0	0
0	0	0	0	0	0	1	0	0	0	2	0	0	0
0	0	0	0	0	0	0	0	0	0	0	0	0	0
0	0	0	0	0	0	0	0	0	0	0	0	0	0
0	0	0	0	0	0	0	0	0	0	0	0	0	0
0	0	0	0	0	0	0	0	0	0	1	0	0	0
0	0	0	0	0	0	0	0	0	0	0	0	0	0
0													
OTU112	0	1	4	0	0	0	0	0	0	0	0	0	0
0	0	0	1	0	0	0	0	3	0	0	0	0	0
1	39	0	0	0	0	0	0	0	0	3	0	0	0
0	0	14	0	0	0	0	0	29	66	0	62	0	1
10	0	0	0	0	183	2	56	168	0	47	0	0	0
55	1	0	0	0	0	0	266	0	0	0	96	0	1
0	8	0	0	0	0	0	0	0	0	0	0	0	0
0	0	14	4	21	37	26	0	13	1	0	1	0	4
1													
OTU5436	0	3	0	0	0	0	1	0	0	0	0	0	0
0	0	0	0	0	0	1	0	0	0	0	0	0	0
0	0	0	0	0	0	0	0	0	0	38	0	0	0
0	0	0	0	0	0	0	0	0	0	0	0	0	0
0	0	0	0	0	0	0	0	0	1	0	0	0	0
0	0	1	0	2	0	0	0	0	0	0	3	0	1
0	0	0	1	0	0	0	0	0	0	0	0	0	0
0	0	2	1	2	3	2	3	2	1	0	0	0	0
0													
OTU444	0	9	0	0	0	0	0	0	2	0	0	1	0
0	0	0	0	0	0	0	0	0	0	0	5	0	0
1	0	0	0	0	0	0	0	0	0	0	0	0	0

0	0	0	0	0	0	0	0	0	0	0	0	0	0
0	3	0	1	0	0	0	0	0	0	0	0	0	0
0	14	0	0	6	0	0	0	0	0	0	1	0	1
0	0	0	1	0	0	0	0	0	0	0	1	14	0
0	0	4	3	1	6	7	7	27	4	0	0	0	12
0													
OTU7573	0	1	0	0	4	0	2	2	0	2	0	0	1
2	4	3	0	1	1	1	0	0	1	0	2	0	0
1	1	1	2	0	1	0	2	0	0	0	1	8	0
2	2	1	1	0	3	0	0	2	6	0	0	2	0
1	1	1	0	0	3	1	2	0	3	0	1	3	0
0	2	1	0	1	1	0	1	0	1	0	4	0	2
1	0	0	0	1	3	2	0	0	0	1	0	2	0
0	2	2	3	0	0	1	1	1	0	0	0	0	1
2													
OTU2395	0	2	0	0	0	0	0	0	0	0	0	4	0
0	0	0	0	0	0	0	0	0	0	1	0	1	0
0	0	0	0	0	0	1	0	1	1	3	0	0	0
0	0	0	0	0	0	0	0	0	0	1	0	0	0
0	0	0	0	0	0	0	0	0	0	0	2	0	1
0	0	0	0	0	0	1	0	2	0	2	0	0	0
0	0	0	0	0	0	1	0	0	0	0	0	0	0
0	0	0	0	0	0	0	0	0	0	0	0	1	0
0													
OTU12258	0	1	1	0	1	0	0	0	0	0	0	0	0
0	0	0	0	0	0	0	0	0	0	0	0	0	0
0	0	0	0	0	1	0	0	0	0	0	0	1	0
0	0	0	0	0	0	0	0	0	0	0	0	0	0
0	0	0	0	0	0	0	0	0	0	0	0	0	0
0	0	0	0	0	0	0	0	0	0	0	0	0	0
0	0	0	0	0	0	0	0	0	0	0	0	0	0
0	1	0	0	0	0	0	0	0	0	0	0	0	0
0													

OTU14264	0	1	0	0	1	0	0	1	0	0	0	1	0
0	0	0	0	0	0	0	0	0	0	0	0	0	0
0	0	0	0	0	0	1	1	0	0	0	0	0	0
0	0	0	0	0	0	1	0	0	0	0	0	0	0
0	0	0	0	0	0	0	0	0	0	0	0	0	0
0	0	0	0	0	0	1	0	0	1	0	0	0	0
0	0	0	0	0	0	0	0	0	0	0	0	0	0
0	0	0	0	0	0	0	0	0	0	0	0	0	0
0													
OTU1128	0	3	0	0	3	2	1	0	0	1	0	8	0
0	1	1	1	1	0	4	0	0	0	0	0	2	0
3	0	0	0	0	1	0	0	3	0	0	0	23	0
14	0	0	0	0	2	0	10	0	0	0	0	0	2
0	3	0	1	0	0	0	0	0	0	0	0	2	0
0	8	4	0	0	1	0	0	2	8	0	1	0	1
0	0	0	0	0	2	0	6	0	1	0	10	16	0
1	1	1	11	0	0	0	5	2	5	2	4	0	12
4													
OTU12160	0	1	0	0	0	0	0	0	0	0	0	0	0
0	0	0	0	0	0	0	0	0	0	0	0	0	0
0	0	0	0	0	0	0	0	0	0	0	0	0	0
0	0	0	0	0	0	0	0	0	0	0	0	0	0
0	0	0	0	0	0	0	0	0	0	0	0	0	0
0	0	0	0	0	0	0	0	0	0	0	0	0	0
0	0	0	0	0	0	0	0	0	0	0	0	0	0
0	0	0	0	0	0	0	0	0	0	0	0	0	0
0													
OTU3480	0	1	0	0	0	0	0	0	2	2	0	0	0
0	0	0	0	0	0	0	0	0	0	0	0	0	0
0	0	0	0	0	0	0	0	0	0	0	0	0	0
0	0	0	0	0	0	0	0	0	0	0	0	0	0
0	0	0	0	0	0	0	0	0	0	0	0	0	0
0	0	0	0	0	0	0	0	0	0	0	0	0	0

0	0	0	0	0	0	0	0	0	0	0	2	0	0
0	0	1	2	1	0	1	1	1	0	0	0	0	2
0													
OTU775	0	1	0	0	0	0	0	0	0	0	0	0	0
0	0	0	0	0	0	0	0	0	0	0	0	0	0
0	0	0	0	0	0	0	0	0	0	0	0	0	0
0	0	0	0	0	0	0	0	0	0	0	0	0	0
0	0	0	0	0	0	0	0	0	0	0	0	0	0
0	0	3	0	0	0	0	0	0	0	0	0	0	0
0	0	0	0	0	0	0	1	0	0	0	0	0	0
0	1	0	0	0	0	0	4	0	0	0	0	0	0
0													
OTU12162	0	1	0	0	0	0	0	0	0	0	0	0	0
0	0	0	0	0	0	0	0	0	0	0	0	0	0
0	0	0	0	0	0	0	0	0	0	0	0	0	0
0	0	0	0	0	0	0	0	0	0	0	0	0	0
0	0	0	0	0	0	0	0	0	0	0	0	0	0
0	0	0	0	0	0	0	0	0	0	0	0	0	0
0	0	0	0	0	0	0	0	0	0	0	0	0	0
0	0	0	0	0	0	0	0	0	0	0	0	0	0
0													
OTU4579	0	1	0	0	3	0	0	0	1	0	0	0	0
0	0	0	0	1	0	0	0	0	0	0	0	0	0
0	0	0	0	0	0	0	0	0	0	0	0	0	0
0	0	0	0	0	0	0	1	0	0	0	0	0	0
0	1	0	0	0	0	0	0	0	0	0	0	0	0
0	0	0	0	0	0	0	0	0	0	0	0	0	0
0	0	0	0	0	0	0	0	0	0	0	0	0	0
0	0	0	0	0	0	0	0	2	0	0	0	0	0
0													
OTU12120	0	1	1	0	3	1	1	0	0	0	0	0	0
1	2	0	0	0	0	0	0	0	0	1	0	0	0
0	0	1	0	0	0	0	0	0	0	0	0	0	0

0	0	0	0	0	0	0	0	0	0	0	0	0	0
0	0	0	0	0	0	0	0	0	0	0	0	0	0
0	0	1	1	0	2	0	0	1	0	0	0	1	0
0	0	0	0	0	0	0	0	0	0	0	0	0	0
0	0	0	0	0	0	0	0	0	0	1	0	0	0
0													
OTU385	0	2	0	0	0	0	2	0	9	0	0	0	0
0	0	5	0	0	0	0	0	0	1	0	1	0	0
0	0	1	0	0	0	0	0	0	2	2	14	0	0
0	0	0	5	0	0	18	1	0	0	0	0	0	0
0	0	0	0	0	0	0	0	0	0	0	2	0	0
0	2	4	0	0	0	0	1	0	2	16	0	0	0
0	1	0	0	0	0	0	2	0	0	0	2	2	0
0	2	9	6	5	5	6	8	5	2	1	0	0	5
0													
OTU1221	0	1	0	0	0	0	0	0	0	0	0	0	0
0	0	0	0	0	0	0	0	0	0	0	0	0	0
0	0	0	0	0	0	0	0	0	0	1	0	0	0
0	0	0	0	0	0	0	0	0	0	0	0	0	0
0	0	0	0	0	0	0	0	0	0	0	0	0	0
0	1	0	0	0	0	0	0	0	0	0	0	0	0
0	0	0	0	0	0	0	0	0	0	0	3	2	0
0	1	0	0	3	2	0	1	0	0	0	0	0	0
0													
OTU15661	0	1	0	0	0	0	0	1	0	0	0	2	5
0	0	0	0	0	0	1	0	0	0	0	0	0	0
0	0	0	0	0	0	0	1	0	0	0	0	0	0
2	0	0	1	0	0	0	0	0	0	0	0	0	0
0	0	0	0	0	0	0	0	0	0	0	0	0	0
0	0	0	0	1	0	1	0	0	1	0	0	0	0
0	0	0	0	1	0	0	0	0	0	1	0	0	0
0	0	0	1	0	0	0	0	0	0	2	0	2	0
0													

OTU7920	0	1	17	0	0	0	0	0	0	0	0	0	0
0	0	0	0	0	0	0	0	0	0	0	0	0	0
0	0	0	0	0	0	0	0	0	0	0	1	0	0
0	0	0	0	4	0	0	0	0	0	0	0	0	0
0	0	0	0	0	0	0	0	0	0	0	0	0	0
1	0	0	0	0	0	0	0	0	0	0	0	0	0
0	1	0	0	0	0	0	1	0	0	0	0	0	0
0	0	0	0	0	5	0	0	0	0	0	0	0	0
0													
OTU1724	0	2	0	0	2	6	0	0	0	0	0	2	0
1	2	0	0	0	0	2	2	0	0	0	0	0	0
0	0	0	0	0	1	0	3	0	2	0	0	0	4
0	0	0	0	0	0	0	0	0	0	1	0	0	0
1	0	0	1	0	0	0	0	0	0	0	0	1	0
0	0	1	10	0	0	1	0	16	0	0	1	0	0
0	0	4	0	1	0	11	1	2	1	0	0	0	0
10	0	0	0	0	0	0	0	1	0	4	6	5	0
0													
OTU12161	0	1	0	0	0	0	0	0	0	0	0	0	0
0	0	0	0	0	0	0	0	0	0	0	0	0	0
0	0	0	0	0	0	0	0	0	0	0	0	0	0
0	0	0	0	0	0	0	0	0	0	0	0	0	0
0	0	0	0	0	0	0	0	0	0	0	0	0	0
0	0	0	0	0	0	0	0	0	0	0	0	0	0
0	0	0	0	0	0	0	0	0	0	0	0	0	0
0	0	0	0	0	0	0	0	0	0	0	0	0	0
0													
OTU8469	0	1	0	0	0	0	0	0	0	0	0	0	0
0	0	0	0	0	0	0	0	0	0	0	0	0	0
0	0	0	0	0	0	0	0	0	0	0	0	0	0
0	0	0	0	0	0	0	0	0	0	0	0	0	0
0	0	0	0	0	0	0	0	0	0	0	0	0	0
0	0	0	0	0	0	0	0	0	0	0	0	0	0

0	0	0	0	0	0	0	0	0	0	0	0	0	0
0	0	0	0	0	0	0	0	0	0	0	0	0	0
0													
OTU8423	0	4	1	1	2	2	2	2	2	4	1	2	0
0	3	0	2	7	5	1	0	3	0	1	3	0	1
1	1	5	2	0	0	0	8	0	2	0	0	8	1
5	2	1	2	2	2	1	0	0	5	0	1	2	2
2	2	3	0	1	0	1	1	3	2	0	0	2	0
3	2	3	2	6	2	0	3	0	1	0	5	1	0
3	2	0	0	0	2	0	2	1	0	4	3	0	2
2	3	1	0	2	1	0	3	0	2	1	1	2	5
7													
OTU14263	0	1	0	0	0	0	0	0	0	0	0	0	0
0	0	0	0	0	0	0	0	0	0	0	0	0	0
0	0	0	0	0	0	0	0	0	0	0	0	0	0
0	0	0	0	0	0	0	0	0	0	0	0	0	0
0	0	0	0	0	0	0	0	0	0	0	0	0	0
0	0	0	0	0	0	0	0	0	0	0	0	0	0
0	0	0	0	0	0	0	0	0	0	0	0	0	0
0	0	0	0	0	0	0	0	0	0	0	0	0	0
0													
OTU4141	0	2	0	0	0	0	0	0	0	0	0	0	0
0	1	0	0	0	0	1	0	0	0	0	0	0	0
0	0	0	0	0	0	0	0	0	0	0	0	0	0
0	0	0	0	0	0	0	0	0	0	0	0	0	0
0	0	0	0	0	0	0	0	0	0	0	0	0	0
0	0	0	0	0	0	0	0	0	0	0	0	0	0
0	0	0	0	0	0	0	0	0	0	0	0	0	0
0	0	0	0	0	0	0	0	0	0	0	0	0	0
0													
OTU190	0	1	0	4	0	0	2	1	0	2	0	8	4
3	1	1	115	0	2	1	1	91	0	4	3	4	13
6	181	0	0	46	2	9	5	0	0	5	2	2	0

	0	0	6	0	1	0	0	0	3	3	6	0	1	0
	3	0	12	0	13	2	3	6	4	9	7	17	1	23
	17	2	0	0	0	1	0	3	1	0	2	2	2	0
	0	8	0	0	1	0	8	0	0	0	1	0	1	0
	0	1	4	1	0	2	2	0	4	3	1	6	35	1
	0													
OTU4088		0	4	0	0	0	0	0	0	3	0	0	0	1
	0	0	0	0	0	0	0	3	1	0	0	0	0	0
	0	0	0	0	0	0	0	0	0	0	0	0	0	0
	0	0	0	0	0	0	0	0	0	0	0	1	0	0
	0	0	0	0	0	0	0	0	0	0	0	0	0	0
	0	0	1	0	0	0	0	2	0	0	0	0	0	2
	0	1	0	0	0	0	0	0	0	0	0	0	0	0
	0	0	0	0	0	0	0	0	0	0	0	0	0	0
	0													
OTU14262		0	1	0	0	0	0	0	0	0	0	0	0	0
	0	0	0	0	0	0	0	0	0	0	0	0	0	0
	0	0	0	0	0	0	0	0	0	0	0	0	0	0
	0	0	0	0	0	0	0	0	0	0	0	0	0	0
	0	0	0	0	0	0	0	0	0	0	0	0	0	0
	0	0	0	0	0	0	0	0	0	0	0	0	0	0
	0	0	0	0	0	0	0	0	0	0	0	0	0	0
	0	0	0	0	0	0	0	0	0	0	0	0	0	0
	0													
OTU10815		0	1	0	0	0	0	0	0	0	0	0	0	0
	0	0	0	0	0	0	0	0	0	0	0	0	0	0
	0	0	0	0	0	0	0	0	0	0	0	0	0	0
	0	0	0	0	0	0	0	0	0	0	0	0	0	0
	0	0	0	0	0	0	0	0	0	0	0	0	0	0
	0	0	0	0	0	0	0	0	0	0	0	0	0	0
	0	0	0	0	0	0	0	0	0	0	0	0	0	0
	0	0	0	0	0	0	0	0	0	0	0	0	0	0
	0													

OTU1603	0	2	0	0	0	0	0	0	0	0	0	0	0
0	0	0	0	0	0	0	1	0	1	0	0	0	0
0	0	0	0	0	0	0	0	0	0	1	0	0	0
0	0	0	0	0	0	0	0	0	0	0	0	0	0
0	0	0	0	0	0	1	0	1	0	0	0	0	0
0	0	0	1	0	0	0	1	0	0	0	0	1	0
2	0	0	0	0	0	0	0	0	2	0	0	0	0
0	0	0	0	0	0	0	0	0	0	1	0	0	0
0													
OTU6439	0	1	0	0	0	0	0	0	0	0	0	0	0
0	0	0	0	0	0	0	0	0	0	0	0	0	0
0	0	0	0	0	0	0	0	0	0	0	0	0	0
0	0	0	0	0	0	0	0	0	0	0	0	0	0
0	1	0	0	0	0	0	0	0	0	0	0	0	0
0	0	0	0	0	0	0	0	0	0	0	0	0	0
0	0	0	0	0	0	0	0	0	0	0	0	0	0
0	0	0	0	0	0	0	0	0	0	0	0	0	0
0													
OTU1826	0	1	0	0	0	0	0	0	0	0	0	1	1
0	0	1	0	0	0	0	0	0	0	0	0	0	2
0	0	0	0	1	3	0	0	0	0	0	0	0	0
0	0	0	0	0	0	0	0	0	0	0	0	0	1
0	0	0	0	0	0	0	0	0	1	0	1	0	0
0	0	0	0	0	0	1	0	0	0	0	0	0	0
1	0	0	0	0	0	1	0	0	1	0	0	0	0
0	0	0	0	0	0	0	0	0	0	2	2	1	0
0													
OTU10020	0	3	0	0	0	0	0	0	0	0	0	0	0
0	0	1	0	0	0	0	0	0	0	0	0	0	0
0	0	0	0	0	0	0	0	0	0	0	0	0	0
0	0	0	0	0	0	0	0	0	0	0	0	0	0
0	0	0	0	0	0	0	0	0	0	0	0	0	0
0	0	0	0	0	0	0	1	0	0	0	0	0	0

0	2	0	0	0	0	0	0	0	0	0	0	1	0
0	0	2	0	0	1	0	0	0	0	0	0	0	0
0													
OTU536	0	3	0	3	3	0	1	1	2	0	0	0	0
0	3	0	0	6	0	3	0	0	0	0	0	0	0
3	0	0	0	0	0	0	0	0	0	0	0	0	0
3	0	0	1	0	0	0	3	0	0	0	0	0	0
0	0	0	0	0	0	0	1	0	0	0	0	1	0
0	0	0	0	0	4	0	1	0	0	0	0	2	1
0	0	0	3	0	1	0	2	1	0	0	0	0	0
0	2	0	2	5	3	0	1	0	1	1	0	0	0
0													
OTU4815	0	1	1	0	0	0	0	0	0	2	0	0	0
0	0	0	0	0	0	0	0	0	0	0	0	2	0
1	0	0	0	0	0	0	0	0	0	1	0	0	1
1	0	0	0	0	1	0	3	0	0	0	0	0	0
0	0	0	1	0	0	0	0	0	0	0	0	2	0
0	0	0	0	0	0	0	0	0	0	1	0	0	0
0	0	0	0	1	0	0	0	0	0	0	0	1	0
0	0	0	0	0	0	0	2	0	1	0	0	0	0
1													
OTU1201	0	1	0	0	1	0	0	1	0	0	0	0	0
0	0	0	0	0	0	0	0	0	0	0	0	0	1
0	0	0	0	0	0	0	0	0	0	0	0	0	0
0	0	0	0	0	0	0	0	0	0	0	0	0	0
0	0	0	0	0	0	0	1	0	0	0	0	0	0
0	2	0	0	0	0	0	0	0	0	2	0	0	0
0	0	0	0	0	0	0	0	0	0	0	0	1	0
0	7	0	19	2	4	71	0	16	0	1	1	0	39
1													
OTU8476	0	1	0	0	0	0	0	0	0	0	0	0	0
0	0	0	0	0	0	0	0	0	0	0	0	0	0
0	0	0	0	0	0	0	0	0	0	0	0	0	0

0	0	0	0	0	0	0	0	0	0	0	0	0	0
0	0	0	0	0	0	0	0	0	0	0	0	0	0
0	0	0	0	0	0	0	0	0	0	0	0	0	0
0	0	0	0	0	0	0	0	0	0	0	0	0	0
0	0	0	0	0	0	0	0	0	0	0	0	0	0
0													
OTU4420	0	1	0	0	0	0	0	0	0	13	10	0	0
0	0	0	0	0	8	2	320	0	0	1	0	1	186
13	3	0	0	0	0	0	0	0	1	0	0	2	0
0	37	0	0	1	0	0	0	0	0	5	2	0	0
0	20	0	0	0	0	0	1	1	0	2	0	0	0
0	0	1	0	0	0	0	1	0	0	0	4	0	1
0	1	0	3	0	0	0	0	0	0	0	0	1	0
0	5	2	3	0	1	1	13	1	4	0	0	0	12
0													
OTU6689	0	5	0	0	0	1	0	1	0	0	0	1	0
0	0	0	0	0	0	0	0	1	0	0	0	0	0
0	1	0	0	0	0	0	0	0	0	0	0	0	0
1	0	0	1	0	0	0	0	0	0	0	0	0	0
0	0	0	2	0	0	1	0	0	0	0	1	0	1
0	0	1	2	0	0	0	0	2	1	2	0	1	0
1	0	1	0	2	0	5	0	0	0	0	0	0	0
1	0	0	0	0	0	0	0	0	0	4	5	5	0
0													
OTU10818	0	1	0	0	0	0	0	0	0	0	0	0	0
0	0	0	0	0	0	0	0	0	0	0	0	0	0
0	0	0	0	0	0	0	0	0	0	0	0	0	0
0	0	0	0	0	0	0	0	0	0	0	0	0	0
0	0	0	0	0	0	0	0	0	0	0	0	0	0
0	0	0	0	0	0	0	0	0	0	0	0	0	0
0	0	0	0	0	0	0	0	0	0	0	0	0	0
0	0	0	0	0	0	0	0	0	0	0	0	0	0
0													

OTU12172	0	1	0	0	0	0	0	0	0	0	0	0	0
0	0	0	0	0	0	0	0	0	0	0	0	0	0
0	0	0	0	0	0	0	0	0	0	0	0	0	0
0	0	0	0	0	0	0	0	0	0	0	0	0	0
0	0	0	0	0	0	0	0	0	0	0	0	0	0
0	0	0	0	0	0	0	0	0	0	0	0	0	0
0	0	0	0	0	0	0	0	0	0	0	0	0	0
0	0	0	0	0	0	0	0	0	0	0	0	0	0
0													
OTU429	0	1	0	0	1	0	0	1	3	0	0	0	0
0	0	0	0	0	0	0	0	0	0	0	8	0	0
0	0	0	0	0	0	0	0	0	0	1	0	0	0
0	0	0	0	0	0	0	1	0	0	0	0	0	1
0	0	0	0	0	0	0	0	0	0	0	0	0	0
0	0	0	0	0	0	0	0	0	0	0	0	0	0
0	0	0	0	0	0	0	0	0	0	0	1	1	0
0	0	6	24	27	3	10	0	0	7	0	1	0	5
0													
OTU102	0	3	8	0	1	10	3	2	10	0	13	2	92
116	4	24	1	21	75	8	3	0	0	6	2	3	33
2	1	2	242	0	19	0	322	0	47	2	2	5	98
4	0	2	0	306	8	79	1	0	0	323	2	0	3
1	5	25	19	3	4	4	9	2	5	0	251	5	3
5	0	0	39	0	0	19	0	0	0	0	0	2	0
0	8	0	0	3	1	1	1	12	8	53	0	0	66
34	0	0	0	0	3	0	0	1	0	4	1	20	0
2													
OTU609	0	1	0	0	0	0	0	0	1	0	0	0	0
0	0	0	0	0	0	0	0	0	0	0	0	0	0
0	0	0	0	0	0	0	0	0	0	0	0	0	0
0	0	0	0	0	0	0	0	0	0	0	0	0	0
0	0	0	0	0	0	0	0	0	0	0	3	0	0
0	0	0	0	0	0	0	0	0	0	0	0	0	0

0	0	0	1	0	0	0	0	0	0	0	0	3	0
1	2	0	3	0	2	0	5	1	0	0	0	0	1
0													
OTU9270	0	1	0	0	0	0	0	0	0	0	0	0	0
0	0	0	0	0	2	0	0	0	0	0	0	0	0
0	0	0	0	0	1	0	0	0	0	0	2	0	0
0	0	0	0	0	0	0	0	0	0	0	0	0	0
0	1	0	0	0	0	0	0	0	0	0	0	0	0
0	0	0	0	0	0	0	0	0	0	0	0	0	0
0	0	0	0	0	0	0	0	0	0	1	0	0	0
0	0	0	0	0	0	0	0	0	0	0	0	0	0
0													
OTU10817	0	1	0	0	0	0	0	0	0	0	0	0	0
0	0	0	0	0	0	0	0	0	0	0	0	0	0
0	0	0	0	0	0	0	0	0	0	0	0	0	0
0	0	0	0	0	0	0	0	0	0	0	0	0	0
0	0	0	0	0	0	0	0	0	0	0	0	0	0
0	0	0	0	0	0	0	0	0	0	0	0	0	0
0	0	0	0	0	0	0	0	0	0	0	0	0	0
0	0	0	0	0	0	0	0	0	0	0	0	0	0
0													
OTU10816	0	1	0	0	0	0	0	0	0	0	0	0	0
0	0	0	0	0	0	0	0	0	0	0	0	0	0
0	0	0	0	0	0	0	0	0	0	0	0	0	0
0	0	0	0	0	0	0	0	0	0	0	0	0	0
0	0	0	0	0	0	0	0	0	0	0	0	0	0
0	0	0	0	0	0	0	0	0	0	0	0	0	0
0	0	0	0	0	0	0	0	0	0	0	0	0	0
0	0	0	0	0	0	0	0	0	0	0	0	0	0
0													
OTU14253	0	1	0	0	0	0	0	0	0	0	0	0	0
0	0	0	0	0	0	0	0	0	0	0	0	0	0
0	0	0	0	0	0	0	0	0	0	0	0	0	0

0	0	0	0	0	0	0	0	0	0	0	0	0	0
0	0	0	0	0	0	0	0	0	0	0	0	0	0
0	0	0	0	0	0	0	0	0	0	0	0	0	0
0	0	0	0	0	0	0	0	0	0	0	0	0	0
0	0	0	0	0	0	0	0	0	0	0	0	0	0
0													
OTU14638	0	1	0	1	1	0	1	0	0	0	1	0	0
0	0	1	0	1	0	0	0	0	2	0	1	0	0
0	0	2	1	0	1	1	0	0	0	0	1	0	0
0	0	0	0	2	0	0	0	1	0	1	0	2	0
0	2	0	0	0	0	0	0	0	0	0	0	0	0
1	0	0	1	0	0	0	0	0	0	0	0	0	0
0	0	1	0	0	0	0	0	0	0	0	0	0	1
0	0	0	0	0	0	0	1	0	0	0	0	0	0
0													
OTU12171	0	1	0	0	0	0	0	0	0	0	0	0	0
0	0	0	0	0	0	0	0	0	0	0	0	0	0
0	0	0	0	0	0	0	0	0	0	0	0	0	0
0	0	0	0	0	0	0	0	0	0	0	0	0	0
0	0	0	0	0	0	0	0	0	0	0	0	0	0
0	0	0	0	0	0	0	0	0	0	0	0	0	0
0	0	0	0	0	0	0	0	0	0	0	0	0	0
0	0	0	0	0	0	0	0	0	0	0	0	0	0
0													
OTU7538	0	1	0	0	0	0	0	1	0	0	0	0	0
0	0	0	0	0	0	0	0	0	0	0	0	0	0
0	0	0	0	0	0	0	0	0	0	0	0	0	0
0	12	0	0	0	0	0	0	0	0	0	0	0	0
0	0	0	0	0	0	0	0	0	0	0	0	0	0
0	0	0	1	0	0	0	0	0	0	0	0	1	0
0	0	0	0	1	0	0	0	0	1	1	0	0	0
4	0	0	0	0	0	0	0	0	0	0	0	0	0
0													

OTU12164	0	1	0	0	0	0	0	0	0	0	0	0	0
0	0	0	0	0	0	0	0	0	0	0	0	0	0
0	0	0	0	0	0	0	0	0	0	0	0	0	0
0	0	0	0	0	0	0	0	0	0	0	0	0	0
0	0	0	0	0	0	0	0	0	0	0	0	0	0
0	0	0	0	0	0	0	0	0	0	0	0	0	0
0	0	0	0	0	0	0	0	0	0	0	0	0	0
0	0	0	0	0	0	0	0	0	0	0	0	0	0
0													
OTU10809	0	1	0	0	0	0	0	0	0	0	0	0	0
0	0	0	0	0	0	0	0	0	0	0	0	0	0
0	0	0	0	0	0	0	0	0	0	0	0	1	0
0	0	0	0	0	0	0	0	0	0	0	0	0	0
0	0	0	0	0	0	0	0	0	0	0	0	0	0
0	0	0	0	0	0	0	0	0	0	0	0	0	0
0	0	0	0	0	0	0	0	0	0	0	0	0	0
0	0	0	0	0	0	0	0	0	0	0	0	0	0
0													
OTU15745	0	1	0	0	0	1	0	0	0	0	0	0	0
0	0	0	0	0	0	0	0	0	0	0	0	0	0
0	0	0	0	0	0	0	0	0	0	0	0	0	0
0	0	0	0	0	0	0	0	0	0	0	0	0	0
0	0	0	0	0	0	0	0	0	0	0	1	0	0
0	0	0	0	0	0	0	0	0	0	0	0	0	0
0	0	0	0	0	0	0	0	0	0	0	0	0	0
0	0	0	0	0	0	0	0	0	0	0	0	0	0
0													
OTU15534	0	1	0	1	0	0	0	2	2	0	12	0	1
0	0	6	0	6	3	0	1	1	0	0	0	0	1
0	0	2	6	0	0	0	0	0	2	0	0	0	0
0	0	1	0	0	0	0	0	0	0	2	0	0	0
0	1	0	1	0	0	0	2	0	0	0	0	1	0
0	0	0	0	0	0	0	0	0	0	0	0	0	0

0	0	0	0	0	0	0	0	0	0	0	0	0	0
0	0	4	0	0	0	0	0	0	2	0	0	0	0
0													
OTU10787	0	1	1	0	0	0	0	0	0	0	0	0	0
0	0	0	0	0	0	0	0	0	0	0	0	0	0
0	0	0	0	0	0	0	0	0	0	0	0	0	0
0	0	0	0	0	0	0	0	0	0	0	0	0	0
0	0	0	0	0	0	0	0	0	0	0	0	0	0
0	0	0	0	0	0	0	0	0	0	0	0	0	0
0	0	0	0	0	0	0	0	0	0	0	0	0	0
0	0	0	0	0	0	0	0	0	0	0	0	0	0
0													
OTU6390	0	1	0	0	0	0	0	0	0	0	0	0	0
0	0	0	0	0	0	0	0	0	0	0	0	0	0
0	0	0	0	0	0	0	0	0	0	0	0	0	0
0	0	0	0	0	0	1	0	0	0	0	0	0	0
0	0	0	0	0	0	0	0	0	0	0	0	0	0
0	0	0	1	0	0	1	0	0	0	2	0	0	0
0	0	0	0	0	0	0	0	0	0	0	0	0	0
0	0	0	0	0	0	0	0	0	0	0	0	0	0
0													
OTU3770	0	1	0	1	0	1	1	0	0	0	0	0	0
1	1	0	0	0	0	1	0	0	3	0	2	0	0
0	0	1	0	0	0	0	0	0	0	0	1	0	0
0	0	0	0	0	0	1	0	0	0	0	0	0	0
0	0	0	0	0	0	0	0	0	0	0	0	0	0
0	0	0	0	0	0	0	0	0	0	0	0	0	0
0	0	0	0	0	0	0	0	0	0	0	0	0	0
0	0	0	0	0	0	0	0	0	0	0	0	0	0
0													
OTU455	0	1	2	10	3	3	1	0	1	0	0	0	0
0	1	0	2	0	0	0	0	0	0	0	0	0	0
0	0	0	0	6	0	3	0	1	0	1	0	0	0

0	0	0	0	2	0	0	0	1	1	1	0	0	0
0	0	1	4	2	0	0	3	0	1	0	0	2	0
0	0	3	0	0	0	0	3	0	0	0	0	0	0
0	0	0	0	0	0	0	0	0	0	0	0	0	0
0	0	0	0	0	0	0	0	0	0	0	0	0	0
0													
OTU10808	0	1	0	0	0	0	0	0	0	0	0	0	0
0	0	0	0	0	0	0	0	0	0	0	0	0	0
0	0	0	0	1	0	0	0	0	0	0	0	0	0
0	0	0	0	0	0	0	0	0	0	0	0	0	0
0	0	0	0	0	0	0	0	0	0	0	0	0	0
0	0	0	0	0	0	0	0	0	0	0	0	0	0
0	0	0	0	0	0	0	0	0	0	0	0	0	0
0	0	0	0	0	0	0	0	0	0	0	0	0	0
0													
OTU13294	0	1	0	0	0	0	0	0	0	0	0	0	0
0	0	0	0	0	0	0	0	0	0	0	0	0	0
0	0	0	0	0	0	0	0	0	0	0	0	1	0
0	0	0	0	0	0	0	0	0	0	0	0	0	0
0	0	0	0	0	0	0	0	0	0	0	0	0	0
0	0	0	0	0	0	0	0	0	0	0	0	0	0
0	0	0	0	0	0	0	0	0	0	0	0	0	0
0	0	0	0	0	0	0	0	0	0	0	0	0	0
0													
OTU8474	0	1	0	0	0	0	0	0	0	0	0	0	0
0	0	0	0	0	0	0	0	0	0	0	0	0	0
0	0	0	0	0	0	0	0	0	0	0	0	0	0
0	0	0	0	0	0	0	0	0	0	0	0	0	0
0	0	0	0	0	0	0	0	0	0	0	0	0	0
0	0	0	0	0	0	0	0	0	0	0	0	0	0
0	0	0	0	0	0	0	0	0	0	0	0	0	0
0	0	0	0	0	0	0	0	0	0	0	0	0	0
0													

OTU10812	0	1	0	0	0	0	0	0	0	0	0	0	0
0	0	0	0	0	0	0	0	0	0	0	0	0	0
0	0	0	0	0	0	0	0	0	0	0	0	0	0
0	0	0	0	0	0	0	0	0	0	0	0	0	0
0	0	0	0	0	0	0	0	0	0	0	0	0	0
0	0	0	0	0	0	0	0	0	0	0	0	0	0
0	0	0	0	0	0	0	0	0	0	0	0	0	0
0	0	0	0	0	0	0	0	0	0	0	0	0	0
0													
OTU501	0	6	4	0	0	0	0	0	1	0	0	1	0
0	0	0	0	0	0	0	0	0	0	0	1	0	0
0	0	0	0	0	0	0	0	0	0	0	0	0	0
0	0	0	2	0	0	0	0	0	0	0	0	0	0
0	0	0	0	1	0	0	0	0	0	0	0	0	0
0	0	0	0	0	0	0	0	0	1	0	0	0	2
0	1	0	0	0	0	0	0	0	0	0	1	0	0
0	0	0	24	7	2	0	2	2	3	0	0	0	3
0													
OTU12170	0	1	0	0	0	0	0	0	0	0	0	0	0
0	0	0	0	0	0	0	0	0	0	0	0	0	0
0	0	0	0	0	0	0	0	0	0	0	0	0	0
0	0	0	0	0	0	0	0	0	0	0	0	0	0
0	0	0	0	0	0	0	0	0	0	0	0	0	0
0	0	0	0	0	0	0	0	0	0	0	0	0	0
0	0	0	0	0	0	0	0	0	0	0	0	0	0
0	0	0	0	0	0	0	0	0	0	0	0	0	0
0													
OTU2237	0	1	0	0	0	0	0	1	0	0	0	0	0
0	0	0	0	0	0	0	0	0	0	0	0	0	0
0	0	0	0	0	0	0	0	0	0	0	0	0	0
0	0	0	0	0	0	0	0	0	0	0	0	0	0
0	0	0	0	0	0	0	0	0	0	0	0	0	0
0	1	0	0	0	0	0	0	0	0	0	0	0	0

0	0	0	0	0	0	0	0	0	0	0	0	0	0
0	0	0	0	0	0	0	0	0	0	0	0	0	0
0													
OTU14128	0	2	4	0	1	2	0	1	1	1	0	0	0
0	0	3	0	0	0	0	1	0	0	0	1	0	0
3	0	0	1	0	0	0	0	0	2	0	0	0	0
0	0	0	0	0	0	0	0	0	0	0	0	0	0
0	0	0	1	0	0	0	0	0	0	0	0	0	0
0	0	3	1	2	4	0	0	4	0	0	1	3	0
0	0	0	0	1	0	1	0	0	1	1	0	0	0
1	0	0	0	0	0	0	0	0	0	2	2	0	0
0													
OTU1650	0	3	0	0	0	0	0	0	1	0	0	0	0
0	0	0	0	0	0	2	0	0	0	0	0	0	0
0	0	0	0	0	0	0	0	0	0	0	0	2	0
2	0	0	0	0	0	0	0	0	0	0	0	0	0
0	1	0	0	0	0	0	0	0	0	0	0	1	0
0	0	0	0	0	0	0	0	0	0	0	0	0	6
0	0	0	2	0	0	0	1	0	0	0	0	0	0
0	0	0	3	0	0	2	0	0	0	0	0	0	0
0													
OTU10813	0	1	0	0	0	0	0	0	0	0	0	0	0
0	0	0	0	0	0	0	0	0	0	0	0	0	0
0	0	0	0	0	0	0	0	0	0	0	0	0	0
0	0	0	0	0	0	0	0	0	0	0	0	0	0
0	0	0	0	0	0	0	0	0	0	0	0	0	0
0	0	0	0	0	0	0	0	0	0	0	0	0	0
0	0	0	0	0	0	0	0	0	0	0	0	0	0
0	0	0	0	0	0	0	0	0	0	0	0	0	0
0													
OTU8574	0	1	0	0	0	0	0	0	0	0	0	0	0
0	0	0	0	0	0	0	0	0	0	0	0	0	0
0	0	0	0	0	0	0	0	0	0	0	0	0	0

0	0	0	0	0	0	0	0	0	0	0	0	0	0
0	0	0	0	0	0	0	0	0	0	0	0	0	0
0	0	0	0	0	0	0	0	0	0	0	0	0	0
0	0	0	0	0	0	0	0	0	0	0	0	0	0
0	0	0	0	0	0	0	0	0	0	0	0	0	0
0													
OTU9400	0	1	0	1	1	1	1	0	2	0	0	0	0
0	0	1	0	1	6	0	0	0	11	0	0	0	1
0	0	3	1	0	0	1	1	0	0	0	1	0	1
0	0	0	0	1	0	0	0	0	0	3	0	0	1
0	0	0	0	0	0	0	0	0	0	0	0	0	1
1	0	0	1	0	0	0	0	0	0	0	0	0	0
0	0	0	0	0	0	0	0	0	0	0	0	0	0
0	0	0	0	0	0	0	0	0	0	0	0	0	0
0													
OTU711	0	1	0	0	0	0	0	0	0	0	0	0	0
0	0	0	0	0	0	0	0	0	0	0	0	0	0
0	0	0	0	0	0	1	0	0	0	0	0	0	0
0	0	0	0	0	1	0	0	0	0	0	0	0	0
0	2	0	0	0	0	0	0	0	0	0	0	4	0
1	0	0	0	0	0	0	0	0	0	0	0	0	0
0	0	0	0	0	0	0	0	0	0	0	0	0	0
0	0	0	0	0	0	0	0	0	0	0	0	0	0
0													
OTU9840	0	1	0	0	0	1	0	3	0	0	0	0	0
0	0	0	0	0	0	0	0	0	0	0	0	0	0
0	0	0	0	0	0	1	0	0	0	0	0	0	0
0	0	0	1	0	0	0	2	0	0	0	0	0	0
0	1	0	1	0	0	0	0	0	0	0	2	0	0
0	0	0	0	0	0	0	0	0	0	0	0	0	0
0	0	0	2	0	0	0	0	0	0	0	0	0	0
0	0	0	0	0	0	0	0	0	0	0	0	0	0
0													

OTU12169 0 1 0 0 0 0 0 0 0 0 0 0 0
0 0 0 0 0 0 0 0 0 0 0 0 0 0
0 0 0 0 0 0 0 0 0 0 0 0 0 0
0 0 0 0 0 0 0 0 0 0 0 0 0 0
0 0 0 0 0 0 0 0 0 0 0 0 0 0
0 0 0 0 0 0 0 0 0 0 0 0 0 0
0 0 0 0 0 0 0 0 0 0 0 0 0 0
0 0 0 0 0 0 0 0 0 0 0 0 0 0
0
OTU2015 0 1 0 0 0 0 0 0 0 0 0 0 0
0 0 0 0 0 0 0 0 0 0 0 0 0 0
0 0 0 0 0 0 0 0 0 0 0 0 0 0
0 0 0 0 0 0 0 0 0 0 0 0 0 0
0 0 0 0 0 0 0 0 1 0 0 0 0 0
0 0 0 0 0 0 0 0 0 0 0 0 0 0
0 0 0 0 0 0 0 0 0 0 0 0 0 0
0 0 0 0 0 0 0 0 0 0 0 0 0 0
0
OTU12165 0 1 0 0 0 0 0 0 0 0 0 0 0
0 0 0 0 0 0 0 0 0 0 0 0 0 0
0 0 0 0 0 0 0 0 0 0 0 0 0 0
0 0 0 0 0 0 0 0 0 0 0 0 0 0
0 0 0 0 0 0 0 0 0 0 0 0 0 0
0 0 0 0 0 0 0 0 0 0 0 0 0 0
0 0 0 0 0 0 0 0 0 0 0 0 0 0
0 0 0 0 0 0 0 0 0 0 0 0 0 0
0
OTU10810 0 1 0 0 0 0 0 0 0 0 0 0 0
0 0 0 0 0 0 0 0 0 0 0 0 0 0
0 0 0 0 0 0 0 0 0 0 0 0 0 0
0 0 0 0 0 0 0 0 0 0 0 0 0 0
0 0 0 0 0 0 0 0 0 0 0 0 0 0
0 0 0 0 0 0 0 0 0 0 0 0 0 0

0	0	0	0	0	0	0	0	0	0	0	0	0	0
0	0	0	0	0	0	0	0	0	0	0	0	0	0
0													
OTU12168	0	1	0	0	0	1	0	0	0	0	0	0	0
0	0	0	0	0	0	0	0	0	0	0	2	0	0
0	0	0	0	0	0	0	0	0	0	0	0	0	0
0	0	0	0	0	0	0	0	0	0	0	0	0	0
0	0	0	0	0	0	0	0	0	0	0	0	0	0
0	0	0	0	0	0	0	0	0	0	0	0	0	0
0	0	0	0	0	0	0	0	0	0	0	0	0	0
0	0	0	0	0	0	0	0	0	0	0	0	0	0
0													
OTU14252	0	1	0	0	0	0	0	0	0	0	0	0	0
0	0	0	0	0	0	0	0	0	0	0	0	0	0
0	0	0	0	0	0	0	0	0	0	0	0	0	0
0	0	0	0	0	0	0	0	0	0	0	0	0	0
0	0	0	0	0	0	0	0	0	0	0	0	0	0
0	0	0	0	0	0	0	0	0	0	0	0	0	0
0	0	0	0	0	0	0	0	0	0	0	0	0	0
0	0	0	0	0	0	0	0	0	0	0	0	0	0
0													
OTU12166	0	1	0	0	0	0	0	0	0	0	0	0	0
0	0	0	0	0	0	0	0	0	0	0	0	0	0
0	0	0	0	0	0	0	0	0	0	0	0	0	0
0	0	0	0	0	0	0	0	0	0	0	0	0	0
0	0	0	0	0	0	0	0	0	0	0	0	0	0
0	0	0	0	0	0	0	0	0	0	0	0	0	0
0	0	0	0	0	0	0	0	0	0	0	0	0	0
0	0	0	0	0	0	0	0	0	0	0	0	0	0
0													
OTU12167	0	1	0	0	0	0	0	0	0	0	0	0	0
0	0	0	0	0	0	0	0	0	0	0	0	0	0
0	0	0	0	0	0	0	0	0	0	0	0	0	0

0	0	0	0	0	0	0	0	0	0	0	0	0	0
0	0	0	0	0	0	0	0	0	0	0	0	0	0
0	0	0	0	0	0	0	0	0	0	0	0	0	0
0	0	0	0	0	0	0	0	0	0	0	0	0	0
0	0	0	0	0	0	0	0	0	0	0	0	0	0
0													
OTU10811	0	1	0	0	0	0	0	0	0	0	0	0	0
0	0	0	0	0	0	0	0	0	0	0	0	0	0
0	0	0	0	0	0	0	0	0	0	0	0	0	0
0	0	0	0	0	0	0	0	0	0	0	0	0	0
0	0	0	0	0	0	0	0	0	0	0	0	0	0
0	0	0	0	0	0	0	0	0	0	0	0	0	0
0	0	0	0	0	0	0	0	0	0	0	0	0	0
0	0	0	0	0	0	0	0	0	0	0	0	0	0
0													
OTU12196	0	1	0	0	0	0	0	0	0	0	0	0	0
0	0	0	0	0	0	0	0	0	0	0	0	0	0
0	0	0	0	0	0	0	0	0	0	0	0	0	0
0	0	0	0	0	0	0	0	0	0	0	0	0	0
0	0	0	0	0	0	0	0	0	0	0	0	0	0
0	0	0	0	0	0	0	0	0	0	0	0	0	0
0	0	0	0	0	0	0	0	0	0	0	0	0	0
0	0	0	0	0	0	0	0	0	0	0	0	0	0
0													
OTU9882	0	1	0	0	0	0	0	0	2	0	0	0	0
0	0	0	0	0	0	0	0	0	0	0	0	0	0
0	0	0	0	0	0	0	0	0	0	0	0	0	0
1	0	0	0	0	0	0	0	0	0	0	0	0	0
0	0	0	1	0	0	0	0	0	0	0	0	0	0
0	0	0	0	0	0	0	0	0	0	0	0	0	0
0	0	0	0	0	1	0	1	0	0	0	0	0	0
0	0	0	0	0	0	0	0	0	0	0	0	0	0
0													

OTU5885	0	1	0	0	0	0	0	0	0	0	0	0	0
0	0	0	0	0	0	0	0	0	0	0	0	0	0
0	0	0	0	0	0	0	0	1	0	0	0	0	0
0	0	0	0	0	0	0	0	0	0	0	0	0	0
0	0	0	0	0	0	1	0	0	0	0	0	0	0
0	0	1	0	0	0	0	0	0	0	0	0	0	0
0	0	0	0	0	0	0	0	0	0	0	0	0	0
1	0	0	0	0	0	0	0	0	0	0	0	0	0
0													
OTU12197	0	1	0	0	0	0	0	0	0	0	0	0	0
0	0	0	0	0	0	0	0	0	0	0	0	0	0
0	0	0	0	0	0	0	0	0	0	0	0	0	0
0	0	0	0	0	0	0	0	0	0	0	0	0	0
0	0	0	0	0	0	0	0	0	0	0	0	0	0
0	0	0	0	0	0	0	0	0	0	0	0	0	0
0	0	0	0	0	0	0	0	0	0	0	0	0	0
0	0	0	0	0	0	0	0	0	0	0	0	0	0
0													
OTU499	0	1	1	1	0	1	2	2	2	1	1	0	0
0	3	0	1	0	0	4	2	1	0	1	0	3	0
1	1	0	0	0	0	1	1	0	0	0	0	2	0
0	0	0	0	0	3	0	0	0	0	0	0	0	0
0	1	0	2	0	0	0	0	0	0	0	1	2	0
0	4	8	2	1	1	0	2	1	0	0	1	1	0
0	1	0	3	1	0	1	3	0	1	0	1	1	0
0	1	2	6	2	0	1	3	2	1	0	1	0	10
3													
OTU10796	0	1	0	0	0	0	0	0	0	0	0	0	0
0	0	0	0	0	0	0	0	0	0	0	0	0	0
0	0	0	0	0	0	0	0	0	0	0	0	0	0
0	0	0	0	0	0	0	0	0	0	0	0	0	0
0	0	0	0	0	0	0	0	0	0	0	0	0	0
0	0	0	0	0	0	0	0	0	0	0	0	0	0

0	0	0	0	0	0	0	0	0	0	0	0	0	0
0	0	0	0	0	0	0	0	0	0	0	0	0	0
0													
OTU7799	0	2	0	0	0	0	1	0	0	0	0	0	0
0	0	0	0	0	0	1	0	0	0	0	0	0	0
0	0	0	0	0	0	0	0	0	0	0	0	0	0
0	0	0	0	0	0	0	0	0	0	0	0	0	0
0	0	0	0	0	0	0	0	0	0	0	0	0	0
0	0	0	0	0	0	0	0	0	0	0	0	0	0
0	0	0	0	0	0	0	0	0	0	0	0	0	0
0	0	0	0	0	0	0	0	0	0	0	0	0	0
0													
OTU12195	0	1	0	0	0	0	0	0	0	0	0	0	0
0	0	0	0	0	0	0	0	0	0	0	0	0	0
0	0	0	0	0	0	0	0	0	0	0	0	0	0
0	0	0	0	0	0	0	0	0	0	0	0	0	0
0	0	0	0	0	0	0	0	0	0	0	0	0	0
0	0	0	0	0	0	0	0	0	0	0	0	0	0
0	0	0	0	0	0	0	0	0	0	0	0	0	0
0	0	0	0	0	0	0	0	0	0	0	0	0	0
0													
OTU10797	0	1	0	0	0	0	0	0	0	0	0	0	0
0	0	0	0	0	0	0	0	0	0	0	0	0	0
0	0	0	0	0	0	0	0	0	0	0	0	0	0
0	0	0	0	0	0	0	0	0	0	0	0	0	0
0	0	0	0	0	0	0	0	0	0	0	0	0	0
0	0	0	0	0	0	0	0	0	0	0	0	0	0
0	0	0	0	0	0	0	0	0	0	0	0	0	0
0	0	0	0	0	0	0	0	0	0	0	0	0	0
0													
OTU1486	0	1	0	0	0	0	0	0	0	0	0	0	0
0	0	0	0	0	0	0	0	1	0	0	0	0	0
0	0	0	0	0	0	0	0	0	0	0	0	0	0

0 0 0 0 0 0 0 0 0 0 0 0 0 0
0 0 0 0 0 0 0 0 0 0 0 0 0 0
0 0 0 1 0 0 0 0 0 0 0 0 0 0
0 0 0 0 0 0 0 0 1 0 0 0 0 0
0 0 0 0 0 0 0 0 0 0 0 0 0 0
0
OTU71 0 5 31 0 9 12 3 8 8 24 0 0 0 0
0 0 0 0 0 5 0 0 0 0 1 1 0 0
0 0 0 0 0 0 0 1 3 0 0 0 0 28
0 1 0 0 1 0 11 0 1 0 1 1 0 0
0 0 2 0 1 2 0 3 0 0 0 46 0 0
638 7 0 16 1 0 1 0 0 14 7 0 80 1
2 0 3 2 0 0 20 0 0 0 477 177 0 0
215 321 158 61 147 15 159 205 297 0 6 0 66 250
OTU14256 0 1 0 0 0 0 0 0 0 0 0 0 0
0 0 0 0 0 0 0 0 0 0 0 0 0 0
0 0 0 0 0 0 0 0 0 0 0 0 0 0
0 0 0 0 0 0 0 0 0 0 0 0 0 0
0 0 0 0 0 0 0 0 0 0 0 0 0 0
0 0 0 0 0 0 0 0 0 0 0 0 0 0
0 0 0 0 0 0 0 0 0 0 0 0 0 0
0 0 0 0 0 0 0 0 0 0 0 0 0 0
0
OTU4021 0 1 0 0 0 0 0 0 0 0 0 0 0
0 0 0 0 0 0 0 0 0 0 0 0 0 0
0 0 0 1 0 0 0 0 0 0 0 0 0 0
0 0 0 0 0 0 0 0 0 0 0 0 0 0
0 0 0 0 0 0 0 0 0 0 0 0 0 0
0 0 0 0 0 0 0 0 0 0 0 1 0 0
0 0 0 0 0 0 0 0 0 0 0 0 0 0
0 0 0 0 0 0 0 0 0 0 0 0 0 0
0

OTU492	0	2	0	0	0	0	1	0	0	0	0	0	0
0	0	0	0	0	0	0	0	0	0	0	0	0	0
1	0	0	0	0	0	0	0	0	0	0	0	0	0
0	0	0	0	0	0	0	0	0	0	0	0	0	0
0	0	0	0	0	0	0	0	0	0	1	0	5	0
0	0	0	0	0	0	0	0	0	0	0	133	0	2
0	0	0	0	0	0	0	0	0	0	0	0	0	0
0	2	0	0	0	0	0	0	0	0	0	0	0	0
0													
OTU14596	0	2	0	0	0	0	0	0	0	0	0	0	0
0	0	0	0	0	0	0	0	0	0	0	0	0	0
0	0	0	0	0	0	0	0	0	0	0	0	0	0
0	0	0	0	0	0	0	0	0	0	0	0	0	0
0	0	0	0	0	0	0	0	0	0	0	0	0	0
0	0	0	0	0	0	0	0	0	0	0	0	0	0
0	0	0	0	0	0	0	0	0	0	0	0	0	0
0	0	0	0	0	0	0	0	0	0	0	0	0	0
0													
OTU3606	0	1	4	0	0	0	0	1	0	0	0	0	0
0	1	0	0	0	0	2	0	0	0	0	0	0	0
2	1	0	0	0	0	1	0	0	0	0	0	5	0
2	0	0	0	0	0	0	0	0	0	0	1	0	0
0	0	0	1	0	0	0	0	0	0	0	0	0	0
0	0	5	0	0	0	0	0	1	1	0	1	0	1
0	0	0	4	0	0	0	0	0	0	0	0	0	0
0	0	0	1	0	1	0	0	0	1	0	0	0	0
0													
OTU12193	0	1	0	0	0	0	0	0	0	0	0	0	0
0	0	0	0	0	0	0	0	0	0	0	0	0	0
0	0	0	0	0	0	0	0	0	0	0	0	0	0
0	0	0	0	0	0	0	0	0	0	0	0	0	0
0	0	0	0	0	0	0	0	0	0	0	0	0	0
0	0	0	0	0	0	0	0	0	0	0	0	0	0

0	0	0	0	0	0	0	0	0	0	0	0	0	0
0	0	0	0	0	0	0	0	0	0	0	0	0	0
0													
OTU277	0	1	0	0	0	1	0	0	0	12	0	0	0
0	0	0	0	0	0	0	0	0	0	0	0	0	0
0	0	0	0	0	0	0	0	0	0	0	0	0	1
1	0	0	0	0	0	0	2	0	0	0	0	0	0
0	0	0	0	0	0	0	0	0	0	0	0	0	0
0	37	0	0	0	0	0	0	0	0	2	1	0	2
0	0	0	0	0	0	0	0	0	0	0	5	4	0
0	56	0	26	55	53	4	84	24	35	0	2	0	24
9													
OTU8802	0	1	0	0	0	0	0	0	0	0	0	0	0
0	0	0	0	0	0	0	1	0	0	0	0	0	0
0	0	0	0	0	1	0	0	0	0	0	0	0	0
0	0	0	0	0	0	0	0	0	1	0	0	0	0
0	0	0	0	0	0	0	0	0	0	0	0	0	0
0	0	0	0	0	0	0	0	0	0	0	0	0	0
0	0	0	0	0	1	0	0	0	0	0	0	0	0
0	0	0	0	0	0	0	0	0	0	0	0	0	0
0													
OTU12457	0	1	0	0	0	0	0	0	3	1	0	0	0
0	0	0	0	0	0	0	0	0	0	0	0	0	0
2	0	0	0	0	0	0	0	0	0	0	0	0	0
0	0	0	0	0	0	0	0	0	0	0	1	0	0
0	0	0	0	0	0	0	0	0	0	0	0	0	0
0	0	0	0	0	0	0	2	0	1	0	1	0	0
0	0	0	0	0	0	0	0	0	0	0	0	0	0
0	0	0	0	0	0	0	0	0	0	0	0	0	0
0													
OTU10793	0	1	0	0	0	0	0	0	0	0	0	0	0
0	0	0	0	0	0	0	0	0	0	0	0	0	0
0	0	0	0	0	0	0	0	0	0	0	0	0	0

0	0	0	0	0	0	0	0	0	0	0	0	0	0
0	0	0	0	0	0	0	0	0	0	0	0	0	0
0	0	0	0	0	0	0	0	0	0	0	0	0	0
0	0	0	0	0	0	0	0	0	0	0	0	0	0
0	0	0	0	0	0	0	0	0	0	0	0	0	0
0													
OTU671	0	1	0	0	1	0	1	0	0	0	2	0	0
0	0	0	0	1	0	0	0	0	0	0	0	0	0
0	0	0	0	0	1	0	0	0	2	0	0	0	0
0	0	0	0	0	0	0	0	2	0	0	0	0	0
0	0	0	0	0	0	0	0	0	0	0	0	1	1
1	0	0	0	0	0	0	0	0	0	0	0	0	3
0	0	0	0	0	0	0	0	0	0	0	0	0	0
0	0	0	0	0	1	0	0	0	0	0	0	0	0
0													
OTU8662	0	1	0	1	0	0	0	0	0	0	0	0	0
0	0	1	0	0	0	0	0	0	0	0	0	0	0
1	0	0	0	0	0	1	0	1	0	0	0	0	0
0	0	0	0	0	1	0	2	0	0	1	0	0	2
0	0	0	0	0	0	0	0	0	0	0	0	0	0
0	0	0	0	0	0	0	0	0	0	0	0	0	0
0	0	0	1	1	0	0	0	1	0	0	3	1	0
0	0	0	0	0	0	0	0	0	1	0	0	0	4
0													
OTU2588	0	2	0	0	3	1	0	2	0	0	0	0	1
0	2	0	0	2	0	0	0	0	0	0	0	0	0
0	2	1	0	0	0	0	2	0	0	1	0	5	0
0	0	0	2	0	0	0	0	0	1	2	0	0	0
0	0	2	0	0	1	0	1	1	0	1	0	1	1
0	1	0	0	0	2	0	2	0	1	1	1	0	0
1	0	0	0	0	1	0	1	0	1	0	0	0	1
0	0	1	0	2	1	0	0	2	0	0	0	0	0
1													

OTU14257	0	1	6	0	0	0	0	0	0	0	0	0	0
0	0	0	0	0	0	0	0	0	0	0	0	0	0
0	0	0	0	0	0	0	0	0	0	0	0	0	1
0	0	0	0	0	0	0	0	0	0	0	0	0	0
0	0	0	0	0	0	0	0	0	0	0	0	0	0
0	0	0	0	0	0	0	0	0	0	0	0	0	0
0	0	0	0	0	0	0	0	0	0	0	0	0	0
0	0	0	0	0	2	0	0	0	2	0	0	0	0
0													
OTU8578	0	1	0	0	0	0	0	0	0	0	0	0	0
0	0	0	0	0	0	0	0	0	0	0	0	0	0
0	0	0	0	0	0	0	0	0	0	0	0	0	0
0	0	0	0	0	0	0	0	0	0	0	0	0	0
0	0	0	0	0	0	0	0	0	0	0	0	0	0
0	0	0	0	0	0	0	0	0	0	0	0	0	0
0	0	0	0	0	0	0	0	0	0	0	0	0	0
0	0	0	0	0	0	0	0	0	0	0	0	0	0
0													
OTU12194	0	1	0	0	0	0	0	0	0	0	0	0	0
0	1	0	0	0	0	0	0	0	0	0	0	0	1
0	0	0	1	0	0	0	0	0	0	0	0	0	0
1	0	0	0	0	0	0	0	0	0	0	0	0	0
0	0	0	0	0	0	0	0	0	0	0	0	0	0
0	0	0	0	0	0	0	0	0	0	1	0	0	0
0	0	0	0	0	0	0	0	0	0	0	0	0	0
0	0	0	0	0	0	0	0	0	0	0	0	0	0
0													
OTU248	0	2	44	0	0	0	0	0	11	3	0	0	0
0	0	0	0	0	0	0	0	0	0	0	0	0	0
0	0	0	0	0	0	0	0	0	0	0	0	0	0
0	0	2	0	0	0	0	0	0	0	0	0	0	0
0	0	0	0	0	0	0	0	0	0	0	0	0	0
0	9	0	0	0	0	0	4	0	0	1	0	0	0

0 1 0 1 0 1 0 0 0 0 0 0 3 0
0 18 5 12 38 6 25 9 7 3 0 2 0 55
11
OTU3125 0 1 0 0 0 0 0 0 0 0 0 0 0
0 0 0 0 0 0 0 0 0 0 0 0 0 0
0 0 0 0 1 0 0 0 0 0 0 0 0 0
0 0 1 0 0 0 0 0 0 0 0 0 1 0
0 0 0 0 0 0 0 0 0 0 0 0 0 0
0 0 0 0 0 0 0 0 0 0 1 0 0 0
1 0 0 0 0 0 0 0 0 0 0 0 0 0
0 0 0 0 0 0 0 0 0 0 0 0 0 0
0
OTU10795 0 1 0 0 1 0 0 0 0 0 0 0 0
0 0 0 0 0 0 0 0 0 0 0 0 0 0
0 0 0 0 0 0 0 0 0 0 0 0 0 0
0 0 0 1 0 0 0 0 0 0 0 0 0 0
0 0 0 0 0 0 0 0 0 0 0 0 0 0
0 0 0 0 0 1 0 0 0 0 0 0 0 0
0 0 0 0 0 0 1 0 0 0 0 0 0 0
0 0 0 0 0 0 0 0 0 0 0 0 0 0
0
OTU16783 0 1 0 0 0 0 0 0 0 0 0 0 0
0 0 0 0 0 0 0 0 0 0 0 0 0 0
0 0 0 0 0 0 0 0 0 0 0 0 0 0
0 0 0 0 1 0 0 0 0 0 0 0 0 0
0 0 0 0 0 0 0 0 0 0 0 0 0 0
1 0 0 0 0 0 0 0 0 0 0 0 0 0
0 0 0 0 0 0 0 0 0 0 0 0 0 0
0 0 0 0 0 0 0 0 0 0 0 0 0 0
0
OTU9386 0 1 0 0 0 0 0 0 0 0 0 0 0
0 0 0 0 0 0 0 0 0 0 0 0 0 0
0 0 1 0 0 0 0 0 0 0 0 0 0 0

0 0 0 0 0 0 0 0 0 0 0 0 0 0
0 0 0 0 0 0 0 0 0 0 0 0 0 0
0 0 0 0 0 0 0 0 0 0 0 0 0 0
0 0 0 0 0 0 0 0 0 0 0 0 0 0
0 0 0 0 0 0 0 0 0 0 0 0 0 0
0
OTU6919 0 1 3 0 1 2 4 1 7 6 1 0 0
0 0 0 0 0 0 0 0 1 0 0 0 0 0
0 0 0 0 0 0 1 0 1 0 0 0 0 0
0 0 1 0 0 0 0 1 0 0 0 0 0 0
0 2 0 0 0 0 0 4 11 0 1 0 1 0
0 4 1 0 0 0 0 0 0 4 0 0 0 2
0 0 0 37 0 0 0 5 0 0 0 8 30 0
0 10 11 3 4 3 19 3 7 11 0 0 0 3
11
OTU16740 0 1 0 0 0 0 0 0 0 0 0 0 0
0 0 0 0 0 0 0 0 0 0 0 0 0 0
0 0 0 0 0 0 0 0 0 0 0 0 0 0
0 0 1 0 0 0 0 0 0 0 0 0 0 0
0 0 0 0 0 0 0 0 0 0 0 0 0 0
0 0 0 0 0 0 0 0 0 0 0 0 0 0
0 0 0 0 0 0 0 0 0 0 0 0 0 0
0 0 0 0 0 0 0 0 0 0 0 0 0 0
0
OTU10794 0 2 0 0 0 0 0 0 0 0 0 0 0
0 0 0 0 0 0 0 0 0 0 0 0 0 0
0 0 0 0 0 0 0 0 0 0 0 0 0 0
0 0 0 0 0 0 0 0 0 0 0 0 0 0
0 0 0 0 0 0 0 0 0 0 0 0 0 0
0 0 0 0 0 0 0 0 0 0 0 0 0 0
0 0 0 0 0 0 0 0 0 0 0 0 0 0
0 0 0 0 0 0 0 0 0 0 0 0 0 0
0

OTU174 0 1 0 0 0 0 0 0 0 0 22 0 0
0 0 1 0 0 1 0 2 0 6 0 4 0 394
0 0 10 8 0 1 0 0 0 50 0 0 0 0
0 0 0 0 0 0 0 0 0 0 0 0 0 0
0 0 0 0 0 0 0 2 0 0 0 0 0 0
0 0 0 0 0 0 0 0 0 0 0 0 0 0
0 0 0 0 0 0 0 0 0 0 0 0 0 0
0 0 0 0 0 0 0 0 0 0 0 0 0 0
0
OTU12203 0 1 0 0 0 0 0 0 0 0 0 0 0
0 0 0 0 0 0 0 0 0 0 0 0 0 0
0 0 0 0 0 0 0 0 0 0 0 0 0 0
0 0 0 0 0 0 0 0 0 0 0 0 0 0
0 0 0 0 0 0 0 0 0 0 0 0 0 0
0 0 0 0 0 0 0 0 0 0 0 0 0 0
0 0 0 0 0 0 0 0 0 0 0 0 0 0
0 0 0 0 0 0 0 0 0 0 0 0 0 0
0
OTU12204 0 1 0 0 0 0 0 0 0 0 0 0 0
0 0 0 0 0 0 0 0 0 0 0 0 0 0
0 0 0 0 0 0 0 0 0 0 0 0 0 0
0 0 0 0 0 0 0 0 0 0 0 0 0 0
0 0 0 0 0 0 0 0 0 0 0 0 0 0
0 0 0 0 0 0 0 0 0 0 0 0 0 0
0 0 0 0 0 0 0 0 0 0 0 0 0 0
0 0 0 0 0 0 0 0 0 0 0 0 0 0
0
OTU1734 0 1 0 0 1 0 0 0 0 0 0 0 0
0 1 0 0 0 1 0 0 0 0 0 0 0 0
0 0 0 0 0 0 0 0 0 0 0 1 0 4
0 0 0 1 0 0 0 0 0 0 0 0 0 0
0 0 0 0 0 0 0 0 0 0 0 0 0 0
0 0 0 0 0 2 3 0 1 0 0 0 0 0

0	0	1	0	1	0	0	0	0	0	0	0	0	1
0	0	0	0	0	0	0	0	0	0	0	0	0	0
0													
OTU10802	0	1	0	0	0	0	0	0	0	0	0	0	0
0	0	0	0	0	0	0	0	0	0	0	0	0	0
0	0	0	0	0	0	0	0	0	0	0	0	0	0
0	0	0	0	0	0	0	0	0	0	0	0	0	0
0	0	0	0	0	0	0	0	0	0	0	0	0	0
0	0	0	0	0	0	0	0	0	0	0	0	0	0
0	0	0	0	0	0	0	0	0	0	0	0	0	0
0	0	0	0	0	0	0	0	0	0	0	0	0	0
0													
OTU8575	0	1	0	0	0	0	0	0	0	0	0	0	0
0	0	0	0	0	0	0	0	0	0	0	0	0	0
0	0	0	0	0	0	0	0	0	0	0	0	0	0
0	0	0	0	0	0	0	0	0	0	0	0	0	0
0	0	0	0	0	0	0	0	0	0	0	0	0	0
0	0	0	0	0	0	0	0	0	0	0	0	0	0
0	0	0	0	0	0	0	0	0	0	0	0	0	0
0	0	0	0	0	0	0	0	0	0	0	0	0	0
0													
OTU1192	0	1	0	0	0	0	0	0	1	0	0	0	0
0	0	0	0	0	0	0	0	0	0	0	0	0	0
0	0	0	0	0	0	0	0	0	0	0	0	0	0
0	0	0	0	0	0	0	0	0	0	0	1	0	0
0	0	0	0	0	0	0	0	0	0	0	0	0	0
0	0	0	0	8	0	0	0	0	3	0	0	0	0
0	0	0	0	0	0	0	0	0	0	0	0	0	0
0	0	0	0	0	0	0	0	0	0	0	0	0	0
0													
OTU14254	0	1	0	0	0	0	0	0	0	0	0	0	0
0	0	0	0	0	0	0	0	0	0	0	0	0	0
0	0	0	0	0	0	0	0	0	0	0	0	0	0

0 0 0 0 0 0 0 0 0 0 0 0 0 0
0 0 0 0 0 0 0 0 0 0 0 0 0 0
0 0 0 0 0 0 0 0 0 0 0 0 0 0
0 0 0 0 0 0 0 0 0 0 0 0 0 0
0 0 0 0 0 0 0 0 0 0 0 0 0 0
0
OTU12201 0 1 0 0 0 0 0 0 0 0 0 0 0
0 0 0 0 0 0 0 0 0 0 0 0 0 0
0 0 0 0 0 0 0 0 0 0 0 0 0 0
0 0 0 0 0 0 0 0 0 0 0 0 0 0
0 0 0 0 0 0 0 0 0 0 0 0 0 0
0 0 0 0 0 0 0 0 0 0 0 0 0 0
0 0 0 0 0 0 0 0 0 0 0 0 0 0
0 0 0 0 0 0 0 0 0 0 0 0 0 0
0
OTU10799 0 1 0 0 0 0 0 0 0 0 0 0 0
0 0 0 0 0 0 0 0 0 0 0 0 0 0
0 0 0 0 0 0 0 0 0 0 0 0 0 0
0 0 0 0 0 0 0 0 0 0 0 0 0 0
0 0 0 0 0 0 0 0 0 0 0 0 0 0
0 0 0 0 0 0 0 0 0 0 0 0 0 0
0 0 0 0 0 0 0 0 0 0 0 0 0 0
0 0 0 0 0 0 0 0 0 0 0 0 0 0
0
OTU14255 0 1 0 0 0 0 0 0 0 0 0 0 0
0 0 0 0 0 0 0 0 0 0 0 0 0 0
0 0 0 0 0 0 0 0 0 0 0 0 0 0
0 0 0 0 0 0 0 0 0 0 0 0 0 0
0 0 0 0 0 0 0 0 0 0 0 0 0 0
0 0 0 0 0 0 0 1 0 0 0 0 0 0
0 0 0 0 0 0 0 0 0 0 0 0 0 0
0 0 0 0 0 0 0 0 0 0 0 0 0 0
0

OTU3185	0	1	0	0	0	0	0	0	2	2	3	0	1
0	2	0	4	1	0	0	1	1	1	0	1	2	0
1	0	0	0	0	0	0	0	0	0	2	0	1	0
0	1	0	0	1	0	0	1	0	5	0	1	0	0
1	1	1	2	0	2	0	0	1	0	0	1	1	1
2	0	0	0	1	2	0	3	0	0	0	3	0	0
3	0	0	1	0	2	0	0	0	0	2	1	2	0
1	2	0	0	1	0	0	1	1	1	0	2	1	0
1													
OTU12202	0	1	0	0	0	0	0	0	0	0	0	0	0
0	0	0	0	0	0	0	0	0	0	0	0	0	0
0	0	0	0	0	0	0	0	0	0	0	0	0	0
0	0	0	0	0	0	0	0	0	0	0	0	0	0
0	0	0	0	0	0	0	0	0	0	0	0	0	0
0	0	0	0	0	0	0	0	0	0	0	0	0	0
0	0	0	0	0	0	0	0	0	0	0	0	0	0
0	0	0	0	0	0	0	0	0	0	0	0	0	0
0													
OTU10801	0	1	0	0	0	0	0	0	0	0	0	0	0
0	0	0	0	0	0	0	0	0	0	0	0	0	0
0	0	0	0	0	0	0	0	0	0	0	0	0	0
0	0	0	0	0	0	0	0	0	0	0	0	0	0
0	0	0	0	0	0	0	0	0	0	0	0	0	0
0	0	0	0	0	0	0	0	0	0	0	0	0	0
0	0	0	0	0	0	0	0	0	0	0	0	0	0
0	0	0	0	0	0	0	0	0	0	0	0	0	0
0													
OTU8576	0	1	0	0	0	0	0	0	0	0	0	0	0
0	0	0	0	0	0	0	0	0	0	0	0	0	0
0	0	0	0	0	0	0	0	0	0	0	0	0	0
0	0	0	0	0	0	0	0	0	0	0	0	0	0
0	0	0	0	0	0	0	0	0	0	0	0	0	0
0	0	0	0	0	0	0	0	0	0	0	0	0	0

0	0	0	0	0	0	0	0	0	0	0	0	0	0
0	0	0	0	0	0	0	0	0	0	0	0	0	0
0													
OTU8577	0	1	0	0	0	0	0	0	0	0	0	0	0
0	0	0	0	0	0	0	0	0	0	0	0	0	0
0	0	0	0	0	0	0	0	0	0	0	0	0	0
0	0	0	0	0	0	0	0	0	0	0	0	0	0
0	0	0	0	0	0	0	0	0	0	0	0	0	0
0	0	0	0	0	0	0	0	0	0	0	0	0	0
0	0	0	0	0	0	0	0	0	0	0	0	0	0
0	0	0	0	0	0	0	0	0	0	0	0	0	0
0													
OTU10800	0	1	0	0	0	0	0	0	0	0	0	0	0
0	0	0	0	0	0	0	0	0	0	0	0	0	0
0	0	0	0	0	0	0	0	0	0	0	0	0	0
0	0	0	0	0	0	0	0	0	0	0	0	0	0
0	0	0	0	0	0	0	0	0	0	0	0	0	0
0	0	0	0	0	0	0	0	0	0	0	0	0	0
0	0	0	0	0	0	0	0	0	0	0	0	0	0
0	0	0	0	0	0	0	0	0	0	0	0	0	0
0													
OTU14566	0	1	4	0	0	0	0	0	0	0	0	0	0
0	0	0	0	0	0	0	0	0	0	0	0	0	0
0	0	0	0	0	0	0	0	0	0	0	2	0	1
0	0	0	0	2	0	0	0	0	0	0	0	0	0
0	0	0	0	0	0	0	0	0	0	0	0	0	0
0	0	0	0	0	0	0	0	0	0	0	0	0	0
0	2	0	0	0	0	0	1	0	0	0	0	0	0
0	0	0	0	0	4	0	0	0	1	0	0	0	0
0													
OTU6148	0	1	0	0	0	0	0	0	0	0	0	0	0
0	0	0	0	0	0	0	0	0	0	0	0	0	0
0	0	0	0	0	0	0	0	0	0	0	0	0	0

0	0	0	0	0	0	0	0	0	0	0	0	0	0
0	0	0	0	0	0	0	0	0	0	0	0	1	0
0	0	0	0	0	0	0	0	0	0	0	0	0	0
0	0	0	0	0	0	0	0	0	0	0	0	0	0
0	0	0	0	0	0	0	0	0	0	0	0	0	0
0													
OTU2335	0	1	0	0	0	0	0	0	0	0	0	0	0
0	0	0	0	0	0	0	0	0	0	0	0	0	0
0	0	0	0	0	0	0	0	0	0	0	0	0	0
0	0	0	0	0	0	0	0	0	0	0	0	0	0
0	0	0	0	0	0	0	0	0	0	0	0	1	0
0	0	0	0	0	0	0	0	0	0	0	0	0	0
0	0	0	0	0	0	0	0	0	0	0	0	0	0
0	0	0	0	0	0	0	0	0	0	0	0	0	0
0													
OTU11684	0	1	0	0	0	0	1	2	0	0	1	0	0
0	0	0	0	2	4	0	0	0	0	0	1	0	0
0	0	1	0	0	0	1	0	1	0	0	1	0	0
0	0	2	1	0	0	0	0	0	0	0	0	0	0
0	0	0	0	0	0	0	0	0	0	0	0	0	0
0	0	0	0	0	0	0	0	0	1	1	0	0	0
0	0	3	0	0	0	0	0	0	0	0	0	0	0
0	0	0	0	0	0	0	0	0	0	0	1	0	0
0													
OTU3803	0	1	0	0	0	0	0	0	0	0	0	0	1
0	0	0	0	0	0	0	0	0	0	0	1	0	0
0	0	0	1	0	2	0	0	0	0	0	0	0	0
0	0	0	0	0	0	0	0	0	0	0	0	0	1
0	0	0	0	0	0	0	0	0	0	0	0	0	0
0	0	0	1	0	0	0	0	0	0	0	0	0	0
1	0	0	0	0	0	0	0	0	0	0	0	0	0
0	0	0	0	0	0	0	0	0	0	0	0	2	0
0													

OTU14600	0	1	0	0	0	0	0	0	0	0	0	0	0
0	0	0	0	0	0	0	0	0	0	0	0	0	0
0	0	0	0	0	0	0	0	0	0	0	0	0	0
0	0	0	0	0	0	0	0	0	0	0	0	0	0
0	0	0	0	0	0	0	0	0	0	0	0	0	0
0	0	0	0	0	0	0	0	0	0	0	0	0	0
0	0	0	0	0	0	0	0	0	0	0	0	0	0
0	0	0	0	0	0	0	0	0	0	0	0	0	0
0													
OTU1133	0	1	0	0	0	0	0	0	0	0	0	0	0
0	0	0	0	1	0	0	1	0	5	0	1	0	0
0	0	0	0	0	1	0	0	0	0	0	0	0	0
0	0	0	0	0	0	0	0	0	0	0	0	0	0
0	0	0	0	0	0	0	0	0	0	0	0	0	0
0	0	0	0	1	0	0	0	0	0	0	0	0	0
0	0	0	0	0	0	0	1	0	0	0	0	0	0
0	0	0	0	0	0	0	0	0	0	0	0	0	0
0													
OTU8569	0	1	0	0	0	0	0	0	0	0	0	0	0
0	0	0	0	0	0	0	0	0	0	0	0	0	0
0	0	0	0	0	0	0	0	0	0	0	0	0	0
0	0	0	0	0	0	0	0	0	0	0	0	0	0
0	0	0	0	0	0	0	0	0	0	0	0	0	0
0	0	0	0	0	0	0	0	0	0	0	0	0	0
0	0	0	0	0	0	0	0	0	0	0	0	0	0
0	0	0	0	0	0	0	0	0	0	0	0	0	0
0													
OTU5239	0	1	0	0	0	0	0	0	0	1	0	0	0
0	0	0	0	0	0	4	0	0	0	0	0	0	0
0	0	0	0	0	0	0	0	0	0	0	0	1	0
11	0	0	0	0	0	0	0	0	0	0	0	0	0
0	0	0	0	0	0	0	0	0	0	0	0	0	0
0	0	0	0	0	0	0	0	0	4	0	0	0	0

0	0	0	1	0	1	0	0	0	0	0	0	0	0
0	0	0	0	0	0	0	0	0	0	0	0	0	0
0													
OTU10803	0	1	0	0	0	0	0	0	0	0	0	0	0
0	0	0	0	0	0	0	0	0	0	0	0	0	0
0	0	0	0	0	0	0	0	0	0	0	0	0	0
0	0	0	0	0	0	0	0	0	0	0	0	0	0
0	0	0	0	0	0	0	0	0	0	0	0	0	0
0	0	0	0	0	0	0	0	0	0	0	0	0	0
0	0	0	0	0	0	0	0	0	0	0	0	0	0
0	0	0	0	0	0	0	0	0	0	0	0	0	0
0													
OTU12198	0	1	0	0	0	0	0	0	0	0	0	0	0
0	0	0	0	0	0	0	0	0	0	0	0	0	0
0	0	0	0	0	0	0	0	0	0	0	0	0	0
0	0	0	0	0	0	0	0	0	0	0	0	0	0
0	0	0	0	0	0	0	0	0	0	0	0	0	0
0	0	0	0	0	0	0	0	0	0	0	0	0	0
0	0	0	0	0	0	0	0	0	0	0	0	0	0
0	0	0	0	0	0	0	0	0	0	0	0	0	0
0													
OTU7655	0	1	5	2	1	0	0	9	4	3	2	0	0
0	1	5	0	0	2	0	0	0	0	0	0	0	0
0	0	1	2	0	0	0	0	0	1	0	0	0	3
0	0	0	0	0	0	0	0	0	0	0	0	0	0
0	1	0	1	0	0	0	0	1	0	0	0	1	0
0	0	8	0	4	0	0	0	0	11	0	2	0	4
0	1	0	3	0	5	0	0	0	0	0	0	0	0
0	0	2	0	0	1	0	0	0	1	0	0	0	0
0													
OTU12199	0	1	0	0	0	0	0	0	0	0	0	0	0
0	0	0	0	0	0	0	0	0	0	0	0	0	0
0	0	0	0	0	0	0	0	0	0	0	0	0	0

0 0 0 0 0 0 0 0 0 0 0 0 0 0
0 0 0 0 0 0 0 0 0 0 0 0 0 0
0 0 0 0 0 0 0 0 0 0 0 0 0 0
0 0 0 0 0 0 0 0 0 0 0 0 0 0
0 0 0 0 0 0 0 0 0 0 0 0 0 0
0
OTU14599 0 1 0 0 0 0 0 0 0 0 0 0 0
0 0 0 0 0 0 0 0 0 0 0 0 0 0
0 0 0 0 0 0 0 0 0 0 1 0 0 0
1 0 0 0 0 0 0 0 0 0 0 0 0 0
0 0 0 0 0 0 0 0 0 0 0 0 0 0
0 0 0 0 0 0 0 0 0 0 0 0 0 0
0 0 0 0 0 0 0 1 0 0 0 0 0 0
0 0 0 0 0 0 0 0 0 0 0 0 1 0
0
OTU8160 0 1 0 0 0 0 0 0 0 2 0 0 0
0 0 0 0 0 0 1 0 0 0 0 0 0 0
0 0 0 0 0 0 0 0 0 0 0 0 0 0
1 0 0 0 1 0 0 0 0 0 0 0 0 0
0 0 0 1 0 0 0 0 0 0 0 0 1 0
0 0 0 0 0 0 0 0 0 2 0 0 0 0
0 0 0 0 1 0 0 0 0 0 0 0 0 0
0 0 0 0 0 0 0 0 0 0 0 1 0 0
1
OTU10926 0 1 0 1 0 0 0 0 0 0 0 0 0
0 0 0 0 0 0 0 0 1 0 0 0 1 0
0 0 0 0 0 0 0 0 0 0 0 0 0 0
0 0 0 0 1 1 0 0 0 0 0 0 0 1
0 0 0 0 1 0 0 0 0 0 0 2 0 0
0 0 1 0 0 0 0 0 0 0 0 0 0 0
0 0 0 0 0 0 1 0 0 0 0 0 0 1
0 0 0 0 0 0 0 0 0 1 0 0 2 0
0

OTU3840	0	2	0	0	0	0	1	0	1	2	0	0	0
0	0	6	2	13	0	1	0	1	16	5	61	0	6
0	0	3	0	0	24	1	0	0	4	9	13	0	2
1	1	6	5	2	0	0	0	2	0	3	0	15	0
3	0	0	0	0	0	4	0	0	1	0	3	0	1
0	0	0	0	0	0	8	0	2	0	16	0	0	5
11	1	1	0	6	1	0	0	14	0	1	0	0	0
0	0	0	0	0	0	0	0	0	0	2	0	1	0
0													
OTU1623	0	1	0	0	0	0	0	0	0	0	0	0	0
0	0	0	0	0	0	0	0	0	0	0	0	0	0
0	0	0	0	0	0	0	0	0	0	0	0	0	0
0	0	0	0	0	0	0	0	0	0	0	0	0	0
0	0	0	0	0	3	0	1	1	0	0	0	0	0
0	0	0	0	0	0	0	1	0	0	0	2	0	1
0	0	0	0	0	0	0	0	0	0	0	0	0	0
0	0	2	0	0	2	0	0	0	0	0	0	0	0
0													
OTU11336	0	1	11	0	0	0	0	0	0	0	0	0	0
0	0	0	0	0	0	0	0	0	0	0	0	0	0
0	0	0	0	0	0	0	0	0	0	0	2	0	1
0	0	0	0	0	0	0	0	0	0	0	0	0	0
0	0	0	0	0	0	0	0	0	0	0	0	0	0
0	0	0	0	0	0	0	0	0	0	0	0	0	0
0	0	0	1	0	0	0	0	0	0	0	0	0	0
0	0	0	0	0	2	0	0	0	0	0	0	0	0
0													
OTU8567	0	1	0	0	0	0	0	0	0	0	0	0	0
0	0	0	0	0	0	0	0	0	0	0	0	0	0
0	0	0	0	0	0	0	0	0	0	0	0	0	0
0	0	0	0	0	0	0	0	0	0	0	0	0	0
0	0	0	0	0	0	0	0	0	0	0	0	0	0
0	0	0	0	0	0	0	0	0	0	0	0	0	0

0	0	0	0	0	0	0	0	0	0	0	0	0	0
0	0	0	0	0	0	0	0	0	0	0	0	0	0
0													
OTU285	0	1	0	0	0	0	1	1	0	0	0	2	0
0	0	0	0	0	0	0	0	0	0	0	0	1	12
0	0	0	0	0	0	0	0	0	0	0	0	0	0
2	0	0	0	0	0	0	1	0	1	0	0	0	1
0	0	1	0	1	0	0	0	0	0	0	0	0	0
0	10	0	0	0	0	0	0	0	0	0	3	0	0
0	6	0	2	0	0	0	0	0	0	0	57	24	0
0	80	0	101	38	45	25	6	5	0	0	1	0	7
14													
OTU8568	0	1	0	0	0	0	0	0	0	0	0	0	0
0	0	0	0	0	0	0	0	0	0	0	0	0	0
0	0	0	0	0	0	0	0	0	0	0	0	0	0
0	0	0	0	0	0	0	0	0	0	0	0	0	0
0	0	0	0	0	0	0	0	0	0	0	0	0	0
0	0	0	0	0	0	0	0	0	1	0	0	0	0
0	0	0	0	0	0	0	0	0	0	0	0	0	0
0	0	0	0	0	0	0	0	0	0	0	0	0	0
0													
OTU10798	0	2	0	0	0	0	0	0	0	0	0	0	0
0	0	0	0	0	0	0	0	0	0	0	0	0	0
0	0	0	0	0	0	0	0	0	0	0	0	0	0
0	0	0	0	0	0	0	0	0	0	0	0	0	0
0	0	0	0	0	0	0	0	0	0	0	0	0	0
0	0	0	0	0	0	0	0	0	0	0	0	0	0
0	0	0	0	0	0	0	0	0	0	0	0	0	0
0	0	0	0	0	0	0	0	0	0	0	0	0	0
0													
OTU11734	0	1	2	0	0	2	1	4	11	1	63	0	2
0	0	33	0	26	3	0	4	0	3	0	0	0	3
0	0	9	27	0	0	0	0	0	9	0	1	0	4

0	0	1	0	6	0	1	0	0	0	3	0	0	0
0	8	0	2	0	3	0	6	3	0	0	0	7	0
6	0	1	0	0	0	0	2	0	0	0	0	0	0
1	5	0	1	0	0	0	2	0	0	0	0	2	0
0	0	21	0	0	17	1	0	0	3	0	0	0	0
0													
OTU14595	0	1	0	0	0	0	0	0	0	0	0	0	0
0	0	0	0	0	0	0	0	0	0	0	0	0	0
0	0	0	0	0	0	0	0	0	0	0	0	0	0
0	0	0	0	0	0	0	0	0	0	0	0	0	0
0	0	0	0	0	0	0	0	0	0	0	0	0	0
0	0	0	0	0	0	0	0	0	0	0	0	0	0
0	0	0	0	0	0	0	0	0	0	0	0	0	0
0	0	0	0	0	0	0	0	0	0	0	0	0	0
0													
OTU108	0	1	16	4	0	1	5	3	17	2	0	3	0
1	3	3	3	0	0	5	29	13	1	7	1	6	0
135	77	0	0	6	3	1	0	0	1	0	0	19	0
0	1	74	1	32	1	0	0	241	29	13	38	125	1
55	1	1	1	2	151	46	24	160	16	114	1	7	1
4	0	5	0	32	0	0	23	1	7	1	112	1	10
3	10	2	11	3	5	19	31	0	0	1	0	0	0
0	0	0	0	1	3	0	0	0	0	0	0	4	0
0													
OTU8565	0	1	0	0	0	0	0	0	0	0	0	0	0
0	0	0	0	0	0	0	0	0	0	0	0	0	0
0	0	0	0	0	0	0	0	0	0	0	0	0	0
0	0	0	0	0	0	0	0	0	0	0	0	0	0
0	0	0	0	0	0	0	0	0	0	0	0	0	0
0	0	0	0	0	0	0	0	0	0	0	0	0	0
0	0	0	0	0	0	0	0	0	0	0	0	0	0
0	0	0	0	0	0	0	0	0	0	0	0	0	0
0													

OTU12200 0 1 0 0 0 0 0 0 0 0 0 0 0
0 0 0 0 0 0 0 0 0 0 0 0 0 0
0 0 0 0 0 0 0 0 0 0 0 0 0 0
0 0 0 0 0 0 0 0 0 0 0 0 0 0
0 0 0 0 0 0 0 0 0 0 0 0 0 0
0 0 0 0 0 0 0 0 0 0 0 0 0 0
0 0 0 0 0 0 0 0 0 0 0 0 0 0
0 0 0 0 0 0 0 0 0 0 0 0 0 0
0
OTU12535 0 1 0 0 0 0 0 0 0 0 4 1 30
3 0 7 0 0 0 0 20 0 1 0 3 0 4
1 0 0 6 0 7 0 3 0 3 0 1 0 0
0 0 0 1 0 0 2 0 0 0 0 0 0 0
0 0 0 0 0 0 0 0 0 0 0 0 0 0
0 0 0 1 2 0 1 0 2 1 0 0 0 0
0 0 1 0 0 0 1 0 3 1 4 0 0 0
5 0 0 0 0 0 0 0 0 0 1 2 1 0
0
OTU1634 0 1 0 0 0 0 0 0 0 0 0 0 0
0 0 0 0 0 0 0 0 0 0 0 0 0 0
0 0 0 0 0 0 0 0 0 0 0 0 0 0
0 0 0 0 0 0 0 0 0 0 0 0 0 0
0 0 0 0 0 0 0 0 0 0 0 0 0 0
0 0 0 0 0 0 0 0 0 0 0 0 0 0
0 0 0 0 0 0 0 0 0 0 0 0 0 0
0 0 0 0 0 2 0 0 0 0 0 0 1 0
0
OTU2858 0 1 1 0 0 1 1 0 0 1 0 0 0
0 0 0 0 0 0 0 0 0 0 0 0 0 0
0 0 0 0 0 0 0 0 0 0 0 0 0 0
0 0 0 0 0 0 0 1 0 0 0 0 0 0
0 0 0 0 0 0 0 0 0 0 0 0 0 0
0 5 0 0 0 0 0 0 0 1 0 0 0 0

0	0	0	3	0	0	0	0	0	0	0	7	19	0
0	1	3	9	3	3	0	4	0	2	0	0	0	5
18													
OTU5313	0	1	0	0	0	0	0	0	0	0	0	0	0
0	0	0	0	1	0	0	0	1	0	0	0	0	0
0	0	0	0	0	0	0	0	0	1	0	0	0	0
0	1	0	0	0	0	0	0	0	0	0	0	0	0
0	0	0	0	0	0	0	0	0	0	0	0	0	0
0	0	0	0	1	0	0	0	0	0	0	1	0	0
0	0	0	0	0	0	0	0	0	0	0	0	0	0
0	0	0	0	0	0	0	0	0	0	0	0	0	0
0													
OTU5346	0	2	0	1	0	3	0	0	0	0	0	1	0
0	2	0	0	0	0	1	0	0	0	1	0	5	0
0	0	0	0	0	0	0	1	1	0	0	0	0	0
0	0	0	2	0	0	0	0	0	0	0	0	0	0
0	0	0	0	0	0	0	0	0	0	0	0	0	0
0	0	1	0	0	0	0	0	0	0	0	0	0	0
0	0	0	0	0	0	1	0	0	0	0	0	0	1
3	0	0	0	0	0	0	0	1	0	0	0	0	0
0													
OTU12188	0	1	0	0	0	0	0	0	0	0	0	0	0
0	0	0	0	0	0	0	0	0	0	0	0	0	0
0	0	0	0	0	0	0	0	0	0	0	0	0	0
0	0	0	0	0	0	0	0	0	0	0	0	0	0
0	0	0	0	0	0	0	0	0	0	0	0	0	0
0	0	0	0	0	0	0	0	0	0	0	0	0	0
0	0	0	0	0	0	0	0	0	0	0	0	0	0
0	0	0	0	0	0	0	0	0	0	0	0	0	0
0													
OTU521	0	2	0	0	0	0	0	0	1	3	0	0	0
0	0	0	0	0	0	8	0	1	0	0	0	3	0
14	2	0	0	2	1	2	0	0	0	0	0	6	0

0	0	0	0	0	0	0	0	0	4	10	10	0	0
0	0	0	5	2	4	2	7	10	0	1	0	0	1
0	1	0	0	0	0	0	1	0	0	0	5	0	4
0	4	0	0	0	0	0	0	0	0	0	0	0	0
0	0	1	2	4	1	3	0	0	0	0	0	0	1
1													
OTU8003	0	1	1	0	1	0	0	0	1	2	0	0	1
0	0	0	0	1	0	0	0	0	1	0	1	0	0
2	0	1	1	0	2	0	1	0	1	0	0	3	0
1	0	0	0	0	1	0	0	0	2	0	0	0	0
0	0	2	0	0	1	0	0	0	0	1	1	0	0
1	1	0	1	2	1	0	1	1	0	0	0	0	0
1	0	0	0	1	1	0	1	0	1	0	0	0	0
1	0	0	0	0	1	0	2	0	0	1	0	0	1
0													
OTU5536	0	1	0	0	0	0	0	0	0	0	0	0	1
4	0	0	0	0	0	0	0	0	0	0	0	0	0
0	0	3	0	0	0	0	0	0	0	0	0	0	0
0	0	0	0	0	0	1	0	0	0	0	0	0	0
0	0	0	0	0	0	0	0	0	0	0	0	0	0
0	0	0	0	0	0	1	0	0	0	0	0	0	0
0	0	1	0	0	0	0	0	0	0	0	0	0	2
0	0	0	0	0	0	0	0	0	0	0	1	1	0
0													
OTU5262	0	1	0	0	1	1	0	0	0	1	0	1	0
0	0	1	0	0	0	0	0	0	0	0	0	0	0
0	0	0	0	0	0	0	1	0	0	0	0	1	1
0	0	0	1	0	0	0	0	0	2	0	1	0	0
0	0	1	1	0	0	0	0	0	0	0	0	0	0
1	0	0	0	1	0	0	2	1	0	0	2	0	0
0	0	0	1	0	0	0	1	0	0	0	0	0	0
1	0	0	0	0	0	0	0	0	0	0	0	0	1
1													

OTU685	0	1	0	0	0	1	0	0	4	2	0	0	0
0	0	0	0	0	0	0	0	0	0	0	0	0	0
0	0	0	0	0	0	0	0	0	0	0	0	0	0
1	0	0	0	0	0	0	0	0	0	0	0	0	0
0	0	0	0	0	0	0	0	1	0	0	0	0	0
0	0	6	0	0	0	0	0	0	0	0	1	0	0
0	0	0	1	0	0	0	0	0	0	0	3	3	0
0	3	1	0	0	0	0	1	0	3	0	0	0	1
0													
OTU14597	0	1	0	0	0	0	0	0	0	0	0	0	0
0	0	0	0	0	0	0	0	0	0	0	0	0	0
0	0	0	0	0	0	0	0	0	0	0	0	0	0
0	0	0	0	0	0	0	0	0	0	0	0	0	0
0	0	0	0	0	0	0	0	0	0	0	0	0	0
0	0	0	0	0	0	0	0	0	0	0	0	0	0
0	0	0	0	0	0	0	0	0	0	0	0	0	0
0	0	0	0	0	0	0	0	0	0	0	0	0	0
0													
OTU14598	0	1	0	0	0	0	0	0	0	0	0	0	0
0	0	0	0	0	0	0	0	0	0	0	0	0	0
0	0	0	0	0	0	0	0	0	0	0	0	0	0
0	0	0	0	0	0	0	0	0	0	0	0	0	0
0	0	0	0	0	0	0	0	0	0	0	0	0	0
0	0	0	0	0	0	0	0	0	0	0	0	0	0
0	0	0	0	0	0	0	0	0	0	0	0	0	0
0	0	0	0	0	0	0	0	0	0	0	0	0	0
0													
OTU12186	0	1	0	0	0	0	0	0	0	0	0	0	0
0	0	0	0	0	0	0	0	0	0	0	0	0	0
0	0	0	0	0	0	0	0	0	0	0	0	0	0
0	0	0	0	0	0	0	0	0	0	0	0	0	0
0	0	0	0	0	0	0	0	0	0	0	0	0	0
0	0	0	0	0	0	0	0	0	0	0	0	0	0

0	0	0	0	0	0	0	0	0	0	0	0	0	0
0	0	0	0	0	0	0	0	0	0	0	0	0	0
0													
OTU4910	0	1	0	0	1	0	0	2	0	0	0	1	0
0	0	2	0	1	0	0	1	1	0	0	1	0	0
0	0	0	0	0	0	0	3	0	0	0	0	1	1
1	2	0	0	0	0	0	0	0	1	0	0	0	0
0	0	0	1	0	2	0	1	0	0	2	0	0	0
1	0	1	0	0	0	1	2	0	0	0	0	0	0
0	0	0	0	0	1	0	0	0	0	0	0	0	0
0	0	0	0	0	0	0	0	0	0	0	0	0	0
0													
OTU4889	0	1	0	0	0	0	0	0	0	0	0	0	0
0	0	0	1	0	0	0	0	0	0	0	0	0	0
0	2	0	0	0	0	0	0	0	0	0	0	0	0
0	0	0	0	0	0	0	0	0	0	1	0	0	0
0	0	0	0	1	0	0	1	0	0	0	1	0	0
1	0	0	0	0	0	0	0	2	0	0	0	0	0
1	0	0	0	1	0	0	0	0	1	0	0	0	0
0	0	0	0	0	0	0	0	0	0	0	0	0	0
0													
OTU882	0	1	1	0	0	0	0	0	0	0	0	0	0
0	0	0	0	0	0	0	0	0	0	0	0	0	0
0	0	0	0	0	0	0	0	0	0	0	0	0	0
0	0	0	0	0	0	0	0	0	0	0	0	0	0
0	0	0	0	0	0	0	0	0	0	0	0	0	0
0	0	1	0	1	0	0	0	0	0	0	0	0	0
0	0	0	0	0	0	0	0	0	0	0	0	0	0
0	0	0	0	0	0	0	0	0	0	0	0	0	0
0													
OTU12187	0	1	0	0	0	0	0	0	0	0	0	0	0
0	0	0	0	0	0	0	0	0	0	0	0	0	0
0	0	0	0	0	0	0	0	0	0	0	0	0	0

0 0 0 0 0 0 0 0 0 0 0 0 0 0

0 0 0 0 0 0 0 0 0 0 0 0 0 0

0 0 0 0 0 0 0 0 0 0 0 0 0 0

0 0 0 0 0 0 0 0 0 0 0 0 0 0

0 0 0 0 0 0 0 0 0 0 0 0 0 0

0

OTU1111 0 2 0 0 0 2 0 0 1 0 0 0 0

0 0 0 0 0 0 0 0 0 0 0 0 0 0

0 0 0 0 0 0 0 0 0 0 0 0 0 0

0 0 0 0 0 0 0 1 0 0 0 0 0 0

0 0 0 0 0 0 0 0 0 0 0 0 0 0

0 4 1 0 3 0 0 0 0 0 0 10 0 0

0 0 0 0 0 0 0 1 0 0 0 1 1 0

0 0 9 2 8 4 4 2 4 2 0 0 0 6

2

OTU11362 0 1 2 0 0 9 13 0 7 9 0 0 0

0 0 0 0 0 0 0 0 0 0 0 0 0 0

1 0 0 0 0 0 0 0 0 0 1 0 0 0

4 0 0 0 0 0 0 2 0 0 0 0 0 0

0 0 0 2 0 0 0 1 1 0 0 0 1 0

1 8 3 0 7 1 0 1 0 17 1 2 0 2

0 1 0 12 0 5 0 7 0 1 0 10 3 0

0 3 3 7 6 3 1 5 3 8 0 0 0 5

6

OTU1335 0 1 7 1 0 2 10 0 1 1 0 0 0

0 1 0 0 1 0 0 0 0 0 0 0 0 0

1 0 0 0 0 0 0 0 1 0 0 0 0 0

0 0 0 3 0 0 0 0 0 0 0 1 0 0

0 0 0 0 0 0 0 0 0 0 0 0 0 0

0 0 0 0 11 0 0 0 0 2 0 0 1 1

0 0 0 1 0 0 0 1 0 2 0 0 0 0

0 0 0 0 0 0 0 0 0 0 0 0 0 0

0

OTU8991	0	1	0	0	0	0	0	0	0	0	0	0	0
0	0	0	0	0	0	0	0	0	0	0	0	0	0
0	0	0	0	0	0	0	0	0	0	0	0	0	0
1	0	0	0	0	0	0	0	0	0	0	0	0	0
0	0	0	0	0	0	0	0	0	0	0	0	0	0
0	0	0	0	0	0	0	0	0	0	0	0	0	0
0	0	0	0	0	0	0	0	0	0	0	0	0	0
0	0	0	0	0	0	0	0	0	0	0	0	0	0
0													
OTU7004	0	1	0	0	0	0	0	2	0	0	0	0	1
0	0	2	0	3	5	0	0	0	4	0	0	0	0
0	0	1	0	0	1	0	1	1	0	0	7	0	3
0	0	0	2	0	0	3	0	0	0	0	0	0	0
0	2	0	0	0	0	0	0	0	0	0	0	0	0
0	0	0	0	0	1	1	0	0	0	1	0	0	0
0	0	0	1	0	0	0	0	0	2	2	0	0	0
0	0	0	0	0	0	0	0	0	0	0	1	0	0
0													
OTU14605	0	1	0	0	0	0	0	0	0	0	0	0	0
0	0	0	0	0	0	0	0	0	0	0	0	0	0
0	0	0	0	0	0	0	0	0	0	0	0	0	0
0	0	0	0	0	0	0	0	0	0	0	0	0	0
0	0	0	0	0	0	0	0	0	0	0	0	0	0
0	0	0	0	0	0	0	0	0	0	0	0	0	0
0	0	0	0	0	0	0	0	0	0	0	0	0	0
0	0	0	0	0	0	0	0	0	0	0	0	0	0
0													
OTU3420	0	1	0	0	0	0	0	0	0	0	0	0	0
0	0	0	0	1	0	0	0	6	0	3	3	0	0
2	3	0	2	1	0	0	0	0	0	1	0	0	0
0	0	12	0	0	0	0	0	1	1	1	0	0	0
0	0	0	0	0	2	2	2	0	0	0	0	0	1
1	0	0	0	0	0	0	0	0	0	0	0	0	0

1	10	0	0	0	0	2	0	0	0	0	0	0	0
0	0	0	0	0	0	0	0	0	0	1	0	0	0
0													
OTU12189	0	1	0	0	0	0	0	0	0	0	0	0	0
0	0	0	0	0	0	0	0	0	0	0	0	0	0
0	0	0	0	0	0	0	0	0	0	0	0	0	0
0	0	0	0	0	0	0	0	0	0	0	0	0	0
0	0	0	0	0	0	0	0	0	0	0	0	0	0
0	0	0	0	0	0	0	0	0	0	0	0	0	0
0	0	0	0	0	0	0	0	0	0	0	0	0	0
0	0	0	0	0	0	0	0	0	0	0	0	0	0
0													
OTU14550	0	1	2	0	0	0	0	0	0	0	0	0	0
0	0	0	0	0	0	0	0	0	0	0	0	0	0
0	0	0	0	0	0	0	0	0	0	0	0	0	0
0	0	0	0	2	0	0	0	0	0	0	0	0	0
0	0	0	0	0	0	0	0	0	0	0	0	0	0
0	0	0	0	0	0	0	0	0	0	0	0	0	0
0	0	0	0	0	0	0	0	0	0	0	0	0	0
0	0	0	0	0	0	0	0	0	0	0	0	0	0
0													
OTU12190	0	1	0	0	0	0	0	0	0	0	0	0	0
0	0	0	0	0	0	0	0	0	0	0	0	0	0
0	0	0	0	0	0	0	0	0	0	0	0	0	0
0	0	0	0	0	0	0	0	0	0	0	0	0	0
0	0	0	0	0	0	0	0	0	0	0	0	0	0
0	0	0	0	0	0	0	0	0	0	0	0	0	0
0	0	0	0	0	0	0	0	0	0	0	0	0	0
0	0	0	0	0	0	0	0	0	0	0	0	0	0
0													
OTU14603	0	1	0	0	0	0	0	0	0	0	0	0	0
0	0	0	0	0	0	0	0	0	0	0	0	0	0
0	0	0	0	0	0	0	0	0	0	0	0	0	0

0	0	0	0	0	0	0	0	0	0	0	0	0	0
0	0	0	0	0	0	0	0	0	0	0	0	0	0
0	0	0	0	0	0	0	0	0	0	0	0	0	0
0	0	0	0	0	0	0	0	0	0	0	0	0	0
0	0	0	0	0	0	0	0	0	0	0	0	0	0
0													
OTU14604	0	1	0	0	0	1	0	0	0	0	0	0	0
0	0	0	0	0	0	0	0	0	0	0	0	0	0
0	0	0	0	0	0	0	0	0	0	0	0	0	0
0	0	0	0	0	0	0	0	0	0	0	0	0	0
0	0	0	0	0	0	0	0	0	0	0	0	0	0
0	0	0	0	0	0	0	0	0	0	0	0	0	0
0	0	0	0	0	0	0	0	0	0	0	0	0	0
0	0	0	0	0	0	0	0	0	0	0	0	0	0
0													
OTU851	0	1	0	0	0	0	0	0	0	1	0	0	0
0	0	0	0	0	0	1	1	0	0	0	0	0	0
0	0	0	0	0	0	0	0	0	0	1	0	0	0
3	0	0	0	0	3	1	2	0	0	0	0	0	0
0	0	0	1	0	0	0	0	0	0	0	0	0	0
0	0	0	0	0	0	0	0	1	0	1	0	0	1
4	0	0	0	0	0	0	0	0	0	0	0	2	0
0	3	0	0	0	0	0	1	0	1	0	0	0	0
0													
OTU793	0	1	0	0	0	0	0	0	0	0	0	0	0
0	0	0	0	0	0	0	0	0	0	0	0	0	0
0	0	0	0	0	0	0	0	0	0	0	0	0	0
0	0	0	0	0	0	0	0	0	0	0	0	0	0
0	0	0	0	0	0	0	0	0	0	0	0	0	0
0	4	0	0	0	0	0	0	0	0	0	0	0	0
0	0	0	0	0	0	0	0	0	0	0	0	0	0
0	1	0	0	2	0	0	0	0	0	0	0	0	1
0													

OTU10835		0	1	0	0	0	0	0	0	0	0	0	0	0
	0	0	0	0	0	0	0	0	0	0	0	0	0	0
	0	0	0	0	0	0	0	0	0	0	0	0	0	0
	0	0	0	0	0	0	0	0	0	0	0	0	0	0
	0	0	0	0	0	0	0	0	0	0	0	0	0	0
	0	0	0	0	0	0	0	0	0	0	0	0	0	0
	0	0	0	0	0	0	0	0	0	0	0	0	0	0
	0	0	0	0	0	0	0	0	0	0	0	0	0	0
	0													
OTU10565		0	1	0	0	0	1	1	0	0	0	0	0	0
	1	0	0	1	0	0	0	0	0	0	0	0	0	0
	0	0	0	0	0	0	0	1	0	0	0	0	0	0
	0	0	2	1	0	0	0	0	0	1	0	0	0	0
	0	0	0	1	0	0	0	0	0	1	0	0	0	0
	0	0	0	0	0	1	0	2	0	1	0	1	0	0
	1	0	0	1	0	0	0	0	0	0	0	0	1	0
	0	0	0	1	0	0	0	0	0	1	1	0	1	0
	0													
OTU8571		0	1	0	0	0	0	0	0	0	0	0	0	0
	0	0	0	0	0	0	0	0	0	0	0	0	0	0
	0	0	0	0	0	0	0	0	0	0	0	0	0	0
	0	0	0	0	0	0	0	0	0	0	0	0	0	0
	0	0	0	0	0	0	0	0	0	0	0	0	0	0
	0	0	1	0	0	0	0	0	0	0	0	0	0	0
	0	0	0	0	0	0	0	0	0	0	0	0	0	0
	0	0	0	0	0	0	0	0	0	0	0	0	0	0
	0													
OTU223		0	1	0	0	0	0	0	1	0	4	0	0	0
	0	1	0	0	0	0	0	1	0	0	0	0	0	0
	0	0	0	0	1	0	1	0	0	0	0	0	0	0
	0	0	0	0	0	0	0	2	0	0	0	0	0	1
	0	0	0	0	0	0	0	0	0	0	0	0	1	0
	0	15	0	0	0	0	0	0	0	0	1	0	0	0

0	0	0	0	0	0	0	3	0	0	0	0	0	0
0	3	177	5	70	120	105	1	5	6	0	5	0	7
66													
OTU2244	0	1	22	0	0	0	0	0	0	0	0	0	0
0	0	0	0	0	0	1	0	0	0	0	0	0	0
0	0	0	0	0	0	0	0	0	0	15	0	0	0
1	0	0	0	0	0	0	0	0	0	0	0	0	0
0	0	0	0	0	0	0	0	0	0	0	0	0	0
0	0	1	0	0	0	0	2	0	0	0	4	0	0
0	2	0	0	0	0	0	0	0	0	0	1	0	0
0	0	0	0	0	1	6	3	1	0	0	0	0	7
0													
OTU12183	0	1	0	0	0	0	0	0	0	0	0	0	0
0	0	0	0	0	0	0	0	0	0	0	0	0	0
0	0	0	0	0	0	0	0	0	0	0	0	0	0
3	0	0	0	0	0	0	0	0	0	0	0	0	0
0	0	0	0	0	0	0	0	0	0	0	0	0	0
0	1	1	0	0	0	0	0	0	0	0	0	0	0
0	0	0	1	0	0	0	0	0	0	0	4	1	0
0	0	0	1	0	0	0	1	1	0	0	0	0	1
0													
OTU14606	0	1	0	0	0	0	0	0	0	0	0	0	0
0	0	0	0	0	0	0	0	0	1	0	0	0	0
0	0	0	0	0	0	0	0	0	0	0	0	0	0
0	0	0	0	0	0	0	0	0	0	0	0	0	0
0	0	0	0	0	0	0	0	0	0	0	0	0	0
0	0	0	0	0	0	0	0	0	0	0	0	0	0
0	0	0	0	0	0	0	0	0	0	0	0	0	0
0	0	0	0	0	0	0	0	0	0	0	0	0	0
0													
OTU684	0	1	0	1	0	0	0	2	2	0	6	0	12
4	0	0	0	8	8	0	8	1	320	7	50	3	3
0	1	28	0	0	28	36	2	0	0	7	21	1	1

0	0	22	32	2	0	110	0	0	0	1	67	12	1
6	0	2	0	0	0	2	0	0	2	0	4	0	1
0	0	2	6	0	1	55	0	2	2	85	0	2	0
22	0	36	0	4	0	1	0	51	0	2	0	0	0
3	0	0	0	0	0	0	0	0	0	15	0	0	0
1													
OTU14607	0	1	0	0	0	0	0	0	0	0	0	0	0
0	0	0	0	0	0	0	0	0	0	0	0	0	0
0	0	0	0	0	0	0	0	0	0	0	0	0	0
0	0	0	0	0	0	0	0	0	0	0	0	0	0
0	0	0	0	0	0	0	0	0	0	0	0	0	0
0	0	0	0	0	0	0	0	0	0	0	0	0	0
0	0	0	0	0	0	0	0	0	0	0	0	0	0
0	0	0	0	0	0	0	0	0	0	0	0	0	0
0													
OTU10175	0	1	0	0	0	0	0	1	0	1	0	0	0
2	0	0	1	1	0	0	0	0	0	0	0	0	0
1	1	0	0	0	0	0	0	2	0	0	0	0	0
0	0	0	0	1	0	0	0	0	1	0	0	0	0
0	0	0	0	1	0	0	0	1	0	1	0	0	0
0	1	1	0	2	0	0	1	1	0	0	0	0	1
1	0	1	0	0	0	0	1	0	1	0	0	0	0
0	0	1	0	1	0	0	2	0	0	0	0	0	0
2													
OTU10836	0	1	0	0	0	0	0	0	0	0	0	0	0
0	0	0	0	0	0	0	0	0	0	0	0	0	0
0	0	0	0	0	0	0	0	0	0	0	0	0	0
0	0	0	0	0	0	0	0	0	0	0	0	0	0
0	0	0	0	0	0	0	0	0	0	0	0	0	0
0	0	0	0	0	0	0	0	0	0	0	0	0	0
0	0	0	0	0	0	0	0	0	0	0	0	0	0
0	0	0	0	0	0	0	0	0	0	0	0	0	0
0													

OTU14601	0	1	0	0	0	0	0	0	0	0	0	0	0
0	0	0	0	0	0	0	0	0	0	0	0	0	0
0	0	0	0	0	0	0	0	0	0	0	0	0	0
0	0	0	0	0	0	0	0	0	0	0	0	0	0
0	0	0	0	0	0	0	0	0	0	0	0	0	0
0	0	0	0	0	0	0	0	0	0	0	0	0	0
0	0	0	0	0	0	0	0	0	0	0	0	0	0
0	0	0	0	0	0	0	0	0	0	0	0	0	0
0													
OTU8240	0	1	0	0	1	0	0	0	0	0	0	0	0
0	0	0	0	0	0	0	0	0	0	0	0	0	0
0	0	0	0	0	0	0	0	0	0	0	0	3	0
0	0	0	0	0	0	0	0	0	1	0	1	0	0
0	0	0	0	0	0	0	0	1	0	0	0	0	0
0	0	0	0	0	0	0	1	0	0	0	1	0	0
0	0	0	0	0	1	0	1	0	0	0	0	1	0
0	0	1	0	0	0	0	0	0	2	0	0	0	0
0													
OTU418	0	2	0	0	0	0	0	0	0	0	0	0	0
0	0	0	0	0	0	0	0	0	0	0	0	1	0
0	0	0	0	0	0	0	0	0	0	0	0	1	0
0	0	0	0	0	0	0	0	0	0	0	0	0	0
0	3	0	0	0	0	0	0	0	0	0	0	0	0
0	16	0	0	1	0	0	3	0	0	3	1	0	0
0	0	0	0	0	0	0	2	0	0	0	0	5	0
0	1	10	38	11	21	8	1	35	10	0	0	0	30
26													
OTU8595	0	1	0	0	0	0	0	0	0	0	0	0	0
0	0	0	0	0	0	0	0	0	0	0	0	0	0
0	0	0	0	0	0	0	0	0	0	0	0	0	0
0	0	0	0	0	0	0	0	0	0	0	0	0	0
0	0	0	0	0	0	0	0	0	0	0	0	0	0
0	0	0	0	0	0	0	0	0	0	0	0	0	0

0	0	0	0	0	0	0	0	0	0	0	0	0	0
0	0	0	0	0	0	0	0	0	0	0	0	0	0
0													
OTU12184	0	1	0	0	0	0	0	0	0	0	0	0	0
0	0	0	0	0	0	0	0	0	0	0	0	0	0
0	0	0	0	0	0	0	0	0	0	0	0	0	0
0	0	0	0	0	0	0	0	0	0	0	0	0	0
0	0	0	0	0	0	0	0	0	0	0	0	0	0
0	0	0	0	0	0	0	0	0	0	0	0	0	0
0	0	0	0	0	0	0	0	0	0	0	0	0	0
0	0	0	0	0	0	0	0	0	0	0	0	0	0
0													
OTU12185	0	1	0	0	0	0	0	0	0	0	0	0	0
0	0	0	0	0	0	0	0	0	0	0	0	0	0
0	0	0	0	0	0	0	0	0	0	0	0	0	0
0	0	0	0	0	0	0	0	0	0	0	0	0	0
0	0	0	0	0	0	0	0	0	0	0	0	0	0
0	0	0	0	0	0	0	0	0	0	0	0	0	0
0	0	0	0	0	0	0	0	0	0	0	0	0	0
0	0	0	0	0	0	0	0	0	0	0	0	0	0
0													
OTU14553	0	1	7	0	0	0	0	0	0	0	0	0	0
0	0	0	0	0	0	0	0	0	0	0	0	0	0
0	0	0	0	0	0	0	0	0	0	0	0	0	1
0	0	0	0	0	0	0	0	0	0	0	0	0	0
0	0	0	0	0	0	0	0	0	0	0	0	0	0
0	0	0	0	0	0	0	0	0	0	0	0	0	0
0	0	0	0	0	0	0	0	0	0	0	0	0	0
0	0	0	0	0	0	0	0	0	0	0	0	0	0
0													
OTU10840	0	1	0	0	0	0	0	0	0	0	0	0	0
0	0	0	0	0	0	0	0	0	0	0	0	0	0
2	0	0	0	0	0	0	0	0	0	1	0	0	0

0	0	0	0	0	0	0	0	0	0	0	0	0	0
0	0	0	0	0	0	0	0	0	0	0	0	0	0
0	0	0	0	0	0	0	1	0	0	0	0	0	0
0	0	0	0	0	0	0	0	0	0	0	0	0	0
0	0	1	0	0	0	0	0	0	0	0	0	0	0
0													
OTU14602	0	1	0	0	0	0	0	0	0	0	0	0	0
0	0	0	0	0	0	0	0	0	0	0	0	0	0
0	0	0	0	0	0	0	0	0	1	0	0	0	0
0	0	0	0	0	0	0	0	0	0	0	0	0	0
0	0	0	0	0	0	0	0	0	0	0	0	0	0
0	0	0	0	0	0	0	0	1	0	0	0	0	0
0	0	0	0	0	0	0	0	0	0	0	0	0	0
0	0	0	0	0	0	0	0	0	0	0	0	0	0
0													
OTU10841	0	1	0	0	0	0	0	0	0	0	0	0	0
0	0	0	0	0	0	0	0	0	0	0	0	0	0
0	0	0	0	0	0	0	0	0	0	0	0	0	0
0	0	0	0	0	0	0	0	0	0	0	0	0	0
0	0	0	0	0	0	0	0	0	0	0	0	0	0
0	0	0	0	0	0	0	0	0	0	0	0	0	0
0	0	0	0	0	0	0	0	0	0	0	0	0	0
0	0	0	0	0	0	0	0	0	0	0	0	0	0
0													
OTU772	0	1	0	0	0	0	0	0	0	0	0	0	0
0	0	0	0	0	0	0	0	0	0	0	0	0	0
0	0	0	0	0	0	0	0	0	0	14	0	0	0
0	0	0	0	0	0	0	0	0	0	0	0	0	1
0	0	0	0	0	0	0	0	0	0	0	0	0	0
0	0	0	0	0	0	0	0	0	0	0	0	0	2
0	0	0	0	0	0	0	0	0	0	0	0	0	0
0	0	0	0	0	0	0	0	0	0	0	0	0	0
0													

OTU10839	0	1	0	0	0	0	0	0	0	0	0	0	0
0	0	0	0	0	0	0	0	0	0	0	0	0	0
0	0	0	0	0	0	0	0	0	0	0	0	0	0
0	0	0	0	0	0	0	0	0	0	0	0	0	0
0	0	0	0	0	0	0	0	0	0	0	0	0	0
0	0	0	0	0	0	0	0	0	0	0	0	0	0
0	0	0	0	0	0	0	0	0	0	0	0	0	0
0	0	0	0	0	0	0	0	0	0	0	0	0	0
0													
OTU9319	0	1	0	0	0	0	0	0	0	0	0	0	0
0	0	0	0	0	0	0	0	2	0	0	0	0	0
0	0	0	0	0	0	0	0	0	0	0	0	0	0
3	0	0	0	0	0	0	0	0	0	0	0	0	0
0	0	0	0	0	0	0	0	0	0	0	0	1	0
0	0	0	0	0	0	0	1	0	3	0	0	0	0
0	0	0	0	0	0	0	0	0	0	0	2	0	0
1	10	0	2	7	2	3	2	0	0	0	0	0	0
2													
OTU8596	0	1	0	0	0	0	0	0	0	0	0	0	0
0	0	0	0	0	0	0	0	0	0	0	0	0	0
0	0	0	0	0	0	0	0	0	0	0	0	0	0
0	0	0	0	0	0	0	0	0	0	0	0	0	0
0	0	0	0	0	0	0	0	0	0	0	0	0	0
0	0	0	0	0	0	0	0	0	0	0	0	0	0
0	0	0	0	0	0	0	0	0	0	0	0	0	0
0	0	0	0	0	0	0	0	0	0	0	0	0	0
0													
OTU870	0	1	0	0	0	0	0	0	0	0	0	0	0
0	0	0	0	0	0	0	0	0	0	0	0	0	0
0	0	0	0	0	0	0	0	0	0	0	0	0	0
0	0	0	0	0	1	0	0	0	0	0	0	0	0
0	3	0	0	0	0	0	0	0	0	0	0	0	0
0	0	0	0	0	0	0	0	0	0	0	0	0	0

0	0	0	0	0	0	0	0	0	0	0	0	0	0
0	0	0	0	0	0	0	0	0	0	0	0	0	1
0													
OTU1620	0	1	1	1	0	1	2	2	4	1	0	0	0
0	0	0	0	0	0	0	0	0	0	0	0	1	0
1	0	0	0	0	0	0	0	0	0	0	0	0	0
23	1	0	6	0	7	0	0	0	1	0	2	0	0
0	2	0	2	0	0	0	0	0	0	0	0	0	0
0	18	1	0	11	0	0	1	0	14	0	12	0	2
0	0	0	0	0	13	0	0	0	0	0	30	15	0
0	16	8	7	21	2	10	18	3	6	0	1	0	10
3													
OTU4576	0	1	0	0	0	0	0	0	0	0	0	0	0
0	0	0	0	2	0	0	0	0	0	0	0	0	0
0	0	0	0	0	0	0	0	0	0	0	0	0	0
0	0	0	0	0	0	0	0	0	0	0	1	0	0
0	0	0	0	0	0	3	0	0	0	0	0	0	0
0	0	0	0	0	0	0	0	0	0	0	0	0	0
0	0	0	0	0	0	0	0	0	0	0	0	0	0
0	0	0	0	0	0	0	0	0	0	0	0	0	0
0													
OTU12192	0	1	0	0	0	0	0	0	0	0	0	0	0
0	0	0	0	0	0	0	0	0	0	0	0	0	0
0	0	0	0	0	0	0	0	0	0	0	0	0	0
0	0	0	0	0	0	0	0	0	0	0	0	0	0
0	0	0	0	0	0	0	0	0	0	0	0	0	0
0	0	0	0	0	0	0	0	0	0	0	0	0	0
0	0	0	0	0	0	0	0	0	0	0	0	0	0
0	0	0	0	0	0	0	0	0	0	0	0	0	0
0													
OTU8593	0	1	0	0	0	0	0	0	0	0	0	0	0
0	0	0	0	0	0	0	0	0	0	0	0	0	0
0	0	0	0	0	0	0	0	0	0	0	0	0	0

0	0	0	0	0	0	0	0	0	0	0	0	0	0
0	0	0	0	0	0	0	0	0	0	0	0	0	0
0	0	0	0	0	0	0	0	0	0	0	0	0	0
0	0	0	0	0	0	0	0	0	0	0	0	0	0
0	0	0	0	0	0	0	0	0	0	0	0	0	0
0													
OTU1680	0	1	0	2	0	0	0	0	0	0	0	2	0
0	1	0	4	0	0	1	0	3	0	13	7	1	0
2	5	0	0	7	0	4	0	1	2	1	1	1	0
0	1	0	2	0	0	1	1	0	1	0	2	1	0
1	0	2	1	0	0	0	1	0	1	1	0	0	1
0	0	1	1	0	5	1	2	1	0	6	0	4	1
4	2	1	0	3	0	1	0	0	0	0	0	0	1
0	0	0	1	0	0	0	0	0	0	6	1	1	0
0													
OTU5139	0	1	0	4	0	1	0	0	0	0	2	0	0
0	0	0	0	4	1	0	0	0	0	3	0	0	0
0	0	0	0	0	0	0	2	2	0	0	2	3	0
0	0	0	0	0	0	0	7	0	0	2	0	1	8
0	1	0	0	0	0	1	0	0	0	0	0	0	3
0	0	0	0	0	2	0	0	0	0	0	0	0	0
1	0	0	0	1	0	0	0	0	1	0	0	0	4
0	0	0	0	0	0	0	0	0	0	0	0	0	0
0													
OTU16667	0	1	2	0	0	1	0	0	1	2	0	4	0
1	0	4	0	1	0	1	2	0	0	0	0	0	0
1	0	0	0	0	0	0	1	3	0	0	0	1	0
0	1	0	0	0	3	0	4	0	0	3	0	0	8
1	7	0	1	1	0	0	0	0	1	0	0	0	1
0	0	0	0	0	0	0	0	3	1	0	0	1	2
1	0	0	0	3	2	0	1	0	3	4	0	1	0
3	1	0	1	0	0	0	4	0	1	2	0	1	2
2													

OTU10842	0	1	0	0	0	0	0	0	0	0	0	0	0
0	0	0	0	0	0	0	0	0	0	0	0	0	0
0	0	0	0	0	0	0	0	0	0	0	0	0	0
0	0	0	0	0	0	0	0	0	0	0	0	0	0
0	0	0	0	0	0	0	0	0	0	0	0	0	0
0	0	0	0	0	0	0	0	0	0	0	0	0	0
0	0	0	0	0	0	0	0	0	0	0	0	0	0
0	0	0	0	0	0	0	0	0	1	0	0	0	0
0													
OTU12191	0	1	0	0	0	0	0	0	0	0	0	0	0
0	0	0	0	0	0	0	0	0	0	0	0	0	0
0	0	0	0	0	0	0	0	0	0	0	0	0	0
0	0	0	0	0	0	0	0	0	0	0	0	0	0
0	0	0	0	0	0	0	0	0	0	0	0	0	0
0	0	0	0	0	0	0	0	0	0	0	0	0	0
0	0	0	0	0	0	0	0	0	0	0	0	0	0
0	0	0	0	0	0	0	0	0	0	0	0	0	0
0													
OTU646	0	1	0	0	2	0	1	0	0	0	0	0	0
0	0	0	0	0	0	0	0	0	0	0	0	2	0
0	3	0	0	1	0	2	1	0	0	0	0	0	0
0	0	0	0	0	0	0	0	0	0	0	0	0	1
0	0	0	0	0	0	0	0	0	0	0	0	1	0
0	0	4	0	0	0	0	0	0	0	0	0	0	0
0	3	0	1	0	0	5	1	0	0	0	0	0	0
1	0	0	0	0	0	0	0	2	6	1	1	0	0
0													
OTU8594	0	1	0	0	0	0	0	0	0	1	0	0	0
0	0	1	0	0	0	0	0	0	0	0	0	0	0
0	0	0	0	0	0	0	0	0	0	0	0	0	0
0	0	0	0	0	0	0	0	0	0	0	0	0	0
0	0	0	0	0	0	0	0	0	0	0	0	0	0
0	0	0	0	0	0	0	0	0	0	0	0	0	0

1	0	0	0	0	0	0	0	0	0	0	0	0	0
0	0	0	0	0	1	0	0	0	0	0	0	0	0
0													
OTU14589	0	1	0	0	0	0	0	0	0	0	0	0	0
0	0	0	0	0	0	0	0	0	0	0	0	0	0
0	0	0	0	0	0	0	0	0	0	0	0	0	0
0	0	0	0	0	0	0	0	0	0	0	0	0	0
0	0	0	0	0	0	0	0	0	0	0	0	0	0
0	0	0	0	0	0	0	0	0	0	0	0	0	0
0	0	0	0	0	0	0	0	0	0	0	0	0	0
0	0	0	0	0	0	0	0	0	0	0	0	0	0
0													
OTU5003	0	1	0	0	0	0	0	1	0	2	3	0	0
0	1	1	0	2	2	2	0	1	1	0	1	0	0
1	1	2	0	0	0	1	1	0	0	0	0	1	2
1	0	1	1	1	1	0	0	0	0	0	0	0	0
0	0	0	1	0	2	0	0	0	0	0	0	0	0
1	3	3	0	1	0	0	2	0	0	0	1	0	0
0	0	1	1	0	1	1	1	0	1	4	0	0	0
1	0	2	1	0	0	0	2	0	1	0	0	0	1
0													
OTU14588	0	1	0	0	0	0	0	0	0	0	0	0	0
0	0	0	0	0	0	0	0	0	0	0	0	0	0
0	0	0	0	0	0	0	0	0	0	0	0	0	0
0	0	0	0	0	0	0	0	0	0	0	0	0	0
0	0	0	0	0	0	0	0	0	0	0	0	0	0
0	0	0	0	0	0	0	0	0	0	0	0	0	0
0	0	0	0	0	0	0	0	0	0	0	0	0	0
0	0	0	0	0	0	0	0	0	0	0	0	0	0
0													
OTU7473	0	1	3	0	0	6	2	0	1	0	0	2	1
1	1	5	0	1	0	0	6	0	0	0	0	0	4
0	0	0	3	0	0	1	0	1	0	0	1	0	0

0 0 1 1 0 5 0 0 0 0 2 0 0 0
0 0 1 7 1 0 2 1 1 0 0 1 1 0
0 0 1 4 4 0 0 1 1 0 0 2 0 0
2 3 1 0 0 0 3 1 0 16 5 0 1 4
1 0 0 1 1 0 0 0 3 3 0 10 7 2
4
OTU1877 0 1 1 0 1 0 0 1 2 1 1 0 1
0 0 2 0 1 0 0 2 0 1 0 0 0 0
0 0 1 0 0 0 1 0 0 2 0 3 0 0
3 0 0 0 0 0 1 1 0 4 1 1 0 0
0 0 1 0 0 0 0 1 2 0 1 0 1 0
2 0 1 1 4 1 0 2 0 0 0 0 0 1
1 1 0 1 1 0 0 1 0 0 1 0 0 1
0 0 1 0 2 1 1 1 0 0 3 0 0 0
1
OTU1316 0 0 26 1 3 0 14 7 38 11 0 0 2
0 0 0 0 0 1 13 2 0 0 0 0 0 0
1 0 1 0 0 0 0 0 0 2 0 0 0 0
16 5 0 3 0 0 0 0 0 0 0 1 0 0
0 14 0 0 0 0 0 0 0 0 1 0 0 0
0 0 0 0 35 0 0 1 1 48 4 0 3 21
2 0 0 10 11 30 0 0 0 0 1 3 0 0
0 0 0 0 0 0 0 1 0 0 4 0 1 0
0
OTU10771 0 0 3 0 0 0 0 0 0 0 0 0 0
0 0 0 0 0 0 0 0 0 0 0 0 0 0
0 0 0 0 0 0 0 0 0 0 0 0 0 0
0 0 0 0 1 0 0 0 0 0 0 0 0 0
0 0 0 0 0 0 0 0 0 0 0 0 0 0
0 0 0 0 0 0 0 0 0 0 0 0 0 0
0 0 0 0 0 0 0 0 0 0 0 0 0 0
0 0 0 0 0 0 0 0 0 0 0 0 0 0
0

OTU764	0	0	41	0	0	0	0	0	0	0	0	0	0
0	0	0	0	0	0	0	0	0	0	0	0	0	0
0	0	0	0	0	0	0	0	0	0	0	3	0	0
0	0	0	0	5	0	0	0	0	0	0	0	0	0
0	0	0	0	0	0	0	0	0	0	0	0	0	0
0	0	0	0	0	0	0	0	0	0	0	0	0	0
0	2	0	0	0	0	0	0	0	0	0	0	0	0
0	0	0	0	0	2	0	0	0	0	0	0	0	0
0													
OTU407	0	0	5	0	0	0	0	0	0	0	1	0	7
0	0	5	11	0	0	0	0	20	0	0	0	1	0
5	3	0	0	7	0	4	1	0	0	1	0	0	0
1	1	11	0	6	8	0	0	0	0	0	0	0	0
3	0	0	6	0	6	0	1	1	6	13	0	6	1
2	16	0	0	0	0	0	0	0	0	0	1	2	0
0	1	1	1	0	0	0	1	30	0	0	1	0	14
0	3	0	0	34	0	4	0	0	0	0	3	1	0
0													
OTU10837	0	0	1	0	0	0	0	0	0	0	0	0	0
0	0	0	0	0	0	0	0	0	0	0	0	0	0
0	0	0	0	0	0	0	0	0	0	0	0	0	0
0	0	0	0	0	0	0	0	0	0	0	0	0	0
0	0	0	0	0	0	0	0	0	0	0	0	0	0
0	0	0	0	0	0	0	0	0	0	0	0	0	0
0	0	0	0	0	0	0	0	0	0	0	0	0	0
0	0	0	0	0	0	0	0	0	0	0	0	0	0
0													
OTU10838	0	0	1	0	0	0	0	0	0	0	0	0	0
0	0	0	0	0	0	0	0	0	0	0	0	0	0
0	0	0	0	0	0	0	0	0	0	0	0	0	0
0	0	0	0	0	0	0	0	0	0	0	0	0	0
0	0	0	0	0	0	0	0	0	0	0	0	0	0
0	0	0	0	0	0	0	0	0	0	0	0	0	0

0	0	0	0	0	0	0	0	0	0	0	0	0	0
0	0	0	0	0	0	0	0	0	0	0	0	0	0
0													
OTU15697	0	0	1	0	0	0	4	0	1	0	1	0	3
1	9	6	0	0	4	0	1	0	0	2	0	0	4
0	1	0	0	2	0	0	0	0	7	0	20	1	0
0	1	0	0	0	0	0	0	0	0	0	0	0	0
0	0	0	0	0	0	0	0	0	0	0	1	0	0
1	0	1	0	1	0	0	0	0	0	0	0	3	0
0	0	0	1	0	0	0	0	0	1	0	0	0	0
1	0	0	0	0	0	0	1	0	0	0	0	3	0
0													
OTU260	0	0	2	0	1	0	2	2	36	3	0	0	0
0	0	0	0	0	0	0	0	0	0	0	0	0	0
0	0	0	1	0	0	0	0	1	0	0	0	0	0
1	0	0	0	0	0	0	1	0	0	0	0	0	0
0	0	0	1	0	0	1	0	0	0	0	0	0	0
3	51	0	0	2	0	0	1	0	11	1	10	0	9
0	0	0	3	0	2	0	8	0	0	0	12	15	0
0	13	2	52	46	14	1	64	10	28	0	2	0	2
62													
OTU500	0	3	0	3	5	0	5	12	7	0	18	1	0
0	0	32	0	0	4	85	33	0	19	0	0	0	78
163	0	0	26	0	8	4	5	0	50	0	24	0	120
1	30	3	123	1	1	0	4	8	235	27	75	3	7
1	14	9	46	2	69	15	39	4	52	10	79	65	41
2	13	8	22	0	0	5	1	0	2	279	2	608	0
5	9	0	4	0	1	494	0	5	7	1	1	3	36
0	30	7	7	6	8	4	3	3	0	1	144	8	10
OTU8597	0	0	1	0	0	0	0	0	0	0	0	0	0
0	0	0	0	0	0	0	0	0	0	0	0	0	0
0	0	0	0	0	0	0	0	0	0	0	0	0	0
0	0	0	0	0	0	0	0	0	0	0	0	0	0

0	0	0	0	0	0	0	0	0	0	0	0	0	0
0	0	0	0	0	0	0	0	0	0	0	0	0	0
0	0	0	0	0	0	0	0	0	0	0	0	0	0
0	0	0	0	0	0	0	0	0	0	0	0	0	0
0													
OTU10830	0	0	7	0	0	0	0	0	0	0	0	0	0
0	0	0	0	0	0	0	0	0	0	0	0	0	0
0	0	0	0	0	0	0	0	0	0	0	0	0	0
0	0	0	0	0	0	0	0	0	0	0	0	0	0
0	0	0	0	0	0	0	0	0	0	0	0	0	0
0	0	0	0	0	0	0	0	0	0	0	0	0	0
0	1	0	0	0	0	0	0	0	0	0	0	0	0
0	0	0	0	0	1	0	0	0	0	0	0	0	0
0													
OTU416	0	0	31	0	0	2	22	6	6	3	0	0	0
0	4	0	0	0	0	0	0	0	0	0	0	0	0
2	0	0	0	0	0	0	0	0	0	0	0	23	0
0	0	1	0	0	0	0	0	0	0	0	0	0	0
0	0	0	0	0	0	0	0	0	0	0	0	1	0
0	0	0	0	2	0	0	1	0	5	0	0	0	11
0	1	0	2	0	0	0	0	0	0	0	0	0	0
0	0	0	0	0	0	0	0	0	0	0	0	0	0
0													
OTU498	0	0	1	0	0	0	0	0	5	1	0	0	0
0	0	0	0	0	0	0	0	0	0	0	0	0	0
0	0	0	0	0	0	0	0	0	0	0	0	0	0
4	0	0	0	0	0	0	0	0	0	0	0	0	0
0	0	0	0	0	0	0	0	0	0	0	0	0	0
0	1	0	0	0	0	0	0	0	0	0	1	0	0
0	0	0	0	0	0	0	4	0	0	0	0	3	0
0	2	1	2	3	0	0	0	0	3	0	0	0	5
0													

OTU5803	0	0	1	0	0	0	0	1	0	0	0	1	0
0	0	0	0	0	0	0	0	0	0	0	0	0	0
0	0	0	0	0	0	0	0	0	0	0	0	2	0
0	0	0	0	0	1	1	1	1	3	0	0	0	0
0	0	0	0	0	0	0	0	1	0	1	0	0	0
1	0	0	0	2	0	0	0	0	1	0	1	0	1
0	0	0	0	0	2	0	2	1	0	0	1	0	0
0	0	1	1	0	1	0	0	0	0	0	0	0	1
0													
OTU8598	0	0	1	0	0	0	0	0	0	0	0	0	0
0	0	0	0	0	0	0	0	0	0	0	0	0	0
0	0	0	0	0	0	0	0	0	0	0	0	0	0
0	0	0	0	0	0	0	0	0	0	0	0	0	0
0	0	0	0	0	0	0	0	0	0	0	0	0	0
0	0	0	0	0	0	0	0	0	0	0	0	0	0
0	0	0	0	0	0	0	0	0	0	0	0	0	0
0	0	0	0	0	0	0	0	0	0	0	0	0	0
0													
OTU7420	0	0	2	0	0	0	0	0	0	0	0	0	0
0	0	0	0	0	0	0	0	0	0	0	0	0	0
0	0	0	0	0	0	0	0	0	0	0	0	0	0
0	0	0	0	2	0	0	0	0	0	0	0	0	0
0	0	0	0	0	0	0	0	0	0	0	0	0	0
0	0	0	0	0	0	0	0	0	0	0	0	0	0
0	1	0	0	0	0	0	0	0	0	0	0	0	0
0	0	0	0	0	0	0	0	0	1	0	0	0	0
0													
OTU11455	0	0	2	0	3	0	1	0	0	1	0	1	0
0	0	0	0	1	0	0	1	0	0	0	1	0	0
0	0	1	0	0	2	0	0	0	0	0	3	0	2
0	0	1	0	1	0	0	0	1	1	0	0	0	0
0	0	0	1	0	2	0	0	0	0	0	0	1	0
0	1	0	0	1	1	1	0	0	0	0	1	0	0

1	0	0	0	0	2	1	1	0	1	1	0	0	0
0	0	1	0	4	0	0	0	0	2	1	0	0	0
1													
OTU414	0	0	1	0	0	0	0	0	0	1	0	0	0
0	0	5	0	1	0	0	7	0	0	0	2	0	0
0	0	0	0	0	2	0	0	0	6	1	0	0	0
0	0	0	1	2	0	2	0	0	0	3	0	0	0
0	0	0	0	0	0	1	0	0	0	0	2	0	0
0	0	0	1	0	0	1	2	1	0	0	4	0	0
0	0	0	0	0	0	0	0	0	0	0	1	0	0
0	0	2	0	0	4	0	1	1	0	0	0	4	0
0													
OTU6241	0	0	1	0	0	0	0	0	0	0	0	1	0
0	0	0	0	0	0	0	0	0	0	1	0	0	0
0	0	0	0	0	0	0	0	0	0	0	0	0	0
0	0	0	0	1	0	0	1	0	0	0	0	0	0
0	0	0	0	0	0	0	0	0	0	0	0	0	0
0	0	0	0	0	0	0	0	0	1	0	0	0	0
0	0	0	0	0	0	0	0	0	1	0	0	0	0
0	0	0	0	0	0	0	0	0	0	0	0	1	0
0													
OTU2906	0	0	1	0	0	3	0	0	0	0	0	0	0
0	0	0	0	0	0	2	0	0	0	0	0	0	0
0	0	0	0	0	0	0	0	0	0	1	0	0	0
0	0	0	0	0	0	0	0	0	0	0	0	0	0
0	0	0	0	0	0	0	0	0	0	0	0	0	0
0	0	0	0	0	0	0	0	0	0	0	0	0	0
0	0	0	0	0	0	0	0	0	0	0	0	0	0
0	0	0	2	0	0	0	0	2	1	0	0	0	0
0													
OTU320	0	0	16	0	0	0	0	2	10	5	0	0	1
0	0	0	11	2	0	2	0	3	2	4	1	25	0
99	2	0	0	5	2	7	0	0	0	11	0	23	0

	19	2	1	0	0	3	0	4	7	3	3	8	0	0
	0	2	0	1	0	0	2	3	16	0	0	1	3	0
	1	0	0	0	1	8	0	0	0	0	0	15	0	15
	0	1	0	0	0	0	0	0	0	0	0	0	0	0
	0	1	0	0	0	0	0	0	0	0	0	0	0	0
	0													
OTU28	0	0	2	0	0	3	1	0	0	0	4	1	7	1
	0	0	1	0	2	9	1	641	0	517	0	0	59	14
	181	0	0	1059	0	129	6	1	2	11	0	2	0	0
	0	707	0	403	0	1	0	374	1830	32	22	102	10	476
	4	19	5	436	1399	66	708	406	1059	4589	298	60	16	1211
	0	1	2	0	0	0	2	0	8	0	2	1	4	1
	6	0	0	0	0	0	0	68	3	0	0	0	0	19
	0	0	6	0	1	0	0	0	0	0	0	4	1	0
OTU12294		0	0	1	0	0	0	0	0	0	0	0	0	0
	0	0	0	0	0	0	0	0	0	0	0	0	0	0
	0	0	0	0	0	0	0	0	0	0	0	0	0	0
	0	0	0	0	0	0	0	0	0	0	0	0	0	0
	0	0	0	0	0	0	0	0	0	0	0	0	0	0
	0	0	0	0	0	0	0	0	0	0	0	0	0	0
	0	0	0	0	0	0	0	0	0	0	0	0	0	0
	0	0	0	0	0	0	0	0	0	0	0	0	0	0
	0													
OTU12295		0	0	1	0	0	0	0	0	0	0	0	0	0
	0	0	0	0	0	0	0	0	0	0	0	0	0	0
	0	0	0	0	0	0	0	0	0	0	0	0	0	0
	0	0	0	0	0	0	0	0	0	0	0	0	0	0
	0	0	0	0	0	0	0	0	0	0	0	0	0	0
	0	0	0	0	0	0	0	0	0	0	0	0	0	0
	0	0	0	0	0	0	0	0	0	0	0	0	0	0
	0	0	0	0	0	0	0	0	0	0	0	0	0	0
	0													

OTU99 0 0 10 1 1 4 1 1 14 5 0 1 0 0
0 0 0 0 0 0 0 0 0 0 0 0 0 0
0 0 0 0 0 0 0 2 0 0 0 0 1 0
1 0 0 0 0 0 2 0 0 0 0 0 2 0
1 0 1 0 0 0 0 1 0 1 0 2 0 0
65 1 0 0 0 0 1 0 0 11 0 0 0 0
0 0 1 0 0 0 0 0 0 0 237 127 0 0
113 104 0 226 45 82 245 85 125 1 9 0 9 23
OTU720 0 0 2 0 1 0 1 0 0 2 0 1 0
0 0 0 0 0 0 0 0 0 0 0 0 0 0
0 0 0 0 0 0 0 0 0 0 0 0 0 0
0 0 0 1 0 0 0 0 0 0 1 0 0 0
0 0 0 0 0 0 0 0 0 0 0 0 0 0
0 2 0 0 0 0 0 0 0 0 0 0 0 0
0 0 1 1 0 0 0 0 0 2 0 1 2 0
0 3 0 2 1 0 0 5 1 3 0 1 0 2
0
OTU2496 0 0 5 0 1 0 0 2 0 1 0 17 0
0 0 0 0 0 0 1 0 0 0 0 1 0 0
0 0 0 0 0 0 0 9 0 3 0 0 0 0
2 9 0 2 0 3 1 3 0 0 6 0 0 1
0 11 0 2 0 0 0 0 0 0 0 0 0 0
0 0 6 9 0 0 0 0 0 1 2 0 8 0
0 0 0 3 0 0 2 0 0 20 5 0 0 0
6 0 0 0 0 0 0 0 1 2 6 2 0 0
0
OTU10823 0 0 1 0 0 0 0 0 0 0 0 0 0
0 0 0 0 0 0 0 0 0 0 0 0 0 0
0 0 0 0 0 0 0 0 0 0 0 0 0 0
0 0 0 0 0 0 0 0 0 0 0 0 0 0
0 0 0 0 0 0 0 0 0 0 0 0 0 0
0 0 0 0 0 0 0 0 0 0 0 0 0 0
0 0 0 0 0 0 0 0 0 0 0 0 0 0

0	0	0	0	0	0	0	0	0	0	0	0	0	0
0													
OTU3757	0	0	3	17	0	0	1	6	4	5	1	0	1
0	1	0	5	2	2	5	0	1	0	28	118	4	0
0	0	0	62	1	0	0	0	0	0	1	0	0	0
0	0	0	0	0	0	0	0	26	3	6	0	0	2
1	13	0	0	0	0	0	0	0	6	0	0	0	0
0	1	0	21	0	23	0	1	0	2	0	2	0	5
18	0	24	8	0	0	8	1	0	0	0	0	0	0
6	0	0	0	0	0	0	0	0	0	7	0	0	0
0													
OTU159	0	0	22	0	0	5	8	28	183	10	0	0	0
0	0	0	0	0	0	0	0	0	0	0	0	0	0
0	0	0	0	0	0	0	0	0	0	0	0	2	0
0	0	1	0	0	0	0	0	0	0	0	15	0	0
0	0	0	1	0	0	0	0	8	0	2	0	1	0
0	3	119	0	1	0	0	21	0	11	1	0	0	5
0	3	0	0	0	0	0	561	0	0	0	0	3	0
0	0	2	6	2	7	2	1	15	2	0	1	0	0
0													
OTU11405	0	0	1	0	0	0	0	0	0	0	0	3	1
1	0	1	1	0	0	0	0	2	0	0	0	0	0
0	2	0	2	0	0	0	1	6	0	2	0	0	0
0	0	0	0	2	0	0	0	2	0	1	2	0	0
1	1	2	0	1	1	0	0	0	1	1	4	0	1
0	0	0	3	0	0	0	0	1	0	0	0	0	1
0	0	0	0	0	0	6	8	0	0	1	0	0	2
1	0	0	0	0	0	0	0	0	0	0	0	1	0
0													
OTU14591	0	0	1	0	0	0	0	0	0	0	0	0	0
0	0	0	0	0	0	0	0	0	0	0	0	0	0
0	0	0	0	0	0	0	0	0	0	0	0	0	0
0	0	0	0	0	0	0	0	0	0	0	0	0	0

0	0	0	0	0	0	0	0	0	0	0	0	0	0
0	0	0	0	0	0	0	0	0	0	0	0	0	0
0	0	0	0	0	0	0	0	0	0	0	0	0	0
0	0	0	0	0	0	0	0	0	0	0	0	0	0
0													
OTU7128	0	0	1	0	0	1	0	1	0	1	1	1	0
0	1	1	0	0	0	0	0	0	0	0	0	0	0
0	0	1	0	0	1	0	1	0	0	0	0	0	1
0	0	1	0	0	0	1	0	0	1	0	0	0	0
0	0	1	0	0	0	0	0	1	0	0	0	0	0
1	0	0	1	2	0	0	1	0	0	0	1	0	1
0	0	0	1	0	0	0	1	0	0	0	0	0	0
1	0	0	0	1	1	0	0	1	1	0	0	0	0
2													
OTU393	0	0	1	5	3	1	2	0	5	1	0	1	0
0	0	0	0	0	0	2	0	0	0	0	0	0	0
0	0	0	0	0	0	0	1	1	0	1	0	3	0
0	0	0	0	0	1	0	1	0	0	1	1	0	1
0	4	0	2	0	0	0	0	0	0	0	0	2	0
0	2	0	0	2	0	0	0	0	3	0	1	0	2
0	0	0	1	0	2	4	2	0	0	0	1	0	1
1	1	3	3	3	5	1	0	3	2	0	0	1	3
0													
OTU5275	0	0	14	0	0	0	0	0	0	0	0	0	0
0	0	0	0	0	0	0	0	0	0	0	0	0	0
0	0	0	0	0	0	0	0	0	0	0	0	0	2
0	0	0	0	2	0	0	0	0	0	0	0	0	0
0	0	0	0	0	0	0	0	0	0	0	0	0	0
0	0	1	0	0	0	0	0	0	0	0	0	0	0
0	1	0	0	0	0	0	0	0	0	0	0	0	0
0	0	0	0	0	0	0	0	0	0	0	0	0	0
0													

OTU9698		0	0	1	0	1	0	0	0	1	0	0	0	0
	0	0	0	0	0	0	0	0	0	1	0	0	0	0
	0	0	3	0	0	1	0	0	0	0	0	0	0	0
	0	0	0	0	0	1	1	0	0	0	0	0	1	0
	1	0	0	0	0	0	0	0	0	0	0	0	0	0
	0	0	0	0	0	0	0	0	0	0	0	1	0	0
	0	0	0	0	0	0	0	1	0	0	0	0	0	0
	0	0	1	0	0	0	0	0	0	0	0	0	0	0
	0													
OTU10763		0	0	16	0	0	0	0	0	0	0	0	0	0
	0	0	0	0	0	0	0	0	0	0	0	0	0	0
	0	0	0	0	0	0	0	0	0	0	0	0	0	0
	0	0	0	0	0	0	0	0	0	0	0	0	0	0
	0	0	0	0	0	0	0	0	0	0	0	0	0	0
	0	0	0	0	0	0	0	0	0	0	0	0	0	0
	0	0	0	0	0	0	0	0	0	0	0	0	0	0
	0	0	0	0	0	0	0	0	0	1	0	0	0	0
	0													
OTU32	0	0	2	72	0	3	7	0	1	24	15	0	0	0
	1	100	2	273	2	138	2	0	13	0	30	1	263	117
	0	14	1816	0	152	0	0	0	8	0	80	0	0	82
	44	0	3	0	0	0	4	1	0	0	9	0	0	0
	1	0	2	0	1	0	3	0	0	1	0	2	1	0
	0	0	0	0	1	3004	0	0	16	4	60	41	2	6
	0	3	4	2	12	4	0	4	1092	0	0	0	0	0
	0	1	0	0	0	0	0	0	0	10	0	2	0	0
OTU265		0	0	16	0	0	1	0	6	7	75	0	17	0
	0	0	3	0	0	4	0	0	0	1	0	0	0	0
	0	1	0	50	0	0	0	0	1	0	1	0	0	22
	1	0	0	0	12	1	0	1	0	0	0	0	0	0
	0	0	0	1	0	0	7	0	0	0	0	0	0	1
	0	13	0	0	0	4	0	0	32	0	0	0	0	0
	0	3	0	0	0	0	0	0	0	4	0	3	20	0

0	3	4	31	5	3	1	10	2	3	0	3	1	144
0													
OTU2279	0	0	1	0	0	1	0	0	1	0	0	0	0
0	0	0	0	1	0	0	0	1	0	0	0	0	0
0	0	1	0	0	0	0	0	0	2	0	1	0	0
1	0	1	0	0	0	0	0	0	1	0	0	0	0
0	0	0	2	0	0	0	0	0	0	0	0	1	0
0	0	0	1	0	0	0	1	1	0	0	0	0	0
0	0	0	0	0	0	0	2	0	0	0	1	0	0
1	0	0	0	0	0	0	0	2	0	0	0	0	1
0													
OTU12298	0	0	4	0	0	0	0	0	0	0	0	0	0
0	0	0	0	0	0	0	0	0	0	0	0	0	0
0	0	0	0	0	0	0	0	0	0	0	0	0	1
0	0	0	0	1	0	0	0	0	0	0	0	0	0
0	0	0	0	0	0	0	0	0	0	0	0	0	0
0	0	0	0	0	0	0	0	0	0	0	0	0	0
0	0	0	0	0	0	0	0	0	0	0	0	0	0
0	0	0	0	0	4	0	0	0	3	0	0	0	0
0													
OTU6755	0	0	1	0	0	0	0	0	0	0	0	0	0
0	0	0	0	0	0	0	0	0	0	0	0	0	0
0	0	0	0	0	0	0	0	0	0	0	0	0	0
0	0	0	0	0	0	0	0	0	0	0	0	0	0
0	0	0	0	0	0	0	0	0	0	0	0	0	0
0	0	0	0	0	0	0	0	0	0	0	0	0	0
0	0	0	1	0	0	0	0	0	0	0	0	0	0
0	0	0	0	0	0	0	0	0	0	0	0	0	0
0													
OTU4520	0	0	10	2	0	0	0	7	6	1	0	3	0
4	2	2	3	0	0	6	0	0	0	0	0	0	0
6	0	0	0	0	0	0	0	3	0	0	0	1	1
7	3	0	0	6	5	0	8	0	0	0	4	0	1

0	8	0	16	0	1	0	0	1	2	0	0	2	1
6	3	0	1	1	0	0	2	8	6	0	2	0	2
0	2	0	2	11	0	8	11	0	1	0	3	5	0
0	5	1	3	0	0	3	1	4	1	0	0	0	5
1													
OTU2623	0	0	4	0	0	0	0	1	3	4	0	0	0
0	0	0	0	0	0	0	0	0	0	0	0	0	0
1	0	0	0	0	0	0	0	0	0	0	0	0	0
0	0	0	0	0	0	0	0	0	0	0	0	0	0
0	0	0	0	0	0	0	0	0	0	0	0	0	0
0	0	0	0	0	0	0	0	0	2	0	0	0	1
0	0	0	0	0	0	0	1	0	0	0	0	0	0
0	0	0	0	0	0	0	0	0	0	0	0	0	0
0													
OTU2951	0	0	10	0	0	1	2	2	8	12	0	3	0
0	0	0	1	0	0	2	2	0	0	1	0	0	0
6	0	0	0	0	0	0	1	0	0	0	0	3	0
5	4	0	0	0	0	0	1	0	0	0	1	0	0
0	0	0	8	0	0	0	1	1	0	0	0	17	0
0	1	2	0	0	0	0	1	0	3	0	6	0	8
0	0	0	2	0	0	0	1	0	0	0	4	0	0
0	3	3	2	4	4	3	1	2	1	0	0	0	7
5													
OTU8971	0	0	8	0	0	2	0	0	14	3	0	1	0
0	0	0	0	0	0	0	0	1	0	0	0	0	0
12	0	0	0	0	0	0	0	0	0	0	0	16	0
7	1	0	0	0	0	0	0	0	0	0	3	0	1
0	10	0	3	1	0	0	0	0	0	1	0	1	1
0	5	0	0	0	0	0	0	0	1	0	1	0	1
0	2	0	4	0	1	0	4	0	0	0	1	5	0
0	3	5	0	4	0	3	2	2	0	0	0	0	3
3													

OTU10824	0	0	1	0	0	0	0	0	0	0	0	0	0
0	0	0	0	0	0	0	0	0	0	0	0	0	0
0	0	0	0	0	0	0	0	0	0	0	0	0	0
0	0	0	0	0	0	0	0	0	0	0	0	0	0
0	0	0	0	0	0	0	0	0	0	0	0	0	0
0	0	0	0	0	0	0	0	0	0	0	0	0	0
0	0	0	0	0	0	0	0	0	0	0	0	0	0
0	0	0	0	0	0	0	0	0	0	0	0	0	0
0													
OTU222	0	0	12	0	0	0	1	0	0	0	1	8	0
0	0	0	0	0	1	0	0	0	0	0	8	0	1
0	0	0	1	0	0	0	0	0	260	0	0	0	0
0	25	0	5	0	1	3	0	0	0	0	0	0	0
0	5	0	0	0	0	0	1	1	0	0	0	2	0
0	0	0	1	6	13	26	0	9	0	104	1	1	0
79	2	1	0	12	1	0	0	1	1	0	0	0	0
0	2	0	0	1	0	0	4	0	0	10	0	0	1
0													
OTU13316	0	0	4	0	2	0	0	0	0	0	0	0	0
0	0	0	0	0	0	0	0	0	0	0	0	0	0
0	0	0	0	0	0	0	0	0	0	0	2	0	0
0	0	0	0	0	0	0	0	0	0	0	0	0	0
0	0	0	0	0	0	0	0	0	0	0	0	0	0
0	0	0	0	0	0	0	0	0	0	0	0	0	0
0	0	0	0	0	0	0	0	0	0	0	0	0	0
0	0	0	0	0	0	0	0	0	0	0	0	0	0
0													
OTU12296	0	0	1	0	0	0	0	0	0	0	0	0	0
0	0	0	0	0	0	0	0	0	0	0	0	0	0
0	0	0	0	0	0	0	0	0	0	0	0	0	0
0	0	0	0	0	0	0	0	0	0	0	0	0	0
0	0	0	0	0	0	0	0	0	0	0	0	0	0
0	0	0	0	0	0	0	0	0	0	0	0	0	0

0	0	0	0	0	0	0	0	0	0	0	0	0	0
0	0	0	0	0	0	0	0	0	0	0	0	0	0
0													
OTU2584	0	0	1	2	1	0	1	0	0	0	0	0	0
0	1	0	0	0	0	0	0	0	0	0	0	0	0
0	0	0	0	1	0	0	0	0	0	0	0	0	0
0	0	1	0	3	0	0	0	0	0	0	0	0	0
0	0	0	0	0	0	0	0	0	0	0	0	0	0
0	0	0	0	0	0	0	0	0	0	0	0	0	0
0	0	0	0	0	0	0	0	0	0	0	0	0	0
0	0	1	0	0	0	0	0	0	0	0	0	0	0
0													
OTU9148	0	0	2	0	0	0	0	0	0	0	0	0	0
0	0	0	0	0	0	0	0	0	0	0	0	0	0
0	0	0	0	0	0	0	0	0	0	1	0	0	0
2	0	0	0	0	0	0	0	0	0	0	0	0	0
0	0	0	0	0	0	0	0	0	0	0	0	0	0
0	0	0	0	0	0	0	0	0	0	0	0	0	0
0	0	0	0	0	0	0	0	0	0	0	0	0	0
0	2	0	0	4	0	1	0	0	0	0	0	0	0
1													
OTU12297	0	0	1	0	0	0	0	0	0	0	0	0	0
0	0	0	0	0	0	0	0	0	0	0	0	0	0
0	0	0	0	0	0	0	0	0	0	0	0	0	0
0	0	0	0	0	0	0	0	0	0	0	0	0	0
0	0	0	0	0	0	0	0	0	0	0	0	0	0
0	0	0	0	0	0	0	0	0	0	0	0	0	0
0	0	0	0	0	0	0	0	0	0	0	0	0	0
0	0	0	0	0	0	0	0	0	0	0	0	0	0
0													
OTU10825	0	0	1	0	0	0	0	0	0	0	0	0	0
0	0	0	0	0	0	0	0	0	0	0	0	0	0
0	0	0	0	0	0	0	0	0	0	0	0	0	0

0	0	0	0	0	0	0	0	0	0	0	0	0	0
0	0	0	0	0	0	0	0	0	0	0	0	0	0
0	0	0	0	0	0	0	0	0	0	0	0	0	0
0	0	0	0	0	0	0	0	0	0	0	0	0	0
0	0	0	0	0	0	0	0	0	0	0	0	0	0
0													
OTU14594	0	0	1	0	0	0	0	0	0	0	0	0	0
0	0	0	0	0	0	0	0	0	0	0	0	0	0
0	0	0	0	0	0	0	0	0	0	0	0	0	0
0	0	0	0	0	0	0	0	0	0	0	0	0	0
0	0	0	0	0	0	0	0	0	0	0	0	0	0
0	0	0	0	0	0	0	0	0	0	0	0	0	0
0	0	0	0	0	0	0	0	0	0	0	0	0	0
0	0	0	0	0	0	0	0	0	0	0	0	0	0
0													
OTU5260	0	0	4	1	1	0	0	1	1	0	0	0	0
1	0	0	1	3	0	0	0	0	0	0	0	0	0
1	1	1	1	1	0	0	0	1	2	1	2	3	1
0	1	0	0	0	3	0	0	0	1	1	1	2	1
1	0	0	0	1	0	0	0	0	0	1	0	0	0
0	5	0	2	1	1	0	1	0	1	0	2	0	1
0	0	1	0	0	2	0	1	1	1	0	0	0	0
0	2	0	0	0	1	2	0	0	1	1	0	0	0
1													
OTU1337	0	0	1	0	0	0	1	0	4	2	0	0	0
0	0	0	0	0	0	0	0	0	0	0	0	0	0
0	0	0	0	0	0	0	0	0	0	0	0	4	4
1	0	0	0	0	1	0	0	0	0	0	0	0	1
0	0	0	0	0	0	1	0	0	0	0	0	0	0
0	6	0	0	2	0	0	0	0	0	0	1	0	0
0	0	0	3	0	0	0	2	0	0	0	6	2	0
0	11	11	28	18	15	12	15	6	6	0	1	0	26
9													

OTU8587	0	0	1	0	0	0	0	0	0	0	0	0	0
0	0	0	0	0	0	0	0	0	0	0	0	0	0
0	0	0	0	0	0	0	0	0	0	0	0	0	0
0	0	0	0	0	0	0	0	0	0	0	0	0	0
0	0	0	0	0	0	0	0	0	0	0	0	0	0
0	0	0	0	0	0	0	0	0	0	0	0	0	0
0	0	0	0	0	0	0	0	0	0	0	0	0	0
0	0	0	0	0	0	0	0	0	0	0	0	0	0
0													
OTU10759	0	0	2	0	0	0	0	0	0	0	0	0	0
0	0	0	0	0	0	0	0	0	0	0	0	0	0
0	0	0	0	0	0	0	0	0	0	0	0	0	0
0	0	0	0	0	0	0	0	0	0	0	0	0	0
0	0	0	0	0	0	0	0	0	0	0	0	0	0
0	0	0	0	0	0	0	0	0	0	0	0	0	0
0	0	0	0	0	0	0	0	0	0	0	0	0	0
0	0	0	0	0	0	0	0	0	0	0	0	0	0
0													
OTU8588	0	0	1	0	0	0	0	0	0	0	0	0	0
0	0	0	0	0	0	0	0	0	0	0	0	0	0
0	0	0	0	0	0	0	0	0	0	0	0	0	0
0	0	0	0	0	0	0	0	0	0	0	0	0	0
0	0	0	0	0	0	0	0	0	0	0	0	0	0
0	0	0	0	0	0	0	0	0	0	0	0	0	0
0	0	0	0	0	0	0	0	0	0	0	0	0	0
0	0	0	0	0	0	0	0	0	0	0	0	0	0
0													
OTU14592	0	0	1	0	0	0	1	0	0	1	0	0	0
0	0	1	0	0	0	0	0	0	0	0	0	0	0
0	0	0	0	0	0	0	0	0	0	0	0	0	0
0	1	0	0	0	0	0	0	0	0	0	1	0	0
0	0	0	0	0	0	0	0	0	0	0	0	1	0
0	0	0	0	0	0	0	0	0	0	0	0	0	0

0	0	0	0	0	0	0	0	0	0	0	0	0	0
0	0	0	0	1	0	0	0	0	0	1	0	0	0
0													
OTU375	0	0	4	4	0	0	0	0	0	0	0	0	0
0	0	0	0	2	0	3	0	0	0	2	0	25	0
0	0	0	0	0	0	0	0	0	5	0	0	1	0
0	2	0	0	0	0	0	0	0	0	0	0	0	6
0	9	0	0	0	0	0	0	0	0	0	0	6	0
0	0	0	0	0	0	0	0	0	0	0	0	0	0
0	0	0	0	0	0	0	0	0	0	0	0	0	0
0	0	0	1	0	0	0	0	0	0	0	0	0	0
0													
OTU16442	0	0	2	0	1	0	1	0	0	1	1	0	1
1	0	2	0	0	0	0	0	0	0	0	0	0	1
0	0	0	2	0	1	0	0	0	0	0	0	0	1
1	0	0	1	0	0	0	0	0	0	0	0	0	0
1	1	0	0	1	0	0	0	1	0	0	0	1	0
0	0	1	0	2	0	0	0	1	0	1	1	0	0
1	0	0	0	0	0	1	0	0	0	0	0	0	0
0	1	0	0	0	0	0	0	0	1	0	0	0	0
2													
OTU10819	0	0	1	0	0	0	0	0	0	0	0	0	0
0	0	0	0	0	0	0	0	0	0	0	0	0	0
0	0	0	0	0	0	0	0	0	0	0	0	0	0
0	0	0	0	0	0	0	0	0	0	0	0	0	0
0	0	0	0	0	0	0	0	0	0	0	0	0	0
0	0	0	0	0	0	0	0	0	0	0	0	0	0
0	0	0	0	0	0	0	0	0	0	0	0	0	0
0	0	0	0	0	0	0	0	0	0	0	0	0	0
0													
OTU257	0	0	25	0	0	69	39	0	0	10	0	0	0
0	23	0	1	0	0	5	0	0	0	0	0	12	0
0	1	0	0	4	0	0	0	0	0	4	0	0	0

3	0	1	0	0	0	0	0	0	0	0	0	0	0
0	0	0	0	0	0	0	0	0	0	0	0	2	0
0	0	0	0	0	0	0	1	0	2	0	3	0	25
0	3	0	0	0	0	0	0	0	0	0	0	0	0
0	0	0	1	0	0	0	0	0	0	0	0	0	0
4													
OTU14593	0	0	1	0	0	0	0	0	0	0	0	0	0
0	0	0	0	0	0	0	0	0	0	0	0	0	0
0	0	0	0	0	0	0	0	0	0	0	0	0	0
0	0	0	0	0	0	0	0	0	0	0	0	0	0
0	0	0	0	0	0	0	0	0	0	0	0	0	0
0	0	0	0	0	0	0	0	0	0	0	0	0	0
0	0	0	0	0	0	0	0	0	0	0	0	0	0
0	0	0	0	0	0	0	0	0	0	0	0	0	0
0													
OTU1067	0	0	4	0	0	0	1	2	6	2	0	0	0
0	0	0	0	0	0	2	0	0	0	0	0	0	0
1	0	0	0	0	0	0	0	0	0	0	0	0	0
11	0	0	0	0	0	0	0	0	0	0	2	0	0
0	1	0	0	0	0	0	0	0	0	0	0	0	0
0	0	0	0	1	0	0	0	0	6	0	6	0	2
0	0	0	0	0	9	0	0	0	0	0	0	0	0
0	0	0	0	0	0	0	0	0	0	0	0	0	0
0													
OTU9642	0	0	8	0	0	0	0	2	0	0	0	12	0
1	0	2	0	0	0	0	0	0	0	0	0	0	0
0	0	1	0	1	0	1	1	0	6	0	0	0	0
0	6	0	5	1	0	0	0	0	0	0	0	0	0
0	0	0	0	0	0	1	0	0	1	0	0	0	0
0	0	12	5	0	0	0	0	0	3	1	1	1	0
0	1	0	0	0	0	3	0	0	17	0	0	0	0
3	0	0	0	0	0	0	0	0	0	3	1	2	0
0													

OTU389	0	0	2	3	5	2	4	4	2	2	1	1	1
0	4	0	0	4	0	0	0	0	0	1	0	0	0
0	0	0	1	0	0	0	2	2	1	1	0	0	3
1	0	0	1	0	1	0	2	0	1	0	0	0	2
0	3	0	2	0	0	1	0	0	0	0	0	0	0
0	1	0	2	0	2	1	0	2	2	3	1	2	2
0	0	0	2	0	1	1	4	0	6	1	0	3	0
0	1	1	1	0	0	1	3	0	0	0	3	0	0
0													
OTU10831	0	0	3	0	0	0	0	0	0	0	0	0	0
0	0	0	0	0	0	0	0	0	0	0	0	0	0
0	0	0	0	0	0	0	0	0	0	0	0	0	0
0	0	0	0	0	0	0	0	0	0	0	0	0	0
0	0	0	0	0	0	0	0	0	0	0	0	0	0
0	0	0	0	0	0	0	0	0	0	0	0	0	0
0	0	0	0	0	0	0	0	0	0	0	0	0	0
0	0	0	0	0	0	0	0	0	0	0	0	0	0
0													
OTU5973	0	0	2	0	1	1	1	0	0	0	0	1	0
0	3	1	1	1	2	0	1	1	2	0	0	0	0
0	1	2	0	0	0	0	0	0	0	0	0	0	0
0	1	1	0	0	2	0	0	1	0	0	0	1	0
1	0	1	1	0	1	1	0	0	1	0	0	0	0
3	1	0	1	0	0	0	2	0	1	0	4	0	0
1	0	0	0	1	5	0	0	0	0	0	0	1	0
1	1	0	0	0	0	0	1	0	0	1	0	0	1
3													
OTU15982	0	0	1	1	3	2	0	0	0	3	1	3	0
0	0	1	1	1	3	0	1	1	3	0	0	0	1
1	1	0	2	0	1	0	3	0	0	0	2	3	2
2	2	0	1	1	2	0	0	1	3	0	0	0	1
0	2	1	0	0	0	1	1	0	0	1	3	3	1
2	1	0	0	2	1	0	2	0	1	0	3	0	1

1	2	1	0	1	3	0	2	2	0	1	0	0	0
0	2	0	0	1	0	0	1	2	1	1	0	0	0
3													
OTU12292	0	0	1	0	0	0	0	0	0	0	0	0	0
0	0	0	0	0	0	0	0	0	0	0	0	0	0
0	0	0	0	0	0	0	0	0	0	0	0	0	0
0	0	0	0	0	0	0	0	0	0	0	0	0	0
0	0	0	0	0	0	0	0	0	0	0	0	0	0
0	0	0	0	0	0	0	0	0	0	0	0	0	0
0	0	0	0	0	0	0	0	0	0	0	0	0	0
0	0	0	0	0	0	0	0	0	0	0	0	0	0
0													
OTU2319	0	0	1	0	0	0	0	0	0	0	0	1	4
1	0	0	0	0	0	0	0	0	0	0	0	0	0
0	0	0	1	0	1	0	4	0	0	0	0	0	1
0	0	0	0	3	0	3	0	0	0	2	0	0	0
0	0	1	0	0	0	0	0	0	0	0	8	0	0
0	0	0	1	0	0	0	0	0	0	0	0	1	0
0	0	0	0	0	0	0	0	0	0	1	0	0	0
1	0	0	0	0	0	0	0	0	0	0	0	0	0
0													
OTU6021	0	0	1	0	1	1	0	0	0	0	0	1	0
0	0	0	0	0	0	0	0	0	0	0	1	0	0
0	0	0	0	0	0	0	0	0	0	0	0	0	1
0	1	0	0	0	0	0	0	0	0	0	0	0	0
0	0	0	0	0	0	0	0	0	0	0	0	0	0
0	0	1	0	0	0	0	0	1	0	0	1	0	0
0	0	0	0	0	0	0	0	0	0	0	0	0	0
0	0	0	0	0	0	0	0	0	0	0	0	0	0
0													
OTU10822	0	0	1	0	0	0	0	0	0	0	0	0	0
0	0	0	0	0	0	0	0	0	0	0	0	0	0
0	0	0	0	0	0	0	0	0	0	0	0	0	0

0	0	0	0	0	0	0	0	0	0	0	0	0	0
0	0	0	0	0	0	0	0	0	0	0	0	0	0
0	0	0	0	0	0	0	0	0	0	0	0	0	0
0	0	0	0	0	0	0	0	0	0	0	0	0	0
0	0	0	0	0	0	0	0	0	0	0	0	0	0
0													
OTU8584	0	0	2	1	0	0	1	0	0	0	0	0	0
0	0	0	0	0	0	0	0	0	0	1	0	0	0
0	0	0	0	0	0	0	0	0	0	0	0	0	0
0	0	0	0	0	0	0	0	0	0	0	0	0	0
0	0	0	0	0	0	0	0	0	0	0	2	0	0
0	0	0	0	0	0	0	0	0	0	0	0	0	0
0	0	0	0	3	0	0	0	0	0	0	0	0	0
0	0	0	0	0	0	0	0	0	0	0	0	0	0
0													
OTU12293	0	0	1	0	0	0	0	0	0	0	0	0	0
0	0	0	0	0	0	0	0	0	0	0	0	0	0
0	0	0	0	0	0	0	0	0	0	0	0	0	0
0	0	0	0	0	0	0	0	0	0	0	0	0	0
0	0	0	0	0	0	0	0	0	0	0	0	0	0
0	0	0	0	0	0	0	0	0	0	0	0	0	0
0	0	0	0	0	0	0	0	0	0	0	0	0	0
0	0	0	0	0	0	0	0	0	0	0	0	0	0
0													
OTU229	0	0	2	1	0	0	1	4	3	3	0	1	0
0	1	0	0	0	0	0	0	0	0	0	0	0	0
0	0	0	0	0	0	0	0	0	0	0	0	0	0
3	0	0	0	0	0	0	15	0	0	0	1	0	6
0	2	0	0	0	0	0	0	1	0	0	0	2	0
1	72	0	0	2	0	0	1	0	1	3	2	0	1
0	0	0	1	0	0	0	0	0	1	0	21	31	0
0	116	95	241	285	148	253	57	62	190	0	7	0	532
337													

OTU7707	0	0	1	0	0	0	0	0	0	0	0	1	0
0	0	0	0	0	0	0	0	0	0	0	0	0	0
0	0	0	0	0	0	0	0	0	0	0	0	0	0
0	0	0	0	1	0	0	0	0	0	1	0	0	0
0	0	0	0	0	0	0	0	0	0	0	0	0	0
0	0	0	0	0	0	0	0	0	0	0	0	0	0
0	0	0	0	0	0	0	0	0	0	0	0	0	0
0	0	0	0	0	0	0	0	0	0	0	0	0	0
0													
OTU12291	0	0	1	0	0	0	0	0	0	0	0	0	0
0	0	0	0	0	0	0	0	0	0	0	0	0	0
0	0	0	0	0	0	0	0	0	0	0	0	0	0
0	0	0	0	0	0	0	0	0	0	0	0	0	0
0	0	0	0	0	0	0	0	0	0	0	0	0	0
0	0	0	0	0	0	0	0	0	0	0	0	0	0
0	0	0	0	0	0	0	0	0	0	0	0	0	0
0	0	0	0	0	0	0	0	0	0	0	0	0	0
0													
OTU8583	0	0	1	0	0	0	0	0	0	0	0	0	0
0	0	0	0	0	0	0	0	0	0	0	0	0	0
0	0	0	0	0	0	0	0	0	0	0	0	0	0
0	0	0	0	0	0	0	0	0	0	0	0	0	0
0	0	0	0	0	0	0	0	0	0	0	0	0	0
0	0	0	0	0	0	0	0	0	0	0	0	0	0
0	0	0	0	0	0	0	0	0	0	0	0	0	0
0	0	0	0	0	0	0	0	0	0	0	0	0	0
0													
OTU10821	0	0	1	0	0	0	0	0	0	0	0	0	0
0	0	0	0	0	0	0	0	0	0	0	0	0	0
0	0	0	0	0	0	0	0	0	0	0	0	0	0
0	0	0	0	0	0	0	0	0	0	0	0	0	0
0	0	0	0	0	0	0	0	0	0	0	0	0	0
0	0	0	0	0	0	0	0	0	0	0	0	0	0

0	0	0	0	0	0	0	0	0	0	0	0	0	0
0	0	0	0	0	0	0	0	0	0	0	0	0	0
0													
OTU8581	0	0	1	0	0	0	0	0	0	0	0	0	0
0	0	0	0	0	0	0	0	0	0	0	0	0	0
0	0	0	0	0	0	0	0	0	0	0	0	0	0
0	0	0	0	0	0	0	0	0	0	0	0	0	0
0	0	0	0	0	0	0	0	0	0	0	0	0	0
0	0	0	0	0	0	0	0	0	0	0	0	0	0
0	0	0	0	0	0	0	0	0	0	0	0	0	0
0	0	0	0	0	0	0	0	0	0	0	0	0	0
0													
OTU4468	0	0	1	1	0	0	0	0	1	0	0	0	0
0	0	1	0	0	0	4	0	0	0	0	0	0	0
0	0	0	0	0	0	0	0	0	0	0	0	0	0
0	0	0	0	0	0	0	0	0	0	0	0	0	0
0	0	0	1	0	0	0	0	0	0	0	0	1	0
0	0	0	0	2	0	0	0	0	1	0	1	0	0
0	0	1	0	0	0	0	1	0	0	0	0	0	0
0	1	0	0	0	0	0	0	0	0	0	0	0	0
0													
OTU8580	0	0	1	0	0	0	0	0	0	0	0	0	0
0	0	0	0	0	0	0	0	0	0	0	0	0	0
0	0	0	0	0	0	0	0	0	0	0	0	0	0
0	0	0	0	0	0	0	0	0	0	0	0	0	0
0	0	0	0	0	0	0	0	0	0	0	0	0	0
0	0	0	0	0	0	0	0	0	0	0	0	0	0
0	0	0	0	0	0	0	0	0	0	0	0	0	0
0	0	0	0	0	0	0	0	0	0	0	0	0	0
0													
OTU8582	0	0	1	0	0	0	0	0	0	0	0	0	0
0	0	0	0	0	0	0	0	0	0	0	0	0	0
0	0	0	0	0	0	0	0	0	0	0	0	0	0

0	0	0	0	0	0	0	0	0	0	0	0	0	0
0	0	0	0	0	0	0	0	0	0	0	0	0	0
0	0	0	0	0	0	0	0	0	0	0	0	0	0
0	0	0	0	0	0	0	0	0	0	0	0	0	0
0	0	0	0	0	0	0	0	0	0	0	0	0	0
0													
OTU963	0	0	1	0	0	0	0	0	0	0	0	0	0
0	0	0	0	0	0	0	0	0	0	0	0	0	0
0	0	0	0	0	0	0	0	0	0	0	0	0	0
0	0	0	0	0	0	1	0	0	0	0	0	0	0
0	1	0	0	0	0	0	0	0	0	0	0	3	0
0	0	1	0	0	0	0	0	0	1	0	1	0	0
1	1	0	2	0	0	0	0	0	0	0	0	0	0
0	0	0	0	0	0	0	0	0	0	0	0	0	0
0													
OTU10820	0	0	1	0	0	0	0	0	0	0	0	0	0
0	0	0	0	0	0	0	0	0	0	0	0	0	0
0	0	0	0	0	0	0	0	0	0	0	0	0	0
0	0	0	0	0	0	0	0	0	0	0	0	0	0
0	0	0	0	0	0	0	0	0	0	0	0	0	0
0	0	0	0	0	0	0	0	0	0	0	0	0	0
0	0	0	0	0	0	0	0	0	0	0	0	0	0
0	0	0	0	0	0	0	0	0	0	0	0	0	0
0													
OTU13668	0	0	1	1	1	0	0	0	1	0	0	0	0
0	0	0	0	0	0	0	1	0	0	0	0	0	0
0	0	0	0	0	0	0	0	0	0	0	0	0	0
0	0	0	0	0	0	0	0	0	0	0	0	0	0
0	0	0	0	0	0	0	0	0	0	0	0	0	1
0	0	1	0	0	0	0	0	0	0	0	0	0	0
0	1	0	0	0	0	1	0	0	0	0	0	0	0
0	0	0	0	0	0	0	0	0	0	0	0	0	0
0													

OTU10834	0	0	2	0	0	0	0	0	0	0	0	0	0
0	0	0	0	0	0	0	0	0	0	0	0	0	0
0	0	0	0	0	0	0	0	0	0	0	0	0	0
0	0	0	0	0	0	0	0	0	0	0	0	0	0
0	0	0	0	0	0	0	0	0	0	0	0	0	0
0	0	0	0	0	0	0	0	0	0	0	0	0	0
0	0	0	0	0	0	0	0	0	0	0	0	0	0
0	0	0	0	0	0	0	0	0	0	0	0	0	0
0													
OTU863	0	0	1	0	0	0	0	0	1	1	0	0	0
0	0	0	0	0	0	1	0	0	0	0	0	0	0
0	0	0	0	0	1	0	0	0	0	0	0	0	0
0	1	1	0	0	0	0	0	0	0	0	0	0	1
0	0	0	0	0	0	0	0	0	0	0	0	1	0
0	0	0	0	0	0	0	0	0	0	0	0	0	0
0	0	0	0	0	0	0	0	0	0	0	0	0	0
0	1	0	1	0	0	0	0	0	0	0	0	0	0
1													
OTU8585	0	0	1	0	0	0	0	0	0	0	0	0	0
0	0	0	0	0	0	0	0	0	0	0	0	0	0
0	0	0	0	0	0	0	0	0	0	0	0	0	0
0	0	0	0	0	0	0	0	0	0	0	0	0	0
0	0	0	0	0	0	0	0	0	0	0	0	0	0
0	0	0	0	0	0	0	0	0	0	0	0	0	0
0	0	0	0	0	0	0	0	0	0	0	0	0	0
0	0	0	0	0	0	0	0	0	0	0	0	0	0
0													
OTU475	0	0	1	0	2	0	0	0	2	1	0	0	0
0	0	0	0	0	0	0	0	0	0	0	0	0	0
0	0	0	0	0	0	0	0	1	0	0	0	0	0
0	1	0	0	0	1	0	1	0	0	0	0	0	0
0	0	0	0	0	0	0	0	0	0	0	0	0	0
0	8	0	0	0	0	0	0	0	2	0	0	0	0

0	0	0	0	0	0	0	1	0	0	0	8	3	0
0	5	2	0	1	2	0	3	2	3	0	0	0	1
6													
OTU14590	0	0	1	0	0	0	0	0	0	0	0	0	0
0	0	0	0	0	0	0	0	0	0	0	0	0	0
0	0	0	0	0	0	0	0	0	0	0	0	0	0
0	0	0	0	0	0	0	0	0	0	0	0	0	0
0	0	0	0	0	0	0	0	0	0	0	0	0	0
0	0	0	0	0	0	0	0	0	0	0	0	0	0
0	0	0	0	0	0	0	0	0	0	0	0	0	0
0	0	0	0	0	0	0	0	0	0	0	0	0	0
0													
OTU303	0	0	1	0	0	0	0	0	0	0	0	0	0
0	0	0	0	0	0	7	1	0	0	0	0	0	0
0	0	0	0	0	0	0	0	0	6	0	0	2	1
1	7	0	0	0	54	0	0	0	0	0	0	0	8
0	0	0	0	0	0	0	0	0	0	0	0	8	0
0	1	0	0	12	0	0	0	0	1	0	1	0	28
1	1	0	60	0	2	0	0	0	0	0	0	13	0
0	1	0	0	0	0	0	0	0	0	0	0	0	0
0													
OTU14618	0	0	1	0	0	0	0	0	0	0	0	0	0
0	0	0	0	0	0	0	0	0	0	0	0	0	0
0	0	0	0	0	0	0	0	0	0	0	0	0	0
0	0	0	0	0	0	0	0	0	0	0	0	0	0
0	0	0	0	0	0	0	0	0	0	0	0	0	0
0	0	0	0	0	0	0	0	0	0	0	0	0	0
0	0	0	0	0	0	0	0	0	0	0	0	0	0
0	0	0	0	0	0	0	0	0	0	0	0	0	0
0													
OTU12302	0	0	1	0	0	0	0	0	0	0	0	0	0
0	0	0	0	0	0	0	0	0	0	0	0	0	0
0	0	0	0	0	0	0	0	0	0	0	0	0	0

0	0	0	0	0	0	0	0	0	0	0	0	0	0
0	0	0	0	0	0	0	0	0	0	0	0	0	0
0	0	0	0	0	0	0	0	0	0	0	0	0	0
0	0	0	0	0	0	0	0	0	0	0	0	0	0
0	0	0	0	0	0	0	0	0	0	0	0	0	0
0													
OTU9536	0	0	1	0	2	0	0	0	1	0	0	0	0
0	0	0	0	0	0	0	0	0	0	0	0	10	0
0	0	0	0	0	0	1	0	0	0	0	0	1	0
0	0	0	0	0	0	0	0	0	0	0	1	0	0
0	0	0	0	0	0	0	0	0	0	0	0	0	0
0	0	0	0	0	0	0	0	1	0	0	0	0	0
0	0	0	1	0	0	0	0	0	0	0	0	0	0
0	0	0	0	0	0	0	0	0	0	0	0	0	0
0													
OTU328	0	0	4	0	0	0	1	1	4	3	0	0	1
0	0	0	0	0	0	0	0	0	0	1	0	2	0
94	1	0	0	0	0	0	0	0	0	0	0	0	0
1	0	0	0	0	0	0	1	0	0	0	21	0	1
0	1	0	0	0	0	0	1	0	0	0	0	8	0
0	4	0	0	2	0	0	1	0	18	0	6	0	3
0	2	0	2	0	0	0	0	0	0	0	1	1	0
0	7	10	11	9	8	2	2	2	5	0	0	0	10
6													
OTU12305	0	0	1	0	0	0	0	0	0	0	0	0	0
0	0	0	0	0	0	0	0	0	0	0	0	0	0
0	0	0	0	0	0	0	0	0	0	0	0	0	0
0	0	0	0	0	0	0	0	0	0	0	0	0	0
0	0	0	0	0	0	0	0	0	0	0	0	0	0
0	0	0	0	0	0	0	0	0	0	0	0	0	0
0	0	0	0	0	0	0	0	0	0	0	0	0	0
0	0	0	0	0	0	0	0	0	0	0	0	0	0
0													

OTU10832	0	0	1	0	0	0	0	0	0	0	0	0	0
0	0	0	0	0	0	0	0	0	0	0	0	0	0
0	0	0	0	0	0	0	0	0	0	0	0	0	0
0	0	0	0	0	0	0	0	0	0	0	0	0	0
0	0	0	0	0	0	0	0	0	0	0	0	0	0
0	0	0	0	0	0	0	0	0	0	0	0	0	0
0	0	0	0	0	0	0	0	0	0	0	0	0	0
0	0	0	0	0	0	0	0	0	0	0	0	0	0
0													
OTU5312	0	0	1	0	0	1	0	2	0	0	0	1	1
0	0	0	0	0	1	0	1	0	0	0	0	0	0
0	0	0	0	0	0	0	0	0	1	0	0	0	0
0	0	0	2	0	3	0	0	0	0	0	0	0	0
0	0	0	2	0	0	0	0	0	0	0	0	0	0
0	0	5	0	2	0	1	0	0	0	1	0	1	1
0	1	0	0	0	0	0	0	0	0	0	0	0	0
0	0	0	2	0	0	0	0	0	0	0	0	0	0
0													
OTU753	0	0	2	0	0	0	0	1	0	0	0	0	0
0	0	0	0	0	0	0	0	0	0	0	0	0	0
0	0	0	0	0	0	0	0	0	0	0	0	0	0
0	0	0	2	0	0	0	0	0	0	0	0	0	0
0	0	0	0	0	0	0	0	0	0	0	0	0	0
0	0	0	0	0	12	3	0	0	0	0	1	0	0
0	0	0	0	0	0	0	0	0	0	0	0	0	0
0	0	0	0	0	0	0	0	0	0	0	0	0	0
0													
OTU12304	0	0	1	0	0	0	0	0	0	0	0	0	0
0	0	0	0	0	0	0	0	0	0	0	0	0	0
0	0	0	0	0	0	0	0	0	0	0	0	0	0
0	0	0	0	0	0	0	0	0	0	0	0	0	0
0	0	0	0	0	0	0	0	0	0	0	0	0	0
0	0	1	0	0	0	0	0	0	0	0	0	0	0

0	0	0	0	0	0	0	0	0	0	0	0	0	0
0	0	0	0	1	0	0	0	0	2	0	0	0	2
0													
OTU15810	0	0	1	0	2	1	3	3	0	0	0	0	1
1	2	0	0	1	0	0	1	0	0	0	0	0	1
0	0	3	1	0	0	0	1	0	0	0	4	1	1
0	2	1	1	0	0	0	0	2	0	0	0	0	1
0	2	0	2	0	1	0	1	1	1	2	0	1	0
0	0	2	0	3	1	0	1	2	1	0	2	1	2
1	0	0	0	0	0	0	2	0	1	1	0	0	0
1	1	0	0	1	0	0	2	0	0	0	0	2	0
3													
OTU761	0	0	1	0	0	0	0	0	0	0	0	1	0
0	0	0	0	1	0	0	1	0	0	0	0	0	0
1	0	4	0	0	0	0	0	0	0	0	0	0	0
1	0	0	0	0	0	0	0	1	0	0	0	0	0
0	0	0	1	2	0	0	0	0	0	0	0	0	0
0	0	0	0	0	0	0	0	1	0	0	0	0	0
0	1	0	0	0	0	0	0	0	0	0	1	0	0
0	0	0	0	0	0	0	0	1	0	0	0	0	0
0													
OTU14160	0	0	2	0	0	2	1	1	1	0	1	0	0
1	0	5	1	1	0	2	17	0	4	7	5	2	6
6	0	0	0	1	0	0	0	0	2	0	0	0	0
4	2	0	0	6	1	0	0	0	0	5	0	0	0
0	0	1	1	5	0	0	1	1	1	0	5	0	2
0	2	3	10	0	9	1	0	5	0	0	5	0	0
1	1	0	0	0	0	5	0	0	1	0	0	0	0
0	0	0	7	0	2	0	0	2	0	3	0	2	2
0													
OTU12303	0	0	1	0	0	0	0	0	0	0	0	0	0
0	0	0	0	0	0	0	0	0	0	0	0	0	0
0	0	0	0	0	0	0	0	0	0	0	0	0	0

0 0 0 0 0 0 0 0 0 0 0 0 0 0
0 0 0 0 0 0 0 0 0 0 0 0 0 0
0 0 0 0 0 0 0 0 0 0 0 0 0 0
0 0 0 0 0 0 0 0 0 0 0 0 0 0
0 0 0 0 0 0 0 0 0 0 0 0 0 0
0
OTU641 0 0 1 1 0 1 0 0 3 0 0 0 0
0 0 0 0 0 0 0 0 0 0 0 0 0 0
0 0 0 0 0 0 0 0 0 1 0 0 0 0
0 0 1 0 0 0 0 0 0 0 0 1 0 0
0 1 0 1 0 0 0 0 0 0 0 0 1 0
1 0 4 0 9 0 0 0 0 1 1 0 0 0
0 0 0 2 0 0 0 4 0 0 0 0 0 0
0 0 2 0 0 0 0 0 0 1 0 0 0 0
0
OTU9343 0 0 1 0 0 0 0 0 0 0 0 0 0
0 0 1 0 0 0 0 3 0 0 0 0 0 0
0 0 0 0 0 0 0 0 0 0 0 0 0 0
0 0 0 0 0 0 0 0 0 0 0 0 0 0
0 2 0 0 0 0 0 0 0 0 0 0 0 0
0 0 0 0 0 0 0 0 0 0 0 0 0 0
0 0 0 0 0 0 0 0 0 0 0 0 1 0
0 0 0 0 0 0 0 0 0 0 0 0 1 0
0
OTU617 0 0 1 0 0 0 1 2 0 2 0 1 0
0 1 0 0 0 0 0 0 0 0 0 0 0 2
0 0 0 0 1 0 0 0 3 0 0 0 0 0
0 0 0 0 0 1 0 1 0 0 1 0 0 0
0 0 0 2 0 0 0 0 0 0 1 0 1 0
0 0 1 0 0 0 0 0 0 0 0 0 0 0
0 0 0 2 2 0 0 0 1 0 0 2 1 0
0 0 0 0 0 0 0 0 0 0 0 0 0 0
0

OTU12953 0 0 2 4 6 0 2 0 0 0 1 1 0
0 0 3 30 0 1 1 7 40 0 2 0 0 2
7 2 0 1 6 0 3 23 1 7 2 0 0 0
1 1 2 1 38 12 2 0 30 1 7 0 6 0
48 1 30 8 7 26 12 8 17 103 15 19 8 7
30 0 0 0 0 0 0 0 0 2 0 0 0 0
1 7 0 0 0 2 8 0 0 5 12 0 0 4
3 0 0 0 0 0 8 0 3 0 2 12 4 0
0
OTU8590 0 0 1 0 0 0 0 0 0 0 0 0 0
0 0 0 0 0 0 0 0 0 0 0 0 0 0
0 0 0 0 0 0 0 0 0 0 0 0 0 0
0 0 0 0 0 0 0 0 0 0 0 0 0 0
0 0 0 0 0 0 0 0 0 0 0 0 0 0
0 0 0 0 0 0 0 0 0 0 0 0 0 0
0 0 0 0 0 0 0 0 0 0 0 0 0 0
0 0 0 0 0 0 0 0 0 0 0 0 0 0
0
OTU12300 0 0 1 0 0 0 0 0 0 0 0 0 0
0 0 0 0 0 0 0 0 0 0 0 0 0 0
0 0 0 0 0 0 0 0 0 0 0 0 0 0
0 0 0 0 0 0 0 0 0 0 0 0 0 0
0 0 0 0 0 0 0 0 0 0 0 0 0 0
0 0 0 0 0 0 0 0 0 0 0 0 0 0
0 0 0 0 0 0 0 0 0 0 0 0 0 0
0 0 0 0 0 0 0 0 0 0 0 0 0 0
0
OTU274 0 0 2 0 0 0 1 2 8 1 0 2 0
0 0 1 1 0 0 0 0 0 0 0 0 1 0
12 0 0 0 1 0 0 0 0 0 0 0 0 0
14 0 0 0 0 0 0 0 1 0 0 0 2 0
0 0 0 0 0 0 0 0 0 1 0 0 2 0
1 8 1 0 2 0 0 0 2 10 0 6 0 12

0	1	0	0	0	1	0	0	1	1	3	7	10	1
0	25	4	8	10	4	0	10	0	0	2	0	0	1
0													
OTU648	0	0	3	0	0	0	0	0	0	0	0	0	0
0	0	0	0	0	0	0	0	0	0	0	0	0	0
0	0	0	0	0	0	0	0	0	0	0	0	0	0
0	1	0	2	0	0	0	0	0	0	0	0	0	0
0	0	0	0	0	0	0	0	0	0	0	0	0	0
1	0	0	0	4	0	0	0	0	3	0	2	0	3
0	2	0	1	0	0	0	0	0	0	0	0	0	0
0	0	0	0	0	0	0	0	0	0	0	0	0	0
0													
OTU14619	0	0	1	0	0	0	0	0	0	0	0	0	0
0	0	0	0	0	0	0	0	0	0	0	0	0	0
0	0	0	0	0	0	0	0	0	0	0	0	0	0
0	0	0	0	0	0	0	0	0	0	0	0	0	0
0	0	0	0	0	0	0	0	0	0	0	0	0	0
0	0	0	0	0	0	0	0	0	0	0	0	0	0
0	0	0	0	0	0	0	0	0	0	0	0	0	0
0	0	0	0	0	0	0	0	0	0	0	0	0	0
0													
OTU10827	0	0	1	0	0	0	0	0	0	0	0	0	0
0	0	0	0	0	0	0	0	0	0	0	0	0	0
0	0	0	0	0	0	0	0	0	0	0	0	0	0
0	0	0	0	0	0	0	0	0	0	0	0	0	0
0	0	0	0	0	0	0	0	0	0	0	0	0	0
0	0	0	0	0	0	0	0	0	0	0	0	0	0
0	0	0	0	0	0	0	0	0	0	0	0	0	0
0	0	0	0	0	0	0	0	0	0	0	0	0	0
0													
OTU181	0	0	7	19	5	1	93	5	60	24	0	1	0
0	15	0	9	0	0	22	0	0	0	0	0	1	0
2	0	0	0	0	0	0	0	3	1	1	0	21	0

11	0	1	1	0	0	0	1	0	0	0	1	0	0
0	0	0	1	0	0	0	0	0	0	0	0	1	0
1	7	0	0	0	0	0	2	0	25	0	0	0	27
0	2	0	42	1	44	0	43	0	0	0	3	6	0
0	9	17	22	11	40	6	4	0	14	2	0	1	8
11													
OTU7784	0	0	2	0	0	2	1	1	4	1	0	0	0
0	0	0	0	0	0	1	0	0	0	0	0	0	0
1	0	0	0	0	0	0	0	0	0	1	0	2	0
2	1	0	1	0	0	0	0	0	0	0	0	0	0
0	2	0	0	0	0	0	0	0	0	0	0	0	0
0	1	0	0	1	0	0	0	0	0	0	1	0	0
0	0	0	6	0	0	0	4	0	0	0	9	4	0
0	6	5	6	1	1	18	6	4	6	0	0	0	5
5													
OTU11305	0	0	1	0	0	0	0	0	0	0	0	0	0
0	0	0	0	0	0	0	0	0	0	0	0	0	0
0	0	0	0	0	0	0	0	0	0	0	0	0	0
0	0	0	0	0	0	0	0	0	0	0	0	0	0
0	0	0	0	0	0	0	0	0	0	0	0	0	0
0	0	0	0	0	0	0	0	0	0	0	0	0	0
0	0	0	0	0	0	0	0	0	0	0	0	0	0
0	0	0	0	0	2	0	0	0	0	0	0	0	0
0													
OTU5038	0	0	4	0	0	0	2	4	0	3	0	0	0
0	2	0	0	0	0	1	0	0	0	0	0	0	0
1	0	0	0	0	0	0	0	0	0	0	0	0	0
1	0	1	0	0	0	0	0	0	0	0	0	0	0
0	7	0	0	0	0	0	0	0	0	0	0	0	0
0	0	0	0	1	0	0	0	0	2	0	0	0	0
0	1	0	6	0	0	0	0	0	0	0	0	0	0
0	0	0	0	0	0	0	0	0	0	0	0	0	0
0													

OTU8591	0	0	1	0	0	0	0	0	0	0	0	0	0
0	0	0	0	0	0	0	0	0	0	0	0	0	0
0	0	0	0	0	0	0	0	0	0	0	0	0	0
0	0	0	0	0	0	0	0	0	0	0	0	0	0
0	0	0	0	0	0	0	0	0	0	0	0	0	0
0	0	0	0	0	0	0	0	0	0	0	0	0	0
0	0	0	0	0	0	0	0	0	0	0	0	0	0
0	0	0	0	0	0	0	0	0	0	0	0	0	0
0													
OTU12301	0	0	1	0	1	0	0	0	0	0	0	0	0
0	0	0	0	0	0	0	0	0	0	0	0	0	0
0	0	0	0	0	0	0	0	0	0	0	0	0	0
0	0	0	0	0	0	0	0	0	0	0	0	0	0
0	0	0	0	0	0	0	0	0	0	1	0	0	0
0	0	0	0	0	0	0	0	0	0	0	0	0	0
0	1	0	0	0	0	0	0	0	0	0	0	0	0
0	0	0	0	0	0	0	0	0	0	0	0	0	0
0													
OTU7771	0	0	2	0	0	0	0	2	4	2	0	0	0
0	0	0	0	0	0	1	0	0	0	0	0	0	0
0	0	0	0	0	0	0	0	0	0	0	0	1	0
6	0	0	0	0	0	0	0	0	0	0	1	0	0
0	0	0	0	0	0	0	0	0	0	0	0	0	0
0	0	0	0	0	0	0	0	0	1	0	0	0	1
0	1	0	3	0	3	0	1	0	0	0	0	0	0
0	0	0	0	0	0	0	0	0	0	0	0	0	0
0													
OTU298	0	0	3	0	0	1	0	7	1	1	0	6	3
12	2	0	0	0	1	1	0	1	0	0	0	0	0
0	0	0	13	1	0	0	7	0	2	0	0	0	2
13	0	0	3	0	0	0	4	0	0	5	1	0	0
0	3	1	4	1	0	1	0	0	1	0	0	0	0
0	0	11	6	6	1	0	4	3	3	0	1	13	3

2	0	0	1	0	2	0	1	0	10	1	1	0	0
5	1	0	1	1	3	1	1	2	5	2	1	6	9
1													
OTU14617	0	0	1	0	0	0	0	0	0	0	0	0	0
0	0	0	0	0	0	0	0	0	0	0	0	0	0
0	0	0	0	0	0	0	0	0	0	0	0	0	0
0	0	0	0	0	0	0	0	0	0	0	0	0	0
0	0	0	0	0	0	0	0	0	0	0	0	0	0
0	0	0	0	0	0	0	0	0	0	0	0	0	0
0	0	0	0	0	0	0	0	0	0	0	0	0	0
0	0	0	0	0	0	0	0	0	0	0	0	0	0
0													
OTU10826	0	0	1	0	1	0	0	0	0	0	0	0	0
0	0	0	0	0	0	0	0	0	0	0	0	0	0
0	0	0	0	0	0	0	0	1	0	0	0	0	0
0	0	0	0	0	0	0	0	0	0	0	0	0	0
0	0	0	0	0	0	0	0	0	0	0	0	0	0
0	0	0	0	0	0	0	0	0	0	0	0	0	0
0	0	0	0	0	0	0	0	0	0	0	0	0	0
0	0	0	0	0	0	0	0	0	0	0	0	0	0
0													
OTU6720	0	0	1	0	0	1	0	0	0	0	0	0	0
0	0	1	0	0	0	0	0	0	0	0	0	0	0
0	0	0	0	0	0	0	0	0	2	0	0	0	0
0	1	0	0	0	0	0	0	0	0	1	0	0	0
0	0	0	1	0	0	0	0	0	0	0	5	0	0
0	0	0	0	0	0	0	0	1	0	0	0	1	0
0	0	1	0	2	0	1	0	0	3	0	0	0	0
0	0	0	0	0	0	0	0	0	0	1	1	0	0
0													
OTU10855	0	0	1	0	1	0	0	0	0	0	0	0	0
0	0	0	0	0	0	0	0	0	0	0	0	0	0
0	0	0	0	0	0	0	0	0	0	0	0	0	0

0	0	0	0	0	0	0	0	0	0	0	0	0	0
0	0	0	0	0	0	0	0	0	0	0	0	0	0
0	0	0	0	0	0	0	0	0	0	0	0	0	0
0	0	0	0	0	0	0	0	0	0	0	0	0	0
0	0	0	0	0	0	0	0	0	0	0	0	0	0
0													
OTU13347	0	0	1	0	0	0	0	0	0	0	0	0	0
0	0	0	0	0	0	0	0	0	0	0	0	0	0
0	0	0	0	0	0	0	0	0	0	0	0	0	4
0	0	0	0	0	0	0	0	0	0	0	0	0	0
0	0	0	0	0	0	0	0	0	0	0	0	0	0
0	0	0	0	0	0	0	0	0	0	0	0	0	0
0	0	0	0	0	0	0	0	0	0	0	0	0	0
0	0	0	0	0	1	0	0	0	0	0	0	0	0
0													
OTU202	0	0	3	0	0	0	16	0	0	22	0	0	0
0	6	0	0	0	0	0	0	0	0	0	0	0	0
28	0	0	0	0	0	0	0	0	0	0	0	0	0
0	0	0	0	0	0	0	8	0	0	0	32	2	0
0	0	0	0	0	0	1	0	2	0	0	0	22	0
1	0	0	0	5	0	0	5	0	2	0	20	0	24
0	2	0	130	0	108	0	0	1	0	0	2	0	0
0	14	0	3	5	1	3	1	0	13	0	0	0	2
2													
OTU10829	0	0	1	0	0	0	0	0	0	0	0	0	0
0	0	0	0	0	0	0	0	0	0	0	0	0	0
0	0	0	0	0	0	0	0	0	0	0	0	0	0
0	0	0	0	0	0	0	0	0	0	0	0	0	0
0	0	0	0	0	0	0	0	0	0	0	0	0	0
0	0	0	0	0	0	0	0	0	0	0	0	0	0
0	0	0	0	0	0	0	0	0	0	0	0	0	0
0	0	0	0	0	0	0	0	0	0	0	0	0	0
0													

OTU10828	0	0	1	0	0	0	0	0	0	0	0	0	0
0	0	0	0	0	0	0	0	0	0	0	0	0	0
0	0	0	0	0	0	0	0	0	0	0	0	0	0
0	0	0	0	0	0	0	0	0	0	0	0	0	0
0	0	0	0	0	0	0	0	0	0	0	0	0	0
0	0	0	0	0	0	0	0	0	0	0	0	0	0
0	0	0	0	0	0	0	0	0	0	0	0	0	0
0	0	0	0	0	0	0	0	0	0	0	0	0	0
0													
OTU505	0	0	2	0	0	0	0	0	1	0	0	0	0
0	0	0	0	0	0	1	0	0	0	1	0	1	0
1	0	0	0	1	0	0	0	0	0	0	0	0	0
0	0	0	0	1	1	0	2	0	1	2	0	0	3
0	1	3	0	1	0	0	0	0	0	0	6	2	0
0	0	2	0	0	0	0	0	0	0	0	0	0	0
0	0	0	0	0	0	0	0	0	0	0	0	0	0
0	0	0	0	0	0	0	0	0	0	0	0	0	0
0													
OTU2338	0	0	1	0	1	0	0	0	0	0	0	0	0
0	1	0	0	2	1	0	0	0	0	0	0	0	0
1	0	1	0	0	0	0	0	0	0	0	0	1	0
1	2	1	0	1	0	0	0	0	2	0	1	0	0
1	0	3	2	1	0	1	0	0	0	0	0	1	0
0	0	0	0	2	0	0	2	1	0	0	1	0	0
0	0	0	0	0	1	0	0	0	0	0	0	0	0
0	0	0	0	0	0	0	1	0	0	0	0	0	1
1													
OTU284	0	0	1	0	2	1	3	9	7	14	0	0	0
0	0	0	0	0	1	39	0	1	0	0	0	2	0
1	0	0	0	0	0	2	0	0	0	2	0	4	0
0	0	2	0	0	0	0	0	0	0	0	1	0	0
0	2	0	0	0	0	0	0	0	0	0	0	1	0
0	0	0	0	1	0	0	1	0	0	0	0	0	0

0	1	0	2	0	3	0	1	0	0	0	0	0	0
0	2	0	5	1	1	0	0	9	9	2	0	0	7
0													
OTU1442	0	0	3	1	2	0	2	1	3	2	0	0	0
0	0	0	0	0	0	0	0	0	0	0	0	0	0
2	0	0	0	0	0	0	0	1	0	0	0	1	0
0	2	0	2	0	0	0	0	0	0	0	1	0	0
0	0	0	1	0	0	0	0	0	0	0	0	1	0
0	0	0	0	12	0	0	1	0	6	0	1	2	5
0	1	0	1	0	1	0	1	0	0	0	0	0	0
0	0	0	1	0	0	0	0	0	0	1	0	0	0
0													
OTU3734	0	0	1	0	0	0	1	0	0	0	0	0	0
0	0	0	0	1	0	0	0	0	0	0	2	0	0
0	0	0	0	0	0	0	0	0	0	0	0	0	0
0	0	0	0	0	0	0	0	0	0	0	0	0	0
0	0	0	0	0	0	0	0	0	0	0	0	0	0
0	0	0	0	0	0	0	0	0	0	1	0	0	0
0	0	0	1	0	0	0	0	0	0	0	0	0	0
0	0	0	0	0	0	0	0	0	0	0	0	0	0
0													
OTU8536	0	0	1	0	0	0	0	0	0	0	0	0	0
0	0	0	0	0	0	0	0	0	0	0	0	0	0
0	0	0	0	0	0	0	0	0	0	0	0	0	0
0	0	0	0	0	0	0	0	0	0	0	0	0	0
0	0	0	0	0	0	0	0	0	0	0	0	0	0
0	0	0	0	0	0	0	0	0	0	0	0	0	0
0	0	0	0	0	0	0	0	0	0	0	0	0	0
0	0	0	0	0	0	0	0	0	0	0	0	0	0
0													
OTU1172	0	0	3	0	0	0	0	0	0	0	0	0	0
0	0	0	0	0	0	0	0	0	0	0	0	0	0
0	0	0	0	0	0	0	0	0	0	0	0	0	0

0 0 0 0 0 0 0 0 0 0 0 0 0 0
0 0 0 0 0 0 0 0 0 0 0 0 0 0
0 0 0 0 0 0 0 0 0 0 0 0 0 0
0 0 0 0 0 0 0 0 0 0 0 0 0 0
0 0 0 0 0 0 0 0 0 0 0 0 0 0
0
OTU8537 0 0 1 0 0 0 0 0 0 0 0 0 0
0 0 0 0 0 0 0 0 0 0 0 0 0 0
0 0 0 0 0 0 0 0 0 0 0 0 0 0
0 0 0 0 0 0 0 0 0 0 0 0 0 0
0 0 0 0 0 0 0 0 0 0 0 0 0 0
0 0 0 0 0 0 0 0 0 0 0 0 0 0
0 0 0 0 0 0 0 0 0 0 0 0 0 0
0 0 0 0 0 0 0 0 0 0 0 0 0 0
0
OTU14622 0 0 1 0 0 0 0 0 0 0 0 0 0
0 0 0 0 0 0 0 0 0 0 0 0 0 0
0 0 0 0 0 0 0 0 0 0 0 0 0 0
0 0 0 0 0 0 0 0 0 0 0 0 0 0
0 0 0 0 0 0 0 0 0 0 0 0 0 0
0 0 0 0 0 0 0 0 0 0 0 0 0 0
0 0 0 0 0 0 0 0 0 0 0 0 0 0
0 0 0 0 0 0 0 0 0 0 0 0 0 0
0
OTU8533 0 0 1 0 0 0 0 0 0 0 0 0 0
0 0 0 0 0 0 0 0 0 0 0 0 0 0
0 0 0 0 0 0 0 0 0 0 0 0 0 0
0 0 0 0 0 0 0 0 0 0 0 0 0 0
0 0 0 0 0 0 0 0 0 0 0 0 0 0
0 0 0 0 0 0 0 0 0 0 0 0 0 0
0 0 0 0 0 0 0 0 0 0 0 0 0 0
0 0 0 0 0 0 0 0 0 0 0 0 0 0
0

OTU3049		0	0	2	4	0	1	0	1	2	2	1	4	0
	0	1	10	5	0	0	2	0	8	0	1	1	3	0
	29	4	0	0	0	0	0	0	2	0	0	0	7	0
	1	15	5	0	0	0	0	0	2	11	1	10	0	0
	4	3	0	4	0	0	0	5	5	1	7	0	6	0
	3	0	0	4	0	0	0	4	1	3	0	29	0	2
	0	2	0	3	0	1	0	1	0	3	0	1	1	0
	0	0	0	2	0	0	0	1	0	2	1	0	3	0
	1													
OTU12280		0	0	1	0	0	0	0	0	0	0	0	0	0
	0	0	0	0	0	0	0	0	0	0	0	0	0	0
	0	0	0	0	0	0	0	0	0	0	0	0	0	0
	0	0	0	0	0	0	0	0	0	0	0	0	0	0
	0	0	0	0	0	0	0	0	0	0	0	0	0	0
	0	0	0	0	0	0	0	0	0	0	0	0	0	0
	0	0	0	0	0	0	0	0	0	0	0	0	0	0
	0	0	0	0	0	0	0	0	0	0	0	0	0	0
	0													
OTU7304		0	0	1	0	0	0	0	0	0	0	0	0	0
	0	0	0	0	0	0	0	0	0	0	0	0	0	0
	0	0	0	0	0	1	0	0	0	0	0	0	0	0
	0	0	0	0	0	0	0	0	0	0	0	0	0	0
	0	0	0	0	0	0	0	0	0	0	0	0	0	0
	0	0	0	0	0	0	1	0	0	0	0	0	0	0
	0	0	0	0	0	0	0	0	0	0	0	0	0	0
	0	0	0	0	0	0	0	0	0	0	0	0	0	0
	0													
OTU4938		0	0	1	0	0	0	2	0	0	0	0	0	0
	0	4	0	0	0	0	0	0	0	0	0	0	0	0
	0	0	0	0	0	0	0	0	0	0	0	0	0	0
	3	0	0	0	0	5	0	8	0	0	0	0	0	0
	0	1	0	1	0	0	0	0	0	0	0	0	3	0
	0	2	0	0	0	1	0	0	0	2	0	0	0	1

0	0	0	0	0	2	0	0	0	1	0	0	1	0
0	0	0	0	0	0	0	2	0	0	0	0	0	0
0													
OTU8534	0	0	1	0	0	0	0	0	0	0	0	0	0
0	0	0	0	0	0	0	0	0	0	0	0	0	0
0	0	0	0	0	0	0	0	0	0	0	0	0	0
0	0	0	0	0	0	0	0	0	0	0	0	0	0
0	0	0	0	0	0	0	0	0	0	0	0	0	0
0	0	0	0	0	0	0	0	0	0	0	0	0	0
0	0	0	0	0	0	0	0	0	0	0	0	0	0
0	0	0	0	0	0	0	0	0	0	0	0	0	0
0													
OTU1473	0	0	1	0	0	0	0	0	6	0	0	0	0
0	0	0	0	0	0	0	0	0	0	0	0	0	0
0	0	0	0	0	0	0	0	0	0	0	0	0	1
0	0	0	0	1	0	0	1	6	0	2	2	0	0
0	0	0	0	0	0	0	0	1	0	0	0	7	0
0	0	0	0	0	0	0	0	0	0	0	0	1	0
0	0	0	0	0	0	0	0	0	0	0	0	0	0
0	0	1	1	4	18	13	0	0	1	1	0	1	0
0													
OTU2410	0	0	2	0	1	1	0	0	0	0	1	2	1
2	0	1	1	0	0	0	1	0	0	0	3	0	6
0	0	0	0	2	0	0	0	0	0	0	0	0	0
0	2	0	4	3	0	0	0	0	0	1	0	0	0
0	0	1	0	3	0	1	0	0	1	0	16	0	3
0	0	0	2	0	0	2	0	1	0	0	0	1	0
1	0	0	0	6	0	4	0	0	14	3	0	0	0
0	0	0	0	0	0	0	0	0	0	7	0	2	0
0													
OTU12370	0	0	1	0	0	0	0	0	0	0	0	0	0
0	1	0	0	0	0	0	0	0	0	0	0	0	0
0	0	0	0	0	0	0	0	0	0	0	0	0	0

0	0	0	0	0	0	0	0	1	0	0	0	0	0
0	0	0	0	0	0	0	0	0	0	0	0	0	0
0	0	0	0	0	0	0	0	0	0	0	0	0	0
0	0	0	0	0	0	0	0	0	0	0	0	0	0
0	0	0	0	0	0	0	0	0	0	0	0	0	0
0													
OTU14624	0	0	1	0	0	0	0	0	0	0	0	0	0
0	0	0	0	0	0	0	0	0	0	0	0	0	0
0	0	0	0	0	0	0	0	0	0	0	0	0	0
0	0	0	0	0	0	0	0	0	0	0	0	0	0
0	0	0	0	0	0	0	0	0	0	0	0	0	0
0	0	0	0	0	0	0	0	0	0	0	0	0	0
0	0	0	0	0	0	0	0	0	0	0	0	0	0
0	0	0	0	0	0	0	0	0	0	0	0	0	0
0													
OTU8535	0	0	1	0	0	0	0	0	0	0	0	0	0
0	0	0	0	0	0	0	0	0	0	0	0	0	0
0	0	0	0	0	0	0	0	0	0	0	0	0	0
0	0	0	0	0	0	0	0	0	0	0	0	0	0
0	0	0	0	0	0	0	0	0	0	0	0	0	0
0	0	0	0	0	0	0	0	0	0	0	0	0	0
0	0	0	0	0	0	0	0	0	0	0	0	0	0
0	0	0	0	0	0	0	0	0	0	0	0	0	0
0													
OTU565	0	0	3	0	0	0	0	0	0	0	0	0	0
0	0	0	0	0	0	0	0	0	0	0	0	0	0
4	0	0	0	0	0	0	0	0	0	0	0	0	0
0	2	1	0	0	0	0	0	0	0	0	1	0	0
0	0	0	0	0	0	1	0	0	0	0	0	0	0
0	15	9	0	0	0	0	0	0	0	0	0	0	0
11	1	2	0	1	0	1	0	0	0	0	1	1	0
0	30	0	0	0	0	0	1	0	0	0	0	0	0
0													

OTU8543	0	0	2	0	0	0	0	0	0	3	0	0	0
0	0	0	0	0	0	0	0	0	0	0	0	0	0
0	0	0	0	0	0	0	0	0	0	0	0	0	0
0	0	0	0	0	0	0	0	0	0	0	0	0	0
0	0	0	0	0	0	0	0	0	0	0	0	0	0
0	0	0	0	0	0	0	0	0	0	0	0	0	0
0	0	0	0	0	0	0	0	0	0	0	0	0	0
0	0	0	0	0	0	0	0	0	0	0	0	0	0
0													
OTU14623	0	0	1	0	0	0	0	0	0	0	0	0	0
0	0	0	0	0	0	0	0	0	0	0	0	0	0
0	0	0	0	0	0	0	0	0	0	0	0	0	0
0	0	0	0	0	0	0	0	0	0	0	0	0	0
0	0	0	0	0	0	0	0	0	0	0	0	0	0
0	0	0	0	0	0	0	0	0	0	0	0	0	0
0	0	0	0	0	0	0	0	0	0	0	0	0	0
0	0	0	0	0	0	0	0	0	0	0	0	0	0
0													
OTU14620	0	0	1	0	0	0	0	0	0	0	0	0	0
0	0	0	0	0	0	0	0	0	0	0	0	0	0
0	0	0	0	0	0	0	0	0	0	0	0	0	0
0	0	0	0	0	0	0	0	0	0	0	0	0	0
0	0	0	0	0	0	0	0	0	0	0	0	0	0
0	0	0	0	0	0	0	0	0	0	0	0	0	0
0	0	0	0	0	0	0	0	0	0	0	0	0	0
0	0	0	0	0	0	0	0	0	0	0	0	0	0
0													
OTU12277	0	0	1	0	0	0	0	0	1	0	0	0	0
0	0	0	0	0	0	0	0	0	0	0	0	0	0
0	0	0	0	0	0	0	0	0	0	0	0	0	0
0	0	0	0	0	0	0	0	0	0	0	0	0	0
0	0	0	0	0	0	0	0	0	0	0	0	0	0
0	0	0	0	0	0	0	0	0	0	0	0	0	0

0	0	0	0	0	0	0	0	0	0	0	0	0	0
0	0	0	0	0	0	0	0	0	0	0	0	0	0
0													
OTU12279	0	0	1	0	0	0	0	0	0	0	0	0	0
0	0	0	0	0	0	0	0	0	0	0	0	0	0
0	0	0	0	0	0	0	0	0	0	0	0	0	0
0	0	0	0	0	0	0	0	0	0	0	0	0	0
0	0	0	0	0	0	0	0	0	0	0	0	0	0
0	0	0	0	0	0	0	0	0	0	0	0	0	0
0	0	0	0	0	0	1	0	0	0	0	0	0	0
0	0	0	0	0	0	0	0	0	0	0	0	0	0
0													
OTU14621	0	0	1	0	0	0	0	0	0	0	0	0	0
0	0	0	0	0	0	0	0	1	0	0	0	0	0
0	0	0	0	0	0	0	0	0	0	0	0	0	0
0	0	0	0	0	0	0	0	0	0	0	0	0	0
0	0	0	0	0	0	0	0	0	0	0	1	0	0
0	0	0	0	0	0	0	0	0	0	0	0	0	0
0	0	0	0	1	0	0	0	0	0	0	0	0	0
0	0	0	0	0	0	0	0	0	0	0	0	0	0
0													
OTU14225	0	0	1	2	2	1	2	0	2	2	4	0	0
0	2	1	0	1	0	0	1	1	0	0	0	0	0
0	1	2	1	0	2	1	0	1	0	0	0	0	1
1	0	3	3	1	0	0	0	0	1	0	1	0	1
0	0	0	1	0	2	0	1	3	0	1	0	1	0
0	1	2	0	2	1	0	1	1	1	1	7	1	1
1	1	0	0	0	2	2	4	0	0	1	1	1	0
0	1	0	0	0	1	0	1	0	1	0	0	0	2
4													
OTU5473	0	0	1	0	2	0	0	0	0	1	0	0	0
0	0	1	0	0	0	4	0	0	0	0	0	0	0
0	0	0	0	0	0	0	0	0	0	0	0	2	0

	0	0	0	0	4	0	0	1	0	0	4	0	0	0
	0	0	0	0	0	0	0	1	0	0	0	1	0	0
	0	2	3	0	0	1	0	0	0	2	0	0	0	0
	0	2	0	0	1	0	0	0	0	0	0	0	0	0
	0	0	1	1	0	0	0	0	0	2	0	0	0	1
	0													
OTU869		0	0	2	0	1	0	24	1	7	2	0	2	0
	0	2	0	0	0	0	6	0	0	0	0	0	1	0
	25	0	0	0	0	0	0	0	0	0	0	0	1	0
	11	0	0	0	0	0	0	2	1	0	0	0	0	0
	0	1	0	1	0	0	0	0	0	0	0	0	3	0
	0	7	5	0	0	0	0	0	0	8	0	4	0	17
	1	0	0	0	0	10	0	0	0	0	0	4	0	1
	0	1	26	61	21	17	31	15	25	6	0	0	0	26
	5													
OTU8541		0	0	1	0	0	0	0	0	0	0	0	0	0
	0	0	0	0	0	0	0	0	0	0	0	0	0	0
	0	0	0	0	0	0	0	0	0	0	0	0	0	0
	0	0	0	0	0	0	0	0	0	0	0	0	0	0
	0	0	0	0	0	0	0	0	0	0	0	0	0	0
	0	0	0	0	0	0	0	0	0	0	0	0	0	0
	0	0	0	0	0	0	0	0	0	0	0	0	0	0
	0	0	0	0	0	0	0	0	0	0	0	0	0	0
	0													
OTU10762		0	0	1	0	0	0	0	0	0	0	0	0	0
	0	0	0	0	0	0	0	0	0	0	0	0	0	0
	0	0	0	0	0	0	0	0	0	0	0	0	0	0
	0	0	0	0	0	0	0	0	0	0	0	0	0	0
	0	0	0	0	0	0	0	0	0	0	0	0	0	0
	0	0	0	0	0	0	0	0	0	0	0	0	0	0
	0	0	0	0	0	0	0	0	0	0	0	0	0	0
	0	0	0	0	0	0	0	0	0	0	0	0	0	0
	0													

OTU12278 0 0 1 0 0 0 0 0 0 0 0 0 0
0 0 0 0 0 0 0 0 0 0 0 0 0 0
0 0 0 0 0 0 0 0 0 0 0 0 0 0
0 0 0 0 0 0 0 0 0 0 0 0 0 0
0 0 0 0 0 0 0 0 0 0 0 0 0 0
0 0 0 0 0 0 0 0 0 0 0 0 0 0
0 0 0 0 0 0 0 0 0 0 0 0 0 0
0 0 0 0 0 0 0 0 0 0 0 0 0 0
0
OTU14568 0 0 2 0 0 0 0 0 0 0 0 0 0
0 0 0 0 0 0 0 0 0 0 0 0 0 0
0 0 0 0 0 0 0 0 0 0 0 0 0 0
0 0 0 0 0 0 0 0 0 0 0 0 0 0
0 0 0 0 0 0 0 0 0 0 0 0 0 0
0 0 0 0 0 0 0 0 0 0 0 0 0 0
0 0 0 0 0 0 0 0 0 0 0 0 0 0
0 0 0 0 0 0 0 0 0 0 0 0 0 0
0
OTU410 0 0 1 0 0 0 4 0 6 7 1 0 0
0 1 0 1 0 0 2 0 0 0 0 0 0 0
2 0 0 0 0 0 0 0 0 0 0 0 1 0
3 0 0 0 0 5 0 0 0 0 0 2 0 0
0 0 0 0 0 0 0 0 0 0 0 0 1 0
0 0 4 0 0 0 0 0 0 4 0 11 0 0
0 0 0 9 0 3 0 0 0 0 0 0 0 0
0 1 0 0 1 1 1 0 2 1 0 0 0 4
0
OTU12288 0 0 2 0 0 0 0 0 0 0 0 0 0
0 0 0 0 0 0 0 0 0 0 0 0 0 0
0 0 0 0 0 0 0 0 0 0 0 0 0 0
0 0 0 0 0 0 0 0 0 0 0 0 0 0
0 0 0 0 0 0 0 0 0 0 0 0 0 0
0 0 0 0 0 0 0 0 0 0 0 0 0 0

0	0	0	0	0	0	0	0	0	0	0	0	0	0
0	0	0	0	0	0	0	0	0	0	0	0	0	0
0													
OTU7263	0	0	1	2	2	2	0	0	2	1	0	1	1
0	4	1	0	1	0	1	2	1	2	0	0	2	1
0	0	3	4	1	0	0	2	0	0	1	0	2	4
2	1	0	4	1	1	0	2	3	3	0	1	0	0
0	2	2	0	0	1	0	0	2	1	2	2	1	1
3	2	2	1	3	1	0	4	2	0	1	8	1	0
1	0	0	1	1	4	0	3	2	0	2	0	2	1
0	1	1	1	4	0	0	3	1	0	1	0	0	2
4													
OTU9063	0	0	1	0	0	0	0	0	0	0	0	0	0
0	0	0	0	0	0	0	0	0	0	0	0	0	0
0	0	0	0	0	0	0	0	0	0	0	2	0	2
0	0	0	0	0	0	0	0	0	0	0	0	0	0
0	0	0	0	0	0	0	0	0	0	0	0	0	0
0	0	0	0	0	0	0	0	0	0	0	0	0	0
0	0	0	0	0	0	0	0	0	0	0	0	0	0
0	0	0	0	0	0	0	0	0	0	0	0	0	0
0													
OTU986	0	0	8	0	0	0	0	0	0	0	0	0	0
0	0	0	0	0	0	0	0	0	0	0	0	0	0
0	0	0	0	0	0	0	0	0	0	0	0	0	0
0	0	0	0	0	0	0	0	0	0	0	0	0	0
0	0	0	0	0	0	0	0	0	0	0	0	0	0
0	0	0	0	0	0	0	0	0	0	0	0	0	0
0	0	0	0	0	0	0	0	0	0	0	0	0	0
0	0	0	0	0	0	0	0	0	0	0	0	0	0
0													
OTU13585	0	0	1	0	0	0	0	0	0	0	0	0	0
0	0	0	0	0	0	0	0	0	0	0	0	0	0
0	0	0	0	0	0	0	0	0	0	0	0	0	0

0 0 0 0 0 0 0 0 0 0 0 0 0 0
0 0 0 0 0 0 0 0 0 0 0 0 0 0
0 0 0 0 0 0 0 0 0 0 1 0 0 0
0 0 0 0 0 0 0 0 0 0 0 0 0 0
0 0 0 0 0 0 0 0 0 0 0 0 0 0
0
OTU701 0 0 1 0 0 0 0 0 1 0 0 0 0
0 0 0 0 0 0 0 0 0 0 0 0 0 0
0 0 0 0 0 0 0 0 0 0 0 0 0 0
0 0 0 0 0 0 0 0 0 0 0 0 0 0
0 0 0 0 0 0 0 0 0 0 0 0 0 0
0 4 0 0 0 0 0 0 0 0 0 0 0 0
0 0 0 0 0 0 0 0 0 0 0 0 0 0
0 2 2 0 3 7 0 0 0 0 0 0 0 0
0
OTU8539 0 0 1 0 0 0 0 0 0 0 0 0 0
0 0 0 0 0 0 0 0 0 0 0 0 0 0
0 0 0 0 0 0 0 0 0 0 0 0 0 0
0 0 0 0 0 0 0 0 0 0 0 0 0 0
0 0 0 0 0 0 0 0 0 0 0 0 0 0
0 0 0 0 0 0 0 0 0 0 0 0 0 0
0 0 0 0 0 0 0 0 0 0 0 0 0 0
0 0 0 0 0 0 0 0 0 0 0 0 0 0
0
OTU12287 0 0 1 0 0 0 0 0 0 0 0 0 0
0 0 0 0 0 0 0 0 0 0 0 0 0 0
0 0 0 0 0 0 0 0 0 0 0 0 0 0
0 0 0 0 0 0 0 0 0 0 0 0 0 0
0 0 0 0 0 0 0 0 0 0 0 0 0 0
0 0 0 0 0 0 0 0 0 0 0 0 0 0
0 0 0 0 0 0 0 0 0 0 0 0 0 0
0 0 0 0 0 0 0 0 0 0 0 0 0 0
0

OTU7500	0	0	2	0	0	3	0	0	0	0	0	0	0
0	0	0	0	1	0	0	0	0	0	0	0	0	0
0	0	0	0	0	0	0	0	0	0	0	0	0	0
0	0	0	0	0	0	0	0	0	0	0	0	0	0
0	0	0	0	0	0	0	0	0	0	0	1	0	0
0	0	0	0	0	0	0	0	1	0	0	1	1	0
0	0	0	0	1	0	1	0	0	0	0	0	0	0
0	0	0	0	0	0	0	0	0	0	0	0	0	0
1													
OTU12289	0	0	1	0	0	0	0	0	0	0	0	0	0
0	0	0	0	0	0	0	0	0	0	0	0	0	0
0	0	0	0	0	0	0	0	0	0	0	0	0	0
0	0	0	0	0	0	0	0	0	0	0	0	0	0
0	0	0	0	0	0	0	0	0	0	0	0	0	0
0	0	0	0	0	0	0	0	0	0	0	0	0	0
0	0	0	0	0	0	0	0	0	0	0	0	0	0
0	0	0	0	0	0	0	0	0	0	0	0	0	0
0													
OTU10766	0	0	1	0	0	0	0	0	0	0	0	0	0
0	0	0	0	0	0	0	0	0	0	0	0	0	0
0	0	0	0	0	0	0	0	0	0	0	0	0	0
0	0	0	0	0	0	0	0	0	0	0	0	0	0
0	0	0	0	0	0	0	0	0	0	0	0	0	0
0	0	0	0	0	0	0	0	0	0	0	0	0	0
0	0	0	0	0	0	0	0	0	0	0	0	0	0
0	0	0	0	0	0	0	0	0	0	0	0	0	0
0													
OTU373	0	0	2	0	4	1	3	1	1	0	0	6	1
0	6	0	0	0	1	3	1	0	0	0	0	1	0
0	0	0	0	0	1	0	1	0	0	0	1	0	0
2	19	0	4	2	0	1	10	0	1	0	0	0	1
1	11	0	0	1	0	0	0	0	0	0	1	13	0
1	0	1	0	4	0	0	2	0	0	1	2	0	2

2	5	1	3	1	1	1	0	0	4	0	0	0	2
2	0	0	0	0	0	0	0	0	0	0	0	1	0
0													
OTU12290	0	0	1	0	0	0	0	0	0	0	0	0	0
0	0	0	0	0	0	0	0	0	0	0	0	0	0
0	0	0	0	0	0	0	0	0	0	0	0	0	0
0	0	0	0	0	0	0	0	0	0	0	0	0	0
0	0	0	0	0	0	0	0	0	0	0	0	0	0
0	0	0	0	0	0	0	0	0	0	0	0	0	0
0	0	0	0	0	0	0	0	0	0	0	0	0	0
0	0	0	0	0	0	0	0	0	0	0	0	0	0
0													
OTU10769	0	0	1	0	0	0	0	0	0	0	0	0	0
0	0	0	0	0	0	0	0	0	0	0	0	0	0
0	0	0	0	0	0	0	0	0	0	0	0	0	0
0	0	0	0	0	0	0	0	0	0	0	0	0	0
0	0	0	0	0	0	0	0	0	0	0	0	0	0
0	0	0	0	0	0	0	0	0	0	0	0	0	0
0	0	0	0	0	0	0	0	0	0	0	0	0	0
0	0	0	0	0	0	0	0	0	0	0	0	0	0
0													
OTU11958	0	0	1	0	0	0	0	0	0	0	0	0	0
0	0	0	0	0	0	0	0	2	0	0	1	0	2
0	0	0	0	0	0	0	0	0	1	0	0	1	0
2	0	0	0	0	0	0	0	0	2	0	0	0	0
0	0	0	1	0	0	0	0	0	1	0	0	2	0
0	0	0	0	0	0	0	0	0	0	0	0	0	0
0	0	0	0	0	1	0	0	0	0	1	0	0	0
0	1	0	0	0	0	0	0	0	0	0	0	0	0
3													
OTU8538	0	0	1	0	0	0	0	0	0	0	0	0	0
0	0	0	0	0	0	0	0	0	0	0	0	0	0
0	0	0	0	0	0	0	0	0	0	0	0	0	0

0	0	0	0	0	0	0	0	0	0	0	0	0	0
0	0	0	0	0	0	0	0	0	0	0	0	0	0
0	0	0	0	0	0	0	0	0	0	0	0	0	0
0	0	0	0	0	0	0	0	0	0	0	0	0	0
0	0	0	0	0	0	0	0	0	0	0	0	0	0
0													
OTU6237	0	0	3	1	6	1	0	8	0	0	0	1	0
0	1	0	0	0	0	0	0	0	0	0	0	1	1
2	0	0	0	0	0	3	0	1	0	0	0	2	0
0	0	0	2	0	1	0	4	0	0	0	1	0	0
0	0	0	1	0	0	0	2	0	0	0	5	0	0
0	0	1	3	0	1	0	0	0	0	0	0	0	1
0	0	2	6	2	0	2	0	0	0	0	0	0	0
4	0	0	0	0	0	0	0	0	0	0	0	0	0
0													
OTU8530	0	0	1	0	0	0	0	0	0	0	0	0	0
0	0	0	0	0	0	0	0	0	0	0	0	0	0
0	0	0	0	0	0	0	0	0	0	0	0	0	0
0	0	0	0	0	0	0	0	0	0	0	0	0	0
0	0	0	0	0	0	0	0	0	0	0	0	0	0
0	0	0	0	0	0	0	0	0	0	0	0	0	0
0	0	0	0	0	0	0	0	0	0	0	0	0	0
0	0	0	0	0	0	0	0	0	0	0	0	0	0
0													
OTU10768	0	0	1	0	0	0	0	0	0	0	0	0	0
0	0	0	0	0	0	0	0	0	0	0	0	0	0
0	0	0	0	0	0	0	0	0	0	0	0	0	0
0	0	0	0	0	0	0	0	0	0	0	0	0	0
0	0	0	0	0	0	0	0	0	0	0	0	0	0
0	0	0	0	0	0	0	0	0	0	0	0	0	0
0	0	0	0	0	0	0	0	0	0	0	0	0	0
0	0	0	0	0	0	0	0	0	0	0	0	0	0
0													

OTU5485	0	0	1	0	0	0	0	0	0	0	0	4	0
0	0	0	1	0	0	0	0	2	0	0	1	1	0
0	0	0	0	0	0	1	0	0	0	0	0	0	0
0	0	0	1	1	0	3	1	0	0	0	0	0	0
0	0	0	1	0	0	0	0	0	0	0	0	0	0
0	0	0	0	0	0	0	1	0	0	0	0	1	0
0	0	0	0	0	0	0	0	0	1	0	0	0	0
0	0	0	0	0	0	0	0	1	1	0	0	1	0
0													
OTU438	0	0	1	0	0	0	0	0	0	0	0	0	0
1	0	0	0	0	0	1	0	0	0	0	0	0	0
0	0	0	0	0	0	0	0	0	0	32	0	0	0
11	0	0	0	0	0	0	0	0	0	0	0	0	0
0	2	0	0	0	0	0	0	0	0	0	0	3	0
1	0	0	0	0	0	0	0	0	0	0	0	0	0
0	1	0	0	0	0	0	2	0	0	0	0	0	0
0	4	1	4	5	0	9	1	6	3	0	0	0	2
0													
OTU16678	0	0	1	0	0	0	1	0	0	4	0	0	0
0	4	0	0	0	0	4	0	0	0	0	0	0	0
0	0	1	0	0	0	0	0	0	0	0	0	0	0
3	0	0	0	0	3	0	9	0	0	0	0	0	0
0	1	0	0	0	0	0	0	0	0	0	0	8	0
0	4	1	0	0	0	0	0	0	2	0	0	1	3
0	0	0	0	0	3	0	0	0	0	0	2	4	0
0	2	0	1	1	0	1	2	0	1	0	0	0	0
0													
OTU12283	0	0	1	0	0	0	0	0	0	0	0	0	0
0	0	0	0	0	0	0	0	0	0	0	0	0	0
0	0	0	0	0	0	0	0	0	0	0	0	0	0
0	0	0	0	0	0	0	0	0	0	0	0	0	0
0	0	0	0	0	0	0	0	0	0	0	0	0	0
0	0	0	0	0	0	0	0	0	0	0	0	0	0

0	0	0	0	0	0	0	0	0	0	0	0	0	0
0	0	1	0	0	0	0	0	0	0	0	0	0	0
0													
OTU613	0	0	4	0	0	3	5	0	0	0	0	0	0
0	0	0	0	0	0	1	0	0	0	0	0	0	0
0	0	0	0	0	0	2	0	0	0	0	0	0	0
4	0	0	0	0	0	0	1	0	0	0	0	0	0
0	1	0	0	0	0	0	0	0	0	0	0	1	0
0	1	0	0	0	0	0	0	0	0	0	0	0	0
0	0	0	0	0	0	0	0	0	0	0	5	1	0
0	2	0	1	0	1	0	0	0	0	0	0	0	1
5													
OTU6004	0	0	1	4	0	0	2	0	1	2	0	0	0
1	2	0	2	0	0	0	1	1	0	0	1	0	1
0	2	2	0	0	0	0	0	0	0	0	2	3	0
0	1	1	1	0	1	0	1	1	2	1	0	1	0
0	0	2	1	3	0	0	0	0	1	2	1	1	0
5	0	1	0	2	1	0	1	0	0	0	5	0	2
1	0	0	0	2	2	0	1	0	0	1	1	2	1
0	1	0	2	0	0	0	2	0	0	1	0	0	0
2													
OTU12527	0	0	1	0	0	1	0	0	0	0	9	0	0
2	0	3	0	4	0	0	2	0	0	0	0	0	1
0	0	2	4	0	0	0	0	0	0	0	0	0	0
0	0	2	0	1	1	1	1	1	0	1	0	0	0
0	2	0	1	0	0	0	0	0	0	0	1	0	0
0	0	0	0	0	0	0	0	0	0	0	1	0	0
1	0	0	0	0	0	0	0	0	0	0	0	1	0
0	0	4	0	0	3	0	1	0	0	0	1	0	0
1													
OTU9828	0	0	1	0	0	0	0	0	1	1	0	0	0
0	0	0	0	0	0	0	0	0	0	0	0	0	0
0	0	0	0	0	0	0	0	0	0	0	0	0	0

0 0 0 0 0 0 0 0 0 0 0 0 0 0
0 0 0 0 0 0 0 0 0 0 0 0 0 0
0 0 0 0 0 0 0 1 0 0 0 4 0 0
0 0 0 1 0 0 0 0 0 0 0 0 1 0
0 0 0 0 0 0 0 0 0 0 0 0 0 0
0
OTU12281 0 0 1 0 0 0 0 0 0 0 0 0 0
0 0 0 0 0 0 0 0 0 0 0 0 0 0
0 0 0 0 0 0 0 0 0 0 0 0 0 0
0 0 0 0 0 0 0 0 0 0 0 0 0 0
0 0 0 0 0 0 0 0 0 0 0 0 0 0
0 0 0 0 0 0 0 0 0 0 0 0 0 0
0 0 0 0 0 0 0 0 0 0 0 0 0 0
0 0 0 0 0 0 0 0 0 0 0 0 0 0
0
OTU353 0 0 1 6 0 0 0 0 0 0 1 4 14
5 0 0 0 1 2 0 0 0 0 0 0 0 5
0 0 0 3 0 0 0 18 2 0 0 0 0 1
0 1 0 0 9 0 1 0 0 2 0 1 8 1
11 0 0 2 0 3 1 0 3 0 1 0 2 0
6 0 0 0 0 0 0 0 1 0 0 1 0 0
0 1 0 0 0 0 0 0 2 1 7 0 0 2
11 0 0 0 0 0 0 0 2 0 5 35 4 0
0
OTU12282 0 0 1 0 0 0 0 0 0 0 0 0 0
0 0 0 0 0 0 0 0 0 0 0 0 0 0
0 0 0 0 0 0 0 0 0 0 0 0 0 0
0 0 0 0 0 0 0 0 0 0 0 0 0 0
0 0 0 0 0 0 0 0 0 0 0 0 0 0
0 0 0 0 0 0 0 0 0 0 0 0 0 0
0 0 0 0 0 0 0 0 0 0 0 0 0 0
0 0 0 0 0 0 0 0 0 0 0 0 0 0
0

OTU600	0	0	1	0	0	0	1	0	0	1	0	1	0
0	0	0	0	0	0	0	0	0	0	0	0	0	0
0	0	0	0	0	0	0	0	0	0	0	0	0	0
1	0	0	0	0	0	0	0	0	0	0	0	0	0
0	1	0	0	0	0	0	0	0	0	0	0	0	0
0	1	0	0	2	0	0	0	0	0	0	0	1	0
0	0	0	1	0	0	0	0	0	0	0	1	5	0
0	4	1	3	1	0	0	3	0	5	0	0	0	0
2													
OTU10196	0	0	1	0	0	0	0	0	0	0	0	0	0
0	0	0	1	0	0	0	0	0	0	1	1	1	0
0	0	0	0	1	0	1	0	0	0	1	1	0	0
0	0	2	0	3	1	0	0	1	1	1	0	0	0
0	0	0	0	1	2	0	2	5	0	3	0	0	1
2	0	0	0	0	0	0	0	1	0	0	1	0	0
0	13	0	0	0	0	0	0	0	0	0	0	0	0
0	0	1	0	0	1	0	0	0	0	0	0	0	0
0													
OTU1171	0	0	2	0	0	0	0	0	0	0	0	0	0
0	0	0	0	0	0	0	0	0	0	0	0	0	0
0	0	0	0	0	0	0	0	0	0	0	0	0	0
0	0	0	0	0	0	0	0	0	0	0	0	0	0
0	0	0	0	0	0	0	0	0	0	0	0	0	0
0	0	0	0	0	0	0	0	0	0	0	0	0	0
0	0	0	0	0	0	0	0	0	0	0	0	0	0
0	0	0	0	0	0	0	0	0	0	0	0	0	0
0													
OTU2357	0	0	1	0	0	0	0	0	0	0	0	0	0
0	0	0	0	0	0	0	0	0	0	0	0	0	0
0	0	0	0	0	0	0	0	0	0	0	0	0	0
0	1	0	0	0	0	0	0	0	0	0	0	0	0
0	0	1	0	0	0	0	0	0	0	0	0	0	0
1	0	0	0	0	0	0	0	0	0	0	2	0	0

0	0	0	0	0	0	0	0	0	0	0	0	0	0
0	0	0	0	0	0	0	0	0	0	0	0	0	0
0													
OTU13079	0	0	1	0	0	0	0	0	0	0	0	0	0
0	0	0	0	0	0	0	0	0	0	0	0	0	0
0	0	0	1	0	0	0	0	0	0	0	0	0	0
0	0	0	0	0	0	0	0	0	0	0	0	0	0
0	0	0	0	0	0	0	0	0	0	0	0	0	0
0	0	0	0	0	0	0	0	0	0	0	0	0	0
0	0	0	0	0	0	0	0	0	0	0	0	0	0
0	0	0	0	0	0	0	0	0	0	0	0	0	0
0													
OTU12286	0	0	2	0	0	0	0	0	0	0	0	0	0
0	2	0	0	0	0	0	0	0	0	0	0	0	0
0	0	0	0	0	0	0	0	0	0	0	0	0	0
0	0	0	0	0	0	0	0	0	1	0	0	0	0
1	0	0	0	0	0	0	0	0	0	0	0	0	0
1	0	0	0	0	0	0	0	0	0	1	0	0	0
0	0	0	1	0	0	0	0	0	0	0	0	0	1
0	0	0	0	0	0	0	0	0	0	0	0	0	0
0													
OTU1514	0	0	1	0	0	0	0	0	0	0	0	0	0
0	0	0	0	0	0	0	0	0	0	1	0	0	0
0	1	0	0	1	0	0	0	0	0	0	0	0	0
0	0	0	0	0	1	0	1	0	0	0	1	1	0
0	0	1	0	0	0	0	3	1	0	1	1	0	0
0	0	0	0	0	0	0	2	0	0	0	5	0	0
0	0	0	0	0	0	0	0	0	0	0	0	0	0
0	0	0	0	0	0	1	0	0	0	1	0	0	0
0													
OTU12574	0	0	2	0	0	0	0	1	0	1	0	0	0
0	0	0	0	0	0	0	0	1	0	0	0	0	0
0	0	0	1	0	2	0	0	0	0	0	0	0	0

0	0	0	0	1	0	0	0	1	1	0	0	0	0
0	0	0	0	0	0	0	1	0	0	0	0	0	0
0	0	0	0	1	0	0	1	0	0	0	2	0	0
0	0	1	0	0	1	0	1	0	1	0	0	1	0
0	1	0	0	3	0	0	0	0	0	1	0	0	0
0													
OTU12284	0	0	1	0	0	0	0	0	0	0	0	0	0
0	0	0	0	0	0	0	0	0	0	0	0	0	0
0	0	0	0	0	0	0	0	0	0	0	0	0	0
0	0	0	0	0	0	0	0	0	0	0	0	0	0
0	0	0	0	0	0	0	0	0	0	0	0	0	0
0	0	0	0	0	0	0	0	0	0	0	0	0	0
0	0	0	0	0	0	0	0	0	0	0	0	0	0
0	0	0	0	0	0	0	0	0	0	0	0	0	0
0													
OTU10765	0	0	1	0	0	0	0	0	0	0	0	0	0
0	0	0	0	0	0	0	0	0	0	0	0	0	0
0	0	0	0	0	0	0	0	0	0	0	0	0	0
0	0	0	0	0	0	0	0	0	0	0	0	0	0
0	0	0	0	0	0	0	0	0	0	0	0	0	0
0	0	0	0	0	0	0	0	0	0	0	0	0	0
0	0	0	0	0	0	0	0	0	0	0	0	0	0
0	0	0	0	0	0	0	0	0	0	0	0	0	0
0													
OTU8528	0	0	1	0	0	0	0	0	0	0	0	0	0
0	0	0	0	0	0	0	0	0	0	0	0	0	0
0	0	0	0	0	0	0	0	0	0	0	0	0	0
0	0	0	0	0	0	0	0	0	0	0	0	0	0
0	0	0	0	0	0	0	0	0	0	0	1	0	0
0	0	0	0	0	0	0	0	0	0	0	0	0	0
0	0	0	0	0	0	0	0	0	0	0	0	0	0
0	0	0	0	0	0	0	0	0	0	0	0	0	0
0													

OTU5514 0 0 1 0 0 0 0 0 0 0 0 0 0
0 0 0 0 0 0 0 0 0 0 0 0 0 0
0 0 0 0 0 0 0 0 0 0 0 0 0 0
0 0 0 0 1 0 0 0 0 0 0 0 0 0
0 0 0 0 0 0 0 0 0 0 0 0 0 0
0 0 0 0 0 0 0 0 0 0 0 0 0 0
0 0 0 0 0 0 0 0 0 0 0 0 0 0
0 0 0 0 0 0 0 0 0 0 0 0 0 0
0
OTU15934 0 0 2 2 0 0 0 3 0 1 0 0 0
0 0 0 0 0 0 3 0 0 0 0 0 0 0
0 0 0 0 0 0 0 0 0 0 0 0 2 0
1 0 0 0 0 0 0 0 0 0 0 0 0 0
0 0 0 0 0 0 0 0 0 0 0 0 0 0
0 0 2 0 1 0 0 0 1 0 0 0 0 0
0 0 0 0 0 0 0 0 0 0 0 0 0 0
0 0 0 0 0 0 0 0 0 0 0 0 0 0
0
OTU14608 0 0 1 0 0 0 0 0 0 0 0 0 0
0 0 0 0 0 0 0 0 0 0 0 0 0 0
0 0 0 0 0 0 0 0 0 0 0 0 0 0
0 0 0 0 0 0 0 0 0 0 0 0 0 0
0 0 0 0 0 0 0 0 0 0 0 0 0 0
0 0 0 0 0 0 0 0 0 0 0 0 0 0
0 0 0 0 0 0 0 0 0 0 0 0 0 0
0 0 0 0 0 0 0 0 0 0 0 0 0 0
0
OTU10764 0 0 1 0 0 0 0 0 0 0 0 0 0
0 0 0 0 0 0 0 0 0 0 0 0 0 0
0 0 0 0 0 0 0 0 0 0 0 0 0 0
0 0 0 0 0 0 0 0 0 0 0 0 0 0
0 0 0 0 0 0 0 0 0 0 0 0 0 0
0 0 0 0 0 0 0 0 0 0 0 0 0 0

0	0	0	0	0	0	0	0	0	0	0	0	0	0
0	0	0	0	0	0	0	0	0	0	0	0	0	0
0													
OTU4655	0	0	1	0	0	1	0	0	0	0	0	0	1
2	0	2	0	1	0	0	1	0	0	0	1	0	0
0	1	0	0	0	0	0	0	0	0	0	0	0	0
0	0	0	1	1	0	0	0	0	0	0	0	1	0
0	0	0	0	0	0	0	0	0	0	0	1	0	0
1	0	0	0	0	0	0	0	2	0	1	0	0	0
0	0	0	0	0	0	0	0	0	0	0	0	0	0
0	0	0	0	0	1	0	0	0	0	0	1	1	0
0													
OTU12318	0	0	1	0	0	0	0	0	0	0	0	0	0
0	0	0	0	0	0	0	0	0	0	0	1	0	0
0	0	0	0	0	0	0	0	0	0	0	0	0	0
0	0	0	0	0	0	0	0	0	0	0	0	0	0
0	0	0	0	0	0	0	0	0	0	0	0	0	0
0	0	0	0	0	0	0	0	0	0	0	0	0	0
0	0	0	0	0	0	0	0	0	0	0	0	1	0
0	0	0	0	0	0	0	0	0	0	0	0	0	0
0													
OTU14610	0	0	1	0	0	0	0	0	0	0	0	0	0
0	0	0	0	0	0	0	0	0	0	0	0	0	0
0	0	0	0	0	0	0	0	0	0	0	0	0	0
0	0	0	0	0	0	0	0	0	0	0	0	0	0
0	0	0	0	0	0	0	0	0	0	0	0	0	0
0	0	0	0	0	0	0	0	0	0	0	0	0	0
0	0	0	0	0	0	0	0	0	0	0	0	0	0
0	0	0	0	0	0	0	0	0	0	0	0	0	0
0													
OTU14611	0	0	1	0	0	0	0	0	0	0	0	0	0
0	0	0	0	0	0	0	0	0	0	0	0	0	0
0	0	0	0	0	0	0	0	0	0	0	0	0	0

0 0 0 0 0 0 0 0 0 0 0 0 0 0
0 0 0 0 0 0 0 0 0 0 0 0 0 0
0 0 0 0 0 0 0 0 0 0 0 0 0 0
0 0 0 0 0 0 0 0 0 0 0 0 0 0
0 0 0 0 0 0 0 0 0 0 0 0 0 0
0
OTU10755 0 0 1 0 0 0 0 0 0 0 0 0 0
0 0 0 0 0 0 0 0 0 0 0 0 0 0
0 0 0 0 0 0 0 0 0 0 0 0 0 0
0 0 0 0 0 0 0 0 0 0 0 0 0 0
0 0 0 0 0 0 0 0 0 0 0 0 0 0
0 0 0 0 0 0 0 0 0 0 0 0 0 0
0 0 0 0 0 0 0 0 0 0 0 0 0 0
0 0 0 0 0 0 0 0 0 0 0 0 0 0
0
OTU1566 0 0 2 2 0 1 3 0 4 1 0 1 0
0 1 1 0 1 0 1 2 0 0 0 0 1 0
0 0 0 0 0 0 0 1 0 1 0 0 0 0
0 1 1 3 0 0 0 1 0 0 0 0 0 0
0 0 0 0 0 0 0 0 0 0 0 1 2 0
0 4 0 0 0 0 0 1 10 6 1 1 0 3
0 1 0 2 6 1 0 3 0 17 0 3 45 0
0 22 30 55 1 14 16 20 29 86 11 1 6 22
14
OTU6062 0 0 1 0 0 0 0 0 0 0 0 0 0
0 0 0 0 0 0 0 0 0 0 0 0 0 0
0 0 0 0 0 0 0 0 0 0 0 0 0 0
0 0 0 0 0 0 0 0 0 0 0 0 0 0
0 0 0 0 0 0 0 0 0 0 0 0 0 0
0 0 1 0 0 0 0 0 0 0 0 0 0 0
0 0 0 0 0 0 0 0 0 0 0 0 0 0
0 0 0 0 0 0 0 0 0 0 0 0 0 0
0

OTU889	0	0	2	1	0	4	4	1	1	2	0	11	0
1	2	1	0	0	0	0	1	0	1	0	1	1	0
0	0	0	0	0	2	0	5	0	7	0	0	0	0
0	13	0	1	0	0	0	0	0	0	0	0	0	0
0	0	0	1	0	0	0	0	0	0	0	0	0	0
0	0	7	2	9	1	0	0	4	2	1	0	2	0
0	0	1	1	2	0	1	0	0	1	5	0	0	0
5	0	0	0	0	0	0	0	0	0	7	2	8	0
0													
OTU8529	0	0	1	0	0	0	0	0	0	0	0	0	0
0	0	0	0	0	0	0	0	0	0	0	0	0	0
0	0	0	0	0	0	0	0	0	0	0	0	0	0
0	0	0	0	0	0	0	0	0	0	0	0	0	0
0	0	0	0	0	0	0	0	0	0	0	0	0	0
0	0	0	0	0	0	0	0	0	0	0	0	0	0
0	0	0	0	0	0	0	0	0	0	0	0	0	0
0	0	0	0	0	0	0	0	0	0	0	0	0	0
0													
OTU14609	0	0	1	0	0	0	0	0	0	0	0	0	0
0	0	0	0	0	0	0	0	0	0	0	0	0	0
0	0	0	0	0	0	0	0	0	0	0	0	0	0
0	0	0	0	0	0	0	0	0	0	0	0	0	0
0	0	0	0	0	0	0	0	0	0	0	0	0	0
0	0	0	0	0	0	0	0	0	0	0	0	0	0
0	0	0	0	0	0	0	0	0	0	0	0	0	0
0	0	0	0	0	0	0	0	0	0	0	0	0	0
0													
OTU12316	0	0	1	0	0	0	0	0	0	0	0	0	0
0	0	0	0	0	0	0	0	0	0	0	0	0	0
0	0	0	0	0	0	0	0	0	0	0	0	0	0
0	0	0	0	0	0	0	0	0	0	0	0	0	0
0	0	0	0	0	0	0	0	0	0	0	0	0	0
0	0	0	0	0	0	0	0	0	0	0	0	0	0

0	0	0	0	0	0	0	0	0	0	0	0	0	0
0	0	0	0	0	0	0	0	0	0	0	0	0	0
0													
OTU12317	0	0	1	0	0	0	0	0	0	0	0	0	0
0	0	0	0	0	0	0	0	0	0	0	0	0	0
0	0	0	0	0	0	0	0	0	0	0	0	0	0
0	0	0	0	0	0	0	0	0	0	0	0	0	0
0	0	0	0	0	0	0	0	0	0	0	0	0	0
0	0	0	0	0	0	0	0	0	0	0	0	0	0
0	0	0	0	0	0	0	0	0	0	0	0	0	0
0	0	0	0	0	0	0	0	0	0	0	0	0	0
0													
OTU16089	0	0	1	0	0	1	0	0	0	0	0	0	0
0	0	0	0	0	0	0	0	0	0	0	0	0	0
0	0	0	0	0	0	0	0	0	1	0	1	0	0
0	0	0	0	0	0	0	0	0	0	0	0	0	0
0	1	0	0	0	0	0	0	0	0	0	0	0	0
0	0	0	0	0	0	0	0	0	0	0	0	0	0
0	0	0	0	0	0	0	0	0	0	0	0	0	0
0	0	0	0	0	0	0	0	0	0	0	0	0	0
0													
OTU1387	0	0	1	0	0	0	0	0	0	0	0	1	0
0	0	0	0	0	0	0	0	0	0	0	0	0	0
0	0	0	0	0	0	0	0	0	0	0	0	0	0
0	0	0	0	0	0	0	0	0	0	0	0	0	0
0	0	0	0	0	0	0	0	0	0	0	0	0	0
0	0	1	0	0	0	0	0	0	0	0	0	0	0
0	0	0	0	0	0	0	0	0	0	0	0	0	0
0	0	0	0	0	0	0	0	0	0	0	0	0	0
0													
OTU5466	0	0	1	0	0	0	0	0	0	0	0	0	0
0	0	0	0	0	0	0	0	0	0	0	0	0	0
0	0	0	0	3	0	2	0	0	0	0	0	1	0

0	0	0	0	0	1	0	0	0	0	1	0	1	0
0	0	0	0	0	0	0	0	0	0	0	0	0	0
0	0	1	0	0	0	0	0	0	1	0	0	0	0
0	0	0	0	0	0	0	0	0	0	0	0	0	0
0	0	0	0	0	0	0	0	0	0	0	0	0	0
0													
OTU12319	0	0	2	0	0	0	0	0	0	0	0	0	0
0	0	0	0	0	0	0	0	0	0	0	0	0	0
0	0	0	0	0	0	0	0	0	0	0	0	0	0
0	0	0	0	0	0	0	0	1	0	0	0	0	0
0	0	0	0	0	0	0	0	0	0	0	0	0	0
0	0	0	0	0	0	0	0	0	0	0	0	0	0
0	1	0	0	0	0	0	0	0	0	0	0	0	0
0	0	0	0	0	0	0	0	0	0	0	0	0	0
0													
OTU4806	0	0	1	0	0	0	0	0	0	0	0	0	0
0	1	0	0	0	0	0	0	0	0	0	0	0	0
0	0	1	0	0	0	0	0	1	0	0	0	0	0
0	0	0	0	0	0	0	4	0	0	0	0	0	0
0	0	0	1	0	0	0	0	0	0	0	0	0	0
0	0	0	1	0	0	0	0	0	1	0	0	0	0
1	0	0	0	0	1	0	0	0	0	1	0	0	0
1	0	0	0	0	0	0	0	0	0	0	0	0	0
0													
OTU15319	0	0	1	0	0	0	0	0	0	0	0	0	0
0	0	0	1	0	0	0	2	0	0	1	0	0	0
0	0	0	1	0	0	1	0	0	0	0	1	0	0
0	0	0	0	3	0	0	0	2	0	0	2	5	0
1	0	0	1	1	1	0	0	5	2	0	1	0	0
0	0	0	0	0	0	0	3	1	0	0	2	0	0
0	0	0	0	0	0	0	0	0	0	0	0	0	0
1	0	0	0	0	0	0	0	0	0	0	0	1	0
0													

OTU4364	0	0	2	0	1	4	6	0	0	3	0	0	0
0	0	0	0	0	0	5	0	0	0	0	0	1	0
1	0	0	0	0	0	1	0	0	0	0	0	3	0
0	0	0	0	2	0	0	1	0	0	2	0	0	1
0	2	0	0	0	1	0	0	0	0	0	1	0	0
0	0	2	0	4	1	0	1	0	2	1	0	0	4
0	0	0	1	0	1	0	0	0	0	0	0	0	0
0	0	2	0	0	0	0	1	0	0	0	0	0	0
0													
OTU14341	0	0	1	0	0	0	0	0	0	2	0	0	0
0	0	0	0	0	0	0	0	0	0	0	0	0	0
0	0	0	0	0	0	0	0	0	0	0	0	0	0
0	0	0	0	0	0	0	0	0	0	0	0	0	0
0	0	0	0	0	0	0	0	0	0	0	0	0	0
0	0	0	0	0	0	0	0	0	0	0	0	0	0
0	0	0	0	0	0	0	0	0	0	0	0	0	0
0	0	0	0	0	0	0	0	0	0	1	0	0	0
0													
OTU3654	0	0	1	0	0	0	0	0	1	0	0	0	0
0	0	0	0	0	0	0	0	0	0	0	0	12	0
17	0	0	0	0	0	3	0	0	0	0	0	3	0
0	0	0	0	0	0	0	0	0	0	0	0	0	0
0	0	0	0	0	0	0	0	3	0	0	0	0	0
0	0	0	0	0	0	0	0	0	1	0	0	0	0
0	3	0	0	0	0	0	0	0	0	0	0	0	0
0	0	0	0	0	0	0	0	0	0	0	0	2	0
0													
OTU14616	0	0	1	0	0	0	0	0	0	0	0	0	0
0	0	0	0	0	0	0	0	0	0	0	0	0	0
0	0	0	0	0	0	0	0	0	0	0	0	0	0
0	0	0	0	0	0	0	0	0	0	0	0	0	0
0	0	0	0	0	0	0	0	0	0	0	0	0	0
0	0	0	0	0	0	0	0	0	0	0	0	0	0

0	0	0	0	0	0	0	0	0	0	0	0	0	0
0	0	0	0	0	0	0	0	0	0	0	0	0	0
0													
OTU8532	0	0	1	0	0	0	0	0	0	0	0	0	0
0	0	0	0	0	0	0	0	0	0	0	0	0	0
0	0	0	0	0	0	0	0	0	0	0	0	0	0
0	0	0	0	0	0	0	0	0	0	0	0	0	0
0	0	0	0	0	0	0	0	0	0	0	0	0	0
0	0	0	0	0	0	0	0	0	0	0	0	0	0
0	0	0	0	0	0	0	0	0	0	0	0	0	0
0	0	0	0	0	0	0	0	0	0	0	0	0	0
0													
OTU12473	0	0	1	0	0	0	0	0	2	0	0	0	0
0	0	0	0	0	0	0	0	0	0	0	0	0	0
0	0	0	0	0	0	0	0	0	0	0	0	0	0
0	0	0	0	0	0	0	0	0	0	0	0	0	0
0	0	0	0	0	0	0	0	0	0	0	0	0	0
0	0	0	0	1	0	0	0	0	0	0	0	0	0
0	0	0	0	0	0	0	2	0	0	0	0	0	0
0	0	0	0	0	0	0	0	0	0	0	0	0	0
0													
OTU11121	0	0	1	0	0	0	0	0	4	2	0	1	0
0	0	0	0	0	0	1	0	0	0	0	0	0	0
1	0	0	0	0	0	0	0	0	0	0	0	0	0
0	0	0	0	0	0	0	0	0	0	0	0	0	0
0	0	0	0	0	0	0	0	0	0	0	0	0	0
0	0	0	0	0	1	0	0	0	0	0	5	0	0
0	0	0	1	0	0	0	0	0	0	0	0	2	0
0	0	0	0	0	0	0	0	0	1	2	0	0	0
0													
OTU2365	0	0	1	0	1	1	0	0	0	0	1	0	1
0	1	0	1	1	0	0	1	1	3	4	1	0	0
1	0	2	0	0	0	2	1	0	0	0	1	0	0

0 0 0 0 0 1 1 1 1 0 2 0 2 0
2 2 2 0 0 0 2 0 0 1 0 0 0 5
1 0 0 3 1 0 1 0 0 0 0 0 0 0
0 0 0 0 1 0 0 0 0 0 1 0 0 1
0 0 0 0 0 0 0 3 0 0 0 0 1 0
0
OTU12312 0 0 1 0 0 0 0 0 0 0 0 0 0
0 0 0 0 0 0 0 0 0 0 0 0 0 0
0 0 0 0 0 0 0 0 0 0 0 0 0 0
0 0 0 0 0 0 0 0 0 0 0 0 0 0
0 0 0 0 0 0 0 0 0 0 0 0 0 0
0 0 0 0 0 0 0 0 0 0 0 0 0 0
0 0 0 0 0 0 0 0 0 0 0 0 0 0
0 0 0 0 0 0 0 0 0 0 0 0 0 0
0
OTU14615 0 0 1 0 0 0 0 0 0 0 0 0 0
0 0 0 0 0 0 0 0 0 0 0 0 0 0
0 0 0 0 0 0 0 0 0 0 0 0 0 0
0 0 0 0 0 0 0 0 0 0 0 0 0 0
0 0 0 0 0 0 0 0 0 0 0 0 0 0
0 0 0 0 0 0 0 0 0 0 0 0 0 0
0 0 0 0 0 0 0 0 0 0 0 0 0 0
0 0 0 0 0 0 0 0 0 0 0 0 0 0
0
OTU8531 0 0 1 0 0 0 0 0 0 0 0 0 0
0 0 0 0 0 0 0 0 0 0 0 0 0 0
0 0 0 0 0 0 0 0 0 0 0 0 0 0
0 0 0 0 0 0 0 0 0 0 0 0 0 0
0 0 0 0 0 0 0 0 0 0 0 0 0 0
0 0 0 0 0 0 0 0 0 0 0 0 0 0
0 0 0 0 0 0 0 0 0 0 0 0 0 0
0 0 0 0 0 0 0 0 0 0 0 0 0 0
0

OTU8963	0	0	1	0	0	0	0	0	0	0	0	0	0
0	0	0	0	0	0	0	0	0	1	0	0	0	0
0	0	0	0	0	0	0	0	0	0	0	3	0	0
0	0	0	0	0	0	0	0	0	0	0	0	0	0
0	0	0	0	0	0	0	0	0	0	0	0	0	0
0	0	0	0	0	0	0	0	0	0	0	0	0	0
0	0	0	0	0	0	0	0	0	0	0	0	0	0
0	0	0	0	0	2	0	0	0	0	0	0	0	0
0													
OTU2939	0	0	1	0	2	0	0	1	0	1	1	1	0
0	0	2	1	0	1	0	1	1	1	0	0	0	0
1	0	2	0	2	1	2	2	0	0	0	1	1	0
1	1	0	1	0	0	0	0	1	2	0	1	0	0
1	1	0	0	0	0	0	0	1	1	1	1	0	1
1	2	0	0	2	1	1	0	1	0	0	3	1	1
0	0	1	0	1	1	0	0	0	1	0	0	0	0
0	1	0	2	0	0	1	0	0	1	1	0	1	1
3													
OTU864	0	0	1	0	0	0	1	0	0	0	0	0	0
0	0	0	0	0	0	0	0	0	0	0	0	0	0
0	0	0	0	0	0	0	0	0	0	0	0	0	0
0	0	0	0	0	1	1	0	0	0	0	0	0	0
0	2	0	0	0	0	0	0	0	0	0	0	1	0
0	0	0	0	0	0	0	0	0	1	0	0	0	0
0	0	0	0	0	1	0	0	0	0	0	0	3	0
0	0	1	1	3	0	0	1	0	0	0	0	0	0
0													
OTU3063	0	0	1	0	0	0	0	0	0	0	0	0	0
0	0	0	0	0	0	0	0	0	0	0	0	0	0
0	0	0	0	0	0	0	0	0	0	0	0	0	0
1	0	0	0	0	2	0	0	0	0	0	0	0	0
0	0	0	0	0	0	0	0	0	0	0	0	0	0
0	1	0	0	0	0	0	0	0	0	0	0	0	0

0	0	0	0	0	0	0	0	0	0	0	0	0	0
0	3	4	0	8	1	1	0	2	1	0	0	0	0
0													
OTU7571	0	0	1	0	3	0	0	1	1	3	0	0	0
0	2	2	0	2	1	1	0	0	0	0	1	1	0
0	0	3	0	2	0	0	0	1	0	0	2	2	1
2	1	1	0	0	0	0	2	1	1	0	0	0	0
1	0	0	3	0	0	0	1	0	1	0	0	0	1
0	3	0	1	0	0	0	0	0	0	0	4	1	0
0	0	0	1	2	1	0	0	1	1	0	1	1	0
0	1	0	1	0	1	0	2	1	0	1	0	0	0
4													
OTU14613	0	0	1	0	0	0	0	0	0	0	0	0	0
0	0	0	0	0	0	0	0	0	0	0	0	0	0
0	0	0	0	0	0	0	0	0	0	0	0	0	0
0	0	0	0	0	0	0	0	0	0	0	0	0	0
0	0	0	0	0	0	0	0	0	0	0	0	0	0
0	0	0	0	0	0	0	0	0	0	0	0	0	0
0	0	0	0	0	0	0	0	0	0	0	0	0	0
0	0	0	0	0	0	0	0	0	0	0	0	0	0
0													
OTU5245	0	0	1	0	0	0	0	0	0	0	0	0	0
0	0	0	0	0	0	1	0	0	0	0	0	0	0
0	0	0	0	0	0	0	0	0	0	0	0	0	0
1	0	0	0	0	0	0	0	0	0	0	0	0	0
0	0	0	0	0	0	0	0	0	0	0	0	0	0
0	0	2	0	0	0	0	0	0	0	2	0	0	0
0	0	0	2	0	0	0	0	0	0	0	0	0	0
0	0	0	0	0	0	0	0	0	0	0	0	0	0
0													
OTU14462	0	0	1	0	1	0	1	1	0	0	1	1	0
1	0	1	0	0	0	0	0	1	1	0	0	1	1
0	0	2	0	1	1	0	0	0	0	1	2	0	0

0	0	2	1	1	0	0	1	0	1	0	1	0	0
0	0	1	0	0	1	0	1	0	1	0	0	2	0
0	0	0	0	0	0	1	2	0	0	0	3	0	0
0	1	0	0	0	1	0	1	0	0	1	0	0	0
1	0	0	0	0	0	0	1	0	0	0	0	0	0
3													
OTU985	0	0	1	3	3	4	3	4	11	6	0	0	0
0	0	0	0	0	0	8	0	3	0	0	0	1	0
3	2	0	0	0	0	1	0	0	0	2	0	18	0
7	0	0	0	0	0	0	3	0	1	0	2	0	0
0	0	0	21	0	0	0	0	0	2	0	0	12	0
3	10	6	0	0	0	0	2	0	3	0	0	0	0
0	1	0	20	0	1	0	2	0	0	0	0	1	0
0	0	7	9	3	1	0	3	0	1	0	0	2	1
1													
OTU10754	0	0	1	0	0	0	0	0	0	0	0	0	0
0	0	0	0	0	0	0	0	0	0	0	0	0	0
0	0	0	0	0	0	0	0	0	0	0	0	0	0
0	0	0	0	0	0	0	0	0	0	0	0	0	0
0	0	0	0	0	0	0	0	0	0	0	0	0	0
0	0	0	0	0	0	0	0	0	0	0	0	0	0
0	0	0	0	0	0	0	0	0	0	0	0	0	0
0	0	0	0	0	0	0	0	0	0	0	0	0	0
0													
OTU12313	0	0	1	0	0	0	0	0	0	0	0	0	0
0	0	0	0	0	0	0	0	0	0	0	0	0	0
0	0	0	0	0	0	0	0	0	0	0	0	0	0
0	0	0	0	0	0	0	0	0	0	0	0	0	0
0	0	0	0	0	0	0	0	0	0	0	0	0	0
0	0	0	0	0	0	0	0	0	0	0	0	0	0
0	0	0	0	0	0	0	0	0	0	0	0	0	0
0	0	0	0	0	0	0	0	0	0	0	0	0	0
0													

OTU12315	0	0	1	0	0	0	0	0	0	0	0	0	0
0	0	0	0	0	0	0	0	0	0	0	0	0	0
0	0	0	0	0	0	0	0	0	0	0	0	0	0
0	0	0	0	0	0	0	0	0	0	0	0	0	0
0	0	0	0	0	0	0	0	0	0	0	0	0	0
0	0	0	0	0	0	0	0	0	0	0	0	0	0
0	0	0	0	0	0	0	0	0	0	0	0	0	0
0	0	0	0	0	0	0	0	0	0	0	0	0	0
0													
OTU12314	0	0	1	0	0	0	0	0	0	0	0	0	0
0	0	0	0	0	0	0	0	0	0	0	0	0	0
0	0	0	0	0	0	0	0	0	0	0	0	0	0
0	0	0	0	0	0	0	0	0	0	0	0	0	0
0	0	0	0	0	0	0	0	0	0	0	0	0	0
0	0	0	0	0	0	0	0	0	0	0	0	0	0
0	0	0	0	0	0	0	0	0	0	0	0	0	0
0	0	0	0	0	0	0	0	0	0	0	0	0	0
0													
OTU10752	0	0	1	0	0	0	0	0	0	0	0	0	0
0	0	0	0	0	0	0	0	0	0	0	0	0	0
0	0	0	0	0	0	0	0	0	0	0	0	0	0
0	0	0	0	0	0	0	0	0	0	0	0	0	0
0	0	0	0	0	0	0	0	0	0	0	0	0	0
0	0	0	0	0	0	0	0	0	0	0	0	0	0
0	0	0	0	0	0	0	0	0	0	0	0	0	0
0	0	0	0	0	0	0	0	0	0	0	0	0	0
0													
OTU10753	0	0	1	0	0	0	0	0	0	0	0	0	0
0	0	0	0	0	0	0	0	0	0	0	0	0	0
0	0	0	0	0	0	0	0	0	0	0	0	0	0
0	0	0	0	0	0	0	0	0	0	0	0	0	0
0	0	0	0	0	0	0	0	0	0	0	0	0	0
0	0	0	0	0	0	0	0	0	0	0	0	0	0

0	0	0	0	0	0	0	0	0	0	0	0	0	0
0	0	0	0	0	0	0	0	0	0	0	0	0	0
0													
OTU9678	0	0	1	0	0	0	0	0	0	0	0	0	0
0	0	0	0	0	0	0	0	0	0	0	0	0	1
0	0	0	0	0	0	1	1	0	0	0	0	0	0
0	0	0	0	0	0	0	0	0	0	0	0	0	0
0	0	0	0	0	0	0	0	0	0	0	2	0	0
0	0	0	0	0	0	0	0	1	0	0	0	1	0
0	0	0	0	0	0	0	0	0	0	0	0	0	1
0	0	0	0	0	0	0	0	0	0	0	0	0	0
0													
OTU11460	0	0	1	0	0	0	0	0	3	0	1	0	1
0	0	0	0	0	0	0	36	0	0	0	0	0	0
16	0	0	0	0	0	0	1	0	12	1	0	0	0
0	1	0	1	0	0	0	0	0	0	0	0	0	1
0	10	0	0	0	0	0	0	2	0	0	0	1	0
0	1	1	1	0	1	0	1	0	0	1	0	0	0
1	0	0	3	0	0	0	1	0	0	2	2	1	0
0	1	2	0	5	2	3	7	2	2	0	0	0	2
0													
OTU12321	0	0	1	0	0	0	0	0	0	0	0	0	0
0	0	0	0	0	0	0	0	0	0	0	0	0	0
0	0	0	0	0	0	0	0	0	0	0	0	0	0
0	0	0	0	0	0	0	0	0	0	0	0	0	0
0	0	0	0	0	0	0	0	0	0	0	0	0	0
0	0	0	0	0	0	0	0	0	0	0	0	0	0
0	0	0	0	0	0	0	0	0	0	0	0	0	0
0	0	0	0	0	0	0	0	0	0	0	0	0	0
0													
OTU8544	0	0	2	0	0	0	0	0	0	0	0	0	0
0	0	0	0	0	0	0	0	0	0	0	0	0	0
0	0	0	0	0	0	0	0	0	0	0	0	0	0

0	0	0	0	0	0	0	0	0	0	0	0	0	0
0	0	0	0	0	0	0	0	0	0	0	0	0	0
0	0	0	0	0	0	0	0	0	0	0	0	0	0
0	0	0	0	0	0	0	0	0	0	0	0	0	0
0	0	0	0	0	0	0	0	0	0	0	0	0	0
0													
OTU384	0	0	1	0	0	0	0	0	0	0	4	0	0
0	0	0	0	0	1	7	0	0	0	0	0	0	64
6	0	0	0	0	0	0	0	0	0	0	0	0	0
13	0	0	0	0	0	0	0	0	0	0	0	0	0
0	0	0	0	0	0	0	0	0	0	0	0	0	0
0	0	0	0	0	0	0	0	0	0	0	0	0	0
4	0	0	0	3	2	0	0	0	0	0	0	0	0
0	0	0	0	0	0	0	0	0	0	0	0	0	0
0													
OTU8559	0	0	1	0	0	0	0	0	0	0	0	0	0
0	0	0	0	0	0	0	0	0	0	0	0	0	0
0	0	0	0	0	0	0	0	0	0	0	0	0	0
0	0	0	0	0	0	0	0	0	0	0	0	0	0
0	0	0	0	0	0	0	0	0	0	0	0	0	0
0	0	0	0	0	0	0	0	0	0	0	0	0	0
0	0	0	0	0	0	0	0	0	0	0	0	0	0
0	0	0	0	0	0	0	0	0	0	0	0	0	0
0													
OTU7501	0	0	1	0	0	0	0	0	0	1	0	0	0
0	0	0	0	1	0	0	0	0	0	0	0	0	0
0	0	0	0	0	0	0	1	0	0	1	0	0	1
0	2	0	1	0	1	0	0	0	0	0	0	0	1
1	0	0	0	0	0	0	0	0	1	1	0	0	0
1	0	0	0	0	0	0	0	0	0	1	2	0	1
0	0	0	0	0	0	0	0	0	0	0	0	0	0
1	1	1	0	0	1	0	0	0	0	1	0	0	0
1													

OTU14614 0 0 1 0 0 0 0 0 0 0 0 0 0
0 0 0 0 0 0 0 0 0 0 0 0 0 0
0 0 0 0 0 0 0 0 0 0 0 0 0 0
0 0 0 0 0 0 0 0 0 0 0 0 0 0
0 0 0 0 0 0 0 0 0 0 0 0 0 0
0 0 0 0 0 0 0 0 0 0 0 0 0 0
0 0 0 0 0 0 0 0 0 0 0 0 0 0
0 0 0 0 0 0 0 0 0 0 0 0 0 0
0
OTU2843 0 0 7 0 0 0 0 0 1 0 0 0 0
0 0 0 0 0 0 0 0 0 0 0 0 0 0
0 0 0 1 0 0 0 0 0 0 0 2 0 0
0 0 1 0 1 0 0 0 0 0 0 0 0 0
0 0 0 0 0 0 0 0 0 0 0 0 0 0
0 0 0 0 0 0 0 0 0 0 0 0 0 0
0 0 0 0 0 0 0 0 0 0 0 0 0 0
0 0 0 0 0 0 0 0 0 1 0 0 0 0
0
OTU8562 0 0 1 0 0 0 0 0 0 0 0 0 0
0 0 0 0 0 0 0 0 0 0 0 0 0 0
0 0 0 0 0 0 0 0 0 0 0 0 0 0
0 0 0 0 0 0 0 0 0 0 0 0 0 0
0 0 0 0 0 0 0 0 0 0 0 0 0 0
0 0 0 0 0 0 0 0 0 0 0 0 0 0
0 0 0 0 0 0 0 0 0 0 0 0 0 0
0 0 0 0 0 0 0 0 0 0 0 0 0 0
0
OTU13633 0 0 1 0 0 0 0 0 0 0 0 2 0
0 0 7 0 8 0 0 8 0 0 2 0 0 1
0 0 0 0 0 0 0 4 0 19 0 1 0 0
0 1 0 11 0 0 0 0 0 0 0 0 0 0
0 0 0 0 0 0 0 0 0 0 0 0 0 0
0 1 0 3 0 0 2 0 0 0 0 0 15 0

0	0	0	0	1	0	0	0	0	19	4	1	0	1
3	0	0	0	1	0	0	0	0	0	1	1	7	0
0													
OTU8560	0	0	1	0	0	0	0	0	0	0	0	0	0
0	0	0	0	0	0	0	0	0	0	0	0	0	0
0	0	0	0	0	0	0	0	0	0	0	0	0	0
0	0	0	0	0	0	0	0	0	0	0	0	0	0
0	0	0	0	0	0	0	0	0	0	0	0	0	0
0	0	0	0	0	0	0	0	0	0	0	0	0	0
0	0	0	0	0	0	0	0	0	0	0	0	0	0
0	0	0	0	0	0	0	0	0	0	0	0	0	0
0													
OTU8561	0	0	1	0	0	0	0	0	0	0	0	0	0
0	0	0	0	0	0	0	0	0	0	0	0	0	0
0	0	0	0	0	0	0	0	0	0	0	0	0	0
0	0	0	0	0	0	0	0	0	0	0	0	0	0
0	0	0	0	0	0	0	0	0	0	0	0	0	0
0	0	0	0	1	0	0	0	1	0	0	1	0	0
0	0	0	0	0	0	0	1	0	0	0	0	0	0
0	0	0	0	0	0	0	0	0	0	0	0	0	0
0													
OTU308	0	0	1	0	0	0	1	0	0	0	0	0	0
0	0	1	0	0	0	1	1	0	24	0	1	0	0
0	0	0	0	0	22	15	0	0	0	0	0	0	0
0	0	0	0	1	1	2	2	1	1	0	104	0	1
0	0	0	4	1	1	1	9	9	1	4	0	2	0
1	6	0	0	0	0	0	0	0	0	0	173	0	0
0	0	0	0	0	0	0	5	0	0	0	1	1	0
0	7	0	0	6	6	21	1	8	1	1	0	0	30
1													
OTU14007	0	0	1	0	0	0	0	0	0	0	0	0	0
0	0	0	0	0	0	0	0	0	0	0	0	0	0
0	0	0	0	0	0	0	0	0	0	0	0	0	0

0 0 0 0 0 0 0 0 0 0 0 0 0 0
0 0 0 0 0 0 0 0 1 0 0 3 0 0
0 0 0 0 0 0 0 0 0 0 0 0 0 0
0 0 0 0 0 0 0 0 0 0 0 0 0 0
0 0 0 0 0 0 0 0 0 0 0 0 0 0
0
OTU524 0 0 2 0 0 0 0 0 0 2 0 5 0
0 0 0 0 0 0 0 0 0 0 0 0 0 1
0 0 0 0 0 0 0 0 0 0 0 0 0 1
1 5 0 0 0 2 0 0 0 0 0 0 0 0
0 1 0 0 0 2 0 0 0 0 0 0 3 0
0 0 2 0 2 0 0 0 1 1 2 0 0 0
1 0 0 1 0 0 0 0 0 0 0 0 0 0
0 0 0 1 0 0 0 3 1 0 1 0 0 0
0
OTU10761 0 0 1 0 0 0 0 0 0 0 0 0 0
0 0 0 0 0 0 0 0 0 0 0 0 0 0
0 0 0 0 0 0 0 0 0 0 0 0 0 0
0 0 0 0 0 0 0 0 0 0 0 0 0 0
0 0 0 0 0 0 0 0 0 0 0 0 0 0
0 0 0 0 0 0 0 0 0 0 0 0 0 0
0 0 0 0 0 0 0 0 0 0 0 0 0 0
0 0 0 0 0 0 0 0 0 0 0 0 0 0
0
OTU1069 0 0 3 0 0 0 0 0 1 2 0 0 0
0 0 0 0 0 0 0 0 0 0 0 0 0 0
0 0 0 0 0 0 0 0 0 0 0 0 0 0
0 0 0 0 0 0 0 0 0 0 0 0 0 0
0 0 0 0 0 0 0 0 0 0 0 0 1 0
0 0 0 0 0 0 0 16 0 0 0 1 0 4
0 0 0 0 0 0 0 0 0 0 0 0 0 0
0 0 0 0 0 0 0 0 0 0 0 0 0 0
0

OTU5353	0	0	1	0	0	0	0	0	0	0	0	0	3
64	0	0	1	0	0	9	0	11	0	1	0	0	0
0	26	0	0	13	0	9	9	1	0	127	0	4	1
0	0	108	0	119	0	0	0	36	1	7	0	2	0
11	0	9	1	17	12	23	0	0	19	110	8	2	27
26	2	1	0	0	0	0	0	0	0	1	0	0	0
0	0	0	0	0	0	0	0	4	0	0	0	0	6
1	1	9	5	3	8	4	1	1	4	0	1	0	14
6													
OTU10760	0	0	1	0	0	0	0	0	0	0	0	0	0
0	0	0	0	0	0	0	0	0	0	0	0	0	0
0	0	0	0	0	0	0	0	0	0	0	0	0	0
0	0	0	0	0	0	0	0	0	0	0	0	0	0
0	0	0	0	0	0	0	0	0	0	0	0	0	0
0	0	0	0	0	0	0	0	0	0	0	0	0	0
0	0	0	0	0	0	0	0	0	0	0	0	0	0
0	0	0	0	0	0	0	0	0	0	0	0	0	0
0													
OTU14559	0	0	1	0	0	0	0	0	0	0	0	0	0
0	0	0	0	0	0	0	0	0	0	0	0	0	0
0	0	0	0	0	0	0	0	0	0	0	0	0	0
0	0	0	0	0	0	0	0	0	0	0	0	0	0
0	0	0	0	0	0	0	0	0	0	0	0	0	0
0	0	0	0	0	0	0	0	0	0	0	0	0	0
0	0	0	0	0	0	0	0	0	0	0	0	0	0
0	0	0	0	0	0	0	0	0	0	0	0	0	0
0													
OTU10756	0	0	1	0	0	0	0	0	0	0	0	0	0
0	0	0	0	0	0	0	0	0	0	0	0	0	0
0	0	0	0	0	0	0	0	0	0	0	0	0	0
0	0	0	0	0	0	0	0	0	0	0	0	0	0
0	0	0	0	0	0	0	0	0	0	0	0	0	0
0	0	0	0	0	0	0	0	0	0	0	0	0	0

0	0	0	0	0	0	0	0	0	0	0	0	0	0
0	0	0	0	0	0	0	0	0	0	0	0	0	0
0													
OTU2655	0	0	1	0	0	0	0	0	0	0	0	0	0
0	0	0	0	0	0	0	0	1	0	0	0	0	0
0	0	0	0	0	0	0	0	0	0	0	0	0	0
0	0	0	0	0	0	0	0	0	0	0	0	0	0
0	0	0	0	0	0	0	0	0	0	0	0	1	0
0	0	2	0	0	0	0	0	0	0	0	0	0	0
0	0	0	0	0	0	0	2	0	0	0	0	0	0
0	0	0	0	0	0	0	0	0	0	0	0	0	0
0													
OTU3269	0	0	1	0	1	0	0	0	0	0	0	0	0
0	1	0	0	0	0	0	0	0	0	0	0	0	1
0	0	0	0	0	0	0	0	0	0	0	0	0	0
0	0	1	0	0	0	0	0	0	0	0	0	0	0
0	0	0	0	0	0	0	0	0	1	1	0	0	0
0	0	0	0	1	0	0	0	0	0	0	0	0	0
0	0	0	0	0	1	0	0	0	0	0	0	1	0
0	1	0	0	1	0	0	0	0	0	0	0	0	0
0													
OTU861	0	0	1	0	0	0	0	0	1	0	0	0	0
0	1	0	0	0	0	0	0	0	0	0	0	0	0
0	0	0	0	0	0	0	0	0	0	0	0	0	0
0	0	0	0	0	0	0	0	0	0	1	0	0	0
0	1	0	0	0	0	0	0	0	0	0	0	0	0
0	0	0	0	0	0	0	0	0	0	0	0	0	0
0	0	0	0	0	0	0	0	0	0	0	0	0	0
0	0	0	0	0	0	0	0	0	0	0	0	0	0
0													
OTU14558	0	0	1	0	0	0	0	0	0	0	0	0	0
0	0	0	0	0	0	0	0	0	0	0	0	0	0
0	0	0	0	0	0	0	0	0	0	0	0	0	0

0	0	0	0	0	0	0	0	0	0	0	0	0	0
0	0	0	0	0	0	0	0	0	0	0	0	0	0
0	0	0	0	0	0	0	0	0	0	0	0	0	0
0	0	0	0	0	0	0	0	0	0	0	0	0	0
0	0	0	0	0	0	0	0	0	0	0	0	0	0
0													
OTU16253	0	0	1	0	1	2	2	4	3	1	3	1	0
1	2	1	1	2	1	0	0	1	1	1	1	0	2
1	1	1	0	1	1	0	1	0	0	1	4	5	0
3	2	1	1	1	2	3	1	1	3	0	0	1	0
1	2	0	1	1	1	1	2	0	2	1	0	0	0
1	0	0	0	2	0	2	2	2	0	0	6	0	1
1	1	0	3	0	5	0	0	1	0	0	1	1	0
0	3	1	1	1	2	0	0	3	1	1	0	0	1
0													
OTU6286	0	0	1	0	1	0	0	0	0	0	0	0	0
0	1	2	0	0	0	0	0	0	1	0	1	0	0
0	0	1	0	0	3	0	1	0	0	0	1	0	0
0	0	0	0	0	0	0	0	0	0	1	0	0	0
1	0	0	0	1	0	0	0	0	0	0	0	0	2
1	0	0	0	0	0	0	0	0	0	0	0	0	0
0	0	0	1	1	0	0	0	0	0	0	0	0	0
0	0	0	0	0	0	0	0	0	0	0	0	0	0
0													
OTU7896	0	0	4	0	0	0	0	2	2	1	0	0	0
0	1	0	0	0	0	1	0	0	0	0	0	0	0
0	0	0	0	0	0	0	0	0	0	0	0	0	0
0	0	0	0	0	0	0	1	0	0	0	0	0	0
0	0	0	0	0	0	0	0	0	0	0	0	0	0
0	0	0	0	0	0	0	0	0	0	0	0	0	0
0	0	0	0	0	3	0	0	0	0	0	0	0	0
0	0	0	0	0	0	0	0	0	0	0	0	0	0
0													

OTU8564	0	0	2	0	1	0	0	0	0	2	0	0	0
0	3	0	0	0	1	7	3	0	0	0	0	1	0
1	0	0	0	0	1	0	0	1	0	1	0	0	0
1	1	0	0	0	0	0	0	0	0	1	0	0	0
0	1	0	1	0	0	1	0	0	0	0	0	3	0
0	0	0	0	0	0	0	0	2	0	0	1	0	0
0	0	0	1	0	2	0	0	0	0	3	1	2	2
2	1	0	0	0	1	0	0	0	1	0	1	1	1
0													
OTU14569	0	0	3	0	0	0	0	0	0	0	0	0	0
0	0	0	0	0	0	0	0	0	0	0	0	0	0
0	0	0	0	0	0	0	0	0	0	0	0	0	0
0	0	0	0	1	0	0	0	0	0	0	0	0	0
0	0	0	0	0	0	0	0	0	0	0	0	0	0
0	0	0	0	0	0	0	0	0	0	0	0	0	0
0	0	0	0	0	0	0	0	0	0	0	0	0	0
0	0	0	0	0	0	0	0	0	0	0	0	0	0
0													
OTU10758	0	0	2	0	0	0	0	0	0	0	0	0	0
0	0	0	0	0	0	0	0	0	0	0	0	0	0
0	0	0	0	0	0	0	0	0	0	0	0	0	0
0	0	0	0	0	0	0	0	0	0	0	0	0	0
0	0	0	0	0	0	0	0	0	0	0	0	0	0
0	0	0	0	0	0	0	0	0	0	0	0	0	0
0	0	0	0	0	0	0	0	0	0	0	0	0	0
0	0	0	0	0	0	0	0	0	0	0	0	0	0
0													
OTU141	0	0	2	0	0	0	0	0	0	11	0	0	0
0	0	0	0	0	0	0	0	0	0	0	0	0	0
0	0	0	0	0	0	0	0	1	0	0	0	0	0
0	0	0	0	0	0	0	1	0	0	0	0	0	0
0	0	0	0	0	0	1	0	1	0	0	0	0	0
0	228	0	0	0	0	0	0	0	0	0	0	0	3

0 0 0 16 0 0 0 0 0 0 0 107 107 0
0 64 3 7 46 2 0 128 1 0 0 5 0 0
17
OTU7673 0 0 1 0 0 0 0 0 0 0 0 0 0
0 0 0 0 0 0 0 0 0 0 0 0 0 0
0 0 0 0 0 0 0 0 0 0 0 0 0 0
1 0 0 0 0 0 0 0 0 0 0 0 0 0
0 0 0 0 0 0 0 0 0 0 0 0 0 0
0 0 0 0 0 0 0 0 0 0 0 0 0 0
0 0 0 0 2 0 0 1 0 1 0 1 0 0
0 1 0 4 0 4 2 1 2 4 0 0 5 3
2
OTU10757 0 0 1 0 0 0 0 0 0 0 0 0 0
0 0 0 0 0 0 0 0 0 0 0 0 0 0
0 0 0 0 0 0 0 0 0 0 0 0 0 0
0 0 0 0 0 0 0 0 0 0 0 0 0 0
0 0 0 0 0 0 0 0 0 0 0 0 0 0
0 0 0 0 0 0 0 0 0 0 0 0 0 0
0 0 0 0 0 0 0 0 0 0 0 0 0 0
0 0 0 0 0 0 0 0 0 0 0 0 0 0
0
OTU987 0 0 2 0 0 0 0 0 0 0 0 0 0
0 0 0 0 0 0 0 0 0 0 0 0 0 0
0 0 0 0 0 0 0 0 0 0 0 0 0 0
0 0 0 0 0 0 0 0 0 0 0 0 0 0
0 0 0 0 0 0 0 0 0 0 0 0 0 0
0 0 0 0 0 0 0 0 0 0 0 0 0 0
0 0 0 0 0 0 0 0 0 0 0 0 0 0
0 0 0 0 0 0 0 0 0 0 0 0 0 0
0
OTU1084 0 0 1 0 0 0 0 0 0 0 0 0 0
0 0 0 0 0 0 0 0 0 0 0 0 0 0
0 0 0 0 0 0 0 0 0 0 0 0 0 0

0 0 0 0 2 0 0 0 0 0 0 0 0 0
0 0 0 0 0 0 0 0 0 0 0 0 0 0
0 0 0 0 0 0 0 0 0 0 0 0 0 0
0 0 0 0 0 0 0 0 0 0 0 0 0 0
0 0 0 0 0 0 0 0 0 0 0 0 0 0
0
OTU13701 0 0 1 0 0 0 0 0 0 0 0 0 0
0 0 1 0 0 0 0 0 0 0 0 0 0 0
0 0 0 0 0 0 0 2 0 0 0 0 0 0
0 0 0 0 0 0 0 0 0 0 0 0 0 0
0 0 0 0 0 0 0 0 0 0 0 0 0 0
0 0 0 0 0 0 0 0 0 0 0 0 0 0
0 0 0 0 0 0 0 0 0 0 2 0 0 1
0 0 0 0 0 0 0 0 0 0 0 0 0 0
0
OTU563 0 0 1 2 0 1 0 1 2 2 0 0 0
0 0 1 0 0 0 1 2 0 0 0 1 0 0
0 0 0 0 0 0 0 0 0 1 0 0 1 0
0 0 0 0 0 1 0 1 0 0 0 0 0 0
0 2 0 1 1 0 0 0 0 0 0 0 1 0
0 1 5 0 3 0 0 0 0 1 0 0 0 1
1 1 0 0 0 0 0 3 0 0 0 3 1 0
0 5 2 1 0 2 1 0 0 6 0 0 0 1
1
OTU9139 0 0 1 0 1 0 0 0 0 0 0 0 1
0 0 0 0 1 0 0 0 0 1 0 0 0 0
0 1 0 0 0 0 0 0 0 0 0 1 2 0
0 0 0 0 0 1 0 0 0 0 0 0 0 0
0 0 0 0 0 0 0 0 0 0 0 0 0 0
0 0 0 0 1 0 0 0 0 0 0 0 0 0
0 0 0 0 0 1 0 0 0 1 0 0 0 0
0 0 0 0 0 0 0 0 0 0 0 0 0 0
0

OTU8563	0	0	1	0	0	0	0	0	0	0	0	0	0
0	0	0	0	0	0	0	0	0	0	0	0	0	0
0	0	0	0	0	0	0	0	0	0	0	0	0	0
0	0	0	0	0	0	0	0	0	0	0	0	0	0
0	0	0	0	0	0	0	0	0	0	0	0	0	0
0	0	0	0	0	0	0	0	0	0	0	0	0	0
0	0	0	0	0	0	0	0	0	0	0	0	0	0
0	0	0	0	0	0	0	0	0	0	0	0	0	0
0													
OTU10782	0	0	1	0	0	1	0	0	0	0	0	1	1
0	0	0	0	0	0	1	0	0	0	0	0	0	0
1	0	0	0	0	0	0	1	0	1	0	0	1	0
1	1	0	0	0	0	0	0	0	0	1	1	0	0
0	0	0	0	0	0	0	0	0	0	0	1	0	0
0	0	0	1	0	1	1	0	6	0	0	0	0	0
0	0	0	0	0	1	1	0	0	0	0	0	0	0
1	0	0	0	0	0	0	0	0	0	0	2	0	1
0													
OTU5677	0	0	1	0	0	0	0	0	0	0	0	0	0
0	0	0	0	0	0	0	1	0	0	0	0	1	0
3	0	0	0	0	0	0	0	0	0	0	0	0	0
1	0	0	2	0	0	0	0	2	0	2	26	0	0
0	0	0	0	1	1	8	0	6	0	0	0	0	0
0	0	0	1	0	1	3	9	1	0	0	1	3	1
0	0	0	0	1	0	3	0	0	1	0	0	0	0
0	0	0	0	0	0	0	0	0	0	4	0	0	0
0													
OTU949	0	0	2	0	0	0	2	0	0	0	0	0	0
0	0	0	0	0	0	0	0	1	0	0	0	0	0
0	0	0	0	0	0	0	0	0	0	0	0	0	0
0	0	0	0	0	0	0	0	0	0	0	0	0	0
0	0	0	2	0	0	0	0	0	0	0	0	0	0
0	1	0	0	0	0	0	0	0	0	0	0	0	4

0	0	0	0	0	0	0	0	0	0	0	0	0	0
0	1	0	0	1	0	0	0	0	0	0	0	0	0
0													
OTU12465	0	0	1	0	5	1	31	16	34	31	0	0	0
0	3	0	0	0	0	3	0	0	0	0	0	1	0
10	0	0	0	0	0	0	0	3	0	1	0	0	0
17	0	0	0	0	1	0	1	1	0	0	0	0	0
0	1	0	1	0	0	0	1	2	0	0	0	14	0
1	1	4	0	18	0	0	1	0	18	1	7	0	13
0	2	0	50	0	4	0	10	0	0	1	4	20	0
0	1	3	5	1	1	1	9	2	14	0	0	0	4
8													
OTU1523	0	0	1	0	0	0	0	1	0	0	0	0	0
0	0	0	2	0	0	0	0	2	0	0	0	0	0
0	3	0	0	0	0	5	0	0	0	0	0	3	0
0	1	0	0	0	1	0	0	0	0	0	1	0	0
0	1	0	1	0	0	1	0	0	1	0	0	1	0
0	5	0	0	0	0	0	3	0	0	0	1	0	17
0	0	0	0	1	1	0	2	0	0	0	2	10	0
0	8	23	14	13	2	5	15	10	13	0	1	2	12
6													
OTU10784	0	0	1	0	0	0	0	0	0	0	0	0	0
0	0	0	0	0	0	0	0	0	0	0	0	0	0
0	0	0	0	0	0	0	0	0	0	0	0	0	0
0	0	0	0	0	0	0	0	0	0	0	0	0	0
0	0	0	0	0	0	0	0	0	0	0	0	0	0
0	0	0	0	0	0	0	0	0	0	0	0	0	0
0	0	0	0	0	0	0	0	0	0	0	0	0	0
0	0	0	0	0	0	0	0	0	0	0	0	0	0
0													
OTU4039	0	0	1	0	0	0	0	0	0	0	0	0	0
1	1	0	0	0	0	0	0	0	0	0	1	1	0
0	0	1	0	1	0	2	1	0	0	0	0	0	0

0	0	0	0	0	0	2	0	1	3	0	0	0	0
0	1	0	0	0	0	0	0	0	0	0	0	0	0
1	0	0	1	0	1	0	0	1	0	0	0	0	0
0	0	0	1	0	0	0	0	0	0	0	0	0	0
0	0	0	0	0	0	0	0	0	0	0	1	0	0
1													
OTU4277	0	0	1	0	0	0	0	0	0	1	0	0	0
0	0	0	0	0	0	0	0	0	0	0	0	0	0
0	0	0	0	0	0	0	0	0	0	0	0	0	0
0	0	0	0	0	0	0	0	0	0	0	0	0	0
0	0	0	0	0	0	0	0	0	0	0	0	0	0
0	0	0	0	0	0	0	0	0	0	0	0	0	0
0	0	0	0	0	0	0	0	0	0	0	0	0	0
0	0	0	0	0	0	0	0	0	0	0	0	0	0
0													
OTU11981	0	0	5	0	0	4	0	0	0	0	0	0	1
0	0	4	0	0	0	0	0	0	1	0	1	0	0
0	0	0	0	0	0	0	0	0	3	0	0	0	1
0	1	0	0	0	0	0	0	1	0	0	0	0	0
0	0	0	0	0	0	0	0	0	0	0	1	1	0
1	0	1	0	0	0	0	0	0	0	0	1	0	0
0	0	0	1	0	0	0	0	0	1	0	0	0	0
0	0	0	0	0	0	0	0	0	0	1	0	0	0
0													
OTU12306	0	0	1	0	0	0	0	0	0	0	0	0	0
0	0	0	0	0	0	0	0	0	0	0	0	0	0
0	0	0	0	0	0	0	0	0	0	0	0	0	0
0	0	0	0	0	0	0	0	0	0	0	0	0	0
0	0	0	0	0	0	0	0	0	0	0	0	0	0
0	0	0	0	0	0	0	0	0	0	0	0	0	0
0	0	0	0	0	0	0	0	0	0	0	0	0	0
0	0	0	0	0	0	0	0	0	0	0	0	0	0
0													

OTU10785	0	0	1	0	0	0	0	0	0	0	0	0	0
0	0	0	0	0	0	0	0	0	0	0	0	0	0
0	0	0	0	0	0	0	0	0	0	0	0	0	0
0	0	0	0	0	0	0	0	0	0	0	0	0	0
0	0	0	0	0	0	0	0	0	0	0	0	0	0
0	0	0	0	0	0	0	0	0	0	0	0	0	0
0	0	0	0	0	0	0	0	0	0	0	0	0	0
0	0	0	0	0	0	0	0	0	0	0	0	0	0
0													
OTU8545	0	0	2	0	0	0	0	0	0	0	0	0	0
0	0	0	0	0	0	0	0	0	0	0	0	0	0
0	0	0	0	0	0	0	0	0	0	0	0	0	0
0	0	1	0	0	0	0	0	0	0	0	0	0	0
0	0	0	0	0	0	0	0	0	0	0	0	0	0
0	0	0	0	0	0	0	0	0	0	0	0	0	0
0	0	0	0	0	0	0	0	0	0	0	0	0	0
0	0	0	0	0	1	0	0	0	0	0	0	0	0
0													
OTU8548	0	0	1	0	0	0	0	0	0	0	0	0	0
0	0	0	0	0	0	0	0	0	0	0	0	0	0
0	0	0	0	0	0	0	0	0	0	0	0	0	0
0	0	0	0	0	0	0	0	0	0	0	0	0	0
0	0	0	0	0	0	0	0	0	0	0	0	0	0
0	0	0	0	0	0	0	0	0	0	0	0	0	0
0	0	0	0	0	0	0	0	0	0	0	0	0	0
0	0	0	0	0	0	0	0	0	0	0	0	0	0
0													
OTU14565	0	0	1	0	0	0	0	0	0	0	0	0	0
0	0	0	0	0	0	0	0	0	0	0	0	0	0
0	0	0	0	0	0	0	0	0	0	0	0	0	0
0	0	0	0	0	0	0	0	0	0	0	0	0	0
0	0	0	0	0	0	0	0	0	0	0	0	0	0
0	0	0	0	0	0	0	0	0	0	0	0	0	0

0	0	0	0	0	0	0	0	0	0	0	0	0	0
0	0	0	0	0	0	0	0	0	0	0	0	0	0
0													
OTU14564	0	0	1	0	0	0	0	0	0	0	0	0	0
0	0	0	0	0	0	0	0	0	0	0	0	0	0
0	0	0	0	0	0	0	0	0	0	0	0	0	0
0	0	0	0	0	0	0	0	0	0	0	0	0	0
0	0	0	0	0	0	0	0	0	0	0	0	0	0
0	0	0	0	0	0	0	0	0	0	0	0	0	0
0	0	0	0	0	0	0	0	0	0	0	0	0	0
0	0	0	0	0	0	0	0	0	0	0	0	0	0
0													
OTU8546	0	0	1	0	0	0	0	0	0	0	0	0	0
0	0	0	0	0	0	0	0	0	0	0	0	0	0
0	0	0	0	0	0	0	0	0	0	0	0	0	0
0	0	0	0	0	0	0	0	0	0	0	0	0	0
0	0	0	0	0	0	0	0	0	0	0	0	0	0
0	0	0	0	0	0	0	0	0	0	0	0	0	0
0	0	0	0	0	0	0	0	0	0	0	0	0	0
0	0	0	0	0	0	0	0	0	0	0	0	0	0
0													
OTU6564	0	0	1	0	0	0	0	0	0	0	0	0	2
0	0	0	0	0	0	0	0	1	0	0	0	1	0
0	1	0	0	0	0	1	0	0	0	0	0	1	0
0	0	1	1	2	0	1	0	0	0	1	0	0	0
0	0	1	0	0	0	0	2	0	0	0	1	0	3
0	0	1	0	0	0	1	1	0	0	2	0	0	0
0	2	0	0	2	0	1	0	0	0	0	0	0	0
0	0	0	0	1	0	0	0	2	0	0	0	0	0
0													
OTU754	0	0	1	0	0	0	1	0	8	0	0	0	0
0	0	0	0	0	0	0	0	0	0	0	0	0	0
0	0	0	0	2	0	1	0	0	0	0	0	0	0

0 0 0 0 5 0 0 0 0 1 0 0 0 0
0 0 1 0 2 0 0 0 0 2 0 0 0 2
0 0 0 0 0 0 0 0 0 0 0 0 0 0
0 0 0 0 0 0 0 0 0 0 0 0 0 0
0 0 0 1 0 0 0 0 0 2 0 0 0 0
0
OTU10783 0 0 1 0 0 0 0 0 0 0 0 0 0
0 0 0 0 0 0 0 0 0 0 0 0 0 0
0 0 0 0 0 0 0 0 0 0 0 0 0 0
0 0 0 0 0 0 0 0 0 0 0 0 0 0
0 0 0 0 0 0 0 0 0 0 0 0 0 0
0 0 0 0 0 0 0 0 0 0 0 0 0 0
0 0 0 0 0 0 0 0 0 0 0 0 0 0
0 0 0 0 0 0 0 0 0 0 0 0 0 0
0
OTU1029 0 0 2 0 0 0 0 0 0 0 0 0 0
0 0 0 0 0 0 0 0 0 0 0 0 0 0
0 0 0 0 0 0 0 0 0 0 0 0 0 0
0 0 0 0 0 0 0 0 0 0 0 0 0 0
0 0 0 0 0 0 0 0 0 0 0 0 0 0
0 0 0 0 0 0 0 0 0 0 0 0 0 0
0 0 0 0 0 0 0 0 0 0 0 0 0 0
0 0 0 0 0 0 0 0 0 0 0 0 0 0
0
OTU14567 0 0 1 0 0 0 0 0 0 0 0 0 0
0 0 0 0 0 0 0 0 0 0 0 0 0 0
0 0 0 0 0 0 0 0 0 0 0 0 0 0
0 0 0 0 0 0 0 0 0 0 0 0 0 0
0 0 0 0 0 0 0 0 0 0 0 0 0 0
0 0 0 0 0 0 0 0 0 0 0 0 0 0
0 0 0 0 0 0 0 0 0 0 0 0 0 0
0 0 0 0 0 0 0 0 0 0 0 0 0 0
0

OTU10780	0	0	1	0	0	0	0	0	0	0	0	0	0
0	0	0	0	0	0	0	0	0	0	0	0	0	0
0	0	0	0	0	0	0	0	0	0	0	0	0	0
0	0	0	0	0	0	0	0	0	0	0	0	0	0
0	0	0	0	0	0	0	0	0	0	0	0	0	0
0	0	0	0	0	0	0	0	0	0	0	0	0	0
0	0	0	0	0	0	0	0	0	0	0	0	0	0
0	0	0	0	0	0	0	0	0	0	0	0	0	0
0													
OTU2058	0	0	1	0	0	0	0	0	0	0	0	0	0
0	0	0	0	2	0	0	0	0	0	3	0	0	0
0	1	0	0	1	1	0	0	0	1	0	0	0	0
0	0	2	0	0	0	0	0	1	0	0	0	3	0
1	0	1	0	0	0	1	2	1	0	0	0	0	0
0	0	0	0	0	0	0	0	0	0	0	0	0	0
0	2	0	0	0	0	0	0	0	0	0	0	0	0
0	0	0	0	0	0	0	0	0	0	0	0	0	0
0													
OTU12759	0	0	1	1	0	0	0	0	0	0	0	0	0
0	0	0	0	0	0	0	0	0	0	0	0	0	1
0	0	0	0	0	0	0	1	0	0	0	0	0	1
0	0	0	0	0	0	1	3	0	0	0	0	0	0
0	0	0	0	0	0	0	0	0	0	0	0	0	0
0	1	0	0	0	0	0	0	0	0	1	0	0	0
0	0	0	0	2	0	0	0	0	0	0	0	0	0
1	0	0	0	0	0	0	0	0	0	0	0	0	0
0													
OTU3008	0	0	1	0	0	0	1	0	1	0	0	1	0
0	0	0	0	0	0	0	0	0	0	0	0	0	0
0	0	0	0	0	0	0	0	0	0	0	0	0	0
1	0	0	1	0	0	0	0	0	0	0	0	0	0
0	0	0	0	0	0	0	0	0	0	0	0	0	0
0	0	0	0	0	0	0	0	0	1	0	1	0	1

0	0	0	0	0	0	0	0	0	0	0	1	0	0
0	1	0	0	3	0	1	0	0	0	0	0	0	0
0													
OTU8549	0	0	1	0	0	0	0	0	0	0	0	0	0
0	0	0	0	0	0	0	0	0	0	0	0	0	0
0	0	0	0	0	0	0	0	0	0	0	0	0	0
0	0	0	0	0	0	0	0	0	0	0	0	0	0
0	0	0	0	0	0	0	0	0	0	0	0	0	0
0	0	0	0	0	0	0	0	0	0	0	0	0	0
0	0	0	0	0	0	0	0	0	0	0	0	0	0
0	0	0	0	0	0	0	0	0	0	0	0	0	0
0													
OTU8550	0	0	1	0	0	0	0	0	0	0	0	0	0
0	0	0	0	0	0	0	0	0	0	0	0	0	0
0	0	0	0	0	0	0	0	0	0	0	0	0	0
0	0	0	0	0	0	0	0	0	0	0	0	0	0
0	0	0	0	0	0	0	0	0	0	0	0	0	0
0	0	0	0	0	0	0	0	0	0	0	0	0	0
0	0	0	0	0	0	0	0	0	0	0	0	0	0
0	0	0	0	0	0	0	0	0	0	0	0	0	0
0													
OTU10779	0	0	1	0	0	0	0	0	0	0	0	0	0
0	0	0	0	0	0	0	0	0	0	0	0	0	0
0	0	0	0	0	0	0	0	0	0	0	0	0	0
0	0	0	0	0	0	0	0	0	0	0	0	0	0
0	0	0	0	0	0	0	0	0	0	0	0	0	0
0	0	0	0	0	0	0	0	0	0	0	0	0	0
0	0	0	0	0	0	0	0	0	0	0	0	0	0
0	0	0	0	0	0	0	0	0	0	0	0	0	0
0													
OTU3296	0	0	1	0	0	0	1	0	0	0	0	2	0
0	0	0	0	1	0	0	0	0	1	0	0	0	0
2	0	0	0	0	0	0	0	0	1	0	0	0	0

0 0 0 0 1 0 0 0 0 1 0 1 0 0
0 0 1 0 0 0 0 0 0 0 0 0 0 0
0 0 0 0 0 0 0 0 0 0 0 0 0 1
0 0 0 0 0 1 0 1 0 0 0 0 0 0
0 1 0 0 0 0 0 0 0 0 1 0 0 0
0
OTU10605 0 0 1 0 0 0 0 0 0 0 0 0 0
0 0 0 0 0 0 0 0 0 0 0 0 0 0
0 0 0 0 0 0 0 0 0 0 0 0 0 0
0 0 1 0 0 0 0 0 0 0 0 0 0 0
0 0 0 0 0 0 0 0 0 0 0 0 0 0
0 0 0 0 1 0 0 1 0 0 0 0 0 0
0 0 0 0 0 0 0 0 0 0 0 1 0 0
0 0 0 0 0 0 0 0 0 1 0 0 0 0
0
OTU1042 0 0 1 0 0 0 0 0 0 1 0 0 0
0 0 0 0 0 0 0 0 0 0 0 0 0 0
0 0 0 0 0 0 0 0 0 0 0 0 0 0
0 0 0 0 0 0 0 0 0 0 0 0 0 0
0 0 0 0 0 0 0 0 0 0 0 0 0 0
0 3 0 0 0 0 0 0 0 0 0 0 0 0
0 0 0 3 0 0 0 0 0 0 0 1 0 0
0 1 1 1 1 0 0 0 0 1 0 0 0 0
0
OTU179 0 0 3 0 0 0 0 0 0 4 0 15 0
0 0 16 2 0 1 0 0 1 3 0 0 0 1
2 0 2 67 12 0 0 0 0 95 0 0 0 0
1 36 0 47 2 0 0 5 1 23 0 11 2 0
0 0 0 0 0 2 10 7 46 3 1 0 1 0
2 11 1 0 1 7 2 7 30 2 0 58 94 2
0 22 3 0 0 0 0 0 0 12 0 8 4 0
0 34 1 58 3 29 2 6 2 12 1 2 2 29
33

OTU545	0	0	2	0	0	0	0	0	3	0	0	0	0
0	0	0	0	0	0	3	0	2	0	0	0	1	0
23	1	0	0	0	0	8	0	0	0	0	0	1	0
0	0	0	0	1	0	0	0	1	1	0	6	0	0
0	0	0	1	1	21	0	1	38	1	1	0	1	0
0	0	0	0	0	0	0	20	0	1	0	15	0	0
0	0	0	0	0	0	1	0	1	0	0	0	0	0
0	0	5	1	0	6	3	0	1	1	0	0	0	0
4													
OTU14562	0	0	1	0	0	0	0	0	0	0	0	0	0
0	0	0	0	0	0	0	0	0	0	0	0	0	0
0	0	0	0	0	0	0	0	0	0	0	0	0	0
0	0	0	0	0	0	0	0	0	0	0	0	0	0
0	0	0	0	0	0	0	0	0	0	0	0	0	0
0	0	0	0	0	0	0	0	0	0	0	0	0	0
0	0	0	0	0	0	0	0	0	0	0	0	0	0
0	0	0	0	0	0	0	0	0	0	0	0	0	0
0													
OTU11714	0	0	1	0	2	1	0	0	0	1	1	0	1
0	0	0	1	2	1	1	1	1	1	0	0	0	1
0	0	1	1	0	0	0	1	0	1	0	0	2	0
1	0	0	0	2	0	1	0	0	0	0	0	2	0
1	1	1	1	0	0	0	0	0	1	0	0	1	0
2	0	3	0	0	0	0	2	1	0	0	1	0	0
0	0	0	0	1	1	0	1	0	1	0	0	1	0
1	1	1	2	0	0	1	1	0	1	1	0	0	0
2													
OTU7625	0	0	1	1	0	1	0	0	10	39	0	0	0
0	0	0	1	0	0	2	0	0	0	0	0	4	0
0	0	0	0	0	0	2	0	0	0	0	0	2	0
66	0	0	0	0	0	0	0	0	0	0	7	0	1
0	0	0	0	0	0	0	1	3	0	0	0	2	0
1	0	0	0	1	0	0	1	0	1	0	7	0	4

0	0	0	0	0	0	0	1	0	0	0	0	0	0
0	1	0	0	0	0	0	0	0	0	1	0	0	0
0													
OTU14563	0	0	1	0	0	0	0	0	0	0	0	0	0
0	0	0	0	0	0	0	0	0	0	0	0	0	0
0	0	0	0	0	0	0	0	0	0	0	0	0	0
0	0	0	0	0	0	0	0	0	0	0	0	0	0
0	0	0	0	0	0	0	0	0	0	0	0	0	0
0	0	0	0	0	0	0	0	0	0	0	0	0	0
0	0	0	0	0	0	0	0	0	0	0	0	0	0
0	0	0	0	0	0	0	0	0	0	0	0	0	0
0													
OTU14560	0	0	2	0	0	2	0	0	0	0	0	0	0
0	0	0	0	0	0	0	1	0	0	0	0	0	0
0	0	0	0	0	0	0	0	0	0	0	0	0	0
0	0	0	0	0	0	0	0	0	0	0	0	0	0
0	0	0	0	0	0	0	0	0	0	0	0	0	0
0	0	0	0	3	2	0	0	0	0	0	0	1	0
0	0	0	0	0	0	0	0	0	0	0	0	0	0
0	0	0	0	0	0	0	0	0	0	0	0	0	0
0													
OTU10781	0	0	1	0	0	0	0	1	0	0	0	0	0
0	0	0	0	0	0	0	0	0	0	0	0	0	0
0	0	0	0	0	0	0	0	0	0	0	0	0	0
0	0	0	0	0	0	0	0	0	0	0	0	0	0
0	1	0	0	0	0	0	0	0	0	0	0	0	0
0	0	0	0	0	0	0	0	0	0	0	0	0	0
0	0	0	1	0	0	0	0	0	0	0	0	0	0
0	0	0	0	0	0	0	0	0	0	0	0	0	0
0													
OTU12308	0	0	1	0	0	0	0	0	0	0	0	0	0
0	0	0	0	0	0	0	0	0	0	0	0	0	0
0	0	0	0	0	0	0	0	0	0	0	0	0	0

0	0	0	0	0	0	0	0	0	0	0	0	0	0
0	0	0	0	0	0	0	0	0	0	0	0	0	0
0	0	0	0	0	0	0	0	0	0	0	0	0	0
0	0	0	0	0	0	0	0	0	0	0	0	0	0
0	0	0	0	0	0	0	0	0	0	0	0	0	0
0													
OTU12307	0	0	1	0	0	0	0	0	0	0	0	0	0
0	0	0	0	0	0	0	0	0	0	0	0	0	0
0	0	0	0	0	0	0	0	0	0	0	0	0	0
0	0	0	0	0	0	0	0	0	0	0	0	0	0
0	0	0	0	0	0	0	0	0	0	0	0	0	0
0	0	0	0	0	0	0	0	0	0	0	0	0	0
0	0	0	0	0	0	0	0	0	0	0	0	0	0
0	0	0	0	0	0	0	0	0	0	0	0	0	0
0													
OTU5487	0	0	1	1	2	0	1	0	1	0	0	0	0
0	0	0	0	0	0	0	1	0	4	0	0	0	0
0	0	1	1	0	1	1	1	1	1	0	0	0	0
0	0	0	0	1	0	0	0	0	0	0	0	2	0
0	0	1	0	0	0	0	0	0	0	0	0	0	3
1	0	0	1	0	0	0	0	0	0	0	0	1	0
0	0	0	0	0	0	0	0	0	0	0	0	0	3
0	0	0	0	0	0	0	0	0	0	0	1	0	0
0													
OTU15829	0	0	1	0	0	0	0	0	1	0	0	0	0
0	0	0	0	0	0	0	0	0	0	0	0	0	0
0	0	0	0	0	0	0	0	0	0	0	0	0	0
0	0	0	0	0	0	0	0	0	0	0	0	0	0
0	0	0	0	0	0	0	0	0	0	0	0	0	0
0	0	0	0	0	0	0	0	0	0	0	0	0	0
0	0	0	0	0	0	0	0	0	0	0	0	0	0
0	0	0	0	0	0	0	0	0	0	0	0	0	0
0													

OTU14561	0	0	1	0	0	0	0	0	0	0	0	0	0
0	0	0	0	0	0	0	0	0	0	0	0	0	0
0	0	0	0	0	0	0	0	0	0	0	0	0	0
0	0	0	0	0	0	0	0	0	0	0	0	0	0
0	0	0	0	0	0	0	0	0	0	0	0	0	0
0	0	0	0	0	0	0	0	0	0	0	0	0	0
0	0	0	0	0	0	0	0	0	0	0	0	0	0
0	0	0	0	0	0	0	0	0	0	0	0	0	0
0													
OTU8557	0	0	1	0	0	0	0	0	0	0	0	0	0
0	0	0	0	0	0	0	0	0	0	0	0	0	0
0	0	0	0	0	0	0	0	0	0	0	0	0	0
0	0	0	0	0	0	0	0	0	0	0	0	0	0
0	0	0	0	0	0	0	0	0	0	0	0	0	0
0	0	0	0	0	0	0	0	0	0	0	0	0	0
0	0	0	0	0	0	0	0	0	0	0	0	0	0
0	0	0	0	0	0	0	0	0	0	0	0	0	0
0													
OTU9653	0	0	1	0	0	1	0	3	3	0	0	1	1
0	1	0	0	0	0	0	0	1	0	0	0	1	0
0	0	0	0	0	0	0	0	0	0	0	0	0	0
1	0	0	1	0	0	0	0	0	0	0	0	0	0
0	0	0	0	0	0	0	0	0	0	0	0	0	0
0	1	1	1	0	0	0	0	0	6	0	0	0	1
1	0	0	0	0	0	0	0	0	0	0	0	0	0
0	0	0	0	0	0	0	0	0	0	1	0	0	0
0													
OTU1953	0	0	1	0	0	0	0	0	0	0	0	0	0
0	1	0	0	0	0	0	0	0	0	0	0	0	0
0	0	0	0	0	0	0	0	0	0	0	0	0	0
0	0	0	0	0	0	0	0	0	0	0	0	0	0
0	0	0	0	0	0	0	0	0	0	0	0	0	0
0	0	0	0	0	0	0	0	0	0	0	0	0	0

0 0 0 0 0 0 0 0 0 0 0 0 0 0
0 0 0 0 0 0 0 0 1 0 0 0 0 0
0
OTU12309 0 0 1 0 0 0 0 0 0 0 0 0 0
0 0 0 0 0 0 0 0 0 0 0 0 0 0
0 0 0 0 0 0 0 0 0 0 0 0 0 0
0 0 0 0 0 0 0 0 0 0 0 0 0 0
0 0 0 0 0 0 0 0 0 0 0 0 0 0
0 0 0 0 0 0 0 0 0 0 0 0 0 0
0 0 0 0 0 0 0 0 0 0 0 0 0 0
0 0 0 0 0 0 0 0 0 0 0 0 0 0
0
OTU12310 0 0 1 0 0 0 0 0 0 0 0 0 0
0 0 0 0 0 0 0 0 0 0 0 0 0 0
0 0 0 0 0 0 0 0 0 0 0 0 0 0
0 0 0 0 0 0 0 0 0 0 0 0 0 0
0 0 0 0 0 0 0 0 0 0 0 0 0 0
0 0 0 0 0 0 0 0 0 0 0 0 0 0
0 0 0 0 0 0 0 0 0 0 0 0 0 0
0 0 0 0 0 0 0 0 0 0 0 0 0 0
0
OTU194 0 0 1 0 0 0 0 0 0 0 4 2 9
3 0 0 0 1 1 0 9 0 13 0 6 0 0
0 0 0 3 0 4 6 20 9 4 30 34 1 0
0 0 0 0 20 13 11 69 6 1 22 41 2 24
2 0 28 8 10 6 1 6 7 0 4 20 7 0
3 0 0 7 0 0 0 0 0 0 0 0 0 0
18 8 2 0 0 0 2 1 4 0 34 0 0 97
7 0 0 0 0 0 0 0 6 0 1 35 17 0
0
OTU2598 0 0 1 0 0 4 30 2 0 9 0 0 1
0 2 0 73 0 0 0 1 11 0 0 0 1 0
19 13 1 8 15 0 16 0 0 0 0 0 38 0

0 0 0 0 0 2 0 0 0 0 9 1 4 0
0 0 3 5 0 0 0 0 4 0 0 0 1 0
2 0 2 0 0 0 2 7 0 1 0 6 0 15
0 56 0 8 0 18 0 75 0 0 0 1 0 0
0 1 0 0 0 0 2 0 8 2 0 0 2 1
0
OTU2840 0 0 1 0 0 0 0 0 0 0 0 0 0
0 0 0 0 0 0 0 0 0 0 0 0 0 0
0 0 0 0 0 1 0 0 0 0 0 1 0 0
0 0 0 0 0 0 0 0 0 0 0 0 0 0
0 0 0 0 0 0 0 0 0 0 0 0 0 0
0 0 0 0 0 0 0 0 0 0 0 0 0 0
0 0 0 0 0 0 0 0 0 0 0 0 0 0
0 0 1 0 0 0 0 0 0 4 0 0 0 0
0
OTU10792 0 0 1 0 0 0 0 0 0 0 0 0 0
0 0 0 0 0 0 0 0 0 0 0 0 0 0
0 0 0 0 0 0 0 0 0 0 0 0 0 0
0 0 0 0 0 0 0 0 0 0 0 0 0 0
0 0 0 0 0 0 0 0 0 0 0 0 0 0
0 0 0 0 0 0 0 0 0 0 0 0 0 0
0 0 0 0 0 0 0 0 0 0 0 0 0 0
0 0 0 0 0 0 0 0 0 0 0 0 0 0
0
OTU14552 0 0 1 0 0 0 0 0 0 0 0 0 0
0 0 0 0 0 0 0 0 0 0 0 0 0 0
0 0 0 0 0 0 0 0 0 0 0 0 0 0
0 0 0 0 0 0 0 0 0 0 0 0 0 0
0 0 0 0 0 0 0 0 0 0 0 0 0 0
0 0 0 0 0 0 0 0 0 0 0 0 0 0
0 0 0 0 0 0 0 0 0 0 0 0 0 0
0 0 0 0 0 0 0 0 0 0 0 0 0 0
0

OTU9454	0	0	1	0	2	0	0	0	1	0	0	0	0
0	1	0	0	0	0	0	0	0	0	0	0	0	0
0	0	2	0	0	0	0	2	0	0	0	0	0	0
0	0	0	0	0	0	0	0	0	0	0	0	0	1
0	0	0	0	0	0	0	0	0	0	0	0	0	0
1	0	1	0	0	0	0	0	0	1	0	0	0	0
0	0	2	0	0	0	0	0	0	0	0	0	0	0
0	0	0	0	0	0	0	0	0	0	0	0	1	0
0													
OTU192	0	0	1	0	0	0	0	0	40	0	0	1	0
0	0	0	0	0	0	0	1	15	0	0	1	0	0
6	0	0	0	0	0	0	0	1	1	0	0	2	0
8	2	0	0	0	0	0	0	0	0	1	0	2	0
0	1	0	5	1	1	0	0	0	0	0	0	0	0
0	29	0	1	0	0	1	3	0	0	0	7	0	1
2	32	1	9	0	0	0	0	0	0	0	8	10	0
0	42	28	38	44	43	32	12	6	2	0	1	0	5
0													
OTU921	0	0	1	0	0	1	0	0	2	1	0	0	0
0	0	0	0	0	0	0	0	0	0	0	0	0	0
0	0	0	0	0	0	0	0	0	0	0	0	0	0
3	0	0	0	0	0	0	1	0	0	0	0	0	0
0	0	0	0	0	0	0	0	0	0	0	0	0	0
0	1	0	0	1	0	0	0	0	3	0	0	0	2
0	0	0	0	0	0	0	2	0	0	0	1	1	0
0	4	7	3	5	2	1	4	1	4	0	0	0	3
0													
OTU8558	0	0	1	0	0	0	0	0	0	0	0	0	0
0	0	0	0	0	0	0	0	0	0	0	0	0	0
0	0	0	0	0	0	0	0	0	0	0	0	0	0
0	0	0	0	0	0	0	0	0	0	0	0	0	0
0	0	0	0	0	0	0	0	0	0	0	0	0	0
0	0	0	0	0	0	0	0	0	0	0	0	0	0

0	0	0	0	0	0	0	0	0	0	0	0	0	0
0	0	0	0	0	0	0	0	0	0	0	0	0	0
0													
OTU457	0	0	2	0	0	4	18	0	0	10	0	0	0
0	10	0	0	0	0	0	0	0	0	0	0	0	0
0	0	0	0	0	0	0	0	0	0	0	0	0	0
7	13	0	0	0	0	0	0	0	0	2	0	0	0
0	0	0	0	3	0	0	0	0	1	0	0	5	2
0	2	0	2	6	0	0	0	0	5	0	0	2	0
0	0	0	1	1	0	0	0	0	0	0	3	4	0
0	4	2	1	8	8	2	0	4	1	0	0	0	4
0													
OTU6570	0	0	1	0	0	1	2	1	0	8	0	1	0
0	0	0	0	0	0	5	0	0	0	0	0	0	0
1	0	0	0	0	0	0	0	0	0	0	0	0	0
1	0	0	0	0	1	0	0	0	0	0	1	0	0
0	0	0	0	0	0	0	0	0	0	0	0	0	0
0	2	0	0	0	0	0	0	0	0	0	1	0	0
0	0	0	4	0	3	0	0	0	0	0	0	0	0
0	0	0	0	0	1	0	0	0	0	0	0	0	1
0													
OTU14548	0	0	3	0	0	0	0	0	0	0	0	0	0
0	0	0	0	0	0	0	0	0	0	0	0	0	0
0	0	0	0	0	0	0	0	0	0	0	0	0	0
0	0	0	0	0	0	0	0	0	0	0	0	0	0
0	0	0	0	0	0	0	0	0	0	0	0	0	0
0	0	0	0	0	0	0	0	0	0	0	0	0	0
0	0	0	0	0	0	0	0	0	0	0	0	0	0
0	0	0	0	0	0	0	0	0	0	0	0	0	0
0													
OTU376	0	0	1	0	0	0	8	0	4	0	0	0	0
0	0	0	1	0	0	0	0	2	0	0	0	0	0
0	18	0	0	2	0	6	0	1	0	7	0	1	0

1	0	0	0	4	0	0	0	5	4	0	0	1	0
0	0	7	1	5	198	0	0	0	6	0	0	1	1
1	1	0	0	0	0	0	7	0	0	0	0	0	5
0	0	0	0	0	0	0	1	0	0	0	0	1	0
0	0	37	13	0	1	30	3	6	12	0	0	0	25
2													
OTU3954	0	0	1	0	0	0	0	0	0	0	0	0	0
0	0	0	0	0	0	0	0	0	0	0	0	0	0
0	1	0	0	0	0	0	0	0	0	0	0	0	0
0	0	0	0	0	0	0	0	0	0	0	0	0	0
0	0	0	0	0	0	0	0	0	0	0	0	0	0
1	0	0	0	0	0	0	1	0	0	0	0	0	0
0	2	0	0	0	0	0	0	0	0	0	0	0	0
0	0	0	0	0	0	0	0	0	0	0	0	0	0
0													
OTU11899	0	0	1	0	0	0	1	1	0	2	0	0	0
0	0	1	0	0	5	0	3	0	1	0	3	2	10
1	0	0	0	0	0	0	0	0	5	1	1	0	3
2	1	0	0	0	2	1	0	0	0	1	0	0	0
0	0	0	0	0	0	0	0	0	0	0	0	9	0
0	2	2	0	7	2	3	0	0	1	0	0	1	3
0	0	0	3	2	4	1	0	0	1	0	3	2	1
0	9	0	2	2	0	1	2	2	8	3	1	0	4
2													
OTU3752	0	0	1	0	0	0	1	0	0	0	0	0	0
0	0	0	0	0	0	0	0	0	3	4	1	0	0
0	0	0	0	0	0	0	0	0	0	0	0	0	0
0	0	1	0	0	0	1	0	0	0	0	0	0	0
1	1	0	0	0	0	1	0	0	0	0	0	0	1
0	0	0	0	0	0	0	0	0	0	0	0	0	0
0	0	1	0	0	0	0	0	0	0	0	0	0	0
0	0	0	0	0	0	0	0	0	0	0	0	0	0
0													

OTU11143	0	0	1	1	2	0	0	0	1	2	0	0	0
0	1	2	2	2	0	0	0	1	0	0	0	0	0
0	0	2	1	0	0	0	0	0	0	0	0	1	1
0	0	0	0	0	0	0	0	2	3	0	1	0	0
0	0	1	0	0	1	1	0	2	1	1	0	0	0
3	0	0	0	3	0	0	2	2	0	0	1	0	0
1	0	0	1	0	3	0	1	0	0	1	0	0	0
0	0	0	1	0	0	1	0	0	0	0	0	0	0
2													
OTU2499	0	0	1	0	0	0	0	0	0	1	0	0	0
0	0	0	0	0	0	0	0	0	0	0	0	0	0
0	0	0	0	0	0	0	0	0	0	0	0	0	0
0	0	0	0	0	1	0	0	0	0	0	0	0	0
0	0	0	0	0	0	0	0	0	0	0	0	0	0
0	0	0	0	0	0	0	0	0	1	0	0	1	0
0	0	0	0	0	0	0	0	0	1	0	0	0	0
0	0	0	0	0	0	0	0	0	0	0	0	0	0
0													
OTU10788	0	0	1	0	0	0	0	0	0	0	0	0	0
0	0	0	0	0	0	0	0	0	0	0	0	0	0
0	0	0	0	0	0	0	0	0	0	0	0	0	0
0	0	0	0	0	0	0	0	0	0	0	0	0	0
0	0	0	0	0	0	0	0	0	0	0	0	0	0
0	0	0	0	0	0	0	0	0	0	0	0	0	0
0	0	0	0	0	0	0	0	0	0	0	0	0	0
0	0	0	0	0	0	0	0	0	0	0	0	0	0
0													
OTU10789	0	0	1	0	0	0	0	0	0	0	0	0	0
0	0	0	0	0	0	0	0	0	0	0	0	0	0
0	0	0	0	0	0	0	0	0	0	0	0	0	0
0	0	0	0	0	0	0	0	0	0	0	0	0	0
0	0	0	0	0	0	0	0	0	0	0	0	0	0
0	0	0	0	0	0	0	0	0	0	0	0	0	0

0	0	0	0	0	0	0	0	0	0	0	0	0	0
0	0	0	0	0	0	0	0	0	0	0	0	0	0
0													
OTU2040	0	0	1	0	0	0	2	0	0	0	0	0	0
0	0	0	0	0	0	0	0	0	0	0	0	0	0
0	0	0	0	0	0	0	0	0	0	0	0	1	0
0	3	0	0	0	0	0	0	0	0	2	0	0	1
0	0	0	0	0	0	0	0	0	2	0	0	0	0
0	1	0	0	0	0	0	0	0	0	0	0	0	0
0	0	0	0	0	0	0	0	0	0	0	0	0	0
0	0	0	0	0	0	0	1	0	0	0	0	0	1
0													
OTU5678	0	0	1	0	0	0	0	0	0	0	0	0	0
0	0	0	0	0	0	0	1	0	0	1	0	0	0
3	2	0	0	0	0	0	0	0	0	1	0	0	0
0	0	0	0	1	0	0	0	0	0	0	1	4	0
0	0	1	0	1	0	0	0	3	0	0	0	1	0
2	0	0	0	0	0	0	2	0	0	0	1	2	1
1	0	0	0	0	0	0	0	0	0	0	0	0	0
0	0	0	0	0	0	0	0	0	0	0	0	0	0
0													
OTU11799	0	0	1	0	0	0	0	0	1	0	0	0	0
0	1	0	0	0	0	0	0	0	0	0	0	0	0
9	0	0	0	0	0	0	0	0	0	0	0	0	0
0	0	0	0	0	0	0	0	0	0	0	2	0	0
0	0	0	0	0	0	0	0	1	0	0	0	0	0
0	0	0	0	0	0	0	0	0	0	0	0	0	0
0	0	0	1	0	0	0	0	0	0	0	0	0	0
0	0	0	0	0	0	0	0	0	0	0	0	0	0
0													
OTU4608	0	0	1	0	0	0	0	0	0	1	0	0	0
0	0	0	0	0	0	0	0	0	0	0	0	0	0
0	0	0	0	0	0	0	0	0	0	0	0	1	0

0	0	0	0	0	0	0	0	0	0	0	0	0	1
0	0	0	0	0	0	0	0	0	0	0	0	0	0
0	0	0	0	0	0	0	0	0	0	0	0	0	1
0	0	0	0	0	1	0	1	0	0	0	1	0	0
0	0	0	0	0	0	0	0	0	0	0	0	0	3
0													
OTU8551	0	0	1	0	0	0	0	0	0	0	0	0	0
0	0	0	0	0	0	0	0	0	0	0	0	0	0
0	0	0	0	0	0	0	0	0	0	0	0	0	0
0	0	0	0	0	0	0	0	0	0	0	0	0	0
0	0	0	0	0	0	0	0	0	0	0	0	0	0
0	0	0	0	0	0	0	0	0	0	0	0	0	0
0	0	0	0	0	0	0	0	0	0	0	0	0	0
0	0	0	0	0	0	0	0	0	0	0	0	0	0
0													
OTU10786	0	0	1	0	0	0	0	0	0	0	0	0	0
0	0	0	0	0	0	0	0	0	0	0	0	0	0
0	0	0	0	0	0	0	0	0	0	0	0	0	0
0	0	0	0	0	0	0	0	0	0	0	0	0	0
0	0	0	0	0	0	0	0	0	0	0	0	0	0
0	0	0	0	0	0	0	0	0	0	0	0	0	0
0	0	0	0	0	0	0	0	0	0	0	0	0	0
0	0	0	0	0	0	0	0	0	0	0	0	0	0
0													
OTU7600	0	0	1	0	1	0	1	0	0	0	0	0	0
0	1	0	0	0	0	0	0	0	0	0	0	0	0
0	0	0	0	0	0	0	0	2	0	0	0	0	0
0	0	0	0	0	0	0	0	0	0	0	0	0	0
0	0	0	0	0	0	0	0	0	0	0	0	0	0
0	0	0	0	3	0	0	0	0	0	0	0	1	0
0	0	0	0	0	0	0	1	0	0	0	0	0	0
0	0	0	0	0	0	0	0	0	0	0	0	0	0
0													

OTU2057	0	0	1	0	0	0	0	0	1	1	0	0	0
0	0	1	0	0	1	0	1	0	1	0	1	1	0
0	0	1	0	0	0	1	0	0	0	0	0	1	0
0	0	1	0	0	0	0	0	0	1	0	0	0	0
0	0	1	0	0	2	0	0	2	0	1	0	0	0
0	0	0	0	2	0	0	1	0	1	1	0	0	0
0	0	0	0	0	0	0	0	0	0	0	0	1	0
0	0	0	0	0	1	0	0	0	0	0	0	0	0
1													
OTU554	0	0	1	0	0	0	2	0	4	0	0	0	0
0	0	0	0	0	0	0	0	0	0	0	0	1	0
0	0	0	0	0	0	0	0	1	0	0	0	0	0
0	0	0	0	0	0	0	0	0	0	0	0	0	0
0	0	0	1	0	0	0	0	0	0	0	0	2	0
0	7	0	0	0	0	0	2	0	0	0	2	0	4
0	1	0	2	0	0	0	0	0	0	0	5	4	0
0	8	3	0	2	63	15	0	2	0	0	0	0	0
0													
OTU13022	0	0	1	0	0	0	0	0	0	0	0	0	0
0	0	0	0	0	0	0	0	0	0	0	0	0	0
0	0	0	0	0	0	0	0	0	0	2	0	0	0
0	0	0	0	0	0	0	0	0	0	0	0	0	0
0	0	0	0	0	0	0	0	0	0	0	0	0	0
0	1	0	0	0	0	0	0	0	0	0	0	0	0
0	0	0	0	0	0	0	0	0	0	0	0	2	0
0	2	5	3	4	9	2	2	2	4	0	0	0	2
0													
OTU8554	0	0	1	0	0	0	0	0	0	0	0	0	0
0	0	0	0	0	0	0	0	0	0	0	0	0	0
0	0	0	0	0	0	0	0	0	0	0	0	0	0
0	0	0	0	0	0	0	0	0	0	0	0	0	0
0	0	0	0	0	0	0	0	0	0	0	0	0	0
0	0	0	0	0	0	0	0	0	0	0	0	0	0

0	0	0	0	0	0	0	0	0	0	0	0	0	0
0	0	0	0	0	0	0	0	0	0	0	0	0	0
0													
OTU3399	0	0	1	0	0	0	0	0	0	0	0	0	0
0	0	0	0	1	0	0	0	0	2	0	0	0	0
0	0	0	0	0	0	0	0	0	0	0	0	0	0
0	0	0	0	0	0	0	0	0	0	0	0	0	0
0	0	0	0	0	1	0	0	0	0	0	0	0	0
0	0	0	0	0	0	0	0	0	0	0	0	0	0
0	1	0	0	0	0	0	0	0	0	0	0	0	0
0	0	0	0	0	0	0	0	0	0	0	0	0	0
0													
OTU14116	0	0	1	5	1	0	0	0	1	0	0	3	1
0	0	10	6	0	0	1	13	4	1	1	0	4	0
6	3	0	1	1	0	1	0	1	2	1	2	3	0
2	0	0	6	0	0	0	0	2	1	0	0	1	0
1	0	3	1	0	0	1	0	1	2	0	0	0	0
1	0	0	14	0	1	0	5	0	1	0	0	0	1
0	3	0	0	0	0	1	4	0	0	0	0	0	2
0	0	0	0	0	0	0	0	0	0	0	0	0	0
0													
OTU10791	0	0	1	0	0	0	0	0	0	0	0	0	0
0	0	0	0	0	0	0	0	0	0	0	0	0	0
0	0	0	0	0	0	0	0	0	0	0	0	0	0
0	0	0	0	0	0	0	0	0	0	0	0	0	0
0	0	0	0	0	0	0	0	0	0	0	0	0	0
0	0	0	0	0	0	0	0	0	0	0	0	0	0
0	0	0	0	0	0	0	0	0	0	0	0	0	0
0	0	0	0	0	0	0	0	0	0	0	0	0	0
0													
OTU14547	0	0	1	0	0	0	0	0	0	0	0	0	0
0	0	0	0	0	0	0	0	0	0	0	0	0	0
0	0	0	0	0	0	0	0	0	0	0	0	0	0

0 0 0 0 0 0 0 0 0 0 0 0 0 0

0 0 0 0 0 0 0 0 0 0 0 0 0 0

0 0 0 0 0 0 0 0 0 0 0 0 0 0

0 0 0 0 0 0 0 0 0 0 0 0 0 0

0 0 0 0 0 0 0 0 0 0 0 0 0 0

0

OTU8553 0 0 1 0 0 0 0 0 0 0 0 0 0

0 0 0 0 0 0 0 0 0 0 0 0 0 0

0 0 0 0 0 0 0 0 0 0 0 0 0 0

0 0 0 0 0 0 0 0 0 0 0 0 0 0

0 0 0 0 0 0 0 0 0 0 0 0 0 0

0 0 0 0 0 0 0 0 0 0 0 0 0 0

0 0 0 0 0 0 0 0 0 0 0 0 0 0

0 0 0 0 0 0 0 0 0 0 0 0 0 0

0

OTU10790 0 0 1 0 0 0 0 0 0 0 0 0 0

0 0 0 0 0 0 0 0 0 0 0 1 0 0

0 0 0 0 0 0 0 0 0 0 0 0 0 0

0 0 0 0 0 0 0 0 0 0 0 0 0 0

0 0 0 0 0 1 0 0 0 0 0 0 0 0

0 0 0 0 0 0 0 0 0 0 0 0 0 0

0 0 0 0 0 1 0 0 0 0 0 0 0 0

0 0 0 0 0 0 0 0 0 0 0 0 0 0

0

OTU13557 0 0 1 0 0 0 0 0 0 0 0 0 0

0 0 0 0 0 0 0 0 0 0 0 0 0 0

0 0 0 0 0 0 0 0 0 0 0 0 0 0

0 0 0 0 0 0 0 0 0 0 0 0 0 0

0 0 0 0 0 0 0 0 0 0 0 0 0 0

0 0 0 0 0 0 0 0 0 0 0 1 0 0

0 0 0 0 0 0 0 0 0 0 0 0 0 0

0 0 0 0 0 0 0 0 0 0 0 0 0 0

0

OTU12259 0 0 1 0 0 0 0 0 0 0 0 0 0
0 0 0 0 0 0 0 0 0 0 0 0 0 0
0 0 0 0 0 0 0 0 0 0 0 0 0 0
0 0 0 0 0 0 0 0 0 0 0 0 0 0
0 0 0 0 0 0 0 0 0 0 0 0 0 0
0 0 0 0 0 0 0 0 0 0 0 0 0 0
0 0 0 0 0 0 0 0 0 0 0 0 0 0
0 0 0 0 0 0 0 0 0 0 0 0 0 0
0
OTU576 0 0 1 0 0 1 0 0 0 0 0 0 1
0 0 0 0 0 0 1 0 0 0 0 0 0 0
1 0 0 0 0 0 0 0 0 0 1 0 0 0
0 0 0 0 0 36 0 0 0 0 0 0 0 0
0 1 0 0 0 0 0 0 0 0 0 0 0 0
0 0 0 0 0 0 0 0 0 0 0 0 0 0
0 0 0 0 0 0 0 0 0 1 0 0 0 0
0 0 0 1 0 0 0 0 0 0 0 0 0 0
0
OTU6254 0 0 1 1 0 0 0 0 0 0 0 0 0
0 0 0 0 0 0 0 0 0 0 1 2 0 0
0 0 0 0 0 0 0 0 0 0 0 0 0 0
0 0 0 0 0 0 0 0 1 0 0 0 0 0
0 0 0 0 0 0 0 0 0 0 0 0 0 0
0 0 0 0 0 1 0 0 0 0 0 1 0 1
0 0 1 0 0 0 0 0 0 0 0 0 0 0
0 0 0 0 0 0 0 0 0 0 0 0 0 0
0
OTU14549 0 0 1 0 0 0 0 0 0 0 0 0 0
0 0 0 0 0 0 0 0 0 0 0 0 0 0
0 0 0 0 0 0 0 0 0 0 0 0 0 0
0 0 0 0 0 0 0 0 0 0 0 0 0 0
0 0 0 0 0 0 0 0 0 0 0 0 0 0
0 0 0 0 0 0 0 0 0 0 0 0 0 0

0	0	0	0	0	0	0	0	0	0	0	0	0	0
0	0	0	0	0	0	0	0	0	0	0	0	0	0
0													
OTU10773	0	0	1	0	0	0	2	0	0	0	0	5	1
3	0	0	0	0	0	2	0	1	0	0	2	0	0
2	0	0	0	0	0	3	1	1	0	0	0	0	0
0	4	0	0	0	0	0	1	0	0	0	1	0	0
0	0	0	0	0	0	1	0	0	0	0	1	0	0
0	0	0	0	4	2	0	0	7	0	1	0	0	0
1	0	2	0	1	0	2	0	0	0	1	0	0	0
2	0	0	1	0	0	0	0	1	0	3	6	1	0
0													
OTU8352	0	0	1	0	0	0	0	0	0	0	0	0	0
0	0	0	0	0	0	0	0	0	0	0	0	0	0
0	0	0	0	0	0	0	0	0	0	0	0	0	0
0	0	0	0	0	0	0	0	0	0	0	0	0	0
0	0	0	0	0	0	0	0	0	0	0	0	0	0
0	0	0	0	0	0	0	0	0	0	0	0	0	0
0	0	0	0	0	0	0	0	0	0	0	0	0	0
0	0	0	0	0	0	0	0	0	0	0	0	0	0
0													
OTU10772	0	0	1	0	0	0	0	0	0	0	0	0	0
0	0	0	0	0	0	0	0	0	0	0	0	0	0
0	0	0	0	0	0	0	0	0	0	0	0	0	0
0	0	0	0	0	0	0	0	0	0	0	0	0	0
0	0	0	0	0	0	0	0	0	0	0	0	0	0
0	0	0	0	0	0	0	0	0	0	0	0	0	0
0	0	0	0	0	0	0	0	0	0	0	0	0	0
0	0	0	0	0	0	0	0	0	0	0	0	0	0
0													
OTU14551	0	0	1	0	0	0	0	0	0	0	0	0	0
0	0	0	0	0	0	0	0	0	0	0	0	0	0
0	0	0	0	0	0	0	0	0	0	0	0	0	0

	0	0	0	0	0	0	0	0	0	0	0	0	0
0	0	0	0	0	0	0	0	0	0	0	0	0	0
0	0	0	0	0	0	0	0	0	0	0	0	0	0
0	0	0	0	0	0	0	0	0	0	0	0	0	0
0	0	0	0	0	0	0	0	0	0	0	0	0	0
0													
OTU10913	0	0	2	8	0	0	6	6	1	2	0	0	0
0	2	0	0	0	1	0	0	0	0	0	0	0	0
0	0	0	0	0	0	0	0	0	0	0	0	0	0
2	0	1	3	0	1	0	0	0	0	0	0	1	0
0	0	1	0	0	0	0	0	0	0	0	0	0	0
0	2	0	0	0	9	0	0	0	3	0	0	0	0
0	0	0	0	0	0	0	0	0	1	2	4	2	0
0	2	8	2	3	2	0	5	4	5	0	0	0	2
3													
OTU14035	0	0	1	0	0	0	0	0	0	0	0	0	0
0	0	0	0	0	0	0	0	0	0	0	0	0	0
0	0	0	0	0	0	0	0	0	0	0	0	0	0
1	0	0	0	0	0	0	0	0	0	0	0	0	0
0	0	0	0	0	0	0	0	0	0	0	0	0	0
0	0	0	0	0	0	0	1	0	0	0	0	0	0
0	0	0	0	0	0	0	0	0	0	0	0	0	0
0	0	0	0	0	0	0	0	0	0	0	0	0	0
0													
OTU6035	0	0	1	1	0	0	0	3	0	0	0	0	0
0	0	0	0	0	0	2	0	0	0	0	0	0	0
0	0	0	0	0	0	0	0	0	0	0	0	0	0
1	0	0	0	0	1	0	0	0	0	0	0	0	0
0	0	0	0	0	0	0	0	0	0	0	0	0	0
0	0	3	0	0	0	0	0	0	0	0	0	0	6
0	0	0	0	0	0	0	4	0	0	0	0	0	0
0	0	0	0	0	0	0	0	0	0	0	0	0	0
0													

OTU5005 0 0 1 0 0 0 0 0 0 0 0 0 0
0 0 0 0 0 0 0 0 0 0 0 0 0 0
0 0 0 0 0 0 0 0 0 0 0 1 0 1
0 0 0 0 0 0 0 0 0 0 0 0 0 0
0 0 0 0 0 1 0 0 0 0 0 0 0 0
0 0 0 0 0 0 0 0 0 0 0 0 0 0
0 0 0 0 0 0 0 0 0 0 0 0 0 0
0 0 0 0 0 0 0 0 0 0 0 0 0 0
0
OTU8353 0 0 1 0 0 0 0 0 0 0 0 0 0
0 0 0 0 0 0 0 0 0 0 0 0 0 0
0 0 0 0 0 0 0 0 0 0 0 0 0 0
0 0 0 0 0 0 0 0 0 0 0 0 0 0
0 0 0 0 0 0 0 0 0 0 0 0 0 0
0 0 0 0 0 0 0 0 0 0 0 0 0 0
0 0 0 0 0 0 0 0 0 0 0 0 0 0
0 0 0 0 0 0 0 0 0 0 0 0 0 0
0
OTU14556 0 0 1 0 0 0 0 0 0 0 0 0 0
0 0 0 0 0 0 0 0 0 0 0 0 0 0
0 0 0 0 0 0 0 0 0 0 0 0 0 0
0 0 0 0 0 0 0 0 0 0 0 0 0 0
0 0 0 0 0 0 0 0 0 0 0 0 0 0
0 0 0 0 0 0 0 0 0 0 0 0 0 0
0 0 0 0 0 0 0 0 0 1 0 0 0 0
0 0 0 0 0 0 0 0 0 0 0 0 0 0
0
OTU8317 0 0 1 1 0 1 1 1 1 1 2 0 0
1 4 1 0 0 0 0 0 0 2 0 1 0 1
0 0 2 0 0 0 1 1 1 0 3 0 1 0
0 1 1 0 0 0 0 0 2 4 1 0 0 0
1 0 0 0 0 2 0 0 0 2 0 0 0 0
0 0 0 0 1 1 0 1 0 0 0 1 0 0

0	0	0	0	0	0	0	1	1	1	0	0	0	0
0	1	1	0	1	0	0	1	0	0	0	0	0	0
3													
OTU8349	0	0	1	0	0	0	0	0	0	0	0	0	0
0	0	0	0	0	0	0	0	0	0	0	0	0	0
0	0	0	0	0	0	0	0	0	0	0	0	0	0
0	0	0	0	0	0	0	0	0	0	0	0	0	0
0	0	0	0	0	0	0	0	0	0	0	0	0	0
0	0	0	0	0	0	0	0	0	0	0	0	0	0
0	0	0	0	0	0	0	0	0	0	0	0	0	0
0	0	0	0	0	0	0	0	0	0	0	0	0	0
0													
OTU14557	0	0	1	0	0	0	0	0	0	0	0	0	0
0	0	0	0	0	0	0	0	0	0	0	0	0	0
0	0	0	0	0	0	0	0	0	0	0	0	0	0
0	0	0	0	0	0	0	0	0	0	0	0	0	0
0	0	0	0	0	0	0	0	0	0	0	0	0	0
0	0	0	0	0	0	0	0	0	0	0	0	0	0
0	0	0	0	0	0	0	0	0	0	0	0	0	0
0	0	0	0	0	0	0	0	0	0	0	0	0	0
0													
OTU989	0	0	2	0	0	0	0	0	0	0	0	0	0
0	0	0	0	0	0	0	0	0	0	0	0	0	0
0	0	0	0	0	0	0	0	0	0	0	0	0	0
0	0	0	0	0	0	0	0	0	0	0	0	0	0
0	0	0	0	0	0	0	0	0	0	0	0	0	0
0	0	0	0	0	0	0	0	0	0	0	0	0	0
0	0	0	0	0	0	0	0	0	0	0	0	0	0
0	0	0	0	0	0	0	0	0	0	0	0	0	0
0													
OTU1246	0	0	1	1	1	0	1	1	0	0	0	1	0
0	0	0	0	0	0	0	0	0	0	0	0	0	0
0	0	0	0	0	0	0	0	0	0	0	0	0	1

0	0	0	0	0	0	0	0	0	0	0	0	0	0
0	0	0	0	0	0	0	0	0	0	0	0	0	0
0	1	0	0	1	0	0	0	0	1	0	0	0	1
0	0	0	0	0	0	0	0	0	0	0	0	0	0
0	0	0	0	0	0	0	0	0	0	0	0	0	0
0													
OTU12938	0	0	0	3	1	0	1	1	0	0	1	7	0
2	5	0	1	1	0	2	0	0	0	1	1	11	2
3	0	0	0	0	0	0	8	1	0	1	0	6	0
14	0	0	2	0	0	0	1	0	0	1	0	0	2
0	3	1	0	0	0	0	0	0	1	0	0	0	0
0	0	5	1	0	0	0	0	0	0	0	0	1	0
4	0	0	0	0	1	2	3	5	1	6	0	2	0
1	0	0	0	1	0	0	0	1	0	0	0	1	0
0													
OTU10917	0	0	0	65	0	0	0	1	0	0	0	1	0
0	0	0	0	0	0	2	1	0	0	2	0	0	1
1	0	0	0	0	0	0	0	0	0	0	0	3	0
1	0	0	0	2	0	0	1	0	0	10	0	0	0
0	0	0	1	0	6	0	1	8	3	7	0	1	0
0	1	0	0	1	0	0	7	0	0	0	0	0	1
0	7	0	0	0	0	31	2	1	0	2	0	0	0
1	0	0	0	0	0	0	0	0	1	0	0	2	1
0													
OTU412	0	0	0	4	0	0	0	0	0	0	0	0	0
0	0	3	0	3	4	0	0	0	0	0	0	2	2
0	0	0	2	3	0	1	0	0	3	0	0	0	0
0	0	0	0	0	0	0	0	2	0	0	0	0	5
0	0	0	0	2	0	0	0	0	0	0	2	0	0
0	0	0	1	0	5	2	0	0	0	3	0	1	1
6	1	0	0	1	0	2	0	0	1	0	0	0	0
0	0	0	0	0	0	0	0	0	0	1	0	2	0
0													

OTU631	0	0	0	1	0	1	0	0	2	0	4	0	4
3	0	3	0	0	2	0	3	0	0	0	0	0	0
0	0	5	2	0	0	0	1	0	1	0	0	0	3
1	0	0	0	0	0	0	0	0	0	0	0	0	0
0	0	0	2	0	0	0	0	0	0	0	0	2	0
0	0	0	0	2	0	2	0	0	0	0	0	1	0
0	0	0	0	0	0	2	0	1	1	2	0	0	0
1	0	0	0	0	0	0	0	0	0	1	0	2	0
0													
OTU9959	0	0	0	3	0	0	0	0	0	0	19	4	28
61	0	1	0	3	9	7	2	0	0	0	2	0	2
0	0	19	1	1	17	0	15	9	1	0	4	0	42
0	5	0	4	0	0	15	0	0	0	0	0	1	3
0	1	0	0	1	0	0	3	0	1	0	3	0	0
0	0	0	8	0	0	20	0	17	0	10	0	6	0
43	1	24	0	37	1	5	1	51	3	16	1	0	9
13	1	0	0	0	1	0	0	2	0	12	9	1	0
0													
OTU8355	0	0	0	1	0	0	0	0	0	0	0	0	0
0	0	0	0	0	0	0	0	0	0	0	0	0	0
0	0	0	0	0	0	0	0	0	0	0	0	0	0
0	0	0	0	0	0	0	0	0	0	0	0	0	0
0	0	0	0	0	0	0	0	0	0	0	0	0	0
0	1	0	0	0	1	0	0	0	0	0	0	0	0
0	0	0	0	0	0	0	0	0	0	0	0	0	0
0	0	0	0	0	0	0	0	0	0	0	0	0	0
0													
OTU1797	0	0	0	2	2	1	2	1	1	3	2	0	1
0	0	2	0	2	0	0	1	0	0	0	0	1	0
1	0	1	2	0	0	0	2	2	1	1	3	4	1
2	0	2	0	0	0	0	1	4	2	1	0	0	0
0	0	1	1	0	1	1	0	0	1	3	1	4	0
1	1	1	0	2	0	0	0	0	1	0	7	0	3

2	0	0	1	0	2	0	1	0	1	2	1	1	0
0	1	2	1	2	0	0	0	0	2	0	1	1	2
0													
OTU11211	0	0	0	25	0	0	0	0	0	0	1	0	1
0	0	0	0	37	0	0	0	0	1	0	0	0	0
0	0	0	0	0	0	0	1	0	0	0	0	0	0
0	0	0	0	0	0	0	0	0	0	0	0	0	0
0	0	0	1	0	0	0	0	0	0	0	0	0	0
0	0	0	0	0	0	0	0	0	0	0	0	0	0
0	0	0	0	7	0	0	0	7	0	0	0	0	1
0	0	0	0	0	0	0	0	0	0	0	0	0	0
6													
OTU168	0	0	0	2	1	0	0	0	4	1	1	2	6
3	0	58	2	3	13	2	38	0	11	114	6	3	6
2	2	2	6	6	14	7	0	0	17	2	0	14	5
0	0	28	7	2	2	2	1	0	21	14	1	0	0
1	0	0	0	0	3	0	22	2	0	8	1	1	0
0	0	0	5	1	21	0	14	2	1	19	2	3	2
1	0	8	0	9	0	8	2	3	11	7	0	0	1
10	0	2	3	0	0	0	0	0	0	3	4	9	0
0													
OTU552	0	0	0	2	2	2	1	0	0	0	0	0	0
0	0	0	0	0	0	4	0	0	0	0	0	0	0
0	0	0	0	0	0	0	0	0	0	0	0	0	0
0	0	0	0	1	0	0	0	0	0	1	0	0	0
0	0	0	0	0	0	0	0	0	0	0	0	0	1
0	7	0	0	1	0	0	0	0	0	1	0	0	0
0	0	0	0	0	0	0	0	0	0	0	9	3	0
0	0	4	2	0	6	9	0	17	5	1	0	0	0
0													
OTU396	0	0	0	33	0	0	0	0	0	0	2	0	2
5	0	0	0	0	10	0	1	1	1	0	0	0	2
0	0	0	0	0	1	0	0	0	0	0	0	0	2

0	0	0	2	0	0	3	0	0	0	0	0	0	0
0	0	0	0	0	0	0	0	0	0	0	0	0	0
0	0	0	0	0	0	1	0	0	0	0	0	0	0
6	0	0	0	0	0	0	0	34	0	1	0	0	2
1	0	0	0	0	0	0	0	1	0	0	1	1	0
0													
OTU8083	0	0	0	1	0	0	0	0	0	0	0	0	0
0	1	0	0	0	0	0	0	0	0	0	0	0	1
0	0	0	0	0	0	0	0	0	0	0	0	0	0
0	0	0	0	0	0	0	0	0	0	0	0	0	0
0	0	0	0	0	0	0	0	0	0	0	0	0	0
0	0	0	0	0	0	0	0	0	0	0	0	0	0
1	0	0	0	0	0	0	0	0	0	0	0	0	0
0	0	0	0	0	0	0	0	0	0	0	0	0	0
0													
OTU263	0	0	0	30	70	0	0	0	1	1	0	1	0
6	0	0	0	53	2	0	0	0	0	0	0	0	0
0	0	1	0	0	2	0	0	0	0	3	0	1	18
1	0	0	0	0	1	0	1	8	0	0	0	0	0
0	0	0	2	0	4	0	0	0	0	0	0	0	0
0	0	0	0	0	9	2	0	0	0	0	0	0	0
2	0	0	0	1	0	0	0	9	0	0	0	0	0
0	0	0	1	0	0	2	3	0	1	0	0	0	1
0													
OTU14554	0	0	0	1	0	0	0	0	0	0	0	0	0
0	0	0	0	0	0	0	0	0	0	0	0	0	0
0	0	0	0	0	0	0	0	0	0	0	0	0	0
0	0	0	0	0	0	0	0	0	0	0	0	0	0
0	0	0	0	0	0	0	0	0	0	0	0	0	0
0	0	0	0	0	0	0	0	0	0	0	0	0	0
0	0	0	0	0	0	0	0	0	0	0	0	0	0
0	0	0	0	0	0	0	0	0	0	0	0	0	0
0													

OTU10770	0	0	0	1	0	0	0	0	0	0	0	0	0
0	0	0	0	0	0	0	0	0	0	0	0	0	0
0	0	0	0	0	0	0	0	0	0	0	0	0	0
0	0	0	0	0	0	0	0	0	0	0	0	0	0
0	0	0	0	0	0	0	0	0	0	0	0	0	0
0	0	0	0	0	0	0	0	0	0	0	0	0	0
0	0	0	0	0	0	0	0	0	0	0	0	0	0
0	0	0	0	0	0	0	0	0	0	0	0	0	0
0													
OTU5457	0	0	0	4	1	1	0	6	6	0	2	0	1
2	2	6	1	0	0	0	4	0	0	0	1	2	6
0	0	0	5	2	0	13	1	1	0	0	0	0	0
4	4	0	0	5	5	0	1	0	0	1	0	0	0
0	4	4	4	0	0	1	0	0	0	0	2	1	1
0	0	0	5	0	0	2	0	0	1	0	0	7	0
0	0	3	1	0	0	12	0	3	0	3	0	0	1
1	0	1	0	2	0	0	2	0	0	1	4	0	0
0													
OTU483	0	0	0	1	0	0	0	0	0	0	0	0	0
0	0	3	0	1	0	0	1	0	0	0	2	0	0
0	0	0	12	0	4	0	0	0	3	0	0	0	0
0	3	0	2	0	0	0	0	0	0	1	0	0	0
0	0	0	0	0	0	0	0	0	0	1	1	0	0
0	0	0	0	0	1	1	0	0	0	0	0	1	0
0	0	0	0	0	0	0	0	0	0	0	0	0	0
0	0	0	0	0	0	0	0	0	0	0	0	0	0
0													
OTU5521	0	0	0	6	2	0	0	0	0	1	44	16	20
20	5	6	0	13	1	12	1	0	0	0	1	1	1
0	0	4	2	0	26	0	32	22	3	0	18	0	3
0	14	0	4	0	0	38	2	0	0	6	0	0	21
0	1	2	4	0	0	0	6	0	0	0	3	1	0
0	0	1	10	1	3	11	0	22	0	4	0	10	1

54	0	12	0	65	3	0	0	19	4	37	3	0	12
32	1	0	0	0	0	0	0	1	1	26	21	0	0
0													
OTU1837	0	0	0	1	0	0	0	0	1	0	0	1	0
0	0	1	1	1	0	0	0	15	0	0	0	0	0
0	0	0	0	1	0	2	1	0	1	0	0	0	0
0	0	0	0	8	8	1	0	1	0	1	0	2	0
8	1	10	2	2	1	6	1	2	20	0	7	2	6
2	0	0	1	0	0	1	0	0	0	0	0	0	0
1	3	0	0	0	0	5	0	0	0	4	0	0	0
2	0	0	0	0	0	0	0	1	0	0	9	1	0
0													
OTU148	0	0	0	1	0	0	1	0	0	0	30	2	180
337	0	5	1	4	1	0	0	0	0	1	0	0	1
0	0	11	1	0	0	0	24	0	1	0	0	0	0
0	7	5	0	0	0	6	0	0	0	0	0	0	0
0	1	1	0	0	0	0	0	0	0	0	0	5	0
31	0	2	12	49	14	33	1	3	24	1	17	3	20
4	393	32	25	5	17	92	1	295	4	7	0	0	550
5	2	0	0	0	0	0	0	0	0	1	8	51	0
0													
OTU15902	0	0	0	1	0	0	0	0	0	0	4	1	1
18	0	1	1	0	15	0	0	0	0	0	0	0	0
0	0	13	3	0	0	0	2	0	0	0	0	0	3
0	0	14	1	3	0	1	0	3	0	0	0	4	0
3	0	4	0	0	0	3	1	1	3	1	1	0	1
3	0	0	0	0	0	1	0	0	1	0	0	0	0
0	1	0	0	0	0	0	0	16	1	0	0	0	2
2	1	0	0	0	0	0	0	0	0	0	0	2	0
0													
OTU583	0	0	0	4	1	4	0	0	0	0	0	0	0
0	0	0	0	2	0	0	0	0	0	0	1	0	0
1	0	2	1	0	0	0	0	0	0	0	0	1	2

6	0	0	0	0	0	0	4	0	0	0	0	0	0
0	4	0	3	0	0	0	0	0	0	0	0	2	0
0	0	0	1	0	2	0	0	0	1	3	0	0	2
1	0	0	0	1	0	0	0	0	0	0	0	1	0
0	0	0	0	0	0	0	0	0	0	0	1	0	0
0													
OTU7709	0	0	0	1	0	0	0	0	0	0	0	0	0
1	0	0	0	0	0	0	0	0	0	0	1	0	0
0	0	0	0	0	0	0	0	0	0	0	0	0	0
1	0	0	1	0	0	0	0	0	0	0	0	0	0
0	0	1	1	0	0	0	0	0	1	0	0	1	0
0	0	2	0	0	0	0	0	0	0	3	0	0	1
0	0	0	0	0	0	0	0	0	0	0	1	1	1
0	0	0	0	0	2	0	0	1	1	1	1	0	0
0													
OTU14555	0	0	0	1	0	1	0	0	0	0	0	0	0
0	0	0	0	0	0	0	0	0	0	0	0	0	0
0	0	0	0	0	0	0	0	0	0	0	0	0	0
0	0	0	0	0	0	0	0	0	0	0	0	0	0
0	0	0	0	0	0	0	0	0	0	0	0	0	0
0	0	0	0	0	0	0	0	0	0	0	0	0	0
0	0	0	0	0	0	0	0	0	0	0	0	0	0
0	0	0	0	0	0	0	0	0	0	0	0	0	0
0													
OTU8354	0	0	0	1	0	0	0	0	0	0	0	0	0
0	0	0	0	0	0	0	0	0	0	0	0	0	0
0	0	0	0	0	0	0	0	0	0	0	0	0	0
0	0	0	0	0	0	0	0	0	0	0	0	0	0
0	0	0	0	0	0	0	0	0	0	0	0	0	0
0	0	0	0	0	0	0	0	0	0	0	0	0	0
0	0	0	0	0	0	0	0	0	0	0	0	0	0
0	0	0	0	0	0	0	0	0	0	0	0	0	0
0													

OTU14580 0 0 0 1 0 0 0 0 0 0 0 0 0
0 0 0 0 0 0 0 0 0 0 0 0 0 0
0 0 0 0 0 0 0 0 0 0 0 0 0 0
0 0 0 0 0 0 0 0 0 0 0 0 0 0
0 0 0 0 0 0 0 0 0 0 0 0 0 0
0 0 0 0 0 0 0 0 0 0 0 0 0 0
0 0 0 0 0 0 0 0 0 0 0 0 0 0
0 0 0 0 0 0 0 0 0 0 0 0 0 0
0
OTU10777 0 0 0 2 5 0 0 0 0 0 0 0 0
0 0 0 0 0 0 0 0 0 0 1 0 0 0
0 0 0 0 0 0 0 0 0 0 0 0 0 0
0 0 1 0 0 0 0 0 0 0 0 0 0 0
0 0 0 0 1 0 0 0 0 0 0 0 0 0
0 0 0 0 0 0 0 0 0 0 0 0 0 0
0 0 0 0 0 0 0 0 0 0 0 0 0 0
0 0 0 0 0 0 0 0 0 0 0 0 0 0
0
OTU404 0 0 0 2 1 2 0 0 0 0 0 0 0
1 1 0 0 0 0 1 1 2 0 0 0 0 0
5 0 0 0 0 0 1 0 3 1 0 0 4 0
0 1 0 0 2 6 0 0 2 0 2 1 0 0
2 5 0 2 2 1 0 0 0 0 1 2 5 0
0 0 0 1 2 0 0 0 0 2 0 0 0 0
0 0 0 1 0 0 1 0 0 2 1 0 3 0
1 0 0 0 0 0 2 0 0 0 1 0 2 2
0
OTU2424 0 0 0 4 0 0 0 0 0 0 0 0 0
0 0 0 0 0 0 0 0 0 0 0 0 0 0
0 0 0 0 0 0 0 0 1 0 0 0 0 1
0 0 0 0 1 0 0 0 0 0 0 0 0 0
0 0 0 0 0 0 0 0 0 0 0 0 0 0
0 2 1 0 0 0 0 0 0 0 0 0 0 0

0	0	0	0	0	0	0	1	0	4	0	0	0	0
0	0	2	0	0	1	0	1	0	0	0	0	3	0
0													
OTU4532	0	0	0	2	0	0	0	1	0	0	0	0	0
0	1	0	0	0	0	0	1	0	0	0	0	0	0
0	0	0	0	0	0	0	0	0	0	0	0	0	0
0	0	0	0	0	0	0	0	0	0	0	0	0	0
0	0	0	0	0	0	0	0	0	0	0	0	0	0
0	0	0	0	0	0	0	1	0	0	0	0	0	0
0	0	0	0	1	0	0	0	0	0	1	0	0	0
0	0	1	0	0	0	0	0	0	0	0	0	0	0
0													
OTU13122	0	0	0	2	0	0	0	1	1	0	1	1	0
1	0	0	7	0	0	0	0	2	0	0	0	0	0
0	4	9	0	16	0	4	0	0	1	0	0	0	0
0	0	26	0	2	9	1	2	26	0	1	1	41	1
67	2	17	2	0	6	57	7	2	11	6	2	6	5
11	0	0	0	0	0	0	0	0	0	1	0	0	0
0	1	0	0	0	0	0	0	5	0	2	0	0	49
3	5	27	0	11	16	3	1	26	1	0	0	1	1
0													
OTU12262	0	0	0	1	0	0	0	0	0	0	0	0	0
0	0	0	0	0	0	0	0	0	0	0	0	0	0
0	0	0	0	0	0	0	0	0	0	0	0	0	0
0	0	0	0	0	0	0	0	0	0	0	0	0	0
0	0	0	0	0	0	0	0	0	0	0	0	0	0
0	0	0	0	0	0	0	0	0	0	0	0	0	0
0	0	0	0	0	0	0	0	0	0	0	0	0	0
0	0	0	0	0	0	0	0	0	0	0	0	0	0
0													
OTU6118	0	0	0	1	0	0	0	0	0	0	0	0	0
0	0	0	0	0	0	0	0	0	0	0	0	0	0
0	0	0	0	0	0	0	0	0	0	0	0	0	0

0	0	0	0	0	0	0	0	0	0	0	0	0	0
0	0	0	0	0	0	0	0	0	0	0	1	0	0
0	0	0	0	0	0	0	0	0	0	0	0	0	0
1	0	0	0	0	0	0	0	0	0	0	0	0	0
0	0	0	0	0	0	0	0	0	0	0	0	0	0
0													
OTU8756	0	0	0	2	0	0	0	0	0	0	0	0	0
0	0	0	0	0	0	0	0	0	0	0	0	0	0
0	0	0	0	0	0	1	0	0	0	0	0	0	0
0	0	0	1	0	0	0	0	0	0	0	0	0	0
0	0	0	0	0	0	0	0	0	0	0	1	0	0
0	0	0	0	0	0	0	0	0	0	0	0	0	0
0	0	0	0	0	0	0	0	0	0	0	0	0	0
0	0	0	0	0	0	0	0	0	0	0	0	0	0
0													
OTU8347	0	0	0	1	0	0	0	0	0	0	0	0	0
0	0	0	0	0	0	0	0	0	0	0	0	0	0
0	0	0	0	0	0	0	0	0	0	0	0	0	0
0	0	0	0	0	0	0	0	0	0	0	0	0	0
0	0	0	0	0	0	0	0	0	0	0	0	0	0
0	0	0	0	0	0	0	0	0	0	0	0	0	0
0	0	0	0	0	0	0	0	0	0	0	0	0	0
0	0	0	0	0	0	0	0	0	0	0	0	0	0
0													
OTU519	0	0	0	3	0	0	0	0	7	0	0	0	0
1	0	0	0	0	0	1	2	0	0	0	0	0	0
0	1	0	0	0	0	1	0	0	0	0	0	1	0
0	1	2	0	2	0	0	0	0	0	0	5	0	0
0	0	0	1	0	0	0	0	0	0	0	0	1	0
1	1	0	0	0	0	0	0	0	0	0	0	0	0
0	0	0	0	0	0	5	0	0	0	0	0	0	0
0	0	2	6	2	0	1	0	1	7	0	0	0	4
0													

OTU334 0 0 0 2 0 0 1 2 1 0 1 0 2
2 0 2 0 4 3 3 5 0 0 20 26 0 15
0 1 0 0 13 0 4 2 0 0 0 2 2 0
0 0 0 0 2 0 0 1 0 0 4 0 0 6
0 6 4 0 1 0 0 2 0 4 0 7 0 5
0 0 0 2 0 0 3 0 2 4 5 0 0 0
7 0 6 3 1 0 0 0 14 0 2 0 0 7
1 0 0 0 0 0 0 0 1 0 9 3 0 0
0
OTU4854 0 0 0 1 2 0 0 0 0 0 0 0 0
0 0 0 0 0 0 0 0 0 0 0 0 0 0
0 0 0 0 0 0 0 0 0 0 0 0 0 0
0 0 0 0 0 0 0 0 0 0 0 0 0 0
0 0 0 0 0 0 0 0 0 0 0 0 0 0
0 0 0 0 0 0 0 0 0 0 0 0 0 0
0 0 0 0 0 0 0 0 0 0 0 0 0 0
0 0 0 0 0 0 0 0 0 0 0 1 0 0
0
OTU14581 0 0 0 1 0 0 0 0 0 0 0 0 0
0 0 0 0 0 0 0 0 0 0 0 0 0 0
0 0 0 0 0 0 0 0 0 0 0 0 0 0
0 0 0 0 0 0 0 0 0 0 0 0 0 0
0 0 0 0 0 0 0 0 0 0 0 0 0 0
0 0 0 0 0 0 0 0 0 0 0 0 0 0
0 0 0 0 0 0 0 0 0 0 0 0 0 0
0 0 0 0 0 0 0 0 0 0 0 0 0 0
0
OTU12261 0 0 0 1 0 1 0 0 0 0 0 0 0
0 0 0 0 0 0 0 0 0 0 0 0 0 0
0 0 0 0 0 0 0 0 0 0 0 0 0 0
0 0 0 0 0 0 0 0 0 0 0 0 0 0
0 0 0 0 0 0 0 0 0 0 0 0 0 0
0 0 0 0 0 0 0 0 0 0 0 0 0 0

0	0	0	0	0	0	0	0	0	0	0	0	0	0
0	0	0	0	0	0	0	0	0	0	0	0	0	0
0													
OTU702	0	0	0	1	0	0	0	0	1	1	0	1	0
1	0	1	0	0	0	0	0	1	0	0	0	0	0
0	0	1	1	0	1	0	1	0	1	0	1	0	0
0	0	2	0	0	3	1	0	0	0	0	0	0	1
0	0	0	0	0	0	0	0	0	0	0	0	1	0
0	1	0	0	0	1	0	0	0	0	0	0	0	0
0	0	1	0	0	0	0	0	0	0	0	0	0	0
0	0	0	0	0	0	0	0	0	0	0	0	1	0
0													
OTU8348	0	0	0	1	0	0	0	0	0	0	0	0	0
0	0	0	0	0	0	0	0	0	0	0	0	0	0
0	0	0	0	0	0	0	0	0	0	0	0	0	0
0	0	0	0	0	0	0	0	0	0	0	0	0	0
0	0	0	0	0	0	0	0	0	0	0	0	0	0
0	0	0	0	0	0	0	0	0	0	0	0	0	0
0	0	0	0	0	0	0	0	0	0	0	0	0	0
0	0	0	0	0	0	0	0	0	0	0	0	0	0
0													
OTU10778	0	0	0	1	0	0	0	0	0	0	0	0	0
0	0	0	0	0	0	0	0	0	0	0	0	0	0
0	0	0	0	0	0	0	0	0	0	0	0	0	0
0	0	0	0	0	0	0	0	0	0	0	0	0	0
0	0	0	0	0	0	0	0	0	0	0	0	0	0
0	0	0	0	0	0	0	0	0	0	0	0	0	0
0	0	0	0	0	0	0	0	0	0	0	0	0	0
0	0	0	0	0	0	0	0	0	0	0	0	0	0
0													
OTU6975	0	0	0	5	0	0	0	4	3	0	7	0	4
16	0	3	19	7	5	0	5	13	0	0	2	0	1
0	9	12	3	32	0	6	8	0	2	0	0	0	2

16	0	5	1	2	42	7	3	23	0	4	0	76	0
77	4	2	1	0	7	65	8	8	8	3	4	14	4
7	1	0	0	0	2	4	0	0	0	9	0	0	0
0	0	0	0	0	0	0	0	8	0	0	0	4	63
7	2	130	1	67	75	20	0	149	3	0	3	10	4
0													
OTU10774	0	0	0	1	0	0	0	0	0	0	0	0	0
0	0	0	0	0	0	0	0	0	0	0	0	0	0
0	0	0	0	0	0	0	0	0	0	0	0	0	0
0	0	0	0	0	0	0	0	0	0	0	0	0	0
0	0	0	0	0	0	0	0	0	0	0	0	0	0
0	0	0	0	0	0	0	0	0	0	0	0	0	0
0	0	0	0	0	0	0	0	0	0	0	0	0	0
0	0	0	0	0	0	0	0	0	0	0	0	0	0
0													
OTU567	0	0	0	1	0	0	0	0	0	0	1	0	7
1	0	15	7	0	11	0	0	0	0	0	0	0	6
1	0	0	19	0	2	2	1	0	1	0	0	0	2
0	0	0	3	3	0	0	0	1	0	0	0	2	1
3	3	0	1	0	1	0	0	1	0	0	0	0	0
1	0	1	0	0	0	0	0	0	0	0	1	0	0
0	2	1	0	0	0	0	0	1	0	0	0	0	0
0	0	0	0	0	0	0	0	0	0	0	11	0	0
0													
OTU15835	0	0	0	1	0	1	2	2	0	0	0	0	0
0	0	0	0	0	0	0	0	0	0	0	0	0	1
0	0	0	0	0	0	0	0	1	0	0	0	0	0
0	0	0	0	0	3	0	0	1	1	0	0	0	0
0	0	0	0	0	0	0	0	0	0	0	1	1	0
0	0	0	0	0	0	0	0	0	0	0	0	0	0
3	0	0	0	0	0	0	2	0	0	0	0	0	0
0	0	0	0	0	1	0	0	0	0	0	0	0	0
0													

OTU2442 0 0 0 2 0 0 2 0 1 0 0 0 0
0 0 0 0 1 0 3 7 0 0 0 0 0 0
3 1 0 0 2 0 0 0 0 1 3 0 0 0
0 1 0 3 0 0 0 5 0 0 0 0 0 0
0 7 0 2 0 0 0 0 0 0 0 0 1 0
0 0 2 0 0 0 0 0 0 0 0 1 0 0
0 0 0 0 0 0 0 0 0 0 0 1 0 0
0 4 3 1 2 2 1 1 4 3 0 0 0 0
2
OTU1449 0 0 0 1 0 0 0 0 1 0 0 0 0
0 0 0 0 0 0 0 0 0 0 0 0 0 0
0 0 0 0 0 0 0 0 0 0 4 0 0 0
0 0 0 0 0 0 0 1 0 0 0 0 0 0
0 1 0 0 0 0 0 0 0 0 0 0 0 0
0 8 2 0 0 0 0 0 0 0 1 0 0 0
1 0 0 0 0 0 0 0 0 0 0 0 5 0
0 5 26 5 2 13 15 1 16 4 0 0 0 18
5
OTU10775 0 0 0 1 0 0 0 0 0 0 0 0 0
0 0 0 0 0 0 0 0 0 0 0 0 0 0
0 0 0 0 0 0 0 0 0 0 0 0 0 0
0 0 0 0 0 0 0 0 0 0 0 0 0 0
0 0 0 0 0 0 0 0 0 0 0 0 0 0
0 0 0 0 0 0 0 0 0 0 0 0 0 0
0 0 0 0 0 0 0 0 0 0 0 0 0 0
0 0 0 0 0 0 0 0 0 0 0 0 0 0
0
OTU8346 0 0 0 1 0 0 0 0 0 0 0 0 0
0 0 0 0 0 0 0 0 0 0 0 0 0 0
0 0 0 0 0 0 0 0 0 0 0 0 0 0
0 0 0 0 0 0 0 0 0 0 0 0 0 0
0 0 0 0 0 0 0 0 0 0 0 0 0 0
0 0 0 0 0 0 0 0 0 0 0 0 0 0

0	0	0	0	0	0	0	0	0	0	0	0	0	0
0	0	0	0	0	0	0	0	0	0	0	0	0	0
0													
OTU10944	0	0	0	1	0	0	0	0	0	0	0	0	1
0	0	0	0	0	0	0	0	0	0	0	0	0	0
0	0	0	0	0	0	0	0	0	0	0	0	0	0
0	0	0	0	0	0	0	0	0	0	0	0	0	0
0	0	0	0	0	0	0	0	0	0	0	0	0	0
0	0	0	0	0	0	0	0	0	0	0	0	0	0
0	0	0	0	0	0	0	0	0	0	0	0	0	0
0	0	0	0	0	0	0	0	0	0	0	0	1	0
0													
OTU11316	0	0	0	1	0	1	0	0	0	0	0	0	0
0	0	0	0	0	0	0	0	0	0	0	0	0	0
0	0	0	0	0	0	0	0	0	0	0	0	0	0
0	1	0	0	0	0	0	0	1	1	0	0	0	0
0	0	0	1	0	0	0	0	0	0	0	0	0	0
0	0	0	0	0	0	0	0	0	0	0	0	0	0
0	0	0	0	0	0	0	0	0	0	0	0	0	0
0	0	0	0	1	1	1	0	0	0	0	0	0	0
0													
OTU14539	0	0	0	1	1	0	0	0	1	0	0	0	0
0	0	0	0	0	0	0	0	0	0	0	0	0	0
0	0	0	0	0	0	0	0	0	0	0	0	2	0
0	0	0	0	0	0	0	1	0	0	0	0	0	0
0	0	0	1	0	0	0	0	0	0	0	0	0	0
0	0	0	0	0	0	0	1	0	0	0	0	0	1
0	0	0	0	0	2	2	1	0	0	0	0	0	0
0	0	0	2	0	0	0	0	0	0	0	0	0	0
0													
OTU2744	0	0	0	3	0	0	0	0	0	0	15	6	11
6	1	2	0	8	0	3	2	0	0	0	1	1	0
0	0	2	1	0	7	0	25	20	0	0	12	0	0

0 12 0 5 0 2 27 3 0 0 1 0 0 10
0 1 1 1 1 0 0 1 0 0 0 2 0 0
0 1 0 5 0 1 1 0 17 0 0 0 6 1
31 0 6 0 42 0 1 1 9 0 17 2 0 6
17 1 0 0 0 0 0 0 2 1 8 11 1 0
0
OTU14952 0 0 0 1 0 0 0 0 0 0 0 0 0
0 0 0 0 0 0 0 0 0 1 0 0 0 0
0 0 0 0 0 0 0 0 0 0 0 0 0 0
0 0 0 0 0 0 0 0 0 0 0 0 0 0
0 0 0 0 0 0 0 1 0 0 0 0 0 0
0 0 0 0 0 0 0 0 0 0 0 0 0 0
0 0 0 0 0 0 0 0 0 0 0 1 0 0
0 0 0 0 0 0 0 0 0 1 0 0 0 0
0
OTU8384 0 0 0 1 2 2 0 0 1 0 1 0 0
0 1 0 0 2 1 0 1 1 0 2 0 1 0
0 0 0 0 0 0 0 3 1 0 1 1 0 0
2 0 1 1 0 0 0 0 0 1 1 0 2 2
0 0 2 1 0 1 0 0 0 1 2 1 1 0
0 0 0 0 0 1 0 1 0 0 0 1 0 1
0 0 1 0 0 2 1 0 0 0 1 0 1 0
0 0 0 0 0 0 0 1 0 0 0 0 0 0
3
OTU1976 0 0 0 1 0 0 0 0 0 0 1 0 0
0 0 1 0 1 3 0 0 0 0 0 0 0 0
0 0 0 1 0 0 0 1 0 0 0 0 0 2
0 0 1 0 2 0 0 0 0 0 0 0 1 0
1 0 2 0 0 0 2 0 0 0 0 0 0 0
0 0 0 0 0 0 1 0 0 0 0 0 0 0
0 0 0 0 0 0 0 0 2 0 0 0 0 1
1 0 0 0 0 0 0 0 0 0 0 2 0 0
0

OTU549 0 0 0 8 0 1 1 1 3 0 0 1 0
0 0 2 3 0 0 0 1 1 0 1 1 0 0
0 4 0 1 0 0 0 0 1 1 0 0 4 0
1 0 0 11 0 0 0 0 1 2 1 0 3 0
0 0 1 1 0 0 4 2 2 0 0 3 1 1
0 5 0 6 0 0 0 0 3 0 0 2 1 0
0 0 0 0 0 0 33 2 0 2 0 3 5 0
11 2 2 2 4 6 2 8 3 4 1 0 3 4
6
OTU1304 0 0 0 2 0 0 0 0 0 0 0 0 0
0 0 0 0 0 0 1 0 0 0 0 0 0 0
0 0 0 0 0 0 0 0 0 0 0 0 2 0
0 0 0 0 0 0 0 0 0 0 0 0 0 0
0 0 0 0 0 0 0 0 0 0 0 0 0 0
0 0 0 0 4 0 0 0 0 2 0 0 0 0
0 0 0 0 0 0 0 0 0 0 0 0 0 0
0 0 0 0 0 0 0 0 0 0 0 0 0 0
0
OTU5745 0 0 0 2 0 0 0 0 0 0 0 0 0
0 0 0 0 0 0 1 0 0 0 0 0 0 0
0 0 0 0 0 0 0 0 0 1 0 0 1 0
1 0 0 0 0 0 0 0 0 0 1 0 0 0
0 0 0 0 0 0 0 2 0 1 0 0 0 0
0 0 0 0 0 0 0 0 1 0 0 0 0 0
0 0 0 0 0 0 0 0 0 0 0 0 0 0
0 0 0 0 0 0 0 0 0 0 0 0 1 0
2
OTU10776 0 0 0 1 0 0 0 0 0 0 0 0 0
0 0 0 0 0 0 0 0 0 0 0 0 0 0
0 0 0 0 0 0 0 0 0 0 0 0 0 0
0 0 0 0 0 0 0 0 0 0 0 0 0 0
0 0 0 0 0 0 0 0 0 0 0 0 0 0
0 0 0 0 0 0 0 0 0 0 0 0 0 0

0	0	0	0	0	0	0	0	0	0	0	0	0	0
0	0	0	0	0	0	0	0	0	0	0	0	0	0
0													
OTU12265	0	0	0	3	1	0	0	0	0	0	0	0	0
0	1	0	1	0	0	0	0	0	0	0	0	0	0
0	0	0	0	0	0	0	0	0	0	0	0	0	0
0	0	0	0	0	0	0	1	0	0	0	0	0	0
0	3	0	0	0	0	0	0	0	0	0	0	0	0
0	0	0	0	0	0	0	0	0	0	0	0	0	0
0	0	0	0	0	0	0	0	0	0	0	0	0	0
0	0	0	0	0	0	0	0	0	0	0	2	0	0
0													
OTU13619	0	0	0	1	1	0	0	0	1	0	0	0	0
0	0	0	0	0	0	0	0	0	0	0	0	0	0
0	0	0	0	0	1	0	0	0	0	0	0	0	1
0	0	0	0	0	0	0	0	0	0	0	0	0	0
0	0	0	0	0	0	0	0	0	0	0	0	1	0
0	0	0	0	2	0	0	1	0	0	0	1	0	0
0	0	0	0	0	0	0	0	0	0	0	0	1	0
0	1	0	0	0	0	0	0	0	0	0	0	0	0
0													
OTU2706	0	0	0	2	0	0	0	0	0	0	0	0	0
0	0	0	0	0	0	0	4	0	0	0	0	0	0
0	0	0	0	0	0	0	0	0	0	0	0	1	0
0	0	0	0	0	0	0	0	0	0	0	0	0	0
0	0	0	0	0	0	0	0	0	0	0	0	0	0
0	0	0	0	0	0	0	0	0	0	0	0	0	0
0	0	0	0	0	0	0	0	0	0	0	0	1	0
0	1	0	1	1	1	0	1	0	0	0	0	0	0
0													
OTU956	0	0	0	2	0	0	0	0	0	0	0	0	0
0	0	0	0	0	0	0	0	0	0	0	0	0	0
0	0	0	0	0	0	0	0	0	0	0	0	0	0

0	0	0	0	0	0	0	0	0	0	0	0	0	0
0	0	0	0	0	0	0	0	0	0	0	0	0	0
0	0	0	0	0	0	0	0	0	0	0	0	0	0
0	0	0	0	0	0	0	0	0	0	0	0	0	0
0	0	0	0	0	0	0	0	0	0	0	0	0	0
0													
OTU655	0	0	0	1	2	0	0	0	0	1	0	1	0
0	0	0	0	0	0	0	2	0	0	0	0	0	0
0	0	0	0	0	0	0	0	0	0	0	0	0	0
3	5	0	3	1	0	0	0	0	0	0	0	0	0
0	0	0	0	0	0	0	0	0	0	0	0	0	0
0	5	0	1	0	0	1	0	5	1	0	0	0	0
1	0	0	0	1	1	1	0	0	0	2	1	0	1
0	3	1	0	7	3	0	2	0	0	0	2	0	0
0													
OTU14586	0	0	0	1	0	0	0	0	0	0	0	0	0
0	0	0	0	0	0	0	0	0	0	0	0	0	0
0	0	0	0	0	0	0	0	0	0	0	0	0	0
0	0	0	0	0	0	0	0	0	0	0	0	0	0
0	0	0	0	0	0	0	0	0	0	0	0	0	0
0	0	0	0	0	0	0	0	0	0	0	0	0	0
0	0	0	0	0	0	0	0	0	0	0	0	0	0
0	0	0	0	0	0	0	0	0	0	0	0	0	0
0													
OTU2533	0	0	0	7	0	0	0	0	0	0	0	0	0
0	0	0	0	0	0	0	0	0	0	0	0	0	0
0	0	0	0	0	0	1	0	0	0	0	0	0	0
0	0	0	0	0	0	0	0	0	0	0	0	0	0
0	0	0	0	0	1	0	0	0	0	3	1	0	0
0	0	0	0	0	0	0	0	0	0	0	0	0	0
0	0	0	0	0	0	7	0	0	0	0	0	0	0
0	0	0	0	0	0	0	0	0	0	0	0	0	0
0													

OTU12249	0	0	0	1	0	0	0	0	0	0	0	0	0
0	0	0	0	0	0	0	0	0	0	0	0	0	0
0	0	0	0	0	0	0	0	0	0	0	0	0	0
0	0	0	0	0	0	0	0	0	0	0	0	0	0
0	0	0	0	0	0	0	0	0	0	0	0	0	0
0	0	0	0	0	0	0	0	0	0	0	0	0	0
0	0	0	0	0	0	0	0	0	0	0	0	0	0
0	0	0	0	0	0	0	0	0	0	0	0	0	0
0													
OTU6778	0	0	0	1	0	0	0	0	0	0	0	0	0
0	0	2	0	1	0	0	0	0	0	0	0	0	0
0	0	0	1	0	0	0	0	0	0	0	0	0	0
0	0	0	0	0	0	0	1	0	0	0	0	0	0
0	0	0	1	0	1	0	0	0	0	0	0	0	0
0	0	0	0	0	0	0	0	0	0	0	0	0	0
0	0	0	0	0	0	0	0	0	1	0	0	0	0
1	0	6	0	0	0	1	1	0	0	0	0	0	0
0													
OTU10908	0	0	0	1	0	0	0	0	0	0	0	0	0
0	0	0	0	0	0	0	0	0	0	0	0	0	0
0	0	0	0	0	0	0	0	0	0	0	0	0	0
0	0	0	0	0	0	0	0	0	0	0	0	0	0
0	0	0	0	0	0	0	0	0	0	0	0	0	0
0	0	0	0	0	0	0	0	0	0	0	0	0	0
0	0	0	0	0	0	0	0	0	0	0	0	0	0
0	0	0	0	0	0	0	0	0	0	0	0	0	0
0													
OTU14584	0	0	0	1	0	0	0	0	0	0	0	0	0
0	0	0	0	0	0	0	0	0	0	0	0	0	0
0	0	0	0	0	0	0	0	0	0	0	0	0	0
0	0	0	0	0	0	0	0	0	0	0	0	0	0
0	0	0	0	0	0	0	0	0	0	0	0	0	0
0	0	0	0	0	0	0	0	0	0	0	0	0	0

0	0	0	0	0	0	0	0	0	0	0	0	0	0
0	0	0	0	0	0	0	0	0	0	0	0	0	0
0													
OTU12250	0	0	0	1	0	0	0	0	0	0	0	0	0
0	0	0	0	0	0	0	0	0	0	0	0	0	0
0	0	0	0	0	0	0	0	0	0	0	0	0	0
0	0	0	0	0	0	0	0	0	0	0	0	0	0
0	0	0	0	0	0	0	0	0	0	0	0	0	0
0	0	0	0	0	0	0	0	0	0	0	0	0	0
0	0	0	0	0	0	0	0	0	0	0	0	0	0
0	0	0	0	0	0	0	0	0	0	0	0	0	0
0													
OTU1528	0	0	0	2	0	1	1	2	0	0	0	1	0
0	1	0	0	1	2	0	0	0	0	0	0	0	1
3	0	0	5	0	0	0	0	0	1	1	0	0	1
1	0	0	0	0	0	1	0	0	0	0	0	0	0
0	0	0	2	0	1	0	0	0	1	0	0	0	0
0	0	5	1	0	4	1	1	3	0	0	0	5	0
0	2	0	1	2	0	0	0	0	1	0	0	1	1
2	1	0	0	0	0	0	0	0	0	0	0	0	0
0													
OTU11363	0	0	0	1	0	0	0	0	0	0	0	0	0
0	0	0	1	0	0	0	0	0	0	0	0	0	0
1	0	0	0	0	0	0	0	0	0	0	0	0	0
0	0	1	0	0	5	0	0	2	0	0	1	0	0
24	1	0	1	0	4	16	4	5	2	2	0	4	1
2	1	0	0	0	0	0	0	0	0	1	0	0	0
0	0	0	0	1	0	0	0	0	0	0	0	1	0
0	0	2	0	12	6	5	1	17	1	0	1	0	0
0													
OTU14585	0	0	0	1	0	0	0	0	0	0	0	0	0
0	0	0	0	0	0	0	0	0	0	0	0	0	0
0	0	0	0	0	0	0	0	0	0	0	0	0	0

0 0 0 0 0 0 0 0 0 0 0 0 0 0
0 0 0 0 0 0 0 0 0 0 0 0 0 0
0 0 0 0 0 0 0 0 0 0 0 0 0 0
0 0 0 0 0 0 0 0 0 0 0 0 0 0
0 0 0 0 0 0 0 0 0 0 0 0 0 0
0

OTU4330 0 0 0 2 1 0 1 0 0 0 0 0 0
0 0 1 0 1 4 0 0 0 2 0 0 0 0
0 0 2 1 0 0 0 0 0 0 0 0 4 2
0 2 0 0 0 0 0 0 0 0 0 0 1 0
0 0 0 1 0 1 1 0 2 0 0 1 0 0
1 0 0 0 3 1 1 0 0 0 0 2 1 0
0 1 0 0 1 1 0 0 1 0 2 0 1 0
0 1 1 0 1 0 1 1 0 0 0 0 0 3
2

OTU9350 0 0 0 1 0 0 0 0 0 0 0 0 0
0 0 0 0 0 0 0 1 0 0 0 0 1 0
0 0 0 0 0 0 0 1 0 0 0 0 0 0
0 0 0 0 0 0 0 0 0 0 0 0 0 0
0 0 0 0 0 0 0 0 0 0 0 0 0 0
0 0 0 0 0 0 0 0 0 0 0 0 0 0
0 0 0 0 0 0 0 0 0 0 0 0 0 0
0 0 0 0 0 0 0 0 0 0 0 0 0 0
0

OTU419 0 0 0 6 0 0 0 2 17 0 0 0 0
0 0 0 0 0 0 2 0 0 0 0 0 0 0
16 0 0 0 0 0 0 0 1 0 0 0 0 0
2 0 0 0 0 2 0 1 0 0 0 1 0 2
0 1 0 7 0 0 0 0 0 0 0 0 28 0
1 5 1 0 19 0 0 0 0 0 1 0 4 9
3 0 0 0 0 0 0 0 0 0 0 1 31 0
0 10 1 8 17 1 0 24 0 30 0 2 0 9
2

OTU14587 0 0 0 1 0 0 0 0 0 0 0 0 0
0 0 0 0 0 0 0 0 0 0 0 0 0 0
0 0 0 0 0 0 0 0 0 0 0 0 0 0
0 0 0 0 0 0 0 0 0 0 0 0 0 0
0 0 0 0 0 0 0 0 0 0 0 0 0 0
0 0 0 0 0 0 0 0 0 0 0 0 0 0
0 0 0 0 0 0 0 0 0 0 0 0 0 0
0 0 0 0 0 0 0 0 0 0 0 0 0 0
0
OTU268 0 0 0 1 0 0 0 0 0 0 1 0 0
0 1 0 0 19 0 0 0 0 0 0 0 0 0
0 1 0 0 0 0 0 0 0 0 9 0 0 0
0 0 0 0 5 0 0 0 0 1 1 0 1 6
5 0 0 0 3 12 0 0 0 0 0 0 0 2
0 0 0 37 0 0 1 0 0 0 0 0 0 0
166 0 8 0 1 0 0 0 0 0 0 0 0 0
0 0 0 0 0 0 0 0 0 0 0 0 0 0
0
OTU12996 0 0 0 3 0 0 0 0 0 0 3 0 6
21 0 0 0 2 13 0 0 0 1 0 0 0 1
0 1 7 1 4 3 0 8 0 1 0 0 0 6
0 0 1 1 0 0 2 0 4 0 0 0 4 0
0 0 9 0 0 0 3 0 0 2 3 1 0 1
2 0 0 2 1 4 3 0 0 0 0 0 0 0
1 0 0 0 0 0 1 0 13 0 4 0 0 13
8 1 0 0 0 0 0 0 1 1 0 5 4 0
0
OTU14741 0 0 0 1 0 0 0 0 0 0 0 0 0
0 0 0 0 0 0 1 0 0 0 0 0 0 0
0 0 0 0 0 0 0 0 0 0 0 0 0 0
0 0 0 0 0 0 0 0 0 0 0 0 0 0
0 0 0 0 0 0 0 0 0 0 0 0 0 0
0 0 0 0 0 0 0 0 0 1 0 0 0 0

0	0	0	0	0	0	0	1	0	0	0	0	2	0
0	0	2	1	0	3	2	0	2	0	0	0	0	0
0													
OTU1786	0	0	0	3	2	2	0	0	0	1	0	13	0
0	0	1	0	0	0	0	3	0	0	0	1	0	0
0	0	0	0	0	0	0	0	1	5	2	0	8	0
0	9	0	0	0	2	0	0	2	0	1	0	0	0
0	0	0	17	0	0	0	0	0	0	0	0	13	0
0	1	0	0	2	0	0	0	0	0	0	3	0	6
1	1	0	2	0	0	0	2	0	5	1	0	3	0
0	7	0	6	4	2	2	4	7	6	0	0	0	11
3													
OTU14582	0	0	0	1	0	0	0	0	0	0	0	0	0
0	0	0	0	0	0	0	0	0	0	0	0	0	0
0	0	0	0	0	0	0	0	0	0	0	0	0	0
0	0	0	0	0	0	0	0	0	0	0	0	0	0
0	0	0	0	0	0	0	0	0	0	0	0	0	0
0	0	0	0	0	0	0	0	0	0	0	0	0	0
0	0	0	0	0	0	0	0	0	0	0	0	0	0
0	0	0	0	0	0	0	0	0	0	0	0	0	0
0													
OTU10904	0	0	0	3	0	0	0	0	0	0	0	0	0
0	0	0	0	0	0	0	2	0	0	0	0	0	0
0	0	0	0	0	0	0	0	0	0	0	0	0	0
0	0	0	0	0	0	0	0	0	0	0	0	0	0
0	0	0	2	0	0	0	0	0	0	0	0	0	0
1	0	0	1	0	0	0	0	0	0	0	1	0	0
0	0	0	0	0	0	0	0	0	0	0	0	0	0
0	0	0	0	0	0	0	0	0	0	0	0	0	0
0													
OTU12248	0	0	0	1	0	0	0	0	0	0	0	0	0
0	0	0	0	0	0	0	0	0	0	0	0	0	0
0	0	0	0	0	0	0	0	0	0	0	0	0	0

0	0	0	0	0	0	0	0	0	0	0	0	0	0
0	0	0	0	0	0	0	0	0	0	0	0	0	0
0	0	0	0	0	0	0	0	0	0	0	0	0	0
0	0	0	0	0	0	0	0	0	0	0	0	0	0
0	0	0	0	0	0	0	0	0	0	0	0	0	0
0													
OTU10907	0	0	0	1	0	0	0	0	0	0	0	0	0
0	0	0	0	0	0	0	0	0	0	0	0	0	0
0	0	0	0	0	0	0	0	0	0	0	0	0	0
0	0	0	0	0	0	0	0	0	0	0	0	0	0
0	0	0	0	0	0	0	0	0	0	0	0	0	0
0	0	0	0	0	0	0	0	0	0	0	0	0	0
0	0	0	0	0	0	0	0	0	0	0	0	0	0
0	0	0	0	0	0	0	0	0	0	0	0	0	0
0													
OTU14583	0	0	0	1	0	0	0	0	0	0	0	0	0
0	0	0	0	0	0	0	0	0	0	0	0	0	0
0	0	0	0	0	0	0	0	0	0	0	0	0	0
0	0	0	0	0	0	0	0	0	0	0	0	0	0
0	0	0	0	0	0	0	0	0	0	0	0	0	0
0	0	0	0	0	0	0	0	0	0	0	0	0	0
0	0	0	0	0	0	0	0	0	0	0	0	0	0
0	0	0	0	0	0	0	0	0	0	0	0	0	0
0													
OTU9026	0	0	0	1	0	0	0	0	0	0	0	0	0
0	1	0	0	1	0	0	0	0	0	0	1	0	0
0	0	1	0	0	0	0	0	0	0	0	1	0	1
0	0	0	0	0	0	0	0	0	2	0	0	0	0
0	0	0	0	0	0	0	1	0	0	0	0	0	0
0	0	0	0	1	0	0	1	0	0	0	2	1	1
0	0	0	0	1	1	0	0	1	0	0	1	0	0
0	0	0	0	0	0	0	0	0	0	0	0	0	0
0													

OTU10906	0	0	0	1	0	0	0	0	0	0	0	0	0
0	0	0	0	0	0	0	0	0	0	0	0	0	0
0	0	0	0	0	0	0	0	0	0	0	0	0	0
0	0	0	0	0	0	0	0	0	0	0	0	0	0
0	0	1	0	1	0	0	0	0	0	0	0	0	0
0	0	0	0	0	0	0	0	0	0	0	0	0	0
0	0	0	0	0	0	0	0	0	0	0	0	0	0
0	0	0	0	0	0	0	0	0	0	0	0	0	0
0													
OTU6989	0	0	0	1	0	0	0	0	0	0	0	0	0
0	0	0	0	0	0	0	0	0	0	0	0	1	0
1	0	0	0	0	0	0	0	0	0	0	0	0	0
0	0	0	0	0	0	0	0	0	0	0	0	0	0
0	0	0	0	0	0	0	0	0	0	0	0	0	0
0	0	0	0	0	0	0	0	0	0	0	0	0	0
0	0	0	0	0	0	0	0	0	0	0	0	0	0
1	0	0	0	0	0	0	0	0	0	0	0	0	0
0													
OTU1043	0	0	0	3	0	0	0	0	0	0	1	1	0
0	0	1	0	2	0	0	0	0	0	0	0	0	0
0	0	0	1	0	0	1	0	0	3	0	0	0	0
5	0	0	0	0	2	2	1	0	0	0	0	0	0
0	1	2	0	0	0	1	0	0	0	0	1	0	0
0	0	0	1	0	0	0	0	0	1	0	0	0	0
1	1	0	2	0	0	0	1	0	0	0	0	5	0
0	0	1	1	4	0	0	5	2	0	1	1	0	0
0													
OTU10905	0	0	0	1	0	0	0	0	0	0	0	0	0
0	0	0	0	0	0	0	0	0	0	0	0	0	0
0	0	0	0	0	0	0	0	0	0	0	0	0	0
0	0	0	0	0	0	0	0	0	0	0	0	0	0
0	0	0	0	0	0	0	0	0	0	0	0	0	0
0	0	0	0	0	0	0	0	0	0	0	0	0	0

0	0	0	0	0	0	0	0	0	0	0	0	0	0
0	0	0	0	0	0	0	0	0	0	0	0	0	0
0													
OTU8272	0	0	0	1	0	0	0	0	0	0	0	0	0
0	0	0	0	0	0	0	0	0	1	0	0	0	0
0	0	0	0	0	0	0	0	0	0	0	0	0	0
0	0	0	0	0	0	0	0	0	0	0	0	0	0
0	0	0	0	0	0	0	0	0	0	0	0	0	0
0	0	0	0	1	0	0	0	0	0	0	0	0	0
0	0	0	0	0	0	0	0	0	0	0	0	0	0
0	0	0	0	0	0	0	0	0	1	0	0	0	0
0													
OTU10912	0	0	0	1	0	0	0	0	0	0	0	0	0
0	0	0	0	0	0	0	0	0	0	0	0	0	0
0	0	0	0	0	0	0	0	0	0	0	0	0	0
0	0	0	0	0	0	0	0	0	0	0	0	0	0
0	0	0	0	0	0	0	0	0	0	0	0	0	0
0	0	0	0	0	0	0	0	0	0	0	0	0	0
0	0	0	0	0	0	0	0	0	0	0	0	0	0
0	0	0	0	0	0	0	0	0	0	0	0	0	0
0													
OTU12257	0	0	0	1	0	0	0	0	0	0	0	0	0
0	0	0	0	0	0	0	0	0	0	0	0	0	0
0	0	0	0	0	0	0	0	0	0	0	0	0	0
0	0	0	0	0	0	0	0	0	0	0	0	0	0
0	0	0	0	0	0	0	0	0	0	0	0	0	0
0	0	0	0	0	0	0	0	0	0	0	0	0	0
0	0	0	0	0	0	0	0	0	0	0	0	0	0
0	0	0	0	0	0	0	0	0	0	0	0	0	0
0													
OTU1148	0	0	0	2	0	0	0	1	2	0	0	0	0
0	0	0	0	0	0	1	0	0	0	0	0	2	0
0	0	0	0	0	0	0	0	1	0	0	0	0	0

0	0	0	0	0	1	0	0	0	0	0	0	0	0
0	0	0	0	0	0	0	0	0	0	0	0	0	0
0	0	0	0	0	0	0	0	0	0	2	0	0	0
0	0	0	0	0	0	2	0	0	0	0	0	0	0
0	0	0	0	0	0	0	0	0	0	0	0	0	0
0													
OTU16443	0	0	0	1	0	0	0	0	0	0	0	0	0
0	0	0	0	1	0	0	0	0	0	0	0	0	0
0	0	0	0	0	0	0	0	0	0	0	0	0	0
0	0	0	0	0	0	0	0	0	0	0	0	0	1
0	0	0	1	0	0	0	0	0	0	0	0	0	0
0	0	0	0	0	0	0	0	0	0	0	0	0	0
1	0	0	0	0	0	0	0	0	0	0	0	0	0
0	0	0	0	0	0	0	0	0	0	0	0	0	0
0													
OTU14571	0	0	0	1	0	0	0	0	0	0	0	0	0
0	0	0	0	0	0	0	0	0	0	0	0	0	0
0	0	0	0	0	0	0	0	0	0	0	0	0	0
0	0	0	0	0	0	0	0	0	0	0	0	0	0
0	0	0	0	0	0	0	0	0	0	0	0	0	0
0	0	0	0	0	0	0	0	0	0	0	0	0	0
0	0	0	0	0	0	0	0	0	0	0	0	0	0
0	0	0	0	0	0	0	0	0	0	0	0	0	0
0													
OTU8365	0	0	0	1	0	0	0	0	0	0	0	0	0
0	0	0	0	0	0	0	0	0	0	0	0	0	0
0	0	0	0	0	0	0	0	0	0	0	0	0	0
0	0	0	0	0	0	0	0	0	0	0	0	0	0
0	0	0	0	0	0	0	0	0	0	0	0	0	0
0	0	0	0	0	0	0	0	0	0	0	0	0	0
0	0	0	0	0	0	0	0	0	0	0	0	0	0
0	0	0	0	0	0	0	0	0	0	0	0	0	0
0													

OTU3615	0	0	0	1	0	0	1	0	1	10	0	6	0
0	0	0	0	0	0	0	0	0	0	0	0	0	0
2	0	0	0	0	2	0	6	0	0	0	0	0	0
0	0	0	0	0	0	0	0	0	0	0	0	0	4
0	0	0	0	0	0	0	0	0	1	0	0	1	0
0	1	0	0	0	0	0	0	0	2	0	0	0	1
0	0	2	0	0	0	0	0	0	0	2	0	0	0
1	0	0	1	0	0	0	0	0	0	2	0	0	0
0													
OTU2583	0	0	0	2	0	0	0	0	0	0	0	0	0
0	2	0	0	0	0	0	0	1	0	0	0	0	0
13	0	0	0	0	0	0	0	0	0	0	0	0	0
7	1	0	0	0	0	0	3	0	0	4	1	0	0
0	0	0	0	0	0	0	0	0	0	0	0	0	0
0	0	0	21	0	1	2	0	2	4	0	1	0	0
6	0	65	0	1	1	4	0	0	0	1	0	0	0
0	0	0	0	0	0	0	0	0	0	0	0	0	0
0													
OTU12256	0	0	0	1	0	0	0	0	0	0	0	0	0
0	0	0	0	0	0	0	0	0	0	0	0	0	0
0	0	0	0	0	0	0	0	0	0	0	0	0	0
0	0	0	0	0	0	0	0	0	0	0	0	0	0
0	0	0	0	0	0	0	0	0	0	0	0	0	0
0	0	0	0	0	0	0	0	0	0	0	0	0	0
0	0	0	0	0	0	0	0	0	0	0	0	0	0
0	0	0	0	0	0	0	0	0	0	0	0	0	0
0													
OTU7077	0	0	0	1	0	0	0	2	0	0	0	0	0
0	0	0	0	0	0	0	0	0	0	0	0	0	0
0	0	0	0	0	2	0	0	0	0	0	0	0	0
0	1	0	6	0	0	0	5	0	0	0	0	0	0
0	0	1	0	0	0	2	0	4	0	0	1	0	0
0	356	0	0	0	3	1	0	0	0	3	0	1	0

0	1	0	0	2	0	0	0	0	2	0	432	86	0
0	431	2	113	42	1	28	23	30	13	1	6	1	4
115													
OTU8331	0	0	0	3	1	0	0	0	0	0	0	0	0
0	0	0	0	5	0	0	0	0	0	0	0	0	0
0	0	0	0	0	0	0	0	0	0	0	0	0	0
0	0	0	0	0	0	0	0	0	0	0	0	0	0
0	0	0	0	0	0	0	0	0	0	0	0	0	0
0	0	0	0	0	0	0	0	0	0	0	0	0	0
0	0	0	0	0	0	0	0	0	0	0	0	0	0
0	0	0	0	0	0	0	0	0	0	0	0	0	0
0													
OTU10914	0	0	0	1	0	0	0	0	0	0	0	0	0
0	0	0	0	0	0	0	0	0	0	0	0	0	0
0	0	0	0	0	0	0	0	0	0	0	0	0	0
0	0	0	0	0	0	0	0	0	0	0	0	0	0
0	0	0	0	0	0	0	0	0	0	0	0	0	0
0	0	0	0	0	0	0	0	0	0	0	0	0	0
0	0	0	0	0	0	0	0	0	0	0	0	0	0
0	0	0	0	0	0	0	0	0	0	0	0	0	0
0													
OTU15708	0	0	0	1	0	0	2	0	0	0	0	3	0
0	0	0	0	2	0	1	0	0	0	0	0	1	0
0	0	0	0	0	0	1	0	0	0	0	0	0	0
0	0	0	0	0	0	0	1	0	0	0	0	0	0
0	2	1	0	0	0	0	0	0	0	0	0	0	0
0	0	0	1	1	0	0	0	1	0	0	0	0	0
0	0	0	0	0	0	0	0	0	0	0	1	0	0
0	0	0	0	1	0	0	0	0	0	3	0	0	1
0													
OTU350	0	0	0	1	0	0	0	0	0	0	2	0	0
0	0	0	0	0	0	4	7	1	0	0	0	0	4
11	0	0	0	0	0	0	0	1	0	0	0	6	0

5	0	0	0	0	0	0	0	0	3	0	14	0	3
0	0	0	1	1	0	0	2	0	0	0	0	6	0
1	15	0	0	0	0	0	2	0	4	0	6	0	2
15	5	0	2	1	5	2	0	0	0	0	2	12	0
0	3	5	0	24	2	6	14	2	0	0	3	0	0
9													
OTU10915	0	0	0	1	0	0	0	0	0	0	0	0	0
0	0	0	0	0	0	0	0	0	0	0	0	0	0
0	0	0	0	0	0	0	0	0	0	0	0	0	0
0	0	0	0	0	0	0	0	0	0	0	0	0	0
0	0	0	0	0	0	0	0	0	0	0	0	0	0
0	0	0	0	0	0	0	0	0	0	0	0	0	0
0	0	0	0	0	0	0	0	0	0	0	0	0	0
0	0	0	0	0	0	0	0	0	0	0	0	0	0
0													
OTU12253	0	0	0	1	0	0	0	0	0	0	0	0	0
0	0	0	0	0	0	0	0	0	0	0	0	0	0
0	0	0	0	0	0	0	0	0	0	0	0	0	0
0	0	0	0	0	0	0	0	0	0	0	0	0	0
0	0	0	0	0	0	0	0	0	0	0	0	0	0
0	0	0	0	0	0	0	0	0	0	0	0	0	0
0	0	0	0	0	0	0	0	0	0	0	0	0	0
0	0	0	0	0	0	0	0	0	0	0	0	0	0
0													
OTU14130	0	0	0	1	0	0	0	0	0	0	3	0	3
1	0	0	0	0	0	0	0	0	0	0	0	0	0
0	0	0	0	0	0	1	0	0	0	0	0	0	0
0	0	0	0	0	3	2	0	0	0	0	0	0	3
0	0	0	0	0	0	0	0	0	0	0	0	1	0
0	0	0	2	0	0	0	0	0	0	0	0	1	0
0	0	0	0	0	0	0	0	1	0	1	0	0	0
0	0	0	0	0	0	0	0	1	0	0	0	0	0
0													

OTU12114	0	0	0	1	0	1	1	0	0	0	0	0	0
0	0	0	0	0	0	0	0	0	0	0	0	0	0
0	0	0	0	0	0	0	0	4	0	0	0	0	0
0	0	0	0	0	1	0	1	0	0	0	0	0	0
0	0	0	2	0	0	0	0	0	0	0	0	0	0
0	0	0	1	0	0	0	0	0	0	0	0	0	0
0	0	0	0	0	0	2	0	0	0	0	0	0	0
0	0	0	0	0	0	0	0	0	0	0	0	0	0
0													
OTU12348	0	0	0	1	0	0	0	0	1	0	0	1	0
0	0	0	0	0	0	0	0	0	0	0	0	2	0
0	0	0	0	0	0	0	1	0	0	0	0	0	0
14	1	0	0	0	1	0	0	0	0	0	0	0	0
0	0	0	0	1	0	0	0	0	0	0	0	2	0
0	0	0	0	0	0	0	0	0	2	0	0	0	0
0	0	0	0	0	0	0	1	0	0	1	0	0	0
1	0	0	0	0	0	0	0	0	0	0	0	0	0
0													
OTU12251	0	0	0	1	0	0	0	0	0	0	0	0	0
0	0	0	0	0	0	0	0	0	0	0	0	0	0
0	0	0	0	0	0	0	0	0	0	0	0	0	0
0	0	0	0	0	0	0	0	0	0	0	0	0	0
0	0	0	0	0	0	0	0	0	0	0	0	0	0
0	0	0	0	0	0	0	0	0	0	0	0	0	0
0	0	0	0	0	0	0	0	0	0	0	0	0	0
0	0	0	0	0	0	0	0	0	0	0	0	0	0
0													
OTU10859	0	0	0	2	1	0	1	1	1	0	0	0	0
1	0	1	0	1	0	0	0	3	0	0	0	1	0
1	1	1	0	0	0	1	0	1	0	1	0	1	0
1	0	2	0	0	0	0	0	0	0	0	0	1	0
0	0	0	3	1	0	0	2	1	2	0	1	1	0
1	1	0	0	1	1	0	0	0	0	0	4	0	0

1	2	1	0	0	0	0	0	0	1	0	0	0	0
0	1	0	0	1	0	0	0	0	0	2	0	0	0
1													
OTU1182	0	0	0	2	0	0	0	2	0	1	0	0	0
0	0	0	0	0	0	0	1	0	0	0	0	0	0
1	0	0	0	0	0	0	1	0	0	0	0	0	0
0	2	0	0	0	0	0	0	0	0	0	0	0	0
0	0	0	0	0	0	1	0	0	0	0	0	0	0
0	0	0	1	0	0	0	0	0	0	0	0	0	0
0	0	0	0	0	2	1	0	0	0	0	0	0	0
0	1	2	0	0	0	0	0	0	1	0	0	0	2
0													
OTU1438	0	0	0	1	0	0	0	0	0	0	0	0	0
0	0	0	0	0	0	0	0	0	0	0	0	0	0
0	0	0	0	0	0	0	0	0	0	0	0	0	0
0	0	0	0	0	0	0	0	0	0	0	0	0	0
0	0	0	0	0	0	0	0	0	0	0	0	0	0
0	0	0	1	0	0	0	0	0	0	0	0	0	0
0	0	0	0	1	0	0	0	0	0	0	0	0	0
0	0	0	0	0	0	0	0	0	0	0	0	0	0
0													
OTU12252	0	0	0	1	0	0	0	0	0	0	0	0	0
0	0	0	0	0	0	0	0	0	0	0	0	0	0
0	0	0	0	0	0	0	0	0	0	0	0	0	0
0	0	0	0	0	0	0	0	0	0	0	0	0	0
0	0	0	0	0	0	0	0	0	0	0	0	0	0
0	0	0	0	0	0	0	0	0	0	0	0	0	0
0	0	0	0	0	0	0	0	0	0	0	0	0	0
0	0	0	0	0	0	0	0	0	0	0	0	0	0
0													
OTU604	0	0	0	4	0	0	2	0	0	0	0	0	0
0	1	25	8	5	2	2	0	0	1	6	35	2	0
0	0	1	4	4	3	10	0	0	24	0	0	0	0

0	0	0	7	0	0	5	0	0	0	0	21	0	5
0	0	0	0	0	0	0	0	0	0	0	1	0	0
0	0	0	0	0	4	0	0	0	0	0	0	1	0
0	0	0	0	0	0	1	0	0	0	0	0	0	0
1	0	0	0	0	0	0	0	0	0	2	0	0	0
0													
OTU14572	0	0	0	1	0	0	0	0	0	0	0	0	0
0	0	0	0	0	0	0	0	0	0	0	0	0	0
0	0	0	0	0	0	0	0	0	0	0	0	0	0
0	0	0	0	0	0	0	0	0	0	0	0	0	0
0	0	0	0	0	0	0	0	0	0	0	0	0	0
0	0	0	0	0	0	0	0	0	0	0	0	0	0
0	0	0	0	0	0	0	0	0	0	0	0	0	0
0	0	0	0	0	0	0	0	0	0	0	0	0	0
0													
OTU10911	0	0	0	1	0	0	0	0	0	0	0	0	0
0	0	0	0	0	0	0	0	0	0	0	0	0	0
0	0	0	0	0	0	0	0	0	0	0	0	0	0
0	0	0	0	0	0	0	0	0	0	0	0	0	0
0	0	0	0	0	0	0	0	0	0	0	0	0	0
0	0	0	0	0	0	0	0	0	0	0	0	0	0
0	0	0	0	0	0	0	0	0	0	0	0	0	0
0	0	0	0	0	0	0	0	0	0	0	0	0	0
0													
OTU13850	0	0	0	2	2	0	1	1	0	0	1	0	0
0	0	1	0	1	1	0	1	0	10	3	0	0	0
0	0	1	1	0	0	0	0	0	0	0	2	1	0
0	0	1	0	0	0	0	1	0	0	5	0	4	1
0	1	0	0	1	0	1	0	0	1	0	0	0	6
0	0	1	0	1	0	0	0	0	2	0	0	0	1
0	0	0	0	0	0	0	0	0	0	0	0	0	1
0	0	0	0	0	0	0	1	0	0	0	0	0	0
0													

OTU2278	0	0	0	1	0	0	0	0	0	0	0	0	0
0	0	0	0	0	0	0	0	0	0	0	0	0	0
0	0	0	0	0	0	0	0	0	0	0	0	0	0
0	0	0	0	0	1	0	0	0	0	0	0	0	0
0	2	0	0	0	0	0	0	0	0	0	0	2	0
0	0	0	0	0	0	0	0	0	0	0	0	0	0
0	0	0	0	0	0	0	0	0	0	0	1	0	0
0	1	0	0	0	0	0	0	0	0	0	0	0	0
0													
OTU11404	0	0	0	1	0	0	0	0	0	0	0	0	0
0	0	0	0	0	0	0	0	0	0	0	0	0	0
0	0	0	0	0	0	0	0	0	0	0	0	0	0
0	0	0	0	0	0	0	0	0	0	0	0	0	0
0	0	0	0	0	0	0	0	0	0	0	0	0	0
0	0	0	0	0	0	0	0	0	0	0	0	0	0
0	0	0	0	0	0	0	0	0	0	0	0	0	1
1	1	0	0	1	0	0	0	0	0	0	0	0	0
0													
OTU12254	0	0	0	1	0	0	0	0	0	0	0	0	0
0	0	0	0	0	0	0	0	0	0	0	0	0	0
0	0	0	0	0	0	0	0	0	0	0	0	0	0
0	0	0	0	0	0	0	0	0	0	0	0	0	0
0	0	0	0	0	0	0	0	0	0	0	0	0	0
0	0	0	0	0	0	0	0	0	0	0	0	0	0
0	0	0	0	0	0	0	0	0	0	0	0	0	0
0	0	0	0	0	0	0	0	0	0	0	0	0	0
0													
OTU14722	0	0	0	1	0	0	0	0	0	0	2	3	0
1	0	0	0	2	0	3	1	0	0	0	0	0	0
0	0	0	0	0	2	0	6	5	0	0	1	0	0
0	0	0	0	0	0	3	1	0	0	1	0	0	5
0	0	0	1	0	0	0	0	0	0	0	0	0	0
0	0	0	0	0	0	2	0	3	0	1	0	1	0

5	0	2	0	8	0	0	0	1	1	3	0	0	1
8	0	0	0	0	0	0	0	0	0	4	3	0	0
0													
OTU15096	0	0	0	1	0	0	0	0	0	0	0	0	0
0	0	0	0	0	0	0	0	2	0	0	0	0	0
0	0	0	0	0	0	0	0	0	0	0	0	0	0
1	0	0	0	0	1	0	0	5	0	0	0	0	0
1	0	0	0	0	0	2	0	1	0	0	0	2	0
1	0	0	0	0	0	0	0	0	0	1	0	0	0
0	0	0	0	0	0	0	0	0	0	0	0	0	0
0	0	2	0	2	5	2	0	7	0	0	0	0	3
0													
OTU10909	0	0	0	1	0	0	0	0	0	0	0	0	0
0	0	0	0	0	0	0	0	0	0	0	0	0	0
0	0	0	0	0	0	0	0	0	0	0	0	0	0
0	0	0	0	0	0	0	0	0	0	0	0	0	0
0	0	0	0	0	0	0	0	0	0	0	0	0	0
0	0	0	0	0	0	0	0	0	0	0	0	0	0
0	0	0	0	0	0	0	0	0	0	0	0	0	0
0	0	0	0	0	0	0	0	0	0	0	0	0	0
0													
OTU10910	0	0	0	1	0	0	0	0	0	0	0	0	0
0	0	0	0	0	0	0	0	0	0	0	0	0	0
0	0	0	0	0	0	0	0	0	0	0	0	0	0
0	0	0	0	0	0	0	0	0	0	0	0	0	0
0	0	0	0	0	0	0	0	0	0	0	0	0	0
0	0	0	0	0	0	0	0	0	0	0	0	0	0
0	0	0	0	0	0	0	0	0	0	0	0	0	0
0	0	0	0	0	0	0	0	0	0	0	0	0	0
0													
OTU8368	0	0	0	1	0	0	0	0	0	0	0	0	0
0	0	0	0	0	0	0	0	0	0	0	0	0	0
0	0	0	0	0	0	0	0	0	0	0	0	0	0

0	0	0	0	0	0	0	0	0	0	0	0	0	0
0	0	0	0	0	0	0	0	0	0	0	0	0	0
0	0	0	0	0	0	0	0	0	0	0	0	0	0
0	0	0	0	0	0	0	0	0	0	0	0	0	0
0	0	0	0	0	0	0	0	0	0	0	0	0	0
0													
OTU12274	0	0	0	1	0	0	0	0	0	0	0	0	0
0	0	0	0	0	0	0	0	0	0	0	0	0	0
0	0	0	0	0	0	0	0	0	0	0	0	0	0
0	0	0	0	0	0	0	0	0	0	0	0	0	0
0	0	0	0	0	0	0	0	0	0	0	0	0	0
0	0	0	0	0	0	0	0	0	0	0	0	0	0
0	0	0	0	0	0	0	0	0	0	0	0	0	0
0	0	0	0	0	0	0	0	0	0	0	0	0	0
0													
OTU12073	0	0	0	1	1	0	0	0	0	0	0	0	0
0	0	0	0	0	0	0	0	0	0	0	0	0	1
0	0	1	0	0	0	0	0	0	0	0	0	0	0
0	0	0	0	0	0	0	0	0	0	0	0	1	0
0	0	0	0	0	0	1	0	0	0	0	0	0	0
0	0	0	0	0	0	0	0	0	1	0	0	0	0
0	0	0	0	0	0	0	0	0	0	0	0	0	0
0	0	0	0	0	0	0	0	0	0	0	0	0	0
0													
OTU15980	0	0	0	1	0	0	0	0	0	0	0	0	0
0	0	1	1	0	0	0	0	0	3	0	0	0	0
0	0	1	0	0	0	1	1	0	1	0	1	0	0
0	0	0	0	0	0	0	1	1	0	0	0	1	0
0	0	2	0	0	0	0	0	0	0	1	0	0	2
0	0	2	1	0	0	0	0	0	0	0	0	0	0
0	0	0	0	0	0	0	0	0	0	0	1	0	1
0	0	0	0	0	0	0	0	0	0	0	0	0	0
0													

OTU12273	0	0	0	1	0	0	0	0	0	0	0	0	0
0	0	0	0	0	0	0	0	0	0	0	0	0	0
0	0	0	0	0	0	0	0	0	0	0	0	0	0
0	0	0	0	0	0	0	0	0	0	0	0	0	0
0	0	0	0	0	0	0	0	0	0	0	0	0	0
0	0	0	0	0	0	0	0	0	0	0	0	0	0
0	0	0	0	0	0	0	0	0	0	0	0	0	0
0	0	0	0	0	0	0	0	0	0	0	0	0	0
0													
OTU5424	0	0	0	1	0	0	0	0	0	0	0	0	0
0	0	0	0	0	0	0	0	0	0	0	0	0	0
0	0	0	0	0	0	0	0	0	1	0	0	0	0
0	0	0	0	0	1	0	0	0	0	0	0	0	0
0	0	0	0	0	0	0	0	0	0	0	0	0	0
0	0	1	0	0	0	0	0	0	0	0	0	0	0
0	0	0	0	0	0	0	0	0	0	0	0	0	0
0	0	0	0	1	0	0	0	0	0	0	0	0	0
0													
OTU7748	0	0	0	1	1	0	0	0	0	0	1	0	0
0	0	0	0	0	0	0	0	0	0	0	0	0	0
0	0	0	0	0	0	0	0	0	0	0	0	1	0
0	1	0	0	0	0	0	0	0	2	0	0	0	0
0	0	0	0	0	0	0	0	0	0	0	0	0	0
0	0	0	0	0	0	0	0	0	1	0	0	0	0
0	0	0	0	0	2	0	0	0	0	0	0	0	0
0	0	0	0	0	0	1	0	0	0	0	0	0	0
0													
OTU4115	0	0	0	1	0	1	1	0	3	0	40	2	23
0	1	3	0	6	0	0	1	0	0	0	0	0	0
0	0	0	7	0	0	0	1	0	0	0	0	0	0
0	1	0	0	5	2	5	0	0	1	1	0	0	1
0	4	0	2	0	0	0	3	0	1	0	0	5	0
3	0	0	0	0	0	1	0	0	0	0	0	4	0

1	0	0	0	0	0	1	1	10	1	5	0	0	8
1	0	2	0	0	3	0	0	2	0	2	3	2	0
0													
OTU8868	0	0	0	3	0	0	3	0	1	0	11	0	2
4	0	2	1	4	31	0	0	0	0	0	1	0	1
0	0	19	5	1	0	0	1	0	0	0	0	0	12
0	0	9	0	4	0	0	0	9	0	1	0	0	0
1	0	1	0	0	2	0	1	0	0	2	0	0	2
0	0	0	0	0	0	1	0	0	0	0	0	0	0
0	0	0	0	0	0	0	0	0	0	0	0	0	3
0	0	0	0	0	0	0	0	0	0	0	1	0	0
0													
OTU8234	0	0	0	2	0	0	0	0	0	0	0	1	1
1	0	1	16	0	0	0	0	1	0	12	0	0	3
0	0	0	2	4	1	0	0	2	5	3	0	0	2
0	0	3	1	0	0	0	0	4	2	0	0	3	0
2	0	2	0	1	2	2	0	0	1	5	3	1	4
2	0	1	0	0	0	5	0	2	0	4	0	0	0
0	0	4	0	3	0	0	0	0	0	0	0	0	0
2	0	0	0	0	0	0	0	0	0	1	1	2	0
0													
OTU2367	0	0	0	1	0	0	0	0	3	1	0	0	0
0	0	0	0	0	0	1	0	1	0	0	0	0	0
1	0	1	1	0	0	1	0	0	0	0	0	0	0
0	0	9	0	2	1	0	0	11	8	1	0	2	0
1	1	1	0	7	10	4	14	6	2	1	0	5	5
3	1	0	0	0	0	0	0	0	1	0	4	0	5
0	8	0	5	0	0	0	6	0	0	0	1	1	0
0	1	0	0	2	3	0	0	0	8	0	0	0	0
2													
OTU14570	0	0	0	1	0	0	0	0	0	0	0	0	0
0	0	0	0	0	0	0	0	0	0	0	0	0	0
0	0	0	0	0	0	0	0	0	0	0	0	0	0

0	0	0	0	0	0	0	0	0	0	0	0	0	0
0	0	0	0	0	0	0	0	0	0	0	0	0	0
0	0	0	0	0	0	0	0	0	0	0	0	0	0
0	0	0	0	0	0	0	0	0	0	0	0	0	0
0	0	0	0	0	0	0	0	0	0	0	0	0	0
0													
OTU3342	0	0	0	2	1	0	0	1	0	0	0	2	0
0	0	0	0	0	0	0	0	0	0	0	0	0	0
0	0	0	0	0	0	0	0	0	0	0	0	1	0
0	0	0	0	0	0	0	0	0	0	0	1	0	1
0	1	0	0	0	0	0	0	0	0	0	0	0	0
0	1	0	0	0	0	0	0	0	2	0	0	0	2
0	0	0	2	0	2	0	1	0	0	0	0	0	0
0	0	0	0	0	0	0	0	0	0	1	0	0	0
0													
OTU4418	0	0	0	1	0	0	0	0	0	0	1	2	3
2	0	2	2	1	4	0	6	1	1	3	1	0	1
0	0	4	6	2	13	2	1	3	2	0	3	0	7
0	0	0	2	1	0	2	0	0	2	0	0	0	0
2	0	1	0	0	0	0	0	0	0	0	0	0	0
1	0	0	5	0	5	1	0	4	0	5	0	0	0
0	0	5	0	0	0	0	0	0	0	1	0	0	0
1	0	0	0	0	0	0	0	0	0	2	4	12	0
0													
OTU14577	0	0	0	1	0	0	0	0	0	0	0	0	0
0	0	0	0	0	0	0	0	0	0	0	0	0	0
0	0	0	0	0	0	0	0	0	0	0	0	0	0
0	0	0	0	0	0	0	0	0	0	0	0	0	0
0	0	0	0	0	0	0	0	0	0	0	0	0	0
0	0	0	0	0	0	0	0	0	0	0	0	0	0
0	0	0	0	0	0	0	0	0	0	0	0	0	0
0	0	0	0	0	0	0	0	0	0	0	0	0	0
0													

OTU12275 0 0 0 1 0 0 0 0 0 0 0 0 0
0 0 0 0 0 0 0 0 0 0 0 0 0 0
0 0 0 0 0 0 0 0 0 0 0 0 0 0
0 0 0 0 0 0 0 0 0 0 0 0 0 0
0 0 0 0 0 0 0 0 0 0 0 0 0 0
0 0 0 0 0 0 0 0 0 0 0 0 0 0
0 0 0 0 0 0 0 0 0 0 0 0 0 0
0 0 0 0 0 0 0 0 0 0 0 0 0 0
0
OTU8367 0 0 0 1 0 1 0 0 0 1 0 0 0
0 0 0 0 1 0 0 0 0 0 0 0 0 0
0 0 0 0 0 0 0 0 1 0 0 0 0 0
0 0 0 0 0 0 0 0 0 0 2 0 0 0
0 3 0 0 0 0 0 0 0 0 0 0 0 0
0 0 0 0 0 0 0 0 0 0 0 0 0 0
0 0 0 0 0 1 0 0 0 0 0 0 0 0
0 0 0 0 0 0 0 0 0 0 0 0 0 0
0
OTU14647 0 0 0 2 0 2 0 1 1 2 0 0 0
0 1 0 0 0 0 0 0 0 0 0 0 0 0
0 0 0 0 0 0 0 0 0 0 0 0 0 0
0 0 0 0 0 0 0 0 0 0 0 0 0 0
0 0 0 0 0 0 0 0 0 0 0 0 0 0
0 0 0 0 0 0 0 1 0 0 0 0 0 0
0 0 0 0 0 0 1 2 0 0 0 0 0 0
0 0 0 0 0 0 0 0 0 0 0 0 0 0
0
OTU371 0 0 0 1 0 0 1 0 0 0 0 0 0
0 0 0 0 0 0 3 0 0 0 0 0 0 0
2 0 0 0 0 0 0 0 0 0 66 0 0 0
0 0 0 0 0 0 0 0 0 0 0 0 0 0
0 0 0 0 0 33 0 0 0 0 0 0 0 0
0 0 0 1 0 0 0 0 0 0 0 0 0 0

0	0	0	0	0	0	0	0	0	0	0	0	0	0
0	0	0	0	0	0	0	0	0	0	0	0	0	0
0													
OTU15540	0	0	0	1	3	0	0	0	0	0	1	0	0
0	0	0	0	0	1	0	0	0	0	0	0	0	0
0	0	0	2	1	1	0	1	0	0	0	0	0	0
0	2	0	0	0	0	0	0	0	0	0	0	0	0
0	0	0	1	0	0	0	1	0	0	2	0	0	0
0	0	0	0	1	0	0	1	0	0	0	0	0	0
0	0	0	0	0	0	0	0	0	0	0	0	0	0
0	0	0	0	0	0	0	1	1	0	0	0	0	1
2													
OTU14576	0	0	0	1	0	0	0	0	0	0	0	0	0
0	0	0	0	0	0	0	0	0	0	0	0	0	0
0	0	0	0	0	0	0	0	0	0	0	0	0	0
0	0	0	0	0	0	0	0	0	0	0	0	0	0
0	0	0	0	0	0	0	0	0	0	0	0	0	0
0	0	0	0	0	0	0	0	0	0	0	0	0	0
0	0	0	0	0	0	0	0	0	0	0	0	0	0
0	0	0	0	0	0	0	0	0	0	0	0	0	0
0													
OTU3493	0	0	0	1	0	0	0	0	0	0	0	0	0
0	0	0	1	0	0	0	0	0	0	0	0	0	0
0	0	0	0	0	0	0	0	0	0	0	0	0	0
0	0	0	0	0	0	0	0	0	0	0	0	0	0
0	0	0	0	0	0	1	0	0	0	0	0	0	0
0	0	1	0	0	0	0	0	0	0	0	0	0	0
0	0	0	0	0	0	0	0	0	0	0	0	0	0
0	0	0	0	0	0	0	0	0	0	1	0	0	0
0													
OTU506	0	0	0	1	0	1	0	0	0	0	0	0	0
0	0	0	0	0	0	0	0	0	0	0	0	0	0
0	0	0	0	0	0	0	0	0	0	0	0	0	0

0	0	0	0	0	0	0	0	0	0	0	0	0	0
0	1	0	0	0	0	0	0	0	0	0	0	0	0
0	1	0	0	1	0	0	0	0	0	0	0	0	0
0	0	0	0	0	0	0	1	0	0	0	6	4	0
0	10	1	4	10	0	0	11	0	2	0	0	0	1
4													
OTU10900	0	0	0	1	0	0	0	0	0	0	0	0	0
0	0	0	0	0	0	0	0	0	0	0	0	0	0
0	0	0	0	0	0	0	0	0	0	0	0	0	0
0	0	0	0	0	0	0	0	0	0	0	0	0	0
0	0	0	0	0	0	0	0	0	0	0	0	0	0
0	0	0	0	0	0	0	0	0	0	0	0	0	0
0	0	0	0	0	0	0	0	0	0	0	0	0	0
0	0	0	0	0	0	0	0	0	0	0	0	0	0
0													
OTU14579	0	0	0	1	0	0	0	0	0	0	0	0	0
0	0	0	0	0	0	0	0	0	0	0	0	0	0
0	0	0	0	0	0	0	0	0	0	0	0	0	0
0	0	0	0	0	0	0	0	0	0	0	0	0	0
0	0	0	0	0	0	0	0	0	0	0	0	0	0
0	0	0	0	0	0	0	0	0	0	0	0	0	0
0	0	0	0	0	0	0	0	0	0	0	0	0	0
0	0	0	0	0	0	0	0	0	0	0	0	0	0
0													
OTU10649	0	0	0	1	0	1	0	0	0	0	1	0	1
8	0	8	8	16	2	0	6	5	1	6	0	1	0
0	9	2	4	4	2	1	2	0	0	1	5	0	4
0	0	0	1	0	0	1	0	0	0	1	0	1	2
3	0	2	0	2	0	1	1	4	1	1	0	0	1
2	0	0	0	0	2	8	2	5	0	5	0	2	0
0	0	4	0	0	0	0	0	1	0	1	0	0	2
4	0	0	0	0	0	0	0	0	0	3	1	10	0
0													

OTU9241		0	0	0	1	0	0	0	0	0	0	0	0	0
	1	0	0	0	0	3	0	0	0	0	0	0	0	1
	0	0	2	1	1	0	0	0	0	0	0	0	0	1
	0	0	0	0	1	0	0	0	2	0	0	0	3	0
	1	0	0	0	0	0	1	0	0	1	0	1	0	0
	0	0	0	0	0	0	0	0	0	0	0	0	0	0
	0	0	0	0	0	0	1	0	4	0	0	0	0	2
	1	0	0	0	0	0	0	0	0	0	0	3	0	0
	0													
OTU16092		0	0	0	1	0	0	0	0	0	0	0	2	0
	0	2	5	1	5	0	4	3	0	0	0	3	3	0
	2	0	0	0	0	6	1	2	3	0	1	4	10	0
	0	1	0	0	3	5	0	18	0	0	6	1	1	40
	0	37	5	4	0	0	2	0	0	2	0	2	2	3
	0	0	0	3	1	0	1	0	10	0	0	0	5	1
	7	0	0	1	5	1	1	0	0	1	2	0	0	0
	2	0	0	0	0	0	0	0	0	0	0	1	0	1
	0													
OTU13638		0	0	0	2	0	0	0	0	0	0	0	0	0
	1	0	1	0	0	0	0	1	0	0	0	1	0	0
	0	0	0	0	0	0	0	0	0	0	0	0	0	2
	0	0	0	1	0	0	0	0	0	0	1	0	0	0
	0	1	1	0	0	0	0	1	0	1	1	1	0	1
	0	2	0	0	1	0	0	0	0	0	0	3	0	1
	0	0	0	0	0	0	0	1	0	0	0	1	0	0
	0	1	0	0	1	0	0	0	0	0	0	0	0	1
	1													
OTU2992		0	0	0	2	0	0	2	1	0	0	24	7	10
	17	4	1	0	12	1	7	2	0	0	0	1	2	1
	0	0	1	0	0	27	0	35	32	4	0	9	0	0
	0	9	0	5	0	2	40	2	0	0	8	0	0	29
	0	2	4	2	0	0	0	2	0	0	0	2	0	0
	0	0	0	11	0	1	10	0	14	0	1	1	8	1

42	0	10	1	63	2	4	0	9	3	31	2	0	12
36	2	0	0	0	1	0	0	2	0	24	24	1	0
0													
OTU14578	0	0	0	1	0	0	0	0	0	0	0	0	0
0	0	0	0	0	0	0	0	0	0	0	0	0	0
0	0	0	0	0	0	0	0	0	0	0	0	0	0
0	0	0	0	0	0	0	0	0	0	0	0	0	0
0	0	0	0	0	0	0	0	0	0	0	0	0	0
0	0	0	0	0	0	0	0	0	0	0	0	0	0
0	0	0	0	0	0	0	0	0	0	0	0	0	0
0	0	0	0	0	0	0	0	0	0	0	0	0	0
0													
OTU8358	0	0	0	1	0	0	0	0	0	0	0	0	0
0	0	0	0	0	0	0	0	0	0	0	0	0	0
0	0	0	0	0	0	0	0	0	0	0	0	0	0
0	0	0	0	0	0	0	0	0	0	0	0	0	0
0	0	0	0	0	0	0	0	0	0	0	0	0	0
0	0	0	0	0	0	0	0	0	0	0	0	0	0
0	0	0	0	0	0	0	0	0	0	0	0	0	0
0	0	0	0	0	0	0	0	0	0	0	0	0	0
0													
OTU14441	0	0	0	1	6	0	2	0	0	0	1	0	0
0	1	0	0	5	0	1	0	0	0	0	0	0	0
0	0	0	0	0	2	0	0	3	0	0	0	1	0
0	1	0	0	0	2	0	4	0	0	0	0	0	3
0	3	1	0	0	0	2	0	0	2	0	1	0	1
0	0	0	1	1	1	0	0	0	0	0	0	0	0
0	0	2	0	1	0	0	0	0	0	0	1	0	0
0	0	0	0	0	0	0	0	0	0	0	1	0	0
0													
OTU504	0	0	0	1	0	0	0	0	0	0	0	0	0
0	0	0	0	0	0	0	0	0	0	0	0	0	0
0	0	0	0	0	0	1	0	0	0	0	0	0	0

0 5 0 0 0 0 0 0 0 0 0 1 0 0
0 0 0 0 0 0 0 0 0 0 0 0 0 0
0 2 0 0 0 0 0 0 0 0 0 1 0 0
0 0 0 0 0 0 0 0 0 0 0 1 1 0
0 8 5 4 7 2 5 6 2 13 0 0 0 18
8
OTU8357 0 0 0 1 0 0 0 0 0 0 0 0 0
0 0 0 0 0 0 0 0 0 0 0 0 0 0
0 0 0 0 0 0 0 0 0 0 0 0 0 0
0 0 0 0 0 0 0 0 0 0 0 0 0 0
0 0 0 0 0 0 0 0 0 0 0 0 0 0
0 0 0 0 0 0 0 0 0 0 0 0 0 0
0 0 0 0 0 0 0 0 0 0 0 0 0 0
0 0 0 0 0 0 0 0 0 0 0 0 0 0
0
OTU10895 0 0 0 1 0 1 0 0 0 0 0 0 0
0 0 0 0 0 0 0 0 0 0 0 0 0 0
0 0 0 0 0 0 0 0 0 0 0 0 0 0
0 0 0 0 0 0 0 0 0 0 0 0 0 0
0 0 0 0 0 0 0 0 0 0 0 0 0 0
0 0 1 0 0 0 0 0 0 0 0 0 0 0
0 0 0 0 0 0 0 0 0 0 0 0 2 0
0 0 0 0 0 0 0 0 0 0 0 0 0 0
0
OTU7936 0 0 0 1 0 0 0 0 0 1 0 1 1
1 1 2 0 0 0 0 0 0 0 0 0 0 0
0 0 0 0 0 0 0 1 0 1 0 0 0 0
0 2 0 0 0 0 0 0 0 0 0 0 0 0
0 0 0 0 0 0 0 0 0 0 0 0 0 0
0 0 0 0 0 0 0 0 0 0 0 0 0 0
0 0 0 0 0 0 0 0 0 2 2 0 0 0
0 0 1 0 0 0 0 0 0 0 0 2 0 0
0

OTU6430	0	0	0	1	1	0	0	0	0	0	0	0	0
0	0	0	0	0	0	0	0	0	0	0	0	0	0
0	0	0	0	0	0	0	0	0	0	0	0	0	0
1	0	0	0	0	0	0	0	0	0	0	0	0	1
0	3	0	0	0	0	0	0	0	0	0	0	2	0
0	0	0	0	0	1	0	0	0	1	0	0	0	0
0	0	0	0	0	0	0	0	0	0	0	0	0	0
0	0	0	0	0	0	0	0	0	1	0	0	1	0
0													
OTU8356	0	0	0	1	0	0	0	0	0	0	0	0	0
0	0	0	0	0	0	0	0	0	0	0	0	0	0
0	0	0	0	0	0	0	0	0	0	0	0	0	0
0	0	0	0	0	0	0	0	0	0	0	0	0	0
0	0	0	0	0	0	0	0	0	0	0	0	0	0
0	0	0	0	0	0	0	0	0	0	0	0	0	0
0	0	0	0	0	0	0	0	0	0	0	0	0	0
0	0	0	0	0	0	0	0	0	0	0	0	0	0
0													
OTU10896	0	0	0	1	0	0	0	0	0	0	0	0	0
0	0	0	0	0	0	0	0	0	0	0	0	0	0
0	0	0	0	0	0	0	0	0	0	0	0	0	0
0	0	0	0	0	0	0	0	0	0	0	0	0	0
0	0	0	0	0	0	0	0	0	0	0	0	0	0
0	0	0	0	0	0	0	0	0	0	0	0	0	0
0	0	0	0	0	0	0	0	0	0	0	0	0	0
0	0	0	0	0	0	0	0	0	0	0	0	0	0
0													
OTU467	0	0	0	1	0	0	0	0	0	0	2	0	0
0	0	2	0	0	57	0	2	0	5	0	0	0	1
0	0	0	0	0	0	0	1	0	0	0	0	0	2
0	0	0	0	0	0	0	0	0	0	0	0	0	0
0	0	0	0	0	0	0	2	0	0	1	0	0	0
0	0	0	0	0	0	0	0	0	0	0	0	0	0

0	0	0	0	0	0	0	0	0	0	1	0	0	0
0	0	0	0	0	0	0	0	0	0	0	0	0	0
0													
OTU7195	0	0	0	1	1	1	0	2	0	0	1	1	0
0	1	0	0	0	0	1	0	0	0	0	0	0	0
0	0	0	0	0	0	0	0	2	0	0	0	1	0
1	0	0	0	0	3	0	3	0	0	1	1	0	0
0	1	1	2	0	0	0	0	0	0	0	0	2	0
0	0	0	0	1	0	0	0	0	2	0	0	0	0
0	0	0	0	1	1	0	2	0	0	0	0	3	0
0	0	0	0	0	0	0	0	0	0	0	0	0	1
0													
OTU14575	0	0	0	1	0	0	0	0	0	0	0	0	0
0	0	0	0	0	0	0	0	0	0	0	0	0	0
0	0	0	0	0	0	0	0	0	0	0	0	0	0
0	0	0	0	0	0	0	0	0	0	0	0	0	0
0	0	0	0	0	0	0	0	0	0	0	0	0	0
0	0	0	0	0	0	0	0	0	0	0	0	0	0
0	0	0	0	0	0	0	0	0	0	0	0	0	0
0	0	0	0	0	0	0	0	0	0	0	0	0	0
0													
OTU10898	0	0	0	1	0	0	0	0	0	0	0	0	0
0	0	0	0	0	0	0	0	0	0	0	0	0	0
0	0	0	0	0	0	0	0	0	0	0	0	0	0
0	0	0	0	0	0	0	0	0	0	0	0	0	0
0	0	0	0	0	0	0	0	0	0	0	0	0	0
0	0	0	0	0	0	0	0	0	0	0	0	0	0
0	0	0	0	0	0	0	0	0	0	0	0	0	0
0	0	0	0	0	0	0	0	0	0	0	0	0	0
0													
OTU10899	0	0	0	1	0	0	0	0	0	0	0	0	0
0	0	0	0	0	0	0	0	0	0	0	0	0	0
0	0	0	0	0	0	0	0	0	0	0	0	0	0

0 0 0 0 0 0 0 0 0 0 0 0 0 0
0 0 0 0 0 0 0 0 0 0 0 0 0 0
0 0 0 0 0 0 0 0 0 0 0 0 0 0
0 0 0 0 0 0 0 0 0 0 0 0 0 0
0 0 0 0 0 0 0 0 0 0 0 0 0 0
0
OTU14573 0 0 0 1 0 0 0 0 0 0 0 0 0
0 0 0 0 0 0 0 0 0 0 0 0 0 0
0 0 0 0 0 0 0 0 0 0 0 0 0 0
0 0 0 0 0 0 0 0 0 0 0 0 0 0
0 0 0 0 0 0 0 0 0 0 0 0 0 0
0 0 0 0 0 0 0 0 0 0 0 0 0 0
0 0 0 0 0 0 0 0 0 0 0 0 0 0
0 0 0 0 0 0 0 0 0 0 0 0 0 0
0
OTU15974 0 0 0 1 0 0 0 0 0 0 0 1 0
1 0 0 0 0 0 1 0 1 0 0 0 1 0
3 0 0 0 0 0 0 1 0 0 0 0 0 0
0 0 0 0 0 2 0 0 0 0 0 0 0 0
0 4 1 0 0 0 0 0 0 2 0 0 0 0
0 0 0 0 0 0 0 0 0 0 0 1 1 0
0 0 0 0 0 0 0 0 0 0 0 0 0 0
0 0 0 0 0 0 0 0 0 0 1 0 0 0
0
OTU14574 0 0 0 1 0 0 0 0 0 0 0 0 0
0 0 0 0 0 0 0 0 0 0 0 0 0 0
0 0 0 0 0 0 0 0 0 0 0 0 0 1
0 0 0 0 0 0 0 0 0 0 0 0 0 0
0 0 0 0 0 0 0 0 0 0 0 0 0 0
0 0 0 0 0 0 0 0 0 0 0 0 0 0
0 0 0 0 0 0 0 0 0 0 0 0 0 0
0 0 0 0 0 0 0 0 0 0 0 0 0 0
0

OTU225 0 0 0 1 0 0 0 0 0 0 16 0 3
9 3 0 0 10 1 0 0 0 20 0 1 0 90
0 0 5 0 0 13 0 0 31 0 0 36 0 1
0 0 0 1 1 0 1 3 0 0 0 0 0 29
0 0 2 0 0 1 0 0 0 3 0 0 0 1
0 0 3 1 1 0 3 0 1 1 4 0 0 0
6 0 5 0 1 0 1 0 11 0 0 0 0 5
0 0 0 0 0 0 0 0 0 0 0 0 0 0
0
OTU14668 0 0 0 1 0 0 0 0 0 0 0 0 0
0 0 0 0 0 0 0 0 0 0 0 0 0 0
0 0 0 0 0 0 0 0 0 0 0 0 0 0
0 0 0 0 0 0 0 0 0 0 0 0 0 0
0 0 0 0 0 0 0 0 0 0 0 0 0 0
0 0 0 0 0 0 0 0 0 0 0 0 0 0
0 0 0 0 0 0 0 0 0 0 0 0 0 0
0 0 0 0 0 0 0 0 0 0 0 0 0 0
0
OTU8362 0 0 0 1 0 0 0 0 0 0 0 0 0
0 0 0 0 0 0 0 0 0 0 0 0 0 0
0 0 0 0 0 0 0 0 0 0 0 0 0 0
0 0 0 0 0 0 0 0 0 0 0 0 0 0
0 0 0 0 0 0 0 0 0 0 0 0 0 0
0 0 0 0 0 0 0 0 0 0 0 0 0 0
0 0 0 0 0 0 0 0 0 0 0 0 0 0
0 0 0 0 0 0 0 0 0 0 0 0 0 0
0
OTU10901 0 0 0 1 0 0 0 0 0 0 0 0 0
0 0 0 0 0 0 0 0 0 0 0 0 0 0
0 0 0 0 0 0 0 0 0 0 0 0 0 0
0 0 0 0 0 0 0 0 0 0 0 0 0 0
0 0 0 0 0 0 0 0 0 0 0 0 0 0
0 0 0 0 0 0 0 0 0 0 0 0 0 0

0	0	0	0	0	0	0	0	0	0	0	0	0	0
0	0	0	0	0	0	0	0	0	0	0	0	0	0
0													
OTU8363	0	0	0	1	0	0	0	0	0	0	0	0	0
0	0	0	0	0	0	0	0	0	0	0	0	0	0
0	0	0	0	0	0	0	0	0	0	0	0	0	0
0	0	0	0	0	0	0	0	0	0	0	0	0	0
0	0	0	0	0	0	0	0	0	0	0	0	0	0
0	0	0	0	0	0	0	0	0	0	0	0	0	0
0	0	0	0	0	0	0	0	0	0	0	0	0	0
0	0	0	0	0	0	0	0	0	0	0	0	0	0
0													
OTU299	0	0	0	3	0	2	0	0	0	0	5	0	7
4	0	3	8	1	1	0	8	0	0	0	0	0	9
1	0	1	2	0	3	0	8	0	3	1	0	0	6
0	20	3	4	6	0	1	1	0	1	6	0	4	0
3	1	4	0	0	1	1	1	0	8	2	2	0	2
0	0	0	0	0	5	2	0	18	0	1	0	1	0
0	0	5	0	5	0	0	0	6	0	19	0	0	30
1	0	0	0	0	0	0	0	0	0	2	0	11	0
0													
OTU12960	0	0	0	1	1	0	0	0	0	0	0	0	0
0	0	0	0	0	0	0	0	0	0	5	0	0	0
0	0	0	0	0	0	0	0	0	0	0	0	0	0
0	0	0	0	2	0	0	0	1	0	5	0	2	0
1	0	5	0	2	0	5	0	0	0	0	0	0	0
0	0	0	0	0	0	0	0	0	0	0	0	0	0
0	0	0	0	0	0	0	0	0	0	0	0	0	0
0	0	0	0	0	0	0	0	0	0	0	0	0	0
0													
OTU4018	0	0	0	1	0	0	0	0	0	0	0	0	0
2	0	0	0	0	14	0	0	0	1	0	0	0	1
0	1	2	4	2	0	1	0	0	0	0	0	0	1

0	0	1	1	0	0	0	0	1	0	1	0	0	0
1	0	1	0	0	0	3	3	0	0	0	0	0	0
0	0	0	0	0	0	1	0	0	0	0	0	0	0
0	0	0	0	0	0	0	0	3	0	3	0	0	0
0	0	0	0	0	0	0	0	0	0	0	1	0	0
0													
OTU10902	0	0	0	1	0	0	0	0	0	0	0	0	0
0	0	0	0	0	0	0	0	0	0	0	0	0	0
0	0	0	0	0	0	0	0	0	0	0	0	0	0
0	0	0	0	0	0	0	0	0	0	0	0	0	0
0	0	0	0	0	0	0	0	0	0	0	0	0	0
0	0	0	0	0	0	0	0	0	0	0	0	0	0
0	0	0	0	0	0	0	0	0	0	0	0	0	0
0	0	0	0	0	0	0	0	0	0	0	0	0	0
0													
OTU14847	0	0	0	2	0	0	0	0	2	0	0	0	1
5	1	0	0	0	0	0	0	0	0	0	0	0	0
2	1	0	0	0	0	1	4	0	0	1	0	2	0
0	0	1	0	0	24	1	12	0	1	6	0	2	21
3	6	16	4	11	1	3	1	0	6	0	0	8	8
1	7	0	0	2	0	2	0	0	4	1	2	0	14
0	5	0	1	0	2	0	3	7	0	0	1	12	25
1	7	0	0	7	2	15	53	9	30	1	0	2	3
3													
OTU10903	0	0	0	3	0	1	0	0	0	1	0	1	0
0	0	0	0	0	0	0	3	0	0	0	0	0	0
0	0	0	0	0	0	0	1	0	0	0	0	2	0
0	0	0	0	0	0	0	0	1	0	0	0	0	0
0	0	0	3	0	0	1	0	0	0	0	0	0	0
0	0	0	5	0	0	0	0	0	1	1	0	1	0
0	0	0	0	0	0	14	0	0	0	0	0	0	0
7	0	0	0	0	0	0	0	0	0	2	0	2	0
0													

OTU14670	0	0	0	1	0	0	0	0	0	0	0	1	0
0	0	0	0	1	0	0	0	0	1	0	0	0	0
0	0	0	0	0	0	0	0	0	0	0	0	0	0
0	0	0	1	0	0	0	0	0	0	0	0	0	0
0	0	0	0	0	0	0	0	0	0	0	0	0	0
0	0	0	0	0	0	0	0	0	0	0	0	0	0
0	0	0	0	0	0	0	0	0	0	1	0	0	0
0	0	0	0	0	0	0	0	0	0	0	0	0	0
0													
OTU689	0	0	0	2	0	0	0	0	0	0	0	1	0
0	0	1	0	0	0	0	3	0	0	0	0	0	0
0	0	0	2	0	0	0	0	0	2	0	0	2	0
0	0	0	0	0	1	0	0	0	0	0	0	0	0
0	1	0	2	0	0	0	0	0	0	0	0	2	0
0	0	1	0	0	0	0	0	0	0	0	0	0	0
0	1	0	0	0	0	0	1	1	0	0	0	0	0
0	0	0	0	1	0	0	0	0	0	0	0	0	0
0													
OTU12276	0	0	0	1	0	0	0	0	0	0	0	0	0
0	0	0	0	0	0	0	0	0	0	0	0	0	0
0	0	0	0	0	0	0	0	0	0	0	0	1	0
0	0	0	0	0	0	0	0	0	0	0	0	0	0
0	0	0	0	0	0	0	0	0	0	0	0	0	0
0	0	0	0	0	0	0	0	0	0	0	0	0	0
0	0	0	0	0	0	0	0	0	0	0	0	0	0
0	0	0	0	0	0	0	0	0	0	0	0	0	0
0													
OTU14669	0	0	0	1	0	0	0	0	0	0	0	0	1
0	0	0	0	0	0	0	0	0	0	0	0	0	0
0	0	0	0	0	0	0	0	0	0	0	0	0	0
0	0	0	0	0	0	0	0	0	0	0	0	0	0
0	0	0	0	0	0	0	0	0	0	0	0	0	0
0	0	0	0	0	0	1	0	0	0	0	0	0	0

0	0	0	0	0	0	0	0	0	0	0	0	0	0
0	0	0	0	0	0	0	0	0	0	0	1	0	0
0													
OTU15733	0	0	0	1	0	1	0	0	0	0	0	0	0
0	0	0	0	0	0	0	0	0	0	0	0	0	0
0	0	0	0	0	0	0	0	0	0	0	0	0	0
0	0	0	0	0	0	0	0	0	0	0	0	0	0
0	0	0	1	0	0	0	0	0	0	0	0	0	0
0	0	0	0	0	0	0	0	0	0	0	0	0	0
0	0	0	0	1	0	0	0	0	0	0	0	0	0
0	0	0	0	0	0	0	0	0	0	0	0	0	0
0													
OTU11820	0	0	0	1	0	0	0	0	0	0	0	0	0
0	0	0	0	0	0	0	1	0	0	0	0	0	0
0	0	1	0	0	0	0	0	0	0	0	0	0	1
0	0	0	1	0	0	0	0	0	1	0	0	0	0
0	0	0	0	0	0	0	0	0	1	0	0	1	0
1	0	0	0	1	0	1	2	0	0	0	0	0	0
0	0	0	0	0	0	0	0	0	0	0	0	0	0
0	0	0	0	0	1	0	0	0	0	0	1	0	0
0													
OTU14310	0	0	0	1	0	0	0	0	0	0	8	3	5
9	0	0	0	0	0	3	0	0	0	0	1	0	0
0	0	0	0	0	2	0	2	9	0	0	0	0	6
0	0	0	0	0	0	4	1	0	0	0	0	0	2
0	0	0	1	0	0	0	1	0	0	0	0	0	0
0	0	0	1	0	0	1	0	5	0	0	0	1	0
9	0	2	0	7	0	0	0	3	0	0	1	0	1
2	0	0	0	0	0	0	0	0	0	3	2	0	0
0													
OTU3253	0	0	0	2	0	0	0	0	0	0	0	0	0
0	0	0	0	0	0	0	0	0	0	0	0	0	0
0	0	0	0	0	0	0	0	0	0	0	0	0	0

0	0	0	0	0	0	0	0	0	0	0	0	0	0
0	0	0	0	0	0	0	0	0	0	0	0	0	0
0	0	0	0	0	0	0	0	0	0	0	0	0	0
0	0	0	0	2	0	0	0	1	0	0	0	0	0
0	0	0	0	0	0	0	0	0	0	0	0	0	0
0													
OTU3521	0	0	0	3	1	1	0	1	1	0	0	0	0
0	0	0	0	0	0	0	0	0	0	0	0	0	8
0	0	0	0	0	0	0	0	2	0	0	0	1	0
1	0	0	0	1	0	0	2	0	0	0	0	0	0
0	0	1	4	0	0	0	0	0	0	0	1	4	0
0	0	1	0	2	2	0	0	0	0	1	1	0	1
0	0	0	1	0	2	2	0	0	0	0	0	0	0
0	0	0	0	0	0	0	0	0	0	0	0	0	0
0													
OTU4761	0	0	0	1	0	0	0	0	0	0	0	0	1
0	0	0	0	0	0	0	0	0	0	0	0	0	0
0	0	1	0	0	0	1	1	0	0	0	0	0	0
0	0	0	0	0	0	1	0	2	0	0	1	0	0
1	0	2	0	0	0	0	0	0	1	1	2	0	0
0	0	0	1	0	0	0	0	0	0	0	0	0	0
0	0	0	0	0	0	7	0	0	0	0	0	0	4
0	0	0	0	0	0	0	0	0	0	0	0	1	0
0													
OTU369	0	0	0	1	0	0	0	0	0	0	4	0	10
1	0	0	0	4	2	1	2	0	18	0	2	1	0
0	0	3	0	0	0	0	0	0	0	0	0	0	1
0	0	0	2	1	1	2	0	0	0	0	0	0	0
0	0	0	0	0	0	0	0	0	1	0	0	0	0
0	0	0	0	0	0	2	0	1	0	1	0	1	0
0	0	0	0	1	0	0	0	19	0	1	0	0	1
0	0	0	0	0	0	0	0	0	0	0	1	1	0
0													

OTU8360	0	0	0	1	0	0	0	0	0	0	0	0	0
0	0	0	0	0	0	0	0	0	0	0	0	0	1
0	0	0	0	0	0	0	0	0	0	0	0	1	0
0	0	0	0	0	0	0	0	0	0	0	0	0	0
0	0	0	1	0	0	0	0	0	0	0	0	1	0
0	0	0	0	0	0	0	0	0	1	0	0	0	0
0	0	0	0	0	1	1	0	0	0	0	0	0	0
0	0	0	0	0	0	0	0	0	0	0	0	0	0
0													
OTU185	0	0	0	1	0	0	1	0	0	0	98	5	7
1	0	13	0	6	4	0	0	0	0	0	0	0	0
0	0	0	0	0	10	0	64	3	0	0	1	0	0
0	4	0	0	0	0	3	0	0	0	8	0	2	31
0	13	0	0	0	0	0	7	0	0	0	0	0	0
0	0	0	0	0	0	0	0	25	0	0	0	3	0
3	1	3	0	33	2	0	0	1	0	22	0	0	22
4	0	0	0	0	0	0	0	2	0	2	8	0	0
0													
OTU7611	0	0	0	1	0	0	0	0	0	2	0	0	0
0	1	0	0	0	1	0	0	0	0	0	0	0	0
0	0	0	0	0	0	0	0	0	0	0	1	4	0
0	0	0	1	0	0	0	0	0	0	0	0	0	0
0	0	0	0	1	1	1	0	0	0	0	0	1	0
1	0	0	0	1	0	0	0	0	0	0	0	0	0
2	0	0	0	0	0	0	0	0	0	0	0	0	0
0	0	0	0	0	1	0	0	0	0	0	0	0	1
3													
OTU7485	0	0	0	2	0	0	0	0	0	0	0	0	0
0	0	0	0	0	0	0	1	0	0	0	0	0	0
0	0	0	0	0	0	0	0	0	0	0	0	0	0
0	0	0	0	0	1	0	0	0	0	0	0	0	0
0	0	0	0	0	0	0	0	1	0	0	0	0	0
0	0	0	0	0	0	0	0	0	0	0	1	0	0

0	0	0	0	0	0	0	0	0	0	0	0	0	0
0	0	0	0	0	0	0	0	0	0	0	0	0	0
0													
OTU10615	0	0	0	4	0	1	0	0	0	0	0	7	0
0	0	1	0	0	0	6	1	0	0	0	1	0	0
0	0	0	0	0	0	0	0	0	3	1	0	4	0
1	5	0	0	0	1	0	0	0	2	1	0	0	0
0	1	0	9	0	0	0	0	0	0	0	0	8	0
0	1	0	0	0	0	1	0	0	0	0	1	0	3
0	0	0	0	0	0	0	0	0	0	0	1	1	0
0	4	1	2	5	0	0	2	0	1	0	0	0	2
2													
OTU14666	0	0	0	1	0	0	0	0	0	0	0	0	0
0	0	0	0	0	0	0	0	0	0	0	0	0	0
0	0	0	0	0	0	0	0	0	0	0	0	0	0
0	0	0	0	0	0	0	0	0	0	0	0	0	0
0	0	0	0	0	0	0	0	0	0	0	0	0	0
0	0	0	0	0	0	0	0	0	0	0	0	0	0
0	0	0	0	0	0	0	0	0	0	0	0	0	0
0	0	0	0	0	0	0	0	0	0	0	0	0	0
0													
OTU388	0	0	0	1	1	1	1	0	0	0	1	4	0
3	0	5	0	1	6	2	0	0	0	0	4	0	1
0	0	3	0	0	0	0	0	3	0	0	2	0	0
1	3	0	0	0	0	1	4	0	0	0	0	0	12
0	5	0	4	0	0	0	0	0	0	0	0	0	0
0	0	13	1	0	1	0	0	0	3	0	0	6	1
0	0	2	0	1	5	1	0	0	1	2	0	0	1
1	0	0	0	0	0	0	1	1	0	1	0	0	1
0													
OTU469	0	0	0	1	0	0	0	0	0	0	0	0	0
0	0	0	0	0	0	1	0	0	0	0	0	0	0
0	0	0	0	1	0	1	0	0	0	2	0	0	0

0	0	0	0	1	1	0	0	0	0	0	0	0	0
0	0	1	0	0	0	0	0	0	0	0	0	1	1
0	1	1	0	0	0	0	0	0	0	0	0	0	0
0	0	0	0	0	0	0	0	0	0	0	0	0	0
0	0	3	7	0	3	2	6	0	7	0	0	0	7
1													
OTU8359	0	0	0	1	0	0	0	0	0	0	0	0	0
0	0	0	0	0	0	0	0	0	0	0	0	0	0
0	0	0	0	0	0	0	0	0	0	0	0	0	0
0	0	0	0	0	0	0	0	0	0	0	0	0	0
0	0	0	0	0	0	0	0	0	0	0	0	0	0
0	0	0	0	0	0	0	0	0	0	0	0	0	0
0	0	0	0	0	0	0	0	0	0	0	0	0	0
0	0	0	0	0	0	0	0	0	0	0	0	0	0
0													
OTU8221	0	0	0	2	0	0	0	0	0	0	0	0	0
0	0	0	0	0	0	0	0	0	0	0	0	0	0
0	0	0	0	0	0	0	0	0	0	0	0	0	0
0	0	0	0	1	0	0	0	0	0	0	0	0	0
0	0	0	0	0	0	0	0	0	0	0	0	0	0
0	0	0	0	0	0	0	0	0	0	0	0	0	0
0	0	0	0	0	0	0	1	0	0	0	0	0	0
0	0	0	0	0	0	0	0	0	1	0	0	0	0
0													
OTU14667	0	0	0	1	0	0	0	0	0	0	0	0	0
0	0	0	0	0	0	0	0	0	0	0	0	0	0
0	0	0	0	0	0	0	0	0	0	0	0	0	0
0	0	0	0	0	0	0	0	0	0	0	0	0	0
0	0	0	0	0	0	0	0	0	0	0	0	0	0
0	0	0	0	0	0	0	0	0	0	0	0	0	0
0	0	0	0	0	0	0	0	0	0	0	0	0	0
0	0	0	0	0	0	0	0	0	0	0	0	0	0
0													

OTU839	0	0	0	2	0	0	0	1	0	1	0	1	0
2	0	0	0	0	0	2	0	0	0	0	0	1	3
0	0	0	0	0	0	0	3	0	0	0	0	2	0
1	0	0	0	1	2	0	0	0	0	2	0	0	0
0	1	0	1	1	0	0	0	0	0	0	1	1	0
0	1	2	0	4	0	0	0	4	0	0	0	0	1
0	0	0	0	2	0	1	0	0	0	2	0	2	0
1	3	0	1	0	0	0	2	0	0	0	1	0	0
1													
OTU7653	0	0	0	2	0	0	0	0	2	0	0	0	0
0	0	0	0	0	1	0	0	0	0	0	0	0	0
0	0	0	0	0	0	0	0	1	0	0	0	0	0
0	1	0	0	0	0	0	0	0	0	0	1	0	0
0	0	0	1	0	0	0	1	1	1	0	0	0	0
0	0	0	0	0	0	0	0	0	0	0	0	0	0
0	0	0	2	0	0	0	0	0	0	0	0	0	0
0	2	3	2	1	1	2	0	0	1	0	0	0	4
0													
OTU1143	0	0	0	1	2	1	0	0	0	0	0	0	0
0	0	0	0	0	1	0	0	0	0	0	1	0	0
0	0	0	0	0	0	0	0	0	0	0	0	0	0
1	0	0	0	2	0	0	0	0	0	0	1	0	0
0	1	0	0	0	1	0	0	1	0	1	0	0	0
0	0	0	0	0	0	0	0	0	0	0	0	0	1
0	1	0	0	0	0	0	0	0	0	0	0	0	0
0	0	1	0	0	0	0	0	0	0	0	0	0	0
0													
OTU6194	0	0	0	1	0	0	0	0	1	0	12	0	0
0	0	3	0	0	55	0	0	0	2	0	1	0	0
0	0	21	3	0	1	0	0	0	1	0	0	0	17
0	0	11	0	2	0	0	0	9	0	1	0	1	0
0	0	0	0	0	2	0	5	0	0	2	0	1	0
3	0	0	0	0	0	1	0	0	0	0	0	0	0

0	2	0	0	0	1	0	0	0	0	0	0	0	1
1	0	0	0	0	0	0	0	0	0	0	1	0	0
0													
OTU776	0	0	0	1	0	1	0	9	0	0	4	0	18
6	0	18	0	0	42	0	10	0	2	2	0	0	0
0	0	57	4	2	8	0	48	3	1	0	4	0	58
0	1	0	2	0	0	28	0	0	0	0	0	0	1
0	0	0	0	0	0	0	1	0	0	0	0	0	0
0	0	0	24	0	1	2	0	15	0	2	0	0	0
0	0	88	0	0	1	0	0	15	39	31	0	0	10
13	0	3	0	0	0	0	0	8	0	1	48	9	0
0													
OTU9898	0	0	0	1	0	0	0	0	1	1	0	0	1
0	0	0	0	0	0	0	0	0	0	0	1	0	0
0	0	0	0	0	0	0	1	0	0	0	0	0	0
0	0	0	0	0	0	0	0	0	0	0	0	0	0
0	0	0	0	0	0	0	0	0	0	0	0	0	0
0	0	0	0	0	0	0	0	0	0	0	0	0	0
0	0	0	0	0	1	0	0	0	0	0	0	1	0
0	0	0	0	0	0	0	0	0	0	0	0	0	0
0													
OTU16749	0	0	0	1	0	0	0	0	3	0	1	0	0
0	0	1	0	1	0	0	2	0	0	0	0	0	0
0	0	0	0	0	0	0	0	0	0	0	0	0	3
0	0	1	0	2	0	0	0	0	0	0	0	0	0
0	0	0	0	0	0	0	0	0	0	0	0	0	0
0	0	0	0	0	0	0	0	0	0	0	0	0	0
0	0	0	0	0	0	0	0	0	0	0	0	0	0
0	0	0	0	0	0	0	0	0	0	0	0	0	0
0													
OTU13489	0	0	0	1	0	0	0	0	0	0	0	0	0
0	0	0	0	0	0	0	0	0	0	0	0	0	0
0	0	0	0	0	0	0	0	0	0	0	0	0	1

0	0	0	0	0	0	0	0	0	0	0	0	0	0
0	0	0	0	0	0	0	0	0	0	0	0	0	0
0	0	0	1	0	0	0	0	1	0	0	0	0	0
0	0	2	0	0	0	0	0	0	0	0	0	0	1
1	0	0	0	0	0	0	0	0	0	0	2	2	0
0													
OTU1153	0	0	0	1	0	0	0	0	0	0	0	0	0
0	1	1	3	1	3	0	0	0	5	4	1	0	0
0	0	0	0	0	0	1	0	1	1	0	3	0	0
0	1	0	0	1	0	0	0	3	0	1	1	3	0
0	0	2	0	1	0	1	1	0	0	0	0	0	8
1	0	2	0	0	1	0	0	0	1	0	0	0	0
1	1	1	0	0	0	0	0	0	0	0	0	0	3
0	0	0	0	0	0	0	1	0	0	0	0	0	0
0													
OTU1563	0	0	0	1	0	0	0	0	0	0	4	0	0
0	0	0	0	0	1	0	1	0	0	0	0	0	0
0	0	1	1	0	0	1	0	0	0	0	0	0	0
0	0	0	0	0	0	0	0	0	0	0	0	0	0
0	1	0	0	0	0	0	0	0	0	0	0	2	0
1	0	0	0	0	0	0	0	0	0	1	0	0	0
0	0	0	1	0	0	0	0	0	0	0	0	0	0
0	0	0	0	0	2	0	0	0	0	0	0	0	1
0													
OTU12267	0	0	0	1	1	0	0	0	0	0	0	0	0
0	0	0	0	0	0	0	0	0	0	0	0	0	0
0	0	0	0	0	0	0	0	0	0	0	0	0	0
0	0	0	0	0	0	0	0	0	0	0	0	0	0
0	0	0	0	0	0	0	0	0	0	0	0	0	0
0	0	0	0	0	0	0	0	0	0	0	0	0	0
0	0	0	0	0	0	0	0	0	0	0	0	0	0
0	0	0	0	0	0	0	0	0	0	0	0	0	0
0													

OTU8319	0	0	0	1	0	0	0	0	0	0	0	0	0
0	0	0	0	0	0	0	0	0	0	0	0	0	0
0	0	0	0	0	0	0	0	0	0	0	0	0	0
0	0	0	0	0	0	0	0	0	0	0	0	0	0
0	0	0	0	0	0	0	0	0	0	0	0	0	0
0	0	0	0	0	0	0	0	0	0	0	0	0	0
0	0	0	0	0	0	0	0	0	0	0	0	0	0
0	0	0	0	0	0	0	0	0	0	0	0	0	0
0													
OTU2652	0	0	0	1	0	0	0	0	1	0	0	0	0
0	0	0	0	0	0	0	0	0	0	0	0	0	0
1	0	0	0	0	0	0	0	0	1	0	0	0	0
3	0	0	0	0	2	0	0	0	0	0	0	0	0
2	0	0	0	0	0	2	0	2	0	0	0	2	0
0	0	0	0	0	0	0	0	0	0	0	0	0	0
0	0	0	0	0	0	0	0	0	0	0	0	0	1
0	0	9	0	4	3	0	0	10	0	0	1	0	0
0													
OTU9477	0	0	0	1	0	0	0	0	0	0	1	0	0
0	0	0	0	0	0	0	0	0	0	0	0	0	0
0	0	0	1	0	0	0	0	0	0	0	0	0	1
0	0	0	0	0	0	0	0	2	0	0	0	0	0
0	0	0	0	0	0	0	0	0	0	0	0	0	0
0	0	0	0	0	0	0	0	0	0	0	0	0	0
0	1	0	0	0	0	0	0	0	0	0	0	0	0
0	0	0	0	0	0	0	0	0	0	0	0	0	0
0													
OTU12268	0	0	0	1	0	0	0	0	0	0	0	0	0
0	0	0	0	0	0	0	0	0	0	0	0	0	0
0	0	0	0	0	0	0	0	0	0	0	0	0	0
0	0	0	0	0	0	0	0	0	0	0	0	0	0
0	0	0	0	0	0	0	0	0	0	0	0	0	0
0	0	0	0	0	0	0	0	0	0	0	0	0	0

0	0	0	0	0	0	0	0	0	0	0	0	0	0
0	0	0	0	0	0	0	0	0	0	0	0	0	0
0													
OTU12269	0	0	0	1	0	0	0	0	0	0	0	0	0
0	0	0	0	0	0	0	0	0	0	0	0	0	0
0	0	0	0	0	0	0	0	0	0	0	0	0	0
0	0	0	0	0	0	0	0	0	0	0	0	0	0
0	0	0	0	0	0	0	0	0	0	0	0	0	0
0	0	0	0	0	0	0	0	0	0	0	0	0	0
0	0	0	0	0	0	0	0	0	0	0	0	0	0
0	0	0	0	0	0	0	0	0	0	0	0	0	0
0													
OTU10929	0	0	0	1	0	0	0	0	0	0	0	0	0
0	0	0	0	0	0	0	0	0	0	0	0	0	0
0	0	0	0	0	0	0	0	0	0	0	0	0	0
0	0	0	0	0	0	0	0	0	0	0	0	0	0
0	0	0	0	0	0	0	0	0	0	0	0	0	0
0	0	0	0	0	0	0	0	0	0	0	0	0	0
0	0	0	0	0	0	0	0	0	0	0	0	0	0
0	0	0	0	0	0	0	0	0	0	0	0	0	0
0													
OTU8647	0	0	0	1	0	0	0	0	0	0	0	0	0
1	0	0	0	0	0	0	0	0	0	0	0	0	0
0	0	0	0	0	0	0	0	3	0	0	0	0	0
0	0	0	0	0	0	0	0	0	0	0	0	0	0
0	0	0	0	0	0	0	0	0	0	0	0	0	0
0	0	0	0	0	0	0	0	0	0	0	0	0	0
0	0	0	0	0	1	0	0	0	0	0	0	0	0
0	0	1	0	0	0	0	0	1	1	0	0	0	1
0													
OTU15836	0	0	0	1	0	0	0	2	0	0	0	1	0
0	1	0	0	0	0	0	0	0	0	0	1	0	0
0	1	1	0	0	0	0	0	0	0	0	0	3	0

0	1	0	1	0	1	0	0	1	3	0	0	1	0
0	0	0	1	1	0	0	1	0	0	0	0	0	1
3	2	0	1	0	1	0	0	0	0	0	1	0	1
0	0	0	1	0	1	1	1	0	0	0	0	0	0
0	0	0	2	2	0	0	0	0	0	0	0	0	0
2													
OTU8217	0	0	0	1	0	0	0	0	1	0	0	0	0
0	0	0	0	0	0	0	0	0	0	0	0	0	0
0	0	0	0	0	0	0	0	0	0	0	0	0	0
0	0	0	0	0	0	0	0	0	0	0	0	0	0
0	0	0	0	0	0	0	0	0	0	0	0	0	0
0	0	0	0	0	0	0	0	0	0	0	0	0	0
0	0	0	0	0	0	0	0	0	0	0	0	0	0
0	0	3	2	1	7	4	0	3	2	0	0	0	1
0													
OTU10930	0	0	0	1	0	0	0	0	0	0	0	0	0
0	0	0	0	0	0	0	0	0	0	0	0	0	0
0	0	0	0	0	0	0	0	0	0	0	0	0	0
0	0	0	0	0	0	0	0	0	0	0	0	0	0
0	0	0	0	0	0	0	0	0	0	0	0	0	0
0	0	0	0	0	0	0	0	0	0	0	0	0	0
0	0	0	0	0	0	0	0	0	0	0	0	0	0
0	0	0	0	0	0	0	0	0	0	0	0	0	0
0													
OTU13622	0	0	0	1	0	0	0	0	0	0	0	0	0
0	0	0	0	0	0	1	0	0	0	0	0	0	0
0	0	0	0	0	0	0	0	0	0	1	0	1	0
2	0	0	0	1	0	0	0	2	0	1	1	0	0
1	0	0	0	0	0	0	1	3	0	0	0	1	0
0	0	0	0	0	0	0	1	1	0	0	1	0	0
0	1	0	0	0	0	0	0	0	0	0	0	0	0
0	0	0	0	0	0	0	0	0	0	0	0	0	0
0													

OTU16240 0 0 0 1 0 0 0 0 0 0 0 0 0
0 0 0 0 0 0 0 0 0 0 0 0 0 0
0 0 0 0 0 0 0 0 0 0 0 0 0 0
0 0 5 0 0 0 0 0 0 0 0 0 0 0
0 0 3 0 2 0 1 0 0 5 0 0 0 13
0 0 0 0 0 0 0 0 0 0 0 0 0 0
0 0 0 0 0 0 0 0 0 0 0 0 0 0
0 0 0 0 0 0 0 0 1 0 0 0 0 0
0
OTU12266 0 0 0 1 0 0 0 0 0 0 0 0 0
0 0 0 0 0 0 0 0 0 0 0 0 0 0
0 0 0 0 0 0 0 0 0 0 0 0 0 0
0 0 0 0 0 0 0 0 0 0 0 0 0 0
0 0 0 0 0 0 0 0 0 0 0 0 0 0
0 0 0 0 0 0 0 0 0 0 0 0 0 0
0 0 0 0 0 0 0 0 0 0 0 0 0 0
0 0 0 0 0 0 0 0 0 0 0 0 0 0
0
OTU918 0 0 0 2 0 0 0 0 2 0 0 0 0
1 0 0 0 1 0 1 0 0 0 0 0 0 0
0 0 0 0 0 0 0 0 0 0 0 0 0 2
0 0 0 1 0 0 0 0 0 0 0 0 0 0
0 2 0 0 0 0 0 0 0 0 0 0 0 0
0 0 0 0 0 0 0 0 0 0 0 0 0 0
0 0 0 0 0 0 0 0 0 0 0 0 0 0
0 1 0 0 0 0 0 0 0 0 0 0 0 0
0
OTU12264 0 0 0 1 0 0 0 0 0 0 0 0 0
0 0 0 0 0 0 0 0 0 0 0 0 0 0
0 0 0 0 0 0 0 0 0 0 0 0 0 0
0 0 0 0 0 0 0 0 0 0 0 0 0 0
0 0 0 0 0 0 0 0 0 0 0 0 0 0
0 0 0 0 0 0 0 0 0 0 0 0 0 0

0	0	0	0	0	0	0	0	0	0	0	0	0	0
0	0	0	0	0	0	0	0	0	0	0	0	0	0
0													
OTU8318	0	0	0	1	0	0	0	0	0	0	0	0	0
0	0	0	0	0	0	0	0	0	0	0	0	0	0
0	0	0	0	0	0	0	0	0	0	0	0	0	0
0	0	0	0	0	0	0	0	0	0	0	0	0	0
0	0	0	0	0	0	0	0	0	0	0	0	0	0
0	0	0	0	0	0	0	0	0	0	0	0	0	0
0	0	0	0	0	0	0	0	0	0	0	0	0	0
0	0	0	0	0	0	0	0	0	0	0	0	0	0
0													
OTU5304	0	0	0	1	0	0	1	0	0	0	0	0	0
0	1	0	0	0	0	0	0	0	0	0	0	0	0
0	0	0	0	0	0	0	0	2	0	0	1	0	0
0	0	0	0	0	0	0	3	0	0	0	0	0	3
0	0	0	0	0	0	0	0	0	0	0	0	0	0
0	0	0	0	0	0	0	0	0	0	0	0	0	0
0	0	0	0	0	0	0	0	0	2	0	0	0	2
0	0	0	0	0	0	0	0	0	0	0	2	0	0
0													
OTU12263	0	0	0	1	0	0	0	0	0	0	0	0	0
0	0	0	0	0	0	0	0	0	0	0	0	0	0
0	0	0	0	0	0	0	0	0	0	0	0	0	0
0	0	0	0	0	0	0	0	0	0	0	0	0	0
0	0	0	0	0	0	0	0	0	0	0	0	0	0
0	0	0	0	0	0	0	0	0	0	0	0	0	0
0	0	0	0	0	0	0	0	0	0	0	0	0	0
0	0	0	0	0	0	0	0	0	0	0	0	0	0
0													
OTU992	0	0	0	1	2	0	0	0	0	0	0	0	0
0	0	0	0	0	0	0	0	0	0	0	0	0	0
0	0	0	0	0	1	0	1	1	0	0	0	0	0

2	0	0	0	0	1	0	0	0	0	0	0	0	0
0	1	1	0	0	0	0	0	0	0	0	0	0	0
0	0	0	0	0	0	0	0	0	1	0	0	0	0
0	0	0	0	0	0	0	0	0	0	0	0	0	0
0	0	0	0	0	0	0	0	0	0	0	1	0	0
0													
OTU8325	0	0	0	1	0	0	0	0	0	0	0	0	0
0	0	0	0	0	0	0	0	0	0	0	0	0	0
0	0	0	0	0	0	0	0	0	0	0	0	0	0
0	0	0	0	0	0	0	0	0	0	0	0	0	0
0	0	0	0	0	0	0	0	0	0	0	0	0	0
0	0	0	0	0	0	0	0	0	0	0	0	0	0
0	0	0	0	0	0	0	0	0	0	0	0	0	0
0	0	0	0	0	0	0	0	0	0	0	0	0	0
0													
OTU13998	0	0	0	2	0	0	0	0	0	0	0	0	0
0	0	0	0	0	0	0	0	0	0	0	0	0	0
0	0	0	1	0	1	0	0	0	0	0	1	0	0
0	0	0	0	0	0	0	2	0	0	1	0	0	0
0	1	0	0	0	0	0	0	0	0	0	0	1	0
0	0	0	0	0	0	0	0	0	0	0	0	0	0
0	0	0	0	0	0	0	0	0	0	0	0	0	0
0	0	0	0	0	0	0	0	0	0	1	0	1	0
0													
OTU500	0	0	0	1	0	0	0	0	2	1	0	0	0
0	0	0	0	0	0	0	0	0	0	0	0	0	0
4	0	0	0	0	0	0	0	0	0	0	0	0	0
0	0	0	0	0	0	0	0	0	2	1	4	0	0
0	3	0	0	0	0	0	1	0	0	0	1	1	0
0	4	0	0	0	0	0	0	2	4	0	3	0	1
0	0	0	4	0	3	3	6	0	0	0	2	2	0
0	5	2	1	2	3	1	0	1	1	0	0	0	2
1													

OTU8326 0 0 0 1 0 0 0 0 0 0 0 0 0
0 0 0 0 0 0 0 0 0 0 0 0 0 0
0 0 0 0 0 0 0 0 0 0 0 0 0 0
0 0 0 0 0 0 0 0 0 0 0 0 0 0
0 0 0 0 0 0 0 0 0 0 0 0 0 0
0 0 0 0 0 0 0 0 0 0 0 0 0 0
0 0 0 0 0 0 0 0 0 0 0 0 0 0
0 0 0 0 0 0 0 0 0 0 0 0 0 0
0
OTU8323 0 0 0 1 0 0 0 0 0 0 0 0 0
0 0 0 0 0 0 0 0 0 0 0 0 0 0
0 0 0 0 0 0 0 0 0 0 0 0 0 0
0 0 0 0 0 0 0 0 0 0 0 0 0 0
0 0 0 0 0 0 0 0 0 0 0 0 0 0
0 0 0 0 0 0 0 0 0 0 0 0 0 0
0 0 0 0 0 0 0 0 0 0 0 0 0 0
0 0 0 0 0 0 0 0 0 0 0 0 0 0
0
OTU8324 0 0 0 1 0 0 0 0 0 0 0 0 0
0 0 0 0 0 0 0 0 0 0 0 0 0 0
0 0 0 0 0 0 0 0 0 0 0 0 0 0
0 0 0 0 0 0 0 0 0 0 0 0 0 0
0 0 0 0 0 0 0 0 0 0 0 0 0 0
0 0 0 0 0 0 0 0 0 0 0 0 0 0
0 0 0 0 0 0 0 0 0 0 0 0 0 0
0 0 0 0 0 0 0 0 0 0 0 0 0 0
0
OTU10927 0 0 0 1 0 0 0 0 0 0 0 0 0
0 0 0 0 0 0 0 0 0 0 0 0 0 0
0 0 0 0 0 0 0 0 0 0 0 0 0 0
0 0 0 0 0 0 0 0 0 0 0 0 0 0
0 0 0 0 0 0 0 0 0 0 0 0 0 0
0 0 0 0 0 0 0 0 0 0 0 0 0 0

	0	0	0	0	0	0	0	0	0	0	0	0	0	0
	0	0	0	0	0	0	0	0	0	0	0	0	0	0
	0													
OTU834		0	0	0	1	0	0	0	0	0	0	0	0	0
	0	0	0	0	0	0	0	0	0	0	0	0	0	0
	1	0	0	0	0	0	0	0	0	0	0	0	0	0
	0	0	0	0	0	0	0	0	0	0	0	0	0	0
	0	0	0	4	0	0	0	0	0	0	0	0	0	0
	0	0	0	0	0	0	0	0	0	5	0	0	0	0
	0	0	0	0	0	0	0	0	0	0	0	0	0	0
	0	0	0	0	0	0	0	0	0	0	0	0	0	0
	0													
OTU6463		0	0	0	1	0	0	0	0	0	0	0	0	1
	4	0	0	0	0	4	0	0	0	0	0	0	0	0
	0	0	1	1	0	0	0	0	0	0	0	0	0	0
	1	0	0	0	0	0	0	0	1	0	0	0	2	0
	2	0	0	0	0	0	0	0	0	0	1	1	0	0
	0	0	0	0	0	0	0	0	0	0	0	0	0	0
	0	0	1	0	0	0	0	0	2	0	0	0	0	0
	0	0	0	0	0	0	0	0	0	0	0	2	0	0
	0													
OTU13189		0	0	0	1	0	0	1	0	0	0	0	1	0
	0	1	1	0	0	0	0	0	0	0	0	0	0	0
	0	0	0	0	0	0	0	0	0	0	0	0	0	0
	3	1	0	0	0	0	0	0	0	0	0	0	0	0
	0	0	0	0	0	0	0	0	0	0	0	0	0	0
	0	0	0	0	0	0	0	0	1	0	0	0	1	0
	0	0	0	0	0	0	0	0	0	0	0	0	0	0
	0	0	0	0	0	0	0	0	0	0	1	0	0	0
	0													
OTU10928		0	0	0	1	0	0	0	0	0	0	0	0	0
	0	0	0	0	0	0	0	0	0	0	0	0	0	0
	0	0	0	0	0	0	0	0	0	0	0	0	0	0

0	0	0	0	0	0	0	0	0	0	0	0	0	0
0	0	0	0	0	0	0	0	0	0	0	0	0	0
0	0	0	0	0	0	0	0	0	0	0	0	0	0
0	0	0	0	0	0	0	0	0	0	0	0	0	0
0	0	0	0	0	0	0	0	0	0	0	0	0	0
0													
OTU759	0	0	0	1	1	3	0	8	0	0	1	1	4
0	1	0	6	1	1	1	0	7	0	10	0	5	0
0	3	0	1	1	0	5	1	2	2	12	0	5	1
2	1	0	1	3	0	0	8	0	0	0	1	2	0
1	0	0	0	2	0	0	0	0	0	0	0	1	1
0	46	0	0	0	0	0	0	0	0	3	0	1	1
0	2	0	0	1	0	0	2	21	0	0	12	5	1
0	2	137	93	30	30	63	26	35	24	0	1	1	40
67													
OTU1793	0	0	0	1	0	0	0	0	0	0	0	1	0
1	0	0	0	0	0	0	0	0	0	0	1	0	0
0	0	0	0	0	0	0	0	0	1	0	0	0	0
0	0	0	0	0	0	0	0	0	0	1	1	0	0
0	0	0	0	0	0	1	1	0	0	1	0	0	1
0	0	1	0	1	0	0	0	0	0	0	1	0	0
0	0	0	0	0	0	0	1	0	0	0	0	0	1
0	0	0	0	0	1	0	1	0	2	0	0	0	1
0													
OTU8320	0	0	0	1	0	0	0	0	0	0	0	0	0
0	0	0	0	0	0	0	0	0	0	0	0	0	0
0	0	0	0	0	0	0	0	0	0	0	0	0	0
0	0	0	0	0	0	0	0	0	0	0	0	0	0
0	0	0	0	0	0	0	0	0	0	0	0	0	0
0	0	0	0	0	0	0	0	0	0	0	0	0	0
0	0	0	0	0	0	0	0	0	0	0	0	0	0
0	0	0	0	0	0	0	0	0	0	0	0	0	0
0													

OTU4542	0	0	0	1	0	0	0	0	0	0	1	0	0
0	0	2	0	1	0	0	0	0	0	0	0	0	0
0	0	0	0	0	0	0	1	0	0	0	0	0	0
0	0	0	0	0	0	0	0	0	0	0	0	0	1
0	0	0	0	1	0	0	0	0	0	0	0	0	0
0	0	0	0	0	0	0	0	1	0	0	0	0	0
0	0	0	0	1	0	0	0	1	0	0	0	0	0
1	0	0	0	0	0	0	0	1	0	1	0	1	0
0													
OTU14663	0	0	0	1	0	0	0	0	0	0	0	0	0
0	0	0	0	0	0	0	0	0	0	0	0	0	0
0	0	0	0	0	0	0	0	0	0	0	0	0	0
0	0	0	0	0	0	0	0	0	0	0	0	0	0
0	0	0	0	0	0	0	0	0	0	0	0	0	0
0	0	0	0	0	0	0	0	0	0	0	0	0	0
0	0	0	0	0	0	0	0	0	0	0	0	0	0
0	0	0	0	0	0	0	0	0	0	0	0	0	0
0													
OTU12270	0	0	0	1	0	0	0	0	0	0	0	0	0
0	0	0	0	0	0	0	0	0	0	0	0	0	0
0	0	0	0	0	0	0	0	0	0	0	0	0	0
0	0	0	0	0	0	0	0	0	0	0	0	0	0
0	0	0	0	0	0	0	0	0	0	0	0	0	0
0	0	0	0	0	0	0	0	0	0	0	0	0	0
0	0	0	0	0	0	0	0	0	0	0	0	0	0
0	0	0	0	0	0	0	0	0	0	0	0	0	0
0													
OTU12271	0	0	0	1	0	0	0	0	0	0	0	0	0
0	0	0	0	0	0	0	0	0	0	0	0	0	0
0	0	0	0	0	0	0	0	0	0	0	0	0	0
0	0	0	0	0	0	0	0	0	0	0	0	0	0
0	0	0	0	0	0	0	0	0	0	0	0	0	0
0	0	0	0	0	0	0	0	0	0	0	0	0	0

0	0	0	0	0	0	0	0	0	0	0	0	0	0
0	0	0	0	0	0	0	0	0	0	0	0	0	0
0													
OTU2556	0	0	0	1	0	0	0	0	0	0	0	2	0
0	0	0	0	0	0	0	1	2	0	0	0	0	0
0	0	0	0	0	0	0	0	1	0	2	0	0	0
2	0	0	0	0	0	0	0	0	0	0	0	0	0
0	0	0	2	1	0	0	0	0	0	0	0	0	0
0	0	1	0	0	0	0	0	0	0	0	0	0	1
0	0	0	0	0	0	2	1	0	0	0	0	0	0
0	0	0	0	0	0	0	0	0	0	0	0	0	0
0													
OTU12272	0	0	0	1	0	0	0	0	0	0	0	0	0
0	0	0	0	0	0	0	0	0	0	0	0	0	0
0	0	0	0	0	0	0	0	0	0	0	0	0	0
0	0	0	0	0	0	0	0	0	0	0	0	0	0
0	0	0	0	0	0	0	0	0	0	0	0	0	0
0	0	0	0	0	0	0	0	0	0	0	0	0	0
0	0	0	0	0	0	0	0	0	0	0	0	0	0
0	0	0	0	0	0	0	0	0	0	0	0	0	0
0													
OTU6271	0	0	0	1	0	0	0	1	0	0	2	2	0
0	0	0	0	0	0	2	1	76	0	41	0	0	7
2	5	0	0	88	0	26	0	0	0	1	0	0	0
0	0	71	0	0	0	0	0	5	161	2	3	5	1
10	0	0	1	10	17	1	42	30	15	528	43	6	0
33	0	0	0	0	0	0	0	0	1	0	0	0	0
0	1	0	0	0	0	0	0	10	0	0	0	0	1
3	0	0	1	0	0	0	0	0	0	0	0	0	0
0													
OTU848	0	0	0	1	10	0	0	0	0	0	1	0	0
0	0	0	0	0	0	0	0	0	0	0	0	0	0
0	0	0	0	0	0	0	0	0	0	0	0	0	0

0	0	0	0	0	0	0	0	0	0	0	0	0	0
0	0	0	0	0	0	0	0	0	0	0	0	0	0
0	0	0	0	0	0	0	0	0	0	0	0	0	0
0	0	0	0	0	0	0	0	1	0	0	0	0	1
0	0	0	0	0	0	0	0	0	0	1	0	0	0
0													
OTU6587	0	0	0	2	0	0	0	0	0	0	0	0	0
0	0	1	0	0	0	0	0	0	0	0	0	0	0
0	0	0	0	0	0	0	0	1	0	0	0	0	0
0	0	0	0	0	0	0	1	0	0	0	0	0	1
0	0	0	0	0	0	0	0	0	0	0	0	0	0
0	0	0	0	0	1	0	0	0	0	0	0	0	0
0	0	0	0	0	1	0	0	0	0	0	0	0	0
1	0	0	0	0	0	0	0	0	0	0	0	0	1
0													
OTU10932	0	0	0	1	0	0	0	0	0	0	0	0	0
0	0	0	0	0	0	0	0	0	0	0	0	0	0
0	0	0	0	0	0	0	0	0	0	0	0	0	0
0	0	0	0	0	0	0	0	0	0	0	0	0	0
0	0	0	0	0	0	0	0	0	0	0	0	0	0
0	0	0	0	0	0	0	0	0	0	0	0	0	0
0	0	0	0	0	0	0	0	0	0	0	0	0	0
0	0	0	0	0	0	0	0	0	0	0	0	0	0
0													
OTU5993	0	0	0	1	2	0	0	0	15	0	0	0	0
0	0	4	1	0	0	0	0	0	2	0	0	0	2
0	0	0	7	1	0	8	0	0	0	0	0	0	0
0	0	93	0	8	36	0	12	21	26	12	1	11	4
70	9	112	3	45	27	33	42	23	43	4	2	6	61
48	2	1	3	0	0	1	0	0	0	3	1	1	6
0	13	5	4	1	0	0	3	32	0	0	0	5	9
0	9	1	0	38	20	41	41	36	28	0	0	1	0
0													

OTU10931	0	0	0	1	0	0	0	0	0	0	0	0	0
0	0	0	0	0	0	0	0	0	0	0	0	0	0
0	0	0	0	0	0	0	0	0	0	0	0	0	0
0	0	0	0	0	0	0	0	0	0	0	0	0	0
0	0	0	0	0	0	0	0	0	0	0	0	0	0
0	0	0	0	0	0	0	0	0	0	0	0	0	0
0	0	0	0	0	0	0	0	0	0	0	0	0	0
0	0	0	0	0	0	0	0	0	0	0	0	0	0
0													
OTU139	0	0	0	1	0	0	0	2	0	0	0	2	0
0	0	0	0	0	0	0	0	0	0	0	0	0	0
0	0	2	0	0	0	0	0	0	0	0	0	0	0
0	0	0	0	0	0	0	3	0	0	0	0	0	1
0	0	0	0	0	0	1	0	1	0	0	0	0	0
1	34	0	0	0	0	0	0	0	0	4	0	0	0
0	0	0	0	1	0	0	0	0	0	1	7	10	0
0	92	146	68	25	11	63	14	49	33	0	0	0	274
53													
OTU5577	0	0	0	1	0	0	0	0	0	0	0	0	0
0	0	0	0	0	0	0	0	0	0	0	0	0	0
0	0	0	0	0	0	0	0	0	0	0	0	0	0
0	1	0	0	0	0	0	0	0	0	0	0	0	0
0	0	0	0	0	0	0	0	0	0	0	0	0	0
0	0	0	0	0	0	0	0	0	0	0	1	0	0
0	0	0	0	0	0	0	0	0	0	0	0	0	0
0	0	0	0	0	0	0	0	0	0	0	0	0	0
0													
OTU2363	0	0	0	1	1	0	0	0	0	0	0	0	0
0	1	0	0	0	0	0	0	0	0	0	0	0	0
0	0	1	0	2	0	0	0	0	0	0	0	0	1
0	0	5	0	0	0	0	0	0	0	0	0	1	0
0	1	2	0	1	0	0	0	0	1	0	0	0	3
0	0	0	0	1	1	0	0	0	0	0	0	0	0

0	0	0	0	0	0	0	0	0	0	0	0	0	1
0	0	0	0	0	0	0	0	0	0	0	9	0	0
0													
OTU7489	0	0	0	1	0	0	0	0	0	0	1	0	0
0	0	0	0	0	1	1	0	0	0	0	1	0	0
0	0	0	1	0	1	0	1	0	0	0	1	0	0
0	0	0	0	0	0	0	0	0	0	1	0	0	0
0	0	0	0	0	0	0	1	0	0	0	0	0	0
0	0	0	0	0	0	0	0	0	0	0	1	0	0
0	0	0	0	0	0	0	0	1	0	0	0	0	0
1	0	0	0	0	0	0	0	0	0	0	1	0	1
0													
OTU1306	0	0	0	1	0	0	1	0	0	0	0	0	1
0	0	0	0	0	1	0	0	1	0	0	0	2	3
0	0	0	0	0	0	0	0	0	0	0	0	1	0
1	0	0	0	0	0	0	0	0	0	0	0	0	0
0	0	0	0	0	0	0	0	0	0	0	0	0	0
0	0	0	0	0	0	0	0	0	0	0	0	0	0
0	0	0	0	0	0	0	0	0	0	1	0	0	0
0	0	0	0	1	0	0	0	0	0	1	0	0	0
0													
OTU550	0	0	0	1	0	0	0	0	0	0	0	0	0
0	0	0	0	0	0	0	0	0	0	0	0	1	0
0	0	0	0	0	0	0	0	0	0	0	0	0	0
0	0	0	0	0	0	0	0	0	0	0	0	0	0
0	0	0	0	0	0	0	0	0	0	0	0	0	0
0	2	0	0	0	0	0	0	0	0	0	1	1	0
0	0	0	0	1	0	0	0	0	0	0	0	0	0
0	6	4	1	3	0	0	4	5	6	0	1	0	6
0													
OTU12056	0	0	0	1	0	0	0	0	0	0	0	0	0
0	0	0	0	0	0	0	0	0	0	0	0	0	0
0	0	0	0	0	0	0	0	0	0	0	0	0	0

0	0	0	0	0	0	0	0	0	0	0	0	0	0
0	0	0	0	0	0	0	0	0	0	0	0	0	0
0	0	0	0	0	0	0	0	0	0	0	0	0	0
0	0	0	0	0	0	0	0	0	0	0	0	0	0
0	0	0	0	0	0	0	0	0	0	0	0	0	0
0													
OTU9631	0	0	0	1	3	3	1	0	1	3	1	2	1
0	4	0	0	0	2	2	0	0	0	0	1	1	1
1	0	1	0	0	1	0	2	2	0	2	0	2	1
3	0	1	0	0	1	2	4	0	0	1	0	0	0
0	2	0	5	0	0	0	0	0	0	0	0	0	0
0	0	2	0	2	1	0	0	2	5	0	1	0	3
2	2	0	0	0	5	0	4	0	0	1	1	1	0
1	0	0	0	0	0	0	0	0	3	2	0	0	1
0													
OTU14664	0	0	0	1	0	0	0	0	0	0	0	0	0
0	0	0	0	0	0	0	0	0	0	0	0	0	0
0	0	0	0	0	0	0	0	0	0	0	0	0	0
0	0	0	0	0	0	0	0	0	0	0	0	0	0
0	0	0	0	0	0	0	0	0	0	0	0	0	0
0	0	0	0	0	0	0	0	0	0	0	0	0	0
0	0	0	0	0	0	0	0	0	0	0	0	0	0
0	0	0	0	0	0	0	0	0	0	0	0	0	0
0													
OTU4794	0	0	0	1	3	0	2	1	0	1	0	0	0
1	3	0	0	2	4	1	1	1	2	1	0	1	0
1	0	2	1	1	0	0	1	0	1	0	1	3	0
0	2	0	2	4	1	0	0	1	1	1	1	0	0
0	0	0	3	0	2	0	1	0	1	0	0	2	0
4	1	1	2	5	1	0	3	1	2	2	4	1	3
1	1	0	2	2	3	0	0	0	0	3	2	1	0
0	1	0	1	0	0	0	2	0	0	0	0	1	2
3													

OTU6420 0 0 0 1 0 1 0 0 0 1 0 0 0
0 1 0 0 0 1 1 0 0 0 0 0 0 2
0 0 1 1 0 1 0 0 0 0 0 0 1 0
0 0 1 0 0 0 0 0 0 1 0 0 0 0
0 1 0 1 0 1 0 0 0 0 2 1 0 0
1 0 0 0 0 0 0 0 0 0 0 1 0 0
0 0 0 0 0 0 0 0 0 0 0 0 0 0
0 1 0 0 0 0 0 0 0 0 0 0 0 1
1
OTU6064 0 0 0 1 0 0 0 0 0 1 0 0 0
0 0 0 0 0 0 0 2 0 0 0 0 0 0
0 0 0 0 1 0 0 0 0 0 0 0 0 0
0 0 0 2 0 0 0 0 0 0 0 0 0 0
0 0 0 0 0 0 0 0 0 0 0 0 0 1
0 0 2 0 1 3 2 0 1 0 0 0 0 0
0 0 0 0 0 0 0 0 0 0 0 0 0 0
0 0 0 0 0 0 0 1 0 0 0 0 0 0
0
OTU10145 0 0 0 1 0 0 0 0 0 0 0 0 0
0 0 0 0 0 0 0 0 0 0 0 0 0 0
0 0 0 0 0 0 0 0 0 1 0 0 0 0
0 0 0 0 0 0 0 0 0 0 0 0 0 0
0 0 0 0 0 0 0 0 0 0 0 0 1 0
0 0 0 0 0 0 0 0 0 0 0 0 0 0
0 0 0 0 0 0 0 0 0 0 0 0 0 0
0 0 0 0 0 0 0 0 0 0 1 0 0 0
0
OTU16187 0 0 0 1 0 1 0 0 0 0 0 0 0
0 0 0 0 0 0 0 1 0 0 0 0 0 1
0 0 0 0 0 0 0 0 0 0 0 0 0 0
0 0 0 0 0 0 0 0 0 0 1 0 0 0
0 0 0 1 0 0 0 0 0 0 0 0 0 0
0 0 0 0 0 0 1 0 0 0 1 0 0 0

0	0	0	0	0	0	0	0	0	0	0	0	0	0
0	0	0	0	0	0	0	0	0	0	0	1	0	0
0													
OTU14665	0	0	0	1	0	0	0	0	0	0	0	0	0
0	0	0	0	0	0	0	0	0	0	0	0	0	0
0	0	0	0	0	0	0	0	0	0	0	0	0	0
0	0	0	0	0	0	0	0	0	0	0	0	0	0
0	0	0	0	0	0	0	0	0	0	0	0	0	0
0	0	0	0	0	0	0	0	0	0	0	0	0	0
0	0	0	0	0	0	0	0	0	0	0	0	0	0
0	0	0	0	0	0	0	0	0	0	0	0	0	0
0													
OTU401	0	0	0	1	2	0	0	0	17	7	0	9	0
0	0	4	0	0	2	24	3	0	0	0	0	0	0
7	0	0	1	0	0	0	2	2	1	2	0	2	0
0	4	0	0	1	0	0	0	0	1	0	2	0	0
0	0	0	1	0	0	1	0	0	1	0	0	1	0
0	2	0	1	2	0	0	0	0	1	0	0	0	0
0	1	0	4	1	0	2	7	0	0	1	8	1	1
2	4	7	8	2	3	6	10	0	6	4	0	2	7
0													
OTU1193	0	0	0	1	0	0	0	1	0	0	0	0	0
0	0	0	0	0	0	0	0	0	0	0	0	0	0
0	0	0	0	0	0	0	0	0	0	0	0	0	0
0	0	0	0	0	0	0	0	0	0	0	0	0	0
0	0	0	0	0	0	0	0	0	0	0	0	0	0
0	0	0	0	2	0	0	0	0	0	0	0	0	1
0	1	0	0	0	0	0	0	0	0	0	0	0	0
0	0	0	0	0	0	0	0	0	0	0	0	0	0
0													
OTU511	0	0	0	1	0	0	0	1	3	5	0	2	1
0	0	0	0	0	2	1	0	0	0	0	0	0	1
1	0	1	0	0	1	0	0	0	0	0	0	0	0

4	0	0	2	2	15	0	1	0	0	0	0	1	0
0	0	0	82	0	0	2	0	0	1	0	0	12	0
0	50	0	0	0	2	0	7	1	0	1	5	0	1
0	0	0	2	1	1	0	0	0	1	1	2	3	2
0	15	41	13	8	10	5	5	16	162	0	0	0	7
3													
OTU8315	0	0	0	1	0	3	1	0	0	0	0	0	0
0	0	0	0	0	0	0	0	0	0	0	0	0	0
0	0	0	0	0	0	0	0	2	0	0	0	0	0
0	0	0	0	0	0	0	2	0	0	0	0	0	0
0	0	0	1	0	0	0	0	0	0	0	0	1	0
0	0	0	0	0	0	0	0	0	1	0	0	0	0
0	0	0	0	0	0	0	0	0	0	0	0	0	0
0	0	0	0	0	0	0	0	0	0	0	0	0	0
0													
OTU8305	0	0	0	0	3	0	0	0	0	0	0	0	0
0	2	0	0	0	0	0	0	0	0	0	0	0	0
0	0	0	0	0	0	0	0	0	0	0	0	0	0
0	0	0	0	0	0	0	0	0	0	0	0	0	0
0	0	0	0	0	0	0	0	0	0	0	0	0	3
0	0	0	0	0	0	0	0	0	0	0	0	0	0
0	0	0	0	0	0	0	0	0	0	0	0	0	0
0	0	0	0	0	0	0	0	0	0	0	3	0	0
0													
OTU6218	0	0	0	0	1	0	0	0	0	1	0	0	0
1	1	0	0	0	0	1	0	0	0	1	0	0	1
0	0	1	0	0	0	0	0	0	0	1	0	0	0
1	0	0	0	0	0	0	0	1	0	0	0	0	1
0	0	0	0	0	0	0	0	0	1	0	0	0	0
2	0	0	0	0	0	1	1	0	0	0	1	0	0
1	0	0	0	0	0	0	0	0	1	1	1	0	1
0	0	0	0	0	1	0	0	0	0	0	0	0	0
0													

OTU8314 0 0 0 0 1 0 0 0 0 0 0 0 0
0 0 0 0 0 0 0 0 0 0 0 0 0 0
0 0 0 0 0 0 0 0 0 0 0 0 0 0
0 0 0 0 0 0 0 0 0 0 0 0 0 0
0 0 0 0 0 0 0 0 0 0 0 0 0 0
0 0 0 0 0 0 0 0 0 0 0 0 0 0
0 0 0 0 0 0 0 0 0 0 0 0 0 0
0 0 0 0 0 0 0 0 0 0 0 0 0 0
0
OTU294 0 0 0 0 17 0 0 0 1 0 0 0 0
0 1 0 0 0 0 0 0 0 0 0 0 0 0
0 0 0 0 0 0 0 0 0 0 0 0 0 0
0 0 0 0 0 0 0 0 0 0 0 0 0 0
0 0 0 14 0 0 0 0 0 0 0 0 6 0
0 5 0 0 0 0 0 0 0 0 1 2 0 0
0 0 0 0 0 0 0 0 0 0 0 5 9 0
0 13 10 20 3 3 0 0 7 36 0 1 0 7
3
OTU13010 0 0 0 0 1 0 0 2 0 0 0 1 0
0 2 0 0 2 0 0 0 0 0 0 0 0 0
0 0 2 0 0 0 0 0 0 1 0 0 0 0
0 0 1 0 1 0 0 0 0 0 0 1 0 0
1 0 0 0 0 0 0 0 1 0 0 0 0 0
1 0 1 0 0 0 0 1 0 0 1 0 0 0
0 0 0 0 0 1 1 0 0 0 0 0 1 0
0 0 0 1 0 0 0 0 0 0 0 0 0 0
0
OTU7599 0 0 0 0 2 0 0 1 0 0 0 1 0
0 0 0 0 0 0 0 3 0 0 0 0 0 1
0 0 0 1 0 0 0 3 0 0 0 0 0 0
0 1 0 3 0 0 0 0 0 0 0 0 0 0
0 0 0 1 1 0 0 0 0 0 0 0 0 0
0 0 0 0 0 0 2 0 1 0 0 0 6 0

0	2	2	0	7	0	0	0	0	1	2	0	0	0
9	0	0	0	0	0	0	0	0	0	0	0	0	0
0													
OTU10646	0	0	0	0	1	0	1	0	0	0	0	1	0
0	0	0	0	0	0	0	0	0	0	0	0	0	0
0	0	0	0	0	0	0	0	0	0	0	0	0	0
0	0	0	0	0	0	0	0	0	0	0	0	0	0
0	0	0	0	0	0	0	0	0	0	0	0	0	0
0	0	0	0	0	0	0	0	0	0	0	0	0	0
0	0	0	0	0	0	0	0	0	1	1	0	0	0
0	0	0	0	0	0	0	0	0	0	5	0	0	0
0													
OTU1766	0	0	0	0	1	0	0	0	0	0	0	0	0
2	0	0	0	0	0	0	0	0	0	0	0	0	0
0	0	0	0	0	0	0	6	2	0	0	1	0	3
0	1	0	1	0	1	0	0	0	0	0	0	0	0
0	0	0	1	0	0	0	0	0	0	0	3	0	0
0	0	0	5	0	0	7	0	1	0	1	0	2	0
0	0	7	0	37	0	1	0	2	0	1	0	0	0
0	0	0	0	0	0	0	0	0	0	0	2	0	0
0													
OTU12628	0	0	0	0	1	0	1	0	1	1	0	0	0
0	0	0	0	1	1	0	0	0	0	0	0	0	0
0	0	3	1	0	1	0	0	0	1	0	1	2	1
0	0	2	0	0	0	0	0	0	1	1	0	0	1
0	0	1	0	0	0	1	1	0	1	1	0	0	0
0	0	0	0	0	0	0	0	0	0	1	1	0	0
2	0	0	1	0	0	0	0	0	0	0	0	0	0
0	0	0	1	0	0	0	1	0	0	0	0	0	0
0													
OTU2646	0	0	0	0	1	0	0	0	0	0	0	0	0
0	0	0	0	0	0	0	0	0	0	0	0	0	0
0	0	0	0	0	0	0	1	0	0	0	0	0	0

0 0 0 0 0 0 0 0 0 0 0 0 0 0
0 0 0 0 0 0 0 0 0 0 0 0 0 0
0 0 0 0 1 0 0 0 0 0 0 0 0 0
0 0 0 0 1 0 0 1 0 0 0 0 0 0
0 0 0 0 0 0 0 0 0 0 0 0 0 0
0
OTU1476 0 0 0 0 1 0 0 1 0 0 0 3 0
0 1 2 0 0 0 0 0 0 0 0 1 0 0
0 0 0 0 0 0 0 0 1 4 0 0 0 0
0 0 0 1 0 0 2 0 0 0 0 0 0 0
0 0 0 0 0 0 0 0 0 0 0 0 0 0
0 0 0 2 0 0 0 0 0 0 3 0 1 0
0 0 0 0 0 0 0 0 0 3 1 0 0 0
0 0 0 0 0 0 0 0 0 0 1 0 0 0
0
OTU10916 0 0 0 0 1 0 0 0 0 0 0 0 0
0 0 0 0 0 0 0 0 0 0 0 0 0 0
0 0 0 0 0 0 0 0 0 0 0 0 0 0
0 0 0 0 0 0 0 0 0 0 0 0 0 0
0 0 0 0 0 0 0 0 0 0 0 0 0 0
0 0 0 0 0 0 0 0 0 0 0 0 0 0
0 0 0 0 0 0 0 0 0 0 0 0 0 0
0 0 0 0 0 0 0 0 0 0 0 0 0 0
0
OTU3161 0 0 0 0 2 0 1 0 0 0 0 1 0
0 0 0 0 0 1 0 1 0 0 1 0 0 0
0 0 0 0 0 0 0 0 0 0 1 1 3 1
0 0 0 1 0 0 0 0 2 0 0 0 0 0
0 0 0 1 1 1 0 0 1 0 1 1 1 0
1 0 0 0 1 0 0 1 0 0 2 1 0 0
0 0 0 0 0 0 0 0 0 1 0 0 0 0
0 0 0 1 1 0 0 0 0 1 1 0 0 0
2

OTU14446 0 0 0 0 1 1 0 0 0 0 0 0 0
0 0 0 0 0 0 0 0 0 0 0 0 0 0
0 0 0 0 0 0 0 0 0 0 0 0 0 0
0 0 0 0 0 0 0 1 0 0 0 0 0 0
0 0 0 0 0 0 0 0 0 0 0 0 0 0
0 0 0 0 0 0 0 0 0 0 0 0 0 0
0 0 0 0 0 0 0 0 0 0 0 0 0 0
0 0 0 0 0 0 0 0 0 0 0 0 0 0
0
OTU16710 0 0 0 0 1 2 0 0 0 0 0 0 0
0 0 0 0 0 0 0 0 0 0 0 0 0 0
0 0 0 0 0 0 0 0 1 0 0 0 0 0
0 0 0 0 0 1 0 2 0 0 0 0 0 0
0 0 0 1 0 0 0 0 0 0 0 0 0 0
0 0 0 0 0 0 0 0 0 0 0 0 0 0
0 0 0 0 0 0 0 0 0 0 0 0 0 0
0 0 0 0 0 0 0 0 0 0 0 0 0 0
0
OTU2726 0 0 0 0 1 1 0 0 0 0 0 0 0
0 0 0 0 0 0 0 0 0 0 0 0 0 0
1 0 0 0 0 0 0 0 0 0 0 0 1 0
0 0 0 0 0 1 0 1 0 0 0 0 0 0
0 0 0 0 0 0 0 0 0 0 0 0 0 0
0 0 1 0 0 0 0 0 0 0 0 0 0 0
0 0 1 1 0 1 0 1 0 0 0 0 0 0
0 1 0 0 0 0 0 0 0 0 0 0 0 0
0
OTU10924 0 0 0 0 1 0 0 0 0 0 0 0 0
0 0 0 0 0 0 0 0 0 0 0 0 0 0
0 0 0 0 0 0 0 0 0 0 0 0 0 0
0 0 0 0 0 0 0 0 0 0 0 0 0 0
0 0 0 0 0 0 0 0 0 0 0 0 0 0
0 0 0 0 0 0 0 0 0 0 0 0 0 0

0 0 0 0 0 0 0 0 0 0 0 0 0 0
0 0 0 0 0 0 0 0 0 0 0 0 0 0
0
OTU2845 0 0 0 0 1 0 0 0 0 0 1 0 0
1 0 0 0 0 0 0 0 0 0 0 0 0 0
0 0 0 0 0 0 0 0 0 4 0 0 0 0
0 0 0 0 0 0 0 0 0 0 0 0 0 0
0 0 0 0 0 0 0 0 0 0 0 0 0 0
0 0 0 0 0 1 0 0 0 0 0 0 0 0
0 0 0 0 0 0 0 0 0 0 1 0 0 0
0 0 0 0 0 0 0 0 0 0 0 0 0 0
0
OTU635 0 0 0 0 1 0 0 0 0 0 2 0 0
0 0 0 0 0 0 0 0 0 0 0 0 0 12
0 0 0 0 0 0 0 0 0 2 0 0 0 0
0 0 0 0 0 0 0 0 0 0 0 0 0 0
0 0 0 0 0 0 0 0 0 0 0 0 0 0
0 0 0 0 0 0 0 0 0 0 0 0 0 0
0 0 0 0 0 0 0 0 0 0 0 0 0 0
0 0 0 0 0 0 0 0 0 0 0 0 0 0
0
OTU10923 0 0 0 0 1 0 0 0 0 0 0 0 0
0 0 0 0 0 0 0 0 0 0 0 0 0 0
0 0 0 0 0 0 0 0 0 0 0 0 0 0
0 0 0 0 0 0 0 0 0 0 0 0 0 0
0 0 0 0 0 0 0 0 0 0 0 0 0 0
0 0 0 0 0 0 0 0 0 0 0 0 0 0
0 0 0 0 0 0 0 0 0 0 0 0 0 0
0 0 0 0 0 0 0 0 0 0 0 0 0 0
0
OTU14682 0 0 0 0 1 0 0 0 0 0 0 0 0
0 0 0 0 0 0 0 0 0 0 0 0 0 0
0 0 0 0 0 0 0 0 0 0 0 0 0 0

0	0	0	0	0	0	0	0	0	0	0	0	0	0
0	0	0	0	0	0	0	0	0	0	0	0	0	0
0	0	0	0	0	0	0	0	0	0	0	0	0	0
0	0	0	0	0	0	0	0	0	0	0	0	0	0
0	0	0	0	0	0	0	0	0	0	0	0	0	0
0													
OTU11699	0	0	0	0	1	2	0	0	0	4	1	0	0
3	2	0	0	4	0	0	0	0	2	0	1	0	0
0	0	1	0	0	1	0	1	0	0	0	1	0	0
3	1	0	1	1	0	0	0	1	1	0	0	0	0
0	0	1	0	0	1	0	0	0	1	1	2	0	0
1	1	0	1	2	0	0	1	1	0	1	0	1	0
0	1	0	1	0	1	1	0	0	0	0	1	1	0
0	0	0	1	0	1	0	1	0	1	1	0	0	0
1													
OTU14681	0	0	0	0	1	0	0	0	0	0	0	0	0
0	0	0	0	1	0	0	0	0	0	0	0	0	0
0	0	0	0	0	0	0	0	0	1	0	0	0	0
0	0	0	0	0	0	0	0	0	0	0	0	0	0
0	0	0	0	0	0	0	0	0	0	0	0	0	0
0	0	0	0	0	0	0	0	0	0	0	0	0	0
0	0	0	0	0	0	0	0	0	0	0	0	0	0
0	0	0	0	0	0	0	0	0	0	0	0	0	0
0													
OTU5535	0	0	0	0	2	0	0	0	0	0	0	1	0
0	0	0	0	0	0	0	0	0	0	0	0	0	0
0	0	0	0	0	0	0	0	0	1	0	1	0	0
0	0	0	0	0	0	0	2	0	0	0	0	0	0
0	2	0	0	0	0	0	0	0	0	0	0	0	0
0	0	0	0	1	0	0	0	0	0	0	0	0	0
0	0	0	0	0	0	0	0	0	0	0	0	1	0
0	0	1	0	0	0	0	0	0	0	1	0	0	0
0													

OTU12059	0	0	0	0	1	0	0	0	0	0	0	0	0
0	0	0	0	0	0	0	0	0	0	0	0	0	0
0	0	0	0	0	1	0	1	0	1	0	0	0	1
0	0	0	0	0	0	1	0	0	0	0	0	0	0
0	0	0	0	0	0	0	0	0	0	0	0	0	0
0	0	0	0	0	0	0	0	0	0	0	0	0	0
0	0	0	0	0	0	0	0	0	0	0	0	0	0
0	0	0	0	0	0	0	0	0	0	0	0	1	0
0													
OTU12058	0	0	0	0	1	0	0	0	0	0	0	0	0
0	0	0	0	0	0	0	0	0	0	0	0	0	0
0	0	0	0	0	0	0	0	0	0	0	0	0	0
0	0	0	0	0	0	0	0	0	0	0	0	0	0
0	0	0	0	0	0	0	0	0	0	0	0	0	0
0	0	0	0	0	0	0	0	0	0	0	0	0	0
0	0	0	0	0	0	0	0	0	0	0	0	0	0
0	0	0	0	0	0	0	0	0	0	0	0	0	0
0													
OTU525	0	0	0	0	1	2	3	3	0	1	0	0	0
0	1	0	0	1	1	0	0	0	0	0	0	0	0
0	0	1	0	0	0	0	0	0	0	0	0	0	0
1	0	0	0	1	0	0	1	0	0	0	0	0	0
0	2	0	2	0	0	0	0	0	0	0	0	1	0
1	0	8	0	0	0	0	0	0	1	0	0	0	2
0	0	0	0	2	2	0	1	0	0	0	0	0	0
0	1	0	0	0	0	0	0	0	0	0	0	0	0
0													
OTU6376	0	0	0	0	1	0	0	2	0	0	0	0	3
4	0	9	0	0	6	0	2	0	0	0	0	0	0
0	0	12	1	0	2	0	8	0	0	0	1	0	9
0	0	0	0	0	0	2	0	0	0	0	0	0	0
0	0	0	0	0	0	0	0	0	0	0	0	0	0
0	0	0	6	0	0	0	0	6	0	0	0	0	0

0	0	20	0	0	0	0	0	0	9	7	0	0	3
2	0	0	0	0	0	0	0	0	0	0	15	2	0
0													
OTU15634	0	0	0	0	1	0	1	0	0	1	0	0	0
0	0	1	0	0	0	0	0	0	0	0	1	0	0
0	1	0	1	0	0	0	0	0	0	0	0	0	0
0	0	0	0	0	0	0	0	0	0	0	0	0	0
0	0	0	0	0	0	0	0	0	0	0	0	0	0
1	0	1	0	0	0	0	0	0	0	0	0	0	0
0	0	0	0	1	1	0	0	0	0	0	0	0	1
0	0	0	0	0	0	0	0	0	0	0	0	0	0
0													
OTU850	0	0	0	0	1	0	0	0	0	0	0	0	0
0	0	0	0	0	0	0	0	0	0	1	0	0	0
0	0	0	0	0	0	1	0	1	0	1	0	1	0
1	0	0	0	1	4	0	1	0	0	0	0	0	0
0	0	0	0	0	1	0	0	0	0	0	0	1	1
0	6	0	0	0	0	0	0	0	0	0	0	0	0
0	3	0	1	0	0	0	0	0	0	0	2	2	0
0	3	3	4	1	2	1	4	0	5	0	0	0	9
0													
OTU8344	0	0	0	0	1	0	0	0	2	0	0	0	0
0	0	0	0	0	0	0	0	0	0	0	0	0	0
0	0	0	0	0	0	0	0	0	0	0	0	0	0
0	0	0	0	0	0	0	0	0	0	0	0	0	0
0	0	0	0	0	0	0	0	0	0	0	0	0	0
0	0	0	0	1	0	0	0	0	0	0	0	0	0
0	0	0	0	0	0	0	0	0	0	0	0	0	0
0	0	0	0	0	0	0	0	0	0	0	0	0	0
0													
OTU14680	0	0	0	0	1	0	0	0	0	1	0	0	0
0	0	0	0	0	0	0	0	0	0	0	0	0	0
0	0	0	0	0	0	0	0	0	0	0	0	0	0

0 0 0 0 1 0 0 0 0 0 0 0 0 0
0 0 0 0 0 0 0 0 0 0 0 0 0 0
0 0 0 0 0 0 0 0 0 0 0 0 0 0
0 0 0 0 0 0 0 0 0 0 0 0 0 0
0 0 0 0 0 0 0 0 0 0 0 0 0 0
0
OTU6423 0 0 0 0 1 0 0 0 0 0 0 0 0
0 0 0 0 1 0 0 0 0 0 0 0 0 0
0 1 0 0 0 0 0 0 0 0 0 0 0 0
0 0 0 0 0 0 0 1 0 0 1 0 0 0
0 2 0 0 0 0 0 0 0 0 0 0 0 0
0 2 0 0 0 0 0 0 0 0 0 0 0 0
0 0 0 0 0 0 0 0 0 0 0 0 0 0
0 0 1 0 0 0 1 0 7 1 0 0 0 0
0
OTU1383 0 0 0 0 1 0 0 0 0 0 0 0 0
0 2 0 0 0 0 0 0 0 0 0 0 0 0
0 0 0 0 0 0 0 0 0 0 0 1 0 0
0 0 0 0 0 1 0 0 0 0 0 0 0 0
0 0 0 0 0 0 0 0 0 0 0 0 0 0
0 0 0 0 0 0 0 0 0 0 0 0 1 0
0 0 0 0 0 0 0 0 0 0 2 0 0 0
0 0 0 0 0 0 0 0 0 0 1 0 0 0
0
OTU10918 0 0 0 0 1 0 0 0 0 0 0 0 0
0 0 0 0 0 0 0 0 0 0 0 0 0 0
0 0 0 0 0 0 0 0 0 0 0 0 0 0
0 0 0 0 0 0 0 0 0 0 0 0 0 0
0 0 0 0 0 0 0 0 0 0 0 0 0 0
0 0 0 0 0 0 0 0 0 0 0 0 0 0
0 0 0 0 0 0 0 0 0 0 0 0 0 0
0 0 0 0 0 0 0 0 0 0 0 0 0 0
0

OTU10922 0 0 0 0 1 0 0 0 0 2 0 0 0
0 0 0 0 0 0 0 0 0 0 0 0 0 0
0 0 0 0 0 0 0 0 0 0 0 0 0 0
0 0 0 0 0 0 0 0 0 0 0 0 0 0
0 0 0 0 1 1 0 0 0 0 0 0 0 0
0 0 0 0 0 0 0 0 0 0 0 0 0 0
0 0 0 0 0 0 0 1 0 0 0 0 0 0
0 0 0 0 0 0 0 0 0 1 0 0 0 0
0
OTU10919 0 0 0 0 1 0 0 0 1 0 0 0 0
0 0 0 0 0 0 0 0 0 0 0 0 0 0
0 0 0 0 0 0 0 0 0 0 0 0 0 0
0 0 0 0 0 0 0 0 0 0 0 0 0 0
0 0 0 0 0 0 0 0 0 0 0 0 0 0
0 0 0 0 0 0 0 0 0 0 0 0 0 0
0 0 0 0 0 0 0 0 0 0 0 0 0 0
0 0 0 0 0 0 0 0 0 0 0 0 0 0
0
OTU10920 0 0 0 0 1 0 0 0 0 0 0 0 0
0 0 0 0 0 0 0 0 0 0 0 0 0 0
0 0 0 0 0 0 0 0 0 0 0 0 0 0
0 0 0 0 0 0 0 0 0 0 0 0 0 0
0 0 0 0 0 0 0 0 0 0 0 0 0 0
0 0 0 0 0 0 0 0 0 0 0 0 0 0
0 0 0 0 0 0 0 0 0 0 0 0 0 0
0 0 0 0 0 0 0 0 0 0 0 0 0 0
0
OTU3334 0 0 0 0 2 0 0 0 0 0 0 0 0
0 0 0 0 0 0 0 0 0 0 0 0 0 0
0 0 0 0 0 0 0 0 0 0 0 0 0 0
0 0 0 0 0 0 0 0 0 0 0 0 0 0
0 0 0 0 0 0 0 0 0 0 0 0 0 0
0 0 0 0 0 0 0 0 0 0 0 0 0 0

0 0 0 0 0 1 0 0 0 0 0 0 0 0
0 0 0 0 0 0 0 0 0 0 0 0 0 0
0
OTU12057 0 0 0 0 1 0 0 0 0 0 0 0 0
0 0 0 0 0 0 0 0 0 0 0 0 0 0
0 0 0 0 0 0 0 0 0 0 0 0 0 0
0 0 0 0 0 0 0 0 0 0 0 0 0 0
0 0 0 0 0 0 0 0 0 0 0 0 0 0
0 0 0 0 0 0 0 0 0 0 0 0 0 0
0 0 0 0 0 0 0 0 0 0 0 0 0 0
0 0 0 0 0 0 0 0 0 0 0 0 0 0
0
OTU10406 0 0 0 0 1 0 0 0 0 0 0 0 0
0 0 0 0 0 0 0 0 0 0 0 0 0 0
0 0 0 0 0 0 0 0 0 0 0 0 0 0
0 0 0 0 0 0 0 0 0 0 0 0 0 0
0 0 0 0 0 0 0 0 0 0 0 0 0 0
0 0 0 0 1 0 1 0 0 0 0 0 0 0
0 0 0 0 0 0 0 0 0 0 0 0 0 0
0 0 0 0 0 0 0 0 0 0 0 0 0 0
0
OTU10921 0 0 0 0 1 0 0 0 0 0 0 0 0
0 0 0 0 0 0 0 0 0 0 0 0 0 0
0 0 0 0 0 0 0 0 0 0 0 0 0 0
0 0 0 0 0 0 0 0 0 0 0 0 0 0
0 0 0 0 0 0 0 0 0 0 0 0 0 0
0 0 0 0 0 0 0 0 0 0 0 0 0 0
0 0 0 0 0 0 0 0 0 0 0 0 0 0
0 0 0 0 0 0 0 0 0 0 0 0 0 0
0
OTU16196 0 0 0 0 1 0 0 0 0 0 0 0 0
0 0 0 0 0 0 0 0 0 0 0 0 0 0
0 0 0 0 0 0 0 0 0 0 0 0 0 0

0	0	0	0	0	0	0	0	0	0	1	0	0	0
0	0	0	0	0	0	0	0	0	0	0	0	0	0
0	0	0	0	0	0	0	0	0	0	0	0	0	0
0	0	0	0	0	0	0	0	0	0	0	0	0	0
0	0	0	0	0	0	0	0	0	0	0	0	0	0
0													
OTU8341	0	0	0	0	1	0	0	0	0	0	0	0	0
0	0	0	0	0	0	0	0	0	0	0	0	0	0
0	0	0	0	0	0	0	0	0	0	0	0	0	0
0	0	0	0	0	0	0	0	0	0	0	0	0	0
0	0	0	0	0	0	0	0	0	0	0	0	0	0
0	0	0	0	0	0	0	0	0	0	0	0	0	0
0	0	0	0	0	0	0	0	0	0	0	0	0	0
0	0	0	0	0	0	0	0	0	0	0	0	0	0
0													
OTU8343	0	0	0	0	1	0	0	0	0	0	0	0	0
0	0	0	0	0	0	0	0	0	0	0	0	0	0
0	0	0	0	0	0	0	0	0	0	0	0	0	0
0	0	0	0	0	0	0	0	0	0	0	0	0	0
0	0	0	0	0	0	0	0	0	0	0	0	0	0
0	0	0	0	0	0	0	0	0	0	0	0	0	0
0	0	0	0	0	0	0	0	0	0	0	0	0	0
0	0	0	0	0	0	0	0	0	0	0	0	0	0
0													
OTU1055	0	0	0	0	1	0	0	0	0	0	0	0	1
0	0	0	1	7	0	0	0	3	0	3	1	0	0
0	0	0	0	3	1	1	0	1	0	0	0	0	0
0	0	0	1	0	0	0	0	1	0	0	1	0	2
1	0	0	0	0	0	0	0	0	1	0	0	0	1
0	0	0	0	0	0	0	0	0	0	0	0	0	0
0	0	0	0	0	1	1	0	0	0	0	0	0	0
0	0	0	0	0	0	0	0	0	0	0	1	0	0
0													

OTU4707 0 0 0 0 1 0 0 0 0 0 0 1 0
0 0 0 0 0 0 1 0 0 0 0 0 0 0
0 0 0 0 0 2 0 0 1 1 1 0 0 0
0 0 0 0 0 0 1 1 0 0 1 0 0 4
0 0 1 0 0 0 0 0 0 0 0 0 0 0
0 0 0 0 0 0 0 0 0 0 0 0 0 0
0 0 0 0 1 0 0 0 0 0 0 0 0 0
0 0 0 0 0 0 0 0 0 0 1 1 0 1
0
OTU8342 0 0 0 0 2 0 0 0 0 0 0 0 0
0 0 0 0 0 0 0 0 0 0 0 0 0 0
0 0 0 1 0 0 0 0 0 0 0 0 0 0
0 0 0 0 0 0 0 0 0 0 0 0 0 0
0 0 0 0 0 0 0 0 0 0 0 0 0 0
0 0 0 0 0 0 0 0 0 0 0 0 0 0
0 0 0 0 0 0 0 0 0 0 0 0 0 0
0 0 0 0 0 0 0 0 0 0 0 0 0 0
0
OTU5365 0 0 0 0 1 0 0 0 0 0 0 0 0
0 0 0 3 0 0 0 0 2 0 0 0 2 0
2 1 0 0 0 0 4 1 0 0 4 0 1 0
0 0 0 0 0 2 0 0 0 1 0 0 0 0
1 0 2 0 1 0 0 0 0 1 0 0 0 2
0 0 0 0 0 0 0 0 0 0 0 0 0 0
0 0 0 0 0 0 0 0 0 0 1 0 0 0
1 0 0 1 0 0 0 0 0 0 0 0 0 1
0
OTU1919 0 0 0 0 1 0 0 0 2 0 0 0 0
0 0 0 0 0 0 0 1 1 0 0 0 0 0
0 0 0 0 0 0 0 0 0 1 0 0 0 0
0 0 0 0 0 2 1 1 0 0 0 1 0 0
0 0 0 0 0 0 0 0 1 0 0 0 2 0
0 0 0 0 0 0 0 0 0 0 0 0 0 0

0	0	0	0	0	0	0	0	0	0	0	0	0	0
1	0	2	0	4	2	1	0	3	0	0	0	0	0
0													
OTU10275	0	0	0	0	1	0	0	1	0	0	0	0	0
0	0	0	0	0	0	0	0	0	0	0	0	0	0
0	0	0	0	0	0	0	0	0	0	0	0	0	0
0	0	1	0	0	0	0	0	0	0	0	0	0	0
0	0	0	0	0	0	0	0	1	0	0	0	0	0
1	0	0	0	0	0	0	0	0	0	0	0	0	0
0	0	0	0	0	0	0	0	0	0	0	0	0	0
0	0	0	0	0	0	0	0	0	0	0	0	0	0
0													
OTU10857	0	0	0	0	1	0	0	0	0	0	0	0	0
0	0	0	0	0	0	0	0	0	0	0	0	0	0
0	0	0	0	0	0	0	0	0	0	0	0	0	0
0	0	0	0	0	0	0	0	0	0	0	0	0	0
0	0	0	0	0	0	0	0	0	0	0	0	0	0
0	0	0	0	0	0	0	0	0	0	0	0	0	0
0	0	0	0	0	0	0	0	0	0	0	0	0	0
0	0	0	0	0	0	0	0	0	0	0	0	0	0
0													
OTU7568	0	0	0	0	2	1	0	0	0	0	0	0	1
0	1	2	0	0	1	0	0	1	0	0	0	0	0
0	1	1	0	1	1	0	1	2	0	0	0	2	0
0	0	0	0	0	0	0	0	0	2	0	2	0	0
0	1	0	0	1	0	0	0	0	2	1	1	2	0
4	0	1	0	0	0	0	2	0	0	0	2	0	0
1	0	0	0	1	0	0	0	0	0	2	0	0	0
1	0	0	0	1	0	0	1	1	1	0	0	0	0
2													
OTU14691	0	0	0	0	1	0	0	0	0	0	0	0	0
0	0	0	0	0	0	0	0	0	0	0	0	0	0
0	0	0	0	0	0	0	0	0	0	0	0	0	0

0 0 0 0 0 0 0 0 0 0 0 0 0 0

0 0 0 0 0 0 0 0 0 0 0 0 0 0

0 0 0 0 0 0 0 0 0 0 0 0 0 0

0 0 0 0 0 0 0 0 0 0 0 0 0 0

0 0 0 0 0 0 0 0 0 0 0 0 0 0

0

OTU8330 0 0 0 0 1 0 0 0 0 0 0 0 0

0 0 0 0 0 0 0 0 0 0 0 0 0 0

0 0 0 0 0 0 0 0 0 0 0 0 0 0

0 0 0 0 0 0 0 0 0 0 0 0 0 0

0 0 0 0 0 0 0 0 0 0 0 0 0 0

0 0 0 0 0 0 0 0 0 0 0 0 0 0

0 0 0 0 0 0 0 0 0 0 0 0 0 0

0 0 0 0 0 0 0 0 0 0 0 0 0 0

0

OTU12048 0 0 0 0 1 0 0 0 0 0 0 0 0

0 0 0 0 0 0 0 0 0 0 0 0 0 0

0 0 0 0 0 0 0 0 0 0 0 0 0 0

0 0 0 0 0 0 0 0 0 0 0 0 0 0

0 0 0 0 0 0 0 0 0 0 0 0 0 0

0 0 0 0 0 0 0 0 0 0 0 0 0 0

0 0 0 0 0 0 0 0 0 0 0 0 0 0

0 0 0 0 0 0 0 0 0 0 0 0 0 0

0

OTU1010 0 0 0 0 1 0 0 0 0 0 0 0 0

0 0 0 0 0 0 0 0 0 0 0 0 0 0

0 0 5 1 0 0 0 0 0 0 0 0 0 7

0 0 0 0 0 0 0 0 0 0 0 0 0 0

0 0 2 0 0 0 0 0 0 0 0 0 0 0

0 0 0 0 0 0 1 0 0 5 0 0 0 0

2 0 0 0 0 0 0 0 0 1 0 3 0 0

0 0 0 0 0 0 0 0 0 0 0 0 1 0

10

OTU14684 0 0 0 0 1 0 0 0 0 0 0 0 0
0 0 0 0 0 0 0 0 0 0 0 0 0 0
0 0 0 0 0 0 0 0 0 0 0 0 0 0
0 0 0 0 0 0 0 0 0 0 0 0 0 0
0 0 0 0 0 0 0 0 0 0 0 0 0 0
0 0 0 0 0 0 0 0 0 0 0 0 0 0
0 0 0 0 0 0 0 0 0 0 0 0 0 0
0 0 0 0 0 0 0 0 0 0 0 0 0 0
0
OTU12047 0 0 0 0 1 0 0 0 0 0 0 0 0
0 0 0 0 0 0 0 0 0 0 0 0 0 0
0 0 0 0 0 0 0 0 0 0 0 0 0 0
0 0 0 0 0 0 0 0 0 0 0 0 0 0
0 0 0 0 0 0 0 0 0 0 0 0 0 0
0 0 0 0 0 0 0 0 0 0 0 0 0 0
0 0 0 0 0 0 0 0 0 0 0 0 0 0
0 0 0 0 0 0 0 0 0 0 0 0 0 0
0
OTU10853 0 0 0 0 1 0 0 0 0 0 0 0 0
0 0 0 0 0 0 0 0 0 0 0 0 0 0
0 0 0 0 0 0 0 0 0 0 0 0 0 0
0 0 0 0 0 0 0 0 0 0 0 0 0 0
0 0 0 0 0 0 0 0 0 0 0 0 0 0
0 0 0 0 0 0 0 0 0 0 0 0 0 0
0 0 0 0 0 0 0 0 0 0 0 0 0 0
0 0 0 0 0 0 0 0 0 0 0 0 0 0
0
OTU8328 0 0 0 0 1 0 0 0 0 0 0 0 0
0 0 0 0 0 0 0 0 0 0 0 0 0 0
0 0 0 0 0 0 0 0 0 0 0 0 0 0
0 0 0 0 0 0 0 0 0 0 0 0 0 0
0 0 0 0 0 0 0 0 0 0 0 0 0 0
0 0 0 0 0 0 0 0 0 0 0 0 0 0

0	0	0	0	0	0	0	0	0	0	0	0	0	0
0	0	0	0	0	0	0	0	0	0	0	0	0	0
0													
OTU8329	0	0	0	0	1	0	0	0	0	0	0	0	0
0	0	0	0	0	0	0	0	0	0	0	0	0	0
0	0	0	0	0	0	0	0	0	0	0	0	0	0
0	0	0	0	0	0	0	0	0	0	0	0	0	0
0	0	0	0	0	0	0	0	0	0	0	0	0	0
0	0	0	0	0	0	0	0	0	0	0	0	0	0
0	0	0	0	0	0	0	0	0	0	0	0	0	0
0	0	0	0	0	0	0	0	0	0	0	0	0	0
0													
OTU2445	0	0	0	0	1	0	0	0	0	0	42	1	20
5	1	17	0	1	1	0	6	0	0	0	0	0	9
0	0	3	4	1	4	0	9	0	0	0	0	0	0
0	0	0	0	59	2	10	0	0	0	5	1	0	0
6	1	18	0	8	0	14	3	0	4	0	2	0	4
0	0	0	2	0	0	0	0	0	0	0	0	0	0
0	0	0	0	1	0	0	0	146	0	6	0	0	72
3	0	1	0	0	0	0	0	0	0	4	22	0	0
0													
OTU12520	0	0	0	0	1	0	0	1	1	3	2	0	0
0	5	1	0	2	1	0	1	0	2	2	2	0	0
0	0	1	1	0	1	0	1	0	0	0	3	0	1
1	1	2	0	0	0	0	0	1	0	0	0	1	0
0	1	0	1	0	1	0	0	0	0	0	0	1	0
2	2	0	0	3	0	0	1	0	0	1	3	0	1
1	1	0	0	1	1	0	3	1	1	0	0	2	0
1	2	1	0	0	0	0	1	0	1	0	0	0	0
3													
OTU1271	0	0	0	0	1	0	0	0	0	0	0	0	0
0	0	0	0	0	0	0	0	0	0	0	1	0	0
0	0	0	0	0	0	0	0	0	0	0	4	0	1

0	0	0	0	0	0	0	0	0	0	0	0	0	0
0	0	0	0	0	0	0	0	0	0	0	0	0	0
0	0	0	0	0	1	3	0	0	0	1	0	0	0
0	0	1	0	0	0	0	0	0	1	0	0	0	0
0	0	0	0	0	0	0	0	0	0	0	2	0	0
0													
OTU14690	0	0	0	0	1	0	0	0	0	0	0	0	0
0	0	0	0	0	0	0	0	0	0	0	0	0	0
0	0	0	0	0	0	0	0	0	0	0	0	0	0
0	0	0	0	0	0	0	0	0	0	0	0	0	0
0	0	0	0	0	0	0	0	0	0	0	0	0	0
0	0	0	0	0	0	0	0	0	0	0	0	0	0
0	0	0	0	0	0	0	0	0	0	0	0	0	0
0	0	0	0	0	0	0	0	0	0	0	0	0	0
0													
OTU14688	0	0	0	0	1	0	0	0	0	0	0	0	0
0	0	0	0	0	0	0	0	0	0	0	0	0	0
0	0	0	0	0	0	0	0	0	0	0	0	0	0
0	0	0	0	0	0	0	0	0	0	0	0	0	0
0	0	0	0	0	0	0	0	0	0	0	0	0	0
0	0	0	0	0	0	0	0	0	0	0	0	0	0
0	0	0	0	0	0	0	0	0	0	0	0	0	0
0	0	0	0	0	0	0	0	0	0	0	0	0	0
0													
OTU14689	0	0	0	0	1	0	0	0	0	0	0	0	0
0	0	0	0	0	0	0	0	0	0	0	0	0	0
0	0	0	0	0	0	0	0	0	0	0	0	0	0
0	0	0	0	0	0	0	0	0	0	0	0	0	0
0	0	0	0	0	0	0	0	0	0	0	0	0	0
0	0	0	0	0	0	0	0	0	0	0	0	0	0
0	0	0	0	0	0	0	0	0	0	0	0	0	0
0	0	0	0	0	0	0	0	0	0	0	0	0	0
0													

OTU12043 0 0 0 0 1 0 0 0 0 0 0 0 0
0 0 0 0 0 0 0 0 0 0 0 0 0 0
0 0 0 0 0 0 0 0 0 0 0 0 0 0
0 0 0 0 0 0 0 0 0 0 0 0 0 0
0 0 0 0 0 0 0 0 0 0 0 0 0 0
0 0 0 0 0 0 0 0 0 0 0 0 0 0
0 0 0 0 0 0 0 0 0 0 0 0 0 0
0 0 0 0 0 0 0 0 0 0 0 0 0 0
0
OTU5480 0 0 0 0 1 0 0 0 0 0 0 0 1
3 0 0 0 2 0 0 0 0 0 0 0 0 0
0 0 0 1 0 0 0 0 0 0 0 2 0 0
0 0 0 0 0 0 1 0 0 0 0 0 0 0
0 0 0 0 0 0 0 0 0 0 0 0 0 0
0 0 0 0 0 0 1 0 0 0 0 0 0 0
7 0 0 0 1 0 0 0 0 0 1 0 0 1
1 0 0 0 0 0 0 0 0 0 0 4 0 0
0
OTU1672 0 0 0 0 1 0 0 0 0 0 0 0 0
0 0 0 0 0 0 0 0 0 0 0 0 0 0
0 0 1 0 0 0 0 0 0 0 0 0 0 0
0 0 0 0 0 0 0 0 0 0 0 0 0 0
0 0 0 0 0 0 0 0 0 0 0 0 0 0
0 0 0 0 0 0 0 0 0 1 0 0 0 0
0 0 0 0 0 0 0 0 0 0 0 1 0 0
0 0 0 0 0 0 0 0 0 0 0 0 0 0
0
OTU14685 0 0 0 0 1 0 0 0 0 0 0 0 0
0 0 0 0 0 0 0 0 0 0 0 0 0 0
0 0 0 0 0 0 0 0 0 0 0 0 0 0
0 0 0 0 0 0 0 0 0 0 0 0 0 0
0 0 0 0 0 0 0 0 0 0 0 0 0 0
0 0 0 0 0 0 0 0 0 0 0 0 0 0

0 0 0 0 0 0 0 0 0 0 0 0 0 0
0 0 0 0 0 0 0 0 0 0 0 0 0 0
0
OTU5129 0 0 0 0 1 0 0 0 0 0 0 0 0
0 0 0 0 0 0 0 0 0 0 0 0 0 0
0 0 0 0 0 0 0 0 0 0 0 0 1 0
0 0 0 0 0 0 0 0 0 0 0 0 0 0
0 0 0 0 0 0 0 0 0 0 0 0 0 0
0 0 0 0 0 0 0 0 0 0 0 0 0 0
0 0 0 0 0 0 0 0 0 0 0 0 0 0
0 0 0 0 0 0 0 0 0 0 0 0 0 0
0
OTU14686 0 0 0 0 1 0 0 0 0 0 0 0 0
0 0 0 0 0 0 0 0 0 0 0 0 0 0
0 0 0 0 0 0 0 0 0 0 0 0 0 0
0 0 0 0 0 0 0 0 0 0 0 0 0 0
0 0 0 0 0 0 0 0 0 0 0 0 0 0
0 0 0 0 0 0 0 0 0 0 0 0 0 0
0 0 0 0 0 0 0 0 0 0 0 0 0 0
0 0 0 0 0 0 0 0 0 0 0 0 0 0
0
OTU12046 0 0 0 0 1 0 0 0 0 0 0 0 0
0 0 0 0 0 0 0 1 0 0 0 0 0 0
0 0 0 0 0 0 0 0 0 0 0 0 0 0
0 0 0 0 0 0 0 0 0 0 0 0 0 0
0 0 0 0 0 0 0 0 0 0 0 0 0 0
0 0 0 0 0 0 0 0 0 0 0 0 0 0
0 0 0 0 0 0 0 0 0 0 0 0 0 0
0 0 0 0 0 0 0 0 0 0 0 0 0 0
0
OTU14687 0 0 0 0 1 0 0 0 0 0 0 0 0
0 0 0 0 0 0 0 0 0 0 0 0 0 0
0 0 0 0 0 0 0 0 0 0 0 0 0 0

0	0	0	0	0	0	0	0	0	0	0	0	0	0
0	0	0	0	0	0	0	0	0	0	0	0	0	0
0	0	0	0	0	0	0	0	0	0	0	0	0	0
0	0	0	0	0	0	0	0	0	0	0	0	0	0
0	0	0	0	0	0	0	0	0	0	0	0	0	0
0													
OTU15532	0	0	0	0	1	0	0	0	0	0	0	0	0
0	0	1	0	0	0	0	0	0	0	0	1	0	0
0	0	0	1	0	0	0	0	0	0	0	0	0	0
0	0	0	0	0	0	0	0	0	0	0	0	0	0
0	0	0	0	0	0	0	0	0	0	0	0	0	0
0	0	0	0	0	0	0	0	0	0	0	0	0	0
0	0	0	0	0	0	0	0	0	0	0	0	0	0
0	0	0	0	0	0	0	0	0	0	0	0	0	0
0													
OTU10856	0	0	0	0	1	0	0	0	0	0	0	0	0
0	0	0	0	0	0	0	0	0	0	0	0	0	0
0	0	0	0	0	0	0	0	0	0	0	0	0	0
0	0	0	0	0	0	0	0	0	0	0	0	0	0
0	0	0	0	0	0	0	0	0	0	0	0	0	0
0	0	0	0	0	0	0	0	0	0	0	0	0	0
0	0	0	0	0	0	0	0	0	0	0	0	0	0
0	0	0	0	0	0	0	0	0	0	0	0	0	0
0													
OTU14674	0	0	0	0	1	0	0	0	0	0	0	0	0
0	0	0	0	0	0	0	0	0	0	0	0	0	0
0	0	0	0	0	0	0	0	0	0	0	0	0	0
0	0	0	0	0	0	0	0	0	0	0	0	0	0
0	0	0	0	0	0	0	0	0	0	0	0	0	0
0	0	0	0	0	0	0	0	0	0	0	0	0	0
0	0	0	0	0	0	0	0	0	0	0	0	0	0
0	0	0	0	0	0	0	0	0	0	0	0	0	0
0													

OTU12044 0 0 0 0 1 0 0 0 0 0 0 0 0
0 0 0 0 0 0 0 0 0 0 0 0 0 0
0 0 0 0 0 0 0 0 0 0 0 0 0 0
0 0 0 0 0 0 0 0 0 0 0 0 0 0
0 0 0 0 0 0 0 0 0 0 0 0 0 0
0 0 0 0 0 0 0 0 0 0 0 0 0 0
0 0 0 0 0 0 0 0 0 0 0 0 0 0
0 0 0 0 0 0 0 0 0 0 0 0 0 0
0
OTU12045 0 0 0 0 1 0 0 0 0 0 0 0 0
0 0 0 0 0 0 0 0 0 0 0 0 0 0
0 0 0 0 0 0 0 0 0 0 0 0 0 0
0 0 0 0 0 0 0 0 0 0 0 0 0 0
0 0 0 0 0 0 0 0 0 0 0 0 0 0
0 0 0 0 0 0 0 0 0 0 0 0 0 0
0 0 0 0 0 0 0 0 0 0 0 0 0 0
0 0 0 0 0 0 0 0 0 0 0 0 0 0
0
OTU14673 0 0 0 0 1 0 0 0 0 0 0 0 0
0 0 0 0 0 0 0 0 0 0 0 0 0 0
0 0 0 0 0 0 0 0 0 0 0 0 0 0
0 0 0 0 0 0 0 0 0 0 0 0 0 0
0 0 0 0 0 0 0 0 0 0 0 0 0 0
0 0 0 0 0 0 0 0 0 0 0 0 0 0
0 0 0 0 0 0 0 0 0 0 0 0 0 0
0 0 0 0 0 0 0 0 0 0 0 0 0 0
0
OTU8336 0 0 0 0 1 0 0 0 0 0 0 0 0
0 0 0 0 0 0 0 0 0 0 0 0 0 0
0 0 0 0 0 0 0 0 0 0 0 0 0 0
0 0 0 0 0 0 0 0 0 0 0 0 0 0
0 0 0 0 0 0 0 0 0 0 0 0 0 0
0 0 0 0 0 0 0 0 0 0 0 0 0 0

0	0	0	0	0	0	0	0	0	0	0	0	0	0
0	0	0	0	0	0	0	0	0	0	0	0	0	0
0													
OTU10854	0	0	0	0	1	0	0	0	0	0	0	0	0
0	0	0	0	0	0	0	0	0	0	0	0	0	0
0	0	0	0	0	0	0	0	0	0	0	0	0	0
0	0	0	0	0	0	0	0	0	0	0	0	0	0
0	0	0	0	0	0	0	0	0	0	0	0	0	0
0	0	0	0	0	0	0	0	0	0	0	0	0	0
0	0	0	0	0	0	0	0	0	0	0	0	0	0
0	0	0	0	0	0	0	0	0	0	0	0	0	0
0													
OTU7402	0	0	0	0	1	0	0	0	0	0	0	0	0
0	0	0	0	0	0	0	0	0	0	0	1	0	0
0	0	0	0	0	0	1	0	0	0	0	0	0	0
0	0	0	0	0	0	0	0	0	0	2	0	0	0
0	0	0	0	0	0	2	0	0	0	0	0	0	0
0	0	0	0	0	0	0	0	0	0	0	0	0	0
0	0	1	0	1	0	0	0	0	0	0	0	0	0
0	0	0	0	0	0	0	0	0	0	0	0	0	0
0													
OTU6445	0	0	0	0	1	0	0	0	0	0	0	0	0
0	0	0	0	2	0	0	0	0	0	0	0	0	0
0	0	0	0	0	1	0	0	0	0	0	0	0	0
0	0	0	0	0	0	0	0	0	0	0	0	0	0
0	3	0	1	0	0	0	0	0	0	0	0	0	0
1	0	0	0	1	0	0	0	0	0	0	0	0	0
0	0	0	0	0	0	0	0	0	0	0	0	0	0
0	0	0	0	0	0	0	0	0	0	0	0	0	0
0													
OTU1175	0	0	0	0	1	0	0	0	0	0	0	0	0
0	0	0	0	0	0	0	0	0	0	0	0	0	0
0	0	0	0	0	0	0	0	0	0	0	0	0	0

0	0	0	0	0	0	0	0	0	0	0	0	0	1
0	0	0	0	0	0	0	0	0	0	0	0	0	0
0	0	0	0	0	0	0	0	0	0	0	0	0	0
0	0	0	0	0	0	0	1	0	0	0	0	0	0
0	0	1	1	0	2	0	1	0	2	0	0	0	3
0													
OTU2368	0	0	0	0	2	1	0	0	1	0	1	0	0
0	0	0	0	1	0	0	0	0	2	0	2	0	1
0	0	0	1	0	1	0	0	1	0	1	1	2	1
0	1	1	1	0	0	0	0	0	1	0	0	0	0
0	0	0	0	1	0	0	1	0	0	0	2	0	0
3	1	1	0	0	1	0	1	0	0	0	1	0	0
0	0	1	0	0	1	0	1	0	1	3	1	0	0
0	1	0	1	0	0	0	0	0	0	1	0	1	0
2													
OTU14709	0	0	0	0	1	0	0	0	0	0	0	0	0
1	0	0	0	0	0	0	0	0	0	0	0	0	0
0	0	0	0	0	0	0	0	0	0	0	0	0	0
0	0	0	0	0	0	0	0	0	0	0	0	0	0
0	0	0	0	0	0	0	0	0	0	0	0	0	0
0	0	0	1	0	0	0	0	0	0	0	0	0	0
0	0	0	0	0	0	0	0	0	0	0	0	0	2
0	0	0	0	0	0	0	0	0	0	0	0	0	0
0													
OTU9932	0	0	0	0	1	0	1	0	0	0	0	0	0
0	0	0	0	0	0	0	0	0	0	0	0	0	0
0	0	0	0	0	0	0	0	0	0	0	0	1	0
0	0	0	0	0	0	0	0	0	0	0	0	1	0
0	0	0	0	0	0	0	0	0	0	0	0	0	0
0	0	0	0	0	0	0	0	0	1	0	1	0	1
0	0	0	0	0	0	0	0	0	0	0	0	0	0
0	0	0	0	0	0	0	0	0	0	0	0	0	1
0													

OTU4414 0 0 0 0 1 0 0 0 0 0 0 0 0
0 0 0 0 0 1 0 0 0 0 0 0 0 1
0 0 0 0 0 0 0 0 0 0 0 0 0 0
0 0 0 0 0 0 0 0 0 0 0 0 0 0
0 0 0 0 0 0 0 0 0 0 0 0 0 0
0 0 1 0 0 0 0 0 0 0 0 0 0 1
0 0 0 0 0 0 0 0 0 0 0 0 0 0
0 0 0 0 0 0 0 0 0 0 0 0 0 0
0
OTU10866 0 0 0 0 1 0 0 0 0 0 0 0 0
0 0 0 0 0 0 0 0 0 0 0 0 0 0
0 0 0 0 0 0 0 0 0 0 0 0 0 0
0 0 0 0 0 0 0 0 0 0 0 0 0 0
0 0 0 0 0 0 0 0 0 0 0 0 0 0
0 0 0 0 0 0 0 0 0 0 0 0 0 0
0 0 0 0 0 0 0 0 0 0 0 0 0 0
0 0 0 0 0 0 0 0 0 0 0 0 0 0
0
OTU10865 0 0 0 0 1 0 0 0 0 0 0 0 0
0 0 0 0 0 0 0 0 0 0 0 0 0 0
0 0 0 0 0 0 0 0 0 0 0 0 0 0
0 0 0 0 0 0 0 0 0 0 0 0 0 0
0 0 0 0 0 0 0 0 0 0 0 0 0 0
0 0 0 0 0 0 0 0 0 0 0 0 0 0
0 0 0 0 0 0 0 0 0 0 0 0 0 0
0 0 0 0 0 0 0 0 0 0 0 0 0 0
0
OTU8337 0 0 0 0 1 0 0 0 0 0 0 0 0
0 0 0 0 0 0 0 0 0 0 0 0 0 0
0 0 0 0 0 0 0 0 0 0 0 0 0 0
0 0 0 0 0 0 0 0 0 0 0 0 0 0
0 0 0 0 0 0 0 0 0 0 0 0 0 0
0 0 0 0 0 0 0 0 0 0 0 0 0 0

0	0	0	0	0	0	0	0	0	0	0	0	0	0
0	0	0	0	0	0	0	0	0	0	0	0	0	0
0													
OTU12053	0	0	0	0	1	0	0	0	0	0	0	0	0
0	0	0	0	0	0	0	0	0	0	0	0	0	0
0	0	0	0	0	0	0	0	0	0	0	0	0	0
0	0	0	0	0	0	0	0	0	0	0	0	0	0
0	0	0	0	0	0	0	0	0	0	0	0	0	0
0	0	0	0	0	0	0	0	0	0	0	0	0	0
0	0	0	0	0	0	0	0	0	0	0	0	0	0
0	0	0	0	0	0	0	0	0	0	0	0	0	0
0													
OTU12476	0	0	0	0	1	0	0	1	0	0	1	0	0
0	0	0	0	0	0	0	0	0	0	0	0	0	0
0	0	0	0	0	0	0	0	0	0	0	0	0	0
0	0	0	0	0	0	0	0	1	0	0	0	0	0
0	0	0	0	0	0	0	0	0	0	0	0	0	0
0	0	0	0	1	0	0	1	0	0	0	0	0	0
0	1	0	0	0	0	0	0	0	0	0	0	0	0
0	0	0	0	0	0	0	0	0	0	0	0	0	0
0													
OTU14675	0	0	0	0	1	0	0	0	0	0	0	0	0
0	0	0	0	0	0	0	0	0	0	0	0	0	0
0	0	0	0	0	0	0	0	0	0	0	0	0	0
0	0	0	0	0	0	0	0	0	0	0	0	0	0
0	0	0	0	0	0	0	0	0	0	0	0	0	0
0	0	0	0	0	0	0	0	0	0	0	0	0	0
0	0	0	0	0	0	0	0	0	0	0	0	0	0
0	0	0	0	0	0	0	0	0	0	0	0	0	0
0													
OTU10863	0	0	0	0	1	0	0	0	0	0	0	0	0
0	0	0	0	0	0	0	0	0	0	0	0	0	0
0	0	0	0	0	0	0	0	0	0	0	0	0	0

0 0 0 0 0 0 0 0 0 0 0 0 0 0
0 0 0 0 0 0 0 0 0 0 0 0 0 0
0 0 0 0 0 0 0 0 0 0 0 0 0 0
0 0 0 0 0 0 0 0 0 0 0 0 0 0
0 0 0 0 0 0 0 0 0 0 0 0 0 0
0
OTU2413 0 0 0 0 1 0 0 0 0 0 0 0 0
1 0 0 0 0 0 0 0 0 0 0 0 0 0
0 0 0 0 0 0 0 0 0 0 0 0 1 0
0 0 0 0 0 0 0 0 0 0 0 0 0 0
0 0 0 0 0 0 0 0 0 0 0 0 0 0
0 0 0 0 0 0 0 0 0 0 0 0 0 0
0 0 0 0 0 0 0 0 0 1 0 0 0 0
0 0 0 0 0 0 0 0 0 0 0 0 0 0
0
OTU8332 0 0 0 0 1 0 0 0 0 0 0 0 0
0 0 0 0 0 0 0 0 0 0 0 0 0 0
0 0 0 0 0 0 0 0 0 0 0 0 0 0
0 0 0 0 0 0 0 0 0 0 0 0 0 0
0 0 0 0 0 0 0 0 0 0 0 0 0 0
0 0 0 0 0 0 0 0 0 0 0 0 0 0
0 0 0 0 0 0 0 0 0 0 0 0 0 0
0 0 0 0 0 0 0 0 0 0 0 0 0 0
0
OTU11067 0 0 0 0 1 0 0 0 0 0 0 0 0
0 0 0 0 0 0 0 0 0 0 2 0 0 0
0 0 0 0 1 0 0 0 0 0 0 0 0 1
0 0 0 0 0 0 0 0 0 0 0 0 0 0
0 0 0 0 0 0 0 0 0 0 0 0 0 0
0 0 0 0 0 0 0 0 0 0 0 0 0 0
0 0 0 0 0 0 0 0 0 0 1 0 0 0
0 0 0 0 0 0 0 0 0 0 0 0 0 0
0

OTU10864	0	0	0	0	1	0	0	0	0	0	0	0	0
0	0	0	0	0	0	0	0	0	0	0	0	0	0
0	0	0	0	0	0	0	0	0	0	0	0	0	0
0	0	0	0	0	0	0	0	0	0	0	0	0	0
0	0	0	0	0	0	0	0	0	0	0	0	0	0
0	0	0	0	0	0	0	0	0	0	0	0	0	0
0	0	0	0	0	0	0	0	0	0	0	0	0	0
0	0	0	0	0	0	0	0	0	0	0	0	0	0
0													
OTU7916	0	0	0	0	1	0	0	0	0	0	0	0	0
0	0	1	0	0	0	0	0	0	0	0	0	0	0
0	0	0	0	0	0	0	0	0	0	0	0	0	0
0	0	0	0	0	0	0	0	0	0	0	0	0	0
0	0	0	0	0	0	0	0	0	0	0	0	0	0
0	0	0	0	0	0	0	0	0	0	0	0	0	0
0	0	0	0	0	0	0	0	0	0	0	0	0	0
0	0	0	0	0	0	0	0	0	0	0	0	0	0
0													
OTU8289	0	0	0	0	1	0	0	0	0	0	0	0	0
0	0	0	0	0	0	0	0	0	0	0	0	0	0
0	0	0	0	0	0	0	0	0	0	0	0	0	0
0	0	0	0	0	0	0	0	0	0	0	0	0	0
0	0	0	0	0	0	0	0	0	0	0	0	0	0
0	0	0	0	0	0	0	0	0	0	0	0	0	0
0	0	0	0	0	0	0	0	0	0	0	0	0	0
0	0	0	0	0	0	0	0	0	0	0	1	0	0
0													
OTU216	0	0	0	0	1	0	0	0	3	0	0	0	0
7	0	0	12	0	0	3	2	0	0	0	0	0	0
2	1	35	3	0	1	0	3	0	0	124	6	1	0
0	2	0	0	13	2	1	0	3	2	0	0	1	1
0	1	2	2	2	5	0	0	2	0	52	9	1	0
5	0	8	2	0	0	0	1	3	0	1	9	0	1

4	29	4	0	0	0	2	0	0	0	8	0	0	0
2	0	0	0	0	0	0	0	1	0	0	1	1	6
1													
OTU10867	0	0	0	0	1	0	1	0	0	1	0	0	0
0	0	0	0	0	0	0	0	0	0	0	0	0	0
0	0	0	0	0	0	0	0	0	0	0	0	0	0
0	0	0	0	0	0	0	0	0	0	0	0	0	0
0	0	0	0	0	0	0	0	0	0	0	0	0	0
0	0	0	0	0	0	0	0	0	0	0	0	0	0
0	0	0	0	0	0	0	0	0	0	0	0	0	0
0	0	0	0	0	0	0	0	0	0	0	0	0	0
0													
OTU14671	0	0	0	0	1	0	0	0	0	0	0	0	0
0	0	0	0	0	0	0	0	0	0	0	0	0	0
0	0	0	0	0	0	0	0	0	0	0	0	0	0
0	0	0	0	0	0	0	0	0	0	0	0	0	0
0	0	0	0	0	0	0	0	0	0	0	0	0	0
0	0	0	0	0	0	0	0	0	0	0	0	0	0
0	0	0	0	0	0	0	0	0	0	0	0	0	0
0	0	0	0	0	0	0	0	0	0	0	0	0	0
0													
OTU8333	0	0	0	0	1	0	0	0	0	0	0	0	0
0	0	0	0	0	0	0	0	0	0	0	1	0	0
0	0	0	0	0	0	0	0	0	1	0	0	0	0
0	0	0	0	0	0	0	0	0	0	0	0	0	0
0	0	0	0	0	0	0	0	0	0	0	0	1	0
0	0	0	0	0	0	0	0	0	0	0	0	0	0
0	0	0	0	0	0	0	0	0	0	0	0	0	0
0	0	0	0	0	0	0	0	0	0	0	0	0	0
0													
OTU9193	0	0	0	0	1	1	0	0	0	0	0	0	0
0	0	0	0	0	0	0	0	0	0	0	1	0	0
0	0	0	0	0	0	0	0	0	0	0	0	0	0

0	0	0	0	0	0	0	0	0	0	0	0	0	0
0	0	0	0	0	0	0	0	0	0	0	0	0	0
0	0	0	0	0	0	0	0	0	0	0	0	0	0
0	0	0	0	0	0	0	0	0	0	0	0	0	0
0	0	0	0	0	0	0	0	0	0	0	0	0	0
0													
OTU4777	0	0	0	0	1	2	0	0	0	2	0	1	0
0	1	0	0	2	0	0	0	2	0	0	0	0	0
0	0	0	0	0	0	0	0	0	0	0	0	0	1
0	0	0	0	0	0	0	1	3	0	0	0	0	0
0	0	0	0	0	0	0	0	0	0	1	0	1	0
0	0	0	1	2	0	0	0	0	0	0	1	0	0
0	0	1	0	0	2	0	0	0	0	0	0	0	1
0	0	0	0	0	1	0	0	0	1	0	0	0	0
0													
OTU4986	0	0	0	0	1	0	0	0	0	0	1	2	0
0	0	1	0	2	1	0	1	0	1	0	0	0	0
0	0	0	0	0	0	0	0	0	0	0	0	0	0
0	0	0	0	0	1	0	0	1	0	0	0	0	0
0	0	0	0	0	1	0	0	1	0	0	0	0	0
2	0	0	0	1	0	0	0	0	0	0	0	0	0
0	1	0	0	0	1	0	0	0	0	0	0	0	0
2	0	0	0	0	0	0	0	0	0	0	0	0	0
2													
OTU7220	0	0	0	0	1	0	0	1	0	0	0	0	0
0	0	0	0	0	0	0	0	0	0	0	0	0	0
0	0	0	0	0	0	0	0	0	0	0	0	0	0
0	0	0	1	0	0	0	0	0	0	0	0	0	0
0	0	0	0	0	0	0	0	0	0	0	0	0	0
0	0	2	0	0	0	0	0	1	0	0	0	0	0
0	0	0	0	0	0	0	0	0	0	0	0	0	0
0	0	0	0	0	0	0	0	0	0	0	0	0	0
0													

OTU8111	0	0	0	0	3	0	3	0	0	2	0	0	1
2	2	1	0	3	0	2	0	0	1	0	1	0	1
0	0	2	1	0	2	0	2	1	1	1	0	3	1
1	1	0	1	0	0	1	1	0	1	1	0	0	0
1	1	0	3	0	0	0	1	0	0	1	1	0	0
1	1	0	0	1	0	0	4	0	2	0	1	0	0
0	0	0	0	1	3	0	3	0	0	1	0	1	0
0	1	0	0	0	0	1	2	2	2	2	0	0	0
3													
OTU2452	0	0	0	0	1	0	0	0	0	0	0	0	0
0	0	0	0	0	0	0	0	0	0	0	0	0	0
0	0	0	1	0	0	0	0	0	0	0	0	0	0
0	0	0	0	0	0	0	0	0	0	0	0	0	0
0	0	0	0	0	0	0	0	0	0	0	0	0	0
0	0	0	0	0	0	0	2	0	0	0	0	0	0
0	0	0	0	0	0	0	1	0	0	0	0	0	0
0	0	1	0	0	0	0	0	0	0	0	0	0	0
1													
OTU12055	0	0	0	0	1	0	0	0	0	0	0	0	0
0	0	0	0	0	0	0	0	0	0	0	0	0	0
0	0	0	0	0	0	0	0	0	0	0	0	0	0
0	0	0	0	0	0	0	0	0	0	0	0	0	0
0	0	0	0	0	0	0	0	0	0	0	0	0	0
0	0	0	0	0	0	0	0	0	0	0	0	0	0
0	0	0	0	0	0	0	0	0	0	0	0	0	0
0	0	0	0	0	0	0	0	0	0	0	0	0	0
0													
OTU10860	0	0	0	0	1	1	0	0	0	0	0	0	0
0	0	0	0	0	0	0	1	0	0	0	0	0	0
0	0	0	0	0	0	0	0	0	0	0	0	0	0
0	0	0	1	0	0	0	0	0	0	0	0	0	0
0	0	0	2	0	0	0	0	0	0	0	0	0	0
0	0	0	0	0	0	0	0	0	0	0	0	0	0

0	0	0	0	0	0	0	0	0	0	0	0	0	0
0	0	0	0	0	0	0	0	0	0	0	0	0	0
0													
OTU12051	0	0	0	0	1	0	0	0	0	0	0	0	0
0	0	0	0	0	0	0	0	0	0	0	0	0	0
0	0	0	0	0	0	0	0	0	0	0	0	0	0
0	0	0	0	0	0	0	0	0	0	0	0	0	0
0	0	0	0	0	0	0	0	0	0	0	0	0	0
0	0	0	0	0	0	0	0	0	0	0	0	0	0
0	0	0	0	0	0	0	0	0	0	0	0	0	0
0	0	0	0	0	0	0	0	0	0	0	0	0	0
0													
OTU14098	0	0	0	0	1	0	0	0	0	0	0	0	0
0	0	0	0	0	0	0	0	0	0	0	0	0	0
0	0	1	0	0	0	0	1	0	0	0	0	0	0
0	0	0	0	0	0	0	0	0	0	0	0	0	0
0	0	0	0	0	0	0	0	1	0	0	0	0	0
0	0	0	0	0	0	1	0	0	1	0	0	0	0
0	0	0	0	0	0	0	0	0	0	0	0	0	0
0	0	0	0	0	0	0	0	0	0	0	0	0	0
0													
OTU14672	0	0	0	0	1	0	0	0	0	0	0	0	0
0	0	0	0	0	0	0	0	0	0	0	0	0	0
0	0	0	0	0	0	0	0	0	0	0	0	0	0
0	0	0	0	0	0	0	0	0	0	0	0	0	0
0	0	0	0	0	0	0	0	0	0	0	0	0	0
0	0	0	0	0	0	0	0	0	0	0	0	0	0
0	0	0	0	0	0	0	0	0	0	0	0	0	0
0	0	0	0	0	0	0	0	0	0	0	0	0	0
0													
OTU12050	0	0	0	0	1	0	0	0	0	0	0	0	0
0	0	0	0	0	0	0	0	0	0	0	0	0	0
0	0	0	0	0	0	0	0	0	0	0	0	0	0

0	0	0	0	0	0	0	0	0	0	0	0	0	0
0	0	0	0	0	0	0	0	0	0	0	0	0	0
0	0	0	0	0	0	0	0	0	0	0	0	0	0
0	0	0	0	0	0	0	0	0	0	0	0	0	0
0	0	0	0	0	0	0	0	0	0	0	0	0	0
0													
OTU12049	0	0	0	0	1	0	0	0	0	0	0	0	0
0	0	0	0	0	0	0	0	0	0	0	0	0	0
0	0	0	0	0	0	0	0	0	0	0	0	0	0
0	0	0	0	0	0	0	0	0	0	0	0	0	0
0	0	0	0	0	0	0	0	0	0	0	0	0	0
0	0	0	0	0	0	0	0	0	0	0	0	0	0
0	0	0	0	0	0	0	0	0	0	0	0	0	0
0	0	0	0	0	0	0	0	0	0	0	0	0	0
0													
OTU5130	0	0	0	0	1	1	0	0	1	0	1	1	0
0	0	0	0	0	0	2	0	0	0	0	0	0	0
0	0	0	0	0	0	0	0	0	0	0	0	3	0
0	0	0	0	0	3	0	3	0	0	0	0	0	1
0	2	0	0	0	0	0	0	0	0	0	0	2	0
0	0	0	0	1	0	0	0	1	0	0	0	0	1
0	0	0	0	0	1	0	0	0	0	0	0	0	0
0	0	0	1	0	0	0	0	0	0	0	0	0	0
0													
OTU1472	0	0	0	0	1	0	1	0	0	0	0	0	0
0	0	0	0	0	0	0	1	0	0	0	1	0	0
0	0	0	0	0	0	0	1	1	0	0	0	1	0
0	0	0	0	0	0	0	0	0	0	0	0	0	0
0	0	0	0	0	0	0	0	0	0	0	0	0	0
0	0	0	3	0	0	0	0	0	0	0	0	0	0
0	0	0	0	0	0	2	0	0	0	0	0	0	0
0	0	0	0	0	0	0	0	0	0	0	1	0	0
0													

OTU1000	0	0	0	0	2	0	0	0	0	0	0	0	0
0	0	0	0	0	0	0	0	0	0	0	0	0	0
0	0	0	0	0	0	0	0	0	0	0	0	0	0
0	0	0	0	0	0	0	0	0	0	0	0	0	0
0	0	0	0	0	0	0	0	0	0	0	0	0	0
0	0	0	0	0	0	0	0	0	0	0	0	0	0
0	0	0	0	0	0	0	0	0	0	0	0	0	0
0	0	0	0	0	0	0	0	0	0	0	0	0	0
0													
OTU10858	0	0	0	0	1	0	0	0	0	0	0	0	0
0	0	0	0	0	0	0	0	0	0	0	0	0	0
0	0	0	0	0	0	0	0	0	0	0	0	0	0
0	0	0	0	0	0	0	0	0	0	0	0	0	0
0	0	0	0	0	0	0	0	0	0	0	0	0	0
0	0	0	0	0	0	0	0	0	0	0	0	0	0
0	0	0	0	0	0	0	0	0	0	0	0	0	0
0	0	0	0	0	0	0	0	0	0	0	0	0	0
0													
OTU14678	0	0	0	0	1	1	0	0	0	0	1	0	0
0	0	0	0	0	0	0	0	0	0	0	0	0	0
0	0	0	0	0	0	0	0	0	0	0	0	0	0
0	0	0	0	0	0	0	0	0	0	0	0	0	0
0	0	0	0	0	0	0	0	0	0	0	0	0	0
0	0	0	0	0	0	0	0	0	0	0	0	0	0
0	0	0	0	0	0	0	0	0	0	0	0	0	0
0	0	0	0	0	0	0	0	0	0	0	0	0	0
0													
OTU3318	0	0	0	0	3	0	0	0	0	0	0	0	0
0	0	0	0	0	0	0	0	0	0	0	0	0	0
0	0	0	0	0	0	0	1	2	0	0	0	0	0
0	0	0	0	0	0	0	0	0	0	0	0	0	1
0	0	0	0	0	0	0	0	0	0	0	0	0	0
0	0	0	0	2	0	0	0	0	0	0	0	0	0

0 0 0 0 3 1 0 0 0 0 0 0 0 0
0 0 0 0 0 0 0 0 0 0 3 0 0 0
0
OTU12052 0 0 0 0 1 0 0 0 0 0 0 0 0
0 0 0 0 0 0 0 0 0 0 0 0 0 0
0 0 0 0 0 0 0 0 0 0 0 0 0 0
0 0 0 0 0 0 0 0 0 0 0 0 0 0
0 0 0 0 0 0 0 0 0 0 0 0 0 0
0 0 0 0 0 0 0 0 0 0 0 0 0 0
0 0 0 0 0 0 0 0 0 0 0 0 0 0
0 0 0 0 0 0 0 0 0 0 0 0 0 0
0
OTU8424 0 0 0 0 1 0 0 0 0 0 0 0 0
0 0 0 0 0 0 0 0 0 0 0 0 0 0
0 0 0 0 0 0 0 0 0 0 0 0 0 0
0 0 0 0 0 0 0 0 0 0 0 0 0 0
0 0 0 0 0 0 0 0 0 0 0 0 0 0
0 0 0 0 0 0 0 0 0 0 0 0 0 0
0 0 0 0 0 0 0 0 0 0 0 0 0 0
0 0 0 0 0 0 0 0 0 0 0 0 0 0
0
OTU559 0 0 0 0 1 0 0 0 0 0 0 0 0
0 0 0 0 0 0 0 0 0 0 0 0 0 0
0 0 0 0 0 0 0 0 0 0 0 0 0 0
0 0 0 0 0 0 0 0 0 0 0 0 0 0
0 0 0 0 0 0 0 0 0 0 0 0 0 0
0 0 0 0 0 0 0 0 0 0 0 0 0 1
0 0 0 0 0 0 0 0 0 0 0 0 0 0
0 0 0 0 0 0 21 2 1 0 0 0 0 2
0
OTU10862 0 0 0 0 1 0 0 0 0 0 0 0 0
0 0 0 0 0 0 0 0 0 0 0 0 0 0
0 0 0 0 0 0 0 0 0 0 0 0 0 0

0 0 0 0 0 0 0 0 0 0 0 0 0 0
0 0 0 0 0 0 0 0 0 0 0 0 0 0
0 0 0 0 0 0 0 0 0 0 0 0 0 0
0 0 0 0 0 0 0 0 0 0 0 0 0 0
0 0 0 0 0 0 0 0 0 0 0 0 0 0
0
OTU3387 0 0 0 0 1 1 0 0 0 1 0 0 0
0 0 0 0 0 0 0 0 0 0 0 0 0 0
0 0 0 0 0 1 0 0 0 1 0 0 0 0
0 0 0 0 0 0 0 0 0 0 0 0 0 0
0 0 0 0 0 0 0 0 0 0 0 0 0 0
0 0 0 0 0 0 0 0 0 0 1 0 0 0
0 1 0 0 0 0 0 0 0 0 0 0 0 0
0 0 0 0 0 0 0 1 1 0 0 0 0 0
0
OTU14677 0 0 0 0 1 0 0 0 0 0 0 0 0
0 0 0 0 0 0 0 0 0 0 0 0 0 0
0 0 0 0 0 0 0 0 0 0 0 0 0 0
0 0 0 0 0 0 0 0 0 0 0 0 0 0
0 0 0 0 0 0 0 0 0 0 0 0 0 0
0 0 0 0 0 0 0 0 0 0 0 0 0 0
0 0 0 0 0 0 0 0 0 0 0 0 0 0
0 0 0 0 0 0 0 0 0 0 0 0 0 0
0
OTU12074 0 0 0 0 1 0 0 0 0 0 0 0 0
0 0 0 0 0 0 0 0 0 0 0 0 0 0
0 0 0 0 0 0 0 0 0 0 0 0 0 0
0 0 0 0 0 0 0 0 0 0 0 0 0 0
0 0 0 0 0 0 0 0 0 0 0 0 0 0
0 0 0 0 0 0 0 0 0 0 0 0 0 0
0 0 0 0 0 0 0 0 0 0 0 0 0 0
0 0 0 0 0 0 0 0 0 0 0 0 0 0
0

OTU4801 0 0 0 0 1 0 0 0 0 0 0 0 0
0 0 0 0 0 0 0 0 0 0 0 0 0 0
0 0 0 0 0 2 0 0 0 0 0 0 0 0
0 0 0 0 0 0 0 1 0 0 0 0 0 0
0 0 0 0 0 0 1 0 0 0 0 0 0 0
0 0 0 0 0 0 0 0 0 0 0 0 0 0
0 0 0 0 0 0 1 0 0 0 0 0 0 0
0 0 0 0 0 0 0 0 0 0 0 0 0 0
0
OTU6507 0 0 0 0 1 1 0 1 0 0 0 0 1
0 0 0 0 0 0 3 0 0 0 0 0 0 0
0 0 0 0 0 0 0 0 0 0 0 0 0 0
0 0 0 0 0 0 0 0 0 0 0 0 0 0
0 1 0 0 0 0 0 0 0 0 0 0 0 0
0 0 0 0 0 0 0 0 0 0 0 1 0 0
0 0 0 0 0 0 0 0 0 0 0 0 0 0
0 0 0 0 0 0 0 0 0 0 0 0 0 0
0
OTU12075 0 0 0 0 1 0 0 0 0 0 0 0 0
0 0 0 0 0 0 0 0 0 0 0 0 0 0
0 0 0 0 0 0 0 0 0 0 0 0 0 0
0 0 0 0 0 0 0 0 0 0 0 0 0 0
0 0 0 0 0 0 0 0 0 0 0 0 0 0
0 0 0 0 0 0 0 0 0 0 0 0 0 0
0 0 0 0 0 0 0 0 0 0 0 0 0 0
0 0 0 0 0 0 0 0 0 0 0 0 0 0
0
OTU14679 0 0 0 0 1 0 0 0 0 0 0 0 0
0 0 0 0 0 0 0 0 0 0 0 0 0 0
0 0 0 0 0 0 0 0 0 0 0 0 0 0
0 0 0 0 0 0 0 0 0 0 0 0 0 0
0 0 0 0 0 0 0 0 0 0 0 0 0 0
0 0 0 0 0 0 0 0 0 0 0 0 0 0

0	0	0	0	0	0	0	0	0	0	0	0	0	0
0	0	0	0	0	0	0	0	0	0	0	0	0	0
0													
OTU8421	0	0	0	0	1	2	0	0	0	0	0	0	0
0	0	0	0	0	0	0	0	0	0	0	0	0	0
0	0	0	0	0	0	0	0	0	0	0	0	0	0
0	0	0	0	0	0	0	0	0	0	0	0	0	0
0	0	0	0	0	0	0	0	0	0	0	0	0	0
0	0	0	0	0	0	0	0	0	0	0	0	0	0
0	0	0	0	0	0	0	0	0	0	0	0	0	0
0	0	0	0	0	0	0	0	0	0	0	0	0	0
0													
OTU5979	0	0	0	0	1	0	0	0	1	0	0	1	0
0	0	0	0	0	0	0	0	0	0	0	0	0	0
0	1	0	0	0	0	0	0	0	0	1	0	1	1
0	0	0	0	0	0	0	0	0	0	0	0	0	0
0	0	1	0	0	0	0	0	1	0	1	0	0	0
1	0	0	0	1	0	0	0	0	0	0	1	0	0
0	0	0	0	0	0	0	0	0	0	0	0	0	0
0	0	0	0	0	0	0	2	0	0	0	0	0	0
0													
OTU10861	0	0	0	0	1	0	0	0	0	0	0	0	0
0	0	0	0	0	0	0	0	0	0	0	0	0	0
0	0	0	0	0	0	0	0	0	0	0	0	0	0
0	0	0	0	0	0	0	0	0	0	0	0	0	0
0	0	0	0	0	0	0	0	0	0	0	0	0	0
0	0	0	0	0	0	0	0	0	0	0	0	0	0
0	0	0	0	0	0	0	0	0	0	0	0	0	0
0	0	0	0	0	0	0	0	0	0	0	0	0	0
0													
OTU12076	0	0	0	0	1	0	0	0	0	0	0	0	0
0	0	0	0	0	0	0	0	0	0	0	0	0	0
0	0	0	0	0	0	0	0	0	0	0	0	0	0

0 0 0 0 0 0 0 0 0 0 0 0 0 0
0 0 0 0 0 0 0 0 0 0 0 0 0 0
0 0 0 0 0 0 0 0 0 0 0 0 0 0
0 0 0 0 0 0 0 0 0 0 0 0 0 0
0 0 0 0 0 0 0 0 0 0 0 0 0 0
0
OTU14427 0 0 0 0 1 0 0 1 0 0 0 0 0
0 0 0 0 0 0 0 0 0 1 0 0 0 0
0 0 0 0 0 0 0 0 0 0 0 0 0 0
0 0 0 0 0 0 0 0 0 0 0 0 0 0
0 0 0 0 0 0 0 0 0 0 0 0 0 0
0 0 1 0 0 0 0 0 0 0 0 0 0 0
0 0 0 0 0 0 0 0 0 0 0 0 0 0
0 0 0 0 0 0 0 0 0 0 0 0 0 0
0
OTU8422 0 0 0 0 1 0 0 0 0 0 0 0 0
0 0 0 0 0 0 0 0 0 0 0 0 0 0
0 0 0 0 0 0 0 0 0 0 0 0 0 0
0 0 0 0 0 0 0 0 0 0 0 0 0 0
0 0 0 0 0 0 0 0 0 0 0 0 0 0
0 0 0 0 0 0 0 0 0 0 0 0 0 0
0 0 0 0 0 0 0 0 0 0 0 0 0 0
0 0 0 0 0 0 0 0 0 0 0 0 0 0
0
OTU11693 0 0 0 0 1 0 0 0 0 0 0 0 0
0 0 1 0 1 2 0 0 0 0 0 0 0 0
0 0 1 0 0 0 0 0 0 1 0 2 0 1
0 0 0 0 0 0 0 0 0 0 0 0 0 0
0 0 0 0 0 0 0 0 0 0 0 0 0 0
0 0 0 0 0 0 0 0 0 0 0 0 0 0
0 0 0 0 0 0 0 0 0 0 0 0 0 0
0 0 0 0 0 0 0 0 0 0 0 0 0 0
0

OTU10876 0 0 0 0 1 4 0 0 0 0 0 0 0
0 1 0 0 0 0 3 0 0 0 0 0 0 1
0 0 0 0 1 0 0 0 0 0 0 0 0 0
0 0 0 2 0 0 0 0 0 0 0 0 1 0
0 0 0 1 0 0 0 0 0 0 0 0 0 0
0 1 0 0 0 0 0 0 0 0 0 0 0 0
0 0 0 0 0 0 0 0 0 0 0 0 0 0
0 0 0 1 1 0 0 3 0 1 0 0 0 1
0
OTU10845 0 0 0 0 1 0 0 0 0 0 0 0 0
0 0 0 1 0 0 0 0 0 0 0 0 0 0
0 0 0 0 0 0 0 0 1 0 0 0 0 0
0 0 0 0 0 0 0 0 0 0 1 0 0 0
0 0 0 0 0 0 0 0 0 0 0 0 1 0
1 0 0 0 2 0 0 0 0 0 0 0 0 0
0 0 0 0 0 0 0 0 0 0 0 0 0 0
0 0 0 0 0 0 1 0 0 0 0 0 0 0
0
OTU5461 0 0 0 0 1 1 0 0 0 0 0 0 0
0 0 0 0 0 0 0 0 0 0 0 0 1 0
0 0 0 0 0 0 1 0 2 0 0 0 1 0
0 0 0 0 0 2 0 1 0 0 0 0 0 0
0 1 0 0 0 0 0 0 0 0 0 0 1 0
0 0 0 0 0 1 0 0 0 0 0 0 0 0
0 0 0 1 0 1 0 0 0 0 0 0 0 0
0 0 0 0 0 0 0 0 0 0 0 0 0 0
0
OTU8430 0 0 0 0 1 0 0 0 0 0 0 0 0
0 0 0 0 0 0 0 0 0 0 0 0 0 0
0 0 0 0 0 0 0 0 0 0 0 0 0 0
0 0 0 0 0 0 0 0 0 0 0 0 0 0
0 0 0 0 0 0 0 0 0 0 0 0 0 0
0 0 0 0 0 0 0 0 0 0 0 0 0 0

0	0	0	0	0	0	0	0	0	0	0	0	0	0
0	0	0	0	0	0	0	0	0	0	0	0	0	0
0													
OTU12747	0	0	0	0	1	0	0	0	0	0	0	0	0
0	0	0	1	0	0	0	0	0	0	0	0	0	0
0	0	0	0	0	0	0	1	0	0	2	0	0	0
0	0	0	0	0	0	1	0	0	0	0	0	0	0
0	1	0	0	0	0	0	0	0	0	0	0	0	0
0	0	0	0	0	0	0	0	0	0	0	0	0	0
0	0	0	0	0	0	0	0	0	0	0	0	0	1
0	0	0	0	0	0	0	0	0	0	0	0	0	0
0													
OTU8429	0	0	0	0	1	0	0	0	0	0	0	0	0
0	0	0	0	0	0	0	0	0	0	0	0	0	0
0	0	0	0	0	0	0	0	0	0	0	0	0	0
0	0	0	0	0	0	0	0	0	0	0	0	0	0
0	0	0	0	0	0	0	0	0	0	0	0	0	0
0	0	0	0	0	0	0	0	0	0	0	0	0	0
0	0	0	0	0	0	0	0	0	0	0	0	0	0
0	0	0	0	0	0	0	0	0	0	0	0	0	0
0													
OTU10844	0	0	0	0	1	0	0	0	0	0	0	0	0
0	0	0	0	0	0	0	0	0	0	0	0	0	0
0	0	0	0	0	0	0	0	0	0	0	0	0	0
0	0	0	0	0	0	0	0	0	0	0	0	0	0
0	0	0	0	0	0	0	0	0	0	0	0	0	0
0	0	0	0	0	0	0	0	0	0	0	0	0	0
0	0	0	0	0	0	0	0	0	0	0	0	0	0
0	0	0	0	0	0	0	0	0	0	0	0	0	0
0													
OTU8428	0	0	0	0	1	0	0	0	0	0	0	0	0
0	0	0	0	0	0	0	0	0	0	0	0	0	0
0	0	0	0	0	0	0	0	0	0	0	0	0	0

0	0	0	0	0	0	0	0	0	0	0	0	0	0
0	0	0	0	0	0	0	0	0	0	0	0	0	0
0	0	0	0	0	0	0	0	0	0	0	0	0	0
0	0	0	0	0	0	0	0	0	0	0	0	0	0
0	0	0	0	0	0	0	0	0	0	0	0	0	0
0													
OTU10890	0	0	0	0	2	0	0	1	0	0	1	0	0
0	2	0	0	1	0	0	2	1	0	0	0	0	0
1	0	0	0	0	1	0	0	0	0	0	0	1	0
0	1	0	0	0	0	0	0	0	2	0	0	1	0
1	0	0	0	0	0	0	0	1	0	1	1	1	0
0	0	1	0	1	0	0	2	1	1	0	2	0	0
0	0	0	0	0	0	1	0	0	0	0	0	0	1
0	0	1	0	2	0	0	1	0	0	0	0	0	0
1													
OTU10847	0	0	0	0	1	0	0	0	0	0	0	0	0
0	0	0	0	0	0	0	0	0	0	0	0	0	0
0	0	0	0	0	0	0	0	0	0	0	0	0	0
0	0	0	0	0	0	0	0	0	0	0	0	0	0
0	0	0	0	0	0	0	0	0	0	0	0	0	0
0	0	0	0	0	0	0	0	0	0	0	0	0	0
0	0	0	0	0	0	0	0	0	0	0	0	0	0
0	0	0	0	0	0	0	0	0	0	0	0	0	0
0													
OTU11737	0	0	0	0	2	0	0	0	0	0	3	0	0
0	0	0	0	2	0	0	0	0	2	0	0	0	0
0	1	2	1	0	0	0	0	0	0	0	0	1	0
0	0	0	0	0	0	0	0	0	0	0	0	0	0
0	0	0	0	0	0	0	0	0	0	2	1	1	0
0	0	0	0	2	0	0	2	0	0	0	4	0	0
0	0	0	0	0	0	0	0	0	1	1	0	0	1
0	0	0	0	1	0	1	1	0	1	0	0	0	0
1													

OTU10846 0 0 0 0 1 0 0 0 0 0 0 0 0
0 0 0 0 0 0 0 0 0 0 0 0 0 0
0 0 0 0 0 0 0 0 0 0 0 0 0 0
0 0 0 0 0 0 0 0 0 0 0 0 0 0
0 0 0 0 0 0 0 0 0 0 0 0 0 0
0 0 0 0 0 0 0 0 0 0 0 0 0 0
0 0 0 0 0 0 0 0 0 0 0 0 0 0
0 0 0 0 0 0 0 0 0 0 0 0 0 0
0
OTU6200 0 0 0 0 2 0 0 0 0 0 0 0 0
0 0 0 0 0 0 0 0 0 0 0 0 0 0
0 0 0 0 0 0 0 0 0 0 0 0 0 0
0 0 0 0 0 0 0 0 3 0 1 0 0 0
0 1 0 0 0 0 0 0 0 0 0 0 0 0
0 0 0 0 0 0 0 0 0 0 0 0 0 0
0 0 0 0 0 0 0 0 0 0 0 0 0 0
0 0 0 0 0 0 0 0 0 0 0 0 0 0
0
OTU7624 0 0 0 0 1 0 0 0 0 4 0 0 0
0 0 0 0 0 0 0 0 0 0 0 0 0 0
0 0 0 0 0 0 0 0 0 0 0 0 0 0
0 0 0 0 0 0 0 0 0 0 0 0 0 0
0 0 0 0 0 1 0 0 0 0 0 0 0 0
0 0 0 0 0 0 0 0 0 0 0 0 0 0
0 0 0 0 0 0 0 0 0 0 0 0 0 0
0 0 0 1 1 0 0 0 0 0 0 0 0 1
0
OTU9953 0 0 0 0 1 3 0 1 0 0 0 0 0
0 0 0 0 0 0 0 0 0 0 0 0 0 0
0 0 0 1 0 0 0 0 0 0 0 0 0 0
0 0 1 0 0 0 0 3 0 0 0 0 0 0
0 1 0 1 0 0 0 0 0 0 1 0 2 1
0 0 0 2 0 0 0 0 0 1 0 0 0 3

1	0	0	3	2	0	0	0	0	0	0	0	0	1
1	0	0	0	0	0	0	0	0	0	0	0	0	0
0													
OTU8427	0	0	0	0	1	0	0	0	0	0	0	0	0
0	0	0	0	0	0	0	0	0	0	0	0	0	0
0	0	0	0	0	0	0	0	0	0	0	0	0	0
0	0	0	0	0	0	0	0	0	0	0	0	0	0
0	0	0	0	0	0	0	0	0	0	0	0	0	0
0	0	0	0	2	0	0	0	0	0	1	0	0	0
0	0	0	0	0	0	0	0	0	0	0	0	0	0
0	0	0	0	0	0	0	0	0	0	0	0	0	0
0													
OTU8370	0	0	0	0	2	1	1	0	0	0	1	0	0
0	1	1	0	0	0	0	0	2	1	1	0	0	0
0	2	0	1	0	0	1	0	0	0	2	0	2	0
1	0	1	0	1	0	0	1	0	3	0	0	0	1
2	0	1	0	0	0	1	0	0	1	0	1	1	0
1	0	0	0	2	1	0	0	0	1	0	1	0	0
1	0	0	0	0	0	0	1	0	0	0	1	0	0
0	0	0	0	0	0	0	0	1	0	2	0	0	1
0													
OTU14639	0	0	0	0	1	0	0	0	0	0	0	0	0
0	0	0	0	0	0	0	0	0	0	0	0	0	0
0	0	0	0	0	0	0	0	0	0	0	0	0	0
0	0	0	0	0	0	0	0	0	0	0	0	0	0
0	0	0	0	0	0	0	0	0	0	0	0	0	0
0	0	0	0	0	0	0	0	0	0	0	0	0	0
0	0	0	0	0	0	0	0	0	0	0	0	0	0
0	0	0	0	0	0	0	0	0	0	0	0	0	0
0													
OTU10843	0	0	0	0	1	0	0	0	0	0	0	0	0
0	0	0	0	0	0	0	0	0	0	0	0	0	0
0	0	0	0	0	0	0	0	0	0	0	0	0	0

0	0	0	0	0	0	0	0	0	0	0	0	0	0
0	0	0	0	0	0	0	0	0	0	0	0	0	0
0	0	0	0	0	0	0	0	0	0	0	0	0	0
0	0	0	0	0	0	0	0	0	0	0	0	0	0
0	0	0	0	0	0	0	0	0	0	0	0	0	0
0													
OTU13414	0	0	0	0	1	0	0	0	0	1	0	0	0
0	1	0	0	0	0	0	0	0	0	0	0	0	0
0	0	0	0	0	0	0	0	0	0	0	0	0	0
0	0	0	0	0	0	0	0	0	0	0	0	0	0
0	0	0	0	0	0	0	0	0	0	0	0	0	0
0	0	0	0	0	0	0	0	0	0	0	0	0	0
0	1	0	0	0	0	0	0	0	0	0	0	0	0
0	0	0	0	0	0	0	0	0	0	0	0	0	0
0													
OTU8426	0	0	0	0	1	0	0	0	0	0	0	0	0
0	0	0	0	0	0	0	0	0	0	0	0	0	0
0	0	0	0	0	0	0	0	0	0	0	0	0	0
0	0	0	0	0	0	0	0	0	0	0	0	0	0
0	0	0	0	0	0	0	0	0	0	0	0	0	0
0	0	0	0	0	0	0	0	0	0	0	0	0	0
0	0	0	0	0	0	0	0	0	0	0	0	0	0
1	0	0	0	0	0	0	0	0	0	1	0	0	0
0													
OTU8425	0	0	0	0	1	0	0	0	0	0	0	0	0
0	0	0	0	0	0	0	0	0	0	0	0	0	0
0	0	0	0	0	0	0	0	0	0	0	0	0	0
0	0	0	0	0	0	0	0	0	0	0	0	0	0
0	0	0	0	0	0	0	0	0	0	0	0	0	0
0	0	0	0	0	0	0	0	0	0	0	0	0	0
0	0	0	0	0	0	0	0	0	0	0	0	0	0
0	0	0	0	0	0	0	0	0	0	0	0	0	0
0													

OTU13092 0 0 0 0 1 0 0 0 0 1 0 1 0
0 0 1 1 0 0 0 0 0 0 0 2 1 0
0 0 0 1 0 0 0 0 0 0 0 0 0 0
1 0 1 1 0 0 0 0 0 1 0 0 0 0
0 0 0 0 0 0 0 0 0 0 0 0 0 1
0 0 0 0 0 0 0 0 0 0 0 0 0 0
0 0 0 0 0 0 0 0 1 0 0 0 0 0
0 0 0 0 1 1 0 0 0 0 0 1 0 0
0
OTU8415 0 0 0 0 1 0 0 0 0 0 0 0 0
0 0 0 0 0 0 0 0 0 0 0 0 0 0
0 0 0 0 0 0 0 0 0 0 0 0 0 0
0 0 0 0 0 0 0 0 0 0 0 0 0 0
0 0 0 0 0 0 0 0 0 0 0 0 0 0
0 0 0 0 0 0 0 0 0 0 0 0 0 0
0 0 0 0 0 0 0 0 0 0 0 0 0 0
0 0 0 0 0 0 0 0 0 0 0 0 0 0
0
OTU14640 0 0 0 0 1 0 0 0 0 0 0 0 0
0 0 0 0 0 0 0 0 0 0 0 0 0 0
0 0 0 0 0 0 0 0 0 0 0 0 0 0
0 0 0 0 0 0 0 0 0 0 0 0 0 0
0 0 0 0 0 0 0 0 0 0 0 0 0 0
0 0 0 0 0 0 0 0 0 0 0 0 0 0
0 0 0 0 0 0 0 0 0 0 0 0 0 0
0 0 0 0 0 0 0 0 0 0 0 0 0 0
0
OTU2964 0 0 0 0 1 0 0 0 0 1 0 0 0
0 0 0 0 0 0 0 0 0 0 0 0 0 0
0 0 0 0 0 0 0 0 0 0 0 0 0 0
0 0 0 0 0 0 0 0 0 0 0 0 0 0
0 0 0 0 0 0 0 0 0 0 0 0 0 0
0 0 0 0 0 0 0 0 0 0 0 0 0 1

0	0	0	0	0	0	0	0	0	0	0	0	0	0
0	0	0	0	0	0	0	0	0	0	0	0	0	0
0													
OTU13957	0	0	0	0	2	0	0	0	0	0	1	0	0
0	0	0	0	0	0	0	0	0	1	3	0	0	0
0	3	1	0	0	0	0	0	0	0	0	0	0	0
0	0	0	0	2	0	0	0	1	0	0	0	5	0
0	1	3	0	5	0	4	0	0	3	0	1	0	1
0	0	0	1	0	0	0	0	0	0	0	0	0	0
0	0	1	0	0	0	0	0	0	0	0	0	0	1
0	0	0	0	0	0	0	0	0	0	0	0	0	0
0													
OTU12078	0	0	0	0	1	0	0	0	0	0	0	0	0
0	0	0	0	0	0	0	0	0	0	0	0	0	0
0	0	0	0	0	0	0	0	0	0	0	0	0	0
0	0	0	0	0	0	0	0	0	0	0	0	0	0
0	0	0	0	0	0	0	0	0	0	0	0	0	0
0	0	0	0	0	0	0	0	0	0	0	0	0	0
0	0	0	0	0	0	0	0	0	0	0	0	0	0
0	0	0	0	0	0	0	0	0	0	0	0	0	0
0													
OTU8417	0	0	0	0	1	0	0	0	0	0	0	0	0
0	0	0	0	0	0	0	0	0	0	0	0	0	0
0	0	0	0	0	0	0	0	0	0	0	0	0	0
0	0	0	0	0	0	0	0	0	0	0	0	0	0
0	0	0	0	0	0	0	0	0	0	0	0	0	0
0	0	0	0	0	0	0	0	0	0	0	0	0	0
0	0	0	0	0	0	0	0	0	0	0	0	0	0
0	0	0	0	0	0	0	0	0	0	0	0	0	0
0													
OTU14636	0	0	0	0	1	0	0	0	0	0	0	0	0
0	0	0	0	0	0	0	0	0	0	0	0	0	0
0	0	0	0	0	0	0	0	0	0	0	0	0	0

0	0	0	0	0	0	0	0	0	0	0	0	0	0
0	0	0	0	0	0	0	0	0	0	0	0	0	0
0	0	0	0	0	0	0	0	0	0	0	0	0	0
0	0	0	0	0	0	0	0	0	0	0	0	0	0
0	0	0	0	0	0	0	0	0	0	0	0	0	0
0													
OTU15677	0	0	0	0	1	0	0	0	0	0	0	1	0
0	0	0	0	0	0	0	0	0	0	0	0	0	0
0	0	0	0	0	0	0	0	0	0	0	0	0	0
0	0	0	0	0	0	0	0	0	0	0	0	0	0
0	0	0	0	0	0	0	0	0	0	0	0	0	0
0	0	0	0	0	0	0	0	0	0	0	0	0	0
0	0	0	0	0	0	0	0	0	0	0	0	0	0
0	0	0	0	0	0	0	0	0	0	0	0	0	0
0													
OTU3804	0	0	0	0	1	0	0	0	0	0	0	0	0
0	0	0	0	0	0	0	0	0	0	0	0	0	0
0	0	0	0	0	1	0	0	0	0	0	0	0	0
0	0	0	0	0	0	0	0	0	0	0	0	0	0
0	0	0	0	0	0	0	0	0	0	0	0	0	0
0	0	0	0	0	0	0	0	0	0	0	0	0	0
0	0	0	0	0	1	0	0	0	0	0	0	0	0
0	0	0	0	0	0	0	0	0	0	0	0	0	0
0													
OTU645	0	0	0	0	1	0	0	0	0	0	1	0	0
0	0	1	1	1	0	0	1	1	0	1	0	0	0
0	0	0	0	0	0	0	0	0	2	0	0	0	0
0	2	5	1	0	0	0	0	0	0	0	0	0	1
0	0	0	0	0	0	0	0	0	0	0	2	0	0
0	0	1	1	0	0	0	0	0	0	1	0	0	0
0	0	0	0	0	0	0	0	0	0	0	0	0	0
0	0	0	0	0	0	0	0	0	0	0	0	0	0
0													

OTU14637 0 0 0 0 1 0 0 0 0 0 0 0 0
0 0 0 0 0 0 0 0 0 0 0 0 0 0
0 0 0 0 0 0 0 0 0 0 0 0 0 0
0 0 0 0 0 0 0 0 0 0 0 0 0 0
0 0 0 0 0 0 0 0 0 0 0 0 0 0
0 0 0 0 0 0 0 0 0 0 0 0 0 0
0 0 0 0 0 0 0 0 0 0 0 0 0 0
0 0 0 0 0 0 0 0 0 0 0 0 0 0
0
OTU7880 0 0 0 0 1 0 0 1 2 0 0 0 0
0 0 0 1 0 0 0 0 0 0 0 0 0 0
0 0 0 0 0 0 1 0 0 0 0 0 0 0
0 0 3 0 2 1 0 3 0 0 0 0 0 0
1 0 0 0 1 0 0 0 0 0 0 0 0 0
0 0 0 1 0 0 0 0 0 0 0 0 0 0
0 0 0 0 0 0 1 1 0 0 0 0 1 0
0 0 2 0 0 0 1 0 1 2 1 0 0 0
0
OTU10848 0 0 0 0 1 0 0 0 0 0 0 1 0
0 0 1 0 1 0 0 0 0 1 0 0 0 0
0 0 0 0 0 0 0 0 0 0 1 0 0 0
0 0 0 0 0 1 0 0 0 1 0 0 0 0
0 0 0 1 0 0 0 0 0 0 0 0 0 0
0 0 0 0 0 0 0 0 0 0 0 0 0 0
0 0 0 0 0 0 0 0 0 0 0 0 0 0
0 0 0 0 1 0 0 0 1 0 0 0 0 0
0
OTU941 0 0 0 0 1 0 0 0 0 0 5 1 0
0 0 3 0 0 0 0 1 0 0 0 0 0 1
0 0 0 1 0 0 4 1 0 0 0 0 0 0
0 1 0 0 4 4 0 1 0 0 0 0 0 0
0 1 0 0 1 0 0 1 0 0 0 1 0 0
1 0 0 0 0 0 0 0 0 0 0 0 0 0

0	0	0	0	1	0	0	0	0	0	0	0	0	1
5	0	0	0	0	0	0	0	0	0	0	0	0	0
0													
OTU11676	0	0	0	0	1	0	0	0	0	0	0	1	0
0	1	0	1	0	1	0	0	0	0	0	1	0	0
0	0	0	0	0	1	0	0	0	0	0	0	1	1
0	0	0	0	0	0	0	0	0	0	0	0	0	0
0	0	0	0	0	0	0	0	0	0	0	0	0	0
1	0	0	0	0	1	0	0	0	0	0	2	0	0
0	0	0	0	0	1	0	0	0	0	0	0	0	0
0	0	0	0	0	0	0	1	1	0	0	0	0	0
0													
OTU8414	0	0	0	0	1	0	0	0	0	0	0	0	0
0	0	0	0	0	0	0	0	0	0	0	0	0	0
0	0	0	0	0	0	0	0	0	0	0	0	0	0
0	0	0	0	0	0	0	0	0	0	0	0	0	0
0	0	0	0	0	0	0	0	0	0	0	0	0	0
0	0	0	0	0	0	0	0	0	0	0	0	0	0
0	0	0	0	0	0	0	0	0	0	0	0	0	0
0	0	0	0	0	0	0	0	0	0	0	0	0	0
0													
OTU8413	0	0	0	0	1	0	0	0	0	0	0	0	0
0	0	0	0	0	0	0	0	0	0	0	0	0	0
0	0	0	0	0	0	0	0	0	0	0	0	0	0
0	0	0	0	0	0	0	0	0	0	0	0	0	0
0	0	0	0	0	0	0	0	0	0	0	0	0	0
0	0	0	0	0	0	0	0	0	0	0	0	0	0
0	0	0	0	0	0	0	0	0	0	0	0	0	0
0	0	0	0	0	0	0	0	0	0	0	0	0	0
0													
OTU12077	0	0	0	0	1	0	0	0	0	0	0	0	0
0	0	0	0	0	0	0	0	0	0	0	0	0	0
0	0	0	0	0	0	0	0	0	0	0	0	0	0

0	0	0	0	0	0	0	0	0	0	0	0	0	0
0	0	0	0	0	0	0	0	0	0	0	0	0	0
0	0	0	0	0	0	0	0	0	0	0	0	0	0
0	0	0	0	0	0	0	0	0	0	0	0	0	0
0	0	0	0	0	0	0	0	0	0	0	0	0	0
0													
OTU10849	0	0	0	0	1	0	0	0	0	0	0	0	0
0	0	0	0	0	0	0	0	0	0	0	0	0	0
0	0	0	0	0	0	0	0	0	0	0	0	0	0
0	0	0	0	0	0	0	0	0	0	0	0	0	0
0	0	0	0	0	0	0	0	0	0	0	0	0	0
0	0	0	0	0	0	0	0	0	0	0	0	0	0
0	0	0	0	0	0	0	0	0	0	0	0	0	0
0	0	0	0	0	0	0	0	0	0	0	0	0	0
0													
OTU15224	0	0	0	0	2	0	0	0	0	0	0	0	0
0	1	0	0	0	0	0	2	0	0	0	0	0	0
1	0	0	0	0	0	1	1	0	0	0	0	0	0
0	1	0	0	0	0	0	0	0	0	0	0	0	0
0	0	0	0	0	0	0	0	0	0	0	0	0	0
0	0	0	2	0	0	1	0	1	0	0	0	0	0
0	0	0	0	0	0	0	0	0	0	0	0	0	0
0	0	0	0	0	0	0	0	0	0	1	1	0	0
0													
OTU10850	0	0	0	0	1	0	0	0	0	0	0	0	0
0	0	0	0	0	0	0	0	0	0	0	0	0	0
0	0	0	0	0	0	0	0	0	0	0	0	0	0
0	0	0	0	0	0	0	0	0	0	0	0	0	0
0	0	0	0	0	0	0	0	0	0	0	0	0	0
0	0	0	0	0	0	0	0	0	0	0	0	0	0
0	0	0	0	0	0	0	0	0	0	0	0	0	0
0	0	0	0	0	0	0	0	0	0	0	0	0	0
0													

OTU15875	0	0	0	0	1	0	0	0	0	0	0	0	0
0	0	0	1	0	0	0	0	0	0	0	0	0	0
0	1	1	0	0	0	0	0	0	0	0	0	0	0
0	1	0	0	1	0	0	0	0	0	0	0	2	0
1	1	0	0	0	0	0	0	0	1	0	0	0	0
0	0	0	0	0	0	0	0	0	0	0	0	0	0
0	0	1	0	0	0	0	0	0	0	0	0	0	0
0	0	0	0	0	0	0	0	0	0	0	0	0	0
0													
OTU7424	0	0	0	0	2	0	0	0	0	0	0	0	0
0	0	0	0	0	0	0	0	0	0	0	0	0	0
0	0	1	1	0	0	0	0	0	0	0	0	0	0
0	0	0	0	1	0	0	0	0	0	0	0	0	0
0	0	0	1	0	0	0	0	0	0	0	0	0	0
0	0	0	0	0	0	0	0	0	0	0	0	0	1
0	0	1	0	1	0	0	0	0	0	0	0	0	0
0	0	0	0	0	0	0	0	0	0	0	0	0	0
0													
OTU13312	0	0	0	0	1	0	0	1	1	0	0	0	0
0	1	0	0	0	0	0	1	0	0	0	0	0	0
0	0	1	0	0	0	1	0	1	0	0	1	0	0
0	2	0	0	0	0	0	0	0	0	0	0	0	0
0	0	0	0	0	0	0	0	0	0	0	0	0	0
0	0	1	1	0	0	0	0	0	0	0	0	1	0
0	0	0	0	0	0	0	1	1	0	0	0	0	0
0	0	0	0	0	1	0	0	1	1	0	0	0	0
0													
OTU8412	0	0	0	0	1	0	0	0	0	0	0	0	0
0	0	0	0	0	0	0	0	0	0	0	0	0	0
0	0	0	0	0	0	0	0	0	0	0	0	0	0
0	0	0	0	0	0	0	0	0	0	0	0	0	0
0	0	0	0	0	0	0	0	0	0	0	0	0	0
0	0	0	0	0	0	0	0	0	0	0	0	0	0

0	0	0	0	0	0	0	0	0	0	0	0	0	0
0	0	0	0	0	0	0	0	0	0	0	0	0	0
0													
OTU10852	0	0	0	0	1	0	0	0	0	0	0	0	0
0	0	0	0	0	0	0	0	0	0	0	0	0	0
0	0	0	0	0	0	0	0	0	0	0	0	0	0
0	0	0	0	0	0	0	0	0	0	0	0	0	0
0	0	0	0	0	0	0	0	0	0	0	0	0	0
0	0	0	0	0	0	0	0	0	0	0	0	0	0
0	0	0	0	0	0	0	0	0	0	0	0	0	0
0	0	0	0	0	0	0	0	0	0	0	0	0	0
0													
OTU3937	0	0	0	0	1	1	0	0	0	2	0	0	1
0	0	0	0	0	0	0	0	1	0	0	0	0	0
1	0	1	0	0	0	0	0	0	0	0	0	0	1
0	0	1	0	0	0	0	0	0	0	0	0	0	0
0	0	1	1	0	0	0	1	0	0	0	1	0	0
0	0	0	0	1	2	0	0	1	1	0	0	0	1
0	0	0	1	1	0	0	0	0	0	0	0	0	0
0	1	0	0	0	0	1	0	0	0	1	0	1	0
0													
OTU9334	0	0	0	0	1	0	0	0	0	1	0	0	0
0	1	0	0	0	0	0	5	0	0	0	0	0	0
3	1	0	0	0	0	0	0	0	4	0	0	0	0
0	0	0	0	0	0	0	0	0	0	0	0	1	0
0	0	0	0	0	0	0	0	0	0	0	0	1	0
1	0	0	0	0	0	0	0	0	13	0	18	0	0
0	0	0	3	0	6	0	0	0	0	0	0	0	0
0	0	0	0	0	0	0	0	0	0	0	0	0	0
0													
OTU4489	0	0	0	0	1	0	0	0	0	2	0	0	0
0	0	1	0	0	0	0	0	0	0	1	0	2	0
1	0	0	0	0	0	0	0	0	0	0	0	1	0

0 0 0 0 0 0 0 0 0 0 0 0 0 0
0 0 0 0 0 0 0 0 0 0 0 0 0 0
0 0 0 0 1 0 0 0 0 0 0 0 0 0
0 0 0 0 0 1 0 0 0 0 0 0 0 0
0 0 0 0 0 0 0 0 0 0 0 0 0 0
0
OTU910 0 0 0 0 1 0 0 0 0 0 0 0 0
0 0 0 0 0 0 0 0 0 0 0 0 0 0
0 0 0 0 0 0 1 0 0 0 0 0 0 0
0 0 0 0 0 0 0 0 1 0 0 0 0 0
0 0 0 0 0 0 0 0 0 0 0 0 0 0
0 0 0 0 0 0 0 0 0 0 0 0 0 0
0 0 0 0 0 0 0 0 0 0 0 0 0 0
0 0 0 0 0 0 0 0 0 2 0 0 0 0
0
OTU1380 0 0 0 0 1 0 0 0 1 0 0 0 0
0 0 0 0 0 0 0 0 0 0 0 0 0 0
0 0 0 0 0 0 0 0 0 0 0 0 1 0
1 0 0 0 0 0 0 0 0 0 0 0 0 0
0 0 0 0 0 0 0 0 0 0 0 0 0 0
0 0 0 0 0 0 0 0 0 0 1 0 0 0
0 0 0 0 0 0 0 0 0 0 0 0 0 0
0 0 0 0 3 0 0 0 0 0 0 0 0 0
0
OTU10851 0 0 0 0 1 0 0 0 0 0 0 0 0
0 0 0 0 0 0 0 0 0 0 0 0 0 0
0 0 0 0 0 0 0 0 0 0 0 0 0 0
0 0 0 0 0 0 0 0 0 0 0 0 0 0
0 0 0 0 0 0 0 0 0 0 0 0 0 0
0 0 0 0 0 0 0 0 0 0 0 0 0 0
0 0 0 0 0 0 0 0 0 0 0 0 0 0
0 0 0 0 0 0 0 0 0 0 0 0 0 0
0

OTU8420 0 0 0 0 1 0 0 0 0 0 0 0 0

0 0 0 0 0 0 1 0 0 0 0 0 0 0

0 0 0 0 0 0 0 0 0 0 0 0 0 0

0 0 0 0 0 0 0 0 0 0 0 0 0 0

0 0 0 0 0 0 0 0 0 0 0 0 0 0

0 0 0 0 0 0 0 0 0 0 0 0 0 0

0 0 0 0 0 0 0 0 0 0 0 0 0 0

0 0 0 0 0 0 0 0 0 0 0 0 0 0

0

OTU14643 0 0 0 0 1 0 0 0 0 0 0 0 0

0 0 0 0 0 0 0 0 0 0 0 0 0 0

0 0 0 0 0 0 0 0 0 0 0 0 0 0

0 0 0 0 0 0 0 0 0 0 0 0 0 0

0 0 0 0 0 0 0 0 0 0 0 0 0 0

0 0 0 0 0 0 0 0 0 0 0 0 0 0

0 0 0 0 0 0 0 0 0 0 0 0 0 0

0 0 0 0 0 0 0 0 0 0 0 0 0 0

0

OTU7782 0 0 0 0 2 0 1 0 1 0 0 0 0

0 0 0 0 0 0 0 0 0 0 0 0 0 0

0 0 0 0 0 0 0 0 0 0 0 0 0 0

0 0 0 0 0 0 0 0 0 0 0 0 0 0

0 0 0 0 0 0 0 0 0 0 0 0 0 0

0 0 0 0 0 0 0 0 0 0 0 0 0 0

0 2 0 0 1 0 0 0 0 0 0 0 0 0

0 0 0 0 0 0 0 0 0 0 0 0 1 0

0

OTU12064 0 0 0 0 1 0 0 0 0 0 0 0 0

0 0 0 0 0 0 0 0 0 0 0 0 0 0

0 0 0 0 0 0 0 0 0 0 0 0 0 0

0 0 0 0 0 0 0 0 0 0 0 0 0 0

0 0 0 0 0 0 0 0 0 0 0 0 0 0

0 0 0 0 0 0 0 0 0 0 0 0 0 0

0	0	0	0	0	0	0	0	0	0	0	0	0	0
0	0	0	0	0	0	0	0	0	0	0	0	0	0
0													
OTU10885	0	0	0	0	1	0	0	0	0	0	0	0	0
0	0	0	0	0	0	0	0	0	0	0	0	0	0
0	0	0	0	0	0	0	0	0	0	0	0	0	0
0	0	0	0	0	0	0	0	0	0	0	0	0	0
0	0	0	0	0	0	0	0	0	0	0	0	0	0
0	0	0	0	0	0	0	0	0	0	0	0	0	0
0	0	0	0	0	0	0	0	0	0	0	0	0	0
0	0	0	0	0	0	0	0	0	0	0	0	0	0
0													
OTU3780	0	0	0	0	1	0	0	0	0	0	0	0	0
0	0	0	0	0	0	0	0	0	1	0	0	0	0
0	0	1	0	0	0	0	0	0	0	0	0	0	0
0	0	0	0	0	0	0	0	1	0	0	0	0	0
0	0	0	0	0	0	0	0	0	0	0	0	1	0
0	0	0	0	0	0	0	0	0	0	0	0	0	0
0	0	0	0	0	0	0	0	0	0	0	0	0	0
0	0	1	0	0	0	0	0	0	0	0	0	0	0
1													
OTU10884	0	0	0	0	1	0	0	0	0	0	0	0	0
0	0	0	0	0	0	0	0	0	0	1	0	0	0
0	0	0	0	0	0	0	0	0	0	0	0	0	0
0	0	0	0	0	0	0	0	0	0	0	0	0	0
0	0	0	0	0	0	0	0	0	0	0	0	0	0
0	0	0	0	0	0	0	0	0	0	0	0	0	0
0	0	0	0	0	0	0	0	0	0	0	0	0	0
0	0	0	0	0	0	0	0	0	0	0	0	0	0
0													
OTU12062	0	0	0	0	1	0	0	0	0	0	0	0	0
0	0	0	0	0	0	0	0	0	0	0	0	0	0
0	0	0	0	0	0	0	0	0	0	0	0	0	0

0	0	0	0	0	0	0	0	0	0	0	0	0	0
0	0	0	0	0	0	0	0	0	0	0	0	0	0
0	0	0	0	0	0	0	0	0	0	0	0	0	0
0	0	0	0	0	0	0	0	0	0	0	0	0	0
0	0	0	0	0	0	0	0	0	0	0	0	0	0
0													
OTU10063	0	0	0	0	1	0	0	0	0	0	0	0	0
0	0	0	0	0	0	0	0	0	0	1	0	0	0
0	0	0	0	0	0	0	0	0	0	0	0	0	0
0	0	0	0	0	0	0	0	1	0	0	0	1	0
1	0	0	0	0	0	0	0	0	1	0	0	0	0
0	0	0	0	0	0	0	0	0	0	0	0	0	0
0	0	0	0	0	0	0	0	0	0	0	0	0	0
0	0	0	0	0	0	0	0	0	0	0	0	0	0
0													
OTU12063	0	0	0	0	1	0	0	0	0	0	0	0	0
0	0	0	0	0	0	0	0	0	0	0	0	0	0
0	0	0	0	0	0	0	0	0	0	0	0	0	0
0	0	0	0	0	0	0	0	0	0	0	0	0	0
0	0	0	0	0	0	0	0	0	0	0	0	0	0
0	0	0	0	0	0	0	0	0	0	0	1	0	0
0	0	0	0	0	0	0	0	0	0	0	0	0	0
0	0	0	0	0	0	0	0	0	0	0	0	0	0
0													
OTU10882	0	0	0	0	1	0	0	0	0	0	0	0	0
0	0	0	0	0	0	0	0	0	0	0	0	0	0
0	0	0	0	0	0	0	0	0	0	0	0	0	0
0	0	0	0	0	0	0	0	0	0	0	0	0	0
0	0	0	0	0	0	0	0	0	0	0	0	0	0
0	0	0	0	0	0	0	0	0	0	0	0	0	0
0	0	0	0	0	0	0	0	0	0	0	0	0	0
0	0	0	0	0	0	0	0	0	0	0	0	0	0
0													

OTU14644 0 0 0 0 1 0 0 0 0 0 0 0 0
0 0 0 0 1 0 0 0 0 2 1 0 0 0
0 0 0 0 0 1 0 0 0 0 0 0 0 0
0 0 0 0 0 0 0 0 0 0 2 0 0 0
0 0 0 0 0 0 1 0 0 1 0 0 0 3
0 0 1 0 0 0 0 0 0 0 0 0 0 0
0 0 0 0 0 0 0 0 0 0 0 1 0 0
0 0 0 0 0 0 0 0 0 0 0 0 0 0
0
OTU10883 0 0 0 0 1 0 0 0 0 0 0 0 0
0 0 0 0 0 0 0 0 0 0 0 0 0 0
0 0 0 0 0 0 0 0 0 0 0 0 0 0
0 0 0 0 0 0 0 0 0 0 0 0 0 0
0 0 0 0 0 0 0 0 0 0 0 0 0 0
0 0 0 0 0 0 0 0 0 0 0 0 0 0
0 0 0 0 0 0 0 0 0 0 0 0 0 0
0 0 0 0 0 0 0 0 0 0 0 0 0 0
0
OTU13480 0 0 0 0 1 0 1 0 0 0 0 0 0
0 1 0 0 0 0 1 0 0 1 0 0 0 1
0 0 0 0 0 1 0 0 3 0 0 0 0 2
0 0 0 0 0 0 0 1 0 0 0 0 0 0
0 0 0 0 0 0 0 0 0 0 0 0 0 0
0 0 0 4 0 1 0 0 2 0 0 0 0 0
1 0 1 0 0 0 1 0 1 0 0 0 0 1
1 0 0 0 0 0 0 0 0 0 0 3 2 0
0
OTU14642 0 0 0 0 1 0 0 0 0 0 0 0 0
0 0 0 0 0 0 0 0 0 0 0 0 0 0
0 0 0 0 0 0 0 0 0 0 0 0 0 0
0 0 0 0 0 0 0 0 0 0 0 0 0 0
0 0 0 0 0 0 0 0 0 0 0 0 0 0
0 0 0 0 0 0 0 0 0 0 0 0 0 0

0	0	0	0	0	0	0	0	0	0	0	0	0	0
0	0	0	0	0	0	0	0	0	0	0	0	0	0
0													
OTU10888	0	0	0	0	1	0	0	0	0	0	0	0	0
0	0	0	0	0	0	0	0	0	0	0	0	0	0
0	0	0	0	0	0	0	0	0	0	0	0	0	0
0	0	0	0	0	0	0	0	0	0	0	0	0	0
0	0	0	0	0	0	0	0	0	0	0	0	0	0
0	0	0	0	0	0	0	0	0	0	0	0	0	0
0	0	0	0	0	0	0	0	0	0	0	0	0	0
0	0	0	0	0	0	0	0	0	0	0	0	0	0
0													
OTU8419	0	0	0	0	1	0	0	0	0	0	0	0	0
0	0	0	0	0	0	0	0	0	0	0	0	0	0
0	0	0	0	0	0	0	0	0	0	0	0	0	0
0	0	0	0	0	0	0	0	0	0	0	0	0	0
0	0	0	0	0	0	0	0	0	0	0	0	0	0
0	0	0	0	0	0	0	0	0	0	0	0	0	0
0	0	0	0	0	0	0	0	0	0	0	0	0	0
0	0	0	0	0	0	0	0	0	0	0	0	0	0
0													
OTU14477	0	0	0	0	1	0	3	0	0	0	0	0	0
0	0	0	0	0	0	0	0	0	0	0	0	0	0
1	0	0	0	0	0	0	0	0	0	0	0	0	0
0	0	1	0	0	0	0	0	0	0	0	0	0	0
0	0	0	0	0	0	0	1	0	0	0	0	0	0
0	0	0	0	0	0	0	0	0	0	0	1	0	0
0	0	0	1	0	0	0	0	0	0	0	0	0	0
0	0	0	0	0	0	0	0	0	0	0	0	0	0
0													
OTU3404	0	0	0	0	1	0	0	1	1	0	1	1	0
1	4	0	0	1	0	0	0	0	0	0	0	0	0
0	1	1	0	1	0	0	0	0	0	1	0	1	1

0 0 0 2 0 1 0 0 0 2 0 0 0 0
0 0 0 0 0 0 0 1 0 0 0 2 1 1
2 0 0 0 1 0 0 1 0 0 0 1 0 0
0 1 0 0 0 0 0 0 0 0 2 0 0 0
0 3 0 0 1 0 0 0 0 0 0 0 0 0
3
OTU10887 0 0 0 0 1 0 0 0 0 0 0 0 0
0 0 0 0 0 0 0 0 0 0 0 0 0 0
0 0 0 0 0 0 0 0 0 0 0 0 0 0
0 0 0 0 0 0 0 0 0 0 0 0 0 0
0 0 0 0 0 0 0 0 0 0 0 0 0 0
0 0 0 0 0 0 0 0 0 0 0 0 0 0
0 0 0 0 0 0 0 0 0 0 0 0 0 0
0 0 0 0 0 0 0 0 0 0 0 0 0 0
0
OTU12065 0 0 0 0 1 0 0 0 0 0 0 0 0
0 0 0 0 0 0 0 0 0 0 0 0 0 0
0 0 0 0 0 0 0 0 0 0 0 0 0 0
0 0 0 0 0 0 0 0 0 0 0 0 0 0
0 0 0 0 0 0 0 0 0 0 0 0 0 0
0 0 0 0 0 0 0 0 0 0 0 0 0 0
0 0 0 0 0 0 0 0 0 0 0 0 0 0
0 0 0 0 0 0 0 0 0 0 0 0 0 0
0
OTU14641 0 0 0 0 1 0 0 0 0 0 0 0 0
0 0 0 0 0 0 0 0 0 0 0 0 0 0
0 0 0 0 0 0 0 0 0 0 0 0 0 0
0 0 0 0 0 0 0 0 0 0 0 0 0 0
0 0 0 0 0 0 0 0 0 0 0 0 0 0
0 0 0 0 0 0 0 0 0 0 0 0 0 0
0 0 0 0 0 0 0 0 0 0 0 0 0 0
0 0 0 0 0 0 0 0 0 0 0 0 0 0
0

OTU10886	0	0	0	0	1	0	0	0	0	0	0	0	0
0	0	0	0	0	0	0	0	0	0	0	0	0	0
0	0	0	0	0	0	0	0	0	0	0	0	0	0
0	0	0	0	0	0	0	0	0	0	0	0	0	0
0	0	0	0	0	0	0	0	0	0	0	0	0	0
0	0	0	0	0	0	0	0	0	0	0	0	0	0
0	0	0	0	0	0	0	0	0	0	0	0	0	0
0	0	0	0	0	0	0	0	0	0	0	0	0	0
0													
OTU8442	0	0	0	0	1	0	0	0	0	0	0	0	0
0	0	0	0	0	0	0	0	0	0	0	0	0	0
0	0	0	0	0	0	0	0	0	0	0	0	0	0
0	0	0	0	0	0	0	0	0	0	0	0	0	0
0	0	0	0	0	0	0	0	0	0	0	0	0	0
0	0	0	0	0	0	0	0	0	0	0	0	0	0
0	0	0	0	0	0	0	0	0	0	0	0	0	0
0	0	0	0	0	0	0	0	0	0	0	0	0	0
0													
OTU13012	0	0	0	0	1	0	0	0	0	0	0	0	0
0	0	0	0	0	0	0	0	0	0	0	0	0	0
0	0	0	1	0	0	0	0	0	1	0	0	0	0
0	0	0	0	0	0	0	0	0	0	0	0	0	0
0	0	0	0	0	0	0	0	0	0	0	0	0	0
0	0	0	0	0	0	0	0	0	0	0	0	0	0
0	0	0	0	0	0	0	0	0	0	0	0	0	0
0	0	0	0	0	0	0	0	0	0	0	0	0	0
0													
OTU8440	0	0	0	0	1	0	0	0	0	0	0	0	0
0	1	0	0	1	0	0	0	0	0	0	0	0	0
0	0	0	0	1	0	0	0	0	0	0	0	0	0
0	0	0	0	0	0	0	0	0	0	0	0	0	1
0	0	0	0	0	0	0	0	0	1	0	0	0	0
1	0	0	0	0	0	0	0	0	0	0	0	1	0

0 0 1 0 0 0 0 0 0 0 0 0 1 0

0 1 0 0 0 1 0 0 0 0 0 0 0 0

1

OTU15321 0 0 0 0 1 0 0 0 0 0 0 0 0

0 0 0 0 0 0 0 0 0 0 1 0 0 0

0 0 0 0 0 0 0 0 0 0 0 0 0 0

0 0 0 0 0 0 2 0 0 0 0 0 2 0

0 0 0 0 1 0 1 0 0 0 0 0 0 0

0 0 0 0 0 0 0 0 0 0 0 0 0 0

0 0 0 0 0 0 0 0 0 0 0 0 0 0

0 0 0 0 0 0 0 0 0 0 0 0 0 0

0

OTU8441 0 0 0 0 1 0 0 0 0 0 0 0 0

0 0 0 0 0 0 0 0 0 0 0 0 0 0

0 0 0 0 0 0 0 0 0 0 0 0 0 0

0 0 0 0 0 0 0 0 0 0 0 0 0 0

0 0 0 0 0 0 0 0 0 0 0 0 0 0

0 0 0 0 0 0 0 0 0 0 0 0 0 0

0 0 0 0 0 0 0 0 0 0 0 0 0 0

0 0 0 0 0 0 0 0 0 0 0 0 0 0

0

OTU12060 0 0 0 0 1 0 0 0 0 0 0 0 0

0 0 0 0 0 0 0 0 0 0 0 0 0 0

0 0 0 0 0 0 0 0 0 0 0 0 0 0

1 0 0 0 0 1 0 1 0 0 0 0 0 0

0 0 0 0 0 0 0 0 0 0 0 0 0 0

0 0 0 0 0 0 0 0 0 0 0 0 0 0

0 0 0 0 0 0 0 0 0 0 0 0 0 0

0 0 0 0 0 0 0 0 0 0 1 0 0 0

0

OTU10361 0 0 0 0 2 0 0 0 0 1 0 0 0

0 1 0 0 0 0 0 0 0 0 0 0 0 0

0 0 0 1 0 0 1 0 0 0 0 1 2 0

	0	0	0	1	0	0	0	0	0	0	0	0	0	0
	0	0	1	0	0	0	0	0	0	0	0	1	1	0
	1	0	1	0	0	0	0	1	0	0	0	0	0	0
	0	0	0	0	0	1	0	1	0	0	0	0	0	0
	0	0	0	0	0	0	0	0	0	0	0	0	0	1
	4													
OTU1973	0	0	0	0	1	0	0	0	0	0	0	0	0	
	0	0	0	4	0	0	0	0	1	0	0	0	0	0
	0	1	0	0	1	0	0	0	0	0	0	0	0	0
	0	0	0	0	0	2	0	0	1	0	0	0	4	0
	5	0	0	0	0	0	9	0	0	0	0	0	0	0
	0	0	0	0	0	0	0	0	0	0	1	0	0	0
	0	0	0	0	0	0	0	0	0	0	0	0	0	7
	0	0	0	0	0	0	0	0	0	0	0	0	0	0
	0													
OTU12518	0	0	0	0	1	0	0	0	0	1	0	0	0	
	0	0	1	0	0	0	0	0	0	0	0	0	0	0
	0	0	0	0	0	0	0	0	0	0	0	0	0	0
	0	0	0	0	0	0	0	0	0	0	0	0	0	0
	0	0	0	0	0	0	0	0	0	0	0	1	0	0
	1	0	0	0	0	0	0	0	0	0	0	0	0	0
	0	0	0	0	0	0	0	0	0	1	0	0	0	0
	0	0	0	0	0	0	0	0	0	0	0	0	0	0
	1													
OTU7590	0	0	0	0	1	0	0	0	0	0	0	0	0	
	1	0	0	0	0	0	0	0	0	0	0	0	0	0
	0	0	0	0	0	0	0	0	0	0	0	0	0	0
	0	0	0	0	0	0	0	0	0	0	0	0	0	0
	0	0	0	0	0	0	0	0	0	0	0	0	0	0
	0	0	0	0	0	0	1	0	0	0	0	0	1	0
	0	0	0	0	0	0	0	0	1	0	0	0	0	0
	0	0	0	0	0	0	0	0	0	0	0	0	0	0
	0													

OTU14629	0	0	0	0	1	0	0	0	0	0	0	0	0
0	0	0	0	0	0	0	0	0	0	0	0	0	0
0	0	0	0	0	0	0	0	0	0	0	0	0	0
0	0	0	0	0	0	0	0	0	0	0	0	0	0
0	0	0	0	0	0	0	0	0	0	0	0	0	0
0	0	0	0	0	0	0	0	0	0	0	0	0	0
0	0	0	0	0	0	0	0	0	0	0	0	0	0
0	0	0	0	0	0	0	0	0	0	0	0	0	0
0													
OTU8443	0	0	0	0	1	0	0	0	0	0	0	0	0
0	0	0	0	0	0	0	0	0	0	0	0	0	0
0	0	0	0	0	0	0	0	0	0	0	0	0	0
0	0	0	0	0	0	0	0	0	0	0	0	0	0
0	0	0	0	0	0	0	0	0	0	0	0	0	0
0	0	0	0	0	0	0	0	0	0	0	0	0	0
0	0	0	0	0	0	0	0	0	0	0	0	0	0
0	0	0	0	0	0	0	0	0	0	0	0	0	0
0													
OTU10879	0	0	0	0	1	1	0	0	0	0	0	0	0
0	0	0	0	0	0	0	0	0	0	0	0	0	0
0	0	0	0	0	0	0	0	0	0	0	0	0	0
0	0	0	0	0	0	0	0	0	0	0	0	0	0
0	0	0	0	0	0	0	0	0	0	0	0	0	0
0	0	0	0	0	0	0	0	0	0	0	0	0	0
0	0	0	0	0	0	0	0	0	0	0	0	0	0
0	0	0	0	0	0	0	0	0	0	0	0	0	0
0													
OTU14630	0	0	0	0	1	0	0	0	0	0	0	0	0
0	0	0	0	0	0	0	0	0	0	0	0	0	0
0	0	0	0	0	0	0	0	0	0	0	0	0	0
0	0	0	0	0	0	0	0	0	0	0	0	0	0
0	0	0	0	0	0	0	0	0	0	0	0	0	0
0	0	0	0	0	0	0	0	0	0	0	0	0	0

0	0	0	0	0	0	0	0	0	0	0	0	0	0
0	0	0	0	0	0	0	0	0	0	0	0	0	0
0													
OTU10881	0	0	0	0	1	0	0	0	0	0	0	0	0
0	0	0	0	0	0	0	0	0	0	0	0	0	0
0	0	0	0	0	0	0	0	0	0	0	0	0	0
0	0	0	0	0	0	0	0	0	0	0	0	0	0
0	0	0	0	0	0	0	0	0	0	0	0	0	0
0	0	0	0	0	0	0	0	0	0	0	0	0	0
0	0	0	0	0	0	0	0	0	0	0	0	0	0
0	0	0	0	0	0	0	0	0	0	0	0	0	0
0													
OTU12061	0	0	0	0	1	0	0	0	0	0	0	0	0
0	0	0	0	0	0	0	0	0	0	0	0	0	0
0	0	0	0	0	0	0	0	0	0	0	0	0	0
0	0	0	0	0	0	0	0	0	0	0	0	0	0
0	0	0	0	0	0	0	0	0	0	0	0	0	0
0	0	0	0	0	0	0	0	0	0	0	0	0	0
0	0	0	0	0	0	0	0	0	0	0	0	0	0
0	0	0	0	0	0	0	0	0	0	0	0	0	0
0													
OTU10880	0	0	0	0	1	0	0	0	0	0	0	0	0
0	0	0	0	0	0	0	0	0	0	0	0	0	0
0	0	0	0	0	0	0	0	0	0	0	0	0	0
0	0	0	0	0	0	0	0	0	0	0	0	0	0
0	0	0	0	0	0	0	0	0	0	0	0	0	0
0	0	0	0	0	0	0	0	0	0	0	0	0	0
0	0	0	0	0	0	0	0	0	0	0	0	0	0
0	0	0	0	0	0	0	0	0	0	0	0	0	0
0													
OTU6205	0	0	0	0	1	0	0	0	0	0	0	0	0
0	0	0	0	0	1	0	0	0	0	0	1	0	0
0	0	0	0	0	0	0	0	0	0	0	1	0	0

0	0	0	0	0	0	0	0	1	0	0	0	0	0
0	0	0	0	0	0	0	0	0	0	0	0	0	0
0	0	0	0	0	0	0	0	0	0	0	0	0	0
0	0	0	0	0	0	0	0	0	0	0	0	0	0
0	0	0	0	0	0	0	0	0	0	0	0	0	0
0													
OTU14628	0	0	0	0	1	0	0	0	0	0	0	0	0
0	0	0	0	0	0	0	0	0	0	0	0	0	0
0	0	0	0	0	0	0	0	0	0	0	0	0	0
0	0	0	0	0	0	0	0	0	0	0	0	0	0
0	0	0	0	0	0	0	0	0	0	0	0	0	0
0	0	0	0	0	0	0	0	0	0	0	0	0	0
0	0	0	0	0	0	0	0	0	0	0	0	0	0
0	0	0	0	0	0	0	0	0	0	0	0	0	0
0													
OTU14627	0	0	0	0	1	0	0	0	0	0	0	0	0
0	0	0	0	0	0	0	0	0	0	0	0	0	0
0	0	0	0	0	0	0	0	0	0	0	0	0	0
0	0	0	0	0	0	0	0	0	0	0	0	0	1
0	0	0	0	0	0	0	0	0	0	0	0	0	0
0	0	0	0	0	0	0	0	0	0	0	0	0	0
0	0	0	0	0	0	0	0	0	0	0	0	0	0
0	0	0	0	0	0	0	0	0	0	0	0	0	0
0													
OTU4340	0	0	0	0	1	0	0	0	0	0	0	0	0
0	0	0	0	0	1	0	0	0	0	0	0	0	0
0	0	1	0	0	0	0	0	0	0	0	0	0	0
0	0	0	1	0	0	0	0	0	0	0	0	0	0
0	0	0	0	0	0	0	0	0	0	0	0	0	0
0	0	0	1	0	0	0	0	0	0	0	0	0	0
0	0	1	0	0	0	0	0	0	0	0	0	0	0
0	0	0	0	0	0	0	0	0	0	0	0	0	0
0													

OTU12070	0	0	0	0	1	0	0	0	0	0	0	0	0
0	0	0	0	0	0	0	0	0	0	0	1	0	0
0	0	0	0	0	0	0	0	0	0	0	0	0	0
0	0	0	0	0	0	0	1	0	1	0	0	0	0
0	0	0	1	0	0	0	0	0	0	0	0	0	0
0	0	0	0	0	0	0	0	0	0	0	0	0	0
0	0	0	0	0	0	0	0	0	0	0	0	0	0
0	0	0	0	0	0	0	0	0	0	0	0	0	0
0													
OTU12069	0	0	0	0	1	0	0	0	0	0	0	0	0
0	0	0	0	0	0	0	0	0	0	0	0	0	0
0	0	0	0	0	0	0	0	0	0	0	0	0	0
0	0	0	0	0	0	0	0	0	0	0	0	0	0
0	0	0	0	0	0	0	0	0	0	0	0	0	0
0	0	0	0	0	0	0	0	0	0	0	0	0	0
0	0	0	0	0	0	0	0	0	0	0	0	0	0
0	0	0	0	0	0	0	0	0	0	0	0	0	0
0													
OTU14632	0	0	0	0	1	0	0	0	0	0	0	0	0
0	0	0	0	0	0	0	0	0	0	0	0	0	0
0	0	0	0	0	0	0	0	0	0	0	0	0	0
0	0	0	0	0	0	0	0	0	0	0	0	0	0
0	0	0	0	0	0	0	0	0	0	0	0	0	0
0	0	0	0	0	0	0	0	0	0	0	0	0	0
0	0	0	0	0	0	0	0	0	0	0	0	0	0
0	0	0	0	0	0	0	0	0	0	0	0	0	0
0													
OTU13145	0	0	0	0	1	0	0	0	0	0	1	0	0
0	0	0	0	0	0	0	0	0	0	0	0	0	0
0	0	0	0	0	1	1	0	0	0	0	0	0	0
0	0	0	0	0	0	0	0	0	0	0	0	0	0
0	0	1	0	0	0	0	0	0	0	0	0	0	0
0	0	0	0	0	0	0	0	0	0	0	0	0	0

0	0	0	0	0	0	0	0	0	0	1	0	0	0
0	0	0	0	0	0	0	0	0	0	0	0	0	0
0													
OTU10891	0	0	0	0	1	0	0	0	0	0	0	0	0
0	0	0	0	0	0	0	0	0	0	0	0	0	0
0	0	0	0	0	0	0	0	0	0	0	0	0	0
0	0	0	0	0	0	0	0	0	0	0	0	0	0
0	0	0	0	0	0	0	0	0	0	0	0	0	0
0	0	0	0	0	0	0	0	0	0	0	0	0	0
0	0	0	0	0	0	0	0	0	0	0	0	0	0
0	0	0	0	0	0	0	0	0	0	0	0	0	0
0													
OTU16802	0	0	0	0	1	0	0	0	0	0	0	0	0
0	0	0	0	0	0	0	0	0	0	0	0	0	1
0	0	0	0	0	0	0	0	0	0	0	0	0	0
0	0	0	0	1	0	0	0	0	0	0	0	0	0
0	0	0	0	0	0	0	0	0	0	0	0	0	0
0	0	0	0	0	0	0	0	0	0	0	0	0	0
0	0	0	0	0	0	0	0	0	0	0	0	0	0
0	0	0	0	0	0	1	0	0	0	0	0	0	0
0													
OTU14631	0	0	0	0	1	0	0	0	0	0	0	0	0
0	0	0	0	0	0	0	0	0	0	0	0	0	0
0	0	0	0	0	0	0	0	0	0	0	0	0	0
0	0	0	0	0	0	0	0	0	0	0	0	0	0
0	0	0	0	0	0	0	0	0	0	0	0	0	0
0	0	0	0	0	0	0	0	0	0	0	0	0	0
0	0	0	0	0	0	0	0	0	0	0	0	0	0
0	0	0	0	0	0	0	0	0	0	0	0	0	0
0													
OTU10892	0	0	0	0	1	0	0	0	0	0	0	0	0
0	0	0	0	0	0	0	0	0	0	0	0	0	0
0	0	0	0	0	0	0	0	0	0	0	0	0	0

0	0	0	0	0	0	0	0	0	0	0	0	0	0
0	0	0	0	0	0	0	0	0	0	0	0	0	0
0	0	0	0	0	0	0	0	0	0	0	0	0	0
0	0	0	0	0	0	0	0	0	0	0	0	0	0
0	0	0	0	0	0	0	0	0	0	0	0	0	0
0													
OTU10894	0	0	0	0	1	0	0	0	0	0	1	1	0
0	0	0	3	1	0	0	0	0	4	16	0	0	0
0	5	1	1	2	0	0	0	1	0	1	2	1	0
0	0	0	0	0	4	4	2	1	0	0	0	12	0
5	1	14	0	6	0	24	0	0	9	0	0	0	31
1	0	0	5	2	2	0	0	0	0	1	0	0	0
4	0	2	0	0	0	0	0	0	0	0	1	0	11
0	0	0	0	0	0	0	1	0	0	0	1	0	0
0													
OTU12072	0	0	0	0	1	0	0	0	0	0	0	0	0
0	0	0	0	0	0	0	0	0	0	0	0	0	0
0	0	0	0	0	0	0	0	0	0	0	0	0	0
0	0	0	0	0	0	0	0	0	0	0	0	0	0
0	0	0	0	0	0	0	0	0	0	0	0	0	0
0	0	0	0	0	0	0	0	0	0	0	0	0	0
0	0	0	0	0	0	0	0	0	0	0	0	0	0
0	0	0	0	0	0	0	0	0	0	0	0	0	0
0													
OTU12404	0	0	0	0	1	1	0	1	0	2	2	0	0
0	0	0	1	0	0	0	0	0	0	0	0	0	0
0	0	0	0	0	1	0	0	0	1	0	0	0	0
1	0	0	0	0	0	0	0	0	0	0	0	0	0
0	0	0	1	0	0	0	0	0	1	0	0	0	0
1	2	0	0	0	1	0	0	0	1	0	2	0	0
0	0	0	2	1	0	0	0	0	0	1	0	1	0
0	0	0	1	0	0	1	0	0	0	0	0	0	1
2													

OTU8449 0 0 0 0 1 0 0 0 0 0 0 0 0
0 0 0 0 1 0 0 0 0 0 0 0 0 0
0 0 0 0 0 0 0 0 0 0 0 0 0 0
0 0 0 0 0 0 0 0 0 0 0 0 0 0
0 0 0 0 0 0 0 0 0 0 0 0 0 0
0 0 0 0 0 0 0 0 0 0 0 0 0 0
0 0 0 0 0 0 0 0 0 0 0 0 0 0
0 0 0 0 0 0 0 0 0 0 0 0 0 0
0
OTU10893 0 0 0 0 1 0 0 0 0 0 0 0 0
0 0 0 0 0 0 0 0 0 0 0 0 0 0
0 0 0 0 0 0 0 0 0 0 0 0 0 0
0 0 0 0 0 0 0 0 0 0 0 0 0 0
0 0 0 0 0 0 0 0 0 0 0 0 0 0
0 0 0 0 0 0 0 0 0 0 0 0 0 0
0 0 0 0 0 0 0 0 0 0 0 0 0 0
0 0 0 0 0 0 0 0 0 0 0 0 0 0
0
OTU3423 0 0 0 0 1 0 0 0 0 0 0 0 0
0 0 0 0 0 0 0 0 0 0 0 0 1 0
0 0 0 0 0 0 0 0 0 0 0 0 0 0
0 0 0 0 0 0 0 0 0 0 0 0 0 0
0 0 0 0 0 0 0 0 0 0 0 0 0 0
0 0 0 0 0 0 0 0 0 0 0 0 0 0
0 1 0 0 0 0 0 0 0 0 0 0 0 0
0 0 0 0 0 0 0 0 0 0 0 0 0 0
0
OTU12071 0 0 0 0 1 0 0 0 0 0 0 0 0
0 0 0 0 0 0 0 0 0 0 0 0 0 0
0 0 0 0 0 0 0 0 0 0 0 0 0 0
0 0 0 0 0 0 0 0 0 0 0 0 0 0
0 0 0 0 0 0 0 0 0 0 0 0 0 0
0 0 0 0 0 0 0 0 0 0 0 0 0 0

0	0	0	0	0	0	0	0	0	0	0	0	0	0
0	0	0	0	0	0	0	0	0	0	0	0	0	0
0													
OTU1742	0	0	0	0	1	0	0	0	0	0	0	0	0
0	0	0	0	0	0	1	1	0	0	0	0	0	1
0	0	0	0	0	0	0	0	0	1	0	0	0	0
0	0	0	0	0	0	0	0	1	0	0	0	0	0
0	0	0	0	0	0	0	0	0	0	0	0	0	0
1	0	0	0	0	0	0	0	0	0	0	0	0	0
0	0	0	0	0	0	0	0	0	0	0	0	0	0
0	0	0	0	0	1	1	0	0	0	0	0	0	0
0													
OTU14626	0	0	0	0	1	0	0	0	0	0	0	0	0
0	0	0	0	0	0	0	0	0	0	0	0	0	0
0	0	0	0	0	0	0	0	0	0	0	0	0	0
0	0	0	0	0	0	0	0	0	0	0	0	0	0
0	0	0	0	0	0	0	0	0	0	0	0	0	0
0	0	0	0	0	0	0	0	0	0	0	0	0	0
0	0	0	0	0	0	0	0	0	0	0	0	0	0
0	0	0	0	0	0	0	0	0	0	0	0	0	0
0													
OTU8447	0	0	0	0	1	0	0	0	0	0	0	0	0
0	0	0	0	0	0	0	0	0	0	0	0	0	0
0	0	0	0	0	0	0	0	0	0	0	0	0	0
0	0	0	0	0	0	0	0	0	0	0	0	0	0
0	0	0	0	0	0	0	0	0	0	1	0	0	0
0	0	0	0	2	0	0	0	0	0	0	0	0	0
0	0	0	0	0	0	0	0	0	0	0	0	0	0
0	0	0	0	0	0	0	0	0	0	0	0	0	0
0													
OTU8446	0	0	0	0	1	0	0	0	0	0	0	0	0
0	0	0	0	0	0	0	0	0	0	0	0	0	0
0	0	0	0	0	0	0	0	0	0	0	0	0	0

0	0	0	0	0	0	0	0	0	0	0	0	0	0
0	0	0	0	0	0	0	0	0	0	0	0	0	0
0	0	0	0	0	0	0	0	0	0	0	0	0	0
0	0	0	0	0	0	0	0	0	0	0	0	0	0
0	0	0	0	0	0	0	0	0	0	0	0	0	0
0													
OTU1100	0	0	0	0	1	0	0	0	0	0	0	0	0
0	0	0	0	0	0	0	0	0	0	0	0	0	0
0	0	0	0	0	0	1	0	0	0	0	0	0	0
0	0	0	0	0	0	0	2	0	0	0	0	0	0
0	0	5	0	0	3	0	0	0	0	0	0	0	1
0	0	0	0	0	0	0	0	0	0	0	0	0	0
0	0	0	0	0	0	0	0	0	0	0	0	0	0
0	0	0	0	0	0	0	0	0	0	0	0	0	0
0													
OTU9068	0	0	0	0	1	0	0	0	0	0	0	0	1
0	0	0	0	0	0	0	0	0	1	0	0	0	0
0	0	0	0	0	0	0	1	0	0	0	0	0	1
0	0	0	0	0	1	0	0	0	0	0	0	0	0
0	0	0	0	1	1	0	0	0	0	1	0	0	0
1	0	0	0	0	0	0	0	0	1	0	1	0	0
1	0	0	0	0	0	0	0	0	0	0	0	0	0
0	1	0	0	1	0	0	0	0	0	0	0	0	0
1													
OTU14625	0	0	0	0	1	0	0	0	0	0	0	0	0
0	0	0	0	0	0	0	0	0	0	0	0	0	0
0	0	0	0	0	0	0	0	0	0	0	0	0	0
0	0	0	0	0	0	0	0	0	0	0	0	0	0
0	0	0	0	0	0	0	0	0	0	0	0	0	0
0	0	0	0	0	0	0	0	0	0	0	0	0	0
0	0	0	0	0	0	0	0	0	0	0	0	0	0
0	0	0	0	0	0	0	0	0	0	0	0	0	0
0													

OTU12066	0	0	0	0	1	0	0	0	0	0	0	0	0
0	0	0	0	0	0	0	0	0	0	0	0	0	0
0	0	0	0	0	0	0	0	0	0	0	0	0	0
0	0	0	0	0	0	0	0	0	0	0	0	0	0
0	0	0	0	0	0	0	0	0	0	0	0	0	0
0	0	0	0	0	0	0	0	0	0	0	0	0	0
0	0	0	0	0	0	0	0	0	0	0	0	0	0
0	0	0	0	0	0	0	0	0	0	0	0	0	0
0													
OTU10889	0	0	0	0	1	0	0	0	0	0	0	0	0
0	0	0	0	0	0	0	0	0	0	0	0	0	0
0	0	0	0	0	0	0	0	0	0	0	0	0	0
0	0	0	0	0	0	0	0	0	0	0	0	0	0
0	0	0	0	0	0	0	0	0	0	0	0	0	0
0	0	0	0	0	0	0	0	0	0	0	0	0	0
0	0	0	0	0	0	0	0	0	0	0	0	0	0
0	0	0	0	0	0	0	0	0	0	0	0	0	0
0													
OTU1949	0	0	0	0	1	0	0	0	0	0	0	1	0
0	0	0	0	0	0	0	0	0	0	0	0	0	0
0	0	0	0	0	0	0	0	0	0	0	0	0	0
0	0	0	0	0	0	0	0	0	0	0	0	1	0
0	0	0	0	0	0	0	0	0	1	0	0	0	0
0	0	0	0	0	0	0	0	0	0	0	0	0	0
0	0	0	0	0	0	0	0	0	0	0	0	0	0
0	0	0	0	0	0	0	0	0	0	0	1	0	0
0													
OTU12067	0	0	0	0	1	0	0	0	0	0	0	0	0
0	0	0	0	0	0	0	0	0	0	0	0	0	0
0	0	0	0	0	0	0	0	0	0	0	0	0	0
0	0	0	0	0	0	0	0	0	0	0	0	0	0
0	0	0	0	0	0	0	0	0	0	0	0	0	0
0	0	0	0	0	0	0	0	0	0	0	0	0	0

0	0	0	0	0	0	0	0	0	0	0	0	0	0
0	0	0	0	0	0	0	0	0	0	0	0	0	0
0													
OTU13326	0	0	0	0	1	1	0	0	0	0	0	0	0
0	0	0	0	0	0	0	0	0	0	0	0	0	0
0	0	0	1	0	0	0	1	0	0	0	1	0	0
0	0	0	0	0	0	0	0	0	0	0	0	0	1
0	0	0	0	0	0	0	0	0	0	0	0	0	1
0	0	0	0	0	0	0	0	0	0	0	0	0	0
0	0	0	0	0	0	0	0	0	0	0	0	0	0
0	0	0	0	0	0	0	0	0	0	0	0	0	0
0													
OTU14635	0	0	0	0	1	0	0	0	0	0	0	0	0
0	0	0	0	0	0	0	0	0	0	0	0	0	0
0	0	0	0	0	0	0	0	0	0	0	0	0	0
0	0	0	0	0	0	0	0	0	0	0	0	0	0
0	0	0	0	0	0	0	0	0	0	0	0	0	0
0	0	0	0	0	0	0	0	0	0	0	0	0	0
0	0	0	0	0	0	0	0	0	0	0	0	0	0
0	0	0	0	0	0	0	0	0	0	0	0	0	0
0													
OTU12068	0	0	0	0	1	0	0	0	0	0	0	0	0
0	0	0	0	0	0	0	0	0	0	0	0	0	0
0	0	0	0	0	0	0	0	0	0	0	0	0	0
0	0	0	0	0	0	0	0	0	0	0	0	0	0
0	0	0	0	0	0	0	0	0	0	0	0	0	0
0	0	0	0	0	0	0	0	0	0	0	0	0	0
0	0	0	0	0	0	0	0	0	0	0	0	0	0
0	0	0	0	0	0	0	0	0	0	0	0	0	0
0													
OTU5199	0	0	0	0	1	0	0	0	0	0	2	0	1
0	0	0	0	0	0	0	0	0	0	0	0	0	0
0	0	0	0	0	0	0	0	0	0	0	1	0	0

0 0 0 0 0 0 1 0 0 0 0 0 0 0

0 0 0 0 0 0 0 0 0 0 0 0 0 0

0 0 0 0 0 0 0 0 1 0 0 0 0 0

0 0 0 0 0 1 0 0 0 0 0 0 0 0

0 0 0 0 0 0 0 0 0 0 0 0 0 0

0

OTU10870 0 0 0 0 1 0 0 0 0 0 0 0 0

0 0 0 0 0 0 0 0 0 0 0 0 0 0

0 0 0 0 0 0 0 0 0 0 0 0 0 0

0 0 0 0 0 0 0 0 0 0 0 0 0 0

0 0 0 0 0 0 0 0 0 0 0 0 0 0

0 0 0 0 0 0 0 0 0 0 0 0 0 0

0 0 0 0 0 0 0 0 0 0 0 0 0 0

0 0 0 0 0 0 0 0 0 0 0 0 0 0

0

OTU12015 0 0 0 0 1 0 0 0 0 0 0 0 0

0 0 0 0 0 0 0 0 0 0 0 0 0 0

0 0 0 0 0 0 0 0 0 0 0 0 0 0

0 0 0 0 0 0 0 0 0 0 0 0 0 0

0 0 0 0 0 0 0 0 0 0 0 0 0 0

0 0 0 0 0 0 0 0 0 0 0 0 0 0

0 0 0 0 0 0 0 0 0 0 0 0 0 0

0 0 0 0 0 0 0 0 0 0 0 0 0 0

0

OTU3203 0 0 0 0 1 0 0 0 0 1 0 0 0

0 0 0 0 2 0 0 0 0 0 0 0 0 0

0 0 0 0 0 0 0 0 0 0 0 0 1 0

0 0 0 0 0 0 0 0 0 0 0 0 0 0

0 0 0 0 0 0 0 1 0 0 0 0 0 0

0 0 0 0 0 0 0 0 0 0 0 1 0 1

0 1 0 0 0 1 0 0 0 0 0 0 0 0

0 0 0 0 0 0 0 0 0 0 0 0 0 0

0

OTU12014	0	0	0	0	1	0	0	0	0	0	0	0	0
0	0	0	0	0	0	0	0	0	0	0	0	0	0
0	0	0	0	0	0	0	0	0	0	0	0	0	0
0	0	0	0	0	0	0	0	0	0	0	0	0	0
0	0	0	0	0	0	0	0	0	0	0	0	0	0
1	0	0	0	0	0	0	0	0	0	0	0	0	0
0	0	0	0	0	0	0	0	0	0	0	0	0	0
0	0	0	0	0	0	1	0	0	0	0	0	0	0
0													
OTU10869	0	0	0	0	1	0	0	0	0	0	0	0	0
0	0	0	0	0	0	0	0	0	0	0	0	0	0
0	0	0	0	0	0	0	0	0	0	0	0	0	0
0	0	0	0	0	0	0	0	0	0	0	0	0	0
0	0	0	0	0	0	0	0	0	0	0	0	0	0
0	0	0	0	0	0	0	0	0	0	0	0	0	0
0	0	0	0	0	0	0	0	0	0	0	0	0	0
0	0	0	0	0	0	0	0	0	0	0	0	0	0
0													
OTU14633	0	0	0	0	1	0	0	0	1	1	0	1	0
0	0	0	0	1	0	0	0	0	0	0	0	0	0
0	0	0	0	0	0	0	0	0	0	0	0	0	0
0	0	0	0	0	0	0	0	0	0	0	0	0	0
0	0	0	0	0	0	0	0	0	0	0	0	0	0
0	0	0	0	0	0	0	0	0	0	0	0	0	0
0	0	0	0	0	0	0	0	0	0	0	0	0	0
0	1	0	0	0	0	0	0	0	0	0	0	0	0
0													
OTU3669	0	0	0	0	1	0	0	0	0	0	0	0	0
0	0	0	0	0	0	0	0	0	1	0	0	0	0
0	0	1	0	0	0	0	0	0	0	0	1	0	0
0	0	0	0	0	0	0	0	0	0	0	0	0	0
0	0	0	0	0	0	0	0	0	0	0	0	0	0
0	0	0	0	0	0	0	0	0	0	0	0	0	0

0 0 0 0 0 0 0 0 0 0 0 0 0 0
0 0 0 0 0 0 0 0 0 0 0 0 0 0
0
OTU15834 0 0 0 0 1 0 0 1 0 0 0 0 0
0 0 0 0 0 0 0 0 0 0 0 0 0 0
0 0 0 0 0 0 0 0 0 0 0 0 0 0
0 0 0 0 0 0 0 0 0 0 0 0 0 0
0 0 0 0 0 0 0 0 0 0 0 0 0 0
0 0 0 0 0 0 0 0 0 0 0 0 0 0
0 0 0 0 0 0 0 0 0 0 0 0 0 0
0 0 0 0 0 0 0 0 0 0 0 0 0 0
0
OTU8432 0 0 0 0 1 0 0 0 0 0 0 0 0
0 0 0 0 0 0 0 0 0 0 0 0 0 0
0 0 0 0 0 0 0 0 0 0 0 0 0 0
0 0 0 0 0 0 0 0 0 0 0 0 0 0
0 0 0 0 0 0 0 0 0 0 0 0 0 0
0 0 0 0 0 0 0 0 0 0 0 0 0 0
0 0 0 0 0 0 0 0 0 0 0 0 0 0
0 0 0 0 0 0 0 0 0 0 0 0 0 0
0
OTU625 0 0 0 0 2 0 0 0 0 0 0 0 0
1 0 1 0 1 0 0 0 1 0 0 0 0 0
0 0 0 0 0 0 0 0 1 0 0 0 0 0
0 2 1 0 0 0 0 0 0 5 0 0 0 0
1 0 0 0 0 0 0 0 0 2 2 0 0 0
0 0 0 0 1 0 0 0 0 0 0 1 0 0
1 0 0 0 1 0 0 0 49 0 2 0 0 10
0 0 0 0 0 0 0 0 0 0 0 0 0 0
0
OTU10871 0 0 0 0 1 0 0 0 0 0 0 0 0
0 0 0 0 0 0 0 0 0 0 0 0 0 0
0 0 0 0 0 0 0 0 0 0 0 0 0 0

0	0	0	0	0	0	0	0	0	0	0	0	0	0
0	0	0	0	0	0	0	0	0	0	0	0	0	0
0	0	0	0	0	0	0	0	0	0	0	0	0	0
0	0	0	0	0	0	0	0	0	0	0	0	0	0
0	0	0	0	0	0	0	0	0	0	0	0	0	0
0													
OTU449	0	0	0	0	1	0	0	0	0	0	2	0	0
0	0	0	0	0	0	0	2	0	7	0	0	0	20
0	0	17	13	0	1	0	0	0	3	0	0	0	0
0	0	0	0	0	0	0	0	0	0	0	0	0	0
0	0	0	0	0	0	0	1	0	0	0	0	0	0
0	0	0	0	0	0	0	0	0	0	0	1	0	0
0	0	0	0	0	0	0	0	0	0	0	0	0	0
0	0	0	0	0	0	0	0	0	0	0	0	0	0
0													
OTU6985	0	0	0	0	1	0	0	0	0	0	0	0	0
0	0	0	0	0	0	0	0	0	0	0	0	0	0
0	0	0	0	0	0	0	0	0	0	0	0	0	0
0	0	0	0	0	0	0	0	0	0	0	0	0	0
0	0	0	0	0	0	0	0	0	0	0	0	0	0
0	0	0	0	0	0	0	0	0	0	0	0	0	0
0	0	0	0	0	0	0	0	0	0	0	0	0	0
1	0	0	0	0	0	0	0	0	0	0	0	0	0
0													
OTU1458	0	0	0	0	1	1	0	0	0	1	0	0	0
0	1	0	0	0	0	0	0	0	0	1	0	0	0
0	0	1	0	0	0	0	0	0	0	0	1	1	0
1	0	0	0	0	0	0	0	0	1	0	0	1	0
0	0	0	0	0	0	0	1	0	0	0	0	0	0
0	0	1	0	1	0	0	0	2	0	0	1	0	0
0	1	1	0	0	0	0	0	0	0	0	1	0	0
0	0	0	0	0	0	0	0	0	0	0	0	0	1
2													

OTU12016 0 0 0 0 1 0 0 0 0 0 0 0 0
0 0 0 0 0 0 0 0 0 0 0 0 0 0
0 0 0 0 0 0 0 0 0 0 0 0 0 0
0 0 0 0 0 0 0 0 0 0 0 0 0 0
0 0 0 0 0 0 0 0 0 0 0 0 0 0
0 0 0 0 0 0 0 0 0 0 0 0 0 0
0 0 0 0 0 0 0 0 0 0 0 0 0 0
0 0 0 0 0 0 0 0 0 0 0 0 0 0
0
OTU6855 0 0 0 0 1 1 0 0 0 0 0 0 0
0 0 1 0 0 0 0 0 0 0 0 0 0 0
0 0 0 0 0 0 0 0 0 0 0 0 0 0
0 0 0 0 0 0 0 0 0 0 2 0 0 0
0 0 1 1 0 0 0 0 0 0 0 0 0 0
1 0 0 0 0 0 0 0 0 0 0 0 0 0
0 0 0 1 0 1 0 0 0 0 1 0 0 0
0 0 0 0 0 0 0 0 0 0 0 0 0 0
0
OTU3293 0 0 0 0 1 0 1 0 0 3 1 0 0
0 3 1 0 0 1 0 0 0 0 0 0 0 0
0 1 0 0 0 0 0 0 0 1 0 0 0 1
0 0 0 0 1 0 0 0 0 0 0 1 0 0
0 0 2 0 0 0 0 1 0 1 0 1 0 1
0 1 0 0 0 0 1 0 0 0 0 0 0 0
0 1 0 0 0 1 0 0 0 0 0 0 0 0
1 0 0 0 2 0 0 1 0 1 0 0 0 0
3
OTU10868 0 0 0 0 1 0 0 0 0 0 0 0 0
0 0 0 0 0 0 0 0 0 0 0 0 0 0
0 0 0 0 0 0 0 0 0 0 0 0 0 0
0 0 0 0 0 0 0 0 0 0 0 0 0 0
0 0 0 0 0 0 0 0 0 0 0 0 0 0
0 0 0 0 0 0 0 0 0 0 0 0 0 0

0	0	0	0	0	0	0	0	0	0	0	0	0	0
0	0	0	0	0	0	0	0	0	0	0	0	0	0
0													
OTU346	0	0	0	0	0	3	1	0	0	0	5	0	0
6	0	0	0	0	43	0	1	0	0	0	0	0	14
0	0	0	0	0	0	0	4	0	2	0	0	0	7
0	0	0	2	0	0	0	0	0	0	0	0	0	0
0	2	0	0	0	0	0	0	0	0	0	0	0	0
0	0	0	0	0	0	0	0	0	0	0	1	0	0
0	0	0	0	0	0	0	0	8	1	0	0	0	0
0	0	0	0	0	0	0	0	0	0	0	2	0	0
0													
OTU14448	0	0	0	0	0	12	0	0	14	0	0	0	0
0	1	0	0	0	0	0	0	0	0	0	0	0	0
0	0	0	0	0	0	0	0	0	0	0	0	3	0
16	0	0	0	0	0	0	0	0	0	0	0	0	0
0	0	0	1	0	0	0	0	0	0	0	0	0	0
0	0	0	0	1	0	0	0	0	0	0	0	0	0
0	0	0	1	0	0	0	7	0	0	0	0	0	0
0	0	0	0	0	0	0	0	0	6	0	0	0	0
0													
OTU14634	0	0	0	0	0	1	0	0	0	0	0	0	0
0	0	0	0	0	0	0	0	0	0	0	0	0	0
0	0	0	0	0	0	0	0	0	0	0	0	0	0
0	0	0	0	0	0	0	0	0	0	0	0	0	0
0	0	0	0	0	0	0	0	0	0	0	0	0	0
0	0	0	0	0	0	0	0	0	0	0	0	0	0
0	0	0	0	0	0	0	0	0	0	0	0	0	0
0	0	0	0	0	0	0	0	0	0	0	0	0	0
0													
OTU8436	0	0	0	0	0	1	0	0	0	0	0	0	0
0	0	0	0	0	0	0	0	0	0	0	0	0	0
0	0	0	0	0	0	0	0	0	0	0	0	0	0

0	0	0	0	0	0	0	0	0	0	0	0	0	0
0	0	0	0	0	0	0	0	0	0	0	0	0	0
0	0	0	0	0	0	0	0	0	0	0	0	0	0
0	0	0	0	0	0	0	0	0	0	0	0	0	0
0	0	0	0	0	0	0	0	0	0	0	0	0	0
0													
OTU4402	0	0	0	0	0	9	16	0	1	3	0	0	0
0	17	3	0	0	3	0	14	0	0	0	0	0	0
0	1	0	0	0	0	0	0	0	4	0	0	0	0
0	324	0	4	0	0	0	0	0	0	0	0	0	0
0	4	0	0	0	0	0	0	0	0	0	0	4	0
0	5	0	0	4	0	3	0	0	7	0	0	2	0
1	0	0	3	0	0	0	0	0	0	0	2	3	0
0	1	1	0	1	1	1	3	2	13	1	0	0	3
0													
OTU8437	0	0	0	0	0	1	0	0	0	0	0	0	0
0	0	0	0	0	0	0	0	0	0	0	0	0	0
0	0	0	0	0	0	0	0	0	0	0	0	0	0
0	0	0	0	0	0	0	0	0	0	0	0	0	0
0	0	0	0	0	0	0	0	0	0	0	0	0	0
0	0	0	0	0	0	0	0	0	0	0	0	0	0
0	0	0	0	0	0	0	0	0	0	0	0	0	0
0	0	0	0	0	0	0	0	0	0	0	0	0	0
0													
OTU15444	0	0	0	0	0	1	1	0	0	3	1	0	38
8	0	0	0	0	191	37	0	1	0	4	0	101	0
0	0	0	0	0	0	51	0	0	26	1	0	52	1
3	0	0	11	0	0	0	0	0	0	0	3	0	0
0	0	0	0	0	0	0	1	0	0	0	0	0	0
0	0	27	4	0	0	0	0	0	0	0	0	0	0
0	0	0	150	0	0	0	0	10	0	0	0	0	0
0	0	0	0	0	0	0	0	0	0	3	0	0	0
0													

OTU8438 0 0 0 0 0 1 0 0 0 0 0 0 0
0 0 0 0 0 0 0 0 0 0 0 0 0 0
0 0 0 0 0 0 0 0 0 0 0 0 0 0
0 0 0 0 0 0 0 0 0 0 0 0 0 0
0 0 0 0 0 0 0 0 0 0 0 0 0 0
0 0 0 0 0 0 0 0 0 0 0 0 0 0
0 0 0 0 0 0 0 0 0 0 0 0 0 0
0 0 0 0 0 0 0 0 0 0 0 0 0 0
0
OTU8439 0 0 0 0 0 1 0 0 0 0 0 0 0
0 0 0 0 0 0 0 0 0 0 0 0 0 0
0 0 0 0 0 0 0 0 0 0 0 0 0 0
0 0 0 0 0 0 0 0 0 0 0 0 0 0
0 0 0 0 0 0 0 0 0 0 0 0 0 0
0 0 0 0 0 0 0 0 0 0 0 0 0 0
0 0 0 0 0 0 0 0 0 0 0 0 0 0
0 0 0 0 0 0 0 0 0 0 0 0 0 0
0
OTU11952 0 0 0 0 0 2 2 1 0 0 2 0 0
0 0 0 0 2 0 1 0 0 0 0 0 11 13
1 0 0 1 2 0 0 0 0 0 0 0 0 0
0 0 0 3 0 1 0 0 0 0 0 0 0 0
0 0 0 0 0 0 0 0 0 1 0 0 0 0
0 0 1 0 0 0 0 0 0 0 1 0 1 0
5 0 0 0 0 0 0 1 0 0 1 0 0 0
0 0 0 0 0 0 0 0 0 0 0 0 0 0
0
OTU1004 0 0 0 0 0 1 0 0 0 0 0 0 0
0 0 0 0 0 0 0 0 0 0 1 0 0 0
0 0 0 0 0 0 0 0 0 0 0 0 0 0
0 0 0 0 0 0 0 0 0 0 0 0 0 0
0 0 0 0 0 0 0 0 0 0 0 0 0 0
0 0 0 0 0 0 0 0 0 0 0 0 0 0

0	1	0	0	0	0	0	0	0	0	0	0	0	0
0	0	0	0	0	0	0	0	0	0	0	0	0	0
0													
OTU14657	0	0	0	0	0	1	0	0	0	0	0	0	0
0	0	0	0	0	0	0	0	0	0	0	0	0	0
0	0	0	0	0	0	0	0	0	0	0	0	0	0
0	0	0	0	0	0	0	0	0	0	0	0	0	0
0	0	0	0	0	0	0	0	0	0	0	0	0	0
0	0	0	0	0	0	0	0	0	0	0	0	0	0
0	0	0	0	0	0	0	0	0	0	0	0	0	0
0	0	0	0	0	0	0	0	0	0	0	0	0	0
0													
OTU424	0	0	0	0	0	3	0	0	0	0	2	0	0
0	0	0	0	0	36	0	3	0	0	0	0	0	1
0	0	0	0	0	0	0	1	0	0	0	0	0	3
0	0	0	1	0	0	0	1	0	0	1	0	0	1
0	1	0	0	0	0	0	0	0	0	0	0	0	0
0	0	0	0	0	0	0	0	0	0	0	0	0	0
0	0	0	0	0	0	0	0	0	0	0	0	0	0
0	0	0	0	0	0	0	0	0	0	0	0	0	0
0													
OTU15366	0	0	0	0	0	1	1	0	0	0	0	0	0
0	0	0	0	0	0	0	0	0	0	0	0	0	0
0	0	0	0	0	1	0	0	0	0	0	0	0	0
0	1	0	0	0	0	0	0	0	1	0	0	0	0
0	0	0	0	0	0	0	0	2	0	0	0	0	1
0	0	0	0	0	0	0	0	0	0	0	0	0	0
0	0	0	0	0	0	1	0	0	0	0	0	0	0
0	0	0	0	0	0	0	0	0	0	0	0	0	0
0													
OTU6063	0	0	0	0	0	1	1	1	0	0	1	0	1
1	0	0	0	0	0	0	1	0	2	0	1	0	0
1	0	0	0	1	0	0	0	0	0	0	0	0	1

0	0	0	0	0	1	0	0	0	1	0	0	1	0
0	0	0	0	0	1	1	1	0	0	1	0	0	0
0	0	1	1	0	0	1	0	0	0	0	0	0	0
0	0	0	0	0	0	0	0	0	0	0	0	1	0
0	0	0	0	1	1	0	0	0	0	0	0	0	1
3													
OTU14078	0	0	0	0	0	1	0	0	0	0	0	0	0
0	0	0	0	0	0	0	0	0	0	0	0	0	0
0	0	0	0	0	0	0	0	0	0	0	0	0	0
0	0	0	0	0	0	0	0	0	0	0	0	0	0
0	0	0	0	0	0	0	0	0	0	0	0	0	0
0	0	0	0	0	0	0	0	0	1	0	0	0	0
0	0	0	0	0	0	0	0	0	0	0	0	0	0
0	0	0	0	0	0	0	0	0	0	0	0	0	0
0													
OTU3940	0	0	0	0	0	1	0	0	0	0	0	0	0
0	0	0	0	0	0	0	0	0	0	0	0	0	0
0	0	1	0	0	0	0	0	0	0	0	0	0	0
0	0	0	0	0	0	0	0	0	0	0	1	0	0
0	0	0	0	0	0	0	0	0	0	0	0	0	0
0	0	0	0	0	0	0	0	0	1	0	0	0	0
0	0	0	0	0	0	0	0	0	0	1	0	1	0
0	0	0	0	0	0	0	0	0	0	0	0	0	0
0													
OTU3261	0	0	0	0	0	1	0	1	2	2	1	5	0
0	0	2	0	0	2	1	1	0	0	0	0	3	0
0	0	1	0	1	0	0	0	0	0	2	0	3	1
0	3	1	1	0	2	0	0	0	0	1	0	0	0
0	1	0	3	0	0	0	0	0	0	0	0	0	1
0	0	1	0	0	0	1	0	0	0	0	0	0	2
0	0	0	2	0	0	0	0	0	0	2	0	0	0
1	1	1	1	1	0	0	1	1	0	0	0	0	0
0													

OTU542	0	0	0	0	0	1	0	0	0	0	0	0	0
0	0	0	0	0	0	0	0	0	0	0	0	0	0
0	0	0	0	0	0	0	0	0	0	0	0	0	0
0	1	0	0	0	1	0	10	0	0	0	0	0	0
0	1	0	0	0	0	0	0	0	0	0	0	5	0
0	0	0	0	0	0	0	0	0	1	0	0	0	0
0	0	0	1	0	0	0	0	0	0	0	0	1	0
0	7	1	0	7	0	0	0	0	0	1	1	0	0
4													
OTU8434	0	0	0	0	0	1	0	0	0	0	0	0	0
0	0	0	0	0	0	0	0	0	0	0	0	0	0
0	0	0	0	0	0	0	0	0	0	0	0	0	0
0	0	0	0	0	0	0	0	0	0	0	0	0	0
0	0	0	0	0	0	0	0	0	0	0	0	0	0
0	0	0	0	0	0	0	0	0	0	0	0	0	0
0	0	0	0	0	0	0	0	0	0	0	0	0	0
0	0	0	0	0	0	0	0	0	0	0	0	0	0
0													
OTU8435	0	0	0	0	0	1	0	0	0	0	0	0	0
0	0	0	0	0	0	0	0	0	0	0	0	0	0
0	0	0	0	0	0	0	0	0	0	0	0	0	0
0	0	0	0	0	0	0	0	0	0	0	0	0	0
0	0	0	0	0	0	0	0	0	0	0	0	0	0
0	0	0	0	0	0	0	0	0	0	0	0	0	0
0	0	0	0	0	0	0	0	0	0	0	0	0	0
0	0	0	0	0	0	0	0	0	0	0	0	0	0
0													
OTU12020	0	0	0	0	0	1	0	0	0	0	0	0	0
0	0	0	0	0	0	0	0	0	0	0	0	0	0
0	0	0	0	0	0	0	0	0	0	0	0	0	0
0	0	0	0	0	0	0	0	0	0	0	0	0	0
0	0	0	0	0	0	0	0	0	0	0	0	0	0
0	0	0	0	0	0	0	0	0	0	0	0	0	0

0	0	0	0	0	0	0	0	0	0	0	0	0	0
0	0	0	0	0	0	0	0	0	0	0	0	0	0
0													
OTU10877	0	0	0	0	0	1	0	0	0	0	0	0	0
0	0	0	0	0	0	0	0	0	0	0	0	0	0
0	0	0	0	0	0	0	0	0	0	0	0	0	0
0	0	0	0	0	0	0	0	0	0	0	0	0	0
0	0	0	0	0	0	0	0	0	0	0	0	0	0
0	0	0	0	0	0	0	0	0	0	0	0	0	0
0	0	0	0	0	0	0	0	0	0	0	0	0	0
0	0	0	0	0	0	0	0	0	0	0	0	0	0
0													
OTU8382	0	0	0	0	0	1	0	0	0	0	0	0	0
0	0	0	0	0	0	0	0	0	0	0	0	0	0
0	0	0	0	0	0	0	0	0	0	0	0	0	0
0	0	0	0	0	0	0	0	0	0	0	0	0	0
0	0	0	0	0	0	0	0	0	0	0	0	0	0
0	0	0	0	0	0	0	0	0	0	0	0	0	0
0	0	0	0	0	0	0	0	0	0	0	0	0	0
0	0	0	0	0	0	0	0	0	0	0	0	0	0
0													
OTU10878	0	0	0	0	0	1	0	0	0	0	0	0	0
0	0	0	0	0	0	0	0	0	0	0	0	0	0
0	0	0	0	0	0	0	0	0	0	0	0	0	0
0	0	0	0	0	0	0	0	0	0	0	0	0	0
0	0	0	0	0	0	0	0	0	0	0	0	0	0
0	0	0	0	0	0	0	0	0	0	0	0	0	0
0	0	0	0	0	0	0	0	0	0	0	0	0	0
0	0	0	0	0	0	0	0	0	0	0	0	0	0
0													
OTU10875	0	0	0	0	0	1	0	0	0	0	0	0	0
0	0	0	0	0	0	0	0	0	0	0	0	0	0
0	0	0	0	0	0	0	0	0	0	0	0	0	0

0	0	0	0	0	0	0	0	0	0	0	0	0	0
0	0	0	0	0	0	0	0	0	0	0	0	0	0
0	0	0	0	0	0	0	0	0	0	0	0	0	0
0	0	0	0	0	0	0	0	0	0	0	0	0	0
0	0	0	0	0	0	0	0	0	0	0	0	0	0
0													
OTU491	0	0	0	0	0	4	0	0	0	2	0	0	1
0	0	0	0	5	0	0	0	0	0	0	8	0	0
0	0	0	0	0	77	0	0	0	0	0	0	0	0
0	0	0	0	0	23	0	0	1	0	0	0	0	0
0	0	0	0	0	0	0	0	0	0	0	0	0	0
0	0	0	0	0	0	0	0	0	0	0	0	0	0
0	0	0	0	0	0	0	0	0	0	0	0	0	0
0	0	0	0	0	0	0	0	0	0	0	0	0	0
0													
OTU12017	0	0	0	0	0	1	0	0	0	0	0	0	0
0	0	0	0	0	0	0	0	0	0	0	0	0	0
0	0	0	0	0	0	0	0	0	0	0	0	0	0
0	0	0	0	0	0	0	0	0	0	0	0	0	0
0	0	0	0	0	0	0	0	0	0	0	0	0	0
0	0	0	0	0	0	0	0	0	0	0	0	0	0
0	0	0	0	0	0	0	0	0	0	0	0	0	0
0	0	0	0	0	0	0	0	0	0	0	0	0	0
0													
OTU515	0	0	0	0	0	1	0	0	0	0	0	0	0
0	0	0	0	0	0	0	0	0	0	0	0	0	0
0	0	0	0	0	0	0	0	0	2	0	0	0	0
0	0	0	0	0	0	0	0	0	0	0	0	0	0
0	0	0	0	0	0	0	0	0	0	0	0	0	0
0	1	0	0	0	0	0	0	0	0	0	0	0	0
0	0	0	0	0	0	0	1	0	0	0	0	1	0
0	8	3	0	2	35	16	0	10	0	0	0	0	31
11													

OTU14655	0	0	0	0	0	1	0	0	0	0	0	0	0
0	0	0	0	0	0	0	0	0	0	0	0	0	0
0	0	0	0	0	0	0	0	0	0	0	0	0	0
0	0	0	0	0	0	0	0	0	0	0	0	0	0
0	0	0	0	0	0	0	0	0	0	0	0	0	0
0	0	0	0	0	0	0	0	0	0	0	0	0	0
0	0	0	0	0	0	0	0	0	0	0	0	0	0
0	0	0	0	0	0	0	0	0	0	0	0	0	0
0													
OTU8383	0	0	0	0	0	1	0	0	0	0	0	0	0
0	0	0	0	0	0	0	0	0	0	0	0	0	0
0	0	0	0	0	0	0	0	0	0	0	0	0	0
0	0	0	0	0	0	0	0	0	0	0	0	0	0
0	0	0	0	0	0	0	0	0	0	0	0	0	0
0	0	0	0	0	0	0	0	0	0	0	0	0	0
0	0	0	0	0	0	0	0	0	0	0	0	0	0
0	0	0	0	0	0	0	0	0	0	0	0	0	0
0													
OTU14656	0	0	0	0	0	1	0	0	0	0	0	0	0
0	0	0	0	0	0	0	0	0	0	0	0	0	0
0	0	0	0	0	0	0	0	0	0	0	0	0	0
0	0	0	0	0	0	0	0	0	0	0	0	0	0
0	0	0	0	0	0	0	0	0	0	0	0	0	0
0	0	0	0	0	0	0	0	0	0	0	0	0	0
0	0	0	0	0	0	0	0	0	0	0	0	0	0
0	0	0	0	0	0	0	0	0	0	0	0	0	0
0													
OTU743	0	0	0	0	0	1	1	0	0	1	0	0	0
0	0	0	0	0	0	0	0	0	0	0	0	0	0
0	0	0	0	0	0	0	0	0	0	0	0	0	0
1	0	0	0	0	1	0	0	0	0	0	0	0	0
0	1	0	0	0	0	0	0	0	0	0	0	0	0
0	0	0	0	0	0	0	0	0	0	0	0	0	0

0	0	0	0	0	1	0	0	0	0	0	1	0	0
0	0	0	0	0	1	0	1	0	1	0	0	0	0
0													
OTU592	0	0	0	0	0	2	0	0	0	0	3	0	0
0	0	0	0	0	1	0	1	0	0	0	0	0	6
0	0	0	0	0	0	0	2	0	0	0	0	0	1
0	0	0	7	0	0	0	0	0	0	0	0	0	0
0	0	0	0	0	0	0	0	0	0	0	0	0	0
0	0	0	0	0	0	0	0	0	0	0	0	1	0
0	0	0	0	0	0	0	0	0	0	0	0	0	0
0	0	0	0	0	0	0	0	0	0	0	0	0	0
0													
OTU8380	0	0	0	0	0	1	0	0	0	0	0	0	0
0	0	0	0	0	0	0	0	0	0	0	0	0	0
0	0	0	0	0	0	0	0	0	0	0	0	0	0
0	0	0	0	0	0	0	0	0	0	0	0	0	0
0	0	0	0	0	0	0	0	0	0	0	0	0	0
0	0	0	0	0	0	0	0	0	0	0	0	0	0
0	0	0	0	0	0	0	0	0	0	0	0	0	0
0	0	0	0	0	0	0	0	0	0	0	0	0	0
0													
OTU12019	0	0	0	0	0	1	0	0	0	0	0	0	0
0	0	0	0	0	0	0	0	0	0	0	0	0	0
0	0	0	0	0	0	0	0	0	0	0	0	0	0
0	0	0	0	0	0	0	0	0	0	0	0	0	0
0	0	0	0	0	0	0	0	0	0	0	0	0	0
0	0	0	0	0	0	0	1	0	0	0	0	0	0
0	0	0	0	0	0	0	0	0	0	0	0	0	0
0	0	0	0	0	0	0	0	0	1	0	0	0	0
0													
OTU760	0	0	0	0	0	4	0	0	0	0	0	0	0
0	0	0	0	0	0	4	0	0	0	0	0	0	0
0	0	0	0	0	0	0	0	0	0	0	0	2	0

0	0	0	0	0	0	0	7	0	0	0	0	0	0
0	0	0	0	0	0	0	0	0	0	0	0	1	0
0	0	0	0	0	0	0	0	0	0	0	0	0	0
0	0	0	0	0	0	0	0	0	0	0	0	0	0
0	0	0	0	0	0	0	0	0	0	0	0	0	0
0													
OTU8378	0	0	0	0	0	1	0	0	0	0	0	0	0
0	0	0	0	0	0	0	0	0	0	0	0	0	0
0	0	0	0	0	0	0	0	0	0	0	0	0	0
0	0	0	0	0	0	0	0	0	0	0	0	0	0
0	0	0	0	0	0	0	0	0	0	0	0	0	0
0	0	0	0	0	0	0	0	0	0	0	0	0	0
0	0	0	0	0	0	0	0	0	0	0	0	0	0
0	0	0	0	0	0	0	0	0	0	0	0	0	0
0													
OTU264	0	0	0	0	0	3	0	0	0	0	1	0	1
0	0	0	0	0	0	0	2	0	0	1	0	0	0
0	0	72	0	0	0	0	0	0	0	0	16	2	0
0	0	0	0	0	0	0	1	0	0	10	0	0	0
0	0	0	0	73	33	0	0	0	1	0	56	0	142
11	0	0	0	0	0	0	0	0	0	0	0	0	0
0	0	0	0	0	0	0	0	2	0	0	0	0	0
69	0	3	0	0	0	0	0	0	0	0	0	0	0
0													
OTU12018	0	0	0	0	0	1	0	0	0	0	0	0	0
0	0	0	0	0	0	0	0	0	0	0	0	0	0
0	0	0	0	0	0	0	0	0	0	0	0	0	0
0	0	0	0	0	0	0	0	0	0	0	0	0	0
0	0	0	0	0	0	0	0	0	0	0	0	0	0
0	0	0	0	0	0	0	0	0	0	0	0	0	0
0	0	0	0	0	0	0	0	0	0	0	0	0	0
0	0	0	0	0	0	0	0	0	0	0	0	0	0
0													

OTU14661	0	0	0	0	0	1	0	0	0	0	0	0	0
0	0	0	0	0	0	0	0	0	0	0	0	0	0
0	0	0	0	0	0	0	0	0	0	0	0	0	0
0	0	0	0	0	0	0	0	0	0	0	0	0	0
0	0	0	0	0	0	0	0	0	0	0	0	0	0
0	0	0	0	0	0	0	0	0	0	0	0	0	0
0	0	0	0	0	0	0	0	0	0	0	0	0	0
0	0	0	0	0	0	0	0	0	0	0	0	0	0
0													
OTU1008	0	0	0	0	0	2	0	0	0	0	0	0	0
0	0	0	0	0	0	0	0	0	0	0	0	0	0
0	0	0	0	0	0	0	0	0	0	0	0	0	0
0	0	0	0	0	0	0	0	0	0	0	0	0	0
0	0	0	0	0	0	0	0	0	0	0	0	0	0
0	0	0	0	0	0	0	0	0	0	0	0	0	0
0	0	0	0	0	0	0	0	0	0	0	0	0	0
0	0	0	0	0	0	0	0	0	0	0	0	0	0
0													
OTU10874	0	0	0	0	0	1	0	0	0	0	0	0	0
0	0	0	0	0	0	0	0	0	0	0	0	0	0
0	0	0	0	0	0	0	0	0	0	0	0	0	0
0	0	0	0	0	0	0	0	0	0	0	0	0	0
0	0	0	0	0	0	0	0	0	0	0	0	0	0
0	0	0	0	0	0	0	0	0	0	0	0	0	0
0	0	0	0	0	0	0	0	0	0	0	0	0	0
0	0	0	0	0	0	0	0	0	0	0	0	0	0
0													
OTU15664	0	0	0	0	0	1	0	0	0	0	0	1	0
0	0	0	0	0	0	0	0	0	0	0	0	0	0
0	0	0	0	0	0	0	0	0	0	0	0	0	0
0	1	0	0	0	0	0	0	0	0	0	0	0	0
0	0	0	0	0	0	0	0	0	0	0	0	0	0
0	0	0	0	0	0	0	0	1	0	0	0	0	0

0 0 0 0 0 0 0 0 0 0 0 0 0 0
0 0 0 0 0 0 0 0 0 0 0 0 0 0
0
OTU10872 0 0 0 0 0 1 0 0 0 0 0 0 0
0 0 0 0 0 0 0 0 0 0 0 0 0 0
0 0 0 0 0 0 0 0 0 0 0 0 0 0
0 0 0 0 0 0 0 0 0 0 0 0 0 0
0 0 0 0 0 0 0 0 0 0 0 0 0 0
0 0 0 0 0 0 0 0 0 0 0 0 0 0
0 0 0 0 0 0 0 0 0 0 0 0 0 0
0 0 0 0 0 0 0 0 0 0 0 0 0 0
0
OTU10873 0 0 0 0 0 1 0 0 0 0 0 0 0
0 0 0 0 0 0 0 0 0 0 0 0 0 0
0 0 0 0 0 0 0 0 0 0 0 0 0 0
0 0 0 0 0 0 0 0 0 0 0 0 0 0
0 0 0 0 0 0 0 0 0 0 0 0 0 0
0 0 0 0 0 0 0 0 0 0 0 0 0 0
0 0 0 0 0 0 0 0 0 0 0 0 0 0
0 0 0 0 0 0 0 0 0 0 0 0 0 0
0
OTU1007 0 0 0 0 0 1 0 0 0 0 0 0 0
0 0 0 0 0 0 0 1 1 0 0 0 0 0
0 0 0 0 0 0 0 0 0 0 0 0 0 0
0 0 0 0 0 1 0 0 0 0 0 0 0 0
0 0 0 0 0 0 0 0 0 0 0 0 1 0
0 1 0 0 0 0 0 0 0 0 0 0 0 0
0 0 0 0 0 0 0 0 0 0 0 0 0 0
0 0 0 0 0 0 0 0 0 0 0 0 0 0
0
OTU14660 0 0 0 0 0 1 0 0 0 0 0 0 0
0 0 0 0 0 0 0 0 0 0 0 0 0 0
0 0 0 0 0 0 0 0 0 0 0 0 0 0

0	0	0	0	0	0	0	0	0	0	0	0	0	0
0	0	0	0	0	0	0	0	0	0	0	0	0	0
0	0	0	0	0	0	0	0	0	0	0	0	0	0
0	0	0	0	0	0	0	0	0	0	0	0	0	0
0	0	0	0	0	0	0	0	0	0	0	0	0	0
0													
OTU12007	0	0	0	0	0	1	0	0	0	0	0	0	0
0	0	0	0	0	0	0	0	0	0	0	0	0	0
0	0	0	0	0	0	0	0	0	0	0	0	0	0
0	0	0	0	0	0	0	0	0	0	0	0	0	0
0	0	0	0	0	0	0	0	0	0	0	0	0	0
0	0	0	0	0	0	0	0	0	0	0	0	0	0
0	0	0	0	0	0	0	0	0	0	0	0	0	0
0	0	0	0	0	0	0	0	0	0	0	0	0	0
0													
OTU14662	0	0	0	0	0	1	0	0	0	0	0	0	0
0	0	0	0	0	0	0	0	0	0	0	0	0	0
0	0	0	0	0	0	0	0	0	0	0	0	0	0
0	0	0	0	0	0	0	0	0	0	0	0	0	0
0	0	0	0	0	0	0	0	0	0	0	0	0	0
0	0	0	0	0	0	0	0	0	0	0	0	0	0
0	0	0	0	0	0	0	0	0	0	0	0	0	0
0	0	0	0	0	0	0	0	0	0	0	0	0	0
0													
OTU6659	0	0	0	0	0	1	0	0	0	0	0	0	0
0	2	0	0	0	0	1	0	0	0	0	0	2	2
0	0	1	0	0	0	0	0	0	0	0	0	0	0
0	0	0	0	0	2	0	0	0	1	2	0	0	0
0	0	0	0	0	0	0	0	0	0	0	0	1	0
0	0	0	0	0	0	0	0	0	1	1	0	0	1
1	0	0	0	0	0	0	2	0	0	0	0	0	0
0	0	0	0	0	0	0	0	0	0	0	0	0	0
0													

OTU12006 0 0 0 0 0 1 0 0 0 0 0 0 0
0 0 0 0 0 0 0 0 0 0 0 0 0 0
0 0 0 0 0 0 0 0 0 0 0 0 0 0
0 0 0 0 0 0 0 0 0 0 0 0 0 0
0 0 0 0 0 0 0 0 0 0 0 0 0 0
0 0 0 0 0 0 0 0 0 0 0 0 0 0
0 0 0 0 0 0 0 0 0 0 0 0 0 0
0 0 0 0 0 0 0 0 0 0 0 0 0 0
0
OTU8387 0 0 0 0 0 1 0 0 0 0 0 0 0
0 0 0 0 0 0 0 0 0 0 0 0 0 0
0 0 0 0 0 1 0 0 0 0 0 0 0 0
0 0 0 0 0 0 0 0 0 0 0 0 0 0
0 0 0 0 0 0 0 0 0 0 0 0 0 0
0 0 0 0 0 0 0 0 0 0 0 0 0 0
0 0 0 0 0 0 0 0 0 0 0 0 0 1
0 0 0 0 0 0 0 0 0 0 0 0 0 0
0
OTU7045 0 0 0 0 0 3 0 0 14 0 0 0 0
0 0 0 0 0 0 0 0 0 0 0 0 0 0
0 0 0 0 0 0 0 0 2 0 0 0 0 0
0 0 0 0 0 0 0 0 0 0 0 0 0 0
0 0 0 0 0 0 0 0 0 0 0 0 0 0
0 0 0 0 0 0 0 0 0 0 0 0 0 0
0 0 0 0 0 0 0 0 0 0 0 6 3 0
0 4 0 0 1 0 0 0 1 0 0 0 0 0
2
OTU6013 0 0 0 0 0 1 0 0 0 0 0 0 0
0 0 0 0 0 0 0 0 0 0 0 0 0 0
0 0 0 0 0 0 0 0 0 0 0 0 1 0
0 0 0 2 0 0 0 0 0 0 0 0 0 0
0 0 0 0 0 0 0 0 0 0 0 0 0 0
0 0 2 0 0 0 0 0 0 0 0 0 0 0

0	0	0	0	0	0	0	0	0	0	0	0	0	0
0	0	0	0	1	0	0	0	0	0	0	0	0	0
0													
OTU8388	0	0	0	0	0	1	0	0	0	0	0	0	0
0	0	0	0	0	0	0	0	0	0	0	0	0	0
0	0	0	0	0	0	0	0	0	0	0	0	0	0
0	0	0	0	0	0	0	0	0	0	0	0	0	0
0	0	0	0	0	0	0	0	0	0	0	0	0	0
0	0	0	0	0	0	0	0	0	0	0	0	0	0
0	0	0	0	0	0	0	0	0	0	0	0	0	0
0	0	0	0	0	0	0	0	0	0	0	0	0	0
0													
OTU12010	0	0	0	0	0	1	0	0	0	0	0	0	0
0	0	0	0	0	0	0	0	0	0	0	0	0	0
0	0	0	0	0	0	0	0	0	0	0	0	0	0
0	0	0	0	0	0	0	0	0	0	0	0	0	0
0	0	0	0	0	0	0	0	0	0	0	0	0	0
0	0	0	0	0	0	0	0	0	0	0	0	0	0
0	0	0	0	0	0	0	0	0	0	0	0	0	0
0	0	0	0	0	0	0	0	0	0	0	0	0	0
0													
OTU640	0	0	0	0	0	1	0	0	1	17	0	0	0
0	0	0	0	0	0	0	0	0	0	0	0	1	0
3	0	0	0	0	0	0	0	0	0	0	0	0	0
0	0	0	0	0	1	0	0	0	0	0	0	0	0
0	2	0	0	0	0	0	0	0	0	0	0	1	0
0	0	0	0	1	0	0	0	0	0	0	0	0	0
0	0	0	0	0	0	0	1	0	0	0	1	0	0
0	0	3	1	2	0	1	1	0	1	0	0	0	1
0													
OTU361	0	0	0	0	0	1	0	0	0	0	14	0	50
9	0	0	0	0	2	0	0	0	6	0	0	0	0
0	0	1	0	0	1	0	1	0	0	0	3	0	0

0	0	0	0	0	0	1	0	0	0	0	0	0	0
0	0	0	0	0	0	0	1	0	0	0	0	0	0
0	0	0	1	0	0	0	0	0	0	1	0	1	0
0	0	3	0	2	0	0	0	14	0	0	0	0	0
2	0	0	0	0	0	0	0	1	0	0	9	0	0
0													
OTU11584	0	0	0	0	0	1	0	0	1	0	0	0	0
1	0	1	0	0	0	0	0	0	1	0	0	0	0
0	0	0	0	0	0	0	0	0	0	0	0	1	0
0	1	0	0	0	0	0	0	0	0	0	0	0	0
1	0	0	0	0	0	0	0	0	0	0	0	0	0
1	0	0	0	0	0	0	0	0	0	0	0	0	0
0	0	0	0	0	0	0	0	0	0	0	0	0	0
0	0	0	0	0	0	0	0	0	1	0	0	0	0
1													
OTU12008	0	0	0	0	0	1	0	0	0	0	0	0	0
0	0	0	0	0	0	0	0	0	0	0	0	0	0
0	0	0	0	0	0	0	0	0	0	0	0	0	0
0	0	0	0	0	0	0	0	0	0	0	0	0	0
0	0	0	0	0	0	0	0	0	0	0	0	0	0
0	0	0	0	0	0	0	0	0	0	0	0	0	0
0	0	0	0	0	0	0	0	0	0	0	0	0	0
0	0	0	0	0	0	0	0	0	0	0	0	0	0
0													
OTU12009	0	0	0	0	0	1	0	0	0	0	0	0	0
0	0	0	0	0	0	0	0	0	0	0	0	0	0
0	0	0	0	0	0	0	0	0	0	0	0	0	0
0	0	0	0	0	0	0	0	0	0	0	0	0	0
0	0	0	0	0	0	0	0	0	0	0	0	0	0
0	0	0	0	0	0	0	0	0	0	0	0	0	0
0	0	0	0	0	0	0	0	0	0	0	0	0	0
0	0	0	0	0	0	0	0	0	0	0	0	0	0
0													

OTU7181 0 0 0 0 0 1 0 0 0 0 0 0 0
0 0 0 0 0 0 0 0 0 0 0 0 0 0
0 0 0 0 0 0 0 0 0 0 0 0 0 0
0 0 0 0 0 0 0 0 0 0 0 0 0 0
0 0 0 0 0 0 0 0 0 0 0 0 0 0
0 0 0 0 0 0 0 0 0 1 0 0 0 0
0 0 0 0 0 0 0 0 0 0 0 0 0 0
0 0 0 0 0 0 0 0 0 0 0 0 0 0
0
OTU14658 0 0 0 0 0 1 0 0 0 0 0 0 0
0 0 0 0 0 0 0 0 0 0 0 0 0 0
0 0 0 0 0 0 0 0 0 0 0 0 0 0
0 0 0 0 0 0 0 0 0 0 0 0 0 0
0 0 0 0 0 0 0 0 0 0 0 0 0 0
0 0 0 0 0 0 0 0 0 0 0 0 0 0
0 0 0 0 0 0 0 0 0 0 0 0 0 0
0 0 0 0 0 0 0 0 0 0 0 0 0 0
0
OTU12003 0 0 0 0 0 1 0 0 0 0 0 0 0
0 0 0 0 0 0 0 0 0 0 0 0 0 0
0 0 0 0 0 0 0 0 0 0 0 0 0 0
0 0 0 0 0 0 0 0 0 0 0 0 0 0
0 0 0 0 0 0 0 0 0 0 0 0 0 0
0 0 0 0 0 0 0 0 0 0 0 0 0 0
0 0 0 0 0 0 0 0 0 0 0 0 0 0
0 0 0 0 0 0 0 0 0 0 0 0 0 0
0
OTU12004 0 0 0 0 0 1 0 0 0 0 0 0 0
0 0 0 0 0 0 0 0 0 0 0 0 0 0
0 0 0 0 0 0 0 0 0 0 0 0 0 0
0 0 0 0 0 0 0 0 0 0 0 0 0 0
0 0 0 0 0 0 0 0 0 0 0 0 0 0
0 0 0 0 0 0 0 0 0 0 0 0 0 0

0 0 0 0 0 0 0 0 0 0 0 0 0 0
0 0 0 0 0 0 0 0 0 0 0 0 0 0
0
OTU15732 0 0 0 0 0 1 0 0 0 0 0 0 0
0 0 0 0 0 0 0 0 0 0 0 0 0 0
0 0 0 0 0 0 0 0 0 0 0 0 0 0
0 0 0 0 0 0 0 0 0 0 0 0 0 0
0 0 0 0 0 0 0 0 0 0 0 0 0 0
0 0 0 0 0 0 0 0 0 0 0 0 0 0
0 0 0 0 0 0 0 0 0 0 0 0 0 0
0 0 0 0 0 0 0 0 0 0 0 0 0 0
0
OTU8386 0 0 0 0 0 1 0 0 0 0 0 0 0
0 0 0 0 0 0 0 0 0 0 0 0 0 0
0 0 0 0 0 0 0 0 0 0 0 0 0 0
0 0 0 0 0 0 0 0 0 0 0 0 0 0
0 0 0 0 0 0 0 0 0 0 0 0 0 0
0 0 0 0 0 0 0 0 0 0 0 0 0 0
0 0 0 0 0 0 0 0 0 0 0 0 0 0
0 0 0 0 0 0 0 0 0 0 0 0 0 0
0
OTU14659 0 0 0 0 0 1 0 0 0 0 0 0 0
0 0 0 0 0 0 0 0 0 0 0 0 0 0
0 0 0 0 0 0 0 0 0 0 0 0 0 0
0 0 0 0 0 0 0 0 0 0 0 0 0 0
0 0 0 0 0 0 0 0 0 0 0 0 0 0
0 0 0 0 0 0 0 0 0 0 0 0 0 0
0 0 0 0 0 0 0 0 0 0 0 0 0 0
0 0 0 0 0 0 0 0 0 0 0 0 0 0
0
OTU12002 0 0 0 0 0 1 0 0 0 0 0 0 0
0 0 0 0 0 0 0 0 0 0 0 0 0 0
0 0 0 0 0 0 0 0 0 0 0 0 0 0

0	0	0	0	0	0	0	0	0	0	0	0	0	0
0	0	0	0	0	0	0	0	0	0	0	0	0	0
0	0	0	0	0	0	0	0	0	0	0	0	0	0
0	0	0	0	0	0	0	0	0	0	0	0	0	0
0	0	0	0	0	0	0	0	0	0	0	0	0	0
0													
OTU9675	0	0	0	0	0	1	0	0	0	0	0	0	0
0	0	0	0	0	0	0	0	0	0	0	0	0	0
0	0	0	0	0	0	1	0	0	0	0	0	0	0
0	0	0	0	0	0	0	1	0	0	0	0	0	0
0	0	0	0	0	0	0	0	1	0	0	0	0	1
0	0	0	0	0	0	0	1	0	0	0	1	0	0
0	1	0	0	0	0	0	0	0	0	0	0	0	1
0	0	0	0	0	0	1	0	1	0	0	1	0	0
0													
OTU12005	0	0	0	0	0	1	0	0	0	0	0	0	0
0	0	0	0	0	0	0	0	0	0	0	0	0	0
0	0	0	0	0	0	0	0	0	0	0	0	0	0
0	0	0	0	0	0	0	0	0	0	0	0	0	0
0	0	0	0	0	0	0	0	0	0	0	0	0	0
0	0	0	0	0	0	0	0	0	0	0	0	0	0
0	0	0	0	0	0	0	0	0	0	0	0	0	0
0	0	0	0	0	0	0	0	0	0	0	0	0	0
0													
OTU8883	0	0	0	0	0	2	1	0	1	0	0	0	0
0	0	0	0	0	0	3	0	0	0	0	0	7	0
1	0	0	0	0	0	0	0	0	0	0	0	1	0
2	0	0	1	0	0	0	3	0	0	0	0	0	0
0	0	0	1	0	0	0	0	0	0	0	0	0	0
0	0	0	0	0	0	0	0	0	0	0	0	0	0
0	0	0	0	0	0	0	1	0	0	0	0	0	0
0	0	0	0	0	0	0	0	0	0	0	0	0	0
0													

OTU8385	0	0	0	0	0	1	0	0	0	0	0	0	0
0	0	0	0	0	0	0	0	0	0	0	0	0	0
0	0	0	0	0	0	0	0	0	0	0	0	0	0
0	0	0	0	0	0	0	0	0	0	0	0	0	0
0	0	0	0	0	0	0	0	0	0	0	0	0	0
0	0	0	0	0	0	0	0	0	0	0	0	0	0
0	0	0	0	0	0	0	0	0	0	0	0	0	0
0	0	0	0	0	0	0	0	0	0	0	0	0	0
0													
OTU1360	0	0	0	0	0	1	0	0	2	0	0	0	0
0	0	0	0	0	0	0	0	0	0	0	0	0	0
0	0	0	1	0	0	0	0	0	0	0	0	0	0
0	0	0	0	1	1	0	0	0	0	0	0	0	0
0	1	0	0	1	0	0	0	0	1	0	0	0	1
0	0	0	0	0	0	0	0	0	0	0	0	0	0
0	0	0	0	0	0	0	0	0	0	0	0	0	0
0	0	0	0	1	1	3	1	0	1	0	0	0	0
0													
OTU8371	0	0	0	0	0	1	0	0	0	0	0	0	0
0	0	0	0	0	0	0	0	0	0	0	0	0	0
0	0	0	0	0	0	0	0	0	0	0	0	0	0
0	0	0	0	0	0	0	0	0	0	0	0	0	0
0	0	0	0	0	0	0	0	0	0	0	0	0	0
0	0	0	0	0	0	0	0	0	0	0	0	0	0
0	0	0	0	0	0	0	0	0	0	0	0	0	0
0	0	0	0	0	0	0	0	0	0	0	0	0	0
0													
OTU1580	0	0	0	0	0	1	0	0	0	0	0	0	5
0	0	2	0	0	2	0	1	0	0	2	0	0	0
0	0	0	0	0	0	0	3	0	0	0	0	0	1
0	2	0	0	0	0	4	0	0	0	0	0	0	1
0	0	0	0	0	0	0	0	0	0	0	0	0	0
0	0	0	4	0	0	0	0	0	0	0	0	0	0

0	0	3	0	0	0	0	0	0	8	9	0	0	1
10	0	0	0	0	0	0	0	1	0	1	6	3	0
0													
OTU3812	0	0	0	0	0	1	0	0	0	0	0	0	0
0	1	0	0	0	0	1	0	0	0	0	0	0	0
0	0	1	0	0	1	1	0	0	0	0	1	1	0
0	0	0	0	0	1	0	0	0	1	0	0	0	0
0	0	0	0	0	0	0	0	1	1	2	0	0	0
0	0	0	0	0	0	0	0	0	0	0	1	0	1
0	0	0	0	0	0	0	0	0	1	0	1	0	0
0	1	1	0	0	0	0	0	0	0	1	0	1	0
1													
OTU15721	0	0	0	0	0	1	4	2	0	3	0	5	0
0	2	0	0	0	0	1	0	0	0	0	0	0	0
0	0	0	0	0	0	0	2	0	1	0	0	0	0
2	5	0	0	0	0	0	0	0	0	0	0	0	0
0	0	0	0	0	0	0	0	0	0	0	0	0	0
0	0	0	1	0	0	0	0	2	0	0	0	1	0
0	0	1	0	1	0	0	1	0	0	0	0	0	0
0	0	0	0	0	0	0	0	0	1	3	1	3	0
0													
OTU12023	0	0	0	0	0	2	0	0	0	0	0	0	0
0	0	0	0	0	0	0	0	0	0	0	0	0	0
0	0	0	0	0	0	0	0	0	0	0	0	0	0
0	0	0	0	0	0	0	0	0	0	0	0	0	0
0	0	0	0	0	0	0	0	0	0	0	0	0	0
1	0	0	0	0	0	0	0	0	0	0	0	0	0
0	0	0	0	0	2	0	0	0	0	0	0	0	0
0	0	0	0	0	0	0	0	0	0	0	0	0	0
0													
OTU15738	0	0	0	0	0	1	0	0	0	0	0	0	0
0	0	0	0	0	0	0	0	0	0	0	0	0	0
0	0	0	0	0	0	0	0	0	0	0	0	0	0

0	0	0	0	0	0	0	0	0	0	0	0	0	0
0	0	0	0	0	0	0	0	0	0	0	0	0	0
0	0	0	0	0	0	0	0	0	0	0	0	0	0
0	0	0	0	0	0	0	0	0	0	0	0	0	0
0	0	0	0	0	0	0	0	0	0	0	0	0	0
0													
OTU12363	0	0	0	0	0	2	0	0	0	1	0	3	0
0	5	0	0	0	0	0	0	0	0	0	0	0	0
0	0	0	0	0	0	0	0	0	0	0	0	0	0
0	0	0	1	0	0	0	0	0	0	0	0	0	0
0	0	0	0	0	0	0	0	0	0	0	0	0	0
0	0	0	0	2	0	0	0	0	0	0	0	0	0
0	0	0	0	0	0	0	0	0	0	0	0	3	0
0	0	0	0	0	0	0	0	0	1	0	0	0	0
0													
OTU6692	0	0	0	0	0	1	0	0	0	0	0	0	0
0	0	0	0	3	0	0	0	0	0	3	0	0	0
0	0	0	0	1	0	0	0	0	1	0	0	0	0
0	0	0	1	0	0	0	0	0	0	0	0	0	0
0	1	1	0	0	0	1	0	0	0	0	0	0	0
0	0	0	0	0	0	0	0	0	0	0	0	0	0
0	0	0	0	0	0	1	0	0	0	0	0	0	0
0	0	0	0	0	0	0	0	0	0	0	0	0	0
0													
OTU15737	0	0	0	0	0	1	0	0	0	0	0	0	0
0	0	0	0	0	0	0	0	0	0	0	0	0	0
0	0	0	0	0	0	0	0	0	0	0	0	0	0
0	0	0	0	0	0	0	0	0	0	0	0	0	0
0	0	0	0	0	0	0	0	0	0	0	0	0	0
0	0	0	0	0	0	0	0	0	0	0	0	0	0
0	0	0	0	0	0	0	0	0	0	0	0	0	0
0	0	0	0	0	0	0	0	0	0	0	0	0	0
0													

OTU803	0	0	0	0	0	3	0	0	0	0	0	0	0
0	0	0	0	0	0	0	4	0	0	0	0	0	1
0	0	0	0	0	0	0	0	0	0	0	0	0	0
0	0	0	0	0	0	0	0	0	0	0	0	0	0
0	0	0	0	0	0	0	0	0	0	0	0	0	0
0	0	0	0	0	0	0	0	0	0	0	0	0	0
0	0	0	0	0	0	0	0	0	0	0	0	0	0
0	0	0	0	1	0	0	0	0	0	0	0	0	0
0													
OTU12013	0	0	0	0	0	1	0	0	0	0	0	0	0
0	0	0	0	0	0	0	0	0	0	0	0	0	0
0	0	0	0	0	0	0	0	0	0	0	0	0	0
0	0	0	0	0	0	0	0	0	0	0	0	0	0
0	0	0	0	0	0	0	0	0	0	0	0	0	0
0	0	0	0	0	0	0	0	0	0	0	0	0	0
0	0	0	0	0	0	0	0	0	0	0	0	0	0
0	0	0	0	0	0	0	0	0	0	0	0	0	0
0													
OTU14646	0	0	0	0	0	1	0	0	0	0	0	0	0
0	0	0	0	0	0	0	0	0	0	0	0	0	0
0	0	0	0	0	0	0	0	0	0	0	0	0	0
0	0	0	0	0	0	0	0	0	0	0	0	0	0
0	0	0	0	0	0	0	0	0	0	0	0	0	0
0	0	0	0	0	0	0	0	0	0	0	0	0	0
0	0	0	0	0	0	0	0	0	0	0	0	0	0
0	0	0	0	0	0	0	0	0	0	0	0	0	0
0													
OTU6094	0	0	0	0	0	1	0	0	9	0	1	0	3
0	0	3	13	0	0	0	0	53	0	1	0	12	12
13	1	0	0	3	0	30	2	1	0	3	0	17	0
0	0	0	0	26	27	1	112	3	41	34	13	0	74
2	4	9	52	86	68	0	116	145	17	99	60	18	149
73	0	4	0	1	0	0	89	0	3	0	4	0	0

0	108	0	0	0	0	0	31	0	0	0	0	0	2
0	0	0	0	0	0	0	0	0	0	2	0	29	0
0													
OTU13954	0	0	0	0	0	1	1	0	0	0	2	0	0
0	0	0	1	0	1	0	0	0	1	0	1	0	0
0	0	0	0	0	0	0	0	0	0	0	2	0	0
0	1	0	0	0	0	0	0	0	0	2	0	2	0
0	0	0	0	0	0	1	0	0	1	0	0	0	2
0	0	0	0	0	0	0	0	0	0	1	0	0	0
0	0	0	0	0	0	0	0	0	0	0	0	0	0
0	0	0	0	0	0	0	0	0	0	0	0	0	0
0													
OTU5045	0	0	0	0	0	2	0	0	0	0	0	0	0
0	0	0	0	0	0	0	0	0	0	0	0	0	0
0	0	0	0	0	0	0	0	0	0	0	0	0	0
0	0	0	1	1	0	0	0	0	0	0	0	0	0
0	2	0	0	1	0	0	0	0	0	0	2	0	1
0	0	0	0	0	0	0	0	1	0	0	0	0	0
0	0	0	0	1	0	0	0	0	0	0	0	0	0
0	0	0	0	0	0	0	0	0	0	2	0	0	0
0													
OTU15739	0	0	0	0	0	1	0	0	0	0	0	0	0
0	0	0	0	0	0	0	0	0	0	0	0	0	0
0	0	0	0	0	0	0	0	0	0	0	0	0	0
0	0	0	0	0	0	0	0	0	0	0	0	0	0
0	0	0	0	0	0	0	0	0	0	0	0	0	0
0	0	0	0	0	0	0	0	0	0	0	0	0	0
0	0	0	0	0	0	0	0	0	0	0	0	0	0
0	0	0	0	0	0	0	0	0	0	0	0	0	0
0													
OTU531	0	0	0	0	0	1	0	0	0	3	0	0	0
0	1	0	0	0	0	0	0	0	0	0	0	0	0
0	0	0	0	0	0	0	0	1	0	0	0	0	0

0	0	0	0	0	0	0	0	0	0	0	0	0	0
0	0	0	0	0	0	0	0	0	0	0	0	0	0
1	2	0	0	0	0	0	1	0	0	0	11	1	5
0	0	0	0	0	0	0	0	0	0	0	50	0	0
0	68	0	0	15	3	0	18	0	0	1	1	0	0
0													
OTU12011	0	0	0	0	0	1	0	0	0	0	0	0	0
0	0	0	0	0	0	0	0	0	0	0	0	0	0
0	0	0	0	0	0	0	0	0	0	0	0	0	0
0	0	0	0	0	0	0	0	0	0	0	0	0	0
0	0	0	0	0	0	0	0	0	0	0	0	0	0
0	0	0	0	0	0	0	0	0	0	0	0	0	0
0	0	0	0	0	0	0	0	0	0	0	0	0	0
0	0	0	0	0	0	0	0	0	0	0	0	0	0
0													
OTU209	0	0	0	0	0	1	1	0	0	0	4	1	6
9	0	2	5	12	20	3	27	3	5	3	17	6	13
1	1	9	1	9	17	50	6	6	14	3	59	0	1
0	0	0	0	6	0	8	0	0	0	1	0	0	11
0	0	10	0	11	0	3	0	0	7	1	1	0	5
0	0	0	0	0	1	0	0	0	0	1	0	0	0
0	0	3	0	0	0	8	0	20	0	0	0	0	0
0	0	0	1	0	0	0	0	0	1	0	0	9	0
0													
OTU6332	0	0	0	0	0	1	0	0	0	0	0	0	0
0	0	0	0	1	0	0	0	0	0	1	0	0	0
0	0	0	0	0	0	0	0	0	0	0	0	0	0
0	1	0	0	0	1	0	0	0	0	0	0	0	0
0	0	0	0	0	0	0	0	0	0	0	0	0	0
0	0	0	0	0	0	0	0	0	0	0	0	0	0
0	0	0	0	0	0	0	0	0	0	0	0	0	0
0	0	0	0	0	0	0	0	0	0	0	0	0	0
0													

OTU8373	0	0	0	0	0	1	0	0	0	0	0	0	0
0	0	0	0	0	0	0	0	0	0	0	0	0	0
0	0	0	0	0	0	0	0	0	0	0	0	0	0
0	0	0	0	0	0	0	0	0	0	0	0	0	0
0	0	0	0	0	0	0	0	0	0	0	0	0	0
0	0	0	0	0	0	0	0	0	0	0	0	0	0
0	0	0	0	0	0	0	0	0	0	0	0	0	0
0	0	0	0	0	0	0	0	0	0	0	0	0	0
0													
OTU201	0	0	0	0	0	1	0	0	0	0	45	0	45
11	0	4	0	1	118	0	0	0	0	0	0	0	1
0	0	9	0	0	3	0	12	0	0	0	0	0	34
0	4	0	0	0	0	9	0	0	0	0	0	0	0
0	0	0	1	0	0	0	2	0	0	0	0	0	0
0	0	0	1	0	0	0	0	0	0	0	0	2	0
1	0	0	0	4	0	0	0	138	0	11	0	0	37
2	0	0	0	0	1	0	0	1	0	0	4	2	0
0													
OTU234	0	0	0	0	0	1	1	0	1	2	0	1	0
0	1	0	0	0	0	0	0	0	0	0	0	0	0
0	0	0	0	0	0	0	0	17	0	0	0	0	0
0	0	0	0	0	3	0	0	0	0	0	0	0	0
0	0	0	0	0	3	0	7	0	0	1	0	2	0
0	0	25	0	2	0	0	5	0	0	2	0	0	4
0	1	0	6	0	1	0	112	0	0	0	0	1	0
0	1	5	3	0	1	14	0	31	14	0	0	0	0
0													
OTU1988	0	0	0	0	0	1	0	1	0	0	9	2	1
1	0	7	0	5	0	0	1	0	1	0	0	0	1
2	0	0	0	0	0	0	0	0	2	1	0	0	0
2	0	0	0	0	1	3	5	0	0	0	0	0	1
0	4	2	0	0	1	0	0	1	0	0	1	1	0
0	3	0	0	0	0	0	0	0	0	0	0	2	0

2	0	0	0	1	1	3	0	0	0	2	0	10	2
2	1	8	0	5	0	6	11	14	1	0	4	0	0
0													
OTU1179	0	0	0	0	0	1	0	0	0	0	0	0	0
0	0	0	0	0	0	0	0	0	5	0	0	0	0
0	0	0	0	0	0	0	1	0	0	0	3	0	0
0	0	0	0	0	0	1	0	0	0	0	0	0	0
0	0	0	0	0	0	0	0	0	0	0	0	0	0
0	0	0	0	0	0	0	0	0	0	0	0	0	0
0	0	0	0	0	0	0	0	1	0	0	0	0	0
0	0	0	0	0	0	0	0	0	0	0	0	0	0
0													
OTU329	0	0	0	0	0	1	0	0	0	0	0	0	0
0	0	0	56	0	0	0	0	1	0	0	0	1	1
0	6	0	0	7	0	3	0	1	0	9	0	0	0
0	0	0	0	1	0	0	0	1	0	2	0	0	0
2	0	0	0	0	1	1	0	0	0	2	2	0	29
7	0	2	2	0	0	1	0	0	1	0	0	0	0
20	26	0	0	0	0	1	1	0	0	0	0	0	0
0	0	0	0	0	0	0	0	0	0	0	0	0	0
0													
OTU8375	0	0	0	0	0	1	0	0	0	0	0	0	0
0	0	0	0	0	0	0	0	0	0	0	0	0	0
0	0	0	0	0	0	0	0	0	0	0	0	0	0
0	0	0	0	0	0	0	0	0	0	0	0	0	0
0	0	0	0	0	0	0	0	0	0	0	0	0	0
0	0	0	0	0	0	0	0	0	0	0	0	0	0
0	0	0	0	0	0	0	0	0	0	0	0	0	0
0	0	0	0	0	0	0	0	0	0	0	0	0	0
0													
OTU15736	0	0	0	0	0	1	0	0	0	0	0	0	0
0	0	0	0	0	0	0	0	0	0	0	0	0	0
0	0	0	0	0	0	0	0	0	0	0	0	0	0

0	0	0	0	0	0	0	0	0	0	0	0	0	0
0	0	0	0	0	0	0	0	0	0	0	0	0	0
0	0	0	0	0	0	0	0	0	0	0	0	0	0
0	0	0	0	0	0	0	0	0	0	0	0	0	0
0	0	0	0	0	0	0	0	0	0	0	0	0	0
0													
OTU16709	0	0	0	0	0	1	0	0	0	0	0	0	0
0	0	0	0	0	0	0	0	0	0	0	0	0	0
0	0	0	0	0	0	0	0	0	0	0	0	0	0
0	0	0	0	0	0	0	1	0	0	0	0	0	0
0	0	0	0	0	0	0	0	0	0	0	0	0	0
0	0	0	0	0	0	0	0	0	0	0	0	0	1
0	0	0	0	0	0	0	0	0	0	0	0	0	0
0	0	0	0	0	0	0	0	0	0	0	0	0	0
0													
OTU907	0	0	0	0	0	1	0	0	0	0	0	0	0
0	0	0	0	0	0	0	0	0	0	0	0	0	0
0	0	0	0	0	0	0	0	0	0	0	0	1	0
0	0	0	0	0	0	0	0	0	0	0	0	0	0
0	0	0	0	0	0	0	0	0	0	0	0	0	0
0	0	0	0	0	0	0	0	0	0	0	1	0	0
0	0	0	0	0	0	0	0	0	0	0	0	0	0
0	0	0	0	0	0	0	0	0	0	0	0	0	0
0													
OTU356	0	0	0	0	0	2	0	0	0	0	0	0	1
0	1	0	1	0	0	0	0	0	0	0	0	0	0
0	0	0	0	0	0	0	0	0	0	0	0	0	0
0	0	0	0	0	0	1	0	1	0	0	1	0	0
0	1	0	1	0	0	0	0	0	0	0	0	1	0
0	1	0	0	0	0	0	0	0	0	0	0	0	0
0	0	0	0	0	0	0	0	0	0	1	33	67	0
0	5	2	55	7	1	7	3	6	26	0	0	0	12
8													

OTU15734 0 0 0 0 0 1 0 0 0 0 0 0 0
0 0 0 0 0 0 0 0 0 0 0 0 0 0
0 0 0 0 0 0 0 0 0 0 0 0 0 0
0 0 0 0 0 0 0 0 0 0 0 0 0 0
0 0 0 0 0 0 0 0 0 0 0 0 0 0
0 0 0 0 0 0 0 0 0 0 0 0 0 0
0 0 0 0 0 0 0 0 0 0 0 0 0 0
0 0 0 0 0 0 0 0 0 0 0 0 0 0
0
OTU7872 0 0 0 0 0 1 0 1 0 1 0 0 0
0 0 0 0 0 0 0 0 0 0 0 0 0 0
0 0 0 0 0 0 0 0 0 0 0 0 0 0
0 0 0 0 0 0 0 0 0 0 0 0 0 0
0 0 0 0 0 0 0 0 0 0 0 0 0 0
0 0 0 0 0 0 0 0 0 0 0 0 0 0
0 0 0 0 0 0 0 0 0 0 0 0 0 0
0 0 0 0 0 0 0 0 0 0 0 0 0 0
0
OTU14653 0 0 0 0 0 1 0 0 0 0 0 0 0
0 0 0 0 0 0 0 0 0 0 0 0 0 0
0 0 0 0 0 0 0 0 0 0 0 0 0 0
0 0 0 0 0 0 0 0 0 0 0 0 0 0
0 0 0 0 0 0 0 0 0 0 0 0 0 0
0 0 0 0 0 0 0 0 0 0 0 0 0 0
0 0 0 0 0 0 0 0 0 0 0 0 0 0
0 0 0 0 0 0 0 0 0 0 0 0 0 0
0
OTU14652 0 0 0 0 0 1 0 0 0 0 0 0 0
0 0 0 0 0 0 0 0 0 0 0 0 0 0
0 0 0 0 0 0 0 0 0 0 0 0 0 0
0 0 0 0 0 0 0 0 0 0 0 0 0 0
0 0 0 0 0 0 0 0 0 0 0 0 0 0
0 0 0 0 0 0 0 0 0 0 0 0 0 0

0 0 0 0 0 0 0 0 0 0 0 0 0 0
0 0 0 0 0 0 0 0 0 0 0 0 0 0
0
OTU12034 0 0 0 0 0 1 0 0 0 0 0 0 0
0 0 0 0 0 0 0 0 0 0 0 0 0 0
0 0 0 0 0 0 0 0 0 0 0 0 0 0
0 0 0 0 0 0 0 0 0 0 0 0 0 0
0 0 0 0 0 0 0 0 0 0 0 0 0 0
0 0 0 0 0 0 0 0 0 0 0 0 0 0
0 0 0 0 0 0 0 0 0 0 0 0 0 0
0 0 0 0 0 0 0 0 0 0 0 0 0 0
0
OTU15735 0 0 0 0 0 1 0 0 0 0 0 0 0
0 0 0 0 0 0 0 0 0 0 0 0 0 0
0 0 0 0 0 0 0 0 0 0 0 0 0 0
0 0 0 0 0 0 0 0 0 0 0 0 0 0
0 0 0 0 0 0 0 0 0 0 0 0 0 0
0 0 0 0 0 0 0 0 0 0 0 0 0 0
0 0 0 0 0 0 0 0 0 0 0 0 0 0
0 0 0 0 0 0 0 0 0 0 0 0 0 0
0
OTU14651 0 0 0 0 0 1 0 0 0 0 0 0 0
0 0 0 0 0 0 0 0 0 0 0 0 0 0
0 0 0 0 0 0 0 0 0 0 0 0 0 0
0 0 0 0 0 0 0 0 0 0 0 0 0 0
0 0 0 0 0 0 0 0 0 0 0 0 0 0
0 0 0 0 0 0 0 0 0 0 0 0 0 0
0 0 0 0 0 0 0 0 0 0 0 0 0 0
0 0 0 0 0 0 0 0 0 0 0 0 0 0
0
OTU12035 0 0 0 0 0 1 0 0 0 0 0 0 0
0 0 0 0 0 0 0 0 0 0 0 0 0 0
0 0 0 0 0 0 0 0 0 0 0 0 0 0

0	0	0	0	0	0	0	0	0	0	0	0	0	0
0	0	0	0	0	0	0	0	0	0	0	0	0	0
0	0	0	0	0	0	0	0	0	0	0	0	0	0
0	0	0	0	0	0	0	0	0	0	0	0	0	0
0	0	0	0	0	0	0	0	0	0	0	0	0	0
0													
OTU12032	0	0	0	0	0	1	0	0	0	0	0	0	0
0	0	0	0	0	0	0	0	0	0	0	0	0	0
0	0	0	0	0	0	0	0	0	0	0	0	0	0
0	0	0	0	0	0	0	0	0	0	0	0	0	0
0	0	0	0	0	0	0	0	0	0	0	0	0	0
0	0	0	0	0	0	0	0	0	0	0	0	0	0
0	0	0	0	0	0	0	0	0	1	0	0	0	0
0	0	0	0	0	0	0	0	0	0	0	0	0	0
0													
OTU7890	0	0	0	0	0	1	0	1	0	0	0	0	0
0	0	0	0	0	0	0	0	0	0	1	0	1	0
0	1	0	0	0	0	1	0	0	0	1	0	0	0
0	0	0	0	0	1	0	2	1	1	0	0	0	0
0	0	0	0	1	1	0	0	1	0	0	0	0	0
0	0	0	0	0	0	0	0	0	0	0	0	1	0
0	0	0	0	0	0	0	0	0	0	0	0	0	0
0	0	0	0	0	0	0	0	0	0	0	0	0	0
0													
OTU15729	0	0	0	0	0	1	0	0	0	0	0	0	0
0	0	0	0	0	0	0	0	0	0	0	0	0	0
0	0	0	0	0	0	0	0	0	0	0	0	0	0
0	0	0	0	0	0	0	0	0	0	0	0	0	0
0	0	0	0	0	0	0	0	0	0	0	0	0	0
0	0	0	0	0	0	0	0	0	0	0	0	0	0
0	0	0	0	0	0	0	0	0	0	0	0	0	0
0	0	0	0	0	0	0	0	0	0	0	0	0	0
0													

OTU12033	0	0	0	0	0	1	0	0	0	0	0	0	0
0	0	0	0	0	0	0	0	0	0	0	0	0	0
0	0	0	0	0	0	0	0	0	0	0	0	0	0
0	0	0	0	0	0	0	0	0	0	0	0	0	0
0	0	0	0	0	0	0	0	0	0	0	0	0	0
0	0	0	0	0	0	0	0	0	0	0	0	0	0
0	0	0	0	0	0	0	0	0	0	0	0	0	0
0	0	0	0	0	0	0	0	0	0	0	0	0	0
0													
OTU12388	0	0	0	0	0	1	0	0	0	0	12	1	9
27	0	2	1	1	35	0	1	0	1	0	0	0	0
0	0	23	7	4	1	0	2	0	0	0	0	0	10
1	0	8	1	3	0	2	0	3	0	1	0	11	0
10	0	9	0	1	2	6	2	0	2	3	1	0	1
0	0	0	0	0	0	8	0	0	0	0	0	1	0
1	1	1	0	0	0	0	0	14	3	4	0	0	11
4	2	0	0	0	0	0	0	0	0	0	2	4	0
0													
OTU930	0	0	0	0	0	2	0	1	0	0	0	0	0
0	0	0	0	0	0	0	0	0	0	0	0	0	0
0	0	0	0	0	0	0	0	0	0	0	0	0	0
0	0	0	0	0	0	0	0	0	0	0	0	0	0
0	0	0	0	0	0	0	0	0	0	0	0	0	0
0	0	0	0	0	0	0	0	0	0	0	0	0	0
0	0	0	0	0	0	0	0	0	0	0	0	0	0
0	0	0	0	0	0	0	0	0	0	0	0	0	0
0													
OTU8407	0	0	0	0	0	1	0	0	0	0	0	0	0
0	0	0	0	0	0	0	0	0	0	0	0	0	0
0	0	0	0	0	0	0	0	0	0	0	0	0	0
0	0	0	0	0	0	0	0	0	0	0	0	0	0
0	0	0	0	0	0	0	0	0	0	0	0	0	0
0	0	0	0	0	0	0	0	0	0	0	0	0	0

0	0	0	0	0	0	0	0	0	0	0	0	0	0
0	0	0	0	0	0	0	0	0	0	0	0	0	0
0													
OTU8661	0	0	0	0	0	2	0	0	0	0	1	3	0
0	1	0	0	0	0	1	0	0	0	0	0	0	0
0	0	0	0	0	0	0	1	5	0	0	0	0	0
1	1	0	0	0	0	0	0	0	0	0	0	0	1
0	2	0	0	0	0	0	0	0	0	0	0	0	0
0	0	0	0	0	0	0	0	0	0	0	0	0	0
1	0	0	0	2	0	0	0	0	1	0	0	0	0
0	0	0	0	0	0	0	0	0	1	2	0	0	0
0													
OTU14654	0	0	0	0	0	1	0	0	0	0	0	0	0
0	0	0	0	0	0	0	0	0	0	0	0	0	0
0	0	0	0	0	0	0	0	0	0	0	0	0	0
0	0	0	0	0	0	0	0	0	0	0	0	0	0
0	0	0	0	0	0	0	0	0	0	0	0	0	0
0	0	0	0	0	0	0	0	0	0	0	0	0	0
0	0	0	0	0	0	0	0	0	0	0	0	0	0
0	0	0	0	0	0	0	0	0	0	0	0	0	0
0													
OTU12036	0	0	0	0	0	1	0	0	0	0	0	0	0
0	0	0	0	0	0	0	0	0	0	0	0	0	0
0	0	0	0	0	0	0	0	0	0	0	0	0	0
0	0	0	0	0	0	0	0	0	0	0	0	0	0
0	0	0	0	0	0	0	0	0	0	0	0	0	0
0	0	0	0	0	0	0	0	0	0	0	0	0	0
0	0	0	0	0	0	0	0	0	0	0	0	0	0
0	0	0	0	0	0	0	0	0	0	0	0	0	0
0													
OTU15730	0	0	0	0	0	1	0	0	0	0	0	0	0
0	0	0	0	0	0	0	0	0	0	0	0	0	0
0	0	0	0	0	0	0	0	0	0	0	0	0	0

0 0 0 0 0 0 0 0 0 0 0 0 0 0
0 0 0 0 0 0 0 0 0 0 0 0 0 0
0 0 0 0 0 0 0 0 0 0 0 0 0 0
0 0 0 0 0 0 0 0 0 0 0 0 0 0
0 0 0 0 0 0 0 0 0 0 0 0 0 0
0
OTU1372 0 0 0 0 0 1 0 0 0 0 0 0 0
0 0 0 0 0 0 0 0 0 0 0 0 0 0
0 0 0 0 0 0 0 0 0 0 3 0 3 0
0 0 0 0 0 0 0 1 0 0 0 0 0 0
0 0 0 0 0 0 0 0 0 0 0 0 0 0
0 4 0 0 0 0 0 0 0 0 0 0 0 0
0 0 0 0 1 0 0 0 0 0 0 0 1 0
0 0 0 2 1 3 1 3 0 1 0 0 0 14
3
OTU2073 0 0 0 0 0 3 0 0 0 0 0 0 0
1 0 0 4 0 0 0 0 0 0 1 0 1 0
0 0 0 0 0 0 0 1 1 0 3 0 0 0
0 0 0 0 2 7 0 5 0 4 1 0 0 0
1 2 2 0 0 3 0 4 5 0 2 1 8 0
3 0 0 0 0 0 0 0 0 0 0 0 0 0
0 0 0 0 0 0 0 0 0 0 0 0 0 2
0 0 4 0 0 0 0 0 0 0 0 0 0 0
0
OTU8401 0 0 0 0 0 1 0 0 0 0 0 0 0
0 0 0 0 0 0 0 0 0 0 0 0 0 0
0 0 0 0 0 0 0 0 0 0 0 0 0 0
0 0 0 0 0 0 0 0 0 0 0 0 0 0
0 0 0 0 0 0 0 0 0 0 0 0 0 0
0 0 0 0 0 0 0 0 0 0 0 0 0 0
0 0 0 0 0 0 0 0 0 0 0 0 0 0
0 0 0 0 0 0 0 0 0 0 0 0 0 0
0

OTU14648	0	0	0	0	0	1	0	0	0	0	0	0	0
0	0	0	0	0	0	0	0	0	0	0	0	0	0
0	0	0	0	0	0	0	0	0	0	0	0	0	0
0	0	0	0	0	0	0	0	0	0	0	0	0	0
0	0	0	0	0	0	0	0	0	0	0	0	0	0
0	0	0	0	0	0	0	0	0	0	0	0	0	0
0	0	0	0	0	0	0	0	0	0	0	0	0	0
0	0	0	0	0	0	0	0	0	0	0	0	0	0
0													
OTU4529	0	0	0	0	0	1	0	0	0	0	0	0	0
0	0	3	0	0	1	0	1	0	0	0	0	0	0
0	0	1	0	0	0	0	0	0	0	0	0	0	1
0	0	0	0	0	0	0	0	0	0	0	0	0	0
0	0	0	0	0	0	0	0	0	0	0	0	0	0
0	0	0	1	0	0	0	0	0	0	0	0	0	0
0	0	1	0	0	0	0	0	0	1	0	0	0	0
1	0	0	0	0	0	0	0	0	0	0	0	0	0
0													
OTU1871	0	0	0	0	0	1	0	0	0	0	0	0	0
0	0	0	0	0	0	0	0	0	0	0	0	0	0
0	0	0	0	0	0	0	0	0	0	0	0	0	0
0	0	0	0	0	0	0	1	0	0	0	0	0	0
0	0	0	0	0	0	0	0	0	0	0	1	0	0
0	0	0	0	0	0	0	0	0	0	0	0	1	0
0	0	0	0	0	0	0	0	0	0	0	0	0	0
0	0	0	0	0	0	0	0	0	0	0	0	0	0
0													
OTU14439	0	0	0	0	0	2	1	1	1	1	0	1	0
0	0	1	0	2	0	0	1	2	1	0	1	1	1
0	0	3	0	0	1	0	3	0	0	1	1	2	0
1	1	0	0	0	1	0	0	1	4	1	2	0	0
0	0	0	0	0	0	0	0	2	1	0	0	0	0
0	1	0	1	0	0	0	2	2	0	0	2	0	1

1	1	2	0	0	2	0	1	1	1	0	1	2	1
1	3	0	0	0	2	0	0	0	2	0	0	0	3
1													
OTU15727	0	0	0	0	0	1	0	0	0	0	0	0	0
0	0	0	0	0	0	0	0	0	0	0	0	0	0
0	0	0	0	0	0	0	0	0	0	0	0	0	0
0	0	0	0	0	0	0	0	0	0	0	0	0	0
0	0	0	0	0	0	0	0	0	0	0	0	0	0
0	0	0	0	0	0	0	0	0	0	0	0	0	0
0	0	0	0	0	0	0	0	0	0	0	0	0	0
0	0	0	0	0	0	0	0	0	0	0	0	0	0
0													
OTU14649	0	0	0	0	0	1	0	0	0	0	0	0	0
0	0	0	0	0	0	0	0	0	0	0	0	0	0
0	0	0	0	0	0	0	0	0	0	0	0	0	0
0	0	0	0	0	0	0	0	0	0	0	0	0	0
0	0	0	0	0	0	0	0	0	0	0	0	0	0
0	0	0	0	0	0	0	0	0	0	0	0	0	0
0	0	0	0	0	0	0	0	0	0	0	0	0	0
0	0	0	0	0	0	0	0	0	0	0	0	0	0
0													
OTU14650	0	0	0	0	0	1	0	0	0	0	0	0	0
0	0	0	0	0	0	0	0	0	0	0	0	0	0
0	0	0	0	0	0	0	0	0	0	0	0	0	0
0	0	0	0	0	0	0	0	0	0	0	0	0	0
0	0	0	0	0	0	0	0	0	0	0	0	0	0
0	0	0	0	0	0	0	0	0	0	0	0	0	0
0	0	0	0	0	0	0	0	0	0	0	0	0	0
0	0	0	0	0	0	0	0	0	0	0	0	0	0
0													
OTU3879	0	0	0	0	0	1	0	0	1	0	0	0	0
0	0	0	0	0	0	0	0	0	0	0	0	0	0
0	0	0	0	0	0	1	0	0	0	0	0	0	0

1	0	0	0	0	0	0	0	0	0	0	0	0	0
0	0	0	0	0	0	0	0	0	0	0	0	0	1
0	0	0	0	0	0	0	0	0	0	0	0	0	0
0	0	0	0	0	0	0	1	0	0	0	0	0	0
0	0	0	0	0	0	0	0	0	0	0	0	0	0
0													
OTU4825	0	0	0	0	0	1	0	0	0	0	0	0	0
0	0	0	0	0	0	0	0	0	0	0	0	0	0
0	0	0	0	0	0	0	0	0	0	0	0	0	0
0	0	0	0	0	0	0	0	0	0	1	0	0	0
0	0	0	0	0	0	0	0	0	0	0	0	0	0
0	0	0	0	0	0	0	0	0	0	0	0	1	0
0	0	0	0	0	0	0	0	0	0	2	0	0	0
0	0	0	0	0	0	0	0	0	0	0	0	0	0
0													
OTU8404	0	0	0	0	0	1	0	0	0	0	0	0	0
0	0	0	0	0	0	0	0	0	0	0	0	0	0
0	0	0	0	0	0	0	0	0	0	0	0	0	0
0	0	0	0	0	0	0	0	0	0	0	0	0	0
0	0	0	0	0	0	0	0	0	0	0	0	0	0
0	0	0	0	0	0	0	0	0	0	0	0	0	0
0	0	0	0	0	0	0	0	0	0	0	0	0	0
0	0	0	0	0	0	0	0	0	0	0	0	0	0
0													
OTU4121	0	0	0	0	0	1	0	0	0	1	0	0	0
0	0	0	0	0	0	0	0	0	0	0	0	0	0
0	0	0	0	0	0	0	0	0	0	0	0	0	0
0	1	0	0	0	0	0	0	0	0	0	0	0	0
0	0	0	0	0	0	0	0	0	0	0	0	0	0
0	0	0	0	0	0	0	1	0	0	0	0	0	0
0	0	0	0	0	0	0	1	0	0	0	0	0	0
0	0	2	0	1	0	0	0	1	1	0	0	0	0
5													

OTU8403	0	0	0	0	0	1	0	0	0	0	0	0	0
0	0	0	0	0	0	0	0	0	0	0	0	0	0
0	0	0	0	0	0	0	0	0	0	0	0	0	0
0	0	0	0	0	0	0	0	0	0	0	0	0	0
0	0	0	0	0	0	0	0	0	0	0	0	0	0
0	0	0	0	0	0	0	0	0	0	0	0	0	0
0	0	0	0	0	0	0	0	0	0	0	0	0	0
0	0	0	0	0	0	0	0	0	0	0	0	0	0
0													
OTU8402	0	0	0	0	0	1	0	0	0	0	0	0	0
0	0	0	0	0	0	0	0	0	0	0	0	0	0
0	0	0	0	0	0	0	0	0	0	0	0	0	0
0	0	0	0	0	0	0	0	0	0	0	0	0	0
0	0	0	0	0	0	0	0	0	0	0	0	0	0
0	0	0	0	0	0	0	0	0	0	0	0	0	0
0	0	0	0	0	0	0	0	0	0	0	0	0	0
0	0	0	0	0	0	0	0	0	0	0	0	0	0
0													
OTU539	0	0	0	0	0	1	0	0	0	0	0	0	0
0	0	0	0	0	0	0	0	0	0	0	0	0	0
0	0	0	0	1	0	1	0	0	0	1	0	0	0
0	0	0	0	0	0	0	0	0	0	0	1	0	0
0	0	0	0	0	0	0	0	0	0	0	0	0	0
0	4	0	0	0	0	0	5	0	0	0	0	0	0
0	0	0	2	0	0	0	2	0	0	0	1	0	0
0	5	7	2	0	4	1	1	1	3	0	0	0	5
2													
OTU15728	0	0	0	0	0	1	0	0	0	0	0	0	0
0	0	0	0	0	0	0	0	0	0	0	0	0	0
0	0	0	0	0	0	0	0	0	0	0	0	0	0
0	0	0	0	0	0	0	0	0	0	0	0	0	0
0	0	0	0	0	0	0	0	0	0	0	0	0	0
0	0	0	0	0	0	0	0	0	0	0	0	0	0

0	0	0	0	0	0	0	0	0	0	0	0	0	0
0	0	0	0	0	0	0	0	0	0	0	0	0	0
0													
OTU12031	0	0	0	0	0	1	0	0	0	0	0	0	0
0	0	0	0	0	0	0	0	0	0	0	0	0	0
0	0	0	0	0	0	0	0	0	0	0	0	0	0
0	0	0	0	0	0	0	0	0	0	0	0	0	0
0	0	0	0	0	0	0	0	0	0	0	0	0	0
0	0	0	0	0	0	0	0	0	0	0	0	0	0
0	0	0	0	0	0	0	0	0	0	0	0	0	0
0	0	0	0	0	0	0	0	0	0	0	0	0	0
0													
OTU3888	0	0	0	0	0	1	0	1	0	0	0	0	0
0	0	0	0	1	1	0	0	0	1	0	0	0	0
0	0	1	0	0	0	0	1	0	0	0	0	0	2
0	0	0	0	0	0	0	0	0	0	0	0	0	0
0	0	0	0	0	0	0	0	0	0	0	0	0	0
0	0	0	0	0	1	1	0	0	0	0	0	1	0
0	0	0	0	1	0	0	0	0	0	0	0	0	0
0	0	0	0	0	0	0	0	0	0	0	0	0	0
0													
OTU14449	0	0	0	0	0	1	0	0	0	0	0	0	0
0	0	0	0	0	0	0	0	0	0	0	0	0	0
0	0	0	0	0	0	0	0	0	0	0	0	0	0
0	0	0	0	0	0	0	0	0	0	0	0	0	0
0	0	0	0	0	0	0	0	0	0	0	0	0	0
0	0	0	0	0	0	0	0	0	0	0	0	0	0
0	0	0	0	0	0	0	0	0	0	0	0	0	0
0	0	0	0	0	0	0	0	0	0	0	0	0	0
0													
OTU6057	0	0	0	0	0	2	0	1	0	1	0	0	0
0	0	0	0	0	0	0	0	0	0	0	0	1	4
0	0	0	0	0	0	0	0	0	0	0	1	1	0

0 0 0 0 0 0 0 1 0 0 0 0 0 1
0 0 0 1 0 0 0 0 0 0 0 1 2 0
0 0 1 0 0 0 0 0 0 1 1 0 0 0
0 0 0 0 0 0 0 0 0 0 0 0 0 0
0 0 0 0 0 0 0 0 0 0 0 0 0 0
0
OTU1377 0 0 0 0 0 1 0 0 0 0 0 1 1
0 0 0 0 0 0 0 0 0 0 0 0 0 0
0 0 0 0 0 0 0 0 0 3 0 0 0 0
0 0 0 1 0 0 0 0 0 0 0 0 0 0
0 0 0 1 0 0 1 0 0 0 0 8 0 2
0 0 2 0 0 0 0 0 2 0 0 0 1 1
0 0 0 0 3 0 0 0 0 4 0 0 0 0
0 0 0 0 0 0 0 0 0 0 6 0 1 0
1
OTU12042 0 0 0 0 0 2 0 0 0 0 0 0 0
0 0 0 0 0 0 0 0 0 0 0 0 0 0
0 0 0 0 0 0 0 0 0 0 0 0 0 0
0 0 0 0 0 1 0 0 0 0 0 0 0 0
0 0 0 0 0 0 0 0 0 0 0 0 0 0
0 0 0 0 0 0 0 0 0 0 0 0 0 0
0 0 0 0 0 0 0 0 0 0 0 0 0 0
0 0 0 0 0 0 0 0 0 0 0 0 0 0
0
OTU1154 0 0 0 0 0 1 0 0 0 0 3 0 0
0 0 0 0 0 0 0 0 0 0 0 0 0 0
0 0 0 0 0 0 0 0 0 0 0 0 0 0
0 0 0 0 0 0 0 0 0 0 0 0 0 0
0 0 0 0 0 0 0 0 0 0 0 0 0 0
0 0 0 0 0 0 0 0 0 0 0 0 0 0
0 0 0 0 0 0 0 0 0 0 0 0 0 0
0 0 0 0 0 0 0 0 0 0 1 0 0 0
0

OTU6534	0	0	0	0	0	2	0	0	0	0	0	0	0
0	0	1	0	0	0	0	1	0	0	0	0	0	0
0	0	0	0	0	0	0	0	0	0	0	0	0	0
0	0	1	1	0	1	0	0	0	1	0	4	0	0
2	0	0	0	0	1	2	0	2	0	0	0	0	0
0	0	1	0	0	0	0	1	0	0	0	2	0	0
0	2	0	0	0	0	0	0	0	0	0	0	0	0
0	0	0	0	0	1	0	0	0	0	0	0	0	0
0													
OTU12041	0	0	0	0	0	1	0	0	0	0	0	0	0
0	0	0	0	0	0	0	0	0	0	0	0	0	0
0	0	0	0	0	0	0	0	0	0	0	0	0	0
0	0	0	0	0	0	0	0	0	0	0	0	0	0
0	0	0	0	0	0	0	0	0	0	0	0	0	0
0	0	0	0	0	0	0	0	0	0	0	0	0	0
0	0	0	0	0	0	0	0	0	0	0	0	0	0
0	0	0	0	0	0	0	0	0	0	0	0	0	0
0													
OTU8408	0	0	0	0	0	1	0	0	0	0	0	0	0
0	0	0	0	0	0	0	0	0	0	0	0	0	0
0	0	0	0	0	0	0	0	0	0	0	0	0	0
0	0	0	0	0	0	0	0	0	0	0	0	0	0
0	0	0	0	0	0	0	0	0	0	0	0	0	0
0	0	0	0	0	0	0	0	0	0	0	0	0	0
0	0	0	0	0	0	0	0	0	0	0	0	0	0
0	0	0	0	0	0	0	0	0	0	0	0	0	0
0													
OTU8409	0	0	0	0	0	1	0	0	0	0	0	0	0
0	0	0	0	0	0	0	0	0	0	0	0	0	0
0	0	0	0	0	0	0	0	0	0	0	0	0	0
0	0	0	0	0	0	0	0	0	0	0	0	0	0
0	0	0	0	0	0	0	0	0	0	0	0	0	0
0	0	0	0	0	0	0	0	0	0	0	0	0	0

0	0	0	0	0	0	0	0	0	0	0	0	0	0
0	0	0	0	0	0	0	0	0	0	0	0	0	0
0													
OTU1350	0	0	0	0	0	1	0	0	0	0	0	0	0
0	0	0	1	0	0	0	0	2	0	0	0	0	0
0	0	0	1	0	0	0	0	1	0	0	0	0	0
0	0	0	0	1	0	0	3	0	0	0	0	0	0
0	0	0	1	1	0	1	0	0	0	0	2	0	0
0	0	0	5	0	0	0	0	1	0	0	0	0	0
0	0	0	0	0	0	7	1	0	0	0	0	0	0
0	0	0	0	0	0	0	0	0	0	0	0	0	0
0													
OTU12530	0	0	0	0	0	2	0	0	1	1	2	2	0
0	0	0	1	0	1	1	0	0	0	0	0	1	0
0	0	0	0	0	0	0	2	0	0	0	0	2	2
1	0	0	0	1	0	1	0	0	0	0	0	0	1
0	0	2	2	0	1	0	0	0	0	1	0	0	0
1	0	0	0	0	0	1	0	1	0	1	1	0	1
0	0	0	0	0	0	0	0	0	0	1	0	0	0
0	2	1	0	1	1	0	0	0	2	0	0	0	1
0													
OTU14450	0	0	0	0	0	2	0	0	0	0	0	0	0
0	0	0	0	0	0	0	0	0	0	0	0	0	0
0	0	0	0	0	0	0	0	0	0	0	0	0	0
0	0	0	0	0	0	0	0	0	0	0	0	0	0
0	0	0	0	0	0	0	0	0	0	0	0	0	0
0	0	0	0	0	0	0	0	0	0	0	0	0	0
0	0	0	0	0	0	0	0	0	0	0	0	0	0
0	0	0	0	0	0	0	0	0	0	0	0	0	0
0													
OTU15731	0	0	0	0	0	1	0	0	0	0	0	0	0
0	0	0	0	0	0	0	0	0	0	0	0	0	0
0	0	0	0	0	0	0	0	0	0	0	0	0	0

0 0 0 0 0 0 0 0 0 0 0 0 0 0
0 0 0 0 0 0 0 0 0 0 0 0 0 0
0 0 0 0 0 0 0 0 0 0 0 0 0 0
0 0 0 0 0 0 0 0 0 0 0 0 0 0
0 0 0 0 0 0 0 0 0 0 0 0 0 0
0
OTU11523 0 0 0 0 0 1 0 0 0 0 0 0 1
0 1 0 0 2 0 0 1 0 0 0 0 0 0
0 0 0 0 0 1 0 0 0 0 0 0 0 1
0 0 0 0 0 0 0 0 0 1 1 0 0 0
0 0 0 0 0 0 0 0 0 0 0 1 1 0
0 0 0 0 1 0 0 0 0 0 0 1 0 0
0 0 0 0 0 0 0 0 0 0 0 0 0 0
0 0 0 0 1 1 1 0 0 0 0 0 0 0
0
OTU1815 0 0 0 0 0 1 0 0 0 0 0 0 1
0 0 0 0 0 0 0 0 0 0 1 0 0 0
0 0 0 1 0 0 2 0 0 0 1 1 0 0
0 0 0 0 0 0 0 0 0 0 0 0 0 0
0 0 0 0 0 0 0 0 0 0 1 0 0 1
0 0 0 0 0 2 0 1 1 0 3 0 0 1
0 0 2 0 2 0 0 0 0 0 0 0 0 0
1 0 0 0 0 0 0 0 0 0 0 0 0 0
0
OTU8410 0 0 0 0 0 1 0 0 0 0 0 0 0
0 0 0 0 0 0 0 0 0 0 0 0 0 0
0 0 0 0 0 0 0 0 0 0 0 0 0 0
0 0 0 0 0 0 0 0 0 0 0 0 0 0
0 0 0 0 0 0 0 0 0 0 0 0 0 0
0 0 0 0 0 0 0 0 0 0 0 0 0 0
0 0 0 0 0 0 0 0 0 0 0 0 0 0
0 0 0 0 0 0 0 0 0 0 0 0 0 0
0

OTU12039	0	0	0	0	0	1	0	0	0	0	0	0	0
0	0	0	0	0	0	0	0	0	0	0	0	0	0
0	0	0	0	0	0	0	0	0	0	0	0	0	0
0	0	0	0	0	0	0	0	0	0	0	0	0	0
0	0	0	0	0	0	0	0	0	0	0	0	0	0
0	0	0	0	0	0	0	0	0	0	0	0	0	0
0	0	0	0	0	0	0	0	0	0	0	0	0	0
0	0	0	0	0	0	0	0	0	0	0	0	0	0
0													
OTU7580	0	0	0	0	0	1	0	0	0	0	0	0	0
0	0	0	0	0	0	0	0	0	0	0	0	0	0
0	0	1	0	0	0	0	0	0	0	0	0	0	0
0	0	0	0	0	0	0	0	0	0	0	0	0	0
0	0	0	0	1	0	0	0	0	0	0	0	0	0
0	0	1	0	0	0	0	0	0	0	0	2	0	0
0	0	0	0	0	0	0	1	0	0	0	0	0	0
0	0	0	0	0	0	0	0	0	0	0	0	0	0
0													
OTU8392	0	0	0	0	0	1	0	0	0	0	0	0	0
0	0	0	0	0	0	0	0	0	0	0	0	0	0
0	0	0	0	0	0	0	0	0	0	0	0	0	0
0	0	0	0	0	0	0	0	0	0	0	0	0	0
0	0	0	0	0	0	0	0	0	0	0	0	0	0
0	0	0	0	0	0	0	0	0	0	0	0	0	0
0	0	0	0	0	0	0	0	0	0	0	0	0	0
0	0	0	0	0	0	0	0	0	0	0	0	0	0
0													
OTU12037	0	0	0	0	0	1	0	0	0	0	0	0	0
0	0	0	0	0	0	0	0	0	0	0	0	0	0
0	0	0	0	0	0	0	0	0	0	0	0	0	0
0	0	0	0	0	0	0	0	0	0	0	0	0	0
0	0	0	0	0	0	0	0	0	0	0	0	0	0
0	0	0	0	0	0	0	0	0	0	0	0	0	0

0	0	0	0	0	0	0	0	0	0	0	0	0	0
0	0	0	0	0	0	0	0	0	0	0	0	0	0
0													
OTU558	0	0	0	0	0	1	1	0	3	2	0	0	0
0	0	0	0	0	0	0	0	0	0	0	0	0	0
0	0	0	0	0	0	0	0	1	0	0	0	0	0
1	0	0	0	0	0	0	0	0	0	0	0	0	0
0	0	0	0	0	0	0	0	0	0	0	0	0	0
0	3	1	0	0	0	0	0	0	0	0	1	0	2
0	0	0	2	0	0	0	0	0	0	0	2	5	0
0	4	2	5	3	1	0	6	2	6	0	0	0	1
2													
OTU12038	0	0	0	0	0	1	0	0	0	0	0	0	0
0	0	0	0	0	0	0	0	0	0	0	0	0	0
0	0	0	0	0	0	0	0	0	0	0	0	0	0
0	0	0	0	0	0	0	0	0	0	0	0	0	0
0	0	0	0	0	0	0	0	0	0	0	0	0	0
0	0	0	0	0	0	0	0	0	0	0	0	0	0
0	0	0	0	0	0	0	0	0	0	0	0	0	0
0	0	0	0	0	0	0	0	0	0	0	0	0	0
0													
OTU14447	0	0	0	0	0	1	0	0	0	0	1	0	0
0	0	0	0	0	0	0	0	0	0	0	0	0	0
0	0	1	1	0	0	0	0	0	0	0	0	0	0
0	0	0	0	0	0	0	0	0	0	0	0	0	0
0	0	0	0	0	0	0	1	0	0	0	0	0	0
1	0	0	1	0	0	0	0	1	0	0	0	0	0
0	0	0	0	0	0	1	0	0	0	0	0	0	0
0	0	0	0	0	1	0	0	1	0	1	0	0	0
0													
OTU8390	0	0	0	0	0	1	0	0	0	0	0	0	0
0	0	0	0	0	0	0	0	0	0	0	0	0	0
0	0	0	0	0	0	0	0	0	0	0	0	0	0

0 0 0 0 0 0 0 0 0 0 0 0 0 0
0 0 0 0 0 0 0 0 0 0 0 0 0 0
0 0 0 0 0 0 0 0 0 0 0 0 0 0
0 0 0 0 0 0 0 0 0 0 0 0 0 0
0 0 0 0 0 0 0 0 0 0 0 0 0 0
0
OTU2745 0 0 0 0 0 1 0 0 0 0 0 0 0
0 0 0 0 0 0 0 0 0 0 0 0 0 0
0 0 0 2 0 0 0 0 0 0 0 0 1 0
0 0 0 0 0 0 0 0 0 0 0 0 0 0
0 0 0 0 0 0 0 0 0 0 0 0 0 0
0 0 0 0 0 0 0 0 0 0 0 0 0 0
0 0 0 0 0 0 0 0 0 0 0 0 0 0
3 0 0 0 0 0 0 0 0 0 0 0 0 0
0
OTU12040 0 0 0 0 0 1 0 0 0 0 0 0 0
0 0 0 0 0 0 0 0 0 0 0 0 0 0
0 0 0 0 0 0 0 0 0 0 0 0 0 0
0 0 0 0 0 0 0 0 0 0 0 0 0 0
0 0 0 0 0 0 0 0 0 0 0 0 0 0
0 0 0 0 0 0 0 0 0 0 0 0 0 0
0 0 0 0 0 0 0 0 0 0 0 0 0 0
0 0 0 0 0 0 0 0 0 0 0 0 0 0
0
OTU8393 0 0 0 0 0 1 0 0 0 0 0 0 0
0 0 0 0 0 0 0 0 0 0 0 0 0 0
0 0 0 0 0 0 0 0 0 0 0 0 0 0
0 0 0 0 0 0 0 0 0 0 0 0 0 0
0 0 0 0 0 0 0 0 0 0 0 0 0 0
0 0 0 0 0 0 0 0 0 0 0 0 0 0
0 0 0 0 0 0 0 0 0 0 0 0 0 0
0 0 0 0 0 0 0 0 0 0 0 0 0 0
0

OTU749 0 0 0 0 0 1 1 0 2 2 0 0 0
0 0 0 0 0 0 1 0 0 0 0 0 0 0
0 0 0 0 0 0 0 0 0 0 0 0 0 0
0 0 0 0 0 0 0 0 0 0 0 0 0 0
0 0 0 0 0 0 0 0 0 0 0 0 1 0
0 0 1 0 0 0 0 0 0 2 0 1 0 0
0 0 0 1 0 2 0 0 0 0 0 0 0 0
0 1 3 7 3 3 3 2 2 10 0 0 0 0
2
OTU16639 0 0 0 0 0 1 0 0 1 0 0 0 0
0 0 0 0 0 0 0 0 0 0 0 0 0 0
0 0 0 0 0 0 0 0 0 0 0 0 0 0
0 0 0 0 0 1 0 0 0 0 0 0 0 0
0 0 0 0 0 0 0 0 0 0 0 0 0 0
0 0 0 0 0 0 0 0 0 0 0 0 0 0
0 1 0 0 0 0 0 0 0 0 0 0 0 0
0 0 0 0 0 0 0 0 0 0 0 0 0 0
0
OTU14452 0 0 0 0 0 1 0 0 0 0 0 0 0
0 0 0 0 0 0 0 0 0 0 0 0 0 0
0 0 0 0 0 0 0 0 0 0 0 0 0 0
0 0 0 0 0 0 0 0 0 0 0 0 0 0
0 0 0 0 0 0 0 0 0 0 0 0 0 0
0 0 0 0 0 0 0 0 0 0 0 0 0 0
0 0 0 0 0 0 0 0 0 0 0 0 0 0
0 0 0 0 0 0 0 0 0 0 0 0 0 0
0
OTU16768 0 0 0 0 0 1 0 0 0 0 0 0 0
0 0 0 1 0 0 0 0 0 0 0 0 0 0
0 0 0 1 0 0 0 0 0 0 0 0 0 0
0 1 0 0 0 0 0 0 0 0 0 0 0 0
0 0 0 0 0 0 0 0 0 0 0 2 0 0
0 0 0 0 0 0 0 0 0 0 0 0 0 0

0	0	0	0	0	0	0	0	0	2	0	0	0	0
0	0	0	0	0	0	0	0	0	0	0	0	0	0
0													
OTU9471	0	0	0	0	0	1	0	0	0	0	0	0	0
0	0	0	0	0	0	0	0	0	0	0	0	1	0
0	0	0	2	0	0	0	0	0	0	0	0	0	0
0	0	0	0	0	0	0	0	0	0	0	0	0	0
0	0	0	0	0	0	0	0	0	0	0	0	0	0
0	0	0	0	0	0	0	0	0	0	0	0	0	0
0	0	0	0	0	0	1	0	0	0	0	0	0	0
0	0	0	0	0	0	0	0	0	0	0	0	0	0
0													
OTU7871	0	0	0	0	0	1	0	0	0	0	0	0	0
0	0	0	0	0	0	0	0	0	0	0	0	0	0
0	0	0	0	0	0	0	0	0	0	0	0	0	0
0	0	0	0	0	0	0	0	0	0	0	0	0	0
0	0	0	0	0	0	0	0	0	0	0	0	0	0
0	0	0	0	0	0	0	0	0	0	0	0	0	0
0	0	0	0	0	0	0	0	0	0	0	0	0	0
0	0	0	0	0	0	1	0	0	0	0	0	0	0
0													
OTU8389	0	0	0	0	0	1	0	0	0	0	0	0	0
0	0	0	0	0	0	0	0	0	0	0	0	0	0
0	0	0	0	0	0	0	0	0	0	0	0	0	0
0	0	0	0	0	0	0	0	0	0	0	0	0	0
0	0	0	0	0	0	0	0	0	0	0	0	0	0
0	0	0	0	0	0	0	0	0	0	0	0	0	0
0	0	0	0	0	0	0	0	0	0	0	0	0	0
0	0	0	0	0	0	0	0	0	0	0	0	0	0
0													
OTU15754	0	0	0	0	0	1	0	0	0	0	0	0	0
0	0	0	0	0	0	0	0	0	0	0	0	0	0
0	0	0	0	0	0	0	0	0	0	0	0	0	0

0	0	0	0	0	0	0	0	0	0	0	0	0	0
0	0	0	0	0	0	0	0	0	0	0	0	0	0
0	0	0	0	0	0	0	0	0	0	0	0	0	0
0	0	0	0	0	0	0	0	0	0	0	0	0	0
0	0	0	0	0	0	0	0	0	0	0	0	0	0
0													
OTU8398	0	0	0	0	0	1	0	0	0	0	0	0	0
0	0	0	0	0	0	0	0	0	0	0	0	0	0
0	0	0	0	0	0	0	0	0	0	0	0	0	0
0	0	0	0	0	0	0	0	0	0	0	0	0	0
0	0	0	0	0	0	0	0	0	0	0	0	0	0
0	0	0	0	0	0	0	0	0	0	0	0	0	0
0	0	0	0	0	0	0	0	0	0	0	0	0	0
0	0	0	0	0	0	0	0	0	0	0	0	0	0
0													
OTU15753	0	0	0	0	0	1	0	0	0	0	0	0	0
0	0	0	0	1	0	0	0	1	0	0	0	0	0
0	0	0	0	0	0	0	0	0	0	0	1	0	0
0	0	0	0	0	0	0	0	0	0	0	0	0	0
0	0	0	0	0	0	0	0	0	1	0	0	0	0
0	0	0	0	0	0	0	0	0	0	0	0	0	0
0	0	0	0	0	0	0	0	0	0	0	0	0	0
0	0	0	0	0	0	0	0	0	0	0	0	0	0
0													
OTU15752	0	0	0	0	0	1	0	0	0	0	0	0	0
0	0	0	0	0	0	0	0	0	0	0	0	0	0
0	0	0	0	0	0	0	0	0	0	0	0	0	0
0	0	0	0	0	0	0	0	0	0	0	0	0	0
0	0	0	0	0	0	0	0	0	0	0	0	0	0
0	0	0	0	0	0	0	0	0	0	0	0	0	0
0	0	0	0	0	0	0	0	0	0	0	0	0	0
0	0	0	0	0	0	0	0	0	0	0	0	0	0
0													

OTU2207 0 0 0 0 0 1 0 0 0 0 0 1 0
0 0 0 0 0 0 0 0 0 0 0 0 0 1
0 0 0 0 0 0 0 0 0 0 0 0 0 0
0 0 0 0 0 0 0 0 0 0 0 0 0 0
0 0 0 0 0 0 0 0 0 0 1 0 1 0
0 0 1 0 0 0 0 0 0 0 0 0 0 0
0 0 0 0 0 1 0 0 0 0 0 0 2 0
0 0 0 1 0 0 0 0 0 0 0 0 0 0
0
OTU14451 0 0 0 0 0 1 0 0 0 0 0 0 0
0 0 0 0 0 0 0 0 0 0 0 0 0 0
0 0 0 0 0 0 0 0 0 0 0 0 0 0
0 0 0 0 0 0 0 0 0 0 0 0 0 0
0 0 0 0 0 0 0 0 0 0 0 0 0 0
0 0 0 0 0 0 0 0 0 0 0 0 0 0
0 0 0 0 0 0 0 0 0 0 0 0 0 0
0 0 0 0 0 0 0 0 0 0 0 0 0 0
0
OTU15751 0 0 0 0 0 1 0 0 0 0 0 0 0
0 0 0 0 0 0 0 0 0 0 0 0 0 0
0 0 0 0 0 0 0 0 0 0 0 0 0 0
0 0 0 0 0 0 0 0 0 0 0 0 0 0
0 0 0 0 0 0 0 0 0 0 0 0 0 0
0 0 0 0 0 0 0 0 0 0 0 0 0 0
0 0 0 0 0 0 0 0 0 0 0 0 0 0
0 0 0 0 0 0 0 0 0 0 0 0 0 0
0
OTU15716 0 0 0 0 0 2 0 2 0 0 0 0 0
0 0 0 0 0 0 0 0 0 0 0 0 1 0
0 0 0 0 0 0 0 0 0 0 0 0 0 0
0 0 0 0 0 0 0 0 0 0 0 0 0 0
0 0 0 0 0 0 0 0 0 0 0 0 0 0
0 0 0 0 0 0 0 0 0 0 0 0 0 0

0	0	0	0	0	0	3	0	0	0	0	0	0	0
0	0	0	0	0	0	0	0	0	0	0	0	0	0
0													
OTU8399	0	0	0	0	0	1	0	0	0	0	0	0	0
0	0	0	0	0	0	0	0	0	0	0	0	0	0
0	0	0	0	0	0	0	0	0	0	0	0	0	0
0	0	0	0	0	0	0	0	0	0	0	0	0	0
0	0	0	0	0	0	0	0	0	0	0	0	0	0
0	0	0	0	0	0	0	0	0	0	0	0	0	0
0	0	0	0	0	0	0	0	0	0	0	0	0	0
0	0	0	0	0	0	0	0	0	0	0	0	0	0
0													
OTU12021	0	0	0	0	0	1	0	0	0	0	0	0	0
0	0	0	0	0	0	0	0	0	0	0	0	0	0
0	0	0	0	0	0	0	0	0	0	0	0	0	0
0	0	0	0	0	0	0	0	0	0	0	0	0	0
0	0	0	0	0	0	0	0	0	0	0	0	0	0
0	0	0	0	0	0	0	0	0	0	0	0	0	0
0	0	0	0	0	0	0	1	0	0	0	0	0	0
0	0	2	0	0	0	0	0	0	0	0	0	0	0
0													
OTU12022	0	0	0	0	0	1	0	0	0	0	0	0	0
0	0	0	0	0	0	0	0	0	0	0	0	0	0
0	0	0	0	0	0	0	0	0	0	0	0	0	0
0	0	0	0	0	0	0	0	0	0	0	0	0	0
0	0	0	0	0	0	0	0	0	0	0	0	0	0
0	0	0	0	0	0	0	0	0	0	0	0	0	0
0	0	0	0	0	0	0	0	0	0	0	0	0	0
0	0	0	0	0	0	0	0	0	0	0	0	0	0
0													
OTU15750	0	0	0	0	0	1	0	0	0	0	0	0	0
0	0	0	0	0	0	0	0	0	0	0	0	0	0
0	0	0	0	0	0	0	0	0	0	0	0	0	0

0	0	0	0	0	0	0	0	0	0	0	0	0	0
0	0	0	0	0	0	0	0	0	0	0	0	0	0
0	0	0	0	0	0	0	0	0	0	0	0	0	0
0	0	0	0	0	0	0	0	0	0	0	0	0	0
0	0	0	0	0	0	0	0	0	0	0	0	0	0
0													
OTU3439	0	0	0	0	0	2	0	0	0	0	0	0	0
0	0	0	0	0	0	0	0	0	0	0	0	0	0
0	0	0	0	0	0	0	0	0	0	0	0	0	0
0	0	0	0	0	0	0	0	0	0	0	0	0	0
0	0	0	0	0	0	0	0	0	0	0	0	0	0
0	0	0	0	0	0	0	0	0	2	0	0	0	0
0	0	0	1	0	0	0	0	0	0	0	0	0	0
0	0	0	0	0	0	0	0	0	0	0	0	0	0
0													
OTU8396	0	0	0	0	0	1	0	0	0	0	0	0	0
0	0	0	0	0	0	0	0	0	0	0	0	0	0
0	0	0	0	0	0	0	0	0	0	0	0	0	0
0	0	0	0	0	0	0	0	0	0	0	0	0	0
0	0	0	0	0	0	0	0	0	0	0	0	0	0
0	0	0	0	0	0	0	0	0	0	0	0	0	0
0	0	0	0	0	0	0	0	0	0	0	0	0	0
0	0	0	0	0	0	0	0	0	0	0	0	0	0
0													
OTU1127	0	0	0	0	0	1	0	0	0	0	0	0	0
0	0	0	0	0	0	0	0	0	0	0	0	0	0
0	0	0	0	0	0	0	0	0	0	0	0	0	0
0	0	0	0	0	0	0	0	0	0	0	0	0	0
0	0	0	0	0	0	0	0	0	0	0	0	0	0
0	0	0	0	0	0	0	0	0	0	0	0	0	0
0	0	0	0	0	0	0	0	0	0	0	0	0	0
0	1	0	0	0	0	0	0	0	0	0	0	0	0
10													

OTU14436 0 0 0 0 0 1 0 0 0 0 0 0 0
0 0 0 0 0 0 0 0 0 0 0 0 0 0
0 0 0 0 0 0 0 0 0 0 0 0 0 0
0 0 0 0 0 0 0 0 0 0 0 0 0 0
0 0 0 0 0 0 0 0 0 0 0 0 0 0
0 0 0 0 0 0 0 0 0 0 0 0 0 0
0 0 0 0 0 0 0 0 0 0 0 0 0 0
0 0 0 0 0 0 0 0 0 0 0 0 0 0
0
OTU546 0 0 0 0 0 1 0 1 1 0 0 0 0
0 0 0 0 0 0 0 1 1 0 2 1 1 2
1 0 0 0 0 0 1 0 0 0 1 1 0 0
0 0 0 0 1 1 0 2 0 0 0 0 0 0
0 0 0 0 0 1 0 0 1 0 0 0 0 0
0 0 0 0 0 2 0 0 0 0 3 0 0 0
0 0 0 0 0 0 2 0 0 0 0 0 0 0
0 0 0 0 0 0 0 0 0 0 0 0 0 0
0
OTU7439 0 0 0 0 0 1 0 0 0 0 0 0 0
0 0 0 0 0 0 1 0 4 0 0 0 4 0
1 1 0 0 0 0 5 0 1 0 76 0 3 0
0 0 0 0 11 0 0 0 0 1 0 16 0 0
0 0 5 17 1 46 0 1 0 0 4 1 48 1
0 15 5 0 0 0 0 8 0 0 1 3 0 0
1 40 0 0 0 2 0 19 0 0 0 0 2 1
0 0 13 4 3 12 18 4 7 3 0 0 0 12
5
OTU14304 0 0 0 0 0 1 1 1 0 1 1 0 1
0 0 0 0 0 0 1 0 0 0 0 0 0 0
0 0 0 0 0 1 0 0 1 0 0 0 0 0
0 0 0 0 0 0 0 0 0 0 1 0 0 1
0 1 0 0 0 0 0 0 0 0 0 0 0 0
0 0 0 0 0 0 0 0 1 0 0 0 1 0

0	0	0	0	1	0	0	0	0	0	0	0	1	0
0	0	1	0	1	0	0	1	0	0	3	0	1	0
0													
OTU8394	0	0	0	0	0	1	0	0	0	0	0	0	0
0	0	0	0	0	0	0	0	0	0	0	0	0	0
0	0	0	0	0	0	0	0	0	0	0	0	0	0
0	0	0	0	0	0	0	0	0	0	0	0	0	0
0	0	0	0	0	0	0	0	0	0	0	0	0	0
0	0	0	0	0	0	0	0	0	0	0	0	0	0
0	0	0	0	0	0	0	0	0	0	0	0	0	0
0	0	0	0	0	0	0	0	0	0	0	0	0	0
0													
OTU15757	0	0	0	0	0	1	0	0	0	0	0	0	0
0	0	0	0	0	0	0	0	0	0	0	0	0	0
0	0	0	0	0	0	0	0	0	0	0	0	1	0
0	0	0	0	0	0	0	0	0	0	0	0	0	0
0	0	0	0	0	0	0	0	0	0	0	0	0	0
0	0	0	0	0	0	0	0	0	0	0	0	0	0
0	0	0	0	0	0	0	0	0	0	0	0	0	0
0	0	0	0	0	0	0	0	0	0	0	0	0	0
0													
OTU14437	0	0	0	0	0	1	0	0	0	0	0	0	0
0	0	0	0	0	0	0	0	0	0	0	0	0	0
0	0	0	0	0	0	0	0	0	0	0	0	0	0
0	0	0	0	0	0	0	0	0	0	0	0	0	0
0	0	0	0	0	0	0	0	0	0	0	0	0	0
0	0	0	0	0	0	0	0	0	0	0	0	0	0
0	0	0	0	0	0	0	0	0	0	0	0	0	0
0	0	0	0	0	0	0	0	0	0	0	0	0	0
0													
OTU8395	0	0	0	0	0	1	0	0	0	0	0	0	0
0	0	0	0	0	0	0	0	0	0	0	0	0	0
0	0	0	0	0	0	0	0	0	0	0	0	0	0

0	0	0	0	0	0	0	0	0	0	0	0	0	0
0	0	0	0	0	0	0	0	0	0	0	0	0	0
0	0	0	0	0	0	0	0	0	0	0	0	0	0
0	0	0	0	0	0	0	0	0	0	0	0	0	0
0	0	0	0	0	0	0	0	0	0	0	0	0	0
0													
OTU14438	0	0	0	0	0	1	0	0	0	0	0	0	0
0	0	0	0	0	0	0	0	0	0	0	0	0	0
0	0	0	0	0	0	0	0	0	0	0	0	0	0
1	0	0	0	0	0	0	0	0	0	0	0	0	0
0	0	0	0	0	0	0	0	0	0	0	0	0	0
0	0	0	0	0	0	0	0	0	0	0	0	0	0
0	0	0	0	0	0	0	0	0	0	0	0	0	0
0	0	0	0	0	0	0	0	0	0	0	0	0	0
0													
OTU14432	0	0	0	0	0	1	0	0	0	0	0	0	0
0	0	0	0	0	0	0	0	0	0	0	0	0	0
0	0	0	0	0	0	0	0	0	0	0	0	0	0
0	0	0	0	0	0	0	0	0	0	0	0	0	0
0	0	0	0	0	0	0	0	0	0	0	0	0	0
0	0	0	0	0	0	0	0	0	0	0	0	0	0
0	0	0	0	0	0	0	0	0	0	0	0	0	0
0	0	0	0	0	0	0	0	0	0	0	0	0	0
0													
OTU3295	0	0	0	0	0	1	0	0	0	0	0	0	0
0	0	0	0	0	0	0	0	0	0	0	0	0	0
0	0	0	0	0	0	0	0	0	0	0	0	0	0
0	0	0	0	1	0	0	0	0	0	0	0	0	0
0	0	0	0	0	0	0	0	0	0	0	0	0	0
0	0	0	0	0	0	0	0	0	0	0	0	0	0
0	0	0	0	0	1	0	0	0	0	0	0	0	0
0	0	0	0	0	0	0	0	0	0	0	0	0	0
0													

OTU7804 0 0 0 0 0 1 0 0 0 0 0 0 0
0 0 0 0 0 0 0 0 0 0 0 0 0 0
0 0 0 0 0 0 0 0 0 0 0 0 0 0
0 0 0 0 0 0 0 0 0 0 0 0 0 0
0 0 0 0 0 0 0 0 0 0 0 0 0 1
0 0 0 0 0 0 0 0 0 0 0 0 0 0
0 0 0 0 0 0 0 0 0 0 0 0 0 0
0 0 0 0 0 0 0 0 0 0 0 0 0 0
0
OTU12029 0 0 0 0 0 1 0 0 0 0 0 0 0
0 0 0 0 0 0 0 0 0 0 0 0 0 0
0 0 0 0 0 0 0 0 0 0 0 0 0 0
0 0 0 0 0 0 0 0 0 0 0 0 0 0
0 0 0 0 0 0 0 0 0 0 0 0 0 0
0 0 0 0 0 0 0 0 0 0 0 0 0 0
0 0 0 0 0 0 0 0 0 0 0 0 0 0
0 0 0 0 0 0 0 0 0 0 0 0 0 0
0
OTU703 0 0 0 0 0 1 0 0 2 0 0 0 0
1 0 0 2 0 0 1 0 3 0 0 0 0 0
1 0 0 0 1 0 0 0 0 0 1 0 0 0
0 0 0 0 0 0 0 0 0 0 0 0 0 0
0 1 0 0 0 0 0 0 0 3 0 0 0 1
0 0 0 0 1 0 0 0 0 0 0 0 0 0
0 0 0 1 0 0 0 0 0 0 1 0 1 0
0 0 0 0 0 0 0 0 0 0 0 0 0 0
0
OTU15759 0 0 0 0 0 1 0 0 0 0 0 0 0
0 0 0 0 0 0 0 0 0 0 0 0 0 0
0 0 0 0 0 0 0 0 0 0 0 0 0 0
0 0 0 0 0 0 0 0 0 0 0 0 0 0
0 0 0 0 0 0 0 0 0 0 0 0 0 0
0 0 0 0 0 0 0 0 0 0 0 0 0 0

0	0	0	0	0	0	0	0	0	0	0	0	0	0
0	0	0	0	0	0	0	0	0	0	0	0	0	0
0													
OTU12024	0	0	0	0	0	1	0	0	0	0	0	0	0
0	0	0	0	0	0	0	0	0	0	0	0	0	0
0	0	0	0	0	0	0	0	0	0	0	0	0	0
0	0	0	0	0	0	0	0	0	0	0	0	0	0
0	0	0	0	0	0	0	0	0	0	0	0	0	0
0	0	0	0	0	0	0	0	0	0	0	0	0	0
0	0	0	0	0	0	0	0	0	0	0	0	0	0
0	0	0	0	0	0	0	0	0	0	0	0	0	0
0													
OTU758	0	0	0	0	0	1	0	0	0	0	0	0	0
0	0	0	0	0	0	0	0	0	0	0	0	0	0
0	0	0	0	0	0	0	0	0	0	0	0	0	0
1	0	0	0	0	0	0	0	0	0	0	1	0	0
0	0	0	1	0	0	0	0	0	0	0	0	1	0
0	0	1	0	0	0	0	0	0	0	0	0	0	2
0	0	0	1	1	0	0	1	0	0	0	0	1	0
0	1	1	2	0	1	1	0	0	1	1	0	0	2
0													
OTU15758	0	0	0	0	0	1	0	0	0	0	0	0	0
0	0	0	0	0	0	0	0	0	0	0	0	0	0
0	0	0	0	0	0	0	0	0	0	0	0	0	0
0	0	0	0	0	0	0	0	0	0	0	0	0	0
0	0	0	0	0	0	0	0	0	0	0	0	0	0
0	0	0	0	0	0	0	0	0	0	0	0	0	0
0	0	0	0	0	0	0	0	0	0	0	0	0	0
0	0	0	0	0	0	0	0	0	0	0	0	0	0
0													
OTU14324	0	0	0	0	0	2	0	0	0	0	0	0	0
0	0	0	0	0	0	0	0	0	0	0	0	0	0
0	0	0	0	0	0	0	0	0	0	0	0	0	0

0	0	0	0	0	0	0	0	0	0	0	0	0	0
0	0	0	0	0	0	0	0	0	0	0	0	0	0
0	0	0	0	0	0	0	0	0	0	0	0	0	0
0	0	0	0	0	0	0	0	0	0	0	0	0	0
0	0	0	0	0	0	0	0	0	2	0	0	0	0
0													
OTU14434	0	0	0	0	0	1	0	0	0	0	0	0	0
0	0	0	0	0	0	0	0	0	0	0	0	0	0
0	0	0	0	0	0	0	0	0	0	0	0	0	0
0	0	0	0	0	0	0	0	0	0	0	0	0	0
0	0	0	0	0	0	0	0	0	0	0	0	0	0
0	0	0	0	0	0	0	0	0	0	0	0	0	0
0	0	0	0	0	0	0	0	0	0	0	0	0	0
0	0	0	0	0	0	0	0	0	0	0	0	0	0
0													
OTU3895	0	0	0	0	0	1	0	0	0	0	0	0	0
0	0	0	0	0	0	0	0	0	0	0	0	0	0
0	0	0	0	0	0	0	0	0	0	0	0	0	0
0	0	0	0	0	1	0	0	0	0	0	0	0	0
0	0	0	0	0	0	0	0	0	0	0	0	0	0
0	0	0	1	0	0	0	0	0	0	0	0	0	0
0	0	0	0	0	0	0	0	0	0	0	0	0	0
0	0	0	0	0	0	0	0	0	0	0	0	1	0
0													
OTU14435	0	0	0	0	0	1	0	0	0	0	0	0	0
0	0	0	0	0	0	0	0	0	0	0	0	0	0
0	0	0	0	0	0	0	0	0	0	0	0	0	0
0	0	0	0	0	0	0	0	0	0	0	0	0	0
0	0	0	0	0	0	0	0	0	0	0	0	0	0
0	0	0	0	0	0	0	0	0	0	0	0	0	0
0	0	0	0	0	0	0	0	0	0	0	0	0	0
0	0	0	0	0	0	0	0	0	0	0	0	0	0
0													

OTU6955 0 0 0 0 0 2 0 0 8 2 0 2 0
0 0 0 0 0 0 0 0 0 0 0 0 0 0
0 0 0 0 0 0 0 0 1 0 0 0 0 0
0 0 0 0 0 0 0 0 0 0 0 0 0 0
0 3 0 0 0 0 0 0 0 0 0 0 1 0
0 0 0 0 1 0 0 0 0 1 0 0 0 0
0 0 0 4 0 0 0 0 0 0 0 3 0 0
0 2 0 0 0 0 0 0 0 1 1 0 0 0
1
OTU14433 0 0 0 0 0 1 0 0 0 0 0 0 0
0 0 0 0 0 0 0 0 0 0 0 0 0 0
0 0 0 0 0 0 0 0 0 0 0 0 0 0
0 0 0 0 0 0 0 0 0 0 0 0 0 0
0 0 0 0 0 0 0 0 0 0 0 0 0 0
0 0 0 0 0 0 0 0 0 0 0 0 0 0
0 0 0 0 0 0 0 0 0 0 0 0 0 0
0 0 0 0 0 0 0 0 0 0 0 0 0 0
0
OTU15755 0 0 0 0 0 1 0 0 0 0 0 0 0
0 0 0 0 0 0 0 0 0 0 0 0 0 0
0 0 0 0 0 0 0 0 0 0 0 0 0 0
0 0 0 0 0 0 0 0 0 0 0 0 0 0
0 0 0 0 0 0 0 0 0 0 0 0 0 0
0 0 0 0 0 0 0 0 0 0 0 0 0 0
0 0 0 0 0 0 0 0 0 0 0 0 0 0
0 0 0 0 0 0 0 0 0 0 0 0 0 0
0
OTU12028 0 0 0 0 0 1 0 0 0 0 0 0 0
0 0 0 0 0 0 0 0 0 0 0 0 0 0
0 0 0 0 0 0 0 0 0 0 0 0 0 1
0 0 0 0 0 0 0 0 0 0 0 0 0 0
0 0 0 0 0 0 0 0 0 0 0 0 0 0
0 0 0 0 0 0 0 0 0 0 0 0 0 0

0	0	0	0	0	0	0	0	0	0	0	0	0	0
1	0	0	0	0	0	0	0	0	0	0	0	0	0
0													
OTU15049	0	0	0	0	0	1	0	0	0	0	0	0	0
0	0	0	0	0	0	0	0	0	0	0	0	0	0
0	0	0	0	0	0	0	0	0	0	0	0	0	0
0	0	0	0	0	0	0	0	0	0	0	0	0	0
0	0	0	0	0	0	0	0	1	0	0	1	0	0
0	0	0	0	0	0	0	0	1	0	0	0	0	0
0	0	0	0	0	0	0	1	0	1	0	0	0	0
0	0	0	0	0	1	0	0	0	0	0	0	0	0
0													
OTU14443	0	0	0	0	0	1	0	0	0	0	0	0	0
0	0	0	0	0	0	0	0	0	0	0	0	0	0
0	0	0	0	0	0	0	0	0	0	0	0	0	0
0	0	0	0	0	0	0	0	0	0	0	0	0	0
0	0	0	0	0	0	0	0	0	0	0	0	0	0
0	0	0	0	0	0	0	0	0	0	0	0	0	0
0	0	0	0	0	0	0	0	0	0	0	0	0	0
0	0	0	0	0	0	0	0	0	0	0	0	0	0
0													
OTU12025	0	0	0	0	0	1	0	0	0	0	0	0	0
0	0	0	0	0	0	0	0	0	0	0	0	0	0
0	0	0	0	0	0	0	0	0	0	0	0	0	0
0	0	0	0	0	0	0	0	0	0	0	0	0	0
0	0	0	0	0	0	0	0	0	0	0	0	0	0
0	0	0	0	0	0	0	0	0	0	0	0	0	0
0	0	0	0	0	0	0	0	0	0	0	0	0	0
0	0	0	0	0	0	0	0	0	0	0	0	0	0
0													
OTU7810	0	0	0	0	0	1	0	0	0	0	0	0	0
0	0	0	0	0	0	0	0	0	0	0	0	0	0
0	0	0	0	0	0	0	0	0	0	0	0	0	0

0	0	0	0	0	0	0	0	0	0	0	0	0	0
0	0	0	0	0	0	0	0	0	0	0	0	0	0
0	0	0	0	0	0	0	0	0	0	0	0	0	0
0	0	0	0	0	0	0	0	0	0	0	0	0	0
0	0	0	0	0	0	0	0	0	0	0	0	0	0
0													
OTU12026	0	0	0	0	0	1	0	0	0	0	0	0	0
0	0	0	0	0	0	0	0	0	0	0	0	0	0
0	0	0	0	0	0	0	0	0	0	0	0	0	0
0	0	0	0	0	0	0	0	0	0	0	0	0	0
0	0	0	0	0	0	0	0	0	0	0	0	0	0
0	0	0	0	0	0	0	0	0	0	0	0	0	0
0	0	0	0	0	0	0	0	0	0	0	0	0	0
0	0	0	0	0	0	0	0	0	0	0	0	0	0
0													
OTU12027	0	0	0	0	0	1	0	0	0	0	0	0	0
0	0	0	0	0	0	0	0	0	0	0	0	0	0
0	0	0	0	0	0	0	0	0	0	0	0	0	0
0	0	0	0	0	0	0	0	0	0	0	0	0	0
0	0	0	0	0	0	0	0	0	0	0	0	0	0
0	0	0	0	0	0	0	0	0	0	0	0	0	0
0	0	0	0	0	0	0	0	0	0	0	0	0	0
0	0	0	0	0	0	0	0	0	0	0	0	0	0
0													
OTU14442	0	0	0	0	0	1	0	0	0	0	0	0	0
0	0	0	0	0	0	0	0	0	0	0	0	0	0
0	0	0	0	0	0	0	0	0	0	0	0	0	0
0	0	0	0	0	0	0	0	0	0	0	0	0	0
0	0	0	0	0	0	0	0	0	0	0	0	0	0
0	0	0	0	0	0	0	0	0	0	0	0	0	0
0	0	0	0	0	0	0	0	0	0	0	0	0	0
0	0	0	0	0	0	0	0	0	0	0	0	0	0
0													

OTU3272 0 0 0 0 0 1 0 0 0 0 0 0 0
0 2 0 0 0 0 0 0 0 0 0 0 0 0
0 0 0 0 0 0 0 0 0 0 1 0 0 0
0 0 0 0 0 2 0 3 0 0 0 0 0 0
0 5 0 0 1 0 0 0 0 0 0 0 0 0
0 1 0 0 2 0 0 0 0 1 0 0 0 2
0 0 0 0 0 0 0 0 0 0 0 1 1 0
0 3 1 5 1 0 0 10 1 1 0 0 0 0
0
OTU15756 0 0 0 0 0 1 0 0 0 0 0 0 0
0 0 0 0 0 0 0 0 0 0 0 0 0 0
0 0 0 0 0 0 0 0 0 0 0 0 0 0
0 0 0 0 0 0 0 0 0 0 0 0 0 0
0 0 0 0 0 0 0 0 0 0 0 0 0 0
0 0 0 0 0 0 0 0 0 0 0 0 0 0
0 0 0 0 0 0 0 0 0 0 0 0 0 0
0 0 0 0 0 0 0 0 0 0 0 0 0 0
0
OTU7811 0 0 0 0 0 1 0 0 0 0 0 0 0
0 0 0 0 0 0 0 0 0 0 0 0 0 0
0 0 0 0 0 0 0 0 0 0 0 0 0 0
0 0 0 0 0 0 0 0 0 0 0 0 0 0
0 0 0 0 0 0 0 0 0 0 0 0 0 0
0 0 0 0 0 0 0 0 0 0 0 0 0 0
0 0 0 0 0 0 0 0 0 0 0 0 0 0
0 0 0 0 0 0 0 0 0 0 0 0 0 0
0
OTU12133 0 0 0 0 0 1 0 0 0 0 0 0 0
0 0 0 0 0 0 0 0 0 0 0 0 0 0
0 0 0 0 0 0 0 0 0 0 0 0 0 0
0 0 0 0 0 0 0 0 0 0 0 0 0 0
0 0 0 0 0 0 0 0 0 0 0 0 0 0
0 0 0 0 0 0 0 0 0 0 0 0 0 0

0	0	0	0	0	0	0	0	0	0	0	0	0	0
0	0	0	0	0	0	0	0	0	0	0	0	0	0
0													
OTU7806	0	0	0	0	0	1	0	0	0	0	0	0	0
0	0	0	0	0	0	0	0	0	0	0	0	0	0
0	0	0	0	0	0	0	0	0	0	0	0	0	0
0	0	0	0	0	0	0	0	0	0	0	0	0	0
0	0	0	0	0	0	0	0	0	0	0	0	0	0
0	0	0	0	0	0	0	0	0	0	0	0	0	0
0	0	0	0	0	0	0	0	0	0	0	0	0	0
0	0	0	0	0	0	0	0	0	0	0	0	0	0
0													
OTU10137	0	0	0	0	0	1	0	1	0	0	0	1	0
0	0	0	0	0	0	0	0	0	0	0	0	0	0
1	0	0	0	0	0	0	0	0	0	0	0	2	0
1	1	0	0	0	0	0	1	0	0	0	0	0	0
0	0	0	2	0	0	0	0	0	0	0	0	1	0
0	0	0	0	0	0	0	0	0	0	0	0	0	1
0	0	0	0	0	0	0	0	0	0	0	0	0	0
0	0	0	2	0	0	0	0	0	1	0	0	0	2
0													
OTU15746	0	0	0	0	0	2	0	0	0	0	0	0	0
0	0	0	0	0	0	0	0	0	0	0	0	0	0
0	0	0	0	0	0	0	0	0	0	0	0	0	0
0	0	0	0	0	0	0	0	0	0	0	0	0	0
0	0	0	0	0	0	0	0	0	0	0	0	0	0
0	0	0	0	0	0	0	0	0	0	0	0	0	0
0	0	0	0	0	0	0	0	0	0	0	0	0	0
0	0	0	0	0	0	0	0	0	0	0	0	0	0
0													
OTU4702	0	0	0	0	0	1	1	0	0	0	0	1	0
0	0	0	0	0	0	0	0	0	0	0	0	0	0
0	1	0	0	0	0	0	1	0	0	0	0	0	0

0	0	0	0	0	0	0	0	0	0	0	0	0	1
0	0	0	0	0	0	0	0	0	3	0	0	0	0
0	0	1	0	0	0	0	1	0	0	0	0	0	0
0	0	0	0	0	0	0	0	0	0	0	0	0	1
0	0	0	0	0	0	0	0	0	0	0	0	2	0
0													
OTU15747	0	0	0	0	0	1	0	0	0	0	0	0	0
0	0	0	0	0	0	0	0	0	0	0	0	0	0
0	0	0	0	0	0	0	0	0	0	0	0	0	0
0	0	0	0	0	0	0	0	0	0	0	0	0	0
0	0	0	0	0	0	0	0	0	0	0	0	0	0
0	0	0	0	0	0	0	0	0	0	0	0	0	0
0	0	0	0	0	0	0	0	0	0	0	0	0	0
0	0	0	0	0	0	0	0	0	0	0	0	0	0
0													
OTU7805	0	0	0	0	0	1	0	0	0	0	0	0	0
0	0	0	0	0	0	0	0	0	0	0	0	0	0
0	0	0	0	0	0	0	0	0	0	0	0	0	0
0	0	0	0	0	0	0	0	0	0	0	0	0	0
0	0	0	0	0	0	0	0	0	0	0	0	0	0
0	0	0	0	0	0	0	0	0	0	0	0	0	0
0	0	0	0	0	0	0	0	0	0	0	0	0	0
0	0	0	0	0	0	0	0	0	0	0	0	0	0
0													
OTU14445	0	0	0	0	0	1	0	0	0	0	0	0	0
0	0	0	0	0	0	0	0	0	0	0	0	0	0
0	0	0	0	0	0	0	0	0	0	0	0	0	0
0	0	0	0	0	0	0	0	0	0	0	0	0	0
0	0	0	0	0	0	0	0	0	0	0	0	0	0
0	0	0	0	0	0	0	0	0	0	0	0	0	0
0	0	0	0	0	0	0	0	0	0	0	0	0	0
0	0	0	0	0	0	0	0	0	0	0	0	0	0
0													

OTU5062 0 0 0 0 0 1 0 0 0 0 0 0 0

0 0 0 0 0 0 0 0 0 0 0 0 0 0

0 0 0 0 0 0 0 0 0 0 0 0 0 0

0 0 0 0 0 0 0 0 0 0 0 0 0 1

0 0 0 0 0 0 0 0 0 0 0 0 0 0

0 0 0 0 0 0 0 0 0 0 0 0 0 0

0 0 0 0 0 0 0 0 0 0 0 0 0 0

0 0 0 0 0 0 0 0 0 0 0 1 0 0

0

OTU12134 0 0 0 0 0 1 0 0 0 0 0 0 0

0 0 0 0 0 0 0 0 0 0 0 0 0 0

0 0 0 0 0 0 0 0 0 0 0 0 0 0

0 0 0 0 0 0 0 0 0 0 0 0 0 0

0 0 0 0 0 0 0 0 0 0 0 0 0 0

0 0 0 0 0 0 0 0 0 0 0 0 0 0

0 0 0 0 0 0 0 0 0 0 0 0 0 0

0 0 0 0 0 0 0 0 0 0 0 0 0 0

0

OTU14440 0 0 0 0 0 1 0 0 0 0 0 0 0

0 0 0 0 0 0 0 0 0 0 0 0 0 0

0 0 0 0 0 0 0 0 0 0 0 0 0 0

0 0 0 0 0 0 0 0 0 0 0 0 0 0

0 0 0 0 0 0 0 0 0 0 0 0 0 0

0 0 0 0 0 0 0 0 0 0 0 0 0 0

0 0 0 0 0 0 0 0 0 0 0 0 0 0

0 0 0 0 0 0 0 0 0 0 0 0 0 0

0

OTU2512 0 0 0 0 0 1 0 0 0 0 0 0 0

0 0 0 0 0 0 0 1 0 0 0 0 0 0

0 0 0 0 0 0 0 2 0 0 0 0 0 0

0 0 0 0 0 0 1 0 0 0 0 0 0 0

0 0 0 0 0 0 0 0 0 1 0 0 0 3

0 0 0 0 0 0 0 0 0 0 0 0 0 0

0 0 1 0 0 0 0 0 0 1 0 0 0 0
0 0 0 0 0 0 0 0 0 0 0 0 1 0
0
OTU11387 0 0 0 0 0 0 2 0 0 0 0 1 0
0 1 0 0 0 0 0 1 0 0 0 0 0 0
0 0 0 0 0 0 0 0 1 0 0 0 1 0
0 0 0 2 0 0 0 2 0 0 0 0 0 2
0 0 0 1 0 0 0 0 0 0 0 0 0 0
0 0 0 0 0 0 0 0 0 1 0 0 0 0
0 0 0 0 2 0 0 0 0 1 3 1 0 0
1 0 0 1 1 0 0 0 1 0 0 1 1 0
0
OTU3317 0 0 0 0 0 0 1 0 0 0 0 0 0
0 0 0 0 0 0 0 0 0 0 1 0 0 0
0 0 0 0 0 0 0 0 0 0 0 0 0 0
0 0 0 0 0 0 0 0 0 0 0 0 0 0
0 0 0 0 0 0 0 0 0 0 0 0 0 0
0 0 0 0 0 0 0 0 0 0 0 0 0 0
0 0 0 0 1 0 0 0 0 1 0 0 0 0
0 0 0 0 0 0 0 0 0 0 0 0 0 0
0
OTU7438 0 0 0 0 0 0 1 0 0 0 0 0 0
0 0 0 0 0 0 0 0 0 0 0 0 0 0
0 0 0 0 0 0 0 0 0 0 0 0 0 0
0 0 0 0 0 0 0 0 0 0 0 0 0 0
0 0 0 0 0 0 0 0 0 0 0 0 0 0
0 0 0 0 0 0 0 0 0 0 0 0 0 0
0 1 0 0 0 0 0 0 0 0 0 0 0 0
0 0 0 0 0 0 0 0 0 0 0 0 0 0
0
OTU5249 0 0 0 0 0 0 3 2 0 3 0 0 0
0 0 0 0 0 0 0 0 0 0 0 0 0 0
3 0 0 0 0 0 0 0 0 0 0 0 0 0

1 0 0 0 0 0 0 0 0 0 0 1 0 0
0 0 0 0 0 0 0 0 0 0 0 0 0 0
0 0 0 0 0 0 0 0 0 0 0 0 0 0
0 0 0 0 0 0 0 0 0 0 0 1 0 0
0 0 0 0 0 0 0 3 0 0 0 0 0 0
0
OTU7807 0 0 0 0 0 0 1 0 0 0 0 0 0
0 0 0 0 0 0 0 0 0 0 0 0 0 0
0 0 0 0 0 0 0 0 0 0 0 0 0 0
0 0 0 0 0 0 0 0 0 0 0 0 0 0
0 0 0 0 0 0 0 0 0 0 0 0 0 0
0 0 0 0 0 0 0 0 0 0 0 0 0 0
0 0 0 0 0 0 0 0 0 0 0 0 0 0
0 0 0 0 0 0 0 0 0 0 0 0 0 0
0
OTU751 0 0 0 0 0 0 4 0 0 0 0 0 0
0 0 0 0 0 0 1 0 0 0 0 0 0 0
0 0 0 0 0 0 0 0 0 0 0 0 1 0
0 0 0 0 0 0 0 0 0 0 0 0 0 0
0 0 0 0 0 0 0 2 0 0 0 0 1 0
0 0 0 0 0 0 0 0 0 0 0 0 0 7
0 0 0 0 0 0 1 0 0 0 0 0 0 0
0 0 0 0 0 0 0 0 0 0 0 0 0 0
0
OTU725 0 0 0 0 0 0 2 0 0 0 0 0 0
0 0 0 0 0 0 0 0 0 0 0 0 0 0
0 0 0 0 0 0 0 0 0 0 0 0 0 0
0 0 0 0 1 0 0 0 0 0 0 0 0 0
0 0 0 0 0 0 0 0 0 0 0 0 2 0
0 0 1 0 0 0 0 0 0 0 0 0 1 0
0 0 0 0 0 0 0 0 0 1 0 0 1 0
0 2 2 1 1 4 1 0 3 13 0 0 0 8
1

OTU15748	0	0	0	0	0	0	1	0	0	0	0	0	0
0	0	0	0	0	0	0	0	0	0	0	0	0	0
0	0	0	0	0	0	0	0	0	0	0	0	0	0
0	0	0	0	0	0	0	0	0	0	0	0	0	0
0	0	0	0	0	0	0	0	0	0	0	0	0	0
0	0	0	0	0	0	0	0	0	0	0	0	0	0
0	0	0	0	0	0	0	0	0	0	0	0	0	0
0	0	0	0	0	0	0	0	0	0	0	0	0	0
0													
OTU321	0	0	0	0	0	0	1	0	0	2	0	2	0
0	0	0	5	0	0	3	0	2	0	0	0	3	1
0	89	0	0	2	0	15	0	0	0	29	0	0	0
1	1	4	1	0	0	0	0	2	1	0	0	0	0
3	2	21	0	0	49	0	0	0	0	1	0	0	0
0	0	0	0	4	0	0	0	0	0	2	7	1	20
0	0	0	0	0	0	0	0	0	0	0	2	0	0
0	0	18	2	0	0	28	1	0	0	0	0	0	0
0													
OTU12132	0	0	0	0	0	0	1	0	0	0	0	0	0
0	0	0	0	0	0	0	0	0	0	0	0	0	0
0	0	0	0	0	0	0	0	0	0	0	0	0	0
0	0	0	0	0	0	0	0	0	0	0	0	0	0
0	0	0	0	0	0	0	0	0	0	0	0	0	0
0	0	0	0	0	0	0	0	0	0	0	0	0	0
0	0	0	0	0	0	0	0	0	0	0	0	0	0
0	0	0	0	0	0	0	0	0	0	0	0	0	0
0													
OTU428	0	0	0	0	0	0	1	0	0	0	0	0	0
0	0	1	12	1	0	2	0	7	0	1	0	0	0
0	1	1	0	2	1	4	0	2	3	5	3	0	0
0	0	2	0	6	0	0	0	1	5	2	2	0	0
2	6	1	0	2	8	1	2	4	2	14	0	4	0
14	0	2	1	0	2	1	4	1	1	0	5	0	0

0	2	0	2	0	0	0	1	2	0	0	0	0	0
0	0	2	0	0	0	0	0	0	0	0	0	0	2
0													
OTU15742	0	0	0	0	0	0	1	0	0	0	0	0	0
0	0	0	0	0	0	0	0	0	0	0	0	0	0
0	0	0	0	0	0	0	0	0	0	0	0	0	0
0	0	0	0	0	0	0	0	0	0	0	0	0	0
0	0	0	0	0	0	0	0	0	0	0	0	0	0
0	0	0	0	0	0	0	0	0	0	0	0	0	0
0	0	0	0	0	0	0	0	0	0	0	0	0	0
0	0	0	0	0	0	0	0	0	0	0	0	0	0
0													
OTU7081	0	0	0	0	0	0	1	0	0	0	0	0	0
0	0	0	0	0	0	0	0	0	0	0	0	0	0
0	0	0	0	0	0	0	0	0	0	0	0	0	0
0	0	0	0	0	0	0	3	0	0	0	0	0	0
0	0	0	0	0	0	0	0	0	0	0	0	0	0
0	5	0	0	0	0	0	0	0	0	0	0	0	0
0	0	0	0	0	0	0	0	0	0	0	25	43	0
0	3	0	0	0	0	0	25	0	0	0	0	0	1
15													
OTU7789	0	0	0	0	0	0	1	0	0	0	0	0	0
0	0	0	0	0	0	0	0	0	0	0	0	0	0
0	0	0	0	0	0	0	0	0	0	0	0	0	0
0	0	0	0	0	0	0	0	0	0	0	0	0	0
0	0	0	0	0	0	0	0	0	0	0	0	0	0
0	0	0	0	0	0	0	0	0	0	0	0	0	0
0	0	0	0	0	0	0	0	0	0	0	0	0	0
0	0	0	0	0	0	0	0	0	0	0	0	0	0
0													
OTU932	0	0	0	0	0	0	3	1	0	0	0	0	0
0	0	0	0	0	0	0	0	0	0	0	0	0	0
0	0	0	0	0	0	0	0	0	0	0	0	0	0

0 0 0 0 0 0 0 0 0 0 0 0 0 0
0 0 0 0 0 0 0 0 0 0 0 0 0 0
0 0 0 0 0 0 0 0 0 0 0 0 0 0
0 0 0 0 0 0 0 0 0 0 0 0 0 0
0 0 0 0 0 0 0 0 0 0 0 0 0 0
0
OTU7790 0 0 0 0 0 0 1 0 0 0 0 0 0
0 0 0 0 0 0 0 0 0 0 0 0 0 0
0 0 0 0 0 0 0 0 0 0 0 0 0 0
0 0 0 0 0 0 0 0 0 0 0 0 0 0
0 0 0 0 0 0 0 0 0 0 0 0 0 0
0 0 0 0 0 0 0 0 0 0 0 0 0 0
0 0 0 0 0 0 0 0 0 0 0 0 0 0
0 0 0 0 0 0 0 0 0 0 0 0 0 0
0
OTU12130 0 0 0 0 0 0 1 0 0 0 0 0 0
0 0 0 0 0 0 0 0 0 0 0 0 0 0
0 0 0 0 0 0 0 0 0 0 0 0 0 0
0 0 0 0 0 0 0 0 0 0 0 0 0 0
0 0 0 0 0 0 0 0 0 0 0 0 0 0
0 0 0 0 0 0 0 0 0 0 0 0 0 0
0 0 0 0 0 0 0 0 0 0 0 0 0 0
0 0 0 0 0 0 0 0 0 0 0 0 0 0
0
OTU12131 0 0 0 0 0 0 2 0 0 0 0 0 0
0 0 0 0 0 0 0 0 0 0 0 0 0 0
0 0 0 0 0 0 0 0 0 0 0 0 0 0
0 0 0 0 0 0 0 0 0 0 0 0 0 0
0 0 0 0 0 0 0 0 0 0 0 0 0 0
0 0 0 0 0 0 0 0 0 0 0 0 0 0
0 0 0 0 0 0 0 0 0 0 0 0 0 0
0 0 0 0 0 0 0 0 0 0 0 0 0 0
0

OTU1189	0	0	0	0	0	0	5	0	5	10	0	1	0
0	0	0	0	0	0	2	0	0	0	0	0	0	2
0	0	0	0	0	0	0	0	0	0	0	0	0	0
10	0	0	0	0	0	0	1	0	0	0	0	0	0
0	1	0	0	0	1	1	0	0	0	0	0	5	0
1	119	1	0	12	0	0	11	0	7	0	7	0	39
0	7	0	67	0	4	0	36	0	0	0	93	26	0
0	69	6	0	39	11	19	164	21	1	0	3	0	0
16													
OTU15740	0	0	0	0	0	0	1	0	0	0	0	0	0
0	0	0	0	0	0	0	0	0	0	0	0	0	0
0	0	0	0	0	0	0	0	0	0	0	0	0	0
0	0	0	0	0	0	0	0	0	0	0	0	0	0
0	0	0	0	0	0	0	0	0	0	0	0	0	0
0	0	0	0	0	0	0	0	0	0	0	0	0	0
0	0	0	0	0	0	0	0	0	0	0	0	0	0
0	0	0	0	0	0	0	0	0	0	0	0	0	0
0													
OTU7791	0	0	0	0	0	0	1	0	0	0	0	0	0
0	0	0	0	0	0	0	0	0	0	0	0	0	0
0	0	0	0	0	0	0	0	0	0	0	0	0	0
0	0	0	0	0	0	0	0	0	0	0	0	0	0
0	0	0	0	0	0	0	0	0	0	0	0	0	0
0	0	0	0	0	0	0	0	0	0	0	0	0	0
0	0	0	0	0	0	0	0	0	0	0	0	0	0
0	0	0	0	0	0	0	0	0	0	0	0	0	0
0													
OTU13150	0	0	0	0	0	0	1	0	0	0	0	0	0
0	0	0	0	0	1	0	0	0	0	0	0	0	0
0	0	0	0	0	1	0	0	0	0	0	0	0	0
0	0	0	0	0	0	0	0	0	0	0	0	0	0
0	0	0	0	0	0	0	0	0	0	0	0	0	0
0	0	0	0	0	0	0	0	0	0	0	0	0	0

0	0	0	0	0	0	0	0	0	0	0	0	0	0
0	0	0	0	0	0	0	0	0	0	0	0	0	0
0													
OTU15741	0	0	0	0	0	0	1	0	0	0	0	0	0
0	0	0	0	0	0	0	0	0	0	0	0	0	0
0	0	0	0	0	0	0	0	0	0	0	0	0	0
0	0	0	0	0	0	0	0	0	0	0	0	0	0
0	0	0	0	0	0	0	0	0	0	0	0	0	0
0	0	0	0	0	0	0	0	0	0	0	0	0	0
0	0	0	0	0	0	0	0	0	0	0	0	0	0
0	0	0	0	0	0	0	0	0	0	0	0	0	0
0													
OTU614	0	0	0	0	0	0	1	0	2	0	0	0	0
0	0	0	0	0	0	0	0	0	0	0	0	0	0
0	0	0	0	0	0	0	0	0	0	0	0	1	0
0	0	0	0	0	0	0	0	0	0	0	0	0	0
0	1	0	2	0	0	0	0	0	0	0	0	0	0
0	0	0	0	2	0	0	0	0	2	0	0	0	3
0	0	0	0	0	0	0	2	0	0	0	0	1	0
0	0	2	1	1	1	0	2	3	1	0	0	0	0
0													
OTU510	0	0	0	0	0	0	5	1	0	3	0	0	0
0	0	0	1	0	0	0	0	2	0	0	0	0	0
0	10	0	0	0	0	0	0	0	0	0	0	0	0
0	0	0	0	0	0	0	0	0	0	0	0	0	0
0	0	0	0	0	0	0	0	0	0	0	0	0	0
0	0	0	0	0	0	0	5	0	26	0	0	0	0
0	0	0	0	0	0	0	0	0	0	0	0	0	0
0	0	0	0	0	0	0	0	0	0	0	0	0	0
0													
OTU136	0	0	0	0	0	0	1	0	0	0	0	2	2
0	0	0	0	0	0	0	0	0	0	0	0	0	0
0	0	1	0	0	0	0	0	0	0	0	0	0	0

0 0 1 0 0 0 0 6 0 0 0 0 0 0
0 0 0 0 0 0 1 0 1 1 0 0 0 0
0 50 0 0 0 0 0 3 0 0 1 0 0 0
0 0 0 0 0 0 0 0 0 0 0 0 73 0
0 4 0 179 2 4 198 0 7 260 0 0 1 20
80
OTU1340 0 0 0 0 0 0 1 0 0 0 0 0 0
0 1 0 0 0 0 0 0 1 0 0 0 0 0
0 0 1 0 0 0 0 0 0 0 0 0 0 1
0 0 0 0 0 0 0 0 0 1 0 0 0 0
0 0 0 0 0 0 0 0 0 0 0 0 0 0
0 0 0 0 1 0 0 0 0 0 0 0 0 0
0 0 0 0 0 0 0 0 0 0 0 0 0 0
0 0 0 1 0 0 0 0 0 0 0 0 0 0
0
OTU14473 0 0 0 0 0 0 1 0 0 0 0 0 0
0 0 0 0 0 0 0 0 0 0 0 0 0 0
0 0 0 0 0 0 0 0 0 0 0 0 0 0
0 0 0 0 0 0 0 0 0 0 0 0 0 0
0 0 0 0 0 0 0 0 0 0 0 0 0 0
0 0 0 0 0 0 0 0 0 0 0 0 0 0
0 0 0 0 0 0 0 0 0 0 0 0 0 0
0 0 0 0 0 0 0 0 0 0 0 0 0 0
0
OTU6646 0 0 0 0 0 0 1 0 0 0 0 0 0
0 0 0 0 0 0 0 0 0 0 0 0 0 0
0 0 0 0 0 0 0 0 0 0 0 0 1 0
0 0 0 0 0 0 0 0 0 0 0 0 0 0
0 0 0 1 0 0 0 0 0 0 0 0 0 0
0 0 0 0 0 0 0 0 0 0 0 0 0 0
0 0 0 0 0 0 0 1 0 0 0 0 0 0
0 0 0 0 0 0 0 0 0 0 0 0 0 0
0

OTU15744	0	0	0	0	0	0	1	0	0	0	0	0	0
0	0	0	0	0	0	0	0	0	0	0	0	0	0
0	0	0	0	0	0	0	0	0	0	0	0	0	0
0	0	0	0	0	0	0	0	0	0	0	0	0	0
0	0	0	0	0	0	0	0	0	0	0	0	0	0
0	0	0	0	0	0	0	0	0	0	0	0	0	0
0	0	0	0	0	0	0	0	0	0	0	0	0	0
0	0	0	0	0	0	0	0	0	0	0	0	0	0
0													
OTU472	0	0	0	0	0	0	1	0	0	0	0	0	0
0	0	0	1	0	0	37	0	0	0	0	0	0	0
9	0	0	0	0	0	0	0	0	0	0	0	0	0
2	3	0	0	0	0	0	0	0	0	0	2	0	0
0	0	0	0	0	0	0	0	0	0	0	0	4	0
0	6	0	0	0	0	0	0	0	1	1	0	0	0
0	0	0	0	0	0	0	0	0	0	0	22	1	0
0	27	17	8	38	22	6	10	1	2	0	1	0	5
0													
OTU4710	0	0	0	0	0	0	1	0	0	0	0	0	0
0	1	0	0	0	0	0	0	0	0	0	0	0	0
0	0	0	0	0	1	0	0	2	0	1	0	0	0
0	0	0	0	0	1	0	0	0	0	0	0	0	1
0	0	0	0	0	0	0	0	0	0	0	0	0	0
0	0	1	0	0	0	0	0	1	0	0	0	0	0
0	0	0	0	0	0	0	0	0	0	0	0	0	0
0	0	0	0	0	0	0	1	0	0	0	0	0	0
0													
OTU2898	0	0	0	0	0	0	6	0	0	6	0	1	0
0	0	0	0	0	0	0	0	0	0	0	0	0	0
1	0	0	0	0	0	0	0	0	0	0	0	0	0
0	2	0	0	0	4	0	4	0	0	0	1	0	0
0	0	0	1	0	0	0	0	0	0	0	0	12	0
0	8	0	0	3	0	0	0	0	0	1	0	0	0

0	0	0	0	0	1	0	0	0	0	0	10	2	0
0	20	2	3	2	4	4	11	1	6	0	0	0	0
16													
OTU1045	0	0	0	0	0	0	3	0	0	0	0	0	0
0	0	0	0	0	0	0	0	0	0	0	0	0	0
0	0	0	0	0	0	0	0	0	0	0	0	0	0
1	0	0	0	0	0	0	0	0	0	0	0	0	0
0	0	0	0	0	0	0	0	0	0	0	0	0	0
0	0	0	0	3	0	0	0	0	0	0	0	0	1
0	0	0	1	0	1	0	0	0	0	0	0	0	0
0	0	0	0	0	0	0	0	0	0	0	0	0	0
0													
OTU1248	0	0	0	0	0	0	6	0	0	1	0	0	0
0	0	0	0	0	0	0	0	0	0	0	0	0	0
0	0	0	0	0	0	0	0	0	0	0	0	0	0
0	0	0	0	0	0	0	0	0	0	0	0	0	0
0	0	0	0	0	0	0	0	0	0	0	0	0	0
0	9	0	0	1	0	0	0	0	0	0	0	0	0
0	0	0	0	0	0	0	0	0	0	0	1	1	0
0	25	0	0	0	0	0	4	0	0	0	0	0	0
0													
OTU15175	0	0	0	0	0	0	1	0	0	0	0	0	0
0	0	0	0	0	0	0	0	0	0	0	1	0	0
0	0	0	0	0	0	0	0	0	0	0	0	0	0
0	0	0	0	0	0	0	0	0	0	0	0	0	0
0	0	0	0	0	0	0	0	0	0	0	0	0	0
0	0	0	0	0	0	0	0	0	0	0	0	0	0
0	0	0	0	0	0	0	0	0	0	0	0	0	0
0	0	0	0	0	0	0	0	0	0	0	0	0	0
0													
OTU15743	0	0	0	0	0	0	1	0	0	0	0	0	0
0	0	0	0	0	0	0	0	0	0	0	0	0	0
0	0	0	0	0	0	0	0	0	0	0	0	0	0

0	0	0	0	0	0	0	0	0	0	0	0	0	0
0	0	0	0	0	0	0	0	0	0	0	0	0	0
0	0	0	0	0	0	0	0	0	0	0	0	0	0
0	0	0	0	0	0	0	0	0	0	0	0	0	0
0	0	0	0	0	0	0	0	0	0	0	0	0	0
0													
OTU12138	0	0	0	0	0	0	1	0	0	0	0	0	0
0	0	0	0	0	0	0	0	0	0	0	0	0	0
0	0	0	0	0	0	0	0	0	0	0	0	0	0
0	0	0	0	0	0	0	0	0	0	0	0	0	0
0	0	0	0	0	0	0	0	0	0	0	0	0	0
0	0	0	0	0	0	0	0	0	0	0	0	0	0
0	0	0	0	0	0	0	0	0	0	0	0	0	0
0	0	0	0	0	0	0	0	0	0	0	0	0	0
0													
OTU14474	0	0	0	0	0	0	1	0	0	0	0	0	0
0	0	0	0	0	0	0	0	0	0	0	0	0	0
0	0	0	0	0	0	0	0	0	0	0	0	0	0
0	0	0	0	0	0	0	0	0	0	0	0	0	0
0	0	0	0	0	0	0	0	0	0	0	0	0	0
0	0	0	0	0	0	0	0	0	0	0	0	0	0
0	0	0	0	0	0	0	0	0	0	0	0	0	0
0	0	0	0	0	0	0	0	0	0	0	0	0	0
0													
OTU3257	0	0	0	0	0	0	3	0	4	11	0	0	0
0	2	0	0	0	0	0	1	0	0	0	0	0	0
0	0	0	0	0	0	0	0	0	0	0	0	0	0
7	0	0	0	0	0	0	0	0	0	0	0	0	1
0	0	0	0	0	0	0	0	0	0	0	0	1	0
0	0	1	0	0	0	0	1	0	2	0	0	0	2
0	0	0	14	0	0	0	26	0	0	0	1	1	0
0	0	1	0	2	1	3	0	2	1	0	0	0	2
0													

OTU12101 0 0 0 0 0 0 1 2 0 0 0 0 0
0 0 0 1 0 0 0 0 0 0 0 0 0 0
0 1 0 0 0 0 0 0 0 0 0 0 0 0
1 0 0 1 0 0 0 1 0 0 0 0 0 0
0 0 0 0 0 0 0 0 0 0 0 0 0 0
0 0 0 1 0 0 0 0 0 0 0 0 1 0
0 0 0 0 1 0 0 0 0 0 0 0 0 0
0 0 0 0 0 0 0 0 0 0 0 0 0 0
0
OTU2094 0 0 0 0 0 0 1 0 0 0 0 0 0
0 0 0 0 0 0 0 0 0 0 0 0 0 0
0 0 0 0 0 0 0 0 0 0 0 0 0 0
0 0 0 0 0 0 0 0 0 0 0 0 0 0
0 0 0 0 0 0 0 0 0 0 0 0 0 0
0 1 0 0 0 0 0 0 0 0 0 0 0 0
0 0 0 0 0 0 0 0 0 0 0 0 1 0
0 0 0 0 0 0 0 0 0 0 0 0 0 0
0
OTU1287 0 0 0 0 0 0 1 0 0 0 0 0 0
0 0 0 0 0 0 0 0 0 0 0 0 0 0
0 0 0 0 0 0 0 0 0 0 0 0 0 0
0 0 0 0 0 0 0 0 0 0 0 1 0 0
0 0 0 0 0 0 0 0 0 0 1 0 0 0
0 0 0 0 0 0 0 0 0 0 0 0 0 0
0 1 0 1 0 0 0 1 0 0 0 0 0 0
0 1 0 0 0 0 1 0 3 2 0 1 0 0
1
OTU12139 0 0 0 0 0 0 1 0 0 0 0 0 0
0 0 0 0 0 0 0 0 0 0 0 0 0 0
0 0 0 0 0 0 0 0 0 0 0 0 0 0
0 0 0 0 0 0 0 0 0 0 0 0 0 0
0 0 0 0 0 0 0 0 0 0 0 0 0 0
0 0 0 0 0 0 0 0 0 0 0 0 0 0

0 0 0 0 0 0 0 0 0 0 0 0 0 0
0 0 0 0 0 0 0 0 0 0 0 0 0 0
0
OTU6590 0 0 0 0 0 0 1 0 0 0 0 0 0
0 0 0 0 0 0 0 0 0 0 0 0 0 0
0 0 0 0 0 0 0 0 1 0 0 0 0 0
0 0 0 0 0 0 0 0 0 0 0 0 0 1
0 0 0 0 0 0 0 0 0 0 0 0 0 0
0 0 0 0 0 0 0 0 0 0 0 0 0 0
0 0 0 0 0 0 0 0 0 0 0 0 0 0
0 0 0 0 0 0 0 0 0 0 0 0 0 0
0
OTU63 0 0 0 0 0 0 2 0 0 0 355 22 808 373
0 18 0 1 5 0 2 0 5 1 21 0 3 0
0 192 8 0 1 0 64 0 1 0 24 0 56 0
0 0 247 0 0 9 0 0 0 0 0 0 0 0
0 1 0 0 0 0 22 0 0 0 1 0 0 0
0 0 0 0 237 1 0 0 0 29 0 2 0 0
0 3 1 1 0 1 0 83 1 3 0 0 3 5
0 3 0 0 0 0 0 0 0 6 19 1 0 0
OTU14471 0 0 0 0 0 0 1 0 0 0 0 0 0
0 0 0 0 0 0 0 0 0 0 0 0 0 0
0 0 0 0 0 0 0 0 0 0 0 0 0 0
0 0 0 0 0 0 0 0 0 0 0 0 0 0
0 0 0 0 0 0 0 0 0 0 0 0 0 0
0 0 0 0 0 0 0 0 0 0 0 0 0 0
0 0 0 0 0 0 0 0 0 0 0 0 0 0
0 0 0 0 0 0 0 0 0 0 0 0 0 0
0
OTU14470 0 0 0 0 0 0 1 0 0 0 0 0 0
0 0 0 0 0 0 0 0 0 0 0 0 0 0
0 0 0 0 0 0 0 0 0 0 0 0 0 0
0 0 0 0 0 0 0 0 0 0 0 0 0 0

0	0	0	0	0	0	0	0	0	0	0	0	0	0
0	0	0	0	0	0	0	0	0	0	0	0	0	0
0	0	0	0	0	0	0	0	0	0	0	0	0	0
0	0	0	0	0	0	0	0	0	0	0	0	0	0
0													
OTU7798	0	0	0	0	0	0	1	0	0	0	0	0	0
0	0	0	0	0	0	0	0	0	0	0	0	0	0
0	0	0	0	0	0	0	0	0	0	0	0	0	0
0	0	0	0	0	0	0	0	0	0	0	0	0	0
0	0	0	0	0	0	0	0	0	0	0	0	0	0
0	0	0	0	0	0	0	0	0	0	0	0	0	0
0	0	0	0	0	0	0	0	0	0	0	0	0	0
0	0	0	0	0	0	0	0	0	0	0	0	0	0
0													
OTU380	0	0	0	0	0	0	3	1	1	0	44	1	106
21	0	0	1	2	1	0	0	0	0	1	0	0	1
0	0	40	0	0	0	0	13	0	0	0	0	0	0
0	0	0	0	0	0	85	0	0	0	0	0	0	0
2	0	0	0	0	0	0	3	0	0	0	0	0	0
0	0	0	0	0	0	0	0	0	0	0	0	2	0
0	0	0	0	0	0	0	0	2	2	6	3	0	4
1	0	0	3	0	1	1	0	0	0	3	3	0	0
0													
OTU3254	0	0	0	0	0	0	1	0	0	0	0	0	0
0	0	0	0	0	0	0	0	0	0	0	0	0	0
0	0	0	0	0	0	0	0	0	0	0	0	0	0
0	0	0	0	0	0	0	0	0	0	0	0	0	0
0	0	0	0	0	0	0	0	0	0	0	0	2	0
0	1	0	0	0	0	0	0	0	0	0	0	0	0
0	0	0	3	0	0	0	0	0	0	0	0	0	0
0	1	1	0	0	0	0	1	0	1	0	0	0	0
1													

OTU7800	0	0	0	0	0	0	1	0	0	0	0	0	0
0	0	0	0	0	0	0	0	0	0	0	0	0	0
0	0	0	0	0	0	0	0	0	0	0	0	0	0
0	0	0	0	0	0	0	0	0	0	0	0	0	0
0	0	0	0	0	0	0	0	0	0	0	0	0	0
0	0	0	0	0	0	0	0	0	0	0	0	0	0
0	0	0	0	0	0	0	0	0	0	0	0	0	0
0	0	0	0	0	0	0	0	0	0	0	0	0	0
0													
OTU955	0	0	0	0	0	0	1	1	0	0	0	0	0
0	0	0	0	0	0	0	0	0	0	0	0	0	0
0	0	0	0	0	0	0	0	0	0	0	0	0	0
0	1	0	0	0	0	0	0	0	0	0	0	0	2
0	0	0	0	0	0	0	0	0	0	0	0	1	0
0	0	0	0	0	0	0	0	1	0	0	0	0	0
0	0	0	0	0	0	0	0	0	0	0	0	2	0
0	1	0	1	5	0	0	0	0	2	0	0	0	0
0													
OTU10388	0	0	0	0	0	0	1	0	0	0	0	0	0
0	0	0	0	0	0	0	0	0	0	0	0	0	0
0	0	0	0	0	0	0	0	0	0	0	0	0	0
0	0	0	0	0	0	0	0	0	0	0	0	0	0
0	0	0	0	0	0	0	0	0	0	0	0	0	0
0	0	0	0	5	0	0	0	0	1	0	0	0	1
0	0	0	2	0	0	0	0	0	0	0	0	0	0
0	0	0	0	0	0	0	0	0	0	0	0	0	0
0													
OTU13577	0	0	0	0	0	0	2	0	0	2	0	0	0
0	1	0	0	0	0	0	0	0	0	0	0	3	0
0	0	0	3	0	0	2	0	1	0	0	0	1	0
0	0	0	0	0	0	0	6	0	0	0	0	0	0
0	0	0	1	0	0	0	0	3	0	0	0	0	0
0	0	1	0	0	0	0	0	0	1	1	0	0	0

1	0	0	1	0	0	2	0	0	0	0	0	0	0
0	0	0	0	0	0	0	0	0	0	0	0	0	0
0													
OTU12135	0	0	0	0	0	0	1	0	0	0	0	0	0
0	0	0	0	0	0	0	0	0	0	0	0	0	0
0	0	0	0	0	0	0	0	0	0	0	0	0	0
0	0	0	0	0	0	0	0	0	0	0	0	0	0
0	0	0	0	0	0	0	0	0	0	0	0	0	0
0	0	0	0	0	0	0	0	0	0	0	0	0	0
0	0	0	0	0	0	0	0	0	0	0	0	0	0
0	0	0	0	0	0	0	0	0	0	0	0	0	0
0													
OTU15749	0	0	0	0	0	0	1	0	0	0	0	0	0
0	0	0	0	0	0	0	0	0	0	0	0	0	0
0	0	0	0	0	0	0	0	0	0	0	0	0	0
0	0	0	0	0	0	0	0	0	0	0	0	0	0
0	0	0	0	0	0	0	0	0	0	0	0	0	0
0	0	0	1	0	0	0	0	0	0	0	0	0	0
0	0	0	0	0	0	0	0	0	0	0	0	0	0
0	0	0	0	0	0	0	0	0	0	0	0	0	0
0													
OTU7792	0	0	0	0	0	0	1	0	0	0	0	0	0
0	0	0	0	0	0	0	0	0	0	0	0	0	0
0	0	0	0	0	0	0	0	0	0	0	0	0	0
0	0	0	0	0	0	0	0	0	0	0	0	0	0
0	0	0	0	0	0	0	0	0	0	0	0	0	0
0	0	0	0	0	0	0	0	0	0	0	0	0	0
0	0	0	0	0	0	0	0	0	0	0	0	0	0
0	0	0	0	0	0	0	0	0	0	0	0	0	0
0													
OTU1185	0	0	0	0	0	0	2	0	0	0	0	0	0
0	0	0	0	0	0	0	0	0	0	0	0	0	0
0	0	0	0	0	0	0	0	0	0	1	0	1	0

1	0	0	0	0	0	0	0	0	0	1	0	0	0
0	1	0	0	0	0	0	0	0	0	0	0	0	0
0	0	0	0	0	0	0	0	0	1	0	0	0	0
0	0	0	0	0	1	0	0	0	0	0	0	3	0
0	0	0	0	0	0	0	0	0	0	0	0	0	4
0													
OTU7793	0	0	0	0	0	0	1	0	0	0	0	0	0
0	0	0	0	0	0	0	0	0	0	0	0	0	0
0	0	0	0	0	0	0	0	0	0	0	0	0	0
0	0	0	0	0	0	0	0	0	0	0	0	0	0
0	0	0	0	0	0	0	0	0	0	0	0	0	0
0	0	0	0	0	0	0	0	0	0	0	0	0	0
0	0	0	0	0	0	0	0	0	0	0	0	0	0
0	0	0	0	0	0	0	0	0	0	0	0	0	0
0													
OTU12137	0	0	0	0	0	0	1	0	0	0	0	0	0
0	0	0	0	0	0	0	0	0	0	0	0	0	0
0	0	0	0	0	0	0	0	0	0	0	0	0	0
0	0	0	0	0	0	0	0	0	0	0	0	0	0
0	0	0	0	0	0	0	0	0	0	0	0	0	0
0	0	0	0	0	0	0	0	0	0	0	0	0	0
0	0	0	0	0	0	0	0	0	0	0	0	0	0
0	0	0	0	0	0	0	0	0	0	0	0	0	0
0													
OTU7794	0	0	0	0	0	0	1	0	0	0	0	0	0
0	0	0	0	1	0	0	0	0	0	0	0	0	0
0	1	0	0	0	0	0	0	0	0	0	0	1	0
0	0	0	0	2	0	0	0	0	0	0	0	0	0
0	0	0	0	0	0	0	0	0	0	0	0	0	0
0	0	0	0	0	0	0	0	0	0	0	0	0	0
0	0	0	0	0	0	0	0	1	0	0	0	0	1
0	0	0	0	0	0	0	0	0	0	0	0	0	0
0													

OTU14348	0	0	0	0	0	0	2	0	0	0	0	0	0
0	0	0	0	0	0	0	0	0	0	0	0	0	0
0	0	0	0	0	0	0	0	0	0	0	0	0	0
0	0	0	0	0	0	0	0	0	0	0	0	0	0
0	0	0	0	0	0	0	0	0	0	0	0	0	0
0	0	0	0	0	0	0	0	0	0	0	0	0	0
0	0	0	0	0	0	0	0	0	0	0	0	0	0
0	3	2	2	8	1	4	0	1	7	0	0	0	1
0													
OTU7643	0	0	0	0	0	0	2	1	0	2	0	0	0
0	6	0	0	0	0	0	0	0	0	0	0	0	0
0	0	0	0	0	1	0	0	0	0	0	0	0	0
0	0	0	2	0	0	0	0	1	0	0	0	0	0
0	0	0	0	0	0	0	0	0	0	0	0	1	0
0	0	0	0	0	1	0	0	0	0	0	0	1	0
0	1	0	0	0	0	0	0	0	0	0	0	0	0
0	0	0	0	0	0	0	0	0	0	0	0	0	0
0													
OTU5717	0	0	0	0	0	0	2	0	0	0	0	0	0
0	0	0	0	0	0	0	0	0	0	0	0	0	0
0	0	0	0	0	0	0	0	0	0	1	0	0	0
0	0	0	0	1	0	0	0	0	0	0	0	0	0
0	0	0	0	0	0	0	1	0	0	0	0	0	2
0	0	0	0	0	0	0	0	0	0	0	0	0	0
0	0	0	0	0	0	0	0	0	0	0	0	0	0
0	0	0	0	0	0	0	0	0	0	0	0	0	0
0													
OTU7797	0	0	0	0	0	0	1	0	0	0	0	0	0
0	0	0	0	0	0	0	0	0	0	0	0	0	0
0	0	0	0	0	0	0	0	0	0	0	0	0	0
0	0	0	0	0	0	0	0	0	0	0	0	0	0
0	0	0	0	0	0	0	0	0	0	0	0	0	0
0	0	0	0	0	0	0	0	0	0	0	0	0	0

0	0	0	0	0	0	0	0	0	0	0	0	0	0
0	0	0	0	0	0	0	0	0	0	0	0	0	0
0													
OTU12136	0	0	0	0	0	0	1	0	0	0	0	0	0
0	0	0	0	0	0	0	0	0	0	0	0	0	0
0	0	0	0	0	0	0	0	0	0	0	0	0	0
0	0	0	0	0	0	0	0	0	0	0	0	0	0
0	0	0	0	0	0	0	0	0	0	0	0	0	0
0	0	0	0	0	0	0	0	0	0	0	0	0	0
0	0	0	0	0	0	0	0	0	0	0	0	0	0
0	0	0	0	0	0	0	0	0	0	0	0	0	0
0													
OTU7795	0	0	0	0	0	0	1	0	0	0	0	0	0
0	0	0	0	0	0	0	0	0	0	0	0	0	0
0	0	0	0	0	0	0	0	0	0	0	0	0	0
0	0	0	0	0	0	0	0	0	0	0	0	0	0
0	0	0	0	0	0	0	0	0	0	0	0	0	0
0	0	0	0	0	0	0	0	0	0	0	0	0	0
0	0	0	0	0	0	0	0	0	0	0	0	0	0
0	0	0	0	0	0	0	0	0	0	0	0	0	0
0													
OTU12117	0	0	0	0	0	0	1	0	0	0	0	0	0
0	0	0	0	0	0	0	0	0	0	0	0	0	0
0	0	0	0	0	0	0	0	0	0	0	0	0	0
0	0	0	0	0	0	0	0	0	0	0	0	0	0
0	0	0	0	0	0	0	0	0	0	0	0	0	0
0	0	0	0	1	0	0	0	0	0	0	0	0	0
0	0	0	0	0	0	0	0	0	0	0	0	0	0
0	0	0	0	0	0	0	0	0	0	0	0	0	0
0													
OTU566	0	0	0	0	0	0	1	0	0	0	0	0	0
0	0	0	0	0	0	0	0	0	0	0	0	0	0
0	0	0	0	0	0	0	0	0	0	0	0	0	0

5	0	0	0	0	0	0	0	0	0	0	0	0	0
0	0	0	0	0	0	0	0	0	0	0	0	0	0
0	0	27	0	0	0	0	0	0	1	0	0	0	0
0	0	0	0	0	0	0	0	0	0	0	0	1	0
0	0	0	0	0	0	0	0	0	0	0	0	0	0
0													
OTU12116	0	0	0	0	0	0	1	0	0	0	0	0	0
0	0	0	0	0	0	0	0	0	0	0	0	0	0
0	0	0	0	0	0	0	0	0	0	0	0	0	0
0	0	0	0	0	0	0	0	0	0	0	0	0	0
0	0	0	0	0	0	0	0	0	0	0	0	0	0
0	0	0	0	0	0	0	0	0	0	0	0	0	0
0	0	0	0	0	0	0	0	0	0	0	0	0	0
0	0	0	0	0	0	0	0	0	0	0	0	0	0
0													
OTU2063	0	0	0	0	0	0	1	0	0	0	0	0	0
0	0	0	0	0	0	0	0	8	0	4	0	0	1
0	1	0	0	9	0	3	0	0	0	0	0	0	0
0	0	18	0	0	0	0	0	2	14	2	0	0	0
1	0	0	0	0	1	0	9	10	5	107	2	0	0
12	0	0	0	0	0	0	0	0	1	0	0	0	0
0	0	0	0	0	0	0	0	3	0	0	0	0	0
0	0	0	0	0	0	0	0	0	0	0	0	0	0
0													
OTU301	0	0	0	0	0	0	1	0	0	0	0	0	18
0	0	0	0	0	0	0	0	0	0	0	0	0	0
0	0	0	0	0	0	0	0	0	0	0	0	0	0
0	0	1	7	0	0	3	0	0	0	0	0	0	0
0	0	0	0	0	0	0	0	0	0	0	0	0	0
0	0	0	1	0	0	2	0	0	0	29	0	0	0
3	0	7	0	1	0	0	0	183	0	0	0	0	2
1	0	0	0	0	0	0	0	0	0	0	1	1	0
0													

OTU12108 0 0 0 0 0 0 2 2 0 0 0 0 0
0 2 0 1 0 0 0 0 0 0 0 0 0 0
0 0 0 0 0 0 0 0 0 0 0 0 0 0
0 0 0 0 0 0 0 0 0 0 0 0 0 0
0 0 0 1 0 0 0 0 0 0 0 0 0 0
0 0 0 0 0 0 0 0 0 0 0 0 0 0
1 0 0 0 0 0 0 0 0 0 0 0 0 0
0 0 0 0 0 0 0 0 0 0 0 0 0 0
0
OTU12119 0 0 0 0 0 0 1 0 0 0 0 0 0
0 0 0 0 0 0 0 0 0 0 0 0 0 0
0 0 0 0 0 0 0 0 0 0 0 0 0 0
0 0 0 0 0 0 0 0 0 0 0 0 0 0
0 0 0 0 0 0 0 0 0 0 0 0 0 0
0 0 0 0 0 0 0 0 0 0 0 0 0 0
0 0 0 0 0 0 0 0 0 0 0 0 0 0
0 0 0 0 0 0 0 0 0 0 0 0 0 0
0
OTU15703 0 0 0 0 0 0 1 0 0 0 0 0 0
0 0 0 0 0 0 0 0 0 0 0 0 0 0
0 0 0 0 0 0 0 0 0 0 0 0 0 0
0 0 0 0 0 0 0 0 0 0 0 0 0 0
0 0 0 0 0 0 0 0 0 0 0 0 0 0
0 0 0 0 0 0 0 0 0 0 0 0 0 0
0 0 0 0 0 0 0 0 0 0 0 0 0 0
0 0 0 0 0 0 0 0 0 0 0 0 0 0
0
OTU15704 0 0 0 0 0 0 1 0 0 0 0 0 0
0 0 0 0 0 0 0 0 0 0 0 0 0 0
0 0 0 0 0 0 0 0 0 0 0 0 0 0
0 0 0 0 0 0 0 0 0 0 0 0 0 0
0 0 0 0 0 0 0 0 0 0 0 0 0 0
0 0 0 0 0 0 0 0 0 0 0 0 0 0

0	0	0	0	0	0	0	0	0	0	0	0	0	0
0	0	0	0	0	0	0	0	0	0	0	0	0	0
0													
OTU7819	0	0	0	0	0	0	1	0	0	0	0	0	0
0	0	0	0	0	0	0	0	0	0	0	0	0	0
0	0	0	0	0	0	0	0	0	0	0	0	0	0
0	0	0	0	0	0	0	0	0	0	0	0	0	0
0	0	0	0	0	0	0	0	0	0	0	0	0	0
0	0	0	0	0	0	0	0	0	0	0	0	0	0
0	0	0	0	0	0	0	0	0	0	0	0	0	0
0	0	0	0	0	0	0	0	0	0	0	0	0	0
0													
OTU7005	0	0	0	0	0	0	1	0	1	2	0	0	0
0	0	0	0	0	0	1	0	0	0	0	0	0	0
0	0	0	0	0	0	0	0	0	0	0	0	0	0
2	0	0	0	0	3	0	0	0	0	0	0	0	0
0	1	0	0	0	0	0	0	0	0	0	0	0	0
0	5	0	0	2	0	0	1	0	0	0	0	0	0
0	0	0	0	0	1	0	0	0	0	1	0	2	0
0	3	1	3	3	0	0	4	0	3	0	0	0	5
0													
OTU9865	0	0	0	0	0	0	1	0	0	0	0	0	0
0	0	0	0	0	0	0	0	0	0	0	0	0	0
0	0	0	0	0	0	0	0	0	0	0	0	0	0
0	0	0	1	0	0	0	0	0	0	0	0	0	0
0	0	0	0	0	0	0	0	0	0	0	0	0	0
0	0	0	0	0	0	0	0	0	0	0	0	0	0
0	0	0	1	0	0	0	0	0	0	0	0	0	0
0	0	0	0	0	0	0	0	0	0	0	0	0	0
0													
OTU15705	0	0	0	0	0	0	1	0	0	0	0	0	0
0	0	0	0	0	0	0	0	0	0	0	0	0	0
0	0	0	0	0	1	0	0	0	0	0	0	0	0

0	0	0	0	0	0	0	0	0	0	0	0	0	0
0	0	0	0	0	0	0	0	0	0	0	0	0	0
0	0	0	0	0	0	0	0	0	0	0	0	0	0
0	0	0	0	0	0	0	0	0	0	0	0	0	0
0	0	0	0	0	0	0	0	0	0	0	0	0	0
0													
OTU13319	0	0	0	0	0	0	1	4	0	0	0	0	1
0	1	0	0	0	0	0	0	0	0	0	0	1	0
0	0	0	1	0	0	0	0	0	0	1	1	0	0
0	0	0	1	0	0	0	0	0	0	0	0	0	0
0	0	0	0	0	0	0	0	0	0	0	0	0	0
0	0	2	0	0	0	0	0	0	1	2	0	0	0
0	0	0	0	0	0	0	0	0	1	0	0	0	0
0	0	0	0	0	0	0	0	0	0	1	0	0	0
0													
OTU12118	0	0	0	0	0	0	1	0	0	0	0	0	0
0	0	0	0	0	0	0	0	0	0	0	0	0	0
0	0	0	0	0	0	0	0	0	0	0	0	0	0
0	0	0	0	0	0	0	0	0	0	0	0	0	0
0	0	0	0	0	0	0	0	0	0	0	0	0	0
0	0	0	0	0	0	0	0	0	0	0	0	0	0
0	0	0	0	0	0	0	0	0	0	0	0	0	0
0	0	0	0	0	0	0	0	0	0	0	0	0	0
0													
OTU1384	0	0	0	0	0	0	2	0	0	0	1	0	4
1	0	1	0	1	0	0	0	0	0	0	1	0	0
0	0	0	0	0	0	0	0	0	0	0	0	0	0
0	0	0	0	0	0	0	0	0	0	0	0	0	0
0	0	0	0	0	0	0	0	0	0	0	0	0	0
0	0	1	0	0	0	0	0	1	0	0	0	0	0
0	0	1	0	0	0	0	0	0	1	1	0	0	0
0	1	0	0	0	0	1	0	0	0	1	0	0	0
0													

OTU7818 0 0 0 0 0 0 1 0 0 0 0 0 0
0 0 0 0 0 0 0 0 0 0 0 0 0 0
0 0 0 0 0 0 0 0 0 0 0 0 0 0
0 0 0 0 0 0 0 0 0 0 0 0 0 0
0 0 0 0 0 0 0 0 0 0 0 0 0 0
0 0 0 0 0 0 0 0 0 0 0 0 0 0
0 0 0 0 0 0 0 0 0 0 0 0 0 0
0 0 0 0 0 0 0 0 0 0 0 0 0 0
0
OTU688 0 0 0 0 0 0 2 0 0 0 0 0 0
0 1 0 0 0 0 0 0 0 0 0 0 0 0
0 0 0 0 0 0 0 0 0 0 0 0 0 0
0 0 0 0 0 0 0 0 0 0 0 0 0 0
0 0 0 0 0 0 0 0 0 0 0 0 5 0
0 1 0 0 0 0 0 0 0 3 0 0 0 0
0 0 0 2 0 0 0 0 0 0 0 2 2 0
0 3 3 3 4 0 0 3 0 0 0 0 0 1
4
OTU2186 0 0 0 0 0 0 1 2 0 0 0 0 0
0 0 0 0 0 0 0 0 0 0 0 1 0 0
0 0 0 0 0 0 0 0 1 0 0 0 0 0
0 0 0 0 0 0 0 0 0 0 0 0 0 0
0 0 0 0 0 0 0 0 0 0 0 0 0 0
0 0 2 0 0 0 0 0 0 0 0 0 0 0
0 0 0 0 0 1 0 0 0 0 0 0 0 0
0 0 0 0 0 0 0 0 0 0 0 0 0 0
0
OTU14475 0 0 0 0 0 0 1 0 0 0 0 0 0
0 0 0 0 0 0 0 0 0 0 0 0 0 0
0 0 0 0 0 0 0 0 0 0 0 0 0 0
0 0 0 0 0 0 0 0 0 0 0 0 0 0
0 0 0 0 0 0 0 0 0 0 0 0 0 0
0 0 0 0 0 0 0 0 0 0 0 0 0 0

0	0	0	0	0	0	0	0	0	0	0	0	0	0
0	0	0	0	0	0	0	0	0	0	0	0	0	0
0													
OTU14476	0	0	0	0	0	0	1	0	0	0	0	0	0
0	0	0	0	0	0	0	0	0	0	0	0	0	0
0	0	0	0	0	0	0	0	0	0	0	0	0	0
0	0	0	0	0	0	0	0	0	0	0	0	0	0
0	0	0	0	0	0	0	0	0	0	0	0	0	0
0	0	0	0	0	0	0	0	0	0	0	0	0	0
0	0	0	0	0	0	0	0	0	0	0	0	0	0
0	0	0	0	0	0	0	0	0	0	0	0	0	0
0													
OTU15701	0	0	0	0	0	0	1	0	0	0	0	0	0
0	0	0	0	0	0	0	0	0	0	0	0	0	0
0	0	0	0	0	0	0	0	0	0	0	0	0	0
0	0	0	0	0	0	0	0	0	0	0	0	0	0
0	0	0	0	0	0	0	0	0	0	0	0	0	0
0	0	0	0	0	0	0	0	0	0	0	0	0	0
0	0	0	0	0	0	0	0	0	0	0	0	0	0
0	0	0	0	0	0	0	0	0	0	0	0	0	0
0													
OTU958	0	0	0	0	0	0	1	0	0	0	0	1	0
0	0	0	0	0	0	0	0	1	0	1	0	5	0
4	2	0	0	1	0	0	0	0	0	0	0	5	0
15	0	0	0	0	0	0	0	0	0	1	0	0	0
0	1	2	0	1	0	1	2	0	0	0	1	1	0
0	1	1	0	0	2	0	1	0	0	0	0	1	0
0	3	0	0	0	0	0	0	0	0	0	0	0	0
0	1	2	5	2	0	1	0	1	1	0	0	0	2
2													
OTU6378	0	0	0	0	0	0	1	0	4	0	0	0	0
0	0	0	0	0	0	0	0	0	0	0	0	0	1
1	0	0	0	0	0	2	0	0	0	0	0	1	0

0	0	0	0	1	4	0	4	0	1	2	0	0	1
0	0	4	2	0	0	2	1	0	0	0	1	2	0
0	0	0	0	0	0	0	0	0	0	0	0	0	2
0	3	0	4	0	1	0	0	0	0	0	0	0	0
0	0	0	0	0	0	0	0	0	0	0	0	0	0
0													
OTU1299	0	0	0	0	0	0	2	2	1	1	0	0	0
0	0	0	0	0	0	2	0	0	0	0	0	1	0
1	0	0	0	0	0	0	1	0	0	0	0	0	0
5	0	0	0	0	2	0	1	0	0	0	0	0	0
0	0	0	0	0	0	0	0	0	0	0	0	2	0
0	0	0	0	0	0	0	1	0	4	0	2	0	6
0	0	0	1	0	0	0	1	0	0	0	2	0	0
0	0	1	3	1	1	1	0	0	0	0	0	0	0
0													
OTU372	0	0	0	0	0	0	1	0	0	9	0	0	0
0	0	0	1	0	0	5	0	0	0	0	0	0	0
0	0	0	0	12	0	0	0	0	0	1	0	0	0
2	0	0	0	0	0	0	0	0	0	0	0	0	0
0	1	0	0	0	0	0	0	1	0	0	0	1	0
0	1	5	0	21	0	0	1	0	42	0	0	0	4
0	0	0	16	0	0	0	4	0	0	0	0	1	0
0	0	1	0	0	0	0	0	1	60	0	0	0	0
1													
OTU7142	0	0	0	0	0	0	3	0	0	0	0	0	0
0	0	0	0	0	0	0	0	0	0	0	0	0	0
1	0	0	0	0	0	1	0	0	0	0	0	0	0
1	0	0	0	0	0	0	0	0	0	1	0	0	0
0	0	0	0	0	0	0	0	0	0	0	0	1	0
0	0	0	0	0	0	0	1	0	0	0	0	0	2
0	2	0	1	0	0	0	0	0	0	0	0	0	0
0	0	0	0	0	0	0	0	0	0	0	0	0	0
0													

OTU12115 0 0 0 0 0 0 1 0 0 0 0 0 0
0 0 0 0 0 0 0 0 0 0 0 0 0 0
0 0 0 0 0 0 0 0 0 0 0 0 0 0
0 0 0 0 0 0 0 0 0 0 0 0 0 0
0 0 0 0 0 0 0 0 0 0 0 0 0 0
0 0 0 0 0 0 0 0 0 0 0 0 0 0
0 0 0 0 0 0 0 0 0 0 0 0 0 0
0 0 0 0 0 0 0 0 0 0 0 0 0 0
0
OTU15702 0 0 0 0 0 0 1 0 0 0 0 0 0
0 0 0 0 0 0 0 0 0 0 0 0 0 0
0 0 0 0 0 0 0 0 0 0 0 0 0 0
0 0 0 0 0 0 0 0 0 0 0 0 0 0
0 0 0 0 0 0 0 0 0 0 0 0 0 0
0 0 0 0 0 0 0 0 0 0 0 0 0 0
0 0 0 0 0 0 0 0 0 0 0 0 0 0
0 0 0 0 0 0 0 0 0 0 0 0 0 0
0
OTU12112 0 0 0 0 0 0 1 0 0 0 0 0 0
0 0 0 0 0 0 0 0 0 0 0 0 0 0
0 0 0 0 0 0 0 0 0 0 0 0 0 0
0 0 0 0 0 0 0 0 0 0 0 0 0 0
0 0 0 0 0 0 0 0 0 0 0 0 0 0
0 0 0 0 0 0 0 0 0 0 0 0 0 0
0 0 0 0 0 0 0 0 0 0 0 0 0 0
0 0 0 0 0 0 0 0 0 0 0 0 0 0
0
OTU7827 0 0 0 0 0 0 1 0 0 0 0 0 0
0 0 0 0 0 0 0 0 0 0 0 0 0 0
0 0 0 0 0 0 0 0 0 0 0 0 0 0
0 0 0 0 0 0 0 0 0 0 0 0 0 0
0 0 0 0 0 0 0 0 0 0 0 0 0 0
0 0 0 0 0 0 0 0 0 0 0 0 0 0

0	0	0	0	0	0	0	0	0	0	0	0	0	0
0	0	0	0	0	0	0	0	0	0	0	0	0	0
0													
OTU1238	0	0	0	0	0	0	2	0	4	1	0	1	0
0	0	0	1	0	0	0	0	4	0	0	0	1	0
0	0	0	0	0	0	0	1	2	0	2	0	4	0
4	0	0	0	0	2	0	3	0	0	3	1	0	0
0	0	0	2	0	0	0	0	0	0	0	0	0	0
0	2	0	0	0	0	0	1	0	1	0	0	0	0
0	0	0	0	0	3	0	2	0	1	0	7	10	0
0	0	4	22	6	2	10	6	14	3	0	1	0	6
1													
OTU12113	0	0	0	0	0	0	1	0	0	0	0	0	0
0	0	0	0	0	0	0	0	0	0	0	0	0	0
0	0	0	0	0	0	0	0	0	0	0	0	0	0
0	0	0	0	0	0	0	0	0	0	0	0	0	0
0	0	0	0	0	0	0	0	0	0	0	0	0	0
0	0	0	0	0	0	0	0	0	0	0	0	0	0
0	0	0	0	0	0	0	0	0	0	0	0	0	0
0	0	0	0	0	0	0	0	0	0	0	0	0	0
0													
OTU12127	0	0	0	0	0	0	1	0	0	0	0	0	0
0	0	0	0	0	0	0	0	0	0	0	0	0	0
0	0	0	0	0	0	0	0	0	0	0	0	0	0
0	0	0	0	0	0	0	0	0	0	0	0	0	0
0	0	0	0	0	0	0	0	0	0	0	0	0	0
0	0	0	0	0	0	0	0	0	0	0	0	0	0
0	0	0	0	0	0	0	0	0	0	0	0	0	0
0	0	0	0	0	0	0	0	0	0	0	0	0	0
0													
OTU12128	0	0	0	0	0	0	1	0	0	0	0	0	0
0	0	0	0	0	0	0	0	0	0	0	0	0	0
0	0	0	0	0	0	0	0	0	0	0	0	0	0

0	0	0	0	0	0	0	0	0	0	0	0	0	0
0	0	0	0	0	0	0	0	0	0	0	0	0	0
0	0	0	0	0	0	0	0	0	0	0	0	0	0
0	0	0	0	0	0	0	0	0	0	0	0	0	0
0	0	0	0	0	0	0	0	0	0	0	0	0	0
0													
OTU14458	0	0	0	0	0	0	2	0	0	0	0	0	0
0	0	0	0	0	0	0	0	0	0	0	0	0	0
0	0	0	0	0	0	0	0	0	0	0	0	0	0
0	0	0	0	0	0	0	0	0	0	0	0	0	0
0	0	0	0	0	0	0	0	0	0	0	0	0	0
0	0	0	0	0	0	0	0	0	0	0	0	0	0
0	0	0	0	0	0	0	0	0	0	0	0	0	0
0	0	0	0	0	0	0	0	0	0	0	0	0	0
0													
OTU14457	0	0	0	0	0	0	1	0	0	0	0	0	0
0	0	0	0	0	0	0	0	0	0	0	0	0	0
0	0	0	0	0	0	0	0	0	0	0	0	0	0
0	0	0	0	0	0	0	0	0	0	0	0	0	0
0	0	0	0	0	0	0	0	0	0	0	0	0	0
0	0	0	0	0	0	0	0	0	0	0	0	0	0
0	0	0	0	0	0	0	0	0	0	0	0	0	0
0	0	0	0	0	0	0	0	0	0	0	0	0	0
0													
OTU12129	0	0	0	0	0	0	1	0	0	0	0	0	0
0	0	0	0	0	0	0	0	0	0	0	0	0	0
0	0	0	0	0	0	0	0	0	0	0	0	0	0
0	0	0	0	0	0	0	0	0	0	0	0	0	0
0	0	0	0	0	0	0	0	0	0	0	0	0	0
0	0	0	0	0	0	0	0	0	0	0	0	0	0
0	0	0	0	0	0	0	0	0	0	0	0	0	0
0	0	0	0	0	0	0	0	0	0	0	0	0	0
0													

OTU14459 0 0 0 0 0 0 1 0 0 0 0 0 0
0 0 0 0 0 0 0 0 0 0 0 0 0 0
0 0 0 0 0 0 0 0 0 0 0 0 0 0
0 0 0 0 0 0 0 0 0 0 0 0 0 0
0 0 0 0 0 0 0 0 0 0 0 0 0 0
0 0 0 0 0 0 0 0 0 0 0 0 0 0
0 0 0 0 0 0 0 0 0 0 0 0 0 0
0 0 0 0 0 0 0 0 0 0 0 0 0 0
0
OTU12126 0 0 0 0 0 0 1 0 0 0 0 0 0
0 0 0 0 0 0 0 0 0 0 0 0 0 0
0 0 0 0 0 0 0 0 0 0 0 0 0 0
0 0 0 0 0 0 0 0 0 0 0 0 0 0
0 0 0 0 0 0 0 0 0 0 0 0 0 0
0 0 0 0 0 0 0 0 0 0 0 0 0 0
0 0 0 0 0 0 0 0 0 0 0 0 0 0
0 0 0 0 0 0 0 0 0 0 0 0 0 0
0
OTU12125 0 0 0 0 0 0 2 0 0 0 0 0 0
0 0 0 0 0 0 0 0 0 0 0 0 0 0
0 0 0 0 0 0 0 0 0 0 0 0 0 0
0 0 0 0 0 0 0 0 0 0 0 0 0 0
0 0 0 0 0 0 0 0 0 0 0 0 0 0
0 0 0 0 0 0 0 0 0 0 0 0 0 0
0 0 0 0 0 0 0 0 0 0 0 0 0 0
0 0 0 0 0 0 0 0 0 0 0 0 0 0
0
OTU1140 0 0 0 0 0 0 1 0 0 0 0 0 0
0 1 0 0 0 0 0 0 0 0 0 1 0 0
0 0 0 0 0 0 1 0 0 0 0 0 0 0
0 0 0 0 0 0 0 0 0 0 0 0 0 0
0 2 0 0 0 0 0 0 0 0 0 0 0 0
0 0 0 0 0 0 0 0 0 0 0 0 1 0

0 0 0 0 0 0 0 0 0 0 0 0 0 0
0 0 0 0 0 0 0 0 0 0 0 0 0 0
0
OTU1455 0 0 0 0 0 0 1 0 0 0 0 0 0
0 0 0 1 0 0 1 0 0 0 0 0 1 0
0 0 0 0 1 0 0 0 0 0 0 0 0 0
0 0 0 0 0 0 0 0 0 1 0 0 0 0
0 0 0 0 0 0 0 0 0 0 0 0 0 0
0 0 0 0 2 0 0 0 0 0 0 0 0 0
0 0 0 0 0 0 0 0 0 0 0 0 0 0
0 0 0 0 0 0 0 0 1 0 0 0 0 0
0
OTU7820 0 0 0 0 0 0 1 0 0 0 0 0 0
0 0 0 0 0 0 0 0 0 0 0 0 0 0
0 0 0 0 0 0 0 0 0 0 0 0 0 0
0 0 0 0 0 0 0 0 0 0 0 0 0 0
0 0 0 0 0 0 0 0 0 0 0 0 0 0
0 0 0 0 0 0 0 0 0 0 0 0 0 0
0 0 0 0 0 0 0 0 0 0 0 0 0 0
0 0 0 0 0 0 0 0 0 0 0 0 0 0
0
OTU7823 0 0 0 0 0 0 1 0 0 0 0 0 0
0 0 0 0 0 0 0 0 0 0 0 0 0 0
0 0 0 0 0 0 0 0 0 0 0 0 0 0
0 0 0 0 0 0 0 0 0 0 0 0 0 0
0 0 0 0 0 0 0 0 0 0 0 0 0 0
0 0 0 0 0 0 0 0 0 0 0 0 0 0
0 0 0 0 0 0 0 0 0 0 0 0 0 0
0 0 0 0 0 0 0 0 0 0 0 0 0 0
0
OTU14460 0 0 0 0 0 0 1 0 0 0 0 0 0
0 0 0 0 0 0 0 0 0 0 0 0 0 0
0 0 0 0 0 0 0 0 0 0 0 0 0 0

0 0 0 0 0 0 0 0 0 0 0 0 0 0
0 0 0 0 0 0 0 0 0 0 0 0 0 0
0 0 0 0 0 0 0 0 0 0 0 0 0 0
0 0 0 0 0 0 0 0 0 0 0 0 0 0
0 0 0 0 0 0 0 0 0 0 0 0 0 0
0
OTU15710 0 0 0 0 0 0 2 0 0 0 0 0 0
0 0 0 0 0 0 0 0 0 0 0 0 0 0
0 0 0 0 0 0 0 0 0 0 0 0 0 0
0 0 0 0 0 0 0 0 0 0 0 0 0 0
0 0 0 0 0 0 0 0 0 0 0 0 0 0
0 0 0 0 0 0 0 0 0 0 0 0 0 0
0 0 0 0 0 0 0 0 0 0 0 0 0 0
0 0 0 0 0 0 0 0 0 0 0 0 0 0
0
OTU7824 0 0 0 0 0 0 1 0 0 0 0 0 0
0 0 0 0 0 0 0 0 0 0 0 0 0 0
0 0 0 0 0 0 0 0 0 0 0 0 0 0
0 0 0 0 0 0 0 0 0 0 0 0 0 0
0 0 0 0 0 0 0 0 0 0 0 0 0 0
0 0 0 0 0 0 0 0 0 0 0 0 0 0
0 0 0 0 0 0 0 0 0 0 0 0 0 0
0 0 0 0 0 0 0 0 0 0 0 0 0 0
0
OTU7825 0 0 0 0 0 0 1 0 0 0 0 0 0
0 0 0 0 0 0 0 0 0 0 0 0 0 0
0 0 0 0 0 0 0 0 0 0 0 0 0 0
0 0 0 0 0 0 0 0 0 0 0 0 0 0
0 0 0 0 0 0 0 0 0 0 0 0 0 0
0 0 0 0 0 0 0 0 0 0 0 0 0 0
0 0 0 0 0 0 0 0 0 0 0 0 0 0
0 0 0 0 0 0 0 0 0 0 0 0 0 0
0

OTU7822	0	0	0	0	0	0	1	1	0	0	0	0	0
0	0	0	0	0	0	0	0	0	0	0	0	0	0
0	0	0	0	0	0	0	0	0	0	0	0	0	0
0	0	0	0	0	0	0	0	0	0	0	0	0	0
0	0	0	0	0	0	0	0	0	0	0	0	0	0
0	0	0	0	0	0	0	0	0	0	0	0	0	0
0	0	0	0	0	0	0	0	0	0	0	0	0	0
0	0	0	0	0	0	0	0	0	0	0	0	0	0
0													
OTU3072	0	0	0	0	0	0	2	0	1	0	1	0	0
0	0	1	0	1	0	0	0	0	0	0	0	0	0
0	0	1	0	0	0	0	0	0	0	0	0	0	0
1	0	1	0	0	0	0	0	1	2	0	0	0	0
0	0	0	0	0	0	0	1	0	0	0	0	0	1
0	0	1	0	1	0	0	1	0	0	0	1	0	0
1	0	0	0	0	1	0	0	0	0	1	0	0	0
0	1	0	0	0	0	0	0	0	0	0	0	1	0
2													
OTU10621	0	0	0	0	0	0	1	0	8	0	0	0	0
0	1	0	1	0	0	3	0	0	0	1	0	3	0
0	0	0	0	0	0	0	0	0	0	1	0	1	0
7	0	0	1	0	0	0	2	0	0	0	0	0	0
0	1	0	7	0	0	0	0	0	0	0	0	2	0
0	0	1	0	1	0	0	0	0	2	0	2	0	2
0	0	0	1	0	0	0	0	0	0	0	0	1	0
0	0	0	1	0	0	0	1	0	2	0	0	0	0
0													
OTU15712	0	0	0	0	0	0	1	0	0	0	0	0	0
0	0	0	0	0	0	0	0	0	0	0	0	0	0
0	0	0	0	0	0	0	0	0	0	0	0	0	0
0	0	0	0	0	0	0	0	0	0	0	0	0	0
0	0	0	0	0	0	0	0	0	0	0	0	0	0
0	0	0	0	0	0	0	0	0	0	0	0	0	0

0	0	0	0	0	0	0	0	0	0	0	0	0	0
0	0	0	0	0	0	0	0	0	0	0	0	0	0
0													
OTU335	0	0	0	0	0	0	2	0	0	0	0	0	0
0	0	0	0	0	0	0	0	0	0	0	0	0	0
0	0	0	0	0	0	0	0	0	0	0	0	0	0
0	0	0	0	0	0	0	0	0	0	0	0	1	0
0	0	0	0	0	0	0	0	0	0	0	0	0	0
0	5	0	0	9	0	0	3	0	160	0	0	0	3
0	0	0	2	0	0	0	2	0	0	0	0	2	0
0	0	0	0	0	0	0	1	0	0	0	0	0	0
0													
OTU15711	0	0	0	0	0	0	1	0	0	0	0	0	0
0	0	0	0	0	0	0	0	0	0	0	0	0	0
0	0	0	0	0	0	0	0	0	0	0	0	0	0
0	0	0	0	0	0	0	0	0	0	0	0	0	0
0	0	0	0	0	0	0	0	0	0	0	0	0	0
0	0	0	0	0	0	0	0	0	0	0	0	0	0
0	0	0	0	0	0	0	0	0	0	0	0	0	0
0	0	0	0	0	0	0	0	0	1	0	0	0	0
0													
OTU14453	0	0	0	0	0	0	1	0	0	0	0	0	0
0	0	0	0	0	0	0	0	0	0	0	0	0	0
0	0	0	0	0	0	0	0	0	0	0	0	0	0
0	0	0	0	0	0	0	0	0	0	0	0	0	0
0	0	0	0	0	0	0	0	0	0	0	0	0	0
0	0	0	0	0	0	0	0	0	0	0	0	0	0
0	0	0	0	0	0	0	0	0	0	0	0	0	0
0	0	0	0	0	0	0	0	0	0	0	0	0	0
0													
OTU14454	0	0	0	0	0	0	1	0	0	0	0	0	0
0	0	0	0	0	0	0	0	0	0	0	0	0	0
0	0	0	0	0	0	0	0	0	0	0	0	0	0

0 0 0 0 0 0 0 0 0 0 0 0 0 0
0 0 0 0 0 0 0 0 0 0 0 0 0 0
0 0 0 0 0 0 0 0 0 0 0 0 0 0
0 0 0 0 0 0 0 0 0 0 0 0 0 0
0 0 0 0 0 0 0 0 0 0 0 0 0 0
0
OTU6478 0 0 0 0 0 0 1 0 0 0 0 0 0
0 0 0 0 0 0 0 0 0 0 0 0 0 0
0 0 0 0 0 0 0 0 0 0 0 1 0 0
0 0 0 0 0 0 0 0 0 0 0 0 0 0
0 0 1 0 0 0 0 0 0 0 0 0 0 0
0 0 1 0 0 0 1 0 0 0 0 0 0 0
0 0 0 0 0 0 0 0 1 0 0 0 0 0
0 0 0 0 0 0 0 0 0 0 0 0 0 0
0
OTU12121 0 0 0 0 0 0 1 0 0 0 0 0 0
0 0 0 0 0 0 0 0 0 0 0 0 0 0
0 0 0 0 0 0 0 0 0 0 0 0 0 0
0 0 0 0 0 0 0 0 0 0 0 0 0 0
0 0 0 0 0 0 0 0 0 0 0 0 0 0
0 0 0 0 0 0 0 0 0 0 0 0 0 0
0 0 0 0 0 0 0 0 0 0 0 0 0 0
0 0 0 0 0 0 0 0 0 0 0 0 0 0
0
OTU9639 0 0 0 0 0 0 1 0 0 0 0 0 0
0 0 0 0 0 0 0 0 0 0 0 0 0 0
0 0 0 0 0 0 0 0 0 0 0 0 0 0
0 0 0 0 0 0 0 0 0 0 0 0 0 0
0 0 0 0 0 0 0 0 0 0 0 0 0 0
0 0 0 0 0 0 0 0 0 1 0 0 0 0
0 0 0 0 0 0 0 0 0 0 0 0 0 0
0 0 0 0 0 0 0 0 0 0 0 0 0 0
0

OTU14455	0	0	0	0	0	0	1	0	0	0	0	0	0
0	0	0	0	0	0	0	0	0	0	0	0	0	0
0	0	0	0	0	0	0	0	0	0	0	0	0	0
0	0	0	0	0	0	0	0	0	0	0	0	0	0
0	0	0	0	0	0	0	0	0	0	0	0	0	0
0	0	0	0	0	0	0	0	0	0	0	0	0	0
0	0	0	0	0	0	0	0	0	0	0	0	0	0
0	0	0	0	0	0	0	0	0	0	0	0	0	0
0													
OTU14456	0	0	0	0	0	0	1	0	0	0	0	0	0
0	0	0	0	0	0	0	0	0	0	0	0	0	0
0	0	0	0	0	0	0	0	0	0	0	0	0	0
0	0	0	0	0	0	0	0	0	0	0	0	0	0
0	0	0	0	0	0	0	0	0	0	0	0	0	0
0	0	0	0	0	0	0	0	0	0	0	0	0	0
0	0	0	0	0	0	0	0	0	0	0	0	0	0
0	0	0	0	0	0	0	0	0	0	0	0	0	0
0													
OTU7754	0	0	0	0	0	0	2	0	0	1	0	1	0
0	0	0	0	0	0	2	0	0	0	0	0	0	0
1	0	0	0	0	0	0	0	0	0	0	0	1	0
2	0	0	0	0	0	0	0	0	0	0	0	0	0
0	1	0	0	0	0	0	1	0	0	0	0	0	0
0	2	0	0	0	0	0	0	0	0	0	1	0	0
0	0	0	1	0	0	0	0	0	0	0	1	0	0
0	2	0	0	2	0	0	1	1	1	0	0	0	4
0													
OTU12122	0	0	0	0	0	0	1	0	0	0	0	0	0
0	0	0	0	0	0	0	0	0	0	0	0	0	0
0	0	0	0	0	0	0	0	0	0	0	0	0	0
0	0	0	0	0	0	0	0	0	0	0	0	0	0
0	0	0	0	0	0	0	0	0	0	0	0	0	0
0	0	0	0	0	0	0	0	0	0	0	0	0	0

0	0	0	0	0	0	0	0	0	0	0	0	0	0
0	0	0	0	0	0	0	0	0	0	0	0	0	0
0													
OTU3862	0	0	0	0	0	0	1	0	0	0	0	0	4
8	0	0	0	0	2	0	0	0	0	0	0	0	0
0	0	1	1	1	0	0	3	0	0	0	0	0	2
0	0	1	0	2	0	0	0	0	0	0	0	2	0
3	0	4	0	0	1	0	1	0	0	0	0	0	0
0	0	0	0	0	0	1	0	0	0	1	0	0	0
0	0	1	0	0	0	0	0	6	0	1	0	0	4
2	0	0	0	0	0	0	0	0	0	0	0	3	0
0													
OTU12124	0	0	0	0	0	0	1	0	0	0	0	0	0
0	0	0	0	0	0	0	0	0	0	0	0	0	0
0	0	0	0	0	0	0	0	0	0	0	0	0	0
0	0	0	0	0	0	0	0	0	0	0	0	0	0
0	0	0	0	0	0	0	0	0	0	0	0	0	0
0	0	0	0	0	0	0	0	0	0	0	0	0	0
0	0	0	0	0	0	0	0	0	0	0	0	0	0
0	0	0	0	0	0	0	0	0	0	0	0	0	0
0													
OTU14465	0	0	0	0	0	0	1	0	0	0	0	0	0
0	0	0	0	0	0	0	0	0	0	0	0	0	0
0	0	0	0	0	0	0	0	0	0	0	0	0	0
0	0	0	0	0	0	0	0	0	0	0	0	0	0
0	0	0	0	0	0	0	0	0	0	0	0	0	0
0	0	0	0	0	0	0	0	0	0	0	0	0	0
0	0	0	0	0	0	0	0	0	0	0	0	0	0
0	0	0	0	0	0	0	0	0	0	0	0	0	0
0													
OTU15709	0	0	0	0	0	0	1	0	0	0	0	0	0
0	0	0	0	0	0	0	0	0	0	0	0	0	0
0	0	0	0	0	0	0	0	0	0	0	0	0	0

0	0	0	0	0	0	0	0	0	0	0	0	0	0
0	0	0	0	0	0	0	0	0	0	0	0	0	0
0	0	0	0	0	0	0	0	0	0	0	0	0	0
0	0	0	0	0	0	0	0	0	0	0	0	0	0
0	0	0	0	0	0	0	0	0	0	0	0	0	0
0													
OTU14467	0	0	0	0	0	0	1	0	0	0	0	0	0
0	0	0	0	0	0	0	0	0	0	0	0	0	0
0	0	0	0	0	0	0	0	0	0	0	0	0	0
0	0	0	0	0	0	0	0	0	0	0	0	0	0
0	0	0	0	0	0	0	0	0	0	0	0	0	0
0	0	0	0	0	0	0	0	0	0	0	0	0	0
0	0	0	0	0	0	0	0	0	0	0	0	0	0
0	0	0	0	0	0	0	0	0	0	0	0	0	0
0													
OTU485	0	0	0	0	0	0	1	1	1	0	0	0	0
0	0	0	0	0	0	0	0	4	0	2	0	13	0
12	4	0	0	0	0	1	0	0	0	5	0	0	0
0	0	0	0	0	0	0	1	1	30	9	11	0	0
0	0	0	0	1	6	0	12	5	0	3	0	1	0
6	0	0	0	0	0	0	34	0	1	2	15	0	1
0	99	0	0	0	0	0	2	0	0	0	0	3	0
0	3	8	11	32	70	32	1	16	5	0	1	0	17
23													
OTU14466	0	0	0	0	0	0	1	0	0	0	0	0	0
0	0	0	0	0	0	0	0	0	0	0	0	0	0
0	0	0	0	0	0	0	0	0	0	0	0	0	0
0	0	0	0	0	0	0	0	0	0	0	0	0	0
0	0	0	0	0	0	0	0	0	0	0	0	0	0
0	0	0	0	0	0	0	0	0	0	0	0	0	0
0	0	0	0	0	0	0	0	0	0	0	0	0	0
0	0	0	0	0	0	0	0	0	0	0	0	0	0
0													

OTU6636	0	0	0	0	0	0	1	0	14	1	0	0	0
0	0	0	0	0	0	0	0	0	0	0	0	0	0
0	0	0	0	0	0	0	0	0	0	0	0	4	0
9	0	0	0	0	0	0	0	0	0	0	0	0	0
0	0	0	0	0	0	0	0	0	0	0	0	0	0
0	0	0	0	0	0	0	0	0	0	0	0	0	0
0	0	0	3	0	0	0	6	0	0	0	0	0	0
0	0	0	0	0	0	0	0	0	9	0	0	0	0
0													
OTU5377	0	0	0	0	0	0	1	0	0	0	0	2	2
3	3	0	0	1	0	0	0	0	0	0	0	1	0
0	0	0	0	1	0	0	5	1	0	2	0	0	0
0	0	0	0	0	0	3	0	0	0	0	0	0	0
0	0	1	0	0	0	0	0	0	3	0	0	0	0
0	0	0	2	0	0	0	0	1	1	0	0	0	0
15	0	1	0	2	0	0	1	0	1	1	0	0	1
0	0	0	0	0	0	0	0	0	0	0	3	0	0
0													
OTU12123	0	0	0	0	0	0	1	0	0	0	0	0	0
0	0	0	0	0	0	0	0	0	0	0	0	0	0
0	0	0	0	0	0	0	0	0	0	0	0	0	0
0	0	0	0	0	0	0	0	0	0	0	0	0	0
0	0	0	0	0	0	0	0	0	0	0	0	0	0
0	0	0	0	0	0	0	0	0	0	0	0	0	0
0	0	0	0	0	0	0	0	0	0	0	0	0	0
0	0	0	0	0	0	0	0	0	0	0	0	0	0
0													
OTU14464	0	0	0	0	0	0	1	0	0	0	0	0	0
0	0	0	0	0	0	0	0	0	0	0	0	0	0
0	0	0	0	0	0	0	0	0	0	0	0	0	0
0	0	0	0	0	0	0	0	0	0	0	0	0	0
0	0	0	0	0	0	0	0	0	0	0	0	0	0
0	0	0	0	0	0	0	0	0	0	0	0	0	0

0	0	0	0	0	0	0	0	0	0	0	0	0	0
0	0	0	0	0	0	0	0	0	0	0	0	0	0
0													
OTU15706	0	0	0	0	0	0	1	0	0	0	0	0	0
0	0	0	0	0	0	0	0	0	0	0	0	0	0
0	0	0	0	0	0	0	0	0	0	0	0	0	0
0	0	0	0	0	0	0	0	0	0	0	0	0	0
0	0	0	0	0	0	0	0	0	0	0	0	0	0
0	0	0	0	0	0	0	0	0	0	0	0	0	0
0	0	0	0	0	0	0	0	0	0	0	0	0	0
0	0	0	0	0	0	0	0	0	0	0	0	0	0
0													
OTU15707	0	0	0	0	0	0	1	0	0	0	0	0	0
0	0	0	0	0	0	0	0	0	0	0	0	0	0
0	0	0	0	0	0	0	0	0	0	0	0	0	0
0	0	0	0	0	0	0	0	0	0	0	0	0	0
0	0	0	0	0	0	0	0	0	0	0	0	0	0
0	0	0	0	0	0	0	0	0	0	0	0	0	0
0	0	0	0	0	0	0	0	0	0	0	0	0	0
0	0	0	0	0	0	0	0	0	0	0	0	0	0
0													
OTU14468	0	0	0	0	0	0	1	0	0	0	0	0	0
0	0	0	0	0	0	0	0	0	0	0	0	0	0
0	0	0	0	0	0	0	0	0	0	0	0	0	0
0	0	0	0	0	0	0	0	0	0	0	0	0	0
0	0	0	0	0	0	0	0	0	0	0	0	0	0
0	0	0	0	0	0	0	0	0	0	0	0	0	0
0	0	0	0	0	0	0	0	0	0	0	0	0	0
0	0	0	0	0	0	0	0	0	0	0	0	0	0
0													
OTU15236	0	0	0	0	0	0	1	0	1	0	0	0	0
0	0	0	0	0	0	0	0	0	0	0	0	3	0
0	0	0	0	0	0	0	0	0	0	0	0	0	0

0	0	0	0	0	0	0	0	0	0	0	0	0	0
0	0	0	0	0	0	0	0	0	0	0	0	0	0
0	0	0	0	0	0	0	0	0	0	0	0	0	0
0	0	0	0	0	0	0	0	0	0	0	0	0	0
0	0	0	0	0	0	0	0	0	0	0	0	0	0
0													
OTU12149	0	0	0	0	0	0	1	0	0	0	0	0	0
0	0	0	0	0	0	0	0	0	0	0	0	0	0
0	0	0	0	0	0	0	0	0	0	0	0	0	0
0	0	0	0	0	0	0	0	0	0	0	0	0	0
0	0	0	0	0	0	0	0	0	0	0	0	0	0
0	0	0	0	0	0	0	0	0	0	0	0	0	0
0	0	0	0	0	0	0	0	0	0	0	0	0	0
0	0	0	0	0	0	0	0	0	0	0	0	0	0
0													
OTU14469	0	0	0	0	0	0	1	0	0	0	0	0	0
0	0	0	0	0	0	0	0	0	0	0	0	0	0
0	0	0	0	0	0	0	0	0	0	0	0	0	0
0	0	0	0	0	0	0	0	0	0	0	0	0	0
0	0	0	0	0	0	0	0	0	0	0	0	0	0
0	0	0	0	0	0	0	0	0	0	0	0	0	0
0	0	0	0	0	0	0	0	0	0	0	0	0	0
0	0	0	0	0	0	0	0	0	0	0	0	0	0
0													
OTU15696	0	0	0	0	0	0	1	0	0	0	0	0	0
0	0	0	0	0	0	0	0	0	0	0	0	0	0
0	0	0	0	0	0	0	0	0	0	0	0	0	0
0	0	0	0	0	0	0	0	0	0	0	0	0	0
0	0	0	0	0	0	0	0	0	0	0	0	0	0
0	0	0	0	0	0	0	0	0	0	0	0	0	0
0	0	0	0	0	0	0	0	0	0	0	0	0	0
0	0	0	0	0	0	0	0	0	0	0	0	0	0
0													

OTU1379 0 0 0 0 0 0 1 0 0 0 0 0 0
0 0 0 0 0 0 0 0 0 0 0 0 0 0
0 0 0 0 0 0 0 0 0 0 0 0 0 0
0 0 0 0 0 0 0 0 0 0 0 0 0 0
0 0 0 0 0 0 0 0 0 0 0 0 0 0
0 0 0 0 0 0 0 0 0 0 0 0 0 0
0 0 0 0 0 1 0 0 0 0 0 0 1 0
0 1 0 4 0 2 5 2 1 8 0 0 0 10
1
OTU7817 0 0 0 0 0 0 1 0 0 0 0 0 0
0 0 0 0 0 0 0 0 0 0 0 0 0 0
0 0 0 0 0 0 0 0 0 0 0 0 0 0
0 0 0 0 0 0 0 0 0 0 0 0 0 0
0 0 0 0 0 0 0 0 0 0 0 0 0 0
0 0 0 0 0 0 0 0 0 0 0 0 0 0
0 0 0 0 0 0 0 0 0 0 0 0 0 0
0 0 0 0 0 0 0 0 0 0 0 0 0 0
0
OTU15698 0 0 0 0 0 0 1 0 0 0 0 0 0
0 0 0 0 0 0 0 0 0 0 0 0 0 0
0 0 0 0 0 0 0 0 0 0 0 0 0 0
0 0 0 0 0 0 0 0 0 0 0 0 0 0
0 0 0 0 0 0 0 0 0 0 0 0 0 0
0 0 0 0 0 0 0 0 0 0 0 0 0 0
0 0 0 0 0 0 0 0 0 0 0 0 0 0
0 0 0 0 0 0 0 0 0 0 0 0 0 0
0
OTU6228 0 0 0 0 0 0 1 0 0 0 0 0 0
1 1 0 0 0 0 0 0 0 0 0 0 0 0
0 0 0 0 0 0 0 0 0 0 0 0 0 1
0 0 0 0 0 0 0 0 1 0 0 0 0 0
0 0 0 0 0 1 0 0 0 0 0 0 0 0
0 0 0 0 0 0 0 0 0 0 0 0 0 0

0	0	0	0	0	0	0	0	0	0	0	1	0	0
0	0	0	0	0	0	0	0	0	0	0	0	0	0
1													
OTU12150	0	0	0	0	0	0	1	0	0	0	0	0	0
0	0	0	0	0	0	0	0	0	0	0	0	0	0
0	0	0	0	0	0	0	0	0	0	0	0	0	0
0	0	0	0	0	0	0	0	0	0	0	0	0	0
0	0	0	0	0	0	0	0	0	0	0	0	0	0
0	0	0	0	0	0	0	0	0	0	0	0	0	0
0	0	0	0	0	0	0	0	0	0	0	0	0	0
0	0	0	0	0	0	0	0	0	0	0	0	0	0
0													
OTU7815	0	0	0	0	0	0	1	0	0	0	0	0	0
0	0	0	0	0	0	0	0	0	0	0	0	0	0
0	0	0	0	0	0	0	0	0	0	0	0	0	0
0	0	0	0	0	0	0	0	0	0	0	0	0	0
0	0	0	0	0	0	0	0	0	0	0	0	0	0
0	0	0	0	0	0	0	0	0	0	0	0	0	0
0	0	0	0	0	0	0	0	0	0	0	0	0	0
0	0	0	0	0	0	0	0	0	0	0	0	0	0
0													
OTU12148	0	0	0	0	0	0	1	0	0	0	0	0	0
0	0	0	0	0	0	0	0	0	0	0	0	0	0
0	0	0	0	0	0	0	0	0	0	0	0	0	0
0	0	0	0	0	0	0	0	0	0	0	0	0	0
0	0	0	0	0	0	0	0	0	0	0	0	0	0
0	0	0	0	0	0	0	0	0	0	0	0	0	0
0	0	0	0	0	0	0	0	0	0	0	0	0	0
0	0	0	0	0	0	0	0	0	0	0	0	0	0
0													
OTU15693	0	0	0	0	0	0	1	0	0	0	0	0	0
0	0	0	0	0	0	0	0	0	0	0	0	0	0
0	0	0	0	0	0	0	0	0	0	0	0	0	0

0 0 0 0 0 0 0 0 0 0 0 0 0 0
0 0 0 0 0 0 0 0 0 0 0 0 0 0
0 0 0 0 0 0 0 0 0 0 0 0 0 0
0 0 0 0 0 0 0 0 0 0 0 0 0 0
0 0 0 0 0 0 0 0 0 0 0 0 0 0
0
OTU14461 0 0 0 0 0 0 1 0 0 0 0 0 0
0 0 0 0 0 0 0 0 0 0 0 0 0 0
0 0 0 0 0 0 0 0 0 0 0 0 0 0
0 0 0 0 0 0 0 0 0 0 0 0 0 0
0 0 0 0 0 0 0 0 0 0 0 0 0 0
0 0 0 0 0 0 0 0 0 0 0 0 0 0
0 0 0 0 0 0 0 0 0 0 0 0 0 0
0 0 0 0 0 0 0 0 0 0 0 0 0 0
0
OTU7816 0 0 0 0 0 0 1 0 0 0 0 0 0
0 0 0 0 0 0 0 0 0 0 0 0 0 0
0 0 0 0 0 0 0 0 0 0 0 0 0 0
0 0 0 0 0 0 0 0 0 0 0 0 0 0
0 0 0 0 0 0 0 0 0 0 0 0 0 0
0 0 0 0 0 0 0 0 0 0 0 0 0 0
0 0 0 0 0 0 0 0 0 0 0 0 0 0
0 0 0 0 0 0 0 0 0 0 0 0 0 0
0
OTU15695 0 0 0 0 0 0 1 0 0 0 0 0 0
0 0 0 0 0 0 0 0 0 0 0 0 0 0
0 0 0 0 0 0 0 0 0 0 0 0 0 0
0 0 0 0 0 0 0 0 0 0 0 0 0 0
0 0 0 0 0 0 0 0 0 0 0 0 0 0
0 0 0 0 0 0 0 0 0 0 0 0 0 0
0 0 0 0 0 0 0 0 0 0 0 0 0 0
0 0 0 0 0 0 0 0 0 0 0 0 0 0
0

OTU14463 0 0 0 0 0 0 2 0 0 0 0 0 0
0 0 0 0 0 0 0 0 0 0 0 0 0 0
0 0 0 0 0 0 0 0 0 0 0 0 0 0
0 0 0 0 0 0 0 0 0 0 0 0 0 0
0 0 0 0 0 0 0 0 0 0 0 0 0 0
0 0 0 0 0 0 0 0 0 0 0 0 0 0
0 0 0 0 0 0 0 0 0 0 0 0 0 0
0 0 0 0 0 0 0 0 0 0 0 0 0 0
0
OTU11924 0 0 0 0 0 0 1 0 0 0 0 0 0
0 0 0 0 0 0 0 1 0 3 1 0 0 0
0 0 0 0 0 0 0 0 0 0 0 1 0 0
0 0 0 0 0 0 0 0 0 0 0 0 0 0
0 0 0 0 0 0 0 0 0 0 0 0 0 0
0 0 0 0 0 0 1 0 0 0 0 0 0 0
0 0 0 0 0 0 0 0 0 0 0 0 0 0
0 0 0 0 0 0 0 0 0 0 0 0 0 0
0
OTU15694 0 0 0 0 0 0 1 0 0 0 0 0 0
0 0 0 0 0 0 0 0 0 0 0 0 0 0
0 0 0 0 0 0 0 0 0 0 0 0 0 0
0 0 0 0 0 0 0 0 0 0 0 0 0 0
0 0 0 0 0 0 0 0 0 0 0 0 0 0
0 0 0 0 0 0 0 0 0 0 0 0 0 0
0 0 0 0 0 0 0 0 0 0 0 0 0 0
0 0 0 0 0 0 0 0 0 0 0 0 0 0
0
OTU13824 0 0 0 0 0 0 1 0 0 0 0 0 0
0 0 0 0 0 0 0 0 0 0 0 0 0 0
0 0 0 0 0 0 0 0 0 0 0 0 0 1
0 0 0 0 0 0 0 0 0 0 1 0 0 0
0 0 0 0 0 0 0 0 0 0 0 0 0 0
1 0 0 0 0 0 1 0 1 0 1 0 0 0

0 0 0 1 0 0 0 0 0 0 0 0 0 0
0 0 0 0 0 0 0 0 0 0 0 0 0 0
0
OTU7761 0 0 0 0 0 0 1 0 0 0 0 0 0
0 0 0 0 0 0 0 0 0 0 0 0 0 0
0 0 0 0 0 0 0 0 0 0 0 0 0 0
0 0 0 0 0 0 0 0 0 0 0 0 0 0
0 0 0 0 0 0 0 0 0 0 0 0 0 0
0 0 0 0 0 0 0 0 0 0 0 0 0 0
0 0 0 0 0 0 0 0 0 0 0 0 0 0
0 0 0 0 0 0 0 0 0 0 0 0 0 0
0
OTU3224 0 0 0 0 0 0 1 0 0 0 0 0 0
0 0 1 0 1 0 0 0 0 0 0 0 0 0
0 0 0 0 0 2 0 0 0 0 0 0 0 0
0 0 0 0 0 0 0 0 0 0 0 0 0 0
0 0 0 0 0 0 0 0 0 0 0 0 0 1
0 0 0 0 0 1 0 0 0 0 1 0 0 0
0 0 0 0 2 0 0 0 0 0 0 0 0 1
0 0 0 0 0 0 0 0 0 0 0 0 0 0
0
OTU14399 0 0 0 0 0 0 1 0 0 0 0 0 0
0 0 0 0 0 0 0 0 0 0 0 0 0 0
0 0 0 0 0 0 0 0 0 0 0 0 0 0
0 0 0 0 0 0 0 0 0 0 0 0 0 0
0 0 1 0 0 0 0 0 0 0 0 0 0 0
0 0 0 0 0 0 0 0 0 0 0 0 0 0
0 0 0 0 0 0 0 0 0 0 0 0 0 0
0 0 0 0 0 0 0 0 0 0 0 0 0 0
0
OTU10397 0 0 0 0 0 0 1 0 0 0 0 0 0
0 0 0 0 0 0 0 0 0 0 0 0 0 0
0 0 0 0 0 0 0 0 0 0 0 0 0 0

0	0	0	0	0	0	0	0	0	0	0	0	0	0
0	0	0	0	0	0	0	0	0	0	0	0	0	0
0	0	0	0	0	1	0	0	0	0	0	0	0	0
0	0	0	0	0	0	0	0	0	0	0	0	0	0
0	0	0	0	0	0	0	0	0	0	0	0	0	0
0													
OTU12157	0	0	0	0	0	0	1	0	0	0	0	0	0
0	0	0	0	0	0	0	0	0	0	0	0	0	0
0	0	0	0	0	0	0	0	0	0	0	0	0	0
0	0	0	0	0	0	0	0	0	0	0	0	0	0
0	0	0	0	0	0	0	0	0	0	0	0	0	0
0	0	0	0	0	0	0	0	0	0	0	0	0	0
0	0	0	0	0	0	0	0	0	0	0	0	0	0
0	0	0	0	0	0	0	0	0	0	0	0	0	0
0													
OTU12156	0	0	0	0	0	0	1	0	0	0	0	0	0
0	0	0	0	0	0	0	0	0	0	0	0	0	0
0	0	0	0	0	0	0	0	0	0	0	0	0	0
0	0	0	0	0	0	0	0	0	0	0	0	0	0
0	0	0	0	0	0	0	0	0	0	0	0	0	0
0	0	0	0	0	0	0	0	0	0	0	0	0	0
0	0	0	0	0	0	0	0	0	0	0	0	0	0
0	0	0	0	0	0	0	0	0	0	0	0	0	0
0													
OTU4903	0	0	0	0	0	0	1	0	0	0	0	0	0
0	0	0	0	0	0	0	0	0	0	0	0	0	0
0	0	0	0	0	0	0	0	0	0	0	0	0	0
0	0	0	0	0	0	0	0	0	0	1	0	0	0
0	0	0	3	0	0	0	0	0	0	0	0	0	0
0	0	0	0	0	0	0	0	0	0	0	0	0	0
0	0	0	0	0	0	0	0	0	0	0	0	0	0
0	0	0	0	0	0	0	0	0	0	0	0	0	0
0													

OTU12155	0	0	0	0	0	0	1	0	0	0	0	0	0
0	0	0	0	0	0	0	0	0	0	0	0	0	0
0	0	0	0	0	0	0	0	0	0	0	0	0	0
0	0	0	0	0	0	0	0	0	0	0	0	0	0
0	0	0	0	0	0	0	0	0	0	0	0	0	0
0	0	0	0	0	0	0	0	0	0	0	0	0	0
0	0	0	0	0	0	0	0	0	0	0	0	0	0
0	0	0	0	0	0	0	0	0	0	0	0	0	0
0													
OTU14400	0	0	0	0	0	0	1	0	0	0	0	0	0
0	0	0	0	0	0	0	0	0	0	0	0	0	0
0	0	0	0	0	0	0	0	0	0	0	0	0	0
0	0	0	0	0	0	0	0	0	0	0	0	0	0
0	0	0	0	0	0	0	0	0	0	0	0	0	0
0	0	0	0	0	0	0	0	0	0	0	0	0	0
0	0	0	0	0	0	0	0	0	0	0	0	0	0
0	0	0	0	0	0	0	0	0	0	0	0	0	0
0													
OTU13739	0	0	0	0	0	0	1	0	0	1	0	0	0
0	0	0	0	0	0	1	1	0	0	0	0	0	0
0	0	0	0	0	0	0	0	0	0	0	0	0	0
0	0	0	0	0	0	0	0	0	0	0	0	0	0
0	0	0	1	0	0	0	0	0	0	0	0	0	0
0	0	0	0	0	0	0	0	0	0	0	0	0	0
0	0	0	2	1	0	0	1	0	1	0	0	0	0
0	0	0	0	0	0	0	0	0	0	0	0	0	0
0													
OTU15700	0	0	0	0	0	0	1	0	0	0	0	0	0
0	0	0	0	0	0	0	0	0	0	0	0	0	0
0	0	0	0	0	0	0	0	0	0	0	0	0	0
0	0	0	0	0	0	0	0	0	0	0	0	0	0
0	0	0	0	0	0	0	0	0	0	0	0	0	0
0	0	0	0	0	0	0	0	0	0	0	0	0	0

0	0	0	0	0	0	0	0	0	0	0	0	0	0
0	0	0	0	0	0	0	0	0	0	0	0	0	0
0													
OTU12158	0	0	0	0	0	0	1	0	0	0	0	0	0
0	0	0	0	0	0	0	0	0	0	0	0	0	0
0	0	0	0	0	0	0	0	0	0	0	0	0	0
0	0	0	0	0	0	0	0	0	0	0	0	0	0
0	0	0	0	0	0	0	0	0	0	0	0	0	0
0	0	0	0	0	0	0	0	0	0	0	0	0	0
0	0	0	0	0	0	0	0	0	0	0	0	0	0
0	0	0	0	0	0	0	0	0	0	0	0	0	0
0													
OTU7765	0	0	0	0	0	0	1	0	0	0	0	0	0
0	0	0	0	0	0	0	0	0	0	0	0	0	0
0	0	0	0	0	0	0	0	0	0	0	0	0	0
0	0	0	0	0	0	0	0	0	0	0	0	0	0
0	0	0	0	0	0	0	0	0	0	0	0	0	0
0	0	0	0	0	0	0	0	0	0	0	0	0	0
0	0	0	0	0	0	0	0	0	0	0	0	0	0
0	0	0	0	0	0	0	0	0	0	0	0	0	0
0													
OTU1916	0	0	0	0	0	0	1	0	0	0	0	0	0
0	0	0	0	0	0	0	0	0	0	0	0	0	0
0	0	0	0	0	0	0	0	0	0	0	0	0	0
0	0	0	0	0	0	0	0	0	0	0	0	0	0
0	1	0	0	0	0	0	0	0	0	0	0	1	0
0	0	0	0	1	0	0	0	0	0	0	0	0	0
0	0	0	0	1	0	0	0	0	0	0	0	1	0
0	0	0	0	0	0	0	0	1	0	0	0	0	0
0													
OTU14398	0	0	0	0	0	0	1	0	0	0	0	0	0
0	0	0	0	0	0	0	0	0	0	0	0	0	0
0	0	0	0	0	0	0	0	0	0	0	0	0	0

0	0	0	0	0	0	0	0	0	0	0	0	0	0
0	0	0	0	0	0	0	0	0	0	0	0	0	0
0	0	0	0	0	0	0	0	0	0	0	0	0	0
0	0	0	0	0	0	0	0	0	0	0	0	0	0
0	0	0	0	0	0	0	0	0	0	0	0	0	0
0													
OTU12151	0	0	0	0	0	0	1	0	0	0	0	0	0
0	0	0	0	0	0	0	0	0	0	0	0	0	0
0	0	0	0	0	0	0	0	0	0	0	0	0	0
0	0	0	0	0	0	0	0	0	0	0	0	0	0
0	0	0	0	0	0	0	0	0	0	0	0	0	0
0	0	0	0	0	0	0	0	0	0	0	0	0	0
0	0	0	0	0	0	0	0	0	0	0	0	0	0
0	0	0	0	0	0	0	0	0	0	0	0	0	0
0													
OTU12152	0	0	0	0	0	0	1	0	0	0	0	0	0
0	0	0	0	0	0	0	0	0	0	0	0	0	0
0	0	0	0	0	0	0	0	0	0	0	0	0	0
0	0	0	0	0	0	0	0	0	0	0	0	0	0
0	0	0	0	0	0	0	0	0	0	0	0	0	0
0	0	0	0	0	0	0	0	0	0	0	0	0	0
0	0	0	0	0	0	0	0	0	0	0	0	0	0
0	0	0	0	0	0	0	0	0	0	0	0	0	0
0													
OTU988	0	0	0	0	0	0	2	0	0	0	0	0	0
0	0	0	0	0	0	0	0	0	0	0	0	0	0
0	0	0	0	0	0	0	0	0	0	0	0	0	0
0	0	0	0	0	0	0	0	0	0	0	0	0	0
0	0	0	0	0	0	0	0	0	0	0	0	0	0
0	0	0	0	0	0	0	0	0	0	0	0	0	0
0	0	0	0	0	0	0	0	0	0	0	0	0	0
0	0	0	0	0	0	0	0	0	0	0	0	0	0
0													

OTU15699	0	0	0	0	0	0	1	0	0	0	0	0	0
0	0	0	0	0	0	0	0	0	0	0	0	0	0
0	0	0	0	0	0	0	0	0	0	0	0	0	0
0	0	0	0	0	0	0	0	0	0	0	0	0	0
0	0	0	0	0	0	0	0	0	0	0	0	0	0
0	0	0	0	0	0	0	0	0	0	0	0	0	0
0	0	0	0	0	0	0	0	0	0	0	0	0	0
0	0	0	0	0	0	0	0	0	0	0	0	0	0
0													
OTU12153	0	0	0	0	0	0	1	0	0	0	0	0	0
0	0	0	0	0	0	0	0	0	0	0	0	0	0
0	0	0	0	0	0	0	0	0	0	0	0	0	0
0	0	0	0	0	0	0	0	0	0	0	0	0	0
0	0	0	0	0	0	0	0	0	0	0	0	0	0
0	0	0	0	0	0	0	0	0	0	0	0	0	0
0	0	0	0	0	0	0	0	0	0	0	0	0	0
0	0	0	0	0	0	0	0	0	0	0	0	0	0
0													
OTU12154	0	0	0	0	0	0	1	0	0	0	0	0	0
0	0	0	0	0	0	0	0	0	0	0	0	0	0
0	0	0	0	0	0	0	0	0	0	0	0	0	0
0	0	0	0	0	0	0	0	0	0	0	0	0	0
0	0	0	0	0	0	0	0	0	0	0	0	0	0
0	0	0	0	0	0	0	0	0	0	0	0	0	0
0	0	0	0	0	0	0	0	0	0	0	0	0	0
0	0	0	0	0	0	0	0	0	0	0	0	0	0
0													
OTU123	0	0	0	0	0	0	2	0	0	0	3	0	13
3	0	9	0	0	0	0	1	1	0	0	0	0	0
0	3	1	27	3	1	1	0	0	1	0	0	0	3
5	6	0	0	2	0	3	0	0	0	0	0	5	0
0	0	0	0	0	0	6	0	0	0	0	1	0	7
1	1	0	0	1	1	32	0	1	0	0	1	8	1

2	0	4	0	11	0	4	2	741	0	0	2	0	30
0	6	0	0	4	1	21	8	0	2	1	0	29	0
0													
OTU14403	0	0	0	0	0	0	1	0	0	0	0	0	0
0	0	0	0	0	0	0	0	0	0	0	0	0	0
0	0	0	0	0	0	0	0	0	0	0	0	0	0
0	0	0	0	0	0	0	0	0	0	0	0	0	0
0	0	0	0	0	0	0	0	0	0	0	0	0	0
0	0	0	0	0	0	0	0	0	0	0	0	0	0
0	0	0	0	0	0	0	0	0	0	0	0	0	0
0	0	0	0	0	0	0	0	0	0	0	0	0	0
0													
OTU12142	0	0	0	0	0	0	1	0	0	0	0	0	0
0	0	0	0	0	0	0	0	0	1	0	0	0	0
0	0	0	0	0	0	0	0	0	0	0	0	0	0
0	0	0	0	0	0	0	0	0	0	0	0	0	0
0	0	0	0	0	0	0	0	0	0	0	0	0	0
0	0	1	0	0	0	0	0	0	0	0	0	0	0
0	0	0	0	0	0	0	0	0	0	0	0	0	0
0	0	0	0	0	0	0	0	0	0	0	0	0	0
0													
OTU14402	0	0	0	0	0	0	1	0	0	0	0	0	0
0	0	0	0	0	0	0	0	0	0	0	0	0	0
0	0	0	0	0	0	0	0	0	0	0	0	0	0
0	0	0	0	0	0	0	0	0	0	0	0	0	0
0	0	0	0	0	0	0	0	0	0	0	0	0	0
0	0	0	0	0	0	0	0	0	0	0	0	0	0
0	0	0	0	0	0	0	0	0	0	0	0	0	0
0	0	0	0	0	0	0	0	0	0	0	0	0	0
0													
OTU14404	0	0	0	0	0	0	1	0	0	0	0	0	0
0	0	0	0	0	0	0	0	0	0	0	0	0	0
0	0	0	0	0	0	0	0	0	0	0	0	0	0

0	0	0	0	1	0	0	0	0	0	0	0	0	0
0	0	0	0	0	0	0	0	0	0	0	0	0	0
0	0	0	0	0	0	0	0	0	0	0	0	0	0
0	0	0	0	0	0	0	0	0	0	0	0	0	0
0	0	0	0	0	0	0	0	0	0	0	0	0	0
0													
OTU7763	0	0	0	0	0	0	1	0	0	0	0	0	0
0	0	0	0	0	0	0	0	0	0	0	0	0	0
0	0	0	0	0	0	0	0	0	0	0	0	0	0
0	0	0	0	0	0	0	0	0	0	0	0	0	0
0	0	0	0	0	0	0	0	0	0	0	0	0	0
0	0	0	0	0	0	0	0	0	0	0	0	0	0
0	0	0	0	0	0	0	0	0	0	0	0	0	0
0	0	0	0	0	0	0	0	0	0	0	0	0	0
0													
OTU14405	0	0	0	0	0	0	1	0	0	0	0	0	0
0	0	0	0	0	0	0	0	0	0	0	0	0	0
0	0	0	0	0	0	0	0	0	0	0	0	0	0
0	0	0	0	0	0	0	0	0	0	0	0	0	0
0	0	0	0	0	0	0	0	0	0	0	0	0	0
0	0	0	0	0	0	0	0	0	0	0	0	0	0
0	0	0	0	0	0	0	0	0	0	0	0	0	0
0	0	0	0	0	0	0	0	0	0	0	0	0	0
0													
OTU4483	0	0	0	0	0	0	1	0	0	1	0	1	0
0	0	0	0	0	0	5	0	0	0	0	0	0	0
0	0	0	0	0	0	0	0	0	0	0	0	0	0
0	0	0	0	0	0	0	0	0	0	0	0	0	0
0	0	0	1	0	0	0	0	0	0	0	0	1	0
0	0	0	0	0	1	0	0	0	0	0	1	0	1
0	0	0	0	0	0	0	0	0	0	0	2	3	0
0	1	0	0	0	0	0	2	0	2	1	0	0	0
0													

OTU7753 0 0 0 0 0 0 1 0 0 0 0 0 0
0 0 0 0 0 0 0 0 0 0 0 0 0 0
0 0 0 0 0 0 0 0 0 0 0 0 1 0
0 0 0 0 0 0 0 0 0 0 0 0 0 0
0 0 0 0 0 0 0 0 0 0 0 0 0 0
0 0 0 0 0 0 0 0 0 0 0 0 0 0
0 1 0 0 0 0 0 0 0 0 0 0 0 0
0 0 0 0 0 0 0 0 0 0 0 0 0 0
0
OTU13903 0 0 0 0 0 0 1 0 0 0 0 0 0
0 0 0 0 0 0 0 0 0 0 0 0 0 0
0 0 0 0 0 0 0 0 0 0 0 0 0 0
0 0 0 0 0 0 0 0 0 0 0 0 0 0
0 0 0 0 0 0 0 0 0 0 0 0 0 0
0 9 1 0 0 0 0 0 0 0 0 0 0 0
0 0 0 0 0 0 0 0 0 0 0 5 17 0
0 6 0 0 0 1 0 0 0 3 0 0 0 0
0
OTU12143 0 0 0 0 0 0 1 0 0 0 0 0 0
0 0 0 0 0 0 0 0 0 0 0 0 0 0
0 0 0 0 0 0 0 0 0 0 0 0 0 0
0 0 0 0 0 0 0 0 0 0 0 0 0 0
0 0 0 0 0 0 0 0 0 0 0 0 0 0
0 0 0 0 0 0 0 0 0 0 0 0 0 0
0 0 0 0 0 0 0 0 0 0 0 0 0 0
0 0 0 0 0 0 0 0 0 0 0 0 0 0
0
OTU7755 0 0 0 0 0 0 1 0 0 0 0 0 0
0 0 0 0 0 0 0 0 0 0 0 0 0 0
0 0 0 0 0 0 0 0 0 0 0 0 0 0
0 0 0 0 0 0 0 0 0 0 0 0 0 0
0 0 0 0 0 0 0 0 0 0 0 0 0 0
0 0 0 0 0 0 0 0 0 0 0 0 0 0

0	0	0	0	0	0	0	0	0	0	0	0	0	0
0	0	0	0	0	0	0	0	0	0	0	0	0	0
0													
OTU12432	0	0	0	0	0	0	1	0	0	2	0	1	0
1	0	0	0	0	0	0	1	0	0	0	0	0	0
0	0	0	0	0	0	0	0	0	1	0	0	0	0
0	0	0	0	0	0	0	0	0	0	0	0	0	3
0	0	0	0	0	0	0	0	0	0	0	0	0	0
0	0	0	0	0	0	0	0	1	0	0	0	2	0
1	0	5	0	0	0	0	0	2	0	3	0	0	4
3	0	0	0	0	0	0	0	0	0	1	1	1	0
0													
OTU12141	0	0	0	0	0	0	1	0	0	0	0	0	0
0	0	0	0	0	0	0	0	0	0	0	0	0	0
0	0	0	0	0	0	0	0	0	0	0	0	0	0
0	0	0	0	0	0	0	0	0	0	0	0	0	0
0	0	0	0	0	0	0	0	0	0	0	0	0	0
0	0	0	0	0	0	0	0	0	0	0	0	0	0
0	0	0	0	0	0	0	0	0	0	0	0	0	0
0	0	0	0	0	0	0	0	0	0	0	0	0	0
0													
OTU7756	0	0	0	0	0	0	1	0	0	0	0	0	0
0	0	0	0	0	0	0	0	0	0	0	0	0	0
0	0	0	0	0	0	0	0	0	0	0	0	0	0
0	0	1	0	0	0	0	0	0	0	0	0	0	0
0	0	0	0	0	0	0	0	0	0	0	0	0	0
0	0	0	0	0	0	0	0	0	0	0	0	0	0
0	0	0	0	0	0	0	0	0	0	0	0	0	0
0	0	0	0	0	0	0	0	0	0	0	0	0	0
0													
OTU7751	0	0	0	0	0	0	1	0	0	0	0	0	0
0	0	0	0	0	0	0	0	0	0	0	0	0	0
0	0	0	0	0	0	0	0	0	0	0	0	0	0

0	0	0	0	0	0	0	0	0	0	0	0	0	0
0	0	0	0	0	0	0	0	0	0	0	0	0	0
0	0	0	0	0	0	0	0	0	0	0	0	0	0
0	0	0	0	0	0	0	0	0	0	0	0	0	0
0	0	0	0	0	0	0	0	0	0	0	0	0	0
0													
OTU14401	0	0	0	0	0	0	1	0	0	0	0	0	0
0	0	0	0	0	0	0	0	0	0	0	0	0	0
0	0	0	0	0	0	0	0	0	0	0	0	0	0
0	0	0	0	0	0	0	0	0	0	0	0	0	0
0	0	0	0	0	0	0	0	0	0	0	0	0	0
0	0	0	0	0	0	0	0	0	0	0	0	0	0
0	0	0	0	0	0	0	0	0	0	0	0	0	0
0	0	0	0	0	0	0	0	0	0	0	0	0	0
0													
OTU6788	0	0	0	0	0	0	1	0	0	0	0	0	0
0	0	0	0	0	0	1	0	0	0	0	0	0	0
0	0	0	0	0	0	0	0	0	0	0	0	0	0
0	0	0	0	0	0	0	0	0	0	0	0	0	0
0	0	0	0	0	0	0	0	0	0	0	0	0	0
0	0	0	0	0	0	0	0	0	0	0	0	0	0
0	0	0	2	0	0	0	0	0	0	0	0	0	0
0	0	0	0	0	0	0	0	0	0	0	0	0	0
0													
OTU2922	0	0	0	0	0	0	1	2	11	0	0	0	0
0	0	0	0	0	0	0	0	0	0	0	0	0	0
0	0	0	0	0	0	0	0	0	1	0	0	0	0
0	0	0	0	0	0	0	0	0	0	0	0	0	0
0	0	0	0	0	0	0	0	0	0	0	0	0	0
0	0	0	0	2	0	0	1	0	3	0	0	0	0
0	0	0	4	0	0	0	6	0	0	0	1	4	0
0	2	1	3	2	5	2	2	0	2	0	0	0	3
0													

OTU12140 0 0 0 0 0 0 1 0 0 0 0 0 0
0 0 0 0 0 0 0 0 0 0 0 0 0 0
0 0 0 0 0 0 0 0 0 0 0 0 0 0
0 0 0 0 0 0 0 0 0 0 0 0 0 0
0 0 0 0 0 0 0 0 0 0 0 0 0 0
0 0 0 0 0 0 0 0 0 0 0 0 0 0
0 0 0 0 0 0 0 0 0 0 0 0 0 0
0 0 0 0 0 0 0 0 0 0 0 0 0 0
0
OTU774 0 0 0 0 0 0 1 0 0 1 0 0 0
0 0 0 0 0 0 0 0 0 0 0 0 0 0
0 0 0 0 0 0 0 0 0 0 1 0 0 0
0 0 0 0 0 0 0 0 0 0 0 0 0 0
0 0 0 0 0 0 0 0 0 0 0 0 0 0
0 4 0 0 0 0 0 0 0 1 0 0 0 0
0 0 0 1 0 0 0 0 0 0 0 3 4 0
0 0 0 2 0 0 0 2 2 0 0 0 0 1
5
OTU15719 0 0 0 0 0 0 1 0 0 0 0 0 0
0 0 0 0 0 0 0 0 0 0 0 0 0 0
0 0 0 0 0 0 0 0 0 0 0 0 0 0
0 0 0 0 0 0 0 0 0 0 0 0 0 0
0 0 0 0 0 0 0 0 0 0 0 0 0 0
0 0 0 0 0 0 0 0 0 0 0 0 0 0
0 0 0 0 0 0 0 0 0 0 0 0 0 0
0 0 0 0 0 0 0 0 0 0 0 0 0 0
0
OTU15720 0 0 0 0 0 0 1 0 0 0 0 0 0
0 0 0 0 0 0 0 0 0 0 0 0 0 0
0 0 0 0 0 0 0 0 0 0 0 0 0 0
0 0 0 0 0 0 0 0 0 0 0 0 0 0
0 0 0 0 0 0 0 0 0 0 0 0 0 0
0 0 0 0 0 0 0 0 0 0 0 0 0 0

0	0	0	0	0	0	0	0	0	0	0	0	0	0
0	0	0	0	0	0	0	0	0	0	0	0	0	0
0													
OTU14389	0	0	0	0	0	0	1	0	0	0	0	0	0
0	0	0	0	0	0	0	0	0	0	0	0	0	0
0	0	0	0	0	0	0	0	0	0	0	0	0	0
0	0	0	0	0	0	0	0	0	0	0	0	0	0
0	0	0	0	0	0	0	0	0	0	0	0	0	0
0	0	0	0	0	0	0	0	0	0	0	0	0	0
0	0	0	0	0	0	0	0	0	0	0	0	0	0
0	0	0	0	0	0	0	0	0	0	0	0	0	0
0													
OTU14511	0	0	0	0	0	0	1	0	0	1	0	0	0
0	1	0	0	0	0	0	0	0	0	0	0	0	0
0	0	0	0	0	0	0	0	0	0	0	0	0	0
0	0	0	0	0	0	0	0	0	0	0	0	0	0
0	0	0	0	0	0	0	0	0	0	0	0	0	0
0	0	0	0	0	0	0	0	0	0	0	0	0	0
0	0	0	1	0	0	0	0	0	0	0	0	0	0
0	0	0	0	0	0	0	0	0	0	2	0	0	0
0													
OTU12436	0	0	0	0	0	0	1	0	0	2	0	0	0
0	0	0	0	0	0	0	0	0	0	0	0	0	0
0	0	0	0	0	0	0	0	0	0	0	0	0	0
0	0	0	0	0	0	0	0	0	0	0	0	0	0
0	0	0	0	0	0	0	0	0	0	0	0	0	0
0	0	0	0	0	0	0	0	0	0	0	0	0	0
0	0	0	0	0	0	0	0	0	0	0	0	0	0
0	0	0	0	0	0	0	0	0	0	0	0	0	0
0													
OTU7759	0	0	0	0	0	0	1	0	0	2	0	0	0
0	0	0	0	0	0	0	0	0	0	0	0	0	0
0	0	0	0	0	0	0	0	0	0	0	0	0	0

0	0	0	0	0	0	0	0	0	0	0	0	0	0
0	0	0	0	0	0	0	0	0	0	0	0	0	0
0	0	0	0	0	0	0	0	0	0	0	0	0	0
0	0	0	6	0	1	0	0	0	0	0	0	0	0
0	0	0	0	0	0	0	0	0	0	0	0	0	0
0													
OTU11086	0	0	0	0	0	0	1	0	0	0	0	0	0
0	0	0	0	0	0	0	0	0	0	0	0	0	0
0	0	0	0	0	0	0	0	0	0	0	0	0	0
0	0	0	0	0	0	0	0	0	0	0	0	0	1
0	0	1	0	0	0	0	0	0	0	0	0	0	0
0	0	0	0	0	0	1	0	0	0	0	0	0	0
0	0	0	0	0	0	0	0	0	0	0	0	0	3
0	0	0	0	0	0	0	0	0	0	0	0	0	0
0													
OTU14388	0	0	0	0	0	0	1	0	0	0	0	0	0
0	0	0	0	0	0	0	0	0	0	0	0	0	0
0	0	0	0	0	0	0	0	0	0	0	0	0	0
0	0	0	0	0	0	0	0	0	0	0	0	0	0
0	0	0	0	0	0	0	0	0	0	0	0	0	0
0	0	0	0	0	0	0	0	0	0	0	0	0	0
0	0	0	0	0	0	0	0	0	0	0	0	0	0
0	0	0	0	0	0	0	0	0	0	0	0	0	0
0													
OTU606	0	0	0	0	0	0	1	0	0	0	0	0	0
0	0	0	0	0	0	0	0	0	0	0	8	0	0
1	0	3	0	0	0	0	0	0	0	0	0	0	7
0	0	0	0	0	0	0	0	0	0	0	0	0	0
0	0	0	0	0	0	0	0	0	0	0	0	0	0
0	0	0	0	0	0	0	0	0	0	0	0	0	0
0	0	0	0	0	0	0	0	0	0	0	0	0	0
0	0	2	1	1	0	0	0	0	0	0	0	0	0
0													

OTU15725	0	0	0	0	0	0	1	0	0	0	0	0	0
0	0	0	0	0	0	0	0	0	0	0	0	0	0
0	0	0	0	0	0	0	0	0	0	0	0	0	0
0	0	0	0	0	0	0	0	0	0	0	0	0	0
0	0	0	0	0	0	0	0	0	0	0	0	0	0
0	0	0	0	0	0	0	0	0	0	0	0	0	0
0	0	0	0	0	0	0	0	0	0	0	0	0	0
0	0	0	0	0	0	0	0	0	0	0	0	0	0
0													
OTU7760	0	0	0	0	0	0	1	0	0	0	0	0	0
0	0	0	0	0	0	0	0	0	0	0	0	0	0
0	0	0	0	0	0	0	0	0	0	0	0	0	0
0	0	0	0	0	0	0	0	0	0	0	0	0	0
0	0	0	0	0	0	0	0	0	0	0	0	0	0
0	0	0	0	0	0	0	0	0	0	0	0	0	0
0	0	0	0	0	0	0	0	0	0	0	0	0	0
0	0	0	0	0	0	0	0	0	0	0	0	0	0
0													
OTU12146	0	0	0	0	0	0	1	0	0	0	0	0	0
0	0	0	0	0	0	0	0	0	0	0	0	0	0
0	0	0	0	0	0	0	0	0	0	0	0	0	0
0	0	0	0	0	0	0	0	0	0	0	0	0	0
0	0	0	0	0	0	0	0	0	0	0	0	0	0
0	0	0	0	0	0	0	0	0	0	0	0	0	0
0	0	0	0	0	0	0	0	0	0	0	0	0	0
0	0	0	0	0	0	0	0	0	0	0	0	0	0
0													
OTU15726	0	0	0	0	0	0	1	0	0	0	0	0	0
0	0	0	0	0	0	0	0	0	0	0	0	0	0
0	0	0	0	0	0	0	0	0	0	0	0	0	0
0	0	0	0	0	0	0	0	0	0	0	0	0	0
0	0	0	0	0	0	0	0	0	0	0	0	0	0
0	0	0	0	0	0	0	0	0	0	0	0	0	0

0	0	0	0	0	0	0	0	0	0	0	0	0	0
0	0	0	0	0	0	0	0	0	0	0	0	0	0
0													
OTU411	0	0	0	0	0	0	1	0	0	1	0	0	0
0	0	0	0	0	0	0	0	0	0	0	0	0	0
0	0	0	0	0	0	0	0	0	0	0	0	0	0
0	0	0	0	0	0	0	0	0	0	0	0	0	0
0	0	0	0	0	0	0	0	0	0	0	0	0	0
0	0	0	0	0	0	0	0	0	0	0	0	0	0
0	0	0	0	0	0	0	6	0	0	0	0	0	0
0	0	0	0	0	0	13	0	3	19	0	0	0	17
0													
OTU502	0	0	0	0	0	0	2	0	0	0	9	0	0
0	0	0	0	0	3	0	0	0	0	0	0	0	0
0	0	0	0	0	0	0	0	0	0	0	0	0	2
0	0	0	0	0	0	1	0	0	0	0	0	0	0
0	0	0	0	0	0	0	0	0	0	0	1	0	0
0	0	0	1	0	0	16	0	18	0	0	0	0	0
0	0	32	0	12	0	0	0	4	0	0	0	0	0
0	0	0	0	0	0	0	0	0	0	0	1	0	0
0													
OTU3255	0	0	0	0	0	0	1	0	0	0	0	0	0
0	0	0	0	0	0	0	0	0	0	0	0	1	0
0	0	0	0	0	0	0	0	0	1	0	0	0	0
0	0	0	0	0	0	0	0	0	0	0	0	0	0
0	0	0	0	0	0	0	0	0	0	0	0	1	0
0	0	1	0	0	0	0	0	0	1	0	0	0	0
0	0	0	1	0	0	0	0	0	0	0	0	0	0
0	0	0	0	0	0	0	0	0	0	0	0	0	0
0													
OTU7757	0	0	0	0	0	0	1	0	0	0	0	0	0
0	0	0	0	0	0	0	0	0	0	0	0	0	0
0	0	0	0	0	0	0	0	0	0	0	0	0	0

0 0 0 0 0 0 0 0 0 0 0 0 0 0

0 0 0 0 0 0 0 0 0 0 0 0 0 0

0 0 0 0 0 0 0 0 0 0 0 0 0 0

0 0 0 0 0 0 0 0 0 0 0 0 0 0

0 0 0 0 0 0 0 0 0 0 0 0 0 0

0

OTU2633 0 0 0 0 0 0 1 0 0 0 0 0 0

0 0 0 0 0 0 0 0 0 0 0 0 0 0

0 0 0 0 0 0 0 0 0 0 0 0 0 0

0 0 0 0 0 0 0 0 0 0 0 0 0 0

0 0 0 0 0 0 0 0 0 0 0 0 0 0

0 0 0 0 0 0 0 0 0 0 0 0 0 0

0 0 0 0 0 0 0 1 0 0 0 0 0 0

0 0 0 0 0 0 0 0 0 0 0 0 0 0

0

OTU12147 0 0 0 0 0 0 1 0 0 0 0 0 0

0 0 0 0 0 0 0 0 0 0 0 0 0 0

0 0 0 0 0 0 0 0 0 0 0 0 0 0

0 0 0 0 0 0 0 0 0 0 0 0 0 0

0 0 0 0 0 0 0 0 0 0 0 0 0 0

0 0 0 0 0 0 0 0 0 0 0 0 0 0

0 0 0 0 0 0 0 0 0 0 0 0 0 0

0 0 0 0 0 0 0 0 0 0 0 0 0 0

0

OTU12145 0 0 0 0 0 0 1 0 0 0 0 0 0

0 0 0 0 0 0 0 0 0 0 0 0 0 0

0 0 0 0 0 0 0 0 0 0 0 0 0 0

0 0 0 0 0 0 0 0 0 0 0 0 0 0

0 0 0 0 0 0 0 0 0 0 0 0 0 0

0 0 0 0 0 0 0 0 0 0 0 0 0 0

0 0 0 0 0 0 0 0 0 0 0 0 0 0

0 0 0 0 0 0 0 0 0 0 0 0 0 0

0

OTU6419	0	0	0	0	0	0	1	1	0	0	1	0	0
1	0	0	0	0	1	0	1	0	1	0	1	0	0
0	0	0	0	0	0	0	0	0	0	1	2	0	1
0	0	1	0	0	0	0	0	1	0	0	0	0	0
0	1	0	0	0	0	0	0	0	1	2	1	0	0
3	0	1	0	0	0	0	1	0	0	0	3	1	0
0	0	1	1	0	0	0	1	0	0	0	0	0	0
1	2	1	0	1	0	0	0	0	0	2	0	0	0
1													
OTU14385	0	0	0	0	0	0	1	0	0	0	0	0	0
0	0	0	0	0	0	0	0	0	0	0	0	0	0
0	0	0	0	0	0	0	0	0	0	0	0	0	0
0	0	0	0	0	0	0	0	0	0	0	0	0	0
0	0	0	0	0	0	0	0	0	0	0	0	0	0
0	0	0	0	0	0	0	0	0	0	0	0	0	0
0	0	0	0	0	0	0	0	0	0	0	0	0	0
0	0	0	0	0	0	0	0	0	0	0	0	0	0
0													
OTU12144	0	0	0	0	0	0	1	0	0	0	0	0	0
0	0	0	0	0	0	0	0	0	0	0	0	0	0
0	0	0	0	0	0	0	0	0	0	0	0	0	0
0	0	0	0	0	0	0	0	0	0	0	0	0	0
0	0	0	0	0	0	0	0	0	0	0	0	0	0
0	0	0	0	0	0	0	0	0	0	0	0	0	0
0	0	0	0	0	0	0	0	0	0	0	0	0	0
0	0	0	0	0	0	0	0	0	0	0	0	0	0
0													
OTU922	0	0	0	0	0	0	1	0	0	0	2	0	0
1	0	0	0	0	0	0	0	0	0	0	0	0	0
0	0	0	0	0	0	0	0	0	0	0	0	0	0
0	0	0	0	0	0	0	0	0	0	0	0	0	0
0	0	0	0	0	0	0	0	0	0	0	0	0	0
0	0	0	0	0	0	0	0	0	0	0	0	0	0

0	0	0	0	0	0	0	0	0	0	0	0	0	0
0	0	0	0	0	0	0	0	0	0	0	0	0	0
0													
OTU858	0	0	0	0	0	0	1	0	11	0	0	0	0
0	1	0	0	0	0	0	0	0	0	0	0	0	0
0	0	0	0	0	0	0	0	0	0	0	0	0	0
0	0	0	0	0	0	0	0	0	0	0	0	0	0
0	0	0	0	0	0	0	0	0	0	0	0	0	0
0	0	2	0	0	0	0	0	0	0	0	0	0	0
0	0	0	0	0	0	0	0	0	0	0	0	1	0
0	3	0	1	0	0	0	0	0	0	0	0	0	0
0													
OTU15722	0	0	0	0	0	0	1	0	0	0	0	0	0
0	0	0	0	0	0	0	0	0	0	0	0	0	0
0	0	0	0	0	0	0	0	0	0	0	0	0	0
0	0	0	0	0	0	0	0	0	0	0	0	0	0
0	0	0	0	0	0	0	0	0	0	0	0	0	0
0	0	0	0	0	0	0	0	0	0	0	0	0	0
0	0	0	0	0	0	0	0	0	0	0	0	0	0
0	0	0	0	0	0	0	0	0	0	0	0	0	0
0													
OTU11167	0	0	0	0	0	0	1	0	0	1	0	0	0
0	0	0	0	0	0	0	0	0	0	0	0	0	0
0	0	0	0	0	0	0	0	0	0	0	0	0	0
0	0	0	0	0	0	0	0	0	0	0	0	0	0
0	0	0	0	0	0	0	0	0	0	0	0	0	0
0	0	0	0	0	0	0	0	0	0	0	0	0	0
0	0	0	1	0	0	0	3	0	0	0	0	0	0
0	0	0	0	1	0	0	0	0	0	0	0	0	0
0													
OTU14387	0	0	0	0	0	0	1	0	1	0	0	0	0
0	0	0	0	0	1	0	0	0	0	0	0	0	0
1	1	0	0	0	0	0	0	0	0	0	0	0	0

0	0	0	0	0	0	0	0	0	1	0	0	0	0
0	0	0	0	0	0	0	0	0	0	0	0	0	0
0	0	1	0	1	0	0	0	0	0	0	1	0	0
2	0	0	0	0	1	0	0	0	0	0	0	0	0
0	1	0	0	0	0	0	0	0	0	2	0	0	0
0													
OTU14386	0	0	0	0	0	0	1	0	0	0	0	0	0
0	0	0	0	0	0	0	0	0	0	0	0	0	0
0	0	0	0	0	0	0	0	0	0	0	0	0	0
0	0	0	0	0	0	0	0	0	0	0	0	0	0
0	0	0	0	0	0	0	0	0	0	0	0	0	0
0	0	0	0	0	0	0	0	0	0	0	0	0	0
0	0	0	0	0	0	0	0	0	0	0	0	0	0
0	0	0	0	0	0	0	0	0	0	0	0	0	0
0													
OTU2560	0	0	0	0	0	0	1	0	0	0	0	0	0
0	0	0	0	0	0	0	0	0	0	0	0	0	0
0	0	0	1	0	0	0	0	0	0	0	0	0	0
0	0	0	0	1	0	0	1	0	0	0	0	0	0
0	0	0	0	0	0	0	0	0	0	0	0	1	0
0	0	0	0	0	0	0	0	0	1	0	1	0	0
0	0	0	0	2	0	1	0	0	0	0	0	0	0
0	0	0	0	0	0	0	0	0	0	0	0	0	0
0													
OTU7781	0	0	0	0	0	0	1	0	0	0	0	0	0
0	0	0	0	0	0	0	0	0	0	0	0	0	0
0	0	0	0	0	0	0	0	0	0	0	0	0	0
0	0	0	0	0	0	0	0	0	0	0	0	0	0
0	0	0	0	0	0	0	0	0	0	0	0	0	0
0	0	0	0	0	0	0	0	0	0	0	0	0	0
0	0	0	0	0	0	0	0	0	0	0	0	0	0
0	0	0	0	0	0	0	0	0	0	0	0	0	0
0													

OTU15724	0	0	0	0	0	0	1	0	0	0	0	0	0
0	0	0	0	0	0	0	0	0	0	0	0	0	0
0	0	0	0	0	0	0	0	0	0	0	0	0	0
0	0	0	0	0	0	0	0	0	0	0	0	0	0
0	0	0	0	0	0	0	0	0	0	0	0	0	0
0	0	0	0	0	0	0	0	0	0	0	0	0	0
0	0	0	0	0	0	0	0	0	0	0	0	0	0
0	0	0	0	0	0	0	0	0	0	0	0	0	0
0													
OTU15713	0	0	0	0	0	0	1	0	0	0	0	0	0
0	0	0	0	0	0	0	0	0	0	0	0	0	0
0	0	0	0	0	0	0	0	0	0	0	0	0	0
0	0	0	0	0	0	0	0	0	0	0	0	0	0
0	0	0	0	0	0	0	0	0	0	0	0	0	0
0	0	0	0	0	0	0	0	0	0	0	0	0	0
0	0	0	0	0	0	0	0	0	0	0	0	0	0
0	0	0	0	0	0	0	0	0	0	0	0	0	0
0													
OTU12089	0	0	0	0	0	0	1	0	0	0	0	0	0
0	0	0	0	0	0	0	0	0	0	0	0	0	0
0	0	0	0	0	0	0	0	0	0	0	0	0	0
0	0	0	0	0	0	0	0	0	0	0	0	0	0
0	0	0	0	0	0	0	0	0	0	0	0	0	0
0	0	0	0	0	0	0	0	0	0	0	0	0	0
0	0	0	0	0	0	0	0	0	0	0	0	0	0
0	0	0	0	0	0	0	0	0	0	0	0	0	0
0													
OTU14394	0	0	0	0	0	0	1	0	0	0	0	0	0
0	0	0	0	0	0	0	0	0	0	0	0	0	0
0	0	0	0	0	0	0	0	0	0	0	0	0	0
0	0	0	0	0	0	0	0	0	0	0	0	0	0
0	0	0	0	0	0	0	0	0	0	0	0	0	0
0	0	0	0	0	0	0	0	0	0	0	0	0	0

0	0	0	1	0	0	0	0	0	0	0	0	0	0
0	0	0	0	0	0	0	0	0	0	0	0	0	0
0													
OTU14395	0	0	0	0	0	0	1	0	0	0	0	0	0
0	0	0	0	0	0	0	0	0	0	0	0	0	0
0	0	0	0	0	0	0	0	0	0	0	0	0	0
0	0	0	0	0	0	0	0	0	0	0	0	0	0
0	0	0	0	0	0	0	0	0	0	0	0	0	0
0	0	0	0	0	0	0	0	0	0	0	0	0	0
0	0	0	0	0	0	0	0	0	0	0	0	0	0
0	0	0	0	0	0	0	0	0	0	0	0	0	0
0													
OTU9801	0	0	0	0	0	0	1	1	0	0	1	0	0
0	0	0	0	0	0	0	0	0	0	0	0	0	0
0	0	0	0	0	0	0	0	0	0	0	0	0	0
0	0	0	0	0	1	1	0	0	0	0	0	0	0
0	1	0	0	0	0	0	0	0	0	0	0	0	0
0	1	0	0	0	0	0	1	0	0	0	1	0	0
0	0	0	1	0	1	0	1	0	0	1	0	0	0
0	0	0	0	0	0	0	0	0	0	0	0	0	1
0													
OTU3245	0	0	0	0	0	0	1	0	0	0	1	0	0
0	0	1	0	0	0	0	0	0	0	0	0	0	0
0	0	0	0	0	0	0	0	0	0	0	0	0	0
0	0	0	0	0	0	0	0	0	0	0	0	0	0
0	0	0	0	0	0	0	0	0	0	0	0	0	0
0	0	0	0	0	0	0	0	0	0	0	0	0	0
0	0	0	0	0	0	0	0	0	0	0	0	0	0
0	0	0	1	0	0	0	0	0	0	0	0	0	0
0													
OTU7452	0	0	0	0	0	0	2	0	2	0	0	0	0
0	0	0	0	0	0	0	0	1	0	0	0	5	0
3	0	0	0	1	0	1	2	0	0	0	0	0	0

	1	0	0	0	0	0	0	0	5	1	0	0	0	0
	0	0	0	2	1	0	2	3	0	0	0	0	2	0
	0	4	0	0	1	2	1	0	0	0	0	0	0	8
	0	2	0	0	0	1	1	1	0	1	0	0	4	0
	0	1	4	0	1	3	2	2	1	0	0	0	0	3
	3													
OTU14397	0	0	0	0	0	0	1	0	0	0	0	0	0
	0	0	0	0	0	0	0	0	0	0	0	0	0	0
	0	0	0	0	0	0	0	0	0	0	0	0	0	0
	0	0	0	0	0	0	0	0	0	0	0	0	0	0
	0	0	0	0	0	0	0	0	0	0	0	0	0	0
	0	0	0	0	0	0	0	0	0	0	0	0	0	0
	0	0	0	0	0	0	0	0	0	0	0	0	0	0
	0	0	0	0	0	0	0	0	0	0	0	0	0	0
	0													
OTU14396	0	0	0	0	0	0	1	0	0	0	0	0	0
	0	0	0	0	0	0	0	0	0	0	0	0	0	0
	0	0	0	0	0	0	0	0	0	0	0	0	0	0
	0	0	0	0	0	0	0	0	0	0	0	0	0	0
	0	0	0	0	0	0	0	0	0	0	0	0	0	0
	0	0	0	0	0	0	0	0	0	0	0	0	0	0
	0	0	0	0	0	0	0	0	0	0	0	0	0	0
	0	0	0	0	0	0	0	0	0	0	0	0	0	0
	0													
OTU7778	0	0	0	0	0	0	1	0	0	0	0	0	0
	0	0	0	0	0	0	0	0	0	0	0	0	0	0
	0	0	0	0	0	0	0	0	0	0	0	0	0	0
	0	0	0	0	0	0	0	0	0	0	0	0	0	0
	0	0	0	0	0	0	0	0	0	0	0	0	0	0
	0	0	0	0	0	0	0	0	0	0	0	0	0	0
	0	0	0	0	0	0	0	0	0	0	0	0	0	0
	0	0	0	0	0	0	0	0	0	0	0	0	0	0
	0													

OTU12090 0 0 0 0 0 0 1 0 0 0 0 0 0
0 0 0 0 0 0 0 0 0 0 0 0 0 0
0 0 0 0 0 0 0 0 0 0 0 0 0 0
0 0 0 0 0 0 0 0 0 0 0 0 0 0
0 0 0 0 0 0 0 0 0 0 0 0 0 0
0 0 0 0 0 0 0 0 0 0 0 0 0 0
0 0 0 0 0 0 0 0 0 0 0 0 0 0
0 0 0 0 0 0 0 0 0 0 0 0 0 0
0
OTU12091 0 0 0 0 0 0 0 1 0 0 0 0 0
0 0 0 0 0 0 0 0 0 0 0 0 0 0
0 0 0 0 0 0 0 0 0 0 0 0 0 0
0 0 0 0 0 0 0 0 0 0 0 0 0 0
0 0 0 0 0 0 0 0 0 0 0 0 0 0
0 0 0 0 0 0 0 0 0 0 0 0 0 0
0 0 0 0 0 0 0 0 0 0 0 0 0 0
0 0 0 0 0 0 0 0 0 0 0 0 0 0
0
OTU14391 0 0 0 0 0 0 0 1 0 0 0 0 0
0 0 0 0 0 0 0 0 0 0 0 0 0 0
0 0 0 0 0 0 0 0 0 0 0 0 0 0
0 0 0 0 0 0 0 0 0 0 0 0 0 0
0 0 0 0 0 0 0 0 0 0 0 0 0 0
0 0 0 0 0 0 0 0 0 0 0 0 0 0
0 0 0 0 0 0 0 0 0 0 0 0 0 0
0 0 0 0 0 0 0 0 0 0 0 0 0 0
0
OTU7780 0 0 0 0 0 0 0 1 0 0 0 0 0
0 0 0 0 0 0 0 0 0 0 0 0 0 0
0 0 0 0 0 0 0 0 0 0 0 0 0 0
0 0 0 0 0 0 0 0 0 0 0 0 0 0
0 0 0 0 0 0 0 0 0 0 0 0 0 0
0 0 0 0 0 0 0 0 0 0 0 0 0 0

0	0	0	0	0	0	0	0	0	0	0	0	0	0
0	0	0	0	0	0	0	0	0	0	0	0	0	0
0													
OTU7779	0	0	0	0	0	0	0	1	0	0	0	0	0
0	0	0	0	0	0	0	0	0	0	0	0	0	0
0	0	0	0	0	0	0	0	0	0	0	0	0	0
0	0	0	0	0	0	0	0	0	0	0	0	0	0
0	0	0	0	0	0	0	0	0	0	0	0	0	0
0	0	0	0	0	0	0	0	0	0	0	0	0	0
0	0	0	0	0	0	0	0	0	0	0	0	0	0
0	0	0	0	0	0	0	0	0	0	0	0	0	0
0													
OTU14390	0	0	0	0	0	0	0	1	0	0	0	0	0
0	0	0	0	0	0	0	0	0	0	0	0	0	0
0	0	0	0	0	0	0	0	0	0	0	0	0	0
0	0	0	0	0	0	0	0	0	0	0	0	0	0
0	0	0	0	0	0	0	0	0	0	0	0	0	0
0	0	0	0	0	0	0	0	0	0	0	0	0	0
0	0	0	0	0	0	0	0	0	0	0	0	0	0
0	0	0	0	0	0	0	0	0	0	0	0	0	0
0													
OTU643	0	0	0	0	0	0	0	1	0	0	0	1	1
0	0	1	0	0	0	0	1	0	0	1	0	0	1
0	0	1	0	0	0	1	0	0	0	0	0	0	2
1	0	1	1	0	0	0	1	0	0	0	5	0	1
0	0	1	0	0	0	0	0	0	0	0	0	0	0
2	0	0	1	0	0	0	0	0	0	0	0	0	0
0	0	0	0	0	0	0	0	0	1	0	0	0	0
0	2	0	1	1	0	0	0	0	0	1	1	0	0
0													
OTU9488	0	0	0	0	0	0	0	1	0	0	0	0	0
0	0	0	0	0	0	0	0	0	0	0	2	0	0
0	0	0	1	0	0	0	0	0	0	0	0	0	0

0	0	0	0	0	0	0	0	0	0	0	0	0	0
0	0	0	0	0	0	0	0	0	0	0	0	0	0
0	0	0	0	0	0	0	0	0	0	0	0	0	0
0	0	0	0	0	0	0	0	0	0	0	0	0	0
0	0	0	0	0	0	0	0	0	0	0	0	0	0
0													
OTU587	0	0	0	0	0	0	0	12	0	2	0	0	0
0	0	0	0	0	0	0	0	0	0	0	0	0	0
0	0	0	0	0	0	0	0	0	0	0	0	0	0
0	0	0	0	0	0	0	0	0	0	4	1	0	0
0	0	0	0	0	0	0	0	0	9	0	0	0	0
0	0	0	0	0	0	0	0	0	0	0	0	0	0
0	0	0	0	0	0	0	0	0	0	0	0	0	0
0	0	0	0	0	0	0	0	0	0	0	0	0	0
0													
OTU207	0	0	0	0	0	0	0	2	0	2	0	0	0
0	0	21	0	5	1	2	1	0	4	2	84	0	1
0	0	0	0	21	142	4	0	0	36	30	0	0	0
0	0	0	0	0	0	0	0	1	0	0	1	0	0
0	0	0	0	0	0	0	0	0	0	0	0	0	0
0	0	0	0	0	1	0	0	0	0	0	0	0	0
0	0	0	0	0	0	0	0	0	0	0	0	0	0
0	0	0	0	0	0	0	0	1	0	0	0	1	0
0													
OTU459	0	0	0	0	0	0	0	3	1	0	0	0	0
0	0	0	0	0	0	0	0	0	0	0	0	1	0
1	0	0	0	0	0	0	0	0	0	0	0	5	0
0	0	0	0	0	0	0	0	0	0	0	0	0	0
0	4	0	0	0	0	0	0	0	0	0	0	3	0
0	0	0	0	0	0	0	1	0	0	0	0	3	1
0	0	0	0	0	0	0	0	0	0	0	1	1	0
0	1	0	1	1	2	0	3	0	2	0	0	0	2
4													

OTU14392	0	0	0	0	0	0	0	1	0	0	0	0	0
0	0	0	0	0	0	0	0	0	0	0	0	0	0
0	0	0	0	0	0	0	0	0	0	0	0	0	0
0	0	0	0	0	0	0	0	0	0	0	0	0	0
0	0	0	0	0	0	0	0	0	0	0	0	0	0
0	0	0	0	0	0	0	0	0	0	0	0	0	0
0	0	0	0	0	0	0	0	0	0	0	0	0	0
0	0	0	0	0	0	0	0	0	0	0	0	0	0
0													
OTU12087	0	0	0	0	0	0	0	1	0	0	0	0	0
0	0	0	0	0	0	0	0	0	0	0	0	0	0
0	0	0	0	0	0	0	0	0	0	0	0	0	0
0	0	0	0	0	0	0	0	0	0	0	0	0	0
0	0	0	0	0	0	0	0	0	0	0	0	0	0
0	0	0	0	0	0	0	0	0	0	0	0	0	0
0	0	0	0	0	0	0	0	0	0	0	0	0	0
0	0	0	0	0	0	0	0	0	0	0	0	0	0
0													
OTU422	0	0	0	0	0	0	0	3	1	1	0	0	0
0	0	0	0	0	0	6	0	0	0	0	0	0	0
5	0	0	0	0	0	0	0	0	0	0	0	2	0
3	0	0	0	0	0	0	0	0	0	0	0	0	0
0	3	0	0	0	0	0	0	0	0	0	0	1	0
0	0	2	0	1	0	0	0	0	2	0	0	0	29
0	0	0	0	0	0	11	3	0	0	0	0	2	0
0	0	0	0	0	0	0	0	0	0	0	0	0	0
0													
OTU12088	0	0	0	0	0	0	0	1	0	0	0	0	0
0	0	0	0	0	0	0	0	0	0	0	0	0	0
0	0	0	0	0	0	0	0	0	0	0	0	0	0
0	0	0	0	0	0	0	0	0	0	0	0	0	0
0	0	0	0	0	0	0	0	0	0	0	0	0	0
0	0	0	0	0	0	0	0	0	0	0	0	0	0

0	0	0	0	0	0	0	0	0	0	0	0	0	0
0	0	0	0	0	0	0	0	0	0	0	0	0	0
0													
OTU15640	0	0	0	0	0	0	0	1	0	2	0	0	0
0	0	0	0	0	0	0	0	0	0	0	0	0	0
0	0	0	0	0	0	0	0	0	0	0	0	0	0
0	0	0	0	0	0	0	0	0	0	0	0	0	0
0	0	0	0	0	0	0	0	0	0	0	0	0	0
0	0	0	0	0	0	0	0	0	0	0	0	0	0
0	0	0	0	0	0	0	0	0	0	0	0	0	0
0	0	0	0	0	0	0	0	0	0	0	0	0	0
0													
OTU1608	0	0	0	0	0	0	0	1	0	0	0	0	0
0	1	0	0	0	0	0	0	0	0	0	0	0	0
0	0	0	0	0	0	0	0	0	0	0	0	0	0
0	1	0	0	0	0	0	0	0	0	0	0	0	0
0	0	0	0	0	0	0	0	0	0	0	0	0	0
0	0	0	0	0	0	0	0	3	0	0	0	0	0
0	0	1	0	0	0	0	0	0	0	0	0	0	0
1	0	0	0	0	0	0	0	0	0	0	0	0	0
0													
OTU7787	0	0	0	0	0	0	0	1	0	0	0	0	0
0	0	0	0	0	0	0	0	0	0	0	0	0	0
0	0	0	0	0	0	0	0	0	0	0	0	0	0
0	0	0	0	0	0	0	0	0	0	0	0	0	0
0	0	0	0	0	0	0	0	0	0	0	0	0	0
0	0	0	0	0	0	0	0	0	0	0	0	0	0
0	0	0	0	0	0	0	0	0	0	0	0	0	0
0	0	0	0	0	0	0	0	0	0	0	0	0	0
0													
OTU474	0	0	0	0	0	0	0	1	0	6	0	0	0
0	0	0	0	0	0	4	3	0	0	0	0	0	0
0	0	0	0	0	0	0	0	0	0	1	0	0	0

4 1 0 1 0 0 0 0 1 0 3 4 0 0
0 0 3 3 0 0 0 0 1 0 0 0 0 0
0 1 2 1 4 0 1 0 2 1 0 0 0 2
0 2 0 2 0 0 0 6 0 0 0 2 0 0
0 1 5 10 4 10 1 6 0 3 0 1 0 3
0
OTU966 0 0 0 0 0 0 0 1 0 0 0 0 0
0 0 0 0 0 0 0 0 0 0 0 0 0 0
0 0 0 0 0 0 0 0 0 0 0 0 0 0
0 0 0 0 0 0 0 0 0 0 0 0 0 0
0 0 0 0 0 0 0 0 0 0 0 0 0 0
0 3 0 0 0 0 0 0 0 0 1 0 0 0
0 0 0 0 0 0 0 0 0 0 0 1 0 0
0 0 3 0 0 0 0 0 1 0 0 0 0 0
0
OTU15717 0 0 0 0 0 0 0 1 0 0 0 0 0
0 0 0 0 0 0 0 0 0 0 0 0 0 0
0 0 0 0 0 0 0 0 0 0 0 0 0 0
0 0 0 0 0 0 0 0 0 0 0 0 0 0
0 0 0 0 0 0 0 0 0 0 0 0 0 0
0 0 0 0 0 0 0 0 0 0 0 0 0 0
0 0 0 0 0 0 0 0 0 0 0 0 0 0
0 0 0 0 0 0 0 0 0 0 0 0 0 0
0
OTU14420 0 0 0 0 0 0 0 1 0 0 0 0 0
0 0 0 0 0 0 0 0 0 0 0 0 0 0
0 0 0 0 0 0 0 0 0 0 0 0 0 0
0 0 0 0 0 0 0 0 0 0 0 0 0 0
0 0 0 0 0 0 0 0 0 0 0 0 0 0
0 0 0 0 0 0 0 0 0 0 0 0 0 0
0 0 0 0 0 0 0 0 0 0 0 0 0 0
0 0 0 0 0 0 0 0 0 0 0 0 0 0
0

OTU13170	0	0	0	0	0	0	0	1	0	0	0	0	0
0	0	0	0	0	0	0	0	0	0	0	0	0	0
0	0	0	0	0	2	0	0	0	0	0	0	0	0
0	0	0	0	0	0	0	0	0	0	0	0	0	0
0	0	0	0	0	0	0	0	0	0	0	0	0	0
0	0	0	0	0	0	0	0	0	0	0	0	0	0
0	0	0	0	0	0	0	0	0	0	0	0	0	0
0	0	0	0	0	0	0	0	0	0	0	0	0	0
0													
OTU7785	0	0	0	0	0	0	0	1	0	0	0	0	0
0	0	0	0	0	0	0	0	0	0	0	0	0	0
0	0	0	0	0	0	0	0	0	0	0	0	0	0
0	0	0	0	0	0	0	0	0	0	0	0	0	0
0	0	0	0	0	0	0	0	0	0	0	0	0	0
0	0	0	0	0	0	0	0	0	0	0	0	0	0
0	0	0	0	0	0	0	0	0	0	0	0	0	0
0	0	0	0	0	0	0	0	0	0	0	0	0	0
0													
OTU12092	0	0	0	0	0	0	0	1	0	0	0	0	0
0	0	0	0	0	0	0	0	0	0	0	0	0	0
0	0	0	0	0	0	0	0	0	0	0	0	0	0
0	0	0	0	0	0	0	0	0	0	0	0	0	0
0	0	0	0	0	0	0	0	0	0	0	0	0	0
0	0	0	0	0	0	0	0	0	0	0	0	0	0
0	0	0	0	0	0	0	0	0	0	0	0	0	0
0	0	0	0	0	0	0	0	0	0	0	0	0	0
0													
OTU7770	0	0	0	0	0	0	0	1	0	0	0	0	0
0	0	0	0	0	0	0	0	0	0	0	0	0	0
0	0	0	0	0	0	0	0	0	0	0	0	0	0
0	0	0	0	0	0	0	0	0	0	0	0	0	0
0	0	0	0	0	0	0	0	0	0	0	0	0	0
0	0	0	0	0	0	0	0	0	0	0	0	0	0

0	0	0	0	0	0	0	0	0	0	0	0	0	0
0	0	0	0	0	0	0	0	0	0	0	0	0	0
0													
OTU12093	0	0	0	0	0	0	0	1	0	0	0	0	0
0	0	0	0	0	0	0	0	0	0	0	0	0	0
0	0	0	0	0	0	0	0	0	0	0	0	0	0
0	0	0	0	0	0	0	0	0	0	0	0	0	0
0	0	0	0	0	0	0	0	0	0	0	0	0	0
0	0	0	0	0	0	0	0	0	0	0	0	0	0
0	0	0	0	0	0	0	0	0	0	0	0	0	0
0	0	0	0	0	0	0	0	0	0	0	0	0	0
0													
OTU15718	0	0	0	0	0	0	0	1	0	0	0	0	0
0	0	0	0	0	0	0	0	0	0	0	0	0	0
0	0	0	0	0	0	0	0	0	0	0	0	0	0
0	0	0	0	0	0	0	0	0	0	0	0	0	0
0	0	0	0	0	0	0	0	0	0	0	0	0	0
0	0	0	0	0	0	0	0	0	0	0	0	0	0
0	0	0	0	0	0	0	0	0	0	0	0	0	0
0	0	0	0	0	0	0	0	0	0	0	0	0	0
0													
OTU4306	0	0	0	0	0	0	0	1	0	0	0	0	0
0	0	0	0	0	0	0	0	1	0	0	0	0	0
0	0	0	0	0	0	0	0	0	0	0	0	0	0
0	0	0	0	0	0	0	0	0	0	0	0	0	0
0	0	0	1	0	0	0	0	0	0	0	0	0	0
0	0	1	0	0	0	0	0	0	0	0	0	0	0
0	0	0	0	0	0	0	0	0	0	0	0	0	0
0	0	0	0	0	0	0	0	0	0	0	0	1	0
2													
OTU3921	0	0	0	0	0	0	0	1	0	0	0	2	0
0	0	0	0	0	1	0	0	0	0	0	0	0	0
0	0	0	1	0	0	1	0	0	0	0	0	0	0

0	0	0	0	0	0	0	0	0	0	0	0	0	0
0	0	0	0	0	0	0	0	0	0	0	0	0	0
0	0	0	0	0	0	0	0	0	0	0	0	0	0
0	0	0	0	0	0	0	0	0	0	0	0	0	0
0	0	0	0	0	0	0	0	0	0	0	1	0	0
0													
OTU14417	0	0	0	0	0	0	0	1	0	0	0	0	0
0	0	0	0	0	0	0	0	0	0	0	0	0	0
0	0	0	0	0	0	0	0	0	0	0	0	0	0
0	0	0	0	0	0	0	0	0	0	0	0	0	0
0	0	0	0	0	0	0	0	0	0	0	0	0	0
0	0	0	0	0	0	0	0	0	0	0	0	0	0
0	0	0	0	0	0	0	0	0	0	0	0	0	0
0	0	0	0	0	0	0	0	0	0	0	0	0	0
0													
OTU383	0	0	0	0	0	0	0	4	0	0	0	1	0
0	0	0	0	0	0	1	0	0	0	0	0	0	0
0	0	0	0	0	0	0	0	0	0	0	0	0	0
0	0	0	0	0	0	0	0	0	0	0	1	0	0
0	16	0	0	0	0	1	0	0	0	0	0	1	0
0	32	0	0	0	0	0	0	0	0	0	0	0	0
0	0	0	0	0	0	0	0	0	0	0	0	5	0
0	0	12	37	0	9	11	0	5	19	0	0	0	0
23													
OTU2193	0	0	0	0	0	0	0	1	0	0	0	0	0
0	0	0	0	0	0	0	0	0	0	0	0	0	0
0	0	0	0	0	0	0	0	0	0	0	0	0	0
0	0	0	0	0	0	0	0	0	0	0	0	0	0
0	0	0	0	0	0	0	0	0	0	0	0	0	0
0	0	1	0	0	0	0	0	0	0	0	0	0	0
0	0	0	0	0	0	0	0	0	0	0	0	0	0
0	0	0	0	0	0	0	0	0	0	0	0	0	0
0													

OTU14418	0	0	0	0	0	0	0	1	0	0	0	0	0
0	0	0	0	0	0	0	0	0	0	0	0	0	0
0	0	0	0	0	0	0	0	0	0	0	0	0	0
0	0	0	0	0	0	0	0	0	0	0	0	0	0
0	0	0	0	0	0	0	0	0	0	0	0	0	0
0	0	0	0	0	0	0	0	0	0	0	0	0	0
0	0	0	0	0	0	0	0	0	0	0	0	0	0
0	0	0	0	0	0	0	0	0	0	0	0	0	0
0													
OTU15822	0	0	0	0	0	0	0	3	0	0	0	0	0
0	0	0	0	0	0	0	0	0	0	0	0	0	0
0	0	0	0	0	0	0	0	0	0	0	0	0	0
0	1	0	0	0	0	0	0	0	0	1	0	0	0
0	0	0	0	0	0	0	0	0	0	0	0	0	0
0	0	0	1	0	0	0	0	0	0	0	0	0	0
0	0	0	0	0	0	0	0	0	0	0	0	0	0
0	0	0	0	0	0	0	0	0	0	0	0	0	0
0													
OTU11928	0	0	0	0	0	0	0	1	2	0	0	0	0
0	0	0	1	0	0	1	1	2	0	1	0	8	0
0	3	0	0	1	0	0	0	0	1	1	0	0	0
1	0	0	0	0	0	0	0	0	1	0	2	0	0
0	0	0	3	0	0	0	0	0	0	0	0	0	0
0	0	0	4	0	0	0	4	0	0	0	0	0	0
0	0	0	0	0	0	1	1	0	0	0	0	0	0
0	0	0	0	0	0	0	0	0	0	0	0	0	0
0													
OTU14419	0	0	0	0	0	0	0	1	0	0	0	0	0
0	0	0	0	0	0	0	0	0	0	0	0	0	1
0	0	0	0	0	0	0	0	0	0	0	0	0	0
0	0	0	0	0	0	0	0	0	0	0	0	0	0
0	0	0	0	0	0	0	0	0	0	0	0	0	0
0	0	0	0	0	0	0	0	0	0	0	0	0	0

0	0	0	0	0	0	0	0	0	0	0	0	0	0
0	0	0	0	0	0	0	0	0	0	0	0	0	0
0													
OTU7768	0	0	0	0	0	0	0	1	0	0	0	0	0
0	0	0	0	0	0	0	0	0	0	0	0	0	0
0	0	0	0	0	0	0	0	0	0	0	0	0	0
0	0	0	0	0	0	0	0	0	0	0	0	0	0
0	0	0	0	0	0	0	0	0	0	0	0	0	0
0	0	0	0	0	0	0	0	0	0	0	0	0	0
0	0	0	0	0	0	0	0	0	0	0	0	0	0
0	0	0	0	0	0	0	0	0	0	0	0	0	0
0													
OTU7869	0	0	0	0	0	0	0	2	0	0	0	0	0
0	0	0	0	0	0	0	0	0	0	0	0	0	0
0	0	0	0	0	0	0	0	0	0	0	0	0	0
0	0	0	0	0	0	0	0	0	0	0	0	0	0
0	0	0	0	0	0	0	0	0	0	0	0	0	0
0	0	0	0	0	0	0	0	0	0	0	0	0	0
0	0	0	0	0	0	0	0	0	0	0	0	0	0
0	0	0	0	0	0	0	0	0	0	1	0	0	0
0													
OTU7769	0	0	0	0	0	0	0	1	0	0	0	0	0
0	0	0	0	0	0	0	0	0	0	0	0	0	0
0	0	0	0	0	0	0	0	0	0	0	0	0	0
0	0	0	0	0	0	0	0	0	0	0	0	0	0
0	0	0	0	0	0	0	0	0	0	0	0	0	0
0	0	0	0	0	0	0	0	0	0	0	0	0	0
0	0	0	0	0	0	0	0	0	0	0	0	0	0
0	0	0	0	0	0	0	0	0	0	0	0	0	0
0													
OTU14416	0	0	0	0	0	0	0	1	0	0	0	0	0
0	0	0	0	0	0	0	0	0	0	0	0	0	0
0	0	0	0	0	0	0	0	0	0	0	0	0	0

0	0	0	0	0	0	0	0	0	0	0	0	0	0
0	0	0	0	0	0	0	0	0	0	0	0	0	0
0	0	0	0	0	0	0	0	0	0	0	0	0	0
0	0	0	0	0	0	0	0	0	0	0	0	0	0
0	0	0	0	0	0	0	0	0	0	0	0	0	0
0													
OTU15714	0	0	0	0	0	0	0	1	0	0	0	0	0
0	0	0	0	0	0	0	0	0	0	0	0	0	0
0	0	0	0	0	0	0	0	0	0	0	0	0	0
0	0	0	0	0	0	0	0	0	0	0	0	0	0
0	0	0	0	0	0	0	0	0	0	0	0	0	0
0	0	0	0	0	0	0	0	0	0	0	0	0	0
0	0	0	0	0	0	0	0	0	0	0	0	0	0
0	0	0	0	0	0	0	0	0	0	0	0	0	0
0													
OTU10257	0	0	0	0	0	0	0	1	0	0	0	0	0
0	0	0	0	0	0	0	0	0	0	0	0	0	1
0	0	1	1	0	0	0	0	0	0	0	2	0	0
1	0	1	0	0	0	0	0	0	1	0	0	0	0
0	0	0	0	0	0	0	1	0	0	0	0	1	0
2	0	0	0	0	0	1	0	0	0	0	0	0	0
0	0	0	0	0	1	0	0	0	0	0	0	0	0
0	0	1	0	0	1	0	0	0	0	0	0	0	0
0													
OTU15715	0	0	0	0	0	0	0	1	0	0	0	0	0
0	0	0	0	0	0	0	0	0	0	0	0	0	0
0	0	0	0	0	0	0	0	0	0	0	0	0	0
0	0	0	0	0	0	0	0	0	0	0	0	0	0
0	0	0	0	0	0	0	0	0	0	0	0	0	0
0	0	0	0	0	0	0	0	0	0	0	0	0	0
0	0	0	0	0	0	0	0	0	0	0	0	0	0
0	0	0	0	0	0	0	0	0	0	0	0	0	0
0													

OTU2195 0 0 0 0 0 0 0 1 1 2 0 0 0
0 1 0 0 1 0 0 0 1 0 0 1 1 0
1 0 2 0 0 1 0 1 1 0 1 0 1 0
0 1 2 0 0 1 0 0 1 1 0 0 0 0
2 0 1 0 0 1 1 0 0 1 1 1 0 0
1 1 1 0 2 1 1 2 1 1 1 1 1 0
1 1 0 0 1 0 0 0 0 0 0 0 0 1
0 1 0 0 1 0 0 1 1 0 3 0 0 1
1
OTU15811 0 0 0 0 0 0 0 1 0 0 0 0 0
0 0 0 0 0 0 0 0 0 0 0 0 0 0
0 0 0 0 0 0 0 0 0 0 0 0 0 0
0 0 0 0 0 0 0 0 0 0 0 0 0 0
0 0 0 0 0 0 0 0 0 0 0 0 0 0
0 0 0 0 0 0 0 0 0 0 0 0 0 0
0 0 0 0 0 0 0 0 0 0 0 0 0 0
0 0 0 0 0 0 0 0 0 0 0 0 0 0
0
OTU14428 0 0 0 0 0 0 0 1 0 0 0 0 0
0 0 0 0 0 0 0 0 0 0 0 0 0 0
0 0 0 0 0 0 0 0 0 0 0 0 0 0
0 0 0 0 0 0 0 0 0 0 0 0 0 0
0 0 0 0 0 0 0 0 0 0 0 0 0 0
0 0 0 0 0 0 0 0 0 0 0 0 0 0
0 0 0 0 0 0 0 0 0 0 0 0 0 0
0 0 0 0 0 0 0 0 0 0 0 0 0 0
0
OTU7777 0 0 0 0 0 0 0 1 0 0 0 0 0
0 0 0 0 0 0 0 0 0 0 0 0 0 0
0 0 0 0 0 0 0 0 0 0 0 0 0 0
0 0 0 0 0 0 0 0 0 0 0 0 0 0
0 0 0 0 0 0 0 0 0 0 0 0 0 0
0 0 0 0 0 0 0 0 0 0 0 0 0 0

0	0	0	0	0	0	0	0	0	0	0	0	0	0
0	0	0	0	0	0	0	0	0	0	0	0	0	0
0													
OTU15813	0	0	0	0	0	0	0	1	0	0	0	0	0
0	0	0	0	0	0	0	0	0	0	0	0	0	0
0	0	0	0	0	0	0	0	0	0	0	0	0	0
0	0	0	0	0	0	0	0	0	0	0	0	0	0
0	0	0	0	0	0	0	0	0	0	0	0	0	0
0	0	0	0	0	0	0	0	0	0	0	0	0	0
0	0	0	0	0	0	0	0	0	0	0	0	0	0
0	0	0	0	0	0	0	0	0	0	0	0	0	0
0													
OTU15814	0	0	0	0	0	0	0	1	0	0	0	0	0
0	0	0	0	0	0	0	0	0	0	0	0	0	0
0	0	0	0	0	0	0	0	0	0	0	0	0	0
0	0	0	0	0	0	0	0	0	0	0	0	0	0
0	0	0	0	0	0	0	0	0	0	0	0	0	0
0	0	0	0	0	0	0	0	0	0	0	0	0	0
0	0	0	0	0	0	0	0	0	0	0	0	0	0
0	0	0	0	0	0	0	0	0	0	0	0	0	0
0													
OTU14431	0	0	0	0	0	0	0	1	0	0	0	0	0
0	0	0	0	0	0	0	0	0	0	0	0	0	0
0	0	0	0	0	0	0	0	0	0	0	0	0	0
0	0	0	0	0	0	0	0	0	0	0	0	0	0
0	0	0	0	0	0	0	0	0	0	0	0	0	0
0	0	0	0	0	0	0	0	0	0	0	0	0	0
0	0	0	0	0	0	0	0	0	0	0	0	0	0
0	0	0	0	0	0	0	0	0	0	0	0	0	0
0													
OTU14429	0	0	0	0	0	0	0	1	0	0	0	0	0
0	0	0	0	0	0	0	0	0	0	0	0	0	0
0	0	0	0	0	0	0	0	0	0	0	0	0	0

0 0 0 0 0 0 0 0 0 0 0 0 0 0
0 0 0 0 0 0 0 0 0 0 0 0 0 0
0 0 0 0 0 0 0 0 0 0 0 0 0 0
0 0 0 0 0 0 0 0 0 0 0 0 0 0
0 0 0 0 0 0 0 0 0 0 0 0 0 0
0
OTU15812 0 0 0 0 0 0 0 1 0 0 0 0 0
0 0 0 0 0 0 0 0 0 0 0 0 0 0
0 0 0 0 0 0 0 0 0 0 0 0 0 0
0 0 0 0 0 0 0 0 0 0 0 0 0 0
0 0 0 0 0 0 0 0 0 0 0 0 0 0
0 0 0 0 0 0 0 0 0 0 0 0 0 0
0 0 0 0 0 0 0 0 0 0 0 0 0 0
0 0 0 0 0 0 0 0 0 0 0 0 0 0
0
OTU14430 0 0 0 0 0 0 0 1 0 0 0 0 0
0 0 0 0 0 0 0 0 0 0 0 0 0 0
0 0 0 0 0 0 0 0 0 0 0 0 0 0
0 0 0 0 0 0 0 0 0 0 0 0 0 0
0 0 0 0 0 0 0 0 0 0 0 0 0 0
0 0 0 0 0 0 0 0 0 0 0 0 0 0
0 0 0 0 0 0 0 0 0 0 0 0 0 0
0 0 0 0 0 0 0 0 0 0 0 0 0 0
0
OTU14424 0 0 0 0 0 0 0 1 0 0 0 0 0
0 0 0 0 0 0 0 0 0 0 0 0 0 0
0 0 0 0 0 0 0 0 0 0 0 0 0 0
0 0 0 0 0 0 0 0 0 0 0 0 0 0
0 0 0 0 0 0 0 0 0 0 0 0 0 0
0 0 0 0 0 0 0 0 0 0 0 0 0 0
0 0 0 0 0 0 0 0 0 0 0 0 0 0
0 0 0 0 0 0 0 0 0 0 0 0 0 0
0

OTU14423	0	0	0	0	0	0	0	1	0	0	0	0	0
0	0	0	0	0	0	0	0	0	0	0	0	0	0
0	0	0	0	0	0	0	0	0	0	0	0	0	0
0	0	0	0	0	0	0	0	0	0	0	0	0	0
0	0	0	0	0	0	0	0	0	0	0	0	0	0
0	0	0	0	0	0	0	0	0	0	0	0	0	0
0	0	0	0	0	0	0	0	0	0	0	0	0	0
0	0	0	0	0	0	0	0	0	0	0	0	0	0
0													
OTU15807	0	0	0	0	0	0	0	1	0	0	0	0	0
0	0	0	0	0	0	0	0	0	0	0	0	0	0
0	0	0	0	0	0	0	0	0	0	0	0	0	0
0	0	0	0	0	0	0	0	0	0	0	0	0	0
0	0	0	0	0	0	0	0	0	0	0	0	0	0
0	0	0	0	0	0	0	0	0	0	0	0	0	0
0	0	0	0	0	0	0	0	0	0	0	0	0	0
0	0	0	0	0	0	0	0	0	0	0	0	0	0
0													
OTU12079	0	0	0	0	0	0	0	1	0	0	0	0	0
0	0	0	0	0	0	0	0	0	0	0	0	0	0
0	0	0	0	0	0	0	0	0	0	0	0	0	0
0	0	0	0	0	0	0	0	0	0	0	0	0	0
0	0	0	0	0	0	0	0	0	0	0	0	0	0
0	0	0	0	0	0	0	0	0	0	0	0	0	0
0	0	0	0	0	0	0	0	0	0	0	0	0	0
0	0	0	0	0	0	0	0	0	0	0	0	0	0
0													
OTU15808	0	0	0	0	0	0	0	1	0	0	0	0	0
0	0	0	0	0	0	0	0	0	0	0	0	0	0
0	0	0	0	0	0	0	0	0	0	0	0	0	0
0	0	0	0	0	0	0	0	0	0	0	0	0	0
0	0	0	0	0	0	0	0	0	0	0	0	0	0
0	0	0	0	0	0	0	0	0	0	0	0	0	0

0 0 0 0 0 0 0 0 0 0 0 0 0 0
0 0 0 0 0 0 0 0 0 0 0 0 0 0
0
OTU7776 0 0 0 0 0 0 0 1 0 0 0 0 0
0 0 0 0 0 0 0 0 0 0 0 0 0 0
0 0 0 0 0 0 0 0 0 0 0 0 0 0
0 0 0 0 0 0 0 0 0 0 0 0 0 0
0 0 0 0 0 0 0 0 0 0 0 0 0 0
0 0 0 0 0 0 0 0 0 0 0 0 0 0
0 0 0 0 0 0 0 0 0 0 0 0 0 0
0 0 0 0 0 0 0 0 0 0 0 0 0 0
0
OTU7775 0 0 0 0 0 0 0 1 0 0 0 0 0
0 0 0 0 0 0 0 0 0 0 0 0 0 0
0 0 0 0 0 0 0 0 0 0 0 0 0 0
0 0 0 0 0 0 0 0 0 0 0 0 0 0
0 0 0 0 0 0 0 0 0 0 0 0 0 0
0 0 0 0 0 0 0 0 0 0 0 0 0 0
0 0 0 0 0 0 0 0 0 0 0 0 0 0
0 0 0 0 0 0 0 0 0 0 0 0 0 0
0
OTU14421 0 0 0 0 0 0 0 1 0 0 0 0 0
0 0 0 0 0 0 0 0 0 0 0 0 0 0
0 0 0 0 0 0 0 0 0 0 0 0 0 0
0 0 0 0 0 0 0 0 0 0 0 0 0 0
0 0 0 0 0 0 0 0 0 0 0 0 0 0
0 0 0 0 0 0 0 0 0 0 0 0 0 0
0 0 0 0 0 0 0 0 0 0 0 0 0 0
0 0 0 0 0 0 0 0 0 0 0 0 0 0
0
OTU14422 0 0 0 0 0 0 0 1 0 0 0 0 0
0 0 0 0 0 0 0 0 0 0 0 0 0 0
0 0 0 0 0 0 0 0 0 0 0 0 0 0

0	0	0	0	0	0	0	0	0	0	0	0	0	0
0	0	0	0	0	0	0	0	0	0	0	0	0	0
0	0	0	0	0	0	0	0	0	0	0	0	0	0
0	0	0	0	0	0	0	0	0	0	0	0	0	0
0	0	0	0	0	0	0	0	0	0	0	0	0	0
0													
OTU598	0	0	0	0	0	0	0	2	0	0	0	0	0
0	0	0	0	0	0	0	0	0	0	2	0	0	0
5	0	0	0	12	0	0	0	0	0	0	0	0	0
2	0	0	0	0	0	0	0	0	0	0	0	0	0
0	0	0	0	0	0	0	0	0	0	0	0	0	0
0	0	0	0	0	0	0	0	0	0	0	0	0	0
0	0	0	0	0	0	0	0	0	0	0	0	0	0
0	0	0	0	0	0	0	0	5	2	0	0	0	3
0													
OTU7877	0	0	0	0	0	0	0	1	0	0	0	0	0
0	0	0	0	0	0	0	0	0	0	0	0	0	0
0	0	0	0	0	0	0	0	0	0	0	0	0	0
0	0	0	0	0	0	0	0	0	0	0	0	0	0
0	0	0	0	0	0	0	0	0	0	0	0	0	0
0	0	0	0	0	0	0	0	0	0	0	0	0	0
0	0	0	0	0	0	0	0	0	0	0	0	0	0
0	0	0	0	0	0	0	0	0	0	0	0	0	0
0													
OTU14425	0	0	0	0	0	0	0	1	0	0	0	0	0
0	0	0	0	0	0	0	0	0	0	0	0	0	0
0	0	0	0	0	0	0	0	0	0	0	0	0	0
0	0	0	0	0	0	0	0	0	0	0	0	0	0
0	0	0	0	0	0	0	0	0	0	0	0	0	0
0	0	0	0	0	0	0	0	0	0	0	0	0	0
0	0	0	0	0	0	0	0	0	0	0	0	0	0
0	0	0	0	0	0	0	0	0	0	0	0	0	0
0													

OTU8866	0	0	0	0	0	0	0	1	0	0	0	1	0
0	0	0	0	0	0	0	0	0	0	0	0	0	0
0	0	0	0	0	0	0	1	0	0	0	0	1	0
0	0	1	0	0	0	0	0	0	0	0	0	0	0
0	0	0	0	0	0	0	0	0	0	0	0	0	0
0	0	0	0	0	0	0	0	0	0	0	0	0	0
0	0	0	0	0	0	0	0	0	0	0	0	0	0
0	0	0	0	0	0	0	0	0	0	0	0	0	0
0													
OTU9428	0	0	0	0	0	0	0	1	0	0	0	0	0
0	0	0	0	0	0	0	0	0	0	0	0	0	0
0	0	0	1	0	0	0	0	0	0	0	0	0	0
0	0	0	0	0	0	0	0	0	1	0	0	0	0
0	0	0	0	0	0	0	0	0	0	0	0	0	0
0	0	0	0	0	0	0	1	0	0	0	0	0	0
0	0	0	0	0	0	0	1	0	0	0	0	0	0
0	0	0	0	0	0	0	0	0	0	0	0	0	0
0													
OTU15809	0	0	0	0	0	0	0	1	0	0	0	0	0
0	0	0	0	0	0	0	0	0	0	0	0	0	0
0	0	0	0	0	0	0	0	0	0	0	0	0	0
0	0	0	0	0	0	0	0	0	0	0	0	0	0
0	0	0	0	0	0	0	0	0	0	0	0	0	0
0	0	0	0	0	0	0	0	0	0	0	0	0	0
0	0	0	0	0	0	0	0	0	0	0	0	0	0
0	0	0	0	0	0	0	0	0	0	0	0	0	0
0													
OTU7878	0	0	0	0	0	0	0	1	0	0	0	0	0
0	0	0	0	0	0	0	0	0	0	0	0	0	0
0	0	0	0	0	0	0	0	0	0	0	0	0	0
0	0	0	0	0	0	0	0	0	0	0	0	0	0
0	0	0	0	0	0	0	0	0	0	0	0	0	0
0	0	0	0	0	0	0	0	0	0	0	0	0	0

0	0	0	0	0	0	0	0	0	0	0	0	0	0
0	0	0	0	0	0	0	0	0	0	0	0	0	0
0													
OTU4967	0	0	0	0	0	0	0	2	0	0	0	0	0
0	0	0	0	0	0	0	0	0	0	0	1	0	0
0	0	0	0	0	0	0	0	0	0	0	0	0	0
0	0	0	0	0	0	0	0	0	0	1	0	0	0
0	0	0	1	0	1	0	1	0	0	0	0	0	0
1	0	0	0	0	0	0	0	0	0	0	1	0	0
0	0	0	0	0	0	0	0	0	0	0	0	0	0
0	0	0	0	0	0	0	0	0	0	0	0	0	0
0													
OTU14409	0	0	0	0	0	0	0	1	0	0	0	0	0
0	0	0	0	0	0	0	0	0	0	0	0	0	0
0	0	0	0	0	0	0	0	0	0	0	0	0	0
0	0	0	0	0	0	0	0	0	0	0	0	0	0
0	0	0	0	0	0	0	0	0	0	0	0	0	0
0	0	0	0	0	0	0	0	0	0	0	0	0	0
0	0	0	0	0	0	0	0	0	0	0	0	0	0
0	0	0	0	0	0	0	0	0	0	0	0	0	0
0													
OTU7876	0	0	0	0	0	0	0	2	0	0	0	0	0
0	0	0	0	0	0	0	0	0	0	0	0	0	0
0	0	0	0	0	0	0	0	0	0	0	0	0	0
0	0	0	0	0	0	0	0	0	0	0	0	1	0
0	0	0	0	0	0	0	0	0	0	0	0	0	0
0	0	0	0	0	0	0	0	0	0	0	0	0	0
0	0	0	0	0	0	0	0	0	0	0	0	0	0
0	0	0	0	0	0	0	0	0	0	0	0	0	0
0													
OTU12084	0	0	0	0	0	0	0	1	0	0	0	0	0
0	0	0	0	0	0	0	0	0	0	0	0	0	0
0	0	0	0	0	0	0	0	0	0	0	0	0	0

0	0	0	0	0	0	0	0	0	0	0	0	0	0
0	0	0	0	0	0	0	0	0	0	0	0	0	0
0	0	0	0	0	0	0	0	0	0	0	0	0	0
0	0	0	0	0	0	0	0	0	0	0	0	0	0
0	0	0	0	0	0	0	0	0	0	0	0	0	0
0													
OTU12085	0	0	0	0	0	0	0	1	0	0	0	0	0
0	0	0	0	0	0	0	0	0	0	0	0	0	0
0	0	0	0	0	0	0	0	0	0	0	0	0	0
0	0	0	0	0	0	0	0	0	0	0	0	0	0
0	0	0	0	0	0	0	0	0	0	0	0	0	0
0	0	0	0	0	0	0	0	0	0	0	0	0	0
0	0	0	0	0	0	0	0	0	0	0	0	0	0
0	0	0	0	0	0	0	0	0	0	0	0	0	0
0													
OTU7874	0	0	0	0	0	0	0	1	0	0	0	0	0
0	0	0	0	0	0	0	0	0	0	0	0	0	0
0	0	0	0	0	0	0	0	0	0	0	0	0	0
0	0	0	0	0	0	0	0	0	0	0	0	0	0
0	0	0	0	0	0	0	0	0	0	0	0	0	0
0	0	0	1	0	0	0	0	0	0	0	0	0	0
0	0	0	0	0	0	0	0	0	0	0	0	0	0
0	0	1	0	0	0	0	0	0	0	0	0	0	0
0													
OTU9662	0	0	0	0	0	0	0	1	0	0	0	0	0
0	0	0	1	0	1	0	0	0	0	0	0	0	0
1	0	0	0	0	0	0	1	0	0	0	1	0	0
0	0	2	0	1	0	0	0	0	0	0	0	0	0
0	0	0	0	0	0	0	0	0	0	0	0	0	0
0	0	2	1	0	0	0	2	0	2	0	2	0	0
0	0	0	0	0	0	2	0	0	0	0	0	0	0
0	0	0	0	0	1	0	0	0	1	0	0	0	1
0													

OTU6698	0	0	0	0	0	0	0	1	0	0	0	0	0
0	0	0	0	0	0	0	0	0	0	0	0	0	0
0	0	0	0	0	0	0	0	0	0	0	0	0	0
0	0	0	0	0	0	0	0	0	0	0	0	0	0
0	0	0	0	0	0	0	0	0	0	0	0	0	0
0	0	0	0	0	0	0	0	0	0	0	1	0	0
0	0	0	0	0	0	1	0	0	0	0	0	0	0
0	0	0	0	0	0	0	0	0	0	0	0	0	0
0													
OTU14410	0	0	0	0	0	0	0	1	0	0	0	0	0
0	0	0	0	0	0	0	0	0	0	0	0	1	0
0	0	0	0	0	0	1	0	0	0	0	0	0	0
0	2	0	1	0	0	0	0	0	0	0	0	0	0
0	0	0	1	0	0	0	0	0	0	0	1	0	0
0	0	0	0	0	0	0	0	0	0	0	0	0	0
0	0	0	2	0	0	0	0	0	0	0	0	0	0
0	0	0	0	0	0	0	0	0	0	0	0	0	0
0													
OTU14411	0	0	0	0	0	0	0	1	0	0	0	0	0
0	0	0	0	0	0	0	0	0	0	0	0	0	0
0	0	0	0	0	0	0	0	0	0	0	0	0	0
0	0	0	0	0	0	0	0	0	0	0	0	0	0
0	0	0	0	0	0	0	0	0	0	0	0	0	0
0	0	0	0	0	0	0	0	0	0	0	0	0	0
0	0	0	0	0	0	0	0	0	0	0	0	0	0
0	0	0	0	0	0	0	0	0	0	0	0	0	0
0													
OTU14412	0	0	0	0	0	0	0	1	0	0	0	0	0
0	0	0	0	0	0	0	0	0	0	0	0	0	0
0	0	0	0	0	0	0	0	0	0	0	0	0	0
0	0	0	0	0	0	0	0	0	0	0	0	0	0
0	0	0	0	0	0	0	0	0	0	0	0	0	0
0	0	0	0	0	0	0	0	0	0	0	0	0	0

0	0	0	0	0	0	0	0	0	0	0	0	0	0
0	0	0	0	0	0	0	0	0	0	0	0	0	0
0													
OTU12086	0	0	0	0	0	0	0	1	0	0	0	0	0
0	0	0	0	0	0	0	0	0	0	0	0	0	0
0	0	0	0	0	0	0	0	0	0	0	0	0	0
0	0	0	0	0	0	0	0	0	0	0	0	0	0
0	0	0	0	0	0	0	0	0	0	0	0	0	0
0	0	0	0	0	0	0	0	0	0	0	0	0	1
0	0	0	0	0	0	0	0	0	0	0	0	0	0
0	0	0	0	0	0	0	0	0	0	0	0	0	0
0													
OTU13500	0	0	0	0	0	0	0	1	0	0	0	0	0
0	0	0	0	0	0	0	0	0	0	0	0	0	0
0	0	0	0	0	0	0	0	0	0	0	0	0	0
0	0	0	0	0	0	0	0	0	0	0	0	0	0
0	0	0	0	0	0	0	0	0	0	0	0	0	0
0	0	0	0	0	0	0	0	0	0	0	0	0	0
0	1	0	0	0	0	0	0	0	0	0	0	0	0
0	0	0	0	0	0	0	0	0	0	0	0	0	0
0													
OTU15819	0	0	0	0	0	0	0	1	0	0	0	0	0
0	0	0	0	0	0	0	0	0	0	0	0	0	0
0	0	0	0	0	0	0	0	0	0	0	0	0	0
0	0	0	0	0	0	0	0	0	0	0	0	0	0
0	0	0	0	0	0	0	0	0	0	0	0	0	0
0	0	0	0	0	0	0	0	0	0	0	0	0	0
0	0	0	0	0	0	0	0	0	0	0	0	0	0
0	0	0	0	0	0	0	0	0	0	0	0	0	0
0													
OTU14407	0	0	0	0	0	0	0	1	0	0	0	0	0
0	0	0	0	0	0	0	0	0	0	0	0	0	0
0	0	0	0	0	0	0	0	0	0	0	0	0	0

0	0	0	0	0	0	0	0	0	0	0	0	0	0
0	0	0	0	0	0	0	0	0	0	0	0	0	0
0	0	0	0	0	0	0	0	0	0	0	0	0	0
0	0	0	0	0	0	0	0	0	0	0	0	0	0
0	0	0	0	0	0	0	0	0	0	0	0	0	0
0													
OTU7884	0	0	0	0	0	0	0	1	0	0	0	0	0
0	0	0	0	0	0	0	0	0	0	0	0	0	0
0	0	0	0	0	0	0	0	0	0	0	0	0	0
0	0	0	0	0	0	0	0	0	0	0	0	0	0
0	0	0	0	0	0	0	0	0	0	0	0	0	0
0	0	0	0	0	0	0	0	0	0	0	0	0	0
0	0	0	0	0	0	0	0	0	0	0	0	0	0
0	0	0	0	0	0	0	0	0	0	0	0	0	0
0													
OTU7881	0	0	0	0	0	0	0	1	0	0	0	0	0
0	0	0	0	0	0	0	0	0	0	0	0	0	0
0	0	0	0	0	0	0	0	0	0	0	0	0	0
0	0	0	0	0	0	0	0	0	0	0	0	0	0
0	0	0	0	0	0	0	0	0	0	0	0	0	0
0	0	0	0	0	0	0	0	0	0	0	0	0	0
0	0	0	0	0	0	0	0	0	0	0	0	0	0
0	0	0	0	0	0	0	0	0	0	0	0	0	0
0													
OTU12081	0	0	0	0	0	0	0	1	0	0	0	0	0
0	0	0	0	0	0	0	0	0	0	0	0	0	0
0	0	0	0	0	0	0	0	0	0	0	0	0	0
0	0	0	0	0	0	0	0	0	0	0	0	0	0
0	0	0	0	0	0	0	0	0	0	0	0	0	0
0	0	0	0	0	0	0	0	0	0	0	0	0	0
0	0	0	0	0	0	0	0	0	0	0	0	0	0
0	0	0	0	0	0	0	0	0	0	0	0	0	0
0													

OTU15815	0	0	0	0	0	0	0	1	0	0	0	0	0
0	0	0	0	0	0	0	0	0	0	0	0	0	0
0	0	0	0	0	0	0	0	0	0	0	0	0	0
0	0	0	0	0	0	0	0	0	0	0	0	0	0
0	0	0	0	0	0	0	0	0	0	0	0	0	0
0	0	0	0	0	0	0	0	0	0	0	0	0	0
0	0	0	0	0	0	0	0	0	0	0	0	0	0
0	0	0	0	0	0	0	0	0	0	0	0	0	0
0													
OTU3867	0	0	0	0	0	0	0	1	0	0	0	0	0
0	0	0	0	0	0	0	0	0	0	0	0	0	0
0	0	0	0	1	0	0	0	0	0	0	0	0	0
0	0	0	0	0	0	0	0	0	0	0	0	0	0
0	0	0	0	0	0	0	0	0	0	0	0	0	0
0	0	0	0	0	0	0	0	0	0	0	0	0	0
0	0	0	0	0	0	0	0	0	0	0	0	0	0
0	0	0	0	0	0	0	0	0	0	0	0	0	0
0													
OTU15816	0	0	0	0	0	0	0	1	0	0	0	0	0
0	0	0	0	0	0	0	0	0	0	0	0	0	0
0	0	0	0	0	0	0	0	0	0	0	0	0	0
0	0	0	0	0	0	0	0	0	0	0	0	0	0
0	0	0	0	0	0	0	0	0	0	0	0	0	0
0	0	0	0	0	0	0	0	0	0	0	0	0	0
0	0	0	0	0	0	0	0	0	0	0	0	0	0
0	0	0	0	0	0	0	0	0	0	0	0	0	0
0													
OTU14406	0	0	0	0	0	0	0	1	0	0	0	0	0
0	0	0	0	0	0	0	0	0	0	0	0	0	0
0	0	0	0	0	0	0	0	0	0	0	0	0	0
0	0	0	0	0	0	0	0	0	0	0	0	0	0
0	0	0	0	0	0	0	0	0	0	0	0	0	0
0	0	0	0	0	0	0	0	0	0	0	0	0	0

0	0	0	0	0	0	0	0	0	0	0	0	0	0
0	0	0	0	0	0	0	0	0	0	0	0	0	0
0													
OTU14408	0	0	0	0	0	0	0	1	0	0	0	0	0
0	0	0	0	0	0	0	0	0	0	0	0	0	0
0	0	0	0	0	0	0	0	0	0	0	0	0	0
0	0	0	0	0	0	0	0	0	0	0	0	0	0
0	0	0	0	0	0	0	0	0	0	0	0	0	0
0	0	0	0	0	0	0	0	0	0	0	0	0	0
0	0	0	0	0	0	0	0	0	0	0	0	0	0
0	0	0	0	0	0	0	0	0	0	0	0	0	0
0													
OTU15818	0	0	0	0	0	0	0	1	0	0	0	0	0
0	0	0	0	0	0	0	0	0	0	0	0	0	0
0	0	0	0	0	0	0	0	0	0	0	0	0	0
0	0	0	0	0	0	0	0	0	0	0	0	0	0
0	0	0	0	0	0	0	0	0	0	0	0	0	0
0	0	0	0	0	0	0	0	0	0	0	0	0	0
0	0	0	0	0	0	0	0	0	0	0	0	0	0
0	0	0	0	0	0	0	0	0	0	0	0	0	0
0													
OTU7885	0	0	0	0	0	0	0	1	0	0	0	0	0
0	0	0	0	0	0	0	0	0	0	0	0	0	0
0	0	0	0	0	0	0	0	0	0	0	0	0	0
0	0	0	0	0	0	0	0	0	0	0	0	0	0
0	0	0	0	0	0	0	0	0	0	0	0	0	0
0	0	0	0	0	0	0	0	0	0	0	0	0	0
0	0	0	0	0	0	0	0	0	0	0	0	0	0
0	0	0	0	0	0	0	0	0	0	0	0	0	0
0													
OTU13571	0	0	0	0	0	0	0	1	0	0	0	0	0
0	0	0	0	0	0	0	0	0	0	0	0	0	0
0	0	0	0	0	0	0	1	0	0	0	0	1	0

2 0 0 0 0 0 0 0 0 0 0 0 0 0
0 0 0 0 0 0 0 0 0 0 0 0 0 0
0 0 0 0 0 0 0 0 0 1 1 0 0 0
0 0 0 0 0 0 0 0 0 0 0 0 0 0
0 0 0 0 0 0 0 0 0 0 1 0 1 0
0
OTU1058 0 0 0 0 0 0 0 1 0 0 0 1 0
0 0 0 4 0 0 0 0 4 0 0 0 0 0
1 0 0 0 0 0 3 1 0 0 0 0 0 0
0 0 0 0 0 0 0 0 0 0 2 2 0 0
1 1 1 0 0 0 0 0 0 0 0 0 0 0
0 0 0 2 0 0 0 0 2 0 1 0 0 0
0 0 0 0 0 0 0 0 1 0 0 0 0 0
0 0 0 0 0 0 0 0 0 0 1 1 0 0
0
OTU15817 0 0 0 0 0 0 0 1 0 0 0 0 0
0 0 0 0 0 0 0 0 0 0 0 0 0 0
0 0 0 0 0 0 0 0 0 0 0 0 0 0
0 0 0 0 0 0 0 0 0 0 0 0 0 0
0 0 0 0 0 0 0 0 0 0 0 0 0 0
0 0 0 0 0 0 0 0 0 0 0 0 0 0
0 0 0 0 0 0 0 0 0 0 0 0 0 0
0 0 0 0 0 0 0 0 0 0 0 0 0 0
0
OTU12082 0 0 0 0 0 0 0 1 0 0 0 0 0
0 0 0 0 0 0 0 0 0 0 0 0 0 0
0 0 0 0 0 0 0 0 0 0 0 0 0 0
0 0 0 0 0 0 0 0 0 0 0 0 0 0
0 0 0 0 0 0 0 0 0 0 0 0 0 0
0 0 0 0 0 0 0 0 0 0 0 0 0 0
0 0 0 0 0 0 0 0 0 0 0 0 0 0
0 0 0 0 0 0 0 0 0 0 0 0 0 0
0

OTU12083	0	0	0	0	0	0	0	1	0	0	0	0	0
0	0	0	0	0	0	0	0	0	0	0	0	0	0
0	0	0	0	0	0	0	0	0	0	0	0	0	0
0	0	0	0	0	0	0	0	0	0	0	0	0	0
0	0	0	0	0	0	0	0	0	0	0	0	0	0
0	0	0	0	0	0	0	0	0	0	0	0	0	0
0	0	0	0	0	0	0	0	0	0	0	0	0	0
0	0	0	0	0	0	0	0	0	0	0	0	0	0
0													
OTU1049	0	0	0	0	0	0	0	1	0	0	0	1	0
0	0	1	0	1	0	0	0	0	0	0	0	0	0
0	0	0	0	0	0	0	1	0	1	0	0	0	0
0	2	0	0	0	0	0	0	0	0	0	0	0	0
0	0	0	1	0	0	0	0	0	0	0	0	0	0
0	0	0	0	0	0	1	0	0	0	1	0	2	0
0	0	0	0	0	0	0	0	0	0	0	0	0	0
0	0	0	0	0	0	0	1	0	0	0	0	0	0
0													
OTU15803	0	0	0	0	0	0	0	1	0	0	0	0	0
0	0	0	0	0	0	0	0	0	0	0	0	0	0
0	0	0	0	0	0	0	0	0	0	0	0	0	0
0	0	0	0	0	0	0	0	0	0	0	0	0	0
0	0	0	0	0	0	0	0	0	0	0	0	0	0
0	0	0	0	0	0	0	0	0	0	0	0	0	0
0	0	0	0	0	0	0	0	0	0	0	0	0	0
0	0	0	0	0	0	0	0	0	0	0	0	0	0
0													
OTU12105	0	0	0	0	0	0	0	1	0	0	0	0	0
0	0	0	0	0	0	0	0	0	0	0	0	0	0
0	0	0	0	0	0	0	0	0	0	0	0	0	0
0	0	0	0	0	0	0	0	0	0	0	0	0	0
0	0	0	0	0	0	0	0	0	0	0	0	0	0
0	0	0	0	0	0	0	0	0	0	0	0	0	0

0	0	0	0	0	0	0	0	0	0	0	0	0	0
0	0	0	0	0	0	0	0	0	0	0	0	0	0
0													
OTU1279	0	0	0	0	0	0	0	1	0	0	0	0	0
0	2	0	0	2	0	0	0	0	0	0	0	0	0
0	0	1	3	0	0	0	0	0	0	0	0	0	0
1	0	0	0	0	0	0	0	0	0	0	0	0	0
1	0	0	0	0	0	0	0	0	0	0	0	0	0
0	0	3	1	0	8	1	0	1	0	1	0	4	0
0	0	2	1	3	0	0	0	0	0	1	0	0	0
2	0	0	0	0	0	0	0	0	0	0	0	0	0
0													
OTU12104	0	0	0	0	0	0	0	1	0	0	0	0	0
0	0	0	0	0	0	0	0	0	0	0	0	0	0
0	0	0	0	0	0	0	0	0	0	0	0	0	0
0	0	0	0	0	0	0	0	0	0	0	0	0	0
0	0	0	0	0	0	0	0	0	0	0	0	0	0
0	0	0	0	0	0	0	0	0	0	0	0	0	0
0	0	0	0	0	0	0	0	0	0	0	0	0	0
0	0	0	0	0	0	0	0	0	0	0	0	0	0
0													
OTU12107	0	0	0	0	0	0	0	1	0	0	0	0	0
0	0	0	0	0	0	0	0	0	0	0	0	0	0
0	0	0	0	0	0	0	0	0	0	0	0	0	0
0	0	0	0	0	0	0	0	0	0	0	0	0	0
0	0	0	0	0	0	0	0	0	0	0	0	0	0
0	0	0	0	0	0	0	0	0	0	0	0	0	0
0	0	0	0	0	0	0	0	0	0	0	0	0	0
0	0	0	0	0	0	0	0	0	0	0	0	0	0
0													
OTU14414	0	0	0	0	0	0	0	1	0	0	0	0	0
0	0	0	0	0	0	0	0	0	0	0	0	0	0
0	0	0	0	0	0	0	0	0	0	0	0	0	0

0 0 0 0 0 0 0 0 0 0 0 0 0 0
0 0 0 0 0 0 0 0 0 0 0 0 0 0
0 0 0 0 0 0 0 0 0 0 0 0 0 0
0 0 0 0 0 0 0 0 0 0 0 0 0 0
0 0 0 0 0 0 0 0 0 0 0 0 0 0
0
OTU7866 0 0 0 0 0 0 0 1 0 0 0 0 0
0 0 0 0 0 0 0 0 0 0 0 0 0 0
0 0 0 0 0 0 0 0 0 0 0 0 0 0
0 0 0 0 0 0 0 0 0 0 0 0 0 0
0 0 0 0 0 0 0 0 0 0 0 0 0 0
0 0 0 0 0 0 0 0 0 0 0 0 0 0
0 0 0 0 0 0 0 0 0 0 0 0 0 0
0 0 0 0 0 0 0 0 0 0 0 0 0 0
0
OTU14415 0 0 0 0 0 0 0 1 0 0 0 0 0
0 0 0 0 0 0 0 0 0 0 0 0 0 0
0 0 0 0 0 0 0 0 0 0 0 0 0 0
0 0 0 0 0 0 0 0 0 0 0 0 0 0
0 0 0 0 0 0 0 0 0 0 0 0 0 0
0 0 0 0 0 0 0 0 0 0 0 0 0 0
0 0 0 0 0 0 0 0 0 0 0 0 0 0
0 0 0 0 0 0 0 0 0 0 0 0 0 0
0
OTU15805 0 0 0 0 0 0 0 1 0 0 0 0 0
0 0 0 0 0 0 0 0 0 0 0 0 0 0
0 0 0 0 0 0 0 0 0 0 0 0 0 0
0 0 0 0 0 0 0 0 0 0 0 0 0 0
0 0 0 0 0 0 0 0 0 0 0 0 0 0
0 0 0 0 0 0 0 0 0 0 0 0 0 0
0 0 0 0 0 0 0 0 0 0 0 0 0 0
0 0 0 0 0 0 0 0 0 0 0 0 0 0
0

OTU12106 0 0 0 0 0 0 0 1 0 0 0 0 0
0 0 0 0 0 0 0 0 0 0 0 0 0 0
0 0 0 0 0 0 0 0 0 0 0 0 0 0
0 0 0 0 0 0 0 0 0 0 0 0 0 0
0 0 0 0 0 0 0 0 0 0 0 0 0 0
0 0 0 0 0 0 0 0 0 0 0 0 0 0
0 0 0 0 0 0 0 0 0 0 0 0 0 0
0 0 0 0 0 0 0 0 0 0 0 0 0 0
0
OTU15804 0 0 0 0 0 0 0 1 0 0 0 0 0
0 0 0 0 0 0 0 0 0 0 0 0 0 0
0 0 0 0 0 0 0 0 0 0 0 0 0 0
0 0 0 0 0 0 0 0 0 0 0 0 0 0
0 0 0 0 0 0 0 0 0 0 0 0 0 0
0 0 0 0 0 0 0 0 0 0 0 0 0 0
0 0 0 0 0 0 0 0 0 0 0 0 0 0
0 0 0 0 0 0 0 0 0 0 0 0 0 0
0
OTU7867 0 0 0 0 0 0 0 1 0 0 0 0 0
0 0 0 0 0 0 0 0 0 0 0 0 0 0
0 0 0 0 0 0 0 0 0 0 0 0 0 0
0 0 0 0 0 0 0 0 0 0 0 0 0 0
0 0 0 0 0 0 0 0 0 0 0 0 0 0
0 0 0 0 0 0 0 0 0 0 0 0 0 0
0 0 0 0 0 0 0 0 0 0 0 0 0 0
0 0 0 0 0 0 0 0 0 0 0 0 0 0
0
OTU15801 0 0 0 0 0 0 0 1 0 0 0 0 0
0 0 0 0 0 0 0 0 0 0 0 0 0 0
0 0 0 0 0 0 0 0 0 0 0 0 0 0
0 0 0 0 0 0 0 0 0 0 0 0 0 0
0 0 0 0 0 0 0 0 0 0 0 0 0 0
0 0 0 0 0 0 0 0 0 0 0 0 0 0

0	0	0	0	0	0	0	0	0	0	0	0	0	0
0	0	0	0	0	0	0	0	0	0	0	0	0	0
0													
OTU12103	0	0	0	0	0	0	0	2	0	0	0	0	0
0	0	0	0	0	0	0	0	0	0	0	0	0	0
0	0	0	0	0	0	0	0	0	0	0	0	0	0
0	0	0	0	0	0	0	0	0	0	0	0	0	0
0	0	0	0	0	0	0	0	0	0	0	0	0	0
0	0	0	0	0	0	0	0	0	0	0	0	0	0
0	0	0	0	0	0	0	0	0	0	0	0	0	0
0	0	0	0	0	0	0	0	0	0	0	0	0	0
0													
OTU7861	0	0	0	0	0	0	0	1	0	0	0	0	0
0	0	0	0	0	0	0	0	0	0	0	0	0	0
0	0	0	0	0	0	0	0	0	0	0	0	0	0
0	0	0	0	0	0	0	0	0	0	0	0	0	0
0	0	0	0	0	0	0	0	0	0	0	0	0	0
0	0	0	0	0	0	0	0	0	0	0	0	0	0
0	0	0	0	0	0	0	0	0	0	0	0	0	0
0	0	0	0	0	0	0	0	0	0	0	0	0	0
0													
OTU650	0	0	0	0	0	0	0	1	1	0	5	0	8
0	0	1	4	0	3	0	3	0	0	0	0	0	1
0	0	6	2	0	0	0	1	0	1	1	0	0	0
2	3	0	0	0	0	0	0	1	0	0	0	1	0
1	0	0	0	0	1	0	0	0	1	0	0	0	0
0	0	0	0	0	0	1	1	0	0	0	0	0	0
1	0	1	0	1	0	0	0	6	0	0	0	0	3
0	0	0	0	0	0	0	0	0	0	2	2	0	0
0													
OTU14302	0	0	0	0	0	0	0	1	0	0	0	0	0
0	0	0	0	0	0	0	0	0	0	0	0	0	0
0	0	0	0	0	0	0	0	0	0	0	0	0	0

0 0 0 0 0 0 0 0 0 0 0 0 0 0
0 0 0 0 0 0 0 0 0 0 0 0 0 0
0 0 0 0 0 0 0 0 0 0 1 0 0 0
0 0 0 0 0 0 1 0 0 0 0 0 0 0
0 0 0 0 0 0 0 0 0 0 2 0 0 0
0
OTU7865 0 0 0 0 0 0 0 1 0 0 0 0 0
0 0 0 0 0 0 0 0 0 0 0 0 0 0
0 0 0 0 0 0 0 0 0 0 0 0 0 0
0 0 0 0 0 0 0 0 0 0 0 0 0 0
0 0 0 0 0 0 0 0 0 0 0 0 0 0
0 0 0 0 0 0 0 0 0 0 0 0 0 0
0 0 0 0 0 0 0 0 0 0 0 0 0 0
0 0 0 0 0 0 0 0 0 0 0 0 0 0
0
OTU7864 0 0 0 0 0 0 0 1 0 0 0 0 0
0 0 0 0 0 0 0 0 0 0 0 0 0 0
0 0 0 0 0 0 0 0 0 0 0 0 0 0
0 0 0 0 0 0 0 0 0 0 0 0 0 0
0 0 0 0 0 0 0 0 0 0 0 0 0 0
0 0 0 0 0 0 0 0 0 0 0 0 0 0
0 0 0 0 0 0 0 0 0 0 0 0 0 0
0 0 0 0 0 0 0 0 0 0 0 0 0 0
0
OTU15802 0 0 0 0 0 0 0 1 0 0 0 0 0
0 0 0 0 0 0 0 0 0 0 0 0 0 0
0 0 0 0 0 0 0 0 0 0 0 0 0 0
0 0 0 0 0 0 0 0 0 0 0 0 0 0
0 0 0 0 0 0 0 0 0 0 0 0 0 0
0 0 0 0 0 0 0 0 0 0 0 0 0 0
0 0 0 0 0 0 0 0 0 0 0 0 0 0
0 0 0 0 0 0 0 0 0 0 0 0 0 0
0

OTU7863	0	0	0	0	0	0	0	1	0	0	0	0	0
0	0	0	0	0	0	0	0	0	0	0	0	0	0
0	0	0	0	0	0	0	0	0	0	0	0	0	0
0	0	0	0	0	0	0	0	0	0	0	0	0	0
0	0	0	0	0	0	0	0	0	0	0	0	0	0
0	0	0	0	0	0	0	0	0	0	0	0	0	0
0	0	0	0	0	0	0	0	0	0	0	0	0	0
0	0	0	0	0	0	0	0	0	0	0	0	0	0
0													
OTU9122	0	0	0	0	0	0	0	1	0	0	1	0	1
0	0	0	0	0	0	0	0	0	1	0	0	0	0
0	0	1	0	0	0	0	0	0	0	0	1	0	0
0	0	0	0	0	0	0	0	0	0	0	0	0	0
0	0	0	0	0	0	0	0	0	0	0	0	0	0
0	0	0	0	0	0	0	0	0	0	0	0	0	0
0	0	0	0	0	0	0	0	0	0	0	0	0	0
0	0	0	0	0	0	0	0	0	0	0	0	0	0
0													
OTU14413	0	0	0	0	0	0	0	1	0	0	0	0	0
0	0	0	0	0	0	0	0	0	0	0	0	0	0
0	0	0	0	0	0	0	0	0	0	0	0	0	0
0	0	0	0	0	0	0	0	0	0	0	0	0	0
0	0	0	0	0	0	0	0	0	0	0	0	0	0
0	0	0	0	0	0	0	0	0	0	0	0	0	0
0	0	0	0	0	0	0	0	0	0	0	0	0	0
0	0	0	0	0	0	0	0	0	0	0	0	0	0
0													
OTU12109	0	0	0	0	0	0	0	1	0	0	0	0	0
0	0	0	0	0	0	0	0	0	0	0	0	0	0
0	0	0	0	0	0	0	0	0	0	0	0	0	0
0	0	0	0	0	0	0	0	0	0	0	0	0	0
0	0	0	0	0	0	0	0	0	0	0	0	0	0
0	0	0	0	0	0	0	0	0	0	0	0	0	0

0	0	0	0	0	0	0	0	0	0	0	0	0	0
0	0	0	0	0	0	0	0	0	0	0	0	0	0
0													
OTU14523	0	0	0	0	0	0	0	1	0	0	0	0	0
0	0	0	0	0	0	0	0	0	0	0	0	0	0
0	0	0	0	0	0	0	0	0	0	0	0	0	0
0	0	0	0	0	0	0	0	0	0	0	0	0	0
0	0	0	0	0	0	0	0	0	0	0	0	0	0
0	0	0	0	0	0	0	0	0	0	0	0	0	0
0	0	0	0	0	0	0	0	0	0	0	0	0	0
0	0	0	0	0	0	0	0	0	0	0	0	0	0
0													
OTU804	0	0	0	0	0	0	0	1	2	0	0	0	0
0	0	0	0	0	0	0	0	0	0	0	0	0	0
0	0	0	0	0	0	0	0	0	1	0	0	0	0
0	0	0	0	0	0	0	0	0	0	0	0	0	0
0	0	0	0	0	0	0	0	0	0	0	0	0	0
0	0	0	0	0	0	0	0	0	2	0	0	0	1
0	0	0	2	0	0	0	0	0	0	0	2	0	0
0	3	3	1	1	2	3	0	1	1	0	0	0	0
0													
OTU14939	0	0	0	0	0	0	0	3	0	0	0	0	0
0	0	0	0	0	0	0	0	0	0	0	0	1	0
0	0	0	0	0	0	0	0	0	0	0	0	0	0
0	0	0	0	0	0	0	1	0	0	0	0	0	0
0	0	0	0	0	0	0	0	0	0	0	1	0	0
0	0	0	0	0	0	0	0	0	0	2	0	0	0
0	0	0	0	0	0	0	0	0	0	0	0	0	0
0	0	44	10	31	70	41	4	20	7	0	2	0	25
3													
OTU7873	0	0	0	0	0	0	0	1	0	0	0	0	0
0	0	0	0	0	0	0	0	0	0	0	0	0	0
0	0	0	0	0	0	0	0	0	0	0	0	0	0

0	0	0	0	0	0	0	0	0	0	0	0	0	0
0	0	0	0	0	0	0	0	0	0	0	0	0	0
0	0	0	0	0	0	0	0	0	0	0	0	0	0
0	0	0	0	0	0	0	0	0	0	0	0	0	0
0	0	0	0	0	0	0	0	0	0	0	0	0	0
0													
OTU2218	0	0	0	0	0	0	0	2	0	0	0	0	1
0	0	0	0	0	0	0	0	0	0	0	0	1	0
0	0	0	0	0	0	0	0	0	0	0	0	0	0
0	0	0	0	0	0	0	0	0	0	0	0	0	0
0	0	0	0	0	0	0	0	0	0	0	0	0	0
0	0	2	0	0	0	0	0	0	1	0	0	0	0
0	0	0	0	0	0	0	0	0	0	0	0	0	0
0	0	0	0	0	0	0	0	0	0	1	0	0	0
0													
OTU12111	0	0	0	0	0	0	0	1	0	0	0	0	0
0	0	0	0	0	0	0	0	0	0	0	0	0	0
0	0	0	0	0	0	0	0	0	0	0	0	0	0
0	0	0	0	0	0	0	0	0	0	0	0	0	0
0	0	0	0	0	0	0	0	0	0	0	0	0	0
0	0	0	0	0	0	0	0	0	0	0	0	0	0
0	0	0	0	0	0	0	0	0	0	0	0	0	0
0	0	0	0	0	0	0	0	0	0	0	0	0	0
0													
OTU12099	0	0	0	0	0	0	0	2	0	0	0	0	0
0	0	0	0	0	0	0	0	0	0	0	0	1	0
0	0	0	0	0	0	0	0	1	0	0	0	0	0
0	0	0	0	0	0	0	0	0	0	0	0	0	0
0	0	0	0	0	0	0	0	0	0	0	0	0	0
0	0	0	0	2	0	0	0	0	0	0	0	0	0
0	0	0	0	0	0	0	0	0	0	0	0	0	0
0	0	0	0	0	0	0	0	0	0	0	0	0	0
0													

OTU15806 0 0 0 0 0 0 0 1 0 0 0 0 0
0 0 0 0 0 0 0 0 0 0 0 0 0 0
0 0 0 0 0 0 0 0 0 0 0 0 0 0
0 0 0 0 0 0 0 0 0 0 0 0 0 0
0 0 0 0 0 0 0 0 0 0 0 0 0 0
0 0 0 0 0 0 0 0 0 0 0 0 0 0
0 0 0 0 0 0 0 0 0 0 0 0 0 0
0 0 0 0 0 0 0 0 0 0 0 0 0 0
0
OTU7868 0 0 0 0 0 0 0 1 0 0 0 0 0
0 0 0 0 0 0 0 0 0 0 0 0 0 0
0 0 0 0 0 0 0 0 0 0 0 0 0 0
0 0 0 0 0 0 0 0 0 0 0 0 0 0
0 0 0 0 0 0 0 0 0 0 0 0 0 0
0 0 0 0 0 0 0 0 0 0 0 0 0 0
0 0 0 0 0 0 0 0 0 0 0 0 0 0
0 0 0 0 0 0 0 0 0 0 0 0 0 0
0
OTU847 0 0 0 0 0 0 0 1 1 0 0 0 0
0 0 0 0 0 0 0 0 0 0 0 0 0 0
1 0 0 0 1 0 0 0 0 0 0 0 1 0
0 0 0 0 0 2 0 0 0 0 0 0 0 0
0 2 0 3 0 0 0 0 0 0 0 1 6 1
0 0 0 0 0 0 0 0 0 0 0 0 0 0
0 1 0 0 0 0 0 0 0 0 0 0 1 0
0 1 0 0 0 0 0 0 0 0 0 0 0 2
1
OTU14524 0 0 0 0 0 0 0 1 0 0 0 0 0
0 0 0 0 0 0 0 0 0 0 0 0 0 0
0 0 0 0 0 0 0 0 0 0 0 0 0 0
0 0 0 0 0 0 0 0 0 0 0 0 0 0
0 0 0 0 0 0 0 0 0 0 0 0 0 0
0 0 0 0 0 0 0 0 0 0 0 0 0 0

0	0	0	0	0	0	0	0	0	0	0	0	0	0
0	0	0	0	0	0	0	0	0	0	0	0	0	0
0													
OTU9581	0	0	0	0	0	0	0	1	0	0	0	0	0
0	0	0	0	0	0	0	0	0	0	0	0	0	1
0	0	0	0	0	0	0	0	0	0	0	0	0	0
0	0	0	0	0	0	0	0	0	0	0	0	0	0
0	0	0	0	0	0	0	0	0	0	0	0	0	0
0	0	0	0	0	0	0	0	0	0	0	0	0	0
0	0	0	0	0	0	0	0	0	0	0	0	0	0
0	0	0	0	0	0	0	0	0	0	0	0	0	0
0													
OTU14520	0	0	0	0	0	0	0	1	0	0	0	0	0
0	0	0	0	0	0	0	0	0	0	0	0	0	0
0	0	0	0	0	0	0	0	0	0	0	0	0	0
0	0	0	0	0	0	0	0	0	0	0	0	0	0
0	0	0	0	0	0	0	0	0	0	0	0	0	0
0	0	0	0	0	0	0	0	0	0	0	0	0	0
0	0	0	0	0	0	0	0	0	0	0	0	0	0
0	0	0	0	0	0	0	0	0	0	0	0	0	0
0													
OTU7870	0	0	0	0	0	0	0	1	0	0	0	0	0
0	0	0	0	0	0	0	0	0	0	0	0	0	0
0	0	0	0	0	0	0	0	0	0	0	0	0	0
0	0	0	0	0	0	0	0	0	0	0	0	0	0
0	0	0	0	0	0	0	0	0	0	0	0	0	0
0	0	0	0	0	0	0	0	0	0	0	0	0	0
0	0	0	0	0	0	0	0	0	0	0	0	0	0
0	0	0	0	0	0	0	0	0	0	0	0	0	0
0													
OTU14521	0	0	0	0	0	0	0	1	0	0	0	0	0
0	0	0	0	0	0	0	0	0	0	0	0	0	0
0	0	0	0	0	0	0	0	0	0	0	0	0	0

0	0	0	0	0	0	0	0	0	0	0	0	0	0
0	0	0	0	0	0	0	0	0	0	0	0	0	0
0	0	0	0	0	0	0	0	0	0	0	0	0	0
0	0	0	0	0	0	0	0	0	0	0	0	0	0
0	0	0	0	0	0	0	0	0	0	0	0	0	0
0													
OTU10483	0	0	0	0	0	0	0	1	0	0	0	0	0
0	0	0	0	0	0	0	0	0	0	0	0	0	0
0	0	0	0	0	0	0	0	0	0	0	0	0	0
0	0	0	0	0	0	0	0	0	0	0	0	0	0
0	0	0	0	0	0	0	0	0	0	0	0	1	0
0	0	0	0	0	0	0	0	0	0	0	0	0	0
0	0	0	0	0	0	0	0	0	0	0	0	0	0
0	0	0	0	0	0	0	0	0	0	0	0	0	0
0													
OTU14522	0	0	0	0	0	0	0	1	0	0	0	0	0
0	0	0	0	0	0	0	0	0	0	0	0	0	0
0	0	0	0	0	0	0	0	0	0	0	0	0	0
0	1	0	0	0	0	0	0	0	0	0	0	0	0
0	0	0	0	0	0	0	0	0	0	0	0	0	0
0	0	0	0	0	0	0	0	0	0	0	0	0	0
0	0	0	0	0	0	0	0	0	0	0	0	0	0
0	0	0	0	0	0	0	0	0	0	0	0	0	0
0													
OTU5687	0	0	0	0	0	0	0	2	0	3	0	1	0
0	0	3	0	1	0	0	1	0	0	0	0	0	0
1	0	0	0	0	4	0	0	0	0	0	0	2	1
0	1	1	1	0	0	0	0	2	0	0	0	1	2
1	0	0	1	0	1	0	2	0	0	2	0	0	0
1	4	0	0	3	0	0	3	1	1	0	2	0	1
1	0	0	0	0	0	3	0	0	0	1	0	0	0
0	0	0	0	3	1	2	0	0	0	0	0	0	0
0													

OTU5582	0	0	0	0	0	0	0	1	0	0	0	0	0
0	0	0	0	0	0	0	0	0	0	0	0	0	0
0	0	0	0	0	0	0	0	0	0	0	0	0	0
0	1	0	0	0	0	0	0	0	0	0	0	0	0
0	0	0	0	0	0	0	0	0	0	0	0	0	0
0	0	0	0	0	0	0	0	0	0	0	0	0	0
0	0	0	0	0	0	0	0	0	0	0	0	0	0
0	0	0	0	0	0	0	0	0	0	0	0	0	0
0													
OTU7898	0	0	0	0	0	0	0	1	0	0	0	0	0
0	0	0	0	0	0	0	0	0	0	0	0	0	0
0	0	0	0	0	0	0	0	0	0	0	0	0	0
0	0	0	0	0	0	0	0	0	0	0	0	0	0
0	0	0	0	0	0	0	0	0	0	0	0	0	0
0	0	0	0	0	0	0	0	0	0	0	0	0	0
0	0	0	0	0	0	0	0	0	0	0	0	0	0
0	0	0	0	0	0	0	0	0	0	0	0	0	0
0													
OTU13546	0	0	0	0	0	0	0	1	0	0	0	0	1
0	0	0	0	0	1	0	0	0	3	0	0	0	1
0	0	1	0	0	0	0	0	0	0	0	1	0	0
0	0	0	0	0	0	0	0	0	0	0	0	0	0
0	0	0	0	0	0	0	0	0	0	0	0	0	0
0	0	0	0	0	0	0	0	1	0	1	0	0	0
0	0	0	0	0	0	0	0	0	0	0	0	0	0
0	0	0	0	0	0	0	0	0	0	0	1	0	0
0													
OTU15330	0	0	0	0	0	0	0	1	1	0	0	0	0
0	0	1	0	0	0	0	0	0	1	0	0	0	0
0	0	2	1	0	0	0	0	0	0	0	0	0	0
0	0	0	0	0	0	0	0	0	0	0	0	0	0
0	0	0	0	0	0	0	0	0	0	0	0	0	0
1	0	0	0	0	0	0	0	0	0	0	0	0	1

	0	0	0	0	0	0	0	0	0	0	1	0	0	0
	0	0	0	0	0	0	0	0	0	0	0	0	0	0
	0													
OTU12098	0	0	0	0	0	0	0	1	0	0	0	0	0
	0	0	0	0	0	0	0	0	0	0	0	0	0	0
	0	0	0	0	0	0	0	0	0	0	0	0	0	0
	0	0	0	0	0	0	0	0	0	0	0	0	0	0
	0	0	0	0	0	0	0	0	0	0	0	0	0	0
	0	0	0	0	0	0	0	0	0	0	0	0	0	0
	0	0	0	0	0	0	0	0	0	0	0	0	0	0
	0	0	0	0	0	0	0	0	0	0	0	0	0	0
	0													
OTU12097	0	0	0	0	0	0	0	1	0	0	0	0	0
	0	0	0	0	0	0	0	0	0	0	0	0	0	0
	0	0	0	0	0	0	0	0	0	0	0	0	0	0
	0	0	0	0	0	0	0	0	0	0	0	0	0	0
	0	0	0	0	0	0	0	0	0	0	0	0	0	0
	0	0	0	0	0	0	0	0	0	0	0	0	0	0
	0	0	0	0	0	0	0	0	0	0	0	0	0	0
	0	0	0	0	0	0	0	0	0	0	0	0	0	0
	0													
OTU392	0	0	0	0	0	0	0	2	0	6	0	0	0
	0	1	0	0	3	11	14	3	0	0	5	1	1	0
	0	0	0	0	0	6	0	0	0	8	0	0	2	0
	0	0	0	0	2	0	0	0	0	0	2	0	0	0
	0	0	0	0	0	0	0	0	1	0	0	3	0	0
	0	6	2	0	0	0	0	0	0	0	0	0	0	0
	0	0	0	2	0	0	0	0	0	0	0	0	6	0
	0	1	0	5	0	2	0	1	2	3	0	0	0	1
	0													
OTU12096	0	0	0	0	0	0	0	1	0	0	0	0	0
	0	0	0	0	0	0	0	0	0	0	0	0	0	0
	0	0	0	0	0	0	0	0	0	0	0	0	0	0

0	0	0	0	0	0	0	0	0	0	0	0	0	0
0	0	0	0	0	0	0	0	0	0	0	0	0	0
0	0	0	0	0	0	0	0	0	0	0	0	0	0
0	0	0	0	0	0	0	0	0	0	0	0	0	0
0	0	0	0	0	0	0	0	0	0	0	0	0	0
0													
OTU15833	0	0	0	0	0	0	0	1	0	0	0	0	0
0	0	0	0	0	0	0	0	0	0	0	0	0	0
0	0	0	0	0	0	0	0	0	0	0	0	0	0
0	0	0	0	0	0	0	0	0	0	0	0	0	0
0	0	0	0	0	0	0	0	0	0	0	0	0	0
0	0	0	0	0	0	0	0	0	0	0	0	0	0
0	0	0	0	0	0	0	0	0	0	0	0	0	0
0	0	0	0	0	0	0	0	0	0	0	0	0	0
0													
OTU4282	0	0	0	0	0	0	0	1	0	0	1	1	0
0	1	0	0	0	0	0	0	0	0	0	0	0	1
0	0	0	0	0	1	0	0	0	0	0	0	0	0
0	0	0	0	0	0	0	0	0	0	0	0	0	1
0	0	0	0	0	0	0	0	0	0	0	0	0	0
0	0	0	0	0	0	0	0	0	0	0	0	0	0
0	0	0	0	1	0	0	0	0	0	0	0	0	1
0	0	0	0	0	0	0	0	0	0	0	0	0	0
0													
OTU14195	0	0	0	0	0	0	0	1	0	0	0	0	0
0	0	0	0	0	0	0	0	0	0	0	0	0	0
0	0	0	0	0	0	0	0	0	0	0	0	0	0
0	0	0	0	0	0	0	0	0	0	0	0	0	0
0	0	0	0	0	0	0	0	0	0	0	0	0	0
0	0	0	0	1	0	0	0	0	0	0	0	0	0
0	0	0	0	0	0	0	0	0	0	0	0	0	0
0	0	0	0	0	0	0	0	0	0	0	0	0	0
0													

OTU7902 0 0 0 0 0 0 0 1 0 0 0 0 0
0 0 0 0 0 0 0 0 0 0 0 0 0 0
0 0 0 0 0 0 0 0 0 0 0 0 0 0
0 0 0 0 0 0 0 0 0 0 0 0 0 0
0 0 0 0 0 0 0 0 0 0 0 0 0 0
0 0 0 0 0 0 0 0 0 0 0 0 0 0
0 0 0 0 0 0 0 0 0 0 0 0 0 0
0 0 0 0 0 0 0 0 0 0 0 0 0 0
0
OTU14525 0 0 0 0 0 0 0 1 0 0 0 0 0
0 0 0 0 0 1 0 0 0 1 0 0 0 0
0 0 0 0 0 0 0 0 0 0 0 0 0 0
0 0 0 0 0 0 0 0 0 0 0 0 0 0
0 0 0 0 0 0 0 0 0 0 0 0 0 0
0 0 0 0 0 0 0 0 0 0 0 0 0 0
0 0 0 0 0 0 0 0 0 0 0 0 0 0
0 0 0 0 0 0 0 0 0 0 0 0 0 0
0
OTU13797 0 0 0 0 0 0 0 1 0 0 0 0 0
0 0 0 0 0 0 0 0 0 0 0 0 0 0
0 0 0 0 0 0 0 0 0 0 0 0 0 0
0 0 0 0 0 0 0 0 0 0 0 0 0 0
0 0 0 0 0 0 0 0 0 0 0 0 0 0
0 0 0 0 0 0 0 0 0 0 0 0 0 0
0 0 0 0 0 1 0 0 0 0 0 0 0 0
0 0 0 0 0 0 0 0 0 0 0 0 0 0
0
OTU7901 0 0 0 0 0 0 0 1 0 0 0 0 0
0 0 0 0 0 0 0 0 0 0 0 0 0 0
0 0 0 0 0 0 0 0 0 0 0 0 0 0
0 0 0 0 0 0 0 0 0 0 0 0 0 0
0 0 0 0 0 0 0 0 0 0 0 0 0 0
0 0 0 0 0 0 0 0 0 0 0 0 0 0

0	0	0	0	0	0	0	0	0	0	0	0	0	0
0	0	0	0	0	0	0	0	0	0	0	0	0	0
0													
OTU14527	0	0	0	0	0	0	0	1	0	0	0	0	0
0	0	0	0	0	0	0	0	0	0	0	0	0	0
0	0	0	0	0	0	0	0	0	0	0	0	0	0
0	0	0	0	0	0	0	0	0	0	0	0	0	0
0	0	0	0	0	0	0	0	0	0	0	0	0	0
0	0	0	0	0	0	0	0	0	0	0	0	0	0
0	0	0	0	0	0	0	0	0	0	0	0	0	0
0	0	0	0	0	0	0	0	0	0	0	0	0	0
0													
OTU12095	0	0	0	0	0	0	0	1	0	0	0	0	0
0	0	0	0	0	0	0	0	0	0	0	0	0	0
0	0	0	0	0	0	0	0	0	0	0	0	0	0
0	0	0	0	0	0	0	0	0	0	0	0	0	0
0	0	0	0	0	0	0	0	0	0	0	0	0	0
0	0	0	0	0	0	0	0	0	0	0	0	0	0
0	0	0	0	0	0	0	0	0	0	0	0	0	0
0	0	0	0	0	0	0	0	0	0	0	0	0	0
0													
OTU15830	0	0	0	0	0	0	0	1	0	0	0	0	0
0	0	0	0	0	0	0	0	0	0	0	0	0	0
0	0	0	0	0	0	0	0	0	0	0	0	0	0
0	0	0	0	0	0	0	0	0	0	0	0	0	0
0	0	0	0	0	0	0	0	0	0	0	0	0	0
0	0	0	0	0	0	0	0	0	0	0	0	0	0
0	0	0	0	0	0	0	0	0	0	0	0	0	0
0	0	0	0	0	0	0	0	0	0	0	0	0	0
0													
OTU14528	0	0	0	0	0	0	0	2	0	0	0	0	0
0	0	0	0	0	0	0	0	0	0	0	0	0	0
0	0	0	0	0	0	0	0	0	0	0	0	0	0

0	0	0	0	0	0	0	1	0	0	0	0	0	0
0	1	0	0	0	0	0	0	0	0	0	0	1	0
0	0	0	0	0	0	0	0	0	0	0	0	0	0
0	0	0	0	0	0	0	0	0	0	0	0	0	0
0	0	0	0	0	0	0	0	0	0	0	0	0	0
0													
OTU12094	0	0	0	0	0	0	0	1	0	0	0	0	0
0	0	0	0	0	0	0	0	0	0	0	0	0	0
0	0	0	0	0	0	0	0	0	0	0	0	0	0
0	0	0	0	0	0	0	0	0	0	0	0	0	0
0	0	0	0	0	0	0	0	0	0	0	0	1	0
0	0	0	0	0	0	0	0	0	0	0	0	0	0
0	0	0	0	0	0	0	0	0	0	0	0	0	0
0	0	0	0	0	0	0	0	0	0	0	0	0	0
0													
OTU7899	0	0	0	0	0	0	0	1	0	0	0	0	0
0	0	0	0	0	0	0	0	0	0	0	0	0	0
0	0	0	0	0	0	0	0	0	0	0	0	0	0
0	0	0	0	0	0	0	0	0	0	0	0	0	0
0	0	0	0	0	0	0	0	0	0	0	0	0	0
0	0	0	0	0	0	0	0	0	0	0	0	0	0
0	0	0	0	0	0	0	0	0	0	0	0	0	0
0	0	0	0	0	0	0	0	0	0	0	0	0	0
0													
OTU15832	0	0	0	0	0	0	0	1	0	0	0	0	0
0	0	0	0	0	0	0	0	0	0	0	0	0	0
0	0	0	0	0	0	0	0	0	0	0	0	0	0
0	0	0	0	0	0	0	0	0	0	0	0	0	0
0	0	0	0	0	0	0	0	0	0	0	0	0	0
0	0	0	0	0	0	0	0	0	0	0	0	0	0
0	0	0	0	0	0	0	0	0	0	0	0	0	0
0	0	0	0	0	0	0	0	0	0	0	0	0	0
0													

OTU15831	0	0	0	0	0	0	0	1	0	0	0	0	0
0	0	0	0	0	0	0	0	0	0	0	0	0	0
0	0	0	0	0	0	0	0	0	0	0	0	0	0
0	0	0	0	0	0	0	0	0	0	0	0	0	0
0	0	0	0	0	0	0	0	0	0	0	0	0	0
0	0	0	0	0	0	0	0	0	0	0	0	0	0
0	0	0	0	0	0	0	0	0	0	0	0	0	0
0	0	0	0	0	0	0	0	0	0	0	0	0	0
0													
OTU7900	0	0	0	0	0	0	0	1	0	0	0	0	0
0	0	0	0	0	0	0	0	0	0	0	0	0	0
0	0	0	0	0	0	0	0	0	0	0	0	0	0
0	0	0	0	0	0	0	0	0	0	0	0	0	0
0	0	0	0	0	0	0	0	0	0	0	0	0	0
0	0	0	0	0	0	0	0	0	0	0	0	0	0
0	0	0	0	0	0	0	0	0	0	0	0	0	0
0	0	0	0	0	0	0	0	0	0	0	0	0	0
0													
OTU7889	0	0	0	0	0	0	0	1	0	0	0	0	0
0	0	0	0	0	0	0	0	0	0	0	0	0	0
0	0	0	0	0	0	0	0	0	0	0	0	0	0
0	0	0	0	0	0	0	0	0	0	0	0	0	0
0	0	0	0	0	0	0	0	0	0	0	0	0	0
0	0	0	0	0	0	0	0	0	0	0	0	0	0
0	0	0	0	0	0	0	0	0	0	0	0	0	0
0	0	0	0	0	0	0	0	0	0	0	0	0	0
0													
OTU513	0	0	0	0	0	0	0	1	0	1	0	0	0
0	0	0	0	0	0	0	0	0	0	0	1	0	0
1	0	0	0	0	0	0	0	0	0	0	0	0	0
0	0	0	0	0	0	0	0	0	0	0	0	0	0
0	0	0	0	0	0	0	0	0	0	0	0	0	0
0	4	0	0	0	0	0	2	0	1	2	0	0	0

1	0	0	0	0	0	0	1	0	0	0	7	5	0
0	8	3	14	14	57	26	1	16	2	0	0	0	0
3													
OTU12102	0	0	0	0	0	0	0	1	0	0	0	0	0
0	0	0	0	0	0	0	0	0	0	0	0	0	0
0	0	0	0	0	0	0	0	0	0	0	0	0	0
0	0	0	0	0	0	0	0	0	0	0	0	0	0
0	0	0	0	0	0	0	0	0	0	0	0	0	0
0	0	0	0	0	0	0	0	0	0	0	0	0	0
0	0	0	0	0	0	0	0	0	0	0	0	0	0
0	0	0	0	0	0	0	0	0	0	0	0	0	0
0													
OTU14516	0	0	0	0	0	0	0	1	0	0	0	0	0
0	0	0	0	0	0	0	0	0	0	0	0	0	0
0	0	0	0	0	0	0	0	0	0	0	0	0	0
0	0	0	0	0	0	0	0	0	0	0	0	0	0
0	0	0	0	0	0	0	0	0	0	0	0	0	0
0	0	0	0	0	0	0	0	0	0	0	0	0	0
0	0	0	0	0	0	0	0	0	0	0	0	0	0
0	0	0	0	0	0	0	0	0	0	0	0	0	0
0													
OTU7891	0	0	0	0	0	0	0	1	0	0	0	0	0
0	0	0	0	0	0	0	0	0	0	0	0	0	0
0	0	0	0	0	0	0	0	0	0	0	0	0	0
0	0	0	0	0	0	0	0	0	0	0	0	0	0
0	0	0	0	0	0	0	0	0	0	0	0	0	0
0	0	0	0	0	0	0	0	0	0	0	0	0	0
0	0	0	0	0	0	0	0	0	0	0	0	0	0
0	0	0	0	0	0	0	0	0	0	0	0	0	0
0													
OTU12100	0	0	0	0	0	0	0	1	0	0	0	0	0
0	0	0	0	0	0	0	0	0	0	0	0	0	0
0	0	0	0	0	0	0	0	0	0	0	0	0	0

0	0	0	0	0	0	0	0	0	0	0	0	0	0
0	0	0	0	0	0	0	0	0	0	0	0	0	0
0	0	0	0	0	0	0	0	0	0	0	0	0	0
0	0	0	0	0	0	0	0	0	0	0	0	0	0
0	0	0	0	0	0	0	0	0	0	0	0	0	0
0													
OTU7887	0	0	0	0	0	0	0	1	0	0	0	0	0
0	0	0	0	0	0	0	0	0	0	0	0	0	0
0	0	0	0	0	0	0	0	0	0	0	0	0	0
0	0	0	0	0	0	0	0	0	0	0	0	0	0
0	0	0	0	0	0	0	0	0	0	0	0	0	0
0	0	0	0	0	0	0	0	0	0	0	0	0	0
0	0	0	0	0	0	0	0	0	0	0	0	0	0
0	0	0	0	0	0	0	0	0	0	0	0	0	0
0													
OTU14515	0	0	0	0	0	0	0	1	0	0	0	0	0
0	0	0	0	0	0	0	0	0	0	0	0	0	0
0	0	0	0	0	0	0	0	0	0	0	0	0	0
0	0	0	0	0	0	0	0	0	0	0	0	0	0
0	0	0	0	0	0	0	0	0	0	0	0	0	0
0	0	0	0	0	0	0	0	0	0	0	0	0	0
0	0	0	0	0	0	0	0	0	0	0	0	0	0
0	0	0	0	0	0	0	0	0	0	0	0	0	0
0													
OTU13136	0	0	0	0	0	0	0	1	0	0	0	0	0
0	0	0	0	0	0	0	0	0	0	0	0	0	0
0	0	0	0	0	0	1	0	0	0	0	0	0	0
0	0	0	0	0	0	0	0	0	0	0	0	0	0
0	0	0	0	0	0	0	0	0	0	0	0	0	0
0	0	0	0	0	0	0	0	0	0	0	0	0	0
0	0	0	0	0	0	0	0	0	0	0	0	0	0
0	0	0	0	0	0	0	0	0	0	0	0	0	0
0													

OTU7886 0 0 0 0 0 0 0 1 0 0 0 0 0
0 0 0 0 0 0 0 0 0 0 0 0 0 0
0 0 0 0 0 0 0 0 0 0 0 0 0 0
0 0 0 0 0 0 0 0 0 0 0 0 0 0
0 0 0 0 0 0 0 0 0 0 0 0 0 0
0 0 0 0 0 0 0 0 0 0 0 0 0 0
0 0 0 0 0 0 0 0 0 0 0 0 0 0
0 0 0 0 0 0 0 0 0 0 0 0 0 0
0
OTU1119 0 0 0 0 0 0 0 1 0 0 0 0 0
0 0 0 0 0 0 0 0 0 0 0 0 0 0
0 0 0 0 0 0 0 0 0 0 0 0 0 0
0 0 0 0 0 0 0 0 0 0 0 0 0 0
0 0 0 0 0 0 0 0 0 0 0 0 0 0
0 0 0 0 1 0 0 0 0 0 0 0 0 0
0 0 0 0 0 0 0 0 0 0 0 0 0 0
0 0 3 0 2 1 1 0 0 1 0 0 0 0
4
OTU12587 0 0 0 0 0 0 0 1 0 0 0 0 0
0 0 0 0 0 0 0 0 0 0 0 0 0 0
0 0 0 0 0 0 0 0 0 0 0 0 0 0
0 0 0 0 0 0 0 1 0 0 0 0 0 0
0 0 0 0 0 0 0 0 0 0 1 0 0 0
0 0 0 0 0 0 0 0 0 1 0 0 0 0
0 0 0 0 0 0 0 0 0 0 0 0 0 0
0 0 0 0 0 0 0 0 0 0 0 0 0 0
0
OTU3247 0 0 0 0 0 0 0 1 0 0 0 0 0
0 0 0 0 0 0 0 0 0 0 0 0 0 0
0 0 0 0 0 0 0 0 0 0 0 0 0 0
0 0 0 0 0 0 0 0 0 0 0 0 0 0
0 0 0 0 0 0 0 0 0 0 0 0 0 0
0 0 0 0 0 0 0 0 0 0 0 0 0 1

	0	0	0	1	1	0	0	0	0	0	0	0	0	0
	0	0	0	0	0	0	0	0	0	0	0	0	0	0
	0													
OTU1470	0	0	0	0	0	0	0	1	0	0	0	0	0
	0	1	1	0	0	0	0	0	0	1	0	0	0	0
	0	0	0	0	0	2	0	0	3	0	0	1	0	0
	0	0	0	0	0	0	0	0	0	0	0	0	0	0
	0	0	0	0	0	0	0	0	0	0	0	0	0	0
	0	0	0	1	0	0	0	0	0	0	0	0	1	0
	0	0	0	0	0	0	0	0	0	0	0	0	0	1
	0	0	0	0	0	0	0	0	0	0	0	0	1	0
	0													
OTU15837	0	0	0	0	0	0	0	1	0	0	0	0	0
	0	0	0	0	0	0	0	0	0	0	0	0	0	0
	0	0	0	0	0	0	0	0	0	0	0	0	0	0
	0	0	0	0	0	0	0	0	0	0	0	0	0	0
	0	0	0	0	0	0	0	0	0	0	0	0	0	0
	0	0	0	0	0	0	0	0	0	0	0	0	0	0
	0	0	0	0	0	0	0	0	0	0	0	0	0	0
	0	0	0	0	0	0	0	0	0	0	0	0	0	0
	0													
OTU4113	0	0	0	0	0	0	0	1	0	0	0	0	1
	1	0	0	0	1	0	0	0	0	0	0	0	0	0
	0	0	0	0	0	0	0	0	0	0	0	0	0	0
	0	0	0	0	0	0	0	0	0	0	1	0	0	0
	0	0	0	0	0	0	0	0	0	0	0	0	0	0
	0	0	0	0	0	2	2	0	1	0	0	0	0	0
	1	1	0	0	0	0	0	0	0	1	0	0	0	1
	0	0	0	0	0	0	0	0	0	0	0	0	0	0
	0													
OTU15838	0	0	0	0	0	0	0	1	0	0	0	0	0
	0	0	0	0	0	0	0	0	0	0	0	0	0	0
	0	0	0	0	0	0	0	0	0	0	0	0	0	0

0 0 0 0 0 0 0 0 0 0 0 0 0 0
0 0 0 0 0 0 0 0 0 0 0 0 0 0
0 0 0 0 0 0 0 0 0 0 0 0 0 0
0 0 0 0 0 0 0 0 0 0 0 0 0 0
0 0 0 0 0 0 0 0 0 0 0 0 0 0
0
OTU14519 0 0 0 0 0 0 0 1 0 0 0 0 0
0 0 0 0 0 0 0 0 0 0 0 0 0 0
0 0 0 0 0 0 0 0 0 0 0 0 0 0
0 0 0 0 0 0 0 0 0 0 0 0 0 0
0 0 0 0 0 0 0 0 0 0 0 0 0 0
0 0 0 0 0 0 0 0 0 0 0 0 0 0
0 0 0 0 0 0 0 0 0 0 0 0 0 0
0 0 0 0 0 0 0 0 0 0 0 0 0 0
0
OTU2878 0 0 0 0 0 0 0 1 0 0 0 1 0
0 0 0 0 0 0 0 0 0 0 0 0 0 2
0 0 0 0 0 0 1 0 0 1 0 0 0 0
0 0 0 1 0 0 0 0 0 0 1 0 0 0
0 0 0 0 0 0 0 0 0 0 0 2 0 0
0 0 0 0 0 0 0 0 0 2 1 0 1 0
0 0 0 0 0 0 2 0 0 0 0 0 1 0
0 0 0 0 2 0 0 0 1 1 0 0 0 0
0
OTU7894 0 0 0 0 0 0 0 1 0 0 0 0 0
0 0 0 0 0 0 0 0 0 0 0 0 0 0
0 0 0 0 0 0 0 0 0 0 0 0 0 0
0 0 0 0 0 0 0 0 0 0 0 0 0 0
0 0 0 0 0 0 0 0 0 0 0 0 0 0
0 0 0 0 0 0 0 0 0 0 0 0 0 0
0 0 0 0 0 0 0 0 0 0 0 0 0 0
0 0 0 0 0 0 0 0 0 0 0 0 0 0
0

OTU7893 0 0 0 0 0 0 0 1 0 0 0 0 0
0 0 0 0 0 0 0 0 0 0 0 0 0 0
0 0 0 0 0 0 0 0 0 0 0 0 0 0
0 0 0 0 0 0 0 0 0 0 0 0 0 0
0 0 0 0 0 0 0 0 0 0 0 0 0 0
0 0 0 0 0 0 0 0 0 0 0 0 0 0
0 0 0 0 0 0 0 0 0 0 0 0 0 0
0 0 0 0 0 0 0 0 0 0 0 0 0 0
0
OTU4360 0 0 0 0 0 0 0 1 3 1 0 0 0
0 0 0 0 0 0 4 0 0 0 0 0 0 0
2 0 0 0 0 0 0 0 0 0 0 0 0 0
1 0 0 0 0 0 0 0 0 0 0 0 0 0
0 0 0 0 0 0 0 0 0 0 0 0 0 0
0 0 0 0 0 0 0 0 0 0 0 0 0 1
0 0 0 1 0 3 0 1 0 0 0 0 0 0
0 0 0 0 0 0 0 0 0 0 0 0 0 0
0
OTU14538 0 0 0 0 0 0 0 1 1 0 0 0 0
0 0 0 0 0 0 0 0 0 0 0 0 0 0
0 0 0 0 0 0 0 0 0 0 0 0 0 0
0 0 0 0 0 0 0 0 0 0 0 0 0 0
0 0 0 0 0 0 0 0 0 0 0 0 0 0
0 0 0 0 0 0 0 0 0 0 0 0 0 0
0 0 0 0 0 0 0 0 0 0 0 0 0 0
0 0 0 0 0 0 0 0 0 0 0 0 0 0
0
OTU15821 0 0 0 0 0 0 0 1 0 0 0 0 0
0 0 0 0 0 0 0 0 0 0 0 0 0 0
0 0 0 0 0 0 0 0 0 0 0 0 0 0
0 0 0 0 0 0 0 0 0 0 0 0 0 0
0 0 0 0 0 0 0 0 0 0 0 0 0 0
0 0 0 0 0 0 0 0 0 0 0 0 0 0

0	0	0	0	0	0	0	0	0	0	0	0	0	0
0	0	0	0	0	0	0	0	0	0	0	0	0	0
0													
OTU15824	0	0	0	0	0	0	0	1	0	0	0	0	0
0	0	0	0	0	0	0	0	0	0	0	0	0	0
0	0	0	0	0	0	0	0	0	0	0	0	0	0
0	0	0	0	0	0	0	0	0	0	0	0	0	0
0	0	0	0	0	0	0	0	0	0	0	0	0	0
0	0	0	0	0	0	0	0	0	0	0	0	0	0
0	0	0	0	0	0	0	0	0	0	0	0	0	0
0	0	0	0	0	0	0	0	0	0	0	0	0	0
0													
OTU8016	0	0	0	0	0	0	0	1	0	0	0	0	1
0	0	0	0	1	0	0	0	0	0	0	0	0	0
0	0	0	0	0	0	0	0	0	0	0	0	0	0
0	0	0	0	0	1	0	0	0	0	0	0	0	1
0	0	0	0	0	0	0	0	0	0	0	0	0	0
0	0	0	0	0	0	0	0	0	0	0	0	0	0
0	0	0	0	0	0	0	0	0	1	0	0	0	0
0	0	0	0	0	0	0	0	0	0	0	1	0	0
0													
OTU12450	0	0	0	0	0	0	0	1	0	0	0	0	0
0	0	0	0	0	0	0	0	0	0	0	0	0	0
0	0	0	0	0	0	0	0	0	0	0	0	0	0
0	0	0	0	0	0	0	0	0	0	0	0	0	0
0	0	0	0	0	0	0	0	0	0	0	0	0	0
0	0	0	0	0	0	0	0	0	0	0	0	0	0
0	0	0	0	0	0	0	0	0	0	0	0	0	0
0	0	0	0	0	0	0	0	0	0	0	0	0	0
0													
OTU10315	0	0	0	0	0	0	0	1	2	3	0	0	0
0	0	0	0	0	0	0	0	0	0	0	0	10	0
1	0	0	0	0	0	0	0	0	0	0	0	0	0

0	0	0	0	0	0	0	0	0	0	0	0	0	0
0	0	0	0	0	0	0	0	0	0	0	0	0	0
0	5	3	0	1	0	0	0	0	0	0	0	0	0
0	0	0	1	0	0	0	0	0	0	0	0	0	0
0	0	1	7	0	4	0	0	1	0	0	0	0	0
0													
OTU2545	0	0	0	0	0	0	0	1	0	0	0	0	0
0	0	0	0	0	0	0	0	0	0	0	0	0	0
0	0	0	0	0	0	0	0	0	0	0	0	0	0
0	0	0	0	0	0	0	0	0	0	0	0	0	0
0	0	0	0	0	0	0	0	0	0	0	0	0	0
0	0	0	0	0	0	0	0	0	0	0	0	0	0
0	0	0	0	0	0	0	1	0	0	0	0	0	0
0	0	0	0	0	0	0	0	0	0	0	0	0	0
0													
OTU7892	0	0	0	0	0	0	0	1	0	0	0	0	0
0	0	0	0	0	0	0	0	0	0	0	0	0	0
0	0	0	0	0	0	0	0	0	0	0	0	0	0
0	0	0	0	0	0	0	0	0	0	0	0	0	0
0	0	0	0	0	0	0	0	0	0	0	0	0	0
0	0	0	0	0	0	0	0	0	0	0	0	0	0
0	0	0	0	0	0	0	0	0	0	0	0	0	0
0	0	0	0	0	0	0	0	0	0	0	0	0	0
0													
OTU7066	0	0	0	0	0	0	0	1	0	0	0	1	1
7	0	0	1	0	0	0	0	0	0	0	0	0	0
0	0	0	0	1	0	2	1	0	0	0	0	0	0
0	0	0	0	0	2	1	0	0	0	0	0	0	0
0	1	1	0	0	0	0	0	0	0	0	0	0	1
1	0	0	0	0	0	0	0	0	0	0	0	0	0
0	0	0	0	0	0	1	0	1	0	2	0	0	3
0	0	0	0	0	0	0	0	1	0	0	3	1	0
0													

OTU15823	0	0	0	0	0	0	0	1	0	0	0	0	0
0	0	0	0	0	0	0	0	0	0	0	0	0	0
0	0	0	0	0	0	0	0	0	0	0	0	0	0
0	0	0	0	0	0	0	0	0	0	0	0	0	0
0	0	0	0	0	0	0	0	0	0	0	0	0	0
0	0	0	0	0	0	0	0	0	0	0	0	0	0
0	0	0	0	0	0	0	0	0	0	0	0	0	0
0	0	0	0	0	0	0	0	0	0	0	0	0	0
0													
OTU15820	0	0	0	0	0	0	0	0	1	0	0	0	0
0	0	0	0	0	0	0	0	0	0	0	0	0	0
0	0	0	0	0	0	0	0	0	0	0	0	0	0
0	0	0	0	0	0	0	0	0	0	0	0	0	0
0	0	0	0	0	0	0	0	0	0	0	0	0	0
0	0	0	0	0	0	0	0	0	0	0	0	0	0
0	0	0	0	0	0	0	0	0	0	0	0	0	0
0	0	0	0	0	0	0	0	0	0	0	0	0	0
0													
OTU7860	0	0	0	0	0	0	0	0	3	0	0	0	0
0	0	0	0	0	0	0	0	0	0	0	0	0	0
0	0	0	0	0	0	0	0	0	0	0	0	0	0
0	0	0	0	0	0	0	0	0	0	0	0	0	0
0	0	0	0	0	0	0	0	0	0	0	0	0	0
0	0	0	0	0	0	0	0	0	0	0	0	0	0
0	0	0	0	0	0	0	0	0	0	0	0	0	0
0	0	0	0	0	0	0	0	0	0	0	0	0	0
0													
OTU3239	0	0	0	0	0	0	0	0	2	1	0	0	0
0	0	0	0	0	0	0	0	0	0	0	0	0	0
0	0	0	0	0	0	0	0	0	0	0	0	0	0
0	0	10	0	1	2	0	0	4	11	0	0	1	0
5	1	1	0	8	12	2	8	6	4	1	0	0	4
2	0	0	0	0	0	0	0	0	0	0	1	0	5

0	10	1	1	0	0	0	2	0	0	0	0	3	0
0	2	1	0	0	2	2	6	2	5	0	0	0	0
0													
OTU4911	0	0	0	0	0	0	0	0	5	0	0	1	0
1	0	0	0	0	7	0	0	0	0	0	0	0	0
0	0	3	0	0	0	0	0	0	4	0	0	1	2
0	0	0	0	5	44	0	0	0	0	0	1	0	1
0	0	0	5	0	0	0	0	1	0	0	0	14	0
0	21	0	0	0	0	0	0	0	0	1	8	0	0
0	0	0	0	0	0	0	0	0	1	0	7	10	1
0	50	2	6	17	7	8	6	3	16	0	1	0	8
10													
OTU12451	0	0	0	0	0	0	0	0	1	0	0	0	0
0	0	0	0	0	0	0	0	0	0	0	0	0	0
0	0	0	0	0	0	0	0	0	0	0	0	0	0
0	0	0	0	0	0	0	0	0	0	0	0	0	0
0	0	0	0	0	0	0	0	0	0	0	0	0	0
0	0	0	0	0	0	0	0	0	0	0	0	0	0
0	0	0	0	0	0	0	0	0	0	0	0	0	0
0	0	0	0	0	0	0	0	0	0	0	0	0	0
0													
OTU7839	0	0	0	0	0	0	0	0	1	0	0	0	0
0	0	0	0	0	0	0	0	0	0	0	0	0	0
0	0	0	0	0	0	0	0	0	0	0	0	0	0
0	0	0	0	0	0	0	0	0	0	0	0	0	0
0	0	0	0	0	0	0	0	0	0	0	0	0	0
0	0	0	0	0	0	0	0	0	0	0	0	0	0
0	0	0	0	0	0	0	0	0	0	0	0	0	0
0	0	0	0	0	0	0	0	0	0	0	0	0	0
0													
OTU855	0	0	0	0	0	0	0	0	3	0	0	0	0
0	0	0	0	0	0	0	0	0	0	0	0	0	1
0	0	0	0	0	0	0	0	0	0	0	0	0	0

0	0	0	0	0	0	0	0	0	0	0	0	0	0
0	1	0	0	0	0	0	0	0	0	0	0	0	0
0	0	0	0	0	0	0	0	0	0	0	0	0	0
0	0	0	0	0	0	0	0	0	0	0	0	1	0
0	0	0	1	4	0	2	0	0	1	0	1	0	3
13													
OTU405	0	0	0	0	0	0	0	0	2	1	0	1	0
0	0	0	0	0	0	0	0	0	0	0	0	0	0
0	0	0	0	0	0	0	0	0	0	0	0	0	0
2	0	0	0	0	0	0	0	0	0	0	0	0	0
0	0	0	1	0	0	0	0	0	0	0	0	0	0
0	2	0	0	0	0	0	0	0	0	2	0	0	0
0	0	0	10	0	0	0	0	0	0	0	2	0	0
0	0	0	114	0	0	0	0	29	0	0	0	0	9
1													
OTU7840	0	0	0	0	0	0	0	0	1	0	0	0	0
0	0	0	0	0	0	0	0	0	0	0	0	0	0
0	0	0	0	0	0	0	0	0	0	0	0	0	0
0	0	0	0	0	0	0	0	0	0	0	0	0	0
0	0	0	0	0	0	0	0	0	0	0	0	0	0
0	0	0	0	0	0	0	0	0	0	0	0	0	0
0	0	0	0	0	0	0	0	0	0	0	0	0	0
0	0	0	0	0	0	0	0	0	0	0	0	0	0
0													
OTU8915	0	0	0	0	0	0	0	0	1	0	1	0	0
0	0	0	0	0	0	0	0	0	118	0	7	0	0
0	0	10	0	0	7	0	0	0	1	0	5	0	0
0	1	5	3	0	0	3	0	0	0	0	0	0	0
0	0	0	0	0	0	0	0	0	0	0	0	0	0
0	0	0	0	0	0	14	0	0	0	9	0	0	0
1	0	4	0	0	0	0	0	9	0	0	0	0	0
0	0	0	0	0	0	0	0	0	0	0	0	0	0
0													

OTU660	0	0	0	0	0	0	0	0	1	0	0	0	0
0	0	0	0	0	0	0	0	0	0	0	0	0	0
0	0	0	0	0	0	0	0	0	0	0	0	0	0
0	0	0	0	0	0	0	0	0	0	0	0	0	0
0	0	0	0	0	0	0	0	0	0	0	0	0	0
0	1	0	0	0	0	0	0	0	0	0	0	0	0
0	0	0	0	0	0	0	0	0	0	0	0	0	0
0	0	0	2	1	1	0	2	2	2	0	0	0	0
0													
OTU673	0	0	0	0	0	0	0	0	1	0	0	0	0
0	0	0	0	0	0	1	0	1	0	0	0	0	0
0	0	0	0	0	0	0	0	0	0	16	0	0	0
0	0	2	0	0	1	0	1	0	0	0	0	0	0
0	0	0	0	0	0	0	1	0	0	0	0	0	0
0	2	0	0	0	0	0	20	0	0	0	1	0	0
0	4	0	0	0	0	0	1	0	0	0	1	2	0
0	3	4	8	10	4	4	6	3	3	0	1	0	8
5													
OTU14517	0	0	0	0	0	0	0	0	1	0	0	0	0
0	0	0	0	0	0	0	0	0	0	0	0	0	0
0	0	0	0	0	0	0	0	0	0	0	0	0	0
0	0	0	0	0	0	0	0	0	0	0	0	0	0
0	0	0	0	0	0	0	0	0	0	0	0	0	0
0	0	0	0	0	0	0	0	0	0	0	0	0	0
0	0	0	0	0	0	0	0	0	0	0	0	0	0
0	0	0	0	0	0	0	0	0	0	0	0	0	0
0													
OTU15777	0	0	0	0	0	0	0	0	2	0	0	0	0
0	1	0	0	0	0	0	0	0	0	0	0	0	0
0	0	0	0	0	0	0	0	0	0	0	0	0	0
0	0	0	0	0	0	0	0	0	1	0	0	0	0
0	0	0	0	0	0	0	0	0	0	0	0	0	0
0	0	0	0	0	0	0	0	0	0	0	0	0	0

0 0 0 0 0 0 0 0 0 0 0 0 0 0
0 0 0 0 0 0 0 0 0 0 0 0 0 0
0
OTU2170 0 0 0 0 0 0 0 0 53 1 0 0 0
0 0 0 0 0 0 0 0 0 0 0 0 0 0
0 0 0 0 0 0 0 0 0 0 0 0 0 0
0 0 0 0 0 1 0 0 0 0 0 6 0 0
0 0 0 0 0 0 0 0 0 0 0 0 0 0
0 0 28 0 0 0 0 2 0 1 1 0 0 1
0 0 0 0 0 0 0 62 0 0 0 0 2 0
0 0 0 0 0 1 0 0 6 0 0 0 0 0
0
OTU14541 0 0 0 0 0 0 0 0 1 0 0 0 0
0 0 0 0 0 0 0 0 0 0 0 0 0 0
0 0 0 0 0 0 0 0 0 0 0 0 0 0
0 0 0 0 0 0 0 0 0 0 0 0 0 0
0 0 0 0 0 0 0 0 0 0 0 0 0 0
0 0 0 0 0 0 0 0 0 0 0 0 0 0
0 0 0 0 0 0 0 0 0 0 0 0 0 0
0 0 0 0 0 0 0 0 0 0 0 0 0 0
0
OTU7835 0 0 0 0 0 0 0 0 1 0 0 0 0
0 0 0 0 0 0 0 0 0 0 0 0 0 0
0 0 0 0 0 0 0 0 0 0 0 0 0 0
0 0 0 0 0 0 0 0 0 0 0 0 0 0
0 0 0 0 0 0 0 0 0 0 0 0 0 0
0 0 0 0 0 0 0 0 0 0 0 0 0 0
0 0 0 0 0 0 0 0 0 0 0 0 0 0
0 0 0 0 0 0 0 0 0 0 0 0 0 0
0
OTU13093 0 0 0 0 0 0 0 0 1 0 1 0 0
0 5 0 1 0 1 0 1 1 0 0 0 0 1
0 0 2 1 0 0 1 1 0 0 1 1 1 0

0	0	1	1	0	0	0	1	0	1	0	0	1	0
1	0	1	1	1	1	0	0	0	1	0	1	0	0
0	0	0	1	1	0	0	1	0	0	0	1	0	1
0	0	0	0	0	0	0	1	0	0	0	0	0	0
0	4	1	0	1	0	0	0	0	0	0	1	2	0
3													
OTU4784	0	0	0	0	0	0	0	0	1	0	0	0	0
0	0	0	0	0	0	0	0	0	0	0	0	0	0
0	0	0	0	0	0	0	0	0	0	0	0	0	0
0	0	0	0	1	0	0	0	2	1	0	0	0	0
1	0	0	0	0	0	0	0	0	1	0	0	0	0
0	0	0	0	0	0	0	0	0	0	0	0	0	0
0	0	0	0	0	0	0	0	0	0	0	0	0	0
0	0	0	0	0	1	0	0	0	1	0	0	0	0
0													
OTU171	0	0	0	0	0	0	0	0	2	3	0	0	0
0	0	1	1	1	6	3	1	0	76	77	88	2	0
0	0	4	12	17	0	0	0	0	0	1	0	1	0
0	0	0	74	0	15	1	20	8	15	3	0	0	6
2	86	0	0	1	0	9	11	3	0	0	3	7	2
1	0	0	0	0	0	2	0	0	0	22	0	0	0
2	0	1	0	0	0	0	0	0	0	0	0	0	0
0	0	0	3	0	0	0	0	0	0	0	0	0	0
0													
OTU548	0	0	0	0	0	0	0	0	3	0	0	0	0
0	0	5	0	0	0	0	0	0	0	0	0	0	0
0	0	0	0	0	3	0	0	0	11	0	0	0	0
0	3	0	0	0	0	0	0	0	0	0	0	0	0
0	0	1	4	0	0	0	0	0	0	0	0	0	0
1	0	0	0	1	28	0	0	0	0	0	0	0	0
0	0	0	0	0	0	0	0	0	0	0	0	0	0
2	0	0	0	0	0	0	0	0	0	0	0	0	0
0													

OTU14542	0	0	0	0	0	0	0	0	1	0	0	0	0
0	0	0	0	0	0	0	0	0	0	0	0	0	0
0	0	0	0	0	0	0	0	0	0	0	0	0	0
0	0	0	0	0	0	0	0	0	0	0	0	0	0
0	0	0	0	0	0	0	0	0	0	0	0	0	0
0	0	0	0	0	0	0	0	0	0	0	0	0	0
0	0	0	0	0	0	0	0	0	0	0	0	0	0
0	0	0	0	0	0	0	0	0	0	0	0	0	0
0													
OTU12475	0	0	0	0	0	0	0	0	7	0	0	0	0
0	0	0	0	0	0	0	0	0	0	0	0	0	0
0	0	0	0	0	0	0	0	0	0	0	0	0	0
1	0	0	0	0	0	0	0	0	0	0	0	0	0
0	0	0	0	0	0	0	0	0	0	0	0	0	0
0	0	0	0	0	0	0	1	0	2	0	0	0	0
0	0	0	1	0	0	0	0	0	0	0	1	1	0
0	0	0	0	0	0	0	0	0	0	0	0	0	0
0													
OTU11298	0	0	0	0	0	0	0	0	1	0	0	0	0
0	0	0	0	0	0	0	0	0	0	0	0	0	0
0	0	0	0	0	0	0	0	0	0	0	0	0	0
0	0	0	0	0	0	0	0	0	0	0	0	0	0
0	0	0	0	0	0	0	0	0	0	0	0	0	0
0	0	0	0	0	0	0	0	0	0	0	0	0	0
0	0	0	0	0	0	0	0	0	0	0	0	0	0
0	0	0	0	0	1	0	0	0	0	0	0	0	0
0													
OTU7834	0	0	0	0	0	0	0	0	1	0	0	0	0
0	0	0	0	0	0	0	0	0	0	0	0	0	0
0	0	0	0	0	0	0	0	0	0	0	0	0	0
0	0	0	0	0	0	0	0	0	0	0	0	0	0
0	0	0	0	0	0	0	0	0	0	0	0	0	0
0	0	0	0	0	0	0	0	0	0	0	0	0	0

0	0	0	0	0	0	0	0	0	0	0	0	0	0
0	0	0	0	0	0	0	0	0	0	0	0	0	0
0													
OTU7836	0	0	0	0	0	0	0	0	1	0	0	0	0
0	0	0	0	0	0	0	0	0	0	0	0	0	0
0	0	0	0	0	0	0	0	0	0	0	0	0	0
0	0	0	0	0	0	0	0	0	0	0	0	0	0
0	0	0	0	0	0	0	0	0	0	0	0	0	0
0	0	0	0	0	0	0	0	0	0	0	0	0	0
0	0	0	0	0	0	0	0	0	0	0	0	0	0
0	0	0	0	0	0	0	0	0	0	0	0	0	0
0													
OTU1244	0	0	0	0	0	0	0	0	1	0	0	0	0
0	0	0	3	0	0	1	1	0	0	0	0	0	0
0	2	3	0	1	0	2	1	0	0	2	0	0	0
0	2	10	2	3	0	0	0	18	0	0	0	27	0
6	0	13	0	1	18	15	19	2	8	7	0	6	0
6	0	0	0	3	1	1	0	0	0	0	1	1	1
0	2	0	0	0	1	2	0	0	0	0	0	0	12
0	0	0	0	0	0	0	1	0	0	0	0	1	0
0													
OTU9744	0	0	0	0	0	0	0	0	1	2	0	0	0
0	0	0	0	0	0	0	0	0	0	0	0	0	0
0	0	0	0	0	0	0	0	0	0	2	0	0	0
0	0	0	0	0	0	0	0	0	0	0	0	0	0
0	0	0	0	0	0	0	2	0	0	0	0	0	0
0	1	0	0	1	1	0	0	0	0	0	0	1	0
0	0	0	0	0	1	0	0	0	0	0	0	0	0
0	0	0	0	0	0	0	0	0	0	0	0	0	0
0													
OTU7837	0	0	0	0	0	0	0	0	1	0	0	0	0
0	0	0	0	0	0	0	0	0	0	0	0	0	0
0	0	0	0	0	0	0	0	0	0	0	0	0	0

0 0 0 0 0 0 0 0 0 0 0 0 0 0
0 0 0 0 0 0 0 0 0 0 0 0 0 0
0 0 0 0 0 0 0 0 0 0 0 0 0 0
0 0 0 0 0 0 0 0 0 0 0 0 0 0
0 0 0 0 0 0 0 0 0 0 0 0 0 0
0
OTU3990 0 0 0 0 0 0 0 0 1 0 0 0 0
0 0 0 0 0 0 0 0 0 0 0 0 0 0
0 1 0 0 0 0 0 0 0 0 0 0 2 0
0 0 0 0 0 1 1 0 0 0 0 0 0 0
0 0 0 1 0 0 0 0 0 0 0 0 0 0
0 1 2 0 0 0 0 0 0 0 0 0 0 0
0 0 0 0 1 0 0 0 0 0 0 0 2 0
0 2 0 0 0 0 0 1 0 1 0 0 0 1
0
OTU3454 0 0 0 0 0 0 0 0 3 0 0 0 0
0 0 0 0 0 0 0 0 0 0 1 0 1 0
3 0 0 0 1 0 0 0 0 0 0 0 0 0
5 0 0 0 0 0 0 4 0 0 0 0 0 0
0 1 0 0 2 1 0 0 0 0 0 0 0 0
0 1 1 0 0 0 0 1 0 0 0 0 0 1
0 2 0 1 1 0 0 0 0 0 0 0 6 0
0 1 1 5 0 0 0 1 1 2 0 0 1 1
1
OTU706 0 0 0 0 0 0 0 0 7 3 0 1 0
0 1 0 0 0 0 0 0 0 0 0 0 0 0
0 0 0 0 0 0 0 0 0 0 0 0 0 0
9 0 0 0 0 0 0 0 0 0 0 0 0 0
0 0 0 0 0 0 0 3 0 0 0 0 0 0
0 0 0 0 0 0 0 2 0 2 0 1 0 1
1 0 0 0 0 1 0 0 0 0 0 0 0 0
0 0 0 0 0 0 0 0 0 0 0 0 1 0
0

OTU254 0 0 0 0 0 0 0 0 7 0 0 0 0
0 0 0 3 0 0 0 0 1 0 38 0 0 3
0 6 0 0 12 0 9 0 0 0 0 0 0 0
0 0 63 0 23 0 0 0 2 8 8 1 1 0
7 6 13 0 12 6 5 11 0 1 1 36 1 5
2 1 0 0 0 0 0 42 0 0 0 0 0 0
0 8 0 0 0 0 0 0 0 0 0 0 1 0
0 3 1 0 1 3 5 1 0 2 1 0 0 1
0
OTU12453 0 0 0 0 0 0 0 0 1 0 0 0 0
0 0 0 0 0 0 0 0 0 0 0 0 0 0
0 0 0 0 0 0 0 0 0 0 0 0 0 0
0 0 0 0 0 0 0 0 0 0 0 0 0 0
0 0 0 0 0 0 0 0 0 0 0 0 0 0
0 0 0 0 0 0 0 0 0 0 0 0 0 0
0 0 0 0 0 0 0 0 0 0 0 0 0 0
0 0 0 0 0 0 0 0 0 0 0 0 0 0
0
OTU6700 0 0 0 0 0 0 0 0 1 0 0 0 0
0 0 0 0 0 0 0 0 0 0 0 0 0 0
0 0 0 0 0 0 1 0 0 0 0 0 0 0
0 0 1 0 0 1 0 0 0 0 0 0 1 0
1 1 2 0 1 0 1 1 0 2 0 0 0 2
1 0 0 0 0 0 0 0 0 0 0 0 0 1
0 1 0 0 0 0 0 0 3 0 0 0 0 3
0 0 0 0 0 0 0 0 0 0 0 0 0 0
0
OTU1019 0 0 0 0 0 0 0 0 1 0 0 0 0
0 0 0 0 0 0 0 0 0 0 0 0 0 0
0 0 0 0 0 0 0 0 0 0 0 0 0 0
0 0 0 0 3 0 0 0 0 0 0 0 0 0
0 0 0 0 0 0 0 0 0 0 0 0 0 0
0 1 0 0 0 1 0 0 0 0 0 0 0 0

0	0	0	0	0	0	0	0	0	0	0	0	0	0
0	0	0	3	0	0	0	0	0	0	0	0	0	1
0													
OTU5125	0	0	0	0	0	0	0	0	2	0	0	0	0
0	0	0	0	0	0	0	0	0	0	0	0	0	0
0	0	0	0	0	0	0	0	0	0	0	0	4	0
0	0	0	0	0	0	0	0	0	0	0	0	0	0
0	0	0	0	0	0	0	0	0	0	0	0	0	0
0	0	0	0	0	0	0	0	0	0	0	0	0	0
0	0	0	0	0	0	0	0	0	0	0	0	0	0
0	0	0	0	0	0	0	0	0	0	0	0	0	0
0													
OTU14789	0	0	0	0	0	0	0	0	1	0	0	0	0
0	0	0	0	0	0	0	0	0	0	0	0	0	0
0	1	0	0	0	0	0	0	0	0	0	0	0	0
0	0	0	0	0	0	0	1	0	0	0	0	0	0
0	0	0	0	0	0	0	0	1	0	0	0	0	0
0	0	0	0	0	0	0	0	0	0	0	0	0	0
0	0	0	0	0	0	0	0	0	0	0	0	0	0
0	0	1	0	1	0	0	0	0	0	0	0	0	0
0													
OTU15449	0	0	0	0	0	0	0	0	2	0	0	0	1
0	0	0	0	0	0	0	0	0	0	0	0	0	0
0	0	0	0	0	0	1	0	0	0	0	0	0	0
0	0	0	0	0	0	0	0	1	0	0	0	0	0
0	0	0	0	0	0	0	0	0	0	0	0	0	0
0	0	0	0	0	0	0	0	0	0	0	0	0	0
0	0	0	2	0	0	0	0	0	0	0	0	0	2
0	0	0	0	0	0	0	0	0	0	0	0	0	0
0													
OTU12452	0	0	0	0	0	0	0	0	1	0	0	0	0
0	0	0	0	0	0	0	0	0	0	0	0	0	0
0	0	0	0	0	0	0	0	0	0	0	0	0	0

0	0	0	0	0	0	0	0	0	0	0	0	0	0
0	0	0	0	0	0	0	0	0	0	0	0	0	0
0	0	0	0	0	0	0	0	0	0	0	0	0	0
0	0	0	0	0	0	0	0	0	0	0	0	0	0
0	0	0	0	0	0	0	0	0	0	0	0	0	0
0													
OTU7830	0	0	0	0	0	0	0	0	5	0	0	0	0
0	0	0	0	0	0	0	0	0	0	0	0	0	0
0	0	0	0	0	0	0	0	0	0	0	0	0	0
0	0	0	0	0	0	0	0	0	0	0	0	0	0
0	0	0	0	0	0	0	0	0	0	0	0	0	0
0	0	0	0	0	0	0	0	0	0	0	0	0	0
0	0	0	0	0	0	0	0	0	0	0	0	0	0
0	0	0	0	0	0	0	0	1	0	0	0	0	0
0													
OTU15826	0	0	0	0	0	0	0	0	1	0	0	0	0
0	0	1	1	0	0	0	0	0	0	0	0	1	0
0	0	0	0	0	0	0	0	0	0	0	0	0	0
0	0	1	0	0	0	0	0	1	0	0	0	0	0
0	0	0	0	0	0	0	0	0	0	0	0	0	0
0	0	0	0	0	0	0	0	0	0	0	0	0	0
0	0	0	0	0	0	1	0	0	1	0	0	0	0
2	0	0	0	0	0	0	0	0	0	0	0	0	0
0													
OTU14496	0	0	0	0	0	0	0	0	2	0	0	0	0
0	0	0	0	0	0	0	0	0	0	0	0	0	0
0	0	0	0	0	0	0	0	0	0	0	0	0	0
0	0	0	0	0	0	0	0	0	0	0	0	0	0
0	0	0	0	0	0	0	0	0	0	0	0	0	0
0	0	0	0	0	0	0	0	0	0	0	0	0	0
0	0	0	0	0	0	0	0	0	0	0	0	0	0
0	0	0	0	0	0	0	0	0	0	0	0	0	0
0													

OTU7841	0	0	0	0	0	0	0	0	1	0	0	0	0
0	0	0	0	0	0	0	0	0	0	0	0	0	0
0	0	0	0	0	0	0	0	0	0	0	0	0	0
0	0	0	0	0	0	0	0	0	0	0	0	0	0
0	0	0	0	0	0	0	0	0	0	0	0	0	0
0	0	0	0	0	0	0	0	0	0	0	0	0	0
0	0	0	0	0	0	0	0	0	0	0	0	0	0
0	0	0	0	0	0	0	0	0	0	0	0	0	0
0													
OTU15825	0	0	0	0	0	0	0	0	1	0	0	0	0
0	0	0	0	0	0	0	0	0	0	0	0	0	0
0	0	0	0	0	0	0	0	0	0	0	0	0	0
0	0	0	0	0	0	0	0	0	0	0	0	0	0
0	0	0	0	0	0	0	0	0	0	0	0	0	0
0	0	0	0	0	0	0	0	0	0	0	0	0	0
0	0	0	0	0	0	0	0	0	0	0	0	0	0
0	0	0	0	0	0	0	0	0	0	0	0	0	0
0													
OTU7369	0	0	0	0	0	0	0	0	1	1	0	0	0
0	0	0	0	0	1	0	0	0	0	0	0	0	0
0	0	0	0	0	0	0	0	0	0	0	0	0	0
0	0	1	0	0	0	0	0	0	0	0	0	0	0
0	0	0	0	0	0	0	0	0	0	0	0	0	0
0	0	0	0	1	0	0	0	0	0	0	0	0	1
0	0	0	1	0	0	0	0	0	0	0	0	0	0
0	0	0	0	0	0	0	0	0	0	0	0	0	0
0													
OTU241	0	0	0	0	0	0	0	0	4	1	0	1	0
0	1	0	0	0	0	0	0	0	0	0	0	0	0
0	0	0	0	0	0	1	0	0	0	0	0	0	0
1	0	0	1	0	0	0	1	0	0	0	0	0	0
0	0	0	0	0	0	0	0	0	0	0	0	1	0
0	8	0	0	0	0	0	1	0	1	0	1	0	0

0 0 0 3 0 0 0 0 0 0 0 10 19 0
0 15 11 12 37 12 38 17 7 14 0 3 0 9
21
OTU15828 0 0 0 0 0 0 0 0 1 0 0 0 0
0 0 0 0 0 0 0 0 0 0 0 0 0 0
0 0 0 0 0 0 0 0 0 0 0 0 0 0
0 0 0 0 0 0 0 0 0 0 0 0 0 0
0 0 0 0 0 0 0 0 0 0 0 0 0 0
0 0 0 0 0 0 0 0 0 0 0 0 0 0
0 0 0 0 0 0 0 0 0 0 0 0 0 0
0 0 0 0 0 0 0 0 0 0 0 0 0 0
0
OTU7842 0 0 0 0 0 0 0 0 1 0 0 0 0
0 0 0 0 0 0 0 0 0 0 0 0 0 0
0 0 0 0 0 0 0 0 0 0 0 0 0 0
0 0 0 0 0 0 0 0 0 0 0 0 0 0
0 0 0 0 0 0 0 0 0 0 0 0 0 0
0 0 0 0 0 0 0 0 0 0 0 0 0 0
0 0 0 0 0 0 0 0 0 0 0 0 0 0
0 0 0 0 0 0 0 0 0 0 0 0 0 0
0
OTU15339 0 0 0 0 0 0 0 0 3 0 0 0 0
0 0 0 0 0 0 0 0 1 6 0 0 0 0
0 0 0 0 0 0 0 0 0 0 2 0 0 0
0 0 0 0 1 0 0 0 2 0 3 0 0 0
0 0 3 1 0 0 0 8 0 1 0 0 0 0
1 0 0 0 0 0 0 4 1 0 0 0 0 0
0 0 0 0 0 0 1 2 0 0 0 0 0 0
0 0 0 0 2 0 0 1 0 0 0 0 0 0
0
OTU14544 0 0 0 0 0 0 0 0 1 0 0 0 0
0 0 0 0 0 0 0 0 0 0 0 0 0 0
0 0 0 0 0 0 0 0 0 0 0 0 0 0

0	0	0	0	0	0	0	0	0	0	0	0	0	0
0	0	0	0	0	0	0	0	0	0	0	0	0	0
0	0	0	0	0	0	0	0	0	0	0	0	0	0
0	0	0	0	0	0	0	0	0	0	0	0	0	0
0	0	0	0	0	0	0	0	0	0	0	0	0	0
0													
OTU12442	0	0	0	0	0	0	0	0	1	0	0	0	0
0	0	0	0	0	0	0	0	0	0	0	0	0	0
0	0	0	0	0	0	0	0	0	0	0	0	0	0
0	0	0	0	0	0	0	0	0	0	0	0	0	0
0	0	0	0	0	0	0	0	0	0	0	0	0	0
0	0	0	0	0	0	0	0	0	0	0	0	0	0
0	0	0	0	0	0	0	0	0	0	0	0	0	0
0	0	0	0	0	0	0	0	0	0	0	0	0	0
0													
OTU15827	0	0	0	0	0	0	0	0	1	0	0	0	0
0	0	0	0	0	0	0	0	0	0	0	0	0	0
0	0	0	0	0	0	0	0	0	0	0	0	0	0
0	0	0	0	0	1	0	0	0	0	0	0	0	0
0	0	0	0	0	0	0	0	0	0	0	0	0	0
0	0	0	0	0	0	0	0	0	0	0	0	0	0
0	0	0	0	0	0	0	0	0	0	0	0	0	0
0	0	0	0	0	0	0	0	0	0	0	0	0	0
0													
OTU450	0	0	0	0	0	0	0	0	4	0	0	0	0
0	0	0	62	0	0	0	0	12	0	2	0	2	0
0	28	0	0	0	0	0	0	0	0	0	0	0	0
0	0	0	0	0	0	0	0	0	0	0	0	0	0
0	0	0	0	0	3	0	0	0	0	0	0	0	0
0	0	0	0	0	0	0	0	0	0	0	0	0	0
0	12	0	8	0	0	0	0	0	0	0	0	0	0
0	0	0	0	0	0	0	0	0	0	0	0	0	0
0													

OTU2642 0 0 0 0 0 0 0 0 1 0 0 0 0
0 0 0 0 0 0 0 0 0 0 0 0 0 0
0 0 0 0 0 0 0 0 0 0 0 0 0 0
0 0 0 0 1 0 0 0 0 0 0 0 0 0
0 0 0 0 0 0 0 0 0 0 0 0 0 0
0 0 0 0 0 0 0 0 0 0 0 0 0 0
0 0 0 0 0 0 0 1 0 0 0 0 0 0
0 0 0 0 0 0 0 0 0 0 0 0 0 0
0
OTU12443 0 0 0 0 0 0 0 0 1 0 0 0 0
0 0 0 0 0 0 0 0 0 0 0 0 0 0
0 0 0 0 0 0 0 0 0 0 0 0 0 0
0 0 0 0 0 0 0 0 0 0 0 0 0 0
0 0 0 0 0 0 0 0 0 0 0 0 0 0
0 0 0 0 0 0 0 0 0 0 0 0 0 0
0 0 0 0 0 0 0 0 0 0 0 0 0 0
0 0 0 0 0 0 0 0 0 0 0 0 0 0
0
OTU14545 0 0 0 0 0 0 0 0 1 0 0 0 0
0 0 0 0 0 0 0 0 0 0 0 0 0 0
0 0 0 0 0 0 0 0 0 0 0 0 0 0
0 0 0 0 0 0 0 0 0 1 0 0 0 0
0 0 0 0 0 0 0 0 0 0 0 0 0 0
0 0 0 0 0 0 0 0 0 0 0 0 0 0
0 0 0 0 0 0 0 0 0 0 0 0 0 0
0 0 0 0 0 0 0 0 0 0 0 0 0 0
0
OTU7829 0 0 0 0 0 0 0 0 1 0 0 0 0
0 0 0 0 0 0 0 0 0 0 0 0 0 0
0 0 0 0 0 0 0 0 0 0 0 0 0 0
0 0 0 0 0 0 0 0 0 0 0 0 0 0
0 0 0 0 0 0 0 0 0 0 0 0 0 0
0 0 0 0 0 0 0 0 0 0 0 0 0 0

0	0	0	0	0	0	0	0	0	0	0	0	0	0
0	0	0	0	0	0	0	0	0	0	0	0	0	0
0													
OTU12441	0	0	0	0	0	0	0	0	1	0	0	0	0
0	0	0	0	0	0	0	0	0	0	0	0	0	0
0	0	0	0	0	0	0	0	0	0	0	0	0	0
0	0	0	0	0	0	0	0	0	0	0	0	0	0
0	0	0	0	0	0	0	0	0	0	0	0	0	0
0	0	0	0	0	0	0	0	0	0	0	0	0	0
0	0	0	0	0	0	0	0	0	0	0	0	0	0
0	0	0	0	0	0	0	0	0	0	0	0	0	0
0													
OTU2331	0	0	0	0	0	0	0	0	1	0	0	0	0
0	0	0	0	0	0	0	0	0	0	0	0	0	0
0	0	0	0	0	0	0	0	0	0	0	0	0	0
0	0	0	0	0	0	0	0	0	0	0	0	0	0
0	0	0	0	0	0	0	0	0	0	0	0	0	0
0	0	0	0	0	0	0	0	0	0	0	0	0	0
0	0	0	0	0	0	0	0	0	0	0	0	0	0
0	3	0	0	2	0	0	3	0	1	0	0	0	0
0													
OTU15774	0	0	0	0	0	0	0	0	1	0	0	0	0
0	0	0	0	0	0	0	0	0	0	0	0	0	0
0	0	0	0	0	0	0	0	0	0	0	0	0	0
0	0	0	0	0	0	0	0	0	0	0	0	0	0
0	0	0	0	0	0	0	0	0	0	0	0	0	0
0	0	0	0	0	0	0	0	0	0	0	0	0	0
0	0	0	0	0	0	0	0	0	0	0	0	0	0
0	0	0	0	0	0	0	0	0	0	0	0	0	0
0													
OTU15773	0	0	0	0	0	0	0	0	1	0	0	0	0
0	0	0	0	0	0	0	0	0	0	0	0	0	0
0	0	0	0	0	0	0	0	0	0	0	0	0	0

0	0	0	0	0	0	0	0	0	0	0	0	0	0
0	0	0	0	0	0	0	0	0	0	0	0	0	0
0	0	0	0	0	0	0	0	0	0	0	0	0	0
0	0	0	0	0	0	0	0	0	0	0	0	0	0
0	0	0	0	0	0	0	0	0	0	0	0	0	0
0													
OTU14546	0	0	0	0	0	0	0	0	1	0	0	0	0
0	0	0	0	0	0	0	0	0	0	0	0	0	0
0	0	0	0	0	0	0	0	0	0	0	0	0	0
0	0	0	0	0	0	0	0	0	0	0	0	0	0
0	0	0	0	0	0	0	0	0	0	0	0	0	0
0	0	0	0	0	0	0	0	0	0	0	0	0	0
0	0	0	0	0	0	0	0	0	0	0	0	0	0
0	0	0	0	0	0	0	0	0	0	0	0	0	0
0													
OTU1039	0	0	0	0	0	0	0	0	2	0	0	0	0
0	0	0	0	0	0	0	0	0	0	0	0	0	0
0	0	0	0	0	0	0	0	0	0	0	0	0	0
0	0	0	0	0	0	0	0	0	0	0	0	0	0
0	0	0	0	0	0	0	0	0	0	0	0	0	0
0	0	0	0	0	0	0	0	0	0	0	0	0	2
0	0	0	1	0	0	0	0	0	0	0	0	0	0
0	0	0	0	1	0	0	0	0	0	0	0	0	0
0													
OTU7989	0	0	0	0	0	0	0	0	5	0	0	2	0
0	0	3	2	4	0	0	0	0	0	0	0	2	0
0	0	0	1	2	0	0	0	0	0	0	0	0	0
1	0	1	0	0	0	0	1	0	0	0	0	0	0
0	2	0	0	1	0	0	0	0	2	0	0	0	0
0	0	0	1	0	0	0	0	0	0	0	0	2	0
3	0	0	0	0	0	1	0	1	0	1	0	0	1
1	0	0	0	1	0	0	0	0	0	0	1	1	0
0													

OTU15775	0	0	0	0	0	0	0	0	1	0	0	0	0
0	0	0	0	0	0	0	0	0	0	0	0	0	0
0	0	0	0	0	0	0	0	0	0	0	0	0	0
0	0	0	0	0	0	0	0	0	0	0	0	0	0
0	0	0	0	0	0	0	0	0	0	0	0	0	0
0	0	0	0	0	0	0	0	0	0	0	0	0	0
0	0	0	0	0	0	0	1	0	0	0	0	0	0
0	0	0	0	0	0	0	0	0	0	0	0	0	0
0													
OTU623	0	0	0	0	0	0	0	0	2	0	0	0	0
0	0	0	0	0	0	0	1	0	0	0	1	0	0
0	0	0	0	0	0	0	0	0	2	0	0	0	0
0	0	0	0	0	0	0	0	0	0	0	0	0	0
0	0	0	0	0	0	0	0	0	0	0	0	0	0
0	5	0	0	0	0	0	0	0	0	0	1	0	0
0	0	0	0	0	0	0	0	0	0	0	6	6	0
0	8	10	1	2	9	0	3	0	4	0	0	0	0
0													
OTU12438	0	0	0	0	0	0	0	0	1	0	0	0	0
0	0	0	0	0	0	0	0	0	0	0	0	0	0
0	0	0	0	0	0	0	0	0	0	0	0	0	0
0	0	0	0	0	0	0	0	0	0	0	0	0	0
0	0	0	0	0	0	0	0	0	0	0	0	0	0
0	0	0	0	0	0	0	0	0	0	0	0	0	0
0	0	0	0	0	0	0	0	0	0	0	0	0	0
0	0	0	0	0	0	0	0	0	0	0	0	0	0
0													
OTU14543	0	0	0	0	0	0	0	0	1	0	0	0	0
0	0	0	0	0	0	0	0	0	0	0	0	0	0
0	0	1	0	0	0	0	0	0	0	0	0	0	0
0	0	0	0	0	0	0	0	0	0	0	0	0	0
0	0	0	0	0	0	0	0	0	0	0	0	0	0
0	0	0	0	2	0	0	1	0	0	0	2	0	0

0	0	0	0	0	0	0	0	0	0	0	0	0	0
0	0	0	0	0	0	0	0	0	0	0	0	0	0
0													
OTU15772	0	0	0	0	0	0	0	0	1	0	0	0	0
0	0	0	0	0	0	0	0	0	0	0	0	0	0
0	0	0	0	0	0	0	0	0	0	0	0	0	0
0	0	0	0	0	0	0	0	0	0	0	0	0	0
0	0	0	0	0	0	0	0	0	0	0	0	0	0
0	0	0	0	0	0	0	0	0	0	0	0	0	0
0	0	0	0	0	0	0	0	0	0	0	0	0	0
0	0	0	0	0	0	0	0	0	0	0	0	0	0
0													
OTU7832	0	0	0	0	0	0	0	0	1	0	0	0	0
0	0	0	0	0	0	0	0	0	0	0	0	0	0
0	0	0	0	0	0	0	0	0	0	0	0	0	0
0	0	0	0	0	1	0	0	1	0	0	0	0	0
0	0	0	0	0	0	0	0	0	0	0	0	0	0
0	0	0	0	0	0	0	0	0	0	0	0	0	0
0	1	0	0	0	0	0	0	0	0	0	0	0	0
0	0	0	0	0	0	0	0	0	0	0	0	0	0
0													
OTU3905	0	0	0	0	0	0	0	0	1	0	0	0	0
0	0	0	1	0	0	1	0	0	0	0	0	1	0
1	0	0	0	0	0	1	0	0	0	0	0	2	0
0	0	0	0	0	0	0	0	0	0	0	0	0	0
0	0	0	0	0	0	0	0	0	0	0	0	0	0
0	0	0	0	0	0	0	0	0	0	0	1	0	0
0	0	0	2	0	0	0	0	0	0	0	0	1	0
0	0	1	1	0	0	0	0	0	0	0	0	0	3
0													
OTU8609	0	0	0	0	0	0	0	0	1	0	0	0	0
0	0	0	0	0	0	0	0	0	0	0	0	0	0
0	0	1	0	0	1	0	0	0	1	0	1	0	0

0	0	0	0	0	1	0	0	0	0	0	0	0	0
0	0	0	0	0	0	0	1	1	0	0	0	0	0
0	0	0	0	1	0	0	0	0	0	0	0	0	0
0	0	0	0	1	0	0	0	0	0	0	0	0	0
0	1	0	0	0	0	0	0	0	0	0	0	0	0
1													
OTU12439	0	0	0	0	0	0	0	0	1	0	0	0	0
0	0	0	0	0	0	0	0	0	0	0	0	0	0
0	0	0	0	0	0	0	0	0	0	0	0	0	0
0	0	0	0	0	0	0	0	0	0	0	0	0	0
0	0	0	0	0	0	0	0	0	0	0	0	0	0
0	0	0	0	0	0	0	0	0	0	0	0	0	0
0	0	0	0	0	0	0	0	0	0	0	0	0	0
0	0	0	0	0	0	0	0	0	0	0	0	0	0
0													
OTU16189	0	0	0	0	0	0	0	0	2	0	2	0	0
0	1	1	6	0	0	0	0	18	0	0	0	3	3
5	2	0	0	1	0	10	1	0	0	0	0	5	0
0	0	1	0	6	14	0	35	1	16	11	12	0	29
0	1	2	27	28	28	0	50	50	9	35	27	13	58
20	0	1	0	0	0	0	22	0	1	0	0	0	1
0	41	0	0	0	0	0	10	0	0	0	0	0	0
1	0	0	0	0	0	0	0	0	0	0	0	10	0
0													
OTU6086	0	0	0	0	0	0	0	0	1	0	0	0	0
0	0	0	0	0	0	0	0	0	0	0	0	0	0
0	0	0	1	0	0	0	0	0	0	1	0	1	0
0	0	2	0	0	0	0	0	1	4	1	0	0	0
1	0	2	0	2	1	2	1	2	0	0	0	1	1
1	0	0	0	0	0	0	0	0	0	0	1	0	1
0	3	0	0	0	0	0	0	0	0	0	0	0	0
0	0	0	0	2	1	2	1	1	2	0	0	0	0
2													

OTU13626	0	0	0	0	0	0	0	0	1	0	0	0	0
0	0	0	0	0	0	0	0	0	0	0	0	0	0
0	0	0	0	0	0	0	0	0	0	0	0	0	0
0	0	0	0	0	0	0	0	0	0	0	0	0	0
0	0	0	0	0	0	0	0	0	0	0	0	0	0
0	0	0	0	0	0	0	0	0	0	0	1	0	0
0	0	0	0	0	0	0	0	0	0	0	0	0	0
0	0	0	0	0	0	0	0	0	0	0	0	0	0
0													
OTU16346	0	0	0	0	0	0	0	0	1	0	0	0	0
0	0	0	2	0	0	0	0	1	0	5	0	0	0
0	0	0	0	1	0	1	0	0	0	0	1	0	0
3	0	0	0	0	0	0	1	0	0	0	1	0	0
0	0	1	0	1	0	1	0	0	0	0	0	0	0
0	0	0	0	0	0	0	0	0	0	0	0	2	0
1	0	0	0	0	0	0	0	0	1	0	0	0	0
0	0	0	0	0	0	0	0	0	0	0	1	1	0
0													
OTU15780	0	0	0	0	0	0	0	0	1	0	0	0	0
0	0	0	0	0	0	0	0	0	0	0	0	0	0
0	0	0	0	0	0	0	0	0	0	0	0	0	0
0	0	0	0	0	0	0	0	0	0	0	0	0	0
0	0	0	0	0	0	0	0	0	0	0	0	0	0
0	0	0	0	0	0	0	0	0	0	0	0	0	0
0	0	0	0	0	0	0	0	0	0	0	0	0	0
0	0	0	0	0	0	0	0	0	0	0	0	0	0
0													
OTU10948	0	0	0	0	0	0	0	0	8	1	0	0	0
0	0	0	0	0	0	2	0	0	0	0	0	0	0
10	0	0	0	0	0	0	0	0	0	0	0	0	0
0	0	0	1	0	0	1	0	0	0	0	1	0	2
0	0	0	4	0	0	0	0	0	0	0	0	3	0
0	22	0	0	0	0	0	1	0	7	0	8	0	0

0	0	0	6	0	1	0	0	0	0	0	3	3	0
0	23	10	96	15	9	11	8	1	13	0	0	0	20
7													
OTU602	0	0	0	0	0	0	0	0	1	0	0	0	0
0	0	0	1	1	0	0	0	0	1	0	0	0	1
0	0	0	0	0	0	0	0	0	0	5	0	0	0
0	0	0	0	0	0	0	0	0	1	0	0	0	0
1	0	0	0	0	1	2	2	3	0	0	0	1	1
1	0	0	0	0	0	0	2	0	0	0	0	1	0
0	0	0	0	0	0	0	0	0	0	0	0	0	0
0	0	0	0	0	0	1	2	0	0	0	0	0	0
0													
OTU574	0	0	0	0	0	0	0	0	1	0	0	0	0
0	0	0	0	0	0	1	0	1	0	0	0	0	0
0	1	0	0	0	0	1	0	0	0	2	0	0	0
0	0	0	0	1	0	0	0	0	0	0	1	0	0
0	0	1	0	0	1	0	0	0	0	0	0	0	2
1	0	0	0	0	0	0	2	0	0	0	5	0	2
0	1	0	0	0	0	0	0	0	0	0	1	0	0
0	1	1	2	2	9	9	0	2	1	0	0	0	5
7													
OTU914	0	0	0	0	0	0	0	0	1	0	0	0	0
0	0	0	0	0	0	0	0	0	0	0	0	0	0
0	0	0	0	0	0	0	0	0	0	0	0	0	0
0	0	0	0	0	0	0	0	0	0	0	0	0	0
0	0	0	0	0	0	0	0	0	0	0	0	0	0
0	0	0	0	0	0	0	0	0	0	0	0	0	0
0	0	0	0	0	1	0	0	0	0	0	0	0	0
0	2	0	0	3	6	0	0	0	0	0	0	0	4
0													
OTU12448	0	0	0	0	0	0	0	0	1	0	0	0	0
0	0	0	0	0	0	0	0	0	0	0	0	0	0
0	0	0	0	0	0	0	0	0	0	0	0	0	0

0 0 0 0 0 0 0 0 0 0 0 0 0 0

0 0 0 0 0 0 0 0 0 0 0 0 0 0

0 0 0 0 0 0 0 0 0 0 0 0 0 0

0 0 0 0 0 0 0 0 0 0 0 0 0 0

0 0 0 0 0 0 0 0 0 0 0 0 0 0

0

OTU15781 0 0 0 0 0 0 0 0 1 0 0 0 0

0 0 0 0 0 0 0 0 0 0 0 0 0 0

0 0 0 0 0 0 0 0 0 0 0 0 0 0

0 0 0 0 0 0 0 0 0 0 0 0 0 0

0 0 0 0 0 0 0 0 0 0 0 0 0 0

0 0 0 0 0 0 0 0 0 0 0 0 0 0

0 0 0 0 0 0 0 0 0 0 0 0 0 0

0 0 0 0 0 0 0 0 0 0 0 0 0 0

0

OTU3525 0 0 0 0 0 0 0 0 2 0 0 0 0

0 0 0 0 0 0 0 0 0 0 0 0 0 1

0 0 0 0 0 0 2 0 0 0 5 0 3 0

0 0 0 0 0 0 0 0 0 0 0 0 0 0

0 0 0 0 0 0 0 0 0 0 0 0 0 0

0 0 0 0 0 0 0 0 0 0 0 0 0 0

0 0 0 0 0 0 0 0 0 0 0 0 0 0

0 0 0 0 0 0 0 0 1 0 0 0 0 0

0

OTU12449 0 0 0 0 0 0 0 0 1 0 0 0 0

0 0 0 0 0 0 0 0 0 0 0 0 0 0

0 0 0 0 0 0 0 0 0 0 0 0 0 0

0 0 0 0 0 0 0 0 0 0 0 0 0 0

0 0 0 0 0 0 0 0 0 0 0 0 0 0

0 0 0 0 0 0 0 0 0 0 0 0 0 0

0 0 0 0 0 0 0 0 0 0 0 0 0 0

0 0 0 0 0 0 0 0 0 0 0 0 0 0

0

OTU12447	0	0	0	0	0	0	0	0	1	0	0	0	0
0	0	0	0	0	0	0	0	0	0	0	0	0	0
0	0	0	0	0	0	0	0	0	0	0	0	0	0
0	0	0	0	0	0	0	0	0	0	0	0	0	0
0	0	0	0	0	0	0	0	0	0	0	0	0	0
0	0	0	0	0	0	0	0	0	0	0	0	0	0
0	0	0	0	0	0	0	0	0	0	0	0	0	0
0	0	0	0	0	0	0	0	0	0	0	0	0	0
0													
OTU9276	0	0	0	0	0	0	0	0	1	0	0	0	0
2	0	0	0	1	1	0	1	0	0	0	0	0	0
0	0	1	0	0	0	0	2	0	0	0	0	0	0
0	0	1	0	0	0	0	0	1	0	0	0	0	0
1	0	1	0	0	0	0	0	0	0	1	0	0	0
0	0	0	0	0	0	0	0	0	0	0	0	0	0
1	0	0	0	0	0	0	0	1	0	0	0	0	0
0	0	0	0	0	0	0	0	0	0	0	0	0	0
0													
OTU14536	0	0	0	0	0	0	0	0	1	0	0	0	0
0	0	0	0	0	0	0	0	0	0	0	0	0	0
0	0	0	0	0	0	0	0	0	0	0	0	0	0
0	0	0	0	0	0	0	0	0	0	0	0	0	0
0	0	0	0	0	0	0	0	0	0	0	0	0	0
0	0	0	0	0	0	0	0	0	0	0	0	0	0
0	0	0	0	0	0	0	0	0	0	0	0	0	0
0	0	0	0	0	0	0	0	0	0	0	0	0	0
0													
OTU14535	0	0	0	0	0	0	0	0	1	0	0	0	0
0	0	0	0	0	0	0	0	0	0	0	0	0	0
0	0	0	0	0	0	0	0	0	0	0	0	0	0
0	0	0	0	0	0	0	0	0	0	0	0	0	0
0	0	0	0	0	0	0	0	0	0	0	0	0	0
0	0	0	0	0	0	0	0	0	0	0	0	0	0

0 0 0 0 0 0 0 0 0 0 0 0 0 0
0 0 0 0 0 0 0 0 0 0 0 0 0 0
0
OTU9768 0 0 0 0 0 0 0 0 1 0 0 0 0
0 0 0 0 0 0 0 0 0 0 0 0 0 0
0 0 0 0 0 0 0 0 0 0 0 0 0 0
0 0 0 0 0 0 0 0 0 0 0 0 0 0
0 0 0 0 0 0 0 0 0 0 0 0 0 0
0 0 0 0 0 0 0 0 1 0 0 0 0 0
0 0 0 0 0 0 0 0 0 0 0 0 1 0
0 0 0 0 0 0 0 0 0 0 0 0 0 0
0
OTU7844 0 0 0 0 0 0 0 0 1 0 0 0 0
0 0 0 0 0 0 0 0 0 0 0 0 0 0
0 0 0 0 0 0 0 0 0 0 0 0 0 0
0 0 0 0 0 0 0 0 0 0 0 0 0 0
0 0 0 0 0 0 0 0 0 0 0 0 0 0
0 0 0 0 0 0 0 0 0 0 0 0 0 0
0 0 0 0 0 0 0 0 0 0 0 0 0 0
0 0 0 0 0 0 0 0 0 0 0 0 0 0
0
OTU15784 0 0 0 0 0 0 0 0 1 0 0 0 0
0 0 0 0 0 0 0 0 0 0 0 0 0 0
0 0 0 0 0 0 0 0 0 0 0 0 0 0
0 0 0 0 0 0 0 0 0 0 0 0 0 0
0 0 0 0 0 0 0 0 0 0 0 0 0 0
0 0 0 0 0 0 0 0 0 0 0 0 0 0
0 0 0 0 0 0 0 0 0 0 0 0 0 0
0 0 0 0 0 0 0 0 0 0 0 0 0 0
0
OTU12445 0 0 0 0 0 0 0 0 1 0 0 0 0
0 0 0 0 0 0 0 0 0 0 0 0 0 0
0 0 0 0 0 0 0 0 0 0 0 0 0 0

0	0	0	0	0	0	0	0	0	0	0	0	0	0
0	0	0	0	0	0	0	0	0	0	0	0	0	0
0	0	0	0	0	0	0	0	0	0	0	0	0	0
0	0	0	0	0	0	0	0	0	0	0	0	0	0
0	0	0	0	0	0	0	0	0	0	0	0	0	0
0													
OTU4623	0	0	0	0	0	0	0	0	1	0	0	0	0
0	0	0	1	0	0	0	0	0	0	0	0	0	0
0	0	0	0	0	0	0	0	0	0	1	0	0	0
0	0	0	0	0	0	0	0	0	0	0	0	0	0
0	0	0	0	0	0	0	0	0	0	0	0	0	0
0	0	0	0	0	0	0	0	0	0	0	0	0	0
0	0	0	0	0	0	0	0	0	0	0	0	0	0
0	0	2	1	3	6	13	1	2	0	0	0	0	1
0													
OTU7849	0	0	0	0	0	0	0	0	1	0	0	0	0
0	0	0	0	0	0	0	0	0	0	0	0	0	0
0	0	0	0	0	0	0	0	0	0	0	0	0	0
0	0	0	0	0	0	0	0	0	0	0	0	0	0
0	0	0	0	0	0	0	0	0	0	0	0	0	0
0	0	0	0	0	0	0	0	0	0	0	0	0	0
0	0	0	0	0	0	0	0	0	0	0	0	0	0
0	0	0	0	0	0	0	0	0	0	0	0	0	0
0													
OTU10469	0	0	0	0	0	0	0	0	1	0	0	0	0
0	0	0	0	1	0	0	0	0	0	0	0	0	0
0	0	0	0	0	0	0	0	0	0	0	0	0	0
0	0	0	0	0	0	0	0	0	0	0	0	0	0
0	0	0	0	0	0	0	0	0	0	0	0	1	0
0	0	0	0	0	0	0	0	0	0	0	0	0	0
0	0	0	0	0	0	0	0	0	0	0	0	0	0
0	0	0	0	0	0	0	0	0	0	0	0	0	0
0													

OTU15782 0 0 0 0 0 0 0 0 1 0 0 0 0
0 0 0 0 0 0 0 0 0 0 0 0 0 0
0 0 0 0 0 0 0 0 0 0 0 0 0 0
0 0 0 0 0 0 0 0 0 0 0 0 0 0
0 0 0 0 0 0 0 0 0 0 0 0 0 0
0 0 0 0 0 0 0 0 0 0 0 0 0 0
0 0 0 0 0 0 0 0 0 0 0 0 0 0
0 0 0 0 0 0 0 0 0 0 0 0 0 0
0
OTU15783 0 0 0 0 0 0 0 0 1 0 0 0 0
0 0 0 0 0 0 0 0 0 0 0 0 0 0
0 0 0 0 0 0 0 0 0 0 0 0 0 0
0 0 0 0 0 0 0 0 0 0 0 0 0 0
0 0 0 0 0 0 0 0 0 0 0 0 0 0
0 0 0 0 0 0 0 0 0 0 0 0 0 0
0 0 0 0 0 0 0 0 0 0 0 0 0 0
0 0 0 0 0 0 0 0 0 0 0 0 0 0
0
OTU791 0 0 0 0 0 0 0 0 1 1 0 0 0
0 0 0 0 0 0 1 0 0 0 0 0 0 0
0 0 0 0 1 0 0 0 0 0 0 0 0 0
0 0 0 0 0 0 0 0 0 0 0 0 0 0
0 0 0 0 0 0 0 0 0 0 0 0 0 0
0 0 0 0 0 0 0 1 0 0 0 1 0 0
0 0 1 0 0 0 0 0 0 0 0 0 0 0
0 0 0 0 0 0 0 0 0 0 0 0 0 0
0
OTU13763 0 0 0 0 0 0 0 0 1 1 0 0 0
0 1 1 0 0 0 0 0 1 0 0 0 2 0
2 0 0 0 0 0 0 1 1 0 4 0 4 0
8 2 0 0 1 0 0 0 0 0 1 0 0 2
0 3 0 2 0 0 0 0 0 0 0 0 0 0
0 3 0 0 0 0 0 0 0 3 0 0 0 1

0	0	0	1	0	0	0	2	0	0	1	1	4	0
0	2	1	3	1	0	0	0	1	3	1	0	0	3
0													
OTU1450	0	0	0	0	0	0	0	0	1	0	0	0	0
0	0	0	0	0	0	0	0	0	0	0	0	0	0
1	2	0	0	0	0	0	0	0	0	0	0	0	0
2	0	0	0	1	0	0	0	1	1	8	7	0	0
1	1	0	0	2	3	0	6	2	0	0	0	2	0
1	0	0	0	0	0	0	0	0	0	0	4	0	0
0	0	0	0	0	0	0	3	0	0	0	0	1	0
0	0	0	0	1	5	3	0	0	1	0	0	0	0
0													
OTU14531	0	0	0	0	0	0	0	0	1	0	0	0	0
0	0	0	0	0	0	0	0	0	0	0	0	0	0
0	0	0	0	0	0	0	0	0	0	0	0	0	0
0	0	0	0	0	0	0	0	0	0	0	0	0	0
0	0	0	0	0	0	0	0	0	0	0	0	0	0
0	0	0	0	0	0	0	0	0	0	0	0	0	0
0	0	0	0	0	0	0	0	0	0	0	0	0	0
0	0	0	0	0	0	0	0	0	0	0	0	0	0
0													
OTU7850	0	0	0	0	0	0	0	0	1	0	0	0	0
0	0	0	0	0	0	0	0	0	0	0	0	0	0
0	0	0	0	0	0	0	0	0	0	0	0	0	0
0	0	0	0	0	0	0	0	0	0	0	0	0	0
0	0	0	0	0	0	0	0	0	0	0	0	0	0
0	0	0	0	0	0	0	0	0	0	0	0	0	0
0	0	0	0	0	0	0	0	0	0	0	0	0	0
0	0	0	0	0	0	0	0	0	0	0	0	0	0
0													
OTU7458	0	0	0	0	0	0	0	0	1	0	0	0	0
0	0	0	0	0	0	0	0	0	0	0	0	0	0
0	0	0	0	0	0	0	0	0	0	0	0	0	0

0	0	0	0	0	0	0	0	0	0	0	0	0	0
0	0	0	0	0	0	0	0	0	0	0	0	0	0
0	0	0	0	0	0	0	0	0	0	0	0	0	0
0	1	0	0	0	0	0	1	0	0	0	0	0	0
0	0	0	0	0	0	0	0	0	0	0	0	0	0
0													
OTU12444	0	0	0	0	0	0	0	0	1	0	0	0	0
0	0	0	0	0	0	0	0	0	0	0	0	0	0
0	0	0	0	0	0	0	0	0	0	0	0	0	0
0	0	0	0	0	0	0	0	0	0	0	0	0	0
0	0	0	0	0	0	0	0	0	0	0	0	0	0
0	0	0	0	0	0	0	0	0	0	0	0	0	0
0	0	0	0	0	0	0	0	0	0	0	0	0	0
0	0	0	0	0	0	0	0	0	0	0	0	0	0
0													
OTU716	0	0	0	0	0	0	0	0	1	0	0	0	0
0	0	0	0	0	0	0	0	0	0	0	0	0	0
0	0	0	0	0	0	0	0	0	0	0	0	0	0
0	0	0	0	0	0	0	0	0	0	0	0	0	0
0	0	0	0	0	0	0	0	0	0	0	0	0	0
0	0	0	0	0	0	0	0	0	0	0	0	0	0
0	0	0	0	0	0	0	0	0	0	0	0	0	0
0	0	0	0	3	2	0	2	1	0	0	0	0	0
0													
OTU14529	0	0	0	0	0	0	0	0	1	0	0	0	0
0	0	0	0	0	0	0	0	0	0	0	0	0	0
0	0	0	0	0	0	0	0	0	0	0	0	0	0
0	0	0	0	0	0	0	0	0	0	0	0	0	0
0	0	0	0	0	0	0	0	0	0	0	0	0	0
0	0	0	0	0	0	0	0	0	0	0	0	0	0
0	0	0	0	0	0	0	0	0	0	0	0	0	0
0	0	0	0	0	0	0	0	0	0	0	0	0	0
0													

OTU2903	0	0	0	0	0	0	0	0	1	1	0	0	0
0	0	0	0	0	0	0	0	0	0	0	0	1	0
0	0	0	0	0	0	0	0	0	0	0	0	1	0
0	0	0	0	0	0	0	0	0	0	0	0	0	0
0	0	0	0	0	0	0	0	0	0	0	0	0	0
0	1	0	0	0	0	0	0	0	0	0	2	0	0
0	0	0	0	0	0	0	0	0	0	0	3	18	0
0	3	2	5	2	6	23	0	0	6	0	0	0	4
1													
OTU4692	0	0	0	0	0	0	0	0	1	0	0	0	0
0	0	1	0	0	0	0	1	0	1	0	0	0	0
0	0	0	0	0	1	0	0	0	0	0	0	0	0
0	2	0	0	0	0	0	0	0	0	1	2	0	0
0	0	0	0	0	0	0	0	0	0	0	0	0	0
0	0	0	0	0	0	0	0	1	0	0	0	0	0
0	0	0	0	0	0	0	0	0	0	0	0	0	0
0	0	0	0	0	0	0	0	0	0	0	0	0	0
0													
OTU14530	0	0	0	0	0	0	0	0	1	0	0	0	0
0	0	0	0	0	0	0	0	0	0	0	0	0	0
0	0	0	0	0	0	0	0	0	0	0	0	0	0
0	0	0	0	0	0	0	0	0	0	0	0	0	0
0	0	0	0	0	0	0	0	0	0	0	0	0	0
0	0	0	0	0	0	0	0	0	0	0	0	0	0
0	0	0	0	0	0	0	0	0	0	0	0	0	0
0	1	0	0	0	0	0	0	0	0	0	0	0	0
0													
OTU5842	0	0	0	0	0	0	0	0	1	0	0	0	0
0	0	0	0	0	0	0	0	0	0	0	0	1	0
0	0	0	1	0	0	0	0	0	0	0	0	0	0
0	0	3	0	2	3	0	0	1	7	0	0	1	0
4	1	7	0	7	4	1	6	0	4	0	0	1	2
1	2	0	0	0	0	0	0	0	1	1	0	2	2

0	2	1	1	0	0	0	0	0	0	0	0	1	0
0	1	0	0	2	2	3	1	1	2	0	0	0	0
1													
OTU7848	0	0	0	0	0	0	0	0	1	0	0	0	0
0	0	0	0	0	0	0	0	0	0	0	0	0	0
0	0	0	0	0	0	0	0	0	0	0	0	0	0
0	0	0	0	0	0	0	0	0	0	0	0	0	0
0	0	0	0	0	0	0	0	0	0	0	0	0	0
0	0	0	0	0	0	0	0	0	0	0	0	0	0
0	0	0	0	0	0	0	0	0	0	0	0	0	0
0	0	0	0	0	0	0	0	0	0	0	0	0	0
0													
OTU3055	0	0	0	0	0	0	0	0	1	1	0	0	0
0	1	5	1	0	0	0	0	1	0	4	0	0	0
0	0	0	2	2	0	0	0	0	0	1	0	0	0
0	0	0	0	0	0	0	1	0	1	0	0	0	0
0	0	0	0	1	0	2	0	0	1	0	0	0	0
0	0	0	0	0	0	0	0	0	0	2	0	0	0
1	0	0	0	0	0	0	0	0	0	3	0	0	0
0	0	0	0	0	0	0	0	0	0	0	0	2	0
0													
OTU2270	0	0	0	0	0	0	0	0	1	0	0	0	0
0	0	0	0	0	0	0	0	0	0	0	0	0	0
0	0	0	0	0	0	0	0	0	0	0	0	0	0
0	0	0	0	0	0	0	0	0	0	0	0	0	0
0	0	0	0	0	0	0	0	0	0	0	0	1	0
0	0	0	0	0	0	0	0	0	0	0	1	0	0
0	0	0	0	0	0	0	0	0	0	0	0	0	0
0	0	0	0	0	1	0	0	0	0	0	0	0	0
0													
OTU15779	0	0	0	0	0	0	0	0	1	0	0	0	0
0	0	0	0	0	0	0	0	0	0	0	0	0	0
0	0	0	0	0	0	0	0	0	0	0	0	0	0

0 0 0 0 0 0 0 0 0 0 0 0 0 0
0 0 0 0 0 0 0 0 0 0 0 0 0 0
0 0 0 0 0 0 0 0 0 0 0 0 0 0
0 0 0 0 0 0 0 0 0 0 0 0 0 0
0 0 0 0 0 0 0 0 0 0 0 0 0 0
0
OTU12446 0 0 0 0 0 0 0 0 1 0 0 0 0
0 0 0 0 0 0 0 0 0 0 0 0 0 0
0 0 0 0 0 0 0 0 0 0 0 0 0 0
0 0 0 0 0 0 0 0 0 0 0 0 0 0
0 0 0 0 0 0 0 0 0 0 0 0 0 0
0 0 1 0 0 0 0 0 0 0 0 0 0 0
0 0 0 0 0 0 0 0 0 0 0 0 0 0
0 0 0 0 0 0 0 0 0 0 0 0 0 0
0
OTU15121 0 0 0 0 0 0 0 0 1 0 0 0 0
0 0 0 0 0 0 0 0 0 0 0 0 0 0
0 0 0 0 0 0 0 0 0 0 0 0 0 0
0 0 0 0 0 0 0 0 0 0 0 0 0 0
0 0 0 0 0 0 0 0 0 0 0 0 0 0
0 1 0 0 0 0 0 0 0 0 0 0 0 0
0 0 0 1 0 0 0 0 0 0 0 0 0 0
0 4 1 0 0 1 2 0 0 0 0 0 0 0
0
OTU14534 0 0 0 0 0 0 0 0 1 0 0 0 0
0 0 0 0 0 0 0 0 0 0 0 0 0 0
0 0 0 0 0 0 0 0 0 0 0 0 0 0
0 0 0 0 0 0 0 0 0 0 0 0 0 0
0 0 0 0 0 0 0 0 0 0 0 0 0 0
0 0 0 0 0 0 0 0 0 0 0 0 0 0
0 0 0 0 0 0 0 0 0 0 0 0 0 0
0 0 0 0 0 0 0 0 0 0 0 0 0 0
0

OTU1265	0	0	0	0	0	0	0	0	1	1	0	1	1
0	1	3	0	0	0	0	0	0	0	0	0	0	4
0	0	0	0	0	0	1	0	0	1	0	0	0	0
0	0	0	3	2	0	2	1	0	1	3	0	1	0
0	0	0	3	0	0	2	0	0	0	1	2	0	1
0	2	0	0	1	2	2	0	3	0	1	0	6	0
4	3	2	1	2	0	1	0	0	0	0	0	1	0
1	0	0	0	0	0	0	0	1	2	0	1	0	1
0													
OTU873	0	0	0	0	0	0	0	0	1	0	0	0	1
1	1	1	0	0	0	0	0	0	0	0	0	0	1
0	0	0	0	0	2	0	0	0	0	1	0	1	0
0	0	0	0	0	0	0	0	0	0	1	0	0	0
0	0	0	0	0	0	0	0	0	0	0	4	0	0
0	0	0	0	0	2	0	0	0	0	1	0	2	0
0	0	0	0	0	0	1	0	0	0	0	0	2	0
0	0	0	0	1	0	0	1	0	0	0	0	1	0
0													
OTU3891	0	0	0	0	0	0	0	0	1	0	3	1	3
7	0	2	0	0	0	0	4	0	1	0	1	0	0
0	0	1	1	0	0	0	1	0	1	0	0	0	1
0	1	0	0	0	1	0	0	0	0	2	0	1	0
0	0	0	0	0	0	0	0	0	0	0	2	0	0
0	0	0	0	0	1	1	0	3	1	0	0	1	0
0	0	0	0	0	0	4	1	2	0	0	0	0	1
1	0	0	0	0	0	0	0	2	0	1	2	0	0
0													
OTU9699	0	0	0	0	0	0	0	0	1	0	0	0	0
0	0	0	0	0	0	0	0	0	0	0	0	0	0
0	0	0	0	0	0	0	0	0	0	0	0	0	0
0	0	0	0	0	0	0	0	0	0	0	0	0	0
0	0	0	0	0	0	0	0	0	0	0	0	0	0
0	0	0	0	0	0	0	0	0	0	0	1	0	0

0	0	0	0	0	0	0	0	0	4	0	0	0	0
0	0	0	0	0	0	0	0	0	0	0	2	0	0
0													
OTU15778	0	0	0	0	0	0	0	0	1	0	0	0	0
1	0	0	0	0	0	0	1	0	0	0	0	0	0
0	0	0	0	0	0	0	0	0	0	0	0	0	0
0	0	0	0	0	0	0	0	0	0	0	0	0	0
0	0	0	0	0	0	0	0	0	0	0	0	0	0
0	0	0	0	0	0	0	0	0	0	0	0	0	0
0	0	0	0	0	0	0	0	0	0	0	0	0	0
0	0	0	0	0	0	0	0	0	0	0	0	0	0
0													
OTU14532	0	0	0	0	0	0	0	0	1	0	0	0	0
0	0	0	0	0	0	0	0	0	0	0	0	0	0
0	0	0	0	0	0	0	0	0	0	0	0	0	0
0	0	0	0	0	0	0	0	0	0	0	0	0	0
0	0	0	0	0	0	0	0	0	0	0	0	0	0
0	0	0	0	0	0	0	0	0	0	0	0	0	0
0	0	0	0	0	0	0	0	0	0	0	0	0	0
0	0	0	0	0	0	0	0	0	0	0	0	0	0
0													
OTU11076	0	0	0	0	0	0	0	0	1	1	0	0	0
0	0	0	0	0	0	0	0	0	0	0	0	0	0
0	0	0	0	0	0	0	0	0	0	0	0	0	0
2	0	0	0	0	0	0	0	0	0	0	0	0	0
0	0	0	0	0	0	0	0	0	0	0	0	0	0
0	0	0	0	0	0	0	0	0	0	0	0	0	0
0	0	0	1	0	0	0	0	0	0	0	0	2	0
0	0	0	0	0	0	0	1	0	0	0	0	0	0
0													
OTU14533	0	0	0	0	0	0	0	0	1	0	0	0	0
0	0	0	0	0	0	0	0	0	0	0	0	0	0
0	0	0	0	0	0	0	0	0	0	0	0	0	0

0	0	0	0	0	0	0	0	0	0	0	0	0	0
0	0	0	0	0	0	0	0	0	0	0	0	0	0
0	0	0	0	0	0	0	0	0	0	0	0	0	0
0	0	0	0	0	0	0	0	0	0	0	0	0	0
0	0	0	0	0	0	0	0	0	0	0	0	0	0
0													
OTU15776	0	0	0	0	0	0	0	0	2	0	0	0	0
0	0	0	0	0	0	0	0	0	0	0	0	0	0
0	0	0	0	0	0	0	0	0	0	0	0	0	0
1	0	0	0	0	0	0	0	0	0	0	0	0	0
0	0	0	0	0	0	0	0	0	0	0	0	0	0
0	0	0	0	0	0	0	0	0	0	0	0	0	0
0	0	0	0	0	0	0	0	0	0	0	0	0	0
0	0	0	0	0	0	0	0	0	0	0	0	0	0
0													
OTU3945	0	0	0	0	0	0	0	0	1	0	0	0	0
0	0	0	1	0	10	1	0	46	0	0	0	0	0
0	105	0	0	0	0	23	0	0	0	1	0	0	0
0	0	0	0	0	0	0	0	0	0	0	0	0	0
0	0	0	0	0	0	0	5	75	0	0	0	0	0
6	0	4	0	0	0	0	0	0	1	0	1	0	0
0	0	0	0	0	0	0	0	0	0	0	0	0	0
0	2	1	1	3	0	5	1	4	0	0	0	0	5
4													
OTU2189	0	0	0	0	0	0	0	0	2	0	0	0	0
0	0	0	0	0	0	0	0	0	0	0	0	0	0
0	0	0	0	0	0	0	0	0	0	0	0	0	0
0	0	0	0	0	0	0	0	0	0	0	0	0	0
0	0	0	0	0	0	0	0	0	0	0	0	0	0
0	0	1	0	0	0	0	0	0	0	0	0	0	0
0	0	0	0	0	0	0	0	0	0	0	0	0	0
0	0	0	0	0	0	0	0	0	0	0	0	0	0
0													

OTU2949	0	0	0	0	0	0	0	0	1	0	0	0	0
0	0	0	0	0	0	0	0	0	0	0	0	0	0
0	0	0	0	0	0	0	0	0	0	0	0	0	0
0	0	0	0	0	0	0	0	0	0	0	0	0	0
0	0	0	0	0	0	0	0	0	0	0	0	0	0
0	1	0	0	0	0	0	0	0	0	0	0	0	0
0	0	0	0	0	0	0	0	0	1	0	0	0	0
0	0	0	4	44	2	0	1	2	5	0	3	0	0
3													
OTU7858	0	0	0	0	0	0	0	0	1	0	0	0	0
0	0	0	0	0	0	0	0	0	0	0	0	0	0
0	0	0	0	0	0	0	0	0	0	0	0	0	0
0	0	0	0	0	0	0	0	0	0	0	0	0	0
0	0	0	0	0	0	0	0	0	0	0	0	0	0
0	0	0	0	0	0	0	0	0	0	0	0	0	0
0	0	0	0	0	0	0	0	0	0	0	0	0	0
0	0	0	0	0	0	0	0	0	0	0	0	0	0
0													
OTU15762	0	0	0	0	0	0	0	0	1	0	0	0	0
0	0	0	0	0	0	0	0	0	0	0	0	0	0
0	0	0	0	0	0	0	0	0	0	0	0	0	0
0	0	0	0	0	0	0	0	0	0	0	0	0	0
0	0	0	0	0	0	0	0	0	0	0	0	0	0
0	0	0	0	0	0	0	0	0	0	0	0	0	0
0	0	0	0	0	0	0	0	0	0	0	0	0	0
0	0	0	0	0	0	0	0	0	0	0	0	0	0
0													
OTU15764	0	0	0	0	0	0	0	0	1	0	0	0	0
0	0	0	0	0	0	0	0	0	0	0	0	0	0
0	0	0	0	0	0	0	0	0	0	0	0	0	0
0	0	0	0	0	0	0	0	0	0	0	0	0	0
0	0	0	0	0	0	0	0	0	0	0	0	0	0
0	0	0	0	0	0	0	0	0	0	0	0	0	0

0	0	0	0	0	0	0	0	0	0	0	0	0	0
0	0	0	0	0	0	0	0	0	0	0	0	0	0
0													
OTU12474	0	0	0	0	0	0	0	0	1	0	0	0	0
0	0	0	0	0	0	0	0	0	0	0	0	0	0
0	0	0	0	0	0	0	0	0	0	0	0	0	0
0	0	0	0	0	0	0	0	0	0	0	0	0	0
0	0	0	0	0	0	0	0	0	0	0	0	0	0
0	0	0	0	0	0	0	0	0	0	0	0	0	0
0	0	0	0	0	0	0	0	0	0	0	0	0	0
0	0	0	0	0	0	0	0	0	0	0	0	0	0
0													
OTU15763	0	0	0	0	0	0	0	0	1	0	0	0	0
0	0	0	0	0	0	0	0	0	0	0	0	0	0
0	0	0	0	0	0	0	0	0	0	0	0	0	0
0	0	0	0	0	0	0	0	0	0	0	0	0	0
0	0	0	0	0	0	0	0	0	0	0	0	0	0
0	0	0	0	0	0	0	0	0	0	0	0	0	0
0	0	0	0	0	0	0	0	0	0	0	0	0	0
0	0	0	0	0	0	0	0	0	0	0	0	0	0
0													
OTU7859	0	0	0	0	0	0	0	0	1	0	0	0	0
0	0	0	0	0	0	0	0	0	0	0	0	0	1
0	0	0	0	0	0	0	0	0	0	0	0	0	0
0	0	0	0	0	0	0	0	0	0	0	0	0	0
0	0	0	0	0	0	0	0	0	0	0	0	0	0
0	0	0	0	0	0	0	0	0	0	0	0	0	0
0	0	0	0	0	0	0	0	0	0	0	0	0	0
0	0	0	0	0	0	0	0	0	0	0	0	0	0
0													
OTU16048	0	0	0	0	0	0	0	0	1	0	0	0	0
0	0	0	0	0	0	0	0	0	0	0	0	0	0
0	0	0	0	0	0	0	0	0	0	1	0	1	0

0	0	0	0	0	0	0	0	0	0	0	0	0	1
0	0	0	0	0	0	0	0	0	0	0	0	0	0
0	0	0	0	0	0	0	0	0	0	0	0	0	0
0	0	0	0	0	0	0	0	0	0	0	0	0	0
0	0	0	0	0	0	0	0	0	0	0	0	0	0
0													
OTU8212	0	0	0	0	0	0	0	0	1	0	0	0	0
0	0	0	0	0	0	0	0	0	0	0	0	0	0
0	0	0	1	0	0	0	0	0	0	0	0	0	0
0	0	0	0	0	0	0	0	0	1	0	0	0	0
0	0	0	0	0	0	0	0	0	0	0	0	0	0
0	0	0	0	0	0	0	0	0	1	0	0	0	0
1	0	0	0	0	0	1	0	0	0	0	0	0	0
0	0	0	0	0	0	0	0	0	1	0	0	0	0
1													
OTU317	0	0	0	0	0	0	0	0	1	0	0	0	0
0	0	1	0	0	0	0	3	0	54	0	0	0	12
0	0	13	74	0	0	0	0	0	1	0	0	0	0
0	0	0	0	1	0	0	0	0	0	0	0	0	0
0	1	0	0	0	0	0	0	0	0	0	0	0	0
0	0	0	0	0	0	0	0	0	0	0	0	0	0
0	0	0	0	0	0	0	0	0	0	0	0	0	0
0	0	0	0	0	0	0	0	0	0	0	0	0	0
0													
OTU7851	0	0	0	0	0	0	0	0	1	0	0	0	0
0	0	0	0	0	0	0	0	0	0	0	0	0	0
0	0	0	0	0	0	0	0	0	0	0	0	0	0
0	0	0	0	0	0	0	0	0	0	0	0	0	0
0	0	0	0	0	0	0	0	0	0	0	0	0	0
0	0	0	0	0	0	0	0	0	0	0	0	0	0
0	0	0	0	0	0	0	0	0	0	0	0	0	0
0	0	0	0	0	0	0	0	0	0	0	0	0	0
0													

OTU1516 0 0 0 0 0 0 0 0 1 0 0 0 0
0 0 0 0 0 0 0 4 0 0 0 0 0 0
0 0 0 0 0 0 2 0 0 0 0 0 0 0
0 0 2 0 0 0 0 0 0 0 0 0 0 0
0 0 0 0 0 1 0 0 0 0 0 0 0 0
0 0 0 0 0 0 0 5 0 0 0 0 3 0
0 1 0 0 1 0 1 0 0 0 0 0 0 0
0 0 0 0 0 0 0 0 0 0 0 0 0 0
0
OTU12471 0 0 0 0 0 0 0 0 1 0 0 0 0
0 0 0 0 0 0 0 0 0 0 0 0 0 0
0 0 0 0 0 0 0 0 0 0 0 0 0 0
0 0 0 0 0 0 0 0 0 0 0 0 0 0
0 0 0 0 0 0 0 0 0 0 0 0 0 0
0 0 0 0 0 0 0 0 0 0 0 0 0 0
0 0 0 0 0 0 0 0 0 0 0 0 0 0
0 0 0 0 0 0 0 0 0 0 0 0 0 0
0
OTU5411 0 0 0 0 0 0 0 0 1 0 0 0 0
0 0 0 0 0 0 0 0 0 0 0 0 0 0
0 0 0 0 0 0 0 0 0 1 1 0 0 0
0 0 0 0 0 0 0 0 0 0 0 0 0 0
0 0 0 0 0 0 0 0 0 0 0 1 0 0
0 0 0 0 0 1 0 0 1 0 0 0 0 0
0 0 0 0 0 0 0 0 0 0 0 0 1 0
0 0 0 0 0 0 0 0 0 0 0 0 0 0
0
OTU7856 0 0 0 0 0 0 0 0 1 0 0 0 0
0 0 0 0 0 0 0 0 0 0 0 0 0 0
0 0 0 0 0 0 0 0 0 0 0 0 0 0
0 0 0 0 0 0 0 0 0 0 0 0 0 0
0 0 0 0 0 0 0 0 0 0 0 0 0 0
0 0 0 0 0 0 0 0 0 0 0 0 0 0

0	0	0	0	0	0	0	0	0	0	0	0	0	0
0	1	0	0	0	0	0	0	0	0	0	0	0	0
0													
OTU1036	0	0	0	0	0	0	0	0	2	2	0	0	0
0	0	0	0	0	0	0	0	0	0	0	0	0	0
0	0	0	0	0	0	0	0	0	0	0	0	0	0
0	0	0	0	0	0	0	0	0	0	0	0	0	0
0	0	0	0	0	0	0	0	0	0	0	0	0	0
0	0	0	1	0	0	0	0	0	1	0	0	0	0
0	0	0	0	0	0	0	0	0	0	0	0	0	0
0	0	0	0	0	0	0	0	0	0	0	0	0	0
0													
OTU14537	0	0	0	0	0	0	0	0	1	0	0	0	0
0	0	0	0	0	0	0	0	0	0	0	0	0	0
0	0	0	0	0	0	0	0	0	0	0	0	0	0
0	0	0	0	0	0	0	0	0	0	0	0	0	0
0	0	0	0	0	0	0	0	0	0	0	0	0	0
0	0	0	0	0	0	0	0	0	0	0	0	0	0
0	0	0	0	0	0	0	0	0	0	0	0	0	0
0	0	0	0	0	0	0	0	0	0	0	0	0	0
0													
OTU16140	0	0	0	0	0	0	0	0	1	1	0	0	0
0	0	0	0	0	0	0	0	0	0	0	0	1	0
0	0	0	0	0	0	1	0	0	0	0	0	1	0
0	0	0	0	0	0	0	0	1	0	0	0	0	1
0	0	0	0	0	0	0	0	0	0	0	0	0	0
0	1	0	0	0	0	0	0	0	0	0	0	0	0
0	0	1	0	0	0	0	0	1	2	0	0	0	0
0	1	0	0	0	0	0	1	0	0	0	0	0	0
1													
OTU3416	0	0	0	0	0	0	0	0	2	0	0	0	0
0	0	0	0	0	0	0	1	0	0	0	0	1	0
0	0	0	0	0	0	3	0	0	0	13	0	0	0

0 0 0 0 0 0 0 1 0 1 0 3 0 0
0 0 0 2 1 8 0 0 0 1 1 0 8 1
0 3 0 0 0 0 0 0 0 0 0 4 0 0
0 6 0 0 0 1 0 6 0 0 0 0 0 0
0 0 2 2 0 0 5 2 1 2 0 0 0 3
3
OTU7855 0 0 0 0 0 0 0 0 1 0 0 0 0
0 0 0 0 0 0 0 0 0 0 0 0 0 0
0 0 0 0 0 0 0 0 0 0 0 0 0 0
0 0 0 0 0 0 0 0 0 0 0 0 0 0
0 0 0 0 0 0 0 0 0 0 0 0 0 0
0 0 0 0 0 0 0 0 0 0 0 0 0 0
0 0 0 0 0 0 0 0 0 0 0 0 0 0
0 0 0 0 0 0 0 0 0 0 0 0 0 0
0
OTU12472 0 0 0 0 0 0 0 0 2 0 0 0 0
0 0 0 0 0 0 0 0 0 0 0 0 0 0
0 0 0 0 0 0 0 0 0 0 0 0 0 0
0 0 0 0 0 0 0 0 0 0 0 0 0 0
0 0 0 0 0 0 0 0 0 0 0 0 0 0
0 0 0 0 0 0 0 0 0 0 0 0 0 0
0 0 0 0 0 0 0 0 0 0 0 0 0 0
0 0 0 0 0 0 0 0 0 0 0 0 0 0
0
OTU678 0 0 0 0 0 0 0 0 1 0 0 0 0
0 0 0 0 0 0 2 0 0 0 0 0 0 0
0 0 0 0 0 0 0 0 0 0 0 0 0 0
0 0 0 0 0 0 0 0 0 0 0 0 0 0
0 0 0 0 0 0 0 0 0 0 0 0 0 0
0 0 0 0 1 0 0 0 0 0 1 1 1 0
0 0 0 0 0 0 1 0 0 0 0 1 0 0
0 0 0 2 3 1 1 0 1 2 0 0 0 3
0

OTU6235	0	0	0	0	0	0	0	0	1	0	1	0	0
0	0	0	0	0	0	0	0	0	0	0	0	0	0
0	0	0	0	0	0	0	0	0	0	0	1	0	0
0	0	0	0	0	0	0	1	0	0	0	0	0	0
0	0	0	0	0	0	0	0	0	0	0	0	0	0
0	0	0	0	0	0	0	0	0	0	0	0	0	0
0	0	0	0	0	0	0	0	0	0	0	0	0	0
0	0	0	0	0	0	0	0	0	0	0	0	0	0
0													
OTU792	0	0	0	0	0	0	0	0	1	0	0	0	0
0	0	0	0	0	0	0	0	0	0	0	0	0	0
0	0	0	0	0	0	0	0	0	0	0	0	0	0
0	0	0	0	1	0	0	0	1	8	0	0	0	0
4	0	5	1	4	3	1	2	0	1	0	0	1	5
1	0	0	1	1	0	0	0	0	0	0	0	0	4
0	1	0	0	0	0	0	1	1	0	0	0	0	1
0	1	0	0	0	0	1	5	1	4	0	0	0	0
0													
OTU15761	0	0	0	0	0	0	0	0	1	0	0	0	0
0	0	0	0	0	0	0	0	0	0	0	0	0	0
0	0	0	0	0	0	0	0	0	0	0	0	0	0
0	0	0	0	0	0	0	0	0	0	0	0	0	0
0	0	0	0	0	0	0	0	0	0	0	0	0	0
0	0	0	0	0	0	0	0	0	0	0	0	0	0
0	0	0	0	0	0	0	0	0	0	0	0	0	0
0	0	0	0	0	0	0	0	0	0	0	0	0	0
0													
OTU1293	0	0	0	0	0	0	0	0	1	0	0	0	0
0	0	0	0	0	1	0	0	0	0	0	0	0	0
0	0	0	0	0	0	0	0	0	0	0	0	0	0
0	0	0	0	0	0	0	0	0	0	0	0	0	0
0	0	0	0	0	0	0	0	0	0	0	0	0	0
0	0	0	0	0	1	0	0	0	0	0	0	0	0

0	0	0	0	0	0	0	1	0	0	0	0	0	0
0	0	0	0	0	0	0	0	0	0	1	0	0	0
0													
OTU7845	0	0	0	0	0	0	0	0	1	0	0	0	0
0	0	0	0	0	0	0	0	0	0	0	0	0	0
0	0	0	0	0	0	0	0	0	0	0	0	0	0
0	0	0	0	0	0	0	0	0	0	0	0	0	0
0	0	0	0	0	0	0	0	0	0	0	0	0	0
0	0	0	0	0	0	0	0	0	0	0	0	0	0
0	0	0	0	0	0	0	0	0	0	0	0	0	0
0	0	0	0	0	0	0	0	0	0	0	0	0	0
0													
OTU15760	0	0	0	0	0	0	0	0	1	0	0	0	0
0	0	0	0	0	0	0	0	0	0	0	0	0	0
0	0	0	0	0	0	0	0	0	0	0	0	0	0
0	0	0	0	0	0	0	0	0	0	0	0	0	0
0	0	0	0	0	0	0	0	0	0	0	0	0	0
0	0	0	0	0	0	0	0	0	0	0	0	0	0
0	0	0	0	0	0	0	0	0	0	0	0	0	0
0	0	0	0	0	0	0	0	0	0	0	0	0	0
0													
OTU4747	0	0	0	0	0	0	0	0	1	0	0	0	0
0	0	0	1	0	0	0	0	0	0	0	0	0	0
0	1	0	0	0	0	0	0	0	0	0	0	0	0
0	0	0	0	1	0	0	0	5	1	1	0	1	0
1	0	0	0	1	8	0	3	6	0	6	0	0	0
1	0	0	0	0	0	0	5	0	0	0	4	0	0
0	5	0	0	0	0	0	1	0	0	0	0	0	0
0	0	0	0	0	0	0	0	0	0	0	0	0	0
0													
OTU15769	0	0	0	0	0	0	0	0	1	0	0	0	0
0	0	0	0	0	0	0	0	0	0	0	0	0	0
0	0	0	0	0	0	0	0	0	0	0	0	0	0

0	0	0	0	0	0	0	0	0	0	0	0	0	0
0	0	0	0	0	0	0	0	0	0	0	0	0	0
0	0	0	0	0	0	0	0	0	0	0	0	0	0
0	0	0	0	0	0	0	0	0	0	0	0	0	0
0	0	0	0	0	0	0	0	0	0	0	0	0	0
0													
OTU2750	0	0	0	0	0	0	0	0	2	0	0	0	3
1	0	0	0	0	0	0	1	0	56	0	3	0	0
0	0	6	0	0	2	0	0	0	0	0	2	0	0
0	1	2	0	0	0	47	0	0	0	0	0	0	0
0	0	0	0	0	0	0	0	0	0	0	0	0	0
0	0	0	0	0	2	7	0	0	0	9	0	3	0
4	0	5	0	0	0	0	0	10	0	1	0	0	0
1	0	0	0	0	0	0	0	0	0	0	0	0	0
0													
OTU16532	0	0	0	0	0	0	0	0	1	2	0	0	0
0	1	3	0	0	1	0	0	0	0	0	0	0	0
3	0	0	0	0	0	0	0	0	0	0	0	0	7
0	0	0	0	0	0	0	0	0	0	0	1	0	0
0	0	0	0	0	0	0	0	0	0	0	0	0	0
0	2	0	0	0	2	0	0	0	1	0	0	0	0
0	0	0	4	1	0	0	0	0	0	0	0	2	0
0	2	2	1	0	0	2	0	0	0	0	0	0	1
0													
OTU15770	0	0	0	0	0	0	0	0	1	0	0	0	0
0	0	0	0	0	0	0	0	0	0	0	0	0	0
0	0	0	0	0	0	0	0	0	0	0	0	0	0
0	0	0	0	0	0	0	0	0	0	0	0	0	0
0	0	0	0	0	0	0	0	0	0	0	0	0	0
0	0	0	0	0	0	0	0	0	0	0	0	0	0
0	0	0	0	0	0	0	0	0	0	0	0	0	0
0	0	0	0	0	0	0	0	0	0	0	0	0	0
0													

OTU837	0	0	0	0	0	0	0	0	1	0	0	0	0
0	0	0	0	0	0	0	0	0	7	0	0	0	11
0	0	0	7	0	0	0	0	0	0	0	0	0	0
0	0	0	0	0	0	0	0	0	0	0	0	0	0
0	0	0	0	0	0	0	0	0	0	0	0	0	0
0	0	0	0	0	0	0	0	0	0	0	0	0	0
0	0	0	0	0	0	0	0	0	0	0	0	0	0
0	0	0	0	0	0	0	0	0	0	0	0	0	0
0													
OTU14540	0	0	0	0	0	0	0	0	1	0	0	0	0
0	0	0	0	0	0	0	0	0	0	0	0	0	0
0	0	0	0	0	0	0	0	0	0	0	0	0	0
0	0	0	0	0	0	0	0	0	0	0	0	0	0
0	0	0	0	0	0	0	0	0	0	0	0	0	0
0	0	0	0	0	0	0	0	0	0	0	0	0	0
0	0	0	0	0	0	0	0	0	0	0	0	0	0
0	0	0	0	0	0	0	0	0	0	0	0	0	0
0													
OTU14489	0	0	0	0	0	0	0	0	1	0	0	0	0
0	0	0	0	0	0	0	0	0	0	0	0	0	0
0	0	0	0	0	0	0	0	0	0	0	0	0	0
0	0	0	0	0	0	0	0	0	0	0	0	0	0
0	0	0	0	0	0	0	0	0	0	0	0	0	0
0	0	0	0	0	0	0	0	0	0	0	0	0	0
0	0	0	1	0	0	0	0	0	0	0	0	0	0
0	0	0	0	0	0	0	0	0	0	0	0	0	0
0													
OTU2993	0	0	0	0	0	0	0	0	1	0	0	0	0
0	0	0	0	0	0	0	0	0	0	0	0	0	0
0	0	0	0	0	0	0	0	0	0	0	0	0	0
0	0	0	0	0	0	0	0	0	0	0	0	0	0
0	0	0	0	0	0	0	0	0	0	0	0	0	0
0	0	0	0	0	0	0	0	0	0	0	0	0	0

0	0	0	0	0	0	0	1	0	0	0	0	0	0
0	0	0	0	3	0	2	0	0	0	0	0	0	0
0													
OTU1025	0	0	0	0	0	0	0	0	1	0	0	0	0
0	0	0	0	0	0	0	0	0	0	0	0	0	0
0	0	0	0	0	0	0	0	0	0	0	0	0	0
0	0	0	0	0	0	0	0	0	0	0	0	0	0
0	0	0	0	0	0	0	0	0	0	0	0	0	0
0	0	0	0	0	0	0	0	0	0	0	0	0	0
0	0	0	0	0	0	0	0	0	0	0	0	0	0
0	0	0	0	0	6	2	1	0	0	0	0	0	0
0													
OTU15771	0	0	0	0	0	0	0	0	1	0	0	0	0
0	0	0	0	0	0	0	0	0	0	0	0	0	0
0	0	0	0	0	0	0	0	0	0	0	0	0	0
0	0	0	0	0	0	0	0	0	0	0	0	0	0
0	0	0	0	0	0	0	0	0	0	0	0	0	0
0	0	0	0	0	0	0	0	0	0	0	0	0	0
0	0	0	0	0	0	0	0	0	0	0	0	0	0
0	0	0	0	0	0	0	0	0	0	0	0	0	0
0													
OTU544	0	0	0	0	0	0	0	0	1	0	0	0	0
0	0	0	0	1	6	0	1	0	0	1	4	6	1
0	0	0	0	0	0	0	0	1	2	0	0	0	1
0	5	0	0	0	0	0	0	0	0	0	0	0	0
0	0	0	0	0	0	0	0	0	0	0	1	0	0
0	0	0	0	0	5	0	0	3	0	0	0	0	0
0	0	0	0	0	0	0	1	3	0	0	0	0	0
1	0	0	0	0	0	0	0	0	0	1	0	0	0
0													
OTU16495	0	0	0	0	0	0	0	0	1	1	0	0	0
0	0	0	0	0	0	0	0	0	0	0	0	0	0
0	0	0	0	0	0	0	0	0	0	1	0	0	0

5	0	0	1	0	4	0	0	0	0	0	0	0	0
0	0	0	0	0	0	0	0	0	0	0	0	0	0
0	1	0	0	0	0	0	0	0	0	0	0	0	1
0	0	0	0	0	0	0	0	0	0	0	0	0	0
0	0	0	2	1	0	2	0	0	0	0	0	0	0
0													
OTU10472	0	0	0	0	0	0	0	0	2	0	0	0	0
0	0	0	0	0	0	0	0	0	0	0	0	0	0
0	0	0	0	0	0	0	0	0	0	0	0	0	0
0	0	0	0	0	1	0	0	0	1	0	0	0	0
2	0	0	0	0	0	0	0	0	0	0	0	1	0
1	0	0	0	0	0	0	0	0	0	0	0	0	0
0	0	0	0	0	0	0	0	0	0	0	0	0	0
0	0	0	0	1	1	0	0	0	0	0	0	0	0
0													
OTU13909	0	0	0	0	0	0	0	0	1	0	3	0	0
2	0	0	0	1	9	0	0	0	0	0	0	0	0
0	0	10	1	0	1	0	0	0	0	0	0	0	5
0	0	4	0	3	0	0	0	1	0	0	0	0	0
0	0	0	0	0	1	0	3	0	0	2	0	0	0
0	0	0	0	0	0	0	0	0	0	0	0	0	0
0	0	0	0	0	0	0	0	6	0	0	0	0	1
0	0	0	0	0	0	0	0	0	0	0	0	0	0
0													
OTU10209	0	0	0	0	0	0	0	0	1	0	0	0	0
0	0	0	0	0	0	0	0	0	0	0	0	0	0
0	0	0	0	0	0	0	0	0	0	0	0	0	0
0	0	0	0	0	0	0	0	0	0	0	0	0	0
0	0	0	0	0	0	1	0	0	0	0	0	0	0
0	0	0	0	0	0	0	0	0	1	0	0	0	2
0	0	0	0	0	0	0	0	0	0	0	0	0	0
0	0	0	0	0	0	0	1	0	0	0	0	0	0
0													

OTU11719	0	0	0	0	0	0	0	0	1	0	0	0	0
0	0	0	0	2	0	0	0	0	0	0	0	0	0
0	0	0	0	0	0	0	0	0	0	1	0	0	0
0	0	0	0	0	0	0	0	0	0	0	0	0	0
0	0	0	0	0	0	0	0	0	0	0	0	1	0
0	0	0	0	1	0	0	1	0	0	0	0	0	0
0	0	0	0	0	0	0	0	0	0	0	0	0	0
0	0	0	0	0	0	0	0	0	0	0	0	0	0
1													
OTU13234	0	0	0	0	0	0	0	0	1	0	0	0	0
0	0	0	0	0	0	0	0	0	0	0	0	0	0
0	0	0	0	0	0	0	0	0	0	0	0	0	0
0	0	2	0	3	0	0	0	3	0	0	0	0	0
0	0	0	0	0	2	1	0	8	0	4	0	0	0
2	0	0	0	1	1	0	4	0	0	0	1	0	0
0	4	0	0	0	0	0	0	0	0	0	0	0	0
0	0	0	0	0	0	0	0	0	0	0	0	0	0
0													
OTU13820	0	0	0	0	0	0	0	0	1	0	0	0	0
0	0	0	0	0	1	0	1	0	0	1	0	0	0
0	0	0	1	0	0	0	0	2	0	0	0	0	1
0	0	0	1	0	0	0	0	0	2	0	0	0	0
0	0	1	1	1	0	0	0	1	0	0	0	0	0
1	0	1	0	1	0	0	0	1	0	0	0	0	1
0	0	0	0	0	0	0	0	0	0	0	1	0	0
0	0	0	0	0	0	0	1	0	0	1	1	1	0
0													
OTU996	0	0	0	0	0	0	0	0	1	0	0	0	0
0	0	0	0	0	0	0	1	0	1	0	0	0	1
0	0	0	3	0	0	0	0	0	0	0	0	0	0
0	0	0	0	0	0	0	0	0	0	0	0	0	0
0	0	0	0	0	0	0	0	0	0	0	0	0	0
0	0	0	0	0	0	0	0	0	0	0	0	0	0

0	0	0	0	0	0	0	0	0	0	0	0	0	0
0	0	0	0	0	0	0	0	0	0	0	0	0	0
0													
OTU13987	0	0	0	0	0	0	0	0	1	1	0	0	0
0	0	0	0	0	0	0	0	0	0	0	0	0	0
0	0	0	0	0	0	0	0	0	0	0	0	0	0
0	0	0	0	0	0	0	0	0	0	0	0	0	0
0	0	0	0	0	0	0	0	0	0	0	0	1	0
0	0	0	0	0	0	0	0	0	0	0	0	0	0
0	0	0	0	0	0	0	0	0	0	0	0	0	0
0	0	0	0	0	0	0	0	0	0	0	0	0	0
0													
OTU7847	0	0	0	0	0	0	0	0	1	0	0	0	0
0	0	0	0	0	0	0	0	0	0	0	0	0	0
0	0	0	0	0	0	0	0	0	0	0	0	0	0
0	0	0	0	0	0	0	0	0	0	0	0	0	0
0	0	0	0	0	0	0	0	0	0	0	0	0	0
0	0	0	0	0	0	0	0	0	0	0	0	0	0
0	0	0	0	0	0	0	0	0	0	0	0	0	0
0	0	0	0	0	0	0	0	0	0	0	0	0	0
0													
OTU14490	0	0	0	0	0	0	0	0	1	0	0	0	0
0	0	0	0	0	0	0	0	0	0	0	0	0	0
0	0	0	0	0	0	0	0	0	0	0	0	0	0
0	0	0	0	0	0	0	0	0	0	0	0	0	0
0	0	0	0	0	0	0	0	0	0	0	0	0	0
0	0	0	0	0	0	0	0	0	0	0	0	0	0
0	0	0	0	0	0	0	0	0	0	0	0	0	0
0	0	0	0	0	0	0	0	0	0	0	0	0	0
0													
OTU1150	0	0	0	0	0	0	0	0	2	2	0	0	0
0	0	0	0	0	0	2	0	0	0	0	0	0	0
9	0	0	0	0	0	0	0	0	0	0	0	0	0

1 0 0 0 0 0 2 2 0 0 0 0 0 0
0 0 0 5 0 0 0 0 0 0 0 0 1 0
0 17 0 0 1 0 0 0 0 4 1 23 0 0
2 0 0 3 0 4 0 0 0 0 0 3 5 0
0 19 20 84 15 8 8 11 3 8 0 1 0 20
12
OTU15765 0 0 0 0 0 0 0 0 1 0 0 0 0
0 0 0 0 0 0 0 0 0 0 0 0 0 0
0 0 0 0 0 0 0 0 0 0 0 0 0 0
0 0 0 0 0 0 0 0 0 0 0 0 0 0
0 0 0 0 0 0 0 0 0 0 0 0 0 0
0 0 0 0 0 0 0 0 0 0 0 0 0 0
0 0 0 0 0 0 0 0 0 0 0 0 0 0
0 0 0 0 0 0 0 0 0 0 0 0 0 0
0
OTU6920 0 0 0 0 0 0 0 0 2 0 0 0 0
0 0 0 0 0 0 0 0 0 0 0 0 0 0
0 0 0 0 0 0 0 0 0 0 0 0 0 0
0 0 0 0 0 0 0 0 0 0 0 0 0 0
0 0 0 0 0 0 0 0 0 0 0 0 0 0
0 0 0 0 0 0 0 0 0 0 0 0 0 0
0 0 0 0 0 0 0 0 0 0 0 0 0 0
0 1 0 0 0 0 0 0 0 0 0 0 0 0
0
OTU12460 0 0 0 0 0 0 0 0 1 1 0 0 1
0 0 0 0 0 0 0 0 0 0 0 0 0 0
0 0 0 0 0 0 0 0 0 0 0 0 0 0
0 0 0 0 0 0 0 0 0 0 0 0 0 0
0 0 0 0 0 1 0 0 0 0 0 1 0 0
0 0 0 0 0 0 0 0 0 0 0 0 0 0
0 0 0 0 0 0 0 0 0 0 0 0 0 0
0 0 0 0 0 0 0 0 1 0 0 0 0 0
0

OTU9695 0 0 0 0 0 0 0 0 1 0 0 0 0
0 0 0 0 0 0 0 0 0 0 0 0 0 0
0 0 0 0 0 0 0 0 0 0 0 0 0 0
0 0 0 0 0 0 0 0 0 0 0 0 0 0
0 0 0 0 0 0 0 0 0 0 0 0 0 0
0 0 0 0 0 0 0 0 0 0 0 1 0 0
0 0 0 0 0 0 0 0 0 0 0 0 0 0
0 0 0 0 0 0 0 0 0 0 0 0 0 0
0
OTU15766 0 0 0 0 0 0 0 0 1 0 0 0 0
0 0 0 0 0 0 0 0 0 0 0 0 0 0
0 0 0 0 0 0 0 0 0 0 0 0 0 0
0 0 0 0 0 0 0 0 0 0 0 0 0 0
0 0 0 0 0 0 0 0 0 0 0 0 0 0
0 0 0 0 0 0 0 0 0 0 0 0 0 0
0 0 0 0 0 0 0 0 0 0 0 0 0 0
0 0 0 0 0 0 0 0 0 0 0 0 0 0
0
OTU9934 0 0 0 0 0 0 0 0 1 0 1 0 0
0 1 0 0 0 0 0 1 0 0 0 0 0 0
0 0 0 0 0 0 0 0 0 0 0 0 0 0
0 0 0 0 0 0 0 0 0 0 0 0 0 0
0 0 0 0 0 0 0 0 0 0 1 0 0 0
0 0 0 0 0 0 0 0 0 0 0 0 0 1
0 0 0 0 0 0 0 0 0 0 0 0 0 0
0 0 0 0 0 0 0 0 0 0 0 0 0 0
1
OTU12461 0 0 0 0 0 0 0 0 1 0 0 0 0
0 0 0 0 0 0 0 0 0 0 0 0 0 0
0 0 0 0 0 0 0 0 0 0 0 0 0 0
0 0 0 0 0 0 0 0 0 0 0 0 0 0
0 0 0 0 0 0 0 0 0 0 0 0 0 0
0 0 0 0 0 0 0 0 0 0 0 0 0 0

0	0	0	0	0	0	0	0	0	0	0	0	0	0
0	0	0	0	0	0	0	0	0	0	0	0	0	0
0													
OTU15768	0	0	0	0	0	0	0	0	1	0	0	0	0
0	0	0	0	0	0	0	0	0	0	0	0	0	0
0	0	0	0	0	0	0	0	0	0	0	0	0	0
0	0	0	0	0	0	0	0	0	0	0	0	0	0
0	0	0	0	0	0	0	0	0	0	0	0	0	0
0	0	0	0	0	0	0	0	0	0	0	0	0	0
0	0	0	0	0	0	0	0	0	0	0	0	0	0
0	0	0	0	0	0	0	0	0	0	0	0	0	0
0													
OTU12459	0	0	0	0	0	0	0	0	1	0	0	0	0
0	0	0	0	0	0	0	0	0	0	0	0	0	0
0	0	0	0	0	0	0	0	0	0	0	0	0	0
0	0	0	0	0	0	0	0	0	0	0	0	0	0
0	0	0	0	0	0	0	0	0	0	0	0	0	0
0	0	0	0	0	0	0	0	0	0	0	0	0	0
0	0	0	0	0	0	0	0	0	0	0	0	0	0
0	0	0	0	0	0	0	0	0	0	0	0	0	0
0													
OTU15767	0	0	0	0	0	0	0	0	1	0	0	0	0
0	0	0	0	0	0	0	0	0	0	0	0	0	0
0	0	0	0	0	0	0	0	0	0	0	0	0	0
0	0	0	0	0	0	0	0	0	0	0	0	0	0
0	1	0	0	0	0	0	0	0	0	0	0	0	0
0	0	0	0	0	0	0	0	0	0	0	0	0	0
0	0	0	0	0	0	0	0	0	0	0	0	0	0
0	0	0	0	0	0	0	0	0	0	0	0	0	0
0													
OTU7846	0	0	0	0	0	0	0	0	1	0	0	0	0
0	0	0	0	0	0	0	0	0	0	0	0	0	0
0	0	0	0	0	0	0	0	0	0	0	0	0	0

0	0	0	0	0	0	0	0	0	0	0	0	0	0
0	0	0	0	0	0	0	0	0	0	0	0	0	0
0	0	0	0	0	0	0	0	0	0	0	0	0	0
0	0	0	0	0	0	0	0	0	0	0	0	0	0
0	0	0	0	0	0	0	0	0	0	0	0	0	0
0													
OTU15792	0	0	0	0	0	0	0	0	1	0	0	0	0
0	0	0	0	0	0	0	0	0	0	0	0	0	0
0	0	0	0	0	0	0	0	0	0	0	0	0	0
0	0	0	0	0	0	0	0	0	0	0	0	0	0
0	0	0	0	0	0	0	0	0	0	0	0	0	0
0	0	0	0	0	0	0	0	0	0	0	0	0	0
0	0	0	0	0	0	0	0	0	0	0	0	0	0
0	0	0	0	0	0	0	0	0	0	0	0	0	0
0													
OTU1034	0	0	0	0	0	0	0	0	2	0	0	0	0
0	0	0	0	0	0	0	0	0	0	0	0	0	0
0	0	0	0	0	0	0	0	0	0	0	0	0	0
0	0	0	0	0	0	0	0	0	0	0	0	0	0
0	0	0	0	0	0	0	0	0	0	0	0	0	0
0	0	0	0	0	0	0	0	0	0	0	0	0	0
0	0	0	0	0	0	0	0	0	0	0	0	0	0
0	0	0	0	0	0	0	0	0	0	0	0	0	0
0													
OTU12464	0	0	0	0	0	0	0	0	1	0	0	1	1
1	0	0	0	0	0	0	0	1	0	0	0	0	0
0	0	0	2	0	0	0	0	0	0	0	0	0	0
0	0	0	0	0	2	0	0	0	0	0	0	0	0
0	0	1	1	0	0	1	0	0	0	0	0	0	0
0	0	0	0	0	1	0	0	0	0	0	0	0	0
0	0	0	0	0	0	1	0	1	0	3	0	0	0
0	0	0	0	2	0	0	0	1	0	2	0	0	0
0													

OTU12463	0	0	0	0	0	0	0	0	1	0	0	0	0
0	0	0	0	0	0	0	0	0	0	0	0	0	0
0	0	0	0	0	0	0	0	0	0	0	0	0	0
0	0	0	0	0	0	0	0	0	0	0	0	0	0
0	0	0	0	0	0	0	0	0	0	0	0	0	0
0	0	0	0	0	0	0	0	0	0	0	0	0	0
0	0	0	0	0	0	0	0	0	0	0	0	0	0
0	0	0	0	0	0	0	0	0	0	0	0	0	0
0													
OTU9459	0	0	0	0	0	0	0	0	1	1	2	0	0
0	0	0	0	0	0	0	0	1	0	0	0	0	0
1	1	1	0	0	0	0	1	0	0	2	0	1	1
0	0	0	0	0	0	0	0	0	2	0	0	0	0
0	0	0	0	0	0	1	0	0	0	0	0	0	0
0	1	0	0	1	0	0	0	0	0	0	0	0	0
2	0	0	0	0	1	0	2	0	0	0	0	0	0
0	0	0	0	0	0	0	0	1	0	0	0	0	0
0													
OTU1074	0	0	0	0	0	0	0	0	1	0	0	0	0
0	0	0	0	0	0	0	0	0	0	0	0	0	0
0	0	0	0	0	0	0	0	0	0	0	0	0	0
1	0	0	0	0	0	0	0	0	0	0	0	0	0
0	0	0	0	0	0	0	0	0	0	0	0	0	0
0	1	0	0	0	0	0	0	0	0	0	0	0	1
0	0	0	0	0	0	0	0	0	0	0	0	0	0
0	0	3	0	1	0	0	0	1	1	0	0	0	1
0													
OTU14495	0	0	0	0	0	0	0	0	1	0	0	0	0
0	0	0	0	0	0	0	0	0	0	0	0	0	0
0	0	0	0	0	0	0	0	0	0	0	0	0	0
0	0	0	0	0	0	0	0	0	0	0	0	0	0
0	0	0	0	0	0	0	0	0	0	0	0	0	0
0	0	0	0	0	0	0	0	0	0	0	0	0	0

0	0	0	0	0	0	0	0	0	0	0	0	0	0
0	0	0	0	0	0	0	0	0	0	0	0	0	0
0													
OTU9180	0	0	0	0	0	0	0	0	1	0	0	0	0
0	0	0	0	0	0	0	0	0	0	0	0	0	5
0	0	0	0	0	0	0	0	0	0	0	0	0	0
0	0	0	0	0	0	0	0	0	0	0	0	0	0
0	3	0	0	0	0	0	0	0	0	0	0	0	0
2	7	0	0	2	0	0	5	0	2	0	6	0	0
0	9	0	2	0	0	0	0	0	0	0	1	2	0
0	22	2	30	28	11	1	3	10	2	0	1	0	7
43													
OTU12462	0	0	0	0	0	0	0	0	1	0	0	0	0
0	0	0	0	0	0	0	0	0	0	0	0	0	0
0	0	0	0	0	0	0	0	0	0	0	0	0	0
0	0	0	0	0	0	0	0	0	0	0	0	0	0
0	0	0	0	0	0	0	0	0	0	0	0	0	0
0	0	0	0	0	0	0	0	0	0	0	0	0	0
0	0	0	0	0	0	0	0	0	0	0	0	0	0
0	0	0	0	0	0	0	0	0	0	0	0	0	0
0													
OTU9939	0	0	0	0	0	0	0	0	1	0	0	0	0
0	0	0	0	0	0	0	0	0	0	0	0	0	0
0	0	0	0	0	0	0	0	0	0	0	0	0	0
0	0	0	0	0	0	0	0	0	0	0	0	0	0
0	0	0	0	0	0	0	0	0	0	0	0	0	0
0	0	0	0	0	0	0	0	0	0	0	0	0	0
2	0	0	0	0	0	1	0	0	0	0	0	0	0
0	0	0	0	0	0	0	0	0	0	0	0	0	0
0													
OTU7661	0	0	0	0	0	0	0	0	1	0	0	0	0
0	0	0	0	0	0	0	0	0	0	0	0	0	0
0	0	0	0	0	0	0	0	0	0	0	0	0	0

0	0	0	0	0	0	0	0	0	0	0	0	0	0
0	0	0	0	0	0	0	0	0	0	0	0	0	0
0	0	0	0	0	0	0	0	0	0	0	0	0	0
0	0	0	0	0	0	0	0	0	0	0	0	0	0
0	0	0	0	0	0	0	0	0	0	0	0	0	0
0													
OTU6576	0	0	0	0	0	0	0	0	1	0	0	0	1
0	0	0	0	0	0	0	0	0	0	0	0	0	0
0	0	0	0	0	0	0	0	0	0	0	0	0	0
2	0	0	2	0	1	0	1	0	0	0	1	1	0
0	0	0	0	0	0	2	0	0	0	0	0	1	0
0	0	0	0	0	0	0	0	0	0	0	0	0	0
0	0	0	0	0	0	0	0	0	0	0	0	0	0
0	0	1	0	3	2	0	0	0	0	0	0	0	0
0													
OTU15793	0	0	0	0	0	0	0	0	1	0	0	0	0
0	0	0	0	0	0	0	0	0	0	0	0	0	0
0	0	0	0	0	0	0	0	0	0	0	0	0	0
0	0	0	0	0	0	0	0	0	0	0	0	0	0
0	0	0	0	0	0	0	0	0	0	0	0	0	0
0	0	0	0	0	0	0	0	0	0	0	0	0	0
0	0	0	0	0	0	0	0	0	0	0	0	0	0
0	0	0	0	0	0	0	0	0	0	0	0	0	0
0													
OTU366	0	0	0	0	0	0	0	0	2	0	0	0	0
0	0	0	0	0	0	0	0	0	0	0	0	0	0
0	0	0	0	0	0	0	0	0	0	0	0	0	0
0	0	0	0	0	0	0	0	0	0	0	0	0	0
0	0	0	0	0	0	0	0	1	0	0	0	1	0
0	2	2	0	1	0	0	0	0	0	3	1	0	1
0	0	0	1	0	0	0	2	0	0	0	4	7	0
0	3	6	71	0	9	1	1	14	23	0	0	0	3
1													

OTU14499 0 0 0 0 0 0 0 0 1 0 0 0 0
0 0 0 0 0 0 0 0 0 0 0 0 0 0
0 0 0 0 0 0 0 0 0 0 0 0 0 0
0 0 0 0 0 0 0 0 0 0 0 0 0 0
0 0 0 0 0 0 0 0 0 0 0 0 0 0
0 0 0 0 0 0 0 0 0 0 0 0 0 0
0 0 0 0 0 0 0 0 0 0 0 0 0 0
0 0 0 0 0 0 0 0 0 0 0 0 0 0
0
OTU12454 0 0 0 0 0 0 0 0 1 0 0 0 0
0 0 0 0 0 0 0 0 0 0 0 0 0 0
0 0 0 0 0 0 0 0 0 0 0 0 0 0
0 0 0 0 0 0 0 0 0 0 0 0 0 0
0 0 0 0 0 0 0 0 0 0 0 0 0 0
0 0 0 0 0 0 0 0 0 0 0 0 0 0
0 0 0 0 0 0 0 0 0 0 0 0 0 0
0 0 0 0 0 0 0 0 0 0 0 0 0 0
0
OTU12455 0 0 0 0 0 0 0 0 1 0 0 0 0
0 0 0 0 0 0 0 0 0 0 0 0 0 0
0 0 0 0 0 0 0 0 0 0 0 0 0 0
0 0 0 0 0 0 0 0 0 0 0 0 0 0
0 0 0 0 0 0 0 0 0 0 0 0 0 0
0 0 0 0 0 0 0 0 0 0 0 0 0 0
0 0 0 0 0 0 0 0 0 0 0 0 0 0
0 0 0 0 0 0 0 0 0 0 0 0 0 0
0
OTU14498 0 0 0 0 0 0 0 0 1 0 0 0 0
0 0 0 0 0 0 0 0 0 0 0 0 0 0
0 0 0 0 0 0 0 0 0 0 0 0 0 0
0 0 0 0 0 0 0 0 0 0 0 0 0 0
0 0 0 0 0 0 0 0 0 0 0 0 0 0
0 0 0 0 0 0 0 0 0 0 0 0 0 0

0	0	0	0	0	0	0	0	0	0	0	0	0	0
0	0	0	0	0	0	0	0	0	0	0	0	0	0
0													
OTU14497	0	0	0	0	0	0	0	0	1	0	0	0	0
0	0	0	0	0	0	0	0	0	0	0	0	0	0
0	0	0	0	0	0	0	0	0	0	0	0	0	0
0	0	0	0	0	0	0	0	0	0	0	0	0	0
0	0	0	0	0	0	0	0	0	0	0	0	0	0
0	0	0	0	0	0	0	0	0	0	0	0	0	0
0	0	0	0	0	0	0	0	0	0	0	0	0	0
0	0	0	0	0	0	0	0	0	0	0	0	0	0
0													
OTU12456	0	0	0	0	0	0	0	0	1	0	0	0	0
0	0	0	0	0	0	0	0	0	0	0	0	0	0
0	0	0	0	0	0	0	0	0	0	0	0	0	0
0	0	0	0	0	0	0	0	0	0	0	0	0	0
0	0	0	0	0	0	0	0	0	0	0	0	0	0
0	0	0	0	0	0	0	0	0	0	0	0	0	0
0	0	0	0	0	0	0	0	0	0	0	0	0	0
0	0	0	0	0	0	0	0	0	0	0	0	0	0
0													
OTU12421	0	0	0	0	0	0	0	0	2	0	0	0	0
0	0	0	0	0	0	1	0	1	0	8	0	0	0
1	1	0	0	2	0	1	0	0	0	0	0	1	0
0	0	1	0	1	1	0	0	1	2	0	6	0	0
1	0	0	0	0	0	1	4	6	3	3	0	1	0
2	0	0	0	1	0	1	10	0	0	0	2	1	2
0	3	0	0	0	0	0	1	0	0	0	0	0	0
0	0	0	0	0	1	0	0	0	0	1	0	0	1
0													
OTU16350	0	0	0	0	0	0	0	0	1	1	0	0	0
0	0	0	0	0	0	0	0	0	0	0	0	0	0
0	0	0	0	0	0	0	0	0	2	0	0	0	0

1	0	0	0	0	0	0	0	0	0	0	0	0	0
0	0	0	1	0	0	0	0	0	0	0	0	1	0
0	0	0	0	0	0	0	0	0	0	0	1	0	0
0	0	0	0	0	0	0	0	0	0	0	3	3	0
0	9	2	2	7	17	1	5	1	3	0	0	0	1
0													
OTU14491	0	0	0	0	0	0	0	0	1	0	0	0	0
0	0	0	0	0	0	0	0	0	0	0	0	0	0
0	0	0	0	0	0	0	0	0	0	0	0	0	0
0	0	0	0	0	0	0	0	0	0	0	0	0	0
0	0	0	0	0	0	0	0	0	0	0	0	0	0
0	0	0	0	0	0	0	0	0	0	0	0	0	0
0	0	0	0	0	0	0	0	0	0	0	0	0	0
0	0	0	0	0	0	0	0	0	0	0	0	0	0
0													
OTU8853	0	0	0	0	0	0	0	0	1	0	0	1	0
0	0	1	0	0	0	1	0	0	0	0	0	0	0
5	0	0	1	0	0	0	0	1	0	0	0	0	0
0	8	0	3	0	0	0	0	0	0	0	1	0	0
0	0	0	2	0	0	0	0	0	0	0	0	3	1
0	0	0	0	0	0	0	0	0	0	0	0	0	0
0	3	1	1	6	2	0	0	0	1	0	0	0	0
0	0	0	0	0	0	0	0	0	0	0	0	0	0
0													
OTU348	0	0	0	0	0	0	0	0	1	0	0	0	0
1	0	0	0	0	0	0	0	0	0	0	0	0	0
0	0	0	0	0	0	66	0	0	0	0	0	0	0
0	0	0	0	0	0	0	0	0	0	0	119	0	0
0	0	0	0	0	0	0	0	0	0	0	1	0	0
0	0	0	0	0	1	0	0	0	0	0	0	0	1
0	0	0	0	0	0	0	6	0	0	0	0	1	0
0	5	4	0	2	2	12	6	1	5	19	0	0	0
0													

OTU15791	0	0	0	0	0	0	0	0	1	0	0	0	0
0	0	0	0	0	0	0	0	0	0	0	0	0	0
0	0	0	0	0	0	0	0	0	0	0	0	0	0
0	0	0	0	0	0	0	0	0	0	0	0	0	0
0	0	0	0	0	0	0	0	0	0	0	0	0	0
0	0	0	0	0	0	0	0	0	0	0	0	0	0
0	0	0	0	0	0	0	0	0	0	0	0	0	0
0	0	0	0	0	0	0	0	0	0	0	0	0	0
0													
OTU11943	0	0	0	0	0	0	0	0	1	0	0	0	0
0	1	0	0	0	0	0	0	0	0	0	0	2	0
0	0	0	0	0	0	0	0	0	0	0	0	0	0
0	0	0	0	0	0	0	0	0	0	0	0	0	0
0	0	0	0	0	0	0	0	0	0	0	0	0	0
0	0	0	0	0	0	0	0	0	0	0	0	0	0
0	0	0	0	0	0	0	0	0	0	0	0	0	0
0	0	0	0	0	0	0	0	0	0	0	0	0	0
0													
OTU14492	0	0	0	0	0	0	0	0	1	0	0	1	0
0	0	0	0	0	0	0	0	0	0	0	0	0	0
0	0	0	0	0	0	0	0	0	0	0	0	0	0
0	0	0	0	0	0	0	0	0	0	0	0	0	0
0	0	0	0	0	0	0	0	0	0	0	0	0	0
0	0	0	0	0	0	0	0	0	0	0	0	0	0
0	0	0	0	0	0	0	3	0	0	0	0	0	0
0	1	0	0	3	0	0	1	2	0	0	0	0	3
0													
OTU12470	0	0	0	0	0	0	0	0	1	0	0	0	0
0	0	0	0	0	0	0	0	0	0	0	0	0	0
0	0	0	0	0	0	0	0	0	0	0	0	0	0
0	0	0	0	1	0	0	0	0	1	0	0	0	0
0	0	0	0	0	0	0	0	0	0	0	0	1	0
0	0	0	0	0	0	0	1	0	0	0	0	0	0

0	1	0	0	0	0	0	0	0	0	0	0	0	0
0	0	0	0	0	0	0	0	0	0	0	0	0	0
0													
OTU2610	0	0	0	0	0	0	0	0	1	0	0	0	0
0	0	0	0	0	0	0	0	0	0	0	0	0	0
0	0	0	0	0	0	0	0	0	0	0	0	0	0
1	0	0	0	0	0	0	0	0	0	0	0	0	0
0	0	0	0	0	0	0	0	0	0	0	0	0	0
0	0	0	0	0	0	0	0	0	0	0	0	0	0
0	0	0	0	0	0	0	0	0	0	0	0	0	0
0	0	1	0	0	0	0	0	0	0	0	0	0	0
1													
OTU482	0	0	0	0	0	0	0	0	1	0	0	0	0
0	0	0	0	0	0	0	0	0	0	0	0	0	0
0	0	0	0	0	0	0	0	0	3	0	0	0	0
1	0	0	0	0	0	0	0	9	5	0	0	0	0
2	0	0	0	0	16	0	15	9	0	0	0	0	0
1	3	0	0	0	0	0	6	0	0	0	0	0	0
0	0	0	0	0	0	0	0	0	0	0	0	0	0
0	1	3	1	5	8	6	0	5	3	0	0	0	7
3													
OTU14494	0	0	0	0	0	0	0	0	1	0	0	0	0
0	0	0	0	0	0	0	0	0	0	0	0	0	0
0	0	0	0	0	0	0	0	0	0	0	0	0	0
0	0	0	0	0	0	0	0	0	0	0	0	0	0
0	0	0	0	0	0	0	0	0	0	0	0	0	0
0	0	0	0	0	0	0	0	0	0	0	0	0	0
0	0	0	0	0	0	0	0	0	0	0	0	0	0
0	0	0	0	0	0	0	0	0	0	0	0	0	0
0													
OTU14493	0	0	0	0	0	0	0	0	1	0	0	0	0
0	0	0	0	0	0	0	0	0	0	0	0	0	0
0	0	0	0	0	0	0	0	0	0	0	0	0	0

0	0	0	0	0	0	0	0	0	0	0	0	0	0
0	0	0	0	0	0	0	0	0	0	0	0	0	0
0	0	0	0	0	0	0	0	0	0	0	0	0	0
0	0	0	0	0	0	0	0	0	0	0	0	0	0
0	0	0	0	0	0	0	0	0	0	0	0	0	0
0													
OTU8904	0	0	0	0	0	0	0	0	1	0	0	0	0
0	0	0	0	0	0	0	0	0	0	0	0	0	0
0	0	0	0	0	0	0	0	0	0	0	0	0	0
0	0	0	1	0	0	0	2	0	0	0	0	0	1
0	0	0	0	0	0	0	0	0	0	0	0	0	0
0	0	0	0	0	0	0	0	0	0	0	0	0	0
0	0	0	0	0	0	0	0	0	0	0	1	0	0
0	0	0	0	0	0	1	0	0	2	0	0	0	0
0													
OTU12469	0	0	0	0	0	0	0	0	1	0	0	0	0
0	0	0	0	0	0	0	0	0	0	0	0	0	0
1	0	0	0	0	0	0	0	0	0	0	0	0	0
0	0	0	0	0	0	0	0	0	0	0	0	0	0
0	0	0	0	0	0	0	0	0	0	0	0	0	0
0	0	0	0	0	0	0	0	0	0	0	0	0	0
0	0	0	0	0	0	0	0	0	0	0	0	0	0
0	0	0	0	0	0	0	0	0	0	0	0	0	0
0													
OTU14485	0	0	0	0	0	0	0	0	1	0	0	0	0
0	0	0	0	0	0	0	0	0	0	0	0	0	0
0	0	0	0	0	0	0	0	0	0	0	0	0	0
0	0	0	0	0	0	0	0	0	0	0	0	0	0
0	0	0	0	0	0	0	0	0	0	0	0	0	0
0	0	0	0	0	0	0	0	0	0	0	0	0	0
0	0	0	0	0	0	0	0	0	0	0	0	0	0
0	0	0	0	0	0	0	0	0	0	0	0	0	0
0													

OTU14351	0	0	0	0	0	0	0	0	1	0	0	0	0
0	0	0	2	0	0	0	0	2	0	0	0	0	0
1	0	0	0	2	0	0	1	0	0	0	0	0	0
0	0	0	0	0	0	0	0	0	0	0	0	0	0
0	0	3	0	0	0	0	0	0	0	0	0	0	0
0	0	1	0	0	0	0	0	0	0	0	0	0	0
0	0	0	0	0	0	0	0	0	0	1	0	0	0
0	0	0	0	0	0	0	0	0	0	1	0	3	0
0													
OTU1475	0	0	0	0	0	0	0	0	1	0	0	0	0
0	0	0	0	0	0	0	0	0	0	0	3	0	0
0	0	0	0	0	0	0	0	0	0	1	0	0	0
1	0	0	0	0	0	0	0	0	0	0	0	0	0
0	0	0	0	0	0	0	0	0	0	0	0	0	0
0	0	0	0	0	0	0	0	0	0	0	0	0	0
0	0	0	0	0	0	0	0	0	0	0	0	0	0
0	8	0	0	1	3	0	0	0	0	0	0	0	0
0													
OTU14484	0	0	0	0	0	0	0	0	1	0	0	0	0
0	0	0	0	0	0	0	0	0	0	0	0	0	0
0	0	0	0	0	0	0	0	0	0	0	0	0	0
0	0	0	0	0	0	0	0	0	0	0	0	0	0
0	0	0	0	0	0	0	0	0	0	0	0	0	0
0	0	0	0	0	0	0	0	0	0	0	0	0	0
0	0	0	0	0	0	0	0	0	0	0	0	0	0
0	0	0	0	0	0	0	0	0	0	0	0	0	0
0													
OTU486	0	0	0	0	0	0	0	0	3	0	0	0	0
0	0	0	0	0	0	0	0	0	0	0	0	0	0
0	0	0	0	0	0	0	0	0	0	0	0	0	0
0	0	0	0	0	0	0	0	0	0	0	0	0	0
0	0	0	0	0	0	0	0	0	0	0	0	0	0
0	7	0	0	0	0	0	0	0	0	0	0	0	0

0	0	0	0	0	0	0	0	0	0	0	15	0	0
0	4	1	0	0	1	0	5	1	9	0	0	0	5
7													
OTU14483	0	0	0	0	0	0	0	0	1	0	0	0	0
0	0	0	0	0	0	0	0	0	0	0	0	0	0
0	0	0	0	0	0	0	0	0	0	0	0	0	0
0	0	0	0	0	0	0	0	0	0	0	0	0	0
0	0	0	0	0	0	0	0	0	0	0	0	0	0
0	0	0	0	0	0	0	0	0	0	0	0	0	0
0	0	0	0	0	0	0	0	0	0	0	0	0	0
0	0	0	0	0	0	0	0	0	0	0	0	0	0
0													
OTU7666	0	0	0	0	0	0	0	0	1	0	0	0	0
0	1	0	0	0	0	0	0	0	0	0	0	0	0
0	0	0	0	0	0	0	0	0	0	0	0	0	0
0	0	0	0	0	0	0	0	0	0	0	0	0	0
0	0	0	0	0	0	0	0	0	0	0	0	0	0
0	0	0	0	0	0	0	1	0	0	0	0	0	0
0	0	0	0	0	0	0	0	0	0	0	0	0	0
0	0	0	0	0	0	0	0	0	0	0	0	1	0
0													
OTU8659	0	0	0	0	0	0	0	0	1	2	0	0	0
0	1	0	0	1	0	0	0	0	0	0	0	0	0
0	0	1	0	1	0	0	0	1	0	1	0	0	1
0	0	0	0	0	0	0	0	0	1	0	0	0	0
0	0	0	0	0	0	0	0	0	0	0	0	0	0
0	0	0	0	0	0	0	0	0	0	0	0	0	0
1	0	0	0	0	0	0	0	0	1	0	1	0	0
0	0	0	0	1	0	0	0	0	0	0	0	0	0
0													
OTU1151	0	0	0	0	0	0	0	0	1	1	31	2	47
0	0	0	0	0	8	0	0	0	0	0	0	0	0
0	0	0	0	0	0	0	0	0	0	0	0	0	0

0	0	1	0	30	2	2	0	0	0	0	0	1	0
0	0	0	0	0	0	0	3	0	0	0	0	0	0
0	0	3	0	0	1	0	0	0	0	0	0	0	0
0	0	0	0	0	0	1	0	0	4	0	0	0	5
0	0	0	0	1	0	0	0	0	0	1	0	0	0
1													
OTU4443	0	0	0	0	0	0	0	0	1	0	0	0	3
1	0	0	0	0	1	0	0	0	0	0	0	0	0
0	0	0	0	1	0	0	0	0	0	0	0	0	0
0	0	0	0	0	0	0	0	0	0	0	0	0	0
0	0	0	0	0	0	1	0	0	0	0	2	0	2
1	0	0	1	0	1	0	0	0	0	0	0	0	0
0	0	0	0	0	0	0	0	1	0	1	0	0	1
0	0	0	0	0	0	0	0	0	0	1	0	2	0
0													
OTU364	0	0	0	0	0	0	0	0	1	0	0	0	0
0	0	0	0	0	0	0	0	0	0	1	0	0	0
2	0	0	0	0	0	0	0	0	0	0	0	0	0
0	0	0	0	0	0	0	1	0	0	0	0	0	0
0	0	0	0	0	0	0	0	0	0	0	0	0	0
0	6	0	0	0	0	0	0	0	0	1	0	0	0
0	0	0	1	0	0	0	0	0	0	0	0	2	0
0	1	33	7	27	12	0	0	59	0	0	1	0	6
14													
OTU12466	0	0	0	0	0	0	0	0	1	0	0	0	0
0	0	0	0	0	0	0	0	0	0	0	0	0	0
0	0	0	0	0	0	0	0	0	0	0	0	0	0
0	0	0	0	0	0	0	0	0	0	0	0	0	0
0	0	0	0	0	0	0	0	0	0	0	0	0	0
0	0	0	0	0	0	0	0	0	0	0	0	0	0
0	0	0	0	0	0	0	0	0	0	0	0	0	0
0	0	0	0	0	0	0	0	0	0	0	0	0	0
0													

OTU12467	0	0	0	0	0	0	0	0	1	0	0	0	0
0	0	0	0	0	0	0	0	0	0	0	0	0	0
0	0	0	0	0	0	0	0	0	0	0	0	0	0
0	0	0	0	0	0	0	0	0	0	0	0	0	0
0	0	0	0	0	0	0	0	0	0	0	0	0	0
0	0	0	0	0	0	0	0	0	0	0	0	0	0
0	0	0	0	0	0	0	0	0	0	0	0	0	0
0	0	0	0	0	0	0	0	0	0	0	0	0	0
0													
OTU7654	0	0	0	0	0	0	0	0	1	0	0	0	0
0	0	0	0	0	0	0	0	0	0	0	0	0	0
0	0	0	0	0	0	0	0	0	0	0	0	0	0
0	0	0	0	0	0	0	0	0	0	0	0	0	0
0	0	0	0	0	0	0	0	0	0	0	0	0	0
0	0	0	0	0	0	0	0	0	0	0	0	0	0
0	0	0	0	0	0	0	0	0	0	0	0	0	0
0	0	0	0	0	0	0	0	0	0	0	0	0	0
0													
OTU11491	0	0	0	0	0	0	0	0	2	0	0	1	0
0	0	0	0	0	0	0	0	0	0	4	0	0	0
0	0	1	0	0	0	0	0	0	0	0	0	0	0
0	0	0	0	0	0	0	0	0	0	0	0	0	0
0	1	0	0	0	0	0	0	0	0	0	0	0	0
0	0	1	0	1	0	0	0	0	0	0	0	0	0
0	0	0	0	0	0	0	0	0	0	0	1	0	0
0	4	1	1	0	0	0	2	0	0	0	0	0	0
0													
OTU11861	0	0	0	0	0	0	0	0	1	0	0	0	0
0	0	0	1	0	0	0	0	0	0	0	0	0	0
0	1	1	0	0	0	0	0	0	0	0	0	0	0
0	0	0	0	0	0	0	0	0	0	0	0	1	0
0	0	0	0	0	0	0	0	0	0	0	0	0	1
0	0	1	0	0	0	0	0	0	0	0	0	0	0

0	0	0	0	0	0	0	0	0	0	0	0	0	0
0	0	0	0	0	0	0	0	0	0	0	0	0	0
0													
OTU3687	0	0	0	0	0	0	0	0	1	0	7	0	25
5	0	0	0	0	5	0	3	0	248	0	10	0	0
0	0	30	1	0	4	0	1	0	0	0	2	0	1
0	1	18	12	0	0	575	0	0	0	0	0	0	0
0	0	0	0	0	0	0	0	0	0	0	0	0	0
0	0	0	0	1	5	28	0	0	0	78	1	6	0
16	0	53	0	12	0	3	0	47	0	5	0	0	0
2	0	0	0	0	0	0	0	0	0	0	0	0	0
0													
OTU15795	0	0	0	0	0	0	0	0	1	0	0	0	0
0	0	0	0	0	0	0	0	0	0	0	0	0	0
0	0	0	0	0	0	0	0	0	0	0	0	0	0
0	0	0	0	0	0	0	0	0	0	0	0	0	0
0	0	0	0	0	0	0	0	0	0	0	0	0	0
0	0	0	0	0	0	0	0	0	0	0	0	0	0
0	0	0	0	0	0	0	0	0	0	0	0	0	0
0	0	0	0	0	0	0	0	0	0	0	0	0	0
0													
OTU2179	0	0	0	0	0	0	0	0	1	0	0	0	0
0	0	0	0	0	0	0	0	0	0	0	0	0	0
0	0	0	0	0	0	0	0	0	1	0	0	0	0
0	0	0	0	0	0	0	0	0	0	0	0	0	0
0	0	0	0	0	0	0	0	0	0	0	0	0	0
0	0	0	0	0	0	0	0	0	0	0	0	0	0
0	0	0	0	0	0	0	0	0	0	0	0	1	0
0	0	0	0	0	0	1	0	0	0	0	0	0	1
0													
OTU14478	0	0	0	0	0	0	0	0	1	0	0	0	0
0	0	0	0	0	0	0	0	0	0	0	0	0	0
0	0	0	0	0	0	0	0	0	0	0	0	0	0

0	0	0	0	0	0	0	0	0	0	0	0	0	0
0	0	0	0	0	0	0	0	0	0	0	0	0	0
0	0	0	0	0	0	0	0	0	0	0	0	0	0
0	0	0	0	0	0	0	0	0	0	0	0	0	0
0	0	0	0	0	0	0	0	0	0	0	0	0	0
0													
OTU12468	0	0	0	0	0	0	0	0	1	0	0	0	0
0	0	0	0	0	0	0	0	0	0	0	0	0	0
0	0	0	0	0	0	0	0	0	0	0	0	0	0
0	0	0	0	0	0	0	0	0	0	0	0	0	0
0	0	0	0	0	0	0	0	0	0	0	0	0	0
0	0	0	0	0	0	0	0	0	0	0	0	0	0
0	0	0	0	0	0	0	0	0	0	0	0	0	0
0	0	0	0	0	0	0	0	0	0	0	0	0	0
0													
OTU4107	0	0	0	0	0	0	0	0	1	0	0	0	0
0	0	0	0	0	0	0	0	0	0	0	0	0	0
0	0	0	0	0	0	0	0	0	0	0	0	0	0
0	0	0	0	0	1	0	0	0	0	0	0	0	0
0	0	0	0	0	0	0	0	0	0	0	0	2	0
0	0	0	0	0	0	0	0	0	0	1	0	0	0
0	0	0	0	0	0	0	0	0	0	0	0	0	1
0	1	1	1	2	1	1	3	1	2	0	0	0	0
1													
OTU14480	0	0	0	0	0	0	0	0	1	0	0	0	0
0	0	0	0	0	0	0	0	0	0	0	0	0	0
0	0	0	0	0	0	0	0	0	0	0	0	0	0
0	0	0	0	0	0	0	0	0	0	0	0	0	0
0	0	0	0	0	0	0	0	0	0	0	0	0	0
0	0	0	0	0	0	0	0	0	0	0	0	0	0
0	0	0	0	0	0	0	0	0	0	0	0	0	0
0	0	0	0	0	0	0	0	0	0	0	0	0	0
0													

OTU14481 0 0 0 0 0 0 0 0 1 0 0 0 0
0 0 0 0 0 0 0 0 0 0 0 0 0 0
0 0 0 0 0 0 0 0 0 0 0 0 0 0
0 0 0 0 0 0 0 0 0 0 0 0 0 0
0 0 0 0 0 0 0 0 0 0 0 0 0 0
0 0 0 0 0 0 0 0 0 0 0 0 0 0
0 0 0 0 0 0 0 0 0 0 0 0 0 0
0 0 0 0 0 0 0 0 0 0 0 0 0 0
0
OTU14482 0 0 0 0 0 0 0 0 1 0 0 0 0
0 0 0 0 0 0 0 0 0 0 0 0 0 0
0 0 0 0 0 0 0 0 0 0 0 0 0 0
0 0 0 0 0 0 0 0 0 0 0 0 0 0
0 0 0 0 0 0 0 0 0 0 0 0 0 0
0 0 0 0 0 0 0 0 0 0 0 0 0 0
0 0 0 0 0 0 0 0 0 0 0 0 0 0
0 0 0 0 0 0 0 0 0 0 0 0 0 0
0
OTU9001 0 0 0 0 0 0 0 0 1 0 0 0 0
0 0 0 0 0 0 0 0 0 0 0 0 0 0
0 0 0 0 0 0 0 0 0 0 0 0 0 0
1 0 0 0 0 0 0 0 0 0 0 0 0 0
0 0 0 0 0 0 0 0 0 0 0 0 0 0
0 0 0 0 0 0 0 0 0 0 0 0 0 0
0 0 0 0 0 0 0 0 0 0 0 0 0 0
0 0 0 0 0 0 0 0 0 0 0 0 0 0
0
OTU12412 0 0 0 0 0 0 0 0 1 0 0 0 0
0 0 0 0 0 0 0 0 0 0 0 0 0 0
0 0 0 0 0 0 0 0 0 0 0 0 0 0
0 0 0 0 0 0 0 0 0 0 0 0 0 0
0 0 0 0 0 0 0 0 0 0 0 0 0 0
0 0 0 0 0 0 0 0 0 0 0 0 0 0

0	0	0	0	0	0	0	0	0	0	0	0	0	0
0	0	0	0	0	0	0	0	0	0	0	0	0	0
0													
OTU7650	0	0	0	0	0	0	0	0	1	0	0	0	0
0	0	0	0	0	0	0	0	0	0	0	0	0	0
0	0	0	0	0	0	0	0	0	0	0	0	0	0
0	0	0	0	0	0	0	0	0	0	0	0	0	0
0	0	0	0	0	0	0	0	0	0	0	0	0	0
0	0	0	0	0	0	0	0	0	0	0	0	0	0
0	0	0	0	0	0	0	0	0	0	0	0	0	0
0	0	0	0	0	0	0	0	0	0	0	0	0	0
0													
OTU15799	0	0	0	0	0	0	0	0	1	0	0	0	0
0	0	0	0	0	0	0	0	0	0	0	0	0	0
0	0	0	0	0	0	0	0	0	0	0	0	0	0
0	0	0	0	0	0	0	0	0	0	0	0	0	0
0	0	0	0	0	0	0	0	0	0	0	0	0	0
0	0	0	0	0	0	0	0	0	0	0	0	0	0
0	0	0	0	0	0	0	0	0	0	0	0	0	0
0	0	0	0	0	0	0	0	0	0	0	0	0	0
0													
OTU14479	0	0	0	0	0	0	0	0	1	0	0	0	0
0	0	0	0	0	0	0	0	0	0	0	0	0	0
0	0	0	0	0	0	0	0	0	0	0	0	0	0
0	0	0	0	0	0	0	0	0	0	0	0	0	0
0	0	0	0	0	0	0	0	0	0	0	0	0	0
0	0	0	0	0	0	0	0	0	0	0	0	0	0
0	0	0	0	0	0	0	0	0	0	0	0	0	0
0	0	0	0	0	0	0	0	0	0	0	0	0	0
0													
OTU12410	0	0	0	0	0	0	0	0	1	0	0	0	0
0	0	0	0	0	0	0	0	0	0	0	0	0	0
0	0	0	0	0	0	0	0	0	0	0	0	0	0

0	0	0	0	0	0	0	0	0	0	0	0	0	0
0	0	0	0	0	0	0	0	0	0	0	0	0	0
0	0	0	0	0	0	0	0	0	0	0	0	0	0
0	0	0	0	0	0	0	0	0	0	0	0	0	0
0	0	0	0	0	0	0	0	0	0	0	0	0	0
0													
OTU15798	0	0	0	0	0	0	0	0	1	0	0	0	0
0	0	0	0	0	0	0	0	0	0	0	0	0	0
0	0	0	0	0	0	0	0	0	0	0	0	0	0
0	0	0	0	0	0	0	0	0	0	0	0	0	0
0	0	0	0	0	0	0	0	0	0	0	0	0	0
0	0	0	0	0	0	0	0	0	0	0	0	0	0
0	0	0	0	0	0	0	0	0	0	0	0	0	0
0	0	0	0	0	0	0	0	0	0	0	0	0	0
0													
OTU14488	0	0	0	0	0	0	0	0	1	0	0	0	0
0	0	0	0	0	0	0	0	0	0	0	0	0	0
0	0	0	0	0	0	0	0	0	0	0	0	0	0
0	0	0	0	0	0	0	0	0	0	0	0	0	0
0	0	0	0	0	0	0	0	0	0	0	0	0	0
0	0	0	0	0	0	0	0	0	0	0	0	0	0
0	0	0	0	0	0	0	0	0	0	0	0	0	0
0	0	0	0	0	0	0	0	0	0	0	0	0	0
0													
OTU2276	0	0	0	0	0	0	0	0	2	0	0	0	0
0	0	0	0	0	0	1	0	1	0	1	0	0	0
0	0	0	0	0	1	0	1	0	0	0	0	0	0
0	0	1	2	0	0	0	0	0	0	0	0	0	0
0	0	0	0	0	0	0	0	0	0	0	0	2	0
0	0	0	0	0	4	0	0	2	0	0	0	2	0
0	0	0	0	0	0	0	0	0	0	0	0	0	0
1	0	0	0	1	0	0	0	0	1	0	0	1	1
0													

OTU15796 0 0 0 0 0 0 0 0 1 0 0 0 0
0 0 0 0 0 0 0 0 0 0 0 0 0 0
0 0 0 0 0 0 0 0 0 0 0 0 0 0
0 0 0 0 0 0 0 0 0 0 0 0 0 0
0 0 0 0 0 0 0 0 0 0 0 0 0 0
0 0 0 0 0 0 0 0 0 0 0 0 0 0
0 0 0 0 0 0 0 0 0 0 0 0 0 0
0 0 0 0 0 0 0 0 0 0 0 0 0 0
0
OTU15797 0 0 0 0 0 0 0 0 1 0 0 0 0
0 0 0 0 0 0 0 0 0 0 0 0 0 0
0 0 0 0 0 0 0 0 0 0 0 0 0 0
0 0 0 0 0 0 0 0 0 0 0 0 0 0
0 0 0 0 0 0 0 0 0 0 0 0 0 0
0 0 0 0 0 0 0 0 0 0 0 0 0 0
0 0 0 0 0 0 0 0 0 0 0 0 0 0
0 0 0 0 0 0 0 0 0 0 0 0 0 0
0
OTU6781 0 0 0 0 0 0 0 0 1 0 0 0 0
0 0 0 0 0 0 0 0 0 0 0 0 0 0
0 0 0 0 0 0 0 0 0 0 0 0 0 0
0 0 0 0 0 0 0 0 0 0 0 0 0 0
0 0 0 0 0 0 0 0 0 0 0 0 0 0
0 0 0 0 0 0 0 0 0 0 0 0 0 0
0 0 0 1 0 0 0 0 0 0 0 0 0 0
0 0 0 0 0 0 0 0 0 0 0 0 0 0
0
OTU12411 0 0 0 0 0 0 0 0 1 0 0 0 0
0 0 0 0 0 0 0 0 0 0 0 0 0 0
0 0 0 0 0 0 0 0 0 0 0 0 0 0
0 0 0 0 0 0 0 0 0 0 0 0 0 0
0 0 0 0 0 0 0 0 0 0 0 0 0 0
0 0 0 0 0 0 0 0 0 0 0 0 0 0

0	0	0	0	0	0	0	0	0	0	0	0	0	0
0	0	0	0	0	0	0	0	0	0	0	0	0	0
0													
OTU15058	0	0	0	0	0	0	0	0	1	0	0	0	0
0	0	0	0	0	0	0	0	0	0	0	0	0	0
0	0	0	0	0	0	0	0	0	0	0	0	0	0
1	0	0	0	0	0	0	0	0	0	0	0	0	0
0	0	0	0	0	0	0	0	0	0	0	0	0	0
0	0	0	0	0	0	0	0	0	0	0	0	0	0
0	0	0	0	0	0	0	0	0	0	0	0	0	0
0	0	0	0	1	2	0	0	0	0	0	0	0	0
0													
OTU7652	0	0	0	0	0	0	0	0	1	0	1	0	0
0	0	0	0	0	0	0	0	0	0	0	0	0	0
0	0	0	0	0	0	0	0	0	0	0	0	0	0
0	0	0	0	0	0	0	0	0	0	0	0	0	0
0	0	0	0	0	0	0	0	0	0	0	0	0	0
0	0	0	0	0	0	0	0	0	0	0	0	0	0
0	0	0	0	0	0	0	0	0	0	0	0	0	0
0	0	0	0	0	0	0	0	0	0	0	0	0	0
0													
OTU15047	0	0	0	0	0	0	0	0	2	0	0	1	0
0	0	1	0	0	0	0	0	0	0	0	1	7	0
0	7	0	0	0	0	0	0	0	3	0	0	0	0
0	0	0	0	0	0	0	0	9	0	0	3	0	0
0	0	0	3	0	2	0	6	2	0	0	0	0	0
0	0	0	0	0	0	0	0	0	0	0	3	0	2
0	0	0	0	0	0	1	0	0	0	0	0	1	0
0	1	4	0	1	3	1	0	0	0	1	0	0	0
0													
OTU7651	0	0	0	0	0	0	0	0	1	0	0	0	0
0	0	0	0	0	0	0	0	0	0	0	0	0	0
0	0	0	0	0	0	0	0	0	0	0	0	0	0

0	0	0	0	0	0	0	0	0	0	0	0	0	0
0	0	0	0	0	0	0	0	0	0	0	0	0	0
0	0	0	0	0	0	0	0	0	0	0	0	0	0
0	0	0	0	0	0	0	0	0	0	0	0	0	0
0	0	0	0	0	0	0	0	0	0	0	0	0	0
0													
OTU12550	0	0	0	0	0	0	0	0	1	0	0	0	0
0	1	0	0	0	0	0	0	0	0	0	0	0	0
0	0	0	0	0	0	0	0	0	0	0	0	0	0
0	0	0	0	0	0	0	0	1	0	0	0	0	0
0	0	0	0	0	0	0	0	0	0	0	0	0	0
0	0	0	0	0	0	0	0	0	0	0	0	0	0
0	0	0	0	0	0	0	0	0	0	0	0	0	0
1	0	0	0	0	0	0	0	0	0	0	0	0	0
0													
OTU15786	0	0	0	0	0	0	0	0	1	0	0	0	0
0	0	0	0	0	0	0	0	0	0	0	0	0	0
0	0	0	0	0	0	0	0	0	0	0	0	0	0
0	0	0	0	0	0	0	0	0	0	0	0	0	0
0	0	0	0	0	0	0	0	0	0	0	0	0	0
0	0	0	0	0	0	0	0	0	0	0	0	0	0
0	0	0	0	0	0	0	1	0	0	0	0	0	0
0	0	0	0	0	0	0	0	0	0	0	0	0	0
0													
OTU7658	0	0	0	0	0	0	0	0	1	0	0	0	0
0	0	0	0	0	0	0	0	0	0	0	0	0	0
0	0	0	0	0	0	0	0	0	0	0	0	0	0
0	0	0	0	0	0	0	0	0	0	0	0	0	0
0	0	0	0	0	0	0	0	0	0	0	0	0	0
0	0	0	0	0	0	0	0	0	0	0	0	0	0
0	0	0	0	0	0	0	0	0	0	0	0	0	0
0	0	0	0	0	0	0	0	0	0	0	0	0	0
0													

OTU12407	0	0	0	0	0	0	0	0	1	0	0	0	0
0	0	0	0	0	0	0	0	0	0	0	0	0	0
0	0	0	0	0	0	0	0	0	0	0	0	0	0
0	0	0	0	0	0	0	0	0	0	0	0	0	0
0	0	0	0	0	0	0	0	0	0	0	0	0	0
0	0	0	0	0	0	0	0	0	0	0	0	0	0
0	0	0	0	0	0	0	0	0	0	0	0	0	0
0	0	0	0	0	0	0	0	0	0	0	0	0	0
0													
OTU2122	0	0	0	0	0	0	0	0	1	0	0	0	0
0	0	0	0	0	0	0	0	0	0	0	0	0	0
0	0	0	0	0	0	0	0	0	0	0	0	0	0
0	0	1	0	0	0	0	0	1	1	0	0	0	0
1	0	0	0	4	0	2	1	1	1	0	0	0	0
1	0	0	0	0	0	0	0	0	0	0	1	0	1
0	1	0	0	0	0	0	0	0	0	0	0	0	0
0	0	0	0	0	0	0	0	1	0	0	0	0	0
0													
OTU9579	0	0	0	0	0	0	0	0	1	0	0	0	0
0	0	0	0	0	0	0	0	0	0	0	0	0	2
0	0	0	0	0	0	0	1	0	0	1	0	0	0
0	0	0	0	0	0	0	0	0	0	0	0	0	1
0	0	0	0	0	0	0	0	0	0	0	0	0	0
0	0	0	0	0	0	0	0	0	0	0	0	2	0
0	0	0	0	0	0	0	0	0	0	1	0	0	0
0	0	0	0	0	0	0	0	0	0	0	1	0	0
0													
OTU10730	0	0	0	0	0	0	0	0	1	0	0	0	0
0	0	0	0	0	0	0	0	0	0	0	0	0	0
0	0	0	0	0	0	0	0	0	0	0	0	0	0
0	0	0	0	0	0	0	0	0	0	0	0	0	0
0	0	0	0	0	0	0	0	0	0	0	0	0	0
0	0	0	0	0	0	0	0	0	0	0	0	0	0

0	0	0	0	0	0	0	0	0	0	0	0	0	0
0	0	0	0	0	0	0	0	1	0	0	0	0	0
0													
OTU7657	0	0	0	0	0	0	0	0	1	0	0	0	0
0	0	0	0	0	0	0	0	0	0	0	0	0	0
0	0	0	0	0	0	0	0	0	0	0	0	0	0
0	0	0	0	0	0	0	0	0	0	0	0	0	0
0	0	0	0	0	0	0	0	0	0	0	0	0	0
0	0	0	0	0	0	0	0	0	0	0	0	0	0
0	0	0	0	0	0	0	0	0	0	0	0	0	0
0	0	0	0	0	0	0	0	0	0	0	0	0	0
0													
OTU14486	0	0	0	0	0	0	0	0	1	0	0	0	0
0	0	0	0	0	0	0	0	0	0	0	0	0	0
0	0	0	0	0	0	0	0	0	0	0	0	0	0
0	0	0	0	0	0	0	0	0	0	0	0	0	0
0	0	0	0	0	0	0	0	0	0	0	0	0	0
0	0	0	0	0	0	0	0	0	0	0	0	0	0
0	0	0	0	0	0	0	0	0	0	0	0	0	0
0	0	0	0	0	0	0	0	0	0	0	0	0	0
0													
OTU7656	0	0	0	0	0	0	0	0	1	0	0	0	0
0	0	0	0	0	0	0	0	0	0	0	0	0	0
0	0	0	0	0	0	0	0	0	0	0	0	0	0
0	0	0	0	0	0	0	0	0	0	0	0	0	0
0	0	0	0	0	0	0	0	0	0	0	0	0	0
0	0	0	0	0	0	0	0	0	0	0	0	0	1
0	0	0	0	0	0	0	0	0	0	0	0	0	0
0	0	0	0	0	0	0	0	0	0	0	0	0	0
0													
OTU9237	0	0	0	0	0	0	0	0	1	0	0	0	0
0	0	0	0	0	0	1	0	0	0	0	0	0	0
0	0	0	0	0	0	0	0	0	0	0	0	0	0

0	0	0	0	0	0	0	0	0	0	0	0	0	0
0	0	0	0	0	0	0	0	0	0	0	0	0	0
0	0	0	0	0	0	0	0	0	0	0	0	0	0
0	0	0	0	0	0	0	0	0	0	0	0	0	0
0	0	0	0	0	0	0	0	0	0	0	0	0	0
0													
OTU8935	0	0	0	0	0	0	0	0	1	0	0	0	0
0	0	0	0	0	0	0	0	0	0	0	0	23	0
3	0	0	0	0	0	0	0	0	0	0	0	4	0
0	0	0	0	0	0	0	0	0	0	0	0	0	0
0	0	0	0	0	0	0	0	0	0	0	0	0	0
0	0	0	0	0	0	0	0	0	0	0	0	0	0
0	2	0	0	0	0	0	0	0	0	0	0	0	0
0	0	0	0	0	0	0	0	0	0	0	0	0	0
0													
OTU12408	0	0	0	0	0	0	0	0	1	0	0	0	0
0	0	0	0	0	0	0	0	0	0	0	0	0	0
0	0	0	0	0	0	0	0	0	0	0	0	0	0
0	0	0	0	0	0	0	0	0	0	0	0	0	0
0	0	0	0	0	0	0	0	0	0	0	0	0	0
0	0	0	0	0	0	0	0	0	0	0	0	0	0
0	0	0	0	0	0	0	0	0	0	0	0	0	0
0	0	0	0	0	0	0	0	0	0	0	0	0	0
0													
OTU12409	0	0	0	0	0	0	0	0	1	0	0	0	0
0	0	0	0	0	0	0	0	0	0	0	0	0	0
0	0	0	0	0	0	0	0	0	0	0	0	0	0
0	0	0	0	0	0	0	0	0	0	0	0	0	0
0	0	0	0	0	0	0	0	0	0	0	0	0	0
0	0	0	0	0	0	0	0	0	0	0	0	0	0
0	0	0	0	0	0	0	0	0	0	0	0	0	0
0	0	0	0	0	0	0	0	0	0	0	0	0	0
0													

OTU12420 0 0 0 0 0 0 0 0 1 0 0 0 0
0 0 0 0 0 0 0 0 0 0 0 0 0 0
0 0 0 0 0 0 0 0 0 0 0 0 0 0
0 0 0 0 0 0 0 0 0 0 0 0 0 0
0 0 0 0 0 0 0 0 0 0 0 0 0 0
0 0 0 0 0 0 0 0 0 0 0 0 0 0
0 0 0 0 0 0 0 0 0 0 0 0 0 0
0 0 0 0 0 0 0 0 0 0 0 0 0 0
0
OTU14487 0 0 0 0 0 0 0 0 1 0 0 0 0
0 0 0 0 0 0 0 0 0 0 0 0 0 0
0 0 0 0 0 0 0 0 0 0 0 0 0 0
0 0 0 0 0 0 0 0 0 0 0 0 0 0
0 0 0 0 0 0 0 0 0 0 0 0 0 0
0 0 0 0 0 0 0 0 0 0 0 0 0 0
0 0 0 0 0 0 0 0 0 0 0 0 0 0
0 0 0 0 0 0 0 0 0 0 0 0 0 0
0
OTU12419 0 0 0 0 0 0 0 0 1 0 0 0 0
0 0 0 0 0 0 0 0 0 0 0 0 0 0
0 0 0 0 0 0 0 0 0 0 0 0 0 0
0 0 0 0 0 0 0 0 0 0 0 0 0 0
0 0 0 0 0 0 0 0 0 0 0 0 0 0
0 0 0 0 0 0 0 0 0 0 0 0 0 0
0 0 0 0 0 0 0 0 0 0 0 0 0 0
0 0 0 0 0 0 0 0 0 0 0 0 0 0
0
OTU15785 0 0 0 0 0 0 0 0 1 0 0 0 0
0 0 0 0 0 0 0 0 0 0 0 0 0 0
0 0 0 0 0 0 0 0 0 0 0 0 0 0
0 0 0 0 0 0 0 0 0 0 0 0 0 0
0 0 0 0 0 0 0 0 0 0 0 0 0 0
0 0 0 0 0 0 0 0 0 0 0 0 0 0

0	0	0	0	0	0	0	0	0	0	0	0	0	0
0	0	0	0	0	0	0	0	0	0	0	0	0	0
0													
OTU14510	0	0	0	0	0	0	0	0	1	0	0	0	0
0	0	0	0	0	0	0	0	0	0	0	0	0	0
0	0	0	0	0	0	0	0	0	0	0	0	0	0
0	0	0	0	0	0	0	0	0	0	0	0	0	0
0	0	0	0	0	0	0	0	0	0	0	0	0	0
0	0	0	0	0	0	0	0	0	0	0	0	0	0
0	0	0	0	0	0	0	0	0	0	0	0	0	0
0	0	0	0	0	0	0	0	0	0	0	0	0	0
0													
OTU15944	0	0	0	0	0	0	0	0	1	0	0	0	0
0	1	0	0	1	1	0	0	0	0	0	0	0	0
0	1	0	0	0	0	0	0	0	0	0	0	0	0
0	0	0	0	0	1	0	0	0	0	0	1	0	0
0	0	0	0	0	0	0	0	0	0	0	0	0	0
0	0	0	0	1	0	0	0	0	0	1	0	0	0
1	0	0	0	0	0	0	0	0	0	1	0	1	0
0	0	0	0	1	0	0	0	0	0	0	0	0	0
0													
OTU12418	0	0	0	0	0	0	0	0	1	0	0	0	0
0	0	0	0	0	0	0	0	0	0	0	0	0	0
0	0	0	0	0	0	0	0	0	0	0	0	0	0
0	0	0	0	0	0	0	0	0	0	0	0	0	0
0	0	0	0	0	0	0	0	0	0	0	0	0	0
0	0	0	0	0	0	0	0	0	0	0	0	0	0
0	0	0	0	0	0	0	0	0	0	0	0	0	0
0	0	0	0	0	0	0	0	0	0	0	0	0	0
0													
OTU731	0	0	0	0	0	0	0	0	1	1	0	1	0
0	0	0	0	0	0	0	0	0	0	0	0	1	0
0	0	0	0	0	0	0	0	0	0	5	0	1	0

0	0	0	0	0	0	0	2	0	0	0	0	0	0
0	0	0	0	1	0	0	0	0	1	0	0	0	0
0	88	0	0	0	0	0	0	0	0	2	0	0	0
0	0	0	1	0	0	0	2	0	0	0	0	2	0
0	1	58	22	6	18	26	10	23	18	0	0	0	10
35													
OTU15788	0	0	0	0	0	0	0	0	1	0	0	0	0
0	0	0	0	0	0	0	0	0	0	0	0	0	0
0	0	0	0	0	0	0	0	0	0	0	0	0	0
0	0	0	0	0	0	0	0	0	0	0	0	0	0
0	0	0	0	0	0	0	0	0	0	0	0	0	0
0	0	0	0	0	0	0	0	0	0	0	0	0	0
0	0	0	0	0	0	0	0	0	0	0	0	0	0
0	0	0	0	0	0	0	0	0	0	0	0	0	0
0													
OTU4595	0	0	0	0	0	0	0	0	1	0	0	0	0
0	2	0	2	4	0	0	0	2	0	2	0	0	0
0	0	0	0	2	1	0	0	0	0	0	1	0	0
0	0	1	0	0	0	1	0	0	0	0	1	0	3
0	0	1	0	0	0	1	0	0	2	0	0	0	0
1	0	1	1	0	0	1	0	0	0	0	0	0	0
0	0	1	0	0	0	0	0	0	0	0	0	0	0
0	0	0	0	0	0	0	1	0	0	1	0	0	0
0													
OTU2879	0	0	0	0	0	0	0	0	1	0	0	0	0
0	0	0	0	0	0	0	0	0	0	0	0	0	0
0	0	0	0	0	0	0	0	0	0	0	0	0	0
0	0	0	0	0	0	0	0	0	0	0	0	0	0
0	0	0	0	0	0	0	0	0	0	0	0	0	0
0	1	0	0	0	0	0	0	0	0	0	0	0	0
0	0	0	0	0	0	0	0	0	0	0	3	2	0
0	1	0	0	0	1	0	0	0	0	0	0	0	0
3													

OTU2635	0	0	0	0	0	0	0	0	1	0	0	0	0
0	0	0	0	0	0	0	0	0	0	0	0	0	0
0	0	0	0	0	0	0	0	0	0	0	0	0	0
0	0	0	0	0	1	0	0	0	0	0	1	0	0
0	0	0	0	0	0	0	0	0	0	0	0	2	0
0	0	0	0	0	0	0	0	0	0	0	0	0	0
0	0	0	1	0	0	0	0	0	0	0	0	0	0
0	0	1	0	0	0	0	0	0	0	0	0	0	0
0													
OTU15003	0	0	0	0	0	0	0	0	1	0	0	0	0
0	0	0	0	0	0	0	0	0	0	0	0	0	0
0	0	0	0	0	0	0	0	0	0	0	0	1	0
0	0	0	0	0	0	0	0	0	0	0	0	0	1
0	1	0	0	0	0	0	0	0	0	0	0	1	0
0	0	0	0	1	1	0	0	1	0	0	0	0	0
0	0	0	0	0	0	0	0	0	0	0	1	0	0
0	0	0	1	0	0	0	0	0	0	0	0	0	0
0													
OTU1288	0	0	0	0	0	0	0	0	1	0	0	0	0
0	0	0	0	0	0	0	0	0	0	0	0	0	0
0	0	0	0	0	0	0	0	0	0	0	0	0	0
0	0	0	0	0	0	0	0	0	0	0	0	0	0
0	0	0	0	0	0	0	0	0	0	0	0	0	0
0	0	0	0	1	0	0	0	0	1	0	0	0	0
0	0	0	1	0	0	0	0	0	0	0	0	0	0
0	0	0	0	0	0	0	0	0	0	0	0	0	0
0													
OTU11062	0	0	0	0	0	0	0	0	1	0	0	0	0
0	0	1	0	3	0	0	0	0	0	0	0	0	1
0	0	0	0	0	0	2	0	0	1	0	0	0	0
0	0	0	0	0	0	0	0	0	0	0	0	0	0
0	0	0	0	1	0	0	0	0	0	0	0	0	0
0	0	0	0	0	0	0	0	0	0	0	0	1	0

1	0	0	0	0	0	0	0	0	2	0	0	0	0
2	0	0	0	0	0	0	0	0	0	0	0	0	0
0													
OTU12413	0	0	0	0	0	0	0	0	1	0	0	0	0
0	0	0	0	0	0	0	0	0	0	0	0	0	0
0	0	0	0	0	0	0	0	0	0	0	0	0	0
0	0	0	0	0	0	0	0	0	0	0	0	0	0
0	0	0	0	0	0	0	0	0	0	0	0	0	0
0	0	0	0	0	0	0	0	0	0	0	0	0	0
0	0	0	0	0	0	0	0	0	0	0	0	0	0
0	0	0	0	0	0	0	0	0	0	0	0	0	0
0													
OTU15790	0	0	0	0	0	0	0	0	1	0	0	0	0
0	0	0	0	0	0	0	0	0	0	0	0	0	0
0	0	0	0	0	0	0	0	0	0	0	0	0	0
0	0	0	0	0	0	0	0	0	0	0	0	0	0
0	0	0	0	0	0	0	0	0	0	0	0	0	0
0	0	0	0	0	0	0	0	0	0	0	0	0	0
0	0	0	0	0	0	0	0	0	0	0	0	0	0
0	0	0	0	0	0	0	0	0	0	0	0	0	0
0													
OTU15789	0	0	0	0	0	0	0	0	1	0	0	0	0
0	0	0	0	0	0	0	0	0	0	0	0	0	0
0	0	0	0	0	0	0	0	0	0	0	0	0	0
0	0	0	0	0	0	0	0	0	0	0	0	0	0
0	0	0	0	0	0	0	0	0	0	0	0	0	0
0	0	0	0	0	0	0	0	0	0	0	0	0	0
0	0	0	0	0	0	0	0	0	0	0	0	0	0
0	0	0	0	0	0	0	0	0	0	0	0	0	0
0													
OTU14507	0	0	0	0	0	0	0	0	1	0	0	0	0
0	0	0	0	0	0	0	0	0	0	0	0	0	0
0	0	0	0	0	0	0	0	0	0	0	0	0	0

0 0 0 0 0 0 0 0 0 0 0 0 0 0
0 0 0 0 0 0 0 0 0 0 0 0 0 0
0 0 0 0 0 0 0 0 0 0 0 0 0 0
0 0 0 0 0 0 0 0 0 0 0 0 0 0
0 0 0 0 0 0 0 0 0 0 0 0 0 0
0
OTU14508 0 0 0 0 0 0 0 0 1 0 0 0 0
0 0 0 0 0 0 0 0 0 0 0 0 0 0
0 0 0 0 0 0 0 0 0 0 0 0 0 0
0 0 0 0 0 0 0 0 0 0 0 0 0 0
0 0 0 0 0 0 0 0 0 0 0 0 0 0
0 0 0 0 0 0 0 0 0 0 0 0 0 0
0 0 0 0 0 0 0 0 0 0 0 0 0 0
0 0 0 0 0 0 0 0 0 0 0 0 0 0
0
OTU778 0 0 0 0 0 0 0 0 1 0 0 0 0
0 0 0 0 0 0 0 0 0 0 0 0 0 0
0 0 0 0 0 0 0 0 0 0 0 0 0 0
0 0 0 0 0 0 0 0 0 0 0 0 0 0
0 4 0 0 0 0 0 0 0 0 0 0 0 0
0 1 0 0 0 0 0 0 0 0 0 0 0 2
0 0 0 0 0 0 0 0 0 0 0 1 3 0
0 6 1 3 2 0 2 4 1 11 0 0 0 0
3
OTU5140 0 0 0 0 0 0 0 0 1 0 0 0 0
0 0 0 0 0 0 0 0 0 0 0 0 0 0
0 0 0 0 0 0 0 0 0 0 0 0 1 0
0 0 1 0 0 0 0 0 0 0 0 0 0 0
0 0 0 0 0 0 0 0 0 0 0 0 0 0
0 0 0 0 0 0 0 0 0 0 0 0 0 0
0 0 0 0 0 0 0 0 0 0 0 0 0 0
0 0 0 0 0 0 0 0 0 0 0 0 0 0
0

OTU12415 0 0 0 0 0 0 0 0 1 0 0 0 0
0 0 0 0 0 0 0 0 0 0 0 0 0 0
0 0 0 0 0 0 0 0 0 0 0 0 0 0
0 0 0 0 0 0 0 0 0 0 0 0 0 0
0 0 0 0 0 0 0 0 0 0 0 0 0 0
0 0 0 0 0 0 0 0 0 0 0 0 0 0
0 0 0 0 0 0 0 0 0 0 0 0 0 0
0 0 0 0 0 0 0 0 0 0 0 0 0 0
0
OTU14509 0 0 0 0 0 0 0 0 1 0 0 0 0
0 0 0 0 0 0 0 0 0 0 0 0 0 0
0 0 0 0 0 0 0 0 0 0 0 0 0 0
0 0 0 0 0 0 0 0 0 0 0 0 0 0
0 0 0 0 0 0 0 0 0 0 0 0 0 0
0 0 0 0 0 0 0 0 0 0 0 0 0 0
0 0 0 0 0 0 0 0 0 0 0 0 0 0
0 0 0 0 0 0 0 0 0 0 0 0 0 0
0
OTU12417 0 0 0 0 0 0 0 0 0 1 0 0 0
0 0 0 0 0 0 0 0 0 0 0 0 0 0
0 0 0 0 0 0 0 0 0 0 0 0 0 0
0 0 0 0 0 0 0 0 0 0 0 0 0 0
0 0 0 0 0 0 0 0 0 0 0 0 0 0
0 0 0 0 0 0 0 0 0 0 0 0 0 0
0 0 0 0 0 0 0 0 0 0 0 0 0 0
0 0 0 0 0 0 0 0 0 0 0 0 0 0
0
OTU7684 0 0 0 0 0 0 0 0 0 1 0 0 0
0 0 0 0 0 0 0 0 0 0 0 0 0 0
0 0 0 0 0 0 0 0 0 0 0 0 0 0
0 0 0 0 0 0 0 0 0 0 0 0 0 0
0 0 0 0 0 0 0 0 0 0 0 0 0 0
0 0 0 0 0 0 0 0 0 0 0 0 0 0

0	0	0	0	0	0	0	0	0	0	0	0	0	0
0	0	0	0	0	0	0	0	0	0	0	0	0	0
0													
OTU15549	0	0	0	0	0	0	0	0	0	1	0	0	0
0	1	0	0	0	0	0	1	0	0	0	0	0	0
0	0	0	1	0	0	0	0	0	0	0	0	0	0
0	0	0	0	0	0	0	0	0	0	0	0	0	0
0	0	0	0	0	0	0	0	0	0	0	0	0	0
0	0	0	0	0	0	1	1	0	1	0	0	0	0
0	0	0	0	0	0	0	0	0	0	0	1	0	0
0	0	0	0	0	0	0	0	0	0	0	0	0	0
0													
OTU7685	0	0	0	0	0	0	0	0	0	1	0	0	0
0	0	0	0	0	0	0	0	0	0	0	0	4	0
0	0	0	0	0	0	1	0	0	0	0	0	0	0
0	0	0	0	0	0	0	0	0	0	0	0	0	0
0	0	0	0	0	0	0	0	0	0	0	0	0	0
0	0	0	0	0	0	0	0	0	0	0	0	0	0
0	0	0	1	0	0	0	0	0	0	0	0	0	0
0	0	0	1	0	0	0	0	0	0	0	0	0	0
0													
OTU12414	0	0	0	0	0	0	0	0	0	1	0	0	0
0	0	0	0	0	0	0	0	0	0	0	0	0	0
0	0	0	0	0	0	0	0	0	0	0	0	0	0
0	0	0	0	0	0	0	0	0	0	0	0	0	0
0	0	0	0	0	0	0	0	0	0	0	0	0	0
0	0	0	0	0	0	0	0	0	0	0	0	0	0
0	0	0	0	0	0	0	0	0	0	0	0	0	0
0	0	0	0	0	0	0	0	0	0	0	0	0	0
0													
OTU495	0	0	0	0	0	0	0	0	0	16	0	0	0
0	0	0	0	0	0	0	0	0	0	0	0	0	0
0	0	0	0	0	0	0	0	0	0	0	1	0	0

0	0	0	0	1	0	0	0	0	0	0	0	0	0
0	0	0	0	0	0	0	0	0	0	0	0	0	0
0	3	0	0	0	0	0	0	0	0	0	0	0	0
0	0	0	0	0	0	0	0	0	0	0	0	3	0
0	2	12	0	0	3	3	0	1	0	0	0	0	0
6													
OTU12396	0	0	0	0	0	0	0	0	0	1	0	0	0
0	0	0	0	0	0	0	0	0	0	0	0	0	0
0	0	0	0	0	0	0	0	0	0	0	0	0	0
0	0	0	0	0	0	0	0	0	0	0	0	0	0
0	0	0	0	0	0	0	0	0	0	0	0	0	0
0	0	0	0	0	0	0	0	0	0	0	0	0	0
0	0	0	0	0	0	0	0	0	0	0	0	0	0
0	0	0	0	0	0	0	0	0	0	0	0	0	0
0													
OTU823	0	0	0	0	0	0	0	0	0	2	1	0	1
0	0	0	0	0	0	0	0	0	0	0	0	0	0
0	0	0	0	0	0	0	0	0	0	2	0	4	0
2	0	0	0	0	0	0	1	0	0	0	0	0	1
0	0	0	1	0	0	0	0	1	0	0	0	1	0
0	18	0	0	0	0	0	0	0	0	0	1	0	0
0	0	0	1	0	0	0	0	0	0	0	32	19	0
0	1	39	50	23	89	47	10	20	30	0	0	0	45
79													
OTU14512	0	0	0	0	0	0	0	0	0	1	0	0	0
0	0	0	0	0	0	0	0	0	0	0	0	0	0
0	0	0	0	0	0	0	0	0	0	0	0	0	0
0	0	0	0	0	0	0	0	0	0	0	0	0	0
0	0	0	0	0	0	0	0	0	0	0	0	0	0
0	0	0	0	0	0	0	0	0	0	0	0	0	0
0	0	0	0	0	0	0	0	0	0	0	0	0	0
0	0	0	0	0	0	0	0	0	0	0	0	0	0
0													

OTU14514	0	0	0	0	0	0	0	0	0	1	0	0	0
0	0	0	0	0	0	0	0	0	0	0	0	0	0
0	0	0	0	0	0	0	0	0	0	0	0	0	0
0	0	0	0	0	0	0	0	0	0	0	0	0	0
0	0	0	0	0	0	0	0	0	0	0	0	0	0
0	0	0	0	0	0	0	0	0	0	0	0	0	0
0	0	0	0	0	0	0	0	0	0	0	0	0	0
0	0	0	0	0	0	0	0	0	0	0	0	0	0
0													
OTU14513	0	0	0	0	0	0	0	0	0	1	0	0	0
0	0	0	0	0	0	0	0	0	0	0	0	0	0
0	0	0	0	0	0	0	0	0	0	0	0	0	0
0	0	0	0	0	0	0	0	0	0	0	0	0	0
0	0	0	0	0	0	0	0	0	0	0	0	0	0
0	0	0	0	0	0	0	0	0	0	0	0	0	0
0	0	0	0	0	0	0	0	0	0	0	0	0	0
0	0	0	0	0	0	0	0	0	0	0	0	0	0
0													
OTU4249	0	0	0	0	0	0	0	0	0	8	0	0	0
0	0	0	0	0	0	0	0	0	0	0	0	0	0
0	0	0	0	0	0	0	0	0	0	0	0	0	0
0	0	0	0	0	0	0	0	0	0	0	0	0	0
0	0	0	0	0	0	0	0	0	0	0	0	0	0
0	0	0	0	0	0	0	0	0	0	0	0	0	0
0	0	0	0	0	0	0	0	0	0	0	0	0	0
0	0	0	0	0	0	0	0	0	0	0	0	0	0
0													
OTU1704	0	0	0	0	0	0	0	0	0	1	0	0	0
0	0	0	0	0	0	0	0	0	0	0	0	1	0
0	0	0	0	0	0	0	0	0	0	3	0	0	0
0	0	0	0	0	0	0	0	0	0	0	0	0	0
0	0	0	0	0	0	0	0	0	0	0	0	4	0
0	0	0	0	0	0	0	0	0	0	0	0	0	0

0	0	0	0	0	0	0	2	0	0	0	0	0	0
0	0	0	0	0	0	4	2	1	0	0	0	0	2
0													
OTU7682	0	0	0	0	0	0	0	0	0	1	0	0	0
0	0	0	0	0	0	0	0	0	0	0	0	0	0
0	0	0	0	0	0	0	0	0	0	0	0	0	0
0	0	0	0	0	0	0	0	0	0	0	0	0	0
0	0	0	0	0	0	0	0	0	0	0	0	0	0
0	0	0	0	0	0	0	0	0	0	0	0	0	0
0	0	0	0	0	0	0	0	0	0	0	0	0	0
0	0	0	0	0	0	0	0	0	0	0	0	0	0
0													
OTU12397	0	0	0	0	0	0	0	0	0	1	0	0	0
0	0	0	0	0	0	0	0	0	0	0	0	0	0
0	0	0	0	0	0	0	0	0	0	0	0	0	0
0	0	0	0	0	0	0	0	0	0	0	0	0	0
0	0	0	0	0	0	0	0	0	0	0	0	0	0
0	0	0	0	0	0	0	0	0	0	0	0	0	0
0	0	0	0	0	0	0	0	0	0	0	0	0	0
0	0	0	0	0	0	0	0	0	0	0	0	0	0
0													
OTU231	0	0	0	0	0	0	0	0	0	16	0	0	0
0	2	0	0	0	0	0	0	0	0	0	0	0	0
0	0	0	0	0	0	0	0	0	2	0	0	0	0
0	0	0	0	0	0	0	1	0	0	0	0	0	0
0	0	0	0	0	0	0	0	0	0	0	0	0	0
0	29	0	0	0	0	0	0	0	2	0	0	0	2
0	0	0	0	1	0	0	0	0	0	0	39	15	0
0	14	24	0	40	10	37	74	6	2	0	1	0	3
1													
OTU12394	0	0	0	0	0	0	0	0	0	2	0	0	0
0	0	0	0	0	0	0	0	0	0	0	0	0	0
0	0	0	0	0	0	0	0	0	0	0	0	0	0

0	0	0	0	0	0	0	0	0	0	0	0	0	0
0	0	0	0	0	0	0	0	0	0	0	0	0	0
0	0	0	0	0	0	0	0	0	0	0	0	0	0
0	0	0	0	0	0	0	0	0	0	0	0	0	0
0	0	0	0	0	0	0	0	0	0	0	0	0	0
0													
OTU11732	0	0	0	0	0	0	0	0	0	1	0	0	0
0	0	1	0	1	0	5	1	0	0	0	0	0	3
3	0	0	2	0	1	0	0	0	0	0	0	0	0
6	0	0	0	0	0	0	0	0	0	0	1	0	0
0	0	0	0	0	0	0	0	0	0	0	0	0	0
0	0	0	0	0	1	12	0	0	0	0	1	0	0
0	0	0	0	0	0	0	0	0	9	0	0	0	0
0	0	0	0	0	0	0	0	0	0	0	0	0	0
0													
OTU7124	0	0	0	0	0	0	0	0	0	1	0	0	0
0	0	0	0	0	0	0	0	0	0	0	0	0	0
0	0	0	0	0	0	0	0	0	0	0	0	0	0
0	0	0	0	0	0	0	0	0	0	0	0	0	0
0	0	0	0	0	0	0	0	0	0	0	0	0	0
0	0	0	0	0	0	0	0	0	0	0	0	0	0
0	0	0	0	0	0	0	0	0	0	0	0	0	0
0	0	1	0	0	0	0	0	0	0	0	0	0	0
0													
OTU7670	0	0	0	0	0	0	0	0	0	1	0	0	0
0	0	0	0	0	0	0	0	0	0	0	0	0	0
0	0	0	0	0	0	0	0	0	0	0	0	0	0
0	0	0	0	0	0	0	0	0	0	0	0	0	0
0	0	0	0	0	0	0	0	0	0	0	0	0	0
0	0	0	0	0	0	0	0	0	0	0	0	0	0
0	0	0	0	0	0	0	0	0	0	0	0	0	0
0	0	0	0	0	0	0	0	0	0	0	0	0	0
0													

OTU7217	0	0	0	0	0	0	0	0	0	1	0	0	0
0	0	0	0	0	0	1	0	0	0	0	0	0	0
0	0	0	0	0	0	0	0	0	0	0	0	0	0
0	0	0	0	0	0	0	0	0	0	0	0	0	0
0	0	0	0	0	0	0	0	0	0	0	0	0	0
0	0	0	0	1	0	0	0	0	2	0	0	0	0
0	0	0	0	0	0	0	0	0	0	0	0	0	0
0	0	0	0	0	0	0	0	0	1	0	0	0	0
0													
OTU2095	0	0	0	0	0	0	0	0	0	1	0	0	0
0	0	0	0	0	0	0	0	0	0	0	0	0	0
0	0	0	0	0	0	0	0	0	0	0	0	0	0
0	0	0	0	0	0	0	0	0	0	0	0	0	0
0	0	0	0	0	0	0	0	0	0	0	0	2	0
0	1	0	0	0	0	0	0	0	0	0	0	0	0
0	0	0	0	0	0	0	0	0	0	0	0	0	0
0	0	0	0	0	0	0	0	0	1	0	0	0	0
0													
OTU4467	0	0	0	0	0	0	0	0	0	5	0	1	0
0	0	0	0	0	0	7	0	0	0	0	0	0	0
0	0	0	0	0	0	0	0	0	0	0	0	0	0
4	0	0	0	0	0	0	0	0	0	0	0	0	0
0	0	0	0	0	0	0	0	0	0	0	0	0	0
0	0	0	0	0	0	0	0	0	0	0	0	0	0
0	0	0	0	0	0	0	0	0	0	0	0	0	0
0	0	2	4	2	0	0	0	0	0	0	0	0	0
0													
OTU12395	0	0	0	0	0	0	0	0	0	1	0	0	0
0	0	0	0	0	0	0	0	0	0	0	0	0	0
0	0	0	0	0	0	0	0	0	0	0	0	0	0
0	0	0	0	0	0	0	0	0	0	0	0	0	0
0	0	0	0	0	0	0	0	0	0	0	0	0	0
0	0	0	0	0	0	0	0	0	0	0	0	0	0

0	0	0	0	0	0	0	0	0	0	0	0	0	0
0	0	0	0	0	0	0	0	0	0	0	0	0	0
0													
OTU13212	0	0	0	0	0	0	0	0	0	5	0	0	0
0	0	0	0	0	0	0	0	0	0	0	0	0	0
0	0	0	0	0	0	0	0	0	0	0	0	0	0
0	0	0	2	0	0	0	0	0	0	0	0	0	0
0	0	0	0	0	0	0	0	0	0	0	2	0	0
0	0	0	0	0	0	0	0	0	0	0	0	0	0
0	0	0	0	0	0	0	0	0	0	0	0	0	0
0	0	0	0	0	0	0	0	0	0	0	0	0	0
0													
OTU12400	0	0	0	0	0	0	0	0	0	1	0	0	0
0	0	0	0	0	0	0	0	0	0	0	0	0	1
0	0	0	0	0	0	0	0	0	0	0	0	0	0
0	0	0	0	0	0	0	0	0	0	0	0	0	0
0	0	0	0	0	0	0	0	0	0	0	0	0	0
0	0	0	0	0	0	0	0	0	0	0	0	0	0
0	0	0	0	0	0	0	0	0	0	0	0	0	0
0	0	0	0	0	0	0	0	0	0	0	0	0	0
0													
OTU4807	0	0	0	0	0	0	0	0	0	1	0	0	0
0	0	0	0	0	0	0	0	0	0	0	0	0	0
0	0	0	0	0	0	0	0	0	0	0	0	0	0
0	0	0	0	0	0	0	1	0	0	0	0	0	0
0	0	0	0	0	0	0	0	0	0	0	0	0	0
0	0	0	0	0	0	0	0	0	0	0	0	0	0
0	0	0	0	0	0	0	0	0	0	0	0	0	0
0	0	0	0	0	0	0	0	0	0	0	0	0	0
0													
OTU12401	0	0	0	0	0	0	0	0	0	1	0	0	0
0	0	0	0	0	0	0	0	0	0	0	0	0	0
0	0	0	0	0	0	0	0	0	0	0	0	0	0

0 0 0 0 0 0 0 0 0 0 0 0 0 0
0 0 0 0 0 0 0 0 0 0 0 0 0 0
0 0 0 0 0 0 0 0 0 0 0 0 0 0
0 0 0 0 0 0 0 0 0 0 0 0 0 0
0 0 0 0 0 0 0 0 0 0 0 0 0 0
0
OTU7669 0 0 0 0 0 0 0 0 0 1 0 0 0
0 0 0 0 0 0 0 0 0 0 0 0 0 0
0 0 0 0 0 0 0 0 0 0 0 0 0 0
0 0 0 0 0 0 0 0 0 0 0 0 0 0
0 0 0 0 0 0 0 0 0 0 0 0 0 0
0 0 0 0 0 0 0 0 0 0 0 0 0 0
0 0 0 0 0 0 0 0 0 0 0 0 0 0
0 0 0 0 0 0 0 0 0 0 0 0 0 0
0
OTU12983 0 0 0 0 0 0 0 0 0 1 0 0 0
0 0 0 0 0 0 0 0 0 0 0 0 0 0
0 0 0 0 0 0 0 1 0 0 0 0 0 0
0 0 0 0 0 0 0 0 0 0 0 0 0 0
0 0 0 0 0 0 0 0 0 0 0 0 0 0
0 0 0 0 0 0 0 0 0 0 0 0 0 0
0 0 0 0 0 0 0 0 0 0 0 0 0 0
0 0 0 0 0 0 0 0 0 0 0 0 0 0
0
OTU7668 0 0 0 0 0 0 0 0 0 1 0 0 0
0 0 0 0 0 0 0 0 0 0 0 0 0 0
0 0 0 0 0 0 0 0 0 0 0 0 0 0
0 0 0 0 0 0 0 0 0 0 0 0 0 0
0 0 0 0 0 0 0 0 0 0 0 0 0 0
0 0 0 0 0 0 0 0 0 0 0 0 0 0
0 0 0 0 0 0 0 0 0 0 0 0 0 0
0 0 0 0 0 0 0 0 0 0 0 0 0 0
0

OTU1457 0 0 0 0 0 0 0 0 0 1 0 0 0

0 0 0 0 0 0 0 0 0 0 0 0 0 0

0 0 0 0 0 0 0 0 0 0 0 0 0 0

0 0 0 0 0 0 0 0 0 0 0 0 0 0

0 0 0 0 0 0 0 1 0 0 0 0 0 0

0 0 0 0 1 0 0 0 0 0 0 0 0 0

0 0 0 0 0 0 0 0 0 0 0 0 0 0

0 0 0 0 0 0 0 0 0 0 0 0 0 0

0

OTU14502 0 0 0 0 0 0 0 0 0 1 0 0 0

0 0 0 0 0 0 0 0 0 0 0 0 0 0

0 0 0 0 0 0 0 0 0 0 0 0 0 0

0 0 0 0 0 0 0 0 0 0 0 0 0 0

0 0 0 0 0 0 0 0 0 0 0 0 0 0

0 0 0 0 0 0 0 0 0 0 0 0 0 0

0 0 0 0 0 0 0 0 0 0 0 0 0 0

0 0 0 0 0 0 0 0 0 0 0 0 0 0

0

OTU14503 0 0 0 0 0 0 0 0 0 1 0 0 0

0 0 0 0 0 0 0 0 0 0 0 0 0 0

0 0 0 0 0 0 0 0 0 0 0 0 0 0

0 0 0 0 0 0 0 0 0 0 0 0 0 0

0 0 0 0 0 0 0 0 0 0 0 0 0 0

0 0 0 0 0 0 0 0 0 0 0 0 0 0

0 0 0 0 0 0 0 0 0 0 0 0 0 0

0 0 0 0 0 0 0 0 0 0 0 0 0 0

0

OTU15637 0 0 0 0 0 0 0 0 0 1 0 0 0

0 0 0 0 0 0 0 0 0 0 0 0 0 0

0 0 0 0 0 0 0 0 0 0 0 0 0 0

0 0 0 0 0 0 0 0 0 0 0 0 0 0

0 0 0 0 0 0 0 0 0 0 0 0 0 0

0 0 0 0 0 0 0 0 0 0 0 0 0 0

0 0 0 0 0 0 0 0 0 0 0 0 0 0

0 0 0 0 0 0 0 0 0 0 0 0 0 0

0

OTU12405 0 0 0 0 0 0 0 0 0 1 0 0 0

0 0 0 0 0 0 0 0 0 0 0 0 0 0

0 0 0 0 0 0 0 0 0 0 0 0 0 0

0 0 0 0 0 0 0 0 0 0 0 0 0 0

0 0 0 0 0 0 0 0 0 0 0 0 0 0

0 0 0 0 0 0 0 0 0 0 0 0 0 0

0 0 0 0 0 0 0 0 0 0 0 0 0 0

0 0 0 0 0 0 0 0 0 0 0 0 0 0

0

OTU435 0 0 0 0 0 0 0 0 0 1 0 0 0

0 0 0 0 0 0 0 0 0 0 0 0 0 0

0 0 0 0 0 0 0 0 0 0 0 0 0 0

0 0 0 0 0 0 0 1 0 0 0 0 0 0

0 0 0 0 0 0 0 0 0 0 0 0 0 0

0 0 0 0 0 0 0 0 0 0 0 0 0 0

0 0 0 0 0 0 0 0 0 0 0 0 0 0

0 0 0 1 0 0 0 0 1 67 0 0 0 0

26

OTU12403 0 0 0 0 0 0 0 0 0 1 0 0 0

0 0 0 0 0 0 0 0 0 0 0 0 0 0

0 0 0 0 0 0 0 0 0 0 0 0 0 0

0 0 0 0 0 0 0 0 0 0 0 0 0 0

0 0 0 0 0 0 0 0 0 0 0 0 0 0

0 0 0 0 0 0 0 0 0 0 0 0 0 0

0 0 0 0 0 0 0 0 0 0 0 0 0 0

0 0 0 0 0 0 0 0 0 0 0 0 0 0

0

OTU12406 0 0 0 0 0 0 0 0 0 1 0 0 0

0 0 0 0 0 0 0 0 0 0 0 0 0 0

0 0 0 0 0 0 0 0 0 0 0 0 0 0

0	0	0	0	0	0	0	0	0	0	0	0	0	0
0	0	0	0	0	0	0	0	0	0	0	0	0	0
0	0	0	0	0	0	0	0	0	0	0	0	0	0
0	0	0	0	0	0	0	0	0	0	0	0	0	0
0	0	0	0	0	0	0	0	0	0	0	0	0	0
0													
OTU15641	0	0	0	0	0	0	0	0	0	1	0	0	0
0	0	0	0	0	0	0	0	0	0	0	0	0	0
0	0	0	0	0	0	0	0	0	0	0	0	0	0
0	0	0	0	0	0	0	0	0	0	0	0	0	0
0	0	0	0	0	0	0	0	0	0	0	0	0	0
0	0	0	0	0	0	0	0	0	0	0	0	0	0
0	0	0	0	0	0	0	0	0	0	0	0	0	0
0	0	0	0	0	0	0	0	0	0	0	0	0	0
0													
OTU14504	0	0	0	0	0	0	0	0	0	1	0	0	0
0	0	0	0	0	0	0	0	0	0	0	0	0	0
0	0	0	0	0	0	0	0	0	0	0	0	0	0
0	0	0	0	0	0	0	0	0	0	0	0	0	0
0	0	0	0	0	0	0	0	0	0	0	0	0	0
0	0	0	0	0	0	0	0	0	0	0	0	0	0
0	0	0	0	0	0	0	0	0	0	0	0	0	0
0	0	0	0	0	0	0	0	0	0	0	0	0	0
0													
OTU7677	0	0	0	0	0	0	0	0	0	1	0	0	0
0	0	0	0	0	0	0	0	0	0	0	0	0	0
0	0	0	0	0	0	0	0	0	0	0	0	0	0
0	0	0	0	0	0	0	0	0	0	0	0	0	0
0	0	0	0	0	0	0	0	0	0	0	0	0	0
0	0	0	0	0	0	0	0	0	0	0	0	0	0
0	0	0	0	0	0	0	0	0	0	0	0	0	0
0	0	0	0	0	0	0	0	0	0	0	0	0	0
0													

OTU5583	0	0	0	0	0	0	0	0	0	1	0	0	0
0	0	0	0	0	0	0	0	0	0	0	1	0	0
1	0	0	0	0	0	0	0	0	4	0	0	0	0
4	21	0	6	0	0	0	0	0	1	0	0	0	0
0	0	0	0	1	0	0	0	0	0	0	0	0	0
0	0	0	0	1	0	0	1	0	0	0	4	0	1
1	0	0	0	0	0	0	0	0	0	0	0	0	0
0	1	0	0	0	0	0	0	0	0	0	0	0	0
0													
OTU12402	0	0	0	0	0	0	0	0	0	1	0	0	0
0	0	0	0	0	0	0	0	0	0	0	0	0	0
0	0	0	0	0	0	0	0	0	0	0	0	0	0
0	0	0	0	0	0	0	0	0	0	0	0	0	0
0	0	0	0	0	0	0	0	0	0	0	0	0	0
0	0	0	0	0	0	0	0	0	0	0	0	0	0
0	0	0	0	0	0	0	0	0	0	0	0	0	0
0	0	0	0	0	0	0	0	0	0	0	0	0	0
0													
OTU14336	0	0	0	0	0	0	0	0	0	1	0	0	0
0	0	0	0	0	0	0	0	0	0	0	0	1	0
0	0	0	0	0	0	0	0	0	0	0	0	0	0
1	0	0	0	0	0	0	1	0	0	0	0	0	0
0	0	0	0	0	0	0	0	0	0	0	0	0	0
0	1	0	0	0	0	0	0	0	0	0	0	0	0
0	0	0	0	0	0	0	0	0	0	0	0	0	0
0	0	0	8	0	1	2	0	0	9	0	0	0	7
10													
OTU307	0	0	0	0	0	0	0	0	0	2	0	4	0
0	0	4	1	0	0	0	0	0	0	0	0	0	0
29	0	0	0	0	0	0	0	0	0	0	0	0	0
1	15	0	7	0	0	0	0	0	0	0	4	0	0
0	0	0	0	0	8	0	2	25	1	2	0	9	0
1	6	0	0	0	5	1	3	5	1	0	266	4	1

0	14	1	0	0	0	0	0	0	0	0	3	3	0
0	5	0	9	10	4	1	10	1	1	0	1	0	19
4													
OTU1718	0	0	0	0	0	0	0	0	0	1	0	1	0
0	0	0	0	0	0	0	0	0	0	0	0	0	0
0	0	0	0	0	0	0	0	0	0	0	0	0	0
0	0	0	0	0	0	0	0	0	0	0	0	0	0
0	0	0	0	0	0	0	0	0	0	0	0	0	0
0	0	0	0	0	0	0	0	0	0	0	0	0	0
0	0	0	0	0	0	0	0	0	0	0	0	0	0
0	0	0	0	0	2	0	0	0	0	0	0	0	0
0													
OTU7678	0	0	0	0	0	0	0	0	0	1	0	0	0
0	0	0	0	0	0	0	0	0	0	0	0	0	0
0	0	0	0	0	0	0	0	0	0	0	0	0	0
0	0	0	0	0	0	0	0	0	0	0	0	0	0
0	0	0	0	0	0	0	0	0	0	0	0	0	0
0	0	0	0	0	0	0	0	0	0	0	0	0	0
0	0	0	0	0	0	0	0	0	0	0	0	0	0
0	0	0	0	0	0	0	0	0	0	0	0	0	0
0													
OTU12398	0	0	0	0	0	0	0	0	0	1	0	0	0
0	0	0	0	0	0	0	0	0	0	0	0	0	0
0	0	0	0	0	0	0	0	0	0	0	0	0	0
0	0	0	0	0	0	0	0	0	0	0	0	0	0
0	0	0	0	0	0	0	0	0	0	0	0	0	0
0	0	0	0	0	0	0	0	0	0	0	0	0	0
0	0	0	0	0	0	0	0	0	0	0	0	0	0
0	0	0	0	0	0	0	0	0	0	0	0	0	0
0													
OTU15642	0	0	0	0	0	0	0	0	0	1	0	0	0
0	0	0	0	0	0	0	0	0	0	0	0	0	0
0	0	0	0	0	0	0	0	0	0	0	0	0	0

0	0	0	0	0	0	0	0	0	0	0	0	0	0
0	0	0	0	0	0	0	0	0	0	0	0	0	0
0	0	0	0	0	0	0	0	0	0	0	0	0	0
0	0	0	0	0	0	0	0	0	0	0	0	0	0
0	0	0	0	0	0	0	0	0	0	0	0	0	0
0													
OTU15643	0	0	0	0	0	0	0	0	0	1	0	0	0
0	0	0	0	0	0	0	0	0	0	0	0	0	0
0	0	0	0	0	0	0	0	0	0	0	0	0	0
0	0	0	0	0	0	0	0	0	0	0	0	0	0
0	0	0	0	0	0	0	0	0	0	0	0	0	0
0	0	0	0	0	0	0	0	0	0	0	0	0	0
0	0	0	0	0	0	0	0	0	0	0	0	0	0
0	0	0	0	0	0	0	0	0	0	0	0	0	0
0													
OTU14500	0	0	0	0	0	0	0	0	0	1	0	0	0
0	0	0	0	0	0	0	0	0	0	0	0	0	0
0	0	0	0	0	0	0	0	0	0	0	0	0	0
0	0	0	0	0	0	0	0	0	0	0	0	0	0
0	0	0	0	0	0	0	0	0	0	0	0	0	0
0	0	0	0	0	0	0	0	0	0	0	0	0	0
0	0	0	0	0	0	0	0	0	0	0	0	0	0
0	0	0	0	0	0	0	0	0	0	0	0	0	0
0													
OTU14501	0	0	0	0	0	0	0	0	0	1	0	0	0
0	0	0	0	0	0	0	0	0	0	0	0	0	0
0	0	0	0	0	0	0	0	0	0	0	0	0	0
0	0	0	0	0	0	0	0	0	0	0	0	0	0
0	0	0	0	0	0	0	0	0	0	0	0	0	0
0	0	0	0	0	0	0	0	0	0	0	0	0	0
0	0	0	0	0	0	0	0	0	0	0	0	0	0
0	0	0	0	0	0	0	0	0	0	0	0	0	0
0													

OTU7667 0 0 0 0 0 0 0 0 0 2 0 0 0
0 0 0 0 0 0 0 0 0 0 0 0 0 0
0 0 0 0 0 0 0 0 0 0 0 0 0 0
0 0 0 0 0 0 0 0 0 0 0 0 0 0
0 0 0 0 0 0 0 0 0 0 0 0 1 0
0 0 0 0 0 0 0 0 0 1 0 0 0 0
0 0 0 0 0 0 0 0 0 0 0 2 1 0
0 2 0 1 8 13 0 1 0 1 0 0 0 3
2
OTU7671 0 0 0 0 0 0 0 0 0 1 0 0 0
0 0 0 0 0 0 0 0 0 0 0 0 0 0
0 0 0 0 0 0 0 0 0 0 0 0 0 0
0 0 0 0 0 0 0 0 0 0 0 0 0 0
0 0 0 0 0 0 0 0 0 0 0 0 0 0
0 0 0 0 0 0 0 0 0 0 0 0 0 0
0 0 0 0 0 0 0 0 0 0 0 0 0 0
0 0 0 0 0 0 0 0 0 0 0 0 0 0
0
OTU12399 0 0 0 0 0 0 0 0 0 1 0 0 0
0 0 0 0 0 0 0 0 0 0 0 0 0 0
0 0 0 0 0 0 0 0 0 0 0 0 0 0
0 0 0 0 0 0 0 0 0 0 0 0 0 0
0 0 0 0 0 0 0 0 0 0 0 0 0 0
0 0 0 0 0 0 0 0 0 0 0 0 0 0
0 0 0 0 0 0 0 0 0 0 0 0 0 0
0 0 0 0 0 0 0 0 0 0 0 0 0 0
0
OTU15592 0 0 0 0 0 0 0 0 0 2 0 0 0
0 0 0 0 0 0 0 0 0 0 0 0 0 1
0 0 0 1 0 0 0 0 0 0 0 0 0 0
0 0 0 0 0 0 0 0 0 0 0 0 0 0
0 0 0 0 0 0 0 0 0 0 0 0 0 0
0 0 0 0 0 0 0 0 0 0 0 0 0 0

0	0	0	0	0	0	0	0	0	0	0	0	0	0
0	0	0	0	0	0	0	0	0	0	0	0	0	0
0													
OTU1124	0	0	0	0	0	0	0	0	0	1	0	0	0
0	0	0	0	0	0	0	0	0	0	0	0	0	0
0	0	0	0	0	0	0	0	0	0	0	0	0	0
0	0	0	0	0	0	0	0	0	0	0	0	0	0
0	2	0	0	0	0	0	0	0	0	0	1	0	0
0	1	0	0	1	0	0	0	0	0	0	0	0	2
0	0	0	0	0	1	0	0	0	1	0	1	0	0
0	1	0	0	0	0	0	0	0	1	0	0	0	0
0													
OTU14505	0	0	0	0	0	0	0	0	0	1	0	0	0
0	0	0	0	0	0	0	0	0	0	0	0	0	0
0	0	0	0	0	0	0	0	0	0	0	0	0	0
0	0	0	0	0	0	0	0	0	0	0	0	0	0
0	0	0	0	0	0	0	0	0	0	0	0	0	0
0	0	0	0	0	0	0	0	0	0	0	0	0	0
0	0	0	0	0	0	0	0	0	0	0	0	0	0
0	0	0	0	0	0	0	0	0	0	0	0	0	0
0													
OTU11911	0	0	0	0	0	0	0	0	0	1	0	0	0
0	0	0	0	0	0	0	0	0	1	0	0	0	0
0	0	0	0	0	0	1	0	0	0	2	1	0	0
0	0	0	0	0	0	0	0	0	0	0	0	0	0
0	0	0	1	0	0	0	0	0	0	0	0	0	0
1	0	0	0	0	0	1	1	0	0	0	0	0	0
0	0	0	0	0	0	0	0	0	0	0	0	0	0
0	0	0	0	0	0	0	0	0	0	0	0	0	0
0													
OTU15639	0	0	0	0	0	0	0	0	0	1	0	0	0
0	0	0	0	0	0	0	0	0	0	0	0	0	0
0	0	0	0	0	0	0	0	0	0	0	0	0	0

0 0 0 0 0 0 0 0 0 0 0 0 0 0
0 0 0 0 0 0 0 0 0 0 0 0 0 0
0 0 0 0 0 0 0 0 0 0 0 0 0 0
0 0 0 0 0 0 0 0 0 0 0 0 0 0
0 0 0 0 0 0 0 0 0 0 0 0 0 0
0
OTU15638 0 0 0 0 0 0 0 0 0 1 0 0 0
0 0 0 0 0 0 0 0 0 0 0 0 0 0
0 0 0 0 0 0 0 0 0 0 0 0 0 0
0 0 0 0 0 0 0 0 0 0 0 0 0 0
0 0 0 0 0 0 0 0 0 0 0 0 0 0
0 0 0 0 0 0 0 0 0 0 0 0 0 0
0 0 0 0 0 0 0 0 0 0 0 0 0 0
0 0 0 0 0 0 0 0 0 0 0 0 0 0
0
OTU14506 0 0 0 0 0 0 0 0 0 1 0 0 0
0 0 0 0 0 0 0 0 0 0 0 0 0 0
0 0 0 0 0 0 0 0 0 0 0 0 0 0
0 0 0 0 0 0 0 0 0 0 0 0 0 0
0 0 0 0 0 0 0 0 0 0 0 0 0 0
0 0 0 0 0 0 0 0 0 0 0 0 0 0
0 0 0 0 0 0 0 0 0 0 0 0 0 0
0 0 0 0 0 0 0 0 0 0 0 0 0 0
0
OTU7676 0 0 0 0 0 0 0 0 0 1 0 0 0
0 0 0 0 0 0 0 0 0 0 0 0 0 0
0 0 0 0 0 0 0 0 0 0 0 0 0 0
0 0 0 0 0 0 0 0 0 0 0 0 0 0
0 0 0 0 0 0 0 0 0 0 0 0 0 0
0 0 0 0 0 0 0 0 0 0 0 0 0 0
0 0 0 0 0 0 0 0 0 0 0 0 0 0
0 0 0 0 0 0 0 0 0 0 0 0 0 0
0

OTU3083	0	0	0	0	0	0	0	0	0	1	0	0	0
0	0	0	0	0	0	0	0	0	0	0	0	0	0
0	0	0	0	0	0	0	0	0	0	0	0	0	0
0	0	0	0	0	0	0	0	0	0	0	0	0	0
0	0	0	0	0	0	0	0	0	0	0	0	0	0
0	0	0	0	0	0	0	0	0	0	0	0	0	0
0	0	0	0	0	0	0	0	0	0	0	0	0	0
0	0	0	0	1	0	0	0	0	0	0	0	0	0
0													
OTU7617	0	0	0	0	0	0	0	0	0	2	0	0	0
0	0	0	0	0	0	0	0	0	0	0	0	0	0
0	0	0	0	0	0	0	0	0	0	0	0	0	0
0	0	0	0	0	0	0	0	0	0	0	0	0	0
0	0	0	0	0	0	0	0	0	0	0	0	0	0
0	0	0	0	0	0	0	0	0	0	0	0	0	0
0	0	0	0	0	0	0	0	0	0	0	0	0	0
0	0	0	0	0	0	0	0	0	0	0	0	0	0
0													
OTU267	0	0	0	0	0	0	0	0	0	3	0	0	0
0	0	0	0	0	0	0	0	0	0	0	0	0	0
0	0	0	0	0	0	0	0	0	0	0	0	0	0
3	0	0	0	0	0	0	0	0	0	0	0	0	0
0	0	0	1	0	0	0	0	0	0	0	0	0	0
0	0	0	0	1	1	0	0	0	2	0	0	229	0
0	0	0	0	0	0	0	0	0	0	0	0	0	0
0	0	0	0	0	0	0	0	0	0	0	0	0	0
0													
OTU12431	0	0	0	0	0	0	0	0	0	1	0	0	0
0	0	0	0	0	0	0	0	0	0	0	0	0	0
0	0	0	0	0	0	0	0	0	0	0	0	0	0
0	0	0	0	0	0	0	0	0	0	0	0	0	0
0	0	0	0	0	0	0	0	0	0	0	0	0	0
0	0	0	0	0	0	0	0	0	0	0	0	0	0

0	0	0	0	0	0	0	0	0	0	0	0	0	0
0	0	0	0	0	0	0	0	0	0	0	0	0	0
0													
OTU15631	0	0	0	0	0	0	0	0	0	1	0	0	0
0	0	0	0	0	0	0	0	0	0	0	0	0	0
0	0	0	0	0	0	0	0	0	0	0	0	0	0
0	0	0	0	0	0	0	0	0	0	0	0	0	0
0	0	0	0	0	0	0	0	0	0	0	0	0	0
0	0	0	0	0	0	0	0	0	0	0	0	0	0
0	0	0	0	0	0	0	0	0	0	0	0	0	0
0	0	0	0	0	0	0	0	0	0	0	0	0	0
0													
OTU331	0	0	0	0	0	0	0	0	0	1	0	0	0
0	0	1	0	1	0	0	0	0	0	0	0	0	0
0	0	0	0	0	1	0	0	0	1	0	0	0	1
0	0	0	0	0	0	0	1	0	0	0	1	0	0
0	0	0	0	0	0	0	0	0	0	0	0	0	0
0	12	0	0	0	1	0	1	0	0	2	0	1	0
0	0	1	1	0	0	0	0	0	0	3	30	0	1
1	14	1	0	45	3	2	35	0	2	1	2	1	2
0													
OTU11548	0	0	0	0	0	0	0	0	0	1	0	0	0
0	2	0	0	0	0	2	0	0	0	0	0	0	0
0	0	0	0	0	0	0	0	0	0	0	0	0	0
0	0	0	0	0	0	0	0	0	0	0	0	0	0
0	0	0	0	0	0	0	0	0	0	0	0	0	0
0	1	0	0	0	0	0	0	0	0	0	0	0	0
0	0	0	1	0	0	0	0	0	0	0	0	0	0
0	1	0	0	1	0	0	3	1	1	0	1	0	0
0													
OTU12430	0	0	0	0	0	0	0	0	0	1	0	0	0
0	0	0	0	0	0	0	0	0	0	0	0	0	0
0	0	0	0	0	0	0	0	0	0	0	0	0	0

0	0	0	0	0	0	0	0	0	0	0	0	0	0
0	0	0	0	0	0	0	0	0	0	0	0	0	0
0	0	0	0	0	0	0	0	0	0	0	0	0	0
0	0	0	0	0	0	0	0	0	0	0	0	0	0
0	0	0	0	0	0	0	0	0	0	0	0	0	0
0													
OTU5662	0	0	0	0	0	0	0	0	0	2	0	0	1
1	0	0	0	1	15	0	0	0	0	0	0	0	0
0	0	0	0	0	0	0	0	0	1	0	0	0	0
1	0	1	0	0	1	0	1	0	0	0	0	0	1
0	0	0	0	0	3	0	0	0	0	0	0	0	0
0	35	0	0	0	0	0	2	0	0	0	0	0	0
0	0	0	0	0	0	0	0	0	0	0	21	24	1
0	86	7	16	32	7	5	9	4	30	0	3	0	26
10													
OTU7620	0	0	0	0	0	0	0	0	0	1	0	0	0
0	0	0	0	0	0	0	0	0	0	0	0	0	0
0	0	0	0	0	0	0	0	0	0	0	0	0	0
0	0	0	0	0	0	0	0	0	0	0	0	0	0
0	0	0	0	0	0	0	0	0	0	0	0	0	0
0	0	0	0	0	0	0	0	0	0	0	0	0	0
0	0	0	0	0	0	0	0	0	0	0	0	0	0
0	0	0	0	0	0	0	0	0	0	0	0	0	0
0													
OTU7614	0	0	0	0	0	0	0	0	0	1	0	0	0
0	0	0	0	0	0	0	0	0	0	0	0	0	0
0	0	0	0	0	0	0	0	0	0	0	0	0	0
0	0	0	0	0	0	0	0	0	0	0	0	0	0
0	0	0	0	0	0	0	0	0	0	0	0	0	0
0	0	0	0	0	0	0	0	0	0	0	0	0	0
0	0	0	0	0	0	0	0	0	0	0	0	0	0
0	0	0	0	0	0	0	0	0	0	0	0	0	0
0													

OTU15629	0	0	0	0	0	0	0	0	0	1	0	0	0
0	0	0	0	0	0	0	0	0	0	0	0	0	0
0	0	0	0	0	0	0	0	0	0	0	0	0	0
0	0	0	0	0	0	0	0	0	0	0	0	0	0
0	0	0	0	0	0	0	0	0	0	0	0	0	0
0	0	0	0	0	0	0	0	0	0	0	0	0	0
0	0	0	0	0	0	0	0	0	0	0	0	0	0
0	0	0	0	0	0	0	0	0	0	0	0	0	0
0													
OTU7615	0	0	0	0	0	0	0	0	0	1	0	0	0
0	0	0	0	0	0	0	0	0	0	0	0	0	0
0	0	0	0	0	0	0	0	0	0	0	0	0	0
0	0	0	0	0	0	0	0	0	0	0	0	0	0
0	0	0	0	0	0	0	0	0	0	0	0	0	0
0	0	0	0	0	0	0	0	0	0	0	0	0	0
0	0	0	0	0	0	0	0	0	0	0	0	0	0
0	0	0	0	0	0	0	0	0	0	0	0	0	0
0													
OTU2281	0	0	0	0	0	0	0	0	0	3	0	0	0
0	0	0	0	0	0	0	0	0	0	0	0	0	0
0	0	0	0	0	0	0	0	0	0	0	0	0	0
0	0	0	0	0	14	0	1	0	1	0	2	0	0
0	14	0	1	0	0	0	0	0	0	0	0	35	0
0	3	0	0	0	0	0	0	0	0	0	0	0	0
0	0	0	0	0	0	0	1	0	0	0	1	2	0
0	5	0	0	0	0	0	2	2	1	0	0	0	0
0													
OTU4248	0	0	0	0	0	0	0	0	0	1	0	0	0
0	0	0	0	0	0	0	0	0	0	0	0	0	0
0	0	0	0	0	0	0	0	0	0	0	0	0	0
0	0	0	0	0	0	0	0	0	0	0	0	0	0
0	0	0	0	0	0	0	0	0	0	0	0	0	0
0	0	0	0	0	0	0	0	0	0	0	0	0	0

0	0	0	0	0	0	0	0	0	0	0	0	0	0
0	0	0	0	0	0	0	0	0	0	0	0	0	0
0													
OTU2493	0	0	0	0	0	0	0	0	0	1	0	1	0
0	0	0	0	0	0	0	0	0	0	0	0	0	0
0	0	0	0	0	0	0	0	0	1	0	0	0	0
0	0	0	0	0	0	0	0	0	0	0	0	0	0
0	0	0	0	0	0	0	0	0	0	0	0	0	0
0	0	0	0	0	0	0	0	1	0	0	0	0	0
0	0	0	0	0	0	0	0	0	1	4	0	0	0
0	0	0	0	0	0	0	0	0	0	0	1	0	0
0													
OTU413	0	0	0	0	0	0	0	0	0	1	0	0	0
0	0	0	0	0	0	0	0	0	0	0	0	1	0
0	0	0	0	0	0	0	0	0	0	0	0	0	0
0	0	0	0	7	0	0	0	0	0	0	0	0	0
0	0	0	0	0	0	0	0	0	0	0	0	1	0
1	3	1	0	0	0	0	0	0	0	2	0	0	0
0	0	0	0	0	0	0	0	0	0	0	2	6	0
0	4	1	10	2	4	2	0	4	18	0	0	0	15
2													
OTU8167	0	0	0	0	0	0	0	0	0	1	0	1	0
0	0	0	0	0	0	0	0	0	0	0	0	0	0
0	1	0	0	0	0	0	0	0	0	0	0	0	0
0	0	0	0	0	0	0	1	0	0	0	0	0	0
0	0	0	1	0	0	0	0	0	0	0	0	1	0
0	0	0	0	0	0	0	0	0	0	0	0	0	0
0	0	0	0	0	0	0	0	0	0	0	0	0	0
0	22	3	4	0	0	1	0	0	0	1	0	0	0
17													
OTU12435	0	0	0	0	0	0	0	0	0	1	0	0	0
0	0	0	0	0	0	0	0	0	0	0	0	0	0
0	0	0	0	0	0	0	0	0	0	0	0	0	0

0	0	0	0	0	0	0	0	0	0	0	0	0	0
0	0	0	0	0	0	0	0	0	0	0	0	0	0
0	0	0	0	0	0	0	0	0	0	0	0	0	0
0	0	0	0	0	0	0	0	0	0	0	0	0	0
0	0	0	0	0	0	0	0	0	0	0	0	0	0
0													
OTU7616	0	0	0	0	0	0	0	0	0	1	0	0	0
0	0	0	0	0	0	0	0	0	0	0	0	0	0
0	0	0	0	0	0	0	0	0	0	0	0	0	0
0	0	0	0	0	0	0	0	0	0	0	0	0	0
0	0	0	0	0	0	0	0	0	0	0	0	0	0
0	0	0	0	0	0	0	0	0	0	0	0	0	0
0	0	0	0	0	0	0	0	0	0	0	0	0	0
0	0	0	0	0	0	0	0	0	0	0	0	0	0
0													
OTU15630	0	0	0	0	0	0	0	0	0	1	0	0	0
0	0	0	0	0	0	0	0	0	0	0	0	0	0
0	0	0	0	0	0	0	0	0	0	0	0	0	0
0	0	0	0	0	0	0	0	0	0	0	0	0	0
0	0	0	0	0	0	0	0	0	0	0	0	0	0
0	0	0	0	0	0	0	0	0	0	0	0	0	0
0	0	0	0	0	0	0	0	0	0	0	0	0	0
0	0	0	0	0	0	0	0	0	0	0	0	0	0
0													
OTU6653	0	0	0	0	0	0	0	0	0	1	0	3	0
0	0	0	0	0	12	0	0	0	0	0	0	0	1
0	0	0	0	0	0	0	0	0	0	0	0	0	0
0	2	0	13	0	1	0	0	0	0	0	0	0	0
0	0	0	0	0	0	0	0	0	0	0	0	1	0
0	4	1	0	1	0	0	0	0	1	0	0	0	0
0	0	0	2	0	0	0	2	0	0	2	4	3	0
0	5	0	1	6	0	0	5	0	3	0	1	0	0
3													

OTU15635	0	0	0	0	0	0	0	0	0	1	0	0	0
0	0	0	0	0	0	0	0	0	0	0	0	0	0
0	0	0	0	0	0	0	0	0	0	0	0	0	0
0	0	0	0	0	0	0	0	0	0	0	0	0	0
0	0	0	0	0	0	0	0	0	0	0	0	0	0
0	0	0	0	0	0	0	0	0	0	0	0	0	0
0	0	0	0	0	0	0	0	0	0	0	0	0	0
0	0	0	0	0	0	0	0	0	0	0	0	0	0
0													
OTU7627	0	0	0	0	0	0	0	0	0	1	0	0	0
0	0	0	0	0	0	0	0	0	0	0	0	0	0
0	0	0	0	0	0	0	0	0	0	0	0	0	0
0	0	0	0	0	0	0	0	0	0	0	0	0	0
0	0	0	0	0	0	0	0	0	0	0	0	0	0
0	0	0	0	0	0	0	0	0	0	0	0	0	0
0	0	0	0	0	0	0	0	0	0	0	0	0	0
0	0	0	0	0	0	0	0	0	0	0	0	0	0
0													
OTU7626	0	0	0	0	0	0	0	0	0	1	0	0	0
0	0	0	0	0	0	0	0	0	0	0	0	0	0
0	0	0	0	0	0	0	0	0	0	0	0	0	0
0	0	0	0	0	0	0	0	0	0	0	0	0	0
0	0	0	0	0	0	0	0	0	0	0	0	0	0
0	0	0	0	0	0	0	0	0	0	0	0	0	0
0	0	0	0	0	0	0	0	0	0	0	0	0	0
0	0	0	0	0	0	0	0	0	0	0	0	0	0
0													
OTU12437	0	0	0	0	0	0	0	0	0	1	0	0	0
0	0	0	0	0	0	0	0	0	0	0	0	0	0
0	0	0	0	0	0	0	0	0	0	0	0	0	0
0	0	0	0	0	0	0	0	0	0	0	0	0	0
0	0	0	0	0	0	0	0	0	0	0	0	0	0
0	0	0	0	0	0	0	0	0	0	0	0	0	0

0	0	0	0	0	0	0	0	0	0	0	0	0	0
0	0	0	0	0	0	0	0	0	0	0	0	0	0
0													
OTU4246	0	0	0	0	0	0	0	0	0	1	0	0	0
0	0	0	0	0	0	0	0	0	0	0	0	0	0
0	0	0	0	0	0	0	0	0	0	0	0	0	0
0	0	0	0	0	0	0	0	0	0	0	0	0	0
0	0	0	0	0	0	0	0	0	0	0	0	0	0
0	0	0	0	0	0	0	0	0	0	0	0	0	0
0	0	0	0	0	0	0	0	0	0	0	0	0	0
0	0	0	0	0	0	0	0	0	0	0	0	0	0
0													
OTU16095	0	0	0	0	0	0	0	0	0	1	0	0	0
0	0	0	0	0	0	0	0	0	1	1	0	0	0
0	0	0	0	0	0	0	0	0	0	0	0	0	0
0	0	0	0	0	0	0	0	0	0	0	0	0	0
0	1	0	0	0	0	0	0	0	0	0	0	0	0
0	0	0	0	0	0	0	0	0	0	0	0	0	0
0	1	0	0	0	0	0	0	0	0	0	0	0	0
0	0	0	0	0	0	0	0	0	0	0	0	0	0
0													
OTU7628	0	0	0	0	0	0	0	0	0	1	0	0	0
0	0	0	0	0	0	0	0	0	0	0	0	0	0
0	0	0	0	0	0	0	0	0	0	0	0	0	0
0	0	0	0	0	0	0	0	0	0	0	0	0	0
0	0	0	0	0	0	0	0	0	0	0	0	0	0
0	0	0	0	0	0	0	0	0	0	0	0	0	0
0	0	0	0	0	0	0	0	0	0	0	0	0	0
0	0	0	0	0	0	0	0	0	0	0	0	0	0
0													
OTU8236	0	0	0	0	0	0	0	0	0	1	0	0	0
0	0	0	0	0	0	0	0	0	0	0	0	0	0
0	0	0	0	0	0	0	0	0	0	0	0	0	0

0	0	0	0	0	0	0	0	0	0	0	0	0	0
0	0	0	0	0	0	0	0	0	0	0	0	0	0
0	0	0	0	0	0	0	0	0	0	0	0	0	0
0	0	0	0	0	0	0	0	0	0	0	0	0	0
0	0	0	0	0	1	0	0	3	1	0	0	0	3
0													
OTU4245	0	0	0	0	0	0	0	0	0	1	0	0	0
0	0	0	0	0	0	0	0	0	0	0	0	0	0
0	0	0	0	0	0	0	0	0	0	0	0	0	0
0	0	0	0	0	0	0	0	0	0	0	0	0	0
0	0	0	0	0	0	0	0	0	0	0	0	0	0
0	0	0	0	0	0	0	0	0	0	0	0	0	0
0	0	0	0	0	0	0	0	0	0	0	0	0	0
0	0	0	0	0	0	0	0	0	0	0	0	0	0
0													
OTU1373	0	0	0	0	0	0	0	0	0	1	0	0	0
4	0	0	0	0	0	1	0	0	0	0	0	0	0
0	0	0	0	0	0	0	1	0	1	0	1	0	4
0	0	0	0	0	0	0	0	0	0	0	0	0	0
0	0	0	0	0	0	0	0	0	0	0	1	0	0
0	0	0	6	0	0	1	0	5	0	0	0	0	0
2	0	1	0	0	0	1	0	0	0	1	0	0	0
1	0	0	0	0	0	0	0	0	0	1	1	0	0
0													
OTU4043	0	0	0	0	0	0	0	0	0	1	0	0	0
0	0	0	0	1	0	0	0	0	0	0	0	1	0
0	0	1	0	0	0	0	0	0	0	0	1	0	0
0	0	1	0	0	0	0	0	0	1	0	0	0	0
0	1	0	0	0	0	0	0	0	0	0	0	0	0
0	0	0	0	0	0	0	0	0	0	0	1	0	0
0	0	0	1	0	0	0	0	0	0	0	0	1	0
0	0	0	0	1	1	0	0	0	0	0	0	0	0
0													

OTU4247	0	0	0	0	0	0	0	0	0	1	0	0	0
0	0	0	0	0	0	0	0	0	0	0	0	0	0
0	0	0	0	0	0	0	0	0	0	0	0	0	0
0	0	0	0	0	0	0	0	0	0	0	0	0	0
0	0	0	0	0	0	0	0	0	0	0	0	0	0
0	0	0	0	0	0	0	0	0	0	0	0	0	0
0	0	0	0	0	0	0	0	0	0	0	0	0	0
0	0	0	0	0	0	0	0	0	0	0	0	0	0
0													
OTU460	0	0	0	0	0	0	0	0	0	2	0	0	0
0	0	0	0	0	0	0	0	0	0	0	0	1	0
0	0	0	0	0	0	0	0	0	0	0	0	1	0
0	0	0	0	0	0	0	1	0	0	0	0	0	0
0	0	0	0	0	0	0	0	0	0	0	0	1	0
0	2	0	0	1	0	0	0	0	0	0	10	0	1
0	1	0	0	0	0	0	0	0	0	0	9	0	0
0	15	11	0	12	13	15	8	6	2	0	0	0	0
6													
OTU15636	0	0	0	0	0	0	0	0	0	1	0	0	0
0	0	0	0	0	0	0	0	0	0	0	0	0	0
0	0	0	0	0	0	0	0	0	0	0	0	0	0
0	0	0	0	0	0	0	0	0	0	0	0	0	0
0	0	0	0	0	0	0	0	0	0	0	0	0	0
0	0	0	0	0	0	0	0	0	0	0	0	0	0
0	0	0	0	0	0	0	0	0	0	0	0	0	0
0	0	0	0	0	0	0	0	0	0	0	0	0	0
0													
OTU12433	0	0	0	0	0	0	0	0	0	1	0	0	0
0	0	0	0	0	0	0	0	0	0	0	0	0	0
0	0	0	0	0	0	0	0	0	0	0	0	0	0
0	0	0	0	0	0	0	0	0	0	0	0	0	0
0	0	0	0	0	0	0	0	0	0	0	0	0	0
0	0	0	0	0	0	0	0	0	0	0	0	0	0

0 0 0 0 0 0 0 0 0 0 0 0 0 0
0 0 0 0 0 0 0 0 0 0 0 0 0 0
0
OTU15632 0 0 0 0 0 0 0 0 0 1 0 0 0
0 0 0 0 0 0 0 0 0 0 0 0 0 0
0 0 0 0 0 0 0 0 0 0 0 0 0 0
0 0 0 0 0 0 0 0 0 0 0 0 0 0
0 0 0 0 0 0 0 0 0 0 0 0 0 0
0 0 0 0 0 0 0 0 0 0 0 0 0 0
0 0 0 0 0 0 0 0 0 0 0 0 0 0
0 0 0 0 0 0 0 0 0 0 0 0 0 0
0
OTU540 0 0 0 0 0 0 0 0 0 1 0 0 0
0 0 0 0 0 0 1 0 0 0 0 0 0 0
1 0 1 0 0 0 0 0 0 0 0 0 0 0
1 0 0 0 0 0 0 0 0 0 0 1 0 0
0 0 0 5 0 1 0 7 10 1 0 0 0 0
0 2 0 0 0 0 0 0 0 0 0 0 1 0
0 0 0 0 0 0 0 0 0 0 0 6 2 0
0 4 5 1 10 7 1 6 11 2 0 0 0 4
1
OTU7478 0 0 0 0 0 0 0 0 0 1 0 0 0
0 0 0 0 0 0 0 5 0 0 0 0 0 0
0 0 0 0 0 0 0 0 0 0 0 0 0 0
0 0 0 0 0 0 0 0 0 1 0 0 0 0
0 0 0 0 0 0 0 0 0 0 0 0 0 0
0 0 0 0 0 0 0 0 0 1 0 0 0 0
0 1 0 0 0 1 0 0 0 0 0 0 0 0
0 0 0 0 0 0 0 0 0 0 0 0 0 0
0
OTU12434 0 0 0 0 0 0 0 0 0 1 0 0 0
0 0 0 0 0 0 0 0 0 0 0 0 0 0
0 0 0 0 0 0 0 0 0 0 0 0 0 0

0 0 0 0 0 0 0 0 0 0 0 0 0 0
0 0 0 0 0 0 0 0 0 0 0 0 0 0
0 0 0 0 0 0 0 0 0 0 0 0 0 0
0 0 0 0 0 0 0 0 0 0 0 0 0 0
0 0 0 0 0 0 0 0 0 0 0 0 0 0
0
OTU6789 0 0 0 0 0 0 0 0 0 2 0 0 0
0 0 0 0 0 0 1 0 0 0 0 0 1 0
0 0 0 0 0 0 0 0 0 1 0 0 2 0
2 1 0 0 0 0 0 0 0 0 0 0 0 0
0 0 0 0 0 0 0 0 0 0 0 0 0 0
0 0 0 0 0 0 0 0 0 0 0 0 0 0
0 0 0 2 0 0 0 0 0 0 0 0 0 0
0 0 0 0 0 0 0 0 0 0 0 0 0 0
0
OTU4254 0 0 0 0 0 0 0 0 0 1 0 0 0
0 0 0 0 0 0 0 0 0 0 0 0 0 0
0 0 0 0 0 0 0 0 0 0 0 0 0 0
0 0 0 0 0 0 0 0 0 0 0 0 0 0
0 0 0 0 0 0 0 0 0 0 0 0 0 0
0 0 0 0 0 0 0 0 0 0 0 0 0 0
0 0 0 0 0 0 0 0 0 0 0 0 0 0
0 0 0 0 0 0 0 0 0 0 0 0 0 0
0
OTU4255 0 0 0 0 0 0 0 0 0 1 0 0 0
0 0 0 0 0 0 0 0 0 0 0 0 0 0
0 0 0 0 0 0 0 0 0 0 0 0 0 0
0 0 0 0 0 0 0 0 0 0 0 0 0 0
0 0 0 0 0 0 0 0 0 0 0 0 0 0
0 0 0 0 0 0 0 0 0 0 0 0 0 0
0 0 0 0 0 0 0 0 0 0 0 0 0 0
0 0 0 0 0 0 0 0 0 0 0 0 0 0
0

OTU7623	0	0	0	0	0	0	0	0	0	1	0	0	0
0	0	0	0	0	0	0	0	0	0	0	0	0	0
0	0	0	0	0	0	0	0	0	0	0	0	0	0
0	0	0	0	0	0	0	0	0	0	0	0	0	0
0	0	0	0	0	0	0	0	0	0	0	0	0	0
0	0	0	0	0	0	0	0	0	0	0	0	0	0
0	0	0	0	0	0	0	0	0	0	0	0	0	0
0	0	0	0	0	0	0	0	0	0	0	0	0	0
0													
OTU7622	0	0	0	0	0	0	0	0	0	1	0	0	0
0	0	0	0	0	0	0	0	0	0	0	0	0	0
0	0	0	0	0	0	0	0	0	0	0	0	0	0
0	0	0	0	0	0	0	0	0	0	0	0	0	0
0	0	0	0	0	0	0	0	0	0	0	0	0	0
0	0	0	0	0	0	0	0	0	0	0	0	0	0
0	0	0	0	0	0	0	0	0	0	0	0	0	0
0	0	0	0	0	0	0	0	0	0	0	0	0	0
0													
OTU15633	0	0	0	0	0	0	0	0	0	1	0	0	0
0	0	0	0	0	0	0	0	0	0	0	0	0	0
0	0	0	0	0	0	0	0	0	0	0	0	0	0
0	0	0	0	0	0	0	0	0	0	0	0	0	0
0	0	0	0	0	0	0	0	0	0	0	0	0	0
0	0	0	0	0	0	0	0	0	0	0	0	0	0
0	0	0	0	0	0	0	0	0	0	0	0	0	0
0	0	0	0	0	0	0	0	0	0	0	0	0	0
0													
OTU7606	0	0	0	0	0	0	0	0	0	1	0	0	0
0	0	0	0	0	0	0	0	0	0	0	0	0	0
0	0	0	0	0	0	0	0	0	0	0	0	0	0
0	0	0	0	0	0	0	0	0	0	0	0	0	0
0	0	0	0	0	0	0	0	0	0	0	0	0	0
0	0	0	0	0	0	0	0	0	0	0	0	0	0

0	0	0	0	0	0	0	0	0	0	0	0	0	0
0	0	0	0	0	0	0	0	0	0	0	0	0	0
0													
OTU4251	0	0	0	0	0	0	0	0	0	1	0	0	0
0	0	0	0	0	0	0	0	0	0	0	0	0	0
0	0	0	0	0	0	0	0	0	0	0	0	0	0
0	0	0	0	0	0	0	0	0	0	0	0	0	0
0	0	0	0	0	0	0	0	0	0	0	0	0	0
0	0	0	0	0	0	0	0	0	0	0	0	0	0
0	0	0	0	0	0	0	0	0	0	0	0	0	0
0	0	0	0	0	0	0	0	0	0	0	0	0	0
0													
OTU15655	0	0	0	0	0	0	0	0	0	1	0	0	0
0	0	0	0	1	0	0	0	0	0	0	0	1	0
0	0	0	0	0	0	0	0	0	1	0	0	0	0
0	0	0	0	0	1	0	0	0	0	0	0	0	0
0	3	0	0	0	0	0	0	0	0	0	0	1	0
0	0	0	0	0	0	0	0	0	1	0	0	0	0
0	0	0	0	0	0	0	0	0	0	0	0	0	0
0	0	0	0	0	0	0	0	0	0	0	0	2	1
0													
OTU4317	0	0	0	0	0	0	0	0	0	1	0	5	0
0	0	0	0	0	0	0	0	0	0	0	0	0	0
0	0	0	0	0	0	0	0	0	1	0	0	0	0
0	0	0	0	0	1	0	1	0	0	0	0	0	0
0	0	0	0	0	0	0	0	0	0	0	0	0	0
0	0	0	0	0	0	0	0	0	0	0	0	0	0
0	0	0	0	0	0	0	0	0	1	0	0	0	0
0	0	0	0	0	0	0	0	0	0	1	1	0	0
0													
OTU4252	0	0	0	0	0	0	0	0	0	1	0	0	0
0	0	0	0	0	0	0	0	0	0	0	0	0	0
0	0	0	0	0	0	0	0	0	0	0	0	0	0

0	0	0	0	0	0	0	0	0	0	0	0	0	0
0	0	0	0	0	0	0	0	0	0	0	0	0	0
0	0	0	0	0	0	0	0	0	0	0	0	0	0
0	0	0	0	0	0	0	0	0	0	0	0	0	0
0	0	0	0	0	0	0	0	0	0	0	0	0	0
0													
OTU12424	0	0	0	0	0	0	0	0	0	1	0	0	0
0	0	0	0	0	0	0	0	0	0	0	0	0	0
0	0	0	0	0	0	0	0	0	0	0	0	0	0
0	0	0	0	0	0	0	0	0	0	0	0	0	0
0	0	0	0	0	0	0	0	0	0	0	0	0	0
0	0	0	0	0	0	0	0	0	0	0	0	0	0
0	0	0	0	0	0	0	0	0	0	0	0	0	0
0	0	0	0	0	0	0	0	0	0	0	0	0	0
0													
OTU4253	0	0	0	0	0	0	0	0	0	1	0	0	0
0	0	0	0	0	0	0	0	0	0	0	0	0	0
0	0	0	0	0	0	0	0	0	0	0	0	0	0
0	0	0	0	0	0	0	0	0	0	0	0	0	0
0	0	0	0	0	0	0	0	0	0	0	0	0	0
0	0	0	0	0	0	0	0	0	0	0	0	0	0
0	0	0	0	0	0	0	0	0	0	0	0	0	0
0	0	0	0	0	0	0	0	0	0	0	0	0	0
0													
OTU11224	0	0	0	0	0	0	0	0	0	1	0	2	0
0	0	0	0	0	0	0	0	0	0	0	0	0	0
1	0	0	0	0	0	0	0	0	0	0	0	0	0
0	0	0	0	0	0	0	1	0	0	0	0	0	0
0	0	0	0	0	0	0	0	0	0	0	0	0	0
0	5	0	0	0	0	0	0	0	0	0	0	0	0
0	0	0	0	0	0	0	0	0	0	0	0	1	0
0	0	0	1	0	1	3	0	0	1	0	0	0	1
0													

OTU7608 0 0 0 0 0 0 0 0 0 1 0 0 0
0 0 0 0 0 0 0 0 0 0 0 0 0 0
0 0 0 0 0 0 0 0 0 0 0 0 0 0
0 0 0 0 0 0 0 0 0 0 0 0 0 0
0 0 0 0 0 0 0 0 0 0 0 0 0 0
0 0 0 0 0 0 0 0 0 0 0 0 0 0
0 0 0 0 0 0 0 0 0 0 0 0 0 0
0 0 0 0 0 0 0 0 0 0 0 0 0 0
0
OTU5241 0 0 0 0 0 0 0 0 0 3 0 0 0
0 0 0 0 0 0 0 0 0 0 0 0 0 0
0 0 0 0 0 0 0 0 0 0 1 0 0 0
1 0 0 0 0 0 0 0 0 0 1 0 0 0
0 0 0 0 0 0 0 0 0 0 0 0 0 0
0 1 0 0 0 0 0 0 0 0 0 0 0 0
0 0 0 0 0 0 1 0 0 0 0 1 0 0
0 0 0 1 0 1 0 0 1 1 0 1 0 0
1
OTU12423 0 0 0 0 0 0 0 0 0 1 0 0 0
0 0 0 0 0 0 0 0 0 0 0 0 0 0
0 0 0 0 0 0 0 0 0 0 0 0 0 0
0 0 0 0 0 0 0 0 0 0 0 0 0 0
0 0 0 0 0 0 0 0 0 0 0 0 0 0
0 0 0 0 0 0 0 0 0 0 0 0 0 0
0 0 0 0 0 0 0 0 0 0 0 0 0 0
0 0 0 0 0 0 0 0 0 0 0 0 0 0
0
OTU7607 0 0 0 0 0 0 0 0 0 1 0 0 0
0 0 0 0 0 0 0 0 0 0 0 0 0 0
0 0 0 0 0 0 0 0 0 0 0 0 0 0
0 0 0 0 0 0 0 0 0 0 0 0 0 0
0 0 0 0 0 0 0 0 0 0 0 0 0 0
0 0 0 0 0 0 0 0 0 0 0 0 0 0

0 0 0 1 0 0 0 0 0 0 0 0 0 0
0 0 0 0 0 0 0 0 0 0 0 0 0 0
0
OTU12422 0 0 0 0 0 0 0 0 0 1 0 0 0
0 0 0 0 0 0 0 0 0 0 0 0 0 0
0 0 0 0 0 0 0 0 0 0 0 0 0 0
0 0 0 0 0 0 0 0 0 0 0 0 0 0
0 0 0 0 0 0 0 0 0 0 0 0 0 0
0 0 0 0 0 0 0 0 0 0 0 0 0 0
0 0 0 0 0 0 0 0 0 0 0 0 0 0
0 0 0 0 0 0 0 0 0 0 0 0 0 0
0
OTU7213 0 0 0 0 0 0 0 0 0 1 0 0 0
0 0 0 0 0 0 0 0 0 0 0 0 0 0
0 0 0 0 0 0 0 0 0 0 0 0 0 0
0 0 0 0 0 0 0 0 0 0 0 0 0 0
0 0 0 0 0 0 0 0 0 0 0 0 0 0
0 0 0 0 0 0 0 0 0 1 0 0 0 0
0 0 0 0 0 1 0 0 0 0 0 0 0 0
0 0 0 0 0 0 0 0 0 0 0 0 0 0
0
OTU8542 0 0 0 0 0 0 0 0 0 1 0 0 0
0 1 0 0 1 0 0 0 0 0 0 1 1 0
0 0 0 0 0 1 0 0 0 0 0 0 0 0
0 0 0 0 0 0 0 0 0 0 0 0 0 0
0 0 0 0 0 0 0 0 0 1 0 0 0 0
1 0 0 0 0 0 0 0 0 0 0 0 0 0
0 0 0 0 0 0 0 0 0 0 0 0 0 0
0 0 1 0 0 0 0 1 0 0 0 0 0 0
0
OTU6012 0 0 0 0 0 0 0 0 0 1 0 0 0
0 0 0 0 0 0 1 0 0 0 0 0 1 0
0 0 0 0 0 0 0 0 0 0 0 0 0 0

0	0	0	0	0	0	0	0	0	0	0	0	0	0
0	0	0	0	0	0	0	0	0	0	0	0	0	0
2	0	1	1	0	1	0	0	0	0	0	0	0	0
0	0	0	0	1	0	0	0	0	0	0	0	0	0
0	0	0	0	0	0	0	0	0	0	0	0	0	0
0													
OTU7605	0	0	0	0	0	0	0	0	0	1	0	0	0
0	0	0	0	0	0	0	0	0	0	0	0	0	0
0	0	0	0	0	0	0	0	0	0	0	0	0	0
0	0	0	0	0	0	0	0	0	0	0	0	0	0
0	0	0	0	0	0	0	0	0	0	0	0	0	0
0	0	0	0	0	0	0	0	0	0	0	0	0	0
0	0	0	0	0	0	0	0	0	0	0	0	0	0
0	0	0	0	0	0	0	0	0	0	0	0	0	0
0													
OTU15654	0	0	0	0	0	0	0	0	0	1	0	0	0
0	0	1	0	0	2	0	1	0	0	0	0	0	1
0	0	1	0	0	0	0	0	0	0	0	2	0	0
0	0	1	0	1	0	0	0	1	0	0	0	0	0
0	0	0	1	0	0	0	0	0	0	1	0	0	0
0	0	1	0	3	0	0	0	0	0	0	1	0	0
0	1	0	2	0	0	0	0	0	0	0	0	1	0
0	0	0	0	0	0	0	0	0	0	0	0	0	0
0													
OTU12429	0	0	0	0	0	0	0	0	0	1	0	0	0
0	0	0	0	0	0	0	0	0	0	0	0	0	0
0	0	0	0	0	0	0	0	0	0	0	0	0	0
0	0	0	0	0	0	0	0	0	0	0	0	0	0
0	0	0	0	0	0	0	0	0	0	0	0	0	0
0	0	0	0	0	0	0	0	0	0	0	0	0	0
0	0	0	0	0	0	0	0	0	0	0	0	0	0
0	0	0	0	0	0	0	0	0	0	0	0	0	0
0													

OTU7604	0	0	0	0	0	0	0	0	0	1	0	0	0
0	0	0	0	0	0	0	0	0	0	0	0	0	0
0	0	0	0	0	0	0	0	0	0	0	0	0	0
0	0	0	0	0	0	0	0	0	0	0	0	0	0
0	0	0	0	0	0	0	0	0	0	0	0	0	0
0	0	0	0	0	0	0	0	0	0	0	0	0	0
0	0	0	0	0	0	0	0	0	0	0	0	0	0
0	0	0	0	0	0	0	0	0	0	0	0	0	0
0													
OTU4237	0	0	0	0	0	0	0	0	0	1	0	0	0
0	0	0	0	0	0	0	0	0	0	0	0	0	0
0	0	0	0	0	0	0	0	0	0	0	0	0	0
0	0	0	0	0	0	0	0	0	0	0	0	0	0
0	0	0	0	0	0	0	0	0	0	0	0	0	0
0	0	0	0	0	0	0	0	0	0	0	0	0	0
0	0	0	0	0	0	0	0	0	0	0	0	0	0
0	0	0	0	0	0	0	0	0	0	0	0	0	0
0													
OTU2135	0	0	0	0	0	0	0	0	0	1	0	0	0
0	0	0	0	0	0	0	0	0	0	0	0	1	0
0	0	0	0	0	0	0	0	0	0	0	0	0	0
0	0	0	0	0	0	0	1	0	0	0	0	0	0
0	0	0	0	0	0	0	0	0	0	0	0	0	0
2	0	0	0	0	0	0	0	0	0	0	1	0	0
0	0	0	0	0	0	0	0	0	0	0	0	0	0
0	0	0	0	0	0	0	0	0	0	0	0	0	0
0													
OTU8180	0	0	0	0	0	0	0	0	0	1	0	0	0
0	0	0	0	0	0	0	0	0	0	0	0	0	0
0	0	0	0	0	0	0	0	0	0	0	0	0	0
0	0	0	0	0	0	0	0	0	0	0	0	0	0
0	0	0	0	0	0	0	0	0	0	0	0	0	0
0	0	0	0	0	0	0	0	0	0	0	0	0	0

0	0	0	0	0	0	0	0	0	0	0	0	0	0
0	0	0	1	0	0	0	0	1	0	0	0	0	0
3													
OTU4236	0	0	0	0	0	0	0	0	0	1	0	0	0
0	0	0	0	0	0	0	0	0	0	0	0	0	0
0	0	0	0	0	0	0	0	0	0	0	0	0	0
0	0	0	0	0	0	0	0	0	0	0	0	0	0
0	0	0	0	0	0	0	0	0	0	0	0	0	0
0	0	0	0	0	0	0	0	0	0	0	0	0	0
0	0	0	0	0	0	0	0	0	0	0	0	0	0
0	0	0	0	0	0	0	0	0	0	0	0	0	0
0													
OTU12428	0	0	0	0	0	0	0	0	0	1	0	0	0
0	0	0	0	0	0	0	0	0	0	0	0	0	0
0	0	0	0	0	0	0	0	0	0	0	0	0	0
0	0	0	0	0	0	0	0	0	0	0	0	0	0
0	0	0	0	0	0	0	0	0	0	0	0	0	0
0	0	0	0	0	0	0	0	0	0	0	0	0	0
0	0	0	0	0	0	0	0	0	0	0	0	0	0
0	0	0	0	0	0	0	0	0	0	0	0	0	0
0													
OTU15659	0	0	0	0	0	0	0	0	0	1	0	0	0
0	0	0	0	0	0	0	0	0	0	0	0	0	0
0	0	0	0	0	0	0	0	0	0	0	0	0	0
0	0	0	0	0	0	0	0	0	0	0	0	0	0
0	0	0	0	0	0	0	0	0	0	0	0	0	0
0	0	0	0	0	0	0	0	0	0	0	0	0	0
0	0	0	0	0	0	0	0	0	0	0	0	0	0
0	0	0	0	0	0	0	0	0	0	0	0	0	0
0													
OTU4238	0	0	0	0	0	0	0	0	0	1	0	0	0
0	0	0	0	0	0	0	0	0	0	0	0	0	0
0	0	0	0	0	0	0	0	0	0	0	0	0	0

0	0	0	0	0	0	0	0	0	0	0	0	1	0
0	0	0	0	0	0	0	0	0	0	0	0	0	0
0	0	0	0	0	0	0	0	0	0	0	0	0	0
0	0	0	0	0	0	0	0	0	0	0	0	0	0
0	0	0	0	0	0	0	0	0	0	0	0	0	0
0													
OTU1026	0	0	0	0	0	0	0	0	0	2	0	0	0
0	0	0	0	0	0	0	0	0	0	0	0	0	0
0	0	0	0	0	0	0	0	0	0	0	0	0	0
1	0	0	0	0	0	0	0	0	0	0	0	0	0
0	0	0	0	0	0	0	0	0	0	0	0	1	0
0	1	0	0	0	0	0	0	0	1	0	0	1	1
0	0	0	0	0	0	0	0	0	0	0	0	1	0
0	0	0	2	0	0	0	0	0	0	0	0	0	1
0													
OTU4243	0	0	0	0	0	0	0	0	0	1	0	0	0
0	0	0	0	0	0	0	0	0	0	0	0	0	0
0	0	0	0	0	0	0	0	0	0	0	0	0	0
0	0	0	0	0	0	0	0	0	0	0	0	0	0
0	0	0	0	0	0	0	0	0	0	0	0	0	0
0	0	0	0	0	0	0	0	0	0	0	0	0	0
0	0	0	0	0	0	0	0	0	0	0	0	0	0
0	0	0	0	0	0	0	0	0	0	0	0	0	0
0													
OTU4244	0	0	0	0	0	0	0	0	0	1	0	0	0
0	0	0	0	0	0	0	0	0	0	0	0	0	0
0	0	0	0	0	0	0	0	0	0	0	0	0	0
0	0	0	1	0	0	0	0	0	0	0	0	0	0
0	0	0	0	0	0	0	0	0	0	0	1	0	0
0	0	0	0	0	0	0	0	0	0	0	0	0	0
0	0	0	0	0	0	0	0	0	0	0	0	0	0
0	0	0	0	0	0	0	0	0	0	0	0	0	0
0													

OTU10558	0	0	0	0	0	0	0	0	0	2	0	0	0
0	0	0	0	0	0	0	0	0	0	0	0	0	0
0	0	0	0	0	0	0	0	0	0	0	0	0	0
0	0	0	0	0	0	0	0	0	0	0	0	0	0
0	0	0	0	0	0	0	0	0	0	0	0	0	0
0	0	0	0	0	0	0	0	0	0	0	0	0	0
0	0	0	0	0	0	0	0	0	0	0	0	0	0
0	0	0	0	0	0	0	0	0	0	1	0	0	0
0													
OTU12425	0	0	0	0	0	0	0	0	0	1	0	0	0
0	0	0	0	0	0	0	0	0	0	0	0	0	0
0	0	0	0	0	0	0	0	0	0	0	0	0	0
0	0	0	0	0	0	0	0	0	0	0	0	0	0
0	0	0	0	0	0	0	0	0	0	0	0	0	0
0	0	0	0	0	0	0	0	0	0	0	0	0	0
0	0	0	0	0	0	0	0	0	0	0	0	0	0
0	0	0	0	0	0	0	0	0	0	0	0	0	0
0													
OTU7609	0	0	0	0	0	0	0	0	0	1	0	0	0
0	0	0	0	0	0	0	0	0	0	0	0	0	0
0	0	0	0	0	0	0	0	0	0	0	0	0	0
0	0	0	0	0	0	0	0	0	0	0	0	0	0
0	0	0	0	0	0	0	0	0	0	0	0	0	0
0	0	0	0	0	0	0	0	0	0	0	0	0	0
0	0	0	0	0	0	0	0	0	0	0	0	0	0
0	0	0	0	1	0	0	0	0	0	0	0	0	0
0													
OTU2296	0	0	0	0	0	0	0	0	0	2	0	0	0
0	0	0	0	0	0	0	0	0	0	0	0	0	0
0	0	0	0	0	0	0	0	0	0	0	0	0	0
0	0	0	0	0	0	0	0	0	0	0	1	0	0
0	0	0	0	0	0	0	0	0	0	0	0	4	0
0	1	0	0	0	0	0	0	0	0	0	0	0	0

0	0	0	0	0	0	0	0	0	0	0	0	1	0
0	4	1	0	0	0	1	0	0	0	0	0	0	0
0													
OTU15660	0	0	0	0	0	0	0	0	0	1	0	0	0
0	0	0	0	0	0	0	0	0	0	0	0	0	0
0	0	0	0	0	0	0	0	0	0	0	0	0	0
0	0	0	0	0	0	0	0	0	0	0	0	0	0
0	0	0	0	0	0	0	0	0	0	0	0	0	0
0	0	0	0	0	0	0	0	0	0	0	0	0	0
0	0	0	0	0	0	0	0	0	0	0	0	0	0
0	0	0	0	0	0	0	0	0	0	0	0	0	0
0													
OTU4242	0	0	0	0	0	0	0	0	0	1	0	0	0
0	0	0	0	0	0	0	0	0	0	0	0	0	0
0	0	0	0	0	0	0	0	0	0	0	0	0	0
0	0	0	0	0	0	0	0	0	0	0	0	0	0
0	0	0	0	0	0	0	0	0	0	0	0	0	0
0	0	0	0	0	0	0	0	0	0	0	0	0	0
0	0	0	0	0	0	0	0	0	0	0	0	0	0
0	0	0	0	0	0	0	0	0	0	0	0	0	0
0													
OTU12426	0	0	0	0	0	0	0	0	0	1	0	0	0
0	0	0	0	0	0	0	0	0	0	0	0	0	0
0	0	0	0	0	0	0	0	0	0	0	0	0	0
0	0	0	0	0	0	0	0	0	0	0	0	0	0
0	0	0	0	0	0	0	0	0	0	0	0	0	0
0	0	0	0	0	0	0	0	0	0	0	0	0	0
0	0	0	0	0	0	0	0	0	0	0	0	0	0
0	0	0	0	0	0	0	0	0	0	0	0	0	0
0													
OTU15657	0	0	0	0	0	0	0	0	0	1	0	0	0
0	0	0	0	0	0	0	0	0	0	0	0	0	0
0	0	0	0	0	0	0	0	0	0	0	0	0	0

0	0	0	0	0	0	0	0	0	0	0	0	0	0
0	0	0	0	0	0	0	0	0	0	0	0	0	0
0	0	0	0	0	0	0	0	0	0	0	0	0	0
0	0	0	0	0	0	0	0	0	0	0	0	0	0
0	0	0	0	0	0	0	0	0	0	0	0	0	0
0													
OTU14824	0	0	0	0	0	0	0	0	0	1	0	0	0
0	0	0	0	0	0	0	0	0	0	0	0	0	0
0	0	0	0	0	0	0	0	0	0	0	0	0	0
0	1	0	0	0	0	0	0	0	0	0	0	0	0
0	0	0	0	0	0	0	0	0	0	0	0	0	0
0	0	0	0	0	0	0	0	0	0	0	0	0	0
0	0	0	0	0	0	0	0	0	0	0	1	0	0
0	0	0	0	0	0	0	0	0	0	0	0	0	0
0													
OTU15656	0	0	0	0	0	0	0	0	0	1	0	0	0
0	0	0	0	0	0	0	0	0	0	0	0	0	0
0	0	0	0	0	0	0	0	0	0	0	0	0	0
0	0	0	0	0	0	0	0	0	0	0	0	0	0
0	0	0	0	0	0	0	0	0	0	0	0	0	0
0	0	0	0	0	0	0	0	0	0	0	0	0	0
0	0	0	0	0	0	0	0	0	0	0	0	0	0
0	0	0	0	0	0	0	0	0	0	0	0	0	0
0													
OTU7610	0	0	0	0	0	0	0	0	0	1	0	0	0
0	0	0	0	0	0	0	0	0	0	0	0	0	0
0	0	0	0	0	0	0	0	0	0	0	0	0	0
0	0	0	0	0	0	0	0	0	0	0	0	0	0
0	0	0	0	0	0	0	0	0	0	0	0	0	0
0	0	0	0	0	0	0	0	0	0	0	0	0	0
0	0	0	0	0	0	0	0	0	0	0	0	0	0
0	0	0	0	0	0	0	0	0	0	0	0	0	0
0													

OTU4239 0 0 0 0 0 0 0 0 0 1 0 0 0
0 0 0 0 0 0 0 0 0 0 0 0 0 0
0 0 0 0 0 0 0 0 0 0 0 0 0 0
0 0 0 0 0 0 0 0 0 0 0 0 0 0
0 0 0 0 0 0 0 0 0 0 0 0 0 0
0 0 0 0 0 0 0 0 0 0 0 0 0 0
0 0 0 0 0 0 0 0 0 0 0 0 0 0
0 0 0 0 0 0 0 0 0 0 0 0 0 0
0
OTU15658 0 0 0 0 0 0 0 0 0 1 0 0 0
0 0 0 0 0 0 0 0 0 0 0 0 0 0
0 0 0 0 0 0 0 0 0 0 0 0 0 0
0 0 0 0 0 0 0 0 0 0 0 0 0 0
0 0 0 0 0 0 0 0 0 0 0 0 0 0
0 0 0 0 0 0 0 0 0 0 0 0 0 0
0 0 0 0 0 0 0 0 0 0 0 0 0 0
0 0 0 0 0 0 0 0 0 0 0 0 0 0
0
OTU4241 0 0 0 0 0 0 0 0 0 1 0 0 0
0 0 0 0 0 0 0 0 0 0 0 0 0 0
0 0 0 0 0 0 0 0 0 0 0 0 0 0
0 0 0 0 0 0 0 0 0 0 0 0 0 0
0 0 0 0 0 0 0 0 0 0 0 0 0 0
0 0 0 0 0 0 0 0 0 0 0 0 0 0
0 0 0 0 0 0 0 0 0 0 0 0 0 0
0 0 0 0 0 0 0 0 0 0 0 0 0 0
0
OTU4240 0 0 0 0 0 0 0 0 0 1 0 0 0
0 0 0 0 0 0 0 0 0 0 0 0 0 0
0 0 0 0 0 0 0 0 0 0 0 0 0 0
0 0 0 0 0 0 0 0 0 0 0 0 0 0
0 0 0 0 0 0 0 0 0 0 0 0 0 0
0 0 0 0 0 0 0 0 0 0 0 0 0 0

0	0	0	0	0	0	0	0	0	0	0	0	0	0
0	0	0	0	0	0	0	0	0	0	0	0	0	0
0													
OTU11176	0	0	0	0	0	0	0	0	0	1	0	0	0
0	0	0	0	0	0	0	0	0	0	0	0	2	0
0	0	0	0	0	0	0	0	0	0	1	0	1	0
0	0	0	0	0	0	0	0	0	0	0	0	0	0
0	0	0	0	0	0	0	0	0	0	0	0	0	0
0	0	0	0	0	0	0	0	0	0	0	0	0	0
0	0	0	0	0	0	0	1	0	0	0	0	0	0
0	0	0	0	0	0	0	0	1	1	0	0	0	0
0													
OTU7644	0	0	0	0	0	0	0	0	0	1	0	0	0
0	0	0	0	0	0	1	0	0	0	0	0	0	0
0	0	0	0	0	0	0	0	0	0	0	0	0	0
0	0	0	0	0	0	0	0	0	0	0	0	0	0
0	0	0	0	0	0	0	0	0	0	0	0	0	0
0	0	0	0	0	0	0	0	0	0	0	0	0	0
0	0	0	0	0	0	0	0	0	0	0	0	0	0
0	0	0	1	0	0	0	0	0	1	0	0	0	0
0													
OTU12515	0	0	0	0	0	0	0	0	0	1	0	0	0
0	0	0	0	0	0	0	0	0	0	0	0	0	0
0	0	0	0	0	0	0	0	0	0	0	0	0	0
0	0	0	0	0	0	0	0	0	0	0	0	0	0
0	0	0	0	0	0	0	0	0	0	0	0	0	0
0	0	0	0	0	0	0	0	0	0	0	0	0	0
0	0	0	0	0	0	0	0	0	0	0	0	0	0
0	0	0	0	0	0	0	0	0	0	0	0	0	0
0													
OTU4272	0	0	0	0	0	0	0	0	0	1	0	0	0
0	0	0	0	0	0	0	0	0	0	0	0	0	1
0	0	0	0	0	0	0	0	0	0	0	0	0	0

0	0	0	0	0	0	0	0	0	0	0	0	0	0
0	0	0	0	0	0	0	0	0	0	0	0	0	0
0	0	0	0	0	0	0	0	0	0	0	0	0	0
0	0	0	0	0	0	0	0	0	0	0	0	0	0
0	0	0	0	0	0	0	0	0	0	0	0	0	0
0													
OTU5039	0	0	0	0	0	0	0	0	0	1	0	1	0
0	0	0	0	0	0	0	0	0	0	0	0	0	0
0	0	0	0	0	0	0	1	0	0	0	0	0	0
0	0	0	0	0	0	0	0	0	0	0	0	1	0
0	2	0	0	0	0	0	0	0	0	0	0	0	0
0	0	0	0	0	0	0	0	0	0	0	0	0	0
0	0	0	0	0	0	0	0	0	0	0	0	0	0
0	0	0	0	0	0	0	0	0	0	0	0	0	0
0													
OTU15648	0	0	0	0	0	0	0	0	0	1	0	0	0
0	0	0	0	0	0	0	0	0	0	0	0	0	0
0	0	0	0	0	0	0	0	0	0	0	0	0	0
0	0	0	0	0	0	0	0	0	0	0	0	0	0
0	0	0	0	0	0	0	0	0	0	0	0	0	0
0	0	0	0	0	0	0	0	0	0	0	0	0	0
0	0	0	0	0	0	0	0	0	0	0	0	0	0
0	0	0	0	0	0	0	0	0	0	0	0	0	0
0													
OTU1326	0	0	0	0	0	0	0	0	0	1	0	0	0
0	0	0	0	0	0	0	0	0	0	0	0	0	0
0	0	0	0	0	0	0	0	0	0	0	0	0	0
0	0	0	0	0	0	0	0	0	0	0	0	0	0
0	0	0	0	0	0	0	0	0	0	0	0	0	0
0	0	0	0	3	0	0	0	0	0	0	0	0	0
0	0	0	0	0	0	0	0	0	0	0	0	0	0
0	0	0	0	0	0	0	0	0	0	0	0	0	0
0													

OTU12514	0	0	0	0	0	0	0	0	0	1	0	0	0
0	0	0	0	0	0	0	0	0	0	0	0	0	0
0	0	0	0	0	0	0	0	0	0	0	0	0	0
0	0	0	0	0	0	0	0	0	0	0	0	0	0
0	0	0	0	0	0	0	0	0	0	0	0	0	0
0	0	0	0	0	0	0	0	0	0	0	0	0	0
0	0	0	0	0	0	0	0	0	0	0	0	0	0
0	0	0	0	0	0	0	0	0	0	0	0	0	0
0													
OTU603	0	0	0	0	0	0	0	0	0	1	0	0	0
0	0	3	0	0	0	0	1	0	0	0	0	0	0
0	0	0	1	0	0	0	0	1	0	0	0	0	0
0	8	0	0	0	1	0	0	0	0	0	0	1	0
0	0	1	3	0	0	0	0	0	0	0	0	0	0
0	0	0	1	0	0	0	0	0	0	0	0	0	0
0	0	0	1	0	0	1	0	0	1	0	0	0	2
0	0	0	0	0	0	0	5	0	0	0	0	1	0
0													
OTU4271	0	0	0	0	0	0	0	0	0	1	0	0	0
0	0	0	0	0	0	0	0	0	0	0	0	0	0
0	0	0	0	0	0	0	0	0	0	0	0	0	0
0	0	0	0	0	0	0	0	0	0	0	0	0	0
0	0	0	0	0	0	0	0	0	0	0	0	0	0
0	0	0	0	0	0	0	0	0	0	0	0	0	0
0	0	0	0	0	0	0	0	0	0	0	0	0	0
0	0	0	0	0	0	0	0	0	0	0	0	0	0
0													
OTU12513	0	0	0	0	0	0	0	0	0	1	0	0	0
0	0	0	0	0	0	0	0	0	0	0	0	0	0
0	0	0	0	0	0	0	0	0	0	0	0	0	0
0	0	0	0	0	0	0	0	0	0	0	0	0	0
0	0	0	0	0	0	0	0	0	0	0	0	0	0
0	0	0	0	0	0	0	0	0	0	0	0	0	0

0	0	0	0	0	0	0	0	0	0	0	0	0	0
0	0	0	0	0	0	0	0	0	0	0	0	0	0
0													
OTU15649	0	0	0	0	0	0	0	0	0	1	0	0	0
0	0	0	0	0	0	0	0	0	0	0	0	0	0
0	0	0	0	0	0	0	0	0	0	0	0	0	0
0	0	0	0	0	0	0	0	0	0	0	0	0	0
0	0	0	0	0	0	0	0	0	0	0	0	0	0
0	0	0	0	0	0	0	0	0	0	0	0	0	0
0	0	0	0	0	0	0	0	0	0	0	0	0	0
0	0	0	0	0	0	0	0	0	0	0	0	0	0
0													
OTU7642	0	0	0	0	0	0	0	0	0	1	0	0	0
0	0	0	0	0	0	0	0	0	0	0	0	0	0
0	0	0	0	0	0	0	0	0	0	0	0	0	0
0	0	0	0	0	0	0	0	0	0	0	0	0	0
0	0	0	0	0	0	0	0	0	0	0	0	0	0
0	0	0	0	0	0	0	0	0	0	0	0	0	0
0	0	0	0	0	0	0	0	0	0	0	0	0	0
0	0	0	0	0	0	0	0	0	0	0	0	0	0
0													
OTU13762	0	0	0	0	0	0	0	0	0	1	0	0	0
0	0	0	0	0	0	0	0	0	0	0	0	0	0
0	0	0	0	0	0	0	0	0	0	0	0	0	0
0	0	0	0	0	0	0	0	0	0	0	0	0	0
0	0	0	0	0	0	0	0	0	0	0	0	0	0
0	0	0	0	0	0	0	0	0	0	0	0	0	0
0	0	0	1	0	0	0	0	0	0	0	0	0	0
0	0	0	0	0	0	0	0	0	0	0	0	0	0
0													
OTU4269	0	0	0	0	0	0	0	0	0	1	0	0	0
0	0	0	0	0	0	0	0	0	0	0	0	0	0
0	0	0	0	0	0	0	0	0	0	0	0	0	0

0	0	0	0	0	0	0	0	0	0	0	0	0	0
0	0	0	0	0	0	0	0	0	0	0	0	0	0
0	0	0	0	0	0	0	0	0	0	0	0	0	0
0	0	0	0	0	0	0	0	0	0	0	0	0	0
0	0	0	0	0	0	0	0	0	0	0	0	0	0
0													
OTU1149	0	0	0	0	0	0	0	0	0	1	0	0	0
0	1	1	0	0	0	1	0	0	0	0	0	0	0
0	0	0	0	0	0	0	0	0	0	1	0	0	0
0	0	0	0	0	0	0	0	0	0	0	0	0	0
0	0	0	1	0	0	0	0	0	0	0	0	0	0
0	0	1	0	0	0	0	0	0	1	0	0	0	0
0	0	0	0	0	0	0	0	0	0	0	0	0	0
0	0	0	0	0	0	0	0	0	0	0	0	0	0
0													
OTU8717	0	0	0	0	0	0	0	0	0	1	0	0	0
0	0	0	0	0	0	0	0	0	0	0	0	0	0
0	0	0	0	0	0	0	0	0	0	1	0	0	0
0	0	0	0	0	0	0	0	0	0	0	0	0	0
0	0	0	0	0	0	0	0	0	0	0	0	0	0
0	0	0	0	0	0	0	0	0	0	0	0	0	0
0	0	0	0	0	0	0	0	0	0	0	0	0	0
0	0	0	0	0	0	0	0	0	0	0	0	0	0
0													
OTU15644	0	0	0	0	0	0	0	0	0	1	0	0	0
0	0	0	0	0	0	0	0	0	0	0	0	0	0
0	0	0	0	0	0	0	0	0	0	0	0	0	0
0	0	0	0	0	0	0	0	0	0	0	0	0	0
0	0	0	0	0	0	0	0	0	0	0	0	0	0
0	0	0	0	0	0	0	0	0	0	0	0	0	0
0	0	0	0	0	0	0	0	0	0	0	0	0	0
0	0	0	0	0	0	0	0	0	0	0	0	0	0
0													

OTU15645	0	0	0	0	0	0	0	0	0	1	0	0	0
0	0	0	0	0	0	0	0	0	0	0	0	0	0
0	0	0	0	0	0	0	0	0	0	0	0	0	0
0	0	0	0	0	0	0	0	0	0	0	0	0	0
0	0	0	0	0	0	0	0	0	0	0	0	0	0
0	0	0	0	0	0	0	0	0	0	0	0	0	0
0	0	0	0	0	0	0	0	0	0	0	0	0	0
0	0	0	0	0	0	0	0	0	0	0	0	0	0
0													
OTU12521	0	0	0	0	0	0	0	0	0	1	0	0	0
0	0	0	0	0	0	0	0	0	0	0	0	0	0
0	0	0	0	0	0	0	0	0	0	0	0	0	0
0	0	0	0	0	0	0	0	0	0	0	0	0	0
0	0	0	0	0	0	0	0	0	0	0	0	0	0
0	0	0	0	0	0	0	0	0	0	0	0	0	0
0	0	0	0	0	0	0	0	0	0	0	0	0	0
0	0	0	0	0	0	0	0	0	0	0	0	0	0
0													
OTU12519	0	0	0	0	0	0	0	0	0	2	0	0	0
0	0	0	0	0	0	0	0	0	0	0	0	0	0
0	0	0	0	0	0	0	0	0	0	0	0	0	0
0	0	0	0	0	0	0	0	0	0	0	0	0	0
0	0	0	0	0	0	0	0	0	0	0	0	0	0
0	0	0	0	0	0	0	0	0	0	0	0	0	0
0	0	0	0	0	0	0	0	0	0	0	0	0	0
0	0	0	0	0	0	0	0	0	0	0	0	0	0
0													
OTU9309	0	0	0	0	0	0	0	0	0	1	0	0	0
0	0	0	0	0	0	0	1	0	0	0	0	0	0
0	0	0	0	0	0	0	0	0	0	0	0	0	0
0	0	0	0	0	0	0	0	0	0	0	0	0	0
0	0	0	0	0	0	0	0	0	0	0	0	0	0
0	0	0	0	0	0	0	0	0	0	0	0	0	0

0	0	0	0	0	0	0	0	0	0	0	0	0	0
0	0	0	0	0	0	0	0	0	0	0	0	0	0
0													
OTU9645	0	0	0	0	0	0	0	0	0	1	0	0	0
0	0	0	0	0	0	0	0	0	0	0	0	0	0
0	0	0	0	0	0	0	0	0	0	0	0	0	0
0	0	0	0	0	0	0	0	0	0	0	0	0	0
0	0	0	0	0	0	0	0	0	0	0	0	0	0
0	0	0	0	0	0	0	0	0	1	0	0	0	0
0	0	0	0	0	0	0	0	0	0	0	0	0	0
0	0	0	0	0	0	0	0	0	0	0	0	0	0
0													
OTU4276	0	0	0	0	0	0	0	0	0	2	0	0	0
0	0	0	0	0	0	0	0	0	0	0	0	0	0
0	0	0	0	0	0	0	0	0	0	0	0	0	0
0	0	0	0	0	0	0	0	0	0	0	0	0	0
0	0	0	0	0	0	0	0	0	0	0	0	0	0
0	0	0	0	0	0	0	0	0	0	0	0	0	0
0	0	0	0	0	0	0	0	0	0	0	0	0	0
0	0	0	0	0	0	0	0	0	0	0	0	0	0
0													
OTU15647	0	0	0	0	0	0	0	0	0	1	0	0	0
0	0	0	0	0	0	0	0	0	0	0	0	0	0
0	0	0	0	0	0	0	0	0	0	0	0	0	0
0	0	0	0	0	0	0	0	0	0	0	0	0	0
0	0	0	0	0	0	0	0	0	0	0	0	0	0
0	0	0	0	0	0	0	0	0	0	0	0	0	0
0	0	0	0	0	0	0	0	0	0	0	0	0	0
0	0	0	0	0	0	0	0	0	0	0	0	0	0
0													
OTU7647	0	0	0	0	0	0	0	0	0	1	0	0	0
0	0	0	0	0	0	0	0	0	0	0	0	0	0
0	0	0	0	0	0	0	0	0	0	0	0	0	0

0	0	0	0	0	0	0	0	0	0	0	0	0	0
0	0	0	0	0	0	0	0	0	0	0	0	0	0
0	0	0	0	0	0	0	0	0	0	0	0	0	0
0	0	0	0	0	0	0	0	0	0	0	0	0	0
0	0	0	0	0	0	0	0	0	0	0	0	0	0
0													
OTU15653	0	0	0	0	0	0	0	0	0	1	0	0	0
0	0	0	0	0	0	0	0	0	0	0	0	0	0
0	0	0	0	0	0	0	0	0	0	0	0	0	0
0	0	0	0	0	0	0	0	0	0	0	0	0	0
0	0	0	0	0	0	0	0	0	0	0	0	0	0
0	0	0	0	0	0	0	0	0	0	0	0	0	0
0	0	0	0	0	0	0	0	0	0	0	0	0	0
0	0	0	0	0	0	0	0	0	0	0	0	0	0
0													
OTU866	0	0	0	0	0	0	0	0	0	4	0	0	0
0	0	0	0	0	0	0	0	0	0	0	0	0	0
0	0	0	0	0	0	0	0	0	0	0	0	0	0
0	0	0	0	0	0	0	0	0	0	0	0	0	0
0	0	0	0	0	0	0	0	0	0	0	0	0	0
0	0	0	0	0	0	0	0	0	0	0	0	0	0
0	0	0	0	0	0	0	0	0	0	0	0	0	0
0	0	0	0	0	0	0	0	0	6	0	0	0	0
0													
OTU7645	0	0	0	0	0	0	0	0	0	2	0	0	0
0	0	0	0	0	0	0	0	0	0	0	0	0	0
0	0	0	0	0	0	0	0	0	0	0	0	0	0
0	0	0	0	0	0	0	0	0	0	0	0	0	0
0	0	0	0	0	0	0	0	0	0	0	0	0	0
0	0	0	0	0	0	0	0	0	0	0	0	0	0
0	0	0	0	0	0	0	0	0	0	0	0	0	0
0	0	0	0	0	0	0	0	0	0	0	0	0	0
0													

OTU12516	0	0	0	0	0	0	0	0	0	1	0	0	0
0	0	0	0	0	0	0	0	0	0	0	0	0	0
0	0	0	0	0	0	0	0	0	0	0	0	0	0
0	0	0	0	0	0	0	0	0	0	0	0	0	0
0	0	0	0	0	0	0	0	0	0	0	0	0	0
0	0	0	0	0	0	0	0	0	0	0	0	0	0
0	0	0	0	0	0	0	0	0	0	0	0	0	0
0	0	0	0	0	0	0	0	0	0	0	0	0	0
0													
OTU4274	0	0	0	0	0	0	0	0	0	1	0	0	0
0	0	0	0	0	0	0	0	0	0	0	0	0	0
0	0	0	0	0	0	0	0	0	0	0	0	0	0
0	0	0	0	0	0	0	0	0	0	0	0	0	0
0	0	0	0	0	0	0	0	0	0	0	0	0	0
0	0	0	0	0	0	0	0	0	0	0	0	0	0
0	0	0	0	0	0	0	0	0	0	0	0	0	0
0	0	0	0	0	0	0	0	0	0	0	0	0	0
0													
OTU1319	0	0	0	0	0	0	0	0	0	1	0	0	0
0	0	0	0	0	0	0	0	0	0	0	0	0	0
0	0	0	0	0	0	0	0	0	0	0	0	0	0
0	0	0	0	0	0	0	0	0	0	0	0	0	0
0	0	0	0	0	0	0	0	0	0	0	0	0	0
0	0	0	0	1	0	0	0	0	0	0	0	0	0
0	0	0	0	0	0	0	0	0	0	0	0	0	0
0	0	0	0	0	0	0	0	0	0	0	0	0	0
0													
OTU12517	0	0	0	0	0	0	0	0	0	1	0	0	0
0	0	0	0	0	0	0	0	0	0	0	0	0	0
0	0	0	0	0	0	0	0	0	0	0	0	0	0
0	0	0	0	0	0	0	0	0	0	0	0	0	0
0	0	0	0	0	0	0	0	0	0	0	0	0	0
0	0	0	0	0	0	0	0	0	0	0	0	0	0

0 0 0 0 0 0 0 0 0 0 0 0 0 0
0 0 0 0 0 0 0 0 0 0 0 0 0 0
0
OTU6090 0 0 0 0 0 0 0 0 0 1 0 0 0
0 0 0 0 0 0 0 0 0 0 0 0 0 0
0 0 0 0 0 0 0 0 0 0 0 0 0 0
0 0 0 0 0 0 0 0 0 0 0 0 0 0
0 0 0 0 0 0 0 0 0 0 0 0 1 0
0 0 0 0 0 1 0 0 0 0 0 0 0 0
0 0 0 0 0 0 0 0 0 0 0 0 0 0
0 0 0 0 0 0 0 0 0 0 0 0 0 0
0
OTU4260 0 0 0 0 0 0 0 0 0 1 0 0 0
0 0 0 0 0 0 0 0 0 0 0 0 0 0
0 0 0 0 0 0 0 0 0 0 0 0 0 0
0 0 0 0 0 0 0 0 0 0 0 0 0 0
0 0 0 0 0 0 0 0 0 0 0 0 0 0
0 0 0 0 0 0 0 0 0 0 0 0 0 0
0 0 0 0 0 0 0 0 0 0 0 0 0 0
0 0 0 0 0 0 0 0 0 0 0 0 0 0
0
OTU11622 0 0 0 0 0 0 0 0 0 2 0 0 0
0 0 1 0 0 0 0 0 0 0 0 0 0 0
0 0 0 0 0 0 0 0 0 0 0 0 0 0
0 0 0 0 0 0 0 0 0 0 0 0 0 0
0 0 0 0 0 0 0 0 0 0 0 0 0 0
0 0 0 0 0 0 0 0 1 0 0 0 0 0
0 0 0 0 0 0 0 0 0 0 0 0 0 0
0 0 0 0 0 0 0 0 0 0 0 0 0 0
0
OTU15650 0 0 0 0 0 0 0 0 0 1 0 0 0
0 0 0 0 0 0 0 0 0 0 0 0 0 0
0 0 0 0 0 0 0 0 0 0 0 0 0 0

0	0	0	0	0	0	0	0	0	0	0	0	0	0
0	0	0	0	0	0	0	0	0	0	0	0	0	0
0	0	0	0	0	0	0	0	0	0	0	0	0	0
0	0	0	0	0	0	0	0	0	0	0	0	0	0
0	0	0	0	0	0	0	0	0	0	0	0	0	0
0													
OTU12505	0	0	0	0	0	0	0	0	0	1	0	0	0
0	0	0	0	0	0	0	0	0	0	0	0	0	0
0	0	0	0	0	0	0	0	0	0	0	0	0	0
0	0	0	0	0	0	0	0	0	0	0	0	0	0
0	0	0	0	0	0	0	0	0	0	0	0	0	0
0	0	0	0	0	0	0	0	0	0	0	0	0	0
0	0	0	0	0	0	0	0	0	0	0	0	0	0
0	0	0	0	0	0	0	0	0	0	0	0	0	0
0													
OTU15652	0	0	0	0	0	0	0	0	0	1	0	0	0
0	0	0	0	0	0	0	0	0	0	0	0	0	0
0	0	0	0	0	0	0	0	0	0	0	0	0	0
0	0	0	0	0	0	0	0	0	0	0	0	0	0
0	0	0	0	0	0	0	0	0	0	0	0	0	0
0	0	0	0	0	0	0	0	0	0	0	0	0	0
0	0	0	0	0	0	0	0	0	0	0	0	0	0
0	0	0	0	0	0	0	0	0	0	0	0	0	0
0													
OTU15651	0	0	0	0	0	0	0	0	0	1	0	0	0
0	0	0	0	0	0	0	0	0	0	0	0	0	0
0	0	0	0	0	0	0	0	0	0	0	0	0	0
0	0	0	0	0	0	0	0	0	0	0	0	0	0
0	0	0	0	0	0	0	0	0	0	0	0	0	0
0	0	0	0	0	0	0	0	0	0	0	0	0	0
0	0	0	0	0	0	0	0	0	0	0	0	0	0
0	0	0	0	0	0	0	0	0	0	0	0	0	0
0													

OTU4262	0	0	0	0	0	0	0	0	0	1	0	0	0
0	0	0	0	0	0	0	0	0	0	0	0	0	0
0	0	0	0	0	0	0	0	0	0	0	0	0	0
0	0	0	0	0	0	0	0	0	0	0	0	0	0
0	0	0	0	0	0	0	0	0	0	0	0	0	0
0	0	0	0	0	0	0	0	0	0	0	0	0	0
0	0	0	0	0	0	0	0	0	0	0	0	0	0
0	0	0	0	0	0	0	0	0	0	0	0	0	0
0													
OTU12506	0	0	0	0	0	0	0	0	0	1	0	0	0
0	0	0	0	0	0	0	0	0	0	0	0	0	0
0	0	0	0	0	0	0	0	0	0	0	0	0	0
0	0	0	0	0	0	0	0	0	0	0	0	0	0
0	0	0	0	0	0	0	0	0	0	0	0	0	0
0	0	0	0	0	0	0	0	0	0	0	0	0	0
0	0	0	0	0	0	0	0	0	0	0	0	0	0
0	0	0	0	0	0	0	0	0	0	0	0	0	0
0													
OTU7633	0	0	0	0	0	0	0	0	0	1	0	0	0
0	0	0	0	0	0	0	0	0	0	0	0	0	0
0	0	0	0	0	0	0	0	0	0	0	0	0	0
0	0	0	0	0	0	0	0	0	0	0	0	0	0
0	0	0	0	0	0	0	0	0	0	0	0	0	0
0	0	0	0	0	0	0	0	0	0	0	0	0	0
0	0	0	0	0	0	0	0	0	0	0	0	0	0
0	0	0	0	0	0	0	0	0	0	0	0	0	0
0													
OTU7632	0	0	0	0	0	0	0	0	0	1	0	0	0
0	0	0	0	0	0	0	0	0	0	0	0	0	0
0	0	0	0	0	0	0	0	0	0	0	0	0	0
0	0	0	0	0	0	0	0	0	0	0	0	0	0
0	0	0	0	0	0	0	0	0	0	0	0	0	0
0	0	0	0	0	0	0	0	0	0	0	0	0	0

0	0	0	0	0	0	0	0	0	0	0	0	0	0
0	0	0	0	0	0	0	0	0	0	0	0	0	0
0													
OTU15604	0	0	0	0	0	0	0	0	0	1	0	0	0
0	0	0	0	0	0	0	0	0	0	0	0	0	0
0	0	0	0	0	0	0	0	0	0	0	0	0	0
0	0	0	0	0	0	0	0	0	0	0	0	0	0
0	0	0	0	0	0	0	0	0	0	0	0	0	0
0	0	0	0	0	0	0	0	0	0	0	0	0	0
0	0	0	0	0	0	0	0	0	0	0	0	0	0
0	0	0	0	0	0	0	0	0	0	0	0	0	0
0													
OTU15482	0	0	0	0	0	0	0	0	0	1	0	0	0
0	0	0	0	0	0	0	0	0	0	0	0	0	0
0	0	1	0	0	0	0	0	0	0	1	0	1	0
0	0	0	0	0	0	0	0	2	2	0	0	0	0
0	1	0	0	0	0	0	0	0	0	0	0	0	0
0	1	0	0	0	0	0	0	0	0	0	0	0	0
0	0	0	1	0	0	0	0	0	0	0	0	0	0
0	0	0	1	1	0	0	0	0	0	1	0	0	0
0													
OTU4256	0	0	0	0	0	0	0	0	0	1	0	0	0
0	0	0	0	0	0	0	0	0	0	0	0	0	0
0	0	0	0	0	0	0	0	0	0	0	0	0	0
0	0	0	0	0	0	0	0	0	0	0	0	0	0
0	0	0	0	0	0	0	0	0	0	0	0	0	0
0	0	0	0	0	0	0	0	0	0	0	0	0	0
0	0	0	0	0	0	0	0	0	0	0	0	0	0
0	0	0	0	0	0	0	0	0	0	0	0	0	0
0													
OTU4209	0	0	0	0	0	0	0	0	0	1	0	0	0
1	0	0	0	0	1	0	0	0	0	0	0	0	0
0	1	0	0	0	0	0	0	0	0	0	0	1	0

0	0	0	0	0	0	0	0	0	0	0	0	0	0
0	0	0	1	0	0	0	0	0	0	0	0	0	0
0	0	0	0	0	1	0	0	0	0	0	1	0	0
0	0	0	0	0	0	0	0	0	0	1	0	0	1
0	0	0	0	0	1	0	0	0	0	0	0	0	0
2													
OTU12502	0	0	0	0	0	0	0	0	0	2	0	0	0
0	0	0	0	0	0	0	0	0	0	0	0	0	0
0	0	0	0	0	0	0	0	0	0	0	0	0	0
0	0	0	0	0	0	0	0	0	0	0	0	0	0
0	0	0	0	0	0	0	0	0	0	0	0	0	0
0	0	0	0	0	0	0	0	0	0	0	0	0	0
0	0	0	0	0	0	0	0	0	0	0	0	0	0
0	0	0	0	0	0	0	0	0	0	0	0	0	0
0													
OTU7629	0	0	0	0	0	0	0	0	0	1	0	0	0
0	0	0	0	0	0	0	0	0	0	0	0	0	0
0	0	0	0	0	0	0	0	0	0	0	0	0	0
0	0	0	0	0	0	0	0	0	0	0	0	0	0
0	0	0	0	0	0	0	0	0	0	0	0	0	0
0	0	0	0	0	0	0	0	0	0	0	0	0	0
0	0	0	0	0	0	0	0	0	0	0	0	0	0
0	0	0	0	0	0	0	0	0	0	0	0	0	0
0													
OTU12503	0	0	0	0	0	0	0	0	0	1	0	0	0
0	0	0	0	0	0	0	0	0	0	0	0	0	0
0	0	0	0	0	0	0	0	0	0	0	0	0	0
0	0	0	0	0	0	0	0	0	0	0	0	0	0
0	0	0	0	0	0	0	0	0	0	0	0	0	0
0	0	0	0	0	0	0	0	0	0	0	0	0	0
0	0	0	0	0	0	0	0	0	0	0	0	0	0
0	0	0	0	0	0	0	0	0	0	0	0	0	0
0													

OTU7630	0	0	0	0	0	0	0	0	0	1	0	0	0
0	0	0	0	0	0	0	0	0	0	0	0	0	0
0	0	0	0	0	0	0	0	0	0	0	0	0	0
0	0	0	0	0	0	0	0	0	0	0	0	0	0
0	0	0	0	0	0	0	0	0	0	0	0	0	0
0	0	0	0	0	0	0	0	0	0	0	0	0	0
0	0	0	0	0	0	0	0	0	0	0	0	0	0
0	0	0	0	0	0	0	0	0	0	0	0	0	0
0													
OTU15605	0	0	0	0	0	0	0	0	0	1	0	0	0
0	0	0	0	0	0	0	0	0	0	0	0	0	0
0	0	0	0	0	0	0	0	0	0	0	0	0	0
0	0	0	0	0	0	0	0	0	0	0	0	0	0
0	0	0	0	0	0	0	0	0	0	0	0	0	0
0	0	0	0	0	0	0	0	0	0	0	0	0	0
0	0	0	0	0	0	0	0	0	0	0	0	0	0
0	0	0	0	0	0	0	0	0	0	0	0	0	0
0													
OTU7631	0	0	0	0	0	0	0	0	0	1	0	0	0
0	0	0	0	0	0	0	0	0	0	0	0	0	0
0	0	0	0	0	0	0	0	0	0	0	0	0	0
0	0	0	0	0	0	0	0	0	0	0	0	0	0
0	0	0	0	0	0	0	0	0	0	0	0	0	0
0	0	0	0	0	0	0	0	0	0	0	0	0	0
0	0	0	0	0	0	0	0	0	0	0	0	0	0
0	0	0	0	0	0	0	0	0	0	0	0	0	0
0													
OTU4259	0	0	0	0	0	0	0	0	0	1	0	0	0
0	0	0	0	0	0	0	0	0	0	0	0	0	0
0	0	0	0	0	0	0	0	0	0	0	0	0	0
0	0	0	0	0	0	0	0	0	0	0	0	0	0
0	0	0	0	0	0	0	0	0	0	0	0	0	0
0	0	0	0	0	0	0	0	0	0	0	0	0	0

0	0	0	0	0	0	0	0	0	0	0	0	0	0
0	0	0	0	0	0	0	0	0	0	0	0	0	0
0													
OTU12504	0	0	0	0	0	0	0	0	0	1	0	0	0
0	0	0	0	0	0	0	0	0	0	0	0	0	0
0	0	0	0	0	0	0	0	0	0	0	0	0	0
0	0	0	0	0	0	0	0	0	0	0	0	0	0
0	0	0	0	0	0	0	0	0	0	0	0	0	0
0	0	0	0	0	0	0	0	0	0	0	0	0	0
0	0	0	0	0	0	0	0	0	0	0	0	0	0
0	0	0	0	0	0	0	0	0	0	0	0	0	0
0													
OTU4258	0	0	0	0	0	0	0	0	0	1	0	0	0
0	0	0	0	0	0	0	0	0	0	0	0	0	0
0	0	0	0	0	0	0	0	0	0	0	0	0	0
0	0	0	0	0	0	0	0	0	0	0	0	0	0
0	0	0	0	0	0	0	0	0	0	0	0	0	0
0	0	0	0	0	0	0	0	0	0	0	0	0	0
0	0	0	0	0	0	0	0	0	0	0	0	0	0
0	0	0	0	0	0	0	0	0	0	0	0	0	0
0													
OTU4257	0	0	0	0	0	0	0	0	0	1	0	0	0
0	0	0	0	0	0	0	0	0	0	0	0	0	0
0	0	0	0	0	0	0	0	0	0	0	0	0	0
0	0	0	0	0	0	0	0	0	0	0	0	0	0
0	0	0	0	0	0	0	0	0	0	0	0	0	0
0	0	0	0	0	0	0	0	0	0	0	0	0	0
0	0	0	0	0	0	0	0	0	0	0	0	0	0
0	0	0	0	0	0	0	0	0	0	0	0	0	0
0													
OTU12511	0	0	0	0	0	0	0	0	0	1	0	0	0
0	0	0	0	0	0	0	0	0	0	0	0	0	0
0	0	0	0	0	0	0	0	0	0	0	0	0	0

0	0	0	0	0	0	0	0	0	0	0	0	0	0
0	0	0	0	0	0	0	0	0	0	0	0	0	0
0	0	0	0	0	0	0	0	0	0	0	0	0	0
0	0	0	0	0	0	0	0	0	0	0	0	0	0
0	0	0	0	0	0	0	0	0	0	0	0	0	0
0													
OTU7638	0	0	0	0	0	0	0	0	0	1	0	0	0
0	0	0	0	0	0	0	0	0	0	0	0	0	0
0	0	0	0	0	0	0	0	0	0	0	0	0	0
0	0	0	0	0	0	0	0	0	0	0	0	0	0
0	0	0	0	0	0	0	0	0	0	0	0	0	0
0	0	0	0	0	0	0	0	0	0	0	0	0	0
0	0	0	0	0	0	0	0	0	0	0	0	0	0
0	0	0	0	0	0	0	0	0	0	0	0	0	0
0													
OTU333	0	0	0	0	0	0	0	0	0	0	69	0	24
2	0	0	0	0	0	0	4	0	3	0	0	0	0
0	0	68	0	0	0	0	1	0	0	0	0	0	0
0	0	0	0	0	0	0	0	0	0	0	0	0	0
0	0	0	0	0	0	0	4	0	0	0	0	0	0
0	0	0	0	0	0	0	0	0	0	0	0	0	0
0	0	0	0	0	0	0	0	10	0	4	0	0	5
0	0	0	0	0	0	0	0	0	0	0	0	0	0
0													
OTU7640	0	0	0	0	0	0	0	0	0	0	1	0	0
0	0	0	0	0	0	0	0	0	0	0	0	0	0
0	0	0	0	0	0	0	0	0	0	0	0	0	0
0	0	0	0	0	0	0	0	0	0	0	0	0	0
0	0	0	0	0	0	0	0	0	0	0	0	0	0
0	0	0	0	0	0	0	0	0	0	0	0	0	0
0	0	0	0	0	0	0	0	0	0	0	0	0	0
0	0	0	0	0	0	0	0	0	0	0	0	0	0
0													

OTU4265	0	0	0	0	0	0	0	0	0	0	1	0	0
0	0	0	0	0	0	0	0	0	0	0	0	0	0
0	0	0	0	0	0	0	0	0	0	0	0	0	0
0	0	0	0	0	0	0	0	0	0	0	0	0	0
0	0	0	0	0	0	0	0	0	0	0	0	0	0
0	0	0	0	0	0	0	0	0	0	0	0	0	0
0	0	0	0	0	0	0	0	0	0	0	0	0	0
0	0	0	0	0	0	0	0	0	0	0	0	0	0
0													
OTU15603	0	0	0	0	0	0	0	0	0	0	1	0	0
0	0	0	0	0	0	0	0	0	0	0	0	0	0
0	0	0	0	0	0	0	0	0	0	0	0	0	0
0	0	0	0	0	0	0	0	0	0	0	0	0	0
0	0	0	0	0	0	0	0	0	0	0	0	0	0
0	0	0	0	0	0	0	0	0	0	0	0	0	0
0	0	0	0	0	0	0	0	0	0	0	0	0	0
0	0	0	0	0	0	0	0	0	0	0	0	0	0
0													
OTU11950	0	0	0	0	0	0	0	0	0	0	1	0	0
0	0	1	0	0	0	0	0	0	0	0	0	0	1
0	0	1	0	0	0	0	0	0	0	0	0	0	0
0	0	0	0	0	0	0	0	0	0	0	0	0	0
0	0	0	0	0	0	0	0	0	0	0	0	0	0
0	0	0	0	0	0	0	0	0	0	0	0	0	0
0	1	0	0	0	0	0	0	0	0	0	0	0	0
0	0	0	0	0	0	0	0	1	0	0	0	0	0
1													
OTU8098	0	0	0	0	0	0	0	0	0	0	7	0	29
61	0	0	0	0	1	0	0	0	0	0	0	0	0
0	0	3	0	0	0	0	2	0	1	0	0	0	0
0	0	0	0	0	0	0	0	0	0	0	0	0	0
1	0	0	0	0	0	1	1	0	0	0	0	0	0
0	0	0	1	0	0	0	0	0	0	0	0	0	0

0	1	1	0	0	0	14	0	38	1	0	0	0	56
2	0	0	0	0	0	0	0	0	0	0	1	3	0
0													
OTU7639	0	0	0	0	0	0	0	0	0	0	1	0	0
0	0	0	0	0	0	0	0	0	0	0	0	0	0
0	0	0	0	0	0	0	0	0	0	0	0	0	0
0	0	0	0	0	0	0	0	0	0	0	0	0	0
0	0	0	0	0	0	0	0	0	0	0	0	0	0
0	0	0	0	0	0	0	0	0	0	0	0	0	0
0	0	0	0	0	0	0	0	0	0	0	0	0	0
0	0	0	0	0	0	0	0	0	0	0	0	0	0
0													
OTU10333	0	0	0	0	0	0	0	0	0	0	1	1	7
3	0	0	0	0	0	0	0	0	0	0	0	1	0
0	0	1	0	0	0	0	2	0	0	0	0	0	6
0	2	0	0	0	0	0	0	0	0	0	0	0	0
0	0	0	0	0	0	2	0	0	1	0	0	0	0
0	0	0	8	0	0	0	0	0	0	0	0	1	0
1	0	3	0	2	0	0	0	3	0	2	0	0	6
2	0	0	0	0	0	0	0	0	0	0	2	0	0
0													
OTU12499	0	0	0	0	0	0	0	0	0	0	1	2	1
0	0	0	0	0	0	0	0	0	0	0	0	0	0
0	0	0	0	0	0	0	0	0	0	0	0	0	1
0	0	0	0	0	0	0	0	0	0	0	0	0	0
0	0	0	0	0	0	0	0	0	0	0	0	0	0
0	0	0	0	0	0	1	0	0	0	0	0	0	0
0	0	1	0	1	0	0	0	0	0	0	0	0	0
0	0	0	0	0	0	0	0	0	0	1	1	0	0
0													
OTU5359	0	0	0	0	0	0	0	0	0	0	1	0	0
0	0	0	0	0	0	0	0	0	0	0	0	0	0
0	0	0	0	0	0	0	0	1	0	0	0	0	0

0	0	0	2	0	0	1	0	0	0	0	0	0	0
0	2	0	0	0	0	0	0	0	0	0	0	0	0
0	0	0	0	0	0	0	0	0	0	0	0	1	0
0	0	0	0	0	0	0	0	0	0	0	0	0	0
0	0	0	0	0	0	0	0	0	0	0	1	0	0
0													
OTU4267	0	0	0	0	0	0	0	0	0	0	1	0	0
0	0	0	0	0	0	0	0	0	0	0	0	0	0
0	0	0	0	0	0	0	0	0	0	0	0	0	0
0	0	0	0	0	0	0	0	0	0	0	0	0	0
0	0	0	0	0	0	0	0	0	0	0	0	0	0
0	0	0	0	0	0	0	0	0	0	0	0	0	0
0	0	0	0	0	0	0	0	0	0	0	0	0	0
0	0	0	0	0	0	0	0	0	0	0	0	0	0
0													
OTU359	0	0	0	0	0	0	0	0	0	0	35	1	40
0	0	0	0	0	0	0	7	0	0	0	0	0	0
0	0	0	0	0	0	0	0	0	0	0	0	0	0
0	4	0	0	0	0	0	0	0	0	0	0	0	0
0	0	0	0	0	0	0	0	0	0	0	0	1	0
0	1	0	0	0	0	0	0	0	0	0	0	0	0
0	0	0	0	0	0	0	0	0	2	0	1	2	0
0	1	0	0	9	0	1	6	0	0	0	3	0	0
0													
OTU4266	0	0	0	0	0	0	0	0	0	0	1	0	0
0	0	0	0	0	0	0	0	0	0	0	0	0	0
0	0	0	0	0	0	0	0	0	0	0	0	0	0
0	0	0	0	0	0	0	0	0	0	0	0	0	0
0	0	0	0	0	0	0	0	0	0	0	0	0	0
0	0	0	0	0	0	0	0	0	0	0	0	0	0
0	0	0	0	0	0	0	0	0	0	0	0	0	0
0	0	0	0	0	0	0	0	0	0	0	0	0	0
0													

OTU78	0	0	0	0	0	0	0	0	0	0	29	0	19	133
	0	7	9	0	0	0	4	8	0	0	0	1	0	1
	14	66	13	14	2	11	5	0	0	4	5	0	6	0
	4	0	0	20	0	20	0	3	9	30	0	2	2	44
	0	368	0	107	0	10	2	0	51	1	259	0	224	3
	1	0	0	0	1	1	0	1	0	0	0	1	0	16
	0	37	0	0	0	2	0	549	0	4	0	0	6	133
	0	0	0	0	0	0	0	0	0	5	0	64	0	0
OTU7641		0	0	0	0	0	0	0	0	0	0	1	0	0
	0	0	0	0	0	0	0	0	0	0	0	0	0	0
	0	0	0	0	0	0	0	0	0	0	0	0	0	0
	0	0	0	0	0	0	0	0	0	0	0	0	0	0
	0	0	0	0	0	0	0	0	0	0	0	0	0	0
	0	0	0	0	0	0	0	0	0	0	0	0	0	0
	0	0	0	0	0	0	0	0	0	0	0	0	0	0
	0	0	0	0	0	0	0	0	0	0	0	0	0	0
	0													
OTU408		0	0	0	0	0	0	0	0	0	0	12	0	1
	1	0	0	0	2	23	0	0	0	3	0	0	0	0
	0	0	8	1	0	0	0	0	0	0	21	0	0	18
	0	0	0	0	0	0	0	0	0	0	0	0	0	0
	0	0	0	0	0	0	0	0	0	0	0	0	0	0
	0	0	0	4	0	0	0	0	0	0	0	0	0	0
	0	0	0	0	1	0	0	0	3	0	0	0	0	0
	0	0	0	0	0	0	0	0	0	0	0	0	0	0
	0													
OTU4268		0	0	0	0	0	0	0	0	0	0	1	0	0
	0	0	0	0	0	1	0	0	0	0	0	0	0	0
	0	0	0	0	0	0	0	0	0	0	0	0	0	0
	0	0	0	0	0	0	0	0	0	0	0	0	0	0
	0	0	0	0	0	0	0	0	0	0	0	0	0	0
	0	0	0	0	0	0	0	0	0	0	0	0	0	0
	0	0	0	0	0	0	0	0	0	0	0	0	0	0

0	0	0	0	0	0	0	0	0	0	0	0	0	0
0													
OTU12512	0	0	0	0	0	0	0	0	0	0	1	0	0
0	0	0	0	0	0	0	0	0	0	0	0	0	0
0	0	0	0	0	0	0	0	0	0	0	0	0	0
0	0	0	0	0	0	0	0	0	0	0	0	0	0
0	0	0	0	0	0	0	0	0	0	0	0	0	0
0	0	0	0	0	0	0	0	0	0	0	0	0	0
0	0	0	0	0	0	0	0	0	0	0	0	0	0
0	0	0	0	0	0	0	0	0	0	0	0	0	0
0													
OTU2453	0	0	0	0	0	0	0	0	0	0	4	0	4
2	0	1	0	1	1	0	0	0	0	0	0	0	0
0	0	1	0	0	0	0	2	0	0	0	0	0	3
0	0	0	0	0	0	1	0	0	0	0	0	0	0
0	0	0	0	0	0	0	0	0	0	0	0	0	0
0	0	0	0	0	0	0	0	0	0	0	0	1	0
1	0	0	0	0	0	1	0	4	0	1	0	0	0
1	0	0	0	0	0	0	0	0	0	0	1	0	0
0													
OTU4263	0	0	0	0	0	0	0	0	0	0	1	0	0
0	0	0	0	0	0	0	0	0	0	0	0	0	0
0	0	0	0	0	0	0	0	0	0	0	0	0	0
0	0	0	0	0	0	0	0	0	0	0	0	0	0
0	1	0	0	0	0	0	0	0	0	0	0	0	0
0	0	0	0	0	0	0	0	0	0	0	0	0	0
0	0	0	0	0	0	0	0	0	0	0	0	0	0
0	0	0	0	0	0	0	0	0	0	0	0	0	0
0													
OTU13659	0	0	0	0	0	0	0	0	0	0	1	0	0
2	0	0	0	0	0	0	0	0	0	0	0	0	0
0	0	0	0	1	0	0	1	0	0	0	0	0	0
0	0	0	0	0	0	0	0	0	0	0	0	0	0

0	0	0	0	0	0	0	0	0	0	0	0	0	0
0	0	0	0	0	0	0	0	0	0	0	0	0	0
0	0	0	0	0	0	0	0	1	0	0	0	0	1
0	0	0	0	0	0	0	0	0	0	0	0	0	0
0													
OTU674	0	0	0	0	0	0	0	0	0	0	28	0	1
0	0	0	0	0	0	0	0	0	0	0	0	0	0
0	0	0	0	0	0	0	0	0	0	0	0	0	0
0	0	0	0	0	0	0	0	0	0	0	0	0	0
0	0	0	0	0	0	0	0	0	0	0	0	0	0
0	0	0	0	0	0	0	0	0	0	0	0	0	0
0	0	0	0	0	0	0	0	0	0	0	0	0	0
0	0	0	0	0	0	0	0	0	0	0	0	0	0
0													
OTU656	0	0	0	0	0	0	0	0	0	0	4	0	5
0	0	0	0	0	0	0	0	0	0	0	0	0	1
0	0	0	0	0	0	0	0	0	0	0	0	0	0
0	2	0	1	0	0	0	0	0	0	0	0	0	0
0	0	0	0	0	0	0	0	0	0	0	0	0	0
0	0	0	2	0	0	0	0	1	0	0	0	0	0
1	0	1	0	1	0	0	0	1	0	0	0	0	0
0	0	0	0	0	0	0	0	0	0	0	0	0	0
0													
OTU12508	0	0	0	0	0	0	0	0	0	0	1	0	0
0	0	0	0	0	0	0	0	0	0	0	0	0	0
0	0	0	0	0	0	0	0	0	0	0	0	0	0
0	0	0	0	0	0	0	0	0	0	0	0	0	0
0	0	0	0	0	0	0	0	0	0	0	0	0	0
0	0	0	0	0	0	0	0	0	0	0	0	0	0
0	0	0	0	0	0	0	0	0	0	0	0	0	0
0	0	0	0	0	0	0	0	0	0	0	0	0	0
0													

OTU7636 0 0 0 0 0 0 0 0 0 0 1 0 0
0 0 0 0 0 0 0 0 0 0 0 0 0 0
0 0 0 0 0 0 0 0 0 0 0 0 0 0
0 0 0 0 0 0 0 0 0 0 0 0 0 0
0 0 0 0 0 0 0 0 0 0 0 0 0 0
0 0 0 0 0 0 0 0 0 0 0 0 0 0
0 0 0 0 0 0 0 0 0 0 0 0 0 0
0 0 0 0 0 0 0 0 0 0 0 0 0 0
0
OTU15610 0 0 0 0 0 0 0 0 0 0 1 0 0
0 0 0 0 0 0 0 0 0 0 0 0 0 0
0 0 0 0 0 0 0 0 0 0 0 0 0 0
0 0 0 0 0 0 0 0 0 0 0 0 0 0
0 0 0 0 0 0 0 0 0 0 0 0 0 0
0 0 0 0 0 0 0 0 0 0 0 0 0 0
0 0 0 0 0 0 0 0 0 0 0 0 0 0
0 0 0 0 0 0 0 0 0 0 0 0 0 0
0
OTU15609 0 0 0 0 0 0 0 0 0 0 1 0 0
0 0 0 0 0 0 0 0 0 0 0 0 0 0
0 0 0 0 0 0 0 0 0 0 0 0 0 0
0 0 0 0 0 0 0 0 0 0 0 0 0 0
0 0 0 0 0 0 0 0 0 0 0 0 0 0
0 0 0 0 0 0 0 0 0 0 0 0 0 0
0 0 0 0 0 0 0 0 0 0 0 0 0 0
0 0 0 0 0 0 0 0 0 0 0 0 0 0
0
OTU12509 0 0 0 0 0 0 0 0 0 0 1 0 0
0 0 0 0 0 0 0 0 0 0 0 0 0 0
0 0 0 0 0 0 0 0 0 0 0 0 0 0
0 0 0 0 0 0 0 0 0 0 0 0 0 0
0 0 0 0 0 0 0 0 0 0 0 0 0 0
0 0 0 0 0 0 0 0 0 0 0 0 0 0

0	0	0	0	0	0	0	0	0	0	0	0	0	0
0	0	0	0	0	0	0	0	0	0	0	0	0	0
0													
OTU7635	0	0	0	0	0	0	0	0	0	0	1	0	0
0	0	0	0	0	0	0	0	0	0	0	0	0	0
0	0	0	0	0	0	0	0	0	0	0	0	0	0
0	0	0	0	0	0	0	0	0	0	0	0	0	0
0	0	0	0	0	0	0	0	0	0	0	0	0	0
0	0	0	0	0	0	0	0	0	0	0	0	0	0
0	0	0	0	0	0	0	0	0	0	0	0	0	0
0	0	0	0	0	0	0	0	0	0	0	0	0	0
0													
OTU7634	0	0	0	0	0	0	0	0	0	0	2	1	1
5	0	0	0	1	0	0	0	0	0	0	0	0	0
0	0	0	1	1	0	0	0	0	0	0	0	0	0
0	0	0	0	0	0	0	0	0	0	0	0	1	0
0	0	0	0	0	0	0	0	0	1	0	0	0	0
0	0	0	0	0	0	1	0	0	0	0	0	0	0
1	0	0	0	0	0	0	0	0	0	1	0	0	0
1	0	0	0	0	0	0	0	0	0	0	1	1	0
0													
OTU13695	0	0	0	0	0	0	0	0	0	0	1	0	0
0	0	0	0	0	0	0	0	0	0	0	0	0	0
0	0	0	0	0	0	0	1	1	0	0	0	0	0
0	0	0	0	0	0	0	0	0	0	0	0	0	0
0	0	0	0	0	0	0	0	0	0	0	0	0	0
0	0	0	0	0	0	0	0	0	0	0	0	0	0
0	0	0	0	0	0	0	0	1	1	1	0	0	0
0	0	0	0	0	0	0	0	0	0	0	0	0	0
0													
OTU340	0	0	0	0	0	0	0	0	0	0	24	0	6
27	0	0	0	0	0	0	0	0	0	0	0	0	0
0	0	0	0	0	0	0	0	0	0	0	0	0	0

0	0	0	0	0	0	0	0	0	0	0	0	0	0
0	0	0	0	0	0	0	5	0	0	0	0	0	0
0	0	0	0	0	0	0	0	0	0	0	0	1	0
0	0	0	0	0	0	0	0	31	0	0	0	0	3
0	0	0	0	0	0	0	0	0	0	0	0	0	0
0													
OTU478	0	0	0	0	0	0	0	0	0	0	40	0	8
0	0	0	0	0	0	0	0	0	0	0	0	0	0
0	0	0	0	0	0	0	0	0	0	0	0	0	0
0	0	0	0	0	0	0	0	0	0	0	0	0	0
0	0	0	0	0	0	0	1	0	0	0	0	0	0
0	0	0	0	0	0	0	0	0	0	0	0	0	0
0	0	0	0	0	0	0	0	0	0	0	0	0	0
0	0	0	0	0	0	0	0	0	0	0	0	0	0
0													
OTU1259	0	0	0	0	0	0	0	0	0	0	2	4	0
4	0	0	0	0	0	0	0	0	0	0	0	0	0
0	0	1	0	0	1	0	8	1	0	0	0	0	0
0	5	0	1	0	0	1	0	0	0	0	0	0	0
0	0	0	0	0	0	0	0	0	0	0	0	0	0
0	0	0	3	0	0	0	0	4	0	0	0	1	0
0	0	0	0	17	0	0	0	1	2	8	0	0	0
0	0	0	0	0	0	0	0	1	0	6	2	1	0
0													
OTU4201	0	0	0	0	0	0	0	0	0	0	1	0	0
0	0	0	0	0	0	0	0	0	0	0	0	0	0
0	0	0	0	0	0	0	0	0	0	0	0	0	0
0	0	0	0	0	0	0	0	0	0	0	0	0	0
0	0	0	0	0	0	0	0	0	0	0	0	0	0
0	0	0	0	0	0	0	0	0	0	0	0	0	0
0	0	0	0	0	0	0	0	0	0	0	0	0	0
0	0	0	0	0	0	0	0	0	0	0	0	0	0
0													

OTU427	0	0	0	0	0	0	0	0	0	0	9	0	0
1	0	0	0	0	2	0	0	0	0	0	0	0	0
0	0	10	0	0	3	0	0	0	0	0	0	0	0
0	0	0	0	0	0	0	0	0	0	0	0	0	0
0	0	2	0	0	0	0	0	0	0	0	0	0	0
0	0	0	0	0	0	0	0	1	0	0	0	0	0
1	0	1	0	11	0	0	0	87	0	1	0	0	23
0	0	0	0	0	0	0	0	1	0	0	4	0	0
0													
OTU15611	0	0	0	0	0	0	0	0	0	0	1	0	0
0	0	0	0	0	0	0	0	0	0	0	0	0	0
0	0	0	0	0	0	0	0	0	0	0	0	0	0
0	0	0	0	0	0	0	0	0	0	0	0	0	0
0	0	0	0	0	0	0	0	0	0	0	0	0	0
0	0	0	0	0	0	0	0	0	0	0	0	0	0
0	0	0	0	0	0	0	0	0	0	0	0	0	0
0	0	0	0	0	0	0	0	0	0	0	0	0	0
0													
OTU12510	0	0	0	0	0	0	0	0	0	0	1	0	0
0	0	0	0	0	0	0	0	0	0	0	0	0	0
0	0	0	0	0	0	0	0	0	0	0	0	0	0
0	0	0	0	0	0	0	0	0	0	0	0	0	0
0	0	0	0	0	0	0	0	0	0	0	0	0	0
0	0	0	0	0	0	0	0	0	0	0	0	0	0
0	0	0	0	0	0	0	0	0	0	0	0	0	0
0	0	0	0	0	0	0	0	0	0	0	0	0	0
0													
OTU9378	0	0	0	0	0	0	0	0	0	0	1	0	1
1	0	0	0	0	0	0	0	0	0	0	0	0	1
0	0	2	0	0	0	0	2	0	0	0	1	0	0
0	2	0	0	0	0	1	0	0	0	0	0	0	0
0	0	0	0	0	0	0	0	0	0	0	0	0	0
0	0	0	0	0	0	1	0	1	0	0	0	0	0

0	0	0	0	0	0	0	0	1	1	2	0	0	1
0	0	0	0	0	0	0	0	0	0	1	0	0	0
0													
OTU15607	0	0	0	0	0	0	0	0	0	0	1	0	0
0	0	0	0	0	0	0	0	0	0	0	0	0	0
0	0	0	0	0	0	0	0	0	0	0	0	0	0
0	0	0	0	0	0	0	0	0	0	0	0	0	0
0	0	0	0	0	0	0	0	0	0	0	0	0	0
0	0	0	0	0	0	0	0	0	0	0	0	0	0
0	0	0	0	0	0	0	0	0	0	0	0	0	0
0	0	0	0	0	0	0	0	0	0	0	0	0	0
0													
OTU12534	0	0	0	0	0	0	0	0	0	0	1	0	0
0	0	0	0	0	0	0	0	0	0	0	0	0	0
0	0	0	0	0	0	0	0	0	0	0	0	0	0
0	0	0	0	0	0	0	0	0	0	0	0	0	0
0	0	0	0	0	0	0	0	0	0	0	0	0	0
0	0	0	0	0	0	0	0	0	0	0	0	0	0
0	0	0	0	0	0	0	0	0	0	0	0	0	0
0	0	0	0	0	0	0	0	0	0	0	0	0	0
0													
OTU7722	0	0	0	0	0	0	0	0	0	0	1	0	0
0	0	0	0	0	0	0	0	0	0	0	0	0	0
0	0	0	0	0	0	0	0	0	0	0	0	0	0
0	0	0	0	0	0	0	0	0	0	0	0	0	0
0	0	0	0	0	0	0	0	0	0	0	0	0	0
0	0	0	0	0	0	0	0	0	0	0	0	0	0
0	0	0	0	0	0	0	0	0	0	0	0	0	0
0	0	0	0	0	0	0	0	0	0	0	0	0	0
0													
OTU4202	0	0	0	0	0	0	0	0	0	0	1	0	0
0	0	0	0	0	0	0	0	0	0	0	0	0	0
0	0	0	0	0	0	0	0	0	0	0	0	0	0

0	0	0	0	0	0	0	0	0	0	0	0	0	0
0	0	0	0	0	0	0	0	0	0	0	0	0	0
0	0	0	0	0	0	0	0	0	0	0	0	0	0
0	0	0	0	0	0	0	0	0	0	0	0	0	0
0	0	0	0	0	0	0	0	0	0	0	0	0	0
0													
OTU4203	0	0	0	0	0	0	0	0	0	0	1	0	1
0	0	0	0	0	0	0	0	0	0	0	0	0	0
0	0	0	0	0	0	0	0	0	0	0	0	0	0
0	0	0	0	0	0	0	0	0	0	0	0	0	0
0	0	0	0	0	0	0	1	0	0	0	0	0	0
0	0	0	0	0	0	0	0	0	0	0	0	0	0
0	0	0	0	0	0	0	0	0	0	0	0	0	0
0	0	0	0	0	0	0	0	0	0	0	0	0	0
0													
OTU3647	0	0	0	0	0	0	0	0	0	0	1	0	0
0	0	0	0	0	0	0	0	0	0	0	0	0	6
0	0	0	0	0	0	0	0	0	1	0	0	0	0
0	0	0	0	0	0	0	0	0	0	0	0	0	0
0	0	0	0	0	0	0	0	0	0	0	0	0	0
0	0	0	0	0	0	0	0	0	0	0	0	0	0
0	0	0	0	0	0	0	0	0	0	0	0	0	0
0	0	0	0	0	0	0	0	0	0	0	0	0	0
0													
OTU15606	0	0	0	0	0	0	0	0	0	0	1	0	0
0	0	0	0	0	0	0	0	0	0	0	0	0	0
0	0	0	0	0	0	0	0	0	0	0	0	0	0
0	0	0	0	0	0	0	0	0	0	0	0	0	0
0	0	0	0	0	0	0	0	0	0	0	0	0	0
0	0	0	0	0	0	0	0	0	0	0	0	0	0
0	0	0	0	0	0	0	0	0	0	0	0	0	0
0	0	0	0	0	0	0	0	0	0	0	0	0	0
0													

OTU12533	0	0	0	0	0	0	0	0	0	0	1	0	0
0	0	0	0	0	0	0	0	0	0	0	0	0	0
0	0	0	0	0	0	0	0	0	0	0	0	0	0
0	0	0	0	0	0	0	0	0	0	0	0	0	0
0	0	0	0	0	0	0	0	0	0	0	0	0	0
0	0	0	0	0	0	0	0	0	0	0	0	0	0
0	0	0	0	0	0	0	0	0	0	0	0	0	0
0	0	0	0	0	0	0	0	0	0	0	0	0	0
0													
OTU6704	0	0	0	0	0	0	0	0	0	0	2	0	0
0	0	0	0	0	0	0	0	0	0	0	0	0	0
0	0	0	0	0	0	0	0	0	0	0	0	0	0
0	0	0	0	0	0	0	0	0	0	0	0	0	0
0	0	0	0	0	0	0	0	0	0	0	0	0	0
0	0	0	0	0	0	0	0	0	0	0	0	0	0
0	0	0	0	0	0	0	0	20	0	0	0	0	0
0	0	0	0	0	0	0	0	0	0	0	0	0	0
0													
OTU4087	0	0	0	0	0	0	0	0	0	0	1	0	1
1	0	0	0	0	0	0	0	0	0	0	0	0	0
0	0	0	0	0	0	0	1	0	0	0	0	0	0
0	0	0	0	0	0	0	0	0	0	0	0	0	0
0	0	0	0	0	0	0	0	0	0	0	0	0	0
0	0	0	0	0	0	0	0	0	0	0	0	0	0
0	0	0	0	0	0	0	0	0	0	0	0	0	0
0	0	0	0	0	0	1	0	1	0	0	0	0	0
0													
OTU4896	0	0	0	0	0	0	0	0	0	0	1	0	0
0	0	0	0	0	0	0	0	0	0	0	1	0	0
0	0	1	0	0	0	0	0	0	0	0	0	0	0
0	0	0	0	0	0	0	0	0	0	1	0	0	0
0	0	0	1	0	0	0	0	0	0	0	0	0	0
0	0	0	0	0	0	0	0	0	0	0	0	0	0

0	1	0	0	0	0	0	0	0	0	0	0	1	0
1	0	0	0	0	0	0	0	0	0	0	0	0	0
0													
OTU4199	0	0	0	0	0	0	0	0	0	0	1	0	1
1	0	0	0	0	0	0	0	0	0	0	0	0	0
0	0	0	0	0	0	0	0	0	0	0	0	0	0
0	0	0	0	0	0	0	0	0	0	0	0	0	0
0	0	0	0	0	0	0	0	0	0	0	0	0	0
0	0	0	0	0	0	0	0	0	0	0	0	0	0
0	0	0	0	0	0	0	0	0	0	0	0	0	0
0	0	0	0	0	0	0	0	0	0	0	0	0	0
0													
OTU608	0	0	0	0	0	0	0	0	0	0	3	0	1
0	0	0	0	0	2	0	0	0	0	0	0	0	0
0	0	1	0	0	0	0	2	0	0	0	0	0	0
0	1	0	0	0	0	0	0	0	0	0	0	0	0
0	0	0	0	0	0	0	0	0	0	0	0	0	1
0	0	0	0	0	0	0	0	1	0	0	0	0	0
0	0	0	0	0	0	0	0	2	0	0	0	0	16
0	0	0	0	0	0	0	0	0	0	0	0	0	0
0													
OTU520	0	0	0	0	0	0	0	0	0	0	1	0	0
0	0	0	1	0	6	0	0	0	1	0	0	0	0
0	0	0	0	0	0	0	0	0	0	1	0	4	0
0	0	7	5	0	0	0	0	0	0	0	0	0	0
0	0	0	2	0	0	0	0	0	0	0	0	0	0
0	0	0	0	0	0	0	0	1	0	0	0	0	0
0	1	11	0	0	0	0	0	0	0	0	0	0	0
0	0	0	0	0	0	0	0	0	0	0	0	0	0
0													
OTU7201	0	0	0	0	0	0	0	0	0	0	1	0	0
1	0	2	8	2	0	0	1	0	0	8	0	0	0
0	3	2	4	4	4	3	0	1	2	5	0	0	4

0	0	2	1	0	0	0	0	1	0	0	0	0	1
1	0	1	0	3	1	0	0	0	0	0	2	0	2
0	0	0	2	0	1	0	1	1	0	2	0	0	0
0	0	1	0	3	0	0	0	1	0	0	0	0	0
0	0	0	1	0	0	0	0	0	0	3	2	5	0
0													
OTU1136	0	0	0	0	0	0	0	0	0	0	1	0	0
0	0	0	0	0	0	0	0	0	0	0	0	0	4
0	0	0	0	0	0	0	0	0	0	0	0	0	0
0	0	0	0	0	0	0	0	0	0	0	0	0	0
0	0	0	0	0	1	0	0	0	0	0	0	0	0
0	0	0	0	0	0	0	0	0	0	0	0	0	0
0	0	0	0	0	0	0	0	0	0	0	0	0	0
0	0	0	0	0	0	0	0	0	0	0	0	0	0
0													
OTU4198	0	0	0	0	0	0	0	0	0	0	1	0	0
0	0	0	0	0	0	0	0	0	0	0	0	0	0
0	0	0	0	0	0	0	0	0	0	0	0	0	0
0	0	0	0	0	0	0	0	0	0	0	0	0	0
0	0	0	0	0	0	0	0	0	0	0	0	0	0
0	0	0	0	0	0	0	0	0	0	0	0	0	0
0	0	0	0	0	0	0	0	0	0	0	0	0	0
0	0	0	0	0	0	0	0	0	0	0	0	0	0
0													
OTU15608	0	0	0	0	0	0	0	0	0	0	1	0	0
0	0	0	0	0	0	0	0	0	0	0	0	0	0
0	0	0	0	0	0	0	0	0	0	0	0	0	0
0	0	0	0	0	0	0	0	0	0	0	0	0	0
0	0	0	0	0	0	0	0	0	0	0	0	0	0
0	0	0	0	0	0	0	0	0	0	0	0	0	0
0	0	0	0	0	0	0	0	0	0	0	0	0	0
0	0	0	0	0	0	0	0	0	0	0	0	0	0
0													

OTU7719	0	0	0	0	0	0	0	0	0	0	1	0	0
0	0	0	0	0	0	0	0	0	0	0	0	0	0
0	0	0	0	0	0	0	0	0	0	0	0	0	0
0	0	1	0	0	0	0	0	0	0	0	0	0	0
0	0	0	0	0	0	0	0	0	0	0	0	0	0
0	0	0	0	0	0	0	0	0	0	0	1	0	0
0	0	0	0	0	0	0	0	0	0	0	0	0	0
0	0	0	0	0	0	0	0	0	0	0	0	0	0
0													
OTU4200	0	0	0	0	0	0	0	0	0	0	1	0	0
0	0	0	0	0	0	0	0	0	0	0	0	0	0
0	0	0	0	0	0	0	0	0	0	0	0	0	0
0	0	0	0	0	0	0	0	0	0	0	0	0	0
0	0	0	0	0	0	0	0	0	0	0	0	0	0
0	0	0	0	0	0	0	0	0	0	0	0	0	0
0	0	0	0	0	0	0	0	0	0	0	0	0	0
0	0	0	0	0	0	0	0	0	0	0	0	0	0
0													
OTU3964	0	0	0	0	0	0	0	0	0	0	1	0	0
0	0	1	0	0	1	0	0	0	1	0	0	0	0
0	0	1	0	0	0	0	0	0	0	0	0	0	0
0	0	0	0	0	0	0	0	0	0	0	0	0	0
0	0	0	0	0	0	0	0	0	0	0	0	0	0
0	0	0	0	0	0	0	0	0	0	0	0	0	0
0	0	0	0	0	0	0	0	0	0	0	0	0	0
0	0	0	0	0	0	0	0	0	0	0	0	0	0
0													
OTU5042	0	0	0	0	0	0	0	0	0	0	3	0	0
0	0	0	0	1	0	0	0	0	0	0	0	0	0
0	0	0	0	0	0	0	0	0	0	0	0	0	0
0	0	0	0	0	0	0	0	0	0	0	0	0	0
0	1	2	0	0	0	0	0	0	0	0	0	0	0
0	0	0	1	0	0	0	0	0	0	0	0	1	0

0	0	0	0	0	0	0	0	1	0	0	0	0	0
0	0	0	0	0	0	0	0	0	0	0	0	0	0
0													
OTU15599	0	0	0	0	0	0	0	0	0	0	1	0	0
0	0	0	0	0	0	0	0	0	0	0	0	0	0
0	0	0	0	0	0	0	0	0	0	0	0	0	0
0	0	0	0	0	0	0	0	0	0	0	0	0	0
0	0	0	0	0	0	0	0	0	0	0	0	0	0
0	0	0	0	0	0	0	0	0	0	0	0	0	0
0	0	0	0	0	0	0	0	0	0	0	0	0	0
0	0	0	0	0	0	0	0	0	0	0	0	0	0
0													
OTU8095	0	0	0	0	0	0	0	0	0	0	4	0	10
11	0	0	0	0	0	0	0	0	0	0	0	0	0
0	0	1	0	0	0	0	0	0	0	0	0	0	0
0	0	0	0	0	0	0	0	0	0	0	0	0	0
0	0	0	0	0	0	0	0	0	0	0	0	0	0
0	0	0	0	0	1	0	0	0	0	0	0	0	0
0	0	0	0	0	0	0	0	2	0	0	0	0	0
0	0	0	0	0	0	0	0	0	0	0	0	0	0
0													
OTU8112	0	0	0	0	0	0	0	0	0	0	3	0	1
28	0	0	0	0	0	0	0	0	0	0	0	0	0
0	0	0	0	0	0	0	2	0	0	0	0	0	0
0	0	0	0	0	0	0	0	0	0	0	0	0	0
0	0	0	0	0	0	0	0	0	0	0	0	0	0
0	0	0	0	0	0	0	0	0	0	0	0	0	0
0	0	0	0	0	0	0	0	6	0	2	0	0	1
0	0	0	0	0	0	0	0	0	0	0	0	0	0
0													
OTU12531	0	0	0	0	0	0	0	0	0	0	1	0	0
0	0	0	0	0	0	0	0	0	0	0	0	0	0
0	0	0	0	0	0	0	0	0	0	0	0	0	0

0	0	0	0	0	0	0	0	0	0	0	0	0	0
0	0	0	0	0	0	0	0	0	0	0	0	0	0
0	0	0	0	0	0	0	0	0	0	0	0	0	0
0	0	0	0	0	0	0	0	0	0	0	0	0	0
0	0	0	0	0	0	0	0	0	0	0	0	0	0
0													
OTU813	0	0	0	0	0	0	0	0	0	0	13	0	6
0	0	0	0	0	0	0	0	0	0	0	0	0	0
0	0	7	0	0	0	0	0	0	0	0	0	0	0
0	0	0	0	0	0	0	0	0	0	0	0	0	0
0	0	0	0	0	0	0	0	0	0	0	0	0	0
0	0	0	0	0	0	0	0	0	0	0	0	0	0
0	0	8	0	0	0	0	0	4	0	0	0	0	0
0	0	0	0	0	0	0	0	0	0	0	0	0	0
0													
OTU1956	0	0	0	0	0	0	0	0	0	0	4	0	0
0	0	0	0	0	0	0	0	0	0	0	0	0	0
0	0	0	0	0	0	0	0	0	0	0	0	0	0
0	0	0	0	0	0	0	0	0	0	0	0	0	0
0	0	0	0	0	0	0	2	0	0	0	0	0	0
0	0	0	0	0	0	0	0	0	0	0	0	0	0
0	0	0	0	0	0	0	0	0	0	0	0	0	0
0	0	0	0	0	0	0	0	0	0	0	0	0	0
0													
OTU255	0	0	0	0	0	0	0	0	0	0	7	0	0
1	0	1	0	0	10	0	10	0	12	2	1	0	0
1	0	5	0	34	4	0	2	0	0	0	5	0	0
0	1	0	5	0	0	26	0	0	0	0	0	0	0
0	0	0	0	0	0	0	0	0	0	0	1	0	0
0	0	1	5	0	0	1	0	0	0	4	0	0	0
0	0	65	1	0	0	0	0	7	0	1	0	0	0
2	0	1	0	0	0	0	0	2	0	0	1	2	0
0													

OTU417 0 0 0 0 0 0 0 0 0 0 3 0 0
0 0 0 0 0 0 0 0 0 0 0 0 0 52
0 0 1 2 0 0 0 0 0 5 0 0 0 0
0 0 0 0 0 0 0 0 0 0 0 0 0 0
0 0 0 0 0 0 0 0 0 0 0 0 0 0
0 0 0 0 0 0 0 0 0 0 0 0 0 0
0 0 0 0 0 0 0 0 0 0 0 0 0 0
0 0 0 0 0 0 0 0 0 0 0 0 0 0
0
OTU4210 0 0 0 0 0 0 0 0 0 0 1 0 0
0 0 0 0 0 0 0 1 0 0 0 0 0 0
0 0 0 0 0 0 0 0 0 0 0 0 0 0
0 0 0 0 0 0 0 0 0 0 0 0 0 0
0 0 0 0 0 0 0 0 0 0 0 0 0 0
0 0 0 0 0 0 0 0 0 0 0 0 0 0
0 0 0 0 0 0 0 0 0 0 0 0 0 0
0 0 0 0 0 0 0 0 0 0 0 0 0 0
0
OTU7726 0 0 0 0 0 0 0 0 0 0 1 0 0
0 0 0 0 0 0 0 0 0 0 0 0 0 0
0 0 0 0 0 0 0 0 0 0 0 0 0 0
0 0 0 0 0 0 0 0 0 5 0 0 0 0
0 0 0 0 0 0 0 0 0 0 0 0 0 0
0 0 0 0 0 0 0 0 0 0 0 0 0 0
0 0 0 0 0 0 0 0 0 0 0 0 0 0
0 0 0 0 0 0 0 0 0 0 0 0 0 0
0
OTU12532 0 0 0 0 0 0 0 0 0 0 1 0 0
0 0 0 0 0 0 0 0 0 0 0 0 0 0
0 0 0 0 0 0 0 0 0 0 0 0 0 0
0 0 0 0 0 0 0 0 0 0 0 0 0 0
0 0 0 0 0 0 0 0 0 0 0 0 0 0
0 0 0 0 0 0 0 0 0 0 0 0 0 0

0	0	0	0	0	0	0	0	0	0	0	0	0	0
0	0	0	0	0	0	0	0	0	0	0	0	0	0
0													
OTU4207	0	0	0	0	0	0	0	0	0	0	1	0	0
0	0	0	0	0	0	0	0	0	0	0	0	0	0
0	0	0	0	0	0	0	0	0	0	0	0	0	0
0	0	0	0	0	0	0	0	0	0	0	0	0	0
0	0	0	0	0	0	0	0	0	0	0	0	0	0
0	0	0	0	0	0	0	0	0	0	0	0	0	0
0	0	0	0	0	0	0	0	0	0	0	0	0	0
0	0	0	0	0	0	0	0	0	0	0	0	0	0
0													
OTU4206	0	0	0	0	0	0	0	0	0	0	1	0	0
0	0	0	0	0	0	0	0	0	0	0	0	0	0
0	0	0	0	0	0	0	0	0	0	0	0	0	0
0	0	0	0	0	0	0	0	0	0	0	0	0	0
0	0	0	0	0	0	0	0	0	0	0	0	0	0
0	0	0	0	0	0	0	0	0	0	0	0	0	0
0	0	0	0	0	0	0	0	0	0	0	0	0	0
0	0	0	0	0	0	0	0	0	0	0	0	0	0
0													
OTU4205	0	0	0	0	0	0	0	0	0	0	2	0	0
0	0	0	0	0	0	0	0	0	0	0	0	0	0
0	0	0	0	0	0	0	0	0	0	0	0	0	0
0	0	0	0	0	0	0	0	0	0	0	0	0	0
0	0	0	0	0	0	0	0	0	0	0	0	0	0
0	0	0	0	0	0	0	0	0	0	0	0	0	0
0	0	0	0	0	0	0	0	0	0	0	0	0	0
0	0	0	0	0	0	0	0	0	0	0	0	0	0
0													
OTU4204	0	0	0	0	0	0	0	0	0	0	1	0	0
0	0	0	0	0	0	0	0	0	0	0	0	0	0
0	0	0	0	0	0	0	0	0	0	0	0	0	0

0	0	0	0	0	0	0	0	0	0	0	0	0	0
0	0	0	0	0	0	0	0	0	0	0	0	0	0
0	0	0	0	0	0	0	0	0	0	0	0	0	0
0	0	0	0	0	0	0	0	0	0	0	0	0	0
0	0	0	0	0	0	0	0	0	0	0	0	0	0
0													
OTU15600	0	0	0	0	0	0	0	0	0	0	1	0	0
0	0	0	0	0	0	0	0	0	0	0	0	0	0
0	0	0	0	0	0	0	0	0	0	0	0	0	0
0	0	0	0	0	0	0	0	0	0	0	0	0	0
0	0	0	0	0	0	0	0	0	0	0	0	0	0
0	0	0	0	0	0	0	0	0	0	0	0	0	0
0	0	0	0	0	0	0	0	0	0	0	0	0	0
0	0	0	0	0	0	0	0	0	0	0	0	0	0
0													
OTU7728	0	0	0	0	0	0	0	0	0	0	1	0	0
0	0	0	0	0	0	0	0	0	0	0	0	0	0
0	0	0	0	0	0	0	0	0	0	0	0	0	0
0	0	0	0	0	0	0	0	0	0	0	0	0	0
0	0	0	0	0	0	0	0	0	0	0	0	0	0
0	0	0	0	0	0	0	0	0	0	0	0	0	0
0	0	0	0	0	0	0	0	0	0	0	0	0	0
0	0	0	0	0	0	0	0	0	0	0	0	0	0
0													
OTU2599	0	0	0	0	0	0	0	0	0	0	1	0	0
0	0	0	0	0	0	0	0	0	0	0	0	0	1
0	0	0	0	0	0	0	0	0	0	0	0	0	0
0	0	0	0	0	0	0	0	0	0	0	0	0	0
0	0	0	0	0	0	0	0	0	0	0	0	0	0
0	0	0	0	0	0	0	0	0	0	0	0	0	0
0	0	0	0	0	0	0	1	0	0	0	0	0	0
0	0	0	0	0	0	0	0	0	0	0	0	0	0
0													

OTU11855	0	0	0	0	0	0	0	0	0	0	1	0	0
0	0	2	0	0	0	0	0	0	0	0	0	0	0
0	0	5	0	0	0	0	0	0	0	0	0	0	0
0	0	0	0	0	0	0	0	0	0	0	0	0	0
0	0	0	0	0	0	0	0	0	0	0	0	0	0
0	0	0	0	0	0	0	0	0	0	0	0	0	0
0	0	0	0	0	0	0	0	0	0	0	0	0	0
0	0	0	0	0	0	0	0	0	0	0	0	0	0
0													
OTU16455	0	0	0	0	0	0	0	0	0	0	2	0	0
0	0	1	0	1	0	0	0	0	0	0	0	0	0
0	0	0	0	0	0	0	0	0	0	0	0	0	3
0	0	0	0	0	0	0	0	0	0	0	0	0	1
0	0	0	0	0	0	0	0	0	0	0	0	0	0
0	0	0	0	0	0	1	0	1	0	0	0	0	0
2	0	1	0	2	0	1	0	1	0	0	0	0	0
0	0	0	0	0	0	0	0	0	0	0	0	0	0
0													
OTU4208	0	0	0	0	0	0	0	0	0	0	2	0	0
0	0	0	0	0	0	0	0	0	0	0	0	0	0
0	0	0	0	0	0	0	0	0	0	0	0	0	0
0	0	0	0	0	0	0	0	0	0	0	0	0	0
0	0	0	0	0	0	0	0	0	0	0	0	0	0
0	0	0	0	0	0	0	0	0	0	0	0	0	0
0	0	0	0	0	0	0	0	0	0	0	0	0	0
0	0	0	0	0	0	0	0	0	0	0	0	0	0
0													
OTU15959	0	0	0	0	0	0	0	0	0	0	1	0	0
0	1	0	0	0	2	0	0	0	0	0	0	0	0
0	0	0	0	0	0	0	1	0	0	0	0	0	0
0	0	0	0	0	0	1	0	0	0	0	0	0	1
0	0	0	0	0	0	0	0	0	0	0	0	0	0
0	0	0	0	0	0	0	0	0	0	0	0	0	0

0	0	0	0	0	0	0	0	0	0	0	0	0	1
1	0	0	0	0	0	0	0	0	0	0	0	0	0
0													
OTU12541	0	0	0	0	0	0	0	0	0	0	1	0	0
0	0	0	0	0	0	0	0	0	0	0	0	0	0
0	0	0	0	0	0	0	0	0	0	0	0	0	0
0	0	0	0	0	0	0	0	0	0	0	0	0	0
0	0	0	0	0	0	0	0	0	0	0	0	0	0
0	0	0	0	0	0	0	0	0	0	0	0	0	0
0	0	0	0	0	0	0	0	0	0	0	0	0	0
0	0	0	0	0	0	0	0	0	0	0	0	0	0
0													
OTU12539	0	0	0	0	0	0	0	0	0	0	1	0	0
0	0	0	0	0	0	0	0	0	0	0	0	0	0
0	0	0	0	0	0	0	0	0	0	0	0	0	0
0	0	0	0	0	0	0	0	0	0	0	0	0	0
0	0	0	0	0	0	0	0	0	0	0	0	0	0
0	0	0	0	0	0	0	0	0	0	0	0	0	0
0	0	0	0	0	0	0	0	0	0	0	0	0	0
0	0	0	0	0	0	0	0	0	0	0	0	0	0
0													
OTU12540	0	0	0	0	0	0	0	0	0	0	2	0	0
0	0	0	0	0	0	0	0	0	0	0	0	0	0
0	0	0	0	0	0	0	0	0	0	0	0	0	0
0	0	0	0	0	1	0	0	0	0	0	0	0	0
0	0	0	0	0	0	0	0	0	0	0	0	0	0
0	0	0	0	0	0	0	0	0	0	0	0	0	0
0	0	0	0	0	0	0	0	0	0	0	0	0	0
1	0	0	0	0	0	0	0	0	0	0	0	0	0
0													
OTU12543	0	0	0	0	0	0	0	0	0	0	1	0	0
0	0	0	0	0	0	0	0	0	0	0	0	0	0
0	0	0	0	0	0	0	0	0	0	0	0	0	0

0 0 0 0 0 0 0 0 0 0 0 0 0 0
0 0 0 0 0 0 0 0 0 0 0 0 0 0
0 0 0 0 0 0 0 0 0 0 0 0 0 0
0 0 0 0 0 0 0 0 0 0 0 0 0 0
0 0 0 0 0 0 0 0 0 0 0 0 0 0
0
OTU15598 0 0 0 0 0 0 0 0 0 0 1 0 0
0 0 0 0 0 0 0 0 0 0 0 0 0 0
0 0 0 0 0 0 0 0 0 0 0 0 0 0
0 0 0 0 0 0 0 0 0 0 0 0 0 0
0 0 0 0 0 0 0 0 0 0 0 0 0 0
0 0 0 0 0 0 0 0 0 0 0 0 0 0
0 0 0 0 0 0 0 0 0 0 0 0 0 0
0 0 0 0 0 0 0 0 0 0 0 0 0 0
0
OTU4189 0 0 0 0 0 0 0 0 0 0 1 0 0
0 0 0 0 0 0 0 0 0 0 0 0 0 0
0 0 0 0 0 0 0 0 0 0 0 0 0 0
0 0 0 0 0 0 0 0 0 0 0 0 0 0
0 0 0 0 0 0 0 0 0 0 0 0 0 0
0 0 0 0 0 0 0 0 0 0 0 0 0 0
0 0 0 0 0 0 0 0 0 0 0 0 0 0
0 0 0 0 0 0 0 0 0 0 0 0 0 0
0
OTU1954 0 0 0 0 0 0 0 0 0 0 1 0 0
0 0 0 0 0 0 0 0 0 0 0 0 0 0
0 0 0 0 0 0 0 0 0 0 0 0 0 0
0 0 0 0 0 0 0 0 0 0 0 0 0 0
0 0 0 0 0 0 0 1 0 0 0 0 1 0
0 0 0 0 0 0 0 0 0 0 0 0 0 0
0 0 0 0 0 0 0 0 0 0 0 0 0 0
0 0 0 0 0 0 0 0 0 0 0 0 0 0
0

OTU12542	0	0	0	0	0	0	0	0	0	0	1	0	0
0	0	0	0	0	0	0	0	0	0	0	0	0	0
0	0	0	0	0	0	0	0	0	0	0	0	0	0
0	0	0	0	0	0	0	0	0	0	0	0	0	0
0	0	0	0	0	0	0	0	0	0	0	0	0	0
0	0	0	0	0	0	0	0	0	0	0	0	0	0
0	0	0	0	0	0	0	0	0	0	0	0	0	0
0	0	0	0	0	0	0	0	0	0	0	0	0	0
0													
OTU4415	0	0	0	0	0	0	0	0	0	0	1	0	0
0	0	0	0	0	1	0	0	0	0	0	0	0	0
0	0	0	0	0	0	0	0	0	0	0	0	0	0
0	0	0	0	0	0	0	0	0	0	0	0	0	0
0	0	0	0	0	0	0	0	0	0	0	0	0	0
0	0	0	0	0	0	0	0	0	0	0	0	0	0
1	0	0	0	0	1	0	0	0	0	0	0	0	0
0	0	0	0	0	0	0	0	0	0	0	0	0	0
0													
OTU4191	0	0	0	0	0	0	0	0	0	0	1	0	0
0	0	0	0	0	0	0	0	0	0	0	0	0	0
0	0	0	0	0	0	0	0	0	0	0	0	0	0
0	0	0	0	0	0	0	0	0	0	0	0	0	0
0	0	0	0	0	0	0	0	0	0	0	0	0	0
0	0	0	0	0	0	0	0	0	0	0	0	0	0
0	0	0	0	0	0	0	0	0	0	0	0	0	0
0	0	0	0	0	0	0	0	0	0	0	0	0	0
0													
OTU7724	0	0	0	0	0	0	0	0	0	0	3	0	0
0	0	1	0	1	0	0	0	0	0	0	0	0	1
0	0	1	0	0	0	0	0	0	0	0	0	0	0
0	0	0	0	5	0	0	0	0	0	1	0	0	0
0	0	2	0	0	0	1	0	0	0	0	0	0	0
0	0	0	0	0	0	0	0	0	0	0	0	0	0

0	0	0	0	0	0	0	0	1	0	0	0	0	1
0	0	0	0	0	0	0	0	0	0	0	0	0	0
0													
OTU16372	0	0	0	0	0	0	0	0	0	0	1	0	0
0	0	0	0	0	0	1	0	0	0	0	0	0	0
0	0	0	0	0	0	0	0	0	0	0	0	0	0
0	0	0	0	0	0	0	0	0	0	0	0	0	0
0	1	0	0	0	0	0	0	0	0	0	0	0	0
0	0	0	0	0	0	0	0	0	0	0	0	0	0
0	0	0	0	0	0	0	0	0	1	0	0	1	0
0	0	0	0	0	0	0	0	0	0	0	0	0	0
0													
OTU819	0	0	0	0	0	0	0	0	0	0	1	0	5
0	0	0	0	0	0	0	0	0	0	0	0	0	0
0	0	0	0	0	0	0	0	0	0	0	0	0	0
0	0	0	0	0	0	0	0	0	0	0	0	0	0
0	0	0	0	0	0	0	0	0	0	0	0	0	0
0	0	0	0	0	0	0	0	0	0	0	0	0	0
0	0	0	0	0	0	0	0	1	0	0	0	0	0
0	0	0	0	0	0	0	0	0	0	0	0	0	0
0													
OTU11207	0	0	0	0	0	0	0	0	0	0	1	0	3
5	0	0	0	0	0	0	0	0	0	0	0	0	0
0	0	4	0	0	0	0	0	0	0	0	0	0	0
0	0	0	0	0	0	2	0	0	0	0	0	0	0
0	0	0	0	0	0	0	0	0	0	0	0	0	0
0	0	0	0	0	0	0	0	0	0	0	0	0	0
0	0	1	0	1	0	0	0	1	0	1	0	0	0
0	0	0	0	0	0	0	0	0	0	0	1	0	0
0													
OTU12536	0	0	0	0	0	0	0	0	0	0	1	0	0
0	0	0	0	0	0	0	0	0	0	0	0	0	0
0	0	0	0	0	0	0	0	0	0	0	0	0	0

0	0	0	0	0	0	0	0	0	0	0	0	0	0
0	0	0	0	0	0	0	0	0	0	0	0	0	0
0	0	0	0	0	0	0	0	0	0	0	0	0	0
0	0	0	0	0	0	0	0	0	0	0	0	0	0
0	0	0	0	0	0	0	0	0	0	0	0	0	0
0													
OTU11114	0	0	0	0	0	0	0	0	0	0	1	0	0
0	0	0	0	0	0	1	0	0	0	0	0	0	0
0	0	0	0	0	0	0	0	0	0	0	0	0	0
2	0	0	0	0	0	0	0	0	0	0	1	0	0
0	0	0	0	0	0	1	0	1	0	0	0	2	0
0	2	0	0	0	0	0	0	0	0	0	0	0	0
0	0	0	0	0	0	0	0	0	0	0	3	0	0
0	0	0	0	7	2	0	10	0	0	0	0	0	1
3													
OTU7712	0	0	0	0	0	0	0	0	0	0	1	0	0
0	0	0	0	0	0	0	0	0	0	0	0	0	0
0	0	0	0	0	0	0	0	0	0	0	0	0	0
0	0	0	0	0	0	0	0	0	0	0	0	0	0
0	0	0	0	0	0	0	0	0	0	0	0	0	0
0	0	0	0	0	0	0	0	0	0	0	0	0	0
0	0	0	0	0	0	0	0	0	0	0	0	0	0
0	0	0	0	0	0	0	0	0	0	0	0	0	0
0													
OTU3309	0	0	0	0	0	0	0	0	0	0	1	0	0
0	0	0	0	0	0	0	0	0	0	0	0	0	0
0	0	1	0	0	0	0	0	0	0	0	0	0	2
0	0	0	1	0	0	0	0	0	0	0	0	0	0
0	0	0	0	0	0	0	0	0	0	0	0	0	0
0	0	0	0	0	0	0	0	0	0	0	0	0	0
0	0	0	0	1	0	0	0	0	0	0	0	0	0
0	0	0	0	0	0	0	0	0	0	0	0	0	0
0													

OTU4187	0	0	0	0	0	0	0	0	0	0	1	0	0
0	0	0	0	0	0	0	0	0	0	0	0	0	0
0	0	0	0	0	0	0	0	0	0	0	0	0	0
0	0	0	0	0	0	0	0	0	0	0	0	0	0
0	0	0	0	0	0	0	0	0	0	0	0	0	0
0	0	0	0	0	0	0	0	0	0	0	0	0	0
0	0	0	0	0	0	0	0	0	0	0	0	0	0
0	0	0	0	0	0	0	0	0	0	0	0	0	0
0													
OTU1078	0	0	0	0	0	0	0	0	0	0	1	0	0
1	0	0	0	0	0	0	0	0	0	0	0	0	0
0	0	1	0	1	0	0	1	0	0	0	0	0	2
0	0	0	0	0	0	0	0	0	0	0	0	0	0
0	0	3	0	0	0	0	0	0	0	0	0	0	1
0	0	0	0	0	0	0	0	0	0	0	0	0	0
0	0	0	0	0	0	0	0	2	0	0	0	0	0
0	0	0	0	0	0	0	0	0	0	0	0	1	0
0													
OTU2150	0	0	0	0	0	0	0	0	0	0	2	0	0
0	0	0	0	0	0	0	0	0	0	0	0	0	0
0	0	0	0	0	0	0	0	0	0	0	0	0	0
0	0	0	0	0	0	0	0	0	0	0	0	0	0
0	0	0	0	0	0	0	0	0	0	0	0	0	0
1	0	0	0	0	0	0	0	0	0	0	0	0	0
0	0	0	0	0	0	0	0	0	0	1	0	0	0
0	0	0	0	0	0	0	0	0	0	0	0	0	0
0													
OTU16291	0	0	0	0	0	0	0	0	0	0	1	0	0
0	1	1	0	0	0	0	0	0	0	0	0	0	0
0	0	0	0	0	0	0	0	0	0	0	0	0	0
0	0	0	0	0	1	0	0	0	0	0	0	0	0
0	0	0	0	0	0	0	0	1	0	0	0	0	0
0	0	0	0	0	0	0	0	0	0	0	0	0	0

0	0	0	1	0	0	0	0	0	0	0	0	0	0
0	0	0	0	0	0	0	0	0	0	0	0	0	0
0													
OTU12537	0	0	0	0	0	0	0	0	0	0	1	0	0
0	0	0	0	0	0	0	0	0	0	0	0	0	0
0	0	0	0	0	0	0	0	0	0	0	0	0	0
0	0	0	0	0	0	0	0	0	0	0	0	0	0
0	0	0	0	0	0	0	0	0	0	0	0	0	0
0	0	0	0	0	0	0	0	0	0	0	0	0	0
0	0	0	0	0	0	0	0	0	0	0	0	0	0
0	0	0	0	0	0	0	0	0	0	0	0	0	0
0													
OTU12538	0	0	0	0	0	0	0	0	0	0	1	0	0
0	0	0	0	0	0	0	0	0	0	0	0	0	0
0	0	0	0	0	0	0	0	0	0	0	0	0	0
0	0	0	0	0	0	0	0	0	0	0	0	0	0
0	0	0	0	0	0	0	0	0	0	0	0	0	0
0	0	0	0	0	0	0	0	0	0	0	0	0	0
0	0	0	0	0	0	0	0	0	0	0	0	0	0
0	0	0	0	0	0	0	0	0	0	0	0	0	0
0													
OTU15602	0	0	0	0	0	0	0	0	0	0	1	0	0
0	0	0	0	0	0	0	0	0	0	0	0	0	0
0	0	0	0	0	0	0	0	0	0	0	0	0	0
0	0	0	0	0	0	0	0	0	0	0	0	0	0
0	0	0	0	0	0	0	0	0	0	0	0	0	0
0	0	0	0	0	0	0	0	0	0	0	0	0	0
0	0	0	0	0	0	0	0	0	0	0	0	0	0
0	0	0	0	0	0	0	0	0	0	0	0	0	0
0													
OTU11915	0	0	0	0	0	0	0	0	0	0	1	0	0
0	0	0	0	0	0	0	0	0	1	0	0	0	0
0	0	0	0	0	0	0	0	0	0	0	0	0	0

0	0	0	0	0	0	0	0	0	0	0	0	0	0
0	0	0	0	0	0	0	0	0	0	0	0	0	0
0	0	0	0	0	0	0	0	0	0	0	0	0	0
0	0	0	0	0	0	0	0	0	0	0	0	0	0
0	0	0	0	0	0	0	0	0	0	0	0	0	0
0													
OTU561	0	0	0	0	0	0	0	0	0	0	1	0	0
0	0	0	0	0	0	0	0	0	0	0	0	0	64
0	0	1	2	0	0	0	0	0	6	0	0	0	0
0	0	0	0	0	0	0	0	0	0	0	0	0	0
0	0	0	0	0	0	0	0	0	0	0	0	0	0
0	0	0	0	0	0	0	0	0	0	0	0	0	0
0	0	0	0	0	0	0	0	0	0	0	0	0	0
0	0	0	0	0	0	0	0	0	0	0	0	0	0
0													
OTU4188	0	0	0	0	0	0	0	0	0	0	1	0	0
0	0	0	0	0	0	0	0	0	0	0	0	0	0
0	0	0	0	0	0	0	0	0	0	0	0	0	0
0	0	0	0	0	0	0	0	0	0	0	0	0	0
0	0	0	0	0	0	0	0	0	0	0	0	0	0
0	0	0	0	0	0	0	0	0	0	0	0	0	0
0	0	0	0	0	0	0	0	0	0	0	0	0	0
0	0	0	0	0	0	0	0	0	0	0	0	0	0
0													
OTU7711	0	0	0	0	0	0	0	0	0	0	1	0	0
0	0	0	0	0	0	0	0	0	0	0	0	0	0
0	0	0	0	0	0	0	0	0	0	0	0	0	0
0	0	0	0	0	0	0	0	0	0	0	0	0	0
0	0	0	0	0	0	0	0	0	0	0	0	0	0
0	0	0	0	0	0	0	0	0	0	0	0	0	0
0	0	0	0	0	0	0	0	0	0	0	0	0	0
0	0	0	0	0	0	0	0	0	0	0	0	0	0
0													

OTU2469	0	0	0	0	0	0	0	0	0	0	3	0	0
0	0	0	0	0	0	0	0	0	0	0	0	0	0
0	0	0	0	0	0	0	0	0	0	0	0	0	0
0	0	0	0	0	0	0	0	0	0	0	0	0	0
0	0	0	0	0	0	0	0	0	0	0	0	0	0
0	0	0	0	0	0	0	0	0	0	0	0	0	0
0	0	0	0	0	0	0	0	1	0	0	0	0	0
0	0	0	0	0	0	0	0	0	0	0	0	0	0
0													
OTU7708	0	0	0	0	0	0	0	0	0	0	3	0	0
0	0	0	0	0	0	0	0	0	0	0	0	0	0
0	0	0	0	0	0	0	0	0	0	0	0	0	0
0	0	0	0	0	0	0	0	0	0	0	0	0	0
0	0	0	0	0	0	0	1	0	0	0	0	0	0
0	0	0	0	0	0	0	0	0	0	0	0	0	0
0	0	0	0	0	0	0	0	0	0	0	0	0	0
0	0	0	0	0	0	0	0	0	0	0	0	0	0
0													
OTU3688	0	0	0	0	0	0	0	0	0	0	1	0	0
0	0	0	0	0	0	0	0	0	34	0	0	0	0
0	0	6	0	0	0	0	0	0	0	0	0	0	0
0	0	1	1	0	0	0	0	0	0	0	0	0	0
0	0	0	0	0	0	0	0	0	0	0	0	0	0
0	0	0	0	0	0	2	0	0	0	10	0	1	0
0	0	2	0	2	0	0	0	0	0	0	0	0	0
0	0	0	0	0	0	0	0	0	0	0	0	0	0
0													
OTU4196	0	0	0	0	0	0	0	0	0	0	1	0	0
0	0	0	0	0	0	0	0	0	0	0	0	0	0
0	0	0	0	0	0	0	0	0	0	0	0	0	0
0	0	0	0	0	0	0	0	0	0	0	0	0	0
0	0	0	0	0	0	0	0	0	0	0	0	0	0
0	0	0	0	0	0	0	0	0	0	0	0	0	0

0 0 0 0 0 0 0 0 0 0 0 0 0 0
0 0 0 0 0 0 0 0 0 0 0 0 0 0
0
OTU4195 0 0 0 0 0 0 0 0 0 0 1 0 0
0 0 0 0 0 0 0 0 0 0 0 0 0 0
0 0 0 0 0 0 0 0 0 0 0 0 0 0
0 0 0 0 0 0 0 0 0 0 0 0 0 0
0 0 0 0 0 0 0 0 0 0 0 0 0 0
0 0 0 0 0 0 0 0 0 0 0 0 0 0
0 0 0 0 0 0 0 0 0 0 0 0 0 0
0 0 0 0 0 0 0 0 0 0 0 0 0 0
0
OTU12525 0 0 0 0 0 0 0 0 0 0 1 0 0
0 0 0 0 0 0 0 0 0 0 0 0 0 0
0 0 0 0 0 0 0 0 0 0 0 0 0 0
0 0 0 0 0 0 0 0 0 0 0 0 0 0
0 0 0 0 0 0 0 0 0 0 0 0 0 0
0 0 0 0 0 0 0 0 0 0 0 0 0 0
0 0 0 0 0 0 0 0 0 0 0 0 0 0
0 0 0 0 0 0 0 0 0 0 0 0 0 0
0
OTU7710 0 0 0 0 0 0 0 0 0 0 1 0 0
0 0 0 0 0 0 0 0 0 0 0 0 0 0
0 0 0 0 0 0 0 0 0 0 0 0 0 1
0 0 0 0 0 0 1 0 0 0 0 0 0 0
0 0 0 0 0 0 0 0 0 0 0 0 0 0
0 0 0 0 0 0 0 0 0 0 0 0 0 0
0 0 0 0 0 0 0 0 0 0 0 0 0 0
0 0 0 0 0 0 0 0 0 0 0 0 0 0
0
OTU12488 0 0 0 0 0 0 0 0 0 0 2 0 0
0 0 0 0 0 0 0 0 0 0 0 1 0 0
0 0 1 0 0 0 0 1 0 0 0 0 0 0

0 0 0 0 0 0 1 0 0 0 0 0 0 0
0 0 0 0 0 0 0 0 0 0 0 0 0 0
0 0 0 0 0 0 0 0 0 0 0 0 0 0
0 0 1 0 0 0 0 0 0 0 0 0 0 0
0 0 0 0 0 0 0 0 0 0 0 0 0 0
0
OTU4297 0 0 0 0 0 0 0 0 0 0 1 0 1
0 0 0 0 0 0 0 0 0 0 0 0 0 0
0 0 0 0 0 0 0 0 0 0 0 0 0 0
0 0 0 0 0 0 0 0 0 0 0 0 0 0
0 0 0 0 0 0 0 0 0 0 0 0 0 0
0 0 0 0 0 0 0 0 0 0 0 0 0 0
0 0 0 0 0 0 0 0 0 0 0 0 0 0
0 0 0 0 0 0 0 0 0 0 0 0 0 0
0
OTU4383 0 0 0 0 0 0 0 0 0 0 1 0 0
0 0 1 0 1 0 0 0 0 0 0 0 0 0
0 0 0 0 0 0 0 0 0 0 0 0 0 0
0 0 0 0 0 0 0 0 0 0 0 0 0 0
0 0 0 0 0 0 0 0 0 0 0 0 0 0
0 0 0 0 0 0 0 0 0 0 0 0 0 0
0 0 0 0 0 0 0 0 0 0 0 0 1 0
0 0 0 0 0 1 0 0 0 0 0 0 0 0
0
OTU15442 0 0 0 0 0 0 0 0 0 0 1 0 0
0 0 0 0 0 0 0 0 0 1 0 1 0 0
0 1 0 0 0 0 1 0 0 0 0 0 0 0
0 0 0 0 0 0 0 0 0 0 0 0 0 0
0 0 0 0 0 0 0 0 0 0 0 0 0 0
0 0 0 0 0 0 0 0 0 0 0 0 0 0
0 0 0 0 0 0 0 0 0 0 0 0 0 0
0 0 0 0 0 0 0 0 0 0 0 0 0 0
0

OTU5568	0	0	0	0	0	0	0	0	0	0	1	0	0
0	0	0	0	0	1	0	0	0	0	0	0	0	0
0	0	0	0	0	0	0	0	0	0	0	0	0	0
0	2	0	0	0	0	1	0	0	0	0	0	0	0
0	0	0	0	0	0	0	0	0	0	0	0	0	0
0	0	0	0	0	0	0	0	0	0	0	0	0	0
0	0	0	0	0	0	0	0	0	0	0	0	0	0
0	0	0	0	0	0	0	0	0	0	0	2	0	0
0													
OTU476	0	0	0	0	0	0	0	0	0	0	2	0	0
0	0	0	0	0	0	0	0	0	1	0	0	0	39
0	0	1	7	0	0	0	0	0	8	0	0	0	0
0	0	0	0	0	0	0	0	0	0	0	0	0	0
0	0	0	0	0	0	0	1	0	0	0	0	0	0
0	0	0	0	0	0	0	0	0	0	0	0	0	0
0	0	0	0	0	0	0	0	0	0	0	0	0	0
0	0	0	0	0	0	0	0	0	0	0	0	0	0
0													
OTU7917	0	0	0	0	0	0	0	0	0	0	1	0	0
0	0	1	0	0	0	0	0	0	0	0	0	0	0
0	0	0	0	0	0	0	0	0	0	0	0	0	0
0	0	0	0	0	0	0	0	0	0	0	0	0	0
0	0	0	0	0	0	0	0	0	0	0	0	0	0
0	0	0	0	0	0	0	0	0	0	0	0	0	0
0	0	0	0	0	0	0	0	0	0	0	0	0	0
0	0	0	0	0	0	0	0	0	0	0	0	0	0
0													
OTU11307	0	0	0	0	0	0	0	0	0	0	1	0	0
0	0	0	0	0	0	0	0	0	0	0	0	0	0
0	0	0	0	0	0	0	0	0	0	0	0	0	0
0	0	0	0	0	0	0	0	0	0	0	0	0	0
0	0	0	0	0	0	0	0	0	0	0	0	0	0
0	0	0	0	0	0	0	0	0	0	0	0	0	0

0	0	0	0	0	0	0	0	0	0	0	0	0	0
0	0	0	0	0	1	0	0	0	0	0	0	0	0
0													
OTU4194	0	0	0	0	0	0	0	0	0	0	1	0	0
0	0	0	0	0	0	0	0	0	0	0	0	0	0
0	0	0	0	0	0	0	0	0	0	0	0	0	0
0	0	0	0	0	0	0	0	0	0	0	0	0	0
0	0	0	0	0	0	0	0	0	0	0	0	0	0
0	0	0	0	0	0	0	0	0	0	0	0	0	0
0	0	0	0	0	0	0	0	0	0	0	0	0	0
0	0	0	0	0	0	0	0	0	0	0	0	0	0
0													
OTU7717	0	0	0	0	0	0	0	0	0	0	1	0	0
0	0	0	0	0	0	0	0	0	0	0	0	0	0
0	0	0	0	0	0	0	0	0	0	0	0	0	0
0	0	0	0	0	0	0	0	0	0	0	0	0	0
0	0	0	0	0	0	0	0	0	0	0	0	0	0
0	0	0	0	0	0	0	0	0	0	0	0	0	0
0	0	0	0	0	0	0	0	0	0	0	0	0	0
0	0	0	0	0	0	0	0	0	0	0	0	0	0
0													
OTU4193	0	0	0	0	0	0	0	0	0	0	1	0	0
0	0	0	0	0	0	0	0	0	0	0	0	0	0
0	0	0	0	0	0	0	0	0	0	0	0	0	0
0	0	0	0	0	0	0	0	0	0	0	0	0	0
0	0	0	0	0	0	0	0	0	0	0	0	0	0
0	0	0	0	0	0	0	0	0	0	0	0	0	0
0	0	0	0	0	0	0	0	0	0	0	0	0	0
0	0	0	0	0	0	0	0	0	0	0	0	0	0
0													
OTU7716	0	0	0	0	0	0	0	0	0	0	1	0	0
0	0	0	0	0	0	0	0	0	0	0	0	0	0
0	0	0	0	0	0	0	0	0	0	0	0	0	0

0 0 0 0 0 0 0 0 0 0 0 0 0 0
0 0 0 0 0 0 0 0 0 0 0 0 0 0
0 0 0 0 0 0 0 0 0 0 0 0 0 0
0 0 0 0 0 0 0 0 0 0 0 0 0 0
0 0 0 0 0 0 0 0 0 0 0 0 0 0
0
OTU12524 0 0 0 0 0 0 0 0 0 0 1 0 0
0 0 0 0 0 0 0 0 0 0 0 0 0 0
0 0 0 0 0 0 0 0 0 0 0 0 0 0
0 0 0 0 0 0 0 0 0 0 0 0 0 0
0 0 0 0 0 0 0 0 0 0 0 0 0 0
0 0 0 0 0 0 0 0 0 0 0 0 0 0
0 0 0 0 0 0 0 0 0 0 0 0 0 0
0 0 0 0 0 0 0 0 0 0 0 0 0 0
0
OTU4217 0 0 0 0 0 0 0 0 0 0 3 0 1
0 0 0 0 0 0 0 0 0 0 0 0 0 0
0 0 0 0 0 0 0 0 0 0 0 0 0 0
0 0 0 0 0 0 0 0 0 0 0 0 0 0
0 0 0 0 0 0 0 1 0 0 0 0 0 0
0 0 0 0 0 0 0 0 0 0 0 0 0 0
0 0 0 0 0 0 0 0 0 0 0 0 0 0
0 0 0 0 0 0 0 0 0 0 0 0 0 0
0
OTU7718 0 0 0 0 0 0 0 0 0 0 1 0 0
0 0 0 0 0 0 0 0 0 0 0 0 0 0
0 0 0 0 0 0 0 0 0 0 0 0 0 0
0 0 0 0 0 0 0 0 0 0 0 0 0 0
0 0 0 0 0 0 0 0 0 0 0 0 0 0
0 0 0 0 0 0 0 0 0 0 0 0 0 0
0 0 0 0 0 0 0 0 0 0 0 0 0 0
0 0 0 0 0 0 0 0 0 0 0 0 0 0
0

OTU15623	0	0	0	0	0	0	0	0	0	0	1	0	0
0	0	0	0	0	0	0	0	0	0	0	0	0	0
0	0	0	0	0	0	0	0	0	0	0	0	0	0
0	0	0	0	0	0	0	0	0	0	0	0	0	0
0	0	0	0	0	0	0	0	0	0	0	0	0	0
0	0	0	0	0	0	0	0	0	0	0	0	0	0
0	0	0	0	0	0	0	0	0	0	0	0	0	0
0	0	0	0	0	0	0	0	0	0	0	0	0	0
0													
OTU12522	0	0	0	0	0	0	0	0	0	0	1	0	0
0	0	0	0	0	0	0	0	0	0	0	0	0	0
0	0	0	0	0	0	0	0	0	0	0	0	0	0
0	0	0	0	0	0	0	0	0	0	0	0	0	0
0	0	0	0	0	0	0	0	0	0	0	0	0	0
0	0	0	0	0	0	0	0	0	0	0	0	0	0
0	0	0	0	0	0	0	0	0	0	0	0	0	0
0	0	0	0	0	0	0	0	0	0	0	0	0	0
0													
OTU2577	0	0	0	0	0	0	0	0	0	0	1	1	1
1	0	0	0	0	0	2	0	0	0	0	0	0	0
0	0	0	0	0	0	0	2	0	1	0	0	0	0
0	0	0	1	0	0	2	0	0	0	0	0	0	1
0	0	0	0	0	0	0	0	0	0	0	0	0	0
0	0	0	2	0	0	0	0	0	0	5	0	1	0
0	0	0	0	0	0	3	0	0	1	3	0	0	0
0	0	0	0	0	0	0	0	0	0	0	2	0	0
0													
OTU12523	0	0	0	0	0	0	0	0	0	0	1	0	0
0	0	0	0	0	0	0	0	0	0	0	0	0	0
0	0	0	0	0	0	0	0	0	0	0	0	0	0
0	0	0	0	0	0	0	0	0	0	0	0	0	0
0	0	0	0	0	0	0	0	0	0	0	0	0	0
0	0	0	0	0	0	0	0	0	0	0	0	0	0

0	0	0	0	0	0	0	0	0	0	0	0	0	0
0	0	0	0	0	0	0	0	0	0	0	0	0	0
0													
OTU11410	0	0	0	0	0	0	0	0	0	0	1	0	0
0	0	0	0	0	0	0	0	0	0	0	0	0	0
0	0	0	0	0	0	0	0	0	0	0	0	0	0
0	0	0	0	0	0	0	0	0	0	0	0	0	0
0	0	0	0	0	0	0	0	0	0	0	0	0	0
0	0	0	0	0	0	0	0	0	0	0	0	0	0
0	0	0	0	0	0	0	0	0	0	0	0	0	0
1	0	0	0	0	0	0	0	0	0	0	0	0	0
0													
OTU1657	0	0	0	0	0	0	0	0	0	0	1	0	0
0	0	0	0	0	0	0	0	0	0	0	0	0	0
0	0	0	0	0	0	0	0	0	0	0	0	0	0
0	0	0	0	0	0	0	0	0	0	0	0	0	0
0	0	0	0	0	0	0	0	0	0	0	0	0	0
0	0	0	0	0	0	0	0	0	1	0	0	0	0
0	0	0	0	0	0	0	0	0	0	0	0	0	0
0	0	0	0	0	0	0	0	0	0	0	0	0	0
0													
OTU15625	0	0	0	0	0	0	0	0	0	0	1	0	0
0	0	0	0	0	0	0	0	0	0	0	0	0	0
0	0	0	0	0	0	0	0	0	0	0	0	0	0
0	0	0	0	0	0	0	0	0	0	0	0	0	0
0	0	0	0	0	0	0	0	0	0	0	0	0	0
0	0	0	0	0	0	0	0	0	0	0	0	0	0
0	0	0	0	0	0	0	0	0	0	0	0	0	0
0	0	0	0	0	0	0	0	0	0	0	0	0	0
0													
OTU7713	0	0	0	0	0	0	0	0	0	0	2	0	0
0	0	0	0	0	0	0	0	0	0	0	0	0	0
0	0	0	0	0	0	0	0	0	0	0	0	0	0

0 0 0 0 0 0 0 0 0 0 0 0 0 0
0 0 0 0 0 0 0 0 0 0 0 0 0 0
0 0 0 0 0 0 0 0 0 0 0 0 0 0
0 0 0 0 0 0 0 0 0 0 0 0 0 0
0 0 0 0 0 0 0 0 0 0 0 0 0 0
0
OTU5171 0 0 0 0 0 0 0 0 0 0 1 0 0
0 0 0 0 0 0 0 0 0 0 0 0 0 0
0 0 0 0 0 0 0 0 0 0 0 1 0 0
0 0 0 0 0 0 0 0 0 0 0 0 0 0
0 0 0 0 0 0 0 0 0 0 0 0 0 0
0 0 0 0 0 0 0 0 0 0 0 0 0 0
0 0 0 0 0 0 0 0 0 0 0 0 0 0
0 0 0 0 0 0 0 0 0 0 0 0 0 0
0
OTU7714 0 0 0 0 0 0 0 0 0 0 1 0 0
0 0 0 0 0 0 0 0 0 0 0 0 0 0
0 0 0 0 0 0 0 0 0 0 0 0 0 0
0 0 0 0 0 0 0 0 0 0 0 0 0 0
0 0 0 0 0 0 0 0 0 0 0 0 0 0
0 0 0 0 0 0 0 0 0 0 0 0 0 0
0 0 0 0 0 0 0 0 0 0 0 0 0 0
0 0 0 0 0 0 0 0 0 0 0 0 0 0
0
OTU4228 0 0 0 0 0 0 0 0 0 0 1 0 0
0 0 1 0 0 0 0 1 0 0 0 0 0 0
0 0 0 0 0 0 0 0 0 0 0 0 0 0
0 0 0 0 0 0 0 0 0 0 0 0 0 0
0 1 0 0 0 0 0 0 0 0 0 0 0 0
0 0 0 0 0 0 0 0 0 0 0 0 0 0
0 0 0 0 0 0 0 0 0 0 0 0 0 0
0 0 0 0 0 0 0 0 0 0 0 0 0 0
0

OTU1011	0	0	0	0	0	0	0	0	0	0	1	0	0
0	0	0	0	0	0	0	0	0	0	0	0	0	0
0	0	0	0	0	0	0	0	0	0	0	0	0	0
0	0	0	0	0	0	0	0	0	0	0	0	0	0
0	0	0	0	0	0	0	0	0	0	0	0	0	0
0	0	0	0	0	0	0	0	0	0	0	1	0	1
0	0	0	0	0	0	0	0	0	2	0	0	0	0
0	0	0	0	0	0	0	0	0	0	0	0	0	0
0													
OTU4225	0	0	0	0	0	0	0	0	0	0	1	0	0
0	0	0	0	0	0	0	0	0	0	0	0	0	0
0	0	0	0	0	0	0	0	0	0	0	0	0	0
0	0	0	0	0	0	0	0	0	0	0	0	0	0
0	0	0	0	0	0	0	0	0	0	0	0	0	0
0	0	0	0	0	0	0	0	0	0	0	0	0	0
0	0	0	0	0	0	0	0	0	0	0	0	0	0
0	0	0	0	0	0	0	0	0	0	0	0	0	0
0													
OTU12529	0	0	0	0	0	0	0	0	0	0	1	0	0
0	0	0	0	0	0	0	0	0	0	0	0	0	0
0	0	0	0	0	0	0	0	0	0	0	0	0	0
0	0	0	0	0	0	0	0	0	0	0	0	0	0
0	0	0	0	0	0	0	0	0	0	0	0	0	0
0	0	0	0	0	0	0	0	0	0	0	0	0	0
0	0	0	0	0	0	0	0	0	0	0	0	0	0
0	0	0	0	0	0	0	0	0	0	0	0	0	0
0													
OTU12336	0	0	0	0	0	0	0	0	0	0	1	0	2
1	0	0	0	0	0	0	0	0	0	0	0	0	0
0	0	0	0	0	0	0	0	0	0	0	0	0	0
0	0	0	0	0	0	2	0	0	0	0	0	0	0
0	0	0	0	0	0	0	0	0	0	0	0	0	0
0	0	0	0	0	0	0	0	1	0	0	0	0	0

0	0	0	0	0	0	0	0	0	0	0	0	0	0
0	0	0	0	0	0	0	0	0	0	0	0	0	0
0													
OTU12528	0	0	0	0	0	0	0	0	0	0	1	0	0
0	0	0	0	0	0	0	0	0	0	0	0	0	0
0	0	0	0	0	0	0	0	0	0	0	0	0	0
0	0	0	0	0	0	0	0	0	0	0	0	0	0
0	0	0	0	0	0	0	0	0	0	0	0	0	0
0	0	0	0	0	0	0	0	0	0	0	0	0	0
0	0	0	0	0	0	0	0	0	0	0	0	0	0
0	0	0	0	0	0	0	0	0	0	0	0	0	0
0													
OTU653	0	0	0	0	0	0	0	0	0	0	1	0	0
0	0	1	0	0	0	0	0	0	0	0	0	0	19
0	0	0	0	0	0	0	0	0	0	0	0	0	0
0	0	0	0	0	0	0	0	0	0	0	0	0	0
0	0	0	0	0	0	0	0	0	0	0	0	0	0
0	0	0	0	0	0	0	0	0	0	0	0	0	0
0	0	0	0	0	0	0	0	0	0	0	0	0	0
0	0	0	0	0	0	0	0	0	0	0	0	0	0
0													
OTU7745	0	0	0	0	0	0	0	0	0	0	2	0	0
0	0	0	1	0	6	0	0	0	0	0	0	0	0
0	0	2	0	0	0	0	0	0	0	0	0	0	0
0	0	1	0	2	0	0	0	0	0	0	0	0	0
0	0	0	0	0	1	0	0	0	0	0	0	0	0
0	0	0	0	0	0	0	0	0	0	0	0	0	0
0	0	0	0	0	0	0	0	1	0	0	0	0	0
0	0	0	0	0	0	0	0	0	0	0	0	0	0
0													
OTU355	0	0	0	0	0	0	0	0	0	0	1	0	3
0	0	0	16	1	0	1	0	4	0	0	0	2	0
2	0	0	0	0	0	1	2	0	0	0	0	0	0

	0	0	2	3	0	0	0	0	1	0	0	1	20	0
	89	4	0	5	3	1	0	2	0	1	2	1	1	0
	3	0	0	2	0	2	1	2	2	0	0	0	0	1
	0	3	5	0	1	0	2	2	1	0	0	0	0	4
	0	0	0	0	0	0	0	0	0	0	1	6	3	0
	0													
OTU15620		0	0	0	0	0	0	0	0	0	0	1	0	0
	0	0	0	0	0	0	0	0	0	0	0	0	0	0
	0	0	0	0	0	0	0	0	0	0	0	0	0	0
	0	0	0	0	0	0	0	0	0	0	0	0	0	0
	0	0	0	0	0	0	0	0	0	0	0	0	0	0
	0	0	0	0	0	0	0	0	0	0	0	0	0	0
	0	0	0	0	0	0	0	0	0	0	0	0	0	0
	0	0	0	0	0	0	0	0	0	0	0	0	0	0
	0													
OTU897		0	0	0	0	0	0	0	0	0	0	1	0	0
	0	0	0	0	0	0	0	0	0	0	0	0	0	0
	0	0	0	0	0	0	0	0	0	0	0	0	0	0
	0	0	0	0	0	0	0	0	0	0	0	0	0	0
	0	0	0	0	0	0	0	0	0	0	0	0	0	0
	0	3	0	0	0	0	0	0	0	1	0	0	0	0
	0	0	0	0	0	0	0	0	0	0	0	4	0	0
	0	0	0	0	0	1	0	2	0	0	0	0	0	0
	0													
OTU2461		0	0	0	0	0	0	0	0	0	0	1	0	3
	0	0	0	0	0	0	0	0	0	0	0	0	0	0
	0	0	0	0	0	0	0	0	0	0	0	0	0	0
	0	0	0	0	0	0	0	0	0	0	0	0	0	0
	0	0	0	0	0	0	0	0	0	0	0	0	0	0
	0	1	0	0	0	0	0	0	0	0	0	0	0	0
	0	0	0	0	0	0	0	0	15	0	0	0	0	0
	0	1	0	0	0	0	5	1	0	0	0	0	0	0
	0													

OTU15621	0	0	0	0	0	0	0	0	0	0	1	0	0
0	0	0	0	0	0	0	0	0	0	0	0	0	0
0	0	0	0	0	0	0	0	0	0	0	0	0	0
0	0	0	0	0	0	0	0	0	0	0	0	0	0
0	0	0	0	0	0	0	0	0	0	0	0	0	0
0	0	0	0	0	0	0	0	0	0	0	0	0	0
0	0	0	0	0	0	0	0	0	0	0	0	0	0
0	0	0	0	0	0	0	0	0	0	0	0	0	0
0													
OTU15622	0	0	0	0	0	0	0	0	0	0	1	0	0
0	0	0	0	0	0	0	0	0	0	0	0	0	0
0	0	0	0	0	0	0	0	0	0	0	0	0	0
0	0	0	0	0	0	0	0	0	0	0	0	0	0
0	0	0	0	0	0	0	0	0	0	0	0	0	0
0	0	0	0	0	0	0	0	0	0	0	0	0	0
0	0	0	0	0	0	0	0	0	0	0	0	0	0
0	0	0	0	0	0	0	0	0	0	0	0	0	0
0													
OTU7746	0	0	0	0	0	0	0	0	0	0	1	0	3
1	0	0	0	2	0	0	0	0	0	0	0	0	0
0	0	0	0	0	0	0	0	0	0	0	0	0	0
0	0	0	0	0	0	2	0	0	0	0	0	0	0
0	0	1	0	0	0	0	0	0	0	0	0	0	0
0	0	0	0	0	0	0	0	0	0	0	0	0	0
0	0	0	0	0	0	0	0	1	0	1	0	0	1
0	0	0	0	0	0	0	0	0	0	0	0	0	0
0													
OTU4227	0	0	0	0	0	0	0	0	0	0	1	0	0
0	0	0	0	0	0	0	0	0	0	0	0	0	0
0	0	0	0	0	0	0	0	0	0	0	0	0	0
0	0	0	0	0	0	0	0	0	0	0	0	0	0
0	0	0	0	0	0	0	0	0	0	0	0	0	0
0	0	0	0	0	0	0	0	0	0	0	0	0	0

0	0	0	0	0	0	0	0	0	0	0	0	0	0
0	0	0	0	0	0	0	0	0	0	0	0	0	0
0													
OTU15930	0	0	0	0	0	0	0	0	0	0	1	0	0
0	0	0	0	0	1	0	0	0	0	0	0	0	0
0	0	0	0	0	0	0	0	0	0	0	0	0	0
0	0	0	0	0	0	0	0	0	0	0	0	2	0
0	0	0	0	0	0	0	0	0	0	0	0	0	0
0	0	0	0	0	0	0	0	0	0	0	0	0	0
0	0	0	0	0	0	0	0	0	0	0	0	0	3
0	0	0	0	0	0	0	0	0	0	0	0	0	0
0													
OTU7747	0	0	0	0	0	0	0	0	0	0	1	0	0
0	0	0	0	0	0	0	0	0	0	0	0	0	0
0	0	0	0	0	0	0	0	0	0	0	0	0	0
0	0	0	0	0	0	0	0	0	0	0	0	0	0
0	0	0	0	0	0	0	0	0	0	0	0	0	0
0	0	0	0	0	0	0	0	0	0	0	0	0	0
0	0	0	0	0	0	0	0	0	0	0	0	0	0
0	0	0	0	0	0	0	0	0	0	0	0	0	0
0													
OTU4231	0	0	0	0	0	0	0	0	0	0	1	0	0
0	0	0	0	0	0	0	0	0	0	0	0	0	0
0	0	0	0	0	0	0	0	0	0	0	0	0	0
0	0	0	0	0	0	0	0	0	0	0	0	0	0
0	0	0	0	0	0	0	0	0	0	0	0	0	0
0	0	0	0	0	0	0	0	0	0	0	0	0	0
0	0	0	0	0	0	0	0	0	0	0	0	0	0
0	0	0	0	0	0	0	0	0	0	0	0	0	0
0													
OTU6243	0	0	0	0	0	0	0	0	0	0	1	0	0
0	0	0	0	0	0	0	0	0	0	0	0	0	0
0	0	0	0	0	0	0	0	0	0	0	0	0	0

0	0	0	0	1	0	0	1	0	0	0	0	0	0
0	0	0	0	0	0	0	0	0	0	0	0	0	0
0	0	0	0	0	0	0	0	0	0	0	0	0	0
0	0	0	0	0	0	0	0	0	0	0	0	0	0
0	0	1	0	0	0	0	0	0	0	0	0	0	0
0													
OTU718	0	0	0	0	0	0	0	0	0	0	1	0	0
0	0	0	0	0	0	0	0	0	0	0	0	0	19
0	0	1	0	0	1	0	0	0	6	0	0	0	0
0	0	0	0	0	0	0	0	0	0	0	0	0	0
0	0	0	0	0	0	0	0	0	0	0	0	0	0
0	0	0	0	0	0	0	0	0	0	0	0	0	0
0	0	0	0	0	0	0	0	0	0	0	0	0	0
0	0	0	0	0	0	0	0	0	0	0	0	0	0
0													
OTU12526	0	0	0	0	0	0	0	0	0	0	1	0	0
0	0	0	0	0	0	0	0	0	0	0	0	0	0
0	0	0	0	0	0	0	0	0	0	0	0	0	0
0	0	0	0	0	0	0	0	0	0	0	0	0	0
0	0	0	0	0	0	0	0	0	0	0	0	0	0
0	0	0	0	0	0	0	0	0	0	0	0	0	0
0	0	0	0	0	0	0	0	0	0	0	0	0	0
0	0	0	0	0	0	0	0	0	0	0	0	0	0
0													
OTU8817	0	0	0	0	0	0	0	0	0	0	1	0	0
0	0	0	0	0	0	0	0	0	0	0	0	0	0
0	0	0	0	0	0	0	1	0	0	0	0	0	0
0	0	0	0	0	0	0	0	0	0	0	0	0	0
0	0	0	0	0	0	0	0	0	0	0	0	0	0
0	0	0	0	0	0	0	0	0	0	0	0	0	0
0	0	0	0	0	0	0	0	0	0	0	0	0	0
0	0	0	0	0	0	0	0	0	0	0	0	0	0
0													

OTU4234	0	0	0	0	0	0	0	0	0	0	1	0	0
0	0	0	0	0	0	0	0	0	0	0	0	0	0
0	0	0	0	0	0	0	0	0	0	0	0	0	0
0	0	0	0	0	0	0	0	0	0	0	0	0	0
0	0	0	0	0	0	0	0	0	0	0	0	0	0
0	0	0	0	0	0	0	0	0	0	0	0	0	0
0	0	0	0	0	0	0	0	0	0	0	0	0	0
0	0	0	0	0	0	0	0	0	0	0	0	0	0
0													
OTU7742	0	0	0	0	0	0	0	0	0	0	1	0	0
0	0	0	0	0	0	0	0	0	0	0	0	0	0
0	0	0	0	0	0	0	0	0	0	0	0	0	0
0	0	0	0	0	0	0	0	0	0	0	0	0	0
0	0	0	0	0	0	0	0	0	0	0	0	0	0
0	0	0	0	0	0	0	0	0	0	0	0	0	0
0	0	0	0	0	0	0	0	0	0	0	0	0	0
0	0	0	0	0	0	0	0	0	0	0	0	0	0
0													
OTU7741	0	0	0	0	0	0	0	0	0	0	1	0	0
0	0	0	0	0	0	0	0	0	0	0	0	0	0
0	0	0	0	0	0	0	0	0	0	0	0	0	0
0	0	0	0	0	0	0	0	0	0	0	0	0	0
0	0	0	0	0	0	0	0	0	0	0	0	0	0
0	0	0	0	0	0	0	0	0	0	0	0	0	0
0	0	0	0	0	0	0	0	0	0	0	0	0	0
0	0	0	0	0	0	0	0	0	0	0	0	0	0
0													
OTU7743	0	0	0	0	0	0	0	0	0	0	1	0	0
0	0	0	0	0	0	0	0	0	0	0	0	0	0
0	0	0	0	0	0	0	0	0	0	0	0	0	0
0	0	0	0	0	0	0	0	0	0	0	0	0	0
0	0	0	0	0	0	0	0	0	0	0	0	0	0
0	0	0	0	0	0	0	0	0	0	0	0	0	0

0 0 0 0 0 0 0 0 0 0 0 0 0 0
0 0 0 0 0 0 0 0 0 0 0 0 0 0
0
OTU4233 0 0 0 0 0 0 0 0 0 0 1 0 0
0 0 0 0 0 0 0 0 0 0 0 0 0 0
0 0 0 0 0 0 0 0 0 0 0 0 0 0
0 0 0 0 0 0 0 0 0 0 0 0 0 0
0 0 0 0 0 0 0 0 0 0 0 0 0 0
0 0 0 0 0 0 0 0 0 0 0 0 0 0
0 0 0 0 0 0 0 0 0 0 0 0 0 0
0 0 0 0 0 0 0 0 0 0 0 0 0 0
0
OTU15628 0 0 0 0 0 0 0 0 0 0 1 0 0
0 0 0 0 0 0 0 0 0 0 0 0 0 0
0 0 0 0 0 0 0 0 0 0 0 0 0 0
0 0 0 0 0 0 0 0 0 0 0 0 0 0
0 0 0 0 0 0 0 0 0 0 0 0 0 0
0 0 0 0 0 0 0 0 0 0 0 0 0 0
0 0 0 0 0 0 0 0 0 0 0 0 0 0
0 0 0 0 0 0 0 0 0 0 0 0 0 0
0
OTU4232 0 0 0 0 0 0 0 0 0 0 1 0 0
0 0 0 0 1 0 0 0 0 0 0 0 0 0
2 0 0 0 0 0 0 0 1 0 0 0 0 0
0 1 0 0 0 0 0 0 0 0 0 0 0 0
0 0 1 0 0 0 0 0 0 0 0 1 0 0
0 0 0 0 0 0 0 0 0 0 0 0 0 0
0 0 0 0 2 0 0 0 0 1 0 0 0 0
0 0 0 0 0 0 0 0 0 1 0 0 0 0
0
OTU4101 0 0 0 0 0 0 0 0 0 0 1 0 0
1 0 0 0 0 0 0 0 0 0 0 0 0 0
0 0 0 0 0 0 0 0 0 1 0 0 0 0

0	0	0	0	0	0	0	0	0	0	0	0	0	0
0	0	0	0	0	0	0	0	0	0	0	0	0	0
0	0	0	0	0	0	0	0	0	0	1	0	0	0
0	0	0	0	0	0	0	0	0	0	1	0	0	0
0	0	0	0	0	0	0	0	0	0	0	0	0	0
0													
OTU15627	0	0	0	0	0	0	0	0	0	0	1	0	0
0	0	0	0	0	0	0	0	0	0	0	0	0	0
0	0	0	0	0	0	0	0	0	0	0	0	0	0
0	0	0	0	0	0	0	0	0	0	0	0	0	0
0	0	0	0	0	0	0	0	0	0	0	0	0	0
0	0	0	0	0	0	0	0	0	0	0	0	0	0
0	0	0	0	0	0	0	0	0	0	0	0	0	0
0	0	0	0	0	0	0	0	0	0	0	0	0	0
0													
OTU3555	0	0	0	0	0	0	0	0	0	0	1	0	0
0	0	0	0	0	0	0	0	0	0	0	0	3	0
0	0	0	0	0	0	0	0	0	1	0	0	0	0
0	0	0	0	0	0	0	0	1	0	0	0	0	0
0	0	0	0	0	0	0	0	0	0	0	0	0	0
0	0	0	0	0	0	0	0	0	0	0	0	0	0
0	0	0	0	0	0	0	0	0	0	0	1	0	0
0	0	0	0	0	0	0	0	0	0	0	0	0	0
0													
OTU4229	0	0	0	0	0	0	0	0	0	0	1	0	0
0	0	0	0	0	0	0	0	0	0	0	0	0	0
0	0	0	0	0	0	0	0	0	0	0	0	0	0
0	0	0	0	0	0	0	0	0	0	0	0	0	0
0	0	0	0	0	0	0	0	0	0	0	0	0	0
0	0	0	0	0	0	0	0	0	0	0	0	0	0
0	0	0	0	0	0	0	0	0	0	0	0	0	0
0	0	0	0	0	0	0	0	0	0	0	0	0	0
0													

OTU7744	0	0	0	0	0	0	0	0	0	0	1	0	0
0	0	1	0	0	0	0	0	0	0	0	0	0	0
0	0	0	0	0	0	0	0	0	0	0	0	0	0
0	0	0	0	0	0	0	0	0	0	0	0	0	0
0	0	0	0	0	0	0	0	0	0	0	0	0	0
0	0	0	0	0	0	0	0	0	0	0	0	0	0
0	0	0	0	0	0	0	0	0	0	0	0	0	0
0	0	0	0	0	0	0	0	0	0	0	0	0	0
0													
OTU2485	0	0	0	0	0	0	0	0	0	0	1	0	1
0	0	0	0	0	1	0	0	0	0	0	0	0	0
0	0	0	0	0	0	0	0	0	0	0	0	0	0
0	0	0	0	0	0	1	0	0	0	0	0	0	0
0	0	0	0	0	0	0	0	0	0	0	0	0	0
0	0	0	0	0	0	0	0	0	0	0	0	0	0
0	0	0	0	0	0	0	0	9	0	1	0	0	0
0	0	0	0	0	0	0	0	0	0	0	0	0	0
0													
OTU12485	0	0	0	0	0	0	0	0	0	0	1	0	0
0	0	0	0	0	0	0	0	0	0	0	0	0	0
0	0	0	0	0	0	0	0	0	0	0	0	0	0
0	0	0	0	0	0	0	0	0	0	0	0	0	0
0	0	0	0	0	0	0	0	0	0	0	0	0	0
0	0	0	0	0	0	0	0	0	0	0	0	0	0
0	0	0	0	0	0	0	0	0	0	0	0	0	0
0	0	0	0	0	0	0	0	0	0	0	0	0	0
0													
OTU2115	0	0	0	0	0	0	0	0	0	0	1	1	0
0	1	1	0	2	0	1	0	0	0	0	0	0	0
0	0	0	0	0	0	0	0	0	0	1	0	0	0
0	3	0	0	0	0	1	2	0	0	0	0	0	1
0	2	0	0	0	0	0	0	0	0	0	1	1	0
0	1	0	0	0	0	0	0	1	0	0	0	0	2

0	0	0	0	1	1	0	0	0	1	1	0	0	1
0	0	0	0	0	0	0	1	0	0	0	0	0	1
0													
OTU7750	0	0	0	0	0	0	0	0	0	0	1	0	0
0	0	0	0	0	0	0	0	0	0	0	0	0	0
0	0	0	0	0	0	0	0	0	0	0	0	0	0
0	0	0	0	0	0	0	0	0	0	0	0	0	0
0	0	0	0	0	0	0	0	0	0	0	0	0	0
0	0	0	0	0	0	0	0	0	0	0	0	0	0
0	0	0	0	0	0	0	0	0	0	0	0	0	0
0	0	0	0	0	0	0	0	0	0	0	0	0	0
0													
OTU1354	0	0	0	0	0	0	0	0	0	0	2	0	4
7	0	1	0	3	0	0	1	0	0	0	4	0	0
0	0	9	0	0	1	0	1	0	1	0	6	0	8
0	2	0	2	0	0	2	1	1	0	1	0	0	0
0	0	0	0	0	0	0	0	0	0	0	1	0	0
0	0	0	3	0	0	13	0	1	0	4	0	1	0
20	0	2	0	6	0	1	1	1	0	0	0	0	3
1	0	0	0	0	0	0	0	0	0	4	1	0	0
0													
OTU12486	0	0	0	0	0	0	0	0	0	0	1	0	0
0	0	0	0	0	0	0	0	0	0	0	0	0	0
0	0	0	0	0	0	0	0	0	0	0	0	0	0
0	0	0	0	0	0	0	0	0	0	0	0	0	0
0	0	0	0	0	0	0	0	0	0	0	0	0	0
0	0	0	0	0	0	0	0	0	0	0	0	0	0
0	0	0	0	0	0	0	0	0	0	0	0	0	0
0	0	0	0	0	0	0	0	0	0	0	0	0	0
0													
OTU13657	0	0	0	0	0	0	0	0	0	0	1	0	0
1	0	0	0	0	0	0	0	0	0	0	0	0	0
0	0	1	0	0	2	0	0	0	0	0	1	0	0

0	4	0	0	0	0	1	0	0	0	0	0	0	0
0	0	0	0	0	0	0	0	0	0	0	0	0	0
0	0	0	0	0	0	0	0	1	0	0	0	0	0
0	0	0	0	0	0	0	0	3	0	2	0	0	1
0	0	0	0	0	0	0	0	0	0	0	0	0	0
0													
OTU4215	0	0	0	0	0	0	0	0	0	0	1	0	0
0	0	0	0	0	0	0	0	0	0	0	0	0	0
0	0	0	0	0	0	0	0	0	0	0	0	0	0
0	0	0	0	0	0	0	0	0	0	0	0	0	0
0	0	0	0	0	0	0	0	0	0	0	0	0	0
0	0	0	0	0	0	0	0	0	0	0	0	0	0
0	0	0	0	0	0	0	0	0	0	0	0	0	0
0	0	0	0	0	0	0	0	0	0	0	0	0	0
0													
OTU15626	0	0	0	0	0	0	0	0	0	0	1	0	0
0	0	0	0	0	0	0	0	0	0	0	0	0	0
0	0	0	0	0	0	0	0	0	0	0	0	0	0
0	0	0	0	0	0	0	0	0	0	0	0	0	0
0	0	0	0	0	0	0	0	0	0	0	0	0	0
0	0	0	0	0	0	0	0	0	0	0	0	0	0
0	0	0	0	0	0	0	0	0	0	0	0	0	0
0	0	0	0	0	0	0	0	0	0	0	0	0	0
0													
OTU4081	0	0	0	0	0	0	0	0	0	0	1	0	0
1	0	0	0	0	0	0	0	0	0	0	0	0	0
0	0	0	0	0	0	0	0	0	0	0	1	0	0
0	0	0	0	0	0	0	0	0	0	0	0	0	0
0	0	0	0	0	0	0	0	0	0	0	0	0	0
0	0	0	0	0	0	0	0	0	0	0	0	0	0
0	0	0	0	0	0	0	0	0	0	0	0	0	0
0	0	0	0	0	0	0	0	0	0	0	0	0	0
0													

OTU4219	0	0	0	0	0	0	0	0	0	0	1	0	0
0	0	0	0	0	0	0	0	0	0	0	0	0	0
0	0	0	0	0	0	0	0	0	0	0	0	0	0
0	0	0	0	0	0	0	0	0	0	0	0	0	0
0	0	0	0	0	0	0	0	0	0	0	0	0	0
0	0	0	0	0	0	0	0	0	0	0	0	0	0
0	0	0	0	0	0	0	0	0	0	0	0	0	0
0	0	0	0	0	0	0	0	0	0	0	0	0	0
0													
OTU4218	0	0	0	0	0	0	0	0	0	0	1	0	0
0	0	0	0	0	0	0	0	0	0	0	0	0	0
0	0	0	0	0	0	0	0	0	0	0	0	0	0
0	0	0	0	0	0	0	0	0	0	0	0	0	0
0	0	0	0	0	0	0	0	0	0	0	0	0	0
0	0	0	0	0	0	0	0	0	0	0	0	0	0
0	0	0	0	0	0	0	0	0	0	0	0	0	0
0	0	0	0	0	0	0	0	0	0	0	0	0	0
0													
OTU15616	0	0	0	0	0	0	0	0	0	0	1	0	0
0	0	0	0	0	0	0	0	0	0	0	0	0	0
0	0	0	0	0	0	0	0	0	0	0	0	0	0
0	0	0	0	0	0	0	0	0	0	0	0	0	0
0	0	0	0	0	0	0	0	0	0	0	0	0	0
0	0	0	0	0	0	0	0	0	0	0	0	0	0
0	0	0	0	0	0	0	0	0	0	0	0	0	0
0	0	0	0	0	0	0	0	0	0	0	0	0	0
0													
OTU917	0	0	0	0	0	0	0	0	0	0	1	0	0
1	0	0	0	0	5	0	0	0	0	0	0	0	0
0	0	0	0	0	0	0	0	0	0	0	0	0	0
0	0	0	0	0	0	0	0	0	0	0	0	0	0
0	0	0	0	0	0	0	0	0	0	0	0	0	0
0	0	0	0	0	0	0	0	0	0	0	0	0	0

0	0	0	0	0	0	0	0	0	0	0	0	0	0
0	0	0	0	0	0	0	0	0	0	0	0	0	0
0													
OTU15617	0	0	0	0	0	0	0	0	0	0	1	0	0
0	0	0	0	0	0	0	0	0	0	0	0	0	0
0	0	0	0	0	0	0	0	0	0	0	0	0	0
0	0	0	0	0	0	0	0	0	0	0	0	0	0
0	0	0	0	0	0	0	0	0	0	0	0	0	0
0	0	0	0	0	0	0	0	0	0	0	0	0	0
0	0	0	0	0	0	0	0	0	0	0	0	0	0
0	0	0	0	0	0	0	0	0	0	0	0	0	0
0													
OTU7749	0	0	0	0	0	0	0	0	0	0	1	0	0
0	0	0	0	0	0	0	0	0	0	0	0	0	0
0	0	0	0	0	0	0	0	0	0	0	0	0	0
0	0	0	0	0	0	0	0	0	0	0	0	0	0
0	0	0	0	0	0	0	0	0	0	0	0	0	0
0	0	0	0	0	0	0	0	0	0	0	0	0	0
0	0	0	0	0	0	0	0	0	0	0	0	0	0
0	0	0	0	0	0	0	0	0	0	0	0	0	0
0													
OTU15615	0	0	0	0	0	0	0	0	0	0	1	0	0
0	0	0	0	0	0	0	0	0	0	0	0	0	0
0	0	0	0	0	0	0	0	0	0	0	0	0	0
0	0	0	0	0	0	0	0	0	0	0	0	0	0
0	0	0	0	0	0	0	0	0	0	0	0	0	0
0	0	0	0	0	0	0	0	0	0	0	0	0	0
0	0	0	0	0	0	0	0	0	0	0	0	0	0
0	0	0	0	0	0	0	0	0	0	0	0	0	0
0													
OTU4211	0	0	0	0	0	0	0	0	0	0	1	0	0
0	0	0	0	0	0	0	0	0	0	0	0	0	0
0	0	0	0	0	0	0	0	0	0	0	0	0	0

0 0 0 0 0 0 0 0 0 0 0 0 0 0
0 0 0 0 0 0 0 0 0 0 0 0 0 0
0 0 0 0 0 0 0 0 0 0 0 0 0 0
0 0 0 0 0 0 0 0 0 0 0 0 0 0
0 0 0 0 0 0 0 0 0 0 0 0 0 0
0
OTU10371 0 0 0 0 0 0 0 0 0 0 1 0 0
0 0 0 0 0 0 0 0 0 0 0 0 0 0
0 0 0 0 0 0 0 0 0 0 0 0 0 0
0 0 0 0 0 0 0 0 0 0 0 0 0 0
0 0 0 0 0 0 0 0 0 0 0 0 0 0
0 0 0 0 1 0 0 0 0 0 0 0 0 1
0 0 0 0 0 0 0 0 0 0 0 0 0 0
0 0 0 0 0 0 0 0 0 0 0 0 0 0
0
OTU7733 0 0 0 0 0 0 0 0 0 0 1 0 0
0 0 0 0 0 0 0 0 0 0 0 0 0 0
0 0 0 0 0 0 0 0 0 0 0 0 0 0
0 0 0 0 0 0 0 0 0 0 0 0 0 0
0 0 0 0 0 0 0 0 0 0 0 0 0 0
0 0 0 0 0 0 0 0 0 0 0 0 0 0
0 0 0 0 0 0 0 0 0 0 0 0 0 0
0 0 0 0 0 0 0 0 0 0 0 0 0 0
0
OTU12484 0 0 0 0 0 0 0 0 0 0 1 0 0
0 0 0 0 0 0 0 0 0 0 0 0 0 0
0 0 0 0 0 0 0 0 0 0 0 0 0 0
0 0 0 0 0 0 0 0 0 0 0 0 0 0
0 0 0 0 0 0 0 0 0 0 0 0 0 0
0 0 0 0 0 0 0 0 0 0 0 0 0 0
0 0 0 0 0 0 0 0 0 0 0 0 0 0
0 0 0 0 0 0 0 0 0 0 0 0 0 0
0

OTU4213	0	0	0	0	0	0	0	0	0	0	1	0	0
0	0	0	0	0	0	0	0	0	0	0	0	0	0
0	0	0	0	0	0	0	0	0	0	0	0	0	0
0	0	0	0	0	0	0	0	0	0	0	0	0	0
0	0	0	0	0	0	0	0	0	0	0	0	0	0
0	0	0	0	0	0	0	0	0	0	0	0	0	0
0	0	0	0	0	0	0	0	0	0	0	0	0	0
0	0	0	0	0	0	0	0	0	0	0	0	0	0
0													
OTU4212	0	0	0	0	0	0	0	0	0	0	1	0	0
0	0	0	0	0	0	0	0	0	0	0	0	0	0
0	0	0	0	0	0	0	0	0	0	0	0	0	0
0	0	0	0	0	0	0	0	0	0	0	0	0	0
0	0	0	0	0	0	0	0	0	0	0	0	0	0
0	0	0	0	0	0	0	0	0	0	0	0	0	0
0	0	0	0	0	0	0	0	0	0	0	0	0	1
0	0	0	0	0	0	0	0	0	0	0	0	0	0
0													
OTU7732	0	0	0	0	0	0	0	0	0	0	1	0	0
0	0	0	0	0	0	0	0	0	0	0	0	0	0
0	0	0	0	0	0	0	0	0	0	0	0	0	0
0	0	0	0	0	0	0	0	0	0	0	0	0	0
0	0	0	0	0	0	0	0	0	0	0	0	0	0
0	0	0	0	0	0	0	0	0	0	0	0	0	0
0	0	0	0	0	0	0	0	0	0	0	0	0	0
0	0	0	0	0	0	0	0	0	0	0	0	0	0
0													
OTU12483	0	0	0	0	0	0	0	0	0	0	1	0	0
0	0	0	0	0	0	0	0	0	0	0	0	0	0
0	0	0	0	0	0	0	0	0	0	0	0	0	0
0	0	0	0	0	0	0	0	0	0	0	0	0	0
0	0	0	0	0	0	0	0	0	0	0	0	0	0
0	0	0	0	0	0	0	0	0	0	0	0	0	0

0	0	0	0	0	0	0	0	0	0	0	0	0	0
0	0	0	0	0	0	0	0	0	0	0	0	0	0
0													
OTU2616	0	0	0	0	0	0	0	0	0	0	1	0	0
0	0	0	0	0	0	0	0	0	0	0	0	0	0
0	0	0	0	0	0	1	0	0	0	0	0	0	0
0	0	0	0	0	0	0	0	0	0	0	0	0	0
0	0	0	0	0	0	0	0	0	0	0	0	0	0
0	0	0	0	0	0	0	0	0	0	0	0	0	0
0	0	0	0	0	0	0	0	1	0	0	0	0	0
0	0	0	0	0	0	0	0	0	0	0	0	0	0
0													
OTU4222	0	0	0	0	0	0	0	0	0	0	1	0	0
0	0	0	0	0	0	0	0	0	0	0	0	0	0
0	0	0	0	0	0	0	0	0	0	0	0	0	0
0	0	0	0	0	0	0	0	0	0	0	0	0	0
0	0	0	0	0	0	0	0	0	0	0	0	0	0
0	0	0	0	0	0	0	0	0	0	0	0	0	0
0	0	0	0	0	0	0	0	0	0	0	0	0	0
0	0	0	0	0	0	0	0	0	0	0	0	0	0
0													
OTU920	0	0	0	0	0	0	0	0	0	0	2	0	0
0	0	0	0	0	0	0	0	0	0	0	0	0	0
0	0	0	0	0	0	0	0	0	0	0	0	0	0
0	0	0	0	0	0	0	0	0	0	0	0	0	0
0	0	0	0	0	0	0	0	0	0	0	0	0	0
0	0	0	0	0	0	0	0	0	0	0	0	0	0
0	0	0	0	0	0	0	0	0	0	0	0	0	0
0	0	0	0	0	0	0	0	0	0	0	0	0	0
0													
OTU7734	0	0	0	0	0	0	0	0	0	0	1	0	0
0	0	0	0	0	1	0	0	0	0	0	0	0	0
0	0	0	0	0	0	0	0	0	0	0	0	0	1

0	0	0	0	0	0	0	0	0	0	0	0	0	0
0	0	0	0	0	0	0	0	0	0	0	0	0	0
0	0	0	0	0	0	0	0	0	0	0	0	0	0
0	0	0	0	0	0	0	0	0	0	0	0	0	0
0	0	0	0	0	0	0	0	0	0	0	0	0	0
0													
OTU4223	0	0	0	0	0	0	0	0	0	0	1	0	0
0	0	0	0	0	0	0	0	0	0	0	0	0	0
0	0	0	0	0	0	0	0	0	0	0	0	0	0
0	0	0	0	0	0	0	0	0	0	0	0	0	0
0	0	0	0	0	0	0	0	0	0	0	0	0	0
0	0	0	0	0	0	0	0	0	0	0	0	0	0
0	0	0	0	0	0	0	0	0	0	0	0	0	0
0	0	0	0	0	0	0	0	0	0	0	0	0	0
0													
OTU12492	0	0	0	0	0	0	0	0	0	0	1	0	0
0	0	0	0	0	0	0	0	0	0	0	0	0	0
0	0	0	0	0	0	0	0	0	0	0	0	0	0
0	0	0	0	0	0	0	0	0	0	0	0	0	0
0	0	0	0	0	0	0	0	0	0	0	0	0	0
0	0	0	0	0	0	0	0	0	0	0	0	0	0
0	0	0	0	0	0	0	0	0	0	0	0	0	0
0	0	0	0	0	0	0	0	0	0	0	0	0	0
0													
OTU15612	0	0	0	0	0	0	0	0	0	0	1	0	0
0	0	0	0	0	0	0	0	0	0	0	0	0	0
0	0	0	0	0	0	0	0	0	0	0	0	0	0
0	0	0	0	0	0	0	0	0	0	0	0	0	0
0	0	0	0	0	0	0	0	0	0	0	0	0	0
0	0	0	0	0	0	0	0	0	0	0	0	0	0
0	0	0	0	0	0	0	0	0	0	0	0	0	0
0	0	0	0	0	0	0	0	0	0	0	0	0	0
0													

OTU15614	0	0	0	0	0	0	0	0	0	0	1	0	0
0	0	0	0	0	0	0	0	0	0	0	0	0	0
0	0	0	0	0	0	0	0	0	0	0	0	0	0
0	0	0	0	0	0	0	0	0	0	0	0	0	0
0	0	0	0	0	0	0	0	0	0	0	0	0	0
0	0	0	0	0	0	0	0	0	0	0	0	0	0
0	0	0	0	0	0	0	0	0	0	0	0	0	0
0	0	0	0	0	0	0	0	0	0	0	0	0	0
0													
OTU15613	0	0	0	0	0	0	0	0	0	0	1	0	0
0	0	0	0	0	0	0	0	0	0	0	0	0	0
0	0	0	0	0	0	0	0	0	0	0	0	0	0
0	0	0	0	0	0	0	0	0	0	0	0	0	0
0	0	0	0	0	0	0	0	0	0	0	0	0	0
0	0	0	0	0	0	0	0	0	0	0	0	0	0
0	0	0	0	0	0	0	0	0	0	0	0	0	0
0	0	0	0	0	0	0	0	0	0	0	0	0	0
0													
OTU693	0	0	0	0	0	0	0	0	0	0	1	0	0
0	0	0	0	0	1	0	0	0	0	0	0	0	26
0	0	0	0	0	0	0	0	0	4	0	0	0	0
0	0	0	0	0	0	0	0	0	0	0	0	0	0
0	0	0	0	0	0	0	0	0	0	0	0	0	0
0	0	0	0	0	0	0	0	0	0	0	0	0	0
0	0	0	0	0	0	0	0	0	0	0	0	0	0
0	0	0	0	0	0	0	0	0	0	0	0	0	0
0													
OTU6152	0	0	0	0	0	0	0	0	0	0	1	0	0
0	0	0	0	0	0	0	0	0	0	0	0	0	0
0	0	0	0	0	0	0	0	0	0	0	0	0	0
0	0	0	0	0	0	0	0	0	0	0	0	0	0
0	0	0	0	0	0	0	0	0	0	0	0	1	0
0	0	0	0	0	0	0	0	0	0	0	0	0	0

0	0	0	0	0	0	0	0	0	0	0	0	0	0
0	0	0	0	0	0	0	0	0	0	0	0	0	0
0													
OTU3674	0	0	0	0	0	0	0	0	0	0	1	0	0
0	0	0	0	0	0	0	0	0	22	0	0	0	0
0	0	1	0	0	0	0	0	0	0	0	0	0	0
0	0	0	1	0	0	0	0	0	0	0	0	0	0
0	0	0	0	0	0	0	0	0	0	0	0	0	0
0	0	0	0	0	0	1	0	0	0	0	0	0	0
0	0	0	0	1	0	0	0	3	0	0	0	0	0
0	0	0	0	0	0	0	0	0	0	0	0	0	0
0													
OTU4221	0	0	0	0	0	0	0	0	0	0	2	0	0
0	0	0	0	0	0	0	0	0	0	0	0	0	1
0	0	0	0	0	0	0	0	0	0	0	0	0	0
0	0	0	0	0	0	0	0	0	0	0	0	0	0
0	0	0	0	0	0	0	0	0	0	0	0	0	0
0	0	0	0	0	0	0	0	0	0	0	0	0	0
0	0	0	0	0	0	0	0	0	0	0	0	0	0
0	0	0	0	0	0	0	0	0	0	0	0	0	0
0													
OTU7729	0	0	0	0	0	0	0	0	0	0	1	0	0
0	0	0	0	0	0	0	0	0	0	0	0	0	0
0	0	0	0	0	0	0	0	0	0	0	0	0	0
0	0	0	0	0	0	0	0	0	0	0	0	0	0
0	0	0	0	0	0	0	0	0	0	0	0	0	0
0	0	0	0	0	0	0	0	0	0	0	0	0	0
0	0	0	0	0	0	0	0	0	0	0	0	0	0
0	0	0	0	0	0	0	0	0	0	0	0	0	0
0													
OTU4220	0	0	0	0	0	0	0	0	0	0	1	0	0
0	0	0	0	0	0	0	0	0	0	0	0	0	0
0	0	0	0	0	0	0	0	0	0	0	0	0	0

0	0	0	0	0	0	0	0	0	0	0	0	0	0
0	0	0	0	0	0	0	0	0	0	0	0	0	0
0	0	0	0	0	0	0	0	0	0	0	0	0	0
0	0	0	0	0	0	0	0	0	0	0	0	0	0
0	0	0	0	0	0	0	0	0	0	0	0	0	0
0													
OTU7731	0	0	0	0	0	0	0	0	0	0	1	0	0
0	0	0	0	0	0	0	0	0	0	0	0	0	0
0	0	0	0	0	0	0	0	0	0	0	0	0	0
0	0	0	0	0	0	0	0	0	0	0	0	0	0
0	0	0	0	0	0	0	0	0	0	0	0	0	0
0	0	0	0	0	0	0	0	0	0	0	0	0	0
0	0	0	0	0	0	0	0	0	0	0	0	0	0
0	0	0	0	0	0	0	0	0	0	0	0	0	0
0													
OTU2931	0	0	0	0	0	0	0	0	0	0	1	0	0
0	0	0	0	0	0	0	0	0	0	0	1	0	0
0	0	0	0	0	0	0	0	0	0	0	0	0	0
0	0	0	0	0	0	0	0	0	0	0	0	0	0
0	0	0	0	0	0	0	0	0	0	0	0	0	0
0	0	0	0	0	0	0	0	0	0	0	0	0	0
0	0	0	0	0	0	0	0	0	0	0	1	0	0
0	0	0	0	0	0	0	0	0	0	0	0	0	0
0													
OTU12487	0	0	0	0	0	0	0	0	0	0	1	0	0
0	0	0	0	0	0	0	0	0	0	0	0	0	0
0	0	0	0	0	0	0	0	0	0	0	0	0	0
0	0	0	0	0	0	0	0	0	0	0	0	0	0
0	0	0	0	0	0	0	0	0	0	0	0	0	0
0	0	0	0	0	0	0	0	0	0	0	0	0	0
0	0	0	0	0	0	0	0	0	0	0	0	0	0
0	0	0	0	0	0	0	0	0	0	0	0	0	0
0													

OTU747	0	0	0	0	0	0	0	0	0	0	1	0	0
0	0	0	0	0	0	0	0	0	0	0	0	0	16
0	0	0	0	0	0	0	0	0	3	0	0	0	0
0	0	0	0	0	0	0	0	0	0	0	0	0	0
0	0	0	0	0	0	0	0	0	0	0	0	0	0
0	0	0	0	0	0	0	0	0	0	0	0	0	0
0	0	0	0	0	0	0	0	0	0	0	0	0	0
0	0	0	0	0	0	0	0	0	0	0	0	0	0
0													
OTU7730	0	0	0	0	0	0	0	0	0	0	1	0	0
0	0	0	0	0	0	0	0	0	0	0	0	0	0
0	0	0	0	0	0	0	0	0	0	0	0	0	0
0	0	0	0	0	0	0	0	0	0	0	0	0	0
0	0	0	0	0	0	0	0	0	0	0	0	0	0
0	0	0	0	0	0	0	0	0	0	0	0	0	0
0	0	0	0	0	0	0	0	0	0	0	0	0	0
0	0	0	0	0	0	0	0	0	0	0	0	0	0
0													
OTU7740	0	0	0	0	0	0	0	0	0	0	1	0	0
0	0	0	0	0	0	0	0	0	0	0	0	0	0
0	0	0	0	0	0	0	0	0	0	0	0	0	0
0	0	0	0	0	0	0	0	0	0	0	0	0	0
0	0	0	0	0	0	0	0	0	0	0	0	0	0
0	0	0	0	0	0	0	0	0	0	0	0	0	0
0	0	0	0	0	0	0	0	0	0	0	0	0	0
0	0	0	0	0	0	0	0	0	0	0	0	0	0
0													
OTU1017	0	0	0	0	0	0	0	0	0	0	1	0	0
0	0	0	0	0	2	0	0	0	0	0	0	0	1
0	0	0	0	0	0	0	5	0	0	0	0	0	1
0	0	0	0	0	1	0	0	0	0	0	0	0	0
0	0	0	0	0	0	0	0	0	0	0	0	0	0
0	0	0	0	0	0	0	0	0	0	0	0	0	0

0	0	0	0	0	0	0	0	0	0	0	0	0	0
0	0	0	0	0	0	0	0	0	0	0	0	0	0
0													
OTU12974	0	0	0	0	0	0	0	0	0	0	1	0	0
0	0	0	0	0	0	0	0	0	0	0	0	1	0
0	1	0	0	0	0	0	1	0	0	2	0	0	0
0	0	0	0	0	0	0	0	0	0	0	0	0	0
0	0	0	0	0	0	0	0	0	1	0	0	0	0
0	0	1	0	0	0	0	0	0	0	0	0	0	0
0	0	0	0	0	0	0	0	1	0	0	0	0	0
1	0	0	0	0	0	0	0	0	0	0	0	1	0
0													
OTU12490	0	0	0	0	0	0	0	0	0	0	1	0	0
0	0	0	0	0	0	0	0	0	0	0	0	0	0
0	0	0	0	0	0	0	0	0	0	0	0	0	0
0	0	0	0	0	0	0	0	0	0	0	0	0	0
0	0	0	0	0	0	0	0	0	0	0	0	0	0
0	0	0	0	0	0	0	0	0	0	0	0	0	0
0	0	0	0	0	0	0	0	0	0	0	0	0	0
0	0	0	0	0	0	0	0	0	0	0	0	0	0
0													
OTU7739	0	0	0	0	0	0	0	0	0	0	2	0	0
0	0	0	0	0	0	0	0	0	0	0	0	0	0
0	0	0	0	0	0	0	0	0	0	0	0	0	0
0	0	0	0	0	0	0	0	0	0	0	0	0	0
0	0	0	0	0	0	0	0	0	0	0	0	0	0
0	0	0	0	0	0	0	0	0	0	0	0	0	0
0	0	0	0	0	0	0	0	0	0	0	0	0	0
0	0	0	0	0	0	0	0	0	0	0	0	0	0
0													
OTU12489	0	0	0	0	0	0	0	0	0	0	1	0	0
0	0	0	0	0	0	0	0	0	0	0	0	0	0
0	0	0	0	0	0	0	0	0	0	0	0	0	0

0	0	0	0	0	0	0	0	0	0	0	0	0	0
0	0	0	0	0	0	0	0	0	0	0	0	0	0
0	0	0	0	0	0	0	0	0	0	0	0	0	0
0	0	0	0	0	0	0	0	0	0	0	0	0	0
0	0	0	0	0	0	0	0	0	0	0	0	0	0
0													
OTU13184	0	0	0	0	0	0	0	0	0	0	1	0	0
1	0	0	0	0	0	0	0	0	0	0	0	0	1
0	0	1	0	0	0	0	0	0	0	0	1	0	0
0	2	0	0	0	0	1	0	0	0	0	0	0	0
0	0	0	0	0	0	0	0	0	0	0	0	0	0
0	0	0	0	0	0	2	0	0	0	0	0	1	0
2	0	0	0	4	0	0	0	2	0	0	0	0	0
1	0	0	0	0	0	0	0	0	0	0	1	0	0
0													
OTU12491	0	0	0	0	0	0	0	0	0	0	1	0	0
0	0	0	0	0	0	0	0	0	0	0	0	0	0
0	0	0	0	0	0	0	0	0	0	0	0	0	0
0	0	0	0	0	0	0	0	0	0	0	0	0	0
0	0	0	0	0	0	0	0	0	0	0	0	0	0
0	0	0	0	0	0	0	0	0	0	0	0	0	0
0	0	0	0	0	0	0	0	0	0	0	0	0	0
0	0	0	0	0	0	0	0	0	0	0	0	0	0
0													
OTU15618	0	0	0	0	0	0	0	0	0	0	1	1	0
0	0	0	0	0	0	0	0	0	0	0	0	0	0
0	0	0	0	0	0	0	0	0	0	0	0	0	0
0	0	0	0	0	0	0	0	0	0	0	0	0	0
0	0	0	0	0	0	0	0	0	0	0	0	0	0
0	0	0	0	0	0	0	0	0	0	0	0	0	0
0	0	0	0	0	0	0	0	0	0	0	0	0	0
0	0	0	0	0	0	0	0	0	0	0	0	0	0
0													

OTU4565	0	0	0	0	0	0	0	0	0	0	1	0	0
0	0	0	0	1	0	0	0	0	0	0	0	0	0
0	0	0	0	0	0	0	0	0	0	0	0	0	0
0	0	0	0	0	0	0	0	0	0	0	0	0	0
0	0	0	0	0	0	0	0	0	0	0	0	0	0
0	0	0	0	0	0	0	0	0	0	0	0	0	0
0	0	0	0	0	0	0	0	0	0	0	0	0	0
0	0	0	0	0	0	0	0	0	0	0	0	0	0
0													
OTU14863	0	0	0	0	0	0	0	0	0	0	1	0	0
0	0	0	0	0	0	0	0	0	0	0	0	0	0
0	0	0	0	0	0	0	0	0	0	0	0	0	0
0	0	0	0	0	0	0	0	0	0	0	0	0	0
0	0	0	0	0	0	0	0	0	0	0	0	0	0
0	0	0	0	0	0	0	0	0	0	0	0	0	0
0	0	0	0	0	0	0	0	0	0	0	0	0	1
0	0	0	0	0	0	0	0	0	0	0	0	0	0
0													
OTU13342	0	0	0	0	0	0	0	0	0	0	1	0	0
0	0	0	0	0	0	0	0	0	0	0	0	0	0
0	0	1	1	0	0	0	0	0	0	0	1	0	2
0	1	0	0	1	0	0	0	0	0	0	0	1	0
0	0	0	0	0	0	1	0	0	0	0	0	0	0
0	0	0	0	0	0	0	0	0	0	0	0	0	0
0	0	0	0	0	0	0	0	0	0	0	0	0	0
0	0	0	0	0	0	0	0	0	0	0	0	0	0
0													
OTU7736	0	0	0	0	0	0	0	0	0	0	1	0	0
0	0	0	0	0	0	0	0	0	0	0	0	0	0
0	0	0	0	0	0	0	0	0	0	0	0	0	0
0	0	0	0	0	0	0	0	0	0	0	0	0	0
0	0	0	0	0	0	0	0	0	0	0	0	0	0
0	0	0	0	0	0	0	0	0	0	0	0	0	0

0	0	0	0	0	0	0	0	0	0	0	0	0	0
0	0	0	0	0	0	0	0	0	0	0	0	0	0
0													
OTU9460	0	0	0	0	0	0	0	0	0	0	1	0	0
1	0	0	0	0	1	0	0	0	0	0	0	0	0
0	0	1	0	0	0	0	1	0	0	0	0	0	1
0	0	0	0	0	0	0	0	0	0	0	0	0	0
0	0	0	0	0	0	0	0	0	0	0	0	0	0
0	0	0	0	0	0	1	0	0	0	0	0	0	0
0	0	0	0	0	0	0	0	0	0	1	0	0	0
0	0	0	0	0	0	0	0	1	0	0	2	2	0
0													
OTU4304	0	0	0	0	0	0	0	0	0	0	1	0	0
0	0	0	0	0	0	0	0	0	0	0	0	0	0
0	0	0	0	0	0	0	0	0	0	0	0	0	0
0	0	0	0	0	0	0	0	0	0	0	0	0	0
0	0	0	0	0	0	0	0	0	0	0	0	0	0
0	0	0	0	0	0	0	0	0	0	0	0	0	0
0	0	0	0	0	0	0	0	0	0	0	0	0	0
0	0	0	0	0	0	0	0	0	0	0	0	0	0
0													
OTU7735	0	0	0	0	0	0	0	0	0	0	1	0	0
0	0	0	0	0	0	0	0	0	0	0	0	0	0
0	0	0	0	0	0	0	0	0	0	0	0	0	0
0	0	0	0	0	0	0	0	0	0	0	0	0	0
0	0	0	0	0	0	0	0	0	0	0	0	0	0
0	0	0	0	0	0	0	0	0	0	0	0	0	0
0	0	0	0	0	0	0	0	0	0	0	0	0	0
0	0	0	0	0	0	0	0	0	0	0	0	0	0
0													
OTU4303	0	0	0	0	0	0	0	0	0	0	1	0	0
0	0	0	0	0	0	0	0	0	0	0	0	0	0
0	0	0	0	0	0	0	0	0	0	0	0	0	0

0 0 0 0 0 0 0 0 0 0 0 0 0 0
0 0 0 0 0 0 0 0 0 0 0 0 0 0
0 0 0 0 0 0 0 0 0 0 0 0 0 0
0 0 0 0 0 0 0 0 0 0 0 0 0 0
0 0 0 0 0 0 0 0 0 0 0 0 0 0
0
OTU12479 0 0 0 0 0 0 0 0 0 0 1 0 0
0 0 0 0 0 0 0 0 0 0 0 0 0 0
0 0 0 0 0 0 0 0 0 0 0 0 0 0
0 0 0 0 0 0 0 0 0 0 0 0 0 0
0 0 0 0 0 0 0 0 0 0 0 0 0 0
0 0 0 0 0 0 0 0 0 0 0 0 0 0
0 0 0 0 0 0 0 0 0 0 0 0 0 0
0 0 0 0 0 0 0 0 0 0 0 0 0 0
0
OTU12480 0 0 0 0 0 0 0 0 0 0 1 0 0
0 0 0 0 0 0 0 0 0 0 0 0 0 0
0 0 0 0 0 0 0 0 0 0 0 0 0 0
0 0 0 0 0 0 0 0 0 0 0 0 0 0
0 0 0 0 0 0 0 0 0 0 0 0 0 0
0 0 0 0 0 0 0 0 0 0 0 0 0 0
0 0 0 0 0 0 0 0 0 0 0 0 0 0
0 0 0 0 0 0 0 0 0 0 0 0 0 0
0
OTU4305 0 0 0 0 0 0 0 0 0 0 1 0 0
0 0 0 0 0 0 0 0 0 0 0 0 0 0
0 0 0 0 0 0 0 0 0 0 0 0 0 0
0 0 0 0 0 0 0 0 0 0 0 0 0 0
0 0 0 0 0 0 0 0 0 0 0 0 0 0
0 0 0 0 0 0 0 0 0 0 0 0 0 0
0 0 0 0 0 0 0 0 0 0 0 0 0 0
0 0 0 0 0 0 0 0 0 0 0 0 0 0
0

OTU7737	0	0	0	0	0	0	0	0	0	0	1	0	0
0	0	0	0	0	0	0	0	0	0	0	0	0	0
0	0	0	0	0	0	0	0	0	0	0	0	0	0
0	0	0	0	0	0	0	0	0	0	0	0	0	0
0	0	0	0	0	0	0	0	0	0	0	0	0	0
0	0	0	0	0	0	0	0	0	0	0	0	0	0
0	0	0	0	0	0	0	0	0	0	0	0	0	0
0	0	0	0	0	0	0	0	0	0	0	0	0	0
0													
OTU3126	0	0	0	0	0	0	0	0	0	0	1	0	0
0	0	0	0	0	0	0	0	0	0	0	0	0	0
0	0	0	0	0	0	0	0	0	0	0	0	0	0
0	0	0	0	0	0	0	0	0	0	0	0	0	1
0	0	0	0	0	0	0	0	0	0	0	0	0	1
0	0	0	0	0	0	1	0	0	0	1	0	0	0
0	0	1	0	0	0	0	0	0	0	0	0	0	0
0	0	0	0	0	0	0	0	0	0	0	0	0	0
0													
OTU15619	0	0	0	0	0	0	0	0	0	0	1	0	0
0	0	0	0	0	0	0	0	0	0	0	0	0	0
0	0	0	0	0	0	0	0	0	0	0	0	0	0
0	0	0	0	0	0	0	0	0	0	0	0	0	0
0	0	0	0	0	0	0	0	0	0	0	0	0	0
0	0	0	0	0	0	0	0	0	0	0	0	0	0
0	0	0	0	0	0	0	0	0	0	0	0	0	0
0	0	0	0	0	0	0	0	0	0	0	0	0	0
0													
OTU4308	0	0	0	0	0	0	0	0	0	0	1	0	0
0	0	0	0	0	0	0	0	0	0	0	0	0	0
0	0	0	0	0	0	0	0	0	0	0	0	0	0
0	0	0	0	0	0	0	0	0	0	0	0	0	0
0	0	0	0	0	0	0	0	0	0	0	0	0	0
0	0	0	0	0	0	0	0	0	0	0	0	0	0

0	0	0	0	0	0	0	0	0	0	0	0	0	0
0	0	0	0	0	0	0	0	0	0	0	0	0	0
0													
OTU4309	0	0	0	0	0	0	0	0	0	0	1	0	0
0	0	0	0	0	0	0	0	0	0	0	0	0	0
0	0	0	0	0	0	0	0	0	0	0	0	0	0
0	0	0	0	0	0	0	0	0	0	0	0	0	0
0	0	0	0	0	0	0	0	0	0	0	0	0	0
0	0	0	0	0	0	0	0	0	0	0	0	0	0
0	0	0	0	0	0	0	0	0	0	0	0	0	0
0	0	0	0	0	0	0	0	0	0	0	0	0	0
0													
OTU12477	0	0	0	0	0	0	0	0	0	0	1	0	0
0	0	0	0	0	0	0	0	0	0	0	0	0	0
0	0	0	0	0	0	0	0	0	0	0	0	0	0
0	0	0	0	0	0	0	0	0	0	0	0	0	0
0	0	0	0	0	0	0	0	0	0	0	0	0	0
0	0	0	0	0	0	0	0	0	0	0	0	0	0
0	0	0	0	0	0	0	0	0	0	0	0	0	0
0	0	0	0	0	0	0	0	0	0	0	0	0	0
0													
OTU15682	0	0	0	0	0	0	0	0	0	0	1	0	0
0	0	0	0	0	0	0	0	0	0	0	0	0	0
0	0	0	0	0	0	0	0	0	0	0	0	0	0
0	0	0	0	0	0	0	0	0	0	0	0	0	0
0	0	0	0	0	0	0	0	0	0	0	0	0	0
0	0	0	0	0	0	0	0	0	0	0	0	0	0
0	0	0	0	0	0	0	0	0	0	0	0	0	0
0	0	0	0	0	0	0	0	0	0	0	0	0	0
0													
OTU4749	0	0	0	0	0	0	0	0	0	0	1	0	1
4	0	2	0	0	2	0	0	0	0	0	0	0	0
0	0	0	0	0	0	0	2	0	0	0	1	0	1

0	0	1	0	0	0	0	0	1	0	0	0	0	0
2	0	1	0	0	0	1	0	0	1	0	0	0	0
1	0	0	0	0	0	1	0	0	0	0	0	0	0
0	0	0	0	0	0	0	0	3	0	1	0	0	4
0	0	0	0	0	0	0	0	0	0	0	0	0	0
0													
OTU7691	0	0	0	0	0	0	0	0	0	0	1	0	0
0	0	0	0	0	0	0	0	0	0	0	0	0	0
0	0	0	0	0	0	0	0	0	0	0	0	0	0
0	0	0	0	0	0	0	0	0	0	0	0	0	0
0	0	0	0	0	0	0	0	0	0	0	0	0	0
0	0	0	0	0	0	0	0	0	0	0	0	0	0
0	0	0	0	0	0	0	0	0	0	0	0	0	0
0	0	0	0	0	0	0	0	0	0	0	0	0	0
0													
OTU4311	0	0	0	0	0	0	0	0	0	0	1	0	0
0	0	0	0	0	0	0	0	0	0	0	0	0	0
0	0	0	0	0	0	0	0	0	0	0	0	0	0
0	0	0	0	0	0	0	0	0	0	0	0	0	0
0	0	0	0	0	0	0	0	0	0	0	0	0	0
0	0	0	0	0	0	0	0	0	0	0	0	0	0
0	0	0	0	0	0	0	0	0	0	0	0	0	0
0	0	0	0	0	0	0	0	0	0	0	0	0	0
0													
OTU4312	0	0	0	0	0	0	0	0	0	0	1	0	0
0	0	0	0	0	0	0	0	0	0	0	0	0	0
0	0	0	0	0	0	0	0	0	0	0	0	0	0
0	0	0	0	0	0	0	0	0	0	0	0	0	0
0	0	0	0	0	0	0	0	0	0	0	0	0	0
0	0	0	0	0	0	0	0	0	0	0	0	0	0
0	0	0	0	0	0	0	0	0	0	0	0	0	0
0	0	0	0	0	0	0	0	0	0	0	0	0	0
0													

OTU4310	0	0	0	0	0	0	0	0	0	0	1	0	0
0	0	0	0	0	0	0	0	0	0	0	0	0	0
0	0	0	0	0	0	0	0	0	0	0	0	0	0
0	0	0	0	0	0	0	0	0	0	0	0	0	0
0	0	0	0	0	0	0	0	0	0	0	0	0	0
0	0	0	0	0	0	0	0	0	0	0	0	0	0
0	0	0	0	0	0	0	0	0	0	0	0	0	0
0	0	0	0	0	0	0	0	0	0	0	0	0	0
0													
OTU15684	0	0	0	0	0	0	0	0	0	0	1	0	0
0	0	0	0	0	0	0	0	0	0	0	0	0	0
0	0	0	0	0	0	0	0	0	0	0	0	0	0
0	0	0	0	0	0	0	0	0	0	0	0	0	0
0	0	0	0	0	0	0	0	0	0	0	0	0	0
0	0	0	0	0	0	0	0	0	0	0	0	0	0
0	0	0	0	0	0	0	0	0	0	0	0	0	0
0	0	0	0	0	0	0	0	0	0	0	0	0	0
0													
OTU4160	0	0	0	0	0	0	0	0	0	0	1	0	0
1	0	0	0	0	0	0	0	0	0	0	0	0	0
0	0	0	0	0	0	0	0	0	0	0	0	0	0
0	1	0	0	0	0	0	0	0	0	0	0	0	0
0	0	0	0	0	0	0	0	0	0	0	0	0	0
0	0	0	0	0	0	0	0	0	0	0	0	0	0
0	0	0	0	1	0	0	0	0	0	0	0	0	0
0	0	0	0	0	0	0	0	0	0	0	0	0	0
0													
OTU12478	0	0	0	0	0	0	0	0	0	0	1	0	0
0	0	0	0	0	0	0	0	0	0	0	0	0	0
0	0	0	0	0	0	0	0	0	0	0	0	0	0
0	0	0	0	0	0	0	0	0	0	0	0	0	0
0	0	0	0	0	0	0	0	0	0	0	0	0	0
0	0	0	0	0	0	0	0	0	0	0	0	0	0

0	0	0	0	0	0	0	0	0	0	0	0	0	0
0	0	0	0	0	0	0	0	0	0	0	0	0	0
0													
OTU15683	0	0	0	0	0	0	0	0	0	0	1	0	0
0	0	0	0	0	0	0	0	0	0	0	0	0	0
0	0	0	0	0	0	0	0	0	0	0	0	0	0
0	0	0	0	0	0	0	0	0	0	0	0	0	0
0	0	0	0	0	0	0	0	0	0	0	0	0	0
0	0	0	0	0	0	0	0	0	0	0	0	0	0
0	0	0	0	0	0	0	0	0	0	0	0	0	0
0	0	0	0	0	0	0	0	0	0	0	0	0	0
0													
OTU818	0	0	0	0	0	0	0	0	0	0	0	5	0
0	0	0	0	0	0	0	0	0	0	0	0	0	0
0	0	0	0	0	0	0	0	0	0	0	0	0	0
0	0	0	0	0	0	0	0	0	0	0	0	0	0
0	0	0	0	0	0	0	0	0	0	0	0	0	0
0	0	0	0	0	0	0	0	0	0	0	0	0	0
0	0	0	0	0	0	0	0	0	3	0	0	0	0
4	0	0	0	0	0	0	0	0	0	2	0	1	0
0													
OTU1947	0	0	0	0	0	0	0	0	0	0	0	1	0
0	0	0	0	0	0	0	0	0	0	0	0	0	0
0	1	0	0	0	0	0	0	0	0	0	0	0	0
0	0	0	0	0	0	0	0	0	0	0	0	1	0
0	0	0	0	2	1	0	1	0	0	0	0	0	0
0	0	0	0	0	0	0	0	1	0	0	0	0	0
0	0	0	0	0	0	0	0	0	0	0	0	0	0
0	0	0	0	0	0	0	0	0	0	0	0	0	0
0													
OTU14314	0	0	0	0	0	0	0	0	0	0	0	1	0
0	0	0	0	0	0	0	0	0	0	0	0	0	0
0	0	0	0	0	0	0	1	0	0	0	0	0	0

1	0	0	0	0	0	0	0	0	0	0	0	0	0
0	0	0	0	0	0	0	0	0	0	0	0	0	0
0	0	0	0	0	0	0	0	0	0	0	0	1	0
0	0	0	0	0	0	0	0	0	0	0	0	0	0
0	0	0	0	0	0	0	0	0	0	2	0	0	0
0													
OTU11616	0	0	0	0	0	0	0	0	0	0	0	1	0
0	0	0	3	0	0	0	0	0	0	1	0	0	0
0	1	0	0	0	0	0	1	0	0	0	0	0	1
0	0	1	0	1	0	0	0	0	0	0	0	0	0
0	0	0	0	0	0	0	0	0	0	0	1	0	0
0	0	0	0	0	0	0	0	0	0	0	0	1	0
0	0	0	0	0	0	0	0	0	0	0	0	0	0
0	0	0	0	0	0	0	0	0	0	0	0	1	0
0													
OTU10362	0	0	0	0	0	0	0	0	0	0	0	1	0
0	0	0	0	0	0	0	0	0	0	0	0	0	0
0	0	0	0	1	0	0	0	0	0	0	0	0	0
0	0	0	0	0	0	0	0	0	0	0	1	0	0
0	0	1	0	0	0	0	0	0	0	0	0	0	0
0	0	1	0	0	0	0	0	0	0	0	0	0	0
0	0	0	0	0	0	1	0	0	0	0	0	0	0
0	0	0	0	0	0	0	0	0	0	0	0	1	0
0													
OTU15681	0	0	0	0	0	0	0	0	0	0	0	1	0
0	0	0	0	0	0	0	0	0	0	0	0	0	0
0	0	0	0	0	0	0	0	0	0	0	0	0	0
0	0	0	0	0	0	0	0	0	0	0	0	0	0
0	0	0	0	0	0	0	0	0	0	0	0	0	0
0	0	0	0	0	0	0	0	0	0	0	0	0	0
0	0	0	0	0	0	0	0	0	0	0	0	0	0
0	0	0	0	0	0	0	0	0	0	0	0	0	0
0													

OTU15680 0 0 0 0 0 0 0 0 0 0 0 1 0
0 0 0 0 0 0 1 0 0 0 0 0 1 0
0 0 0 0 0 0 1 0 0 0 0 0 0 0
0 0 0 0 0 0 0 0 0 0 0 0 0 0
0 0 0 0 0 0 0 0 0 0 0 0 0 0
0 0 0 0 0 0 0 0 0 0 0 0 0 0
0 0 0 1 0 0 0 0 0 0 0 0 0 0
0 0 0 0 0 0 0 0 0 0 0 0 0 0
0
OTU8153 0 0 0 0 0 0 0 0 0 0 0 2 1
0 0 0 0 0 0 0 9 0 0 0 0 0 0
0 0 0 1 0 0 0 0 0 0 0 0 0 0
0 2 0 0 0 0 0 0 0 0 0 0 0 0
0 0 0 0 0 0 0 0 0 0 0 1 0 0
0 1 0 0 1 0 0 0 0 0 0 0 0 0
0 0 0 0 0 0 0 0 0 1 0 0 0 0
0 0 0 0 0 0 0 0 0 0 0 0 4 0
0
OTU12482 0 0 0 0 0 0 0 0 0 0 0 1 0
0 0 0 0 0 0 0 0 0 0 0 0 0 0
0 0 0 0 0 0 0 0 0 0 0 0 0 0
0 0 0 0 0 0 0 0 0 0 0 0 0 0
0 0 0 0 0 0 0 0 0 0 0 0 0 0
0 0 0 0 0 0 0 0 0 0 0 0 0 0
0 0 0 0 0 0 0 0 0 0 0 0 0 0
0 0 0 0 0 0 0 0 0 0 0 0 0 0
0
OTU4318 0 0 0 0 0 0 0 0 0 0 0 2 0
0 0 0 0 0 0 0 0 0 0 0 0 0 0
0 0 0 0 0 0 0 0 0 0 0 0 0 0
0 0 0 0 0 0 0 0 0 0 0 0 0 0
0 0 0 0 0 0 0 0 0 0 0 0 0 0
0 0 0 0 0 0 0 0 0 0 0 0 0 0

0	0	0	0	0	0	0	0	0	0	0	0	0	0
0	0	0	0	0	0	0	0	0	0	0	0	0	0
0													
OTU12481	0	0	0	0	0	0	0	0	0	0	0	1	0
0	0	0	0	0	0	0	0	0	0	0	0	0	0
0	0	0	0	0	0	0	0	0	0	0	0	0	0
0	0	0	0	0	0	0	0	0	0	0	0	0	0
0	0	0	0	0	0	0	0	0	0	0	0	0	0
0	0	0	0	0	0	0	0	0	0	0	0	0	0
0	0	0	0	0	0	0	0	0	0	0	0	0	0
0	0	0	0	0	0	0	0	0	0	0	0	0	0
0													
OTU11689	0	0	0	0	0	0	0	0	0	0	0	2	1
13	0	0	0	0	1	0	0	0	0	0	0	0	0
0	0	0	1	1	0	0	0	0	0	0	0	0	0
0	0	0	0	0	0	0	0	0	0	0	0	2	0
2	0	1	0	0	1	1	0	0	0	0	0	0	0
0	0	0	0	0	0	4	0	0	0	0	0	0	0
0	0	0	0	0	0	0	0	4	1	1	0	0	2
0	0	0	0	0	0	0	0	1	0	0	0	0	0
0													
OTU7692	0	0	0	0	0	0	0	0	0	0	0	1	0
0	0	0	0	0	0	0	0	0	0	0	0	1	0
0	0	0	0	0	0	0	0	0	0	0	0	0	0
0	0	0	0	0	0	0	0	0	0	0	0	0	0
0	0	0	0	0	0	0	0	0	0	0	0	0	0
0	0	0	0	0	0	0	0	0	0	0	0	0	0
0	0	0	0	0	0	0	0	0	0	0	0	0	0
0	0	0	0	0	0	0	0	0	0	0	0	0	0
0													
OTU1725	0	0	0	0	0	0	0	0	0	0	0	1	0
0	0	0	0	0	0	0	0	0	0	0	0	0	0
0	0	0	0	0	0	0	0	0	0	0	0	0	0

0	0	0	0	0	0	0	0	0	0	0	0	0	0
0	0	0	0	0	0	0	0	0	0	0	0	0	0
0	0	0	0	0	0	0	0	1	0	1	0	0	0
0	0	0	0	0	0	0	0	0	0	0	0	0	0
0	0	0	0	0	0	0	0	0	0	0	0	0	1
0													
OTU15686	0	0	0	0	0	0	0	0	0	0	0	1	0
0	0	0	0	0	0	0	0	0	0	0	0	0	0
0	0	0	0	0	0	0	0	0	0	0	0	0	0
0	0	0	0	0	0	0	0	0	0	0	0	0	0
0	0	0	0	0	0	0	0	0	0	0	0	0	0
0	0	0	0	0	0	0	0	0	0	0	0	0	0
0	0	0	0	0	0	0	0	0	0	0	0	0	0
0	0	0	0	0	0	0	0	0	0	0	0	0	0
0													
OTU9731	0	0	0	0	0	0	0	0	0	0	0	1	0
0	0	0	0	0	0	0	0	0	0	0	0	0	0
0	0	0	0	0	0	0	0	0	0	0	0	0	0
0	0	0	0	0	0	0	0	0	0	0	0	0	0
0	0	0	0	0	0	0	0	0	0	0	0	0	1
1	0	0	0	0	0	0	1	0	0	0	0	0	0
0	0	0	0	0	0	0	0	0	1	0	0	0	0
0	0	0	0	0	0	0	0	0	0	0	0	0	0
0													
OTU3294	0	0	0	0	0	0	0	0	0	0	0	1	0
0	0	0	1	0	0	2	0	0	0	1	0	0	0
0	0	0	0	0	0	0	0	0	0	0	0	1	0
19	0	0	0	0	0	0	0	0	0	0	0	0	0
0	0	0	0	0	0	0	0	0	0	0	0	0	0
0	0	0	0	0	0	0	0	0	0	0	1	0	0
0	0	0	0	0	0	0	0	0	0	0	0	0	0
0	0	0	6	0	0	0	0	0	0	0	0	0	1
0													

OTU15685	0	0	0	0	0	0	0	0	0	0	0	1	0
0	0	0	0	0	0	0	0	0	0	0	0	0	0
0	0	0	0	0	0	0	0	0	0	0	0	0	0
0	0	0	0	0	0	0	0	0	0	0	0	0	0
0	0	0	0	0	0	0	0	0	0	0	0	0	0
0	0	0	0	0	0	0	0	0	0	0	0	0	0
0	0	0	0	0	0	0	0	0	0	0	0	0	0
0	0	0	0	0	0	0	0	0	0	0	0	0	0
0													
OTU7688	0	0	0	0	0	0	0	0	0	0	0	1	0
0	0	0	0	0	0	0	0	0	0	0	0	0	0
0	0	0	0	0	0	0	0	0	0	0	0	0	0
0	0	0	0	0	0	0	0	0	0	0	0	0	0
0	0	0	0	0	0	0	0	0	0	0	0	0	0
0	0	0	0	0	0	0	0	0	0	0	0	0	0
0	0	0	0	0	0	0	0	0	0	0	0	0	0
0	0	0	0	0	0	0	0	0	0	0	0	0	0
0													
OTU3943	0	0	0	0	0	0	0	0	0	0	0	1	0
0	0	0	0	0	0	0	0	0	0	0	0	0	0
0	0	1	0	0	0	0	0	0	0	0	0	0	0
0	0	0	0	0	0	0	0	0	0	0	0	0	0
0	0	0	0	0	0	0	0	0	0	0	0	0	0
0	0	0	0	0	1	0	0	0	0	0	0	0	0
0	0	0	0	0	0	0	0	0	0	1	0	0	0
0	0	0	0	0	0	0	0	0	0	0	0	0	0
0													
OTU607	0	0	0	0	0	0	0	0	0	0	0	1	0
0	0	0	0	0	0	1	0	0	0	0	0	2	0
1	0	0	0	0	0	1	1	1	0	2	0	12	0
7	0	0	0	0	13	0	0	0	0	0	0	0	0
0	0	0	0	0	0	0	0	0	0	0	0	0	1
0	0	0	0	0	0	0	0	0	0	0	0	0	0

0 0 1 0 0 0 0 0 0 0 0 1 0 0
0 7 0 0 1 0 17 2 0 0 0 0 1 1
7
OTU7687 0 0 0 0 0 0 0 0 0 0 0 1 0
0 0 0 0 0 0 0 0 0 0 0 0 0 0
0 0 0 0 0 0 0 0 0 0 0 0 0 0
0 0 0 0 0 0 0 0 0 0 0 0 0 0
0 0 0 0 0 0 0 0 0 0 0 0 0 0
0 0 0 0 0 0 0 0 0 0 0 0 0 0
0 0 0 0 0 0 0 0 0 0 0 0 0 0
0 0 0 0 0 0 0 0 0 0 0 0 0 0
0
OTU2494 0 0 0 0 0 0 0 0 0 0 0 2 0
0 0 0 0 0 0 0 0 0 0 0 0 0 0
0 0 0 0 0 0 0 0 0 0 0 0 0 0
0 0 0 1 0 0 0 1 0 0 0 0 0 3
0 0 0 0 0 0 0 0 0 0 0 0 1 0
0 0 0 0 0 0 0 0 0 0 0 0 0 0
0 0 0 0 0 0 0 0 0 0 1 0 0 1
0 0 0 0 0 0 0 0 0 0 0 0 1 0
0
OTU1165 0 0 0 0 0 0 0 0 0 0 0 2 0
0 0 0 0 0 0 0 0 0 0 0 0 0 0
0 0 0 0 0 0 0 0 0 0 0 0 0 0
0 0 0 0 0 0 0 0 0 0 0 0 0 0
0 0 0 0 0 0 0 0 0 0 0 0 0 0
0 0 0 0 0 0 0 0 0 0 0 0 0 0
0 0 1 0 0 0 0 0 0 0 0 0 0 0
0 0 0 0 0 0 0 0 0 0 0 0 0 0
0
OTU12498 0 0 0 0 0 0 0 0 0 0 0 1 0
0 0 0 0 0 0 0 0 0 0 0 0 0 0
0 0 0 0 0 0 0 0 0 0 0 0 0 0

0 0 0 0 0 0 0 0 0 0 0 0 0 0
0 0 0 0 0 0 0 0 0 0 0 0 0 0
0 0 0 0 0 0 0 0 0 0 0 0 0 0
0 0 0 0 0 0 0 0 0 0 0 0 0 0
0 0 0 0 0 0 0 0 0 0 0 0 0 0
0
OTU2440 0 0 0 0 0 0 0 0 0 0 0 1 1
0 0 0 0 0 1 0 0 0 0 0 0 0 0
0 0 0 0 0 0 0 0 0 0 0 0 0 0
0 0 0 0 0 0 0 0 0 0 0 0 0 0
0 0 0 0 0 0 0 0 0 0 0 0 0 0
0 0 0 0 0 0 0 0 0 0 0 0 0 0
0 0 0 0 0 0 0 0 6 0 0 0 0 0
0 0 0 0 0 0 0 0 0 0 0 0 0 0
0
OTU709 0 0 0 0 0 0 0 0 0 0 0 1 0
0 0 0 0 0 0 0 1 0 0 0 0 0 0
0 0 0 0 0 1 0 0 0 9 0 0 0 0
0 0 0 0 0 0 0 0 0 0 0 0 0 0
0 0 0 0 0 0 0 0 0 0 0 0 0 0
0 2 0 0 0 0 0 0 0 0 0 0 0 0
0 0 0 0 0 0 0 0 0 0 0 0 0 0
0 0 2 0 0 10 1 1 0 0 0 0 1 0
0
OTU12328 0 0 0 0 0 0 0 0 0 0 0 1 0
5 0 0 0 0 0 0 0 0 0 0 0 0 0
0 0 0 0 0 0 0 0 0 0 0 0 0 0
0 0 0 0 0 0 0 0 0 0 0 0 0 0
0 0 0 0 0 0 0 0 1 0 0 0 0 0
0 0 0 0 0 0 0 0 0 0 0 0 0 0
0 0 0 0 0 0 0 0 0 0 1 0 0 1
0 0 0 0 0 0 0 0 0 0 0 0 0 0
0

OTU9955	0	0	0	0	0	0	0	0	0	0	0	1	0
1	0	2	0	0	0	0	0	1	0	0	0	1	0
0	0	0	0	0	0	0	3	0	0	0	0	0	0
0	0	0	1	0	0	0	0	0	0	1	0	0	1
0	0	0	0	0	0	0	0	0	0	0	0	0	0
0	0	0	1	0	0	0	0	0	0	1	0	0	0
5	0	0	0	1	0	1	0	4	0	5	0	0	0
2	0	0	0	0	0	0	0	0	0	2	0	0	0
0													
OTU1630	0	0	0	0	0	0	0	0	0	0	0	1	6
6	0	0	0	0	1	0	1	0	0	0	0	0	0
0	0	1	0	0	4	0	4	1	1	0	0	0	3
0	0	0	1	0	0	1	0	0	0	0	0	0	0
0	0	1	0	0	0	0	0	0	0	0	0	0	0
0	0	0	2	0	0	3	0	4	1	5	0	0	0
1	0	8	0	5	0	0	0	4	1	0	0	0	0
1	0	0	0	0	0	0	0	0	0	3	1	1	0
0													
OTU12883	0	0	0	0	0	0	0	0	0	0	0	1	0
0	2	0	0	0	0	0	0	0	0	0	0	0	0
0	0	0	0	0	0	0	0	1	0	0	0	0	0
0	0	0	0	0	0	0	0	0	0	0	0	0	0
0	0	0	1	0	0	0	0	0	0	0	1	0	0
1	0	0	0	0	0	0	1	0	0	0	0	0	0
0	0	0	0	0	0	1	0	0	0	0	0	0	0
0	0	0	0	0	0	0	0	0	0	0	0	0	0
0													
OTU5793	0	0	0	0	0	0	0	0	0	0	0	2	4
53	0	0	3	0	5	0	0	0	0	0	0	0	0
0	1	2	0	7	0	0	5	0	0	0	0	0	1
0	0	1	1	0	0	0	0	0	0	0	0	4	0
8	0	6	0	0	0	14	0	0	3	1	2	0	4
1	1	0	1	0	0	9	0	0	1	0	0	0	0

	0	1	0	0	0	0	2	0	29	1	6	0	0	13
	7	0	0	0	0	0	0	0	1	0	0	12	2	0
	0													
OTU12500		0	0	0	0	0	0	0	0	0	0	0	1	0
	0	0	0	0	0	0	0	0	0	0	0	0	0	0
	0	0	0	0	0	0	0	0	0	0	0	0	0	0
	0	0	0	0	0	0	0	0	0	0	0	0	0	0
	0	0	0	0	0	0	0	0	0	0	0	0	0	0
	0	0	0	0	0	0	0	0	0	0	0	0	0	0
	0	0	0	0	0	0	0	0	0	0	0	0	0	0
	0	0	0	0	0	0	0	0	0	0	0	0	0	0
	0													
OTU4319		0	0	0	0	0	0	0	0	0	0	0	1	0
	0	0	0	0	0	0	0	0	0	0	0	0	0	0
	0	0	0	0	0	0	0	0	0	0	0	0	0	0
	0	0	0	0	0	0	0	0	0	0	0	0	0	0
	0	0	0	0	0	0	0	0	0	0	0	0	0	0
	0	0	0	0	0	0	0	0	0	0	0	0	0	0
	0	0	0	0	0	0	0	0	0	0	0	0	0	0
	0	0	0	0	0	0	0	0	0	0	0	0	0	0
	0													
OTU13264		0	0	0	0	0	0	0	0	0	0	0	1	0
	0	0	0	0	0	0	0	0	0	0	0	0	0	0
	0	0	0	0	0	0	0	0	0	0	0	1	0	0
	0	0	0	0	0	0	0	0	0	0	0	0	0	0
	0	0	0	0	0	0	0	0	0	0	0	0	0	0
	0	0	0	0	0	0	0	0	0	0	0	0	0	0
	0	0	0	0	0	0	0	0	0	0	0	0	0	0
	0	0	0	0	0	0	0	0	0	0	0	0	0	0
	0													
OTU14072		0	0	0	0	0	0	0	0	0	0	0	1	3
	0	0	0	0	0	0	0	0	0	0	0	0	0	1
	0	0	0	0	0	0	1	0	0	0	0	0	0	0

0	0	0	0	0	0	0	0	0	0	0	0	0	0
0	0	1	0	0	0	0	0	0	0	0	0	1	0
1	0	0	0	0	0	1	0	1	0	0	0	0	1
0	0	2	0	0	0	0	0	0	1	0	0	0	0
0	0	0	0	0	0	0	0	2	0	0	1	0	0
1													
OTU7689	0	0	0	0	0	0	0	0	0	0	0	1	0
0	0	0	0	0	0	0	0	0	0	0	0	0	0
0	0	0	0	0	0	0	0	0	0	0	0	0	0
0	0	0	0	0	0	0	0	0	0	0	0	0	0
0	0	0	0	0	0	0	0	0	0	0	0	0	0
0	0	0	0	0	0	0	0	0	0	0	0	0	0
1	0	0	0	0	0	0	0	0	0	0	0	0	0
0	0	0	0	0	0	0	0	0	0	0	0	0	0
0													
OTU4320	0	0	0	0	0	0	0	0	0	0	0	1	0
0	0	0	0	0	0	0	0	0	0	0	0	0	0
0	0	0	0	0	0	0	0	0	0	0	0	0	0
0	0	0	0	0	0	0	0	0	0	0	0	0	0
0	0	0	0	0	0	0	0	0	0	0	0	0	0
0	0	0	0	0	0	0	0	0	0	0	0	0	0
0	0	0	0	0	0	0	0	0	0	0	0	0	0
0	0	0	0	0	0	0	0	0	0	0	0	0	0
0													
OTU1499	0	0	0	0	0	0	0	0	0	0	0	1	1
0	0	0	0	1	0	0	1	0	0	0	0	0	0
0	1	0	0	0	0	0	0	0	1	0	0	0	0
1	0	0	0	0	0	0	0	0	0	0	0	0	0
0	1	0	0	0	0	0	0	0	0	0	0	0	0
0	0	0	1	0	0	0	0	0	0	0	0	0	0
0	0	0	0	0	0	0	0	0	0	0	0	0	0
0	0	0	0	0	0	0	0	0	0	0	0	0	0
0													

OTU12501	0	0	0	0	0	0	0	0	0	0	0	1	0
0	0	0	0	0	0	0	0	0	0	0	0	0	0
0	0	0	0	0	0	0	0	0	0	0	0	0	0
0	0	0	0	0	0	0	0	0	0	0	0	0	0
0	0	0	0	0	0	0	0	0	0	0	0	0	0
0	0	0	0	0	0	0	0	0	0	0	0	0	0
0	0	0	0	0	0	0	0	0	0	0	0	0	0
0	0	0	0	0	0	0	0	0	0	0	0	0	0
0													
OTU16730	0	0	0	0	0	0	0	0	0	0	0	1	1
0	0	0	0	0	0	0	0	0	0	0	0	0	0
0	0	0	0	0	0	0	0	0	0	0	0	0	1
0	0	1	0	0	0	0	0	0	0	0	0	0	0
0	0	0	1	0	0	0	0	0	0	0	0	0	0
0	0	0	0	0	0	0	0	0	0	0	0	0	0
0	0	0	0	0	0	0	0	0	0	0	0	0	0
0	0	0	0	0	0	0	0	0	0	0	0	0	0
0													
OTU4324	0	0	0	0	0	0	0	0	0	0	0	1	0
0	0	0	0	0	0	0	0	0	0	0	0	0	0
0	0	0	0	0	0	0	0	0	0	0	0	0	0
0	0	0	0	0	0	0	0	0	0	0	0	0	0
0	0	0	0	0	0	0	0	0	0	0	0	0	0
0	0	0	0	0	0	0	0	0	0	0	0	0	0
0	0	0	0	0	0	0	0	0	0	0	0	0	0
0	0	0	0	0	0	0	0	0	0	0	0	0	0
0													
OTU12969	0	0	0	0	0	0	0	0	0	0	0	1	1
0	0	0	0	0	0	0	0	0	0	0	0	0	1
0	0	0	1	1	1	0	1	2	1	1	0	0	0
0	0	0	1	1	0	1	0	0	0	0	0	0	0
0	0	0	0	0	0	0	0	0	0	0	0	0	0
1	0	0	1	0	0	0	0	0	0	0	0	0	0

0	0	0	0	0	0	0	0	1	0	0	0	0	1
0	0	0	0	0	0	0	0	0	0	1	1	1	0
0													
OTU11975	0	0	0	0	0	0	0	0	0	0	0	2	2
0	2	0	0	0	0	0	0	0	0	0	1	0	0
0	0	0	0	3	1	0	0	0	0	0	0	0	0
0	5	0	0	0	1	2	0	0	0	0	0	0	0
0	0	0	0	0	0	0	0	0	0	0	0	0	0
0	0	0	2	0	0	0	0	0	0	0	0	1	0
2	0	0	0	0	0	0	0	0	0	1	0	0	6
2	0	0	0	0	0	0	0	0	0	1	3	1	0
0													
OTU2414	0	0	0	0	0	0	0	0	0	0	0	1	1
0	0	0	0	0	0	0	0	0	0	0	0	0	0
0	0	0	0	0	0	0	0	0	0	0	0	0	0
0	1	0	0	0	0	0	0	0	0	0	0	0	0
0	0	0	0	0	0	0	0	0	0	0	0	0	0
0	0	0	0	0	0	0	0	0	0	0	0	0	0
0	0	0	0	0	0	0	0	0	1	0	1	0	2
0	0	0	0	0	0	0	0	0	0	0	0	0	0
0													
OTU15679	0	0	0	0	0	0	0	0	0	0	0	1	0
0	0	0	0	0	0	0	0	0	0	0	0	0	0
0	0	0	0	0	0	0	0	0	0	0	0	0	0
1	0	0	0	0	0	0	0	0	0	0	0	0	0
0	0	0	0	0	0	0	0	0	0	0	0	0	0
0	0	0	0	0	0	0	0	0	0	0	0	0	0
0	0	0	0	0	0	0	0	0	0	0	0	0	0
0	0	0	0	0	0	0	0	0	0	0	0	0	0
0													
OTU7703	0	0	0	0	0	0	0	0	0	0	0	1	0
0	0	0	0	0	0	0	0	0	0	0	0	0	0
0	0	0	0	0	0	0	0	0	0	0	0	0	0

0 0 0 0 0 0 0 0 0 0 0 0 0 0
0 0 0 0 0 0 0 0 0 0 0 0 0 0
0 0 0 0 0 0 0 0 0 0 0 0 0 0
0 0 0 0 0 0 0 0 0 0 0 0 0 0
0 0 0 0 0 0 0 0 0 0 0 0 0 0
0
OTU10356 0 0 0 0 0 0 0 0 0 0 0 1 0
0 0 0 0 0 0 0 0 0 0 0 0 0 0
0 0 0 0 0 0 0 0 0 0 0 0 0 0
0 0 0 0 0 0 0 0 0 0 0 0 0 0
0 0 0 0 0 0 0 0 0 0 0 0 0 0
0 0 1 0 0 0 0 0 0 0 0 0 0 0
0 0 0 0 0 0 0 0 0 0 0 0 0 0
0 0 0 0 0 0 0 0 0 0 0 0 0 0
0
OTU7702 0 0 0 0 0 0 0 0 0 0 0 1 0
0 0 0 0 0 0 0 0 0 0 0 0 0 0
0 0 0 0 0 0 0 0 0 0 0 0 0 0
0 0 0 0 0 0 0 0 0 0 0 0 0 0
0 0 0 0 0 0 0 0 0 0 0 0 0 0
0 0 0 0 0 0 0 0 0 0 0 0 0 0
0 0 0 0 0 0 0 0 0 0 0 0 0 0
0 0 0 0 0 0 0 0 0 0 0 0 0 0
0
OTU4322 0 0 0 0 0 0 0 0 0 0 0 1 0
0 0 0 0 0 0 0 0 0 0 0 0 0 0
0 0 0 0 0 0 0 0 0 0 0 0 0 0
0 0 0 0 0 0 0 0 0 0 0 0 0 0
0 0 0 0 0 0 0 0 0 0 0 0 0 0
0 0 0 0 0 0 0 0 0 0 0 0 0 0
0 0 0 0 0 0 0 0 0 0 0 0 0 0
0 0 0 0 0 0 0 0 0 0 0 0 0 0
0

OTU4321 0 0 0 0 0 0 0 0 0 0 0 1 0
0 0 0 0 0 0 0 0 0 0 0 0 0 0
0 0 0 0 0 0 0 0 0 0 0 0 0 0
0 0 0 0 0 0 0 0 0 0 0 0 0 0
0 0 0 0 0 0 0 0 0 0 0 0 0 0
0 0 0 0 0 0 0 0 0 0 0 0 0 0
0 0 0 0 0 0 0 0 0 0 0 0 0 0
0 0 0 0 0 0 0 0 0 0 0 0 0 0
0
OTU12495 0 0 0 0 0 0 0 0 0 0 0 1 0
0 0 0 0 0 0 0 0 0 0 0 0 0 0
0 0 0 0 0 0 0 0 0 0 0 0 0 0
0 0 0 0 0 0 0 0 0 0 0 0 0 0
0 0 0 0 0 0 0 0 0 0 0 0 0 0
0 0 0 0 0 0 0 0 0 0 0 0 0 0
0 0 0 0 0 0 0 0 0 0 0 0 0 0
0 0 0 0 0 0 0 0 0 0 0 0 0 0
0
OTU15678 0 0 0 0 0 0 0 0 0 0 0 1 0
0 0 0 0 0 0 0 0 0 0 0 0 0 0
0 0 0 0 0 0 0 0 0 0 0 0 0 0
0 0 0 0 0 0 0 0 0 0 0 0 0 0
0 0 0 0 0 0 0 0 0 0 0 0 0 0
0 0 0 0 0 0 0 0 0 0 0 0 0 0
0 0 0 0 0 0 0 0 0 0 0 0 0 0
0 0 0 0 0 0 0 0 0 0 0 0 0 0
0
OTU12494 0 0 0 0 0 0 0 0 0 0 0 1 0
0 0 0 0 0 0 0 0 0 0 0 0 0 0
0 0 0 0 0 0 0 0 0 0 0 0 0 0
0 0 0 0 0 0 0 0 0 0 0 0 0 0
0 0 0 0 0 0 0 0 0 0 0 0 0 0
0 0 0 0 0 0 0 0 0 0 0 0 0 0

0	0	0	0	0	0	0	0	0	0	0	0	0	0
0	0	0	0	0	0	0	0	0	0	0	0	0	0
0													
OTU4323	0	0	0	0	0	0	0	0	0	0	0	1	0
0	0	0	0	0	0	0	0	0	0	0	0	0	0
0	0	0	0	0	0	0	0	0	0	0	0	0	0
0	0	0	0	0	0	0	0	0	0	0	0	0	0
0	0	0	0	0	0	0	0	0	0	0	0	0	0
0	0	0	0	0	0	0	0	0	0	0	0	0	0
0	0	0	0	0	0	0	0	0	0	0	0	0	0
0	0	0	0	0	0	0	0	0	0	0	0	0	0
0													
OTU11317	0	0	0	0	0	0	0	0	0	0	0	1	0
0	0	0	0	0	0	0	0	0	0	0	0	0	0
0	0	0	0	0	0	0	0	0	0	0	0	0	0
0	0	0	0	2	0	0	0	0	0	0	0	0	0
0	0	0	0	0	0	0	0	0	0	1	0	0	0
0	0	0	0	0	0	0	0	0	0	0	0	0	0
2	0	0	0	0	0	0	0	0	0	0	0	0	0
0	0	0	0	1	0	0	0	0	0	0	0	0	0
0													
OTU3473	0	0	0	0	0	0	0	0	0	0	0	1	0
0	0	0	0	0	0	0	1	0	0	0	0	0	0
0	0	0	0	0	0	0	1	1	0	0	0	1	0
1	0	0	0	0	0	0	1	0	0	0	0	0	0
0	0	0	0	0	0	0	0	0	0	0	0	0	0
0	0	0	0	0	0	0	0	0	0	0	0	0	0
1	0	2	0	0	0	0	0	0	0	0	0	0	0
0	0	0	0	0	0	0	0	0	0	1	0	0	0
0													
OTU3226	0	0	0	0	0	0	0	0	0	0	0	1	0
0	0	0	0	0	0	0	1	0	0	0	0	0	0
0	0	0	0	0	0	0	0	0	0	0	0	0	0

0 1 0 0 0 0 0 0 0 0 0 0 0 0
0 0 0 0 0 0 0 0 0 0 0 0 0 0
0 0 0 0 0 1 1 0 0 0 0 0 0 0
0 0 0 0 1 0 0 0 1 0 0 0 0 0
0 0 0 0 0 0 0 0 0 0 0 0 0 0
0
OTU15690 0 0 0 0 0 0 0 0 0 0 0 1 0
0 0 0 0 0 0 0 0 0 0 0 0 0 0
0 0 0 0 0 0 0 0 0 0 0 0 0 0
0 0 0 0 0 0 0 0 0 0 0 0 0 0
0 0 0 0 0 0 0 0 0 0 0 0 0 0
0 0 0 0 0 0 0 0 0 0 0 0 0 0
0 0 0 0 0 0 0 0 0 0 0 0 0 0
0 0 0 0 0 0 0 0 0 0 0 0 0 0
0
OTU12989 0 0 0 0 0 0 0 0 0 0 0 1 0
0 0 0 0 0 0 0 0 0 0 0 0 0 0
0 0 0 0 0 0 0 1 0 0 0 0 0 0
0 0 0 0 0 0 0 0 0 0 0 0 0 0
0 0 0 0 0 0 0 0 0 0 0 0 0 0
0 0 0 0 0 0 0 0 0 0 0 0 0 0
0 0 0 0 0 0 0 0 0 0 0 0 0 0
1 0 0 0 0 0 0 0 0 0 0 0 1 0
0
OTU4398 0 0 0 0 0 0 0 0 0 0 0 1 0
0 0 0 0 1 1 0 1 0 0 0 0 0 0
0 0 0 0 0 0 0 0 0 0 0 0 0 0
1 0 0 0 0 0 0 0 0 0 0 0 0 0
0 0 0 0 0 0 0 0 0 0 0 0 0 0
0 0 0 0 0 0 0 1 0 0 0 1 0 0
0 0 0 0 0 0 0 0 0 0 0 0 0 0
0 0 0 0 0 0 0 0 1 0 0 0 0 0
0

OTU4314 0 0 0 0 0 0 0 0 0 0 0 1 0
0 0 0 0 0 0 0 0 0 0 0 0 0 0
0 0 0 0 0 0 0 0 0 0 0 0 0 0
0 0 0 0 0 0 0 0 0 0 0 0 0 0
0 0 0 0 0 0 0 0 0 0 0 0 0 0
0 0 0 0 0 0 0 0 0 0 0 0 0 0
0 0 0 0 0 0 0 0 0 0 0 0 0 0
0 0 0 0 0 0 0 0 0 0 0 0 0 0
0
OTU386 0 0 0 0 0 0 0 0 0 0 0 1 0
0 0 0 0 0 0 0 1 0 0 0 0 0 0
0 0 0 0 0 0 0 0 0 2 1 0 0 0
0 0 0 0 0 0 0 0 0 0 0 0 0 0
0 0 0 0 0 0 0 0 0 0 0 0 0 0
0 2 2 0 2 0 0 0 0 0 0 1 0 1
0 1 0 0 0 0 0 0 0 0 0 1 1 0
0 3 3 8 18 4 7 7 7 6 0 0 0 7
5
OTU12493 0 0 0 0 0 0 0 0 0 0 0 1 0
0 0 0 0 0 0 0 0 0 0 0 0 0 0
0 0 0 0 0 0 0 0 0 0 0 0 0 0
0 0 0 0 0 0 0 0 0 0 0 0 0 0
0 0 0 0 0 0 0 0 0 0 0 0 0 0
0 0 0 0 0 0 0 0 0 0 0 0 0 0
0 0 0 0 0 0 0 0 0 0 0 0 0 0
0 0 0 0 0 0 0 0 0 0 0 0 0 0
0
OTU4313 0 0 0 0 0 0 0 0 0 0 0 1 0
0 0 0 0 0 0 0 0 0 0 0 0 0 0
0 0 0 0 0 0 0 0 0 0 0 0 0 0
0 0 0 0 0 0 0 0 0 0 0 0 0 0
0 0 0 0 0 0 0 0 0 0 0 0 0 0
0 0 0 0 0 0 0 0 0 0 0 0 0 0

0	0	0	0	0	0	0	0	0	0	0	0	0	0
0	0	0	0	0	0	0	0	0	0	0	0	0	0
0													
OTU822	0	0	0	0	0	0	0	0	0	0	0	2	0
0	0	0	0	0	0	0	0	0	0	0	0	0	0
0	0	0	0	0	0	0	0	0	0	0	0	0	0
0	0	0	0	0	0	0	0	0	0	0	0	0	0
0	0	0	0	0	0	0	0	0	0	0	0	0	0
0	0	0	0	0	0	0	0	0	0	0	0	0	0
0	0	0	0	0	0	0	0	0	0	0	0	0	0
0	0	0	0	0	0	0	0	1	3	2	0	1	0
0													
OTU3884	0	0	0	0	0	0	0	0	0	0	0	3	0
0	0	0	0	0	0	0	0	2	0	1	0	4	0
0	0	0	0	1	0	2	1	0	0	13	0	0	0
1	0	0	1	0	0	0	0	0	0	1	0	0	0
0	0	1	0	1	0	4	0	0	2	0	2	0	0
0	0	0	0	0	0	0	0	0	0	0	0	0	0
0	0	0	0	0	0	1	1	1	0	0	0	0	0
0	0	0	0	0	0	0	0	1	0	0	4	4	0
0													
OTU12496	0	0	0	0	0	0	0	0	0	0	0	1	0
0	0	0	0	0	0	0	0	0	0	0	0	0	0
0	0	0	0	0	0	0	0	0	0	0	0	0	0
0	0	0	0	0	0	0	0	0	0	0	0	0	0
0	0	0	0	0	0	0	0	0	0	0	0	0	0
0	0	0	0	0	0	0	0	0	0	0	0	0	0
0	0	0	0	0	0	0	0	0	0	0	0	0	0
0	0	0	0	0	0	0	0	0	0	0	0	0	0
0													
OTU1272	0	0	0	0	0	0	0	0	0	0	0	1	0
0	0	0	0	0	0	0	0	0	0	0	0	0	0
0	0	0	0	0	0	0	0	0	0	0	0	0	0

0	0	0	0	0	0	0	0	0	0	0	0	0	0
0	0	0	0	0	0	0	0	0	0	0	0	0	0
0	0	0	0	0	1	0	0	0	0	0	0	0	1
0	0	0	0	0	0	0	0	0	0	0	0	0	0
0	0	0	0	0	0	0	0	0	0	0	0	0	0
0													
OTU12497	0	0	0	0	0	0	0	0	0	0	0	1	0
0	0	0	0	0	0	0	0	0	0	0	0	0	0
0	0	0	0	0	0	0	0	0	0	0	0	0	0
0	0	0	0	0	0	0	0	0	0	0	0	0	0
0	0	0	0	0	0	0	0	0	0	0	0	0	0
0	0	0	0	0	0	0	0	0	0	0	0	0	0
0	0	0	0	0	0	0	0	0	0	0	0	0	0
0	0	0	0	0	0	0	0	0	0	0	0	0	0
0													
OTU7704	0	0	0	0	0	0	0	0	0	0	0	1	0
0	0	0	0	0	0	0	0	0	0	0	0	0	0
0	0	0	0	0	0	0	0	0	0	0	0	0	0
0	0	0	0	0	0	0	0	0	0	0	0	0	0
0	0	0	0	0	0	0	0	0	0	0	0	0	0
0	0	0	0	0	0	0	0	0	0	0	0	0	0
0	0	0	0	0	1	0	0	0	0	0	0	0	0
0	0	0	0	0	0	0	0	0	0	0	0	0	0
0													
OTU7706	0	0	0	0	0	0	0	0	0	0	0	1	0
0	0	0	0	0	0	0	0	0	0	0	0	0	0
0	0	0	0	0	0	0	0	0	0	0	0	0	0
0	0	0	0	0	0	0	0	0	0	0	0	0	0
0	0	0	0	0	0	0	0	0	0	0	0	0	0
0	0	0	0	0	0	0	0	0	0	0	0	0	0
0	0	0	0	0	0	0	0	0	0	0	0	0	0
0	0	0	0	0	0	0	0	0	0	0	0	0	0
0													

OTU15689 0 0 0 0 0 0 0 0 0 0 0 1 0
0 0 0 0 0 0 0 0 0 0 0 0 0 0
0 0 0 0 0 0 0 0 0 0 0 0 0 0
0 0 0 0 0 0 0 0 0 0 0 0 0 0
0 0 0 0 0 0 0 0 0 0 0 0 0 0
0 0 0 0 0 0 0 0 0 0 0 0 0 0
0 0 0 0 0 0 0 0 0 0 0 0 0 0
0 0 0 0 0 0 0 0 0 0 0 0 0 0
0
OTU7705 0 0 0 0 0 0 0 0 0 0 0 1 0
0 0 0 0 0 0 0 0 0 0 0 0 0 0
0 0 0 0 0 0 0 0 0 0 0 0 0 0
0 0 0 0 0 0 0 0 0 0 0 0 0 0
0 0 0 0 0 0 0 0 0 0 0 0 0 0
0 0 0 0 0 0 0 0 0 0 0 0 0 0
0 0 0 0 0 0 0 0 0 0 0 0 0 0
0 0 0 0 0 0 0 0 0 0 0 0 0 0
0
OTU7695 0 0 0 0 0 0 0 0 0 0 0 1 0
0 0 0 0 0 0 0 0 0 0 0 0 0 0
0 0 0 0 0 0 0 0 0 0 0 0 0 0
0 0 0 0 0 0 0 0 0 0 0 0 0 0
0 0 0 0 0 0 0 0 0 0 0 0 0 0
0 0 0 0 0 0 0 0 0 0 0 0 0 0
0 0 0 0 0 0 0 0 0 0 0 0 0 0
0 0 0 0 0 0 0 0 0 0 0 0 0 0
0
OTU7696 0 0 0 0 0 0 0 0 0 0 0 1 0
0 0 0 0 0 0 0 0 0 0 0 0 0 0
0 0 0 0 0 0 0 0 0 0 0 0 0 0
0 0 0 0 0 0 0 0 0 0 0 0 0 0
0 0 0 0 0 0 0 0 0 0 0 0 0 0
0 0 0 0 0 0 0 0 0 0 0 0 0 0

0	0	0	0	0	0	0	0	0	0	0	0	0	0
0	0	0	0	0	0	0	0	0	0	0	0	0	0
0													
OTU3168	0	0	0	0	0	0	0	0	0	0	0	1	0
0	0	0	0	0	0	0	0	0	0	0	0	0	0
0	0	0	0	0	0	0	0	0	0	0	0	0	0
0	0	0	0	0	0	0	0	0	0	0	0	0	0
0	0	0	0	0	0	0	0	0	0	0	0	0	0
0	0	2	0	0	0	0	0	0	0	1	0	3	0
0	0	0	0	0	0	0	0	0	2	0	0	0	0
1	0	0	0	0	0	0	0	0	0	0	0	0	0
0													
OTU15691	0	0	0	0	0	0	0	0	0	0	0	1	0
0	0	0	0	0	0	0	0	0	0	0	0	0	0
0	0	0	0	0	0	0	0	0	0	0	0	0	0
0	0	0	0	0	0	0	0	0	0	0	0	0	0
0	0	0	0	0	0	0	0	0	0	0	0	0	0
0	0	0	0	0	0	0	0	0	0	0	0	0	0
0	0	0	0	0	0	0	0	0	0	0	0	0	0
0	0	0	0	0	0	0	0	0	0	0	0	0	0
0													
OTU7699	0	0	0	0	0	0	0	0	0	0	0	1	0
0	0	0	0	0	0	0	0	0	0	0	0	0	0
0	0	0	0	0	0	0	0	0	0	0	0	0	0
0	0	0	0	0	0	0	0	0	0	0	0	0	0
0	0	0	0	0	0	0	0	0	0	0	0	0	0
0	0	0	0	0	0	0	0	0	0	0	0	0	0
0	0	0	0	0	0	0	0	0	0	0	0	0	0
0	0	0	0	0	0	0	0	0	0	0	0	0	0
0													
OTU3759	0	0	0	0	0	0	0	0	0	0	0	1	0
0	0	0	0	0	0	0	0	0	0	0	1	0	0
0	0	0	0	0	0	0	0	0	0	0	0	0	0

0	0	0	0	0	1	0	0	0	0	0	0	0	0
0	0	0	0	0	0	0	0	0	0	0	0	0	0
0	0	0	0	0	0	0	0	0	0	0	0	0	0
0	0	0	0	0	0	0	0	0	0	0	0	0	0
0	0	0	0	0	0	0	0	0	0	0	0	0	0
0													
OTU15692	0	0	0	0	0	0	0	0	0	0	0	1	0
0	0	0	0	0	0	0	0	0	0	0	0	0	0
0	0	0	0	0	0	0	0	0	0	0	0	0	0
0	0	0	0	0	0	0	0	0	0	0	0	0	0
0	0	0	0	0	0	0	0	0	0	0	0	0	0
0	0	0	0	0	0	0	0	0	0	0	0	0	0
0	0	0	0	0	0	0	0	0	0	0	0	0	0
0	0	0	0	0	0	0	0	0	0	0	0	0	0
0													
OTU7698	0	0	0	0	0	0	0	0	0	0	0	1	0
0	0	0	0	0	0	0	0	0	0	0	0	0	0
0	0	0	0	0	0	0	0	0	0	0	0	0	0
0	0	0	0	0	0	0	0	0	0	0	0	0	0
0	0	0	0	0	0	0	0	0	0	0	0	0	0
0	0	0	0	0	0	0	0	0	0	0	0	0	0
0	0	0	0	0	0	0	0	0	0	0	0	0	0
0	0	0	0	0	0	0	0	0	0	0	0	0	0
0													
OTU7697	0	0	0	0	0	0	0	0	0	0	0	1	0
0	0	0	0	0	0	0	0	0	0	0	0	0	0
0	0	0	0	0	0	0	0	0	0	0	0	0	0
0	0	0	0	0	0	0	0	0	0	0	0	0	0
0	0	0	0	0	0	0	0	0	0	0	0	0	0
0	0	0	0	0	0	0	0	0	0	0	0	0	0
0	0	0	0	0	0	0	0	0	0	0	0	0	0
0	0	0	0	0	0	0	0	0	0	0	0	0	0
0													

OTU4287	0	0	0	0	0	0	0	0	0	0	0	1	0
0	0	0	0	0	0	0	0	0	0	0	0	0	0
0	0	0	0	0	0	0	0	0	0	0	0	0	0
0	0	0	0	0	0	0	0	0	0	0	0	0	0
0	0	0	0	0	0	0	0	0	0	0	0	0	0
0	0	1	0	0	0	0	0	0	0	0	0	0	0
0	0	0	0	0	0	0	0	0	0	0	0	0	0
0	0	0	0	0	0	0	0	0	0	0	0	0	0
0													
OTU3981	0	0	0	0	0	0	0	0	0	0	0	1	0
1	0	0	0	0	0	0	0	0	0	0	0	0	0
0	1	0	0	0	0	0	0	0	0	0	0	0	0
0	0	0	1	0	0	0	0	0	0	0	0	0	0
0	0	1	0	1	0	0	0	0	0	0	0	0	0
0	0	0	0	0	0	0	0	1	0	0	0	0	0
0	0	0	0	0	0	0	0	1	0	0	0	0	0
0	0	0	0	0	0	0	0	0	0	0	0	0	0
0													
OTU13958	0	0	0	0	0	0	0	0	0	0	0	2	0
0	0	0	1	0	0	0	0	0	0	0	0	0	0
0	0	0	0	0	0	0	0	0	0	0	0	0	0
0	0	0	0	0	0	0	0	0	0	1	0	0	0
0	0	0	0	0	0	0	0	0	7	0	2	0	0
0	0	0	0	0	0	0	0	0	0	0	0	0	0
0	0	0	0	0	0	0	0	0	0	0	0	0	0
0	0	0	0	0	0	0	0	0	0	0	2	1	0
0													
OTU1156	0	0	0	0	0	0	0	0	0	0	0	2	0
0	0	0	0	0	0	0	0	0	0	0	0	0	0
0	0	0	0	0	0	0	0	0	0	0	0	0	0
0	0	0	0	0	0	0	0	0	0	0	0	0	0
0	0	0	0	0	0	0	0	0	0	0	0	0	0
0	0	0	0	0	0	0	0	0	0	0	0	0	0

0	0	0	0	0	0	0	0	0	0	0	0	0	0
0	0	0	0	0	0	0	0	0	0	0	0	0	0
0													
OTU7693	0	0	0	0	0	0	0	0	0	0	0	1	0
0	0	0	0	0	0	0	0	0	0	0	0	0	0
0	0	0	0	0	0	0	0	0	0	0	0	0	0
0	0	0	0	0	0	0	0	0	0	0	0	0	0
0	0	0	0	0	0	0	0	0	0	0	0	0	0
0	0	0	0	0	0	0	0	0	0	0	0	0	0
0	0	0	0	0	0	0	0	0	0	0	0	0	0
0	0	0	0	0	0	0	0	0	0	0	1	0	0
0													
OTU1831	0	0	0	0	0	0	0	0	0	0	0	1	0
0	0	0	0	0	0	0	0	0	0	0	0	0	0
0	0	0	0	0	0	0	0	0	0	0	0	0	0
0	0	0	0	0	0	0	0	0	0	0	0	0	0
0	0	0	0	0	0	0	0	0	1	0	0	0	0
0	0	0	0	0	0	0	0	0	0	0	0	0	0
0	0	0	0	0	0	0	0	0	0	0	0	0	0
0	0	0	0	0	0	0	0	0	0	0	0	0	0
0													
OTU9940	0	0	0	0	0	0	0	0	0	0	0	1	0
0	1	0	0	0	0	0	0	0	1	0	0	0	0
0	0	0	0	0	0	0	1	0	0	0	0	1	1
2	0	0	0	0	0	0	0	0	0	0	0	0	0
0	0	1	0	0	0	0	0	0	0	0	0	0	0
0	0	0	0	0	1	0	0	0	0	0	0	1	1
0	0	0	0	0	0	0	0	0	0	1	0	0	0
0	0	0	0	0	0	0	0	0	0	0	0	0	0
0													
OTU4284	0	0	0	0	0	0	0	0	0	0	0	1	0
0	0	0	0	0	0	0	0	0	0	0	0	0	0
0	0	0	0	0	0	0	0	0	0	0	0	0	0

0	0	0	0	0	0	0	0	0	0	0	0	0	0
0	0	0	0	0	0	0	0	0	0	0	0	0	0
0	0	0	0	0	0	0	0	0	0	0	0	1	0
0	0	0	0	0	0	0	0	0	0	0	0	0	0
0	0	0	0	0	0	0	0	0	0	0	0	0	0
0													
OTU14961	0	0	0	0	0	0	0	0	0	0	0	1	0
0	0	0	0	0	0	0	0	0	0	0	0	0	0
0	0	0	0	0	0	0	0	0	0	0	0	0	0
0	0	0	0	0	0	0	0	0	0	0	0	0	0
0	0	0	0	0	0	0	0	0	0	0	0	0	0
0	0	0	0	0	0	0	0	0	0	0	0	0	0
0	0	0	0	0	0	0	0	0	0	0	0	0	0
0	0	0	0	0	0	0	0	0	1	0	0	0	0
0													
OTU12346	0	0	0	0	0	0	0	0	0	0	0	1	0
0	0	0	0	0	0	0	0	0	0	0	0	0	0
0	0	0	0	0	0	0	0	0	0	0	0	0	0
0	0	0	0	0	0	0	0	0	0	0	0	0	0
0	0	0	0	0	0	0	0	0	0	0	0	0	0
0	0	0	0	0	0	0	0	0	0	0	0	0	0
0	0	0	0	0	0	0	0	0	0	0	0	0	0
0	0	0	0	0	0	0	0	0	0	0	0	0	0
0													
OTU1408	0	0	0	0	0	0	0	0	0	0	0	3	0
1	0	0	1	0	0	0	0	0	0	0	0	0	0
0	0	0	0	0	0	0	0	0	0	0	0	0	0
0	0	0	0	0	0	0	0	0	0	0	0	0	0
0	0	1	0	0	0	0	0	0	1	0	0	0	0
0	0	2	0	0	0	0	0	0	0	0	1	0	0
0	0	0	0	0	0	0	0	0	0	0	0	0	1
0	0	0	0	0	0	0	0	0	0	0	1	1	0
0													

OTU15688	0	0	0	0	0	0	0	0	0	0	0	1	0
0	0	0	0	0	0	0	0	0	0	0	0	0	0
0	0	0	0	0	0	0	0	0	0	0	0	0	0
0	0	0	0	0	0	0	0	0	0	0	0	0	0
0	0	0	0	0	0	0	0	0	0	0	0	0	0
0	0	0	0	0	0	0	0	0	0	0	0	0	0
0	0	0	0	0	0	0	0	0	0	0	0	0	0
0	0	0	0	0	0	0	0	0	0	0	0	0	0
0													
OTU4285	0	0	0	0	0	0	0	0	0	0	0	1	0
0	0	0	0	0	0	0	0	0	0	0	0	0	0
0	0	0	0	0	0	0	0	0	0	0	0	0	0
0	0	0	0	0	0	0	0	0	0	0	0	0	0
0	0	0	0	0	0	0	0	0	0	0	0	0	0
0	0	0	0	0	0	0	0	0	0	0	0	0	0
0	0	0	0	0	0	0	0	0	0	0	0	0	0
0	0	0	0	0	0	0	0	0	0	0	0	0	0
0													
OTU7700	0	0	0	0	0	0	0	0	0	0	0	1	0
0	0	0	0	0	0	0	0	0	0	0	0	0	0
0	0	0	0	0	0	0	0	0	0	0	0	0	0
0	0	0	0	0	0	0	0	0	0	0	0	0	0
0	0	0	0	0	0	0	0	0	0	0	0	0	0
0	0	0	0	0	0	0	0	0	0	0	0	0	0
0	0	0	0	0	0	0	0	0	0	0	0	0	0
0	0	0	0	0	0	0	0	0	0	0	0	0	0
0													
OTU14693	0	0	0	0	0	0	0	0	0	0	0	1	0
1	0	0	0	1	0	0	0	0	0	0	0	0	0
0	0	0	0	0	0	0	2	0	3	0	1	0	0
0	1	0	5	0	0	0	0	0	0	0	0	0	0
0	0	0	0	0	0	0	0	0	0	0	0	0	0
0	0	0	0	0	0	0	0	0	0	0	0	3	0

0	0	0	0	0	0	0	0	0	0	1	0	0	0
7	0	0	0	0	0	0	0	0	0	0	0	1	0
0													
OTU738	0	0	0	0	0	0	0	0	0	0	0	1	0
0	0	0	0	0	0	0	0	0	0	0	0	0	0
0	0	0	0	0	0	2	0	0	0	0	0	0	0
306	0	5	0	0	0	0	1	0	0	0	7	0	0
0	0	0	0	0	0	0	0	0	0	0	0	0	0
0	2	0	0	0	0	0	3	0	20	0	0	0	0
0	4	0	0	0	0	0	0	0	0	0	0	0	0
0	7	0	0	4	1	7	2	0	5	0	0	0	3
14													
OTU4289	0	0	0	0	0	0	0	0	0	0	0	1	0
0	0	0	0	0	0	0	0	0	0	0	0	0	0
0	0	0	0	0	0	0	0	0	0	0	0	0	0
0	0	0	0	0	0	0	0	0	0	0	0	0	0
0	0	0	0	0	0	0	0	0	0	0	0	0	0
0	0	0	0	0	0	0	0	0	0	0	0	0	0
0	0	0	0	0	0	0	0	0	0	0	0	0	0
0	0	0	0	0	0	0	0	0	0	0	0	0	0
0													
OTU9143	0	0	0	0	0	0	0	0	0	0	0	1	0
0	0	0	0	0	0	0	0	0	0	0	0	0	0
0	0	0	0	0	0	0	0	0	0	0	0	0	0
0	0	0	0	0	0	0	0	0	0	0	0	0	0
0	0	0	0	0	0	0	0	0	0	0	0	0	0
0	0	0	0	0	0	0	0	0	0	0	0	0	0
0	0	0	0	0	0	0	0	0	0	1	0	0	0
0	0	0	0	1	0	0	0	0	0	0	0	0	0
0													
OTU14921	0	0	0	0	0	0	0	0	0	0	0	1	0
0	0	0	0	0	0	0	0	0	0	0	0	0	0
0	0	0	0	0	0	0	0	0	0	0	0	0	0

0	0	0	0	0	0	0	0	0	0	0	0	0	1
0	0	0	0	0	0	0	0	0	0	0	0	0	0
0	1	0	0	0	0	0	0	0	0	0	0	0	0
0	0	0	0	0	0	0	0	0	0	0	19	3	0
0	2	0	0	5	1	1	5	0	1	0	1	0	3
2													
OTU12349	0	0	0	0	0	0	0	0	0	0	0	1	0
0	0	0	0	0	0	0	0	0	0	0	0	0	0
0	0	0	0	0	0	0	0	0	0	0	0	0	0
0	0	0	0	0	0	0	0	0	0	0	0	0	0
0	0	0	0	0	0	0	0	0	0	0	0	0	0
0	0	0	0	0	0	0	0	0	0	0	0	0	0
0	0	0	0	0	0	0	0	0	0	0	0	0	0
0	0	0	0	0	0	0	0	0	0	0	0	0	0
0													
OTU15687	0	0	0	0	0	0	0	0	0	0	0	1	0
0	0	0	0	0	0	0	0	0	0	0	0	0	0
0	0	0	0	0	0	0	0	0	0	0	0	0	0
0	0	0	0	0	0	0	0	0	0	0	0	0	0
0	0	0	0	0	0	0	0	0	0	0	0	0	0
0	0	0	0	0	0	0	0	0	0	0	0	0	0
0	0	0	0	0	0	0	0	0	0	0	0	0	0
0	0	0	0	0	0	0	0	0	0	0	0	0	0
0													
OTU4715	0	0	0	0	0	0	0	0	0	0	0	2	0
0	1	0	0	0	0	0	0	0	0	0	0	0	0
0	0	0	0	0	0	0	0	1	0	0	0	0	0
0	0	0	0	0	0	0	1	0	0	0	0	0	2
0	1	0	0	0	0	0	0	0	0	0	0	0	0
0	0	0	0	0	0	0	0	0	0	0	0	0	0
0	0	0	0	0	0	0	0	0	0	0	0	0	0
0	0	0	0	0	0	0	0	0	0	0	0	0	0
0													

OTU13481	0	0	0	0	0	0	0	0	0	0	0	1	0
2	0	0	1	0	0	0	0	0	0	0	0	0	0
0	0	0	0	0	0	0	0	0	0	0	0	0	0
0	0	0	0	0	0	0	0	0	0	0	0	0	0
0	0	1	0	0	0	0	0	0	1	0	0	0	0
0	0	0	0	0	0	0	0	0	0	0	0	0	0
0	0	1	0	1	0	0	0	0	1	1	0	0	2
0	0	0	0	0	0	0	0	0	0	1	1	0	0
0													
OTU610	0	0	0	0	0	0	0	0	0	0	0	1	0
0	0	1	0	1	0	0	6	0	0	0	0	0	0
0	0	0	0	0	0	1	0	0	5	0	0	0	0
0	0	0	1	0	0	0	0	0	0	0	0	0	0
0	0	0	0	0	0	2	0	0	0	0	0	0	0
0	0	0	0	0	0	0	0	0	0	0	0	0	0
0	0	0	0	1	0	0	0	0	0	0	0	2	1
1	0	0	0	0	0	1	1	0	2	1	0	3	4
0													
OTU12347	0	0	0	0	0	0	0	0	0	0	0	1	0
0	0	0	0	0	0	0	0	0	0	0	0	0	0
0	0	0	0	0	0	0	0	0	0	0	0	0	0
0	0	0	0	0	0	0	0	0	0	0	0	0	0
0	0	0	0	0	0	0	0	0	0	0	0	0	0
0	0	0	0	0	0	0	0	0	0	0	0	0	0
0	0	0	0	0	0	0	0	0	0	0	0	0	0
0	0	0	0	0	0	0	0	0	0	0	0	0	0
0													
OTU16812	0	0	0	0	0	0	0	0	0	0	0	1	0
0	0	0	0	0	0	0	0	0	0	0	0	0	0
0	0	0	0	0	0	0	1	0	0	0	0	0	0
0	3	0	1	0	0	0	0	0	0	0	0	0	0
0	0	0	0	0	0	0	0	0	0	0	0	0	0
0	0	0	0	0	0	0	0	2	0	0	0	1	0

1	0	0	0	2	0	0	0	0	0	1	0	0	0
0	0	0	0	0	0	0	0	0	0	0	0	0	0
0													
OTU2757	0	0	0	0	0	0	0	0	0	0	0	1	0
0	0	0	0	0	0	0	0	0	0	0	0	0	0
0	0	0	0	0	0	0	0	0	0	0	0	0	0
0	0	0	0	0	0	0	0	0	0	0	0	0	1
0	0	0	0	0	0	0	0	0	0	0	0	0	0
0	0	0	0	0	0	0	0	0	0	0	0	0	0
0	0	0	0	0	0	1	0	0	0	0	0	0	0
1	0	0	0	0	0	0	0	0	0	0	0	0	0
0													
OTU12341	0	0	0	0	0	0	0	0	0	0	0	1	0
0	0	0	0	0	0	0	0	0	0	0	0	0	0
0	0	0	0	0	0	0	0	0	0	0	0	0	0
0	0	0	0	0	0	0	0	0	0	0	0	0	0
0	0	0	0	0	0	0	0	0	0	0	0	0	0
0	0	0	0	0	0	0	0	0	0	0	0	0	0
0	0	0	0	0	0	0	0	0	0	0	0	0	0
0	0	0	0	0	0	0	0	0	0	0	0	0	0
0													
OTU4278	0	0	0	0	0	0	0	0	0	0	0	1	0
0	0	0	0	0	0	0	0	0	0	0	0	0	0
0	0	0	0	0	0	0	0	0	0	0	0	0	0
0	0	0	0	0	0	0	0	0	0	0	0	0	0
0	0	0	0	0	0	0	0	0	0	0	0	0	0
0	0	0	0	0	0	0	0	0	0	0	0	0	0
0	0	0	0	0	0	0	0	0	0	0	0	0	0
0	0	0	0	0	0	0	0	0	0	0	0	0	0
0													
OTU8064	0	0	0	0	0	0	0	0	0	0	0	1	0
0	0	0	0	0	0	0	0	0	0	0	0	0	0
0	0	0	0	0	0	0	0	0	0	0	0	0	0

0	0	0	0	0	0	0	0	0	0	0	0	0	0
0	0	0	0	0	0	0	0	0	0	0	0	0	0
0	0	0	0	0	0	0	0	0	0	0	0	0	0
0	0	0	0	0	0	0	0	0	0	0	0	0	0
0	0	0	0	0	0	0	0	0	0	0	0	0	0
0													
OTU8065	0	0	0	0	0	0	0	0	0	0	0	1	0
0	0	0	0	0	0	0	0	0	0	0	0	0	0
0	0	0	0	0	0	0	0	0	0	0	0	0	0
0	0	0	0	0	0	0	0	0	0	0	0	0	0
0	0	0	0	0	0	0	0	0	0	0	0	0	0
0	0	0	0	0	0	0	0	0	0	0	0	0	0
0	0	0	0	0	0	0	0	0	0	0	0	0	0
0	0	0	0	0	0	0	0	0	0	0	0	0	0
0													
OTU9726	0	0	0	0	0	0	0	0	0	0	0	1	0
0	0	0	0	0	0	0	1	0	0	0	0	0	0
0	0	0	0	0	0	0	0	0	1	0	0	0	0
0	4	0	1	0	0	0	0	0	0	0	0	0	0
0	0	0	2	0	0	0	0	0	0	0	0	0	0
0	0	0	3	0	0	5	0	0	0	0	0	0	0
0	0	0	0	0	0	0	0	0	0	0	0	0	0
0	0	0	0	0	0	0	0	0	0	0	0	0	0
0													
OTU935	0	0	0	0	0	0	0	0	0	0	0	1	0
0	0	0	0	0	0	0	0	0	0	3	0	0	0
0	0	0	0	0	0	0	0	0	0	0	0	0	0
0	0	0	0	0	0	0	0	0	0	0	0	0	0
0	0	0	0	0	0	0	0	0	1	0	0	0	0
0	0	0	0	0	0	0	0	1	0	0	0	0	0
0	0	0	0	0	0	0	0	0	0	0	0	0	0
0	0	0	0	0	0	1	0	0	0	0	0	0	0
0													

OTU12342	0	0	0	0	0	0	0	0	0	0	0	1	0
0	0	0	0	0	0	0	0	0	0	0	0	0	0
0	0	0	0	0	0	0	0	0	0	0	0	0	0
0	0	0	0	0	0	0	0	0	0	0	0	0	0
0	0	0	0	0	0	0	0	0	0	0	0	0	0
0	0	0	0	0	0	0	0	0	0	0	0	0	0
0	0	0	0	0	0	0	0	0	0	0	0	0	0
0	0	0	0	0	0	0	0	0	0	0	0	0	0
0													
OTU8062	0	0	0	0	0	0	0	0	0	0	0	1	0
0	0	0	0	0	0	0	0	0	0	0	0	0	0
0	0	0	0	0	0	0	0	0	0	0	0	0	0
0	0	0	0	0	0	0	0	0	0	0	0	0	0
0	0	0	0	0	0	0	0	0	0	0	0	0	0
0	0	0	0	0	0	0	0	0	0	0	0	0	0
0	0	0	0	0	0	0	0	0	0	0	0	0	0
0	0	0	0	0	0	0	0	0	0	0	0	0	0
0													
OTU4512	0	0	0	0	0	0	0	0	0	0	0	1	6
1	0	1	11	1	1	0	1	1	0	14	0	0	1
0	3	3	4	5	3	5	3	1	1	2	0	0	0
0	0	3	2	1	0	0	0	0	3	1	0	0	0
2	0	3	0	1	0	3	0	0	2	0	0	0	7
0	0	0	2	0	5	0	0	5	0	1	0	1	0
0	0	4	0	2	0	3	0	2	0	0	0	0	2
2	0	0	0	0	0	0	0	0	0	3	2	6	0
0													
OTU629	0	0	0	0	0	0	0	0	0	0	0	1	0
0	1	0	1	0	0	2	0	0	0	0	0	0	0
0	0	0	0	1	0	1	1	2	0	27	1	1	0
4	0	0	0	0	5	0	0	0	0	0	0	0	0
0	0	0	0	0	0	0	0	0	0	0	0	0	0
0	0	0	0	0	0	0	0	0	0	0	0	1	0

0	0	0	0	0	0	0	0	0	0	0	5	0	1
0	5	0	0	20	0	8	5	0	0	0	2	0	11
5													
OTU12339	0	0	0	0	0	0	0	0	0	0	0	1	0
0	0	0	0	0	0	0	0	0	0	0	0	0	0
0	0	0	0	0	0	0	0	0	0	0	0	0	0
0	0	0	0	0	0	0	0	0	0	0	0	0	0
0	0	0	0	0	0	0	0	0	0	0	0	0	0
0	0	0	0	0	0	0	0	0	0	0	0	0	0
0	0	0	0	0	0	0	0	0	0	0	0	0	0
0	0	0	0	0	0	0	0	0	0	0	0	0	0
0													
OTU458	0	0	0	0	0	0	0	0	0	0	0	1	0
0	0	0	0	0	0	0	0	0	0	0	0	0	0
0	0	0	0	0	0	0	0	0	0	0	0	0	0
0	2	0	0	0	0	0	0	0	0	0	0	0	0
0	0	0	0	0	0	0	0	0	0	0	0	0	0
0	3	0	0	0	0	0	0	0	0	0	0	0	0
0	0	0	0	0	0	0	0	0	0	0	4	0	0
0	23	0	0	11	0	0	0	0	0	2	0	0	0
0													
OTU3064	0	0	0	0	0	0	0	0	0	0	0	1	0
0	0	0	0	0	0	0	0	0	0	0	0	0	0
0	0	0	0	0	0	0	0	0	0	0	0	0	0
0	0	0	0	0	0	0	0	0	0	0	0	0	0
0	0	0	0	0	0	0	0	0	0	0	0	0	0
0	0	0	0	0	0	0	0	0	0	0	0	0	0
1	0	0	0	0	0	0	0	0	0	0	0	0	0
0	0	0	0	0	0	0	0	0	0	0	0	0	0
0													
OTU8063	0	0	0	0	0	0	0	0	0	0	0	1	0
0	0	0	0	0	0	0	0	0	0	0	0	0	0
0	0	0	0	0	0	0	0	0	0	0	0	0	0

0	0	0	0	0	0	0	0	0	0	0	0	0	0
0	0	0	0	0	0	0	0	0	0	0	0	0	0
0	0	0	0	0	0	0	0	0	0	0	0	0	0
0	0	0	0	0	0	0	0	0	0	0	0	0	0
0	0	0	0	0	0	0	0	0	0	0	0	0	0
0													
OTU4280	0	0	0	0	0	0	0	0	0	0	0	1	0
0	0	0	0	0	0	0	0	0	0	0	0	0	0
0	0	0	0	0	0	0	0	0	0	0	0	0	0
0	0	0	0	0	0	0	0	0	0	0	0	0	0
0	0	0	0	0	0	0	0	0	0	0	0	0	0
0	0	0	0	0	0	0	0	0	0	0	0	0	0
0	0	0	0	0	0	0	0	0	0	0	0	0	0
0	0	0	0	0	0	0	0	0	0	0	0	0	0
0													
OTU182	0	0	0	0	0	0	0	0	0	0	0	2	0
0	2	1	1	5	0	1	1	0	0	0	0	1	1
1	0	0	0	0	1	0	1	0	0	0	0	0	0
0	4	0	6	2	1	1	16	0	0	3	4	2	2
1	1	0	0	0	0	1	0	2	0	0	2	0	3
0	940	0	0	0	2	0	0	1	0	8	0	3	0
1	1	1	0	0	0	0	0	0	1	0	1324	195	1
1	1017	3	322	205	7	101	30	72	33	3	3	3	14
188													
OTU9003	0	0	0	0	0	0	0	0	0	0	0	1	0
0	0	0	0	0	0	0	0	0	0	0	0	0	0
0	0	0	0	0	0	0	0	0	0	0	0	0	0
1	0	0	0	0	0	0	0	0	0	0	0	0	0
0	0	0	0	0	0	0	0	0	0	0	0	0	0
0	0	0	0	0	0	0	0	0	0	0	0	0	0
0	0	0	0	0	0	0	0	0	0	0	0	0	0
0	0	0	0	0	0	0	0	0	0	0	0	0	0
0													

OTU12340	0	0	0	0	0	0	0	0	0	0	0	1	0
0	0	0	0	0	0	0	0	0	0	0	0	0	0
0	0	0	0	0	0	0	0	0	0	0	0	0	0
0	0	0	0	0	0	0	0	0	0	0	0	0	0
0	0	0	0	0	0	0	0	0	0	0	0	0	0
0	0	0	0	0	0	0	0	0	0	0	0	0	0
0	0	0	0	0	0	0	0	0	0	0	0	0	0
0	0	0	0	0	0	0	0	0	0	0	0	0	0
0													
OTU15665	0	0	0	0	0	0	0	0	0	0	0	1	0
0	0	0	0	0	0	0	0	0	0	0	0	0	0
0	0	0	0	0	0	0	0	0	0	0	0	0	0
0	0	0	0	0	0	0	0	0	0	0	0	0	0
0	0	0	0	0	0	0	0	0	0	0	0	0	0
0	0	0	0	0	0	0	0	0	0	0	0	0	0
0	0	0	0	0	0	0	0	0	0	0	0	0	0
0	0	0	0	0	0	0	0	0	0	0	0	0	0
0													
OTU8056	0	0	0	0	0	0	0	0	0	0	0	1	0
0	0	0	0	0	0	2	0	0	0	0	0	0	0
0	0	0	0	0	0	0	0	0	0	0	0	0	0
0	0	0	0	0	0	0	0	0	0	0	0	0	0
0	0	0	0	0	0	0	0	0	0	0	0	0	0
0	0	0	0	0	0	0	0	0	0	0	0	0	0
0	0	0	0	0	0	0	2	0	0	0	0	0	0
1	0	0	0	0	0	0	0	0	0	0	0	0	0
0													
OTU12345	0	0	0	0	0	0	0	0	0	0	0	1	0
0	0	0	0	0	0	0	0	0	0	0	0	0	0
0	0	0	0	0	0	0	0	0	0	0	0	0	0
0	0	0	0	0	0	0	0	0	0	0	0	0	0
0	0	0	0	0	0	0	0	0	0	0	0	0	0
0	0	0	0	0	0	0	0	0	0	0	0	0	0

0	0	0	0	0	0	0	0	0	0	0	0	0	0
0	0	0	0	0	0	0	0	0	0	0	0	0	0
0													
OTU2480	0	0	0	0	0	0	0	0	0	0	0	1	0
0	0	1	0	0	0	0	0	0	0	0	0	0	0
0	0	0	0	0	0	1	1	0	0	0	0	0	0
0	0	0	0	0	0	0	0	0	0	0	0	0	1
0	0	0	0	0	0	0	0	0	0	0	0	0	0
0	0	0	0	0	0	0	0	0	0	0	0	1	0
0	0	1	0	0	0	0	0	0	0	5	0	0	2
0	0	0	0	0	0	0	0	0	0	1	1	0	0
0													
OTU8815	0	0	0	0	0	0	0	0	0	0	0	1	0
2	0	0	0	0	1	0	0	0	0	1	3	0	0
0	0	0	0	0	0	0	2	0	0	0	0	0	0
0	0	0	0	0	0	0	0	0	0	0	0	0	0
0	0	0	1	0	0	0	0	0	0	0	1	0	0
0	0	0	0	0	0	0	0	0	0	0	0	3	0
0	0	0	0	0	0	0	0	0	0	1	0	0	0
3	0	0	0	0	0	0	0	0	0	0	0	2	0
0													
OTU16571	0	0	0	0	0	0	0	0	0	0	0	1	0
0	0	0	0	0	0	1	0	1	0	0	0	0	1
0	0	0	0	0	0	0	4	1	0	0	0	0	0
0	0	0	0	1	2	0	0	0	0	1	0	0	0
1	0	1	0	0	0	0	0	0	1	0	1	0	0
0	0	0	0	0	0	0	0	0	0	0	0	0	0
0	0	0	0	0	0	0	0	0	0	1	0	0	0
2	0	0	0	0	0	0	0	0	0	0	1	0	0
0													
OTU15663	0	0	0	0	0	0	0	0	0	0	0	1	0
0	0	0	0	0	0	0	0	0	0	0	0	0	0
0	0	0	0	0	0	0	0	0	0	0	0	0	0

0	0	0	0	0	0	0	0	0	0	0	0	0	0
0	0	0	0	0	0	0	0	0	0	0	0	0	0
0	0	0	0	0	0	0	0	0	0	0	0	0	0
0	0	0	0	0	0	0	0	0	0	0	0	0	0
0	0	0	0	0	0	0	0	0	0	0	0	0	0
0													
OTU4296	0	0	0	0	0	0	0	0	0	0	0	1	0
0	0	0	0	0	0	0	0	0	0	0	0	0	0
0	0	0	0	0	0	0	0	0	0	0	0	0	0
0	0	0	0	0	0	0	0	0	0	0	0	0	0
0	0	0	0	0	0	0	0	0	0	0	0	0	0
0	0	0	0	0	0	0	0	0	0	0	0	0	0
0	0	0	0	0	0	0	0	0	0	0	0	0	0
0	0	0	0	0	0	0	0	0	0	0	0	0	0
0													
OTU12343	0	0	0	0	0	0	0	0	0	0	0	1	0
0	0	0	0	0	0	0	0	0	0	0	0	0	0
0	0	0	0	0	0	0	0	0	0	0	0	0	0
0	0	0	0	0	0	0	0	0	0	0	0	0	0
0	0	0	0	0	0	0	0	0	0	0	0	0	0
0	0	0	0	0	0	0	0	0	0	0	0	0	0
0	0	0	0	0	0	0	0	0	0	0	0	0	0
0	0	0	0	0	0	0	0	0	0	0	0	0	0
0													
OTU12344	0	0	0	0	0	0	0	0	0	0	0	1	0
0	0	0	0	0	0	0	0	0	0	0	0	0	0
0	0	0	0	0	0	0	0	0	0	0	0	0	0
0	0	0	0	0	0	0	0	0	0	0	0	0	0
0	0	0	0	0	0	0	0	0	0	0	0	0	0
0	0	0	0	0	0	0	0	0	0	0	0	0	0
0	0	0	0	0	0	0	0	0	0	0	0	0	0
0	0	0	0	0	0	0	0	0	0	0	0	0	0
0													

OTU3899	0	0	0	0	0	0	0	0	0	0	0	1	0
6	0	0	0	0	0	0	0	0	0	0	0	0	7
0	0	0	0	0	0	0	2	0	0	0	3	0	0
0	0	0	0	0	0	0	0	0	0	0	0	0	0
0	0	1	0	0	0	0	1	0	0	0	0	0	0
0	0	0	0	0	0	0	0	0	0	0	0	0	0
0	0	4	0	0	0	0	0	7	0	1	0	0	0
0	0	0	0	0	0	0	0	0	0	0	1	6	0
0													
OTU15839	0	0	0	0	0	0	0	0	0	0	0	0	1
0	0	0	0	1	0	0	0	0	0	0	0	0	0
0	0	0	1	0	0	0	0	0	0	0	0	0	0
0	0	0	0	0	0	0	0	0	0	0	0	0	0
0	0	0	0	0	0	0	0	0	0	0	0	0	0
1	0	0	0	0	0	0	0	0	0	0	0	0	0
0	0	0	0	0	0	0	0	0	0	0	0	0	0
0	0	0	0	0	1	0	0	0	0	0	0	0	0
0													
OTU5416	0	0	0	0	0	0	0	0	0	0	0	0	1
0	0	0	0	0	0	0	0	0	0	0	0	0	0
0	0	0	0	0	0	0	0	0	3	0	0	1	0
0	0	0	0	0	0	0	0	0	0	0	0	0	0
0	0	0	0	0	0	0	0	0	0	0	0	0	0
0	0	0	0	0	0	0	0	0	0	0	0	0	0
0	0	0	1	0	0	0	0	0	0	0	0	0	0
0	0	0	0	0	0	0	0	0	0	0	0	0	0
0													
OTU2682	0	0	0	0	0	0	0	0	0	0	0	0	3
7	0	1	4	0	1	0	0	0	0	0	0	0	0
0	2	5	1	5	0	7	0	0	0	0	0	0	0
0	1	18	1	8	2	0	0	9	0	1	0	55	0
10	2	31	0	0	10	32	10	1	12	3	1	2	2
2	0	0	2	0	2	3	0	0	0	0	0	0	1

0	0	0	0	0	0	1	0	13	0	0	0	0	49
1	0	0	0	1	1	0	0	0	0	0	1	6	0
0													
OTU2741	0	0	0	0	0	0	0	0	0	0	0	0	2
0	0	0	0	0	0	0	0	0	0	0	0	0	0
0	0	0	0	0	0	0	0	0	0	0	0	0	0
0	1	0	0	0	0	1	0	0	0	0	0	0	1
0	0	0	0	0	0	0	0	0	0	1	1	0	0
0	0	0	0	0	1	0	0	0	0	0	0	0	0
0	0	0	0	1	0	0	0	0	0	0	0	0	0
1	0	0	0	0	0	0	0	0	0	1	0	0	0
0													
OTU739	0	0	0	0	0	0	0	0	0	0	0	0	16
2	0	0	0	0	0	0	0	0	0	0	0	0	0
0	0	2	0	0	0	0	0	0	0	0	0	0	0
0	0	0	0	2	2	2	0	0	0	0	3	0	0
2	0	0	0	0	13	0	0	0	0	1	0	0	0
0	0	0	0	0	0	0	0	0	0	0	0	0	0
0	0	1	0	0	0	0	0	0	1	0	0	0	2
0	0	0	0	0	0	0	0	0	0	0	2	0	0
0													
OTU4291	0	0	0	0	0	0	0	0	0	0	0	0	12
3	0	0	0	0	2	0	0	0	0	0	0	1	0
0	0	0	0	0	0	0	0	0	0	0	0	0	7
0	3	0	0	0	0	0	0	0	0	1	0	0	1
0	0	0	0	0	0	0	0	0	0	0	2	0	0
0	0	0	0	0	0	0	0	0	0	0	0	0	0
0	0	0	0	0	0	0	0	1	0	0	0	0	2
1	0	0	0	0	0	0	0	0	0	0	0	0	0
0													
OTU1012	0	0	0	0	0	0	0	0	0	0	0	0	7
0	0	0	0	0	0	0	0	0	0	0	0	0	1
0	0	0	0	0	0	0	0	0	0	0	0	0	0

0 0 0 0 0 0 0 0 0 0 0 0 0 0
0 0 0 0 0 0 0 0 0 0 0 0 0 0
0 0 0 0 0 0 0 0 0 0 0 0 0 0
0 0 0 0 0 0 0 0 20 0 0 0 0 0
0 0 0 0 0 0 0 0 0 0 0 0 0 0
0
OTU16343 0 0 0 0 0 0 0 0 0 0 0 0 1
0 0 0 0 0 0 0 0 0 0 0 0 0 0
0 0 0 0 0 0 1 0 0 0 1 0 0 0
0 0 0 0 0 0 0 0 0 0 0 0 0 0
0 0 2 0 0 0 0 0 0 1 0 1 0 0
0 0 0 0 0 0 0 0 0 0 0 0 0 0
0 0 2 0 0 0 0 0 1 0 0 0 0 0
0 0 0 0 0 0 0 0 0 0 0 0 2 0
0
OTU15666 0 0 0 0 0 0 0 0 0 0 0 0 1
0 0 0 0 0 0 0 0 0 0 0 0 0 0
0 0 0 0 0 0 0 0 0 0 0 0 0 0
0 0 0 0 0 0 0 0 0 0 0 0 0 0
0 0 0 0 0 0 0 0 0 0 0 0 0 0
0 0 0 0 0 0 0 0 0 0 0 0 0 0
0 0 0 0 0 0 0 0 0 0 0 0 0 0
0 0 0 0 0 0 0 0 0 0 0 0 0 0
0
OTU3626 0 0 0 0 0 0 0 0 0 0 0 0 1
0 0 0 0 0 0 0 0 0 0 0 0 0 1
0 0 0 0 0 0 0 0 0 0 0 0 0 0
0 0 0 0 0 0 0 0 0 0 0 0 0 0
0 0 0 0 0 0 0 0 0 0 0 0 0 0
0 0 0 0 0 0 0 0 0 0 0 0 0 0
0 0 0 0 0 0 0 0 0 0 0 0 0 0
0 0 0 0 0 0 0 0 0 0 0 0 0 0
0

OTU6906	0	0	0	0	0	0	0	0	0	0	0	0	1
2	0	0	0	0	0	0	0	0	0	0	0	0	0
0	0	0	0	1	0	0	0	0	0	0	0	0	1
0	0	1	0	0	0	0	0	0	0	0	0	0	0
0	0	0	0	0	0	0	0	0	0	0	1	0	2
0	0	0	0	0	0	0	0	0	0	0	0	0	0
0	0	0	0	0	0	0	0	0	1	0	0	0	2
0	0	0	0	0	0	0	0	0	0	0	0	0	0
0													
OTU10329	0	0	0	0	0	0	0	0	0	0	0	0	1
0	0	0	0	0	0	0	0	0	0	0	0	0	0
0	0	0	0	0	0	0	0	0	1	0	0	0	0
0	0	0	0	0	0	0	0	0	0	0	0	0	0
0	0	0	0	0	0	0	0	0	0	0	0	0	0
0	0	0	1	0	0	0	0	1	0	0	0	1	0
0	0	0	0	0	0	0	0	0	0	0	0	1	0
0	0	0	0	0	0	0	0	0	0	0	0	0	0
0													
OTU1120	0	0	0	0	0	0	0	0	0	0	0	0	1
2	0	0	0	0	0	0	0	0	0	0	0	0	0
0	0	0	0	0	0	0	0	0	0	0	0	0	1
0	0	0	0	0	0	0	0	0	0	0	0	0	0
0	0	0	0	0	0	0	0	0	0	0	0	0	0
0	0	0	0	0	0	0	0	0	0	0	0	0	0
0	0	0	0	0	0	0	0	0	0	0	0	0	0
0	0	0	0	0	0	0	0	0	0	0	0	0	0
0													
OTU8059	0	0	0	0	0	0	0	0	0	0	0	0	1
0	0	0	0	0	0	0	0	0	0	0	0	0	0
0	0	0	0	0	0	0	0	0	0	0	0	0	0
0	0	0	0	0	0	0	0	0	0	0	0	0	0
0	0	0	0	0	0	0	0	0	0	0	0	0	0
0	0	0	0	0	0	0	0	0	0	0	0	0	0

0	0	0	0	0	0	0	0	0	0	0	0	0	0
0	0	0	0	0	0	0	0	0	0	0	0	0	0
0													
OTU880	0	0	0	0	0	0	0	0	0	0	0	0	9
0	0	0	0	0	0	1	0	0	0	0	0	0	0
2	1	0	0	0	0	0	0	2	0	1	0	0	0
1	1	0	0	0	0	0	1	0	1	0	0	2	0
8	0	0	0	0	0	0	0	0	0	0	0	0	1
0	0	0	1	0	0	0	0	0	0	0	0	0	0
2	0	0	0	0	0	0	0	0	0	1	0	0	0
0	0	0	1	0	0	0	0	0	0	40	0	0	0
0													
OTU15668	0	0	0	0	0	0	0	0	0	0	0	0	1
0	0	0	0	0	0	0	0	0	0	0	0	0	0
0	0	0	0	0	0	0	0	0	0	0	0	0	0
0	0	0	0	0	0	0	0	0	0	0	0	0	0
0	0	0	0	0	0	0	0	0	0	0	0	0	0
0	0	0	0	0	0	0	0	0	0	0	0	0	0
0	0	0	0	0	0	0	0	0	0	0	0	0	0
0	0	0	0	0	0	0	0	0	0	0	0	0	0
0													
OTU15667	0	0	0	0	0	0	0	0	0	0	0	0	1
0	0	0	0	0	0	0	0	0	0	0	0	0	0
0	0	0	0	0	0	0	0	0	0	0	0	0	0
0	0	0	0	0	0	0	0	0	0	0	0	0	0
0	0	0	0	0	0	0	0	0	0	0	0	0	0
0	0	0	0	0	0	0	0	0	0	0	0	0	0
0	0	0	0	0	0	0	0	0	0	0	0	0	0
0	0	0	0	0	0	0	0	0	0	0	0	0	0
0													
OTU8071	0	0	0	0	0	0	0	0	0	0	0	0	1
0	0	0	0	0	0	0	0	0	0	0	0	0	0
0	0	0	0	0	0	0	0	0	0	0	0	0	0

0	0	0	0	0	0	0	0	0	0	0	0	0	0
0	0	0	0	0	0	0	0	0	0	0	0	0	0
0	0	0	0	0	0	0	0	0	0	0	0	0	0
0	0	0	0	0	0	0	0	0	0	0	0	0	0
0	0	0	0	0	0	0	0	0	0	0	0	0	0
0													
OTU8070	0	0	0	0	0	0	0	0	0	0	0	0	1
0	0	0	0	0	0	0	0	0	0	0	0	0	0
0	0	0	0	0	0	0	0	0	0	0	0	0	0
0	0	0	0	0	0	0	0	0	0	0	0	0	0
0	0	0	0	0	0	0	0	0	0	0	0	0	0
0	0	0	0	0	0	0	0	0	0	0	0	0	0
0	0	0	0	0	0	0	0	0	0	0	0	0	0
0	0	0	0	0	0	0	0	0	0	0	0	0	0
0													
OTU12355	0	0	0	0	0	0	0	0	0	0	0	0	1
0	0	0	0	0	0	0	0	0	0	0	0	0	0
0	0	0	0	0	0	0	0	0	0	0	0	0	0
0	0	0	0	0	0	0	0	0	0	0	0	0	0
0	0	0	0	0	0	0	0	0	0	0	0	0	0
0	0	0	0	0	0	0	0	0	0	0	0	0	0
0	0	0	0	0	0	0	0	0	0	0	0	0	0
0	0	0	0	0	0	0	0	0	0	0	0	0	0
0													
OTU1713	0	0	0	0	0	0	0	0	0	0	0	0	4
0	1	0	0	0	0	0	1	0	0	0	0	0	0
0	0	2	0	0	0	0	0	0	0	0	0	0	2
0	0	0	1	0	0	0	0	0	0	0	0	0	0
0	0	0	0	0	0	0	0	0	0	0	0	0	0
0	0	0	1	0	0	2	0	0	0	1	0	0	0
1	0	1	0	0	0	0	0	0	0	0	0	0	0
1	0	0	0	0	0	0	0	0	0	0	0	0	0
0													

OTU4292	0	0	0	0	0	0	0	0	0	0	0	0	1
0	0	0	0	0	0	0	0	0	0	0	0	0	0
0	0	0	0	0	0	0	0	0	0	0	0	0	0
0	0	0	0	0	0	0	0	0	0	0	0	0	0
0	0	0	0	0	0	0	0	0	0	0	0	0	0
0	0	0	0	0	0	0	0	0	0	0	0	0	0
0	0	0	0	0	0	0	0	0	0	0	0	0	0
0	0	0	0	0	0	0	0	0	0	0	0	0	0
0													
OTU657	0	0	0	0	0	0	0	0	0	0	0	0	1
0	0	0	0	0	0	1	0	0	0	2	0	0	0
0	0	0	0	0	0	1	0	0	0	0	0	8	0
0	0	0	0	0	0	1	0	0	0	0	0	0	0
0	0	0	0	0	0	0	0	0	2	0	0	0	0
0	0	0	0	0	0	0	0	0	0	0	0	0	0
0	0	0	0	0	0	0	0	0	0	0	0	0	0
0	0	0	0	0	0	0	0	0	0	0	0	0	0
0													
OTU8068	0	0	0	0	0	0	0	0	0	0	0	0	11
1	0	0	0	0	0	0	0	0	0	0	0	0	0
0	0	0	0	0	0	0	0	0	0	0	0	0	0
0	0	0	0	0	0	0	0	0	0	0	0	0	0
0	0	0	0	0	0	0	0	0	0	0	0	0	0
0	0	0	0	0	0	0	0	0	0	0	0	0	0
0	0	0	0	0	0	0	0	0	0	0	0	0	0
0	0	0	0	0	0	0	0	0	0	0	0	0	0
0													
OTU3488	0	0	0	0	0	0	0	0	0	0	0	0	1
0	0	0	0	0	0	0	0	0	0	0	0	0	0
0	0	0	0	0	0	0	0	0	0	0	0	0	0
0	0	0	0	0	0	0	0	0	0	0	0	0	0
0	0	0	0	0	0	0	0	0	0	0	0	0	0
0	0	0	0	0	0	0	0	0	0	0	0	0	0

0	0	1	0	0	0	0	0	0	0	0	0	0	0
0	0	0	0	0	0	0	0	0	0	0	0	0	0
0													
OTU12356	0	0	0	0	0	0	0	0	0	0	0	0	1
0	0	0	0	0	0	0	0	0	0	0	0	0	0
0	0	0	0	0	0	0	0	0	0	0	0	0	0
0	0	0	0	0	0	0	0	0	0	0	0	0	0
0	0	0	0	0	0	0	0	0	0	0	0	0	0
0	0	0	0	0	0	0	0	0	0	0	0	0	0
0	0	0	0	0	0	0	0	0	0	0	0	0	0
0	0	0	0	0	0	0	0	0	0	0	0	0	0
0													
OTU8072	0	0	0	0	0	0	0	0	0	0	0	0	1
0	0	0	0	0	0	0	0	0	0	0	0	0	0
0	0	0	0	0	0	0	0	0	0	0	0	0	0
0	0	0	0	0	0	0	0	0	0	0	0	0	0
0	0	0	0	0	0	0	0	0	0	0	0	0	0
0	0	0	0	0	0	0	0	0	0	0	0	0	0
0	0	0	0	0	0	0	0	0	0	0	0	0	0
0	0	0	0	0	0	0	0	0	0	0	0	0	0
0													
OTU8201	0	0	0	0	0	0	0	0	0	0	0	0	1
0	0	0	0	0	0	0	0	0	0	0	0	0	0
0	0	0	0	0	0	0	0	0	0	0	0	0	0
0	0	0	0	0	0	0	0	0	0	0	0	0	1
0	0	0	0	0	0	0	0	0	0	0	0	0	0
0	0	0	0	0	0	0	0	0	0	0	0	0	0
0	0	0	0	0	0	0	0	0	1	0	0	0	1
0	0	0	0	0	0	0	0	0	0	0	0	0	0
8													
OTU12357	0	0	0	0	0	0	0	0	0	0	0	0	1
0	0	0	0	0	0	0	0	0	0	0	0	0	0
0	0	0	0	0	0	0	0	0	0	0	0	0	0

0 0 0 0 0 0 0 0 0 0 0 0 0 0
0 0 0 0 0 0 0 0 0 0 0 0 0 0
0 0 0 0 0 0 0 0 0 0 0 0 0 0
0 0 0 0 0 0 0 0 0 0 0 0 0 0
0 0 0 0 0 0 0 0 0 0 0 0 0 0
0
OTU3789 0 0 0 0 0 0 0 0 0 0 0 0 1
0 0 0 0 0 0 1 0 0 0 1 0 0 0
0 0 0 0 0 0 0 0 0 0 0 1 0 0
1 0 0 0 0 0 0 0 0 0 0 0 0 0
0 0 0 0 0 0 0 0 0 0 0 0 0 0
0 0 1 0 0 0 0 0 0 0 0 0 0 0
0 0 0 0 0 0 0 0 0 0 0 0 0 0
0 0 0 0 0 0 0 0 0 0 0 0 0 0
1
OTU4117 0 0 0 0 0 0 0 0 0 0 0 0 2
1 0 0 0 0 0 0 0 0 0 0 0 0 0
0 0 0 0 0 0 0 1 0 0 0 0 0 1
0 2 0 0 0 0 0 0 0 0 0 0 0 0
0 0 0 0 0 0 0 0 0 0 0 0 0 0
0 0 0 3 0 0 0 0 3 0 0 1 0 0
0 0 0 0 1 0 0 0 1 0 0 0 0 0
1 0 0 0 0 0 0 0 0 0 2 0 0 0
0
OTU4295 0 0 0 0 0 0 0 0 0 0 0 0 1
0 0 0 0 0 0 0 0 0 0 0 0 0 0
0 0 0 0 0 0 0 0 0 0 0 0 0 0
0 0 0 0 0 0 0 0 0 0 0 0 0 0
0 0 0 0 0 0 0 0 0 0 0 0 0 0
0 0 0 0 0 0 0 0 0 0 0 0 0 0
0 0 0 0 0 0 0 0 0 0 0 0 0 0
0 0 0 0 0 0 0 0 0 0 0 0 0 0
0

OTU15662	0	0	0	0	0	0	0	0	0	0	0	0	1
0	0	0	0	0	0	0	0	0	0	0	0	0	0
0	0	0	0	0	0	0	0	0	0	0	0	0	0
0	0	0	0	0	0	0	0	0	0	0	0	0	0
0	0	0	0	0	0	0	0	0	0	0	0	0	0
0	0	0	0	0	0	0	0	0	0	0	0	0	0
0	0	0	0	0	0	0	0	0	0	0	0	0	0
0	0	0	0	0	0	0	0	0	0	0	0	0	0
0													
OTU14865	0	0	0	0	0	0	0	0	0	0	0	0	1
0	0	0	0	0	0	0	0	0	0	0	0	0	0
0	0	0	0	0	0	0	0	0	0	0	0	0	0
0	0	0	0	0	0	0	0	0	0	0	0	0	0
0	0	0	1	0	0	0	0	0	0	0	0	0	0
0	0	0	0	0	0	0	0	0	0	0	0	0	0
0	0	0	0	0	0	0	0	1	0	0	0	0	3
0	0	0	0	0	0	0	0	0	0	0	0	0	0
0													
OTU4293	0	0	0	0	0	0	0	0	0	0	0	0	1
0	0	0	0	0	0	0	0	0	0	0	0	0	0
0	0	0	0	0	0	0	0	0	0	0	0	0	0
0	0	0	0	0	0	0	0	0	0	0	0	0	0
0	0	0	0	0	0	0	0	0	0	0	0	0	0
0	0	0	0	0	0	0	0	0	0	0	0	0	0
0	0	0	0	0	0	0	0	0	0	0	0	0	0
0	0	0	0	0	0	0	0	0	0	0	0	0	0
0													
OTU4125	0	0	0	0	0	0	0	0	0	0	0	0	2
0	0	0	0	0	0	0	0	0	0	0	0	0	0
0	0	0	0	0	0	0	0	0	0	0	0	0	0
0	0	0	0	0	0	0	0	0	0	0	0	0	0
0	0	0	0	0	0	0	0	0	0	0	0	0	0
0	0	0	0	0	0	0	0	0	0	0	0	0	0

	0	0	1	0	0	0	0	0	2	0	0	0	0	2
	1	0	0	0	0	0	0	0	0	0	0	0	0	0
	0													
OTU13520	0	0	0	0	0	0	0	0	0	0	0	0	1	
	0	0	0	0	1	0	0	0	0	0	0	0	0	0
	0	0	0	1	0	0	0	0	0	0	0	0	0	0
	0	0	0	0	1	0	1	0	0	0	0	0	0	0
	0	0	0	0	0	0	0	0	0	0	0	3	0	0
	0	0	0	0	0	0	0	0	0	0	0	0	0	0
	0	1	0	0	0	0	0	0	0	0	0	0	0	0
	0	0	0	0	0	0	0	0	0	0	0	0	0	0
	0													
OTU13113	0	0	0	0	0	0	0	0	0	0	0	0	1	
	0	0	0	0	0	1	0	0	0	0	0	0	0	0
	0	0	0	1	0	0	0	0	0	0	0	0	0	0
	0	0	1	0	0	0	0	0	0	0	0	0	0	0
	0	0	0	0	0	0	0	0	0	1	0	0	0	0
	0	0	1	0	0	0	0	1	0	0	0	1	0	0
	0	0	0	0	0	0	0	0	0	0	0	0	0	0
	1	0	0	0	0	0	0	0	0	0	0	0	0	0
	0													
OTU191	0	0	0	0	0	0	0	0	0	0	0	0	1	
	0	0	0	1	0	0	3	0	0	0	4	0	269	0
	1	0	0	0	0	0	10	0	0	0	59	0	0	0
	0	0	0	0	0	0	0	0	0	0	0	0	0	0
	0	0	0	0	0	0	0	0	65	0	1	0	0	0
	0	0	0	0	0	0	0	0	0	0	0	0	0	0
	0	0	0	0	0	0	0	0	0	0	0	0	0	0
	0	0	0	0	0	0	0	0	0	0	0	0	0	0
	0													
OTU4112	0	0	0	0	0	0	0	0	0	0	0	0	1	
	0	0	0	0	0	0	0	0	0	0	0	0	0	0
	0	0	0	0	0	0	0	0	0	0	0	0	0	0

0	0	0	0	0	0	0	0	0	0	0	0	0	0
0	0	0	0	0	0	0	0	0	0	0	1	0	1
0	0	0	0	0	0	0	0	0	0	0	0	0	0
0	0	0	0	0	0	0	0	0	0	0	1	0	0
1	0	0	0	0	0	0	0	0	0	0	0	0	0
0													
OTU12351	0	0	0	0	0	0	0	0	0	0	0	0	1
0	0	0	0	0	0	0	0	0	0	0	0	0	0
0	0	0	0	0	0	0	0	0	0	0	0	0	0
0	0	0	0	0	0	0	0	0	0	0	0	0	0
0	0	0	0	0	0	0	0	0	0	0	0	0	0
0	0	0	0	0	0	0	0	0	0	0	0	0	0
0	0	0	0	0	0	0	0	0	0	0	0	0	0
0	0	0	0	0	0	0	0	0	0	0	0	0	0
0													
OTU4129	0	0	0	0	0	0	0	0	0	0	0	0	2
0	0	0	0	1	0	0	0	1	0	0	0	0	0
0	0	0	0	2	0	0	0	0	0	0	0	0	0
0	0	0	0	0	0	1	0	0	0	0	0	0	1
0	0	0	0	0	0	0	0	0	1	0	0	0	1
0	0	0	0	0	0	0	0	0	0	0	0	0	0
0	0	0	0	0	0	2	0	0	0	0	0	0	0
0	0	0	0	0	0	0	0	0	0	0	0	0	0
0													
OTU1900	0	0	0	0	0	0	0	0	0	0	0	0	4
0	0	0	0	0	1	0	0	0	0	1	0	0	0
0	0	0	2	0	0	0	2	0	2	0	0	0	0
0	0	0	0	2	0	0	0	0	0	0	0	0	0
0	0	0	0	0	0	0	0	0	0	0	10	0	0
0	0	0	0	0	0	0	0	0	0	0	0	0	0
0	0	0	0	0	0	0	0	0	0	2	0	0	0
0	0	0	0	0	0	0	0	0	0	0	0	0	0
0													

OTU11986	0	0	0	0	0	0	0	0	0	0	0	0	2
0	0	0	0	0	0	0	0	0	0	0	0	1	0
0	0	0	0	0	0	0	0	0	0	0	0	0	0
0	0	0	0	0	0	0	0	0	0	0	0	0	0
0	0	0	0	0	0	0	0	0	0	0	0	0	0
0	0	0	2	0	0	0	0	0	0	1	0	0	0
2	0	0	0	0	0	0	0	2	0	0	0	0	0
0	0	0	0	0	0	0	0	0	0	0	0	0	0
0													
OTU940	0	0	0	0	0	0	0	0	0	0	0	0	2
0	0	0	0	0	0	0	0	0	0	0	0	0	0
0	0	0	0	0	0	0	0	0	0	0	0	0	0
0	1	0	1	0	0	0	0	0	0	0	0	28	0
1	0	0	0	0	0	0	0	0	0	0	0	0	0
0	1	0	0	0	0	0	0	0	0	1	0	0	0
0	0	0	0	0	0	0	0	0	0	0	0	0	0
0	0	0	0	0	0	0	0	0	0	4	0	0	0
0													
OTU3885	0	0	0	0	0	0	0	0	0	0	0	0	2
2	0	0	0	0	1	0	0	0	0	0	0	0	0
0	0	1	0	0	0	0	2	0	0	0	0	0	0
0	2	0	0	0	0	0	0	0	0	0	0	0	0
0	0	0	0	0	0	0	0	0	0	0	0	0	0
0	0	0	3	0	0	0	0	0	0	0	0	0	0
0	0	2	0	1	0	0	0	2	0	0	0	0	4
1	0	0	0	0	0	0	0	0	0	1	0	0	0
0													
OTU4114	0	0	0	0	0	0	0	0	0	0	0	0	2
1	0	0	0	0	0	0	0	0	0	0	0	0	0
0	0	0	0	0	0	0	0	0	0	0	0	0	0
0	0	0	0	0	0	0	0	0	0	0	0	0	0
0	0	1	0	0	0	0	0	0	0	0	0	0	0
0	0	0	0	0	0	0	0	0	0	0	0	0	0

0	0	0	0	0	0	0	0	0	0	0	0	0	0
0	0	0	0	0	0	0	0	0	0	0	0	0	0
0													
OTU12350	0	0	0	0	0	0	0	0	0	0	0	0	1
0	0	0	0	0	0	0	0	0	0	0	0	0	0
0	0	0	0	0	0	0	0	0	0	0	0	0	0
0	0	0	0	0	0	0	0	0	0	0	0	0	0
0	0	0	0	0	0	0	0	0	0	0	0	0	0
0	0	0	0	0	0	0	0	0	0	0	0	0	0
0	0	0	0	0	0	0	0	0	0	0	0	0	0
0	0	0	0	0	0	0	0	0	0	0	0	0	0
0													
OTU15672	0	0	0	0	0	0	0	0	0	0	0	0	1
0	0	0	0	0	3	0	0	0	0	0	0	0	0
0	0	0	0	0	0	0	0	0	0	0	0	0	0
0	0	0	0	0	0	0	0	0	0	0	0	0	0
0	0	0	0	0	0	0	0	0	0	0	0	0	0
0	0	0	0	0	0	0	0	0	0	0	0	0	0
0	0	0	0	0	0	0	0	0	0	0	0	0	0
0	0	0	0	0	0	0	0	0	0	0	0	0	0
0													
OTU10298	0	0	0	0	0	0	0	0	0	0	0	0	1
0	0	0	0	0	0	0	0	0	0	0	0	0	0
0	0	0	0	0	0	0	0	0	0	0	0	0	0
0	0	0	0	0	0	0	0	0	0	0	0	0	0
1	0	0	0	0	0	1	0	0	0	0	0	0	0
0	0	0	0	0	0	0	0	0	0	0	0	0	0
0	0	0	0	0	0	0	0	0	0	0	0	0	1
0	0	0	0	0	0	0	0	0	0	0	0	0	0
0													
OTU4110	0	0	0	0	0	0	0	0	0	0	0	0	1
0	0	0	0	0	0	0	0	0	0	0	0	0	0
0	0	0	0	0	0	0	0	0	0	0	0	0	0

0 0 0 0 0 0 0 0 0 0 0 0 0 0
0 0 0 1 0 0 0 0 0 0 0 0 0 0
0 0 0 0 1 0 0 0 0 0 0 0 0 0
0 0 0 0 0 0 0 0 0 0 0 0 0 0
0 0 0 0 0 0 0 0 0 0 0 0 0 0
0
OTU15673 0 0 0 0 0 0 0 0 0 0 0 0 1
0 0 0 0 0 0 0 0 0 0 0 0 0 0
0 0 0 0 0 0 0 0 0 0 0 0 0 0
0 0 0 0 0 0 0 0 0 0 0 0 0 0
0 0 0 0 0 0 0 0 0 0 0 0 0 0
0 0 0 0 0 0 0 0 0 0 0 0 0 0
0 0 0 0 0 0 0 0 0 0 0 0 0 0
0 0 0 0 0 0 0 0 0 0 0 0 0 0
0
OTU9389 0 0 0 0 0 0 0 0 0 0 0 0 1
0 0 0 0 0 0 0 0 0 0 0 0 0 0
0 0 1 0 0 0 0 0 0 0 0 0 0 0
0 0 0 0 0 0 0 0 0 0 0 0 0 0
0 0 0 0 0 0 0 0 0 0 0 0 0 0
0 0 0 1 0 0 0 0 0 0 0 0 1 0
0 0 0 0 0 0 0 0 2 0 0 0 0 0
0 0 0 0 0 0 0 0 0 0 0 0 0 0
0
OTU4111 0 0 0 0 0 0 0 0 0 0 0 0 1
0 0 0 0 0 0 0 0 0 0 0 0 0 0
0 0 0 0 0 0 0 0 0 0 0 0 0 0
0 0 0 0 0 0 0 0 0 0 0 0 0 0
0 0 0 0 0 0 0 0 0 0 0 0 0 0
0 0 0 0 0 0 0 0 0 0 0 0 0 0
0 0 0 0 0 0 0 0 0 0 0 0 0 0
0 0 0 0 0 0 0 0 0 0 0 0 0 0
0

OTU2495 0 0 0 0 0 0 0 0 0 0 0 0 1
0 0 0 0 0 0 0 0 0 0 0 0 0 0
0 0 0 0 0 0 0 0 0 0 0 0 0 0
0 0 0 0 0 0 0 0 0 0 0 0 0 0
0 0 0 0 0 0 0 0 0 0 0 0 0 0
0 0 0 0 0 0 0 0 0 0 0 0 0 0
0 0 0 0 0 0 0 0 0 0 1 0 0 0
0 0 0 0 0 0 0 0 0 0 0 0 0 0
0
OTU5311 0 0 0 0 0 0 0 0 0 0 0 0 1
0 0 0 0 0 0 0 0 0 0 0 0 0 0
0 0 0 0 0 0 0 0 1 0 0 0 0 0
0 0 0 0 0 0 0 0 0 0 0 0 0 0
0 0 0 0 0 0 0 0 0 0 0 0 0 0
0 0 0 0 0 0 0 0 0 0 0 0 0 0
0 0 0 0 0 0 0 0 0 0 0 0 0 0
0 0 0 0 0 0 0 0 0 0 0 0 0 0
0
OTU15671 0 0 0 0 0 0 0 0 0 0 0 0 1
0 0 0 0 0 0 0 0 0 0 0 0 0 0
0 0 0 0 0 0 0 0 0 0 0 0 0 0
0 0 0 0 0 0 0 0 0 0 0 0 0 0
0 0 0 0 0 0 0 0 0 0 0 0 0 0
0 0 0 0 0 0 0 0 0 0 0 0 0 0
0 0 0 0 0 0 0 0 0 0 0 0 0 0
0 0 0 0 0 0 0 0 0 0 0 0 0 0
0
OTU8066 0 0 0 0 0 0 0 0 0 0 0 0 1
0 0 0 0 0 0 0 0 0 0 0 0 0 0
0 0 0 0 0 0 0 0 0 0 0 0 0 0
0 0 0 0 0 0 0 0 0 0 0 0 0 0
0 0 0 0 0 0 0 0 0 0 0 0 0 0
0 0 0 0 0 0 0 0 0 0 0 0 0 0

0	0	0	0	0	0	0	0	0	0	0	0	0	0
0	0	0	0	0	0	0	0	0	0	0	0	0	0
0													
OTU4345	0	0	0	0	0	0	0	0	0	0	0	0	1
0	0	0	0	0	1	0	0	0	0	0	0	0	0
0	0	0	0	0	0	0	0	0	0	0	0	0	0
0	0	0	0	0	0	0	0	0	0	0	0	0	0
0	0	0	0	0	0	0	0	0	0	0	0	0	0
0	0	0	0	0	0	1	0	0	0	1	0	0	0
0	0	0	0	0	0	0	0	0	0	0	0	0	1
0	0	0	0	0	0	0	0	0	0	0	0	0	0
0													
OTU6400	0	0	0	0	0	0	0	0	0	0	0	0	1
0	0	0	0	0	0	0	0	0	0	0	0	0	0
0	0	0	0	0	0	0	2	0	0	1	0	0	0
0	0	0	0	0	2	0	4	0	0	0	0	0	1
0	0	0	0	0	0	0	0	0	0	0	0	0	0
0	0	0	0	0	0	0	0	0	0	0	0	0	0
0	0	0	0	0	0	0	0	0	0	1	0	0	0
0	0	0	0	0	0	0	0	0	0	0	0	0	0
0													
OTU4120	0	0	0	0	0	0	0	0	0	0	0	0	1
0	0	0	0	0	0	0	0	0	0	0	0	0	0
0	0	0	0	0	0	0	0	0	0	0	0	0	0
0	0	0	0	0	0	0	0	0	0	0	0	0	0
0	0	0	0	0	0	0	0	0	0	0	0	0	0
0	0	0	0	0	0	0	0	0	0	0	0	0	0
0	0	0	0	0	0	0	0	0	0	0	0	0	0
0	0	0	0	0	0	0	0	0	0	0	0	0	0
0													
OTU562	0	0	0	0	0	0	0	0	0	0	0	0	1
0	0	2	0	0	0	0	0	0	0	7	3	0	1
1	0	0	0	3	0	0	0	0	1	0	0	1	0

0	0	0	0	2	1	0	0	14	0	0	1	2	1
8	0	0	0	0	7	1	20	2	0	0	23	1	0
0	2	0	0	0	0	0	0	0	0	0	0	1	0
0	0	0	0	0	0	0	1	0	0	0	0	23	0
0	1	0	1	0	0	0	4	1	2	0	0	0	0
0													
OTU12352	0	0	0	0	0	0	0	0	0	0	0	0	1
0	0	0	0	0	0	0	0	0	0	0	0	0	0
0	0	0	0	0	0	0	0	0	0	0	0	0	0
0	0	0	0	0	0	0	0	0	0	0	0	0	0
0	0	0	0	0	0	0	0	0	0	0	0	0	0
0	0	0	0	0	0	0	0	0	0	0	0	0	0
0	0	0	0	0	0	0	0	0	0	0	0	0	0
0	0	0	0	0	0	0	0	0	0	0	0	0	0
0													
OTU4116	0	0	0	0	0	0	0	0	0	0	0	0	1
0	0	0	0	0	0	0	0	0	0	0	0	0	0
0	0	0	0	0	0	0	0	0	0	0	0	0	0
0	0	0	0	0	0	0	0	0	0	0	0	0	0
0	0	0	0	0	0	0	0	0	0	0	0	0	0
0	0	0	0	0	0	0	0	0	0	0	0	0	0
0	0	0	0	0	0	0	0	0	0	0	0	0	0
0	0	0	0	0	0	0	0	0	0	0	0	0	0
0													
OTU12354	0	0	0	0	0	0	0	0	0	0	0	0	1
0	0	0	0	0	0	0	0	0	0	0	0	0	0
0	0	0	0	0	0	0	0	0	0	0	0	0	0
0	0	0	0	0	0	0	0	0	0	0	0	0	0
0	0	0	0	0	0	0	0	0	0	0	0	0	0
0	0	0	0	0	0	0	0	0	0	0	0	0	0
0	0	0	0	0	0	0	0	0	0	0	0	0	0
0	0	0	0	0	0	0	0	0	0	0	0	0	0
0													

OTU13829	0	0	0	0	0	0	0	0	0	0	0	0	1
0	0	0	0	0	1	0	0	0	0	0	0	0	0
0	0	0	0	0	0	1	0	0	0	0	0	0	0
0	0	0	0	0	0	0	0	0	0	0	0	0	0
0	0	0	0	0	0	0	0	0	0	0	0	0	0
1	0	0	0	0	0	0	0	0	0	0	0	0	0
0	0	0	0	0	0	0	0	0	0	0	0	0	0
0	0	0	0	0	0	0	0	0	0	0	0	0	0
0													
OTU12353	0	0	0	0	0	0	0	0	0	0	0	0	1
0	0	0	0	0	0	0	0	0	0	0	0	0	0
0	0	0	0	0	0	0	0	0	0	0	0	0	0
0	0	0	0	0	0	0	0	0	0	0	0	0	0
0	0	0	0	0	0	0	0	0	0	0	0	0	0
0	0	0	0	0	0	0	0	0	0	0	0	0	0
0	0	0	0	0	0	0	0	0	0	0	0	0	0
0	0	0	0	0	0	0	1	0	0	0	0	0	0
0													
OTU15676	0	0	0	0	0	0	0	0	0	0	0	0	1
0	0	0	0	0	0	0	0	0	0	0	0	0	0
0	0	0	0	0	0	0	0	0	0	0	0	0	0
0	0	0	0	0	0	0	0	0	0	0	0	0	0
0	0	0	0	0	0	0	0	0	0	0	0	0	0
0	0	0	0	0	0	0	0	0	0	0	0	0	0
0	0	0	0	0	0	0	0	0	0	0	0	0	0
0	0	0	0	0	0	0	0	0	0	0	0	0	0
0													
OTU172	0	0	0	0	0	0	0	0	0	0	0	0	3
0	0	0	135	0	0	0	0	187	0	3	0	3	0
0	120	0	0	47	0	8	2	0	0	12	0	0	0
0	0	25	0	5	0	0	0	100	31	1	3	1	0
43	0	11	0	9	45	21	37	48	21	170	8	1	3
106	0	0	0	0	0	0	7	0	0	0	1	0	0

0 0 0 0 0 0 0 0 0 0 0 0 0 0
0 0 0 0 0 0 0 0 0 0 0 0 0 0
0
OTU479 0 0 0 0 0 0 0 0 0 0 0 0 1
0 0 0 0 0 0 0 0 0 0 0 0 0 0
0 0 0 0 0 0 53 0 0 0 0 0 0 0
0 0 0 0 0 0 0 0 0 0 0 71 0 0
0 0 0 0 0 0 0 0 0 0 0 1 0 0
0 0 0 0 0 0 0 0 0 0 0 0 0 0
0 0 0 0 0 0 0 0 0 0 0 0 0 0
0 0 0 0 0 0 0 0 0 0 10 0 0 0
0
OTU4105 0 0 0 0 0 0 0 0 0 0 0 0 1
0 0 0 0 0 0 0 0 0 0 0 0 0 0
0 0 0 0 0 0 0 0 0 0 0 0 0 0
0 0 0 0 0 0 0 0 0 0 0 0 0 0
0 0 0 0 0 0 0 1 0 0 0 0 0 0
0 0 0 0 0 0 0 0 0 0 0 0 0 0
0 0 0 0 0 0 0 0 0 0 0 0 0 0
0 0 0 0 0 0 0 0 0 0 0 0 0 0
0
OTU8041 0 0 0 0 0 0 0 0 0 0 0 0 1
0 0 0 0 0 0 0 0 0 0 0 0 0 0
0 0 0 0 0 0 0 0 0 0 0 0 0 0
0 0 0 0 0 0 0 0 0 0 0 0 0 0
0 0 0 0 0 0 0 0 0 0 0 0 0 0
0 0 0 0 0 0 0 0 0 0 0 0 0 0
0 0 0 0 0 0 0 0 0 0 0 0 0 0
0 0 0 0 0 0 0 0 0 0 0 0 0 0
0
OTU5230 0 0 0 0 0 0 0 0 0 0 0 0 1
0 0 0 0 0 1 0 0 0 1 0 0 0 0
0 0 0 0 0 0 0 0 0 0 0 1 0 1

0	0	0	0	0	0	0	0	0	0	0	0	0	0
0	0	0	0	0	0	0	0	0	0	0	0	0	0
0	0	0	0	0	0	0	0	0	0	0	0	0	0
0	0	0	0	0	0	0	0	0	0	0	0	0	0
0	0	0	0	0	0	0	0	0	0	0	0	0	0
0													
OTU15675	0	0	0	0	0	0	0	0	0	0	0	0	1
0	0	0	0	0	0	0	0	0	0	0	0	0	0
0	0	0	0	0	0	0	0	0	0	0	0	0	0
0	0	0	0	0	0	0	0	0	0	0	0	0	0
0	0	0	0	0	0	0	0	0	0	0	0	0	0
0	0	0	0	0	0	0	0	0	0	0	0	0	0
0	0	0	0	0	0	0	0	0	0	0	0	0	0
0	0	0	0	0	0	0	0	0	0	0	0	0	0
0													
OTU15674	0	0	0	0	0	0	0	0	0	0	0	0	1
0	0	0	0	0	0	0	0	0	0	0	0	0	0
0	0	0	0	0	0	0	0	0	0	0	0	0	0
0	0	0	0	0	0	0	0	0	0	0	0	0	0
0	0	0	0	0	0	0	0	0	0	0	0	0	0
0	0	0	0	0	0	0	0	0	0	0	0	0	0
0	0	0	0	0	0	0	0	0	0	0	0	0	0
0	0	0	0	0	0	0	0	0	0	0	0	0	0
0													
OTU3173	0	0	0	0	0	0	0	0	0	0	0	0	1
0	0	0	0	0	0	0	0	0	0	0	0	0	0
0	0	0	0	0	0	0	0	0	0	0	0	0	0
0	0	0	0	0	0	0	0	0	0	0	0	0	0
0	0	0	0	0	0	0	0	0	0	0	0	0	0
0	0	0	0	0	0	0	0	0	0	0	0	1	0
0	0	0	0	0	0	0	0	0	0	0	0	0	0
0	0	0	0	0	0	0	0	0	0	1	0	0	0
0													

OTU4721	0	0	0	0	0	0	0	0	0	0	0	0	2
0	0	0	0	0	0	0	0	0	0	0	0	0	0
0	0	0	0	0	0	0	0	0	0	0	0	0	0
0	0	0	0	0	0	0	0	0	0	0	0	1	0
1	0	0	0	0	0	0	0	0	0	0	0	0	0
0	0	0	0	0	0	0	0	0	0	0	0	0	0
0	0	0	0	0	0	0	0	0	0	0	0	0	0
0	0	0	0	0	0	0	0	0	0	1	0	0	0
0													
OTU4038	0	0	0	0	0	0	0	0	0	0	0	0	2
15	0	1	1	0	2	0	0	0	0	0	0	0	0
0	0	3	0	5	0	0	0	0	0	0	0	0	0
0	0	0	0	1	0	0	0	1	0	0	0	1	0
4	0	0	0	0	0	0	0	0	0	0	0	0	0
0	0	0	0	0	1	1	0	0	0	0	0	0	0
0	0	0	0	0	0	0	0	4	1	0	0	0	8
0	0	0	0	0	0	0	0	0	0	0	3	1	0
0													
OTU8042	0	0	0	0	0	0	0	0	0	0	0	0	1
0	0	0	0	0	0	0	0	0	0	0	0	0	0
0	0	0	0	0	0	0	0	0	0	0	0	0	0
0	0	0	0	0	0	0	0	0	0	0	0	0	0
0	0	0	0	0	0	0	0	0	0	0	0	0	0
0	0	0	0	0	0	0	0	0	0	0	0	0	0
0	0	0	0	0	0	0	0	0	0	0	0	0	0
0	0	0	0	0	0	0	0	0	0	0	0	0	0
0													
OTU16348	0	0	0	0	0	0	0	0	0	0	0	0	1
0	0	0	0	0	0	0	0	0	0	0	0	0	0
0	0	0	0	0	0	0	0	0	0	0	0	0	0
0	0	0	0	0	0	0	0	0	0	0	0	0	0
0	0	0	1	0	0	0	0	0	0	0	0	0	0
0	0	0	0	0	0	0	0	0	0	0	0	0	0

0	0	0	0	0	0	0	0	0	0	0	0	0	0
0	0	0	0	0	0	0	0	0	0	0	0	0	0
0													
OTU9326	0	0	0	0	0	0	0	0	0	0	0	0	1
0	0	0	0	0	0	0	1	0	0	0	0	0	0
0	0	0	0	0	0	0	0	0	0	0	0	0	0
0	0	0	0	0	0	0	0	0	0	0	0	0	0
0	0	0	0	0	0	0	0	0	0	0	0	0	0
0	0	0	0	0	0	0	0	0	0	0	0	0	0
0	0	0	0	0	0	0	0	0	0	0	0	0	0
0	0	0	0	0	0	0	0	0	0	0	0	0	0
0													
OTU929	0	0	0	0	0	0	0	0	0	0	0	0	2
0	0	0	0	0	0	0	0	0	0	0	0	0	0
0	0	0	0	0	0	0	0	0	0	0	0	0	0
0	0	0	0	0	0	0	0	0	0	0	0	9	0
2	0	0	0	0	0	0	0	0	0	0	0	0	0
0	0	0	0	0	0	0	0	0	0	0	0	0	0
1	0	0	0	0	0	0	0	0	0	0	0	0	0
0	0	0	0	0	0	0	0	0	0	1	0	0	0
0													
OTU8040	0	0	0	0	0	0	0	0	0	0	0	0	1
0	0	0	0	0	0	0	0	0	0	0	0	0	0
0	0	0	0	0	0	0	0	0	0	0	0	0	0
0	0	0	0	0	0	0	0	0	0	0	0	0	0
0	0	0	0	0	0	0	0	0	0	0	0	0	0
0	0	0	0	0	0	0	0	0	0	0	0	0	0
0	0	0	0	0	0	0	0	0	0	0	0	0	0
0	0	0	0	0	0	0	0	0	0	0	0	0	0
0													
OTU14075	0	0	0	0	0	0	0	0	0	0	0	0	1
0	2	0	0	0	0	0	0	0	0	0	0	0	0
0	0	0	0	0	0	0	1	0	0	0	0	0	0

0 0 0 4 0 0 4 0 0 0 0 0 0 0
0 0 0 0 0 0 0 0 0 0 0 0 0 0
0 0 0 0 0 3 7 0 5 0 6 0 2 0
0 0 5 0 1 0 0 0 0 0 1 0 0 0
0 0 0 0 0 0 0 0 0 0 1 7 0 0
0
OTU8908 0 0 0 0 0 0 0 0 0 0 0 0 1
0 0 0 5 0 0 1 0 4 0 16 0 0 0
0 8 0 0 12 0 4 0 1 0 2 1 1 0
0 0 1 0 0 1 1 1 0 3 0 1 4 6
1 0 7 0 1 0 5 0 0 4 0 1 0 2
0 0 0 1 0 2 2 0 0 0 2 0 0 0
1 0 0 0 3 0 2 0 1 0 0 0 0 0
0 0 0 0 0 0 0 0 0 0 0 0 0 0
0
OTU4109 0 0 0 0 0 0 0 0 0 0 0 0 1
1 0 0 0 0 0 0 0 0 0 0 0 0 0
0 0 0 0 0 0 0 0 0 0 0 0 0 0
0 0 0 0 0 0 0 0 0 0 0 0 0 0
0 0 0 0 0 0 0 0 0 0 0 0 0 0
0 0 0 0 0 0 0 0 0 0 0 0 0 0
0 0 0 0 0 0 0 0 0 0 0 0 0 0
0 0 0 0 0 0 0 0 0 0 0 0 0 0
0
OTU8046 0 0 0 0 0 0 0 0 0 0 0 0 1
0 0 0 0 0 0 0 0 0 0 0 0 0 0
0 0 0 0 0 0 0 0 0 0 0 0 0 0
0 0 0 0 0 0 0 0 0 0 0 0 0 0
0 0 0 0 0 0 0 0 0 0 0 0 0 0
0 0 0 0 0 0 0 0 0 0 0 0 0 0
0 0 0 0 0 0 0 0 0 0 0 0 0 0
0 0 0 0 0 0 0 0 0 0 0 0 0 0
0

OTU16030 0 0 0 0 0 0 0 0 0 0 0 0 1
0 1 0 0 0 0 0 0 0 0 0 0 0 0
0 0 0 0 1 0 1 0 0 0 0 0 0 0
0 1 0 0 0 0 0 0 0 0 0 0 0 0
0 0 1 0 0 0 0 0 0 0 0 0 0 0
0 0 0 0 0 0 0 0 0 0 0 0 0 0
0 0 0 0 0 0 0 0 0 0 0 0 0 0
0 0 0 0 0 0 0 0 0 0 0 0 0 0
0
OTU16044 0 0 0 0 0 0 0 0 0 0 0 0 1
1 0 0 7 0 0 0 0 0 0 0 0 0 0
0 1 0 0 5 0 0 1 0 0 0 0 0 0
0 0 16 0 1 0 0 0 8 0 0 0 13 0
11 0 6 0 0 1 5 1 0 4 0 0 0 1
2 0 0 0 0 0 0 1 0 0 0 0 0 0
0 0 0 0 0 0 0 0 1 0 0 0 0 5
0 0 0 0 0 0 0 0 0 0 0 1 0 0
0
OTU4106 0 0 0 0 0 0 0 0 0 0 0 0 1
0 0 0 0 0 0 0 0 0 0 0 0 0 0
0 0 0 0 0 0 0 0 0 0 0 0 0 0
0 0 0 0 0 0 0 0 0 0 0 0 0 0
0 0 0 0 0 0 0 0 0 0 0 0 0 0
0 0 0 0 0 0 0 0 0 0 0 0 0 0
0 0 0 0 0 0 0 0 0 0 0 0 0 0
0 0 0 0 0 0 0 0 0 0 0 0 0 0
0
OTU12325 0 0 0 0 0 0 0 0 0 0 0 0 1
0 0 0 0 0 0 0 0 0 0 0 0 0 0
0 0 0 0 0 0 0 0 0 0 0 0 0 0
0 0 0 0 0 0 0 0 0 0 0 0 0 0
0 0 0 0 0 0 0 0 0 0 0 0 0 0
0 0 0 0 0 0 0 0 0 0 0 0 0 0

0	0	0	0	0	0	0	0	0	0	0	0	0	0
0	0	0	0	0	0	0	0	0	0	0	0	0	0
0													
OTU2679	0	0	0	0	0	0	0	0	0	0	0	0	4
39	0	0	0	0	1	0	0	0	0	0	0	0	0
0	0	1	0	0	0	0	0	0	0	0	0	0	1
0	0	30	0	0	0	0	0	5	1	0	0	9	0
22	0	19	0	0	5	12	2	0	4	4	1	0	3
4	0	0	0	0	0	3	0	0	0	0	0	0	0
0	4	1	0	0	0	1	0	24	0	0	0	0	38
1	0	0	0	0	0	0	0	0	0	0	0	2	0
0													
OTU12327	0	0	0	0	0	0	0	0	0	0	0	0	1
0	0	0	0	0	0	0	0	0	0	0	0	0	0
0	0	0	0	0	0	0	0	0	0	0	0	0	0
0	0	0	0	0	0	0	0	0	0	0	0	0	0
0	0	0	0	0	0	0	0	0	0	0	0	0	0
0	0	0	0	0	0	0	0	0	0	0	0	0	0
0	0	0	0	0	0	0	0	0	0	0	0	0	0
0	0	0	0	0	0	0	0	0	0	0	0	0	0
0													
OTU4421	0	0	0	0	0	0	0	0	0	0	0	0	2
2	0	0	0	0	0	0	1	0	0	0	0	0	0
0	0	0	0	0	0	0	0	0	0	0	0	0	0
0	0	0	0	0	0	0	0	0	0	0	0	0	0
0	0	0	0	0	0	0	0	0	0	0	0	0	0
0	0	0	0	0	1	0	0	0	0	0	0	0	0
0	0	0	0	0	0	0	0	0	0	0	0	0	0
0	0	0	0	0	0	0	0	0	0	0	0	0	0
0													
OTU8044	0	0	0	0	0	0	0	0	0	0	0	0	1
0	0	0	0	0	0	0	0	0	0	0	0	0	0
0	0	0	0	0	0	0	0	0	0	0	0	0	0

0	0	0	0	0	0	0	0	0	0	0	0	0	0
0	0	0	0	0	0	0	0	0	0	0	0	0	0
0	0	0	0	0	0	0	0	0	0	0	0	0	0
0	0	0	0	0	0	0	0	0	0	0	0	0	0
0	0	0	0	0	0	0	0	0	0	0	0	0	0
0													
OTU12777	0	0	0	0	0	0	0	0	0	0	0	0	2
0	0	0	0	0	0	0	0	0	10	0	0	0	0
0	0	1	0	0	0	0	1	0	0	0	0	0	0
0	0	0	0	0	0	33	0	0	0	0	0	0	0
0	0	0	0	0	0	0	0	0	0	0	0	0	0
0	0	0	0	0	0	4	0	0	0	8	0	1	0
0	0	5	0	0	0	0	0	3	0	0	0	0	1
1	0	0	0	0	0	0	0	0	0	0	0	0	0
0													
OTU4132	0	0	0	0	0	0	0	0	0	0	0	0	1
0	0	0	0	0	0	0	0	0	0	0	0	0	0
0	0	0	0	0	0	0	0	0	0	0	0	0	0
0	0	0	0	0	0	0	0	0	0	0	0	0	0
0	0	0	0	0	0	0	0	0	0	0	0	0	0
0	0	0	0	0	0	0	0	0	0	0	0	0	0
0	0	0	0	0	0	0	0	0	0	0	0	0	0
0	0	0	0	0	0	0	0	0	0	0	0	0	0
0													
OTU8033	0	0	0	0	0	0	0	0	0	0	0	0	1
0	0	0	0	0	0	0	0	0	0	0	0	0	0
0	0	0	0	0	0	0	0	0	0	0	0	0	0
0	0	0	0	0	0	0	0	0	0	0	0	0	0
0	0	0	0	0	0	0	0	0	0	0	0	0	0
0	0	0	0	0	0	0	0	0	0	0	0	0	0
0	0	0	0	0	0	0	0	0	0	0	0	0	0
0	0	0	0	0	0	0	0	0	0	0	0	0	0
0													

OTU9611 0 0 0 0 0 0 0 0 0 0 0 0 1
0 0 0 0 0 2 0 0 0 0 0 0 0 0
0 0 0 0 0 0 0 0 0 0 0 0 0 0
0 0 0 0 0 0 0 0 0 0 0 0 0 0
0 0 0 0 0 0 0 0 0 0 0 0 0 0
0 0 0 0 0 0 0 0 0 0 2 0 0 0
0 0 1 0 0 0 0 0 0 0 0 0 0 0
0 0 0 0 0 0 0 0 0 0 0 0 0 0
0
OTU15670 0 0 0 0 0 0 0 0 0 0 0 0 2
0 0 0 0 0 5 1 0 0 0 0 0 0 0
0 0 0 0 0 0 0 0 0 0 0 0 0 0
0 0 0 0 0 0 0 0 0 0 0 0 0 0
0 0 0 0 0 0 0 0 0 0 0 0 0 0
0 0 1 0 0 0 0 0 0 0 0 0 0 0
0 0 0 2 0 0 0 0 0 0 0 0 0 0
0 0 0 0 0 0 0 0 0 0 0 0 0 0
0
OTU4763 0 0 0 0 0 0 0 0 0 0 0 0 1
0 0 0 0 0 0 0 0 0 0 0 0 0 0
0 0 0 0 0 0 0 0 0 0 0 0 0 0
0 1 0 0 0 0 0 0 1 0 0 0 0 0
0 0 0 0 0 0 0 0 0 0 0 0 0 0
0 0 0 0 0 0 0 0 0 0 0 0 0 0
0 0 0 0 0 0 0 0 0 0 0 0 0 0
0 0 1 0 0 0 1 0 0 0 0 0 0 0
0
OTU13890 0 0 0 0 0 0 0 0 0 0 0 0 1
0 0 0 0 0 0 0 0 4 0 0 0 0 0
4 42 0 0 0 0 0 0 0 0 1 0 0 0
0 0 0 0 0 0 0 0 1 1 8 9 0 0
0 0 0 2 1 10 0 12 11 0 0 0 1 0
1 0 0 0 0 0 0 0 0 0 2 1 1 0

0	0	0	0	0	0	0	0	0	0	0	0	0	0
0	0	1	1	0	0	0	0	2	9	0	0	0	0
4													
OTU4133	0	0	0	0	0	0	0	0	0	0	0	0	1
0	0	0	0	0	0	0	0	0	0	0	0	0	0
0	0	0	0	0	0	0	0	0	0	0	0	0	0
0	0	0	0	0	0	0	0	0	0	0	0	0	0
0	0	0	0	0	0	0	0	0	0	0	0	0	0
0	0	0	0	0	0	0	0	0	0	0	0	0	0
0	0	0	0	0	0	0	0	0	0	0	0	0	0
0	0	0	0	0	0	0	0	0	0	0	0	0	0
0													
OTU2765	0	0	0	0	0	0	0	0	0	0	0	0	1
0	0	0	0	0	0	0	0	0	0	1	0	0	0
0	0	0	2	0	0	0	1	0	0	0	1	0	0
0	0	0	0	0	0	0	0	0	0	0	0	1	0
0	0	1	0	0	0	0	0	0	0	0	0	0	0
0	0	0	0	0	0	0	0	0	0	0	0	0	0
1	0	0	0	0	0	0	0	0	0	0	0	0	1
0	0	0	0	0	0	0	0	0	0	0	0	0	0
0													
OTU8665	0	0	0	0	0	0	0	0	0	0	0	0	1
0	0	0	0	0	0	0	0	0	0	0	0	0	1
0	0	0	0	0	0	0	0	1	0	0	0	0	0
0	0	0	0	0	0	0	0	0	0	0	0	0	0
0	0	0	0	0	0	0	0	0	0	0	0	0	0
0	0	0	0	0	0	0	0	0	0	1	0	0	0
1	0	0	0	0	0	0	0	0	0	0	0	0	0
0	0	0	0	0	0	0	0	0	0	0	0	0	0
0													
OTU12326	0	0	0	0	0	0	0	0	0	0	0	0	1
0	0	0	0	0	0	0	0	0	0	0	0	0	0
0	0	0	0	0	0	0	0	0	0	0	0	0	0

0 0 0 0 0 0 0 0 0 0 0 0 0 0
0 0 0 0 0 0 0 0 0 0 0 0 0 0
0 0 0 0 0 0 0 0 0 0 0 0 0 0
0 0 0 0 0 0 0 0 0 0 0 0 0 0
0 0 0 0 0 0 0 0 0 0 0 0 0 0
0
OTU4137 0 0 0 0 0 0 0 0 0 0 0 0 1
0 0 0 0 0 0 0 0 0 0 0 0 0 0
0 0 0 0 0 0 0 0 0 0 0 0 0 0
0 0 0 0 0 0 0 0 0 0 0 0 0 0
0 0 0 0 0 0 0 0 0 0 0 0 0 0
0 0 0 0 0 0 0 0 0 0 0 0 0 0
0 0 0 0 0 0 0 0 0 0 0 0 0 0
0 0 0 0 0 0 0 0 0 0 0 0 0 0
0
OTU9191 0 0 0 0 0 0 0 0 0 0 0 0 1
0 0 0 0 0 0 0 1 0 3 0 4 0 0
0 0 1 0 0 0 0 0 0 0 0 1 0 0
0 0 1 0 0 0 0 0 0 0 0 0 0 0
0 0 0 0 0 0 0 0 0 0 1 0 0 0
1 0 1 0 0 0 0 0 0 0 0 0 0 0
0 0 0 0 0 0 0 0 0 0 0 0 0 0
0 0 0 0 0 0 0 0 0 0 0 0 0 0
0
OTU15669 0 0 0 0 0 0 0 0 0 0 0 0 1
0 0 0 0 0 0 0 0 0 0 0 0 0 0
0 0 0 0 0 0 0 0 0 0 0 0 0 0
0 0 0 0 0 0 0 0 0 0 0 0 0 0
0 0 0 0 0 0 0 0 0 0 0 0 0 0
0 0 0 0 0 0 0 0 0 0 0 0 0 0
0 0 0 0 0 0 0 0 0 0 0 0 0 0
0 0 0 0 0 0 0 0 0 0 0 0 0 0
0

OTU5546	0	0	0	0	0	0	0	0	0	0	0	0	1
0	0	0	0	0	0	0	0	0	0	0	0	0	0
0	0	0	0	0	0	0	0	0	0	0	0	0	0
0	0	0	0	0	1	0	0	0	0	0	0	0	0
0	0	0	0	0	0	0	0	0	0	0	0	0	0
0	0	0	0	0	0	0	0	0	0	0	0	0	0
0	0	0	0	0	0	0	0	0	0	0	0	0	0
0	0	0	0	0	0	0	0	0	0	0	0	0	0
0													
OTU220	0	0	0	0	0	0	0	0	0	0	0	0	1
1	0	0	0	0	0	0	0	0	0	0	0	3	0
0	0	0	0	0	0	133	1	0	0	0	0	0	0
0	0	0	1	0	0	0	0	0	1	0	308	0	0
0	0	0	0	0	0	0	0	0	0	0	0	0	0
0	0	0	0	0	1	1	0	0	0	0	0	0	0
0	0	0	0	0	0	1	0	0	0	0	1	0	0
0	0	0	0	0	0	0	1	0	0	56	0	0	0
1													
OTU8924	0	0	0	0	0	0	0	0	0	0	0	0	1
1	0	0	0	0	0	0	0	0	0	0	0	0	0
0	0	0	0	0	0	0	0	0	0	0	0	1	0
0	0	0	0	0	0	0	0	0	0	0	0	0	0
0	0	0	0	0	0	0	0	0	0	0	0	0	0
0	0	0	0	0	0	0	0	0	0	0	0	0	0
0	0	0	0	0	0	0	0	0	0	0	0	0	0
0	0	0	0	0	0	0	0	0	0	0	0	0	0
0													
OTU4135	0	0	0	0	0	0	0	0	0	0	0	0	1
0	0	0	0	0	0	0	0	0	0	0	0	0	0
0	0	0	0	0	0	0	0	0	0	0	0	0	0
0	0	0	0	0	0	0	0	0	0	0	0	0	0
0	0	0	0	0	0	0	0	0	0	0	0	0	0
0	0	0	0	0	0	0	0	0	0	0	0	0	0

0	0	0	0	0	0	0	0	0	0	0	0	0	0
0	0	0	0	0	0	0	0	0	0	0	0	0	0
0													
OTU9286	0	0	0	0	0	0	0	0	0	0	0	0	1
0	0	0	0	0	1	0	0	0	0	0	0	0	0
0	0	0	0	0	0	0	0	0	0	0	0	0	0
0	0	0	0	0	0	0	0	0	0	0	0	0	0
0	0	0	0	0	0	0	0	0	0	0	0	0	0
0	0	0	0	0	0	0	0	0	0	0	0	0	0
0	0	0	0	0	0	0	0	0	0	0	0	0	0
0	0	0	0	0	0	0	0	0	0	0	0	0	0
0													
OTU15966	0	0	0	0	0	0	0	0	0	0	0	0	1
0	0	0	0	0	0	0	0	0	0	0	0	0	0
0	0	0	0	0	0	0	0	0	0	0	0	0	0
0	0	0	0	0	0	0	0	0	0	0	0	0	0
0	0	0	0	0	0	0	0	0	0	0	0	0	0
0	0	0	0	0	0	0	0	0	0	0	0	0	0
0	0	0	0	0	0	0	0	0	0	0	0	0	0
0	0	0	0	0	0	0	0	0	0	0	0	0	0
0													
OTU8037	0	0	0	0	0	0	0	0	0	0	0	0	1
1	0	0	0	0	0	0	0	0	0	0	0	0	0
0	0	0	0	0	0	0	0	0	0	0	0	0	0
0	0	0	0	0	0	0	0	0	0	0	0	0	0
0	0	0	0	0	0	0	0	0	0	0	0	0	0
0	0	0	0	0	0	0	0	0	0	0	0	0	0
0	0	0	0	0	0	0	0	0	0	0	0	0	0
0	0	0	0	0	0	0	0	0	0	0	0	0	0
0													
OTU4124	0	0	0	0	0	0	0	0	0	0	0	0	1
0	0	0	0	0	0	0	0	0	0	0	0	0	0
0	0	0	0	0	0	0	0	0	0	0	0	0	0

0 0 0 0 0 0 0 0 0 0 0 0 0 0
0 0 0 0 0 0 0 0 0 0 0 0 0 0
0 0 0 0 0 0 0 0 0 0 0 0 0 0
0 0 0 0 0 0 0 0 0 0 0 0 0 0
0 0 0 0 0 0 0 0 0 0 0 0 0 0
0
OTU8038 0 0 0 0 0 0 0 0 0 0 0 0 1
0 0 0 0 0 0 0 0 0 0 0 0 0 0
0 0 0 0 0 0 0 0 0 0 0 0 0 0
0 0 0 0 0 0 0 0 0 0 0 0 0 0
0 0 0 0 0 0 0 0 0 0 0 0 0 0
0 0 0 1 0 0 0 0 0 0 0 0 0 0
0 0 0 0 0 0 0 0 0 0 0 0 0 1
0 0 0 0 0 0 0 0 0 0 0 0 0 0
0
OTU4127 0 0 0 0 0 0 0 0 0 0 0 0 1
0 0 0 0 0 0 0 0 0 0 0 0 0 0
0 0 0 0 0 0 0 0 0 0 0 0 0 0
0 0 0 0 0 0 0 0 0 0 0 0 0 0
0 0 0 0 0 0 0 0 0 0 0 0 0 0
0 0 0 0 0 0 0 0 0 0 0 0 0 0
0 0 0 0 0 0 0 0 0 0 0 0 0 0
0 0 0 0 0 0 0 0 0 0 0 0 0 0
0
OTU4126 0 0 0 0 0 0 0 0 0 0 0 0 1
0 0 0 0 0 0 0 0 0 0 0 0 0 0
0 0 0 0 0 0 0 0 0 0 0 0 0 0
0 0 0 0 0 0 0 0 0 0 0 0 0 0
0 0 0 0 0 0 0 0 0 0 0 0 0 0
0 0 0 0 0 0 0 0 0 0 0 0 0 0
0 0 0 0 0 0 0 0 0 0 0 0 0 0
0 0 0 0 0 0 0 0 0 0 0 0 0 0
0

OTU15965	0	0	0	0	0	0	0	0	0	0	0	0	1
6	0	0	0	0	0	0	0	0	0	0	0	0	0
0	0	1	0	0	0	0	1	0	0	0	0	0	0
0	1	0	0	0	0	0	0	0	0	0	0	0	0
0	0	0	0	0	0	0	0	0	0	0	0	0	0
0	0	0	0	0	1	0	0	0	0	0	0	0	0
0	0	0	0	0	0	0	0	0	0	1	0	0	0
1	0	0	0	0	0	0	0	0	0	0	0	0	0
0													
OTU15964	0	0	0	0	0	0	0	0	0	0	0	0	1
0	0	0	0	0	0	0	0	0	0	0	0	0	0
0	0	0	0	0	0	0	0	0	0	0	0	0	0
0	0	0	0	0	0	0	0	0	0	0	0	0	0
0	0	0	0	0	0	0	0	0	0	0	0	0	0
0	0	0	0	0	0	0	0	0	0	0	0	0	0
0	0	0	0	0	0	0	0	0	0	0	0	0	0
0	0	0	0	0	0	0	0	0	0	0	0	0	0
0													
OTU8034	0	0	0	0	0	0	0	0	0	0	0	0	1
0	0	0	0	0	0	0	0	0	0	0	0	0	0
0	0	0	0	0	0	0	0	0	0	0	0	0	0
0	0	0	0	0	0	0	0	0	0	0	0	0	0
0	0	0	0	0	0	0	0	0	0	0	0	0	0
0	0	0	0	0	0	0	0	0	0	0	0	0	0
0	0	0	0	0	0	0	0	0	0	0	0	0	0
0	0	0	0	0	0	0	0	0	0	0	0	0	0
0													
OTU4122	0	0	0	0	0	0	0	0	0	0	0	0	1
0	0	0	0	0	0	0	0	0	0	0	0	0	0
0	0	0	0	0	0	0	0	0	0	0	0	0	0
0	0	0	0	0	0	0	0	0	0	0	0	0	0
0	0	0	0	0	0	0	0	0	0	0	0	0	0
0	0	0	0	0	0	0	0	0	0	0	0	0	0

0	0	0	0	0	0	0	0	0	0	0	0	0	0
0	0	0	0	0	0	0	0	0	0	0	0	0	0
0													
OTU3532	0	0	0	0	0	0	0	0	0	0	0	0	1
0	0	0	0	0	5	0	0	0	0	0	0	0	2
0	0	3	1	0	0	0	0	0	0	0	0	0	1
0	0	0	0	2	0	0	0	2	0	0	0	0	0
0	0	0	0	0	0	2	0	0	0	0	0	1	0
0	0	0	0	0	1	0	0	0	0	0	0	0	0
0	0	0	0	0	0	0	0	0	0	0	0	0	2
0	0	0	0	0	0	0	0	0	0	0	0	0	0
0													
OTU8035	0	0	0	0	0	0	0	0	0	0	0	0	1
0	0	0	0	0	0	0	0	0	0	0	0	0	0
0	0	0	0	0	0	0	0	0	0	0	0	0	0
0	0	0	0	0	0	0	0	0	0	0	0	0	0
0	0	0	0	0	0	0	0	0	0	0	0	0	0
0	0	0	0	0	0	0	0	0	0	0	0	0	0
0	0	0	0	0	0	0	0	0	0	0	0	0	0
0	0	0	0	0	0	0	0	0	0	0	0	0	0
0													
OTU16112	0	0	0	0	0	0	0	0	0	0	0	0	1
0	0	0	0	0	0	0	0	0	0	0	0	0	0
0	0	0	1	0	0	1	0	0	0	0	0	0	0
0	0	1	0	0	0	0	0	0	2	0	0	0	0
1	0	1	0	3	4	1	0	0	0	1	0	1	2
2	0	0	0	0	0	0	0	0	0	0	0	0	0
0	1	0	0	0	0	0	0	1	0	0	0	0	0
0	0	0	0	0	0	1	4	0	3	0	0	0	0
0													
OTU3801	0	0	0	0	0	0	0	0	0	0	0	0	1
0	0	0	0	0	0	0	0	0	0	0	0	0	0
0	1	0	0	0	0	3	0	0	0	0	0	0	0

0	5	0	0	0	0	0	0	0	0	0	0	0	0
0	0	0	0	0	0	0	0	0	1	0	0	1	0
0	0	0	1	0	0	0	0	0	0	0	0	0	0
1	0	0	0	0	0	0	0	0	0	0	0	0	1
0	0	0	0	0	0	0	0	0	0	0	1	2	0
0													
OTU395	0	0	0	0	0	0	0	0	0	0	0	0	1
0	0	0	0	0	0	0	0	0	0	0	0	0	0
0	0	0	0	0	0	22	0	0	0	0	0	0	0
0	0	0	0	0	0	0	0	0	0	0	50	0	0
0	0	0	0	0	0	0	0	0	0	0	0	0	0
0	0	5	0	0	0	0	0	0	0	1	0	0	0
0	0	0	0	0	0	0	0	0	1	0	0	2	0
0	0	0	1	0	0	4	0	6	0	7	0	0	0
0													
OTU13431	0	0	0	0	0	0	0	0	0	0	0	0	1
1	0	0	0	0	0	0	0	0	0	0	0	0	0
0	0	0	0	0	0	0	0	0	0	0	0	0	2
0	0	0	0	0	0	2	0	0	0	0	0	0	1
0	0	0	0	0	0	0	0	0	0	0	0	0	0
0	0	0	0	0	0	0	0	0	0	0	0	0	0
3	0	0	0	4	0	0	0	1	0	1	0	0	0
1	0	0	0	0	0	0	0	0	0	0	1	0	0
0													
OTU4131	0	0	0	0	0	0	0	0	0	0	0	0	1
0	0	0	0	0	0	0	0	0	0	0	0	0	0
0	0	0	0	0	0	0	0	0	0	0	0	0	0
0	0	0	0	0	0	0	0	0	0	0	0	0	0
0	0	0	0	0	0	0	0	0	0	0	0	0	0
0	0	0	0	0	0	0	0	0	0	0	0	0	0
0	0	0	0	0	0	0	0	0	0	0	0	0	0
0	0	0	0	0	0	0	0	0	0	0	0	0	0
0													

OTU12323	0	0	0	0	0	0	0	0	0	0	0	0	1
0	0	0	0	0	0	0	0	0	0	0	0	0	0
0	0	0	0	0	0	0	0	0	0	0	0	0	0
0	0	0	0	0	0	0	0	0	0	0	0	0	0
0	0	0	0	0	0	0	0	0	0	0	0	0	0
0	0	0	0	0	0	0	0	0	0	0	0	0	0
0	0	0	0	0	0	0	0	0	0	0	0	0	0
0	0	0	0	0	0	0	0	0	0	0	0	0	0
0													
OTU12322	0	0	0	0	0	0	0	0	0	0	0	0	1
0	0	0	0	0	0	0	0	0	0	0	0	0	0
0	0	0	0	0	0	0	0	0	0	0	0	0	0
0	0	0	0	0	0	0	0	0	0	0	0	0	0
0	0	0	0	0	0	0	0	0	0	0	0	0	0
0	0	0	0	0	0	0	0	0	0	0	0	0	0
0	0	0	0	0	0	0	0	0	0	0	0	0	0
0	0	0	0	0	0	0	0	0	0	0	0	0	0
0													
OTU8051	0	0	0	0	0	0	0	0	0	0	0	0	1
0	0	0	0	0	0	0	0	0	0	0	0	0	0
0	0	0	0	0	0	0	0	0	0	0	0	0	0
0	0	0	0	0	0	0	0	0	0	0	0	0	0
0	0	0	0	0	0	0	0	0	0	0	0	0	0
0	0	0	0	0	0	0	0	0	0	0	0	0	0
0	0	0	0	0	0	0	0	0	0	0	0	0	0
0	0	0	0	0	0	0	0	0	0	0	0	0	0
0													
OTU15969	0	0	0	0	0	0	0	0	0	0	0	0	1
0	0	0	0	0	0	0	0	0	0	0	0	0	0
0	0	0	0	0	0	0	0	0	0	0	0	0	0
0	0	0	0	0	0	0	0	0	0	0	0	0	0
0	0	0	0	0	0	0	0	0	0	0	0	0	0
0	0	0	0	0	0	0	0	0	0	0	0	0	0

0	0	0	0	0	0	0	0	0	0	0	0	0	0
0	0	0	0	0	0	0	0	0	0	0	0	0	0
0													
OTU5517	0	0	0	0	0	0	0	0	0	0	0	0	1
0	0	0	0	0	0	0	0	0	0	0	0	0	0
0	0	0	0	0	0	0	0	0	0	0	0	0	0
0	0	0	0	0	0	2	0	0	0	0	0	0	0
0	0	0	0	0	0	0	0	0	0	0	0	0	0
0	0	0	0	0	0	0	0	0	0	0	0	0	0
0	0	0	0	0	0	0	0	0	0	0	0	0	0
0	0	0	0	0	0	0	0	0	0	0	0	0	0
0													
OTU8050	0	0	0	0	0	0	0	0	0	0	0	0	1
0	0	0	0	0	0	0	0	0	0	0	0	0	0
0	0	0	0	0	0	0	0	0	0	0	0	0	0
0	0	0	0	0	0	0	0	0	0	0	0	0	0
0	0	0	0	0	0	0	0	0	0	0	0	0	0
0	0	0	0	0	0	0	0	0	0	0	0	0	0
0	0	0	0	0	0	0	0	0	0	0	0	0	0
0	0	0	0	0	0	0	0	0	0	0	0	0	0
0													
OTU4128	0	0	0	0	0	0	0	0	0	0	0	0	1
0	0	0	0	0	0	0	0	0	0	0	0	0	0
0	0	0	0	0	0	0	0	0	0	0	0	0	0
0	0	0	0	0	0	0	0	0	0	0	0	0	0
0	0	0	0	0	0	0	0	0	0	0	0	0	0
0	0	0	0	0	0	0	0	0	0	0	0	0	0
0	0	0	0	0	0	0	0	0	0	0	0	0	0
0	0	0	0	0	0	0	0	0	0	0	0	0	0
0													
OTU15967	0	0	0	0	0	0	0	0	0	0	0	0	1
0	0	0	0	0	0	0	0	0	0	0	0	0	0
0	0	0	0	0	0	0	0	0	0	0	0	0	0

0	0	0	0	0	0	0	0	0	0	0	0	0	0
0	0	0	0	0	0	0	0	0	0	0	0	0	0
0	0	0	0	0	0	0	0	0	0	0	0	0	0
0	0	0	0	0	0	0	0	0	0	0	0	0	0
0	0	0	0	0	0	0	0	0	0	0	0	0	0
0													
OTU15968	0	0	0	0	0	0	0	0	0	0	0	0	1
0	0	0	0	0	0	0	0	0	0	0	0	0	0
0	0	0	0	0	0	0	0	0	0	0	0	0	0
0	0	0	0	0	0	0	0	0	0	0	0	0	0
0	0	0	0	0	0	0	0	0	0	0	0	0	0
0	0	0	0	0	0	0	0	0	0	0	0	0	0
0	0	0	0	0	0	0	0	0	0	0	0	0	0
0	0	0	0	0	0	0	0	0	0	0	0	0	0
0													
OTU239	0	0	0	0	0	0	0	0	0	0	0	0	1
0	1	0	1	0	0	17	0	0	0	0	0	0	0
121	0	0	0	0	0	0	0	0	0	0	0	97	0
1	0	0	0	0	0	0	2	0	0	0	7	0	0
0	0	0	0	0	0	0	0	11	0	0	0	0	0
0	7	1	0	0	0	0	1	0	0	0	0	0	0
0	0	0	1	0	9	0	0	0	0	0	0	1	0
0	0	27	3	1	3	4	4	1	8	0	0	0	2
0													
OTU12324	0	0	0	0	0	0	0	0	0	0	0	0	1
0	0	0	0	0	0	0	0	0	0	0	0	0	0
0	0	0	0	0	0	0	0	0	0	0	0	0	0
0	0	0	0	0	0	0	0	0	0	0	0	0	0
0	0	0	0	0	0	0	0	0	0	0	0	0	0
0	0	0	0	0	0	0	0	0	0	0	0	0	0
0	0	0	0	0	0	0	0	0	0	0	0	0	0
0	0	0	0	0	0	0	0	0	0	0	0	0	0
0													

OTU3685	0	0	0	0	0	0	0	0	0	0	0	0	1
0	0	0	0	0	0	0	0	0	2	0	0	0	0
0	0	0	1	0	0	0	0	0	0	0	0	0	0
0	0	0	0	0	0	0	0	0	0	0	0	0	0
0	0	0	0	0	0	0	0	0	0	0	0	0	0
0	0	0	1	0	0	0	0	0	0	0	0	0	0
0	0	0	0	0	0	0	0	0	0	1	0	0	0
0	0	0	0	0	0	0	0	0	0	0	0	0	0
0													
OTU4549	0	0	0	0	0	0	0	0	0	0	0	0	1
0	0	1	0	0	0	0	0	0	0	0	0	0	0
0	0	0	0	0	0	0	0	0	0	0	0	0	0
0	0	0	0	0	0	0	0	0	0	0	0	0	0
0	0	0	0	0	0	0	0	0	0	0	0	0	0
0	0	0	0	0	0	1	0	0	0	0	0	0	0
0	0	0	0	0	0	0	0	0	0	0	0	0	0
0	0	0	0	0	0	0	0	0	0	0	0	0	0
0													
OTU4667	0	0	0	0	0	0	0	0	0	0	0	0	1
0	0	0	0	0	0	0	0	0	0	0	0	0	0
0	0	0	0	0	0	0	0	0	0	0	0	0	0
0	0	0	0	0	0	0	0	0	0	0	1	0	0
0	0	0	0	0	0	0	0	1	0	1	0	0	0
0	0	0	0	0	0	0	0	0	0	0	0	0	0
0	0	0	0	0	0	0	0	0	0	0	0	0	0
0	0	0	0	0	0	0	0	0	0	0	0	0	0
0													
OTU8055	0	0	0	0	0	0	0	0	0	0	0	0	1
0	0	0	0	0	0	0	0	0	0	0	0	0	0
0	0	0	0	0	0	0	0	0	0	0	0	0	0
0	0	0	0	0	0	0	0	0	0	0	0	0	0
0	0	0	0	0	0	0	0	0	0	0	0	0	0
0	0	0	0	0	0	0	0	0	0	0	0	0	0

0	0	0	0	0	0	0	0	0	0	0	0	0	0
0	0	0	0	0	0	0	0	0	0	0	0	0	0
0													
OTU802	0	0	0	0	0	0	0	0	0	0	0	0	1
0	0	0	1	0	0	0	0	0	0	0	0	0	0
0	0	0	0	0	0	9	0	0	0	0	0	0	0
0	0	0	0	0	0	0	0	0	0	0	26	0	0
0	0	0	0	0	0	0	0	0	0	0	0	0	0
0	0	0	0	0	0	0	0	0	0	0	0	0	0
0	0	0	0	0	0	0	0	0	0	0	0	0	0
0	0	0	0	0	0	0	0	0	0	2	0	0	0
0													
OTU4084	0	0	0	0	0	0	0	0	0	0	0	0	1
0	0	0	0	0	0	0	0	0	0	0	0	0	0
0	0	0	0	0	0	0	0	0	0	0	0	0	0
0	0	0	0	0	0	0	0	0	0	0	0	0	0
0	0	0	0	0	0	0	0	0	0	0	0	0	0
0	0	0	0	0	0	0	0	0	0	0	0	0	0
0	0	0	0	0	0	0	0	0	0	0	0	0	0
0	0	0	0	0	0	0	0	0	0	0	0	0	0
0													
OTU9289	0	0	0	0	0	0	0	0	0	0	0	0	1
0	0	0	0	0	4	0	0	0	0	0	0	0	0
0	0	5	0	0	0	0	0	0	0	0	0	0	0
0	0	0	0	0	0	0	0	0	0	0	0	0	0
0	0	0	0	0	0	0	0	0	0	0	0	0	0
0	1	0	0	0	0	0	0	0	0	0	0	0	0
0	0	1	0	0	0	0	0	1	0	2	0	0	2
0	0	0	0	0	0	0	0	0	0	0	0	0	0
0													
OTU4085	0	0	0	0	0	0	0	0	0	0	0	0	1
0	0	0	0	0	0	0	0	0	0	0	0	0	0
0	0	0	0	0	0	0	0	0	0	0	0	0	0

0	0	0	0	0	0	0	0	0	0	0	0	0	0
0	0	0	0	0	0	0	0	0	0	0	0	0	0
0	0	0	0	0	0	0	0	0	0	0	0	0	0
1	0	0	0	0	0	0	0	0	0	0	0	0	0
0	0	0	0	0	0	0	0	0	0	0	0	0	0
0													
OTU15962	0	0	0	0	0	0	0	0	0	0	0	0	1
0	0	0	0	0	0	0	0	0	0	0	0	0	0
0	0	0	0	0	0	0	0	0	0	0	0	0	0
0	0	0	0	0	0	0	0	0	0	0	0	0	0
0	0	0	0	0	0	0	0	0	0	0	0	0	0
0	0	0	0	0	0	0	0	0	0	0	0	0	0
0	0	0	0	0	0	0	0	0	0	0	0	0	0
0	0	0	0	0	0	0	0	0	0	0	0	0	0
0													
OTU4086	0	0	0	0	0	0	0	0	0	0	0	0	1
0	0	0	0	0	0	0	0	0	0	0	0	0	0
0	0	0	0	0	0	0	0	0	0	0	0	0	0
0	0	0	0	0	0	0	0	0	0	0	0	0	0
0	0	0	0	0	0	0	0	0	0	0	0	0	0
0	0	0	0	0	0	0	0	0	0	0	0	0	0
0	0	0	0	0	0	0	0	0	0	0	0	0	0
0	0	0	0	0	0	0	0	0	0	0	0	0	0
0													
OTU734	0	0	0	0	0	0	0	0	0	0	0	0	1
0	0	0	0	0	0	1	0	0	0	0	0	0	0
0	1	0	0	1	0	0	0	0	0	6	0	2	0
1	0	0	0	0	1	0	0	0	0	0	1	0	0
0	0	0	0	0	0	0	0	0	0	0	0	0	0
0	0	0	0	0	0	0	0	0	0	0	0	0	0
0	0	0	0	0	0	0	0	0	0	0	0	0	0
0	3	0	0	7	0	4	1	0	0	0	0	0	3
5													

OTU1897	0	0	0	0	0	0	0	0	0	0	0	0	0
1	0	0	4	0	0	0	1	0	0	0	0	0	0
0	2	0	0	0	0	2	0	0	0	0	0	0	0
1	0	0	0	0	0	0	1	0	0	0	1	0	0
0	0	0	0	0	0	0	0	0	0	0	2	0	1
1	0	0	0	0	0	0	0	0	0	0	0	0	0
2	0	0	0	0	0	1	0	2	0	2	0	0	0
0	0	0	0	0	0	0	0	0	0	0	1	0	0
0													
OTU627	0	0	0	0	0	0	0	0	0	0	0	0	0
2	0	0	0	0	1	0	0	0	0	0	0	0	0
0	0	0	0	0	0	0	0	0	0	0	0	0	6
0	0	0	0	0	0	0	0	0	0	0	0	0	0
0	0	0	0	0	0	0	0	0	0	0	0	0	0
0	0	0	0	0	0	0	0	0	0	0	0	0	0
0	0	0	0	0	0	0	0	1	0	0	0	0	2
0	0	0	0	0	0	0	0	0	0	0	0	0	0
0													
OTU15961	0	0	0	0	0	0	0	0	0	0	0	0	0
1	0	0	0	0	0	0	0	0	0	0	0	0	0
0	0	0	0	0	0	0	0	0	0	0	0	0	0
0	0	0	0	0	0	0	0	0	0	0	0	0	0
0	0	0	0	0	0	0	0	0	0	0	0	0	0
0	0	0	0	0	0	0	0	0	0	0	0	0	0
0	0	0	0	0	0	0	0	0	0	0	0	0	0
0	0	0	0	0	0	0	0	0	0	0	0	0	0
0													
OTU934	0	0	0	0	0	0	0	0	0	0	0	0	0
1	0	3	1	1	0	0	0	0	0	0	0	0	0
0	0	0	0	0	0	0	1	0	0	0	0	0	0
0	0	2	0	0	0	0	0	0	0	0	0	0	0
0	0	1	0	0	0	1	0	0	1	0	1	0	1
0	0	0	0	0	0	1	0	1	0	0	0	0	0

0	0	0	0	0	0	0	0	2	0	0	0	0	0
0	0	0	0	0	0	0	0	0	0	0	0	3	0
0													
OTU15960	0	0	0	0	0	0	0	0	0	0	0	0	0
1	0	0	0	0	0	0	0	0	0	0	0	0	0
0	0	0	0	0	0	0	0	0	0	0	0	0	0
0	0	0	0	0	0	0	0	0	0	0	0	0	0
0	0	0	0	0	0	0	0	0	0	0	0	0	0
0	0	0	0	0	0	0	0	0	0	0	0	0	0
0	0	0	0	0	0	0	0	0	0	0	0	0	0
0	0	0	0	0	0	0	0	0	0	0	0	0	0
0													
OTU7493	0	0	0	0	0	0	0	0	0	0	0	0	0
1	0	1	5	0	0	0	0	0	0	0	1	0	0
0	0	0	8	7	0	1	2	0	4	1	1	0	0
0	0	0	0	1	0	0	0	2	0	1	0	2	1
2	0	5	0	1	0	2	1	0	0	0	1	0	1
0	0	0	0	0	0	4	0	3	0	8	0	0	0
0	0	0	0	0	0	0	0	5	0	0	0	0	2
1	0	0	0	0	0	0	0	0	0	3	0	15	0
0													
OTU5969	0	0	0	0	0	0	0	0	0	0	0	0	0
2	0	0	0	0	1	0	0	0	0	0	0	0	0
0	0	0	1	0	0	0	0	0	0	0	0	0	0
0	0	0	0	0	0	0	0	1	0	0	0	0	0
0	0	1	0	0	0	0	0	0	0	0	0	0	0
1	0	0	0	0	0	0	0	0	0	0	0	0	0
0	0	0	0	0	0	0	0	0	0	0	0	0	1
0	0	0	0	0	0	0	0	0	0	0	0	0	0
0													
OTU4093	0	0	0	0	0	0	0	0	0	0	0	0	0
1	0	0	0	0	0	0	0	0	0	0	0	0	0
0	0	0	0	0	0	0	0	0	0	0	0	0	1

0 0 0 0 0 0 0 0 0 0 0 0 1 0
0 0 0 0 0 0 0 0 0 0 0 0 0 0
0 0 0 0 0 0 0 0 0 0 0 0 0 0
0 0 0 0 0 0 0 0 0 0 0 0 0 0
0 0 0 0 0 0 0 0 0 0 0 0 0 0
0
OTU6613 0 0 0 0 0 0 0 0 0 0 0 0 0
1 0 0 0 0 0 0 0 0 0 0 0 0 0
0 0 0 0 0 0 0 0 0 0 0 0 0 0
0 0 0 0 0 0 0 0 0 0 0 0 1 0
0 0 0 0 0 0 0 0 0 0 0 0 0 0
0 0 0 0 0 0 0 0 0 0 0 0 0 0
0 0 0 0 0 0 0 0 0 0 0 0 0 0
0 0 0 0 0 0 0 0 0 0 0 0 0 0
0
OTU12338 0 0 0 0 0 0 0 0 0 0 0 0 0
1 0 0 0 0 0 0 0 0 0 0 0 0 0
0 0 0 0 0 0 0 0 0 0 0 0 0 0
0 0 0 0 0 0 0 0 0 0 0 0 0 0
0 0 0 0 0 0 0 0 0 0 0 0 0 0
0 0 0 0 0 0 0 0 0 0 0 0 0 0
0 0 0 0 0 0 0 0 0 0 0 0 0 0
0 0 0 0 0 0 0 0 0 0 0 0 0 0
0
OTU16080 0 0 0 0 0 0 0 0 0 0 0 0 0
2 0 1 0 1 3 0 0 0 0 0 0 0 0
0 0 1 0 0 0 0 1 0 0 0 0 0 2
0 0 1 0 1 0 1 0 1 0 0 0 2 0
1 0 1 0 0 0 0 1 0 0 0 0 0 0
0 0 0 0 0 0 2 0 0 1 0 0 0 0
0 0 0 0 0 0 2 0 2 0 0 0 0 0
0 0 0 0 0 0 0 0 0 0 0 2 0 0
0

OTU4090	0	0	0	0	0	0	0	0	0	0	0	0	0
1	0	0	0	0	0	0	0	0	0	0	0	0	0
0	0	0	0	0	0	0	0	0	0	0	0	0	0
0	0	0	0	0	0	0	0	0	0	0	0	0	0
0	0	0	0	0	0	0	0	0	0	0	0	0	0
0	0	0	0	0	0	0	0	0	0	0	0	0	0
0	0	0	0	0	0	0	0	0	0	0	0	0	0
0	0	0	0	0	0	0	0	0	0	0	0	0	0
0													
OTU8047	0	0	0	0	0	0	0	0	0	0	0	0	0
1	0	0	0	0	0	0	0	0	0	0	0	0	0
0	0	0	0	0	0	0	0	0	0	0	0	0	0
0	0	0	0	0	0	0	0	0	0	0	0	0	0
0	0	0	0	0	0	0	0	0	0	0	0	0	0
0	0	0	0	0	0	0	0	0	0	0	0	0	0
0	0	0	0	0	0	0	0	0	0	0	0	0	0
0	0	0	0	0	0	0	0	0	0	0	0	0	0
0													
OTU4091	0	0	0	0	0	0	0	0	0	0	0	0	0
1	0	0	0	0	0	0	0	0	0	0	0	0	0
0	0	0	0	0	0	0	0	0	0	0	0	0	0
0	0	0	0	0	0	0	0	0	0	0	0	0	0
0	0	0	0	0	0	0	0	0	0	0	0	0	0
0	0	0	0	0	0	0	0	0	0	0	0	0	0
0	0	0	0	0	0	0	0	0	0	0	0	0	0
0	0	0	0	0	0	0	0	0	0	0	0	0	0
0													
OTU4092	0	0	0	0	0	0	0	0	0	0	0	0	0
1	0	0	0	0	0	0	0	0	0	0	0	0	0
0	0	0	0	0	0	0	0	0	0	0	0	0	0
0	0	0	0	0	0	0	0	0	0	0	0	0	0
0	0	0	0	0	0	0	0	0	0	0	0	0	0
0	0	0	0	0	0	0	0	0	0	0	0	0	0

0	0	0	0	0	0	0	0	0	0	0	0	0	0
0	0	0	0	0	0	0	0	0	0	0	0	0	0
0													
OTU1913	0	0	0	0	0	0	0	0	0	0	0	0	0
1	0	0	0	0	0	0	0	0	0	0	0	0	0
0	0	0	0	0	1	0	0	0	0	0	0	0	0
0	0	0	0	0	0	1	0	0	0	0	0	0	0
0	0	0	0	0	0	0	1	0	0	0	0	0	0
0	0	0	0	0	0	0	0	0	0	0	0	0	0
0	0	0	0	0	0	0	0	0	0	0	0	0	0
0	0	0	0	0	0	0	0	0	0	0	0	0	0
0													
OTU16560	0	0	0	0	0	0	0	0	0	0	0	0	0
1	0	0	0	0	0	0	0	0	0	0	0	0	0
0	0	0	0	0	0	0	0	0	1	0	0	0	0
0	0	0	0	0	0	0	0	0	0	0	0	0	0
0	0	0	0	0	0	0	0	0	0	0	0	0	0
0	0	0	0	0	0	0	0	0	0	0	0	0	0
0	0	0	0	0	0	0	0	0	0	1	0	0	0
0	0	0	0	0	0	0	0	0	0	0	0	0	0
0													
OTU4798	0	0	0	0	0	0	0	0	0	0	0	0	0
1	0	0	0	0	0	0	0	0	0	0	0	0	0
0	0	0	0	0	0	0	0	0	0	0	0	0	0
0	0	0	0	0	0	0	1	0	0	0	0	0	0
0	1	0	0	0	0	0	0	0	0	0	0	0	0
0	0	0	0	0	0	0	0	0	0	0	0	0	0
0	0	0	2	0	0	0	0	0	0	0	0	0	0
0	0	0	0	0	0	0	0	0	0	0	0	0	2
0													
OTU577	0	0	0	0	0	0	0	0	0	0	0	0	0
1	0	0	0	0	0	0	0	0	0	0	0	0	0
0	0	0	0	0	0	0	0	0	0	0	0	0	0

0	0	0	0	0	0	0	0	0	0	0	0	0	0
0	0	0	0	0	0	0	0	0	0	0	0	0	0
0	0	0	0	0	0	0	0	0	0	0	0	0	0
0	0	0	0	0	0	0	0	0	0	0	0	0	0
0	0	0	0	0	19	2	0	9	0	0	0	0	0
0													
OTU4164	0	0	0	0	0	0	0	0	0	0	0	0	0
2	0	0	0	0	0	0	0	0	0	0	0	0	0
0	0	0	0	0	0	0	0	0	0	0	0	0	0
0	0	0	0	0	0	0	0	0	0	0	0	0	0
0	0	0	0	0	0	0	0	0	0	0	0	0	0
0	0	0	0	0	0	0	0	0	0	0	0	0	0
0	0	0	0	0	0	0	0	0	0	0	0	0	0
0	0	0	0	0	0	0	0	0	0	0	0	0	0
0													
OTU4758	0	0	0	0	0	0	0	0	0	0	0	0	0
1	0	0	0	0	0	0	0	0	0	0	0	0	0
0	0	0	0	0	0	0	0	0	0	0	0	0	0
0	0	0	0	0	0	0	0	1	0	0	0	0	0
0	0	0	0	0	0	0	0	0	0	0	0	0	0
0	0	0	0	0	0	0	0	0	0	0	0	0	0
0	0	0	0	0	0	0	0	0	0	0	0	0	0
0	0	0	0	0	0	0	0	0	0	0	0	0	0
0													
OTU1795	0	0	0	0	0	0	0	0	0	0	0	0	0
2	0	0	0	0	0	0	0	0	0	0	0	0	0
0	0	0	0	0	0	0	0	0	0	1	0	0	1
0	0	3	0	2	0	0	0	0	0	0	0	0	0
0	0	0	0	0	0	1	0	0	1	1	0	0	0
0	0	0	0	0	0	0	0	0	0	0	0	0	0
0	0	0	0	0	0	0	0	0	0	0	0	0	0
0	0	0	0	0	0	0	0	0	0	0	0	0	0
0													

OTU12335	0	0	0	0	0	0	0	0	0	0	0	0	0
1	0	0	0	0	0	0	0	0	0	0	0	0	0
0	0	0	0	0	0	0	0	0	0	0	0	0	0
0	0	0	0	0	0	0	0	0	0	0	0	0	0
0	0	0	0	0	0	0	0	0	0	0	0	0	0
0	0	0	0	0	0	0	0	0	0	0	0	0	0
0	0	0	0	0	0	0	0	0	0	0	0	0	0
0	0	0	0	0	0	0	0	0	0	0	0	0	0
0													
OTU8049	0	0	0	0	0	0	0	0	0	0	0	0	0
1	0	0	0	0	0	0	0	0	0	0	0	0	0
0	0	0	0	0	0	0	0	0	0	0	0	0	0
0	0	0	0	0	0	0	0	0	0	0	0	0	0
0	0	0	0	0	0	0	0	0	0	0	0	0	0
0	0	0	0	0	0	0	0	0	0	0	0	0	0
0	0	0	0	0	0	0	0	0	0	0	0	0	0
0	0	0	0	0	0	0	0	0	0	0	0	0	0
0													
OTU4076	0	0	0	0	0	0	0	0	0	0	0	0	0
1	0	0	0	0	0	0	0	0	0	0	0	0	0
0	0	0	0	0	0	0	0	0	0	0	0	0	0
0	0	0	0	0	0	0	0	0	0	0	0	0	0
0	0	0	0	0	0	0	0	0	0	0	0	0	0
0	0	0	0	0	0	0	0	0	0	0	0	0	0
0	0	0	0	0	0	0	0	0	0	0	0	0	0
0	0	0	0	0	0	0	0	0	0	0	0	0	0
0													
OTU4079	0	0	0	0	0	0	0	0	0	0	0	0	0
1	0	0	0	0	0	0	0	0	0	0	0	0	0
2	0	0	0	0	0	0	0	0	0	0	0	0	0
0	0	0	0	0	0	0	0	0	0	0	0	0	0
0	0	0	0	0	0	0	0	0	0	0	0	0	0
0	0	0	0	0	0	0	0	0	0	0	0	0	0

0	0	0	0	0	0	0	0	0	0	0	0	0	0
0	0	0	0	0	0	0	0	0	0	0	0	0	0
0													
OTU4078	0	0	0	0	0	0	0	0	0	0	0	0	0
1	0	0	0	0	0	0	0	0	0	0	0	0	0
0	0	0	0	0	0	0	0	0	0	0	0	0	0
0	0	0	0	0	0	0	0	0	0	0	0	0	0
0	0	0	0	0	0	0	0	0	0	0	0	0	0
0	0	0	0	0	0	0	0	0	0	0	0	0	0
0	0	0	0	0	0	0	0	0	0	0	0	0	0
0	0	0	0	0	0	0	0	0	0	0	0	0	0
0													
OTU12337	0	0	0	0	0	0	0	0	0	0	0	0	0
1	0	0	0	0	0	0	0	0	0	0	0	0	0
0	0	0	0	0	0	0	0	0	0	0	0	0	0
0	0	0	0	0	0	0	0	0	0	0	0	0	0
0	0	0	0	0	0	0	0	0	0	0	0	0	0
0	0	0	0	0	0	0	0	0	0	0	0	0	0
0	0	0	0	0	0	0	0	0	0	0	0	0	0
0	0	0	0	0	0	0	0	0	0	0	0	0	0
0													
OTU2830	0	0	0	0	0	0	0	0	0	0	0	0	0
2	0	0	0	0	0	0	0	0	0	0	0	0	0
0	0	1	0	0	0	0	0	0	0	0	0	0	0
0	0	0	0	0	0	0	0	0	0	0	0	0	0
0	0	0	0	0	0	0	0	0	0	0	0	0	0
0	0	0	0	0	0	0	0	0	0	0	0	0	0
0	0	0	0	0	0	0	0	0	0	1	0	0	0
0	0	0	0	0	0	0	0	0	0	0	0	0	0
0													
OTU4073	0	0	0	0	0	0	0	0	0	0	0	0	0
1	0	0	0	0	0	0	0	0	0	0	0	0	0
0	0	0	0	0	0	0	0	0	0	0	0	0	0

0	0	0	0	0	0	0	0	0	0	0	0	0	0
0	0	0	0	0	0	0	0	0	0	0	0	0	0
0	0	0	0	0	0	0	0	0	0	0	0	0	0
0	0	0	0	0	0	0	0	0	0	0	0	0	0
0	0	0	0	0	0	0	0	0	0	0	0	0	0
0													
OTU4097	0	0	0	0	0	0	0	0	0	0	0	0	0
4	0	0	0	0	0	0	0	0	0	0	0	0	0
0	0	0	0	0	0	0	0	0	0	0	0	0	0
0	0	0	0	0	0	0	0	0	0	0	1	0	0
0	0	0	0	0	0	0	0	0	0	0	0	0	0
0	0	0	0	0	0	0	0	0	0	0	0	0	0
0	0	0	0	0	0	0	0	0	0	0	0	0	0
0	0	0	0	0	0	0	0	0	0	0	0	0	0
1													
OTU12330	0	0	0	0	0	0	0	0	0	0	0	0	0
1	0	0	0	0	0	0	0	0	0	0	0	0	0
0	0	0	0	0	0	0	0	0	0	0	0	0	0
0	0	0	0	0	0	0	0	0	0	0	0	0	0
0	0	0	0	0	0	0	0	0	0	0	0	0	0
0	0	0	0	0	0	0	0	0	0	0	0	0	0
0	0	0	0	0	0	0	0	0	0	0	0	0	0
0	0	0	0	0	0	0	0	0	0	0	0	0	0
0													
OTU12329	0	0	0	0	0	0	0	0	0	0	0	0	0
1	0	0	0	0	0	0	0	0	0	0	0	0	0
0	0	0	0	0	0	0	0	0	0	0	0	0	0
0	0	0	0	0	0	0	0	0	0	0	0	0	0
0	0	0	0	0	0	0	0	0	0	0	0	0	0
0	0	0	0	0	0	0	0	0	0	0	0	0	0
0	0	0	0	0	0	0	0	0	0	0	0	0	0
0	0	0	0	0	0	0	0	0	0	0	0	0	0
0													

OTU6097	0	0	0	0	0	0	0	0	0	0	0	0	0
2	0	0	0	0	0	0	0	0	0	0	0	0	0
0	0	0	0	0	0	0	0	0	0	0	0	0	0
0	0	0	0	1	0	0	0	0	0	0	0	0	0
0	0	0	0	0	0	0	0	0	0	0	1	0	1
0	0	0	0	0	0	0	0	0	0	0	0	0	0
0	0	0	0	0	0	0	0	0	0	0	0	0	0
0	0	0	0	0	0	0	0	0	0	0	1	0	0
0													
OTU15963	0	0	0	0	0	0	0	0	0	0	0	0	0
1	0	0	0	0	0	0	0	0	0	0	0	0	0
0	0	0	0	0	0	0	0	0	0	0	0	0	0
0	0	0	0	0	0	0	0	0	0	0	0	0	0
0	0	0	0	0	0	0	0	0	0	0	0	0	0
0	0	0	0	0	0	0	0	0	0	0	0	0	0
0	0	0	0	0	0	0	0	0	0	0	0	0	0
0	0	0	0	0	0	0	0	0	0	0	0	0	0
0													
OTU4075	0	0	0	0	0	0	0	0	0	0	0	0	0
1	0	0	0	0	0	0	0	0	0	0	0	0	0
0	0	0	0	0	0	0	0	0	0	0	0	0	0
0	0	0	0	0	0	0	0	0	0	0	0	0	0
0	0	0	0	0	0	0	0	0	0	0	0	0	0
0	0	0	0	0	0	0	0	0	0	0	0	0	0
0	0	0	0	0	0	0	0	0	0	0	0	0	0
0	0	0	0	0	0	0	0	0	0	0	0	0	0
0													
OTU4074	0	0	0	0	0	0	0	0	0	0	0	0	0
1	0	0	0	0	0	0	0	0	0	0	0	0	0
0	0	0	0	0	0	0	0	0	0	0	0	0	0
0	0	0	0	0	0	0	0	0	0	0	0	0	0
0	0	0	0	0	0	0	0	0	0	0	0	0	0
0	0	0	0	0	0	0	0	0	0	0	0	0	0

0	0	0	0	0	0	0	0	0	0	0	0	0	0
0	0	0	0	0	0	0	0	0	0	0	0	0	0
0													
OTU7428	0	0	0	0	0	0	0	0	0	0	0	0	0
1	0	0	0	0	0	0	0	0	0	0	0	0	0
0	0	0	0	0	0	0	0	0	0	0	0	0	0
0	0	0	0	0	0	0	0	0	0	0	0	0	0
0	0	0	0	0	0	0	0	0	0	0	0	0	0
0	0	0	1	0	0	0	0	0	0	0	0	0	0
0	0	2	0	0	0	0	0	1	0	1	0	0	0
0	0	0	0	0	0	0	0	0	0	0	0	0	0
0													
OTU16525	0	0	0	0	0	0	0	0	0	0	0	0	0
2	0	0	0	0	0	0	0	0	0	0	0	0	0
0	0	0	0	0	0	0	0	0	0	0	0	0	1
0	0	0	0	0	0	0	0	0	0	0	0	0	0
0	0	0	0	0	0	0	0	0	0	0	0	0	0
0	0	0	0	0	0	0	0	0	0	0	0	0	0
0	0	1	0	1	0	0	0	0	0	0	0	0	0
0	0	0	0	0	0	0	0	0	0	0	0	0	0
0													
OTU4083	0	0	0	0	0	0	0	0	0	0	0	0	0
1	0	0	0	0	0	0	0	0	0	0	0	0	0
0	0	0	0	0	0	0	0	0	0	0	0	0	0
0	0	0	0	0	0	0	0	0	0	0	0	0	0
0	0	0	0	0	0	0	0	0	0	0	0	0	0
0	0	0	0	0	0	0	0	0	0	0	0	0	0
0	0	0	0	0	0	0	0	0	0	0	0	0	0
0	0	0	0	0	0	0	0	0	0	0	0	0	0
0													
OTU10651	0	0	0	0	0	0	0	0	0	0	0	0	0
4	0	0	0	0	0	0	0	0	0	0	0	0	0
0	0	0	0	0	0	0	0	0	0	0	0	0	0

0	0	0	0	0	0	0	0	0	0	0	0	0	0
0	0	0	0	0	0	0	0	0	0	0	0	0	0
0	0	0	0	0	0	0	0	0	0	0	0	0	0
0	1	1	0	0	0	0	0	3	0	0	0	0	0
0	0	0	0	0	0	0	0	0	0	0	1	0	0
0													
OTU9267	0	0	0	0	0	0	0	0	0	0	0	0	0
6	0	0	0	1	5	0	0	0	0	0	1	0	0
0	0	6	0	1	0	0	0	0	0	0	0	0	0
0	0	2	0	0	0	0	0	0	0	0	0	0	0
0	0	0	0	0	0	1	1	0	0	0	0	0	0
1	0	0	0	0	0	0	0	0	0	0	0	0	0
0	0	1	0	0	0	0	0	2	0	0	0	0	1
0	0	0	0	0	0	0	0	0	0	0	0	1	0
0													
OTU4080	0	0	0	0	0	0	0	0	0	0	0	0	0
1	0	0	0	0	0	0	0	0	0	0	0	0	0
0	0	0	0	0	0	0	0	0	0	0	0	0	0
0	0	0	0	0	0	0	0	0	0	0	0	0	0
0	0	0	0	0	0	0	0	0	0	0	0	0	0
0	0	0	0	0	0	0	0	0	0	0	0	0	0
0	0	0	0	0	0	0	0	0	0	0	0	0	0
0	0	0	0	0	0	0	0	0	0	0	0	0	0
0													
OTU7422	0	0	0	0	0	0	0	0	0	0	0	0	0
1	0	0	0	0	0	0	0	0	0	0	0	0	0
0	0	0	0	0	0	0	0	0	0	0	0	0	0
0	0	0	0	0	0	0	0	0	0	0	0	0	0
0	0	0	0	0	0	0	0	0	0	0	0	0	0
0	0	0	0	0	0	0	0	0	0	0	0	0	0
0	0	0	0	0	0	0	0	0	0	0	0	0	1
0	0	3	0	0	0	0	0	0	0	0	0	0	0
0													

OTU8103	0	0	0	0	0	0	0	0	0	0	0	0	0
1	0	0	0	0	0	0	0	0	0	0	0	0	0
0	0	0	0	0	0	0	0	0	0	0	0	0	0
0	0	0	0	0	0	0	0	0	0	0	0	0	0
0	0	0	0	0	0	0	0	0	0	0	0	0	0
0	0	0	0	0	0	0	0	0	0	0	0	0	0
0	0	0	0	0	0	0	0	0	0	0	0	0	0
0	0	0	0	0	0	0	0	0	0	0	0	0	0
0													
OTU5444	0	0	0	0	0	0	0	0	0	0	0	0	0
1	0	0	0	0	0	0	0	0	0	0	0	0	0
0	0	0	0	0	0	0	2	1	0	0	0	0	0
0	1	0	0	0	1	0	3	0	0	0	0	1	1
0	0	3	0	0	0	0	0	0	1	0	0	2	1
0	0	0	0	0	0	0	0	0	1	0	0	0	1
0	0	0	0	0	0	0	0	1	0	0	0	1	2
0	1	0	0	0	0	0	0	1	2	0	0	0	0
0													
OTU8102	0	0	0	0	0	0	0	0	0	0	0	0	0
1	0	0	0	0	0	0	0	0	0	0	0	0	0
0	0	0	0	0	0	0	0	0	0	0	0	0	0
0	0	0	0	0	0	0	0	0	0	0	0	0	0
0	0	0	0	0	0	0	0	0	0	0	0	0	0
0	0	0	0	0	0	0	0	0	0	0	0	0	0
0	0	0	0	0	0	0	0	0	0	0	0	0	0
0	0	0	0	0	0	0	0	0	0	0	0	0	0
0													
OTU4082	0	0	0	0	0	0	0	0	0	0	0	0	0
1	0	0	0	0	0	0	0	0	0	0	0	0	0
0	0	0	0	0	0	0	0	0	0	0	0	0	0
0	0	0	0	0	0	0	0	0	0	0	0	0	0
0	0	0	0	0	0	0	0	0	0	0	0	0	0
0	0	0	0	0	0	0	0	0	0	0	0	0	0

0	0	0	0	0	0	0	0	0	0	0	0	0	0
0	0	0	0	0	0	0	0	0	0	0	0	0	0
0													
OTU9165	0	0	0	0	0	0	0	0	0	0	0	0	0
1	0	0	0	0	0	0	0	0	0	0	1	0	0
0	0	0	0	1	0	0	0	0	0	0	0	0	0
0	0	0	0	0	0	1	0	0	0	0	0	0	0
0	0	0	0	0	0	0	0	0	0	0	0	0	0
0	0	0	0	0	0	1	0	0	0	0	0	0	0
2	0	0	0	0	0	0	0	0	0	0	0	0	0
0	0	0	0	0	0	0	0	0	0	0	0	0	0
0													
OTU16519	0	0	0	0	0	0	0	0	0	0	0	0	0
1	0	0	0	0	0	0	0	0	0	0	0	0	0
0	0	0	0	0	0	0	0	0	0	0	0	0	1
[truncated: 587,158 more chars]
